# Large-scale pathway specific polygenic risk and transcriptomic community network analysis identifies novel functional pathways in Parkinson disease

**DOI:** 10.1007/s00401-020-02181-3

**Published:** 2020-06-29

**Authors:** S. Bandres-Ciga, S. Saez-Atienzar, J. J. Kim, M. B. Makarious, F. Faghri, M. Diez-Fairen, H. Iwaki, H. Leonard, J. Botia, M. Ryten, D. Hernandez, J. R. Gibbs, J. Ding, Z. Gan-Or, A. Noyce, L. Pihlstrom, A. Torkamani, A. R. Soltis, C. L. Dalgard, The American Genome Center, S. W. Scholz, B. J. Traynor, D. Ehrlich, C. R. Scherzer, M. Bookman, M. Cookson, C. Blauwendraat, M. A. Nalls, A. B. Singleton, the International Parkinson Disease Genomics Consortium

**Affiliations:** 1 Molecular Genetics Section, Laboratory of Neurogenetics, National Institute on Aging, National Institutes of Health, Bethesda, MD 20892 USA; 2 Neuromuscular Diseases Research Section, Laboratory of Neurogenetics, National Institute on Aging, National Institutes of Health, Bethesda, MD 20892 USA; 3 Fundació Docència i Recerca Mútua Terrassa and Movement Disorders Unit, Department of Neurology, University Hospital Mútua Terrassa, Terrassa, 08221 Barcelona, Spain; 4 Departamento de Ingeniería de la Información y las Comunicaciones, Universidad de Murcia, Murcia, Spain; 5 Department of Molecular Neuroscience, UCL, Institute of Neurology, London, UK; 6 Department of Neurodegenerative Disease, University College London (UCL) Institute of Neurology, London, UK; 7 Department of Neurology and Neurosurgery, McGill University, Montréal, QC Canada; 8 Montreal Neurological Institute, McGill University, Montréal, QC Canada; 9 Department of Human Genetics, McGill University, Montréal, QC Canada; 10 Preventive Neurology Unit, Wolfson Institute of Preventive Medicine, Queen Mary University of London and Department of Neurology, Royal London Hospital, London, UK; 11 Department of Neurology, Oslo University Hospital, Oslo, Norway; 12 The Scripps Research Institute, La Jolla, CA 92037 USA; 13 Department of Anatomy, Physiology & Genetics, Uniformed Services University of the Health Sciences, Bethesda, MA USA; 14 The American Genome Center, Collaborative Health Initiative Research Program, Uniformed Services University of the Health Sciences, Bethesda, MA USA; 15 Neurodegenerative Diseases Research Unit, National Institute of Neurological Disorders and Stroke, Bethesda, MD 20892 USA; 16 Department of Neurology, Johns Hopkins University Medical Center, Baltimore, MD 21287 USA; 17 Parkinson’s Disease Clinic, Office of the Clinical Director, National Institute of Neurological, Disorders and Stroke, National Institutes of Health, Bethesda, MD 20892 USA; 18 Center for Advanced Parkinson Research, Harvard Medical School, Brigham and Women’s Hospital, Boston, MA 0115 USA; 19 Verily Life Sciences, South San Francisco, CA USA; 20 Cell Biology and Gene Expression Section, Laboratory of Neurogenetics, National Institute on Aging, National Institutes of Health, Bethesda, MA USA; 21 Data Tecnica International, Glen Echo, MD 20812 USA

**Keywords:** Parkinson disease, Polygenic risk, Transcriptome community maps, Mendelian randomization

## Abstract

Polygenic inheritance plays a central role in Parkinson disease (PD). A priority in elucidating PD etiology lies in defining the biological basis of genetic risk. Unraveling how risk leads to disruption will yield disease-modifying therapeutic targets that may be effective. Here, we utilized a high-throughput and hypothesis-free approach to determine biological processes underlying PD using the largest currently available cohorts of genetic and gene expression data from International Parkinson’s Disease Genetics Consortium (IPDGC) and the Accelerating Medicines Partnership-Parkinson’s disease initiative (AMP-PD), among other sources. We applied large-scale gene-set specific polygenic risk score (PRS) analyses to assess the role of common variation on PD risk focusing on publicly annotated gene sets representative of curated pathways. We nominated specific molecular sub-processes underlying protein misfolding and aggregation, post-translational protein modification, immune response, membrane and intracellular trafficking, lipid and vitamin metabolism, synaptic transmission, endosomal–lysosomal dysfunction, chromatin remodeling and apoptosis mediated by caspases among the main contributors to PD etiology. We assessed the impact of rare variation on PD risk in an independent cohort of whole-genome sequencing data and found evidence for a burden of rare damaging alleles in a range of processes, including neuronal transmission-related pathways and immune response. We explored enrichment linked to expression cell specificity patterns using single-cell gene expression data and demonstrated a significant risk pattern for dopaminergic neurons, serotonergic neurons, hypothalamic GABAergic neurons, and neural progenitors. Subsequently, we created a novel way of building de novo pathways by constructing a network expression community map using transcriptomic data derived from the blood of PD patients, which revealed functional enrichment in inflammatory signaling pathways, cell death machinery related processes, and dysregulation of mitochondrial homeostasis. Our analyses highlight several specific promising pathways and genes for functional prioritization and provide a cellular context in which such work should be done.

**Electronic supplementary material:**

The online version of this article (10.1007/s00401-020-02181-3) contains supplementary material, which is available to authorized users.

## Introduction

Although a great deal of progress in understanding the genetic underpinnings of familial and sporadic Parkinson disease (PD) has been made, the biological basis and cellular context of this risk remain unclear. We have learned that about 1–2% of PD is associated with a classical Mendelian inheritance pattern, while the majority of disease is driven by a complex set of factors in which polygenic risk seems to play a crucial role [3]. The fact that many of the genes that contain disease-causing mutations also map within risk loci identified by genome-wide association studies (GWAS), supports the notion that common pathways are involved in both forms, and therefore, these pleomorphic genes might interact to regulate downstream common targets in both monogenic and non-monogenic PD [24].

Several common molecular processes have been suggested as critical in PD pathophysiology, including lysosome mediated autophagy, mitochondrial dysfunction, endosomal protein sorting and recycling, immune response, alpha-synuclein aggregation, lipid metabolism and synaptic transmission [2]. A goal in much of this work has been to unify the proteins encoded by PD-linked genes into common pathways. For instance, some success has been seen in this regard within the autosomal recessive genes *PINK1, PRKN*, and *DJ*-*1*, which share a common cellular mechanism: mitochondrial quality control and regulation [12, 18]. However, despite this success, the PD genetics field is still facing the challenge of understanding how genetic risk variants may disrupt biological processes and drive the underlying pathobiology of the disease. In the current era, using genetics to understand the disease process is a key milestone to facilitate the development of targeted therapies.

A priority in elucidating PD etiology lies in defining cumulative risk. GWAS continues to expand the number of genes and loci associated with disease [17], but the majority of these contributors individually exert small effects on PD risk. Current estimates of heritability explained by GWAS loci suggest that there is still an important component of risk yet to be discovered.

Here, we present a novel high-throughput and hypothesis-free approach to detect the existence of PD genetic risk linked to any particular biological pathway. We apply polygenic risk score (PRS) to a total of 2199 curated and well-defined gene sets representative of canonical pathways publicly available in the Molecular Signature Database v7.2 (MSigDB) [26] to define the cumulative effect of pathway-specific genetic variation on PD risk. To assess the impact of rare variation on PD risk explained by significant pathways, we perform gene-set burden analyses in an independent cohort of whole-genome sequencing (WGS) data, including 2101 cases and 2230 controls.

Additionally, we explore cell-type expression specificity enrichment linked to PD etiology by using single-cell RNA sequencing data from brain cells. Furthermore, we use graph-based analyses to generate de novo pathways that could be involved in disease etiology by constructing a transcriptome map of network communities based on RNA sequencing data derived from the blood of 1612 PD patients and 1042 healthy subjects.

Subsequently, we perform summary-data-based Mendelian randomization (SMR) analyses to prioritize genes from significant gene-sets by exploring possible genomic associations with expression quantitative trait loci (eQTL) in public databases and nominate overlapping genes within our transcriptome communities for follow-up functional studies.

Finally, we present a user-friendly platform for the PD research community that enables easy and interactive access to these results (https://pdgenetics.shinyapps.io/pathwaysbrowser/).

## Methods

### Gene set selection representative of canonical pathways

The Molecular Signatures Database (MSigDB database v7.2) is a compilation of annotated gene sets from various sources such as online pathway databases, the biomedical literature, and manual curation by domain experts [15, 26]. We selected the collection “Canonical Pathways” composed of 2199 curated gene sets of pathways annotated from the following databases; Reactome (1499), KEGG (186), BIOCARTA (289), Pathway Interaction Database (196), Matrisome project (10), Signaling Gateway (8), Sigma Aldrich (10), SuperArray SABiosciences (1) (http://software.broadinstitute.org/gsea/msigdb).

### Genotyping data: cohort characteristics, quality control procedures, and study design

**Fig. 1:**
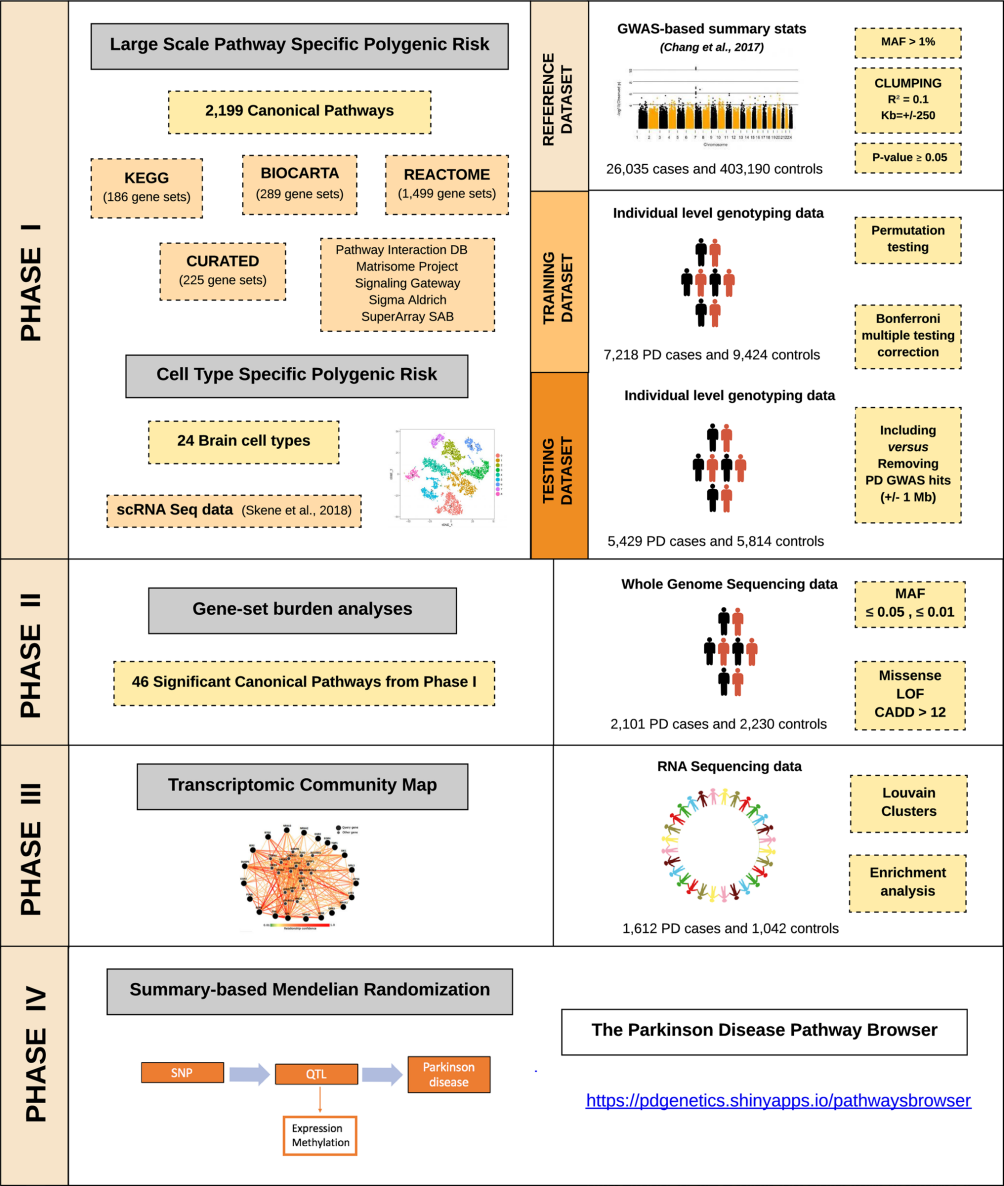
Workflow and rationale summary

 To assess PD risk, summary statistics from Chang et al. [7] PD GWAS meta-analysis involving 26,035 PD cases and 403,190 controls of European ancestry were used as the *reference dataset* for the primary analysis to define risk allele weights. In this study, there were 7,909,453 imputed SNPs tested for association with PD with a minor allele frequency (MAF) > 0.03. Recruitment and genotyping quality control procedures were described in the original report [7]. Individual-level genotyping data not included in Chang et al. [7] and from the last GWAS meta-analysis [17] was then randomly divided as the *training* and *testing datasets*. The *training dataset* used to construct the PRS consisted of 7218 PD cases and 9424 controls, while the *testing dataset* to validate the results consisted of 5429 PD cases and 5814 controls, all of European ancestry (see Fig. 1 for analysis workflow and rationale summary). Demographic and clinical characteristics of the cohorts under study are given in Supplementary Table 1, online resource.

Additional details of these cohorts, along with detailed quality control (QC) methods, can be found in Nalls et al. [17]. For sample QC, in short, individuals with low call rates (< 95%), discordance between genetic and reported sex, heterozygosity outliers (*F*-statistic cutoff of > − 0.15 and < 0.15) and ancestry outliers (± 6 standard deviations from means of eigenvectors 1 and 2 of the 1000 Genomes phase 3 CEU and TSI populations from principal components) were excluded. Further, for genotype QC, variants with a missingness rate of > 5%, minor allele frequency < 0.05, exhibiting Hardy–Weinberg Equilibrium (HWE) < 1E−5 and palindromic SNPs were excluded. Remaining samples were imputed using the Haplotype Reference Consortium (HRC) on the University of Michigan imputation server under default settings with Eagle v2.3 phasing based on *Haplotype Reference Consortium r1.1 2016* (http://www.haplotype-reference-consortium.org), and variants with an imputation quality (*R*^2^ > 0.3) were included.

### Polygenic effect scores for individual biological gene-sets versus PD risk

A polygenic effect score (PES) was generated to estimate polygenic risk for each of the 2199 gene sets representative of biological pathways and then tested for association with PD. PES was calculated based on the weighted allele dose as implemented in *PRSice2* (v2.1.1) (https://github.com/choishingwan/PRSice) [9]. Using the *reference dataset*, we selected variants with a summary statistic *p* value of association less than or equal to 0.05 and with MAF > 1%. We extracted these variants from the *training dataset*, and linkage disequilibrium (LD) clumping was performed using the default *r*^2^ = 0.1 and 250 Kb of distance. Then, 1000 permutations of sample labels were implemented to generate association *p*-value estimates for each gene-set. A *p*-value threshold = 0.05 was considered to prefilter the inclusion of variants in an effort to avoid overfitting when comparing across gene sets as well as improve computational efficiency. The permutation test in the *training dataset* provided a Nagelkerke’s pseudo *r*^2^ value after adjusting for an estimated prevalence of 0.005 (aged population estimate as per Gasser and colleagues), age at onset for cases and age at examination for controls, gender, and 20 PCs to account for population stratification. For those gene-sets surpassing Bonferroni multiple testing correction (*p*-value corrected = 0.05/2199 gene-sets = 2.27E−5), PES was then tested in an independent cohort (*testing dataset)* in a similar way, and overlapping gene-sets significantly associated with PD risk were reported. In an attempt to explore what biological processes were associated with PD risk after excluding known risk factors, the same analyses were performed after removing the 90 known PD GWAS hits [17] and additional SNPs located 1 Mb upstream and downstream from the signal. PES analyses considered that all the variants conferred risk under the additive model and did not cover regulatory regions adjacent to the up or downstream of the genes or intergenic variants.

### Whole-genome sequencing data: cohort characteristics and quality control procedures

The following eight cohorts were utilized in this study; Biofind (https://biofind.loni.usc.edu/), NABEC [11], LNG Path confirmed, PDBP (https://pdbp.ninds.nih.gov/), NIH PD CLINIC, PPMI (https://www.ppmi-info.org/), WELLDERLY and UKBEC. Clinical and demographic characteristics of the cohorts under study are summarised in Supplementary Table 2, online resource. Participants included sporadic PD cases clinically diagnosed by experienced neurologists. PD cases met criteria defined by the UK PD Society Brain Bank. This included 2101 cases and 2230 controls. All individuals were of European descent and were not age- or gender-matched.

DNA sequencing was performed using two vendors: Macrogen and USUHS. For samples sequenced at Macrogen, one microgram of each DNA sample was fragmented by the Covaris System and further prepared according to the Illumina TruSeq DNA Sample preparation guide to obtain a final library of 300–400 bp average insert size. Libraries were multiplexed and sequenced on the Illumina HiSeq X platform. For samples sequenced by USUHS, DNA samples were processed using the Illumina TruSeq DNA PCRFree Sample Preparation kit, starting with 500 ng input and resulting in an average insert size of 310 bp. USUHS processed single-libraries on single lanes on HiSeq X flow cells, and the Macrogen protocol used multiplexing. Paired-end read sequences were processed in accordance with the pipeline standard developed by the Centers for Common Disease Genomics [5]. The GRCh38DH reference genome was used for alignment as specified in the FE standardized pipeline [31]. The Broad Institute’s implementation of this FE standardized pipeline, which incorporates the GATK [8] Best Practices is publicly available and used for WGS processing. Single-nucleotide (SNV) and InDel variants were called from the processed WGS data following the GATK [8] Best Practices [8] using the Broad Institute’s workflow for joint discovery and variant quality score recalibration (VQSR). For quality control, each sample was checked using common methods for genotypes as well and sequence-related metrics. Using Plink v1.9 [6], each sample’s genotype missingness rate (< 95%), heterozygosity rate (exceeding ± 0.15 *F*-stat), and gender were checked. The King v2.1.3 kinship tool (8) was used to check for the presence of duplicate samples. Sequence and alignment related metrics generated by the Broad’s implementation of the FE standardized pipeline were inspected for potential quality problems. This included the sample’s mean sequence depth (< 30×) and contamination rate (> 2%), as reported by VerifyBamID (9), and single nucleotide variant count as reported by Picard’s CollectVariantCallingMetrics (< 3 StDev) based on the sample’s genomic vcf (gvcf). Principal components (PCs) were created for each dataset using PLINK. For the PC calculation, variants were filtered for minor allele frequency (> 0.01), genotype missingness (< 0.05), and HWE (*P*≥1E−6), and minor allele count < 3. GCTA [33] was used to remove cryptically related at the level of first cousins or closer (sharing proportionally more than 12.5% of alleles).

### Gene-set burden analyses

The sequence kernel association test-optimal (SKAT-O) [14] was implemented using default parameters in RVTESTS [35] to determine the difference in the aggregate burden of rare coding genetic variants (minor allele count ≥ 3) between PD cases and controls for the nominated gene-sets by PRS. SKAT-O was applied to aggregate genetic information across defined genomic regions to test for associations with gene-sets of interest under two frequency levels (MAF ≤ 0.03 and MAF ≤ 0.01) and three functional categories (missense, loss of function and Combined Annotation Dependent Depletion (CADD) score > 12 representing between 1 and 10% predicted most pathogenic variants in the genome). Covariates including gender, age at onset (cases), age at enrollment (controls), and 10 PCs were included to adjust the analyses. ANNOVAR was used for variant annotation [30].

### Network expression community map in gene expression data

Baseline peri-diagnostic RNA sequencing data derived from the blood for 1612 PD patients and 1042 healthy subjects available from the Parkinson Progression Marker Initiative (PPMI) was used to construct a network of expression communities based on a graph model with Louvain clusters. This cleaned and normalized data was downloaded from the Accelerating Medicines Partnership for Parkinson’s disease (AMP-PD) on March 1st, 2020. Library preparation, protocol, and transcriptomic quality control procedures can be found in detail in the original source https://amp-pd.org/transcriptomics-data. Prior to analyses, all data for the baseline visit were extracted. Data for each gene was then *z*-transformed to a mean of zero and a standard deviation of one. Scikit-learn’s extraTreeClassifier option was used to extract coding gene features for inclusion in the network builds that are likely to contribute to classifying cases versus controls under default settings in the feature selection phase, leaving 8.3 k protein-coding genes for candidate networks [22]. Following this feature extraction phase, controls were excluded, and case-only correlations were calculated for all remaining gene features. Next, this correlation structure was converted to a graph object using NetworkX [28]. We filtered for network links at positive correlations (upregulated in cases together) between genes greater than or equal to 0.8. Subsequently, the Louvain algorithm was employed to build network communities within this graph object derived from the selected feature set [1].

Finally, pathway enrichment analysis within expression communities was performed to further dissect its biological function using the function g:GOSt from g:ProfileR [19]. The significance of each pathway was tested by hypergeometric tests with Bonferroni correction to calculate the error rate of each network.

### Cell-type polygenic risk enrichment analysis

Single-cell RNA sequencing data [25] based on a total of 9970 cells obtained from several mouse brain regions (neocortex, hippocampus, hypothalamus, striatum, and midbrain) was used to explore cell types associated with PD risk. There are certainly differences between the mouse and the human brain. We used the package EWCE (v. 0.99.2) (https://github.com/NathanSkene/EWCE) to perform mouse to human homolog gene conversion. The package contains a dataset with the human orthologs of Mouse-Genomics-Informatics (MGI) mouse genes (mouse_to_human_homologs list). Out of the 14,579 mouse genes reported in Supplementary Table 4, Skene et al. [25], a total of 13,533 genes (92.82%) were converted to human HGNC symbols. Only genes with a high-confidence (1:1 mapping) were retained. As described in Skene et al. [25], a large fraction of non-matches is reasonable given evolutionary differences between humans and mice. The dataset described by Skene et al. [25], includes the specificity of expression for each gene within each cell type where values range from zero to one and represent the proportion of the total expression of a gene found in one cell type compared to all cell types. The closer the score is to 1, the more specific is the expression in that particular cell type. Taking this into account, PRS *R*^2^ (variance) was calculated within each cell type using *PRSice2* (v2.1.1) as previously described in this manuscript. Cell type expression specificity levels ranging from 0 to 1 were then distributed in deciles. If a particular cell type is associated with PD risk, it is expected to observe a shift in the curve distribution with low PRS R^2^ in non-specific gene sets (i.e., lower deciles) and a higher PRS *R*^2^ in more specific gene sets (i.e., higher deciles). Linear regression adjusted by the number of SNPs included in the PRS was performed to assess the trend of increased PRS *R*^2^ per decile of cell-type expression specificity.

### Summary-data-based Mendelian randomization quantitative trait loci analyses

Two-sample SMR was applied to explore the enrichment of *cis* eQTLs within the 46 gene-sets nominated by our large-scale PRS analysis. The methodology can be interpreted as an analysis to test if the effect size of genetic variants influencing PD risk is mediated by gene expression or methylation to prioritize genes underlying these gene-sets for follow-up functional studies [37]. QTL association summary statistics from well-curated expression datasets were compared to Nalls et al. [17] summary statistics after extracting the gene-set-specific independent SNPs considered as the instrumental variables. Expression datasets used for these analyses include estimates for cis-expression from the Genotype-Tissue Expression (GTEx) Consortium (v6; whole blood and 10 brain regions), the Common Mind Consortium (CMC; dorsolateral prefrontal cortex), the Religious Orders Study and Memory and Aging Project (ROSMAP), and the Brain eQTL Almanac project (Braineac; 10 brain regions). Additionally, we studied expression patterns in blood from the largest eQTL meta-analysis so far [29]. LD pruning and clumping were carried out using default SMR protocols (http://cnsgenomics.com/software/smr). Multi-SMR p-values (gene-level expression summaries for eQTLs) were adjusted by Bonferroni multiple test correction considering the number of genes tested per gene-set, and HEIDI was used to detect pleiotropic associations between the expression levels and PD risk that could be biasing the model at a p-value < 0.01 [32]. Effect estimates represent the change in PD odds ratio per one standard deviation increase in gene expression. Enrichment of *cis* expression was assessed per gene-set and per tissue. The number of genes tested per gene-set were Bonferroni corrected, and a Chisq test was applied to assess whether the proportion of QTLs per gene-set was significantly higher than expected by chance.

## Results

### Large-scale PES analysis nominates biological processes involved in PD risk

**Fig. 2:**
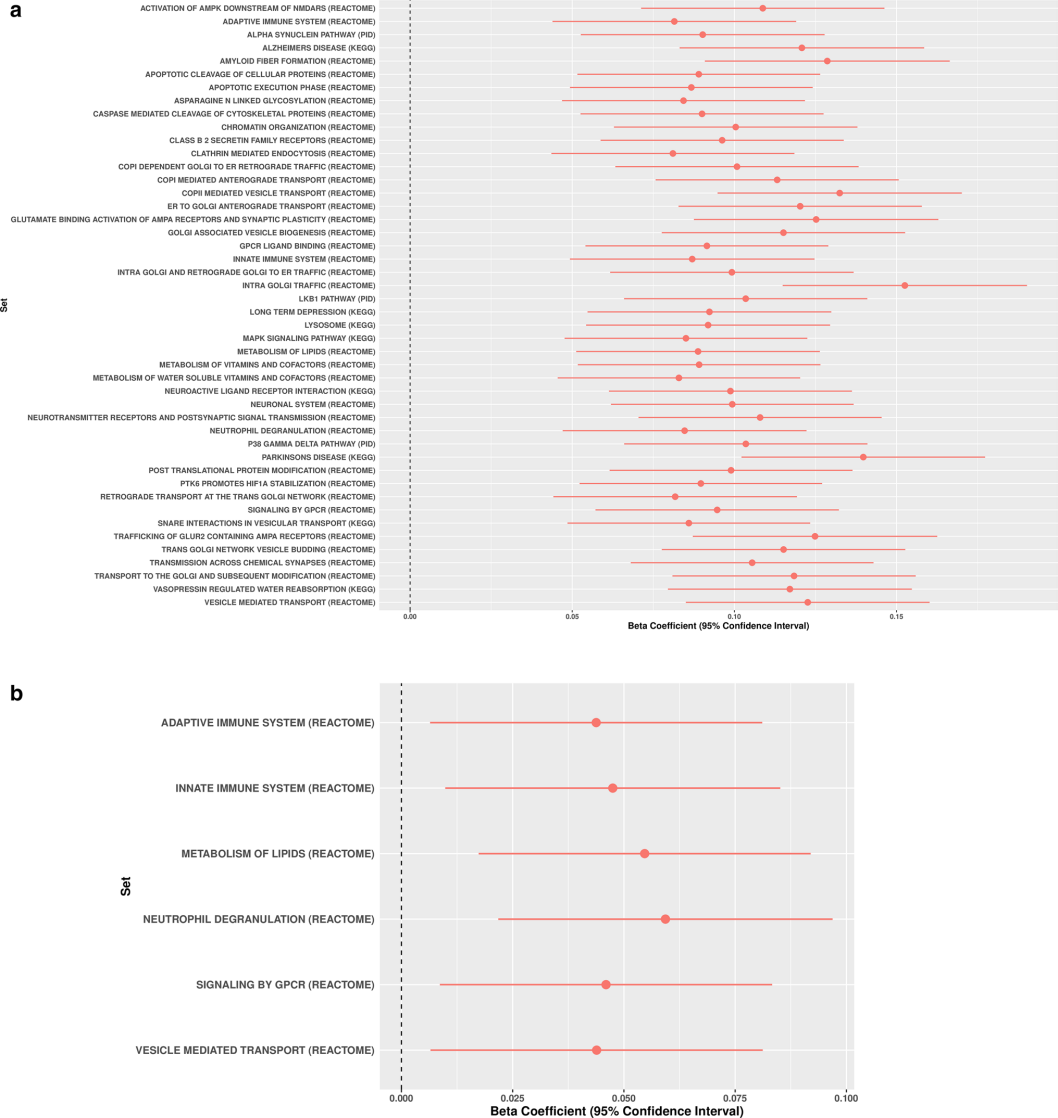

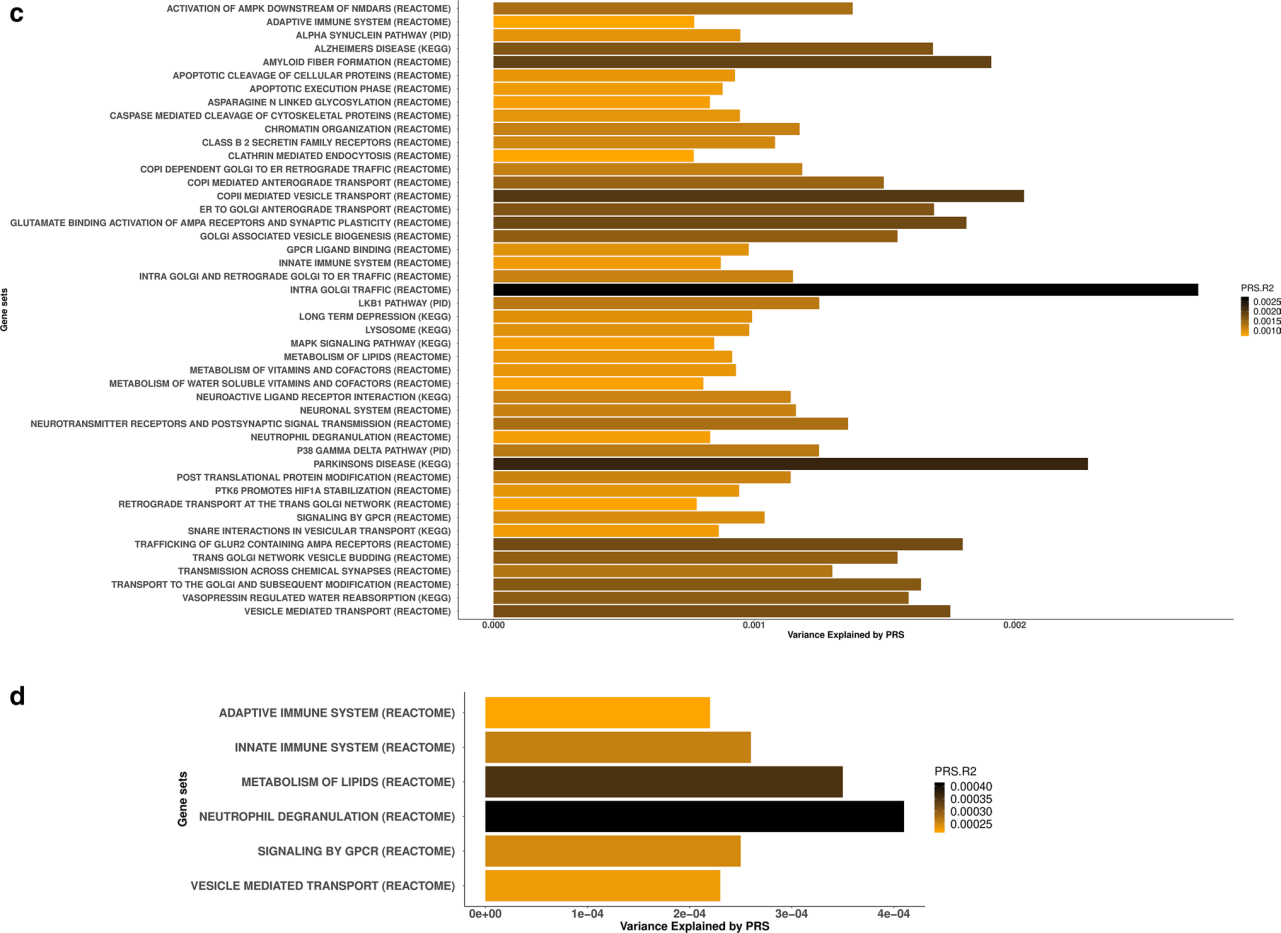
Canonical pathways associated with Parkinson disease risk through common genetic variation based on PES analyses. Forest plots showing polygenic risk score estimates for the significant canonical pathways in the replication phase including (**a**) and removing (**b**) PD known risk loci ± 1Mb upstream and downstream. Estimates of variance explained by PRS for the significant canonical pathways including (**c**) or excluding (**d**) PD known risk loci ± 1Mb upstream and downstream

Out of the 2199 gene sets representative of biological processes included in this report, 279 gene-sets were significantly associated with PD risk in the *training phase* (Bonferroni threshold for significance 0.05/2,199 = 2.27E−5) (Supplementary Table 3, online resource, https://pdgenetics.shinyapps.io/pathwaysbrowser/*)*. Following the same analysis workflow, a total of 46 gene sets were replicated in the *testing phase* and nominated as potentially linked to PD risk through common genetic variation (Table 1, Fig. 2a, b).

**Table 1:** Canonical pathways significantly associated with PD risk in the *discovery* and *replication phases* through common variation

Gene set	*N* genes	Discovery					Replication				
		PRS *R*^2^	Beta	SE	*P*	Num SNP	PRS *R*^2^	Beta	SE	*P*	Num SNP
Activation of AMPK downstream of NMDARS (REACTOME)	29	0.0010	0.0936	0.0162	8.44E−09	18	0.0014	0.1088	0.0191	1.32E−08	9
Adaptive immune system (REACTOME)	811	0.0040	0.1867	0.0164	5.67E−30	455	0.0008	0.0815	0.0192	2.11E−05	169
Alpha synuclein pathway (PID)	32	0.0015	0.1157	0.0164	1.52E−12	34	0.0009	0.0902	0.0192	2.54E−06	17
Alzheimers disease (KEGG)	165	0.0023	0.1410	0.0163	5.91E−18	175	0.0017	0.1208	0.0192	3.34E−10	50
Amyloid fiber formation (REACTOME)	107	0.0031	0.1646	0.0164	7.60E−24	28	0.0019	0.1286	0.0193	2.40E−11	15
Apoptotic cleavage of cellular proteins (REACTOME)	38	0.0019	0.1289	0.0164	3.71E−15	41	0.0009	0.0890	0.0191	3.12E−06	23
Apoptotic execution phase (REACTOME)	52	0.0018	0.1243	0.0164	2.91E−14	48	0.0009	0.0867	0.0191	5.53E−06	26
Asparagine *N* linked glycosylation (REACTOME)	304	0.0020	0.1331	0.0163	3.21E−16	235	0.0008	0.0843	0.0191	1.01E−05	100
Caspase mediated cleavage of cytoskeletal proteins (REACTOME)	12	0.0021	0.1372	0.0164	5.91E−17	15	0.0009	0.0900	0.0191	2.47E−06	13
Chromatin organization (REACTOME)	272	0.0007	0.0773	0.0163	2.11E−06	201	0.0012	0.1004	0.0191	1.56E−07	79
Class B 2 secretin family receptors (REACTOME)	94	0.0015	0.1146	0.0163	1.98E−12	65	0.0011	0.0962	0.0191	4.84E−07	23
Clathrin mediated endocytosis (REACTOME)	145	0.0013	0.1079	0.0162	3.06E−11	151	0.0008	0.0810	0.0191	2.22E−05	81
COPI dependent GOLGI to ER retrograde traffic (REACTOME)	99	0.0016	0.1187	0.0162	2.52E−13	76	0.0012	0.1008	0.0191	1.38E−07	30
COPI mediated anterograde transport (REACTOME)	101	0.0008	0.0832	0.0162	3.00E−07	81	0.0015	0.1132	0.0191	3.23E−09	36
COPII mediated vesicle transport (REACTOME)	68	0.0013	0.1070	0.0163	5.69E−11	46	0.0020	0.1325	0.0192	5.37E−12	20
ER to GOLGI anterograde transport (REACTOME)	154	0.0012	0.1008	0.0163	5.87E−10	114	0.0017	0.1203	0.0191	3.25E−10	48
Glutamate binding activation of AMPA receptors and synaptic plasticity (REACTOME)	31	0.0020	0.1308	0.0163	9.99E−16	48	0.0018	0.1252	0.0192	7.34E−11	12
GOLGI associated vesicle biogenesis (REACTOME)	56	0.0014	0.1118	0.0162	4.87E−12	61	0.0016	0.1151	0.0191	1.79E−09	32
GPCR ligand binding (REACTOME)	454	0.0017	0.1215	0.0163	9.49E−14	263	0.0010	0.0915	0.0191	1.65E−06	88
Innate immune system (REACTOME)	1104	0.0037	0.1790	0.0164	7.05E−28	677	0.0009	0.0870	0.0192	6.11E−06	281
Intra GOLGI and retrograde GOLGI to er traffic (REACTOME)	202	0.0019	0.1275	0.0163	4.52E−15	156	0.0011	0.0992	0.0191	2.19E−07	60
Intra GOLGI traffic (REACTOME)	44	0.0013	0.1049	0.0163	1.13E−10	36	0.0027	0.1526	0.0192	2.05E−15	12
LKB1 pathway (PID)	47	0.0011	0.0988	0.0162	1.12E−09	40	0.0012	0.1035	0.0191	6.25E−08	21
Long term depression (KEGG)	70	0.0017	0.1204	0.0163	1.35E−13	170	0.0010	0.0923	0.0192	1.47E−06	41
Lysosome (KEGG)	121	0.0009	0.0890	0.0164	5.51E−08	85	0.0010	0.0919	0.0192	1.67E−06	46
MAPK signaling pathway (KEGG)	267	0.0013	0.1058	0.0163	8.31E−11	322	0.0008	0.0851	0.0191	8.34E−06	84
Metabolism of lipids (REACTOME)	738	0.0029	0.1585	0.0163	2.99E−22	607	0.0009	0.0888	0.0192	3.58E−06	227
Metabolism of vitamins and cofactors (REACTOME)	189	0.0011	0.0965	0.0162	2.67E−09	201	0.0009	0.0891	0.0191	3.00E−06	86
Metabolism of water soluble vitamins and cofactors (REACTOME)	123	0.0009	0.0881	0.0162	5.47E−08	112	0.0008	0.0829	0.0191	1.38E−05	53
Neuroactive ligand receptor interaction (KEGG)	272	0.0020	0.1316	0.0163	6.58E−16	328	0.0011	0.0988	0.0191	2.34E−07	91
Neuronal system (REACTOME)	411	0.0044	0.1961	0.0164	7.03E−33	880	0.0012	0.0994	0.0191	1.92E−07	191
Neurotransmitter receptors and postsynaptic signal transmission (REACTOME)	204	0.0023	0.1430	0.0163	1.76E−18	390	0.0014	0.1079	0.0191	1.65E−08	98
Neutrophil degranulation (REACTOME)	478	0.0020	0.1316	0.0163	6.89E−16	294	0.0008	0.0847	0.0192	1.01E−05	128
P38 gamma delta pathway (PID)	11	0.0020	0.1334	0.0164	3.34E−16	13	0.0012	0.1035	0.0191	6.26E−08	11
Parkinsons disease (KEGG)	128	0.0029	0.1635	0.0168	2.09E−22	39	0.0023	0.1398	0.0192	3.01E−13	18
Post translational protein modification (REACTOME)	1429	0.0074	0.2568	0.0166	3.27E−54	1165	0.0011	0.0990	0.0191	2.20E−07	391
PTK6 promotes HIF1A stabilization (REACTOME)	6	0.0025	0.1580	0.0178	6.51E−19	18	0.0009	0.0897	0.0191	2.60E−06	13
Retrograde transport at the trans GOLGI network (reactome)	49	0.0008	0.0856	0.0163	1.52E−07	41	0.0008	0.0817	0.0192	1.98E−05	17
Signaling by GPCR (REACTOME)	1184	0.0027	0.1534	0.0164	7.91E−21	884	0.0010	0.0947	0.0191	7.58E−07	282
Snare interactions in vesicular transport (KEGG)	38	0.0008	0.0834	0.0162	2.64E−07	29	0.0009	0.0860	0.0191	6.67E−06	8
Trafficking of GLUR2 containing AMPA receptors (REACTOME)	17	0.0016	0.1184	0.0163	3.61E−13	37	0.0018	0.1249	0.0192	8.29E−11	10
Trans GOLGI network vesicle budding (REACTOME)	72	0.0015	0.1148	0.0162	1.31E−12	76	0.0016	0.1152	0.0191	1.79E−09	37
Transmission across chemical synapses (REACTOME)	269	0.0030	0.1634	0.0163	1.56E−23	517	0.0013	0.1055	0.0191	3.31E−08	124
Transport to the GOLGI and subsequent modification (REACTOME)	185	0.0018	0.1238	0.0163	3.14E−14	154	0.0016	0.1184	0.0191	6.02E−10	64
Vasopressin regulated water reabsorption (KEGG)	44	0.0012	0.1045	0.0163	1.43E−10	55	0.0016	0.1171	0.0192	1.03E−09	16
Vesicle mediated transport (REACTOME)	723	0.0044	0.1964	0.0164	5.90E−33	577	0.0018	0.1226	0.0192	1.57E−10	227

*PRS R2* variance explained by polygenic risk score, *SE* standard error, *P P*-value, *num_SNP* Number of SNPs included in each pathway analysis

Supplementary Table 4, (online resource) summarizes what SNPs within the 90 risk loci located up to 1 Mb upstream and downstream from the GWAS signal were included for each of the 2199 gene-sets as part of the large-scale polygenic risk score analyses for both the training and testing phases.

After excluding the 90 PD risk loci and SNPs located 1 Mb upstream and downstream from the GWAS hits, six gene sets including adaptive immune system, innate immune system, vesicle mediated transport, signaling by G protein-coupled receptors (GPCR) ligand binding, metabolism of lipids and neutrophil degranulation remained significant, suggesting as yet unidentified risk within these gene-sets (Bonferroni threshold for significance 2.27E−5) (Table 2, Fig. 2c, d). For an easy interpretation of these findings, significant gene-sets were clustered in hierarchies according to genetic redundancy, as highlighted in Supplementary Figs. 1, 2, online resource. Additionally, considering genetic pleiotropy across the 46 gene-sets, we prioritized the top 1% of genes involved in multiple pathways as a way of nominating promising PD candidate genes (Supplementary Table 5).

**Table 2:** Canonical pathways significantly associated with PD risk through common variation in the *discovery* and *replication phases* after excluding PD known risk loci ± 1 Mb upstream and downstream

Gene set	Discovery					Replication				
	PRS *R*^2^	Beta	SE	*P*	Num SNP	PRS *R*^2^	Beta	SE	*P*	Num SNP
Adaptive immune system (REACTOME)	0.0028	0.1560	0.0163	1.42E−21	397	0.0002	0.0438	0.0190	2.15E−02	182
Innate immune system (REACTOME)	0.0026	0.1522	0.0163	1.12E−20	621	0.0003	0.0475	0.0192	1.34E−02	332
Vesicle mediated transport (REACTOME)	0.0022	0.1398	0.0163	1.03E−17	515	0.0002	0.0439	0.0190	2.12E−02	259
Signaling by GPCR (REACTOME)	0.0019	0.1299	0.0163	1.86E−15	816	0.0002	0.0460	0.0190	1.58E−02	329
Metabolism of lipids (REACTOME)	0.0018	0.1265	0.0163	8.08E−15	538	0.0004	0.0547	0.0190	4.08E−03	275
Neutrophil degranulation (REACTOME)	0.0012	0.1026	0.0163	2.73E−10	259	0.0004	0.0593	0.0192	1.96E−03	143

*PRS R*^*2*^ variance explained by polygenic risk score, *SE* standard error, *P P*-value, *num_SNP* number of SNPs included in each pathway analysis

In an attempt to define etiological subtypes of PD, we performed Uniform Manifold Approximation and Projection for Dimension Reduction Analysis (UMAP) to explore the possibility of clustering different subgroups of patients that could be enriched for risk in certain molecular pathways. UMAP analysis showed two different clusters of patients according to the pathway-specific PES (subgroup 1 and subgroup 2; Supplementary Fig. 3a, online resource). Subgroup 1 was not enriched on any *LRRK2* G2019S carriers, while all patients from subgroup 2 (*N* = 100) were *LRRK2* G2019S carriers. When *LRRK2* gene boundaries were removed from the analysis and PES were calculated per individual, no subgroups were observed. We assume that since *LRRK2* G2019S is the main risk factor for PD, this variant overweights PES for those pathways in which *LRRK2* plays a role in (Supplementary Fig. 3b, online resource). This would suggest that pathway-specific PES by itself is not an accurate way to define etiological subgroups of the disease since association does not involve prediction. Future multimodality studies are necessary to increase discriminative accuracy given the heterogeneous nature of PD.

### Gene-set-based burden analyses identifies gene-sets involved in PD risk through rare variation

To test whether the same biological processes are enriched by rare coding variants, we implemented gene-set based SKAT-O in a large WGS cohort composed of 2101 PD cases and 2230 controls. Out of the 46 gene-sets significantly associated with PD risk through common variation, 20 were linked through low-frequency genetic variation (MAF ≤ 3%) and 19 through rare variation (MAF ≤ 1%), at a *p*-value < 0.05 (Table 3). At a MAF threshold ≤ 3%, 12 gene-sets remained significantly associated with PD risk when focusing only on missense mutations, 4 when considering only loss of function variants and 6 when filtering by CADD score > 12 (~ among the 1–10% most pathogenic variants in the genome) (Table 3). At a MAF threshold ≤ 1%, 12 gene-sets remained significantly associated with PD risk when focusing only on missense mutations, four when considering only loss of function variants and five when filtering by CADD score > 12 (Table 3). Considering a more stringent p-value (Bonferroni threshold for significance 0.05/46 gene-sets = 0.001), five gene sets including Alzheimer’s disease, Parkinson’s disease, Transmission across chemical synapses, Neuroactive ligand receptor interaction and GPCR ligand binding remained significant at MAF ≤ 3%. When focusing on MAF ≤ 1%, the above mentioned gene sets in addition to Aspargine-N-glycosylation were significantly associated with PD risk.

**Table 3:** Association of canonical pathways and PD risk through rare variation

Gene set	Functional subcategory	MAF < 3		MAF < 1	
		Num SNP	SKAT-O P	Num SNP	SKAT-O P
Activation of AMPK downstream of NMDARS (REACTOME)	Missense	84	0.633	73	0.680
	Loss of function	31	0.015	28	0.892
	CADD > 12%	2	0.491	2	0.491
Adaptive immune system (REACTOME)	Missense	3369	0.061	3128	9.38E−02
	Loss of function	1724	0.003	1589	6.79E−03
	CADD > 12%	93	0.332	86	1
Alpha synuclein pathway (PID)	Missense	130	0.654	121	0.653
	Loss of function	82	0.023	77	1.32E−02
	CADD > 12%	3	0.006	3	5.95E−03
Alzheimers disease (KEGG)	Missense	594	2.15E−10	563	1.02E−06
	Loss of function	375	0.739	343	0.326
	CADD > 12%	13	0.075	13	0.075
Amyloid fiber formation (REACTOME)	Missense	308	0.026	289	0.204
	Loss of function	120	0.086	112	4.86E−02
	CADD > 12%	6	1	4	1
Apoptotic cleavage of cellular proteins (REACTOME)	Missense	356	0.073	332	0.686
	Loss of function	122	0.791	115	0.796
	CADD > 12%	5	0.616	5	0.616
Apoptotic execution phase (REACTOME)	Missense	397	0.111	372	0.631
	Loss of function	128	0.475	120	0.572
	CADD > 12%	7	0.794	7	0.794
Asparagine *N* linked glycosylation (REACTOME)	Missense	2956	0.021	2728	6.14E−04
	Loss of function	1226	0.233	1123	0.448
	CADD > 12%	69	0.544	68	0.427
Caspase mediated cleavage of cytoskeletal proteins (REACTOME)	Missense	159	0.313	151	0.516
	Loss of function	33	0.737	29	0.534
	CADD > 12%	1	0.691	NA	NA
Chromatin organization (REACTOME)	Missense	1213	0.284	1134	0.622
	Loss of function	680	0.813	628	0.777
	CADD > 12%	24	0.794	23	0.594
Class B 2 secretin family receptors (REACTOME)	Missense	327	0.452	298	0.080
	Loss of function	109	0.297	97	0.157
	CADD > 12%	15	0.525	13	0.592
Clathrin mediated endocytosis (REACTOME)	Missense	668	0.196	632	0.240
	Loss of function	410	0.256	363	0.892
	CADD > 12%	15	0.295	15	0.295
Copi dependent GOLGI to ER retrograde traffic (REACTOME)	Missense	576	0.474	525	0.237
	Loss of function	234	0.369	202	0.896
	CADD > 12%	8	0.081	8	0.081
COPI mediated anterograde transport (REACTOME)	Missense	595	0.711	547	0.568
	Loss of function	232	0.671	216	1
	CADD > 12%	13	1	13	1
COPII mediated vesicle transport (REACTOME)	Missense	327	0.742	300	3.13E−01
	Loss of function	149	0.729	140	0.417
	CADD > 12%	5	0.644	5	0.644
ER to Golgi anterograde transport (REACTOME)	Missense	900	0.307	826	3.42E−02
	Loss of function	378	0.426	356	0.808
	CADD > 12%	16	1	16	1
Glutamate binding activation of AMPA receptors and synaptic plasticity (REACTOME)	Missense	83	0.222	75	0.399
	Loss of function	87	0.493	80	0.224
	CADD > 12%	5	0.236	5	0.236
GOLGI associated vesicle biogenesis (REACTOME)	Missense	277	0.224	260	0.428
	Loss of function	118	0.315	110	0.771
	CADD > 12%	8	1	8	1
GPCR ligand binding (REACTOME)	Missense	1769	2.12E−06	1632	2.16E−10
	Loss of function	438	0.185	395	0.445
	CADD > 12%	49	0.540	45	0.605
Innate immune system (REACTOME)	Missense	7162	0.009	6663	2.56E−03
	Loss of function	2965	0.122	2714	0.212
	CADD > 12%	178	0.819	169	0.521
Intra GOLGI and retrograde GOLGI to ER traffic (REACTOME)	Missense	1052	0.241	969	0.065
	Loss of function	441	0.853	389	0.405
	CADD > 12%	20	0.857	18	0.873
Intra GOLGI traffic (REACTOME)	Missense	213	0.284	199	0.077
	Loss of function	76	1	69	0.870
	CADD > 12%	5	0.801	5	0.801
Lkb1 pathway (PID)	Missense	233	0.114	224	0.440
	Loss of function	148	0.576	140	0.928
	CADD > 12%	6	0.270	4	1
Long-term depression (KEGG)	Missense	334	0.073	309	0.444
	Loss of function	195	0.164	166	0.818
	CADD > 12%	8	0.003	7	8.86E−03
Lysosome (KEGG)	Missense	673	0.034	628	0.528
	Loss of function	356	0.400	332	0.390
	CADD > 12%	29	0.272	28	0.837
Mapk signaling pathway (KEGG)	Missense	1156	0.091	1088	0.332
	Loss of function	669	0.691	616	1
	CADD > 12%	28	0.066	25	0.111
Metabolism of lipids (REACTOME)	Missense	3975	0.287	3701	0.204
	Loss of function	1881	0.713	1703	0.874
	CADD > 12%	149	0.782	138	0.640
Metabolism of vitamins and cofactors (REACTOME)	Missense	1252	0.210	1161	0.105
	Loss of function	593	0.765	527	0.872
	CADD > 12%	45	0.151	40	0.196
Metabolism of water soluble vitamins and cofactors (REACTOME)	Missense	740	0.487	688	0.187
	Loss of function	405	0.367	366	0.396
	CADD > 12%	29	0.287	26	0.114
Neuroactive ligand receptor interaction (KEGG)	Missense	1270	1.33E−09	1172	2.15E−10
	Loss of function	378	0.489	344	0.594
	CADD > 12%	37	0.260	33	0.814
Neuronal system (REACTOME)	Missense	1848	0.374	1704	0.606
	Loss of function	872	0.222	787	0.858
	CADD > 12%	47	0.011	40	9.62E−03
Neurotransmitter receptors and postsynaptic signal transmission (REACTOME)	Missense	753	0.931	696	0.870
	Loss of function	396	0.115	364	1
	CADD > 12%	16	0.006	15	7.76E−03
Neutrophil degranulation (REACTOME)	Missense	2715	0.353	2525	0.111
	Loss of function	1207	0.766	1088	0.942
	CADD > 12%	98	0.572	91	0.613
P38 gamma delta pathway (PID)	Missense	62	0.386	56	0.391
	Loss of function	40	0.813	36	0.680
	CADD > 12%	2	0.664	2	0.664
Parkinsons disease (KEGG)	Missense	301	2.15E−10	276	2.72E−09
	Loss of function	185	0.024	175	9.14E−03
	CADD > 12%	11	0.289	11	0.289
Post-translational protein modification (REACTOME)	Missense	9055	0.011	8385	1.54E−03
	Loss of function	3643	0.675	3315	0.638
	CADD > 12%	213	0.471	199	0.467
PTK6 promotes HIF1A stabilization (REACTOME)	Missense	42	0.047	39	5.87E−03
	Loss of function	16	0.309	14	0.256
	CADD > 12%	2	0.170	2	0.170
Retrograde transport at the trans GOLGI network (REACTOME)	Missense	241	0.044	230	1.70E−02
	Loss of function	94	0.902	80	0.854
	CADD > 12%	3	0.499	2	0.792
Signaling by GPCR (REACTOME)	Missense	5789	0.002	5312	1.39E−05
	Loss of function	1623	0.388	1464	0.688
	CADD > 12%	138	0.445	128	0.306
Snare interactions in vesicular transport (KEGG)	Missense	108	0.316	101	0.087
	Loss of function	40	0.531	35	0.590
	CADD > 12%	3	0.034	2	0.072
Trafficking of GLUR2 containing AMPA receptors (REACTOME)	Missense	48	1	45	1
	Loss of function	49	0.095	43	0.920
	CADD > 12%			NA	NA
Trans GOLGI network vesicle budding (REACTOME)	Missense	330	0.118	313	0.229
	Loss of function	160	0.197	148	0.728
	CADD > 12%	11	1	11	1
Transmission across chemical synapses (REACTOME)	Missense	1117	0.441	1028	0.731
	Loss of function	572	0.134	518	0.771
	CADD > 12%	23	9.99E−04	21	7.88E−03
Transport to the GOLGI and subsequent modification (REACTOME)	Missense	1018	0.180	933	2.75E−02
	Loss of function	435	0.505	404	0.865
	CADD > 12%	19	0.672	19	0.672
Vasopressin-regulated water reabsorption (KEGG)	Missense	196	0.318	185	0.199
	Loss of function	84	0.650	78	0.439
	CADD > 12%	2	0.239	2	0.239
Vesicle-mediated transport (REACTOME)	Missense	3655	1	3395	0.484
	Loss of function	1757	1	1584	0.165
	CADD > 12%	87	0.818	80	1

*MAF* minor allele frequency, *SKAT* SNP-set Sequence Kernel Association Test, *P P*-value, *CADD* combined annotation dependent depletion, *NA* non applicable

After removing PD GWAS hits and SNPs located 1 Mb upstream and downstream, innate immune system and signaling by GPCR remained significantly associated with PD suggesting that rare variation within these gene-sets contributes to PD heritability (Supplementary Table 6, online resource).

In an effort to prioritize the top genes within significant gene-sets showing the highest cumulative effect on PD risk, individual gene-based SKAT-O analyses were performed considering a MAF threshold ≤ 3% and three functional categories (missense, loss of function and CADD score > 12). Using this approach, gene-level prioritization is highlighted in Supplementary Table 7, online resource.

### Transcriptome map reveals expression modules linked to PD etiology

**Fig. 3:**
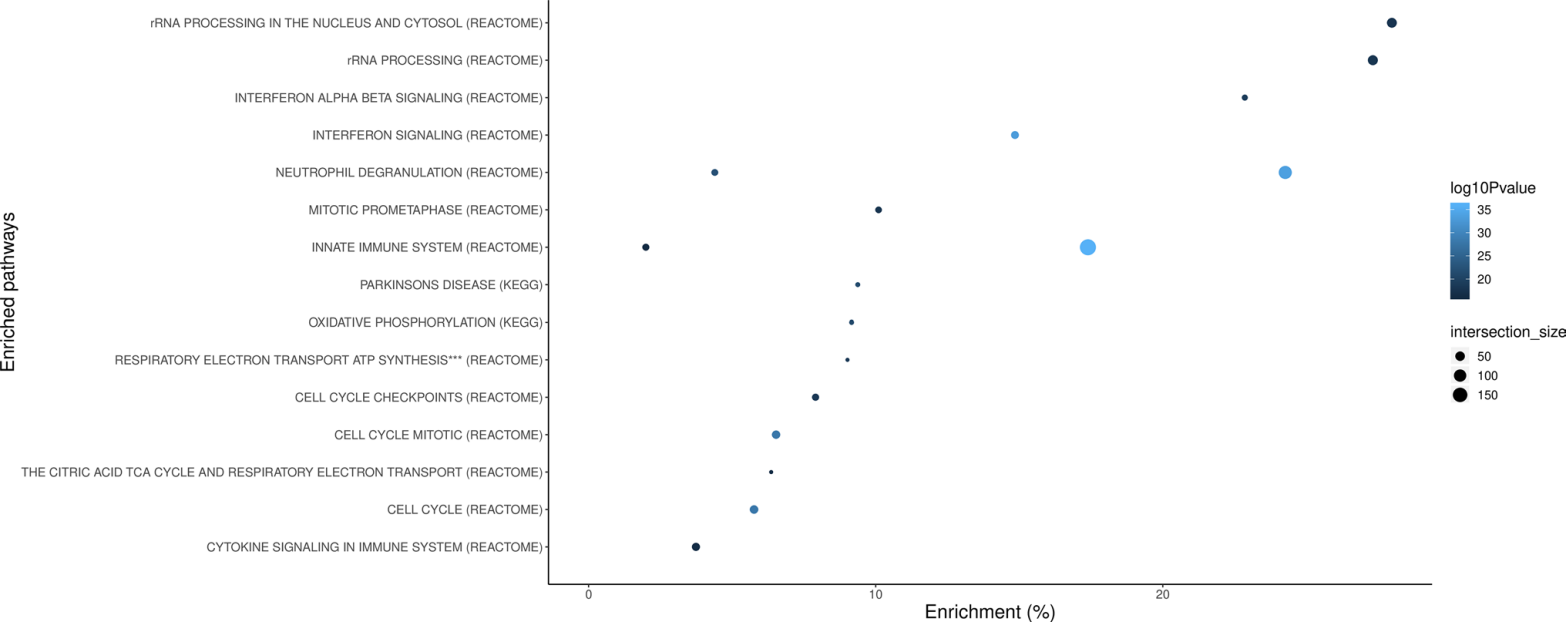
Functional enrichment analyses of transcriptomic community maps. The x-axis represents the gene set enrichment (%) based on the community map gene lists. Intersection size denotes the number of input genes within an enrichment category. Blue color indicates the adjusted association *p*-values on a − log_10_ scale. ***By chemiosmotic coupling and heat production by uncoupling proteins

Using Louvain community detection, we generated transcriptomic networks among PD cases. We identified 54 de novo expression communities (Supplementary Table 8, Supplementary Fig. 4, online resource). Overall, the communities generated were relatively robust, with a modularity score of 0.523 (modularity ranges from − 1 to 1, with closer to 1 suggesting stronger connectivity between network members). The 54 network communities were found to be enriched via hypergeometric tests after Bonferroni correction for processes relating to immune system response, ribosome RNA processing to the nucleus and cytosol, cell cycle, oxidative stress, and mitochondrial impairment (Fig. 3, Supplementary Table 9, online resource).

### Dopaminergic neurons, serotonergic neurons and neural progenitors play a role on PD etiology

We used single-cell RNA sequencing data from 24 different brain cell types [25, 34]. For each of those cell types, genes were clustered into 10 gene sets according to the level of expression specificity, ranging from 0 to 1 (0 means that a gene is not expressed at all and 1 means the gene expression is highly specific for that cell type). Then, PRS was calculated per quintile of specificity within cells. Increased PRS *R*^2^, consistent with increased cell expression specificity, was observed for embryonic dopaminergic neurons, serotonergic neurons, hypothalamic GABAergic neurons, and neural progenitors at *P* < 0.05 in both the *training* and *replication phases* (Supplementary Table 10, online resource).

### Mendelian randomization prioritizes pathways and genes based on their functional consequence

We aimed at nominating genes within significant gene-sets contributing to PD etiology by assessing changes in expression across blood and brain. Out of the 46 gene-sets of interest, 7 showed a significant enrichment of QTLs more than expected by chance in the brain, 1 in substantia nigra and 11 in the blood (Supplementary Table 11, online resource).

SMR revealed functional genomic associations with eQTLs in 201 genes (Supplementary Table 12, online resource) of which 88 were found to be part of the network communities significantly associated with PD in our transcriptome community map (Supplementary Table 13, online resource).

## Discussion

Despite success at uncovering genetic risk factors associated with PD, our understanding of the molecular processes involved in disease is still limited. Using the largest genomic and transcriptomic PD cohorts currently available, our study sought to define both cumulative genetic risk and functional consequences linked to myriad biological pathways in an unbiased and data-directed manner. To our knowledge, there are no previous reports in the PD field where a similar approach has been implemented to explore the contribution of thousands of molecular processes on both the trigger (risk) and the effect (expression changes) in a systematic manner.

Our large-scale PRS analysis identified multiple biological pathways associated with PD risk through common genetic variation. Overall, our results found that molecular processes underlying protein misfolding and aggregation, post-translational protein modification, immune response, membrane and intracellular trafficking, lipid metabolism, synaptic transmission, endosomal–lysosomal dysfunction and apoptosis mediated by initiator and executioner caspases are among the main contributors to PD etiology.

PD heritability remains incompletely deciphered by the genes and variants identified to date [17]. Here, we demonstrate that some of these significant gene-sets contribute to the heritability of PD outside of what is explained by current GWAS [17]. Notably, our genetic analyses provide definitive evidence for the role of several signal transduction mechanisms affecting adaptive and innate immune response, vesicular-mediated transport, and lipid metabolism on the risk for PD even after excluding PD known GWAS loci. The present study suggests that additional targets within these pathways are yet to be identified and prioritizes genes for follow-up functional studies.

A novel aspect of our study is that we nominate pathways whose implication on PD pathology has been poorly studied or debatable before. Our results support the hypothesis that chromatin remodeling and epigenetic mechanisms contribute to the development of PD [13]. An appropriate balance and distribution of active and repressed chromatin is required for proper transcriptional control, maintaining nuclear architecture and genomic stability, as well as regulation of the cell cycle [10]. Dysfunction in the epigenetic machinery has been shown to play a role in the etiology of a number of neurodegenerative and neurodevelopmental disorders either by genetic variation in an epigenetic gene or by changes in DNA methylation or histone modifications [13]. Similarly, our approach supports a role for vitamin metabolism on PD risk. Vitamins are crucial cofactors in the metabolism of carbohydrates, fat, and proteins, and vitamin deficiency has been widely proven to promote oxidative stress and neuroinflammation [16].

Interestingly, some of the nominated gene-sets seem to span the etiological risk spectrum in which both common and rare variation contribute to PD susceptibility. In concordance with previous studies [21], our study identified an increased collective effect of rare lysosomal related variants in PD etiology. Additionally, we found evidence for a burden of rare damaging alleles in a range of specific processes, including neuronal transmission-related pathways and immune response.

The present study represents a significant step forward in our understanding of important connections between genetic factors, functional consequences and PD etiology. We constructed a transcriptome map by clustering de novo pathways relevant to disease pathology. Functional characterization analysis of these expression communities revealed that dysregulation of the immune system and inflammatory response including neutrophil degranulation, interferon alpha beta signaling, and other cytokine-related signaling pathways are key disease processes. Strikingly, when looking at molecular mechanisms significantly associated with PD risk, a cumulative effect of rare loss of function variants was found to be linked to disease through the adaptive immune system pathway. Both inflammation and autoimmune response have been widely studied with regard to PD etiology. Previous genetic studies have identified risk loci spanning key immune-associated genes such as *BST1* (*bone marrow stromal cell antigen 1*), a gene known to play role in neutrophil adhesion and migration, and *HLA* (human leukocyte antigen) [17, 23]. In support of this, it has been reported that α-synuclein-derived fragments act as antigenic epitopes displayed by HLA receptors, where both helper and cytotoxic T-cell responses are present in a high percentage of patients when tested [27].

Our analysis provides compelling evidence that dysregulation in genes that play a pivotal role in mitochondrial homeostasis exists in genetically complex PD. Despite not identifying these pathways as part of the stringent large-scale PRS analysis, our transcriptome community map showed an enrichment for the respiratory electron transport ATP synthesis by chemiosmotic coupling process and mitochondrial oxidative phosphorylation, in concordance with other reports [36]. Among the expression networks to highlight, it should be pointed out an enrichment in cell cycle and cell death machinery related processes and ribosome RNA processing to the nucleus and cytosol.

Our study aimed at pinpointing the specific drivers underlying these significant networks. Focusing on gene-sets linked to PD risk, SMR was applied to prioritize genes whose variation was found to be associated with expression changes linked with PD risk. Interestingly, we managed to replicate 88 of these genes after validating the functional consequence within our transcriptome community map.

Despite genetic efforts, it remains a matter of study in what cell types risk variants are active, which is essential for understanding etiology and experimental modeling. By integrating genetics and single-cell expression data, we found that PD risk is linked to expression specificity patterns in dopaminergic neurons, serotonergic neurons, hypothalamic GABAergic neurons, and neural progenitors, suggesting that these cell types disrupt biological networks that impact PD risk. Although our study failed at replicating specific enrichment patterns for oligodendrocytes and microglia as previously reported using other approaches [20], our results are in concordance with previous literature that applies various methodologies to gain similar conclusions [4].

The strengths of this study include an unbiased effort to link risk variants to biological pathways and characterize the functional consequence. While this study marks major progress in integrating human genetic and functional evidence, much remains to be established. A caveat of this study is that our approach was limited by the canonical gene sets publicly defined that were used for pathway analysis, and the relatively few brain regions studied for cell type analysis, which was based on mice data. We are aware that additional molecular networks and cell types from unsampled regions could contribute to PD. In addition, PRS analyses considered that all the variants conferred risk under the additive model and did not cover regulatory regions adjacent to the up or downstream of the genes or intergenic variants, which may be crucial for the disease. A further limitation of our study is that although we used state-of-the-art methodologies such as SMR to nominate candidate pathways and genes related to PD etiology, QTL datasets and associations are affected by both small sample size and low cis-SNP density. In addition, trans-QTL could not be assessed. Furthermore, our study focused on individuals of European ancestry, given that large sample sizes were required to create this resource. Replication in ancestrally diverse populations would be necessary for future studies. We also assume the limitation that gene redundancy might exist across the tested gene-sets and therefore overrepresentation of certain genes might lead to missing important gene-sets that in turn are associated with PD etiology. We anticipate that substantial collaborative efforts will lead to an improvement in statistical power and accuracy to define gene-sets linked to PD.

In conclusion, our high-throughput and hypothesis-free approach exemplifies a powerful strategy to provide valuable mechanistic insights into PD etiology and pathogenesis. We highlight several promising pathways, cell types, and genes for further functional prioritization, aware that further in-depth investigation will be required to prove a definite link. As part of this study, we created a foundational resource for the PD community that can be applied to other neurodegenerative diseases with complex genetic etiologies (https://pdgenetics.shinyapps.io/pathwaysbrowser/). In future studies, linking specific phenotypic aspects of PD to pathways will constitute a critical effort using large longitudinal cohorts of well clinically characterized PD patients, with the hope of yielding disease-modifying therapeutic targets that are effective across PD subtypes.

## Electronic supplementary material

Below is the link to the electronic supplementary material.

Supplementary material 1 (PDF 8915 kb)
