# Correction to: Large‑scale pathway specific polygenic risk and transcriptomic community network analysis identifies novel functional pathways in Parkinson disease

**DOI:** 10.1007/s00401-021-02309-z

**Published:** 2021-05-04

**Authors:** S. Bandres-Ciga, S. Saez-Atienzar, J. J. Kim, M. B. Makarious, F. Faghri, M. Diez-Fairen, H. Iwaki, H. Leonard, J. Botia, M. Ryten, D. Hernandez, J. R. Gibbs, J. Ding, Z. Gan-Or, A. Noyce, L. Pihlstrom, A. Torkamani, A. R. Soltis, C. L. Dalgard, The American Genome Center, S. W. Scholz, B. J. Traynor, D. Ehrlich, C. R. Scherzer, M. Bookman, M. Cookson, C. Blauwendraat, M. A. Nalls, A. B. Singleton, the International Parkinson Disease Genomics Consortium

**Affiliations:** 1 Molecular Genetics Section, Laboratory of Neurogenetics, National Institute on Aging, National Institutes of Health, Bethesda, MD 20892 USA; 2 Neuromuscular Diseases Research Section, Laboratory of Neurogenetics, National Institute on Aging, National Institutes of Health, Bethesda, MD 20892 USA; 3 Fundació Docència i Recerca Mútua Terrassa and Movement Disorders Unit, Department of Neurology, University Hospital Mútua Terrassa, Terrassa, 08221 Barcelona, Spain; 4 Departamento de Ingeniería de la Información y las Comunicaciones, Universidad de Murcia, Murcia, Spain; 5 Department of Molecular Neuroscience, UCL, Institute of Neurology, London, UK; 6 Department of Neurodegenerative Disease, University College London (UCL) Institute of Neurology, London, UK; 7 Department of Neurology and Neurosurgery, McGill University, Montréal, QC Canada; 8 Montreal Neurological Institute, McGill University, Montréal, QC Canada; 9 Department of Human Genetics, McGill University, Montréal, QC Canada; 10 Preventive Neurology Unit, Wolfson Institute of Preventive Medicine, Queen Mary University of London and Department of Neurology, Royal London Hospital, London, UK; 11 Department of Neurology, Oslo University Hospital, Oslo, Norway; 12 The Scripps Research Institute, La Jolla, CA 92037 USA; 13 Department of Anatomy, Physiology & Genetics, Uniformed Services University of the Health Sciences, Bethesda, MA USA; 14 The American Genome Center, Collaborative Health Initiative Research Program, Uniformed Services University of the Health Sciences, Bethesda, MA USA; 15 Neurodegenerative Diseases Research Unit, National Institute of Neurological Disorders and Stroke, Bethesda, MD 20892 USA; 16 Department of Neurology, Johns Hopkins University Medical Center, Baltimore, MD 21287 USA; 17 Parkinson’s Disease Clinic, Office of the Clinical Director, National Institute of Neurological, Disorders and Stroke, National Institutes of Health, Bethesda, MD 20892 USA; 18 Center for Advanced Parkinson Research, Harvard Medical School, Brigham and Women’s Hospital, Boston, MA 0115 USA; 19 Verily Life Sciences, South San Francisco, CA USA; 20 Cell Biology and Gene Expression Section, Laboratory of Neurogenetics, National Institute on Aging, National Institutes of Health, Bethesda, MA USA; 21 Data Tecnica International, Glen Echo, MD 20812 USA

## Correction to: Acta Neuropathologica (2020) 140:341–358 10.1007/s00401-020-02181-3

The article Large‑scale pathway specific polygenic risk and transcriptomic community network analysis identifies novel functional pathways in Parkinson disease, written by S. Bandres‑Ciga, S. Saez‑Atienzar, J. J. Kim, M. B. Makarious, F. Faghri, M. Diez‑Fairen, H. Iwaki, H. Leonard, J. Botia, M. Ryten, D. Hernandez, J. R. Gibbs, J. Ding, Z. Gan‑Or, A. Noyce, L. Pihlstrom, A. Torkamani, A. R. Soltis, C. L. Dalgard, The American Genome Center, S. W. Scholz, B. J. Traynor, D. Ehrlich, C. R. Scherzer, M. Bookman, M. Cookson, C. Blauwendraat, M. A. Nalls, A. B. Singleton on behalf of the International Parkinson Disease Genomics Consortium, was originally published Online First without Open Access. After publication in volume 140, issue 3, page 341–358 the author decided to opt for Open Choice and to make the article an Open Access publication. Therefore, the copyright of the article has been changed to © The Author(s) 2021 and the article is forthwith distributed under the terms of the “Creative Commons Attribution 4.0 International License, which permits use, sharing, adaptation, distribution and reproduction in any medium or format, as long as you give appropriate credit to the original author(s) and the source, provide a link to the Creative Commons licence, and indicate if changes were made. The images or other third party material in this article are included in the article’s Creative Commons licence, unless indicated otherwise in a credit line to the material. If material is not included in the article’s Creative Commons licence and your intended use is not permitted by statutory regulation or exceeds the permitted use, you will need to obtain permission directly from the copyright holder. To view a copy of this licence, visit http://creativecommons.org/licenses/by/4.0/.”

The original article has been corrected.

