# Supplementary material for: Large-scale pathway specific polygenic risk and transcriptomic community network analysis identifies novel functional pathways in Parkinson disease
Source: Acta Neuropathol. 2020 Jun 29;140(3):341–58. doi: 10.1007/s00401-020-02181-3 (PMC8096770; doi:10.1007/s00401-020-02181-3)
Supplement: Supplementary file 1 — Supplementary material 1 (PDF 8915 kb) [file 401_2020_2181_MOESM1_ESM.pdf]

Supplementary material

Large-scale pathway specific polygenic risk and transcriptomic community network analysis identifies novel functional pathways in Parkinson disease

Bandres-Ciga S[1]*, Saez-Atienzar S[2]*, Kim JJ[1], Makarious MB[1], Faghri F[1], Diez-Fairen M[3], Iwaki H[1], Leonard H[1], Botia J[4,5], Ryten M[6], Hernandez D[1], Gibbs JR[1], Ding J[1], Gan-Or Z[7], Noyce A[8] , Pihlstrom L[9], Torkamani A[10], Soltis AR[12], Dalgard CL[11,12], The American Genome Center, Scholz SW[13,14], Traynor BJ[2,14], Ehrlich D[15], Scherzer CR[16], Bookman M[17], Cookson M[18], Blauwendraat C[1], Nalls MA[1,19], Singleton AB[1], on behalf of the International Parkinson Disease Genomics Consortium.

* Equal contribution

Figure S1. Canonical Pathways associated with PD risk clustered in hierarchies according to genetic redundancy.. 3

Figure S2. Canonical Pathways associated with PD risk clustered in hierarchies according to published literature and public curated databases.. 4

Figure S3. Uniform Manifold Approximation and Projection for Dimension Reduction Analysis... 5

Figure S4. Distribution of transcriptomic community networks according to Louvain cluster detection.. 6

Table S1. Demographic and clinical characteristics of the GWAS cohorts under study.. 7

Table S2. Demographic and clinical characteristics of the WGS cohorts under study.. 8

Table S3. Canonical pathways significantly associated with PD risk in the *discovery phase*.. 9

Table S4. SNPs within 90 risk loci and 1 Mb upstream and downstream from the GWAS signal.. 15

Table S5. Gene prioritization based on pleiotropy across significant gene-sets associated with Parkinson's disease... 264

Table S6. Canonical pathways significantly associated with PD risk through rare variation excluding PD known risk loci +/- 1Mb upstream and downstream 265

Table S7. Gene prioritization per functionality category based on Sequence Kernel Association Test analysis.. 266

Table S8. Genes within expression community maps and modularity ranges.. 334

Table S9. Functional enrichment analysis of network community maps.. 530

Table S10. Cell types significantly associated with PD etiology based on expression specificity enrichment analyses...................................... 531

Table S11. Functional enrichment for expression QTL across pathways significantly associated with PD risk.. 532

Table S12. Functional associations for genes within significant pathways associated with PD risk by SMR 533

Table S13. Genes showing functional inferences through two sample Mendelian randomization and functional consequence in the transcriptome communities.. 651

IPDGC and TAGC Consortia members.. 652

Supplementary Figure 1. Canonical Pathways associated with PD risk clustered in hierarchies according to genetic redundancy

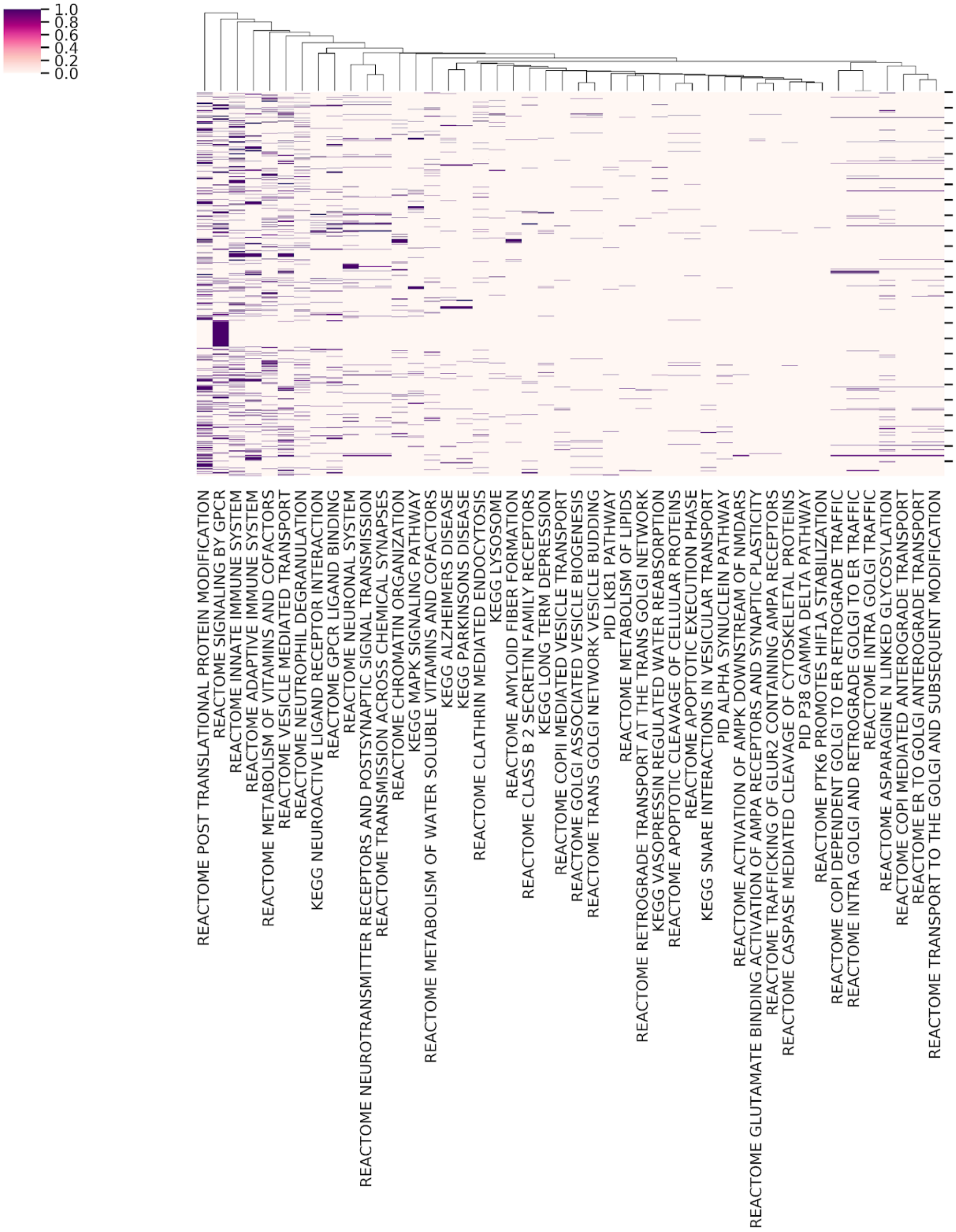

Supplementary Figure 2. Canonical Pathways associated with PD risk clustered in hierarchies according to published literature and public curated databases

Metabolism

Protein metabolism

- Alpha synuclein pathway
- Amyloid fiber formation
- Asparagine N-linked glycosylation
- Post-translational protein modification
- Transport to the Golgi and subsequent modification

Lipid metabolism

Metabolism of vitamins and cofactors

Programmed cell death

- Apoptotic cleavage of cellular proteins
- Apoptotic execution phase
- Caspase mediated cleavage of cytoskeletal proteins

Immune system

- Adaptive immune system
- Innate immune system
- Neutrophil degranulation

Vesicle-mediated transport and membrane trafficking

- Clathrin-mediated endocytosis
- COPI-dependent Golgi-to-ER retrograde traffic
- COPI-mediated anterograde transport
- COPII-mediated vesicle transport
- ER-to-Golgi anterograde transport
- Golgi-associated vesicle biogenesis
- Intra-Golgi and retrograde Golgi-to-ER
- Lysosome
- Retrograde transport at the trans-Golgi network
- SNARE interactions in vesicular transport
- Trans-Golgi network vesicle budding

Signal transduction

- Class B-2 secreting family receptors
- GPCR signaling and ligand binding
- LKB1 pathway
- MAPK signaling pathway
- Neuroactive ligand receptor interaction
- P38 gamma delta pathway
- PTK6 promotes HIF1A stabilization

Neuronal transmission across chemical synapses

- Activation of AMPK downstream of NMDA receptors
- Glutamate binding activation of AMPA receptors and synaptic plasticity
- Neurotransmitter receptors and postsynaptic signal transmission
- Trafficking of GLUR2 containing AMPA receptors
- Transmission across chemical synapses

Miscellaneous

- Chromatin organization
- Alzheimer disease
- Parkinson disease
- Vasopressin-regulated water reabsorption
- Long-term depression

Supplementary Figure 3. Uniform Manifold Approximation and Projection for Dimension Reduction Analysis to explore etiological subtypes of disease

a

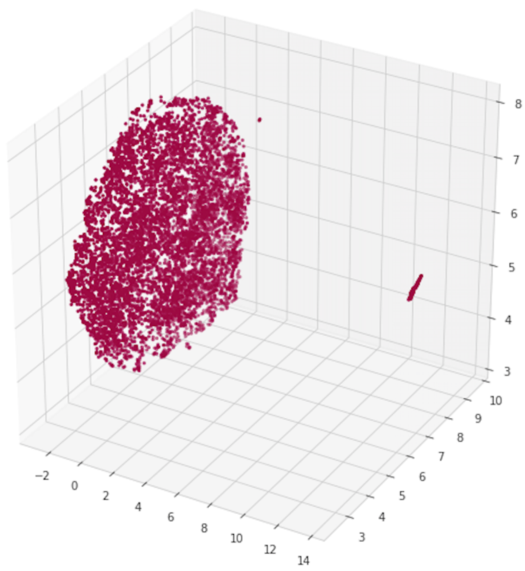

b

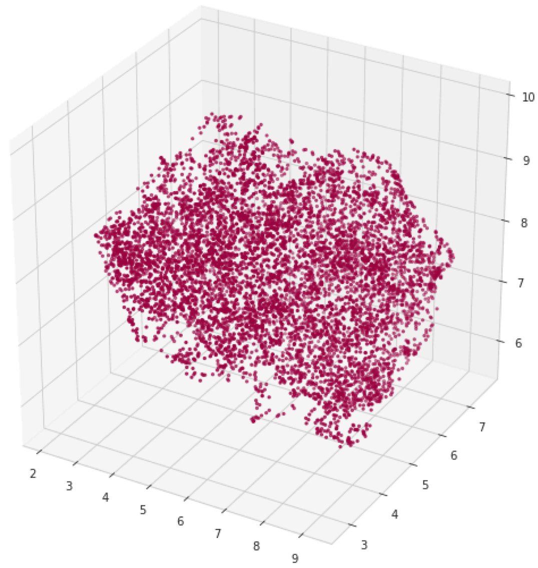

Supplementary Figure 4. Distribution of transcriptomic community networks according to Louvain cluster detection

Colors represent different transcriptomic communities. Circles represent genes within expression networks.

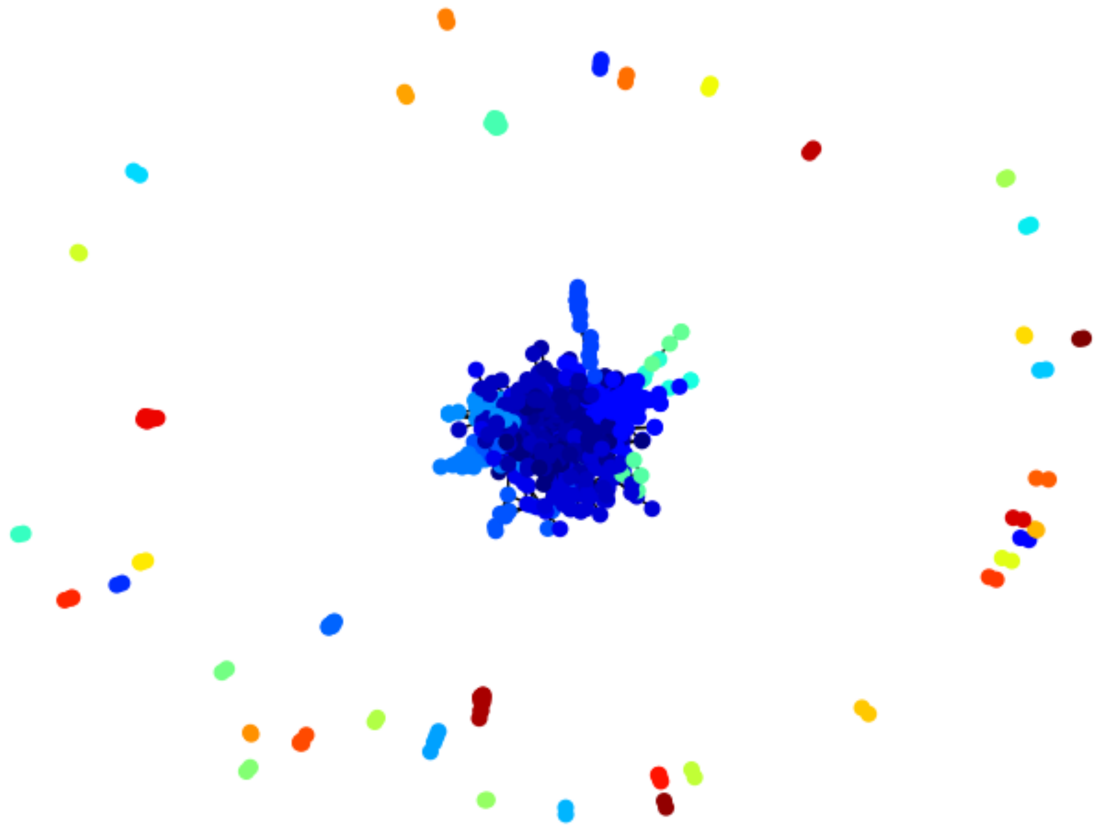

Supplementary Table 1. Demographic and clinical characteristics of the GWAS cohorts under study

Study	Cases (N)	Controls (N)	Total (N)	Female cases (%)	Female control (%)	Case age at onset (mean , SD in years)	Control age at last exam (mean, SD in years)
**Finnish Parkinson's**	386	492	878	45.85	78.9	55.27 (5.64)	92.35 (3.86)
**McGill Parkinson's**	580	905	1485	34.54	48.4	65.71 (9.79)	55.79 (10.69)
**NEUROX-DBGAP**	5429	5814	11243	See PMID: 25064009 for details.			
**Oslo Parkinson's Disease Study**	476	462	938	35.71	42.21	65.32 (9.28)	61.85 (11.06)
**Baylor College of Medicine / University of Maryland**	768	195	963	33.81	69.74	64.83 (10.12)	65.48 (8.31)
**Spanish Parkinson's (IPDGC)**	2108	1331	3439	43.13	54.39	63.92 (12.54)	64.03 (12.59)
**Tubingen Parkinson's Disease cohort (CouragePD)**	658	534	1192	36.04	57.93	59.89 (11.25)	67.48 (8.41)
**UK PDMED (CouragePD)**	1609	5195	6804	32.78	72.67	NA	NA
**Vance (dbGap phs000394)**	619	298	917	27.74	50.84	77.47 (8.40)	81.98 (12.78)
**TOTAL**	12633	15226	27859				

SD, standard deviation; NA, non applicable

Supplementary Table 2. Demographic and clinical characteristics of the WGS cohorts under study

Study	Cases (N)	Controls (N)	Total (N)	Female cases (%)	Female control (%)	Case age at onset (mean , SD in years)	Control age at last exam (mean, SD in years)
**BIOFIND**	91	68	159	35.16	44.11	64.90 (8.14)	65.07 (8.12)
**NABEC**	-	338	338	-	36.68	-	58.98 (15.01)
**NIH LNG PATH**	330	-	330	33.33	-	69.22 (14.94)	-
**PDBP**	798	433	1231	35.83	54.96	62.40 (11.55)	61.92 (10.83)
**NIH PD CLIN**	507	-	507		39.44	55.1 (11.7)	-
**WELLDERLY**	-	1110	1110	-	60.09	-	85.03 (12.63)
**PPMI**	375	176	551	33.86	35.27	63.72 (10.14)	63.79 (10.19)
**UKBEC**	-	105	105	-	23.8	-	68.94 (14.95)
**TOTAL**	2101	2230	4331				

SD: standard deviation

Supplementary Table 3. Canonical pathways significantly associated with PD risk in the *discovery phase*

Gene set	PRS R2	Beta	SE
POST TRANSLATIONAL PROTEIN MODIFICATION (REACTOME)	0.007432	0.2568	0.0166
NABA MATRISOME	0.004983	0.2099	0.0165
VESICLE MEDIATED TRANSPORT (REACTOME)	0.004379	0.1964	0.0164
DISEASE (REACTOME)	0.004366	0.1962	0.0164
NEURONAL SYSTEM (REACTOME)	0.004368	0.1961	0.0164
TRANSPORT OF SMALL MOLECULES (REACTOME)	0.004330	0.1954	0.0164
DEVELOPMENTAL BIOLOGY (REACTOME)	0.004182	0.1924	0.0164
ADAPTIVE IMMUNE SYSTEM (REACTOME)	0.003954	0.1867	0.0164
GENE EXPRESSION TRANSCRIPTION (REACTOME)	0.003896	0.1852	0.0164
INNATE IMMUNE SYSTEM (REACTOME)	0.003657	0.1790	0.0164
GENERIC TRANSCRIPTION PATHWAY (REACTOME)	0.003560	0.1769	0.0164
AMYLOID FIBER FORMATION (REACTOME)	0.003083	0.1646	0.0164
TRANSMISSION ACROSS CHEMICAL SYNAPSES (REACTOME)	0.003045	0.1634	0.0163
PARKINSONS DISEASE (KEGG)	0.002935	0.1635	0.0168
NABA MATRISOME ASSOCIATED	0.002879	0.1591	0.0164
METABOLISM OF LIPIDS (REACTOME)	0.002864	0.1585	0.0163
AXON GUIDANCE (REACTOME)	0.002825	0.1577	0.0164
CLASS I MHC MEDIATED ANTIGEN PROCESSING PRESENTATION (REACTOME)	0.002725	0.1546	0.0163
HEMOSTASIS (REACTOME)	0.002713	0.1545	0.0164
SIGNALING BY GPCR (REACTOME)	0.002667	0.1534	0.0164
CYTOKINE SIGNALING IN IMMUNE SYSTEM (REACTOME)	0.002522	0.1484	0.0163
ANTIGEN PROCESSING: UBIQUITINATION PROTEASOME DEGRADATION (REACTOME)	0.002467	0.1469	0.0163
SIGNALING BY RECEPTOR TYROSINE KINASES (REACTOME)	0.002426	0.1457	0.0163
PTK6 PROMOTES HIF1A STABILIZATION (REACTOME)	0.002543	0.1580	0.0178
NEUROTRANSMITTER RECEPTORS AND POSTSYNAPTIC SIGNAL TRANSMISSION (REACTOME)	0.002338	0.1430	0.0163
ALZHEIMERS DISEASE (KEGG)	0.002263	0.1410	0.0163
EXTRACELLULAR MATRIX ORGANIZATION (REACTOME)	0.002249	0.1403	0.0163
SLC MEDIATED TRANSMEMBRANE TRANSPORT (REACTOME)	0.002203	0.1392	0.0163
SIGNALING BY INTERLEUKINS (REACTOME)	0.002201	0.1385	0.0163
PATHWAYS IN CANCER (KEGG)	0.002155	0.1373	0.0163
CASPASE MEDIATED CLEAVAGE OF CYTOSKELETAL PROTEINS (REACTOME)	0.002125	0.1372	0.0164
NABA CORE MATRISOME	0.002092	0.1355	0.0163
POST TRANSLATIONAL MODIFICATION: SYNTHESIS OF GPI ANCHORED PROTEINS (REACTOME)	0.002087	0.1358	0.0164
ASPARAGINE N LINKED GLYCOSYLATION (REACTOME)	0.002024	0.1331	0.0163
P38 GAMMA DELTA PATHWAY (PID)	0.002019	0.1334	0.0164
NEUROACTIVE LIGAND RECEPTOR INTERACTION (KEGG)	0.001980	0.1316	0.0163
NEUTROPHIL DEGRANULATION (REACTOME)	0.001980	0.1316	0.0163
GLUTAMATE BINDING ACTIVATION OF AMPA RECEPTORS AND SYNAPTIC PLASTICITY (REACTOME)	0.001954	0.1308	0.0163
CELL CYCLE (REACTOME)	0.001927	0.1298	0.0163
SIGNALING BY NON RECEPTOR TYROSINE KINASES (REACTOME)	0.001886	0.1292	0.0164
APOPTOTIC CLEAVAGE OF CELLULAR PROTEINS (REACTOME)	0.001876	0.1289	0.0164
ENDOCYTOSIS (KEGG)	0.001868	0.1280	0.0163
INTRA GOLGI AND RETROGRADE GOLGI TO ER TRAFFIC (REACTOME)	0.001864	0.1275	0.0163
DNA REPAIR (REACTOME)	0.001793	0.1251	0.0163
ION CHANNEL TRANSPORT (REACTOME)	0.001766	0.1243	0.0163
APOPTOTIC EXECUTION PHASE (REACTOME)	0.001753	0.1243	0.0164
TRANSPORT TO THE GOLGI AND SUBSEQUENT MODIFICATION (REACTOME)	0.001749	0.1238	0.0163

PROGRAMMED CELL DEATH (REACTOME)	0.001710	0.1231	0.0164
GOLGI TO ER RETROGRADE TRANSPORT (REACTOME)	0.001699	0.1216	0.0162
DISEASES OF SIGNAL TRANSDUCTION (REACTOME)	0.001699	0.1220	0.0163
NABA ECM GLYCOPROTEINS	0.001703	0.1224	0.0164
GPCR LIGAND BINDING (REACTOME)	0.001683	0.1215	0.0163
LONG TERM DEPRESSION (KEGG)	0.001661	0.1204	0.0163
CALCIUM SIGNALING PATHWAY (KEGG)	0.001655	0.1204	0.0163
COPI DEPENDENT GOLGI TO ER RETROGRADE TRAFFIC (REACTOME)	0.001621	0.1187	0.0162
SIGNALING BY RHO GTPASES (REACTOME)	0.001616	0.1188	0.0163
TIGHT JUNCTION (KEGG)	0.001610	0.1188	0.0163
TRAFFICKING OF GLUR2 CONTAINING AMPA RECEPTORS (REACTOME)	0.001600	0.1184	0.0163
OTHER INTERLEUKIN SIGNALING (REACTOME)	0.001559	0.1166	0.0162
TRANS GOLGI NETWORK VESICLE BUDDING (REACTOME)	0.001521	0.1148	0.0162
ALPHA SYNUCLEIN PATHWAY (PID)	0.001521	0.1157	0.0164
CLASS B 2 SECRETIN FAMILY RECEPTORS (REACTOME)	0.001501	0.1146	0.0163
FOCAL ADHESION (KEGG)	0.001470	0.1135	0.0163
GOLGI ASSOCIATED VESICLE BIOGENESIS (REACTOME)	0.001443	0.1118	0.0162
AXON GUIDANCE (KEGG)	0.001440	0.1123	0.0163
CELL SURFACE INTERACTIONS AT THE VASCULAR WALL (REACTOME)	0.001408	0.1107	0.0162
NABA ECM REGULATORS	0.001340	0.1082	0.0163
CLATHRIN MEDIATED ENDOCYTOSIS (REACTOME)	0.001336	0.1079	0.0162
SIGNALING BY NOTCH (REACTOME)	0.001324	0.1078	0.0163
CELL CYCLE CHECKPOINTS (REACTOME)	0.001324	0.1073	0.0162
INFECTIOUS DISEASE (REACTOME)	0.001299	0.1064	0.0162
COPII MEDIATED VESICLE TRANSPORT (REACTOME)	0.001303	0.1070	0.0163
ACTIVATION OF NMDA RECEPTORS AND POSTSYNAPTIC EVENTS (REACTOME)	0.001297	0.1063	0.0162
DISEASES OF GLYCOSYLATION (REACTOME)	0.001286	0.1060	0.0163
G ALPHA S SIGNALLING EVENTS (REACTOME)	0.001282	0.1062	0.0163
MAPK SIGNALING PATHWAY (KEGG)	0.001276	0.1058	0.0163
INTRA GOLGI TRAFFIC (REACTOME)	0.001260	0.1049	0.0163
VASOPRESSIN REGULATED WATER REABSORPTION (KEGG)	0.001248	0.1045	0.0163
TRANSPORT OF BILE SALTS AND ORGANIC ACIDS METAL IONS AND AMINE COMPOUNDS (REACTOME)	0.001243	0.1049	0.0164
RHO GTPASE EFFECTORS (REACTOME)	0.001241	0.1043	0.0163
TRANSCRIPTIONAL REGULATION BY RUNX2 (REACTOME)	0.001240	0.1041	0.0163
CELL CYCLE MITOTIC (REACTOME)	0.001218	0.1032	0.0163
PHOSPHOLIMETABOLISM (REACTOME)	0.001211	0.1030	0.0163
CHRONIC MYELOID LEUKEMIA (KEGG)	0.001202	0.1026	0.0163
O LINKED GLYCOSYLATION (REACTOME)	0.001179	0.1013	0.0162
SIGNALING BY ERBB2 (REACTOME)	0.001174	0.1011	0.0162
SIGNALING BY WNT (REACTOME)	0.001174	0.1010	0.0162
PLATELET ACTIVATION SIGNALING AND AGGREGATION (REACTOME)	0.001173	0.1013	0.0163
NATURAL KILLER CELL MEDIATED CYTOTOXICITY (KEGG)	0.001172	0.1011	0.0162
ESR MEDIATED SIGNALING (REACTOME)	0.001161	0.1006	0.0162
ER TO GOLGI ANTEROGRADE TRANSPORT (REACTOME)	0.001162	0.1008	0.0163
SIGNALING BY MET (REACTOME)	0.001156	0.1006	0.0163
CMYB PATHWAY (PID)	0.001151	0.1001	0.0162
DEUBIQUITINATION (REACTOME)	0.001142	0.0999	0.0163
UBIQUITIN MEDIATED PROTEOLYSIS (KEGG)	0.001141	0.0999	0.0163
LKB1 PATHWAY (PID)	0.001121	0.0988	0.0162
MHC CLASS II ANTIGEN PRESENTATION (REACTOME)	0.001116	0.0992	0.0163

NEDDYLATION (REACTOME)	0.001116	0.0987	0.0163
G ALPHA Q SIGNALLING EVENTS (REACTOME)	0.001112	0.0986	0.0163
MAPK FAMILY SIGNALING CASCADES (REACTOME)	0.001082	0.0970	0.0162
REGULATION OF ACTIN CYTOSKELETON (KEGG)	0.001080	0.0972	0.0163
RET SIGNALING (REACTOME)	0.001071	0.0964	0.0162
METABOLISM OF VITAMINS AND COFACTORS (REACTOME)	0.001070	0.0965	0.0162
SIGNALING BY NUCLEAR RECEPTORS (REACTOME)	0.001049	0.0957	0.0162
WNT SIGNALING PATHWAY (KEGG)	0.001036	0.0950	0.0162
INOSITOL PHOSPHATE METABOLISM (REACTOME)	0.001028	0.0948	0.0163
ACTIVATION OF AMPK DOWNSTREAM OF NMDARS (REACTOME)	0.001003	0.0936	0.0162
STIMULI SENSING CHANNELS (REACTOME)	0.000990	0.0929	0.0162
SMALL CELL LUNG CANCER (KEGG)	0.000989	0.0928	0.0162
METABOLISM OF AMINO ACIDS AND DERIVATIVES (REACTOME)	0.000983	0.0930	0.0163
INOSITOL PHOSPHATE METABOLISM (KEGG)	0.000975	0.0921	0.0162
CONSTITUTIVE SIGNALING BY ABERRANT PI3K IN CANCER (REACTOME)	0.000963	0.0915	0.0162
SIGNALING BY EGFR (REACTOME)	0.000944	0.0906	0.0162
INTRACELLULAR SIGNALING BY SECOND MESSENGERS (REACTOME)	0.000931	0.0902	0.0163
ERBB1 INTERNALIZATION PATHWAY (PID)	0.000927	0.0899	0.0162
METABOLISM OF CARBOHYDRATES (REACTOME)	0.000918	0.0894	0.0162
MUSCLE CONTRACTION (REACTOME)	0.000916	0.0894	0.0162
DISEASES ASSOCIATED WITH O GLYCOSYLATION OF PROTEINS (REACTOME)	0.000915	0.0894	0.0163
NABA SECRETED FACTORS	0.000902	0.0886	0.0162
PHOSPHATIDYLINOSITOL SIGNALING SYSTEM (KEGG)	0.000898	0.0884	0.0162
METABOLISM OF WATER SOLUBLE VITAMINS AND COFACTORS (REACTOME)	0.000892	0.0881	0.0162
LYSOSOME (KEGG)	0.000894	0.0890	0.0164
ERBB SIGNALING PATHWAY (KEGG)	0.000892	0.0881	0.0162
REGULATION OF INSULIN LIKE GROWTH FACTOR IGF TRANSPORT AND UPTAKE BY INSULIN LIKE GROWTH FACTOR BINDING PROTEINS IGFBPS	0.000889	0.0880	0.0162
LYSINE DEGRADATION (KEGG)	0.000880	0.0875	0.0162
GLYCOSAMINOGLYCAN METABOLISM (REACTOME)	0.000875	0.0873	0.0162
RUNX1 REGULATES TRANSCRIPTION OF GENES INVOLVED IN DIFFERENTIATION OF HSCS (REACTOME)	0.000872	0.0875	0.0163
CELL ADHESION MOLECULES CAMS (KEGG)	0.000870	0.0872	0.0162
POTASSIUM CHANNELS (REACTOME)	0.000871	0.0872	0.0163
NEGATIVE REGULATION OF THE PI3K AKT NETWORK (REACTOME)	0.000868	0.0870	0.0162
NCADHERIN PATHWAY (PID)	0.000866	0.0869	0.0162
NON SMALL CELL LUNG CANCER (KEGG)	0.000865	0.0869	0.0162
GLYCOSYLPHOSPHATIDYLINOSITOL GPI ANCHOR BIOSYNTHESIS	0.000856	0.0867	0.0163
DNA DAMAGE BYPASS (REACTOME)	0.000852	0.0864	0.0163
REELIN PATHWAY (PID)	0.000849	0.0859	0.0162
EPHA MEDIATED GROWTH CONE COLLAPSE (REACTOME)	0.000847	0.0857	0.0162
FACTORS INVOLVED IN MEGAKARYOCYTE DEVELOPMENT AND PLATELET PRODUCTION (REACTOME)	0.000849	0.0861	0.0163
LISTERIA MONOCYTOGENES ENTRY INTO HOST CELLS (REACTOME)	0.000836	0.0854	0.0162
RETROGRADE TRANSPORT AT THE TRANS GOLGI NETWORK (REACTOME)	0.000834	0.0856	0.0163
DUAL INCISION IN TC NER (REACTOME)	0.000822	0.0848	0.0163
GLIOMA (KEGG)	0.000821	0.0846	0.0162
ARF6 PATHWAY (PID)	0.000817	0.0844	0.0162
SPHINGOLIMETABOLISM (KEGG)	0.000822	0.0856	0.0165
COLLAGEN FORMATION (REACTOME)	0.000810	0.0840	0.0162
TCR PATHWAY (PID)	0.000801	0.0836	0.0162
SNARE INTERACTIONS IN VESICULAR TRANSPORT (KEGG)	0.000800	0.0834	0.0162
PROTEIN PROTEIN INTERACTIONS AT SYNAPSES (REACTOME)	0.000794	0.0831	0.0162

COPI MEDIATED ANTEROGRADE TRANSPORT (REACTOME)	0.000793	0.0832	0.0162
ESTROGEN DEPENDENT GENE EXPRESSION (REACTOME)	0.000791	0.0830	0.0162
NEGATIVE REGULATION OF MET ACTIVITY (REACTOME)	0.000791	0.0830	0.0162
NUCLEOTIDE EXCISION REPAIR (REACTOME)	0.000791	0.0831	0.0162
C TYPE LECTIN RECEPTORS CLRS (REACTOME)	0.000789	0.0829	0.0162
L1CAM INTERACTIONS (REACTOME)	0.000789	0.0829	0.0162
SPHINGOLIMETABOLISM (REACTOME)	0.000789	0.0835	0.0163
PI3K AKT SIGNALING IN CANCER (REACTOME)	0.000788	0.0828	0.0162
DEGRADATION OF THE EXTRACELLULAR MATRIX (REACTOME)	0.000783	0.0827	0.0162
P53 DOWNSTREAM PATHWAY (PID)	0.000782	0.0824	0.0162
RECOGNITION OF DNA DAMAGE BY PCNA CONTAINING REPLICATION COMPLEX (REACTOME)	0.000783	0.0829	0.0163
PEPTIDE HORMONE METABOLISM (REACTOME)	0.000781	0.0824	0.0162
SHC1 EVENTS IN ERBB2 SIGNALING (REACTOME)	0.000778	0.0822	0.0162
SYNTHESIS OF IP3 AND IP4 IN THE CYTOSOL (REACTOME)	0.000776	0.0822	0.0162
DNA REPLICATION (REACTOME)	0.000776	0.0823	0.0162
HEDGEHOG SIGNALING PATHWAY (KEGG)	0.000773	0.0819	0.0162
ECADHERIN KERATINOCYTE PATHWAY	0.000769	0.0818	0.0162
MITOTIC METAPHASE AND ANAPHASE (REACTOME)	0.000769	0.0818	0.0162
CELLULAR RESPONSES TO STRESS (REACTOME)	0.000763	0.0817	0.0163
PURINE METABOLISM (KEGG)	0.000762	0.0817	0.0163
RUNX1 REGULATES ESTROGEN RECEPTOR MEDIATED TRANSCRIPTION (REACTOME)	0.000761	0.0815	0.0162
CELL CELL COMMUNICATION (REACTOME)	0.000756	0.0813	0.0162
MITOTIC SPINDLE CHECKPOINT (REACTOME)	0.000754	0.0810	0.0162
REGULATION OF MITOTIC CELL CYCLE (REACTOME)	0.000752	0.0810	0.0162
PDGFRB PATHWAY (PID)	0.000750	0.0810	0.0163
MET PATHWAY (PID)	0.000749	0.0811	0.0163
TXA2PATHWAY (PID)	0.000742	0.0806	0.0163
CELLULAR RESPONSES TO EXTERNAL STIMULI (REACTOME)	0.000741	0.0805	0.0162
TRANSCRIPTION COUPLED NUCLEOTIDE EXCISION REPAIR TC NER (REACTOME)	0.000741	0.0805	0.0163
DNA DOUBLE STRAND BREAK REPAIR (REACTOME)	0.000738	0.0800	0.0162
ION TRANSPORT BY P TYPE ATPASES (REACTOME)	0.000738	0.0803	0.0163
SEMA4D IN SEMAPHORIN SIGNALING (REACTOME)	0.000737	0.0801	0.0162
O GLYCOSYLATION OF TSR DOMAIN CONTAINING PROTEINS (REACTOME)	0.000736	0.0801	0.0162
M PHASE (REACTOME)	0.000735	0.0803	0.0163
T CELL RECEPTOR SIGNALING PATHWAY (KEGG)	0.000735	0.0801	0.0162
NFAT 3PATHWAY (PID)	0.000732	0.0800	0.0163
ER NONGENOMIC PATHWAY (PID)	0.000732	0.0799	0.0162
RUNX1 REGULATES TRANSCRIPTION OF GENES INVOLVED IN BCR SIGNALING (REACTOME)	0.000730	0.0798	0.0162
SIG PIP3 SIGNALING IN CARDIAC MYOCTES	0.000730	0.0798	0.0162
METABOLISM OF NUCLEOTIDES (REACTOME)	0.000728	0.0795	0.0162
BIOLOGICAL OXIDATIONS (REACTOME)	0.000727	0.0796	0.0162
DISORDERS OF TRANSMEMBRANE TRANSPORTERS (REACTOME)	0.000727	0.0795	0.0162
VOLTAGE GATED POTASSIUM CHANNELS (REACTOME)	0.000728	0.0797	0.0162
INLB MEDIATED ENTRY OF LISTERIA MONOCYTOGENES INTO HOST CELL (REACTOME)	0.000721	0.0792	0.0162
TRANSCRIPTIONAL REGULATION BY RUNX3 (REACTOME)	0.000719	0.0792	0.0162
BETA CATENIN INDEPENDENT WNT SIGNALING (REACTOME)	0.000718	0.0790	0.0162
GLYCEROPHOSPHOLIBIOSYNTHESIS (REACTOME)	0.000718	0.0792	0.0162
SHH PATHWAY (BIOCARTA)	0.000715	0.0788	0.0162
AMB2 NEUTROPHILS PATHWAY (PID)	0.000711	0.0787	0.0162
NEUROTROPHIN SIGNALING PATHWAY (KEGG)	0.000708	0.0785	0.0162

TRANSCRIPTIONAL REGULATION BY RUNX1 (REACTOME)	0.000705	0.0783	0.0162
CLASS A 1 RHODOPSIN LIKE RECEPTORS (REACTOME)	0.000702	0.0783	0.0162
LIS1 PATHWAY (BIOCARTA)	0.000688	0.0771	0.0161
SIGNALING BY NTRKS (REACTOME)	0.000686	0.0774	0.0162
SIGNALING BY ERBB4 (REACTOME)	0.000683	0.0772	0.0162
PCP CE PATHWAY (REACTOME)	0.000682	0.0770	0.0162
AP1 PATHWAY (PID)	0.000681	0.0772	0.0162
CHROMATIN ORGANIZATION (REACTOME)	0.000680	0.0773	0.0163
EPH EPHRIN SIGNALING (REACTOME)	0.000679	0.0770	0.0163
METABOLISM OF RNA (REACTOME)	0.000676	0.0768	0.0162
MELANOMA (KEGG)	0.000675	0.0767	0.0162
UCH PROTEINASES (REACTOME)	0.000671	0.0767	0.0163
FCGAMMA RECEPTOR FCGR DEPENDENT PHAGOCYTOSIS (REACTOME)	0.000659	0.0761	0.0163
TRANSCRIPTIONAL REGULATION BY TP53 (REACTOME)	0.000655	0.0754	0.0162
LONG TERM POTENTIATION (KEGG)	0.000655	0.0756	0.0162
IRON UPTAKE AND TRANSPORT (REACTOME)	0.000654	0.0757	0.0163
TRANSLATION (REACTOME)	0.000651	0.0755	0.0163
RHOA PATHWAY (PID)	0.000650	0.0755	0.0163
NABA ECM AFFILIATED	0.000647	0.0751	0.0162
SEMAPHORIN INTERACTIONS (REACTOME)	0.000647	0.0751	0.0162
FC EPSILON RECEPTOR FCERI SIGNALING (REACTOME)	0.000645	0.0751	0.0162
DUAL INCISION IN GG NER (REACTOME)	0.000646	0.0753	0.0163
S PHASE (REACTOME)	0.000644	0.0749	0.0162
PROTEIN REPAIR (REACTOME)	0.000643	0.0753	0.0163
LEUKOCYTE TRANSENDOTHELIAL MIGRATION (KEGG)	0.000634	0.0742	0.0162
EGFR TRANSACTIVATION BY GASTRIN (REACTOME)	0.000634	0.0746	0.0163
AJDISS 2PATHWAY (PID)	0.000629	0.0743	0.0163
ACUTE MYELOID LEUKEMIA (KEGG)	0.000630	0.0742	0.0162
TNFR2 NON CANONICAL NF KB PATHWAY (REACTOME)	0.000627	0.0740	0.0162
A6B1 A6B4 INTEGRIN PATHWAY	0.000624	0.0737	0.0162
HUNTINGTONS DISEASE (KEGG)	0.000621	0.0736	0.0162
BASAL CELL CARCINOMA (KEGG)	0.000620	0.0734	0.0162
G ALPHA I SIGNALLING EVENTS (REACTOME)	0.000621	0.0736	0.0162
EGFR DOWNREGULATION (REACTOME)	0.000617	0.0733	0.0162
ATF2 PATHWAY (PID)	0.000616	0.0733	0.0162
MET ACTIVATES PTK2 SIGNALING (REACTOME)	0.000614	0.0732	0.0162
TRANSCRIPTIONAL REGULATION OF GRANULOPOIESIS (REACTOME)	0.000613	0.0732	0.0162
NOTCH PATHWAY (PID)	0.000612	0.0730	0.0162
PKMTS METHYLATE HISTONE LYSINES (REACTOME)	0.000611	0.0731	0.0162
TRANSCRIPTIONAL REGULATION BY MECP2 (REACTOME)	0.000611	0.0729	0.0162
FATTY ACYL COA BIOSYNTHESIS (REACTOME)	0.000609	0.0735	0.0164
EPITHELIAL CELL SIGNALING IN HELICOBACTER PYLORI INFECTION (KEGG)	0.000607	0.0726	0.0162
EPHA FWDPATHWAY (PID)	0.000605	0.0726	0.0162
HIV INFECTION (REACTOME)	0.000605	0.0726	0.0162
ECADHERIN STABILIZATION PATHWAY (PID)	0.000605	0.0729	0.0163
UNFOLDED PROTEIN RESPONSE UPR (REACTOME)	0.000606	0.0728	0.0163
GRB2 EVENTS IN ERBB2 SIGNALING (REACTOME)	0.000597	0.0720	0.0162
PI3KCI PATHWAY (PID)	0.000595	0.0719	0.0162
REGULATION OF INSULIN SECRETION (REACTOME)	0.000596	0.0721	0.0162
PHASE I FUNCTIONALIZATION OF COMPOUNDS (REACTOME)	0.000594	0.0718	0.0162

REGULATION OF RUNX2 EXPRESSION AND ACTIVITY (REACTOME)	0.000595	0.0720	0.0162
SIGNALING BY THE B CELL RECEPTOR BCR (REACTOME)	0.000590	0.0717	0.0162
RUNX1 REGULATES TRANSCRIPTION OF GENES INVOLVED IN WNT SIGNALING (REACTOME)	0.000588	0.0716	0.0162
PI3K EVENTS IN ERBB2 SIGNALING (REACTOME)	0.000584	0.0713	0.0162
O GLYCAN BIOSYNTHESIS (KEGG)	0.000581	0.0710	0.0162
HEPARAN SULFATE HEPARIN HS GAG METABOLISM (REACTOME)	0.000579	0.0709	0.0162
CDK MEDIATED PHOSPHORYLATION AND REMOVAL OF CDC6 (REACTOME)	0.000578	0.0710	0.0162
APC C:CDH1 MEDIATED DEGRADATION OF CDC20 AND OTHER APC C:CDH1 TARGETED PROTEINS IN LATE MITOSIS EARLY G1 (REACTOME)	0.000578	0.0710	0.0162
GRANULOCYTES PATHWAY (BIOCARTA)	0.000577	0.0708	0.0162
ARGININE AND PROLINE METABOLISM (KEGG)	0.000579	0.0714	0.0163
CARDIAC CONDUCTION (REACTOME)	0.000575	0.0708	0.0162
ECM RECEPTOR INTERACTION (KEGG)	0.000573	0.0709	0.0163
GASTRIN CREB SIGNALLING PATHWAY VIA PKC AND MAPK (REACTOME)	0.000573	0.0709	0.0163
LGI ADAM INTERACTIONS (REACTOME)	0.000570	0.0704	0.0162
INTERLEUKIN 4 AND INTERLEUKIN 13 SIGNALING (REACTOME)	0.000567	0.0702	0.0162
HOMOLOGY DIRECTED REPAIR (REACTOME)	0.000566	0.0700	0.0162
MTOR 4PATHWAY (PID)	0.000566	0.0704	0.0163
AUTOIMMUNE THYROID DISEASE (KEGG)	0.000560	0.0727	0.0169
ACTIVATION OF APC C AND APC C:CDC20 MEDIATED DEGRADATION OF MITOTIC PROTEINS (REACTOME)	0.000557	0.0697	0.0162
HOST INTERACTIONS OF HIV FACTORS (REACTOME)	0.000557	0.0698	0.0163
ERBB4 PATHWAY (PID)	0.000556	0.0696	0.0162
SIGNALING BY PDGF (REACTOME)	0.000555	0.0696	0.0162
RHO GTPASE CYCLE (REACTOME)	0.000554	0.0694	0.0162
RUNX1 REGULATES EXPRESSION OF COMPONENTS OF TIGHT JUNCTIONS (REACTOME)	0.000552	0.0695	0.0162
HER2 PATHWAY (BIOCARTA)	0.000552	0.0693	0.0162
TRANSPORT OF INORGANIC CATIONS ANIONS AND AMINO ACIDS OLIGOPEPTIDES (REACTOME)	0.000551	0.0692	0.0162
IRE1ALPHA ACTIVATES CHAPERONES (REACTOME)	0.000550	0.0695	0.0163
INTEGRIN2 PATHWAY (PID)	0.000546	0.0687	0.0162
DOWNREGULATION OF ERBB2 SIGNALING (REACTOME)	0.000546	0.0689	0.0162
ONCOGENIC MAPK SIGNALING (REACTOME)	0.000545	0.0690	0.0162
SHC1 EVENTS IN ERBB4 SIGNALING (REACTOME)	0.000544	0.0687	0.0162
NUCLEAR RECEPTOR TRANSCRIPTION PATHWAY (REACTOME)	0.000542	0.0689	0.0163

PRS R2, variance explained by polygenic risk score; SE, standard error; P, P-value; num SNP, Number of SNPs included in each pathway analysis

Supplementary Table 4. SNPs within 90 risk loci and 1 Mb upstream and downstream from the GWAS signal included per gene-set

a) TRAINING PHASE

SNP	*Gene*	Gene-set
17:7352976	*V1NB1*	BIOCARTA_ACH_PATHWAY
3:48835245	*IP6K2*	BIOCARTA_AGPCR_PATHWAY
3:49561208	*IP6K2*	BIOCARTA_AGR_PATHWAY
6:112494762	*FYN*	BIOCARTA_AGR_PATHWAY
6:112531401	*FYN*	BIOCARTA_AGR_PATHWAY
6:112561154	*FYN*	BIOCARTA_AGR_PATHWAY
6:112561430	*FYN*	BIOCARTA_AGR_PATHWAY
3:48835245	*IP6K2*	BIOCARTA_AKAP13_PATHWAY
3:49406095	*IP6K2*	BIOCARTA_AKAP13_PATHWAY
3:49424231	*IP6K2*	BIOCARTA_AKAP13_PATHWAY
3:49450137	*IP6K2*	BIOCARTA_AKAP13_PATHWAY
3:48835245	*IP6K2*	BIOCARTA_AKAP95_PATHWAY
5:133558885	*C5orf24*	BIOCARTA_AKAP95_PATHWAY
3:48835245	*IP6K2*	BIOCARTA_AKAPCENTROSOME_PATHWAY
3:49406095	*IP6K2*	BIOCARTA_AKAPCENTROSOME_PATHWAY
3:49424231	*IP6K2*	BIOCARTA_AKAPCENTROSOME_PATHWAY
3:49450137	*IP6K2*	BIOCARTA_AKAPCENTROSOME_PATHWAY
5:133558885	*C5orf24*	BIOCARTA_AKAPCENTROSOME_PATHWAY
5:133558885	*C5orf24*	BIOCARTA_AKT_PATHWAY
8:11558567	*CTSB*	BIOCARTA_ALK_PATHWAY
8:11599269	*CTSB*	BIOCARTA_ALK_PATHWAY
6:31897219	*HLA-DRB5*	BIOCARTA_ALTERNATIVE_PATHWAY
2:168682052	*STK39*	BIOCARTA_AMAN_PATHWAY
8:131145058	*FAM49B*	BIOCARTA_ARAP_PATHWAY
8:131153067	*FAM49B*	BIOCARTA_ARAP_PATHWAY
8:131245821	*FAM49B*	BIOCARTA_ARAP_PATHWAY
8:131328260	*FAM49B*	BIOCARTA_ARAP_PATHWAY
8:131404984	*FAM49B*	BIOCARTA_ARAP_PATHWAY
8:131408202	*FAM49B*	BIOCARTA_ARAP_PATHWAY
8:131455469	*FAM49B*	BIOCARTA_ARAP_PATHWAY
10:104005410	*GBF1*	BIOCARTA_ARAP_PATHWAY
10:104034550	*GBF1*	BIOCARTA_ARAP_PATHWAY
17:76727455	*DNAH17*	BIOCARTA_ARAP_PATHWAY
17:7578671	*V1NB1*	BIOCARTA_ARF_PATHWAY
17:7578671	*V1NB1*	BIOCARTA_ATM_PATHWAY
17:7578671	*V1NB1*	BIOCARTA_ATRBRCA_PATHWAY
3:48835245	*IP6K2*	BIOCARTA_BAD_PATHWAY
6:112137847	*FYN*	BIOCARTA_BIOPEPTIDES_PATHWAY
6:112151452	*FYN*	BIOCARTA_BIOPEPTIDES_PATHWAY
6:112175069	*FYN*	BIOCARTA_BIOPEPTIDES_PATHWAY
16:30510571	*SETD1A*	BIOCARTA_BLYMPHOCYTE_PATHWAY
16:30516492	*SETD1A*	BIOCARTA_BLYMPHOCYTE_PATHWAY
16:30520856	*SETD1A*	BIOCARTA_BLYMPHOCYTE_PATHWAY
17:7578671	*V1NB1*	BIOCARTA_BTG2_PATHWAY
3:49406095	*IP6K2*	BIOCARTA_CARDIACEGF_PATHWAY
3:49424231	*IP6K2*	BIOCARTA_CARDIACEGF_PATHWAY

3:49450137	*IP6K2*	BIOCARTA_CARDIACEGF_PATHWAY
3:48835245	*IP6K2*	BIOCARTA_CARM1_PATHWAY
1:156059276	*GBAP1*	BIOCARTA_CASPASE_PATHWAY
1:226568665	*ITPKB*	BIOCARTA_CASPASE_PATHWAY
3:49406095	*IP6K2*	BIOCARTA_CCR3_PATHWAY
3:49424231	*IP6K2*	BIOCARTA_CCR3_PATHWAY
3:49450137	*IP6K2*	BIOCARTA_CCR3_PATHWAY
17:43390763	*CRHR1_2*	BIOCARTA_CD40_PATHWAY
3:48199695	*IP6K2*	BIOCARTA_CDC25_PATHWAY
3:48214438	*IP6K2*	BIOCARTA_CDC25_PATHWAY
3:49406095	*IP6K2*	BIOCARTA_CDC42RAC_PATHWAY
3:49424231	*IP6K2*	BIOCARTA_CDC42RAC_PATHWAY
3:49450137	*IP6K2*	BIOCARTA_CDC42RAC_PATHWAY
3:48199695	*IP6K2*	BIOCARTA_CELLCYCLE_PATHWAY
3:48214438	*IP6K2*	BIOCARTA_CELLCYCLE_PATHWAY
3:48835245	*IP6K2*	BIOCARTA_CFTR_PATHWAY
1:226568665	*ITPKB*	BIOCARTA_CHEMICAL_PATHWAY
17:7578671	*V1NB1*	BIOCARTA_CHEMICAL_PATHWAY
3:48835245	*IP6K2*	BIOCARTA_CHREBP_PATHWAY
5:133558885	*C5orf24*	BIOCARTA_CHREBP_PATHWAY
3:48835245	*IP6K2*	BIOCARTA_CK1_PATHWAY
5:133558885	*C5orf24*	BIOCARTA_CK1_PATHWAY
6:31895092	*HLA-DRB5*	BIOCARTA_CLASSIC_PATHWAY
6:31897219	*HLA-DRB5*	BIOCARTA_CLASSIC_PATHWAY
6:31895092	*HLA-DRB5*	BIOCARTA_COMP_PATHWAY
6:31897219	*HLA-DRB5*	BIOCARTA_COMP_PATHWAY
3:48835245	*IP6K2*	BIOCARTA_CREB_PATHWAY
3:48835245	*IP6K2*	BIOCARTA_CSK_PATHWAY
5:133558885	*C5orf24*	BIOCARTA_CTCF_PATHWAY
17:7578671	*V1NB1*	BIOCARTA_CTCF_PATHWAY
16:30510571	*SETD1A*	BIOCARTA_CTL_PATHWAY
16:30516492	*SETD1A*	BIOCARTA_CTL_PATHWAY
16:30520856	*SETD1A*	BIOCARTA_CTL_PATHWAY
1:226568665	*ITPKB*	BIOCARTA_D4GDI_PATHWAY
16:31370452	*SETD1A*	BIOCARTA_DC_PATHWAY
1:156059276	*GBAP1*	BIOCARTA_DEATH_PATHWAY
17:43390763	*CRHR1_2*	BIOCARTA_DEATH_PATHWAY
2:95967628	*KCNIP3*	BIOCARTA_DREAM_PATHWAY
3:48835245	*IP6K2*	BIOCARTA_DREAM_PATHWAY
3:49406095	*IP6K2*	BIOCARTA_ECM_PATHWAY
3:49424231	*IP6K2*	BIOCARTA_ECM_PATHWAY
3:49450137	*IP6K2*	BIOCARTA_ECM_PATHWAY
6:112137847	*FYN*	BIOCARTA_ECM_PATHWAY
6:112151452	*FYN*	BIOCARTA_ECM_PATHWAY
6:112175069	*FYN*	BIOCARTA_ECM_PATHWAY
3:49406095	*IP6K2*	BIOCARTA_EDG1_PATHWAY
3:49424231	*IP6K2*	BIOCARTA_EDG1_PATHWAY
3:49450137	*IP6K2*	BIOCARTA_EDG1_PATHWAY
17:7578671	*V1NB1*	BIOCARTA_EFP_PATHWAY
17:7479140	*V1NB1*	BIOCARTA_EIF4_PATHWAY
17:7480325	*V1NB1*	BIOCARTA_EIF4_PATHWAY
17:7479140	*V1NB1*	BIOCARTA_EIF_PATHWAY
17:7480325	*V1NB1*	BIOCARTA_EIF_PATHWAY

6:112137847	*FYN*	BIOCARTA_EPHA4_PATHWAY
6:112151452	*FYN*	BIOCARTA_EPHA4_PATHWAY
6:112175069	*FYN*	BIOCARTA_EPHA4_PATHWAY
17:40392358	*RETREG3*	BIOCARTA_EPO_PATHWAY
5:133558885	*C5orf24*	BIOCARTA_ERK_PATHWAY
1:156059276	*GBAP1*	BIOCARTA_FAS_PATHWAY
1:226568665	*ITPKB*	BIOCARTA_FAS_PATHWAY
6:33287183	*HLA-DRB5*	BIOCARTA_FAS_PATHWAY
19:3142784	*SPPL2B*	BIOCARTA_FMLP_PATHWAY
19:3147326	*SPPL2B*	BIOCARTA_FMLP_PATHWAY
3:48199695	*IP6K2*	BIOCARTA_G1_PATHWAY
3:48214438	*IP6K2*	BIOCARTA_G1_PATHWAY
17:7578671	*V1NB1*	BIOCARTA_G1_PATHWAY
3:48199695	*IP6K2*	BIOCARTA_G2_PATHWAY
3:48214438	*IP6K2*	BIOCARTA_G2_PATHWAY
17:7578671	*V1NB1*	BIOCARTA_G2_PATHWAY
17:44800946	*WNT3*	BIOCARTA_GABA_PATHWAY
17:44833217	*WNT3*	BIOCARTA_GABA_PATHWAY
3:48835245	*IP6K2*	BIOCARTA_GATA3_PATHWAY
17:40392358	*RETREG3*	BIOCARTA_GH_PATHWAY
7:22311894	*GPNMB*	BIOCARTA_GHRELIN_PATHWAY
17:40392358	*RETREG3*	BIOCARTA_GLEEVEC_PATHWAY
3:48835245	*IP6K2*	BIOCARTA_GPCR_PATHWAY
16:30510571	*SETD1A*	BIOCARTA_GRANULOCYTES_PATHWAY
16:30516492	*SETD1A*	BIOCARTA_GRANULOCYTES_PATHWAY
16:30520856	*SETD1A*	BIOCARTA_GRANULOCYTES_PATHWAY
5:133558885	*C5orf24*	BIOCARTA_GSK3_PATHWAY
17:8025979	*V1NB1*	BIOCARTA_HES_PATHWAY
1:156059276	*GBAP1*	BIOCARTA_HIVNEF_PATHWAY
1:226568665	*ITPKB*	BIOCARTA_HIVNEF_PATHWAY
1:227067656	*ITPKB*	BIOCARTA_HIVNEF_PATHWAY
6:33287183	*HLA-DRB5*	BIOCARTA_HIVNEF_PATHWAY
17:43390763	*CRHR1_2*	BIOCARTA_HIVNEF_PATHWAY
6:33287183	*HLA-DRB5*	BIOCARTA_HSP27_PATHWAY
5:133558885	*C5orf24*	BIOCARTA_IGF1MTOR_PATHWAY
3:48835245	*IP6K2*	BIOCARTA_IGF1R_PATHWAY
2:102720323	*MAP4K4*	BIOCARTA_IL1R_PATHWAY
2:102783052	*MAP4K4*	BIOCARTA_IL1R_PATHWAY
17:43390763	*CRHR1_2*	BIOCARTA_IL1R_PATHWAY
17:40392358	*RETREG3*	BIOCARTA_IL22BP_PATHWAY
17:40392358	*RETREG3*	BIOCARTA_IL2_PATHWAY
17:40392358	*RETREG3*	BIOCARTA_IL2RB_PATHWAY
17:40392358	*RETREG3*	BIOCARTA_IL3_PATHWAY
6:112137847	*FYN*	BIOCARTA_IL7_PATHWAY
6:112151452	*FYN*	BIOCARTA_IL7_PATHWAY
6:112175069	*FYN*	BIOCARTA_IL7_PATHWAY
17:40392358	*RETREG3*	BIOCARTA_IL7_PATHWAY
3:49406095	*IP6K2*	BIOCARTA_INTEGRIN_PATHWAY
3:49424231	*IP6K2*	BIOCARTA_INTEGRIN_PATHWAY
3:49450137	*IP6K2*	BIOCARTA_INTEGRIN_PATHWAY
6:112137847	*FYN*	BIOCARTA_INTEGRIN_PATHWAY
6:112151452	*FYN*	BIOCARTA_INTEGRIN_PATHWAY
6:112175069	*FYN*	BIOCARTA_INTEGRIN_PATHWAY

17:7479140	*V1NB1*	BIOCARTA_IRES_PATHWAY
17:7480325	*V1NB1*	BIOCARTA_IRES_PATHWAY
5:133558885	*C5orf24*	BIOCARTA_KERATINOCYTE_PATHWAY
6:33287183	*HLA-DRB5*	BIOCARTA_KERATINOCYTE_PATHWAY
17:43390763	*CRHR1_2*	BIOCARTA_KERATINOCYTE_PATHWAY
16:30510571	*SETD1A*	BIOCARTA_LAIR_PATHWAY
16:30516492	*SETD1A*	BIOCARTA_LAIR_PATHWAY
16:30520856	*SETD1A*	BIOCARTA_LAIR_PATHWAY
6:31895092	*HLA-DRB5*	BIOCARTA_LECTIN_PATHWAY
6:31897219	*HLA-DRB5*	BIOCARTA_LECTIN_PATHWAY
12:122816734	*HIP1R*	BIOCARTA_LIS1_PATHWAY
12:122817580	*HIP1R*	BIOCARTA_LIS1_PATHWAY
12:122853197	*HIP1R*	BIOCARTA_LIS1_PATHWAY
17:7631694	*V1NB1*	BIOCARTA_LIS1_PATHWAY
17:7682605	*V1NB1*	BIOCARTA_LIS1_PATHWAY
16:30510571	*SETD1A*	BIOCARTA_LYM_PATHWAY
16:30516492	*SETD1A*	BIOCARTA_LYM_PATHWAY
16:30520856	*SETD1A*	BIOCARTA_LYM_PATHWAY
16:30510571	*SETD1A*	BIOCARTA_LYMPHOCYTE_PATHWAY
16:30516492	*SETD1A*	BIOCARTA_LYMPHOCYTE_PATHWAY
16:30520856	*SETD1A*	BIOCARTA_LYMPHOCYTE_PATHWAY
3:49406095	*IP6K2*	BIOCARTA_MAL_PATHWAY
3:49424231	*IP6K2*	BIOCARTA_MAL_PATHWAY
3:49450137	*IP6K2*	BIOCARTA_MAL_PATHWAY
2:102357447	*MAP4K4*	BIOCARTA_MAPK_PATHWAY
2:102379173	*MAP4K4*	BIOCARTA_MAPK_PATHWAY
2:102413116	*MAP4K4*	BIOCARTA_MAPK_PATHWAY
6:33287183	*HLA-DRB5*	BIOCARTA_MAPK_PATHWAY
17:43390763	*CRHR1_2*	BIOCARTA_MAPK_PATHWAY
18:48228968	*MEX3C*	BIOCARTA_MAPK_PATHWAY
3:48835245	*IP6K2*	BIOCARTA_MCALPAIN_PATHWAY
6:32789400	*HLA-DRB5*	BIOCARTA_MHC_PATHWAY
6:32791831	*HLA-DRB5*	BIOCARTA_MHC_PATHWAY
6:32797361	*HLA-DRB5*	BIOCARTA_MHC_PATHWAY
6:32802462	*HLA-DRB5*	BIOCARTA_MHC_PATHWAY
16:30510571	*SETD1A*	BIOCARTA_MONOCYTE_PATHWAY
16:30516492	*SETD1A*	BIOCARTA_MONOCYTE_PATHWAY
16:30520856	*SETD1A*	BIOCARTA_MONOCYTE_PATHWAY
3:48835245	*IP6K2*	BIOCARTA_MPR_PATHWAY
5:133558885	*C5orf24*	BIOCARTA_MTOR_PATHWAY
17:7479140	*V1NB1*	BIOCARTA_MTOR_PATHWAY
17:7480325	*V1NB1*	BIOCARTA_MTOR_PATHWAY
16:30510571	*SETD1A*	BIOCARTA_NEUTROPHIL_PATHWAY
16:30516492	*SETD1A*	BIOCARTA_NEUTROPHIL_PATHWAY
16:30520856	*SETD1A*	BIOCARTA_NEUTROPHIL_PATHWAY
3:48835245	*IP6K2*	BIOCARTA_NFAT_PATHWAY
8:11558567	*CTSB*	BIOCARTA_NFAT_PATHWAY
8:11599269	*CTSB*	BIOCARTA_NFAT_PATHWAY
2:102720323	*MAP4K4*	BIOCARTA_NFKB_PATHWAY
2:102783052	*MAP4K4*	BIOCARTA_NFKB_PATHWAY
17:43390763	*CRHR1_2*	BIOCARTA_NFKB_PATHWAY
3:48835245	*IP6K2*	BIOCARTA_NO1_PATHWAY
3:48835245	*IP6K2*	BIOCARTA_NOS1_PATHWAY

17:7101771	*V1NB1*	BIOCARTA_NOS1_PATHWAY
17:43390763	*CRHR1_2*	BIOCARTA_NTHI_PATHWAY
12:39960237	*LRRK2_2*	BIOCARTA_NUCLEARRS_PATHWAY
5:133558885	*C5orf24*	BIOCARTA_P35ALZHEIMERS_PATHWAY
6:33287183	*HLA-DRB5*	BIOCARTA_P38MAPK_PATHWAY
17:7578671	*V1NB1*	BIOCARTA_P53HYPOXIA_PATHWAY
17:7578671	*V1NB1*	BIOCARTA_P53_PATHWAY
3:49406095	*IP6K2*	BIOCARTA_PAR1_PATHWAY
3:49424231	*IP6K2*	BIOCARTA_PAR1_PATHWAY
3:49450137	*IP6K2*	BIOCARTA_PAR1_PATHWAY
4:90677243	*SNCA_2*	BIOCARTA_PARKIN_PATHWAY
4:90700329	*SNCA_2*	BIOCARTA_PARKIN_PATHWAY
4:90703993	*SNCA_2*	BIOCARTA_PARKIN_PATHWAY
4:90738839	*SNCA_2*	BIOCARTA_PARKIN_PATHWAY
4:90757294	*SNCA_2*	BIOCARTA_PARKIN_PATHWAY
1:204876966	*RAB29*	BIOCARTA_PDZS_PATHWAY
1:204940972	*RAB29*	BIOCARTA_PDZS_PATHWAY
11:83219006	*DLG2*	BIOCARTA_PDZS_PATHWAY
11:83232915	*DLG2*	BIOCARTA_PDZS_PATHWAY
11:83282740	*DLG2*	BIOCARTA_PDZS_PATHWAY
11:83387273	*DLG2*	BIOCARTA_PDZS_PATHWAY
11:83538733	*DLG2*	BIOCARTA_PDZS_PATHWAY
11:83544472	*DLG2*	BIOCARTA_PDZS_PATHWAY
11:83578152	*DLG2*	BIOCARTA_PDZS_PATHWAY
11:83647657	*DLG2*	BIOCARTA_PDZS_PATHWAY
11:83669903	*DLG2*	BIOCARTA_PDZS_PATHWAY
11:83736246	*DLG2*	BIOCARTA_PDZS_PATHWAY
11:83762918	*DLG2*	BIOCARTA_PDZS_PATHWAY
11:83823752	*DLG2*	BIOCARTA_PDZS_PATHWAY
11:83839992	*DLG2*	BIOCARTA_PDZS_PATHWAY
11:84104763	*DLG2*	BIOCARTA_PDZS_PATHWAY
11:84169039	*DLG2*	BIOCARTA_PDZS_PATHWAY
11:84221628	*DLG2*	BIOCARTA_PDZS_PATHWAY
11:84312008	*DLG2*	BIOCARTA_PDZS_PATHWAY
11:84324361	*DLG2*	BIOCARTA_PDZS_PATHWAY
11:84360346	*DLG2*	BIOCARTA_PDZS_PATHWAY
11:84403409	*DLG2*	BIOCARTA_PDZS_PATHWAY
17:7101771	*V1NB1*	BIOCARTA_PDZS_PATHWAY
17:42423844	*FAM171A2*	BIOCARTA_PEPI_PATHWAY
17:42426940	*FAM171A2*	BIOCARTA_PEPI_PATHWAY
3:48835245	*IP6K2*	BIOCARTA_PLCE_PATHWAY
17:7578671	*V1NB1*	BIOCARTA_PLK3_PATHWAY
6:33287183	*HLA-DRB5*	BIOCARTA_PML_PATHWAY
17:7578671	*V1NB1*	BIOCARTA_PML_PATHWAY
3:48835245	*IP6K2*	BIOCARTA_PPARA_PATHWAY
17:40392358	*RETREG3*	BIOCARTA_PPARA_PATHWAY
17:42990894	*CRHR1_2*	BIOCARTA_PRION_PATHWAY
10:103185542	*GBF1*	BIOCARTA_PS1_PATHWAY
10:103305087	*GBF1*	BIOCARTA_PS1_PATHWAY
10:103308244	*GBF1*	BIOCARTA_PS1_PATHWAY
3:48199695	*IP6K2*	BIOCARTA_PTC1_PATHWAY
3:48214438	*IP6K2*	BIOCARTA_PTC1_PATHWAY
1:155975202	*GBAP1*	BIOCARTA_PTDINS_PATHWAY

6:73383348	*RIMS1*	BIOCARTA_RACC_PATHWAY
6:73448086	*RIMS1*	BIOCARTA_RACC_PATHWAY
3:49406095	*IP6K2*	BIOCARTA_RACCYCD_PATHWAY
3:49424231	*IP6K2*	BIOCARTA_RACCYCD_PATHWAY
3:49450137	*IP6K2*	BIOCARTA_RACCYCD_PATHWAY
3:49406095	*IP6K2*	BIOCARTA_RAS_PATHWAY
3:49424231	*IP6K2*	BIOCARTA_RAS_PATHWAY
3:49450137	*IP6K2*	BIOCARTA_RAS_PATHWAY
3:48199695	*IP6K2*	BIOCARTA_RB_PATHWAY
3:48214438	*IP6K2*	BIOCARTA_RB_PATHWAY
17:7578671	*V1NB1*	BIOCARTA_RB_PATHWAY
17:76869878	*DNAH17*	BIOCARTA_RECK_PATHWAY
6:112137847	*FYN*	BIOCARTA_REELIN_PATHWAY
6:112151452	*FYN*	BIOCARTA_REELIN_PATHWAY
6:112175069	*FYN*	BIOCARTA_REELIN_PATHWAY
3:49406095	*IP6K2*	BIOCARTA_RHO_PATHWAY
3:49424231	*IP6K2*	BIOCARTA_RHO_PATHWAY
3:49450137	*IP6K2*	BIOCARTA_RHO_PATHWAY
17:7578671	*V1NB1*	BIOCARTA_RNA_PATHWAY
17:43390763	*CRHR1_2*	BIOCARTA_RNA_PATHWAY
3:48835245	*IP6K2*	BIOCARTA_SHH_PATHWAY
10:104278055	*GBF1*	BIOCARTA_SHH_PATHWAY
10:104305501	*GBF1*	BIOCARTA_SHH_PATHWAY
21:38740824	*DYRK1A*	BIOCARTA_SHH_PATHWAY
21:38753354	*DYRK1A*	BIOCARTA_SHH_PATHWAY
21:38770530	*DYRK1A*	BIOCARTA_SHH_PATHWAY
21:38783614	*DYRK1A*	BIOCARTA_SHH_PATHWAY
21:38814867	*DYRK1A*	BIOCARTA_SHH_PATHWAY
3:48199695	*IP6K2*	BIOCARTA_SRCRPTP_PATHWAY
3:48214438	*IP6K2*	BIOCARTA_SRCRPTP_PATHWAY
3:48835245	*IP6K2*	BIOCARTA_STATHMIN_PATHWAY
17:43390763	*CRHR1_2*	BIOCARTA_STRESS_PATHWAY
17:7455536	*V1NB1*	BIOCARTA_TALL1_PATHWAY
17:43390763	*CRHR1_2*	BIOCARTA_TALL1_PATHWAY
6:112137847	*FYN*	BIOCARTA_TCRA_PATHWAY
6:112151452	*FYN*	BIOCARTA_TCRA_PATHWAY
6:112175069	*FYN*	BIOCARTA_TCRA_PATHWAY
6:112137847	*FYN*	BIOCARTA_TCR_PATHWAY
6:112151452	*FYN*	BIOCARTA_TCR_PATHWAY
6:112175069	*FYN*	BIOCARTA_TCR_PATHWAY
16:30510571	*SETD1A*	BIOCARTA_TCYTOTOXIC_PATHWAY
16:30516492	*SETD1A*	BIOCARTA_TCYTOTOXIC_PATHWAY
16:30520856	*SETD1A*	BIOCARTA_TCYTOTOXIC_PATHWAY
5:133558885	*C5orf24*	BIOCARTA_TEL_PATHWAY
17:7578671	*V1NB1*	BIOCARTA_TEL_PATHWAY
17:7578671	*V1NB1*	BIOCARTA_TERT_PATHWAY
3:49406095	*IP6K2*	BIOCARTA_TFF_PATHWAY
3:49424231	*IP6K2*	BIOCARTA_TFF_PATHWAY
3:49450137	*IP6K2*	BIOCARTA_TFF_PATHWAY
16:30510571	*SETD1A*	BIOCARTA_THELPER_PATHWAY
16:30516492	*SETD1A*	BIOCARTA_THELPER_PATHWAY
16:30520856	*SETD1A*	BIOCARTA_THELPER_PATHWAY
17:7578671	*V1NB1*	BIOCARTA_TID_PATHWAY

1:156059276	*GBAP1*	BIOCARTA_TNFR1_PATHWAY
1:226568665	*ITPKB*	BIOCARTA_TNFR1_PATHWAY
17:43390763	*CRHR1_2*	BIOCARTA_TNFR2_PATHWAY
17:43390763	*CRHR1_2*	BIOCARTA_TOLL_PATHWAY
17:40392358	*RETREG3*	BIOCARTA_TPO_PATHWAY
6:112137847	*FYN*	BIOCARTA_TSP1_PATHWAY
6:112151452	*FYN*	BIOCARTA_TSP1_PATHWAY
6:112175069	*FYN*	BIOCARTA_TSP1_PATHWAY
3:49406095	*IP6K2*	BIOCARTA_UCALPAIN_PATHWAY
3:49424231	*IP6K2*	BIOCARTA_UCALPAIN_PATHWAY
3:49450137	*IP6K2*	BIOCARTA_UCALPAIN_PATHWAY
3:48835245	*IP6K2*	BIOCARTA_VIP_PATHWAY
5:133558885	*C5orf24*	BIOCARTA_WNT_PATHWAY
10:103185542	*GBF1*	BIOCARTA_WNT_PATHWAY
10:103305087	*GBF1*	BIOCARTA_WNT_PATHWAY
10:103308244	*GBF1*	BIOCARTA_WNT_PATHWAY
6:32789400	*HLA-DRB5*	KEGG_ABC_TRANSPORTERS
6:32791831	*HLA-DRB5*	KEGG_ABC_TRANSPORTERS
6:32797361	*HLA-DRB5*	KEGG_ABC_TRANSPORTERS
6:32802462	*HLA-DRB5*	KEGG_ABC_TRANSPORTERS
12:39960237	*LRRK2_2*	KEGG_ABC_TRANSPORTERS
12:123453034	*HIP1R*	KEGG_ABC_TRANSPORTERS
14:74764925	*RPS6KL1*	KEGG_ABC_TRANSPORTERS
17:39883520	*RETREG3*	KEGG_ACUTE_MYELOID_LEUKEMIA
17:40392358	*RETREG3*	KEGG_ACUTE_MYELOID_LEUKEMIA
3:49406095	*IP6K2*	KEGG_ADHERENS_JUNCTION
3:49424231	*IP6K2*	KEGG_ADHERENS_JUNCTION
3:49450137	*IP6K2*	KEGG_ADHERENS_JUNCTION
6:112137847	*FYN*	KEGG_ADHERENS_JUNCTION
6:112151452	*FYN*	KEGG_ADHERENS_JUNCTION
6:112175069	*FYN*	KEGG_ADHERENS_JUNCTION
17:41056283	*RETREG3*	KEGG_ADIPOCYTOKINE_SIGNALING_PATHWAY
17:7560294	*V1NB1*	KEGG_ALDOSTERONE_REGULATED_SODIUM_REABSORPTION
6:29798642	*TRIM40*	KEGG_ALLOGRAFT_REJECTION
6:32602372	*HLA-DRB5*	KEGG_ALLOGRAFT_REJECTION
6:32930679	*HLA-DRB5*	KEGG_ALLOGRAFT_REJECTION
6:32934409	*HLA-DRB5*	KEGG_ALLOGRAFT_REJECTION
6:32973303	*HLA-DRB5*	KEGG_ALLOGRAFT_REJECTION
6:32974268	*HLA-DRB5*	KEGG_ALLOGRAFT_REJECTION
1:161171736	*FCGR2A*	KEGG_ALZHEIMERS_DISEASE
1:161875645	*FCGR2A*	KEGG_ALZHEIMERS_DISEASE
1:161930954	*FCGR2A*	KEGG_ALZHEIMERS_DISEASE
1:227067656	*ITPKB*	KEGG_ALZHEIMERS_DISEASE
4:90677243	*SNCA_2*	KEGG_ALZHEIMERS_DISEASE
4:90700329	*SNCA_2*	KEGG_ALZHEIMERS_DISEASE
4:90703993	*SNCA_2*	KEGG_ALZHEIMERS_DISEASE
4:90738839	*SNCA_2*	KEGG_ALZHEIMERS_DISEASE
4:90757294	*SNCA_2*	KEGG_ALZHEIMERS_DISEASE
17:43999203	*WNT3*	KEGG_ALZHEIMERS_DISEASE
17:44005297	*WNT3*	KEGG_ALZHEIMERS_DISEASE
17:44019712	*WNT3*	KEGG_ALZHEIMERS_DISEASE
17:44060492	*WNT3*	KEGG_ALZHEIMERS_DISEASE
17:44065263	*WNT3*	KEGG_ALZHEIMERS_DISEASE

17:44081178	*WNT3*	KEGG_ALZHEIMERS_DISEASE
17:44083297	*WNT3*	KEGG_ALZHEIMERS_DISEASE
6:31758240	*HLA-DRB5*	KEGG_AMINOACYL_TRNA_BIOSYNTHESIS
6:31762311	*HLA-DRB5*	KEGG_AMINOACYL_TRNA_BIOSYNTHESIS
6:33287183	*HLA-DRB5*	KEGG_AMYOTROPHIC_LATERAL_SCLEROSIS_ALS
17:7578671	*V1NB1*	KEGG_AMYOTROPHIC_LATERAL_SCLEROSIS_ALS
6:29798642	*TRIM40*	KEGG_ANTIGEN_PROCESSING_AND_PRESENTATION
6:32602372	*HLA-DRB5*	KEGG_ANTIGEN_PROCESSING_AND_PRESENTATION
6:32789400	*HLA-DRB5*	KEGG_ANTIGEN_PROCESSING_AND_PRESENTATION
6:32791831	*HLA-DRB5*	KEGG_ANTIGEN_PROCESSING_AND_PRESENTATION
6:32797361	*HLA-DRB5*	KEGG_ANTIGEN_PROCESSING_AND_PRESENTATION
6:32802462	*HLA-DRB5*	KEGG_ANTIGEN_PROCESSING_AND_PRESENTATION
6:32930679	*HLA-DRB5*	KEGG_ANTIGEN_PROCESSING_AND_PRESENTATION
6:32934409	*HLA-DRB5*	KEGG_ANTIGEN_PROCESSING_AND_PRESENTATION
6:32973303	*HLA-DRB5*	KEGG_ANTIGEN_PROCESSING_AND_PRESENTATION
6:32974268	*HLA-DRB5*	KEGG_ANTIGEN_PROCESSING_AND_PRESENTATION
8:11712443	*CTSB*	KEGG_ANTIGEN_PROCESSING_AND_PRESENTATION
2:102720323	*MAP4K4*	KEGG_APOPTOSIS
2:102783052	*MAP4K4*	KEGG_APOPTOSIS
3:48835245	*IP6K2*	KEGG_APOPTOSIS
8:22974278	*BIN3*	KEGG_APOPTOSIS
8:23050400	*BIN3*	KEGG_APOPTOSIS
17:7578671	*V1NB1*	KEGG_APOPTOSIS
17:43390763	*CRHR1_2*	KEGG_APOPTOSIS
17:7983799	*V1NB1*	KEGG_ARACHIDONIC_ACID_METABOLISM
1:226108200	*ITPKB*	KEGG_ARGININE_AND_PROLINE_METABOLISM
4:17606956	*LCORL*	KEGG_ARGININE_AND_PROLINE_METABOLISM
1:156059276	*GBAP1*	KEGG_ARRHYTHMOGENIC_RIGHT_VENTRICULAR_CARDIOMYOPATHY_ARVC
3:49561208	*IP6K2*	KEGG_ARRHYTHMOGENIC_RIGHT_VENTRICULAR_CARDIOMYOPATHY_ARVC
10:15562608	*ITGA8*	KEGG_ARRHYTHMOGENIC_RIGHT_VENTRICULAR_CARDIOMYOPATHY_ARVC
10:15569598	*ITGA8*	KEGG_ARRHYTHMOGENIC_RIGHT_VENTRICULAR_CARDIOMYOPATHY_ARVC
10:15652368	*ITGA8*	KEGG_ARRHYTHMOGENIC_RIGHT_VENTRICULAR_CARDIOMYOPATHY_ARVC
17:39883520	*RETREG3*	KEGG_ARRHYTHMOGENIC_RIGHT_VENTRICULAR_CARDIOMYOPATHY_ARVC
17:42454243	*FAM171A2*	KEGG_ARRHYTHMOGENIC_RIGHT_VENTRICULAR_CARDIOMYOPATHY_ARVC
6:32602372	*HLA-DRB5*	KEGG_ASTHMA
6:32930679	*HLA-DRB5*	KEGG_ASTHMA
6:32934409	*HLA-DRB5*	KEGG_ASTHMA
6:32973303	*HLA-DRB5*	KEGG_ASTHMA
6:32974268	*HLA-DRB5*	KEGG_ASTHMA
6:29798642	*TRIM40*	KEGG_AUTOIMMUNE_THYROID_DISEASE
6:32602372	*HLA-DRB5*	KEGG_AUTOIMMUNE_THYROID_DISEASE
6:32930679	*HLA-DRB5*	KEGG_AUTOIMMUNE_THYROID_DISEASE
6:32934409	*HLA-DRB5*	KEGG_AUTOIMMUNE_THYROID_DISEASE
6:32973303	*HLA-DRB5*	KEGG_AUTOIMMUNE_THYROID_DISEASE
6:32974268	*HLA-DRB5*	KEGG_AUTOIMMUNE_THYROID_DISEASE
3:49406095	*IP6K2*	KEGG_AXON_GUIDANCE
3:49424231	*IP6K2*	KEGG_AXON_GUIDANCE
3:49450137	*IP6K2*	KEGG_AXON_GUIDANCE
3:122645304	*KPNA1*	KEGG_AXON_GUIDANCE
3:122720608	*KPNA1*	KEGG_AXON_GUIDANCE
6:112137847	*FYN*	KEGG_AXON_GUIDANCE
6:112151452	*FYN*	KEGG_AXON_GUIDANCE
6:112175069	*FYN*	KEGG_AXON_GUIDANCE

10:104278055	*GBF1*	KEGG_BASAL_CELL_CARCINOMA
10:104305501	*GBF1*	KEGG_BASAL_CELL_CARCINOMA
17:7129974	*V1NB1*	KEGG_BASAL_CELL_CARCINOMA
17:7578671	*V1NB1*	KEGG_BASAL_CELL_CARCINOMA
17:44843651	*WNT3*	KEGG_BASAL_CELL_CARCINOMA
17:44870978	*WNT3*	KEGG_BASAL_CELL_CARCINOMA
17:44871422	*WNT3*	KEGG_BASAL_CELL_CARCINOMA
17:44888209	*WNT3*	KEGG_BASAL_CELL_CARCINOMA
17:44888715	*WNT3*	KEGG_BASAL_CELL_CARCINOMA
17:44906949	*WNT3*	KEGG_BASAL_CELL_CARCINOMA
17:44912901	*WNT3*	KEGG_BASAL_CELL_CARCINOMA
17:44931399	*WNT3*	KEGG_BASAL_CELL_CARCINOMA
17:44947756	*WNT3*	KEGG_BASAL_CELL_CARCINOMA
14:55902186	*GCH1*	KEGG_BASAL_TRANSCRIPTION_FACTORS
1:226568665	*ITPKB*	KEGG_BASE_EXCISION_REPAIR
12:133206648	*FBRSL1*	KEGG_BASE_EXCISION_REPAIR
12:133223685	*FBRSL1*	KEGG_BASE_EXCISION_REPAIR
17:7578671	*V1NB1*	KEGG_BLADDER_CANCER
1:226907849	*ITPKB*	KEGG_CALCIUM_SIGNALING_PATHWAY
1:226910340	*ITPKB*	KEGG_CALCIUM_SIGNALING_PATHWAY
1:226916078	*ITPKB*	KEGG_CALCIUM_SIGNALING_PATHWAY
4:15709252	*BST1*	KEGG_CALCIUM_SIGNALING_PATHWAY
4:15713608	*BST1*	KEGG_CALCIUM_SIGNALING_PATHWAY
4:15724372	*BST1*	KEGG_CALCIUM_SIGNALING_PATHWAY
4:15737882	*BST1*	KEGG_CALCIUM_SIGNALING_PATHWAY
4:15789288	*BST1*	KEGG_CALCIUM_SIGNALING_PATHWAY
4:15816670	*BST1*	KEGG_CALCIUM_SIGNALING_PATHWAY
4:15829612	*BST1*	KEGG_CALCIUM_SIGNALING_PATHWAY
4:114424939	*CAMK2D*	KEGG_CALCIUM_SIGNALING_PATHWAY
4:114494052	*CAMK2D*	KEGG_CALCIUM_SIGNALING_PATHWAY
4:114529492	*CAMK2D*	KEGG_CALCIUM_SIGNALING_PATHWAY
4:114539964	*CAMK2D*	KEGG_CALCIUM_SIGNALING_PATHWAY
4:114597918	*CAMK2D*	KEGG_CALCIUM_SIGNALING_PATHWAY
8:131799767	*FAM49B*	KEGG_CALCIUM_SIGNALING_PATHWAY
8:131845512	*FAM49B*	KEGG_CALCIUM_SIGNALING_PATHWAY
8:131865425	*FAM49B*	KEGG_CALCIUM_SIGNALING_PATHWAY
8:131901170	*FAM49B*	KEGG_CALCIUM_SIGNALING_PATHWAY
16:50300511	*CASC16*	KEGG_CALCIUM_SIGNALING_PATHWAY
16:50317039	*CASC16*	KEGG_CALCIUM_SIGNALING_PATHWAY
17:43195981	*CRHR1_2*	KEGG_CALCIUM_SIGNALING_PATHWAY
19:3142784	*SPPL2B*	KEGG_CALCIUM_SIGNALING_PATHWAY
19:3147326	*SPPL2B*	KEGG_CALCIUM_SIGNALING_PATHWAY
17:7560294	*V1NB1*	KEGG_CARDIAC_MUSCLE_CONTRACTION
1:204876966	*RAB29*	KEGG_CELL_ADHESION_MOLECULES_CAMS
1:204940972	*RAB29*	KEGG_CELL_ADHESION_MOLECULES_CAMS
6:29798642	*TRIM40*	KEGG_CELL_ADHESION_MOLECULES_CAMS
6:32602372	*HLA-DRB5*	KEGG_CELL_ADHESION_MOLECULES_CAMS
6:32930679	*HLA-DRB5*	KEGG_CELL_ADHESION_MOLECULES_CAMS
6:32934409	*HLA-DRB5*	KEGG_CELL_ADHESION_MOLECULES_CAMS
6:32973303	*HLA-DRB5*	KEGG_CELL_ADHESION_MOLECULES_CAMS
6:32974268	*HLA-DRB5*	KEGG_CELL_ADHESION_MOLECULES_CAMS
10:15562608	*ITGA8*	KEGG_CELL_ADHESION_MOLECULES_CAMS
10:15569598	*ITGA8*	KEGG_CELL_ADHESION_MOLECULES_CAMS

10:15652368	*ITGA8*	KEGG_CELL_ADHESION_MOLECULES_CAMS
11:133947732	*IGSF9B*	KEGG_CELL_ADHESION_MOLECULES_CAMS
12:41144387	*LRRK2_2*	KEGG_CELL_ADHESION_MOLECULES_CAMS
12:41147295	*LRRK2_2*	KEGG_CELL_ADHESION_MOLECULES_CAMS
12:41195135	*LRRK2_2*	KEGG_CELL_ADHESION_MOLECULES_CAMS
12:41251554	*LRRK2_2*	KEGG_CELL_ADHESION_MOLECULES_CAMS
12:41255129	*LRRK2_2*	KEGG_CELL_ADHESION_MOLECULES_CAMS
12:41297969	*LRRK2_2*	KEGG_CELL_ADHESION_MOLECULES_CAMS
12:41407403	*LRRK2_2*	KEGG_CELL_ADHESION_MOLECULES_CAMS
16:30510571	*SETD1A*	KEGG_CELL_ADHESION_MOLECULES_CAMS
16:30516492	*SETD1A*	KEGG_CELL_ADHESION_MOLECULES_CAMS
16:30520856	*SETD1A*	KEGG_CELL_ADHESION_MOLECULES_CAMS
21:37854286	*DYRK1A*	KEGG_CELL_ADHESION_MOLECULES_CAMS
21:37859449	*DYRK1A*	KEGG_CELL_ADHESION_MOLECULES_CAMS
21:37860785	*DYRK1A*	KEGG_CELL_ADHESION_MOLECULES_CAMS
21:37894089	*DYRK1A*	KEGG_CELL_ADHESION_MOLECULES_CAMS
21:37899076	*DYRK1A*	KEGG_CELL_ADHESION_MOLECULES_CAMS
3:48199695	*IP6K2*	KEGG_CELL_CYCLE
3:48214438	*IP6K2*	KEGG_CELL_CYCLE
17:7578671	*V1NB1*	KEGG_CELL_CYCLE
17:45215865	*WNT3*	KEGG_CELL_CYCLE
17:45244822	*WNT3*	KEGG_CELL_CYCLE
19:2477820	*SPPL2B*	KEGG_CELL_CYCLE
3:49406095	*IP6K2*	KEGG_CHEMOKINE_SIGNALING_PATHWAY
3:49424231	*IP6K2*	KEGG_CHEMOKINE_SIGNALING_PATHWAY
3:49450137	*IP6K2*	KEGG_CHEMOKINE_SIGNALING_PATHWAY
3:123089448	*KPNA1*	KEGG_CHEMOKINE_SIGNALING_PATHWAY
4:76924571	*FAM47E-STBD1*	KEGG_CHEMOKINE_SIGNALING_PATHWAY
8:131799767	*FAM49B*	KEGG_CHEMOKINE_SIGNALING_PATHWAY
8:131845512	*FAM49B*	KEGG_CHEMOKINE_SIGNALING_PATHWAY
8:131865425	*FAM49B*	KEGG_CHEMOKINE_SIGNALING_PATHWAY
8:131901170	*FAM49B*	KEGG_CHEMOKINE_SIGNALING_PATHWAY
10:121054578	*INPP5F*	KEGG_CHEMOKINE_SIGNALING_PATHWAY
10:121077191	*INPP5F*	KEGG_CHEMOKINE_SIGNALING_PATHWAY
10:121085648	*INPP5F*	KEGG_CHEMOKINE_SIGNALING_PATHWAY
10:121104943	*INPP5F*	KEGG_CHEMOKINE_SIGNALING_PATHWAY
10:121114292	*INPP5F*	KEGG_CHEMOKINE_SIGNALING_PATHWAY
10:121191186	*INPP5F*	KEGG_CHEMOKINE_SIGNALING_PATHWAY
10:121201427	*INPP5F*	KEGG_CHEMOKINE_SIGNALING_PATHWAY
16:50300511	*CASC16*	KEGG_CHEMOKINE_SIGNALING_PATHWAY
16:50317039	*CASC16*	KEGG_CHEMOKINE_SIGNALING_PATHWAY
17:40392358	*RETREG3*	KEGG_CHEMOKINE_SIGNALING_PATHWAY
19:2557113	*SPPL2B*	KEGG_CHEMOKINE_SIGNALING_PATHWAY
19:2563327	*SPPL2B*	KEGG_CHEMOKINE_SIGNALING_PATHWAY
19:2580057	*SPPL2B*	KEGG_CHEMOKINE_SIGNALING_PATHWAY
19:2660983	*SPPL2B*	KEGG_CHEMOKINE_SIGNALING_PATHWAY
17:7578671	*V1NB1*	KEGG_CHRONIC_MYELOID_LEUKEMIA
17:40392358	*RETREG3*	KEGG_CHRONIC_MYELOID_LEUKEMIA
2:101439596	*MAP4K4*	KEGG_CIRCADIAN_RHYTHM_MAMMAL
2:101464631	*MAP4K4*	KEGG_CIRCADIAN_RHYTHM_MAMMAL
2:101547064	*MAP4K4*	KEGG_CIRCADIAN_RHYTHM_MAMMAL
2:101550181	*MAP4K4*	KEGG_CIRCADIAN_RHYTHM_MAMMAL
2:101578010	*MAP4K4*	KEGG_CIRCADIAN_RHYTHM_MAMMAL

2:101603812	*MAP4K4*	KEGG_CIRCADIAN_RHYTHM_MAMMAL
3:49406095	*IP6K2*	KEGG_COLORECTAL_CANCER
3:49424231	*IP6K2*	KEGG_COLORECTAL_CANCER
3:49450137	*IP6K2*	KEGG_COLORECTAL_CANCER
17:7578671	*V1NB1*	KEGG_COLORECTAL_CANCER
6:31897219	*HLA-DRB5*	KEGG_COMPLEMENT_AND_COAGULATION_CASCADES
2:102612026	*MAP4K4*	KEGG_CYTOKINE_CYTOKINE_RECEPTOR_INTERACTION
2:102621855	*MAP4K4*	KEGG_CYTOKINE_CYTOKINE_RECEPTOR_INTERACTION
2:102631503	*MAP4K4*	KEGG_CYTOKINE_CYTOKINE_RECEPTOR_INTERACTION
2:102633387	*MAP4K4*	KEGG_CYTOKINE_CYTOKINE_RECEPTOR_INTERACTION
2:102720323	*MAP4K4*	KEGG_CYTOKINE_CYTOKINE_RECEPTOR_INTERACTION
2:102783052	*MAP4K4*	KEGG_CYTOKINE_CYTOKINE_RECEPTOR_INTERACTION
2:103047986	*MAP4K4*	KEGG_CYTOKINE_CYTOKINE_RECEPTOR_INTERACTION
2:103063855	*MAP4K4*	KEGG_CYTOKINE_CYTOKINE_RECEPTOR_INTERACTION
2:103066346	*MAP4K4*	KEGG_CYTOKINE_CYTOKINE_RECEPTOR_INTERACTION
4:76924571	*FAM47E-STBD1*	KEGG_CYTOKINE_CYTOKINE_RECEPTOR_INTERACTION
8:22974278	*BIN3*	KEGG_CYTOKINE_CYTOKINE_RECEPTOR_INTERACTION
8:23050400	*BIN3*	KEGG_CYTOKINE_CYTOKINE_RECEPTOR_INTERACTION
19:2251817	*SPPL2B*	KEGG_CYTOKINE_CYTOKINE_RECEPTOR_INTERACTION
8:22108784	*BIN3*	KEGG_CYTOSOLIC_DNA_SENSING_PATHWAY
1:156059276	*GBAP1*	KEGG_DILATED_CARDIOMYOPATHY
3:49561208	*IP6K2*	KEGG_DILATED_CARDIOMYOPATHY
3:123089448	*KPNA1*	KEGG_DILATED_CARDIOMYOPATHY
8:131799767	*FAM49B*	KEGG_DILATED_CARDIOMYOPATHY
8:131845512	*FAM49B*	KEGG_DILATED_CARDIOMYOPATHY
8:131865425	*FAM49B*	KEGG_DILATED_CARDIOMYOPATHY
8:131901170	*FAM49B*	KEGG_DILATED_CARDIOMYOPATHY
10:15562608	*ITGA8*	KEGG_DILATED_CARDIOMYOPATHY
10:15569598	*ITGA8*	KEGG_DILATED_CARDIOMYOPATHY
10:15652368	*ITGA8*	KEGG_DILATED_CARDIOMYOPATHY
16:50300511	*CASC16*	KEGG_DILATED_CARDIOMYOPATHY
16:50317039	*CASC16*	KEGG_DILATED_CARDIOMYOPATHY
17:42454243	*FAM171A2*	KEGG_DILATED_CARDIOMYOPATHY
12:133206648	*FBRSL1*	KEGG_DNA_REPLICATION
12:133223685	*FBRSL1*	KEGG_DNA_REPLICATION
6:32179319	*HLA-DRB5*	KEGG_DORSO_VENTRAL_AXIS_FORMATION
6:32186193	*HLA-DRB5*	KEGG_DORSO_VENTRAL_AXIS_FORMATION
7:65425894	*GS1-124K5.11*	KEGG_DRUG_METABOLISM_OTHER_ENZYMES
17:76177567	*DNAH17*	KEGG_DRUG_METABOLISM_OTHER_ENZYMES
3:49162284	*IP6K2*	KEGG_ECM_RECEPTOR_INTERACTION
3:49162583	*IP6K2*	KEGG_ECM_RECEPTOR_INTERACTION
3:49561208	*IP6K2*	KEGG_ECM_RECEPTOR_INTERACTION
6:32031904	*HLA-DRB5*	KEGG_ECM_RECEPTOR_INTERACTION
6:32062687	*HLA-DRB5*	KEGG_ECM_RECEPTOR_INTERACTION
6:33153528	*HLA-DRB5*	KEGG_ECM_RECEPTOR_INTERACTION
6:112494762	*FYN*	KEGG_ECM_RECEPTOR_INTERACTION
6:112531401	*FYN*	KEGG_ECM_RECEPTOR_INTERACTION
6:112561154	*FYN*	KEGG_ECM_RECEPTOR_INTERACTION
6:112561430	*FYN*	KEGG_ECM_RECEPTOR_INTERACTION
10:15562608	*ITGA8*	KEGG_ECM_RECEPTOR_INTERACTION
10:15569598	*ITGA8*	KEGG_ECM_RECEPTOR_INTERACTION
10:15652368	*ITGA8*	KEGG_ECM_RECEPTOR_INTERACTION
17:42454243	*FAM171A2*	KEGG_ECM_RECEPTOR_INTERACTION

1:171837985	*VAMP4*	KEGG_ENDOCYTOSIS
1:171945488	*VAMP4*	KEGG_ENDOCYTOSIS
1:172137119	*VAMP4*	KEGG_ENDOCYTOSIS
1:172386261	*VAMP4*	KEGG_ENDOCYTOSIS
6:29798642	*TRIM40*	KEGG_ENDOCYTOSIS
6:71510024	*RIMS1*	KEGG_ENDOCYTOSIS
6:71539732	*RIMS1*	KEGG_ENDOCYTOSIS
8:17125768	*FGF20*	KEGG_ENDOCYTOSIS
8:131145058	*FAM49B*	KEGG_ENDOCYTOSIS
8:131153067	*FAM49B*	KEGG_ENDOCYTOSIS
8:131245821	*FAM49B*	KEGG_ENDOCYTOSIS
8:131328260	*FAM49B*	KEGG_ENDOCYTOSIS
8:131404984	*FAM49B*	KEGG_ENDOCYTOSIS
8:131408202	*FAM49B*	KEGG_ENDOCYTOSIS
8:131455469	*FAM49B*	KEGG_ENDOCYTOSIS
9:17579690	*SH3GL2_2*	KEGG_ENDOCYTOSIS
9:17587160	*SH3GL2_2*	KEGG_ENDOCYTOSIS
9:17609302	*SH3GL2_2*	KEGG_ENDOCYTOSIS
9:17626762	*SH3GL2_2*	KEGG_ENDOCYTOSIS
9:17627444	*SH3GL2_2*	KEGG_ENDOCYTOSIS
9:17642664	*SH3GL2_2*	KEGG_ENDOCYTOSIS
9:17710876	*SH3GL2_2*	KEGG_ENDOCYTOSIS
9:17732590	*SH3GL2_2*	KEGG_ENDOCYTOSIS
9:17737514	*SH3GL2_2*	KEGG_ENDOCYTOSIS
9:17758945	*SH3GL2_2*	KEGG_ENDOCYTOSIS
10:121054578	*INPP5F*	KEGG_ENDOCYTOSIS
10:121077191	*INPP5F*	KEGG_ENDOCYTOSIS
10:121085648	*INPP5F*	KEGG_ENDOCYTOSIS
10:121104943	*INPP5F*	KEGG_ENDOCYTOSIS
10:121114292	*INPP5F*	KEGG_ENDOCYTOSIS
10:121191186	*INPP5F*	KEGG_ENDOCYTOSIS
10:121201427	*INPP5F*	KEGG_ENDOCYTOSIS
17:7578671	*V1NB1*	KEGG_ENDOMETRIAL_CANCER
11:133947732	*IGSF9B*	KEGG_EPITHELIAL_CELL_SIGNALING_IN_HELICOBACTER_PYLORI_INFECTION
12:124219791	*HIP1R*	KEGG_EPITHELIAL_CELL_SIGNALING_IN_HELICOBACTER_PYLORI_INFECTION
17:43390763	*CRHR1_2*	KEGG_EPITHELIAL_CELL_SIGNALING_IN_HELICOBACTER_PYLORI_INFECTION
4:114424939	*CAMK2D*	KEGG_ERBB_SIGNALING_PATHWAY
4:114494052	*CAMK2D*	KEGG_ERBB_SIGNALING_PATHWAY
4:114529492	*CAMK2D*	KEGG_ERBB_SIGNALING_PATHWAY
4:114539964	*CAMK2D*	KEGG_ERBB_SIGNALING_PATHWAY
4:114597918	*CAMK2D*	KEGG_ERBB_SIGNALING_PATHWAY
17:40392358	*RETREG3*	KEGG_ERBB_SIGNALING_PATHWAY
6:112137847	*FYN*	KEGG_FC_EPSILON_RI_SIGNALING_PATHWAY
6:112151452	*FYN*	KEGG_FC_EPSILON_RI_SIGNALING_PATHWAY
6:112175069	*FYN*	KEGG_FC_EPSILON_RI_SIGNALING_PATHWAY
1:171837985	*VAMP4*	KEGG_FC_GAMMA_R_MEDIATED_PHAGOCYTOSIS
1:171945488	*VAMP4*	KEGG_FC_GAMMA_R_MEDIATED_PHAGOCYTOSIS
1:172137119	*VAMP4*	KEGG_FC_GAMMA_R_MEDIATED_PHAGOCYTOSIS
1:172386261	*VAMP4*	KEGG_FC_GAMMA_R_MEDIATED_PHAGOCYTOSIS
8:131145058	*FAM49B*	KEGG_FC_GAMMA_R_MEDIATED_PHAGOCYTOSIS
8:131153067	*FAM49B*	KEGG_FC_GAMMA_R_MEDIATED_PHAGOCYTOSIS
8:131245821	*FAM49B*	KEGG_FC_GAMMA_R_MEDIATED_PHAGOCYTOSIS
8:131328260	*FAM49B*	KEGG_FC_GAMMA_R_MEDIATED_PHAGOCYTOSIS

8:131404984	*FAM49B*	KEGG_FC_GAMMA_R_MEDIATED_PHAGOCYTOSIS
8:131408202	*FAM49B*	KEGG_FC_GAMMA_R_MEDIATED_PHAGOCYTOSIS
8:131455469	*FAM49B*	KEGG_FC_GAMMA_R_MEDIATED_PHAGOCYTOSIS
3:49162284	*IP6K2*	KEGG_FOCAL_ADHESION
3:49162583	*IP6K2*	KEGG_FOCAL_ADHESION
3:49406095	*IP6K2*	KEGG_FOCAL_ADHESION
3:49424231	*IP6K2*	KEGG_FOCAL_ADHESION
3:49450137	*IP6K2*	KEGG_FOCAL_ADHESION
6:32031904	*HLA-DRB5*	KEGG_FOCAL_ADHESION
6:32062687	*HLA-DRB5*	KEGG_FOCAL_ADHESION
6:33153528	*HLA-DRB5*	KEGG_FOCAL_ADHESION
6:112137847	*FYN*	KEGG_FOCAL_ADHESION
6:112151452	*FYN*	KEGG_FOCAL_ADHESION
6:112175069	*FYN*	KEGG_FOCAL_ADHESION
6:112494762	*FYN*	KEGG_FOCAL_ADHESION
6:112531401	*FYN*	KEGG_FOCAL_ADHESION
6:112561154	*FYN*	KEGG_FOCAL_ADHESION
6:112561430	*FYN*	KEGG_FOCAL_ADHESION
10:15562608	*ITGA8*	KEGG_FOCAL_ADHESION
10:15569598	*ITGA8*	KEGG_FOCAL_ADHESION
10:15652368	*ITGA8*	KEGG_FOCAL_ADHESION
15:62744042	*VPS13C*	KEGG_FOCAL_ADHESION
15:62748823	*VPS13C*	KEGG_FOCAL_ADHESION
15:62755248	*VPS13C*	KEGG_FOCAL_ADHESION
15:62758352	*VPS13C*	KEGG_FOCAL_ADHESION
15:62865256	*VPS13C*	KEGG_FOCAL_ADHESION
15:62875398	*VPS13C*	KEGG_FOCAL_ADHESION
15:62875464	*VPS13C*	KEGG_FOCAL_ADHESION
15:62915346	*VPS13C*	KEGG_FOCAL_ADHESION
15:62968927	*VPS13C*	KEGG_FOCAL_ADHESION
17:42454243	*FAM171A2*	KEGG_FOCAL_ADHESION
4:17465704	*LCORL*	KEGG_FOLATE_BIOSYNTHESIS
4:17503839	*LCORL*	KEGG_FOLATE_BIOSYNTHESIS
14:55348869	*GCH1*	KEGG_FOLATE_BIOSYNTHESIS
8:17161374	*FGF20*	KEGG_FRUCTOSE_AND_MANNOSE_METABOLISM
8:17176046	*FGF20*	KEGG_FRUCTOSE_AND_MANNOSE_METABOLISM
8:17243260	*FGF20*	KEGG_FRUCTOSE_AND_MANNOSE_METABOLISM
8:17260937	*FGF20*	KEGG_FRUCTOSE_AND_MANNOSE_METABOLISM
17:41056283	*RETREG3*	KEGG_GALACTOSE_METABOLISM
3:123089448	*KPNA1*	KEGG_GAP_JUNCTION
8:131799767	*FAM49B*	KEGG_GAP_JUNCTION
8:131845512	*FAM49B*	KEGG_GAP_JUNCTION
8:131865425	*FAM49B*	KEGG_GAP_JUNCTION
8:131901170	*FAM49B*	KEGG_GAP_JUNCTION
16:50300511	*CASC16*	KEGG_GAP_JUNCTION
16:50317039	*CASC16*	KEGG_GAP_JUNCTION
4:114424939	*CAMK2D*	KEGG_GLIOMA
4:114494052	*CAMK2D*	KEGG_GLIOMA
4:114529492	*CAMK2D*	KEGG_GLIOMA
4:114539964	*CAMK2D*	KEGG_GLIOMA
4:114597918	*CAMK2D*	KEGG_GLIOMA
17:7578671	*V1NB1*	KEGG_GLIOMA
4:17606956	*LCORL*	KEGG_GLUTATHIONE_METABOLISM

16:29871730	*CD19*	KEGG_GLYCEROPHOSPHOLIPID_METABOLISM
20:6011934	*CRLS1*	KEGG_GLYCEROPHOSPHOLIPID_METABOLISM
17:41056283	*RETREG3*	KEGG_GLYCOLYSIS_GLUCONEOGENESIS
6:71594785	*RIMS1*	KEGG_GLYCOSAMINOGLYCAN_BIOSYNTHESIS_CHONDROITIN_SULFATE
11:134255325	*IGSF9B*	KEGG_GLYCOSAMINOGLYCAN_BIOSYNTHESIS_CHONDROITIN_SULFATE
6:71594785	*RIMS1*	KEGG_GLYCOSAMINOGLYCAN_BIOSYNTHESIS_HEPARAN_SULFATE
11:134255325	*IGSF9B*	KEGG_GLYCOSAMINOGLYCAN_BIOSYNTHESIS_HEPARAN_SULFATE
13:97182312	*MBNL2*	KEGG_GLYCOSAMINOGLYCAN_BIOSYNTHESIS_HEPARAN_SULFATE
13:97200682	*MBNL2*	KEGG_GLYCOSAMINOGLYCAN_BIOSYNTHESIS_HEPARAN_SULFATE
13:97278310	*MBNL2*	KEGG_GLYCOSAMINOGLYCAN_BIOSYNTHESIS_HEPARAN_SULFATE
13:97441830	*MBNL2*	KEGG_GLYCOSAMINOGLYCAN_BIOSYNTHESIS_HEPARAN_SULFATE
7:65425894	*GS1-124K5.11*	KEGG_GLYCOSAMINOGLYCAN_DEGRADATION
3:160817877	*SPTSSB*	KEGG_GLYCOSPHINGOLIPID_BIOSYNTHESIS_GLOBO_SERIES
2:168682052	*STK39*	KEGG_GLYCOSPHINGOLIPID_BIOSYNTHESIS_LACTO_AND_NEOLACTO_SERIES
3:182976382	*MCCC1*	KEGG_GLYCOSPHINGOLIPID_BIOSYNTHESIS_LACTO_AND_NEOLACTO_SERIES
3:182979912	*MCCC1*	KEGG_GLYCOSPHINGOLIPID_BIOSYNTHESIS_LACTO_AND_NEOLACTO_SERIES
3:183005320	*MCCC1*	KEGG_GLYCOSPHINGOLIPID_BIOSYNTHESIS_LACTO_AND_NEOLACTO_SERIES
1:172386261	*VAMP4*	KEGG_GLYCOSYLPHOSPHATIDYLINOSITOL_GPI_ANCHOR_BIOSYNTHESIS
1:172388441	*VAMP4*	KEGG_GLYCOSYLPHOSPHATIDYLINOSITOL_GPI_ANCHOR_BIOSYNTHESIS
1:172389198	*VAMP4*	KEGG_GLYCOSYLPHOSPHATIDYLINOSITOL_GPI_ANCHOR_BIOSYNTHESIS
3:123089448	*KPNA1*	KEGG_GNRH_SIGNALING_PATHWAY
4:114424939	*CAMK2D*	KEGG_GNRH_SIGNALING_PATHWAY
4:114494052	*CAMK2D*	KEGG_GNRH_SIGNALING_PATHWAY
4:114529492	*CAMK2D*	KEGG_GNRH_SIGNALING_PATHWAY
4:114539964	*CAMK2D*	KEGG_GNRH_SIGNALING_PATHWAY
4:114597918	*CAMK2D*	KEGG_GNRH_SIGNALING_PATHWAY
8:131799767	*FAM49B*	KEGG_GNRH_SIGNALING_PATHWAY
8:131845512	*FAM49B*	KEGG_GNRH_SIGNALING_PATHWAY
8:131865425	*FAM49B*	KEGG_GNRH_SIGNALING_PATHWAY
8:131901170	*FAM49B*	KEGG_GNRH_SIGNALING_PATHWAY
16:50300511	*CASC16*	KEGG_GNRH_SIGNALING_PATHWAY
16:50317039	*CASC16*	KEGG_GNRH_SIGNALING_PATHWAY
6:29798642	*TRIM40*	KEGG_GRAFT_VERSUS_HOST_DISEASE
6:32602372	*HLA-DRB5*	KEGG_GRAFT_VERSUS_HOST_DISEASE
6:32930679	*HLA-DRB5*	KEGG_GRAFT_VERSUS_HOST_DISEASE
6:32934409	*HLA-DRB5*	KEGG_GRAFT_VERSUS_HOST_DISEASE
6:32973303	*HLA-DRB5*	KEGG_GRAFT_VERSUS_HOST_DISEASE
6:32974268	*HLA-DRB5*	KEGG_GRAFT_VERSUS_HOST_DISEASE
2:170012307	*STK39*	KEGG_HEDGEHOG_SIGNALING_PATHWAY
2:170028024	*STK39*	KEGG_HEDGEHOG_SIGNALING_PATHWAY
2:170066424	*STK39*	KEGG_HEDGEHOG_SIGNALING_PATHWAY
10:103185542	*GBF1*	KEGG_HEDGEHOG_SIGNALING_PATHWAY
10:103305087	*GBF1*	KEGG_HEDGEHOG_SIGNALING_PATHWAY
10:103308244	*GBF1*	KEGG_HEDGEHOG_SIGNALING_PATHWAY
10:104278055	*GBF1*	KEGG_HEDGEHOG_SIGNALING_PATHWAY
10:104305501	*GBF1*	KEGG_HEDGEHOG_SIGNALING_PATHWAY
17:44843651	*WNT3*	KEGG_HEDGEHOG_SIGNALING_PATHWAY
17:44870978	*WNT3*	KEGG_HEDGEHOG_SIGNALING_PATHWAY
17:44871422	*WNT3*	KEGG_HEDGEHOG_SIGNALING_PATHWAY
17:44888209	*WNT3*	KEGG_HEDGEHOG_SIGNALING_PATHWAY
17:44888715	*WNT3*	KEGG_HEDGEHOG_SIGNALING_PATHWAY
17:44906949	*WNT3*	KEGG_HEDGEHOG_SIGNALING_PATHWAY
17:44912901	*WNT3*	KEGG_HEDGEHOG_SIGNALING_PATHWAY

17:44931399	*WNT3*	KEGG_HEDGEHOG_SIGNALING_PATHWAY
17:44947756	*WNT3*	KEGG_HEDGEHOG_SIGNALING_PATHWAY
2:102612026	*MAP4K4*	KEGG_HEMATOPOIETIC_CELL_LINEAGE
2:102621855	*MAP4K4*	KEGG_HEMATOPOIETIC_CELL_LINEAGE
2:102631503	*MAP4K4*	KEGG_HEMATOPOIETIC_CELL_LINEAGE
2:102633387	*MAP4K4*	KEGG_HEMATOPOIETIC_CELL_LINEAGE
2:102720323	*MAP4K4*	KEGG_HEMATOPOIETIC_CELL_LINEAGE
2:102783052	*MAP4K4*	KEGG_HEMATOPOIETIC_CELL_LINEAGE
4:15789288	*BST1*	KEGG_HEMATOPOIETIC_CELL_LINEAGE
4:15816670	*BST1*	KEGG_HEMATOPOIETIC_CELL_LINEAGE
4:15829612	*BST1*	KEGG_HEMATOPOIETIC_CELL_LINEAGE
17:42454243	*FAM171A2*	KEGG_HEMATOPOIETIC_CELL_LINEAGE
1:161171736	*FCGR2A*	KEGG_HUNTINGTONS_DISEASE
14:55902186	*GCH1*	KEGG_HUNTINGTONS_DISEASE
17:7101771	*V1NB1*	KEGG_HUNTINGTONS_DISEASE
17:7578671	*V1NB1*	KEGG_HUNTINGTONS_DISEASE
17:7631694	*V1NB1*	KEGG_HUNTINGTONS_DISEASE
17:7682605	*V1NB1*	KEGG_HUNTINGTONS_DISEASE
17:39883520	*RETREG3*	KEGG_HUNTINGTONS_DISEASE
1:156059276	*GBAP1*	KEGG_HYPERTROPHIC_CARDIOMYOPATHY_HCM
3:49561208	*IP6K2*	KEGG_HYPERTROPHIC_CARDIOMYOPATHY_HCM
10:15562608	*ITGA8*	KEGG_HYPERTROPHIC_CARDIOMYOPATHY_HCM
10:15569598	*ITGA8*	KEGG_HYPERTROPHIC_CARDIOMYOPATHY_HCM
10:15652368	*ITGA8*	KEGG_HYPERTROPHIC_CARDIOMYOPATHY_HCM
17:42454243	*FAM171A2*	KEGG_HYPERTROPHIC_CARDIOMYOPATHY_HCM
1:226907849	*ITPKB*	KEGG_INOSITOL_PHOSPHATE_METABOLISM
1:226910340	*ITPKB*	KEGG_INOSITOL_PHOSPHATE_METABOLISM
1:226916078	*ITPKB*	KEGG_INOSITOL_PHOSPHATE_METABOLISM
16:29871730	*CD19*	KEGG_INOSITOL_PHOSPHATE_METABOLISM
17:43195981	*CRHR1_2*	KEGG_INOSITOL_PHOSPHATE_METABOLISM
3:48835245	*IP6K2*	KEGG_INSULIN_SIGNALING_PATHWAY
14:55515304	*GCH1*	KEGG_INSULIN_SIGNALING_PATHWAY
17:41056283	*RETREG3*	KEGG_INSULIN_SIGNALING_PATHWAY
6:32602372	*HLA-DRB5*	KEGG_INTESTINAL_IMMUNE_NETWORK_FOR_IGA_PRODUCTION
6:32930679	*HLA-DRB5*	KEGG_INTESTINAL_IMMUNE_NETWORK_FOR_IGA_PRODUCTION
6:32934409	*HLA-DRB5*	KEGG_INTESTINAL_IMMUNE_NETWORK_FOR_IGA_PRODUCTION
6:32973303	*HLA-DRB5*	KEGG_INTESTINAL_IMMUNE_NETWORK_FOR_IGA_PRODUCTION
6:32974268	*HLA-DRB5*	KEGG_INTESTINAL_IMMUNE_NETWORK_FOR_IGA_PRODUCTION
17:43390763	*CRHR1_2*	KEGG_INTESTINAL_IMMUNE_NETWORK_FOR_IGA_PRODUCTION
14:55515304	*GCH1*	KEGG_JAK_STAT_SIGNALING_PATHWAY
17:40392358	*RETREG3*	KEGG_JAK_STAT_SIGNALING_PATHWAY
6:32602372	*HLA-DRB5*	KEGG_LEISHMANIA_INFECTION
6:32930679	*HLA-DRB5*	KEGG_LEISHMANIA_INFECTION
6:32934409	*HLA-DRB5*	KEGG_LEISHMANIA_INFECTION
6:32973303	*HLA-DRB5*	KEGG_LEISHMANIA_INFECTION
6:32974268	*HLA-DRB5*	KEGG_LEISHMANIA_INFECTION
3:49406095	*IP6K2*	KEGG_LEUKOCYTE_TRANSENDOTHELIAL_MIGRATION
3:49424231	*IP6K2*	KEGG_LEUKOCYTE_TRANSENDOTHELIAL_MIGRATION
3:49450137	*IP6K2*	KEGG_LEUKOCYTE_TRANSENDOTHELIAL_MIGRATION
11:133947732	*IGSF9B*	KEGG_LEUKOCYTE_TRANSENDOTHELIAL_MIGRATION
16:30510571	*SETD1A*	KEGG_LEUKOCYTE_TRANSENDOTHELIAL_MIGRATION
16:30516492	*SETD1A*	KEGG_LEUKOCYTE_TRANSENDOTHELIAL_MIGRATION
16:30520856	*SETD1A*	KEGG_LEUKOCYTE_TRANSENDOTHELIAL_MIGRATION

21:37854286	*DYRK1A*	KEGG_LEUKOCYTE_TRANSENDOTHELIAL_MIGRATION
21:37859449	*DYRK1A*	KEGG_LEUKOCYTE_TRANSENDOTHELIAL_MIGRATION
21:37860785	*DYRK1A*	KEGG_LEUKOCYTE_TRANSENDOTHELIAL_MIGRATION
21:37894089	*DYRK1A*	KEGG_LEUKOCYTE_TRANSENDOTHELIAL_MIGRATION
21:37899076	*DYRK1A*	KEGG_LEUKOCYTE_TRANSENDOTHELIAL_MIGRATION
5:133558885	*C5orf24*	KEGG_LONG_TERM_DEPRESSION
17:43744203	*CRHR1_2*	KEGG_LONG_TERM_DEPRESSION
17:43748712	*CRHR1_2*	KEGG_LONG_TERM_DEPRESSION
17:43784228	*CRHR1_2*	KEGG_LONG_TERM_DEPRESSION
17:43791502	*CRHR1_2*	KEGG_LONG_TERM_DEPRESSION
17:43796210	*CRHR1_2*	KEGG_LONG_TERM_DEPRESSION
17:43843861	*CRHR1_2*	KEGG_LONG_TERM_DEPRESSION
17:43847503	*CRHR1_2*	KEGG_LONG_TERM_DEPRESSION
17:43849514	*CRHR1_2*	KEGG_LONG_TERM_DEPRESSION
4:114424939	*CAMK2D*	KEGG_LONG_TERM_POTENTIATION
4:114494052	*CAMK2D*	KEGG_LONG_TERM_POTENTIATION
4:114529492	*CAMK2D*	KEGG_LONG_TERM_POTENTIATION
4:114539964	*CAMK2D*	KEGG_LONG_TERM_POTENTIATION
4:114597918	*CAMK2D*	KEGG_LONG_TERM_POTENTIATION
8:131799767	*FAM49B*	KEGG_LONG_TERM_POTENTIATION
8:131845512	*FAM49B*	KEGG_LONG_TERM_POTENTIATION
8:131865425	*FAM49B*	KEGG_LONG_TERM_POTENTIATION
8:131901170	*FAM49B*	KEGG_LONG_TERM_POTENTIATION
1:155359992	*GBAP1*	KEGG_LYSINE_DEGRADATION
6:31862876	*HLA-DRB5*	KEGG_LYSINE_DEGRADATION
19:2172266	*SPPL2B*	KEGG_LYSINE_DEGRADATION
19:2202252	*SPPL2B*	KEGG_LYSINE_DEGRADATION
1:155205634	*GBAP1*	KEGG_LYSOSOME
3:182872860	*MCCC1*	KEGG_LYSOSOME
4:77104152	*FAM47E-STBD1*	KEGG_LYSOSOME
4:77124196	*FAM47E-STBD1*	KEGG_LYSOSOME
4:77134786	*FAM47E-STBD1*	KEGG_LYSOSOME
6:32126002	*HLA-DRB5*	KEGG_LYSOSOME
7:65425894	*GS1-124K5.11*	KEGG_LYSOSOME
8:11712443	*CTSB*	KEGG_LYSOSOME
12:123453034	*HIP1R*	KEGG_LYSOSOME
12:124219791	*HIP1R*	KEGG_LYSOSOME
14:88372366	*GALC*	KEGG_LYSOSOME
14:88393900	*GALC*	KEGG_LYSOSOME
14:88415750	*GALC*	KEGG_LYSOSOME
14:88443553	*GALC*	KEGG_LYSOSOME
19:2114694	*SPPL2B*	KEGG_LYSOSOME
19:2122458	*SPPL2B*	KEGG_LYSOSOME
2:102357447	*MAP4K4*	KEGG_MAPK_SIGNALING_PATHWAY
2:102379173	*MAP4K4*	KEGG_MAPK_SIGNALING_PATHWAY
2:102413116	*MAP4K4*	KEGG_MAPK_SIGNALING_PATHWAY
2:102612026	*MAP4K4*	KEGG_MAPK_SIGNALING_PATHWAY
2:102621855	*MAP4K4*	KEGG_MAPK_SIGNALING_PATHWAY
2:102631503	*MAP4K4*	KEGG_MAPK_SIGNALING_PATHWAY
2:102633387	*MAP4K4*	KEGG_MAPK_SIGNALING_PATHWAY
2:102720323	*MAP4K4*	KEGG_MAPK_SIGNALING_PATHWAY
2:102783052	*MAP4K4*	KEGG_MAPK_SIGNALING_PATHWAY
6:33287183	*HLA-DRB5*	KEGG_MAPK_SIGNALING_PATHWAY

17:7578671	*V1NB1*	KEGG_MAPK_SIGNALING_PATHWAY
17:41845031	*FAM171A2*	KEGG_MAPK_SIGNALING_PATHWAY
17:43390763	*CRHR1_2*	KEGG_MAPK_SIGNALING_PATHWAY
17:43999203	*WNT3*	KEGG_MAPK_SIGNALING_PATHWAY
17:44005297	*WNT3*	KEGG_MAPK_SIGNALING_PATHWAY
17:44019712	*WNT3*	KEGG_MAPK_SIGNALING_PATHWAY
17:44060492	*WNT3*	KEGG_MAPK_SIGNALING_PATHWAY
17:44065263	*WNT3*	KEGG_MAPK_SIGNALING_PATHWAY
17:44081178	*WNT3*	KEGG_MAPK_SIGNALING_PATHWAY
17:44083297	*WNT3*	KEGG_MAPK_SIGNALING_PATHWAY
19:2477820	*SPPL2B*	KEGG_MAPK_SIGNALING_PATHWAY
3:123089448	*KPNA1*	KEGG_MELANOGENESIS
4:114424939	*CAMK2D*	KEGG_MELANOGENESIS
4:114494052	*CAMK2D*	KEGG_MELANOGENESIS
4:114529492	*CAMK2D*	KEGG_MELANOGENESIS
4:114539964	*CAMK2D*	KEGG_MELANOGENESIS
4:114597918	*CAMK2D*	KEGG_MELANOGENESIS
8:131799767	*FAM49B*	KEGG_MELANOGENESIS
8:131845512	*FAM49B*	KEGG_MELANOGENESIS
8:131865425	*FAM49B*	KEGG_MELANOGENESIS
8:131901170	*FAM49B*	KEGG_MELANOGENESIS
16:50300511	*CASC16*	KEGG_MELANOGENESIS
16:50317039	*CASC16*	KEGG_MELANOGENESIS
17:7129974	*V1NB1*	KEGG_MELANOGENESIS
17:44843651	*WNT3*	KEGG_MELANOGENESIS
17:44870978	*WNT3*	KEGG_MELANOGENESIS
17:44871422	*WNT3*	KEGG_MELANOGENESIS
17:44888209	*WNT3*	KEGG_MELANOGENESIS
17:44888715	*WNT3*	KEGG_MELANOGENESIS
17:44906949	*WNT3*	KEGG_MELANOGENESIS
17:44912901	*WNT3*	KEGG_MELANOGENESIS
17:44931399	*WNT3*	KEGG_MELANOGENESIS
17:44947756	*WNT3*	KEGG_MELANOGENESIS
17:7578671	*V1NB1*	KEGG_MELANOMA
14:75495059	*RPS6KL1*	KEGG_MISMATCH_REPAIR
13:49890086	*CAB39L*	KEGG_MTOR_SIGNALING_PATHWAY
13:49897815	*CAB39L*	KEGG_MTOR_SIGNALING_PATHWAY
13:49914395	*CAB39L*	KEGG_MTOR_SIGNALING_PATHWAY
13:49927732	*CAB39L*	KEGG_MTOR_SIGNALING_PATHWAY
13:49944031	*CAB39L*	KEGG_MTOR_SIGNALING_PATHWAY
13:49960054	*CAB39L*	KEGG_MTOR_SIGNALING_PATHWAY
13:49976904	*CAB39L*	KEGG_MTOR_SIGNALING_PATHWAY
13:50013818	*CAB39L*	KEGG_MTOR_SIGNALING_PATHWAY
6:29798642	*TRIM40*	KEGG_NATURAL_KILLER_CELL_MEDIATED_CYTOTOXICITY
6:112137847	*FYN*	KEGG_NATURAL_KILLER_CELL_MEDIATED_CYTOTOXICITY
6:112151452	*FYN*	KEGG_NATURAL_KILLER_CELL_MEDIATED_CYTOTOXICITY
6:112175069	*FYN*	KEGG_NATURAL_KILLER_CELL_MEDIATED_CYTOTOXICITY
8:22974278	*BIN3*	KEGG_NATURAL_KILLER_CELL_MEDIATED_CYTOTOXICITY
8:23050400	*BIN3*	KEGG_NATURAL_KILLER_CELL_MEDIATED_CYTOTOXICITY
16:30510571	*SETD1A*	KEGG_NATURAL_KILLER_CELL_MEDIATED_CYTOTOXICITY
16:30516492	*SETD1A*	KEGG_NATURAL_KILLER_CELL_MEDIATED_CYTOTOXICITY
16:30520856	*SETD1A*	KEGG_NATURAL_KILLER_CELL_MEDIATED_CYTOTOXICITY
3:150935203	*MED12L*	KEGG_NEUROACTIVE_LIGAND_RECEPTOR_INTERACTION

6:29565889	*TRIM40*	KEGG_NEUROACTIVE_LIGAND_RECEPTOR_INTERACTION
6:29590715	*TRIM40*	KEGG_NEUROACTIVE_LIGAND_RECEPTOR_INTERACTION
9:33791164	*UBAP2*	KEGG_NEUROACTIVE_LIGAND_RECEPTOR_INTERACTION
17:7352976	*V1NB1*	KEGG_NEUROACTIVE_LIGAND_RECEPTOR_INTERACTION
17:43744203	*CRHR1_2*	KEGG_NEUROACTIVE_LIGAND_RECEPTOR_INTERACTION
17:43748712	*CRHR1_2*	KEGG_NEUROACTIVE_LIGAND_RECEPTOR_INTERACTION
17:43784228	*CRHR1_2*	KEGG_NEUROACTIVE_LIGAND_RECEPTOR_INTERACTION
17:43791502	*CRHR1_2*	KEGG_NEUROACTIVE_LIGAND_RECEPTOR_INTERACTION
17:43796210	*CRHR1_2*	KEGG_NEUROACTIVE_LIGAND_RECEPTOR_INTERACTION
17:43843861	*CRHR1_2*	KEGG_NEUROACTIVE_LIGAND_RECEPTOR_INTERACTION
17:43847503	*CRHR1_2*	KEGG_NEUROACTIVE_LIGAND_RECEPTOR_INTERACTION
17:43849514	*CRHR1_2*	KEGG_NEUROACTIVE_LIGAND_RECEPTOR_INTERACTION
3:49406095	*IP6K2*	KEGG_NEUROTROPHIN_SIGNALING_PATHWAY
3:49424231	*IP6K2*	KEGG_NEUROTROPHIN_SIGNALING_PATHWAY
3:49450137	*IP6K2*	KEGG_NEUROTROPHIN_SIGNALING_PATHWAY
4:114424939	*CAMK2D*	KEGG_NEUROTROPHIN_SIGNALING_PATHWAY
4:114494052	*CAMK2D*	KEGG_NEUROTROPHIN_SIGNALING_PATHWAY
4:114529492	*CAMK2D*	KEGG_NEUROTROPHIN_SIGNALING_PATHWAY
4:114539964	*CAMK2D*	KEGG_NEUROTROPHIN_SIGNALING_PATHWAY
4:114597918	*CAMK2D*	KEGG_NEUROTROPHIN_SIGNALING_PATHWAY
17:7578671	*V1NB1*	KEGG_NEUROTROPHIN_SIGNALING_PATHWAY
2:134902070	*TMEM163*	KEGG_N_GLYCAN_BIOSYNTHESIS
2:134932210	*TMEM163*	KEGG_N_GLYCAN_BIOSYNTHESIS
2:134956835	*TMEM163*	KEGG_N_GLYCAN_BIOSYNTHESIS
2:134986578	*TMEM163*	KEGG_N_GLYCAN_BIOSYNTHESIS
2:134993021	*TMEM163*	KEGG_N_GLYCAN_BIOSYNTHESIS
2:135035038	*TMEM163*	KEGG_N_GLYCAN_BIOSYNTHESIS
2:135040234	*TMEM163*	KEGG_N_GLYCAN_BIOSYNTHESIS
2:135165078	*TMEM163*	KEGG_N_GLYCAN_BIOSYNTHESIS
2:135166385	*TMEM163*	KEGG_N_GLYCAN_BIOSYNTHESIS
4:15709252	*BST1*	KEGG_NICOTINATE_AND_NICOTINAMIDE_METABOLISM
4:15713608	*BST1*	KEGG_NICOTINATE_AND_NICOTINAMIDE_METABOLISM
4:15724372	*BST1*	KEGG_NICOTINATE_AND_NICOTINAMIDE_METABOLISM
4:15737882	*BST1*	KEGG_NICOTINATE_AND_NICOTINAMIDE_METABOLISM
4:15789288	*BST1*	KEGG_NICOTINATE_AND_NICOTINAMIDE_METABOLISM
4:15816670	*BST1*	KEGG_NICOTINATE_AND_NICOTINAMIDE_METABOLISM
4:15829612	*BST1*	KEGG_NICOTINATE_AND_NICOTINAMIDE_METABOLISM
5:102889674	*PAM*	KEGG_NICOTINATE_AND_NICOTINAMIDE_METABOLISM
10:104847713	*GBF1*	KEGG_NICOTINATE_AND_NICOTINAMIDE_METABOLISM
10:104861736	*GBF1*	KEGG_NICOTINATE_AND_NICOTINAMIDE_METABOLISM
10:104926485	*GBF1*	KEGG_NICOTINATE_AND_NICOTINAMIDE_METABOLISM
10:104929716	*GBF1*	KEGG_NICOTINATE_AND_NICOTINAMIDE_METABOLISM
16:50730088	*CASC16*	KEGG_NOD_LIKE_RECEPTOR_SIGNALING_PATHWAY
16:50730446	*CASC16*	KEGG_NOD_LIKE_RECEPTOR_SIGNALING_PATHWAY
16:50745926	*CASC16*	KEGG_NOD_LIKE_RECEPTOR_SIGNALING_PATHWAY
17:7578671	*V1NB1*	KEGG_NON_SMALL_CELL_LUNG_CANCER
1:227067656	*ITPKB*	KEGG_NOTCH_SIGNALING_PATHWAY
3:122289921	*KPNA1*	KEGG_NOTCH_SIGNALING_PATHWAY
6:32179319	*HLA-DRB5*	KEGG_NOTCH_SIGNALING_PATHWAY
6:32186193	*HLA-DRB5*	KEGG_NOTCH_SIGNALING_PATHWAY
17:7129974	*V1NB1*	KEGG_NOTCH_SIGNALING_PATHWAY
12:133206648	*FBRSL1*	KEGG_NUCLEOTIDE_EXCISION_REPAIR
12:133223685	*FBRSL1*	KEGG_NUCLEOTIDE_EXCISION_REPAIR

11:11299050	*RNF141*	KEGG_O_GLYCAN_BIOSYNTHESIS
11:11316049	*RNF141*	KEGG_O_GLYCAN_BIOSYNTHESIS
11:11364660	*RNF141*	KEGG_O_GLYCAN_BIOSYNTHESIS
11:11399582	*RNF141*	KEGG_O_GLYCAN_BIOSYNTHESIS
11:11412180	*RNF141*	KEGG_O_GLYCAN_BIOSYNTHESIS
11:11494181	*RNF141*	KEGG_O_GLYCAN_BIOSYNTHESIS
11:11504200	*RNF141*	KEGG_O_GLYCAN_BIOSYNTHESIS
11:11508780	*RNF141*	KEGG_O_GLYCAN_BIOSYNTHESIS
11:11519365	*RNF141*	KEGG_O_GLYCAN_BIOSYNTHESIS
12:132687184	*FBRSL1*	KEGG_O_GLYCAN_BIOSYNTHESIS
4:114424939	*CAMK2D*	KEGG_OLFACTORY_TRANSDUCTION
4:114494052	*CAMK2D*	KEGG_OLFACTORY_TRANSDUCTION
4:114529492	*CAMK2D*	KEGG_OLFACTORY_TRANSDUCTION
4:114539964	*CAMK2D*	KEGG_OLFACTORY_TRANSDUCTION
4:114597918	*CAMK2D*	KEGG_OLFACTORY_TRANSDUCTION
6:29373213	*TRIM40*	KEGG_OLFACTORY_TRANSDUCTION
6:29407378	*TRIM40*	KEGG_OLFACTORY_TRANSDUCTION
6:29412222	*TRIM40*	KEGG_OLFACTORY_TRANSDUCTION
3:123089448	*KPNA1*	KEGG_OOCYTE_MEIOSIS
4:114424939	*CAMK2D*	KEGG_OOCYTE_MEIOSIS
4:114494052	*CAMK2D*	KEGG_OOCYTE_MEIOSIS
4:114529492	*CAMK2D*	KEGG_OOCYTE_MEIOSIS
4:114539964	*CAMK2D*	KEGG_OOCYTE_MEIOSIS
4:114597918	*CAMK2D*	KEGG_OOCYTE_MEIOSIS
5:133558885	*C5orf24*	KEGG_OOCYTE_MEIOSIS
8:131799767	*FAM49B*	KEGG_OOCYTE_MEIOSIS
8:131845512	*FAM49B*	KEGG_OOCYTE_MEIOSIS
8:131865425	*FAM49B*	KEGG_OOCYTE_MEIOSIS
8:131901170	*FAM49B*	KEGG_OOCYTE_MEIOSIS
10:103185542	*GBF1*	KEGG_OOCYTE_MEIOSIS
10:103305087	*GBF1*	KEGG_OOCYTE_MEIOSIS
10:103308244	*GBF1*	KEGG_OOCYTE_MEIOSIS
16:50300511	*CASC16*	KEGG_OOCYTE_MEIOSIS
16:50317039	*CASC16*	KEGG_OOCYTE_MEIOSIS
17:45215865	*WNT3*	KEGG_OOCYTE_MEIOSIS
17:45244822	*WNT3*	KEGG_OOCYTE_MEIOSIS
1:155205634	*GBAP1*	KEGG_OTHER_GLYCAN_DEGRADATION
1:161171736	*FCGR2A*	KEGG_OXIDATIVE_PHOSPHORYLATION
12:124219791	*HIP1R*	KEGG_OXIDATIVE_PHOSPHORYLATION
4:76407998	*FAM47E-STBD1*	KEGG_P53_SIGNALING_PATHWAY
4:78136299	*FAM47E-STBD1*	KEGG_P53_SIGNALING_PATHWAY
4:78175384	*FAM47E-STBD1*	KEGG_P53_SIGNALING_PATHWAY
17:7578671	*V1NB1*	KEGG_P53_SIGNALING_PATHWAY
19:2477820	*SPPL2B*	KEGG_P53_SIGNALING_PATHWAY
17:7578671	*V1NB1*	KEGG_PANCREATIC_CANCER
6:133025715	*RPS12*	KEGG_PANTOTHENATE_AND_COA_BIOSYNTHESIS
6:133028870	*RPS12*	KEGG_PANTOTHENATE_AND_COA_BIOSYNTHESIS
6:133075265	*RPS12*	KEGG_PANTOTHENATE_AND_COA_BIOSYNTHESIS
1:161171736	*FCGR2A*	KEGG_PARKINSONS_DISEASE
4:90677243	*SNCA_2*	KEGG_PARKINSONS_DISEASE
4:90700329	*SNCA_2*	KEGG_PARKINSONS_DISEASE
4:90703993	*SNCA_2*	KEGG_PARKINSONS_DISEASE
4:90738839	*SNCA_2*	KEGG_PARKINSONS_DISEASE

4:90757294	*SNCA_2*	KEGG_PARKINSONS_DISEASE
12:40614434	*LRRK2_2*	KEGG_PARKINSONS_DISEASE
12:40614656	*LRRK2_2*	KEGG_PARKINSONS_DISEASE
12:40617231	*LRRK2_2*	KEGG_PARKINSONS_DISEASE
12:40653993	*LRRK2_2*	KEGG_PARKINSONS_DISEASE
12:40698422	*LRRK2_2*	KEGG_PARKINSONS_DISEASE
12:40707861	*LRRK2_2*	KEGG_PARKINSONS_DISEASE
12:40734202	*LRRK2_2*	KEGG_PARKINSONS_DISEASE
12:40753796	*LRRK2_2*	KEGG_PARKINSONS_DISEASE
1:155975202	*GBAP1*	KEGG_PATHOGENIC_ESCHERICHIA_COLI_INFECTION
3:49406095	*IP6K2*	KEGG_PATHOGENIC_ESCHERICHIA_COLI_INFECTION
3:49424231	*IP6K2*	KEGG_PATHOGENIC_ESCHERICHIA_COLI_INFECTION
3:49450137	*IP6K2*	KEGG_PATHOGENIC_ESCHERICHIA_COLI_INFECTION
6:112137847	*FYN*	KEGG_PATHOGENIC_ESCHERICHIA_COLI_INFECTION
6:112151452	*FYN*	KEGG_PATHOGENIC_ESCHERICHIA_COLI_INFECTION
6:112175069	*FYN*	KEGG_PATHOGENIC_ESCHERICHIA_COLI_INFECTION
3:49162284	*IP6K2*	KEGG_PATHWAYS_IN_CANCER
3:49162583	*IP6K2*	KEGG_PATHWAYS_IN_CANCER
3:49406095	*IP6K2*	KEGG_PATHWAYS_IN_CANCER
3:49424231	*IP6K2*	KEGG_PATHWAYS_IN_CANCER
3:49450137	*IP6K2*	KEGG_PATHWAYS_IN_CANCER
6:112494762	*FYN*	KEGG_PATHWAYS_IN_CANCER
6:112531401	*FYN*	KEGG_PATHWAYS_IN_CANCER
6:112561154	*FYN*	KEGG_PATHWAYS_IN_CANCER
6:112561430	*FYN*	KEGG_PATHWAYS_IN_CANCER
10:104278055	*GBF1*	KEGG_PATHWAYS_IN_CANCER
10:104305501	*GBF1*	KEGG_PATHWAYS_IN_CANCER
17:7129974	*V1NB1*	KEGG_PATHWAYS_IN_CANCER
17:7578671	*V1NB1*	KEGG_PATHWAYS_IN_CANCER
17:39883520	*RETREG3*	KEGG_PATHWAYS_IN_CANCER
17:40392358	*RETREG3*	KEGG_PATHWAYS_IN_CANCER
17:42454243	*FAM171A2*	KEGG_PATHWAYS_IN_CANCER
17:44843651	*WNT3*	KEGG_PATHWAYS_IN_CANCER
17:44870978	*WNT3*	KEGG_PATHWAYS_IN_CANCER
17:44871422	*WNT3*	KEGG_PATHWAYS_IN_CANCER
17:44888209	*WNT3*	KEGG_PATHWAYS_IN_CANCER
17:44888715	*WNT3*	KEGG_PATHWAYS_IN_CANCER
17:44906949	*WNT3*	KEGG_PATHWAYS_IN_CANCER
17:44912901	*WNT3*	KEGG_PATHWAYS_IN_CANCER
17:44931399	*WNT3*	KEGG_PATHWAYS_IN_CANCER
17:44947756	*WNT3*	KEGG_PATHWAYS_IN_CANCER
7:65425894	*GS1-124K5.11*	KEGG_PENTOSE_AND_GLUCURONATE_INTERCONVERSIONS
5:102889674	*PAM*	KEGG_PEROXISOME
12:39960237	*LRRK2_2*	KEGG_PEROXISOME
12:133295501	*FBRSL1*	KEGG_PEROXISOME
14:74764925	*RPS6KL1*	KEGG_PEROXISOME
1:226907849	*ITPKB*	KEGG_PHOSPHATIDYLINOSITOL_SIGNALING_SYSTEM
1:226910340	*ITPKB*	KEGG_PHOSPHATIDYLINOSITOL_SIGNALING_SYSTEM
1:226916078	*ITPKB*	KEGG_PHOSPHATIDYLINOSITOL_SIGNALING_SYSTEM
16:29871730	*CD19*	KEGG_PHOSPHATIDYLINOSITOL_SIGNALING_SYSTEM
17:43195981	*CRHR1_2*	KEGG_PHOSPHATIDYLINOSITOL_SIGNALING_SYSTEM
7:65425894	*GS1-124K5.11*	KEGG_PORPHYRIN_AND_CHLOROPHYLL_METABOLISM
16:30999462	*SETD1A*	KEGG_PRIMARY_BILE_ACID_BIOSYNTHESIS

6:32789400	*HLA-DRB5*	KEGG_PRIMARY_IMMUNODEFICIENCY
6:32791831	*HLA-DRB5*	KEGG_PRIMARY_IMMUNODEFICIENCY
6:32797361	*HLA-DRB5*	KEGG_PRIMARY_IMMUNODEFICIENCY
6:32802462	*HLA-DRB5*	KEGG_PRIMARY_IMMUNODEFICIENCY
6:112137847	*FYN*	KEGG_PRION_DISEASES
6:112151452	*FYN*	KEGG_PRION_DISEASES
6:112175069	*FYN*	KEGG_PRION_DISEASES
3:48199695	*IP6K2*	KEGG_PROGESTERONE_MEDIATED_OOCYTE_MATURATION
3:48214438	*IP6K2*	KEGG_PROGESTERONE_MEDIATED_OOCYTE_MATURATION
3:123089448	*KPNA1*	KEGG_PROGESTERONE_MEDIATED_OOCYTE_MATURATION
8:131799767	*FAM49B*	KEGG_PROGESTERONE_MEDIATED_OOCYTE_MATURATION
8:131845512	*FAM49B*	KEGG_PROGESTERONE_MEDIATED_OOCYTE_MATURATION
8:131865425	*FAM49B*	KEGG_PROGESTERONE_MEDIATED_OOCYTE_MATURATION
8:131901170	*FAM49B*	KEGG_PROGESTERONE_MEDIATED_OOCYTE_MATURATION
16:50300511	*CASC16*	KEGG_PROGESTERONE_MEDIATED_OOCYTE_MATURATION
16:50317039	*CASC16*	KEGG_PROGESTERONE_MEDIATED_OOCYTE_MATURATION
17:45215865	*WNT3*	KEGG_PROGESTERONE_MEDIATED_OOCYTE_MATURATION
17:45244822	*WNT3*	KEGG_PROGESTERONE_MEDIATED_OOCYTE_MATURATION
17:7578671	*V1NB1*	KEGG_PROSTATE_CANCER
17:7560294	*V1NB1*	KEGG_PROXIMAL_TUBULE_BICARBONATE_RECLAMATION
3:123089448	*KPNA1*	KEGG_PURINE_METABOLISM
5:59236972	*ELOVL7*	KEGG_PURINE_METABOLISM
5:59490965	*ELOVL7*	KEGG_PURINE_METABOLISM
5:59554719	*ELOVL7*	KEGG_PURINE_METABOLISM
5:59705316	*ELOVL7*	KEGG_PURINE_METABOLISM
5:59736085	*ELOVL7*	KEGG_PURINE_METABOLISM
5:59812853	*ELOVL7*	KEGG_PURINE_METABOLISM
8:22108784	*BIN3*	KEGG_PURINE_METABOLISM
8:131799767	*FAM49B*	KEGG_PURINE_METABOLISM
8:131845512	*FAM49B*	KEGG_PURINE_METABOLISM
8:131865425	*FAM49B*	KEGG_PURINE_METABOLISM
8:131901170	*FAM49B*	KEGG_PURINE_METABOLISM
9:34342881	*UBAP2*	KEGG_PURINE_METABOLISM
10:104847713	*GBF1*	KEGG_PURINE_METABOLISM
10:104861736	*GBF1*	KEGG_PURINE_METABOLISM
10:104926485	*GBF1*	KEGG_PURINE_METABOLISM
10:104929716	*GBF1*	KEGG_PURINE_METABOLISM
11:10392432	*RNF141*	KEGG_PURINE_METABOLISM
11:10429998	*RNF141*	KEGG_PURINE_METABOLISM
11:10458098	*RNF141*	KEGG_PURINE_METABOLISM
11:10471738	*RNF141*	KEGG_PURINE_METABOLISM
11:10487997	*RNF141*	KEGG_PURINE_METABOLISM
11:10494132	*RNF141*	KEGG_PURINE_METABOLISM
12:133206648	*FBRSL1*	KEGG_PURINE_METABOLISM
12:133223685	*FBRSL1*	KEGG_PURINE_METABOLISM
14:74477206	*RPS6KL1*	KEGG_PURINE_METABOLISM
16:50300511	*CASC16*	KEGG_PURINE_METABOLISM
16:50317039	*CASC16*	KEGG_PURINE_METABOLISM
8:22108784	*BIN3*	KEGG_PYRIMIDINE_METABOLISM
9:34342881	*UBAP2*	KEGG_PYRIMIDINE_METABOLISM
10:104847713	*GBF1*	KEGG_PYRIMIDINE_METABOLISM
10:104861736	*GBF1*	KEGG_PYRIMIDINE_METABOLISM
10:104926485	*GBF1*	KEGG_PYRIMIDINE_METABOLISM

10:104929716	*GBF1*	KEGG_PYRIMIDINE_METABOLISM
12:133206648	*FBRSL1*	KEGG_PYRIMIDINE_METABOLISM
12:133223685	*FBRSL1*	KEGG_PYRIMIDINE_METABOLISM
14:74477206	*RPS6KL1*	KEGG_PYRIMIDINE_METABOLISM
17:76177567	*DNAH17*	KEGG_PYRIMIDINE_METABOLISM
3:49406095	*IP6K2*	KEGG_REGULATION_OF_ACTIN_CYTOSKELETON
3:49424231	*IP6K2*	KEGG_REGULATION_OF_ACTIN_CYTOSKELETON
3:49450137	*IP6K2*	KEGG_REGULATION_OF_ACTIN_CYTOSKELETON
10:15562608	*ITGA8*	KEGG_REGULATION_OF_ACTIN_CYTOSKELETON
10:15569598	*ITGA8*	KEGG_REGULATION_OF_ACTIN_CYTOSKELETON
10:15652368	*ITGA8*	KEGG_REGULATION_OF_ACTIN_CYTOSKELETON
16:30510571	*SETD1A*	KEGG_REGULATION_OF_ACTIN_CYTOSKELETON
16:30516492	*SETD1A*	KEGG_REGULATION_OF_ACTIN_CYTOSKELETON
16:30520856	*SETD1A*	KEGG_REGULATION_OF_ACTIN_CYTOSKELETON
16:31370452	*SETD1A*	KEGG_REGULATION_OF_ACTIN_CYTOSKELETON
17:42454243	*FAM171A2*	KEGG_REGULATION_OF_ACTIN_CYTOSKELETON
8:17161374	*FGF20*	KEGG_RIBOFLAVIN_METABOLISM
8:17176046	*FGF20*	KEGG_RIBOFLAVIN_METABOLISM
8:17243260	*FGF20*	KEGG_RIBOFLAVIN_METABOLISM
8:17260937	*FGF20*	KEGG_RIBOFLAVIN_METABOLISM
16:50790891	*CASC16*	KEGG_RIG_I_LIKE_RECEPTOR_SIGNALING_PATHWAY
8:22108784	*BIN3*	KEGG_RNA_POLYMERASE
3:49162284	*IP6K2*	KEGG_SMALL_CELL_LUNG_CANCER
3:49162583	*IP6K2*	KEGG_SMALL_CELL_LUNG_CANCER
6:112494762	*FYN*	KEGG_SMALL_CELL_LUNG_CANCER
6:112531401	*FYN*	KEGG_SMALL_CELL_LUNG_CANCER
6:112561154	*FYN*	KEGG_SMALL_CELL_LUNG_CANCER
6:112561430	*FYN*	KEGG_SMALL_CELL_LUNG_CANCER
17:7578671	*V1NB1*	KEGG_SMALL_CELL_LUNG_CANCER
17:42454243	*FAM171A2*	KEGG_SMALL_CELL_LUNG_CANCER
1:171689486	*VAMP4*	KEGG_SNARE_INTERACTIONS_IN_VESICULAR_TRANSPORT
6:132785797	*RPS12*	KEGG_SNARE_INTERACTIONS_IN_VESICULAR_TRANSPORT
6:132830746	*RPS12*	KEGG_SNARE_INTERACTIONS_IN_VESICULAR_TRANSPORT
16:31004169	*SETD1A*	KEGG_SNARE_INTERACTIONS_IN_VESICULAR_TRANSPORT
17:45020426	*WNT3*	KEGG_SNARE_INTERACTIONS_IN_VESICULAR_TRANSPORT
17:45059032	*WNT3*	KEGG_SNARE_INTERACTIONS_IN_VESICULAR_TRANSPORT
17:45089680	*WNT3*	KEGG_SNARE_INTERACTIONS_IN_VESICULAR_TRANSPORT
1:155205634	*GBAP1*	KEGG_SPHINGOLIPID_METABOLISM
14:88372366	*GALC*	KEGG_SPHINGOLIPID_METABOLISM
14:88393900	*GALC*	KEGG_SPHINGOLIPID_METABOLISM
14:88415750	*GALC*	KEGG_SPHINGOLIPID_METABOLISM
14:88443553	*GALC*	KEGG_SPHINGOLIPID_METABOLISM
5:134132269	*C5orf24*	KEGG_SPLICEOSOME
6:30628082	*TRIM40*	KEGG_SPLICEOSOME
6:30631063	*TRIM40*	KEGG_SPLICEOSOME
6:30639369	*TRIM40*	KEGG_SPLICEOSOME
17:41575811	*FAM171A2*	KEGG_SPLICEOSOME
17:42929985	*CRHR1_2*	KEGG_SPLICEOSOME
7:65425894	*GS1-124K5.11*	KEGG_STARCH_AND_SUCROSE_METABOLISM
17:41056283	*RETREG3*	KEGG_STARCH_AND_SUCROSE_METABOLISM
8:11679472	*CTSB*	KEGG_STEROID_BIOSYNTHESIS
10:104591639	*GBF1*	KEGG_STEROID_HORMONE_BIOSYNTHESIS
5:134722833	*C5orf24*	KEGG_SYSTEMIC_LUPUS_ERYTHEMATOSUS

6:31895092	*HLA-DRB5*	KEGG_SYSTEMIC_LUPUS_ERYTHEMATOSUS
6:31897219	*HLA-DRB5*	KEGG_SYSTEMIC_LUPUS_ERYTHEMATOSUS
6:32602372	*HLA-DRB5*	KEGG_SYSTEMIC_LUPUS_ERYTHEMATOSUS
6:32930679	*HLA-DRB5*	KEGG_SYSTEMIC_LUPUS_ERYTHEMATOSUS
6:32934409	*HLA-DRB5*	KEGG_SYSTEMIC_LUPUS_ERYTHEMATOSUS
6:32973303	*HLA-DRB5*	KEGG_SYSTEMIC_LUPUS_ERYTHEMATOSUS
6:32974268	*HLA-DRB5*	KEGG_SYSTEMIC_LUPUS_ERYTHEMATOSUS
8:131799767	*FAM49B*	KEGG_TASTE_TRANSDUCTION
8:131845512	*FAM49B*	KEGG_TASTE_TRANSDUCTION
8:131865425	*FAM49B*	KEGG_TASTE_TRANSDUCTION
8:131901170	*FAM49B*	KEGG_TASTE_TRANSDUCTION
3:49406095	*IP6K2*	KEGG_T_CELL_RECEPTOR_SIGNALING_PATHWAY
3:49424231	*IP6K2*	KEGG_T_CELL_RECEPTOR_SIGNALING_PATHWAY
3:49450137	*IP6K2*	KEGG_T_CELL_RECEPTOR_SIGNALING_PATHWAY
6:112137847	*FYN*	KEGG_T_CELL_RECEPTOR_SIGNALING_PATHWAY
6:112151452	*FYN*	KEGG_T_CELL_RECEPTOR_SIGNALING_PATHWAY
6:112175069	*FYN*	KEGG_T_CELL_RECEPTOR_SIGNALING_PATHWAY
17:43390763	*CRHR1_2*	KEGG_T_CELL_RECEPTOR_SIGNALING_PATHWAY
1:226074321	*ITPKB*	KEGG_TGF_BETA_SIGNALING_PATHWAY
3:49406095	*IP6K2*	KEGG_TGF_BETA_SIGNALING_PATHWAY
3:49424231	*IP6K2*	KEGG_TGF_BETA_SIGNALING_PATHWAY
3:49450137	*IP6K2*	KEGG_TGF_BETA_SIGNALING_PATHWAY
5:133558885	*C5orf24*	KEGG_TGF_BETA_SIGNALING_PATHWAY
19:2251817	*SPPL2B*	KEGG_TGF_BETA_SIGNALING_PATHWAY
17:7578671	*V1NB1*	KEGG_THYROID_CANCER
1:155359992	*GBAP1*	KEGG_TIGHT_JUNCTION
3:49406095	*IP6K2*	KEGG_TIGHT_JUNCTION
3:49424231	*IP6K2*	KEGG_TIGHT_JUNCTION
3:49450137	*IP6K2*	KEGG_TIGHT_JUNCTION
5:133558885	*C5orf24*	KEGG_TIGHT_JUNCTION
11:133947732	*IGSF9B*	KEGG_TIGHT_JUNCTION
21:37854286	*DYRK1A*	KEGG_TIGHT_JUNCTION
21:37859449	*DYRK1A*	KEGG_TIGHT_JUNCTION
21:37860785	*DYRK1A*	KEGG_TIGHT_JUNCTION
21:37894089	*DYRK1A*	KEGG_TIGHT_JUNCTION
21:37899076	*DYRK1A*	KEGG_TIGHT_JUNCTION
4:76924571	*FAM47E-STBD1*	KEGG_TOLL_LIKE_RECEPTOR_SIGNALING_PATHWAY
6:29798642	*TRIM40*	KEGG_TYPE_I_DIABETES_MELLITUS
6:32602372	*HLA-DRB5*	KEGG_TYPE_I_DIABETES_MELLITUS
6:32930679	*HLA-DRB5*	KEGG_TYPE_I_DIABETES_MELLITUS
6:32934409	*HLA-DRB5*	KEGG_TYPE_I_DIABETES_MELLITUS
6:32973303	*HLA-DRB5*	KEGG_TYPE_I_DIABETES_MELLITUS
6:32974268	*HLA-DRB5*	KEGG_TYPE_I_DIABETES_MELLITUS
14:55515304	*GCH1*	KEGG_TYPE_II_DIABETES_MELLITUS
4:76407998	*FAM47E-STBD1*	KEGG_UBIQUITIN_MEDIATED_PROTEOLYSIS
5:133710339	*C5orf24*	KEGG_UBIQUITIN_MEDIATED_PROTEOLYSIS
9:33845203	*UBAP2*	KEGG_UBIQUITIN_MEDIATED_PROTEOLYSIS
10:103185542	*GBF1*	KEGG_UBIQUITIN_MEDIATED_PROTEOLYSIS
10:103305087	*GBF1*	KEGG_UBIQUITIN_MEDIATED_PROTEOLYSIS
10:103308244	*GBF1*	KEGG_UBIQUITIN_MEDIATED_PROTEOLYSIS
17:45215865	*WNT3*	KEGG_UBIQUITIN_MEDIATED_PROTEOLYSIS
17:45244822	*WNT3*	KEGG_UBIQUITIN_MEDIATED_PROTEOLYSIS
6:31758240	*HLA-DRB5*	KEGG_VALINE_LEUCINE_AND_ISOLEUCINE_BIOSYNTHESIS

6:31762311	*HLA-DRB5*	KEGG_VALINE_LEUCINE_AND_ISOLEUCINE_BIOSYNTHESIS
3:182760073	*MCCC1*	KEGG_VALINE_LEUCINE_AND_ISOLEUCINE_DEGRADATION
11:134130174	*IGSF9B*	KEGG_VALINE_LEUCINE_AND_ISOLEUCINE_DEGRADATION
3:49406095	*IP6K2*	KEGG_VASCULAR_SMOOTH_MUSCLE_CONTRACTION
3:49424231	*IP6K2*	KEGG_VASCULAR_SMOOTH_MUSCLE_CONTRACTION
3:49450137	*IP6K2*	KEGG_VASCULAR_SMOOTH_MUSCLE_CONTRACTION
3:123089448	*KPNA1*	KEGG_VASCULAR_SMOOTH_MUSCLE_CONTRACTION
8:131799767	*FAM49B*	KEGG_VASCULAR_SMOOTH_MUSCLE_CONTRACTION
8:131845512	*FAM49B*	KEGG_VASCULAR_SMOOTH_MUSCLE_CONTRACTION
8:131865425	*FAM49B*	KEGG_VASCULAR_SMOOTH_MUSCLE_CONTRACTION
8:131901170	*FAM49B*	KEGG_VASCULAR_SMOOTH_MUSCLE_CONTRACTION
11:10647977	*RNF141*	KEGG_VASCULAR_SMOOTH_MUSCLE_CONTRACTION
11:10648481	*RNF141*	KEGG_VASCULAR_SMOOTH_MUSCLE_CONTRACTION
11:10660840	*RNF141*	KEGG_VASCULAR_SMOOTH_MUSCLE_CONTRACTION
16:50300511	*CASC16*	KEGG_VASCULAR_SMOOTH_MUSCLE_CONTRACTION
16:50317039	*CASC16*	KEGG_VASCULAR_SMOOTH_MUSCLE_CONTRACTION
9:33447547	*UBAP2*	KEGG_VASOPRESSIN_REGULATED_WATER_REABSORPTION
17:44800946	*WNT3*	KEGG_VASOPRESSIN_REGULATED_WATER_REABSORPTION
17:44833217	*WNT3*	KEGG_VASOPRESSIN_REGULATED_WATER_REABSORPTION
12:124219791	*HIP1R*	KEGG_VIBRIO_CHOLERAE_INFECTION
3:49561208	*IP6K2*	KEGG_VIRAL_MYOCARDITIS
6:29798642	*TRIM40*	KEGG_VIRAL_MYOCARDITIS
6:32602372	*HLA-DRB5*	KEGG_VIRAL_MYOCARDITIS
6:32930679	*HLA-DRB5*	KEGG_VIRAL_MYOCARDITIS
6:32934409	*HLA-DRB5*	KEGG_VIRAL_MYOCARDITIS
6:32973303	*HLA-DRB5*	KEGG_VIRAL_MYOCARDITIS
6:32974268	*HLA-DRB5*	KEGG_VIRAL_MYOCARDITIS
6:112137847	*FYN*	KEGG_VIRAL_MYOCARDITIS
6:112151452	*FYN*	KEGG_VIRAL_MYOCARDITIS
6:112175069	*FYN*	KEGG_VIRAL_MYOCARDITIS
16:30510571	*SETD1A*	KEGG_VIRAL_MYOCARDITIS
16:30516492	*SETD1A*	KEGG_VIRAL_MYOCARDITIS
16:30520856	*SETD1A*	KEGG_VIRAL_MYOCARDITIS
3:49406095	*IP6K2*	KEGG_WNT_SIGNALING_PATHWAY
3:49424231	*IP6K2*	KEGG_WNT_SIGNALING_PATHWAY
3:49450137	*IP6K2*	KEGG_WNT_SIGNALING_PATHWAY
4:114424939	*CAMK2D*	KEGG_WNT_SIGNALING_PATHWAY
4:114494052	*CAMK2D*	KEGG_WNT_SIGNALING_PATHWAY
4:114529492	*CAMK2D*	KEGG_WNT_SIGNALING_PATHWAY
4:114539964	*CAMK2D*	KEGG_WNT_SIGNALING_PATHWAY
4:114597918	*CAMK2D*	KEGG_WNT_SIGNALING_PATHWAY
5:133558885	*C5orf24*	KEGG_WNT_SIGNALING_PATHWAY
10:103185542	*GBF1*	KEGG_WNT_SIGNALING_PATHWAY
10:103305087	*GBF1*	KEGG_WNT_SIGNALING_PATHWAY
10:103308244	*GBF1*	KEGG_WNT_SIGNALING_PATHWAY
16:50612930	*CASC16*	KEGG_WNT_SIGNALING_PATHWAY
16:50643086	*CASC16*	KEGG_WNT_SIGNALING_PATHWAY
17:7129974	*V1NB1*	KEGG_WNT_SIGNALING_PATHWAY
17:7578671	*V1NB1*	KEGG_WNT_SIGNALING_PATHWAY
17:44843651	*WNT3*	KEGG_WNT_SIGNALING_PATHWAY
17:44870978	*WNT3*	KEGG_WNT_SIGNALING_PATHWAY
17:44871422	*WNT3*	KEGG_WNT_SIGNALING_PATHWAY
17:44888209	*WNT3*	KEGG_WNT_SIGNALING_PATHWAY

17:44888715	*WNT3*	KEGG_WNT_SIGNALING_PATHWAY
17:44906949	*WNT3*	KEGG_WNT_SIGNALING_PATHWAY
17:44912901	*WNT3*	KEGG_WNT_SIGNALING_PATHWAY
17:44931399	*WNT3*	KEGG_WNT_SIGNALING_PATHWAY
17:44947756	*WNT3*	KEGG_WNT_SIGNALING_PATHWAY
3:49162284	*IP6K2*	NABA_BASEMENT_MEMBRANES
3:49162583	*IP6K2*	NABA_BASEMENT_MEMBRANES
6:112494762	*FYN*	NABA_BASEMENT_MEMBRANES
6:112531401	*FYN*	NABA_BASEMENT_MEMBRANES
6:112561154	*FYN*	NABA_BASEMENT_MEMBRANES
6:112561430	*FYN*	NABA_BASEMENT_MEMBRANES
6:33153528	*HLA-DRB5*	NABA_COLLAGENS
3:49162284	*IP6K2*	NABA_CORE_MATRISOME
3:49162583	*IP6K2*	NABA_CORE_MATRISOME
4:90804532	*SNCA_2*	NABA_CORE_MATRISOME
4:90868355	*SNCA_2*	NABA_CORE_MATRISOME
6:32031904	*HLA-DRB5*	NABA_CORE_MATRISOME
6:32062687	*HLA-DRB5*	NABA_CORE_MATRISOME
6:33153528	*HLA-DRB5*	NABA_CORE_MATRISOME
6:112494762	*FYN*	NABA_CORE_MATRISOME
6:112531401	*FYN*	NABA_CORE_MATRISOME
6:112561154	*FYN*	NABA_CORE_MATRISOME
6:112561430	*FYN*	NABA_CORE_MATRISOME
8:22007293	*BIN3*	NABA_CORE_MATRISOME
14:75057014	*RPS6KL1*	NABA_CORE_MATRISOME
3:122645304	*KPNA1*	NABA_ECM_AFFILIATED
3:122720608	*KPNA1*	NABA_ECM_AFFILIATED
4:15400087	*BST1*	NABA_ECM_AFFILIATED
6:31002527	*TRIM40*	NABA_ECM_AFFILIATED
12:40798608	*LRRK2_2*	NABA_ECM_AFFILIATED
12:40800267	*LRRK2_2*	NABA_ECM_AFFILIATED
12:40837264	*LRRK2_2*	NABA_ECM_AFFILIATED
12:40837331	*LRRK2_2*	NABA_ECM_AFFILIATED
12:40872410	*LRRK2_2*	NABA_ECM_AFFILIATED
12:40918443	*LRRK2_2*	NABA_ECM_AFFILIATED
3:49162284	*IP6K2*	NABA_ECM_GLYCOPROTEINS
3:49162583	*IP6K2*	NABA_ECM_GLYCOPROTEINS
4:90804532	*SNCA_2*	NABA_ECM_GLYCOPROTEINS
4:90868355	*SNCA_2*	NABA_ECM_GLYCOPROTEINS
6:32031904	*HLA-DRB5*	NABA_ECM_GLYCOPROTEINS
6:32062687	*HLA-DRB5*	NABA_ECM_GLYCOPROTEINS
6:112494762	*FYN*	NABA_ECM_GLYCOPROTEINS
6:112531401	*FYN*	NABA_ECM_GLYCOPROTEINS
6:112561154	*FYN*	NABA_ECM_GLYCOPROTEINS
6:112561430	*FYN*	NABA_ECM_GLYCOPROTEINS
8:22007293	*BIN3*	NABA_ECM_GLYCOPROTEINS
14:75057014	*RPS6KL1*	NABA_ECM_GLYCOPROTEINS
1:155027082	*GBAP1*	NABA_ECM_REGULATORS
1:155033317	*GBAP1*	NABA_ECM_REGULATORS
8:11712443	*CTSB*	NABA_ECM_REGULATORS
8:23190941	*BIN3*	NABA_ECM_REGULATORS
8:23212542	*BIN3*	NABA_ECM_REGULATORS
8:23216225	*BIN3*	NABA_ECM_REGULATORS

8:23223713	*BIN3*	NABA_ECM_REGULATORS
9:18502815	*SH3GL2_2*	NABA_ECM_REGULATORS
9:18522254	*SH3GL2_2*	NABA_ECM_REGULATORS
9:18583014	*SH3GL2_2*	NABA_ECM_REGULATORS
9:18623302	*SH3GL2_2*	NABA_ECM_REGULATORS
9:18633417	*SH3GL2_2*	NABA_ECM_REGULATORS
9:18663540	*SH3GL2_2*	NABA_ECM_REGULATORS
9:18721847	*SH3GL2_2*	NABA_ECM_REGULATORS
9:18725820	*SH3GL2_2*	NABA_ECM_REGULATORS
9:33791164	*UBAP2*	NABA_ECM_REGULATORS
17:42855931	*CRHR1_2*	NABA_ECM_REGULATORS
17:76869878	*DNAH17*	NABA_ECM_REGULATORS
1:155027082	*GBAP1*	NABA_MATRISOME_ASSOCIATED
1:155033317	*GBAP1*	NABA_MATRISOME_ASSOCIATED
1:226074321	*ITPKB*	NABA_MATRISOME_ASSOCIATED
3:122645304	*KPNA1*	NABA_MATRISOME_ASSOCIATED
3:122720608	*KPNA1*	NABA_MATRISOME_ASSOCIATED
4:15400087	*BST1*	NABA_MATRISOME_ASSOCIATED
4:76924571	*FAM47E-STBD1*	NABA_MATRISOME_ASSOCIATED
6:31002527	*TRIM40*	NABA_MATRISOME_ASSOCIATED
8:11712443	*CTSB*	NABA_MATRISOME_ASSOCIATED
8:23190941	*BIN3*	NABA_MATRISOME_ASSOCIATED
8:23212542	*BIN3*	NABA_MATRISOME_ASSOCIATED
8:23216225	*BIN3*	NABA_MATRISOME_ASSOCIATED
8:23223713	*BIN3*	NABA_MATRISOME_ASSOCIATED
9:18502815	*SH3GL2_2*	NABA_MATRISOME_ASSOCIATED
9:18522254	*SH3GL2_2*	NABA_MATRISOME_ASSOCIATED
9:18583014	*SH3GL2_2*	NABA_MATRISOME_ASSOCIATED
9:18623302	*SH3GL2_2*	NABA_MATRISOME_ASSOCIATED
9:18633417	*SH3GL2_2*	NABA_MATRISOME_ASSOCIATED
9:18663540	*SH3GL2_2*	NABA_MATRISOME_ASSOCIATED
9:18721847	*SH3GL2_2*	NABA_MATRISOME_ASSOCIATED
9:18725820	*SH3GL2_2*	NABA_MATRISOME_ASSOCIATED
9:33791164	*UBAP2*	NABA_MATRISOME_ASSOCIATED
12:40798608	*LRRK2_2*	NABA_MATRISOME_ASSOCIATED
12:40800267	*LRRK2_2*	NABA_MATRISOME_ASSOCIATED
12:40837264	*LRRK2_2*	NABA_MATRISOME_ASSOCIATED
12:40837331	*LRRK2_2*	NABA_MATRISOME_ASSOCIATED
12:40872410	*LRRK2_2*	NABA_MATRISOME_ASSOCIATED
12:40918443	*LRRK2_2*	NABA_MATRISOME_ASSOCIATED
17:42855931	*CRHR1_2*	NABA_MATRISOME_ASSOCIATED
17:44843651	*WNT3*	NABA_MATRISOME_ASSOCIATED
17:44870978	*WNT3*	NABA_MATRISOME_ASSOCIATED
17:44871422	*WNT3*	NABA_MATRISOME_ASSOCIATED
17:44888209	*WNT3*	NABA_MATRISOME_ASSOCIATED
17:44888715	*WNT3*	NABA_MATRISOME_ASSOCIATED
17:44906949	*WNT3*	NABA_MATRISOME_ASSOCIATED
17:44912901	*WNT3*	NABA_MATRISOME_ASSOCIATED
17:44931399	*WNT3*	NABA_MATRISOME_ASSOCIATED
17:44947756	*WNT3*	NABA_MATRISOME_ASSOCIATED
17:76869878	*DNAH17*	NABA_MATRISOME_ASSOCIATED
19:2251817	*SPPL2B*	NABA_MATRISOME_ASSOCIATED
1:155027082	*GBAP1*	NABA_MATRISOME

1:155033317	*GBAP1*	NABA_MATRISOME
1:226074321	*ITPKB*	NABA_MATRISOME
3:49162284	*IP6K2*	NABA_MATRISOME
3:49162583	*IP6K2*	NABA_MATRISOME
3:122645304	*KPNA1*	NABA_MATRISOME
3:122720608	*KPNA1*	NABA_MATRISOME
4:15400087	*BST1*	NABA_MATRISOME
4:76924571	*FAM47E-STBD1*	NABA_MATRISOME
4:90804532	*SNCA_2*	NABA_MATRISOME
4:90868355	*SNCA_2*	NABA_MATRISOME
6:31002527	*TRIM40*	NABA_MATRISOME
6:32031904	*HLA-DRB5*	NABA_MATRISOME
6:32062687	*HLA-DRB5*	NABA_MATRISOME
6:33153528	*HLA-DRB5*	NABA_MATRISOME
6:112494762	*FYN*	NABA_MATRISOME
6:112531401	*FYN*	NABA_MATRISOME
6:112561154	*FYN*	NABA_MATRISOME
6:112561430	*FYN*	NABA_MATRISOME
8:11712443	*CTSB*	NABA_MATRISOME
8:22007293	*BIN3*	NABA_MATRISOME
8:23190941	*BIN3*	NABA_MATRISOME
8:23212542	*BIN3*	NABA_MATRISOME
8:23216225	*BIN3*	NABA_MATRISOME
8:23223713	*BIN3*	NABA_MATRISOME
9:18502815	*SH3GL2_2*	NABA_MATRISOME
9:18522254	*SH3GL2_2*	NABA_MATRISOME
9:18583014	*SH3GL2_2*	NABA_MATRISOME
9:18623302	*SH3GL2_2*	NABA_MATRISOME
9:18633417	*SH3GL2_2*	NABA_MATRISOME
9:18663540	*SH3GL2_2*	NABA_MATRISOME
9:18721847	*SH3GL2_2*	NABA_MATRISOME
9:18725820	*SH3GL2_2*	NABA_MATRISOME
9:33791164	*UBAP2*	NABA_MATRISOME
12:40798608	*LRRK2_2*	NABA_MATRISOME
12:40800267	*LRRK2_2*	NABA_MATRISOME
12:40837264	*LRRK2_2*	NABA_MATRISOME
12:40837331	*LRRK2_2*	NABA_MATRISOME
12:40872410	*LRRK2_2*	NABA_MATRISOME
12:40918443	*LRRK2_2*	NABA_MATRISOME
14:75057014	*RPS6KL1*	NABA_MATRISOME
17:42855931	*CRHR1_2*	NABA_MATRISOME
17:44843651	*WNT3*	NABA_MATRISOME
17:44870978	*WNT3*	NABA_MATRISOME
17:44871422	*WNT3*	NABA_MATRISOME
17:44888209	*WNT3*	NABA_MATRISOME
17:44888715	*WNT3*	NABA_MATRISOME
17:44906949	*WNT3*	NABA_MATRISOME
17:44912901	*WNT3*	NABA_MATRISOME
17:44931399	*WNT3*	NABA_MATRISOME
17:44947756	*WNT3*	NABA_MATRISOME
17:76869878	*DNAH17*	NABA_MATRISOME
19:2251817	*SPPL2B*	NABA_MATRISOME
1:226074321	*ITPKB*	NABA_SECRETED_FACTORS

4:76924571	*FAM47E-STBD1*	NABA_SECRETED_FACTORS
17:7455536	*V1NB1*	NABA_SECRETED_FACTORS
17:44843651	*WNT3*	NABA_SECRETED_FACTORS
17:44870978	*WNT3*	NABA_SECRETED_FACTORS
17:44871422	*WNT3*	NABA_SECRETED_FACTORS
17:44888209	*WNT3*	NABA_SECRETED_FACTORS
17:44888715	*WNT3*	NABA_SECRETED_FACTORS
17:44906949	*WNT3*	NABA_SECRETED_FACTORS
17:44912901	*WNT3*	NABA_SECRETED_FACTORS
17:44931399	*WNT3*	NABA_SECRETED_FACTORS
17:44947756	*WNT3*	NABA_SECRETED_FACTORS
19:2251817	*SPPL2B*	NABA_SECRETED_FACTORS
3:49162284	*IP6K2*	PID_A6B1_A6B4_INTEGRIN_PATHWAY
3:49162583	*IP6K2*	PID_A6B1_A6B4_INTEGRIN_PATHWAY
6:112494762	*FYN*	PID_A6B1_A6B4_INTEGRIN_PATHWAY
6:112531401	*FYN*	PID_A6B1_A6B4_INTEGRIN_PATHWAY
6:112561154	*FYN*	PID_A6B1_A6B4_INTEGRIN_PATHWAY
6:112561430	*FYN*	PID_A6B1_A6B4_INTEGRIN_PATHWAY
6:112137847	*FYN*	PID_AJDISS_2PATHWAY
6:112151452	*FYN*	PID_AJDISS_2PATHWAY
6:112175069	*FYN*	PID_AJDISS_2PATHWAY
17:39883520	*RETREG3*	PID_AJDISS_2PATHWAY
19:2251817	*SPPL2B*	PID_ALK2_PATHWAY
4:90677243	*SNCA_2*	PID_ALPHA_SYNUCLEIN_PATHWAY
4:90700329	*SNCA_2*	PID_ALPHA_SYNUCLEIN_PATHWAY
4:90703993	*SNCA_2*	PID_ALPHA_SYNUCLEIN_PATHWAY
4:90738839	*SNCA_2*	PID_ALPHA_SYNUCLEIN_PATHWAY
4:90757294	*SNCA_2*	PID_ALPHA_SYNUCLEIN_PATHWAY
6:112137847	*FYN*	PID_ALPHA_SYNUCLEIN_PATHWAY
6:112151452	*FYN*	PID_ALPHA_SYNUCLEIN_PATHWAY
6:112175069	*FYN*	PID_ALPHA_SYNUCLEIN_PATHWAY
8:11353586	*CTSB*	PID_ALPHA_SYNUCLEIN_PATHWAY
8:11363667	*CTSB*	PID_ALPHA_SYNUCLEIN_PATHWAY
8:11381148	*CTSB*	PID_ALPHA_SYNUCLEIN_PATHWAY
10:121054578	*INPP5F*	PID_ALPHA_SYNUCLEIN_PATHWAY
10:121077191	*INPP5F*	PID_ALPHA_SYNUCLEIN_PATHWAY
10:121085648	*INPP5F*	PID_ALPHA_SYNUCLEIN_PATHWAY
10:121104943	*INPP5F*	PID_ALPHA_SYNUCLEIN_PATHWAY
10:121114292	*INPP5F*	PID_ALPHA_SYNUCLEIN_PATHWAY
10:121191186	*INPP5F*	PID_ALPHA_SYNUCLEIN_PATHWAY
10:121201427	*INPP5F*	PID_ALPHA_SYNUCLEIN_PATHWAY
3:49406095	*IP6K2*	PID_AMB2_NEUTROPHILS_PATHWAY
3:49424231	*IP6K2*	PID_AMB2_NEUTROPHILS_PATHWAY
3:49450137	*IP6K2*	PID_AMB2_NEUTROPHILS_PATHWAY
6:32151443	*HLA-DRB5*	PID_AMB2_NEUTROPHILS_PATHWAY
6:112137847	*FYN*	PID_AMB2_NEUTROPHILS_PATHWAY
6:112151452	*FYN*	PID_AMB2_NEUTROPHILS_PATHWAY
6:112175069	*FYN*	PID_AMB2_NEUTROPHILS_PATHWAY
8:11353586	*CTSB*	PID_AMB2_NEUTROPHILS_PATHWAY
8:11363667	*CTSB*	PID_AMB2_NEUTROPHILS_PATHWAY
8:11381148	*CTSB*	PID_AMB2_NEUTROPHILS_PATHWAY
11:133947732	*IGSF9B*	PID_AMB2_NEUTROPHILS_PATHWAY
6:112137847	*FYN*	PID_ANGIOPOIETIN_RECEPTOR_PATHWAY

6:112151452	*FYN*	PID_ANGIOPOIETIN_RECEPTOR_PATHWAY
6:112175069	*FYN*	PID_ANGIOPOIETIN_RECEPTOR_PATHWAY
17:40392358	*RETREG3*	PID_ANGIOPOIETIN_RECEPTOR_PATHWAY
17:7578671	*V1NB1*	PID_AP1_PATHWAY
4:76695927	*FAM47E-STBD1*	PID_ARF_3PATHWAY
8:131145058	*FAM49B*	PID_ARF_3PATHWAY
8:131153067	*FAM49B*	PID_ARF_3PATHWAY
8:131245821	*FAM49B*	PID_ARF_3PATHWAY
8:131328260	*FAM49B*	PID_ARF_3PATHWAY
8:131404984	*FAM49B*	PID_ARF_3PATHWAY
8:131408202	*FAM49B*	PID_ARF_3PATHWAY
8:131455469	*FAM49B*	PID_ARF_3PATHWAY
10:104005410	*GBF1*	PID_ARF_3PATHWAY
10:104034550	*GBF1*	PID_ARF_3PATHWAY
17:45020426	*WNT3*	PID_ARF_3PATHWAY
17:45059032	*WNT3*	PID_ARF_3PATHWAY
17:45089680	*WNT3*	PID_ARF_3PATHWAY
3:49406095	*IP6K2*	PID_ARF6_DOWNSTREAM_PATHWAY
3:49424231	*IP6K2*	PID_ARF6_DOWNSTREAM_PATHWAY
3:49450137	*IP6K2*	PID_ARF6_DOWNSTREAM_PATHWAY
17:42454243	*FAM171A2*	PID_ARF6_PATHWAY
19:3142784	*SPPL2B*	PID_ARF6_PATHWAY
19:3147326	*SPPL2B*	PID_ARF6_PATHWAY
10:15562608	*ITGA8*	PID_ARF6_TRAFFICKING_PATHWAY
10:15569598	*ITGA8*	PID_ARF6_TRAFFICKING_PATHWAY
10:15652368	*ITGA8*	PID_ARF6_TRAFFICKING_PATHWAY
4:76407998	*FAM47E-STBD1*	PID_AR_TF_PATHWAY
6:31862876	*HLA-DRB5*	PID_AR_TF_PATHWAY
5:134722833	*C5orf24*	PID_ATF2_PATHWAY
3:48199695	*IP6K2*	PID_ATM_PATHWAY
3:48214438	*IP6K2*	PID_ATM_PATHWAY
3:48199695	*IP6K2*	PID_ATR_PATHWAY
3:48214438	*IP6K2*	PID_ATR_PATHWAY
5:133558885	*C5orf24*	PID_ATR_PATHWAY
17:7578671	*V1NB1*	PID_AURORA_A_PATHWAY
3:49406095	*IP6K2*	PID_AURORA_B_PATHWAY
3:49424231	*IP6K2*	PID_AURORA_B_PATHWAY
3:49450137	*IP6K2*	PID_AURORA_B_PATHWAY
3:49406095	*IP6K2*	PID_AVB3_INTEGRIN_PATHWAY
3:49424231	*IP6K2*	PID_AVB3_INTEGRIN_PATHWAY
3:49450137	*IP6K2*	PID_AVB3_INTEGRIN_PATHWAY
6:33153528	*HLA-DRB5*	PID_AVB3_INTEGRIN_PATHWAY
3:49406095	*IP6K2*	PID_AVB3_OPN_PATHWAY
3:49424231	*IP6K2*	PID_AVB3_OPN_PATHWAY
3:49450137	*IP6K2*	PID_AVB3_OPN_PATHWAY
17:43390763	*CRHR1_2*	PID_AVB3_OPN_PATHWAY
17:7578671	*V1NB1*	PID_BARD1_PATHWAY
17:7129974	*V1NB1*	PID_BETA_CATENIN_DEG_PATHWAY
16:50300511	*CASC16*	PID_BETA_CATENIN_NUC_PATHWAY
16:50317039	*CASC16*	PID_BETA_CATENIN_NUC_PATHWAY
19:3008151	*SPPL2B*	PID_BETA_CATENIN_NUC_PATHWAY
19:3024077	*SPPL2B*	PID_BETA_CATENIN_NUC_PATHWAY
1:156059276	*GBAP1*	PID_CASPASE_PATHWAY

1:226568665	*ITPKB*	PID_CASPASE_PATHWAY
3:18395010	*SATB1*	PID_CASPASE_PATHWAY
3:18472577	*SATB1*	PID_CASPASE_PATHWAY
17:43390763	*CRHR1_2*	PID_CD40_PATHWAY
6:112137847	*FYN*	PID_CD8_TCR_PATHWAY
6:112151452	*FYN*	PID_CD8_TCR_PATHWAY
6:112175069	*FYN*	PID_CD8_TCR_PATHWAY
17:43390763	*CRHR1_2*	PID_CD8_TCR_PATHWAY
1:227182033	*ITPKB*	PID_CDC42_PATHWAY
1:227270318	*ITPKB*	PID_CDC42_PATHWAY
2:102357447	*MAP4K4*	PID_CERAMIDE_PATHWAY
2:102379173	*MAP4K4*	PID_CERAMIDE_PATHWAY
2:102413116	*MAP4K4*	PID_CERAMIDE_PATHWAY
2:101439596	*MAP4K4*	PID_CIRCADIAN_PATHWAY
2:101464631	*MAP4K4*	PID_CIRCADIAN_PATHWAY
2:101547064	*MAP4K4*	PID_CIRCADIAN_PATHWAY
2:101550181	*MAP4K4*	PID_CIRCADIAN_PATHWAY
2:101578010	*MAP4K4*	PID_CIRCADIAN_PATHWAY
2:101603812	*MAP4K4*	PID_CIRCADIAN_PATHWAY
4:76924571	*FAM47E-STBD1*	PID_CXCR3_PATHWAY
16:30510571	*SETD1A*	PID_CXCR3_PATHWAY
16:30516492	*SETD1A*	PID_CXCR3_PATHWAY
16:30520856	*SETD1A*	PID_CXCR3_PATHWAY
3:49406095	*IP6K2*	PID_CXCR4_PATHWAY
3:49424231	*IP6K2*	PID_CXCR4_PATHWAY
3:49450137	*IP6K2*	PID_CXCR4_PATHWAY
6:112137847	*FYN*	PID_CXCR4_PATHWAY
6:112151452	*FYN*	PID_CXCR4_PATHWAY
6:112175069	*FYN*	PID_CXCR4_PATHWAY
8:11353586	*CTSB*	PID_CXCR4_PATHWAY
8:11363667	*CTSB*	PID_CXCR4_PATHWAY
8:11381148	*CTSB*	PID_CXCR4_PATHWAY
10:15562608	*ITGA8*	PID_CXCR4_PATHWAY
10:15569598	*ITGA8*	PID_CXCR4_PATHWAY
10:15652368	*ITGA8*	PID_CXCR4_PATHWAY
17:40392358	*RETREG3*	PID_CXCR4_PATHWAY
3:48199695	*IP6K2*	PID_E2F_PATHWAY
3:48214438	*IP6K2*	PID_E2F_PATHWAY
17:76177567	*DNAH17*	PID_E2F_PATHWAY
3:49406095	*IP6K2*	PID_ECADHERIN_KERATINOCYTE_PATHWAY
3:49424231	*IP6K2*	PID_ECADHERIN_KERATINOCYTE_PATHWAY
3:49450137	*IP6K2*	PID_ECADHERIN_KERATINOCYTE_PATHWAY
6:112137847	*FYN*	PID_ECADHERIN_KERATINOCYTE_PATHWAY
6:112151452	*FYN*	PID_ECADHERIN_KERATINOCYTE_PATHWAY
6:112175069	*FYN*	PID_ECADHERIN_KERATINOCYTE_PATHWAY
17:39883520	*RETREG3*	PID_ECADHERIN_KERATINOCYTE_PATHWAY
3:49406095	*IP6K2*	PID_ECADHERIN_NASCENT_AJ_PATHWAY
3:49424231	*IP6K2*	PID_ECADHERIN_NASCENT_AJ_PATHWAY
3:49450137	*IP6K2*	PID_ECADHERIN_NASCENT_AJ_PATHWAY
17:39883520	*RETREG3*	PID_ECADHERIN_NASCENT_AJ_PATHWAY
3:49406095	*IP6K2*	PID_ECADHERIN_STABILIZATION_PATHWAY
3:49424231	*IP6K2*	PID_ECADHERIN_STABILIZATION_PATHWAY
3:49450137	*IP6K2*	PID_ECADHERIN_STABILIZATION_PATHWAY

9:33447547	*UBAP2*	PID_ECADHERIN_STABILIZATION_PATHWAY
3:49406095	*IP6K2*	PID_ENDOTHELIN_PATHWAY
3:49424231	*IP6K2*	PID_ENDOTHELIN_PATHWAY
3:49450137	*IP6K2*	PID_ENDOTHELIN_PATHWAY
3:123089448	*KPNA1*	PID_ENDOTHELIN_PATHWAY
8:131799767	*FAM49B*	PID_ENDOTHELIN_PATHWAY
8:131845512	*FAM49B*	PID_ENDOTHELIN_PATHWAY
8:131865425	*FAM49B*	PID_ENDOTHELIN_PATHWAY
8:131901170	*FAM49B*	PID_ENDOTHELIN_PATHWAY
16:50300511	*CASC16*	PID_ENDOTHELIN_PATHWAY
16:50317039	*CASC16*	PID_ENDOTHELIN_PATHWAY
19:3142784	*SPPL2B*	PID_ENDOTHELIN_PATHWAY
19:3147326	*SPPL2B*	PID_ENDOTHELIN_PATHWAY
3:49406095	*IP6K2*	PID_EPHA2_FWD_PATHWAY
3:49424231	*IP6K2*	PID_EPHA2_FWD_PATHWAY
3:49450137	*IP6K2*	PID_EPHA2_FWD_PATHWAY
3:49406095	*IP6K2*	PID_EPHA_FWDPATHWAY
3:49424231	*IP6K2*	PID_EPHA_FWDPATHWAY
3:49450137	*IP6K2*	PID_EPHA_FWDPATHWAY
6:112137847	*FYN*	PID_EPHA_FWDPATHWAY
6:112151452	*FYN*	PID_EPHA_FWDPATHWAY
6:112175069	*FYN*	PID_EPHA_FWDPATHWAY
8:11353586	*CTSB*	PID_EPHA_FWDPATHWAY
8:11363667	*CTSB*	PID_EPHA_FWDPATHWAY
8:11381148	*CTSB*	PID_EPHA_FWDPATHWAY
2:102357447	*MAP4K4*	PID_EPHB_FWD_PATHWAY
2:102379173	*MAP4K4*	PID_EPHB_FWD_PATHWAY
2:102413116	*MAP4K4*	PID_EPHB_FWD_PATHWAY
6:112137847	*FYN*	PID_EPHRINB_REV_PATHWAY
6:112151452	*FYN*	PID_EPHRINB_REV_PATHWAY
6:112175069	*FYN*	PID_EPHRINB_REV_PATHWAY
8:11353586	*CTSB*	PID_EPHRINB_REV_PATHWAY
8:11363667	*CTSB*	PID_EPHRINB_REV_PATHWAY
8:11381148	*CTSB*	PID_EPHRINB_REV_PATHWAY
17:42454243	*FAM171A2*	PID_EPHRINB_REV_PATHWAY
17:40392358	*RETREG3*	PID_EPO_PATHWAY
5:133558885	*C5orf24*	PID_ERBB1_DOWNSTREAM_PATHWAY
9:17579690	*SH3GL2_2*	PID_ERBB1_INTERNALIZATION_PATHWAY
9:17587160	*SH3GL2_2*	PID_ERBB1_INTERNALIZATION_PATHWAY
9:17609302	*SH3GL2_2*	PID_ERBB1_INTERNALIZATION_PATHWAY
9:17626762	*SH3GL2_2*	PID_ERBB1_INTERNALIZATION_PATHWAY
9:17627444	*SH3GL2_2*	PID_ERBB1_INTERNALIZATION_PATHWAY
9:17642664	*SH3GL2_2*	PID_ERBB1_INTERNALIZATION_PATHWAY
9:17710876	*SH3GL2_2*	PID_ERBB1_INTERNALIZATION_PATHWAY
9:17732590	*SH3GL2_2*	PID_ERBB1_INTERNALIZATION_PATHWAY
9:17737514	*SH3GL2_2*	PID_ERBB1_INTERNALIZATION_PATHWAY
9:17758945	*SH3GL2_2*	PID_ERBB1_INTERNALIZATION_PATHWAY
6:112137847	*FYN*	PID_ERBB4_PATHWAY
6:112151452	*FYN*	PID_ERBB4_PATHWAY
6:112175069	*FYN*	PID_ERBB4_PATHWAY
17:7101771	*V1NB1*	PID_ERBB4_PATHWAY
17:40392358	*RETREG3*	PID_ERBB4_PATHWAY
3:49406095	*IP6K2*	PID_ER_NONGENOMIC_PATHWAY

3:49424231	*IP6K2*	PID_ER_NONGENOMIC_PATHWAY
3:49450137	*IP6K2*	PID_ER_NONGENOMIC_PATHWAY
19:3142784	*SPPL2B*	PID_ER_NONGENOMIC_PATHWAY
19:3147326	*SPPL2B*	PID_ER_NONGENOMIC_PATHWAY
3:49406095	*IP6K2*	PID_FAK_PATHWAY
3:49424231	*IP6K2*	PID_FAK_PATHWAY
3:49450137	*IP6K2*	PID_FAK_PATHWAY
6:112137847	*FYN*	PID_FAK_PATHWAY
6:112151452	*FYN*	PID_FAK_PATHWAY
6:112175069	*FYN*	PID_FAK_PATHWAY
8:131145058	*FAM49B*	PID_FAK_PATHWAY
8:131153067	*FAM49B*	PID_FAK_PATHWAY
8:131245821	*FAM49B*	PID_FAK_PATHWAY
8:131328260	*FAM49B*	PID_FAK_PATHWAY
8:131404984	*FAM49B*	PID_FAK_PATHWAY
8:131408202	*FAM49B*	PID_FAK_PATHWAY
8:131455469	*FAM49B*	PID_FAK_PATHWAY
17:59791813	*BRIP1*	PID_FANCONI_PATHWAY
17:59922925	*BRIP1*	PID_FANCONI_PATHWAY
6:112137847	*FYN*	PID_FCER1_PATHWAY
6:112151452	*FYN*	PID_FCER1_PATHWAY
6:112175069	*FYN*	PID_FCER1_PATHWAY
17:40392358	*RETREG3*	PID_FGF_PATHWAY
6:112494762	*FYN*	PID_FOXM1_PATHWAY
6:112531401	*FYN*	PID_FOXM1_PATHWAY
6:112561154	*FYN*	PID_FOXM1_PATHWAY
6:112561430	*FYN*	PID_FOXM1_PATHWAY
17:41056283	*RETREG3*	PID_FOXO_PATHWAY
6:112137847	*FYN*	PID_GLYPICAN_1PATHWAY
6:112151452	*FYN*	PID_GLYPICAN_1PATHWAY
6:112175069	*FYN*	PID_GLYPICAN_1PATHWAY
8:11353586	*CTSB*	PID_GLYPICAN_1PATHWAY
8:11363667	*CTSB*	PID_GLYPICAN_1PATHWAY
8:11381148	*CTSB*	PID_GLYPICAN_1PATHWAY
17:40392358	*RETREG3*	PID_GMCSF_PATHWAY
17:7578671	*V1NB1*	PID_HDAC_CLASSIII_PATHWAY
2:170012307	*STK39*	PID_HEDGEHOG_2PATHWAY
2:170028024	*STK39*	PID_HEDGEHOG_2PATHWAY
2:170066424	*STK39*	PID_HEDGEHOG_2PATHWAY
10:104278055	*GBF1*	PID_HEDGEHOG_GLI_PATHWAY
10:104305501	*GBF1*	PID_HEDGEHOG_GLI_PATHWAY
1:226568665	*ITPKB*	PID_HES_HEY_PATHWAY
4:114424939	*CAMK2D*	PID_HES_HEY_PATHWAY
4:114494052	*CAMK2D*	PID_HES_HEY_PATHWAY
4:114529492	*CAMK2D*	PID_HES_HEY_PATHWAY
4:114539964	*CAMK2D*	PID_HES_HEY_PATHWAY
4:114597918	*CAMK2D*	PID_HES_HEY_PATHWAY
8:11558567	*CTSB*	PID_HES_HEY_PATHWAY
8:11599269	*CTSB*	PID_HES_HEY_PATHWAY
19:1611856	*SPPL2B*	PID_HES_HEY_PATHWAY
17:7578671	*V1NB1*	PID_HIF1A_PATHWAY
15:61016235	*VPS13C*	PID_HIF1_TFPATHWAY
15:61065568	*VPS13C*	PID_HIF1_TFPATHWAY

15:61094298	*VPS13C*	PID_HIF1_TFPATHWAY
15:61140736	*VPS13C*	PID_HIF1_TFPATHWAY
15:61142062	*VPS13C*	PID_HIF1_TFPATHWAY
15:61162571	*VPS13C*	PID_HIF1_TFPATHWAY
15:61191617	*VPS13C*	PID_HIF1_TFPATHWAY
15:61195427	*VPS13C*	PID_HIF1_TFPATHWAY
15:61221682	*VPS13C*	PID_HIF1_TFPATHWAY
15:61298486	*VPS13C*	PID_HIF1_TFPATHWAY
15:61375480	*VPS13C*	PID_HIF1_TFPATHWAY
15:61434581	*VPS13C*	PID_HIF1_TFPATHWAY
15:61467078	*VPS13C*	PID_HIF1_TFPATHWAY
15:61470808	*VPS13C*	PID_HIF1_TFPATHWAY
15:61500263	*VPS13C*	PID_HIF1_TFPATHWAY
6:33287183	*HLA-DRB5*	PID_HIV_NEF_PATHWAY
17:43390763	*CRHR1_2*	PID_HIV_NEF_PATHWAY
14:38061742	*MIPOL1*	PID_HNF3A_PATHWAY
14:38061742	*MIPOL1*	PID_HNF3B_PATHWAY
17:41056283	*RETREG3*	PID_HNF3B_PATHWAY
4:114424939	*CAMK2D*	PID_IFNG_PATHWAY
4:114494052	*CAMK2D*	PID_IFNG_PATHWAY
4:114529492	*CAMK2D*	PID_IFNG_PATHWAY
4:114539964	*CAMK2D*	PID_IFNG_PATHWAY
4:114597918	*CAMK2D*	PID_IFNG_PATHWAY
2:102720323	*MAP4K4*	PID_IL12_2PATHWAY
2:102783052	*MAP4K4*	PID_IL12_2PATHWAY
2:103047986	*MAP4K4*	PID_IL12_2PATHWAY
2:103063855	*MAP4K4*	PID_IL12_2PATHWAY
2:103066346	*MAP4K4*	PID_IL12_2PATHWAY
19:2477820	*SPPL2B*	PID_IL12_2PATHWAY
2:103047986	*MAP4K4*	PID_IL12_STAT4_PATHWAY
2:103063855	*MAP4K4*	PID_IL12_STAT4_PATHWAY
2:103066346	*MAP4K4*	PID_IL12_STAT4_PATHWAY
2:102612026	*MAP4K4*	PID_IL1_PATHWAY
2:102621855	*MAP4K4*	PID_IL1_PATHWAY
2:102631503	*MAP4K4*	PID_IL1_PATHWAY
2:102633387	*MAP4K4*	PID_IL1_PATHWAY
2:102720323	*MAP4K4*	PID_IL1_PATHWAY
2:102783052	*MAP4K4*	PID_IL1_PATHWAY
3:49406095	*IP6K2*	PID_IL2_1PATHWAY
3:49424231	*IP6K2*	PID_IL2_1PATHWAY
3:49450137	*IP6K2*	PID_IL2_1PATHWAY
6:112137847	*FYN*	PID_IL2_1PATHWAY
6:112151452	*FYN*	PID_IL2_1PATHWAY
6:112175069	*FYN*	PID_IL2_1PATHWAY
17:40392358	*RETREG3*	PID_IL2_1PATHWAY
2:103047986	*MAP4K4*	PID_IL23_PATHWAY
2:103063855	*MAP4K4*	PID_IL23_PATHWAY
2:103066346	*MAP4K4*	PID_IL23_PATHWAY
4:76924571	*FAM47E-STBD1*	PID_IL23_PATHWAY
17:7983799	*V1NB1*	PID_IL23_PATHWAY
17:40392358	*RETREG3*	PID_IL2_STAT5_PATHWAY
17:40392358	*RETREG3*	PID_IL3_PATHWAY
17:40392358	*RETREG3*	PID_IL4_2PATHWAY

17:40392358	*RETREG3*	PID_IL5_PATHWAY
19:3142784	*SPPL2B*	PID_IL8_CXCR1_PATHWAY
19:3147326	*SPPL2B*	PID_IL8_CXCR1_PATHWAY
5:133558885	*C5orf24*	PID_IL8_CXCR2_PATHWAY
19:3142784	*SPPL2B*	PID_IL8_CXCR2_PATHWAY
19:3147326	*SPPL2B*	PID_IL8_CXCR2_PATHWAY
1:226568665	*ITPKB*	PID_ILK_PATHWAY
3:49162284	*IP6K2*	PID_INTEGRIN1_PATHWAY
3:49162583	*IP6K2*	PID_INTEGRIN1_PATHWAY
6:33153528	*HLA-DRB5*	PID_INTEGRIN1_PATHWAY
6:112494762	*FYN*	PID_INTEGRIN1_PATHWAY
6:112531401	*FYN*	PID_INTEGRIN1_PATHWAY
6:112561154	*FYN*	PID_INTEGRIN1_PATHWAY
6:112561430	*FYN*	PID_INTEGRIN1_PATHWAY
10:15562608	*ITGA8*	PID_INTEGRIN1_PATHWAY
10:15569598	*ITGA8*	PID_INTEGRIN1_PATHWAY
10:15652368	*ITGA8*	PID_INTEGRIN1_PATHWAY
11:133947732	*IGSF9B*	PID_INTEGRIN2_PATHWAY
16:30510571	*SETD1A*	PID_INTEGRIN2_PATHWAY
16:30516492	*SETD1A*	PID_INTEGRIN2_PATHWAY
16:30520856	*SETD1A*	PID_INTEGRIN2_PATHWAY
16:31370452	*SETD1A*	PID_INTEGRIN2_PATHWAY
6:112494762	*FYN*	PID_INTEGRIN3_PATHWAY
6:112531401	*FYN*	PID_INTEGRIN3_PATHWAY
6:112561154	*FYN*	PID_INTEGRIN3_PATHWAY
6:112561430	*FYN*	PID_INTEGRIN3_PATHWAY
17:42454243	*FAM171A2*	PID_INTEGRIN3_PATHWAY
3:49162284	*IP6K2*	PID_INTEGRIN4_PATHWAY
3:49162583	*IP6K2*	PID_INTEGRIN4_PATHWAY
1:155027082	*GBAP1*	PID_INTEGRIN_A9B1_PATHWAY
1:155033317	*GBAP1*	PID_INTEGRIN_A9B1_PATHWAY
10:15562608	*ITGA8*	PID_INTEGRIN_CS_PATHWAY
10:15569598	*ITGA8*	PID_INTEGRIN_CS_PATHWAY
10:15652368	*ITGA8*	PID_INTEGRIN_CS_PATHWAY
16:30510571	*SETD1A*	PID_INTEGRIN_CS_PATHWAY
16:30516492	*SETD1A*	PID_INTEGRIN_CS_PATHWAY
16:30520856	*SETD1A*	PID_INTEGRIN_CS_PATHWAY
16:31370452	*SETD1A*	PID_INTEGRIN_CS_PATHWAY
17:42454243	*FAM171A2*	PID_INTEGRIN_CS_PATHWAY
3:49406095	*IP6K2*	PID_LIS1_PATHWAY
3:49424231	*IP6K2*	PID_LIS1_PATHWAY
3:49450137	*IP6K2*	PID_LIS1_PATHWAY
12:122816734	*HIP1R*	PID_LIS1_PATHWAY
12:122817580	*HIP1R*	PID_LIS1_PATHWAY
12:122853197	*HIP1R*	PID_LIS1_PATHWAY
1:227067656	*ITPKB*	PID_LKB1_PATHWAY
17:7578671	*V1NB1*	PID_LKB1_PATHWAY
17:43999203	*WNT3*	PID_LKB1_PATHWAY
17:44005297	*WNT3*	PID_LKB1_PATHWAY
17:44019712	*WNT3*	PID_LKB1_PATHWAY
17:44060492	*WNT3*	PID_LKB1_PATHWAY
17:44065263	*WNT3*	PID_LKB1_PATHWAY
17:44081178	*WNT3*	PID_LKB1_PATHWAY

17:44083297	*WNT3*	PID_LKB1_PATHWAY
3:123089448	*KPNA1*	PID_LPA4_PATHWAY
8:131799767	*FAM49B*	PID_LPA4_PATHWAY
8:131845512	*FAM49B*	PID_LPA4_PATHWAY
8:131865425	*FAM49B*	PID_LPA4_PATHWAY
8:131901170	*FAM49B*	PID_LPA4_PATHWAY
16:50300511	*CASC16*	PID_LPA4_PATHWAY
16:50317039	*CASC16*	PID_LPA4_PATHWAY
3:49406095	*IP6K2*	PID_LYSOPHOSPHOLIPID_PATHWAY
3:49424231	*IP6K2*	PID_LYSOPHOSPHOLIPID_PATHWAY
3:49450137	*IP6K2*	PID_LYSOPHOSPHOLIPID_PATHWAY
3:123089448	*KPNA1*	PID_LYSOPHOSPHOLIPID_PATHWAY
8:131799767	*FAM49B*	PID_LYSOPHOSPHOLIPID_PATHWAY
8:131845512	*FAM49B*	PID_LYSOPHOSPHOLIPID_PATHWAY
8:131865425	*FAM49B*	PID_LYSOPHOSPHOLIPID_PATHWAY
8:131901170	*FAM49B*	PID_LYSOPHOSPHOLIPID_PATHWAY
16:50300511	*CASC16*	PID_LYSOPHOSPHOLIPID_PATHWAY
16:50317039	*CASC16*	PID_LYSOPHOSPHOLIPID_PATHWAY
17:43999203	*WNT3*	PID_LYSOPHOSPHOLIPID_PATHWAY
17:44005297	*WNT3*	PID_LYSOPHOSPHOLIPID_PATHWAY
17:44019712	*WNT3*	PID_LYSOPHOSPHOLIPID_PATHWAY
17:44060492	*WNT3*	PID_LYSOPHOSPHOLIPID_PATHWAY
17:44065263	*WNT3*	PID_LYSOPHOSPHOLIPID_PATHWAY
17:44081178	*WNT3*	PID_LYSOPHOSPHOLIPID_PATHWAY
17:44083297	*WNT3*	PID_LYSOPHOSPHOLIPID_PATHWAY
19:3142784	*SPPL2B*	PID_LYSOPHOSPHOLIPID_PATHWAY
19:3147326	*SPPL2B*	PID_LYSOPHOSPHOLIPID_PATHWAY
18:40409806	*RIT2*	PID_MAPK_TRK_PATHWAY
18:40494893	*RIT2*	PID_MAPK_TRK_PATHWAY
18:40543738	*RIT2*	PID_MAPK_TRK_PATHWAY
18:40612823	*RIT2*	PID_MAPK_TRK_PATHWAY
18:40649778	*RIT2*	PID_MAPK_TRK_PATHWAY
18:40671720	*RIT2*	PID_MAPK_TRK_PATHWAY
18:40673380	*RIT2*	PID_MAPK_TRK_PATHWAY
3:49406095	*IP6K2*	PID_MET_PATHWAY
3:49424231	*IP6K2*	PID_MET_PATHWAY
3:49450137	*IP6K2*	PID_MET_PATHWAY
9:17579690	*SH3GL2_2*	PID_MET_PATHWAY
9:17587160	*SH3GL2_2*	PID_MET_PATHWAY
9:17609302	*SH3GL2_2*	PID_MET_PATHWAY
9:17626762	*SH3GL2_2*	PID_MET_PATHWAY
9:17627444	*SH3GL2_2*	PID_MET_PATHWAY
9:17642664	*SH3GL2_2*	PID_MET_PATHWAY
9:17710876	*SH3GL2_2*	PID_MET_PATHWAY
9:17732590	*SH3GL2_2*	PID_MET_PATHWAY
9:17737514	*SH3GL2_2*	PID_MET_PATHWAY
9:17758945	*SH3GL2_2*	PID_MET_PATHWAY
3:49406095	*IP6K2*	PID_MTOR_4PATHWAY
3:49424231	*IP6K2*	PID_MTOR_4PATHWAY
3:49450137	*IP6K2*	PID_MTOR_4PATHWAY
12:122816734	*HIP1R*	PID_MTOR_4PATHWAY
12:122817580	*HIP1R*	PID_MTOR_4PATHWAY
12:122853197	*HIP1R*	PID_MTOR_4PATHWAY

17:7479140	*V1NB1*	PID_MTOR_4PATHWAY
17:7480325	*V1NB1*	PID_MTOR_4PATHWAY
3:48199695	*IP6K2*	PID_MYC_ACTIV_PATHWAY
3:48214438	*IP6K2*	PID_MYC_ACTIV_PATHWAY
5:60273923	*ELOVL7*	PID_MYC_ACTIV_PATHWAY
5:60288288	*ELOVL7*	PID_MYC_ACTIV_PATHWAY
8:22108784	*BIN3*	PID_MYC_ACTIV_PATHWAY
17:7479140	*V1NB1*	PID_MYC_ACTIV_PATHWAY
17:7480325	*V1NB1*	PID_MYC_ACTIV_PATHWAY
17:7578671	*V1NB1*	PID_MYC_ACTIV_PATHWAY
17:76177567	*DNAH17*	PID_MYC_ACTIV_PATHWAY
5:133558885	*C5orf24*	PID_MYC_PATHWAY
3:49406095	*IP6K2*	PID_NCADHERIN_PATHWAY
3:49424231	*IP6K2*	PID_NCADHERIN_PATHWAY
3:49450137	*IP6K2*	PID_NCADHERIN_PATHWAY
17:39883520	*RETREG3*	PID_NCADHERIN_PATHWAY
6:112137847	*FYN*	PID_NEPHRIN_NEPH1_PATHWAY
6:112151452	*FYN*	PID_NEPHRIN_NEPH1_PATHWAY
6:112175069	*FYN*	PID_NEPHRIN_NEPH1_PATHWAY
3:49406095	*IP6K2*	PID_NETRIN_PATHWAY
3:49424231	*IP6K2*	PID_NETRIN_PATHWAY
3:49450137	*IP6K2*	PID_NETRIN_PATHWAY
6:112137847	*FYN*	PID_NETRIN_PATHWAY
6:112151452	*FYN*	PID_NETRIN_PATHWAY
6:112175069	*FYN*	PID_NETRIN_PATHWAY
16:50730088	*CASC16*	PID_NFKAPPAB_CANONICAL_PATHWAY
16:50730446	*CASC16*	PID_NFKAPPAB_CANONICAL_PATHWAY
16:50745926	*CASC16*	PID_NFKAPPAB_CANONICAL_PATHWAY
16:50790891	*CASC16*	PID_NFKAPPAB_CANONICAL_PATHWAY
6:32179319	*HLA-DRB5*	PID_NOTCH_PATHWAY
6:32186193	*HLA-DRB5*	PID_NOTCH_PATHWAY
12:41144387	*LRRK2_2*	PID_NOTCH_PATHWAY
12:41147295	*LRRK2_2*	PID_NOTCH_PATHWAY
12:41195135	*LRRK2_2*	PID_NOTCH_PATHWAY
12:41251554	*LRRK2_2*	PID_NOTCH_PATHWAY
12:41255129	*LRRK2_2*	PID_NOTCH_PATHWAY
12:41297969	*LRRK2_2*	PID_NOTCH_PATHWAY
12:41407403	*LRRK2_2*	PID_NOTCH_PATHWAY
1:161875645	*FCGR2A*	PID_P38_ALPHA_BETA_DOWNSTREAM_PATHWAY
1:161930954	*FCGR2A*	PID_P38_ALPHA_BETA_DOWNSTREAM_PATHWAY
17:7578671	*V1NB1*	PID_P38_ALPHA_BETA_DOWNSTREAM_PATHWAY
6:112137847	*FYN*	PID_P38_ALPHA_BETA_PATHWAY
6:112151452	*FYN*	PID_P38_ALPHA_BETA_PATHWAY
6:112175069	*FYN*	PID_P38_ALPHA_BETA_PATHWAY
8:11353586	*CTSB*	PID_P38_ALPHA_BETA_PATHWAY
8:11363667	*CTSB*	PID_P38_ALPHA_BETA_PATHWAY
8:11381148	*CTSB*	PID_P38_ALPHA_BETA_PATHWAY
17:43999203	*WNT3*	PID_P38_GAMMA_DELTA_PATHWAY
17:44005297	*WNT3*	PID_P38_GAMMA_DELTA_PATHWAY
17:44019712	*WNT3*	PID_P38_GAMMA_DELTA_PATHWAY
17:44060492	*WNT3*	PID_P38_GAMMA_DELTA_PATHWAY
17:44065263	*WNT3*	PID_P38_GAMMA_DELTA_PATHWAY
17:44081178	*WNT3*	PID_P38_GAMMA_DELTA_PATHWAY

17:44083297	*WNT3*	PID_P38_GAMMA_DELTA_PATHWAY
19:1611856	*SPPL2B*	PID_P38_MK2_PATHWAY
19:2477820	*SPPL2B*	PID_P38_MKK3_6PATHWAY
2:102357447	*MAP4K4*	PID_P53_DOWNSTREAM_PATHWAY
2:102379173	*MAP4K4*	PID_P53_DOWNSTREAM_PATHWAY
2:102413116	*MAP4K4*	PID_P53_DOWNSTREAM_PATHWAY
4:76407998	*FAM47E-STBD1*	PID_P53_DOWNSTREAM_PATHWAY
8:22974278	*BIN3*	PID_P53_DOWNSTREAM_PATHWAY
8:23050400	*BIN3*	PID_P53_DOWNSTREAM_PATHWAY
14:38061742	*MIPOL1*	PID_P53_DOWNSTREAM_PATHWAY
17:7578671	*V1NB1*	PID_P53_DOWNSTREAM_PATHWAY
4:76407998	*FAM47E-STBD1*	PID_P53_REGULATION_PATHWAY
5:133558885	*C5orf24*	PID_P53_REGULATION_PATHWAY
6:33287183	*HLA-DRB5*	PID_P53_REGULATION_PATHWAY
17:7578671	*V1NB1*	PID_P53_REGULATION_PATHWAY
1:161171736	*FCGR2A*	PID_P73PATHWAY
4:76407998	*FAM47E-STBD1*	PID_P73PATHWAY
3:49406095	*IP6K2*	PID_P75_NTR_PATHWAY
3:49424231	*IP6K2*	PID_P75_NTR_PATHWAY
3:49450137	*IP6K2*	PID_P75_NTR_PATHWAY
17:7578671	*V1NB1*	PID_P75_NTR_PATHWAY
3:49406095	*IP6K2*	PID_PDGFRB_PATHWAY
3:49424231	*IP6K2*	PID_PDGFRB_PATHWAY
3:49450137	*IP6K2*	PID_PDGFRB_PATHWAY
5:133558885	*C5orf24*	PID_PDGFRB_PATHWAY
6:112137847	*FYN*	PID_PDGFRB_PATHWAY
6:112151452	*FYN*	PID_PDGFRB_PATHWAY
6:112175069	*FYN*	PID_PDGFRB_PATHWAY
8:11353586	*CTSB*	PID_PDGFRB_PATHWAY
8:11363667	*CTSB*	PID_PDGFRB_PATHWAY
8:11381148	*CTSB*	PID_PDGFRB_PATHWAY
17:40392358	*RETREG3*	PID_PDGFRB_PATHWAY
3:122200170	*KPNA1*	PID_PI3KCI_AKT_PATHWAY
3:49406095	*IP6K2*	PID_PI3KCI_PATHWAY
3:49424231	*IP6K2*	PID_PI3KCI_PATHWAY
3:49450137	*IP6K2*	PID_PI3KCI_PATHWAY
6:112137847	*FYN*	PID_PI3KCI_PATHWAY
6:112151452	*FYN*	PID_PI3KCI_PATHWAY
6:112175069	*FYN*	PID_PI3KCI_PATHWAY
8:11353586	*CTSB*	PID_PI3KCI_PATHWAY
8:11363667	*CTSB*	PID_PI3KCI_PATHWAY
8:11381148	*CTSB*	PID_PI3KCI_PATHWAY
17:76727455	*DNAH17*	PID_PI3KCI_PATHWAY
3:49406095	*IP6K2*	PID_PLK1_PATHWAY
3:49424231	*IP6K2*	PID_PLK1_PATHWAY
3:49450137	*IP6K2*	PID_PLK1_PATHWAY
5:133558885	*C5orf24*	PID_PLK1_PATHWAY
3:49406095	*IP6K2*	PID_PRL_SIGNALING_EVENTS_PATHWAY
3:49424231	*IP6K2*	PID_PRL_SIGNALING_EVENTS_PATHWAY
3:49450137	*IP6K2*	PID_PRL_SIGNALING_EVENTS_PATHWAY
16:50612930	*CASC16*	PID_PS1_PATHWAY
16:50643086	*CASC16*	PID_PS1_PATHWAY
3:49406095	*IP6K2*	PID_PTP1B_PATHWAY

3:49424231	*IP6K2*	PID_PTP1B_PATHWAY
3:49450137	*IP6K2*	PID_PTP1B_PATHWAY
6:112137847	*FYN*	PID_PTP1B_PATHWAY
6:112151452	*FYN*	PID_PTP1B_PATHWAY
6:112175069	*FYN*	PID_PTP1B_PATHWAY
8:11353586	*CTSB*	PID_PTP1B_PATHWAY
8:11363667	*CTSB*	PID_PTP1B_PATHWAY
8:11381148	*CTSB*	PID_PTP1B_PATHWAY
17:40392358	*RETREG3*	PID_PTP1B_PATHWAY
17:42454243	*FAM171A2*	PID_PTP1B_PATHWAY
1:155975202	*GBAP1*	PID_RAC1_REG_PATHWAY
5:133558885	*C5orf24*	PID_RB_1PATHWAY
6:32941506	*HLA-DRB5*	PID_RB_1PATHWAY
1:155975202	*GBAP1*	PID_REELIN_PATHWAY
6:112137847	*FYN*	PID_REELIN_PATHWAY
6:112151452	*FYN*	PID_REELIN_PATHWAY
6:112175069	*FYN*	PID_REELIN_PATHWAY
17:43999203	*WNT3*	PID_REELIN_PATHWAY
17:44005297	*WNT3*	PID_REELIN_PATHWAY
17:44019712	*WNT3*	PID_REELIN_PATHWAY
17:44060492	*WNT3*	PID_REELIN_PATHWAY
17:44065263	*WNT3*	PID_REELIN_PATHWAY
17:44081178	*WNT3*	PID_REELIN_PATHWAY
17:44083297	*WNT3*	PID_REELIN_PATHWAY
17:7578671	*V1NB1*	PID_REG_GR_PATHWAY
17:40392358	*RETREG3*	PID_REG_GR_PATHWAY
3:49406095	*IP6K2*	PID_RET_PATHWAY
3:49424231	*IP6K2*	PID_RET_PATHWAY
3:49450137	*IP6K2*	PID_RET_PATHWAY
3:49406095	*IP6K2*	PID_RHOA_PATHWAY
3:49424231	*IP6K2*	PID_RHOA_PATHWAY
3:49450137	*IP6K2*	PID_RHOA_PATHWAY
9:17579690	*SH3GL2_2*	PID_RHOA_PATHWAY
9:17587160	*SH3GL2_2*	PID_RHOA_PATHWAY
9:17609302	*SH3GL2_2*	PID_RHOA_PATHWAY
9:17626762	*SH3GL2_2*	PID_RHOA_PATHWAY
9:17627444	*SH3GL2_2*	PID_RHOA_PATHWAY
9:17642664	*SH3GL2_2*	PID_RHOA_PATHWAY
9:17710876	*SH3GL2_2*	PID_RHOA_PATHWAY
9:17732590	*SH3GL2_2*	PID_RHOA_PATHWAY
9:17737514	*SH3GL2_2*	PID_RHOA_PATHWAY
9:17758945	*SH3GL2_2*	PID_RHOA_PATHWAY
1:155975202	*GBAP1*	PID_RHOA_REG_PATHWAY
3:49406095	*IP6K2*	PID_RHOA_REG_PATHWAY
3:49424231	*IP6K2*	PID_RHOA_REG_PATHWAY
3:49450137	*IP6K2*	PID_RHOA_REG_PATHWAY
19:3142784	*SPPL2B*	PID_S1P_META_PATHWAY
19:3147326	*SPPL2B*	PID_S1P_META_PATHWAY
3:49406095	*IP6K2*	PID_S1P_S1P1_PATHWAY
3:49424231	*IP6K2*	PID_S1P_S1P1_PATHWAY
3:49450137	*IP6K2*	PID_S1P_S1P1_PATHWAY
3:49406095	*IP6K2*	PID_S1P_S1P2_PATHWAY
3:49424231	*IP6K2*	PID_S1P_S1P2_PATHWAY

3:49450137	*IP6K2*	PID_S1P_S1P2_PATHWAY
19:3142784	*SPPL2B*	PID_S1P_S1P2_PATHWAY
19:3147326	*SPPL2B*	PID_S1P_S1P2_PATHWAY
3:49406095	*IP6K2*	PID_S1P_S1P3_PATHWAY
3:49424231	*IP6K2*	PID_S1P_S1P3_PATHWAY
3:49450137	*IP6K2*	PID_S1P_S1P3_PATHWAY
19:3142784	*SPPL2B*	PID_S1P_S1P3_PATHWAY
19:3147326	*SPPL2B*	PID_S1P_S1P3_PATHWAY
3:49406095	*IP6K2*	PID_S1P_S1P4_PATHWAY
3:49424231	*IP6K2*	PID_S1P_S1P4_PATHWAY
3:49450137	*IP6K2*	PID_S1P_S1P4_PATHWAY
3:49406095	*IP6K2*	PID_SHP2_PATHWAY
3:49424231	*IP6K2*	PID_SHP2_PATHWAY
3:49450137	*IP6K2*	PID_SHP2_PATHWAY
19:1611856	*SPPL2B*	PID_SMAD2_3NUCLEAR_PATHWAY
6:33153528	*HLA-DRB5*	PID_SYNDECAN_1_PATHWAY
3:49406095	*IP6K2*	PID_SYNDECAN_2_PATHWAY
3:49424231	*IP6K2*	PID_SYNDECAN_2_PATHWAY
3:49450137	*IP6K2*	PID_SYNDECAN_2_PATHWAY
6:112137847	*FYN*	PID_SYNDECAN_3_PATHWAY
6:112151452	*FYN*	PID_SYNDECAN_3_PATHWAY
6:112175069	*FYN*	PID_SYNDECAN_3_PATHWAY
3:49406095	*IP6K2*	PID_SYNDECAN_4_PATHWAY
3:49424231	*IP6K2*	PID_SYNDECAN_4_PATHWAY
3:49450137	*IP6K2*	PID_SYNDECAN_4_PATHWAY
17:40392358	*RETREG3*	PID_TCPTP_PATHWAY
2:168897564	*STK39*	PID_TCR_PATHWAY
2:168919016	*STK39*	PID_TCR_PATHWAY
2:168931636	*STK39*	PID_TCR_PATHWAY
2:168979290	*STK39*	PID_TCR_PATHWAY
2:169016304	*STK39*	PID_TCR_PATHWAY
2:169057162	*STK39*	PID_TCR_PATHWAY
6:112137847	*FYN*	PID_TCR_PATHWAY
6:112151452	*FYN*	PID_TCR_PATHWAY
6:112175069	*FYN*	PID_TCR_PATHWAY
17:43390763	*CRHR1_2*	PID_TCR_PATHWAY
3:49406095	*IP6K2*	PID_TGFBR_PATHWAY
3:49424231	*IP6K2*	PID_TGFBR_PATHWAY
3:49450137	*IP6K2*	PID_TGFBR_PATHWAY
3:150298317	*MED12L*	PID_TGFBR_PATHWAY
5:133558885	*C5orf24*	PID_TGFBR_PATHWAY
6:33287183	*HLA-DRB5*	PID_TGFBR_PATHWAY
3:49406095	*IP6K2*	PID_THROMBIN_PAR1_PATHWAY
3:49424231	*IP6K2*	PID_THROMBIN_PAR1_PATHWAY
3:49450137	*IP6K2*	PID_THROMBIN_PAR1_PATHWAY
19:3142784	*SPPL2B*	PID_THROMBIN_PAR1_PATHWAY
19:3147326	*SPPL2B*	PID_THROMBIN_PAR1_PATHWAY
3:49406095	*IP6K2*	PID_THROMBIN_PAR4_PATHWAY
3:49424231	*IP6K2*	PID_THROMBIN_PAR4_PATHWAY
3:49450137	*IP6K2*	PID_THROMBIN_PAR4_PATHWAY
19:3142784	*SPPL2B*	PID_THROMBIN_PAR4_PATHWAY
19:3147326	*SPPL2B*	PID_THROMBIN_PAR4_PATHWAY
2:102357447	*MAP4K4*	PID_TNF_PATHWAY

2:102379173	*MAP4K4*	PID_TNF_PATHWAY
2:102413116	*MAP4K4*	PID_TNF_PATHWAY
16:50790891	*CASC16*	PID_TNF_PATHWAY
3:49406095	*IP6K2*	PID_TOLL_ENDOGENOUS_PATHWAY
3:49424231	*IP6K2*	PID_TOLL_ENDOGENOUS_PATHWAY
3:49450137	*IP6K2*	PID_TOLL_ENDOGENOUS_PATHWAY
1:155698425	*GBAP1*	PID_TRAIL_PATHWAY
8:22974278	*BIN3*	PID_TRAIL_PATHWAY
8:23050400	*BIN3*	PID_TRAIL_PATHWAY
3:49406095	*IP6K2*	PID_TRKR_PATHWAY
3:49424231	*IP6K2*	PID_TRKR_PATHWAY
3:49450137	*IP6K2*	PID_TRKR_PATHWAY
18:40409806	*RIT2*	PID_TRKR_PATHWAY
18:40494893	*RIT2*	PID_TRKR_PATHWAY
18:40543738	*RIT2*	PID_TRKR_PATHWAY
18:40612823	*RIT2*	PID_TRKR_PATHWAY
18:40649778	*RIT2*	PID_TRKR_PATHWAY
18:40671720	*RIT2*	PID_TRKR_PATHWAY
18:40673380	*RIT2*	PID_TRKR_PATHWAY
3:49406095	*IP6K2*	PID_TXA2PATHWAY
3:49424231	*IP6K2*	PID_TXA2PATHWAY
3:49450137	*IP6K2*	PID_TXA2PATHWAY
6:112137847	*FYN*	PID_TXA2PATHWAY
6:112151452	*FYN*	PID_TXA2PATHWAY
6:112175069	*FYN*	PID_TXA2PATHWAY
8:11353586	*CTSB*	PID_TXA2PATHWAY
8:11363667	*CTSB*	PID_TXA2PATHWAY
8:11381148	*CTSB*	PID_TXA2PATHWAY
19:3142784	*SPPL2B*	PID_TXA2PATHWAY
19:3147326	*SPPL2B*	PID_TXA2PATHWAY
3:49406095	*IP6K2*	PID_VEGFR1_2_PATHWAY
3:49424231	*IP6K2*	PID_VEGFR1_2_PATHWAY
3:49450137	*IP6K2*	PID_VEGFR1_2_PATHWAY
6:112137847	*FYN*	PID_VEGFR1_2_PATHWAY
6:112151452	*FYN*	PID_VEGFR1_2_PATHWAY
6:112175069	*FYN*	PID_VEGFR1_2_PATHWAY
17:7129974	*V1NB1*	PID_WNT_CANONICAL_PATHWAY
3:49406095	*IP6K2*	PID_WNT_NONCANONICAL_PATHWAY
3:49424231	*IP6K2*	PID_WNT_NONCANONICAL_PATHWAY
3:49450137	*IP6K2*	PID_WNT_NONCANONICAL_PATHWAY
17:7129974	*V1NB1*	PID_WNT_NONCANONICAL_PATHWAY
17:44843651	*WNT3*	PID_WNT_SIGNALING_PATHWAY
17:44870978	*WNT3*	PID_WNT_SIGNALING_PATHWAY
17:44871422	*WNT3*	PID_WNT_SIGNALING_PATHWAY
17:44888209	*WNT3*	PID_WNT_SIGNALING_PATHWAY
17:44888715	*WNT3*	PID_WNT_SIGNALING_PATHWAY
17:44906949	*WNT3*	PID_WNT_SIGNALING_PATHWAY
10:104847713	*GBF1*	REACTOME_ABACAVIR_METABOLISM
10:104861736	*GBF1*	REACTOME_ABACAVIR_METABOLISM
10:104926485	*GBF1*	REACTOME_ABACAVIR_METABOLISM
10:104929716	*GBF1*	REACTOME_ABACAVIR_METABOLISM
10:104847713	*GBF1*	REACTOME_ABACAVIR_TRANSPORT_AND_METABOLISM
10:104861736	*GBF1*	REACTOME_ABACAVIR_TRANSPORT_AND_METABOLISM

10:104926485	*GBF1*	REACTOME_ABACAVIR_TRANSPORT_AND_METABOLISM
10:104929716	*GBF1*	REACTOME_ABACAVIR_TRANSPORT_AND_METABOLISM
6:32151443	*HLA-DRB5*	REACTOME_ABC_FAMILY_PROTEINS_MEDIATED_TRANSPORT
12:39960237	*LRRK2_2*	REACTOME_ABC_FAMILY_PROTEINS_MEDIATED_TRANSPORT
12:123453034	*HIP1R*	REACTOME_ABC_FAMILY_PROTEINS_MEDIATED_TRANSPORT
6:32151443	*HLA-DRB5*	REACTOME_ABC_TRANSPORTER_DISORDERS
14:74764925	*RPS6KL1*	REACTOME_ABC_TRANSPORTER_DISORDERS
12:39960237	*LRRK2_2*	REACTOME_ABC_TRANSPORTERS_IN_LIPID_HOMEOSTASIS
6:31925679	*HLA-DRB5*	REACTOME_ABORTIVE_ELONGATION_OF_HIV_1_TRANSCRIPT_IN_THE_ABSENCE_OF_TAT
6:72646790	*RIMS1*	REACTOME_ACETYLCHOLINE_NEUROTRANSMITTER_RELEASE_CYCLE
6:72648340	*RIMS1*	REACTOME_ACETYLCHOLINE_NEUROTRANSMITTER_RELEASE_CYCLE
6:72686444	*RIMS1*	REACTOME_ACETYLCHOLINE_NEUROTRANSMITTER_RELEASE_CYCLE
6:72716358	*RIMS1*	REACTOME_ACETYLCHOLINE_NEUROTRANSMITTER_RELEASE_CYCLE
6:72904599	*RIMS1*	REACTOME_ACETYLCHOLINE_NEUROTRANSMITTER_RELEASE_CYCLE
6:72913435	*RIMS1*	REACTOME_ACETYLCHOLINE_NEUROTRANSMITTER_RELEASE_CYCLE
6:72930929	*RIMS1*	REACTOME_ACETYLCHOLINE_NEUROTRANSMITTER_RELEASE_CYCLE
6:72982289	*RIMS1*	REACTOME_ACETYLCHOLINE_NEUROTRANSMITTER_RELEASE_CYCLE
6:72986711	*RIMS1*	REACTOME_ACETYLCHOLINE_NEUROTRANSMITTER_RELEASE_CYCLE
6:73008061	*RIMS1*	REACTOME_ACETYLCHOLINE_NEUROTRANSMITTER_RELEASE_CYCLE
6:73060492	*RIMS1*	REACTOME_ACETYLCHOLINE_NEUROTRANSMITTER_RELEASE_CYCLE
19:3142784	*SPPL2B*	REACTOME_ACETYLCHOLINE_REGULATES_INSULIN_SECRETION
19:3147326	*SPPL2B*	REACTOME_ACETYLCHOLINE_REGULATES_INSULIN_SECRETION
1:227067656	*ITPKB*	REACTOME_ACTIVATED_NOTCH1_TRANSMITS_SIGNAL_TO_THE_NUCLEUS
12:41144387	*LRRK2_2*	REACTOME_ACTIVATED_NOTCH1_TRANSMITS_SIGNAL_TO_THE_NUCLEUS
12:41147295	*LRRK2_2*	REACTOME_ACTIVATED_NOTCH1_TRANSMITS_SIGNAL_TO_THE_NUCLEUS
12:41195135	*LRRK2_2*	REACTOME_ACTIVATED_NOTCH1_TRANSMITS_SIGNAL_TO_THE_NUCLEUS
12:41251554	*LRRK2_2*	REACTOME_ACTIVATED_NOTCH1_TRANSMITS_SIGNAL_TO_THE_NUCLEUS
12:41255129	*LRRK2_2*	REACTOME_ACTIVATED_NOTCH1_TRANSMITS_SIGNAL_TO_THE_NUCLEUS
12:41297969	*LRRK2_2*	REACTOME_ACTIVATED_NOTCH1_TRANSMITS_SIGNAL_TO_THE_NUCLEUS
12:41407403	*LRRK2_2*	REACTOME_ACTIVATED_NOTCH1_TRANSMITS_SIGNAL_TO_THE_NUCLEUS
6:112137847	*FYN*	REACTOME_ACTIVATED_NTRK2_SIGNALS_THROUGH_FYN
6:112151452	*FYN*	REACTOME_ACTIVATED_NTRK2_SIGNALS_THROUGH_FYN
6:112175069	*FYN*	REACTOME_ACTIVATED_NTRK2_SIGNALS_THROUGH_FYN
16:50730088	*CASC16*	REACTOME_ACTIVATED_TAK1_MEDIATES_P38_MAPK_ACTIVATION
16:50730446	*CASC16*	REACTOME_ACTIVATED_TAK1_MEDIATES_P38_MAPK_ACTIVATION
16:50745926	*CASC16*	REACTOME_ACTIVATED_TAK1_MEDIATES_P38_MAPK_ACTIVATION
17:43999203	*WNT3*	REACTOME_ACTIVATION_OF_AMPK_DOWNSTREAM_OF_NMDARS
17:44005297	*WNT3*	REACTOME_ACTIVATION_OF_AMPK_DOWNSTREAM_OF_NMDARS
17:44019712	*WNT3*	REACTOME_ACTIVATION_OF_AMPK_DOWNSTREAM_OF_NMDARS
17:44060492	*WNT3*	REACTOME_ACTIVATION_OF_AMPK_DOWNSTREAM_OF_NMDARS
17:44065263	*WNT3*	REACTOME_ACTIVATION_OF_AMPK_DOWNSTREAM_OF_NMDARS
17:44081178	*WNT3*	REACTOME_ACTIVATION_OF_AMPK_DOWNSTREAM_OF_NMDARS
17:44083297	*WNT3*	REACTOME_ACTIVATION_OF_AMPK_DOWNSTREAM_OF_NMDARS
17:45215865	*WNT3*	REACTOME_ACTIVATION_OF_APC_C_AND_APC_C:CDC20_MEDIATED_DEGRADATION_OF_MITOTIC_PROTEINS
17:45244822	*WNT3*	REACTOME_ACTIVATION_OF_APC_C_AND_APC_C:CDC20_MEDIATED_DEGRADATION_OF_MITOTIC_PROTEINS
3:48199695	*IP6K2*	REACTOME_ACTIVATION_OF_ATR_IN_RESPONSE_TO_REPLICATION_STRESS
3:48214438	*IP6K2*	REACTOME_ACTIVATION_OF_ATR_IN_RESPONSE_TO_REPLICATION_STRESS
20:5934513	*CRLS1*	REACTOME_ACTIVATION_OF_ATR_IN_RESPONSE_TO_REPLICATION_STRESS
20:5935522	*CRLS1*	REACTOME_ACTIVATION_OF_ATR_IN_RESPONSE_TO_REPLICATION_STRESS
20:5968737	*CRLS1*	REACTOME_ACTIVATION_OF_ATR_IN_RESPONSE_TO_REPLICATION_STRESS
20:5968964	*CRLS1*	REACTOME_ACTIVATION_OF_ATR_IN_RESPONSE_TO_REPLICATION_STRESS
17:7578671	*V1NB1*	REACTOME_ACTIVATION_OF_BH3_ONLY_PROTEINS
6:31897219	*HLA-DRB5*	REACTOME_ACTIVATION_OF_C3_AND_C5

8:11679472	*CTSB*	REACTOME_ACTIVATION_OF_GENE_EXPRESSION_BY_SREBF_SREBP
17:7101771	*V1NB1*	REACTOME_ACTIVATION_OF_KAINATE_RECEPTORS_UPON_GLUTAMATE_BINDING
19:2557113	*SPPL2B*	REACTOME_ACTIVATION_OF_KAINATE_RECEPTORS_UPON_GLUTAMATE_BINDING
19:2563327	*SPPL2B*	REACTOME_ACTIVATION_OF_KAINATE_RECEPTORS_UPON_GLUTAMATE_BINDING
19:2580057	*SPPL2B*	REACTOME_ACTIVATION_OF_KAINATE_RECEPTORS_UPON_GLUTAMATE_BINDING
19:2660983	*SPPL2B*	REACTOME_ACTIVATION_OF_KAINATE_RECEPTORS_UPON_GLUTAMATE_BINDING
17:76869878	*DNAH17*	REACTOME_ACTIVATION_OF_MATRIX_METALLOPROTEINASES
10:103185542	*GBF1*	REACTOME_ACTIVATION_OF_NF_KAPPAB_IN_B_CELLS
10:103305087	*GBF1*	REACTOME_ACTIVATION_OF_NF_KAPPAB_IN_B_CELLS
10:103308244	*GBF1*	REACTOME_ACTIVATION_OF_NF_KAPPAB_IN_B_CELLS
3:48835245	*IP6K2*	REACTOME_ACTIVATION_OF_NMDA_RECEPTORS_AND_POSTSYNAPTIC_EVENTS
4:114424939	*CAMK2D*	REACTOME_ACTIVATION_OF_NMDA_RECEPTORS_AND_POSTSYNAPTIC_EVENTS
4:114494052	*CAMK2D*	REACTOME_ACTIVATION_OF_NMDA_RECEPTORS_AND_POSTSYNAPTIC_EVENTS
4:114529492	*CAMK2D*	REACTOME_ACTIVATION_OF_NMDA_RECEPTORS_AND_POSTSYNAPTIC_EVENTS
4:114539964	*CAMK2D*	REACTOME_ACTIVATION_OF_NMDA_RECEPTORS_AND_POSTSYNAPTIC_EVENTS
4:114597918	*CAMK2D*	REACTOME_ACTIVATION_OF_NMDA_RECEPTORS_AND_POSTSYNAPTIC_EVENTS
8:131799767	*FAM49B*	REACTOME_ACTIVATION_OF_NMDA_RECEPTORS_AND_POSTSYNAPTIC_EVENTS
8:131845512	*FAM49B*	REACTOME_ACTIVATION_OF_NMDA_RECEPTORS_AND_POSTSYNAPTIC_EVENTS
8:131865425	*FAM49B*	REACTOME_ACTIVATION_OF_NMDA_RECEPTORS_AND_POSTSYNAPTIC_EVENTS
8:131901170	*FAM49B*	REACTOME_ACTIVATION_OF_NMDA_RECEPTORS_AND_POSTSYNAPTIC_EVENTS
11:83219006	*DLG2*	REACTOME_ACTIVATION_OF_NMDA_RECEPTORS_AND_POSTSYNAPTIC_EVENTS
11:83232915	*DLG2*	REACTOME_ACTIVATION_OF_NMDA_RECEPTORS_AND_POSTSYNAPTIC_EVENTS
11:83282740	*DLG2*	REACTOME_ACTIVATION_OF_NMDA_RECEPTORS_AND_POSTSYNAPTIC_EVENTS
11:83387273	*DLG2*	REACTOME_ACTIVATION_OF_NMDA_RECEPTORS_AND_POSTSYNAPTIC_EVENTS
11:83538733	*DLG2*	REACTOME_ACTIVATION_OF_NMDA_RECEPTORS_AND_POSTSYNAPTIC_EVENTS
11:83544472	*DLG2*	REACTOME_ACTIVATION_OF_NMDA_RECEPTORS_AND_POSTSYNAPTIC_EVENTS
11:83578152	*DLG2*	REACTOME_ACTIVATION_OF_NMDA_RECEPTORS_AND_POSTSYNAPTIC_EVENTS
11:83647657	*DLG2*	REACTOME_ACTIVATION_OF_NMDA_RECEPTORS_AND_POSTSYNAPTIC_EVENTS
11:83669903	*DLG2*	REACTOME_ACTIVATION_OF_NMDA_RECEPTORS_AND_POSTSYNAPTIC_EVENTS
11:83736246	*DLG2*	REACTOME_ACTIVATION_OF_NMDA_RECEPTORS_AND_POSTSYNAPTIC_EVENTS
11:83762918	*DLG2*	REACTOME_ACTIVATION_OF_NMDA_RECEPTORS_AND_POSTSYNAPTIC_EVENTS
11:83823752	*DLG2*	REACTOME_ACTIVATION_OF_NMDA_RECEPTORS_AND_POSTSYNAPTIC_EVENTS
11:83839992	*DLG2*	REACTOME_ACTIVATION_OF_NMDA_RECEPTORS_AND_POSTSYNAPTIC_EVENTS
11:84104763	*DLG2*	REACTOME_ACTIVATION_OF_NMDA_RECEPTORS_AND_POSTSYNAPTIC_EVENTS
11:84169039	*DLG2*	REACTOME_ACTIVATION_OF_NMDA_RECEPTORS_AND_POSTSYNAPTIC_EVENTS
11:84221628	*DLG2*	REACTOME_ACTIVATION_OF_NMDA_RECEPTORS_AND_POSTSYNAPTIC_EVENTS
11:84312008	*DLG2*	REACTOME_ACTIVATION_OF_NMDA_RECEPTORS_AND_POSTSYNAPTIC_EVENTS
11:84324361	*DLG2*	REACTOME_ACTIVATION_OF_NMDA_RECEPTORS_AND_POSTSYNAPTIC_EVENTS
11:84360346	*DLG2*	REACTOME_ACTIVATION_OF_NMDA_RECEPTORS_AND_POSTSYNAPTIC_EVENTS
11:84403409	*DLG2*	REACTOME_ACTIVATION_OF_NMDA_RECEPTORS_AND_POSTSYNAPTIC_EVENTS
17:7101771	*V1NB1*	REACTOME_ACTIVATION_OF_NMDA_RECEPTORS_AND_POSTSYNAPTIC_EVENTS
17:43999203	*WNT3*	REACTOME_ACTIVATION_OF_NMDA_RECEPTORS_AND_POSTSYNAPTIC_EVENTS
17:44005297	*WNT3*	REACTOME_ACTIVATION_OF_NMDA_RECEPTORS_AND_POSTSYNAPTIC_EVENTS
17:44019712	*WNT3*	REACTOME_ACTIVATION_OF_NMDA_RECEPTORS_AND_POSTSYNAPTIC_EVENTS
17:44060492	*WNT3*	REACTOME_ACTIVATION_OF_NMDA_RECEPTORS_AND_POSTSYNAPTIC_EVENTS
17:44065263	*WNT3*	REACTOME_ACTIVATION_OF_NMDA_RECEPTORS_AND_POSTSYNAPTIC_EVENTS
17:44081178	*WNT3*	REACTOME_ACTIVATION_OF_NMDA_RECEPTORS_AND_POSTSYNAPTIC_EVENTS
17:44083297	*WNT3*	REACTOME_ACTIVATION_OF_NMDA_RECEPTORS_AND_POSTSYNAPTIC_EVENTS
17:7578671	*V1NB1*	REACTOME_ACTIVATION_OF_NOXA_AND_TRANSLOCATION_TO_MITOCHONDRIA
17:7578671	*V1NB1*	REACTOME_ACTIVATION_OF_PUMA_AND_TRANSLOCATION_TO_MITOCHONDRIA
17:7479140	*V1NB1*	ME_ACTIVATION_OF_THE_MRNA_UPON_BINDING_OF_THE_CAP_BINDING_COMPLEX_AND_EIFS_AND_SUBSEQUENT_BINDIN
17:7480325	*V1NB1*	ME_ACTIVATION_OF_THE_MRNA_UPON_BINDING_OF_THE_CAP_BINDING_COMPLEX_AND_EIFS_AND_SUBSEQUENT_BINDIN
12:133206648	*FBRSL1*	REACTOME_ACTIVATION_OF_THE_PRE_REPLICATIVE_COMPLEX

12:133223685	*FBRSL1*	REACTOME_ACTIVATION_OF_THE_PRE_REPLICATIVE_COMPLEX
20:5934513	*CRLS1*	REACTOME_ACTIVATION_OF_THE_PRE_REPLICATIVE_COMPLEX
20:5935522	*CRLS1*	REACTOME_ACTIVATION_OF_THE_PRE_REPLICATIVE_COMPLEX
20:5968737	*CRLS1*	REACTOME_ACTIVATION_OF_THE_PRE_REPLICATIVE_COMPLEX
20:5968964	*CRLS1*	REACTOME_ACTIVATION_OF_THE_PRE_REPLICATIVE_COMPLEX
20:6011934	*CRLS1*	REACTOME_ACYL_CHAIN_REMODELLING_OF_PG
1:171837985	*VAMP4*	REACTOME_ADAPTIVE_IMMUNE_SYSTEM
1:171945488	*VAMP4*	REACTOME_ADAPTIVE_IMMUNE_SYSTEM
1:172137119	*VAMP4*	REACTOME_ADAPTIVE_IMMUNE_SYSTEM
1:172386261	*VAMP4*	REACTOME_ADAPTIVE_IMMUNE_SYSTEM
3:49740895	*IP6K2*	REACTOME_ADAPTIVE_IMMUNE_SYSTEM
3:121319489	*KPNA1*	REACTOME_ADAPTIVE_IMMUNE_SYSTEM
3:121324643	*KPNA1*	REACTOME_ADAPTIVE_IMMUNE_SYSTEM
3:121340955	*KPNA1*	REACTOME_ADAPTIVE_IMMUNE_SYSTEM
3:122289921	*KPNA1*	REACTOME_ADAPTIVE_IMMUNE_SYSTEM
4:15608677	*BST1*	REACTOME_ADAPTIVE_IMMUNE_SYSTEM
4:15618898	*BST1*	REACTOME_ADAPTIVE_IMMUNE_SYSTEM
4:76407998	*FAM47E-STBD1*	REACTOME_ADAPTIVE_IMMUNE_SYSTEM
4:170015977	*CLCN3*	REACTOME_ADAPTIVE_IMMUNE_SYSTEM
4:170031333	*CLCN3*	REACTOME_ADAPTIVE_IMMUNE_SYSTEM
4:170101917	*CLCN3*	REACTOME_ADAPTIVE_IMMUNE_SYSTEM
4:170161031	*CLCN3*	REACTOME_ADAPTIVE_IMMUNE_SYSTEM
5:133558885	*C5orf24*	REACTOME_ADAPTIVE_IMMUNE_SYSTEM
5:133710339	*C5orf24*	REACTOME_ADAPTIVE_IMMUNE_SYSTEM
6:29798642	*TRIM40*	REACTOME_ADAPTIVE_IMMUNE_SYSTEM
6:32602372	*HLA-DRB5*	REACTOME_ADAPTIVE_IMMUNE_SYSTEM
6:32789400	*HLA-DRB5*	REACTOME_ADAPTIVE_IMMUNE_SYSTEM
6:32791831	*HLA-DRB5*	REACTOME_ADAPTIVE_IMMUNE_SYSTEM
6:32797361	*HLA-DRB5*	REACTOME_ADAPTIVE_IMMUNE_SYSTEM
6:32802462	*HLA-DRB5*	REACTOME_ADAPTIVE_IMMUNE_SYSTEM
6:32930679	*HLA-DRB5*	REACTOME_ADAPTIVE_IMMUNE_SYSTEM
6:32934409	*HLA-DRB5*	REACTOME_ADAPTIVE_IMMUNE_SYSTEM
6:32973303	*HLA-DRB5*	REACTOME_ADAPTIVE_IMMUNE_SYSTEM
6:32974268	*HLA-DRB5*	REACTOME_ADAPTIVE_IMMUNE_SYSTEM
6:112137847	*FYN*	REACTOME_ADAPTIVE_IMMUNE_SYSTEM
6:112151452	*FYN*	REACTOME_ADAPTIVE_IMMUNE_SYSTEM
6:112175069	*FYN*	REACTOME_ADAPTIVE_IMMUNE_SYSTEM
7:66096028	*GS1-124K5.11*	REACTOME_ADAPTIVE_IMMUNE_SYSTEM
7:66136281	*GS1-124K5.11*	REACTOME_ADAPTIVE_IMMUNE_SYSTEM
8:11353586	*CTSB*	REACTOME_ADAPTIVE_IMMUNE_SYSTEM
8:11363667	*CTSB*	REACTOME_ADAPTIVE_IMMUNE_SYSTEM
8:11381148	*CTSB*	REACTOME_ADAPTIVE_IMMUNE_SYSTEM
8:11712443	*CTSB*	REACTOME_ADAPTIVE_IMMUNE_SYSTEM
9:17579690	*SH3GL2_2*	REACTOME_ADAPTIVE_IMMUNE_SYSTEM
9:17587160	*SH3GL2_2*	REACTOME_ADAPTIVE_IMMUNE_SYSTEM
9:17609302	*SH3GL2_2*	REACTOME_ADAPTIVE_IMMUNE_SYSTEM
9:17626762	*SH3GL2_2*	REACTOME_ADAPTIVE_IMMUNE_SYSTEM
9:17627444	*SH3GL2_2*	REACTOME_ADAPTIVE_IMMUNE_SYSTEM
9:17642664	*SH3GL2_2*	REACTOME_ADAPTIVE_IMMUNE_SYSTEM
9:17710876	*SH3GL2_2*	REACTOME_ADAPTIVE_IMMUNE_SYSTEM
9:17732590	*SH3GL2_2*	REACTOME_ADAPTIVE_IMMUNE_SYSTEM
9:17737514	*SH3GL2_2*	REACTOME_ADAPTIVE_IMMUNE_SYSTEM
9:17758945	*SH3GL2_2*	REACTOME_ADAPTIVE_IMMUNE_SYSTEM

9:33845203	*UBAP2*	REACTOME_ADAPTIVE_IMMUNE_SYSTEM
10:103185542	*GBF1*	REACTOME_ADAPTIVE_IMMUNE_SYSTEM
10:103305087	*GBF1*	REACTOME_ADAPTIVE_IMMUNE_SYSTEM
10:103308244	*GBF1*	REACTOME_ADAPTIVE_IMMUNE_SYSTEM
10:104258303	*GBF1*	REACTOME_ADAPTIVE_IMMUNE_SYSTEM
16:30510571	*SETD1A*	REACTOME_ADAPTIVE_IMMUNE_SYSTEM
16:30516492	*SETD1A*	REACTOME_ADAPTIVE_IMMUNE_SYSTEM
16:30520856	*SETD1A*	REACTOME_ADAPTIVE_IMMUNE_SYSTEM
17:43390763	*CRHR1_2*	REACTOME_ADAPTIVE_IMMUNE_SYSTEM
17:45215865	*WNT3*	REACTOME_ADAPTIVE_IMMUNE_SYSTEM
17:45244822	*WNT3*	REACTOME_ADAPTIVE_IMMUNE_SYSTEM
3:123089448	*KPNA1*	REACTOME_ADENYLATE_CYCLASE_ACTIVATING_PATHWAY
8:131799767	*FAM49B*	REACTOME_ADENYLATE_CYCLASE_ACTIVATING_PATHWAY
8:131845512	*FAM49B*	REACTOME_ADENYLATE_CYCLASE_ACTIVATING_PATHWAY
8:131865425	*FAM49B*	REACTOME_ADENYLATE_CYCLASE_ACTIVATING_PATHWAY
8:131901170	*FAM49B*	REACTOME_ADENYLATE_CYCLASE_ACTIVATING_PATHWAY
16:50300511	*CASC16*	REACTOME_ADENYLATE_CYCLASE_ACTIVATING_PATHWAY
16:50317039	*CASC16*	REACTOME_ADENYLATE_CYCLASE_ACTIVATING_PATHWAY
3:123089448	*KPNA1*	REACTOME_ADENYLATE_CYCLASE_INHIBITORY_PATHWAY
8:131799767	*FAM49B*	REACTOME_ADENYLATE_CYCLASE_INHIBITORY_PATHWAY
8:131845512	*FAM49B*	REACTOME_ADENYLATE_CYCLASE_INHIBITORY_PATHWAY
8:131865425	*FAM49B*	REACTOME_ADENYLATE_CYCLASE_INHIBITORY_PATHWAY
8:131901170	*FAM49B*	REACTOME_ADENYLATE_CYCLASE_INHIBITORY_PATHWAY
16:50300511	*CASC16*	REACTOME_ADENYLATE_CYCLASE_INHIBITORY_PATHWAY
16:50317039	*CASC16*	REACTOME_ADENYLATE_CYCLASE_INHIBITORY_PATHWAY
17:39883520	*RETREG3*	REACTOME_ADHERENS_JUNCTIONS_INTERACTIONS
3:151090424	*MED12L*	REACTOME_ADP_SIGNALLING_THROUGH_P2Y_PURINOCEPTOR_12
3:151099733	*MED12L*	REACTOME_ADP_SIGNALLING_THROUGH_P2Y_PURINOCEPTOR_12
19:2557113	*SPPL2B*	REACTOME_ADP_SIGNALLING_THROUGH_P2Y_PURINOCEPTOR_12
19:2563327	*SPPL2B*	REACTOME_ADP_SIGNALLING_THROUGH_P2Y_PURINOCEPTOR_12
19:2580057	*SPPL2B*	REACTOME_ADP_SIGNALLING_THROUGH_P2Y_PURINOCEPTOR_12
19:2660983	*SPPL2B*	REACTOME_ADP_SIGNALLING_THROUGH_P2Y_PURINOCEPTOR_12
19:2557113	*SPPL2B*	REACTOME_ADP_SIGNALLING_THROUGH_P2Y_PURINOCEPTOR_1
19:2563327	*SPPL2B*	REACTOME_ADP_SIGNALLING_THROUGH_P2Y_PURINOCEPTOR_1
19:2580057	*SPPL2B*	REACTOME_ADP_SIGNALLING_THROUGH_P2Y_PURINOCEPTOR_1
19:2660983	*SPPL2B*	REACTOME_ADP_SIGNALLING_THROUGH_P2Y_PURINOCEPTOR_1
19:3142784	*SPPL2B*	REACTOME_ADP_SIGNALLING_THROUGH_P2Y_PURINOCEPTOR_1
19:3147326	*SPPL2B*	REACTOME_ADP_SIGNALLING_THROUGH_P2Y_PURINOCEPTOR_1
3:123089448	*KPNA1*	REACTOME_ADRENALINE_NORADRENALINE_INHIBITS_INSULIN_SECRETION
19:2557113	*SPPL2B*	REACTOME_ADRENALINE_NORADRENALINE_INHIBITS_INSULIN_SECRETION
19:2563327	*SPPL2B*	REACTOME_ADRENALINE_NORADRENALINE_INHIBITS_INSULIN_SECRETION
19:2580057	*SPPL2B*	REACTOME_ADRENALINE_NORADRENALINE_INHIBITS_INSULIN_SECRETION
19:2660983	*SPPL2B*	REACTOME_ADRENALINE_NORADRENALINE_INHIBITS_INSULIN_SECRETION
6:32151443	*HLA-DRB5*	REACTOME_ADVANCED_GLYCOSYLATION_ENDPRODUCT_RECEPTOR_SIGNALING
9:33791164	*UBAP2*	REACTOME_ALPHA_DEFENSINS
6:31897219	*HLA-DRB5*	REACTOME_ALTERNATIVE_COMPLEMENT_ACTIVATION
8:17390689	*FGF20*	REACTOME_AMINO_ACID_TRANSPORT_ACROSS_THE_PLASMA_MEMBRANE
8:17402274	*FGF20*	REACTOME_AMINO_ACID_TRANSPORT_ACROSS_THE_PLASMA_MEMBRANE
8:17427186	*FGF20*	REACTOME_AMINO_ACID_TRANSPORT_ACROSS_THE_PLASMA_MEMBRANE
12:46618863	*SCAF11*	REACTOME_AMINO_ACID_TRANSPORT_ACROSS_THE_PLASMA_MEMBRANE
12:47192323	*SCAF11*	REACTOME_AMINO_ACID_TRANSPORT_ACROSS_THE_PLASMA_MEMBRANE
12:47222402	*SCAF11*	REACTOME_AMINO_ACID_TRANSPORT_ACROSS_THE_PLASMA_MEMBRANE
4:90677243	*SNCA_2*	REACTOME_AMYLOID_FIBER_FORMATION

4:90700329	*SNCA_2*	REACTOME_AMYLOID_FIBER_FORMATION
4:90703993	*SNCA_2*	REACTOME_AMYLOID_FIBER_FORMATION
4:90738839	*SNCA_2*	REACTOME_AMYLOID_FIBER_FORMATION
4:90757294	*SNCA_2*	REACTOME_AMYLOID_FIBER_FORMATION
3:121553904	*KPNA1*	REACTOME_ANCHORING_OF_THE_BASAL_BODY_TO_THE_PLASMA_MEMBRANE
4:15492268	*BST1*	REACTOME_ANCHORING_OF_THE_BASAL_BODY_TO_THE_PLASMA_MEMBRANE
4:15547513	*BST1*	REACTOME_ANCHORING_OF_THE_BASAL_BODY_TO_THE_PLASMA_MEMBRANE
9:34300212	*UBAP2*	REACTOME_ANCHORING_OF_THE_BASAL_BODY_TO_THE_PLASMA_MEMBRANE
10:104258303	*GBF1*	REACTOME_ANCHORING_OF_THE_BASAL_BODY_TO_THE_PLASMA_MEMBRANE
16:19562008	*SYT17*	REACTOME_ANCHORING_OF_THE_BASAL_BODY_TO_THE_PLASMA_MEMBRANE
10:104591639	*GBF1*	REACTOME_ANDROGEN_BIOSYNTHESIS
6:112137847	*FYN*	REACTOME_ANTIGEN_ACTIVATES_B_CELL_RECEPTOR_BCR_LEADING_TO_GENERATION_OF_SECOND_MESSENGERS
6:112151452	*FYN*	REACTOME_ANTIGEN_ACTIVATES_B_CELL_RECEPTOR_BCR_LEADING_TO_GENERATION_OF_SECOND_MESSENGERS
6:112175069	*FYN*	REACTOME_ANTIGEN_ACTIVATES_B_CELL_RECEPTOR_BCR_LEADING_TO_GENERATION_OF_SECOND_MESSENGERS
8:11353586	*CTSB*	REACTOME_ANTIGEN_ACTIVATES_B_CELL_RECEPTOR_BCR_LEADING_TO_GENERATION_OF_SECOND_MESSENGERS
8:11363667	*CTSB*	REACTOME_ANTIGEN_ACTIVATES_B_CELL_RECEPTOR_BCR_LEADING_TO_GENERATION_OF_SECOND_MESSENGERS
8:11381148	*CTSB*	REACTOME_ANTIGEN_ACTIVATES_B_CELL_RECEPTOR_BCR_LEADING_TO_GENERATION_OF_SECOND_MESSENGERS
6:29798642	*TRIM40*	REACTOME_ANTIGEN_PRESENTATION:_FOLDING_ASSEMBLY_AND_PEPTIDE_LOADING_OF_CLASS_I_MHC
6:32789400	*HLA-DRB5*	REACTOME_ANTIGEN_PRESENTATION:_FOLDING_ASSEMBLY_AND_PEPTIDE_LOADING_OF_CLASS_I_MHC
6:32791831	*HLA-DRB5*	REACTOME_ANTIGEN_PRESENTATION:_FOLDING_ASSEMBLY_AND_PEPTIDE_LOADING_OF_CLASS_I_MHC
6:32797361	*HLA-DRB5*	REACTOME_ANTIGEN_PRESENTATION:_FOLDING_ASSEMBLY_AND_PEPTIDE_LOADING_OF_CLASS_I_MHC
6:32802462	*HLA-DRB5*	REACTOME_ANTIGEN_PRESENTATION:_FOLDING_ASSEMBLY_AND_PEPTIDE_LOADING_OF_CLASS_I_MHC
6:29798642	*TRIM40*	REACTOME_ANTIGEN_PROCESSING_CROSS_PRESENTATION
6:32789400	*HLA-DRB5*	REACTOME_ANTIGEN_PROCESSING_CROSS_PRESENTATION
6:32791831	*HLA-DRB5*	REACTOME_ANTIGEN_PROCESSING_CROSS_PRESENTATION
6:32797361	*HLA-DRB5*	REACTOME_ANTIGEN_PROCESSING_CROSS_PRESENTATION
6:32802462	*HLA-DRB5*	REACTOME_ANTIGEN_PROCESSING_CROSS_PRESENTATION
3:49740895	*IP6K2*	REACTOME_ANTIGEN_PROCESSING:_UBIQUITINATION_PROTEASOME_DEGRADATION
3:121319489	*KPNA1*	REACTOME_ANTIGEN_PROCESSING:_UBIQUITINATION_PROTEASOME_DEGRADATION
3:121324643	*KPNA1*	REACTOME_ANTIGEN_PROCESSING:_UBIQUITINATION_PROTEASOME_DEGRADATION
3:121340955	*KPNA1*	REACTOME_ANTIGEN_PROCESSING:_UBIQUITINATION_PROTEASOME_DEGRADATION
3:122289921	*KPNA1*	REACTOME_ANTIGEN_PROCESSING:_UBIQUITINATION_PROTEASOME_DEGRADATION
4:15608677	*BST1*	REACTOME_ANTIGEN_PROCESSING:_UBIQUITINATION_PROTEASOME_DEGRADATION
4:15618898	*BST1*	REACTOME_ANTIGEN_PROCESSING:_UBIQUITINATION_PROTEASOME_DEGRADATION
4:76407998	*FAM47E-STBD1*	REACTOME_ANTIGEN_PROCESSING:_UBIQUITINATION_PROTEASOME_DEGRADATION
4:170015977	*CLCN3*	REACTOME_ANTIGEN_PROCESSING:_UBIQUITINATION_PROTEASOME_DEGRADATION
4:170031333	*CLCN3*	REACTOME_ANTIGEN_PROCESSING:_UBIQUITINATION_PROTEASOME_DEGRADATION
4:170101917	*CLCN3*	REACTOME_ANTIGEN_PROCESSING:_UBIQUITINATION_PROTEASOME_DEGRADATION
4:170161031	*CLCN3*	REACTOME_ANTIGEN_PROCESSING:_UBIQUITINATION_PROTEASOME_DEGRADATION
5:133710339	*C5orf24*	REACTOME_ANTIGEN_PROCESSING:_UBIQUITINATION_PROTEASOME_DEGRADATION
7:66096028	*GS1-124K5.11*	REACTOME_ANTIGEN_PROCESSING:_UBIQUITINATION_PROTEASOME_DEGRADATION
7:66136281	*GS1-124K5.11*	REACTOME_ANTIGEN_PROCESSING:_UBIQUITINATION_PROTEASOME_DEGRADATION
9:33845203	*UBAP2*	REACTOME_ANTIGEN_PROCESSING:_UBIQUITINATION_PROTEASOME_DEGRADATION
10:103185542	*GBF1*	REACTOME_ANTIGEN_PROCESSING:_UBIQUITINATION_PROTEASOME_DEGRADATION
10:103305087	*GBF1*	REACTOME_ANTIGEN_PROCESSING:_UBIQUITINATION_PROTEASOME_DEGRADATION
10:103308244	*GBF1*	REACTOME_ANTIGEN_PROCESSING:_UBIQUITINATION_PROTEASOME_DEGRADATION
17:45215865	*WNT3*	REACTOME_ANTIGEN_PROCESSING:_UBIQUITINATION_PROTEASOME_DEGRADATION
17:45244822	*WNT3*	REACTOME_ANTIGEN_PROCESSING:_UBIQUITINATION_PROTEASOME_DEGRADATION
9:33791164	*UBAP2*	REACTOME_ANTIMICROBIAL_PEPTIDES
3:122200170	*KPNA1*	REACTOME_ANTIVIRAL_MECHANISM_BY_IFN_STIMULATED_GENES
17:7479140	*V1NB1*	REACTOME_ANTIVIRAL_MECHANISM_BY_IFN_STIMULATED_GENES
17:7480325	*V1NB1*	REACTOME_ANTIVIRAL_MECHANISM_BY_IFN_STIMULATED_GENES
17:45215865	*WNT3*	REACTOME_APC_C:CDC20_MEDIATED_DEGRADATION_OF_CYCLIN_B

17:45244822	*WNT3*	REACTOME_APC_C:CDC20_MEDIATED_DEGRADATION_OF_CYCLIN_B
17:45215865	*WNT3*	ME_APC_C:CDH1_MEDIATED_DEGRADATION_OF_CDC20_AND_OTHER_APC_C:CDH1_TARGETED_PROTEINS_IN_LATE_MITOSIS_
17:45244822	*WNT3*	ME_APC_C:CDH1_MEDIATED_DEGRADATION_OF_CDC20_AND_OTHER_APC_C:CDH1_TARGETED_PROTEINS_IN_LATE_MITOSIS_
17:45215865	*WNT3*	REACTOME_APC_CDC20_MEDIATED_DEGRADATION_OF_NEK2A
17:45244822	*WNT3*	REACTOME_APC_CDC20_MEDIATED_DEGRADATION_OF_NEK2A
3:122200170	*KPNA1*	REACTOME_APOPTOSIS_INDUCED_DNA_FRAGMENTATION
1:156059276	*GBAP1*	REACTOME_APOPTOTIC_CLEAVAGE_OF_CELLULAR_PROTEINS
3:18395010	*SATB1*	REACTOME_APOPTOTIC_CLEAVAGE_OF_CELLULAR_PROTEINS
3:18472577	*SATB1*	REACTOME_APOPTOTIC_CLEAVAGE_OF_CELLULAR_PROTEINS
17:43999203	*WNT3*	REACTOME_APOPTOTIC_CLEAVAGE_OF_CELLULAR_PROTEINS
17:44005297	*WNT3*	REACTOME_APOPTOTIC_CLEAVAGE_OF_CELLULAR_PROTEINS
17:44019712	*WNT3*	REACTOME_APOPTOTIC_CLEAVAGE_OF_CELLULAR_PROTEINS
17:44060492	*WNT3*	REACTOME_APOPTOTIC_CLEAVAGE_OF_CELLULAR_PROTEINS
17:44065263	*WNT3*	REACTOME_APOPTOTIC_CLEAVAGE_OF_CELLULAR_PROTEINS
17:44081178	*WNT3*	REACTOME_APOPTOTIC_CLEAVAGE_OF_CELLULAR_PROTEINS
17:44083297	*WNT3*	REACTOME_APOPTOTIC_CLEAVAGE_OF_CELLULAR_PROTEINS
1:156059276	*GBAP1*	REACTOME_APOPTOTIC_EXECUTION_PHASE
3:18395010	*SATB1*	REACTOME_APOPTOTIC_EXECUTION_PHASE
3:18472577	*SATB1*	REACTOME_APOPTOTIC_EXECUTION_PHASE
3:122200170	*KPNA1*	REACTOME_APOPTOTIC_EXECUTION_PHASE
17:43999203	*WNT3*	REACTOME_APOPTOTIC_EXECUTION_PHASE
17:44005297	*WNT3*	REACTOME_APOPTOTIC_EXECUTION_PHASE
17:44019712	*WNT3*	REACTOME_APOPTOTIC_EXECUTION_PHASE
17:44060492	*WNT3*	REACTOME_APOPTOTIC_EXECUTION_PHASE
17:44065263	*WNT3*	REACTOME_APOPTOTIC_EXECUTION_PHASE
17:44081178	*WNT3*	REACTOME_APOPTOTIC_EXECUTION_PHASE
17:44083297	*WNT3*	REACTOME_APOPTOTIC_EXECUTION_PHASE
3:48835245	*IP6K2*	REACTOME_AQUAPORIN_MEDIATED_TRANSPORT
3:123089448	*KPNA1*	REACTOME_AQUAPORIN_MEDIATED_TRANSPORT
8:131799767	*FAM49B*	REACTOME_AQUAPORIN_MEDIATED_TRANSPORT
8:131845512	*FAM49B*	REACTOME_AQUAPORIN_MEDIATED_TRANSPORT
8:131865425	*FAM49B*	REACTOME_AQUAPORIN_MEDIATED_TRANSPORT
8:131901170	*FAM49B*	REACTOME_AQUAPORIN_MEDIATED_TRANSPORT
9:33447547	*UBAP2*	REACTOME_AQUAPORIN_MEDIATED_TRANSPORT
16:50300511	*CASC16*	REACTOME_AQUAPORIN_MEDIATED_TRANSPORT
16:50317039	*CASC16*	REACTOME_AQUAPORIN_MEDIATED_TRANSPORT
19:2557113	*SPPL2B*	REACTOME_AQUAPORIN_MEDIATED_TRANSPORT
19:2563327	*SPPL2B*	REACTOME_AQUAPORIN_MEDIATED_TRANSPORT
19:2580057	*SPPL2B*	REACTOME_AQUAPORIN_MEDIATED_TRANSPORT
19:2660983	*SPPL2B*	REACTOME_AQUAPORIN_MEDIATED_TRANSPORT
17:7983799	*V1NB1*	REACTOME_ARACHIDONIC_ACID_METABOLISM
17:8019125	*V1NB1*	REACTOME_ARACHIDONIC_ACID_METABOLISM
2:134902070	*TMEM163*	REACTOME_ASPARAGINE_N_LINKED_GLYCOSYLATION
2:134932210	*TMEM163*	REACTOME_ASPARAGINE_N_LINKED_GLYCOSYLATION
2:134956835	*TMEM163*	REACTOME_ASPARAGINE_N_LINKED_GLYCOSYLATION
2:134986578	*TMEM163*	REACTOME_ASPARAGINE_N_LINKED_GLYCOSYLATION
2:134993021	*TMEM163*	REACTOME_ASPARAGINE_N_LINKED_GLYCOSYLATION
2:135035038	*TMEM163*	REACTOME_ASPARAGINE_N_LINKED_GLYCOSYLATION
2:135040234	*TMEM163*	REACTOME_ASPARAGINE_N_LINKED_GLYCOSYLATION
2:135165078	*TMEM163*	REACTOME_ASPARAGINE_N_LINKED_GLYCOSYLATION
2:135166385	*TMEM163*	REACTOME_ASPARAGINE_N_LINKED_GLYCOSYLATION
3:121411876	*KPNA1*	REACTOME_ASPARAGINE_N_LINKED_GLYCOSYLATION
3:121412702	*KPNA1*	REACTOME_ASPARAGINE_N_LINKED_GLYCOSYLATION

3:122986429	*KPNA1*	REACTOME_ASPARAGINE_N_LINKED_GLYCOSYLATION
4:76695927	*FAM47E-STBD1*	REACTOME_ASPARAGINE_N_LINKED_GLYCOSYLATION
4:113947327	*CAMK2D*	REACTOME_ASPARAGINE_N_LINKED_GLYCOSYLATION
4:114015252	*CAMK2D*	REACTOME_ASPARAGINE_N_LINKED_GLYCOSYLATION
4:114032570	*CAMK2D*	REACTOME_ASPARAGINE_N_LINKED_GLYCOSYLATION
4:114067233	*CAMK2D*	REACTOME_ASPARAGINE_N_LINKED_GLYCOSYLATION
4:114077386	*CAMK2D*	REACTOME_ASPARAGINE_N_LINKED_GLYCOSYLATION
4:114113772	*CAMK2D*	REACTOME_ASPARAGINE_N_LINKED_GLYCOSYLATION
4:114122398	*CAMK2D*	REACTOME_ASPARAGINE_N_LINKED_GLYCOSYLATION
4:114172749	*CAMK2D*	REACTOME_ASPARAGINE_N_LINKED_GLYCOSYLATION
4:114217997	*CAMK2D*	REACTOME_ASPARAGINE_N_LINKED_GLYCOSYLATION
6:32151443	*HLA-DRB5*	REACTOME_ASPARAGINE_N_LINKED_GLYCOSYLATION
10:104005410	*GBF1*	REACTOME_ASPARAGINE_N_LINKED_GLYCOSYLATION
10:104034550	*GBF1*	REACTOME_ASPARAGINE_N_LINKED_GLYCOSYLATION
10:104258303	*GBF1*	REACTOME_ASPARAGINE_N_LINKED_GLYCOSYLATION
10:121669571	*INPP5F*	REACTOME_ASPARAGINE_N_LINKED_GLYCOSYLATION
10:121683206	*INPP5F*	REACTOME_ASPARAGINE_N_LINKED_GLYCOSYLATION
10:121686714	*INPP5F*	REACTOME_ASPARAGINE_N_LINKED_GLYCOSYLATION
10:121689030	*INPP5F*	REACTOME_ASPARAGINE_N_LINKED_GLYCOSYLATION
17:7491546	*V1NB1*	REACTOME_ASPARAGINE_N_LINKED_GLYCOSYLATION
17:44800946	*WNT3*	REACTOME_ASPARAGINE_N_LINKED_GLYCOSYLATION
17:44833217	*WNT3*	REACTOME_ASPARAGINE_N_LINKED_GLYCOSYLATION
17:45020426	*WNT3*	REACTOME_ASPARAGINE_N_LINKED_GLYCOSYLATION
17:45059032	*WNT3*	REACTOME_ASPARAGINE_N_LINKED_GLYCOSYLATION
17:45089680	*WNT3*	REACTOME_ASPARAGINE_N_LINKED_GLYCOSYLATION
4:114424939	*CAMK2D*	REACTOME_ASSEMBLY_AND_CELL_SURFACE_PRESENTATION_OF_NMDA_RECEPTORS
4:114494052	*CAMK2D*	REACTOME_ASSEMBLY_AND_CELL_SURFACE_PRESENTATION_OF_NMDA_RECEPTORS
4:114529492	*CAMK2D*	REACTOME_ASSEMBLY_AND_CELL_SURFACE_PRESENTATION_OF_NMDA_RECEPTORS
4:114539964	*CAMK2D*	REACTOME_ASSEMBLY_AND_CELL_SURFACE_PRESENTATION_OF_NMDA_RECEPTORS
4:114597918	*CAMK2D*	REACTOME_ASSEMBLY_AND_CELL_SURFACE_PRESENTATION_OF_NMDA_RECEPTORS
11:83219006	*DLG2*	REACTOME_ASSEMBLY_AND_CELL_SURFACE_PRESENTATION_OF_NMDA_RECEPTORS
11:83232915	*DLG2*	REACTOME_ASSEMBLY_AND_CELL_SURFACE_PRESENTATION_OF_NMDA_RECEPTORS
11:83282740	*DLG2*	REACTOME_ASSEMBLY_AND_CELL_SURFACE_PRESENTATION_OF_NMDA_RECEPTORS
11:83387273	*DLG2*	REACTOME_ASSEMBLY_AND_CELL_SURFACE_PRESENTATION_OF_NMDA_RECEPTORS
11:83538733	*DLG2*	REACTOME_ASSEMBLY_AND_CELL_SURFACE_PRESENTATION_OF_NMDA_RECEPTORS
11:83544472	*DLG2*	REACTOME_ASSEMBLY_AND_CELL_SURFACE_PRESENTATION_OF_NMDA_RECEPTORS
11:83578152	*DLG2*	REACTOME_ASSEMBLY_AND_CELL_SURFACE_PRESENTATION_OF_NMDA_RECEPTORS
11:83647657	*DLG2*	REACTOME_ASSEMBLY_AND_CELL_SURFACE_PRESENTATION_OF_NMDA_RECEPTORS
11:83669903	*DLG2*	REACTOME_ASSEMBLY_AND_CELL_SURFACE_PRESENTATION_OF_NMDA_RECEPTORS
11:83736246	*DLG2*	REACTOME_ASSEMBLY_AND_CELL_SURFACE_PRESENTATION_OF_NMDA_RECEPTORS
11:83762918	*DLG2*	REACTOME_ASSEMBLY_AND_CELL_SURFACE_PRESENTATION_OF_NMDA_RECEPTORS
11:83823752	*DLG2*	REACTOME_ASSEMBLY_AND_CELL_SURFACE_PRESENTATION_OF_NMDA_RECEPTORS
11:83839992	*DLG2*	REACTOME_ASSEMBLY_AND_CELL_SURFACE_PRESENTATION_OF_NMDA_RECEPTORS
11:84104763	*DLG2*	REACTOME_ASSEMBLY_AND_CELL_SURFACE_PRESENTATION_OF_NMDA_RECEPTORS
11:84169039	*DLG2*	REACTOME_ASSEMBLY_AND_CELL_SURFACE_PRESENTATION_OF_NMDA_RECEPTORS
11:84221628	*DLG2*	REACTOME_ASSEMBLY_AND_CELL_SURFACE_PRESENTATION_OF_NMDA_RECEPTORS
11:84312008	*DLG2*	REACTOME_ASSEMBLY_AND_CELL_SURFACE_PRESENTATION_OF_NMDA_RECEPTORS
11:84324361	*DLG2*	REACTOME_ASSEMBLY_AND_CELL_SURFACE_PRESENTATION_OF_NMDA_RECEPTORS
11:84360346	*DLG2*	REACTOME_ASSEMBLY_AND_CELL_SURFACE_PRESENTATION_OF_NMDA_RECEPTORS
11:84403409	*DLG2*	REACTOME_ASSEMBLY_AND_CELL_SURFACE_PRESENTATION_OF_NMDA_RECEPTORS
17:7101771	*V1NB1*	REACTOME_ASSEMBLY_AND_CELL_SURFACE_PRESENTATION_OF_NMDA_RECEPTORS
6:33153528	*HLA-DRB5*	REACTOME_ASSEMBLY_OF_COLLAGEN_FIBRILS_AND_OTHER_MULTIMERIC_STRUCTURES
8:11712443	*CTSB*	REACTOME_ASSEMBLY_OF_COLLAGEN_FIBRILS_AND_OTHER_MULTIMERIC_STRUCTURES

8:23190941	*BIN3*	REACTOME_ASSEMBLY_OF_COLLAGEN_FIBRILS_AND_OTHER_MULTIMERIC_STRUCTURES
8:23212542	*BIN3*	REACTOME_ASSEMBLY_OF_COLLAGEN_FIBRILS_AND_OTHER_MULTIMERIC_STRUCTURES
8:23216225	*BIN3*	REACTOME_ASSEMBLY_OF_COLLAGEN_FIBRILS_AND_OTHER_MULTIMERIC_STRUCTURES
8:23223713	*BIN3*	REACTOME_ASSEMBLY_OF_COLLAGEN_FIBRILS_AND_OTHER_MULTIMERIC_STRUCTURES
8:17125768	*FGF20*	REACTOME_ASSEMBLY_OF_THE_HIV_VIRION
10:15199347	*ITGA8*	REACTOME_ASSEMBLY_OF_THE_HIV_VIRION
20:5934513	*CRLS1*	REACTOME_ASSEMBLY_OF_THE_PRE_REPLICATIVE_COMPLEX
20:5935522	*CRLS1*	REACTOME_ASSEMBLY_OF_THE_PRE_REPLICATIVE_COMPLEX
20:5968737	*CRLS1*	REACTOME_ASSEMBLY_OF_THE_PRE_REPLICATIVE_COMPLEX
20:5968964	*CRLS1*	REACTOME_ASSEMBLY_OF_THE_PRE_REPLICATIVE_COMPLEX
1:155205634	*GBAP1*	REACTOME_ASSOCIATION_OF_TRIC_CCT_WITH_TARGET_PROTEINS_DURING_BIOSYNTHESIS
4:15608677	*BST1*	REACTOME_ASSOCIATION_OF_TRIC_CCT_WITH_TARGET_PROTEINS_DURING_BIOSYNTHESIS
4:15618898	*BST1*	REACTOME_ASSOCIATION_OF_TRIC_CCT_WITH_TARGET_PROTEINS_DURING_BIOSYNTHESIS
17:7129974	*V1NB1*	REACTOME_ASYMMETRIC_LOCALIZATION_OF_PCP_PROTEINS
6:71594785	*RIMS1*	REACTOME_A_TETRASACCHARIDE_LINKER_SEQUENCE_IS_REQUIRED_FOR_GAG_SYNTHESIS
11:134255325	*IGSF9B*	REACTOME_A_TETRASACCHARIDE_LINKER_SEQUENCE_IS_REQUIRED_FOR_GAG_SYNTHESIS
1:161875645	*FCGR2A*	REACTOME_ATF4_ACTIVATES_GENES_IN_RESPONSE_TO_ENDOPLASMIC_RETICULUM_STRESS
1:161930954	*FCGR2A*	REACTOME_ATF4_ACTIVATES_GENES_IN_RESPONSE_TO_ENDOPLASMIC_RETICULUM_STRESS
1:161875645	*FCGR2A*	REACTOME_ATF6_ATF6_ALPHA_ACTIVATES_CHAPERONE_GENES
1:161930954	*FCGR2A*	REACTOME_ATF6_ATF6_ALPHA_ACTIVATES_CHAPERONE_GENES
1:161875645	*FCGR2A*	REACTOME_ATF6_ATF6_ALPHA_ACTIVATES_CHAPERONES
1:161930954	*FCGR2A*	REACTOME_ATF6_ATF6_ALPHA_ACTIVATES_CHAPERONES
10:104258303	*GBF1*	REACTOME_AURKA_ACTIVATION_BY_TPX2
16:19562008	*SYT17*	REACTOME_AURKA_ACTIVATION_BY_TPX2
8:17125768	*FGF20*	REACTOME_AUTOPHAGY
12:133295501	*FBRSL1*	REACTOME_AUTOPHAGY
14:55871532	*GCH1*	REACTOME_AUTOPHAGY
17:42990894	*CRHR1_2*	REACTOME_AUTOPHAGY
1:171837985	*VAMP4*	REACTOME_AXON_GUIDANCE
1:171945488	*VAMP4*	REACTOME_AXON_GUIDANCE
1:172137119	*VAMP4*	REACTOME_AXON_GUIDANCE
1:172386261	*VAMP4*	REACTOME_AXON_GUIDANCE
1:204876966	*RAB29*	REACTOME_AXON_GUIDANCE
1:204940972	*RAB29*	REACTOME_AXON_GUIDANCE
1:227067656	*ITPKB*	REACTOME_AXON_GUIDANCE
3:48835245	*IP6K2*	REACTOME_AXON_GUIDANCE
3:49406095	*IP6K2*	REACTOME_AXON_GUIDANCE
3:49424231	*IP6K2*	REACTOME_AXON_GUIDANCE
3:49450137	*IP6K2*	REACTOME_AXON_GUIDANCE
3:49561208	*IP6K2*	REACTOME_AXON_GUIDANCE
4:113947327	*CAMK2D*	REACTOME_AXON_GUIDANCE
4:114015252	*CAMK2D*	REACTOME_AXON_GUIDANCE
4:114032570	*CAMK2D*	REACTOME_AXON_GUIDANCE
4:114067233	*CAMK2D*	REACTOME_AXON_GUIDANCE
4:114077386	*CAMK2D*	REACTOME_AXON_GUIDANCE
4:114113772	*CAMK2D*	REACTOME_AXON_GUIDANCE
4:114122398	*CAMK2D*	REACTOME_AXON_GUIDANCE
4:114172749	*CAMK2D*	REACTOME_AXON_GUIDANCE
4:114217997	*CAMK2D*	REACTOME_AXON_GUIDANCE
6:112137847	*FYN*	REACTOME_AXON_GUIDANCE
6:112151452	*FYN*	REACTOME_AXON_GUIDANCE
6:112175069	*FYN*	REACTOME_AXON_GUIDANCE
8:21565088	*BIN3*	REACTOME_AXON_GUIDANCE

8:21582921	*BIN3*	REACTOME_AXON_GUIDANCE
8:21665306	*BIN3*	REACTOME_AXON_GUIDANCE
8:21668901	*BIN3*	REACTOME_AXON_GUIDANCE
9:17579690	*SH3GL2_2*	REACTOME_AXON_GUIDANCE
9:17587160	*SH3GL2_2*	REACTOME_AXON_GUIDANCE
9:17609302	*SH3GL2_2*	REACTOME_AXON_GUIDANCE
9:17626762	*SH3GL2_2*	REACTOME_AXON_GUIDANCE
9:17627444	*SH3GL2_2*	REACTOME_AXON_GUIDANCE
9:17642664	*SH3GL2_2*	REACTOME_AXON_GUIDANCE
9:17710876	*SH3GL2_2*	REACTOME_AXON_GUIDANCE
9:17732590	*SH3GL2_2*	REACTOME_AXON_GUIDANCE
9:17737514	*SH3GL2_2*	REACTOME_AXON_GUIDANCE
9:17758945	*SH3GL2_2*	REACTOME_AXON_GUIDANCE
12:41144387	*LRRK2_2*	REACTOME_AXON_GUIDANCE
12:41147295	*LRRK2_2*	REACTOME_AXON_GUIDANCE
12:41195135	*LRRK2_2*	REACTOME_AXON_GUIDANCE
12:41251554	*LRRK2_2*	REACTOME_AXON_GUIDANCE
12:41255129	*LRRK2_2*	REACTOME_AXON_GUIDANCE
12:41297969	*LRRK2_2*	REACTOME_AXON_GUIDANCE
12:41407403	*LRRK2_2*	REACTOME_AXON_GUIDANCE
17:7101771	*V1NB1*	REACTOME_AXON_GUIDANCE
17:42454243	*FAM171A2*	REACTOME_AXON_GUIDANCE
1:226568665	*ITPKB*	REACTOME_BASE_EXCISION_REPAIR
12:133206648	*FBRSL1*	REACTOME_BASE_EXCISION_REPAIR
12:133223685	*FBRSL1*	REACTOME_BASE_EXCISION_REPAIR
17:7560294	*V1NB1*	REACTOME_BASIGIN_INTERACTIONS
3:49406095	*IP6K2*	REACTOME_BETA_CATENIN_INDEPENDENT_WNT_SIGNALING
3:49424231	*IP6K2*	REACTOME_BETA_CATENIN_INDEPENDENT_WNT_SIGNALING
3:49450137	*IP6K2*	REACTOME_BETA_CATENIN_INDEPENDENT_WNT_SIGNALING
17:7129974	*V1NB1*	REACTOME_BETA_CATENIN_INDEPENDENT_WNT_SIGNALING
19:2557113	*SPPL2B*	REACTOME_BETA_CATENIN_INDEPENDENT_WNT_SIGNALING
19:2563327	*SPPL2B*	REACTOME_BETA_CATENIN_INDEPENDENT_WNT_SIGNALING
19:2580057	*SPPL2B*	REACTOME_BETA_CATENIN_INDEPENDENT_WNT_SIGNALING
19:2660983	*SPPL2B*	REACTOME_BETA_CATENIN_INDEPENDENT_WNT_SIGNALING
5:133558885	*C5orf24*	REACTOME_BETA_CATENIN_PHOSPHORYLATION_CASCADE
17:42332803	*FAM171A2*	REACTOME_BICARBONATE_TRANSPORTERS
17:42338993	*FAM171A2*	REACTOME_BICARBONATE_TRANSPORTERS
16:30999462	*SETD1A*	REACTOME_BILE_ACID_AND_BILE_SALT_METABOLISM
8:16086683	*FGF20*	REACTOME_BINDING_AND_UPTAKE_OF_LIGANDS_BY_SCAVENGER_RECEPTORS
8:16135246	*FGF20*	REACTOME_BINDING_AND_UPTAKE_OF_LIGANDS_BY_SCAVENGER_RECEPTORS
7:65705034	*GS1-124K5.11*	REACTOME_BIOLOGICAL_OXIDATIONS
7:65824287	*GS1-124K5.11*	REACTOME_BIOLOGICAL_OXIDATIONS
10:104635620	*GBF1*	REACTOME_BIOLOGICAL_OXIDATIONS
17:7491546	*V1NB1*	OSYNTHESIS_OF_THE_N_GLYCAN_PRECURSOR_DOLICHOL_LIPID_LINKED_OLIGOSACCHARIDE_LLO_AND_TRANSFER_TO_A_NA
3:182760073	*MCCC1*	REACTOME_BIOTIN_TRANSPORT_AND_METABOLISM
21:38136802	*DYRK1A*	REACTOME_BIOTIN_TRANSPORT_AND_METABOLISM
21:38168749	*DYRK1A*	REACTOME_BIOTIN_TRANSPORT_AND_METABOLISM
21:38210057	*DYRK1A*	REACTOME_BIOTIN_TRANSPORT_AND_METABOLISM
21:38238679	*DYRK1A*	REACTOME_BIOTIN_TRANSPORT_AND_METABOLISM
21:38328886	*DYRK1A*	REACTOME_BIOTIN_TRANSPORT_AND_METABOLISM
21:38332144	*DYRK1A*	REACTOME_BIOTIN_TRANSPORT_AND_METABOLISM
21:38356061	*DYRK1A*	REACTOME_BIOTIN_TRANSPORT_AND_METABOLISM
2:168682052	*STK39*	REACTOME_BLOOD_GROUP_SYSTEMS_BIOSYNTHESIS

2:101439596	*MAP4K4*	REACTOME_BMAL1:CLOCK_NPAS2_ACTIVATES_CIRCADIAN_GENE_EXPRESSION
2:101464631	*MAP4K4*	REACTOME_BMAL1:CLOCK_NPAS2_ACTIVATES_CIRCADIAN_GENE_EXPRESSION
2:101547064	*MAP4K4*	REACTOME_BMAL1:CLOCK_NPAS2_ACTIVATES_CIRCADIAN_GENE_EXPRESSION
2:101550181	*MAP4K4*	REACTOME_BMAL1:CLOCK_NPAS2_ACTIVATES_CIRCADIAN_GENE_EXPRESSION
2:101578010	*MAP4K4*	REACTOME_BMAL1:CLOCK_NPAS2_ACTIVATES_CIRCADIAN_GENE_EXPRESSION
2:101603812	*MAP4K4*	REACTOME_BMAL1:CLOCK_NPAS2_ACTIVATES_CIRCADIAN_GENE_EXPRESSION
3:182760073	*MCCC1*	REACTOME_BRANCHED_CHAIN_AMINO_ACID_CATABOLISM
11:134130174	*IGSF9B*	REACTOME_BRANCHED_CHAIN_AMINO_ACID_CATABOLISM
8:17125768	*FGF20*	REACTOME_BUDDING_AND_MATURATION_OF_HIV_VIRION
1:154701406	*GBAP1*	REACTOME_CA2PLUS_ACTIVATED_KPLUS_CHANNELS
1:154776412	*GBAP1*	REACTOME_CA2PLUS_ACTIVATED_KPLUS_CHANNELS
1:154801670	*GBAP1*	REACTOME_CA2PLUS_ACTIVATED_KPLUS_CHANNELS
1:154804988	*GBAP1*	REACTOME_CA2PLUS_ACTIVATED_KPLUS_CHANNELS
19:2557113	*SPPL2B*	REACTOME_CA2PLUS_PATHWAY
19:2563327	*SPPL2B*	REACTOME_CA2PLUS_PATHWAY
19:2580057	*SPPL2B*	REACTOME_CA2PLUS_PATHWAY
19:2660983	*SPPL2B*	REACTOME_CA2PLUS_PATHWAY
3:48835245	*IP6K2*	REACTOME_CA_DEPENDENT_EVENTS
3:123089448	*KPNA1*	REACTOME_CA_DEPENDENT_EVENTS
4:114424939	*CAMK2D*	REACTOME_CA_DEPENDENT_EVENTS
4:114494052	*CAMK2D*	REACTOME_CA_DEPENDENT_EVENTS
4:114529492	*CAMK2D*	REACTOME_CA_DEPENDENT_EVENTS
4:114539964	*CAMK2D*	REACTOME_CA_DEPENDENT_EVENTS
4:114597918	*CAMK2D*	REACTOME_CA_DEPENDENT_EVENTS
8:131799767	*FAM49B*	REACTOME_CA_DEPENDENT_EVENTS
8:131845512	*FAM49B*	REACTOME_CA_DEPENDENT_EVENTS
8:131865425	*FAM49B*	REACTOME_CA_DEPENDENT_EVENTS
8:131901170	*FAM49B*	REACTOME_CA_DEPENDENT_EVENTS
16:50300511	*CASC16*	REACTOME_CA_DEPENDENT_EVENTS
16:50317039	*CASC16*	REACTOME_CA_DEPENDENT_EVENTS
6:32151443	*HLA-DRB5*	REACTOME_CALNEXIN_CALRETICULIN_CYCLE
4:114424939	*CAMK2D*	REACTOME_CAMK_IV_MEDIATED_PHOSPHORYLATION_OF_CREB
4:114494052	*CAMK2D*	REACTOME_CAMK_IV_MEDIATED_PHOSPHORYLATION_OF_CREB
4:114529492	*CAMK2D*	REACTOME_CAMK_IV_MEDIATED_PHOSPHORYLATION_OF_CREB
4:114539964	*CAMK2D*	REACTOME_CAMK_IV_MEDIATED_PHOSPHORYLATION_OF_CREB
4:114597918	*CAMK2D*	REACTOME_CAMK_IV_MEDIATED_PHOSPHORYLATION_OF_CREB
3:49462410	*IP6K2*	REACTOME_CARBOXYTERMINAL_POST_TRANSLATIONAL_MODIFICATIONS_OF_TUBULIN
14:76135546	*RPS6KL1*	REACTOME_CARBOXYTERMINAL_POST_TRANSLATIONAL_MODIFICATIONS_OF_TUBULIN
14:76159192	*RPS6KL1*	REACTOME_CARBOXYTERMINAL_POST_TRANSLATIONAL_MODIFICATIONS_OF_TUBULIN
14:76169229	*RPS6KL1*	REACTOME_CARBOXYTERMINAL_POST_TRANSLATIONAL_MODIFICATIONS_OF_TUBULIN
14:76170371	*RPS6KL1*	REACTOME_CARBOXYTERMINAL_POST_TRANSLATIONAL_MODIFICATIONS_OF_TUBULIN
14:76244271	*RPS6KL1*	REACTOME_CARBOXYTERMINAL_POST_TRANSLATIONAL_MODIFICATIONS_OF_TUBULIN
2:95967628	*KCNIP3*	REACTOME_CARDIAC_CONDUCTION
4:114424939	*CAMK2D*	REACTOME_CARDIAC_CONDUCTION
4:114494052	*CAMK2D*	REACTOME_CARDIAC_CONDUCTION
4:114529492	*CAMK2D*	REACTOME_CARDIAC_CONDUCTION
4:114539964	*CAMK2D*	REACTOME_CARDIAC_CONDUCTION
4:114597918	*CAMK2D*	REACTOME_CARDIAC_CONDUCTION
8:11558567	*CTSB*	REACTOME_CARDIAC_CONDUCTION
8:11599269	*CTSB*	REACTOME_CARDIAC_CONDUCTION
14:88681675	*GALC*	REACTOME_CARDIAC_CONDUCTION
14:88709038	*GALC*	REACTOME_CARDIAC_CONDUCTION
14:88738350	*GALC*	REACTOME_CARDIAC_CONDUCTION

14:88769689	*GALC*	REACTOME_CARDIAC_CONDUCTION
14:88778263	*GALC*	REACTOME_CARDIAC_CONDUCTION
17:7560294	*V1NB1*	REACTOME_CARDIAC_CONDUCTION
17:45020426	*WNT3*	REACTOME_CARGO_CONCENTRATION_IN_THE_ER
17:45059032	*WNT3*	REACTOME_CARGO_CONCENTRATION_IN_THE_ER
17:45089680	*WNT3*	REACTOME_CARGO_CONCENTRATION_IN_THE_ER
1:155847427	*GBAP1*	REACTOME_CARGO_RECOGNITION_FOR_CLATHRIN_MEDIATED_ENDOCYTOSIS
1:171689486	*VAMP4*	REACTOME_CARGO_RECOGNITION_FOR_CLATHRIN_MEDIATED_ENDOCYTOSIS
2:170012307	*STK39*	REACTOME_CARGO_RECOGNITION_FOR_CLATHRIN_MEDIATED_ENDOCYTOSIS
2:170028024	*STK39*	REACTOME_CARGO_RECOGNITION_FOR_CLATHRIN_MEDIATED_ENDOCYTOSIS
2:170066424	*STK39*	REACTOME_CARGO_RECOGNITION_FOR_CLATHRIN_MEDIATED_ENDOCYTOSIS
4:77104152	*FAM47E-STBD1*	REACTOME_CARGO_RECOGNITION_FOR_CLATHRIN_MEDIATED_ENDOCYTOSIS
4:77124196	*FAM47E-STBD1*	REACTOME_CARGO_RECOGNITION_FOR_CLATHRIN_MEDIATED_ENDOCYTOSIS
4:77134786	*FAM47E-STBD1*	REACTOME_CARGO_RECOGNITION_FOR_CLATHRIN_MEDIATED_ENDOCYTOSIS
9:17579690	*SH3GL2_2*	REACTOME_CARGO_RECOGNITION_FOR_CLATHRIN_MEDIATED_ENDOCYTOSIS
9:17587160	*SH3GL2_2*	REACTOME_CARGO_RECOGNITION_FOR_CLATHRIN_MEDIATED_ENDOCYTOSIS
9:17609302	*SH3GL2_2*	REACTOME_CARGO_RECOGNITION_FOR_CLATHRIN_MEDIATED_ENDOCYTOSIS
9:17626762	*SH3GL2_2*	REACTOME_CARGO_RECOGNITION_FOR_CLATHRIN_MEDIATED_ENDOCYTOSIS
9:17627444	*SH3GL2_2*	REACTOME_CARGO_RECOGNITION_FOR_CLATHRIN_MEDIATED_ENDOCYTOSIS
9:17642664	*SH3GL2_2*	REACTOME_CARGO_RECOGNITION_FOR_CLATHRIN_MEDIATED_ENDOCYTOSIS
9:17710876	*SH3GL2_2*	REACTOME_CARGO_RECOGNITION_FOR_CLATHRIN_MEDIATED_ENDOCYTOSIS
9:17732590	*SH3GL2_2*	REACTOME_CARGO_RECOGNITION_FOR_CLATHRIN_MEDIATED_ENDOCYTOSIS
9:17737514	*SH3GL2_2*	REACTOME_CARGO_RECOGNITION_FOR_CLATHRIN_MEDIATED_ENDOCYTOSIS
9:17758945	*SH3GL2_2*	REACTOME_CARGO_RECOGNITION_FOR_CLATHRIN_MEDIATED_ENDOCYTOSIS
17:7129974	*V1NB1*	REACTOME_CARGO_RECOGNITION_FOR_CLATHRIN_MEDIATED_ENDOCYTOSIS
8:131145058	*FAM49B*	REACTOME_CARGO_TRAFFICKING_TO_THE_PERICILIARY_MEMBRANE
8:131153067	*FAM49B*	REACTOME_CARGO_TRAFFICKING_TO_THE_PERICILIARY_MEMBRANE
8:131245821	*FAM49B*	REACTOME_CARGO_TRAFFICKING_TO_THE_PERICILIARY_MEMBRANE
8:131328260	*FAM49B*	REACTOME_CARGO_TRAFFICKING_TO_THE_PERICILIARY_MEMBRANE
8:131404984	*FAM49B*	REACTOME_CARGO_TRAFFICKING_TO_THE_PERICILIARY_MEMBRANE
8:131408202	*FAM49B*	REACTOME_CARGO_TRAFFICKING_TO_THE_PERICILIARY_MEMBRANE
8:131455469	*FAM49B*	REACTOME_CARGO_TRAFFICKING_TO_THE_PERICILIARY_MEMBRANE
10:104005410	*GBF1*	REACTOME_CARGO_TRAFFICKING_TO_THE_PERICILIARY_MEMBRANE
10:104034550	*GBF1*	REACTOME_CARGO_TRAFFICKING_TO_THE_PERICILIARY_MEMBRANE
10:104443875	*GBF1*	REACTOME_CARGO_TRAFFICKING_TO_THE_PERICILIARY_MEMBRANE
10:104452818	*GBF1*	REACTOME_CARGO_TRAFFICKING_TO_THE_PERICILIARY_MEMBRANE
8:23050400	*BIN3*	REACTOME_CASPASE_ACTIVATION_VIA_DEATH_RECEPTORS_IN_THE_PRESENCE_OF_LIGAND
8:23050400	*BIN3*	REACTOME_CASPASE_ACTIVATION_VIA_EXTRINSIC_APOPTOTIC_SIGNALLING_PATHWAY
17:43999203	*WNT3*	REACTOME_CASPASE_MEDIATED_CLEAVAGE_OF_CYTOSKELETAL_PROTEINS
17:44005297	*WNT3*	REACTOME_CASPASE_MEDIATED_CLEAVAGE_OF_CYTOSKELETAL_PROTEINS
17:44019712	*WNT3*	REACTOME_CASPASE_MEDIATED_CLEAVAGE_OF_CYTOSKELETAL_PROTEINS
17:44060492	*WNT3*	REACTOME_CASPASE_MEDIATED_CLEAVAGE_OF_CYTOSKELETAL_PROTEINS
17:44065263	*WNT3*	REACTOME_CASPASE_MEDIATED_CLEAVAGE_OF_CYTOSKELETAL_PROTEINS
17:44081178	*WNT3*	REACTOME_CASPASE_MEDIATED_CLEAVAGE_OF_CYTOSKELETAL_PROTEINS
17:44083297	*WNT3*	REACTOME_CASPASE_MEDIATED_CLEAVAGE_OF_CYTOSKELETAL_PROTEINS
6:112137847	*FYN*	REACTOME_CD209_DC_SIGN_SIGNALING
6:112151452	*FYN*	REACTOME_CD209_DC_SIGN_SIGNALING
6:112175069	*FYN*	REACTOME_CD209_DC_SIGN_SIGNALING
6:112137847	*FYN*	REACTOME_CD28_CO_STIMULATION
6:112151452	*FYN*	REACTOME_CD28_CO_STIMULATION
6:112175069	*FYN*	REACTOME_CD28_CO_STIMULATION
17:43390763	*CRHR1_2*	REACTOME_CD28_CO_STIMULATION
6:112137847	*FYN*	REACTOME_CD28_DEPENDENT_PI3K_AKT_SIGNALING

6:112151452	*FYN*	REACTOME_CD28_DEPENDENT_PI3K_AKT_SIGNALING
6:112175069	*FYN*	REACTOME_CD28_DEPENDENT_PI3K_AKT_SIGNALING
17:43390763	*CRHR1_2*	REACTOME_CD28_DEPENDENT_PI3K_AKT_SIGNALING
6:112137847	*FYN*	REACTOME_CD28_DEPENDENT_VAV1_PATHWAY
6:112151452	*FYN*	REACTOME_CD28_DEPENDENT_VAV1_PATHWAY
6:112175069	*FYN*	REACTOME_CD28_DEPENDENT_VAV1_PATHWAY
20:5934513	*CRLS1*	REACTOME_CDC6_ASSOCIATION_WITH_THE_ORC:ORIGIN_COMPLEX
20:5935522	*CRLS1*	REACTOME_CDC6_ASSOCIATION_WITH_THE_ORC:ORIGIN_COMPLEX
20:5968737	*CRLS1*	REACTOME_CDC6_ASSOCIATION_WITH_THE_ORC:ORIGIN_COMPLEX
20:5968964	*CRLS1*	REACTOME_CDC6_ASSOCIATION_WITH_THE_ORC:ORIGIN_COMPLEX
17:45215865	*WNT3*	REACTOME_CDK_MEDIATED_PHOSPHORYLATION_AND_REMOVAL_OF_CDC6
17:45244822	*WNT3*	REACTOME_CDK_MEDIATED_PHOSPHORYLATION_AND_REMOVAL_OF_CDC6
6:112137847	*FYN*	REACTOME_CELL_CELL_COMMUNICATION
6:112151452	*FYN*	REACTOME_CELL_CELL_COMMUNICATION
6:112175069	*FYN*	REACTOME_CELL_CELL_COMMUNICATION
17:39883520	*RETREG3*	REACTOME_CELL_CELL_COMMUNICATION
21:37854286	*DYRK1A*	REACTOME_CELL_CELL_COMMUNICATION
21:37859449	*DYRK1A*	REACTOME_CELL_CELL_COMMUNICATION
21:37860785	*DYRK1A*	REACTOME_CELL_CELL_COMMUNICATION
21:37894089	*DYRK1A*	REACTOME_CELL_CELL_COMMUNICATION
21:37899076	*DYRK1A*	REACTOME_CELL_CELL_COMMUNICATION
17:39883520	*RETREG3*	REACTOME_CELL_CELL_JUNCTION_ORGANIZATION
21:37854286	*DYRK1A*	REACTOME_CELL_CELL_JUNCTION_ORGANIZATION
21:37859449	*DYRK1A*	REACTOME_CELL_CELL_JUNCTION_ORGANIZATION
21:37860785	*DYRK1A*	REACTOME_CELL_CELL_JUNCTION_ORGANIZATION
21:37894089	*DYRK1A*	REACTOME_CELL_CELL_JUNCTION_ORGANIZATION
21:37899076	*DYRK1A*	REACTOME_CELL_CELL_JUNCTION_ORGANIZATION
2:169747216	*STK39*	REACTOME_CELL_CYCLE_CHECKPOINTS
3:48199695	*IP6K2*	REACTOME_CELL_CYCLE_CHECKPOINTS
3:48214438	*IP6K2*	REACTOME_CELL_CYCLE_CHECKPOINTS
5:133558885	*C5orf24*	REACTOME_CELL_CYCLE_CHECKPOINTS
12:122816734	*HIP1R*	REACTOME_CELL_CYCLE_CHECKPOINTS
12:122817580	*HIP1R*	REACTOME_CELL_CYCLE_CHECKPOINTS
12:122853197	*HIP1R*	REACTOME_CELL_CYCLE_CHECKPOINTS
12:123057792	*HIP1R*	REACTOME_CELL_CYCLE_CHECKPOINTS
12:123110365	*HIP1R*	REACTOME_CELL_CYCLE_CHECKPOINTS
12:123110785	*HIP1R*	REACTOME_CELL_CYCLE_CHECKPOINTS
17:7578671	*V1NB1*	REACTOME_CELL_CYCLE_CHECKPOINTS
17:45215865	*WNT3*	REACTOME_CELL_CYCLE_CHECKPOINTS
17:45244822	*WNT3*	REACTOME_CELL_CYCLE_CHECKPOINTS
17:59791813	*BRIP1*	REACTOME_CELL_CYCLE_CHECKPOINTS
17:59922925	*BRIP1*	REACTOME_CELL_CYCLE_CHECKPOINTS
20:5934513	*CRLS1*	REACTOME_CELL_CYCLE_CHECKPOINTS
20:5935522	*CRLS1*	REACTOME_CELL_CYCLE_CHECKPOINTS
20:5968737	*CRLS1*	REACTOME_CELL_CYCLE_CHECKPOINTS
20:5968964	*CRLS1*	REACTOME_CELL_CYCLE_CHECKPOINTS
1:156059276	*GBAP1*	REACTOME_CELL_CYCLE_MITOTIC
1:226497419	*ITPKB*	REACTOME_CELL_CYCLE_MITOTIC
2:169747216	*STK39*	REACTOME_CELL_CYCLE_MITOTIC
3:48199695	*IP6K2*	REACTOME_CELL_CYCLE_MITOTIC
3:48214438	*IP6K2*	REACTOME_CELL_CYCLE_MITOTIC
4:76695927	*FAM47E-STBD1*	REACTOME_CELL_CYCLE_MITOTIC
5:133558885	*C5orf24*	REACTOME_CELL_CYCLE_MITOTIC

10:103185542	*GBF1*	REACTOME_CELL_CYCLE_MITOTIC
10:103305087	*GBF1*	REACTOME_CELL_CYCLE_MITOTIC
10:103308244	*GBF1*	REACTOME_CELL_CYCLE_MITOTIC
10:104258303	*GBF1*	REACTOME_CELL_CYCLE_MITOTIC
12:122816734	*HIP1R*	REACTOME_CELL_CYCLE_MITOTIC
12:122817580	*HIP1R*	REACTOME_CELL_CYCLE_MITOTIC
12:122853197	*HIP1R*	REACTOME_CELL_CYCLE_MITOTIC
12:123057792	*HIP1R*	REACTOME_CELL_CYCLE_MITOTIC
12:123110365	*HIP1R*	REACTOME_CELL_CYCLE_MITOTIC
12:123110785	*HIP1R*	REACTOME_CELL_CYCLE_MITOTIC
12:133206648	*FBRSL1*	REACTOME_CELL_CYCLE_MITOTIC
12:133223685	*FBRSL1*	REACTOME_CELL_CYCLE_MITOTIC
14:74640180	*RPS6KL1*	REACTOME_CELL_CYCLE_MITOTIC
16:19562008	*SYT17*	REACTOME_CELL_CYCLE_MITOTIC
17:7578671	*V1NB1*	REACTOME_CELL_CYCLE_MITOTIC
17:40818584	*RETREG3*	REACTOME_CELL_CYCLE_MITOTIC
17:45215865	*WNT3*	REACTOME_CELL_CYCLE_MITOTIC
17:45244822	*WNT3*	REACTOME_CELL_CYCLE_MITOTIC
17:76177567	*DNAH17*	REACTOME_CELL_CYCLE_MITOTIC
20:5934513	*CRLS1*	REACTOME_CELL_CYCLE_MITOTIC
20:5935522	*CRLS1*	REACTOME_CELL_CYCLE_MITOTIC
20:5968737	*CRLS1*	REACTOME_CELL_CYCLE_MITOTIC
20:5968964	*CRLS1*	REACTOME_CELL_CYCLE_MITOTIC
21:38740824	*DYRK1A*	REACTOME_CELL_CYCLE_MITOTIC
21:38753354	*DYRK1A*	REACTOME_CELL_CYCLE_MITOTIC
21:38770530	*DYRK1A*	REACTOME_CELL_CYCLE_MITOTIC
21:38783614	*DYRK1A*	REACTOME_CELL_CYCLE_MITOTIC
21:38814867	*DYRK1A*	REACTOME_CELL_CYCLE_MITOTIC
1:156059276	*GBAP1*	REACTOME_CELL_CYCLE
1:226497419	*ITPKB*	REACTOME_CELL_CYCLE
2:169747216	*STK39*	REACTOME_CELL_CYCLE
3:48199695	*IP6K2*	REACTOME_CELL_CYCLE
3:48214438	*IP6K2*	REACTOME_CELL_CYCLE
4:76695927	*FAM47E-STBD1*	REACTOME_CELL_CYCLE
5:133558885	*C5orf24*	REACTOME_CELL_CYCLE
6:31716436	*HLA-DRB5*	REACTOME_CELL_CYCLE
10:103185542	*GBF1*	REACTOME_CELL_CYCLE
10:103305087	*GBF1*	REACTOME_CELL_CYCLE
10:103308244	*GBF1*	REACTOME_CELL_CYCLE
10:104258303	*GBF1*	REACTOME_CELL_CYCLE
12:122816734	*HIP1R*	REACTOME_CELL_CYCLE
12:122817580	*HIP1R*	REACTOME_CELL_CYCLE
12:122853197	*HIP1R*	REACTOME_CELL_CYCLE
12:123057792	*HIP1R*	REACTOME_CELL_CYCLE
12:123110365	*HIP1R*	REACTOME_CELL_CYCLE
12:123110785	*HIP1R*	REACTOME_CELL_CYCLE
12:133206648	*FBRSL1*	REACTOME_CELL_CYCLE
12:133223685	*FBRSL1*	REACTOME_CELL_CYCLE
14:74640180	*RPS6KL1*	REACTOME_CELL_CYCLE
14:75495059	*RPS6KL1*	REACTOME_CELL_CYCLE
16:19562008	*SYT17*	REACTOME_CELL_CYCLE
17:40818584	*RETREG3*	REACTOME_CELL_CYCLE
17:45215865	*WNT3*	REACTOME_CELL_CYCLE

17:45244822	*WNT3*	REACTOME_CELL_CYCLE
17:59791813	*BRIP1*	REACTOME_CELL_CYCLE
17:59922925	*BRIP1*	REACTOME_CELL_CYCLE
17:76177567	*DNAH17*	REACTOME_CELL_CYCLE
20:5934513	*CRLS1*	REACTOME_CELL_CYCLE
20:5935522	*CRLS1*	REACTOME_CELL_CYCLE
20:5968737	*CRLS1*	REACTOME_CELL_CYCLE
20:5968964	*CRLS1*	REACTOME_CELL_CYCLE
21:38740824	*DYRK1A*	REACTOME_CELL_CYCLE
21:38753354	*DYRK1A*	REACTOME_CELL_CYCLE
21:38770530	*DYRK1A*	REACTOME_CELL_CYCLE
21:38783614	*DYRK1A*	REACTOME_CELL_CYCLE
21:38814867	*DYRK1A*	REACTOME_CELL_CYCLE
1:155975202	*GBAP1*	REACTOME_CELL_DEATH_SIGNALLING_VIA_NRAGE_NRIF_AND_NADE
1:227067656	*ITPKB*	REACTOME_CELL_DEATH_SIGNALLING_VIA_NRAGE_NRIF_AND_NADE
17:39883520	*RETREG3*	REACTOME_CELL_JUNCTION_ORGANIZATION
21:37854286	*DYRK1A*	REACTOME_CELL_JUNCTION_ORGANIZATION
21:37859449	*DYRK1A*	REACTOME_CELL_JUNCTION_ORGANIZATION
21:37860785	*DYRK1A*	REACTOME_CELL_JUNCTION_ORGANIZATION
21:37894089	*DYRK1A*	REACTOME_CELL_JUNCTION_ORGANIZATION
21:37899076	*DYRK1A*	REACTOME_CELL_JUNCTION_ORGANIZATION
6:112137847	*FYN*	REACTOME_CELL_SURFACE_INTERACTIONS_AT_THE_VASCULAR_WALL
6:112151452	*FYN*	REACTOME_CELL_SURFACE_INTERACTIONS_AT_THE_VASCULAR_WALL
6:112175069	*FYN*	REACTOME_CELL_SURFACE_INTERACTIONS_AT_THE_VASCULAR_WALL
8:23050400	*BIN3*	REACTOME_CELL_SURFACE_INTERACTIONS_AT_THE_VASCULAR_WALL
11:133947732	*IGSF9B*	REACTOME_CELL_SURFACE_INTERACTIONS_AT_THE_VASCULAR_WALL
16:30510571	*SETD1A*	REACTOME_CELL_SURFACE_INTERACTIONS_AT_THE_VASCULAR_WALL
16:30516492	*SETD1A*	REACTOME_CELL_SURFACE_INTERACTIONS_AT_THE_VASCULAR_WALL
16:30520856	*SETD1A*	REACTOME_CELL_SURFACE_INTERACTIONS_AT_THE_VASCULAR_WALL
16:31370452	*SETD1A*	REACTOME_CELL_SURFACE_INTERACTIONS_AT_THE_VASCULAR_WALL
17:7560294	*V1NB1*	REACTOME_CELL_SURFACE_INTERACTIONS_AT_THE_VASCULAR_WALL
2:102357447	*MAP4K4*	REACTOME_CELLULAR_RESPONSES_TO_EXTERNAL_STIMULI
2:102379173	*MAP4K4*	REACTOME_CELLULAR_RESPONSES_TO_EXTERNAL_STIMULI
2:102413116	*MAP4K4*	REACTOME_CELLULAR_RESPONSES_TO_EXTERNAL_STIMULI
4:114424939	*CAMK2D*	REACTOME_CELLULAR_RESPONSES_TO_EXTERNAL_STIMULI
4:114494052	*CAMK2D*	REACTOME_CELLULAR_RESPONSES_TO_EXTERNAL_STIMULI
4:114529492	*CAMK2D*	REACTOME_CELLULAR_RESPONSES_TO_EXTERNAL_STIMULI
4:114539964	*CAMK2D*	REACTOME_CELLULAR_RESPONSES_TO_EXTERNAL_STIMULI
4:114597918	*CAMK2D*	REACTOME_CELLULAR_RESPONSES_TO_EXTERNAL_STIMULI
6:31862876	*HLA-DRB5*	REACTOME_CELLULAR_RESPONSES_TO_EXTERNAL_STIMULI
9:34342881	*UBAP2*	REACTOME_CELLULAR_RESPONSES_TO_EXTERNAL_STIMULI
10:14908849	*ITGA8*	REACTOME_CELLULAR_RESPONSES_TO_EXTERNAL_STIMULI
10:104258303	*GBF1*	REACTOME_CELLULAR_RESPONSES_TO_EXTERNAL_STIMULI
10:121429633	*INPP5F*	REACTOME_CELLULAR_RESPONSES_TO_EXTERNAL_STIMULI
10:121437329	*INPP5F*	REACTOME_CELLULAR_RESPONSES_TO_EXTERNAL_STIMULI
12:133295501	*FBRSL1*	REACTOME_CELLULAR_RESPONSES_TO_EXTERNAL_STIMULI
14:55871532	*GCH1*	REACTOME_CELLULAR_RESPONSES_TO_EXTERNAL_STIMULI
17:7578671	*V1NB1*	REACTOME_CELLULAR_RESPONSES_TO_EXTERNAL_STIMULI
17:40132591	*RETREG3*	REACTOME_CELLULAR_RESPONSES_TO_EXTERNAL_STIMULI
17:45215865	*WNT3*	REACTOME_CELLULAR_RESPONSES_TO_EXTERNAL_STIMULI
17:45244822	*WNT3*	REACTOME_CELLULAR_RESPONSES_TO_EXTERNAL_STIMULI
2:102357447	*MAP4K4*	REACTOME_CELLULAR_RESPONSES_TO_STRESS
2:102379173	*MAP4K4*	REACTOME_CELLULAR_RESPONSES_TO_STRESS

2:102413116	*MAP4K4*	REACTOME_CELLULAR_RESPONSES_TO_STRESS
4:114424939	*CAMK2D*	REACTOME_CELLULAR_RESPONSES_TO_STRESS
4:114494052	*CAMK2D*	REACTOME_CELLULAR_RESPONSES_TO_STRESS
4:114529492	*CAMK2D*	REACTOME_CELLULAR_RESPONSES_TO_STRESS
4:114539964	*CAMK2D*	REACTOME_CELLULAR_RESPONSES_TO_STRESS
4:114597918	*CAMK2D*	REACTOME_CELLULAR_RESPONSES_TO_STRESS
6:31862876	*HLA-DRB5*	REACTOME_CELLULAR_RESPONSES_TO_STRESS
9:34342881	*UBAP2*	REACTOME_CELLULAR_RESPONSES_TO_STRESS
10:14908849	*ITGA8*	REACTOME_CELLULAR_RESPONSES_TO_STRESS
10:104258303	*GBF1*	REACTOME_CELLULAR_RESPONSES_TO_STRESS
10:121429633	*INPP5F*	REACTOME_CELLULAR_RESPONSES_TO_STRESS
10:121437329	*INPP5F*	REACTOME_CELLULAR_RESPONSES_TO_STRESS
17:7578671	*V1NB1*	REACTOME_CELLULAR_RESPONSES_TO_STRESS
17:40132591	*RETREG3*	REACTOME_CELLULAR_RESPONSES_TO_STRESS
17:45215865	*WNT3*	REACTOME_CELLULAR_RESPONSES_TO_STRESS
17:45244822	*WNT3*	REACTOME_CELLULAR_RESPONSES_TO_STRESS
4:114424939	*CAMK2D*	REACTOME_CELLULAR_RESPONSE_TO_HEAT_STRESS
4:114494052	*CAMK2D*	REACTOME_CELLULAR_RESPONSE_TO_HEAT_STRESS
4:114529492	*CAMK2D*	REACTOME_CELLULAR_RESPONSE_TO_HEAT_STRESS
4:114539964	*CAMK2D*	REACTOME_CELLULAR_RESPONSE_TO_HEAT_STRESS
4:114597918	*CAMK2D*	REACTOME_CELLULAR_RESPONSE_TO_HEAT_STRESS
10:14908849	*ITGA8*	REACTOME_CELLULAR_RESPONSE_TO_HEAT_STRESS
10:121429633	*INPP5F*	REACTOME_CELLULAR_RESPONSE_TO_HEAT_STRESS
10:121437329	*INPP5F*	REACTOME_CELLULAR_RESPONSE_TO_HEAT_STRESS
17:40132591	*RETREG3*	REACTOME_CELLULAR_RESPONSE_TO_HEAT_STRESS
2:102357447	*MAP4K4*	REACTOME_CELLULAR_SENESCENCE
2:102379173	*MAP4K4*	REACTOME_CELLULAR_SENESCENCE
2:102413116	*MAP4K4*	REACTOME_CELLULAR_SENESCENCE
6:31862876	*HLA-DRB5*	REACTOME_CELLULAR_SENESCENCE
17:7578671	*V1NB1*	REACTOME_CELLULAR_SENESCENCE
17:45215865	*WNT3*	REACTOME_CELLULAR_SENESCENCE
17:45244822	*WNT3*	REACTOME_CELLULAR_SENESCENCE
10:104258303	*GBF1*	REACTOME_CENTROSOME_MATURATION
16:19562008	*SYT17*	REACTOME_CENTROSOME_MATURATION
17:40818584	*RETREG3*	REACTOME_CENTROSOME_MATURATION
11:10647977	*RNF141*	REACTOME_CGMP_EFFECTS
11:10648481	*RNF141*	REACTOME_CGMP_EFFECTS
11:10660840	*RNF141*	REACTOME_CGMP_EFFECTS
17:42990894	*CRHR1_2*	REACTOME_CHAPERONE_MEDIATED_AUTOPHAGY
4:76924571	*FAM47E-STBD1*	REACTOME_CHEMOKINE_RECEPTORS_BIND_CHEMOKINES
8:11679472	*CTSB*	REACTOME_CHOLESTEROL_BIOSYNTHESIS
6:71594785	*RIMS1*	REACTOME_CHONDROITIN_SULFATE_DERMATAN_SULFATE_METABOLISM
11:134255325	*IGSF9B*	REACTOME_CHONDROITIN_SULFATE_DERMATAN_SULFATE_METABOLISM
1:155359992	*GBAP1*	REACTOME_CHROMATIN_ORGANIZATION
3:183427301	*MCCC1*	REACTOME_CHROMATIN_ORGANIZATION
5:133910742	*C5orf24*	REACTOME_CHROMATIN_ORGANIZATION
6:31862876	*HLA-DRB5*	REACTOME_CHROMATIN_ORGANIZATION
12:46134812	*SCAF11*	REACTOME_CHROMATIN_ORGANIZATION
12:46206268	*SCAF11*	REACTOME_CHROMATIN_ORGANIZATION
17:7155861	*V1NB1*	REACTOME_CHROMATIN_ORGANIZATION
17:44160410	*WNT3*	REACTOME_CHROMATIN_ORGANIZATION
17:44189067	*WNT3*	REACTOME_CHROMATIN_ORGANIZATION
19:2172266	*SPPL2B*	REACTOME_CHROMATIN_ORGANIZATION

19:2202252	*SPPL2B*	REACTOME_CHROMATIN_ORGANIZATION
12:133206648	*FBRSL1*	REACTOME_CHROMOSOME_MAINTENANCE
12:133223685	*FBRSL1*	REACTOME_CHROMOSOME_MAINTENANCE
3:121553904	*KPNA1*	REACTOME_CILIUM_ASSEMBLY
4:15492268	*BST1*	REACTOME_CILIUM_ASSEMBLY
4:15547513	*BST1*	REACTOME_CILIUM_ASSEMBLY
6:30601232	*TRIM40*	REACTOME_CILIUM_ASSEMBLY
6:30612107	*TRIM40*	REACTOME_CILIUM_ASSEMBLY
8:131145058	*FAM49B*	REACTOME_CILIUM_ASSEMBLY
8:131153067	*FAM49B*	REACTOME_CILIUM_ASSEMBLY
8:131245821	*FAM49B*	REACTOME_CILIUM_ASSEMBLY
8:131328260	*FAM49B*	REACTOME_CILIUM_ASSEMBLY
8:131404984	*FAM49B*	REACTOME_CILIUM_ASSEMBLY
8:131408202	*FAM49B*	REACTOME_CILIUM_ASSEMBLY
8:131455469	*FAM49B*	REACTOME_CILIUM_ASSEMBLY
9:34300212	*UBAP2*	REACTOME_CILIUM_ASSEMBLY
10:104005410	*GBF1*	REACTOME_CILIUM_ASSEMBLY
10:104034550	*GBF1*	REACTOME_CILIUM_ASSEMBLY
10:104258303	*GBF1*	REACTOME_CILIUM_ASSEMBLY
10:104443875	*GBF1*	REACTOME_CILIUM_ASSEMBLY
10:104452818	*GBF1*	REACTOME_CILIUM_ASSEMBLY
16:19562008	*SYT17*	REACTOME_CILIUM_ASSEMBLY
2:101439596	*MAP4K4*	REACTOME_CIRCADIAN_CLOCK
2:101464631	*MAP4K4*	REACTOME_CIRCADIAN_CLOCK
2:101547064	*MAP4K4*	REACTOME_CIRCADIAN_CLOCK
2:101550181	*MAP4K4*	REACTOME_CIRCADIAN_CLOCK
2:101578010	*MAP4K4*	REACTOME_CIRCADIAN_CLOCK
2:101603812	*MAP4K4*	REACTOME_CIRCADIAN_CLOCK
10:103185542	*GBF1*	REACTOME_CIRCADIAN_CLOCK
10:103305087	*GBF1*	REACTOME_CIRCADIAN_CLOCK
10:103308244	*GBF1*	REACTOME_CIRCADIAN_CLOCK
15:61016235	*VPS13C*	REACTOME_CIRCADIAN_CLOCK
15:61065568	*VPS13C*	REACTOME_CIRCADIAN_CLOCK
15:61094298	*VPS13C*	REACTOME_CIRCADIAN_CLOCK
15:61140736	*VPS13C*	REACTOME_CIRCADIAN_CLOCK
15:61142062	*VPS13C*	REACTOME_CIRCADIAN_CLOCK
15:61162571	*VPS13C*	REACTOME_CIRCADIAN_CLOCK
15:61191617	*VPS13C*	REACTOME_CIRCADIAN_CLOCK
15:61195427	*VPS13C*	REACTOME_CIRCADIAN_CLOCK
15:61221682	*VPS13C*	REACTOME_CIRCADIAN_CLOCK
15:61298486	*VPS13C*	REACTOME_CIRCADIAN_CLOCK
15:61375480	*VPS13C*	REACTOME_CIRCADIAN_CLOCK
15:61434581	*VPS13C*	REACTOME_CIRCADIAN_CLOCK
15:61467078	*VPS13C*	REACTOME_CIRCADIAN_CLOCK
15:61470808	*VPS13C*	REACTOME_CIRCADIAN_CLOCK
15:61500263	*VPS13C*	REACTOME_CIRCADIAN_CLOCK
3:150935203	*MED12L*	REACTOME_CLASS_A_1_RHODOPSIN_LIKE_RECEPTORS
3:151090424	*MED12L*	REACTOME_CLASS_A_1_RHODOPSIN_LIKE_RECEPTORS
3:151099733	*MED12L*	REACTOME_CLASS_A_1_RHODOPSIN_LIKE_RECEPTORS
4:76924571	*FAM47E-STBD1*	REACTOME_CLASS_A_1_RHODOPSIN_LIKE_RECEPTORS
12:123110365	*HIP1R*	REACTOME_CLASS_A_1_RHODOPSIN_LIKE_RECEPTORS
12:123110785	*HIP1R*	REACTOME_CLASS_A_1_RHODOPSIN_LIKE_RECEPTORS
12:123139394	*HIP1R*	REACTOME_CLASS_A_1_RHODOPSIN_LIKE_RECEPTORS

12:123155263	*HIP1R*	REACTOME_CLASS_A_1_RHODOPSIN_LIKE_RECEPTORS
12:123165949	*HIP1R*	REACTOME_CLASS_A_1_RHODOPSIN_LIKE_RECEPTORS
12:123194636	*HIP1R*	REACTOME_CLASS_A_1_RHODOPSIN_LIKE_RECEPTORS
14:88472612	*GALC*	REACTOME_CLASS_A_1_RHODOPSIN_LIKE_RECEPTORS
20:5290081	*CRLS1*	REACTOME_CLASS_A_1_RHODOPSIN_LIKE_RECEPTORS
17:43744203	*CRHR1_2*	REACTOME_CLASS_B_2_SECRETIN_FAMILY_RECEPTORS
17:43748712	*CRHR1_2*	REACTOME_CLASS_B_2_SECRETIN_FAMILY_RECEPTORS
17:43784228	*CRHR1_2*	REACTOME_CLASS_B_2_SECRETIN_FAMILY_RECEPTORS
17:43791502	*CRHR1_2*	REACTOME_CLASS_B_2_SECRETIN_FAMILY_RECEPTORS
17:43796210	*CRHR1_2*	REACTOME_CLASS_B_2_SECRETIN_FAMILY_RECEPTORS
17:43843861	*CRHR1_2*	REACTOME_CLASS_B_2_SECRETIN_FAMILY_RECEPTORS
17:43847503	*CRHR1_2*	REACTOME_CLASS_B_2_SECRETIN_FAMILY_RECEPTORS
17:43849514	*CRHR1_2*	REACTOME_CLASS_B_2_SECRETIN_FAMILY_RECEPTORS
17:44843651	*WNT3*	REACTOME_CLASS_B_2_SECRETIN_FAMILY_RECEPTORS
17:44870978	*WNT3*	REACTOME_CLASS_B_2_SECRETIN_FAMILY_RECEPTORS
17:44871422	*WNT3*	REACTOME_CLASS_B_2_SECRETIN_FAMILY_RECEPTORS
17:44888209	*WNT3*	REACTOME_CLASS_B_2_SECRETIN_FAMILY_RECEPTORS
17:44888715	*WNT3*	REACTOME_CLASS_B_2_SECRETIN_FAMILY_RECEPTORS
17:44906949	*WNT3*	REACTOME_CLASS_B_2_SECRETIN_FAMILY_RECEPTORS
17:44912901	*WNT3*	REACTOME_CLASS_B_2_SECRETIN_FAMILY_RECEPTORS
17:44931399	*WNT3*	REACTOME_CLASS_B_2_SECRETIN_FAMILY_RECEPTORS
17:44947756	*WNT3*	REACTOME_CLASS_B_2_SECRETIN_FAMILY_RECEPTORS
19:2557113	*SPPL2B*	REACTOME_CLASS_B_2_SECRETIN_FAMILY_RECEPTORS
19:2563327	*SPPL2B*	REACTOME_CLASS_B_2_SECRETIN_FAMILY_RECEPTORS
19:2580057	*SPPL2B*	REACTOME_CLASS_B_2_SECRETIN_FAMILY_RECEPTORS
19:2660983	*SPPL2B*	REACTOME_CLASS_B_2_SECRETIN_FAMILY_RECEPTORS
6:29565889	*TRIM40*	REACTOME_CLASS_C_3_METABOTROPIC_GLUTAMATE_PHEROMONE_RECEPTORS
6:29590715	*TRIM40*	REACTOME_CLASS_C_3_METABOTROPIC_GLUTAMATE_PHEROMONE_RECEPTORS
3:49740895	*IP6K2*	REACTOME_CLASS_I_MHC_MEDIATED_ANTIGEN_PROCESSING_PRESENTATION
3:121319489	*KPNA1*	REACTOME_CLASS_I_MHC_MEDIATED_ANTIGEN_PROCESSING_PRESENTATION
3:121324643	*KPNA1*	REACTOME_CLASS_I_MHC_MEDIATED_ANTIGEN_PROCESSING_PRESENTATION
3:121340955	*KPNA1*	REACTOME_CLASS_I_MHC_MEDIATED_ANTIGEN_PROCESSING_PRESENTATION
3:122289921	*KPNA1*	REACTOME_CLASS_I_MHC_MEDIATED_ANTIGEN_PROCESSING_PRESENTATION
4:15608677	*BST1*	REACTOME_CLASS_I_MHC_MEDIATED_ANTIGEN_PROCESSING_PRESENTATION
4:15618898	*BST1*	REACTOME_CLASS_I_MHC_MEDIATED_ANTIGEN_PROCESSING_PRESENTATION
4:76407998	*FAM47E-STBD1*	REACTOME_CLASS_I_MHC_MEDIATED_ANTIGEN_PROCESSING_PRESENTATION
4:170015977	*CLCN3*	REACTOME_CLASS_I_MHC_MEDIATED_ANTIGEN_PROCESSING_PRESENTATION
4:170031333	*CLCN3*	REACTOME_CLASS_I_MHC_MEDIATED_ANTIGEN_PROCESSING_PRESENTATION
4:170101917	*CLCN3*	REACTOME_CLASS_I_MHC_MEDIATED_ANTIGEN_PROCESSING_PRESENTATION
4:170161031	*CLCN3*	REACTOME_CLASS_I_MHC_MEDIATED_ANTIGEN_PROCESSING_PRESENTATION
5:133710339	*C5orf24*	REACTOME_CLASS_I_MHC_MEDIATED_ANTIGEN_PROCESSING_PRESENTATION
6:29798642	*TRIM40*	REACTOME_CLASS_I_MHC_MEDIATED_ANTIGEN_PROCESSING_PRESENTATION
6:32789400	*HLA-DRB5*	REACTOME_CLASS_I_MHC_MEDIATED_ANTIGEN_PROCESSING_PRESENTATION
6:32791831	*HLA-DRB5*	REACTOME_CLASS_I_MHC_MEDIATED_ANTIGEN_PROCESSING_PRESENTATION
6:32797361	*HLA-DRB5*	REACTOME_CLASS_I_MHC_MEDIATED_ANTIGEN_PROCESSING_PRESENTATION
6:32802462	*HLA-DRB5*	REACTOME_CLASS_I_MHC_MEDIATED_ANTIGEN_PROCESSING_PRESENTATION
7:66096028	*GS1-124K5.11*	REACTOME_CLASS_I_MHC_MEDIATED_ANTIGEN_PROCESSING_PRESENTATION
7:66136281	*GS1-124K5.11*	REACTOME_CLASS_I_MHC_MEDIATED_ANTIGEN_PROCESSING_PRESENTATION
9:33845203	*UBAP2*	REACTOME_CLASS_I_MHC_MEDIATED_ANTIGEN_PROCESSING_PRESENTATION
10:103185542	*GBF1*	REACTOME_CLASS_I_MHC_MEDIATED_ANTIGEN_PROCESSING_PRESENTATION
10:103305087	*GBF1*	REACTOME_CLASS_I_MHC_MEDIATED_ANTIGEN_PROCESSING_PRESENTATION
10:103308244	*GBF1*	REACTOME_CLASS_I_MHC_MEDIATED_ANTIGEN_PROCESSING_PRESENTATION
17:45215865	*WNT3*	REACTOME_CLASS_I_MHC_MEDIATED_ANTIGEN_PROCESSING_PRESENTATION

17:45244822	*WNT3*	REACTOME_CLASS_I_MHC_MEDIATED_ANTIGEN_PROCESSING_PRESENTATION
12:39960237	*LRRK2_2*	REACTOME_CLASS_I_PEROXISOMAL_MEMBRANE_PROTEIN_IMPORT
12:133295501	*FBRSL1*	REACTOME_CLASS_I_PEROXISOMAL_MEMBRANE_PROTEIN_IMPORT
1:155847427	*GBAP1*	REACTOME_CLATHRIN_MEDIATED_ENDOCYTOSIS
1:171689486	*VAMP4*	REACTOME_CLATHRIN_MEDIATED_ENDOCYTOSIS
1:171837985	*VAMP4*	REACTOME_CLATHRIN_MEDIATED_ENDOCYTOSIS
1:171945488	*VAMP4*	REACTOME_CLATHRIN_MEDIATED_ENDOCYTOSIS
1:172137119	*VAMP4*	REACTOME_CLATHRIN_MEDIATED_ENDOCYTOSIS
1:172386261	*VAMP4*	REACTOME_CLATHRIN_MEDIATED_ENDOCYTOSIS
2:170012307	*STK39*	REACTOME_CLATHRIN_MEDIATED_ENDOCYTOSIS
2:170028024	*STK39*	REACTOME_CLATHRIN_MEDIATED_ENDOCYTOSIS
2:170066424	*STK39*	REACTOME_CLATHRIN_MEDIATED_ENDOCYTOSIS
4:925376	*GAK*	REACTOME_CLATHRIN_MEDIATED_ENDOCYTOSIS
4:77104152	*FAM47E-STBD1*	REACTOME_CLATHRIN_MEDIATED_ENDOCYTOSIS
4:77124196	*FAM47E-STBD1*	REACTOME_CLATHRIN_MEDIATED_ENDOCYTOSIS
4:77134786	*FAM47E-STBD1*	REACTOME_CLATHRIN_MEDIATED_ENDOCYTOSIS
9:17579690	*SH3GL2_2*	REACTOME_CLATHRIN_MEDIATED_ENDOCYTOSIS
9:17587160	*SH3GL2_2*	REACTOME_CLATHRIN_MEDIATED_ENDOCYTOSIS
9:17609302	*SH3GL2_2*	REACTOME_CLATHRIN_MEDIATED_ENDOCYTOSIS
9:17626762	*SH3GL2_2*	REACTOME_CLATHRIN_MEDIATED_ENDOCYTOSIS
9:17627444	*SH3GL2_2*	REACTOME_CLATHRIN_MEDIATED_ENDOCYTOSIS
9:17642664	*SH3GL2_2*	REACTOME_CLATHRIN_MEDIATED_ENDOCYTOSIS
9:17710876	*SH3GL2_2*	REACTOME_CLATHRIN_MEDIATED_ENDOCYTOSIS
9:17732590	*SH3GL2_2*	REACTOME_CLATHRIN_MEDIATED_ENDOCYTOSIS
9:17737514	*SH3GL2_2*	REACTOME_CLATHRIN_MEDIATED_ENDOCYTOSIS
9:17758945	*SH3GL2_2*	REACTOME_CLATHRIN_MEDIATED_ENDOCYTOSIS
12:123326598	*HIP1R*	REACTOME_CLATHRIN_MEDIATED_ENDOCYTOSIS
17:7129974	*V1NB1*	REACTOME_CLATHRIN_MEDIATED_ENDOCYTOSIS
10:103185542	*GBF1*	REACTOME_CLEC7A_DECTIN_1_SIGNALING
10:103305087	*GBF1*	REACTOME_CLEC7A_DECTIN_1_SIGNALING
10:103308244	*GBF1*	REACTOME_CLEC7A_DECTIN_1_SIGNALING
17:43390763	*CRHR1_2*	REACTOME_CLEC7A_DECTIN_1_SIGNALING
9:33791164	*UBAP2*	REACTOME_COBALAMIN_CBL_VITAMIN_B12_TRANSPORT_AND_METABOLISM
14:74764925	*RPS6KL1*	REACTOME_COBALAMIN_CBL_VITAMIN_B12_TRANSPORT_AND_METABOLISM
6:33153528	*HLA-DRB5*	REACTOME_COLLAGEN_BIOSYNTHESIS_AND_MODIFYING_ENZYMES
6:33153528	*HLA-DRB5*	REACTOME_COLLAGEN_CHAIN_TRIMERIZATION
6:33153528	*HLA-DRB5*	REACTOME_COLLAGEN_DEGRADATION
8:11712443	*CTSB*	REACTOME_COLLAGEN_DEGRADATION
6:33153528	*HLA-DRB5*	REACTOME_COLLAGEN_FORMATION
8:11712443	*CTSB*	REACTOME_COLLAGEN_FORMATION
8:23190941	*BIN3*	REACTOME_COLLAGEN_FORMATION
8:23212542	*BIN3*	REACTOME_COLLAGEN_FORMATION
8:23216225	*BIN3*	REACTOME_COLLAGEN_FORMATION
8:23223713	*BIN3*	REACTOME_COLLAGEN_FORMATION
6:31895092	*HLA-DRB5*	REACTOME_COMPLEMENT_CASCADE
6:31897219	*HLA-DRB5*	REACTOME_COMPLEMENT_CASCADE
1:161171736	*FCGR2A*	REACTOME_COMPLEX_I_BIOGENESIS
5:60273923	*ELOVL7*	REACTOME_COMPLEX_I_BIOGENESIS
5:60288288	*ELOVL7*	REACTOME_COMPLEX_I_BIOGENESIS
6:112137847	*FYN*	REACTOME_CONSTITUTIVE_SIGNALING_BY_ABERRANT_PI3K_IN_CANCER
6:112151452	*FYN*	REACTOME_CONSTITUTIVE_SIGNALING_BY_ABERRANT_PI3K_IN_CANCER
6:112175069	*FYN*	REACTOME_CONSTITUTIVE_SIGNALING_BY_ABERRANT_PI3K_IN_CANCER
17:45215865	*WNT3*	REACTOME_CONVERSION_FROM_APC_C:CDC20_TO_APC_C:CDH1_IN_LATE_ANAPHASE

17:45244822	*WNT3*	REACTOME_CONVERSION_FROM_APC_C:CDC20_TO_APC_C:CDH1_IN_LATE_ANAPHASE
19:2557113	*SPPL2B*	REACTOME_COOPERATION_OF_PDCL_PHLP1_AND_TRIC_CCT_IN_G_PROTEIN_BETA_FOLDING
19:2563327	*SPPL2B*	REACTOME_COOPERATION_OF_PDCL_PHLP1_AND_TRIC_CCT_IN_G_PROTEIN_BETA_FOLDING
19:2580057	*SPPL2B*	REACTOME_COOPERATION_OF_PDCL_PHLP1_AND_TRIC_CCT_IN_G_PROTEIN_BETA_FOLDING
19:2660983	*SPPL2B*	REACTOME_COOPERATION_OF_PDCL_PHLP1_AND_TRIC_CCT_IN_G_PROTEIN_BETA_FOLDING
10:104005410	*GBF1*	REACTOME_COPI_DEPENDENT_GOLGI_TO_ER_RETROGRADE_TRAFFIC
10:104034550	*GBF1*	REACTOME_COPI_DEPENDENT_GOLGI_TO_ER_RETROGRADE_TRAFFIC
12:39759090	*LRRK2_2*	REACTOME_COPI_DEPENDENT_GOLGI_TO_ER_RETROGRADE_TRAFFIC
12:39826709	*LRRK2_2*	REACTOME_COPI_DEPENDENT_GOLGI_TO_ER_RETROGRADE_TRAFFIC
17:43017194	*CRHR1_2*	REACTOME_COPI_DEPENDENT_GOLGI_TO_ER_RETROGRADE_TRAFFIC
17:43021962	*CRHR1_2*	REACTOME_COPI_DEPENDENT_GOLGI_TO_ER_RETROGRADE_TRAFFIC
17:44800946	*WNT3*	REACTOME_COPI_DEPENDENT_GOLGI_TO_ER_RETROGRADE_TRAFFIC
17:44833217	*WNT3*	REACTOME_COPI_DEPENDENT_GOLGI_TO_ER_RETROGRADE_TRAFFIC
3:122986429	*KPNA1*	REACTOME_COPII_MEDIATED_VESICLE_TRANSPORT
4:76695927	*FAM47E-STBD1*	REACTOME_COPII_MEDIATED_VESICLE_TRANSPORT
10:121669571	*INPP5F*	REACTOME_COPII_MEDIATED_VESICLE_TRANSPORT
10:121683206	*INPP5F*	REACTOME_COPII_MEDIATED_VESICLE_TRANSPORT
10:121686714	*INPP5F*	REACTOME_COPII_MEDIATED_VESICLE_TRANSPORT
10:121689030	*INPP5F*	REACTOME_COPII_MEDIATED_VESICLE_TRANSPORT
17:44800946	*WNT3*	REACTOME_COPII_MEDIATED_VESICLE_TRANSPORT
17:44833217	*WNT3*	REACTOME_COPII_MEDIATED_VESICLE_TRANSPORT
17:45020426	*WNT3*	REACTOME_COPII_MEDIATED_VESICLE_TRANSPORT
17:45059032	*WNT3*	REACTOME_COPII_MEDIATED_VESICLE_TRANSPORT
17:45089680	*WNT3*	REACTOME_COPII_MEDIATED_VESICLE_TRANSPORT
2:135921205	*TMEM163*	REACTOME_COPI_INDEPENDENT_GOLGI_TO_ER_RETROGRADE_TRAFFIC
10:104258303	*GBF1*	REACTOME_COPI_INDEPENDENT_GOLGI_TO_ER_RETROGRADE_TRAFFIC
3:121411876	*KPNA1*	REACTOME_COPI_MEDIATED_ANTEROGRADE_TRANSPORT
3:121412702	*KPNA1*	REACTOME_COPI_MEDIATED_ANTEROGRADE_TRANSPORT
4:76695927	*FAM47E-STBD1*	REACTOME_COPI_MEDIATED_ANTEROGRADE_TRANSPORT
4:113947327	*CAMK2D*	REACTOME_COPI_MEDIATED_ANTEROGRADE_TRANSPORT
4:114015252	*CAMK2D*	REACTOME_COPI_MEDIATED_ANTEROGRADE_TRANSPORT
4:114032570	*CAMK2D*	REACTOME_COPI_MEDIATED_ANTEROGRADE_TRANSPORT
4:114067233	*CAMK2D*	REACTOME_COPI_MEDIATED_ANTEROGRADE_TRANSPORT
4:114077386	*CAMK2D*	REACTOME_COPI_MEDIATED_ANTEROGRADE_TRANSPORT
4:114113772	*CAMK2D*	REACTOME_COPI_MEDIATED_ANTEROGRADE_TRANSPORT
4:114122398	*CAMK2D*	REACTOME_COPI_MEDIATED_ANTEROGRADE_TRANSPORT
4:114172749	*CAMK2D*	REACTOME_COPI_MEDIATED_ANTEROGRADE_TRANSPORT
4:114217997	*CAMK2D*	REACTOME_COPI_MEDIATED_ANTEROGRADE_TRANSPORT
10:104005410	*GBF1*	REACTOME_COPI_MEDIATED_ANTEROGRADE_TRANSPORT
10:104034550	*GBF1*	REACTOME_COPI_MEDIATED_ANTEROGRADE_TRANSPORT
10:104258303	*GBF1*	REACTOME_COPI_MEDIATED_ANTEROGRADE_TRANSPORT
17:44800946	*WNT3*	REACTOME_COPI_MEDIATED_ANTEROGRADE_TRANSPORT
17:44833217	*WNT3*	REACTOME_COPI_MEDIATED_ANTEROGRADE_TRANSPORT
17:45020426	*WNT3*	REACTOME_COPI_MEDIATED_ANTEROGRADE_TRANSPORT
17:45059032	*WNT3*	REACTOME_COPI_MEDIATED_ANTEROGRADE_TRANSPORT
17:45089680	*WNT3*	REACTOME_COPI_MEDIATED_ANTEROGRADE_TRANSPORT
5:133558885	*C5orf24*	REACTOME_COSTIMULATION_BY_THE_CD28_FAMILY
6:32602372	*HLA-DRB5*	REACTOME_COSTIMULATION_BY_THE_CD28_FAMILY
6:112137847	*FYN*	REACTOME_COSTIMULATION_BY_THE_CD28_FAMILY
6:112151452	*FYN*	REACTOME_COSTIMULATION_BY_THE_CD28_FAMILY
6:112175069	*FYN*	REACTOME_COSTIMULATION_BY_THE_CD28_FAMILY
17:43390763	*CRHR1_2*	REACTOME_COSTIMULATION_BY_THE_CD28_FAMILY
4:114424939	*CAMK2D*	REACTOME_CREB1_PHOSPHORYLATION_THROUGH_NMDA_RECEPTOR_MEDIATED_ACTIVATION_OF_RAS_SIGNALING

4:114494052	*CAMK2D*	REACTOME_CREB1_PHOSPHORYLATION_THROUGH_NMDA_RECEPTOR_MEDIATED_ACTIVATION_OF_RAS_SIGNALING
4:114529492	*CAMK2D*	REACTOME_CREB1_PHOSPHORYLATION_THROUGH_NMDA_RECEPTOR_MEDIATED_ACTIVATION_OF_RAS_SIGNALING
4:114539964	*CAMK2D*	REACTOME_CREB1_PHOSPHORYLATION_THROUGH_NMDA_RECEPTOR_MEDIATED_ACTIVATION_OF_RAS_SIGNALING
4:114597918	*CAMK2D*	REACTOME_CREB1_PHOSPHORYLATION_THROUGH_NMDA_RECEPTOR_MEDIATED_ACTIVATION_OF_RAS_SIGNALING
11:83219006	*DLG2*	REACTOME_CREB1_PHOSPHORYLATION_THROUGH_NMDA_RECEPTOR_MEDIATED_ACTIVATION_OF_RAS_SIGNALING
11:83232915	*DLG2*	REACTOME_CREB1_PHOSPHORYLATION_THROUGH_NMDA_RECEPTOR_MEDIATED_ACTIVATION_OF_RAS_SIGNALING
11:83282740	*DLG2*	REACTOME_CREB1_PHOSPHORYLATION_THROUGH_NMDA_RECEPTOR_MEDIATED_ACTIVATION_OF_RAS_SIGNALING
11:83387273	*DLG2*	REACTOME_CREB1_PHOSPHORYLATION_THROUGH_NMDA_RECEPTOR_MEDIATED_ACTIVATION_OF_RAS_SIGNALING
11:83538733	*DLG2*	REACTOME_CREB1_PHOSPHORYLATION_THROUGH_NMDA_RECEPTOR_MEDIATED_ACTIVATION_OF_RAS_SIGNALING
11:83544472	*DLG2*	REACTOME_CREB1_PHOSPHORYLATION_THROUGH_NMDA_RECEPTOR_MEDIATED_ACTIVATION_OF_RAS_SIGNALING
11:83578152	*DLG2*	REACTOME_CREB1_PHOSPHORYLATION_THROUGH_NMDA_RECEPTOR_MEDIATED_ACTIVATION_OF_RAS_SIGNALING
11:83647657	*DLG2*	REACTOME_CREB1_PHOSPHORYLATION_THROUGH_NMDA_RECEPTOR_MEDIATED_ACTIVATION_OF_RAS_SIGNALING
11:83669903	*DLG2*	REACTOME_CREB1_PHOSPHORYLATION_THROUGH_NMDA_RECEPTOR_MEDIATED_ACTIVATION_OF_RAS_SIGNALING
11:83736246	*DLG2*	REACTOME_CREB1_PHOSPHORYLATION_THROUGH_NMDA_RECEPTOR_MEDIATED_ACTIVATION_OF_RAS_SIGNALING
11:83762918	*DLG2*	REACTOME_CREB1_PHOSPHORYLATION_THROUGH_NMDA_RECEPTOR_MEDIATED_ACTIVATION_OF_RAS_SIGNALING
11:83823752	*DLG2*	REACTOME_CREB1_PHOSPHORYLATION_THROUGH_NMDA_RECEPTOR_MEDIATED_ACTIVATION_OF_RAS_SIGNALING
11:83839992	*DLG2*	REACTOME_CREB1_PHOSPHORYLATION_THROUGH_NMDA_RECEPTOR_MEDIATED_ACTIVATION_OF_RAS_SIGNALING
11:84104763	*DLG2*	REACTOME_CREB1_PHOSPHORYLATION_THROUGH_NMDA_RECEPTOR_MEDIATED_ACTIVATION_OF_RAS_SIGNALING
11:84169039	*DLG2*	REACTOME_CREB1_PHOSPHORYLATION_THROUGH_NMDA_RECEPTOR_MEDIATED_ACTIVATION_OF_RAS_SIGNALING
11:84221628	*DLG2*	REACTOME_CREB1_PHOSPHORYLATION_THROUGH_NMDA_RECEPTOR_MEDIATED_ACTIVATION_OF_RAS_SIGNALING
11:84312008	*DLG2*	REACTOME_CREB1_PHOSPHORYLATION_THROUGH_NMDA_RECEPTOR_MEDIATED_ACTIVATION_OF_RAS_SIGNALING
11:84324361	*DLG2*	REACTOME_CREB1_PHOSPHORYLATION_THROUGH_NMDA_RECEPTOR_MEDIATED_ACTIVATION_OF_RAS_SIGNALING
11:84360346	*DLG2*	REACTOME_CREB1_PHOSPHORYLATION_THROUGH_NMDA_RECEPTOR_MEDIATED_ACTIVATION_OF_RAS_SIGNALING
11:84403409	*DLG2*	REACTOME_CREB1_PHOSPHORYLATION_THROUGH_NMDA_RECEPTOR_MEDIATED_ACTIVATION_OF_RAS_SIGNALING
17:7101771	*V1NB1*	REACTOME_CREB1_PHOSPHORYLATION_THROUGH_NMDA_RECEPTOR_MEDIATED_ACTIVATION_OF_RAS_SIGNALING
3:48835245	*IP6K2*	REACTOME_CREB1_PHOSPHORYLATION_THROUGH_THE_ACTIVATION_OF_ADENYLATE_CYCLASE
8:131799767	*FAM49B*	REACTOME_CREB1_PHOSPHORYLATION_THROUGH_THE_ACTIVATION_OF_ADENYLATE_CYCLASE
8:131845512	*FAM49B*	REACTOME_CREB1_PHOSPHORYLATION_THROUGH_THE_ACTIVATION_OF_ADENYLATE_CYCLASE
8:131865425	*FAM49B*	REACTOME_CREB1_PHOSPHORYLATION_THROUGH_THE_ACTIVATION_OF_ADENYLATE_CYCLASE
8:131901170	*FAM49B*	REACTOME_CREB1_PHOSPHORYLATION_THROUGH_THE_ACTIVATION_OF_ADENYLATE_CYCLASE
1:155181232	*GBAP1*	REACTOME_CRISTAE_FORMATION
6:112137847	*FYN*	REACTOME_CRMPS_IN_SEMA3A_SIGNALING
6:112151452	*FYN*	REACTOME_CRMPS_IN_SEMA3A_SIGNALING
6:112175069	*FYN*	REACTOME_CRMPS_IN_SEMA3A_SIGNALING
8:23190941	*BIN3*	REACTOME_CROSSLINKING_OF_COLLAGEN_FIBRILS
8:23212542	*BIN3*	REACTOME_CROSSLINKING_OF_COLLAGEN_FIBRILS
8:23216225	*BIN3*	REACTOME_CROSSLINKING_OF_COLLAGEN_FIBRILS
8:23223713	*BIN3*	REACTOME_CROSSLINKING_OF_COLLAGEN_FIBRILS
5:133558885	*C5orf24*	REACTOME_CTLA4_INHIBITORY_SIGNALING
6:112137847	*FYN*	REACTOME_CTLA4_INHIBITORY_SIGNALING
6:112151452	*FYN*	REACTOME_CTLA4_INHIBITORY_SIGNALING
6:112175069	*FYN*	REACTOME_CTLA4_INHIBITORY_SIGNALING
6:112137847	*FYN*	REACTOME_C_TYPE_LECTIN_RECEPTORS_CLRS
6:112151452	*FYN*	REACTOME_C_TYPE_LECTIN_RECEPTORS_CLRS
6:112175069	*FYN*	REACTOME_C_TYPE_LECTIN_RECEPTORS_CLRS
10:103185542	*GBF1*	REACTOME_C_TYPE_LECTIN_RECEPTORS_CLRS
10:103305087	*GBF1*	REACTOME_C_TYPE_LECTIN_RECEPTORS_CLRS
10:103308244	*GBF1*	REACTOME_C_TYPE_LECTIN_RECEPTORS_CLRS
17:43390763	*CRHR1_2*	REACTOME_C_TYPE_LECTIN_RECEPTORS_CLRS
3:48199695	*IP6K2*	REACTOME_CYCLIN_A_B1_B2_ASSOCIATED_EVENTS_DURING_G2_M_TRANSITION
3:48214438	*IP6K2*	REACTOME_CYCLIN_A_B1_B2_ASSOCIATED_EVENTS_DURING_G2_M_TRANSITION
5:133558885	*C5orf24*	REACTOME_CYCLIN_A_B1_B2_ASSOCIATED_EVENTS_DURING_G2_M_TRANSITION
1:226497419	*ITPKB*	REACTOME_CYCLIN_A:CDK2_ASSOCIATED_EVENTS_AT_S_PHASE_ENTRY

3:48199695	*IP6K2*	REACTOME_CYCLIN_A:CDK2_ASSOCIATED_EVENTS_AT_S_PHASE_ENTRY
3:48214438	*IP6K2*	REACTOME_CYCLIN_A:CDK2_ASSOCIATED_EVENTS_AT_S_PHASE_ENTRY
14:74640180	*RPS6KL1*	REACTOME_CYCLIN_A:CDK2_ASSOCIATED_EVENTS_AT_S_PHASE_ENTRY
2:102612026	*MAP4K4*	REACTOME_CYTOKINE_SIGNALING_IN_IMMUNE_SYSTEM
2:102621855	*MAP4K4*	REACTOME_CYTOKINE_SIGNALING_IN_IMMUNE_SYSTEM
2:102631503	*MAP4K4*	REACTOME_CYTOKINE_SIGNALING_IN_IMMUNE_SYSTEM
2:102633387	*MAP4K4*	REACTOME_CYTOKINE_SIGNALING_IN_IMMUNE_SYSTEM
2:102720323	*MAP4K4*	REACTOME_CYTOKINE_SIGNALING_IN_IMMUNE_SYSTEM
2:102783052	*MAP4K4*	REACTOME_CYTOKINE_SIGNALING_IN_IMMUNE_SYSTEM
2:102826146	*MAP4K4*	REACTOME_CYTOKINE_SIGNALING_IN_IMMUNE_SYSTEM
2:103047986	*MAP4K4*	REACTOME_CYTOKINE_SIGNALING_IN_IMMUNE_SYSTEM
2:103063855	*MAP4K4*	REACTOME_CYTOKINE_SIGNALING_IN_IMMUNE_SYSTEM
2:103066346	*MAP4K4*	REACTOME_CYTOKINE_SIGNALING_IN_IMMUNE_SYSTEM
3:48748989	*IP6K2*	REACTOME_CYTOKINE_SIGNALING_IN_IMMUNE_SYSTEM
3:48766111	*IP6K2*	REACTOME_CYTOKINE_SIGNALING_IN_IMMUNE_SYSTEM
3:122200170	*KPNA1*	REACTOME_CYTOKINE_SIGNALING_IN_IMMUNE_SYSTEM
4:114424939	*CAMK2D*	REACTOME_CYTOKINE_SIGNALING_IN_IMMUNE_SYSTEM
4:114494052	*CAMK2D*	REACTOME_CYTOKINE_SIGNALING_IN_IMMUNE_SYSTEM
4:114529492	*CAMK2D*	REACTOME_CYTOKINE_SIGNALING_IN_IMMUNE_SYSTEM
4:114539964	*CAMK2D*	REACTOME_CYTOKINE_SIGNALING_IN_IMMUNE_SYSTEM
4:114597918	*CAMK2D*	REACTOME_CYTOKINE_SIGNALING_IN_IMMUNE_SYSTEM
5:133558885	*C5orf24*	REACTOME_CYTOKINE_SIGNALING_IN_IMMUNE_SYSTEM
6:29798642	*TRIM40*	REACTOME_CYTOKINE_SIGNALING_IN_IMMUNE_SYSTEM
6:30075864	*TRIM40*	REACTOME_CYTOKINE_SIGNALING_IN_IMMUNE_SYSTEM
6:32151443	*HLA-DRB5*	REACTOME_CYTOKINE_SIGNALING_IN_IMMUNE_SYSTEM
6:32602372	*HLA-DRB5*	REACTOME_CYTOKINE_SIGNALING_IN_IMMUNE_SYSTEM
6:112137847	*FYN*	REACTOME_CYTOKINE_SIGNALING_IN_IMMUNE_SYSTEM
6:112151452	*FYN*	REACTOME_CYTOKINE_SIGNALING_IN_IMMUNE_SYSTEM
6:112175069	*FYN*	REACTOME_CYTOKINE_SIGNALING_IN_IMMUNE_SYSTEM
8:21565088	*BIN3*	REACTOME_CYTOKINE_SIGNALING_IN_IMMUNE_SYSTEM
8:21582921	*BIN3*	REACTOME_CYTOKINE_SIGNALING_IN_IMMUNE_SYSTEM
8:21665306	*BIN3*	REACTOME_CYTOKINE_SIGNALING_IN_IMMUNE_SYSTEM
8:21668901	*BIN3*	REACTOME_CYTOKINE_SIGNALING_IN_IMMUNE_SYSTEM
10:103185542	*GBF1*	REACTOME_CYTOKINE_SIGNALING_IN_IMMUNE_SYSTEM
10:103305087	*GBF1*	REACTOME_CYTOKINE_SIGNALING_IN_IMMUNE_SYSTEM
10:103308244	*GBF1*	REACTOME_CYTOKINE_SIGNALING_IN_IMMUNE_SYSTEM
11:83219006	*DLG2*	REACTOME_CYTOKINE_SIGNALING_IN_IMMUNE_SYSTEM
11:83232915	*DLG2*	REACTOME_CYTOKINE_SIGNALING_IN_IMMUNE_SYSTEM
11:83282740	*DLG2*	REACTOME_CYTOKINE_SIGNALING_IN_IMMUNE_SYSTEM
11:83387273	*DLG2*	REACTOME_CYTOKINE_SIGNALING_IN_IMMUNE_SYSTEM
11:83538733	*DLG2*	REACTOME_CYTOKINE_SIGNALING_IN_IMMUNE_SYSTEM
11:83544472	*DLG2*	REACTOME_CYTOKINE_SIGNALING_IN_IMMUNE_SYSTEM
11:83578152	*DLG2*	REACTOME_CYTOKINE_SIGNALING_IN_IMMUNE_SYSTEM
11:83647657	*DLG2*	REACTOME_CYTOKINE_SIGNALING_IN_IMMUNE_SYSTEM
11:83669903	*DLG2*	REACTOME_CYTOKINE_SIGNALING_IN_IMMUNE_SYSTEM
11:83736246	*DLG2*	REACTOME_CYTOKINE_SIGNALING_IN_IMMUNE_SYSTEM
11:83762918	*DLG2*	REACTOME_CYTOKINE_SIGNALING_IN_IMMUNE_SYSTEM
11:83823752	*DLG2*	REACTOME_CYTOKINE_SIGNALING_IN_IMMUNE_SYSTEM
11:83839992	*DLG2*	REACTOME_CYTOKINE_SIGNALING_IN_IMMUNE_SYSTEM
11:84104763	*DLG2*	REACTOME_CYTOKINE_SIGNALING_IN_IMMUNE_SYSTEM
11:84169039	*DLG2*	REACTOME_CYTOKINE_SIGNALING_IN_IMMUNE_SYSTEM
11:84221628	*DLG2*	REACTOME_CYTOKINE_SIGNALING_IN_IMMUNE_SYSTEM
11:84312008	*DLG2*	REACTOME_CYTOKINE_SIGNALING_IN_IMMUNE_SYSTEM

11:84324361	*DLG2*	REACTOME_CYTOKINE_SIGNALING_IN_IMMUNE_SYSTEM
11:84360346	*DLG2*	REACTOME_CYTOKINE_SIGNALING_IN_IMMUNE_SYSTEM
11:84403409	*DLG2*	REACTOME_CYTOKINE_SIGNALING_IN_IMMUNE_SYSTEM
15:61016235	*VPS13C*	REACTOME_CYTOKINE_SIGNALING_IN_IMMUNE_SYSTEM
15:61065568	*VPS13C*	REACTOME_CYTOKINE_SIGNALING_IN_IMMUNE_SYSTEM
15:61094298	*VPS13C*	REACTOME_CYTOKINE_SIGNALING_IN_IMMUNE_SYSTEM
15:61140736	*VPS13C*	REACTOME_CYTOKINE_SIGNALING_IN_IMMUNE_SYSTEM
15:61142062	*VPS13C*	REACTOME_CYTOKINE_SIGNALING_IN_IMMUNE_SYSTEM
15:61162571	*VPS13C*	REACTOME_CYTOKINE_SIGNALING_IN_IMMUNE_SYSTEM
15:61191617	*VPS13C*	REACTOME_CYTOKINE_SIGNALING_IN_IMMUNE_SYSTEM
15:61195427	*VPS13C*	REACTOME_CYTOKINE_SIGNALING_IN_IMMUNE_SYSTEM
15:61221682	*VPS13C*	REACTOME_CYTOKINE_SIGNALING_IN_IMMUNE_SYSTEM
15:61298486	*VPS13C*	REACTOME_CYTOKINE_SIGNALING_IN_IMMUNE_SYSTEM
15:61375480	*VPS13C*	REACTOME_CYTOKINE_SIGNALING_IN_IMMUNE_SYSTEM
15:61434581	*VPS13C*	REACTOME_CYTOKINE_SIGNALING_IN_IMMUNE_SYSTEM
15:61467078	*VPS13C*	REACTOME_CYTOKINE_SIGNALING_IN_IMMUNE_SYSTEM
15:61470808	*VPS13C*	REACTOME_CYTOKINE_SIGNALING_IN_IMMUNE_SYSTEM
15:61500263	*VPS13C*	REACTOME_CYTOKINE_SIGNALING_IN_IMMUNE_SYSTEM
16:31370452	*SETD1A*	REACTOME_CYTOKINE_SIGNALING_IN_IMMUNE_SYSTEM
16:50730088	*CASC16*	REACTOME_CYTOKINE_SIGNALING_IN_IMMUNE_SYSTEM
16:50730446	*CASC16*	REACTOME_CYTOKINE_SIGNALING_IN_IMMUNE_SYSTEM
16:50745926	*CASC16*	REACTOME_CYTOKINE_SIGNALING_IN_IMMUNE_SYSTEM
17:6667554	*V1NB1*	REACTOME_CYTOKINE_SIGNALING_IN_IMMUNE_SYSTEM
17:7101771	*V1NB1*	REACTOME_CYTOKINE_SIGNALING_IN_IMMUNE_SYSTEM
17:7479140	*V1NB1*	REACTOME_CYTOKINE_SIGNALING_IN_IMMUNE_SYSTEM
17:7480325	*V1NB1*	REACTOME_CYTOKINE_SIGNALING_IN_IMMUNE_SYSTEM
17:7578671	*V1NB1*	REACTOME_CYTOKINE_SIGNALING_IN_IMMUNE_SYSTEM
17:40392358	*RETREG3*	REACTOME_CYTOKINE_SIGNALING_IN_IMMUNE_SYSTEM
17:41845031	*FAM171A2*	REACTOME_CYTOKINE_SIGNALING_IN_IMMUNE_SYSTEM
17:42454243	*FAM171A2*	REACTOME_CYTOKINE_SIGNALING_IN_IMMUNE_SYSTEM
17:43390763	*CRHR1_2*	REACTOME_CYTOKINE_SIGNALING_IN_IMMUNE_SYSTEM
17:59791813	*BRIP1*	REACTOME_CYTOSOLIC_IRON_SULFUR_CLUSTER_ASSEMBLY
17:59922925	*BRIP1*	REACTOME_CYTOSOLIC_IRON_SULFUR_CLUSTER_ASSEMBLY
8:22108784	*BIN3*	REACTOME_CYTOSOLIC_SENSORS_OF_PATHOGEN_ASSOCIATED_DNA
7:65705034	*GS1-124K5.11*	REACTOME_CYTOSOLIC_SULFONATION_OF_SMALL_MOLECULES
7:65824287	*GS1-124K5.11*	REACTOME_CYTOSOLIC_SULFONATION_OF_SMALL_MOLECULES
6:31758240	*HLA-DRB5*	REACTOME_CYTOSOLIC_TRNA_AMINOACYLATION
6:31762311	*HLA-DRB5*	REACTOME_CYTOSOLIC_TRNA_AMINOACYLATION
3:48835245	*IP6K2*	REACTOME_DAG_AND_IP3_SIGNALING
3:123089448	*KPNA1*	REACTOME_DAG_AND_IP3_SIGNALING
4:114424939	*CAMK2D*	REACTOME_DAG_AND_IP3_SIGNALING
4:114494052	*CAMK2D*	REACTOME_DAG_AND_IP3_SIGNALING
4:114529492	*CAMK2D*	REACTOME_DAG_AND_IP3_SIGNALING
4:114539964	*CAMK2D*	REACTOME_DAG_AND_IP3_SIGNALING
4:114597918	*CAMK2D*	REACTOME_DAG_AND_IP3_SIGNALING
8:131799767	*FAM49B*	REACTOME_DAG_AND_IP3_SIGNALING
8:131845512	*FAM49B*	REACTOME_DAG_AND_IP3_SIGNALING
8:131865425	*FAM49B*	REACTOME_DAG_AND_IP3_SIGNALING
8:131901170	*FAM49B*	REACTOME_DAG_AND_IP3_SIGNALING
16:50300511	*CASC16*	REACTOME_DAG_AND_IP3_SIGNALING
16:50317039	*CASC16*	REACTOME_DAG_AND_IP3_SIGNALING
6:112137847	*FYN*	REACTOME_DAP12_INTERACTIONS
6:112151452	*FYN*	REACTOME_DAP12_INTERACTIONS

6:112175069	*FYN*	REACTOME_DAP12_INTERACTIONS
6:112137847	*FYN*	REACTOME_DAP12_SIGNALING
6:112151452	*FYN*	REACTOME_DAP12_SIGNALING
6:112175069	*FYN*	REACTOME_DAP12_SIGNALING
3:48835245	*IP6K2*	REACTOME_DARPP_32_EVENTS
5:59236972	*ELOVL7*	REACTOME_DARPP_32_EVENTS
5:59490965	*ELOVL7*	REACTOME_DARPP_32_EVENTS
5:59554719	*ELOVL7*	REACTOME_DARPP_32_EVENTS
5:59705316	*ELOVL7*	REACTOME_DARPP_32_EVENTS
5:59736085	*ELOVL7*	REACTOME_DARPP_32_EVENTS
5:59812853	*ELOVL7*	REACTOME_DARPP_32_EVENTS
5:133558885	*C5orf24*	REACTOME_DARPP_32_EVENTS
6:112137847	*FYN*	REACTOME_DCC_MEDIATED_ATTRACTIVE_SIGNALING
6:112151452	*FYN*	REACTOME_DCC_MEDIATED_ATTRACTIVE_SIGNALING
6:112175069	*FYN*	REACTOME_DCC_MEDIATED_ATTRACTIVE_SIGNALING
6:32151443	*HLA-DRB5*	REACTOME_DDX58_IFIH1_MEDIATED_INDUCTION_OF_INTERFERON_ALPHA_BETA
16:50790891	*CASC16*	REACTOME_DDX58_IFIH1_MEDIATED_INDUCTION_OF_INTERFERON_ALPHA_BETA
10:103185542	*GBF1*	REACTOME_DEACTIVATION_OF_THE_BETA_CATENIN_TRANSACTIVATING_COMPLEX
10:103305087	*GBF1*	REACTOME_DEACTIVATION_OF_THE_BETA_CATENIN_TRANSACTIVATING_COMPLEX
10:103308244	*GBF1*	REACTOME_DEACTIVATION_OF_THE_BETA_CATENIN_TRANSACTIVATING_COMPLEX
19:3008151	*SPPL2B*	REACTOME_DEACTIVATION_OF_THE_BETA_CATENIN_TRANSACTIVATING_COMPLEX
19:3024077	*SPPL2B*	REACTOME_DEACTIVATION_OF_THE_BETA_CATENIN_TRANSACTIVATING_COMPLEX
17:7479140	*V1NB1*	REACTOME_DEADENYLATION_DEPENDENT_MRNA_DECAY
17:7480325	*V1NB1*	REACTOME_DEADENYLATION_DEPENDENT_MRNA_DECAY
17:7479140	*V1NB1*	REACTOME_DEADENYLATION_OF_MRNA
17:7480325	*V1NB1*	REACTOME_DEADENYLATION_OF_MRNA
1:155975202	*GBAP1*	REACTOME_DEATH_RECEPTOR_SIGNALLING
1:227067656	*ITPKB*	REACTOME_DEATH_RECEPTOR_SIGNALLING
3:49406095	*IP6K2*	REACTOME_DEATH_RECEPTOR_SIGNALLING
3:49424231	*IP6K2*	REACTOME_DEATH_RECEPTOR_SIGNALLING
3:49450137	*IP6K2*	REACTOME_DEATH_RECEPTOR_SIGNALLING
8:23050400	*BIN3*	REACTOME_DEATH_RECEPTOR_SIGNALLING
16:50790891	*CASC16*	REACTOME_DEATH_RECEPTOR_SIGNALLING
19:2335872	*SPPL2B*	REACTOME_DEATH_RECEPTOR_SIGNALLING
19:2346990	*SPPL2B*	REACTOME_DEATH_RECEPTOR_SIGNALLING
19:2352757	*SPPL2B*	REACTOME_DEATH_RECEPTOR_SIGNALLING
10:103185542	*GBF1*	REACTOME_DECTIN_1_MEDIATED_NONCANONICAL_NF_KB_SIGNALING
10:103305087	*GBF1*	REACTOME_DECTIN_1_MEDIATED_NONCANONICAL_NF_KB_SIGNALING
10:103308244	*GBF1*	REACTOME_DECTIN_1_MEDIATED_NONCANONICAL_NF_KB_SIGNALING
17:43390763	*CRHR1_2*	REACTOME_DECTIN_1_MEDIATED_NONCANONICAL_NF_KB_SIGNALING
6:112137847	*FYN*	REACTOME_DECTIN_2_FAMILY
6:112151452	*FYN*	REACTOME_DECTIN_2_FAMILY
6:112175069	*FYN*	REACTOME_DECTIN_2_FAMILY
6:32151443	*HLA-DRB5*	REACTOME_DEFECTIVE_CFTR_CAUSES_CYSTIC_FIBROSIS
6:32179319	*HLA-DRB5*	REACTOME_DEFECTIVE_LFNG_CAUSES_SCDO3
6:32186193	*HLA-DRB5*	REACTOME_DEFECTIVE_LFNG_CAUSES_SCDO3
3:182760073	*MCCC1*	REACTOME_DEFECTS_IN_BIOTIN_BTN_METABOLISM
21:38136802	*DYRK1A*	REACTOME_DEFECTS_IN_BIOTIN_BTN_METABOLISM
21:38168749	*DYRK1A*	REACTOME_DEFECTS_IN_BIOTIN_BTN_METABOLISM
21:38210057	*DYRK1A*	REACTOME_DEFECTS_IN_BIOTIN_BTN_METABOLISM
21:38238679	*DYRK1A*	REACTOME_DEFECTS_IN_BIOTIN_BTN_METABOLISM
21:38328886	*DYRK1A*	REACTOME_DEFECTS_IN_BIOTIN_BTN_METABOLISM
21:38332144	*DYRK1A*	REACTOME_DEFECTS_IN_BIOTIN_BTN_METABOLISM

21:38356061	*DYRK1A*	REACTOME_DEFECTS_IN_BIOTIN_BTN_METABOLISM
14:74764925	*RPS6KL1*	REACTOME_DEFECTS_IN_COBALAMIN_B12_METABOLISM
3:182760073	*MCCC1*	REACTOME_DEFECTS_IN_VITAMIN_AND_COFACTOR_METABOLISM
14:74764925	*RPS6KL1*	REACTOME_DEFECTS_IN_VITAMIN_AND_COFACTOR_METABOLISM
21:38136802	*DYRK1A*	REACTOME_DEFECTS_IN_VITAMIN_AND_COFACTOR_METABOLISM
21:38168749	*DYRK1A*	REACTOME_DEFECTS_IN_VITAMIN_AND_COFACTOR_METABOLISM
21:38210057	*DYRK1A*	REACTOME_DEFECTS_IN_VITAMIN_AND_COFACTOR_METABOLISM
21:38238679	*DYRK1A*	REACTOME_DEFECTS_IN_VITAMIN_AND_COFACTOR_METABOLISM
21:38328886	*DYRK1A*	REACTOME_DEFECTS_IN_VITAMIN_AND_COFACTOR_METABOLISM
21:38332144	*DYRK1A*	REACTOME_DEFECTS_IN_VITAMIN_AND_COFACTOR_METABOLISM
21:38356061	*DYRK1A*	REACTOME_DEFECTS_IN_VITAMIN_AND_COFACTOR_METABOLISM
9:33791164	*UBAP2*	REACTOME_DEFENSINS
5:133558885	*C5orf24*	REACTOME_DEGRADATION_OF_BETA_CATENIN_BY_THE_DESTRUCTION_COMPLEX
10:103185542	*GBF1*	REACTOME_DEGRADATION_OF_BETA_CATENIN_BY_THE_DESTRUCTION_COMPLEX
10:103305087	*GBF1*	REACTOME_DEGRADATION_OF_BETA_CATENIN_BY_THE_DESTRUCTION_COMPLEX
10:103308244	*GBF1*	REACTOME_DEGRADATION_OF_BETA_CATENIN_BY_THE_DESTRUCTION_COMPLEX
19:3008151	*SPPL2B*	REACTOME_DEGRADATION_OF_BETA_CATENIN_BY_THE_DESTRUCTION_COMPLEX
19:3024077	*SPPL2B*	REACTOME_DEGRADATION_OF_BETA_CATENIN_BY_THE_DESTRUCTION_COMPLEX
17:7129974	*V1NB1*	REACTOME_DEGRADATION_OF_DVL
10:103185542	*GBF1*	REACTOME_DEGRADATION_OF_GLI1_BY_THE_PROTEASOME
10:103305087	*GBF1*	REACTOME_DEGRADATION_OF_GLI1_BY_THE_PROTEASOME
10:103308244	*GBF1*	REACTOME_DEGRADATION_OF_GLI1_BY_THE_PROTEASOME
10:104278055	*GBF1*	REACTOME_DEGRADATION_OF_GLI1_BY_THE_PROTEASOME
10:104305501	*GBF1*	REACTOME_DEGRADATION_OF_GLI1_BY_THE_PROTEASOME
1:155027082	*GBAP1*	REACTOME_DEGRADATION_OF_THE_EXTRACELLULAR_MATRIX
1:155033317	*GBAP1*	REACTOME_DEGRADATION_OF_THE_EXTRACELLULAR_MATRIX
6:33153528	*HLA-DRB5*	REACTOME_DEGRADATION_OF_THE_EXTRACELLULAR_MATRIX
8:11712443	*CTSB*	REACTOME_DEGRADATION_OF_THE_EXTRACELLULAR_MATRIX
17:76869878	*DNAH17*	REACTOME_DEGRADATION_OF_THE_EXTRACELLULAR_MATRIX
1:156059276	*GBAP1*	REACTOME_DEPOLYMERISATION_OF_THE_NUCLEAR_LAMINA
9:34342881	*UBAP2*	REACTOME_DETOXIFICATION_OF_REACTIVE_OXYGEN_SPECIES
3:48199695	*IP6K2*	REACTOME_DEUBIQUITINATION
3:48214438	*IP6K2*	REACTOME_DEUBIQUITINATION
3:49406095	*IP6K2*	REACTOME_DEUBIQUITINATION
3:49424231	*IP6K2*	REACTOME_DEUBIQUITINATION
3:49450137	*IP6K2*	REACTOME_DEUBIQUITINATION
3:49740895	*IP6K2*	REACTOME_DEUBIQUITINATION
16:19562008	*SYT17*	REACTOME_DEUBIQUITINATION
16:50730088	*CASC16*	REACTOME_DEUBIQUITINATION
16:50730446	*CASC16*	REACTOME_DEUBIQUITINATION
16:50745926	*CASC16*	REACTOME_DEUBIQUITINATION
16:50790891	*CASC16*	REACTOME_DEUBIQUITINATION
17:7578671	*V1NB1*	REACTOME_DEUBIQUITINATION
1:171837985	*VAMP4*	REACTOME_DEVELOPMENTAL_BIOLOGY
1:171945488	*VAMP4*	REACTOME_DEVELOPMENTAL_BIOLOGY
1:172137119	*VAMP4*	REACTOME_DEVELOPMENTAL_BIOLOGY
1:172386261	*VAMP4*	REACTOME_DEVELOPMENTAL_BIOLOGY
1:204876966	*RAB29*	REACTOME_DEVELOPMENTAL_BIOLOGY
1:204940972	*RAB29*	REACTOME_DEVELOPMENTAL_BIOLOGY
1:226074321	*ITPKB*	REACTOME_DEVELOPMENTAL_BIOLOGY
1:227067656	*ITPKB*	REACTOME_DEVELOPMENTAL_BIOLOGY
3:48835245	*IP6K2*	REACTOME_DEVELOPMENTAL_BIOLOGY
3:49406095	*IP6K2*	REACTOME_DEVELOPMENTAL_BIOLOGY

3:49424231	*IP6K2*	REACTOME_DEVELOPMENTAL_BIOLOGY
3:49450137	*IP6K2*	REACTOME_DEVELOPMENTAL_BIOLOGY
3:49561208	*IP6K2*	REACTOME_DEVELOPMENTAL_BIOLOGY
4:17622910	*LCORL*	REACTOME_DEVELOPMENTAL_BIOLOGY
4:113947327	*CAMK2D*	REACTOME_DEVELOPMENTAL_BIOLOGY
4:114015252	*CAMK2D*	REACTOME_DEVELOPMENTAL_BIOLOGY
4:114032570	*CAMK2D*	REACTOME_DEVELOPMENTAL_BIOLOGY
4:114067233	*CAMK2D*	REACTOME_DEVELOPMENTAL_BIOLOGY
4:114077386	*CAMK2D*	REACTOME_DEVELOPMENTAL_BIOLOGY
4:114113772	*CAMK2D*	REACTOME_DEVELOPMENTAL_BIOLOGY
4:114122398	*CAMK2D*	REACTOME_DEVELOPMENTAL_BIOLOGY
4:114172749	*CAMK2D*	REACTOME_DEVELOPMENTAL_BIOLOGY
4:114217997	*CAMK2D*	REACTOME_DEVELOPMENTAL_BIOLOGY
6:112137847	*FYN*	REACTOME_DEVELOPMENTAL_BIOLOGY
6:112151452	*FYN*	REACTOME_DEVELOPMENTAL_BIOLOGY
6:112175069	*FYN*	REACTOME_DEVELOPMENTAL_BIOLOGY
8:21565088	*BIN3*	REACTOME_DEVELOPMENTAL_BIOLOGY
8:21582921	*BIN3*	REACTOME_DEVELOPMENTAL_BIOLOGY
8:21665306	*BIN3*	REACTOME_DEVELOPMENTAL_BIOLOGY
8:21668901	*BIN3*	REACTOME_DEVELOPMENTAL_BIOLOGY
8:22007293	*BIN3*	REACTOME_DEVELOPMENTAL_BIOLOGY
9:17579690	*SH3GL2_2*	REACTOME_DEVELOPMENTAL_BIOLOGY
9:17587160	*SH3GL2_2*	REACTOME_DEVELOPMENTAL_BIOLOGY
9:17609302	*SH3GL2_2*	REACTOME_DEVELOPMENTAL_BIOLOGY
9:17626762	*SH3GL2_2*	REACTOME_DEVELOPMENTAL_BIOLOGY
9:17627444	*SH3GL2_2*	REACTOME_DEVELOPMENTAL_BIOLOGY
9:17642664	*SH3GL2_2*	REACTOME_DEVELOPMENTAL_BIOLOGY
9:17710876	*SH3GL2_2*	REACTOME_DEVELOPMENTAL_BIOLOGY
9:17732590	*SH3GL2_2*	REACTOME_DEVELOPMENTAL_BIOLOGY
9:17737514	*SH3GL2_2*	REACTOME_DEVELOPMENTAL_BIOLOGY
9:17758945	*SH3GL2_2*	REACTOME_DEVELOPMENTAL_BIOLOGY
12:41144387	*LRRK2_2*	REACTOME_DEVELOPMENTAL_BIOLOGY
12:41147295	*LRRK2_2*	REACTOME_DEVELOPMENTAL_BIOLOGY
12:41195135	*LRRK2_2*	REACTOME_DEVELOPMENTAL_BIOLOGY
12:41251554	*LRRK2_2*	REACTOME_DEVELOPMENTAL_BIOLOGY
12:41255129	*LRRK2_2*	REACTOME_DEVELOPMENTAL_BIOLOGY
12:41297969	*LRRK2_2*	REACTOME_DEVELOPMENTAL_BIOLOGY
12:41407403	*LRRK2_2*	REACTOME_DEVELOPMENTAL_BIOLOGY
16:31004169	*SETD1A*	REACTOME_DEVELOPMENTAL_BIOLOGY
17:7101771	*V1NB1*	REACTOME_DEVELOPMENTAL_BIOLOGY
17:39883520	*RETREG3*	REACTOME_DEVELOPMENTAL_BIOLOGY
17:42454243	*FAM171A2*	REACTOME_DEVELOPMENTAL_BIOLOGY
17:42855931	*CRHR1_2*	REACTOME_DEVELOPMENTAL_BIOLOGY
17:60124582	*BRIP1*	REACTOME_DEVELOPMENTAL_BIOLOGY
19:1611856	*SPPL2B*	REACTOME_DEVELOPMENTAL_BIOLOGY
5:133558885	*C5orf24*	REACTOME_DISASSEMBLY_OF_THE_DESTRUCTION_COMPLEX_AND_RECRUITMENT_OF_AXIN_TO_THE_MEMBRANE
17:7129974	*V1NB1*	REACTOME_DISASSEMBLY_OF_THE_DESTRUCTION_COMPLEX_AND_RECRUITMENT_OF_AXIN_TO_THE_MEMBRANE
17:7491546	*V1NB1*	REACTOME_DISEASES_ASSOCIATED_WITH_N_GLYCOSYLATION_OF_PROTEINS
3:49561208	*IP6K2*	REACTOME_DISEASES_ASSOCIATED_WITH_O_GLYCOSYLATION_OF_PROTEINS
3:122645304	*KPNA1*	REACTOME_DISEASES_ASSOCIATED_WITH_O_GLYCOSYLATION_OF_PROTEINS
3:122720608	*KPNA1*	REACTOME_DISEASES_ASSOCIATED_WITH_O_GLYCOSYLATION_OF_PROTEINS
6:32179319	*HLA-DRB5*	REACTOME_DISEASES_ASSOCIATED_WITH_O_GLYCOSYLATION_OF_PROTEINS
6:32186193	*HLA-DRB5*	REACTOME_DISEASES_ASSOCIATED_WITH_O_GLYCOSYLATION_OF_PROTEINS

9:18502815	*SH3GL2_2*	REACTOME_DISEASES_ASSOCIATED_WITH_O_GLYCOSYLATION_OF_PROTEINS
9:18522254	*SH3GL2_2*	REACTOME_DISEASES_ASSOCIATED_WITH_O_GLYCOSYLATION_OF_PROTEINS
9:18583014	*SH3GL2_2*	REACTOME_DISEASES_ASSOCIATED_WITH_O_GLYCOSYLATION_OF_PROTEINS
9:18623302	*SH3GL2_2*	REACTOME_DISEASES_ASSOCIATED_WITH_O_GLYCOSYLATION_OF_PROTEINS
9:18633417	*SH3GL2_2*	REACTOME_DISEASES_ASSOCIATED_WITH_O_GLYCOSYLATION_OF_PROTEINS
9:18663540	*SH3GL2_2*	REACTOME_DISEASES_ASSOCIATED_WITH_O_GLYCOSYLATION_OF_PROTEINS
9:18721847	*SH3GL2_2*	REACTOME_DISEASES_ASSOCIATED_WITH_O_GLYCOSYLATION_OF_PROTEINS
9:18725820	*SH3GL2_2*	REACTOME_DISEASES_ASSOCIATED_WITH_O_GLYCOSYLATION_OF_PROTEINS
1:156059276	*GBAP1*	REACTOME_DISEASE
1:227067656	*ITPKB*	REACTOME_DISEASE
2:135708115	*TMEM163*	REACTOME_DISEASE
3:48199695	*IP6K2*	REACTOME_DISEASE
3:48214438	*IP6K2*	REACTOME_DISEASE
3:49561208	*IP6K2*	REACTOME_DISEASE
3:122200170	*KPNA1*	REACTOME_DISEASE
3:122645304	*KPNA1*	REACTOME_DISEASE
3:122720608	*KPNA1*	REACTOME_DISEASE
3:182760073	*MCCC1*	REACTOME_DISEASE
4:114424939	*CAMK2D*	REACTOME_DISEASE
4:114494052	*CAMK2D*	REACTOME_DISEASE
4:114529492	*CAMK2D*	REACTOME_DISEASE
4:114539964	*CAMK2D*	REACTOME_DISEASE
4:114597918	*CAMK2D*	REACTOME_DISEASE
5:133558885	*C5orf24*	REACTOME_DISEASE
6:31925679	*HLA-DRB5*	REACTOME_DISEASE
6:32151443	*HLA-DRB5*	REACTOME_DISEASE
6:32179319	*HLA-DRB5*	REACTOME_DISEASE
6:32186193	*HLA-DRB5*	REACTOME_DISEASE
6:112137847	*FYN*	REACTOME_DISEASE
6:112151452	*FYN*	REACTOME_DISEASE
6:112175069	*FYN*	REACTOME_DISEASE
7:65425894	*GS1-124K5.11*	REACTOME_DISEASE
8:17125768	*FGF20*	REACTOME_DISEASE
9:17579690	*SH3GL2_2*	REACTOME_DISEASE
9:17587160	*SH3GL2_2*	REACTOME_DISEASE
9:17609302	*SH3GL2_2*	REACTOME_DISEASE
9:17626762	*SH3GL2_2*	REACTOME_DISEASE
9:17627444	*SH3GL2_2*	REACTOME_DISEASE
9:17642664	*SH3GL2_2*	REACTOME_DISEASE
9:17710876	*SH3GL2_2*	REACTOME_DISEASE
9:17732590	*SH3GL2_2*	REACTOME_DISEASE
9:17737514	*SH3GL2_2*	REACTOME_DISEASE
9:17758945	*SH3GL2_2*	REACTOME_DISEASE
9:18502815	*SH3GL2_2*	REACTOME_DISEASE
9:18522254	*SH3GL2_2*	REACTOME_DISEASE
9:18583014	*SH3GL2_2*	REACTOME_DISEASE
9:18623302	*SH3GL2_2*	REACTOME_DISEASE
9:18633417	*SH3GL2_2*	REACTOME_DISEASE
9:18663540	*SH3GL2_2*	REACTOME_DISEASE
9:18721847	*SH3GL2_2*	REACTOME_DISEASE
9:18725820	*SH3GL2_2*	REACTOME_DISEASE
10:15199347	*ITGA8*	REACTOME_DISEASE
10:103185542	*GBF1*	REACTOME_DISEASE

10:103305087	*GBF1*	REACTOME_DISEASE
10:103308244	*GBF1*	REACTOME_DISEASE
10:104591639	*GBF1*	REACTOME_DISEASE
14:74764925	*RPS6KL1*	REACTOME_DISEASE
16:31004169	*SETD1A*	REACTOME_DISEASE
17:7491546	*V1NB1*	REACTOME_DISEASE
17:40392358	*RETREG3*	REACTOME_DISEASE
17:41056283	*RETREG3*	REACTOME_DISEASE
17:42332803	*FAM171A2*	REACTOME_DISEASE
17:42338993	*FAM171A2*	REACTOME_DISEASE
17:42454243	*FAM171A2*	REACTOME_DISEASE
17:43181287	*CRHR1_2*	REACTOME_DISEASE
17:43184627	*CRHR1_2*	REACTOME_DISEASE
21:38136802	*DYRK1A*	REACTOME_DISEASE
21:38168749	*DYRK1A*	REACTOME_DISEASE
21:38210057	*DYRK1A*	REACTOME_DISEASE
21:38238679	*DYRK1A*	REACTOME_DISEASE
21:38328886	*DYRK1A*	REACTOME_DISEASE
21:38332144	*DYRK1A*	REACTOME_DISEASE
21:38356061	*DYRK1A*	REACTOME_DISEASE
7:65425894	*GS1-124K5.11*	REACTOME_DISEASES_OF_CARBOHYDRATE_METABOLISM
17:41056283	*RETREG3*	REACTOME_DISEASES_OF_CARBOHYDRATE_METABOLISM
3:49561208	*IP6K2*	REACTOME_DISEASES_OF_GLYCOSYLATION
3:122645304	*KPNA1*	REACTOME_DISEASES_OF_GLYCOSYLATION
3:122720608	*KPNA1*	REACTOME_DISEASES_OF_GLYCOSYLATION
6:32179319	*HLA-DRB5*	REACTOME_DISEASES_OF_GLYCOSYLATION
6:32186193	*HLA-DRB5*	REACTOME_DISEASES_OF_GLYCOSYLATION
9:18502815	*SH3GL2_2*	REACTOME_DISEASES_OF_GLYCOSYLATION
9:18522254	*SH3GL2_2*	REACTOME_DISEASES_OF_GLYCOSYLATION
9:18583014	*SH3GL2_2*	REACTOME_DISEASES_OF_GLYCOSYLATION
9:18623302	*SH3GL2_2*	REACTOME_DISEASES_OF_GLYCOSYLATION
9:18633417	*SH3GL2_2*	REACTOME_DISEASES_OF_GLYCOSYLATION
9:18663540	*SH3GL2_2*	REACTOME_DISEASES_OF_GLYCOSYLATION
9:18721847	*SH3GL2_2*	REACTOME_DISEASES_OF_GLYCOSYLATION
9:18725820	*SH3GL2_2*	REACTOME_DISEASES_OF_GLYCOSYLATION
17:7491546	*V1NB1*	REACTOME_DISEASES_OF_GLYCOSYLATION
3:182760073	*MCCC1*	REACTOME_DISEASES_OF_METABOLISM
7:65425894	*GS1-124K5.11*	REACTOME_DISEASES_OF_METABOLISM
10:104591639	*GBF1*	REACTOME_DISEASES_OF_METABOLISM
14:74764925	*RPS6KL1*	REACTOME_DISEASES_OF_METABOLISM
17:41056283	*RETREG3*	REACTOME_DISEASES_OF_METABOLISM
21:38136802	*DYRK1A*	REACTOME_DISEASES_OF_METABOLISM
21:38168749	*DYRK1A*	REACTOME_DISEASES_OF_METABOLISM
21:38210057	*DYRK1A*	REACTOME_DISEASES_OF_METABOLISM
21:38238679	*DYRK1A*	REACTOME_DISEASES_OF_METABOLISM
21:38328886	*DYRK1A*	REACTOME_DISEASES_OF_METABOLISM
21:38332144	*DYRK1A*	REACTOME_DISEASES_OF_METABOLISM
21:38356061	*DYRK1A*	REACTOME_DISEASES_OF_METABOLISM
1:156059276	*GBAP1*	REACTOME_DISEASES_OF_SIGNAL_TRANSDUCTION
1:227067656	*ITPKB*	REACTOME_DISEASES_OF_SIGNAL_TRANSDUCTION
4:114424939	*CAMK2D*	REACTOME_DISEASES_OF_SIGNAL_TRANSDUCTION
4:114494052	*CAMK2D*	REACTOME_DISEASES_OF_SIGNAL_TRANSDUCTION
4:114529492	*CAMK2D*	REACTOME_DISEASES_OF_SIGNAL_TRANSDUCTION

4:114539964	*CAMK2D*	REACTOME_DISEASES_OF_SIGNAL_TRANSDUCTION
4:114597918	*CAMK2D*	REACTOME_DISEASES_OF_SIGNAL_TRANSDUCTION
5:133558885	*C5orf24*	REACTOME_DISEASES_OF_SIGNAL_TRANSDUCTION
6:112137847	*FYN*	REACTOME_DISEASES_OF_SIGNAL_TRANSDUCTION
6:112151452	*FYN*	REACTOME_DISEASES_OF_SIGNAL_TRANSDUCTION
6:112175069	*FYN*	REACTOME_DISEASES_OF_SIGNAL_TRANSDUCTION
17:40392358	*RETREG3*	REACTOME_DISEASES_OF_SIGNAL_TRANSDUCTION
17:42454243	*FAM171A2*	REACTOME_DISEASES_OF_SIGNAL_TRANSDUCTION
6:32151443	*HLA-DRB5*	REACTOME_DISORDERS_OF_TRANSMEMBRANE_TRANSPORTERS
14:74764925	*RPS6KL1*	REACTOME_DISORDERS_OF_TRANSMEMBRANE_TRANSPORTERS
17:42332803	*FAM171A2*	REACTOME_DISORDERS_OF_TRANSMEMBRANE_TRANSPORTERS
17:42338993	*FAM171A2*	REACTOME_DISORDERS_OF_TRANSMEMBRANE_TRANSPORTERS
4:76407998	*FAM47E-STBD1*	REACTOME_DNA_DAMAGE_BYPASS
5:133710339	*C5orf24*	REACTOME_DNA_DAMAGE_BYPASS
6:111628626	*FYN*	REACTOME_DNA_DAMAGE_BYPASS
12:133206648	*FBRSL1*	REACTOME_DNA_DAMAGE_BYPASS
12:133223685	*FBRSL1*	REACTOME_DNA_DAMAGE_BYPASS
1:226568665	*ITPKB*	REACTOME_DNA_DAMAGE_RECOGNITION_IN_GG_NER
16:53747395	*CHD9*	REACTOME_DNA_DAMAGE_REVERSAL
16:53842518	*CHD9*	REACTOME_DNA_DAMAGE_REVERSAL
16:53867798	*CHD9*	REACTOME_DNA_DAMAGE_REVERSAL
16:53887081	*CHD9*	REACTOME_DNA_DAMAGE_REVERSAL
16:53933972	*CHD9*	REACTOME_DNA_DAMAGE_REVERSAL
17:7578671	*V1NB1*	REACTOME_DNA_DAMAGE_TELOMERE_STRESS_INDUCED_SENESCENCE
1:226568665	*ITPKB*	REACTOME_DNA_DOUBLE_STRAND_BREAK_REPAIR
2:17941653	*KCNS3*	REACTOME_DNA_DOUBLE_STRAND_BREAK_REPAIR
2:17963171	*KCNS3*	REACTOME_DNA_DOUBLE_STRAND_BREAK_REPAIR
6:133608051	*RPS12*	REACTOME_DNA_DOUBLE_STRAND_BREAK_REPAIR
6:133646494	*RPS12*	REACTOME_DNA_DOUBLE_STRAND_BREAK_REPAIR
6:133702425	*RPS12*	REACTOME_DNA_DOUBLE_STRAND_BREAK_REPAIR
6:133788110	*RPS12*	REACTOME_DNA_DOUBLE_STRAND_BREAK_REPAIR
12:133206648	*FBRSL1*	REACTOME_DNA_DOUBLE_STRAND_BREAK_REPAIR
12:133223685	*FBRSL1*	REACTOME_DNA_DOUBLE_STRAND_BREAK_REPAIR
17:7578671	*V1NB1*	REACTOME_DNA_DOUBLE_STRAND_BREAK_REPAIR
17:59791813	*BRIP1*	REACTOME_DNA_DOUBLE_STRAND_BREAK_REPAIR
17:59922925	*BRIP1*	REACTOME_DNA_DOUBLE_STRAND_BREAK_REPAIR
6:133608051	*RPS12*	REACTOME_DNA_DOUBLE_STRAND_BREAK_RESPONSE
6:133646494	*RPS12*	REACTOME_DNA_DOUBLE_STRAND_BREAK_RESPONSE
6:133702425	*RPS12*	REACTOME_DNA_DOUBLE_STRAND_BREAK_RESPONSE
6:133788110	*RPS12*	REACTOME_DNA_DOUBLE_STRAND_BREAK_RESPONSE
17:7578671	*V1NB1*	REACTOME_DNA_DOUBLE_STRAND_BREAK_RESPONSE
1:226568665	*ITPKB*	REACTOME_DNA_REPAIR
2:17941653	*KCNS3*	REACTOME_DNA_REPAIR
2:17963171	*KCNS3*	REACTOME_DNA_REPAIR
4:76407998	*FAM47E-STBD1*	REACTOME_DNA_REPAIR
5:133710339	*C5orf24*	REACTOME_DNA_REPAIR
6:111628626	*FYN*	REACTOME_DNA_REPAIR
6:133608051	*RPS12*	REACTOME_DNA_REPAIR
6:133646494	*RPS12*	REACTOME_DNA_REPAIR
6:133702425	*RPS12*	REACTOME_DNA_REPAIR
6:133788110	*RPS12*	REACTOME_DNA_REPAIR
12:133206648	*FBRSL1*	REACTOME_DNA_REPAIR
12:133223685	*FBRSL1*	REACTOME_DNA_REPAIR

16:53747395	*CHD9*	REACTOME_DNA_REPAIR
16:53842518	*CHD9*	REACTOME_DNA_REPAIR
16:53867798	*CHD9*	REACTOME_DNA_REPAIR
16:53887081	*CHD9*	REACTOME_DNA_REPAIR
16:53933972	*CHD9*	REACTOME_DNA_REPAIR
17:7578671	*V1NB1*	REACTOME_DNA_REPAIR
17:59791813	*BRIP1*	REACTOME_DNA_REPAIR
17:59922925	*BRIP1*	REACTOME_DNA_REPAIR
12:133206648	*FBRSL1*	REACTOME_DNA_REPLICATION_INITIATION
12:133223685	*FBRSL1*	REACTOME_DNA_REPLICATION_INITIATION
12:133206648	*FBRSL1*	REACTOME_DNA_REPLICATION_PRE_INITIATION
12:133223685	*FBRSL1*	REACTOME_DNA_REPLICATION_PRE_INITIATION
20:5934513	*CRLS1*	REACTOME_DNA_REPLICATION_PRE_INITIATION
20:5935522	*CRLS1*	REACTOME_DNA_REPLICATION_PRE_INITIATION
20:5968737	*CRLS1*	REACTOME_DNA_REPLICATION_PRE_INITIATION
20:5968964	*CRLS1*	REACTOME_DNA_REPLICATION_PRE_INITIATION
12:133206648	*FBRSL1*	REACTOME_DNA_REPLICATION
12:133223685	*FBRSL1*	REACTOME_DNA_REPLICATION
17:45215865	*WNT3*	REACTOME_DNA_REPLICATION
17:45244822	*WNT3*	REACTOME_DNA_REPLICATION
20:5934513	*CRLS1*	REACTOME_DNA_REPLICATION
20:5935522	*CRLS1*	REACTOME_DNA_REPLICATION
20:5968737	*CRLS1*	REACTOME_DNA_REPLICATION
20:5968964	*CRLS1*	REACTOME_DNA_REPLICATION
20:5934513	*CRLS1*	REACTOME_DNA_STRAND_ELONGATION
20:5935522	*CRLS1*	REACTOME_DNA_STRAND_ELONGATION
20:5968737	*CRLS1*	REACTOME_DNA_STRAND_ELONGATION
20:5968964	*CRLS1*	REACTOME_DNA_STRAND_ELONGATION
6:72646790	*RIMS1*	REACTOME_DOPAMINE_NEUROTRANSMITTER_RELEASE_CYCLE
6:72648340	*RIMS1*	REACTOME_DOPAMINE_NEUROTRANSMITTER_RELEASE_CYCLE
6:72686444	*RIMS1*	REACTOME_DOPAMINE_NEUROTRANSMITTER_RELEASE_CYCLE
6:72716358	*RIMS1*	REACTOME_DOPAMINE_NEUROTRANSMITTER_RELEASE_CYCLE
6:72904599	*RIMS1*	REACTOME_DOPAMINE_NEUROTRANSMITTER_RELEASE_CYCLE
6:72913435	*RIMS1*	REACTOME_DOPAMINE_NEUROTRANSMITTER_RELEASE_CYCLE
6:72930929	*RIMS1*	REACTOME_DOPAMINE_NEUROTRANSMITTER_RELEASE_CYCLE
6:72982289	*RIMS1*	REACTOME_DOPAMINE_NEUROTRANSMITTER_RELEASE_CYCLE
6:72986711	*RIMS1*	REACTOME_DOPAMINE_NEUROTRANSMITTER_RELEASE_CYCLE
6:73008061	*RIMS1*	REACTOME_DOPAMINE_NEUROTRANSMITTER_RELEASE_CYCLE
6:73060492	*RIMS1*	REACTOME_DOPAMINE_NEUROTRANSMITTER_RELEASE_CYCLE
1:226568665	*ITPKB*	REACTOME_DOWNREGULATION_OF_SMAD2_3:SMAD4_TRANSCRIPTIONAL_ACTIVITY
10:103185542	*GBF1*	REACTOME_DOWNSTREAM_SIGNALING_EVENTS_OF_B_CELL_RECEPTOR_BCR
10:103305087	*GBF1*	REACTOME_DOWNSTREAM_SIGNALING_EVENTS_OF_B_CELL_RECEPTOR_BCR
10:103308244	*GBF1*	REACTOME_DOWNSTREAM_SIGNALING_EVENTS_OF_B_CELL_RECEPTOR_BCR
17:40392358	*RETREG3*	REACTOME_DOWNSTREAM_SIGNAL_TRANSDUCTION
6:32602372	*HLA-DRB5*	REACTOME_DOWNSTREAM_TCR_SIGNALING
10:103185542	*GBF1*	REACTOME_DOWNSTREAM_TCR_SIGNALING
10:103305087	*GBF1*	REACTOME_DOWNSTREAM_TCR_SIGNALING
10:103308244	*GBF1*	REACTOME_DOWNSTREAM_TCR_SIGNALING
1:226568665	*ITPKB*	REACTOME_DUAL_INCISION_IN_GG_NER
12:133206648	*FBRSL1*	REACTOME_DUAL_INCISION_IN_GG_NER
12:133223685	*FBRSL1*	REACTOME_DUAL_INCISION_IN_GG_NER
12:133206648	*FBRSL1*	REACTOME_DUAL_INCISION_IN_TC_NER
12:133223685	*FBRSL1*	REACTOME_DUAL_INCISION_IN_TC_NER

20:5934513	*CRLS1*	REACTOME_E2F_ENABLED_INHIBITION_OF_PRE_REPLICATION_COMPLEX_FORMATION
20:5935522	*CRLS1*	REACTOME_E2F_ENABLED_INHIBITION_OF_PRE_REPLICATION_COMPLEX_FORMATION
20:5968737	*CRLS1*	REACTOME_E2F_ENABLED_INHIBITION_OF_PRE_REPLICATION_COMPLEX_FORMATION
20:5968964	*CRLS1*	REACTOME_E2F_ENABLED_INHIBITION_OF_PRE_REPLICATION_COMPLEX_FORMATION
5:133558885	*C5orf24*	REACTOME_E2F_MEDIATED_REGULATION_OF_DNA_REPLICATION
20:5934513	*CRLS1*	REACTOME_E2F_MEDIATED_REGULATION_OF_DNA_REPLICATION
20:5935522	*CRLS1*	REACTOME_E2F_MEDIATED_REGULATION_OF_DNA_REPLICATION
20:5968737	*CRLS1*	REACTOME_E2F_MEDIATED_REGULATION_OF_DNA_REPLICATION
20:5968964	*CRLS1*	REACTOME_E2F_MEDIATED_REGULATION_OF_DNA_REPLICATION
5:133710339	*C5orf24*	REACTOME_E3_UBIQUITIN_LIGASES_UBIQUITINATE_TARGET_PROTEINS
3:122200170	*KPNA1*	REACTOME_EARLY_PHASE_OF_HIV_LIFE_CYCLE
3:49162284	*IP6K2*	REACTOME_ECM_PROTEOGLYCANS
3:49162583	*IP6K2*	REACTOME_ECM_PROTEOGLYCANS
3:49561208	*IP6K2*	REACTOME_ECM_PROTEOGLYCANS
6:32031904	*HLA-DRB5*	REACTOME_ECM_PROTEOGLYCANS
6:32062687	*HLA-DRB5*	REACTOME_ECM_PROTEOGLYCANS
6:112494762	*FYN*	REACTOME_ECM_PROTEOGLYCANS
6:112531401	*FYN*	REACTOME_ECM_PROTEOGLYCANS
6:112561154	*FYN*	REACTOME_ECM_PROTEOGLYCANS
6:112561430	*FYN*	REACTOME_ECM_PROTEOGLYCANS
10:15562608	*ITGA8*	REACTOME_ECM_PROTEOGLYCANS
10:15569598	*ITGA8*	REACTOME_ECM_PROTEOGLYCANS
10:15652368	*ITGA8*	REACTOME_ECM_PROTEOGLYCANS
16:31370452	*SETD1A*	REACTOME_ECM_PROTEOGLYCANS
17:42454243	*FAM171A2*	REACTOME_ECM_PROTEOGLYCANS
9:17579690	*SH3GL2_2*	REACTOME_EGFR_DOWNREGULATION
9:17587160	*SH3GL2_2*	REACTOME_EGFR_DOWNREGULATION
9:17609302	*SH3GL2_2*	REACTOME_EGFR_DOWNREGULATION
9:17626762	*SH3GL2_2*	REACTOME_EGFR_DOWNREGULATION
9:17627444	*SH3GL2_2*	REACTOME_EGFR_DOWNREGULATION
9:17642664	*SH3GL2_2*	REACTOME_EGFR_DOWNREGULATION
9:17710876	*SH3GL2_2*	REACTOME_EGFR_DOWNREGULATION
9:17732590	*SH3GL2_2*	REACTOME_EGFR_DOWNREGULATION
9:17737514	*SH3GL2_2*	REACTOME_EGFR_DOWNREGULATION
9:17758945	*SH3GL2_2*	REACTOME_EGFR_DOWNREGULATION
8:23190941	*BIN3*	REACTOME_ELASTIC_FIBRE_FORMATION
8:23212542	*BIN3*	REACTOME_ELASTIC_FIBRE_FORMATION
8:23216225	*BIN3*	REACTOME_ELASTIC_FIBRE_FORMATION
8:23223713	*BIN3*	REACTOME_ELASTIC_FIBRE_FORMATION
10:15562608	*ITGA8*	REACTOME_ELASTIC_FIBRE_FORMATION
10:15569598	*ITGA8*	REACTOME_ELASTIC_FIBRE_FORMATION
10:15652368	*ITGA8*	REACTOME_ELASTIC_FIBRE_FORMATION
14:75057014	*RPS6KL1*	REACTOME_ELASTIC_FIBRE_FORMATION
8:17125768	*FGF20*	REACTOME_ENDOSOMAL_SORTING_COMPLEX_REQUIRED_FOR_TRANSPORT_ESCRT
6:29798642	*TRIM40*	REACTOME_ENDOSOMAL_VACUOLAR_PATHWAY
13:49890086	*CAB39L*	REACTOME_ENERGY_DEPENDENT_REGULATION_OF_MTOR_BY_LKB1_AMPK
13:49897815	*CAB39L*	REACTOME_ENERGY_DEPENDENT_REGULATION_OF_MTOR_BY_LKB1_AMPK
13:49914395	*CAB39L*	REACTOME_ENERGY_DEPENDENT_REGULATION_OF_MTOR_BY_LKB1_AMPK
13:49927732	*CAB39L*	REACTOME_ENERGY_DEPENDENT_REGULATION_OF_MTOR_BY_LKB1_AMPK
13:49944031	*CAB39L*	REACTOME_ENERGY_DEPENDENT_REGULATION_OF_MTOR_BY_LKB1_AMPK
13:49960054	*CAB39L*	REACTOME_ENERGY_DEPENDENT_REGULATION_OF_MTOR_BY_LKB1_AMPK
13:49976904	*CAB39L*	REACTOME_ENERGY_DEPENDENT_REGULATION_OF_MTOR_BY_LKB1_AMPK
13:50013818	*CAB39L*	REACTOME_ENERGY_DEPENDENT_REGULATION_OF_MTOR_BY_LKB1_AMPK

3:49406095	*IP6K2*	REACTOME_EPHA_MEDIATED_GROWTH_CONE_COLLAPSE
3:49424231	*IP6K2*	REACTOME_EPHA_MEDIATED_GROWTH_CONE_COLLAPSE
3:49450137	*IP6K2*	REACTOME_EPHA_MEDIATED_GROWTH_CONE_COLLAPSE
6:112137847	*FYN*	REACTOME_EPHA_MEDIATED_GROWTH_CONE_COLLAPSE
6:112151452	*FYN*	REACTOME_EPHA_MEDIATED_GROWTH_CONE_COLLAPSE
6:112175069	*FYN*	REACTOME_EPHA_MEDIATED_GROWTH_CONE_COLLAPSE
3:49406095	*IP6K2*	REACTOME_EPHB_MEDIATED_FORWARD_SIGNALING
3:49424231	*IP6K2*	REACTOME_EPHB_MEDIATED_FORWARD_SIGNALING
3:49450137	*IP6K2*	REACTOME_EPHB_MEDIATED_FORWARD_SIGNALING
6:112137847	*FYN*	REACTOME_EPHB_MEDIATED_FORWARD_SIGNALING
6:112151452	*FYN*	REACTOME_EPHB_MEDIATED_FORWARD_SIGNALING
6:112175069	*FYN*	REACTOME_EPHB_MEDIATED_FORWARD_SIGNALING
1:227067656	*ITPKB*	REACTOME_EPH_EPHRIN_MEDIATED_REPULSION_OF_CELLS
6:112137847	*FYN*	REACTOME_EPH_EPHRIN_MEDIATED_REPULSION_OF_CELLS
6:112151452	*FYN*	REACTOME_EPH_EPHRIN_MEDIATED_REPULSION_OF_CELLS
6:112175069	*FYN*	REACTOME_EPH_EPHRIN_MEDIATED_REPULSION_OF_CELLS
1:227067656	*ITPKB*	REACTOME_EPH_EPHRIN_SIGNALING
3:49406095	*IP6K2*	REACTOME_EPH_EPHRIN_SIGNALING
3:49424231	*IP6K2*	REACTOME_EPH_EPHRIN_SIGNALING
3:49450137	*IP6K2*	REACTOME_EPH_EPHRIN_SIGNALING
6:112137847	*FYN*	REACTOME_EPH_EPHRIN_SIGNALING
6:112151452	*FYN*	REACTOME_EPH_EPHRIN_SIGNALING
6:112175069	*FYN*	REACTOME_EPH_EPHRIN_SIGNALING
6:112137847	*FYN*	REACTOME_EPHRIN_SIGNALING
6:112151452	*FYN*	REACTOME_EPHRIN_SIGNALING
6:112175069	*FYN*	REACTOME_EPHRIN_SIGNALING
6:31862876	*HLA-DRB5*	REACTOME_EPIGENETIC_REGULATION_OF_GENE_EXPRESSION
6:33381459	*HLA-DRB5*	REACTOME_EPIGENETIC_REGULATION_OF_GENE_EXPRESSION
3:49406095	*IP6K2*	REACTOME_ERBB2_REGULATES_CELL_MOTILITY
3:49424231	*IP6K2*	REACTOME_ERBB2_REGULATES_CELL_MOTILITY
3:49450137	*IP6K2*	REACTOME_ERBB2_REGULATES_CELL_MOTILITY
6:31862876	*HLA-DRB5*	REACTOME_ERCC6_CSB_AND_EHMT2_G9A_POSITIVELY_REGULATE_RRNA_EXPRESSION
5:133558885	*C5orf24*	REACTOME_ERKS_ARE_INACTIVATED
17:41845031	*FAM171A2*	REACTOME_ERKS_ARE_INACTIVATED
6:32151443	*HLA-DRB5*	REACTOME_ER_QUALITY_CONTROL_COMPARTMENT_ERQC
3:121411876	*KPNA1*	REACTOME_ER_TO_GOLGI_ANTEROGRADE_TRANSPORT
3:121412702	*KPNA1*	REACTOME_ER_TO_GOLGI_ANTEROGRADE_TRANSPORT
3:122986429	*KPNA1*	REACTOME_ER_TO_GOLGI_ANTEROGRADE_TRANSPORT
4:76695927	*FAM47E-STBD1*	REACTOME_ER_TO_GOLGI_ANTEROGRADE_TRANSPORT
4:113947327	*CAMK2D*	REACTOME_ER_TO_GOLGI_ANTEROGRADE_TRANSPORT
4:114015252	*CAMK2D*	REACTOME_ER_TO_GOLGI_ANTEROGRADE_TRANSPORT
4:114032570	*CAMK2D*	REACTOME_ER_TO_GOLGI_ANTEROGRADE_TRANSPORT
4:114067233	*CAMK2D*	REACTOME_ER_TO_GOLGI_ANTEROGRADE_TRANSPORT
4:114077386	*CAMK2D*	REACTOME_ER_TO_GOLGI_ANTEROGRADE_TRANSPORT
4:114113772	*CAMK2D*	REACTOME_ER_TO_GOLGI_ANTEROGRADE_TRANSPORT
4:114122398	*CAMK2D*	REACTOME_ER_TO_GOLGI_ANTEROGRADE_TRANSPORT
4:114172749	*CAMK2D*	REACTOME_ER_TO_GOLGI_ANTEROGRADE_TRANSPORT
4:114217997	*CAMK2D*	REACTOME_ER_TO_GOLGI_ANTEROGRADE_TRANSPORT
10:104005410	*GBF1*	REACTOME_ER_TO_GOLGI_ANTEROGRADE_TRANSPORT
10:104034550	*GBF1*	REACTOME_ER_TO_GOLGI_ANTEROGRADE_TRANSPORT
10:104258303	*GBF1*	REACTOME_ER_TO_GOLGI_ANTEROGRADE_TRANSPORT
10:121669571	*INPP5F*	REACTOME_ER_TO_GOLGI_ANTEROGRADE_TRANSPORT
10:121683206	*INPP5F*	REACTOME_ER_TO_GOLGI_ANTEROGRADE_TRANSPORT

10:121686714	*INPP5F*	REACTOME_ER_TO_GOLGI_ANTEROGRADE_TRANSPORT
10:121689030	*INPP5F*	REACTOME_ER_TO_GOLGI_ANTEROGRADE_TRANSPORT
17:44800946	*WNT3*	REACTOME_ER_TO_GOLGI_ANTEROGRADE_TRANSPORT
17:44833217	*WNT3*	REACTOME_ER_TO_GOLGI_ANTEROGRADE_TRANSPORT
17:45020426	*WNT3*	REACTOME_ER_TO_GOLGI_ANTEROGRADE_TRANSPORT
17:45059032	*WNT3*	REACTOME_ER_TO_GOLGI_ANTEROGRADE_TRANSPORT
17:45089680	*WNT3*	REACTOME_ER_TO_GOLGI_ANTEROGRADE_TRANSPORT
17:42332803	*FAM171A2*	REACTOME_ERYTHROCYTES_TAKE_UP_OXYGEN_AND_RELEASE_CARBON_DIOXIDE
17:42338993	*FAM171A2*	REACTOME_ERYTHROCYTES_TAKE_UP_OXYGEN_AND_RELEASE_CARBON_DIOXIDE
17:40392358	*RETREG3*	REACTOME_ERYTHROPOIETIN_ACTIVATES_STAT5
14:38061742	*MIPOL1*	REACTOME_ESR_MEDIATED_SIGNALING
19:2557113	*SPPL2B*	REACTOME_ESR_MEDIATED_SIGNALING
19:2563327	*SPPL2B*	REACTOME_ESR_MEDIATED_SIGNALING
19:2580057	*SPPL2B*	REACTOME_ESR_MEDIATED_SIGNALING
19:2660983	*SPPL2B*	REACTOME_ESR_MEDIATED_SIGNALING
14:38061742	*MIPOL1*	REACTOME_ESTROGEN_DEPENDENT_GENE_EXPRESSION
17:7479140	*V1NB1*	REACTOME_EUKARYOTIC_TRANSLATION_INITIATION
17:7480325	*V1NB1*	REACTOME_EUKARYOTIC_TRANSLATION_INITIATION
12:133206648	*FBRSL1*	REACTOME_EXTENSION_OF_TELOMERES
12:133223685	*FBRSL1*	REACTOME_EXTENSION_OF_TELOMERES
1:155027082	*GBAP1*	REACTOME_EXTRACELLULAR_MATRIX_ORGANIZATION
1:155033317	*GBAP1*	REACTOME_EXTRACELLULAR_MATRIX_ORGANIZATION
3:49162284	*IP6K2*	REACTOME_EXTRACELLULAR_MATRIX_ORGANIZATION
3:49162583	*IP6K2*	REACTOME_EXTRACELLULAR_MATRIX_ORGANIZATION
3:49561208	*IP6K2*	REACTOME_EXTRACELLULAR_MATRIX_ORGANIZATION
6:30850582	*TRIM40*	REACTOME_EXTRACELLULAR_MATRIX_ORGANIZATION
6:32031904	*HLA-DRB5*	REACTOME_EXTRACELLULAR_MATRIX_ORGANIZATION
6:32062687	*HLA-DRB5*	REACTOME_EXTRACELLULAR_MATRIX_ORGANIZATION
6:33153528	*HLA-DRB5*	REACTOME_EXTRACELLULAR_MATRIX_ORGANIZATION
6:112494762	*FYN*	REACTOME_EXTRACELLULAR_MATRIX_ORGANIZATION
6:112531401	*FYN*	REACTOME_EXTRACELLULAR_MATRIX_ORGANIZATION
6:112561154	*FYN*	REACTOME_EXTRACELLULAR_MATRIX_ORGANIZATION
6:112561430	*FYN*	REACTOME_EXTRACELLULAR_MATRIX_ORGANIZATION
8:11712443	*CTSB*	REACTOME_EXTRACELLULAR_MATRIX_ORGANIZATION
8:23190941	*BIN3*	REACTOME_EXTRACELLULAR_MATRIX_ORGANIZATION
8:23212542	*BIN3*	REACTOME_EXTRACELLULAR_MATRIX_ORGANIZATION
8:23216225	*BIN3*	REACTOME_EXTRACELLULAR_MATRIX_ORGANIZATION
8:23223713	*BIN3*	REACTOME_EXTRACELLULAR_MATRIX_ORGANIZATION
10:15562608	*ITGA8*	REACTOME_EXTRACELLULAR_MATRIX_ORGANIZATION
10:15569598	*ITGA8*	REACTOME_EXTRACELLULAR_MATRIX_ORGANIZATION
10:15652368	*ITGA8*	REACTOME_EXTRACELLULAR_MATRIX_ORGANIZATION
11:133947732	*IGSF9B*	REACTOME_EXTRACELLULAR_MATRIX_ORGANIZATION
14:75057014	*RPS6KL1*	REACTOME_EXTRACELLULAR_MATRIX_ORGANIZATION
16:30510571	*SETD1A*	REACTOME_EXTRACELLULAR_MATRIX_ORGANIZATION
16:30516492	*SETD1A*	REACTOME_EXTRACELLULAR_MATRIX_ORGANIZATION
16:30520856	*SETD1A*	REACTOME_EXTRACELLULAR_MATRIX_ORGANIZATION
16:31370452	*SETD1A*	REACTOME_EXTRACELLULAR_MATRIX_ORGANIZATION
17:42454243	*FAM171A2*	REACTOME_EXTRACELLULAR_MATRIX_ORGANIZATION
17:76869878	*DNAH17*	REACTOME_EXTRACELLULAR_MATRIX_ORGANIZATION
19:2557113	*SPPL2B*	REACTOME_EXTRA_NUCLEAR_ESTROGEN_SIGNALING
19:2563327	*SPPL2B*	REACTOME_EXTRA_NUCLEAR_ESTROGEN_SIGNALING
19:2580057	*SPPL2B*	REACTOME_EXTRA_NUCLEAR_ESTROGEN_SIGNALING
19:2660983	*SPPL2B*	REACTOME_EXTRA_NUCLEAR_ESTROGEN_SIGNALING

3:48835245	*IP6K2*	REACTOME_FACTORS_INVOLVED_IN_MEGAKARYOCYTE_DEVELOPMENT_AND_PLATELET_PRODUCTION
8:11558567	*CTSB*	REACTOME_FACTORS_INVOLVED_IN_MEGAKARYOCYTE_DEVELOPMENT_AND_PLATELET_PRODUCTION
8:11599269	*CTSB*	REACTOME_FACTORS_INVOLVED_IN_MEGAKARYOCYTE_DEVELOPMENT_AND_PLATELET_PRODUCTION
12:39759090	*LRRK2_2*	REACTOME_FACTORS_INVOLVED_IN_MEGAKARYOCYTE_DEVELOPMENT_AND_PLATELET_PRODUCTION
12:39826709	*LRRK2_2*	REACTOME_FACTORS_INVOLVED_IN_MEGAKARYOCYTE_DEVELOPMENT_AND_PLATELET_PRODUCTION
17:7578671	*V1NB1*	REACTOME_FACTORS_INVOLVED_IN_MEGAKARYOCYTE_DEVELOPMENT_AND_PLATELET_PRODUCTION
17:43017194	*CRHR1_2*	REACTOME_FACTORS_INVOLVED_IN_MEGAKARYOCYTE_DEVELOPMENT_AND_PLATELET_PRODUCTION
17:43021962	*CRHR1_2*	REACTOME_FACTORS_INVOLVED_IN_MEGAKARYOCYTE_DEVELOPMENT_AND_PLATELET_PRODUCTION
4:169806784	*CLCN3*	REACTOME_FATTY_ACID_METABOLISM
5:60053220	*ELOVL7*	REACTOME_FATTY_ACID_METABOLISM
5:60085489	*ELOVL7*	REACTOME_FATTY_ACID_METABOLISM
5:60093890	*ELOVL7*	REACTOME_FATTY_ACID_METABOLISM
6:32126002	*HLA-DRB5*	REACTOME_FATTY_ACID_METABOLISM
17:7983799	*V1NB1*	REACTOME_FATTY_ACID_METABOLISM
17:8019125	*V1NB1*	REACTOME_FATTY_ACID_METABOLISM
19:3142784	*SPPL2B*	REACTOME_FATTY_ACIDS_BOUND_TO_GPR40_FFAR1_REGULATE_INSULIN_SECRETION
19:3147326	*SPPL2B*	REACTOME_FATTY_ACIDS_BOUND_TO_GPR40_FFAR1_REGULATE_INSULIN_SECRETION
4:169806784	*CLCN3*	REACTOME_FATTY_ACYL_COA_BIOSYNTHESIS
5:60053220	*ELOVL7*	REACTOME_FATTY_ACYL_COA_BIOSYNTHESIS
5:60085489	*ELOVL7*	REACTOME_FATTY_ACYL_COA_BIOSYNTHESIS
5:60093890	*ELOVL7*	REACTOME_FATTY_ACYL_COA_BIOSYNTHESIS
6:32126002	*HLA-DRB5*	REACTOME_FATTY_ACYL_COA_BIOSYNTHESIS
6:112137847	*FYN*	REACTOME_FC_EPSILON_RECEPTOR_FCERI_SIGNALING
6:112151452	*FYN*	REACTOME_FC_EPSILON_RECEPTOR_FCERI_SIGNALING
6:112175069	*FYN*	REACTOME_FC_EPSILON_RECEPTOR_FCERI_SIGNALING
10:103185542	*GBF1*	REACTOME_FC_EPSILON_RECEPTOR_FCERI_SIGNALING
10:103305087	*GBF1*	REACTOME_FC_EPSILON_RECEPTOR_FCERI_SIGNALING
10:103308244	*GBF1*	REACTOME_FC_EPSILON_RECEPTOR_FCERI_SIGNALING
10:103185542	*GBF1*	REACTOME_FCERI_MEDIATED_NF_KB_ACTIVATION
10:103305087	*GBF1*	REACTOME_FCERI_MEDIATED_NF_KB_ACTIVATION
10:103308244	*GBF1*	REACTOME_FCERI_MEDIATED_NF_KB_ACTIVATION
6:112137847	*FYN*	REACTOME_FCGAMMA_RECEPTOR_FCGR_DEPENDENT_PHAGOCYTOSIS
6:112151452	*FYN*	REACTOME_FCGAMMA_RECEPTOR_FCGR_DEPENDENT_PHAGOCYTOSIS
6:112175069	*FYN*	REACTOME_FCGAMMA_RECEPTOR_FCGR_DEPENDENT_PHAGOCYTOSIS
6:112137847	*FYN*	REACTOME_FCGR_ACTIVATION
6:112151452	*FYN*	REACTOME_FCGR_ACTIVATION
6:112175069	*FYN*	REACTOME_FCGR_ACTIVATION
5:134307319	*C5orf24*	REACTOME_FERTILIZATION
5:134315853	*C5orf24*	REACTOME_FERTILIZATION
17:40392358	*RETREG3*	REACTOME_FGFR1_MUTANT_RECEPTOR_ACTIVATION
11:82582134	*DLG2*	REACTOME_FORMATION_OF_FIBRIN_CLOT_CLOTTING_CASCADE
11:82635123	*DLG2*	REACTOME_FORMATION_OF_FIBRIN_CLOT_CLOTTING_CASCADE
11:82648357	*DLG2*	REACTOME_FORMATION_OF_FIBRIN_CLOT_CLOTTING_CASCADE
2:135708115	*TMEM163*	REACTOME_FORMATION_OF_HIV_ELONGATION_COMPLEX_IN_THE_ABSENCE_OF_HIV_TAT
6:31925679	*HLA-DRB5*	REACTOME_FORMATION_OF_HIV_ELONGATION_COMPLEX_IN_THE_ABSENCE_OF_HIV_TAT
1:226568665	*ITPKB*	REACTOME_FORMATION_OF_INCISION_COMPLEX_IN_GG_NER
17:7578671	*V1NB1*	REACTOME_FORMATION_OF_SENESCENCE_ASSOCIATED_HETEROCHROMATIN_FOCI_SAHF
19:3008151	*SPPL2B*	REACTOME_FORMATION_OF_THE_BETA_CATENIN:TCF_TRANSACTIVATING_COMPLEX
19:3024077	*SPPL2B*	REACTOME_FORMATION_OF_THE_BETA_CATENIN:TCF_TRANSACTIVATING_COMPLEX
17:39883520	*RETREG3*	REACTOME_FORMATION_OF_THE_CORNIFIED_ENVELOPE
6:31925679	*HLA-DRB5*	REACTOME_FORMATION_OF_THE_EARLY_ELONGATION_COMPLEX
4:78136299	*FAM47E-STBD1*	REACTOME_FOXO_MEDIATED_TRANSCRIPTION_OF_CELL_CYCLE_GENES
4:78175384	*FAM47E-STBD1*	REACTOME_FOXO_MEDIATED_TRANSCRIPTION_OF_CELL_CYCLE_GENES

17:41056283	*RETREG3*	REACTOME_FOXO_MEDIATED_TRANSCRIPTION_OF_OXIDATIVE_STRESS_METABOLIC_AND_NEURONAL_GENES
4:78136299	*FAM47E-STBD1*	REACTOME_FOXO_MEDIATED_TRANSCRIPTION
4:78175384	*FAM47E-STBD1*	REACTOME_FOXO_MEDIATED_TRANSCRIPTION
17:41056283	*RETREG3*	REACTOME_FOXO_MEDIATED_TRANSCRIPTION
19:3142784	*SPPL2B*	REACTOME_FREE_FATTY_ACIDS_REGULATE_INSULIN_SECRETION
19:3147326	*SPPL2B*	REACTOME_FREE_FATTY_ACIDS_REGULATE_INSULIN_SECRETION
1:226497419	*ITPKB*	REACTOME_G0_AND_EARLY_G1
3:48199695	*IP6K2*	REACTOME_G0_AND_EARLY_G1
3:48214438	*IP6K2*	REACTOME_G0_AND_EARLY_G1
14:74640180	*RPS6KL1*	REACTOME_G0_AND_EARLY_G1
21:38740824	*DYRK1A*	REACTOME_G0_AND_EARLY_G1
21:38753354	*DYRK1A*	REACTOME_G0_AND_EARLY_G1
21:38770530	*DYRK1A*	REACTOME_G0_AND_EARLY_G1
21:38783614	*DYRK1A*	REACTOME_G0_AND_EARLY_G1
21:38814867	*DYRK1A*	REACTOME_G0_AND_EARLY_G1
5:133558885	*C5orf24*	REACTOME_G1_PHASE
3:48199695	*IP6K2*	REACTOME_G1_S_DNA_DAMAGE_CHECKPOINTS
3:48214438	*IP6K2*	REACTOME_G1_S_DNA_DAMAGE_CHECKPOINTS
17:7578671	*V1NB1*	REACTOME_G1_S_DNA_DAMAGE_CHECKPOINTS
1:226497419	*ITPKB*	REACTOME_G1_S_SPECIFIC_TRANSCRIPTION
3:48199695	*IP6K2*	REACTOME_G1_S_SPECIFIC_TRANSCRIPTION
3:48214438	*IP6K2*	REACTOME_G1_S_SPECIFIC_TRANSCRIPTION
14:74640180	*RPS6KL1*	REACTOME_G1_S_SPECIFIC_TRANSCRIPTION
17:76177567	*DNAH17*	REACTOME_G1_S_SPECIFIC_TRANSCRIPTION
3:48199695	*IP6K2*	REACTOME_G2_M_CHECKPOINTS
3:48214438	*IP6K2*	REACTOME_G2_M_CHECKPOINTS
17:7578671	*V1NB1*	REACTOME_G2_M_CHECKPOINTS
17:59791813	*BRIP1*	REACTOME_G2_M_CHECKPOINTS
17:59922925	*BRIP1*	REACTOME_G2_M_CHECKPOINTS
20:5934513	*CRLS1*	REACTOME_G2_M_CHECKPOINTS
20:5935522	*CRLS1*	REACTOME_G2_M_CHECKPOINTS
20:5968737	*CRLS1*	REACTOME_G2_M_CHECKPOINTS
20:5968964	*CRLS1*	REACTOME_G2_M_CHECKPOINTS
17:7578671	*V1NB1*	REACTOME_G2_M_DNA_DAMAGE_CHECKPOINT
17:59791813	*BRIP1*	REACTOME_G2_M_DNA_DAMAGE_CHECKPOINT
17:59922925	*BRIP1*	REACTOME_G2_M_DNA_DAMAGE_CHECKPOINT
3:123089448	*KPNA1*	REACTOME_GABA_B_RECEPTOR_ACTIVATION
6:29565889	*TRIM40*	REACTOME_GABA_B_RECEPTOR_ACTIVATION
6:29590715	*TRIM40*	REACTOME_GABA_B_RECEPTOR_ACTIVATION
8:131799767	*FAM49B*	REACTOME_GABA_B_RECEPTOR_ACTIVATION
8:131845512	*FAM49B*	REACTOME_GABA_B_RECEPTOR_ACTIVATION
8:131865425	*FAM49B*	REACTOME_GABA_B_RECEPTOR_ACTIVATION
8:131901170	*FAM49B*	REACTOME_GABA_B_RECEPTOR_ACTIVATION
16:50300511	*CASC16*	REACTOME_GABA_B_RECEPTOR_ACTIVATION
16:50317039	*CASC16*	REACTOME_GABA_B_RECEPTOR_ACTIVATION
19:2557113	*SPPL2B*	REACTOME_GABA_B_RECEPTOR_ACTIVATION
19:2563327	*SPPL2B*	REACTOME_GABA_B_RECEPTOR_ACTIVATION
19:2580057	*SPPL2B*	REACTOME_GABA_B_RECEPTOR_ACTIVATION
19:2660983	*SPPL2B*	REACTOME_GABA_B_RECEPTOR_ACTIVATION
21:38984964	*DYRK1A*	REACTOME_GABA_B_RECEPTOR_ACTIVATION
21:39018136	*DYRK1A*	REACTOME_GABA_B_RECEPTOR_ACTIVATION
21:39021814	*DYRK1A*	REACTOME_GABA_B_RECEPTOR_ACTIVATION
21:39039956	*DYRK1A*	REACTOME_GABA_B_RECEPTOR_ACTIVATION

21:39088785	*DYRK1A*	REACTOME_GABA_B_RECEPTOR_ACTIVATION
21:39108160	*DYRK1A*	REACTOME_GABA_B_RECEPTOR_ACTIVATION
21:39120480	*DYRK1A*	REACTOME_GABA_B_RECEPTOR_ACTIVATION
21:39134980	*DYRK1A*	REACTOME_GABA_B_RECEPTOR_ACTIVATION
21:39197323	*DYRK1A*	REACTOME_GABA_B_RECEPTOR_ACTIVATION
21:39217472	*DYRK1A*	REACTOME_GABA_B_RECEPTOR_ACTIVATION
3:123089448	*KPNA1*	REACTOME_GABA_RECEPTOR_ACTIVATION
6:29565889	*TRIM40*	REACTOME_GABA_RECEPTOR_ACTIVATION
6:29590715	*TRIM40*	REACTOME_GABA_RECEPTOR_ACTIVATION
8:131799767	*FAM49B*	REACTOME_GABA_RECEPTOR_ACTIVATION
8:131845512	*FAM49B*	REACTOME_GABA_RECEPTOR_ACTIVATION
8:131865425	*FAM49B*	REACTOME_GABA_RECEPTOR_ACTIVATION
8:131901170	*FAM49B*	REACTOME_GABA_RECEPTOR_ACTIVATION
16:50300511	*CASC16*	REACTOME_GABA_RECEPTOR_ACTIVATION
16:50317039	*CASC16*	REACTOME_GABA_RECEPTOR_ACTIVATION
19:2557113	*SPPL2B*	REACTOME_GABA_RECEPTOR_ACTIVATION
19:2563327	*SPPL2B*	REACTOME_GABA_RECEPTOR_ACTIVATION
19:2580057	*SPPL2B*	REACTOME_GABA_RECEPTOR_ACTIVATION
19:2660983	*SPPL2B*	REACTOME_GABA_RECEPTOR_ACTIVATION
21:38984964	*DYRK1A*	REACTOME_GABA_RECEPTOR_ACTIVATION
21:39018136	*DYRK1A*	REACTOME_GABA_RECEPTOR_ACTIVATION
21:39021814	*DYRK1A*	REACTOME_GABA_RECEPTOR_ACTIVATION
21:39039956	*DYRK1A*	REACTOME_GABA_RECEPTOR_ACTIVATION
21:39088785	*DYRK1A*	REACTOME_GABA_RECEPTOR_ACTIVATION
21:39108160	*DYRK1A*	REACTOME_GABA_RECEPTOR_ACTIVATION
21:39120480	*DYRK1A*	REACTOME_GABA_RECEPTOR_ACTIVATION
21:39134980	*DYRK1A*	REACTOME_GABA_RECEPTOR_ACTIVATION
21:39197323	*DYRK1A*	REACTOME_GABA_RECEPTOR_ACTIVATION
21:39217472	*DYRK1A*	REACTOME_GABA_RECEPTOR_ACTIVATION
6:72646790	*RIMS1*	REACTOME_GABA_SYNTHESIS_RELEASE_REUPTAKE_AND_DEGRADATION
6:72648340	*RIMS1*	REACTOME_GABA_SYNTHESIS_RELEASE_REUPTAKE_AND_DEGRADATION
6:72686444	*RIMS1*	REACTOME_GABA_SYNTHESIS_RELEASE_REUPTAKE_AND_DEGRADATION
6:72716358	*RIMS1*	REACTOME_GABA_SYNTHESIS_RELEASE_REUPTAKE_AND_DEGRADATION
6:72904599	*RIMS1*	REACTOME_GABA_SYNTHESIS_RELEASE_REUPTAKE_AND_DEGRADATION
6:72913435	*RIMS1*	REACTOME_GABA_SYNTHESIS_RELEASE_REUPTAKE_AND_DEGRADATION
6:72930929	*RIMS1*	REACTOME_GABA_SYNTHESIS_RELEASE_REUPTAKE_AND_DEGRADATION
6:72982289	*RIMS1*	REACTOME_GABA_SYNTHESIS_RELEASE_REUPTAKE_AND_DEGRADATION
6:72986711	*RIMS1*	REACTOME_GABA_SYNTHESIS_RELEASE_REUPTAKE_AND_DEGRADATION
6:73008061	*RIMS1*	REACTOME_GABA_SYNTHESIS_RELEASE_REUPTAKE_AND_DEGRADATION
6:73060492	*RIMS1*	REACTOME_GABA_SYNTHESIS_RELEASE_REUPTAKE_AND_DEGRADATION
1:155975202	*GBAP1*	REACTOME_G_ALPHA_12_13_SIGNALLING_EVENTS
3:49406095	*IP6K2*	REACTOME_G_ALPHA_12_13_SIGNALLING_EVENTS
3:49424231	*IP6K2*	REACTOME_G_ALPHA_12_13_SIGNALLING_EVENTS
3:49450137	*IP6K2*	REACTOME_G_ALPHA_12_13_SIGNALLING_EVENTS
19:2557113	*SPPL2B*	REACTOME_G_ALPHA_12_13_SIGNALLING_EVENTS
19:2563327	*SPPL2B*	REACTOME_G_ALPHA_12_13_SIGNALLING_EVENTS
19:2580057	*SPPL2B*	REACTOME_G_ALPHA_12_13_SIGNALLING_EVENTS
19:2660983	*SPPL2B*	REACTOME_G_ALPHA_12_13_SIGNALLING_EVENTS
2:170012307	*STK39*	REACTOME_G_ALPHA_I_SIGNALLING_EVENTS
2:170028024	*STK39*	REACTOME_G_ALPHA_I_SIGNALLING_EVENTS
2:170066424	*STK39*	REACTOME_G_ALPHA_I_SIGNALLING_EVENTS
3:48835245	*IP6K2*	REACTOME_G_ALPHA_I_SIGNALLING_EVENTS
3:123089448	*KPNA1*	REACTOME_G_ALPHA_I_SIGNALLING_EVENTS

3:150935203	*MED12L*	REACTOME_G_ALPHA_I_SIGNALLING_EVENTS
3:151090424	*MED12L*	REACTOME_G_ALPHA_I_SIGNALLING_EVENTS
3:151099733	*MED12L*	REACTOME_G_ALPHA_I_SIGNALLING_EVENTS
4:76924571	*FAM47E-STBD1*	REACTOME_G_ALPHA_I_SIGNALLING_EVENTS
4:114424939	*CAMK2D*	REACTOME_G_ALPHA_I_SIGNALLING_EVENTS
4:114494052	*CAMK2D*	REACTOME_G_ALPHA_I_SIGNALLING_EVENTS
4:114529492	*CAMK2D*	REACTOME_G_ALPHA_I_SIGNALLING_EVENTS
4:114539964	*CAMK2D*	REACTOME_G_ALPHA_I_SIGNALLING_EVENTS
4:114597918	*CAMK2D*	REACTOME_G_ALPHA_I_SIGNALLING_EVENTS
5:59236972	*ELOVL7*	REACTOME_G_ALPHA_I_SIGNALLING_EVENTS
5:59490965	*ELOVL7*	REACTOME_G_ALPHA_I_SIGNALLING_EVENTS
5:59554719	*ELOVL7*	REACTOME_G_ALPHA_I_SIGNALLING_EVENTS
5:59705316	*ELOVL7*	REACTOME_G_ALPHA_I_SIGNALLING_EVENTS
5:59736085	*ELOVL7*	REACTOME_G_ALPHA_I_SIGNALLING_EVENTS
5:59812853	*ELOVL7*	REACTOME_G_ALPHA_I_SIGNALLING_EVENTS
5:133558885	*C5orf24*	REACTOME_G_ALPHA_I_SIGNALLING_EVENTS
6:29565889	*TRIM40*	REACTOME_G_ALPHA_I_SIGNALLING_EVENTS
6:29590715	*TRIM40*	REACTOME_G_ALPHA_I_SIGNALLING_EVENTS
8:131799767	*FAM49B*	REACTOME_G_ALPHA_I_SIGNALLING_EVENTS
8:131845512	*FAM49B*	REACTOME_G_ALPHA_I_SIGNALLING_EVENTS
8:131865425	*FAM49B*	REACTOME_G_ALPHA_I_SIGNALLING_EVENTS
8:131901170	*FAM49B*	REACTOME_G_ALPHA_I_SIGNALLING_EVENTS
10:15199347	*ITGA8*	REACTOME_G_ALPHA_I_SIGNALLING_EVENTS
10:121295884	*INPP5F*	REACTOME_G_ALPHA_I_SIGNALLING_EVENTS
12:123110365	*HIP1R*	REACTOME_G_ALPHA_I_SIGNALLING_EVENTS
12:123110785	*HIP1R*	REACTOME_G_ALPHA_I_SIGNALLING_EVENTS
12:123139394	*HIP1R*	REACTOME_G_ALPHA_I_SIGNALLING_EVENTS
12:123155263	*HIP1R*	REACTOME_G_ALPHA_I_SIGNALLING_EVENTS
12:123165949	*HIP1R*	REACTOME_G_ALPHA_I_SIGNALLING_EVENTS
12:123194636	*HIP1R*	REACTOME_G_ALPHA_I_SIGNALLING_EVENTS
16:50300511	*CASC16*	REACTOME_G_ALPHA_I_SIGNALLING_EVENTS
16:50317039	*CASC16*	REACTOME_G_ALPHA_I_SIGNALLING_EVENTS
17:43181287	*CRHR1_2*	REACTOME_G_ALPHA_I_SIGNALLING_EVENTS
17:43184627	*CRHR1_2*	REACTOME_G_ALPHA_I_SIGNALLING_EVENTS
19:2557113	*SPPL2B*	REACTOME_G_ALPHA_I_SIGNALLING_EVENTS
19:2563327	*SPPL2B*	REACTOME_G_ALPHA_I_SIGNALLING_EVENTS
19:2580057	*SPPL2B*	REACTOME_G_ALPHA_I_SIGNALLING_EVENTS
19:2660983	*SPPL2B*	REACTOME_G_ALPHA_I_SIGNALLING_EVENTS
10:121054578	*INPP5F*	REACTOME_G_ALPHA_Q_SIGNALLING_EVENTS
10:121077191	*INPP5F*	REACTOME_G_ALPHA_Q_SIGNALLING_EVENTS
10:121085648	*INPP5F*	REACTOME_G_ALPHA_Q_SIGNALLING_EVENTS
10:121104943	*INPP5F*	REACTOME_G_ALPHA_Q_SIGNALLING_EVENTS
10:121114292	*INPP5F*	REACTOME_G_ALPHA_Q_SIGNALLING_EVENTS
10:121191186	*INPP5F*	REACTOME_G_ALPHA_Q_SIGNALLING_EVENTS
10:121201427	*INPP5F*	REACTOME_G_ALPHA_Q_SIGNALLING_EVENTS
14:88472612	*GALC*	REACTOME_G_ALPHA_Q_SIGNALLING_EVENTS
19:2557113	*SPPL2B*	REACTOME_G_ALPHA_Q_SIGNALLING_EVENTS
19:2563327	*SPPL2B*	REACTOME_G_ALPHA_Q_SIGNALLING_EVENTS
19:2580057	*SPPL2B*	REACTOME_G_ALPHA_Q_SIGNALLING_EVENTS
19:2660983	*SPPL2B*	REACTOME_G_ALPHA_Q_SIGNALLING_EVENTS
19:3142784	*SPPL2B*	REACTOME_G_ALPHA_Q_SIGNALLING_EVENTS
19:3147326	*SPPL2B*	REACTOME_G_ALPHA_Q_SIGNALLING_EVENTS
20:5290081	*CRLS1*	REACTOME_G_ALPHA_Q_SIGNALLING_EVENTS

3:123089448	*KPNA1*	REACTOME_G_ALPHA_S_SIGNALLING_EVENTS
5:59236972	*ELOVL7*	REACTOME_G_ALPHA_S_SIGNALLING_EVENTS
5:59490965	*ELOVL7*	REACTOME_G_ALPHA_S_SIGNALLING_EVENTS
5:59554719	*ELOVL7*	REACTOME_G_ALPHA_S_SIGNALLING_EVENTS
5:59705316	*ELOVL7*	REACTOME_G_ALPHA_S_SIGNALLING_EVENTS
5:59736085	*ELOVL7*	REACTOME_G_ALPHA_S_SIGNALLING_EVENTS
5:59812853	*ELOVL7*	REACTOME_G_ALPHA_S_SIGNALLING_EVENTS
6:29373213	*TRIM40*	REACTOME_G_ALPHA_S_SIGNALLING_EVENTS
6:29407378	*TRIM40*	REACTOME_G_ALPHA_S_SIGNALLING_EVENTS
6:29412222	*TRIM40*	REACTOME_G_ALPHA_S_SIGNALLING_EVENTS
8:131799767	*FAM49B*	REACTOME_G_ALPHA_S_SIGNALLING_EVENTS
8:131845512	*FAM49B*	REACTOME_G_ALPHA_S_SIGNALLING_EVENTS
8:131865425	*FAM49B*	REACTOME_G_ALPHA_S_SIGNALLING_EVENTS
8:131901170	*FAM49B*	REACTOME_G_ALPHA_S_SIGNALLING_EVENTS
10:121054578	*INPP5F*	REACTOME_G_ALPHA_S_SIGNALLING_EVENTS
10:121077191	*INPP5F*	REACTOME_G_ALPHA_S_SIGNALLING_EVENTS
10:121085648	*INPP5F*	REACTOME_G_ALPHA_S_SIGNALLING_EVENTS
10:121104943	*INPP5F*	REACTOME_G_ALPHA_S_SIGNALLING_EVENTS
10:121114292	*INPP5F*	REACTOME_G_ALPHA_S_SIGNALLING_EVENTS
10:121191186	*INPP5F*	REACTOME_G_ALPHA_S_SIGNALLING_EVENTS
10:121201427	*INPP5F*	REACTOME_G_ALPHA_S_SIGNALLING_EVENTS
16:50300511	*CASC16*	REACTOME_G_ALPHA_S_SIGNALLING_EVENTS
16:50317039	*CASC16*	REACTOME_G_ALPHA_S_SIGNALLING_EVENTS
17:43744203	*CRHR1_2*	REACTOME_G_ALPHA_S_SIGNALLING_EVENTS
17:43748712	*CRHR1_2*	REACTOME_G_ALPHA_S_SIGNALLING_EVENTS
17:43784228	*CRHR1_2*	REACTOME_G_ALPHA_S_SIGNALLING_EVENTS
17:43791502	*CRHR1_2*	REACTOME_G_ALPHA_S_SIGNALLING_EVENTS
17:43796210	*CRHR1_2*	REACTOME_G_ALPHA_S_SIGNALLING_EVENTS
17:43843861	*CRHR1_2*	REACTOME_G_ALPHA_S_SIGNALLING_EVENTS
17:43847503	*CRHR1_2*	REACTOME_G_ALPHA_S_SIGNALLING_EVENTS
17:43849514	*CRHR1_2*	REACTOME_G_ALPHA_S_SIGNALLING_EVENTS
19:2557113	*SPPL2B*	REACTOME_G_ALPHA_S_SIGNALLING_EVENTS
19:2563327	*SPPL2B*	REACTOME_G_ALPHA_S_SIGNALLING_EVENTS
19:2580057	*SPPL2B*	REACTOME_G_ALPHA_S_SIGNALLING_EVENTS
19:2660983	*SPPL2B*	REACTOME_G_ALPHA_S_SIGNALLING_EVENTS
3:123089448	*KPNA1*	REACTOME_G_ALPHA_Z_SIGNALLING_EVENTS
8:131799767	*FAM49B*	REACTOME_G_ALPHA_Z_SIGNALLING_EVENTS
8:131845512	*FAM49B*	REACTOME_G_ALPHA_Z_SIGNALLING_EVENTS
8:131865425	*FAM49B*	REACTOME_G_ALPHA_Z_SIGNALLING_EVENTS
8:131901170	*FAM49B*	REACTOME_G_ALPHA_Z_SIGNALLING_EVENTS
16:50300511	*CASC16*	REACTOME_G_ALPHA_Z_SIGNALLING_EVENTS
16:50317039	*CASC16*	REACTOME_G_ALPHA_Z_SIGNALLING_EVENTS
19:2557113	*SPPL2B*	REACTOME_G_ALPHA_Z_SIGNALLING_EVENTS
19:2563327	*SPPL2B*	REACTOME_G_ALPHA_Z_SIGNALLING_EVENTS
19:2580057	*SPPL2B*	REACTOME_G_ALPHA_Z_SIGNALLING_EVENTS
19:2660983	*SPPL2B*	REACTOME_G_ALPHA_Z_SIGNALLING_EVENTS
4:114899005	*CAMK2D*	REACTOME_GAMMA_CARBOXYLATION_HYPUSINE_FORMATION_AND_ARYLSULFATASE_ACTIVATION
12:133206648	*FBRSL1*	REACTOME_GAP_FILLING_DNA_REPAIR_SYNTHESIS_AND_LIGATION_IN_GG_NER
12:133223685	*FBRSL1*	REACTOME_GAP_FILLING_DNA_REPAIR_SYNTHESIS_AND_LIGATION_IN_GG_NER
19:2557113	*SPPL2B*	REACTOME_G_BETA:GAMMA_SIGNALLING_THROUGH_CDC42
19:2563327	*SPPL2B*	REACTOME_G_BETA:GAMMA_SIGNALLING_THROUGH_CDC42
19:2580057	*SPPL2B*	REACTOME_G_BETA:GAMMA_SIGNALLING_THROUGH_CDC42
19:2660983	*SPPL2B*	REACTOME_G_BETA:GAMMA_SIGNALLING_THROUGH_CDC42

3:49406095	*IP6K2*	REACTOME_G_BETA:GAMMA_SIGNALLING_THROUGH_PI3KGAMMA
3:49424231	*IP6K2*	REACTOME_G_BETA:GAMMA_SIGNALLING_THROUGH_PI3KGAMMA
3:49450137	*IP6K2*	REACTOME_G_BETA:GAMMA_SIGNALLING_THROUGH_PI3KGAMMA
19:2557113	*SPPL2B*	REACTOME_G_BETA:GAMMA_SIGNALLING_THROUGH_PI3KGAMMA
19:2563327	*SPPL2B*	REACTOME_G_BETA:GAMMA_SIGNALLING_THROUGH_PI3KGAMMA
19:2580057	*SPPL2B*	REACTOME_G_BETA:GAMMA_SIGNALLING_THROUGH_PI3KGAMMA
19:2660983	*SPPL2B*	REACTOME_G_BETA:GAMMA_SIGNALLING_THROUGH_PI3KGAMMA
1:226568665	*ITPKB*	REACTOME_GENE_EXPRESSION_TRANSCRIPTION
1:227754686	*ITPKB*	REACTOME_GENE_EXPRESSION_TRANSCRIPTION
1:227804041	*ITPKB*	REACTOME_GENE_EXPRESSION_TRANSCRIPTION
2:135708115	*TMEM163*	REACTOME_GENE_EXPRESSION_TRANSCRIPTION
4:78136299	*FAM47E-STBD1*	REACTOME_GENE_EXPRESSION_TRANSCRIPTION
4:78175384	*FAM47E-STBD1*	REACTOME_GENE_EXPRESSION_TRANSCRIPTION
4:114424939	*CAMK2D*	REACTOME_GENE_EXPRESSION_TRANSCRIPTION
4:114494052	*CAMK2D*	REACTOME_GENE_EXPRESSION_TRANSCRIPTION
4:114529492	*CAMK2D*	REACTOME_GENE_EXPRESSION_TRANSCRIPTION
4:114539964	*CAMK2D*	REACTOME_GENE_EXPRESSION_TRANSCRIPTION
4:114597918	*CAMK2D*	REACTOME_GENE_EXPRESSION_TRANSCRIPTION
5:133558885	*C5orf24*	REACTOME_GENE_EXPRESSION_TRANSCRIPTION
6:28117331	*LOC100131289*	REACTOME_GENE_EXPRESSION_TRANSCRIPTION
6:28223680	*LOC100131289*	REACTOME_GENE_EXPRESSION_TRANSCRIPTION
6:31862876	*HLA-DRB5*	REACTOME_GENE_EXPRESSION_TRANSCRIPTION
6:31925679	*HLA-DRB5*	REACTOME_GENE_EXPRESSION_TRANSCRIPTION
6:32179319	*HLA-DRB5*	REACTOME_GENE_EXPRESSION_TRANSCRIPTION
6:32186193	*HLA-DRB5*	REACTOME_GENE_EXPRESSION_TRANSCRIPTION
6:32941506	*HLA-DRB5*	REACTOME_GENE_EXPRESSION_TRANSCRIPTION
6:33287183	*HLA-DRB5*	REACTOME_GENE_EXPRESSION_TRANSCRIPTION
6:33381459	*HLA-DRB5*	REACTOME_GENE_EXPRESSION_TRANSCRIPTION
8:11353586	*CTSB*	REACTOME_GENE_EXPRESSION_TRANSCRIPTION
8:11363667	*CTSB*	REACTOME_GENE_EXPRESSION_TRANSCRIPTION
8:11381148	*CTSB*	REACTOME_GENE_EXPRESSION_TRANSCRIPTION
8:11558567	*CTSB*	REACTOME_GENE_EXPRESSION_TRANSCRIPTION
8:11599269	*CTSB*	REACTOME_GENE_EXPRESSION_TRANSCRIPTION
8:22108784	*BIN3*	REACTOME_GENE_EXPRESSION_TRANSCRIPTION
8:22974278	*BIN3*	REACTOME_GENE_EXPRESSION_TRANSCRIPTION
8:23050400	*BIN3*	REACTOME_GENE_EXPRESSION_TRANSCRIPTION
12:46134812	*SCAF11*	REACTOME_GENE_EXPRESSION_TRANSCRIPTION
12:46206268	*SCAF11*	REACTOME_GENE_EXPRESSION_TRANSCRIPTION
14:55515304	*GCH1*	REACTOME_GENE_EXPRESSION_TRANSCRIPTION
15:61016235	*VPS13C*	REACTOME_GENE_EXPRESSION_TRANSCRIPTION
15:61065568	*VPS13C*	REACTOME_GENE_EXPRESSION_TRANSCRIPTION
15:61094298	*VPS13C*	REACTOME_GENE_EXPRESSION_TRANSCRIPTION
15:61140736	*VPS13C*	REACTOME_GENE_EXPRESSION_TRANSCRIPTION
15:61142062	*VPS13C*	REACTOME_GENE_EXPRESSION_TRANSCRIPTION
15:61162571	*VPS13C*	REACTOME_GENE_EXPRESSION_TRANSCRIPTION
15:61191617	*VPS13C*	REACTOME_GENE_EXPRESSION_TRANSCRIPTION
15:61195427	*VPS13C*	REACTOME_GENE_EXPRESSION_TRANSCRIPTION
15:61221682	*VPS13C*	REACTOME_GENE_EXPRESSION_TRANSCRIPTION
15:61298486	*VPS13C*	REACTOME_GENE_EXPRESSION_TRANSCRIPTION
15:61375480	*VPS13C*	REACTOME_GENE_EXPRESSION_TRANSCRIPTION
15:61434581	*VPS13C*	REACTOME_GENE_EXPRESSION_TRANSCRIPTION
15:61467078	*VPS13C*	REACTOME_GENE_EXPRESSION_TRANSCRIPTION
15:61470808	*VPS13C*	REACTOME_GENE_EXPRESSION_TRANSCRIPTION

15:61500263	*VPS13C*	REACTOME_GENE_EXPRESSION_TRANSCRIPTION
16:30438930	*SETD1A*	REACTOME_GENE_EXPRESSION_TRANSCRIPTION
16:30510571	*SETD1A*	REACTOME_GENE_EXPRESSION_TRANSCRIPTION
16:30516492	*SETD1A*	REACTOME_GENE_EXPRESSION_TRANSCRIPTION
16:30520856	*SETD1A*	REACTOME_GENE_EXPRESSION_TRANSCRIPTION
16:31753463	*SETD1A*	REACTOME_GENE_EXPRESSION_TRANSCRIPTION
17:7578671	*V1NB1*	REACTOME_GENE_EXPRESSION_TRANSCRIPTION
17:41056283	*RETREG3*	REACTOME_GENE_EXPRESSION_TRANSCRIPTION
17:42454243	*FAM171A2*	REACTOME_GENE_EXPRESSION_TRANSCRIPTION
17:59791813	*BRIP1*	REACTOME_GENE_EXPRESSION_TRANSCRIPTION
17:59922925	*BRIP1*	REACTOME_GENE_EXPRESSION_TRANSCRIPTION
17:59967940	*BRIP1*	REACTOME_GENE_EXPRESSION_TRANSCRIPTION
17:60124582	*BRIP1*	REACTOME_GENE_EXPRESSION_TRANSCRIPTION
19:1611856	*SPPL2B*	REACTOME_GENE_EXPRESSION_TRANSCRIPTION
6:32602372	*HLA-DRB5*	REACTOME_GENERATION_OF_SECOND_MESSENGER_MOLECULES
1:226568665	*ITPKB*	REACTOME_GENERIC_TRANSCRIPTION_PATHWAY
1:227754686	*ITPKB*	REACTOME_GENERIC_TRANSCRIPTION_PATHWAY
1:227804041	*ITPKB*	REACTOME_GENERIC_TRANSCRIPTION_PATHWAY
2:135708115	*TMEM163*	REACTOME_GENERIC_TRANSCRIPTION_PATHWAY
4:78136299	*FAM47E-STBD1*	REACTOME_GENERIC_TRANSCRIPTION_PATHWAY
4:78175384	*FAM47E-STBD1*	REACTOME_GENERIC_TRANSCRIPTION_PATHWAY
4:114424939	*CAMK2D*	REACTOME_GENERIC_TRANSCRIPTION_PATHWAY
4:114494052	*CAMK2D*	REACTOME_GENERIC_TRANSCRIPTION_PATHWAY
4:114529492	*CAMK2D*	REACTOME_GENERIC_TRANSCRIPTION_PATHWAY
4:114539964	*CAMK2D*	REACTOME_GENERIC_TRANSCRIPTION_PATHWAY
4:114597918	*CAMK2D*	REACTOME_GENERIC_TRANSCRIPTION_PATHWAY
5:133558885	*C5orf24*	REACTOME_GENERIC_TRANSCRIPTION_PATHWAY
6:28117331	*LOC100131289*	REACTOME_GENERIC_TRANSCRIPTION_PATHWAY
6:28223680	*LOC100131289*	REACTOME_GENERIC_TRANSCRIPTION_PATHWAY
6:31862876	*HLA-DRB5*	REACTOME_GENERIC_TRANSCRIPTION_PATHWAY
6:31925679	*HLA-DRB5*	REACTOME_GENERIC_TRANSCRIPTION_PATHWAY
6:32179319	*HLA-DRB5*	REACTOME_GENERIC_TRANSCRIPTION_PATHWAY
6:32186193	*HLA-DRB5*	REACTOME_GENERIC_TRANSCRIPTION_PATHWAY
6:32941506	*HLA-DRB5*	REACTOME_GENERIC_TRANSCRIPTION_PATHWAY
6:33287183	*HLA-DRB5*	REACTOME_GENERIC_TRANSCRIPTION_PATHWAY
8:11353586	*CTSB*	REACTOME_GENERIC_TRANSCRIPTION_PATHWAY
8:11363667	*CTSB*	REACTOME_GENERIC_TRANSCRIPTION_PATHWAY
8:11381148	*CTSB*	REACTOME_GENERIC_TRANSCRIPTION_PATHWAY
8:11558567	*CTSB*	REACTOME_GENERIC_TRANSCRIPTION_PATHWAY
8:11599269	*CTSB*	REACTOME_GENERIC_TRANSCRIPTION_PATHWAY
8:22974278	*BIN3*	REACTOME_GENERIC_TRANSCRIPTION_PATHWAY
8:23050400	*BIN3*	REACTOME_GENERIC_TRANSCRIPTION_PATHWAY
12:46134812	*SCAF11*	REACTOME_GENERIC_TRANSCRIPTION_PATHWAY
12:46206268	*SCAF11*	REACTOME_GENERIC_TRANSCRIPTION_PATHWAY
14:55515304	*GCH1*	REACTOME_GENERIC_TRANSCRIPTION_PATHWAY
15:61016235	*VPS13C*	REACTOME_GENERIC_TRANSCRIPTION_PATHWAY
15:61065568	*VPS13C*	REACTOME_GENERIC_TRANSCRIPTION_PATHWAY
15:61094298	*VPS13C*	REACTOME_GENERIC_TRANSCRIPTION_PATHWAY
15:61140736	*VPS13C*	REACTOME_GENERIC_TRANSCRIPTION_PATHWAY
15:61142062	*VPS13C*	REACTOME_GENERIC_TRANSCRIPTION_PATHWAY
15:61162571	*VPS13C*	REACTOME_GENERIC_TRANSCRIPTION_PATHWAY
15:61191617	*VPS13C*	REACTOME_GENERIC_TRANSCRIPTION_PATHWAY
15:61195427	*VPS13C*	REACTOME_GENERIC_TRANSCRIPTION_PATHWAY

15:61221682	*VPS13C*	REACTOME_GENERIC_TRANSCRIPTION_PATHWAY
15:61298486	*VPS13C*	REACTOME_GENERIC_TRANSCRIPTION_PATHWAY
15:61375480	*VPS13C*	REACTOME_GENERIC_TRANSCRIPTION_PATHWAY
15:61434581	*VPS13C*	REACTOME_GENERIC_TRANSCRIPTION_PATHWAY
15:61467078	*VPS13C*	REACTOME_GENERIC_TRANSCRIPTION_PATHWAY
15:61470808	*VPS13C*	REACTOME_GENERIC_TRANSCRIPTION_PATHWAY
15:61500263	*VPS13C*	REACTOME_GENERIC_TRANSCRIPTION_PATHWAY
16:30438930	*SETD1A*	REACTOME_GENERIC_TRANSCRIPTION_PATHWAY
16:30510571	*SETD1A*	REACTOME_GENERIC_TRANSCRIPTION_PATHWAY
16:30516492	*SETD1A*	REACTOME_GENERIC_TRANSCRIPTION_PATHWAY
16:30520856	*SETD1A*	REACTOME_GENERIC_TRANSCRIPTION_PATHWAY
16:31753463	*SETD1A*	REACTOME_GENERIC_TRANSCRIPTION_PATHWAY
17:7578671	*V1NB1*	REACTOME_GENERIC_TRANSCRIPTION_PATHWAY
17:41056283	*RETREG3*	REACTOME_GENERIC_TRANSCRIPTION_PATHWAY
17:42454243	*FAM171A2*	REACTOME_GENERIC_TRANSCRIPTION_PATHWAY
17:59791813	*BRIP1*	REACTOME_GENERIC_TRANSCRIPTION_PATHWAY
17:59922925	*BRIP1*	REACTOME_GENERIC_TRANSCRIPTION_PATHWAY
17:60124582	*BRIP1*	REACTOME_GENERIC_TRANSCRIPTION_PATHWAY
19:1611856	*SPPL2B*	REACTOME_GENERIC_TRANSCRIPTION_PATHWAY
1:226568665	*ITPKB*	REACTOME_GLOBAL_GENOME_NUCLEOTIDE_EXCISION_REPAIR_GG_NER
12:133206648	*FBRSL1*	REACTOME_GLOBAL_GENOME_NUCLEOTIDE_EXCISION_REPAIR_GG_NER
12:133223685	*FBRSL1*	REACTOME_GLOBAL_GENOME_NUCLEOTIDE_EXCISION_REPAIR_GG_NER
2:18091890	*KCNS3*	REACTOME_GLUCAGON_LIKE_PEPTIDE_1_GLP1_REGULATES_INSULIN_SECRETION
2:18130187	*KCNS3*	REACTOME_GLUCAGON_LIKE_PEPTIDE_1_GLP1_REGULATES_INSULIN_SECRETION
2:18141542	*KCNS3*	REACTOME_GLUCAGON_LIKE_PEPTIDE_1_GLP1_REGULATES_INSULIN_SECRETION
2:18143082	*KCNS3*	REACTOME_GLUCAGON_LIKE_PEPTIDE_1_GLP1_REGULATES_INSULIN_SECRETION
2:18176120	*KCNS3*	REACTOME_GLUCAGON_LIKE_PEPTIDE_1_GLP1_REGULATES_INSULIN_SECRETION
2:18247777	*KCNS3*	REACTOME_GLUCAGON_LIKE_PEPTIDE_1_GLP1_REGULATES_INSULIN_SECRETION
2:18299564	*KCNS3*	REACTOME_GLUCAGON_LIKE_PEPTIDE_1_GLP1_REGULATES_INSULIN_SECRETION
2:18315537	*KCNS3*	REACTOME_GLUCAGON_LIKE_PEPTIDE_1_GLP1_REGULATES_INSULIN_SECRETION
2:18335515	*KCNS3*	REACTOME_GLUCAGON_LIKE_PEPTIDE_1_GLP1_REGULATES_INSULIN_SECRETION
2:18455049	*KCNS3*	REACTOME_GLUCAGON_LIKE_PEPTIDE_1_GLP1_REGULATES_INSULIN_SECRETION
2:18472861	*KCNS3*	REACTOME_GLUCAGON_LIKE_PEPTIDE_1_GLP1_REGULATES_INSULIN_SECRETION
3:48835245	*IP6K2*	REACTOME_GLUCAGON_LIKE_PEPTIDE_1_GLP1_REGULATES_INSULIN_SECRETION
3:123089448	*KPNA1*	REACTOME_GLUCAGON_LIKE_PEPTIDE_1_GLP1_REGULATES_INSULIN_SECRETION
8:131799767	*FAM49B*	REACTOME_GLUCAGON_LIKE_PEPTIDE_1_GLP1_REGULATES_INSULIN_SECRETION
8:131845512	*FAM49B*	REACTOME_GLUCAGON_LIKE_PEPTIDE_1_GLP1_REGULATES_INSULIN_SECRETION
8:131865425	*FAM49B*	REACTOME_GLUCAGON_LIKE_PEPTIDE_1_GLP1_REGULATES_INSULIN_SECRETION
8:131901170	*FAM49B*	REACTOME_GLUCAGON_LIKE_PEPTIDE_1_GLP1_REGULATES_INSULIN_SECRETION
19:2557113	*SPPL2B*	REACTOME_GLUCAGON_LIKE_PEPTIDE_1_GLP1_REGULATES_INSULIN_SECRETION
19:2563327	*SPPL2B*	REACTOME_GLUCAGON_LIKE_PEPTIDE_1_GLP1_REGULATES_INSULIN_SECRETION
19:2580057	*SPPL2B*	REACTOME_GLUCAGON_LIKE_PEPTIDE_1_GLP1_REGULATES_INSULIN_SECRETION
19:2660983	*SPPL2B*	REACTOME_GLUCAGON_LIKE_PEPTIDE_1_GLP1_REGULATES_INSULIN_SECRETION
3:48835245	*IP6K2*	REACTOME_GLUCAGON_SIGNALING_IN_METABOLIC_REGULATION
3:123089448	*KPNA1*	REACTOME_GLUCAGON_SIGNALING_IN_METABOLIC_REGULATION
8:131799767	*FAM49B*	REACTOME_GLUCAGON_SIGNALING_IN_METABOLIC_REGULATION
8:131845512	*FAM49B*	REACTOME_GLUCAGON_SIGNALING_IN_METABOLIC_REGULATION
8:131865425	*FAM49B*	REACTOME_GLUCAGON_SIGNALING_IN_METABOLIC_REGULATION
8:131901170	*FAM49B*	REACTOME_GLUCAGON_SIGNALING_IN_METABOLIC_REGULATION
16:50300511	*CASC16*	REACTOME_GLUCAGON_SIGNALING_IN_METABOLIC_REGULATION
16:50317039	*CASC16*	REACTOME_GLUCAGON_SIGNALING_IN_METABOLIC_REGULATION
19:2557113	*SPPL2B*	REACTOME_GLUCAGON_SIGNALING_IN_METABOLIC_REGULATION
19:2563327	*SPPL2B*	REACTOME_GLUCAGON_SIGNALING_IN_METABOLIC_REGULATION

19:2580057	*SPPL2B*	REACTOME_GLUCAGON_SIGNALING_IN_METABOLIC_REGULATION
19:2660983	*SPPL2B*	REACTOME_GLUCAGON_SIGNALING_IN_METABOLIC_REGULATION
19:2557113	*SPPL2B*	REACTOME_GLUCAGON_TYPE_LIGAND_RECEPTORS
19:2563327	*SPPL2B*	REACTOME_GLUCAGON_TYPE_LIGAND_RECEPTORS
19:2580057	*SPPL2B*	REACTOME_GLUCAGON_TYPE_LIGAND_RECEPTORS
19:2660983	*SPPL2B*	REACTOME_GLUCAGON_TYPE_LIGAND_RECEPTORS
10:104591639	*GBF1*	REACTOME_GLUCOCORTICOID_BIOSYNTHESIS
17:41056283	*RETREG3*	REACTOME_GLUCONEOGENESIS
5:133558885	*C5orf24*	REACTOME_GLUCOSE_METABOLISM
17:41056283	*RETREG3*	REACTOME_GLUCOSE_METABOLISM
1:226108200	*ITPKB*	REACTOME_GLUTAMATE_AND_GLUTAMINE_METABOLISM
4:114424939	*CAMK2D*	REACTOME_GLUTAMATE_BINDING_ACTIVATION_OF_AMPA_RECEPTORS_AND_SYNAPTIC_PLASTICITY
4:114494052	*CAMK2D*	REACTOME_GLUTAMATE_BINDING_ACTIVATION_OF_AMPA_RECEPTORS_AND_SYNAPTIC_PLASTICITY
4:114529492	*CAMK2D*	REACTOME_GLUTAMATE_BINDING_ACTIVATION_OF_AMPA_RECEPTORS_AND_SYNAPTIC_PLASTICITY
4:114539964	*CAMK2D*	REACTOME_GLUTAMATE_BINDING_ACTIVATION_OF_AMPA_RECEPTORS_AND_SYNAPTIC_PLASTICITY
4:114597918	*CAMK2D*	REACTOME_GLUTAMATE_BINDING_ACTIVATION_OF_AMPA_RECEPTORS_AND_SYNAPTIC_PLASTICITY
17:7101771	*V1NB1*	REACTOME_GLUTAMATE_BINDING_ACTIVATION_OF_AMPA_RECEPTORS_AND_SYNAPTIC_PLASTICITY
17:44800946	*WNT3*	REACTOME_GLUTAMATE_BINDING_ACTIVATION_OF_AMPA_RECEPTORS_AND_SYNAPTIC_PLASTICITY
17:44833217	*WNT3*	REACTOME_GLUTAMATE_BINDING_ACTIVATION_OF_AMPA_RECEPTORS_AND_SYNAPTIC_PLASTICITY
6:72646790	*RIMS1*	REACTOME_GLUTAMATE_NEUROTRANSMITTER_RELEASE_CYCLE
6:72648340	*RIMS1*	REACTOME_GLUTAMATE_NEUROTRANSMITTER_RELEASE_CYCLE
6:72686444	*RIMS1*	REACTOME_GLUTAMATE_NEUROTRANSMITTER_RELEASE_CYCLE
6:72716358	*RIMS1*	REACTOME_GLUTAMATE_NEUROTRANSMITTER_RELEASE_CYCLE
6:72904599	*RIMS1*	REACTOME_GLUTAMATE_NEUROTRANSMITTER_RELEASE_CYCLE
6:72913435	*RIMS1*	REACTOME_GLUTAMATE_NEUROTRANSMITTER_RELEASE_CYCLE
6:72930929	*RIMS1*	REACTOME_GLUTAMATE_NEUROTRANSMITTER_RELEASE_CYCLE
6:72982289	*RIMS1*	REACTOME_GLUTAMATE_NEUROTRANSMITTER_RELEASE_CYCLE
6:72986711	*RIMS1*	REACTOME_GLUTAMATE_NEUROTRANSMITTER_RELEASE_CYCLE
6:73008061	*RIMS1*	REACTOME_GLUTAMATE_NEUROTRANSMITTER_RELEASE_CYCLE
6:73060492	*RIMS1*	REACTOME_GLUTAMATE_NEUROTRANSMITTER_RELEASE_CYCLE
2:96858914	*KCNIP3*	REACTOME_GLYCEROPHOSPHOLIPID_BIOSYNTHESIS
6:31846234	*HLA-DRB5*	REACTOME_GLYCEROPHOSPHOLIPID_BIOSYNTHESIS
12:123561638	*HIP1R*	REACTOME_GLYCEROPHOSPHOLIPID_BIOSYNTHESIS
12:123585705	*HIP1R*	REACTOME_GLYCEROPHOSPHOLIPID_BIOSYNTHESIS
16:29871730	*CD19*	REACTOME_GLYCEROPHOSPHOLIPID_BIOSYNTHESIS
17:6370241	*V1NB1*	REACTOME_GLYCEROPHOSPHOLIPID_BIOSYNTHESIS
17:6406333	*V1NB1*	REACTOME_GLYCEROPHOSPHOLIPID_BIOSYNTHESIS
20:5575860	*CRLS1*	REACTOME_GLYCEROPHOSPHOLIPID_BIOSYNTHESIS
20:6011934	*CRLS1*	REACTOME_GLYCEROPHOSPHOLIPID_BIOSYNTHESIS
17:41056283	*RETREG3*	REACTOME_GLYCOGEN_STORAGE_DISEASES
5:133558885	*C5orf24*	REACTOME_GLYCOLYSIS
6:71594785	*RIMS1*	REACTOME_GLYCOSAMINOGLYCAN_METABOLISM
7:65425894	*GS1-124K5.11*	REACTOME_GLYCOSAMINOGLYCAN_METABOLISM
11:134255325	*IGSF9B*	REACTOME_GLYCOSAMINOGLYCAN_METABOLISM
13:97182312	*MBNL2*	REACTOME_GLYCOSAMINOGLYCAN_METABOLISM
13:97200682	*MBNL2*	REACTOME_GLYCOSAMINOGLYCAN_METABOLISM
13:97278310	*MBNL2*	REACTOME_GLYCOSAMINOGLYCAN_METABOLISM
13:97441830	*MBNL2*	REACTOME_GLYCOSAMINOGLYCAN_METABOLISM
1:155205634	*GBAP1*	REACTOME_GLYCOSPHINGOLIPID_METABOLISM
3:160817877	*SPTSSB*	REACTOME_GLYCOSPHINGOLIPID_METABOLISM
4:114899005	*CAMK2D*	REACTOME_GLYCOSPHINGOLIPID_METABOLISM
14:88372366	*GALC*	REACTOME_GLYCOSPHINGOLIPID_METABOLISM
14:88393900	*GALC*	REACTOME_GLYCOSPHINGOLIPID_METABOLISM

14:88415750	*GALC*	REACTOME_GLYCOSPHINGOLIPID_METABOLISM
14:88443553	*GALC*	REACTOME_GLYCOSPHINGOLIPID_METABOLISM
12:133295501	*FBRSL1*	REACTOME_GLYOXYLATE_METABOLISM_AND_GLYCINE_DEGRADATION
1:226351254	*ITPKB*	REACTOME_GOLGI_ASSOCIATED_VESICLE_BIOGENESIS
1:226372766	*ITPKB*	REACTOME_GOLGI_ASSOCIATED_VESICLE_BIOGENESIS
3:121411876	*KPNA1*	REACTOME_GOLGI_ASSOCIATED_VESICLE_BIOGENESIS
3:121412702	*KPNA1*	REACTOME_GOLGI_ASSOCIATED_VESICLE_BIOGENESIS
4:925376	*GAK*	REACTOME_GOLGI_ASSOCIATED_VESICLE_BIOGENESIS
9:17579690	*SH3GL2_2*	REACTOME_GOLGI_ASSOCIATED_VESICLE_BIOGENESIS
9:17587160	*SH3GL2_2*	REACTOME_GOLGI_ASSOCIATED_VESICLE_BIOGENESIS
9:17609302	*SH3GL2_2*	REACTOME_GOLGI_ASSOCIATED_VESICLE_BIOGENESIS
9:17626762	*SH3GL2_2*	REACTOME_GOLGI_ASSOCIATED_VESICLE_BIOGENESIS
9:17627444	*SH3GL2_2*	REACTOME_GOLGI_ASSOCIATED_VESICLE_BIOGENESIS
9:17642664	*SH3GL2_2*	REACTOME_GOLGI_ASSOCIATED_VESICLE_BIOGENESIS
9:17710876	*SH3GL2_2*	REACTOME_GOLGI_ASSOCIATED_VESICLE_BIOGENESIS
9:17732590	*SH3GL2_2*	REACTOME_GOLGI_ASSOCIATED_VESICLE_BIOGENESIS
9:17737514	*SH3GL2_2*	REACTOME_GOLGI_ASSOCIATED_VESICLE_BIOGENESIS
9:17758945	*SH3GL2_2*	REACTOME_GOLGI_ASSOCIATED_VESICLE_BIOGENESIS
12:123326598	*HIP1R*	REACTOME_GOLGI_ASSOCIATED_VESICLE_BIOGENESIS
4:76695927	*FAM47E-STBD1*	REACTOME_GOLGI_CISTERNAE_PERICENTRIOLAR_STACK_REORGANIZATION
2:135921205	*TMEM163*	REACTOME_GOLGI_TO_ER_RETROGRADE_TRANSPORT
10:104005410	*GBF1*	REACTOME_GOLGI_TO_ER_RETROGRADE_TRANSPORT
10:104034550	*GBF1*	REACTOME_GOLGI_TO_ER_RETROGRADE_TRANSPORT
10:104258303	*GBF1*	REACTOME_GOLGI_TO_ER_RETROGRADE_TRANSPORT
12:39759090	*LRRK2_2*	REACTOME_GOLGI_TO_ER_RETROGRADE_TRANSPORT
12:39826709	*LRRK2_2*	REACTOME_GOLGI_TO_ER_RETROGRADE_TRANSPORT
17:43017194	*CRHR1_2*	REACTOME_GOLGI_TO_ER_RETROGRADE_TRANSPORT
17:43021962	*CRHR1_2*	REACTOME_GOLGI_TO_ER_RETROGRADE_TRANSPORT
17:44800946	*WNT3*	REACTOME_GOLGI_TO_ER_RETROGRADE_TRANSPORT
17:44833217	*WNT3*	REACTOME_GOLGI_TO_ER_RETROGRADE_TRANSPORT
3:150935203	*MED12L*	REACTOME_GPCR_LIGAND_BINDING
3:151090424	*MED12L*	REACTOME_GPCR_LIGAND_BINDING
3:151099733	*MED12L*	REACTOME_GPCR_LIGAND_BINDING
4:76924571	*FAM47E-STBD1*	REACTOME_GPCR_LIGAND_BINDING
6:29565889	*TRIM40*	REACTOME_GPCR_LIGAND_BINDING
6:29590715	*TRIM40*	REACTOME_GPCR_LIGAND_BINDING
14:88472612	*GALC*	REACTOME_GPCR_LIGAND_BINDING
17:43744203	*CRHR1_2*	REACTOME_GPCR_LIGAND_BINDING
17:43748712	*CRHR1_2*	REACTOME_GPCR_LIGAND_BINDING
17:43784228	*CRHR1_2*	REACTOME_GPCR_LIGAND_BINDING
17:43791502	*CRHR1_2*	REACTOME_GPCR_LIGAND_BINDING
17:43796210	*CRHR1_2*	REACTOME_GPCR_LIGAND_BINDING
17:43843861	*CRHR1_2*	REACTOME_GPCR_LIGAND_BINDING
17:43847503	*CRHR1_2*	REACTOME_GPCR_LIGAND_BINDING
17:43849514	*CRHR1_2*	REACTOME_GPCR_LIGAND_BINDING
17:44843651	*WNT3*	REACTOME_GPCR_LIGAND_BINDING
17:44870978	*WNT3*	REACTOME_GPCR_LIGAND_BINDING
17:44871422	*WNT3*	REACTOME_GPCR_LIGAND_BINDING
17:44888209	*WNT3*	REACTOME_GPCR_LIGAND_BINDING
17:44888715	*WNT3*	REACTOME_GPCR_LIGAND_BINDING
17:44906949	*WNT3*	REACTOME_GPCR_LIGAND_BINDING
17:44912901	*WNT3*	REACTOME_GPCR_LIGAND_BINDING
17:44931399	*WNT3*	REACTOME_GPCR_LIGAND_BINDING

17:44947756	*WNT3*	REACTOME_GPCR_LIGAND_BINDING
19:2557113	*SPPL2B*	REACTOME_GPCR_LIGAND_BINDING
19:2563327	*SPPL2B*	REACTOME_GPCR_LIGAND_BINDING
19:2580057	*SPPL2B*	REACTOME_GPCR_LIGAND_BINDING
19:2660983	*SPPL2B*	REACTOME_GPCR_LIGAND_BINDING
20:5290081	*CRLS1*	REACTOME_GPCR_LIGAND_BINDING
19:2557113	*SPPL2B*	REACTOME_G_PROTEIN_ACTIVATION
19:2563327	*SPPL2B*	REACTOME_G_PROTEIN_ACTIVATION
19:2580057	*SPPL2B*	REACTOME_G_PROTEIN_ACTIVATION
19:2660983	*SPPL2B*	REACTOME_G_PROTEIN_ACTIVATION
3:49406095	*IP6K2*	REACTOME_G_PROTEIN_BETA:GAMMA_SIGNALLING
3:49424231	*IP6K2*	REACTOME_G_PROTEIN_BETA:GAMMA_SIGNALLING
3:49450137	*IP6K2*	REACTOME_G_PROTEIN_BETA:GAMMA_SIGNALLING
19:2557113	*SPPL2B*	REACTOME_G_PROTEIN_BETA:GAMMA_SIGNALLING
19:2563327	*SPPL2B*	REACTOME_G_PROTEIN_BETA:GAMMA_SIGNALLING
19:2580057	*SPPL2B*	REACTOME_G_PROTEIN_BETA:GAMMA_SIGNALLING
19:2660983	*SPPL2B*	REACTOME_G_PROTEIN_BETA:GAMMA_SIGNALLING
6:29565889	*TRIM40*	REACTOME_G_PROTEIN_GATED_POTASSIUM_CHANNELS
6:29590715	*TRIM40*	REACTOME_G_PROTEIN_GATED_POTASSIUM_CHANNELS
19:2557113	*SPPL2B*	REACTOME_G_PROTEIN_GATED_POTASSIUM_CHANNELS
19:2563327	*SPPL2B*	REACTOME_G_PROTEIN_GATED_POTASSIUM_CHANNELS
19:2580057	*SPPL2B*	REACTOME_G_PROTEIN_GATED_POTASSIUM_CHANNELS
19:2660983	*SPPL2B*	REACTOME_G_PROTEIN_GATED_POTASSIUM_CHANNELS
21:38984964	*DYRK1A*	REACTOME_G_PROTEIN_GATED_POTASSIUM_CHANNELS
21:39018136	*DYRK1A*	REACTOME_G_PROTEIN_GATED_POTASSIUM_CHANNELS
21:39021814	*DYRK1A*	REACTOME_G_PROTEIN_GATED_POTASSIUM_CHANNELS
21:39039956	*DYRK1A*	REACTOME_G_PROTEIN_GATED_POTASSIUM_CHANNELS
21:39088785	*DYRK1A*	REACTOME_G_PROTEIN_GATED_POTASSIUM_CHANNELS
21:39108160	*DYRK1A*	REACTOME_G_PROTEIN_GATED_POTASSIUM_CHANNELS
21:39120480	*DYRK1A*	REACTOME_G_PROTEIN_GATED_POTASSIUM_CHANNELS
21:39134980	*DYRK1A*	REACTOME_G_PROTEIN_GATED_POTASSIUM_CHANNELS
21:39197323	*DYRK1A*	REACTOME_G_PROTEIN_GATED_POTASSIUM_CHANNELS
21:39217472	*DYRK1A*	REACTOME_G_PROTEIN_GATED_POTASSIUM_CHANNELS
3:48835245	*IP6K2*	REACTOME_G_PROTEIN_MEDIATED_EVENTS
3:123089448	*KPNA1*	REACTOME_G_PROTEIN_MEDIATED_EVENTS
4:114424939	*CAMK2D*	REACTOME_G_PROTEIN_MEDIATED_EVENTS
4:114494052	*CAMK2D*	REACTOME_G_PROTEIN_MEDIATED_EVENTS
4:114529492	*CAMK2D*	REACTOME_G_PROTEIN_MEDIATED_EVENTS
4:114539964	*CAMK2D*	REACTOME_G_PROTEIN_MEDIATED_EVENTS
4:114597918	*CAMK2D*	REACTOME_G_PROTEIN_MEDIATED_EVENTS
8:131799767	*FAM49B*	REACTOME_G_PROTEIN_MEDIATED_EVENTS
8:131845512	*FAM49B*	REACTOME_G_PROTEIN_MEDIATED_EVENTS
8:131865425	*FAM49B*	REACTOME_G_PROTEIN_MEDIATED_EVENTS
8:131901170	*FAM49B*	REACTOME_G_PROTEIN_MEDIATED_EVENTS
16:50300511	*CASC16*	REACTOME_G_PROTEIN_MEDIATED_EVENTS
16:50317039	*CASC16*	REACTOME_G_PROTEIN_MEDIATED_EVENTS
3:49406095	*IP6K2*	REACTOME_GPVI_MEDIATED_ACTIVATION_CASCADE
3:49424231	*IP6K2*	REACTOME_GPVI_MEDIATED_ACTIVATION_CASCADE
3:49450137	*IP6K2*	REACTOME_GPVI_MEDIATED_ACTIVATION_CASCADE
6:112137847	*FYN*	REACTOME_GPVI_MEDIATED_ACTIVATION_CASCADE
6:112151452	*FYN*	REACTOME_GPVI_MEDIATED_ACTIVATION_CASCADE
6:112175069	*FYN*	REACTOME_GPVI_MEDIATED_ACTIVATION_CASCADE
17:42454243	*FAM171A2*	REACTOME_GRB2:SOS_PROVIDES_LINKAGE_TO_MAPK_SIGNALING_FOR_INTEGRINS

17:40392358	*RETREG3*	REACTOME_GROWTH_HORMONE_RECEPTOR_SIGNALING
3:183427301	*MCCC1*	REACTOME_HATS_ACETYLATE_HISTONES
5:133910742	*C5orf24*	REACTOME_HATS_ACETYLATE_HISTONES
17:7155861	*V1NB1*	REACTOME_HATS_ACETYLATE_HISTONES
17:44160410	*WNT3*	REACTOME_HATS_ACETYLATE_HISTONES
17:44189067	*WNT3*	REACTOME_HATS_ACETYLATE_HISTONES
2:17941653	*KCNS3*	REACTOME_HDR_THROUGH_HOMOLOGOUS_RECOMBINATION_HRR
2:17963171	*KCNS3*	REACTOME_HDR_THROUGH_HOMOLOGOUS_RECOMBINATION_HRR
12:133206648	*FBRSL1*	REACTOME_HDR_THROUGH_HOMOLOGOUS_RECOMBINATION_HRR
12:133223685	*FBRSL1*	REACTOME_HDR_THROUGH_HOMOLOGOUS_RECOMBINATION_HRR
17:59791813	*BRIP1*	REACTOME_HDR_THROUGH_HOMOLOGOUS_RECOMBINATION_HRR
17:59922925	*BRIP1*	REACTOME_HDR_THROUGH_HOMOLOGOUS_RECOMBINATION_HRR
1:226568665	*ITPKB*	REACTOME_HDR_THROUGH_MMEJ_ALT_NHEJ
17:59791813	*BRIP1*	REACTOME_HDR_THROUGH_SINGLE_STRAND_ANNEALING_SSA
17:59922925	*BRIP1*	REACTOME_HDR_THROUGH_SINGLE_STRAND_ANNEALING_SSA
3:48835245	*IP6K2*	REACTOME_HEDGEHOG_OFF_STATE
3:123089448	*KPNA1*	REACTOME_HEDGEHOG_OFF_STATE
8:131799767	*FAM49B*	REACTOME_HEDGEHOG_OFF_STATE
8:131845512	*FAM49B*	REACTOME_HEDGEHOG_OFF_STATE
8:131865425	*FAM49B*	REACTOME_HEDGEHOG_OFF_STATE
8:131901170	*FAM49B*	REACTOME_HEDGEHOG_OFF_STATE
10:103185542	*GBF1*	REACTOME_HEDGEHOG_OFF_STATE
10:103305087	*GBF1*	REACTOME_HEDGEHOG_OFF_STATE
10:103308244	*GBF1*	REACTOME_HEDGEHOG_OFF_STATE
10:104278055	*GBF1*	REACTOME_HEDGEHOG_OFF_STATE
10:104305501	*GBF1*	REACTOME_HEDGEHOG_OFF_STATE
16:50300511	*CASC16*	REACTOME_HEDGEHOG_OFF_STATE
16:50317039	*CASC16*	REACTOME_HEDGEHOG_OFF_STATE
10:104278055	*GBF1*	REACTOME_HEDGEHOG_ON_STATE
10:104305501	*GBF1*	REACTOME_HEDGEHOG_ON_STATE
3:48835245	*IP6K2*	REACTOME_HEMOSTASIS
3:49406095	*IP6K2*	REACTOME_HEMOSTASIS
3:49424231	*IP6K2*	REACTOME_HEMOSTASIS
3:49450137	*IP6K2*	REACTOME_HEMOSTASIS
3:151090424	*MED12L*	REACTOME_HEMOSTASIS
3:151099733	*MED12L*	REACTOME_HEMOSTASIS
4:90804532	*SNCA_2*	REACTOME_HEMOSTASIS
4:90868355	*SNCA_2*	REACTOME_HEMOSTASIS
5:133558885	*C5orf24*	REACTOME_HEMOSTASIS
6:31682029	*HLA-DRB5*	REACTOME_HEMOSTASIS
6:112137847	*FYN*	REACTOME_HEMOSTASIS
6:112151452	*FYN*	REACTOME_HEMOSTASIS
6:112175069	*FYN*	REACTOME_HEMOSTASIS
8:11558567	*CTSB*	REACTOME_HEMOSTASIS
8:11599269	*CTSB*	REACTOME_HEMOSTASIS
8:23050400	*BIN3*	REACTOME_HEMOSTASIS
8:130914130	*FAM49B*	REACTOME_HEMOSTASIS
8:131002284	*FAM49B*	REACTOME_HEMOSTASIS
11:10647977	*RNF141*	REACTOME_HEMOSTASIS
11:10648481	*RNF141*	REACTOME_HEMOSTASIS
11:10660840	*RNF141*	REACTOME_HEMOSTASIS
11:82582134	*DLG2*	REACTOME_HEMOSTASIS
11:82635123	*DLG2*	REACTOME_HEMOSTASIS

11:82648357	*DLG2*	REACTOME_HEMOSTASIS
11:133947732	*IGSF9B*	REACTOME_HEMOSTASIS
12:39759090	*LRRK2_2*	REACTOME_HEMOSTASIS
12:39826709	*LRRK2_2*	REACTOME_HEMOSTASIS
16:30510571	*SETD1A*	REACTOME_HEMOSTASIS
16:30516492	*SETD1A*	REACTOME_HEMOSTASIS
16:30520856	*SETD1A*	REACTOME_HEMOSTASIS
16:31370452	*SETD1A*	REACTOME_HEMOSTASIS
17:7560294	*V1NB1*	REACTOME_HEMOSTASIS
17:7578671	*V1NB1*	REACTOME_HEMOSTASIS
17:42454243	*FAM171A2*	REACTOME_HEMOSTASIS
17:43017194	*CRHR1_2*	REACTOME_HEMOSTASIS
17:43021962	*CRHR1_2*	REACTOME_HEMOSTASIS
17:76969183	*DNAH17*	REACTOME_HEMOSTASIS
19:2557113	*SPPL2B*	REACTOME_HEMOSTASIS
19:2563327	*SPPL2B*	REACTOME_HEMOSTASIS
19:2580057	*SPPL2B*	REACTOME_HEMOSTASIS
19:2660983	*SPPL2B*	REACTOME_HEMOSTASIS
19:3142784	*SPPL2B*	REACTOME_HEMOSTASIS
19:3147326	*SPPL2B*	REACTOME_HEMOSTASIS
6:71594785	*RIMS1*	REACTOME_HEPARAN_SULFATE_HEPARIN_HS_GAG_METABOLISM
7:65425894	*GS1-124K5.11*	REACTOME_HEPARAN_SULFATE_HEPARIN_HS_GAG_METABOLISM
11:134255325	*IGSF9B*	REACTOME_HEPARAN_SULFATE_HEPARIN_HS_GAG_METABOLISM
13:97182312	*MBNL2*	REACTOME_HEPARAN_SULFATE_HEPARIN_HS_GAG_METABOLISM
13:97200682	*MBNL2*	REACTOME_HEPARAN_SULFATE_HEPARIN_HS_GAG_METABOLISM
13:97278310	*MBNL2*	REACTOME_HEPARAN_SULFATE_HEPARIN_HS_GAG_METABOLISM
13:97441830	*MBNL2*	REACTOME_HEPARAN_SULFATE_HEPARIN_HS_GAG_METABOLISM
2:135708115	*TMEM163*	REACTOME_HIV_ELONGATION_ARREST_AND_RECOVERY
6:31925679	*HLA-DRB5*	REACTOME_HIV_ELONGATION_ARREST_AND_RECOVERY
2:135708115	*TMEM163*	REACTOME_HIV_INFECTION
3:122200170	*KPNA1*	REACTOME_HIV_INFECTION
6:31925679	*HLA-DRB5*	REACTOME_HIV_INFECTION
6:112137847	*FYN*	REACTOME_HIV_INFECTION
6:112151452	*FYN*	REACTOME_HIV_INFECTION
6:112175069	*FYN*	REACTOME_HIV_INFECTION
8:17125768	*FGF20*	REACTOME_HIV_INFECTION
10:15199347	*ITGA8*	REACTOME_HIV_INFECTION
10:103185542	*GBF1*	REACTOME_HIV_INFECTION
10:103305087	*GBF1*	REACTOME_HIV_INFECTION
10:103308244	*GBF1*	REACTOME_HIV_INFECTION
17:43181287	*CRHR1_2*	REACTOME_HIV_INFECTION
17:43184627	*CRHR1_2*	REACTOME_HIV_INFECTION
2:135708115	*TMEM163*	REACTOME_HIV_LIFE_CYCLE
3:122200170	*KPNA1*	REACTOME_HIV_LIFE_CYCLE
6:31925679	*HLA-DRB5*	REACTOME_HIV_LIFE_CYCLE
8:17125768	*FGF20*	REACTOME_HIV_LIFE_CYCLE
10:15199347	*ITGA8*	REACTOME_HIV_LIFE_CYCLE
17:43181287	*CRHR1_2*	REACTOME_HIV_LIFE_CYCLE
17:43184627	*CRHR1_2*	REACTOME_HIV_LIFE_CYCLE
17:59791813	*BRIP1*	REACTOME_HOMOLOGOUS_DNA_PAIRING_AND_STRAND_EXCHANGE
17:59922925	*BRIP1*	REACTOME_HOMOLOGOUS_DNA_PAIRING_AND_STRAND_EXCHANGE
1:226568665	*ITPKB*	REACTOME_HOMOLOGY_DIRECTED_REPAIR
2:17941653	*KCNS3*	REACTOME_HOMOLOGY_DIRECTED_REPAIR

2:17963171	*KCNS3*	REACTOME_HOMOLOGY_DIRECTED_REPAIR
12:133206648	*FBRSL1*	REACTOME_HOMOLOGY_DIRECTED_REPAIR
12:133223685	*FBRSL1*	REACTOME_HOMOLOGY_DIRECTED_REPAIR
17:59791813	*BRIP1*	REACTOME_HOMOLOGY_DIRECTED_REPAIR
17:59922925	*BRIP1*	REACTOME_HOMOLOGY_DIRECTED_REPAIR
3:122200170	*KPNA1*	REACTOME_HOST_INTERACTIONS_OF_HIV_FACTORS
6:112137847	*FYN*	REACTOME_HOST_INTERACTIONS_OF_HIV_FACTORS
6:112151452	*FYN*	REACTOME_HOST_INTERACTIONS_OF_HIV_FACTORS
6:112175069	*FYN*	REACTOME_HOST_INTERACTIONS_OF_HIV_FACTORS
10:103185542	*GBF1*	REACTOME_HOST_INTERACTIONS_OF_HIV_FACTORS
10:103305087	*GBF1*	REACTOME_HOST_INTERACTIONS_OF_HIV_FACTORS
10:103308244	*GBF1*	REACTOME_HOST_INTERACTIONS_OF_HIV_FACTORS
3:122200170	*KPNA1*	REACTOME_HOST_INTERACTIONS_WITH_INFLUENZA_FACTORS
4:114424939	*CAMK2D*	REACTOME_HSF1_DEPENDENT_TRANSACTIVATION
4:114494052	*CAMK2D*	REACTOME_HSF1_DEPENDENT_TRANSACTIVATION
4:114529492	*CAMK2D*	REACTOME_HSF1_DEPENDENT_TRANSACTIVATION
4:114539964	*CAMK2D*	REACTOME_HSF1_DEPENDENT_TRANSACTIVATION
4:114597918	*CAMK2D*	REACTOME_HSF1_DEPENDENT_TRANSACTIVATION
13:97182312	*MBNL2*	REACTOME_HS_GAG_BIOSYNTHESIS
13:97200682	*MBNL2*	REACTOME_HS_GAG_BIOSYNTHESIS
13:97278310	*MBNL2*	REACTOME_HS_GAG_BIOSYNTHESIS
13:97441830	*MBNL2*	REACTOME_HS_GAG_BIOSYNTHESIS
7:65425894	*GS1-124K5.11*	REACTOME_HS_GAG_DEGRADATION
10:104258303	*GBF1*	REACTOME_HSP90_CHAPERONE_CYCLE_FOR_STEROID_HORMONE_RECEPTORS_SHR
7:65425894	*GS1-124K5.11*	REACTOME_HYALURONAN_METABOLISM
7:65425894	*GS1-124K5.11*	REACTOME_HYALURONAN_UPTAKE_AND_DEGRADATION
20:5575860	*CRLS1*	REACTOME_HYDROLYSIS_OF_LPC
6:29798642	*TRIM40*	REACTOME_IMMUNOREGULATORY_INTERACTIONS_BETWEEN_A_LYMPHOID_AND_A_NON_LYMPHOID_CELL
16:30510571	*SETD1A*	REACTOME_IMMUNOREGULATORY_INTERACTIONS_BETWEEN_A_LYMPHOID_AND_A_NON_LYMPHOID_CELL
16:30516492	*SETD1A*	REACTOME_IMMUNOREGULATORY_INTERACTIONS_BETWEEN_A_LYMPHOID_AND_A_NON_LYMPHOID_CELL
16:30520856	*SETD1A*	REACTOME_IMMUNOREGULATORY_INTERACTIONS_BETWEEN_A_LYMPHOID_AND_A_NON_LYMPHOID_CELL
8:11558567	*CTSB*	REACTOME_INCRETIN_SYNTHESIS_SECRETION_AND_INACTIVATION
8:11599269	*CTSB*	REACTOME_INCRETIN_SYNTHESIS_SECRETION_AND_INACTIVATION
2:135708115	*TMEM163*	REACTOME_INFECTIOUS_DISEASE
3:122200170	*KPNA1*	REACTOME_INFECTIOUS_DISEASE
6:31925679	*HLA-DRB5*	REACTOME_INFECTIOUS_DISEASE
6:112137847	*FYN*	REACTOME_INFECTIOUS_DISEASE
6:112151452	*FYN*	REACTOME_INFECTIOUS_DISEASE
6:112175069	*FYN*	REACTOME_INFECTIOUS_DISEASE
8:17125768	*FGF20*	REACTOME_INFECTIOUS_DISEASE
9:17579690	*SH3GL2_2*	REACTOME_INFECTIOUS_DISEASE
9:17587160	*SH3GL2_2*	REACTOME_INFECTIOUS_DISEASE
9:17609302	*SH3GL2_2*	REACTOME_INFECTIOUS_DISEASE
9:17626762	*SH3GL2_2*	REACTOME_INFECTIOUS_DISEASE
9:17627444	*SH3GL2_2*	REACTOME_INFECTIOUS_DISEASE
9:17642664	*SH3GL2_2*	REACTOME_INFECTIOUS_DISEASE
9:17710876	*SH3GL2_2*	REACTOME_INFECTIOUS_DISEASE
9:17732590	*SH3GL2_2*	REACTOME_INFECTIOUS_DISEASE
9:17737514	*SH3GL2_2*	REACTOME_INFECTIOUS_DISEASE
9:17758945	*SH3GL2_2*	REACTOME_INFECTIOUS_DISEASE
10:15199347	*ITGA8*	REACTOME_INFECTIOUS_DISEASE
10:103185542	*GBF1*	REACTOME_INFECTIOUS_DISEASE
10:103305087	*GBF1*	REACTOME_INFECTIOUS_DISEASE

10:103308244	*GBF1*	REACTOME_INFECTIOUS_DISEASE
16:31004169	*SETD1A*	REACTOME_INFECTIOUS_DISEASE
17:43181287	*CRHR1_2*	REACTOME_INFECTIOUS_DISEASE
17:43184627	*CRHR1_2*	REACTOME_INFECTIOUS_DISEASE
3:122200170	*KPNA1*	REACTOME_INFLUENZA_INFECTION
5:133558885	*C5orf24*	REACTOME_INHIBITION_OF_REPLICATION_INITIATION_OF_DAMAGED_DNA_BY_RB1_E2F1
17:45215865	*WNT3*	TION_OF_THE_PROTEOLYTIC_ACTIVITY_OF_APC_C_REQUIRED_FOR_THE_ONSET_OF_ANAPHASE_BY_MITOTIC_SPINDLE_CHECI
17:45244822	*WNT3*	TION_OF_THE_PROTEOLYTIC_ACTIVITY_OF_APC_C_REQUIRED_FOR_THE_ONSET_OF_ANAPHASE_BY_MITOTIC_SPINDLE_CHECI
6:31897219	*HLA-DRB5*	REACTOME_INITIAL_TRIGGERING_OF_COMPLEMENT
9:17579690	*SH3GL2_2*	REACTOME_INLB_MEDIATED_ENTRY_OF_LISTERIA_MONOCYTOGENES_INTO_HOST_CELL
9:17587160	*SH3GL2_2*	REACTOME_INLB_MEDIATED_ENTRY_OF_LISTERIA_MONOCYTOGENES_INTO_HOST_CELL
9:17609302	*SH3GL2_2*	REACTOME_INLB_MEDIATED_ENTRY_OF_LISTERIA_MONOCYTOGENES_INTO_HOST_CELL
9:17626762	*SH3GL2_2*	REACTOME_INLB_MEDIATED_ENTRY_OF_LISTERIA_MONOCYTOGENES_INTO_HOST_CELL
9:17627444	*SH3GL2_2*	REACTOME_INLB_MEDIATED_ENTRY_OF_LISTERIA_MONOCYTOGENES_INTO_HOST_CELL
9:17642664	*SH3GL2_2*	REACTOME_INLB_MEDIATED_ENTRY_OF_LISTERIA_MONOCYTOGENES_INTO_HOST_CELL
9:17710876	*SH3GL2_2*	REACTOME_INLB_MEDIATED_ENTRY_OF_LISTERIA_MONOCYTOGENES_INTO_HOST_CELL
9:17732590	*SH3GL2_2*	REACTOME_INLB_MEDIATED_ENTRY_OF_LISTERIA_MONOCYTOGENES_INTO_HOST_CELL
9:17737514	*SH3GL2_2*	REACTOME_INLB_MEDIATED_ENTRY_OF_LISTERIA_MONOCYTOGENES_INTO_HOST_CELL
9:17758945	*SH3GL2_2*	REACTOME_INLB_MEDIATED_ENTRY_OF_LISTERIA_MONOCYTOGENES_INTO_HOST_CELL
1:171837985	*VAMP4*	REACTOME_INNATE_IMMUNE_SYSTEM
1:171945488	*VAMP4*	REACTOME_INNATE_IMMUNE_SYSTEM
1:172137119	*VAMP4*	REACTOME_INNATE_IMMUNE_SYSTEM
1:172386261	*VAMP4*	REACTOME_INNATE_IMMUNE_SYSTEM
1:204876966	*RAB29*	REACTOME_INNATE_IMMUNE_SYSTEM
1:204940972	*RAB29*	REACTOME_INNATE_IMMUNE_SYSTEM
1:226044391	*ITPKB*	REACTOME_INNATE_IMMUNE_SYSTEM
1:226052830	*ITPKB*	REACTOME_INNATE_IMMUNE_SYSTEM
3:49406095	*IP6K2*	REACTOME_INNATE_IMMUNE_SYSTEM
3:49424231	*IP6K2*	REACTOME_INNATE_IMMUNE_SYSTEM
3:49450137	*IP6K2*	REACTOME_INNATE_IMMUNE_SYSTEM
4:15709252	*BST1*	REACTOME_INNATE_IMMUNE_SYSTEM
4:15713608	*BST1*	REACTOME_INNATE_IMMUNE_SYSTEM
4:15724372	*BST1*	REACTOME_INNATE_IMMUNE_SYSTEM
4:15737882	*BST1*	REACTOME_INNATE_IMMUNE_SYSTEM
5:101579003	*PAM*	REACTOME_INNATE_IMMUNE_SYSTEM
5:133558885	*C5orf24*	REACTOME_INNATE_IMMUNE_SYSTEM
6:31895092	*HLA-DRB5*	REACTOME_INNATE_IMMUNE_SYSTEM
6:31897219	*HLA-DRB5*	REACTOME_INNATE_IMMUNE_SYSTEM
6:32151443	*HLA-DRB5*	REACTOME_INNATE_IMMUNE_SYSTEM
6:112137847	*FYN*	REACTOME_INNATE_IMMUNE_SYSTEM
6:112151452	*FYN*	REACTOME_INNATE_IMMUNE_SYSTEM
6:112175069	*FYN*	REACTOME_INNATE_IMMUNE_SYSTEM
6:133025715	*RPS12*	REACTOME_INNATE_IMMUNE_SYSTEM
6:133028870	*RPS12*	REACTOME_INNATE_IMMUNE_SYSTEM
7:65425894	*GS1-124K5.11*	REACTOME_INNATE_IMMUNE_SYSTEM
8:11712443	*CTSB*	REACTOME_INNATE_IMMUNE_SYSTEM
8:22108784	*BIN3*	REACTOME_INNATE_IMMUNE_SYSTEM
9:33791164	*UBAP2*	REACTOME_INNATE_IMMUNE_SYSTEM
10:103185542	*GBF1*	REACTOME_INNATE_IMMUNE_SYSTEM
10:103305087	*GBF1*	REACTOME_INNATE_IMMUNE_SYSTEM
10:103308244	*GBF1*	REACTOME_INNATE_IMMUNE_SYSTEM
11:10392432	*RNF141*	REACTOME_INNATE_IMMUNE_SYSTEM
11:10429998	*RNF141*	REACTOME_INNATE_IMMUNE_SYSTEM

11:10458098	*RNF141*	REACTOME_INNATE_IMMUNE_SYSTEM
11:10471738	*RNF141*	REACTOME_INNATE_IMMUNE_SYSTEM
11:10487997	*RNF141*	REACTOME_INNATE_IMMUNE_SYSTEM
11:10494132	*RNF141*	REACTOME_INNATE_IMMUNE_SYSTEM
11:82582134	*DLG2*	REACTOME_INNATE_IMMUNE_SYSTEM
11:82635123	*DLG2*	REACTOME_INNATE_IMMUNE_SYSTEM
11:82648357	*DLG2*	REACTOME_INNATE_IMMUNE_SYSTEM
12:45755099	*SCAF11*	REACTOME_INNATE_IMMUNE_SYSTEM
12:124219791	*HIP1R*	REACTOME_INNATE_IMMUNE_SYSTEM
16:30510571	*SETD1A*	REACTOME_INNATE_IMMUNE_SYSTEM
16:30516492	*SETD1A*	REACTOME_INNATE_IMMUNE_SYSTEM
16:30520856	*SETD1A*	REACTOME_INNATE_IMMUNE_SYSTEM
16:31370452	*SETD1A*	REACTOME_INNATE_IMMUNE_SYSTEM
16:50730088	*CASC16*	REACTOME_INNATE_IMMUNE_SYSTEM
16:50730446	*CASC16*	REACTOME_INNATE_IMMUNE_SYSTEM
16:50745926	*CASC16*	REACTOME_INNATE_IMMUNE_SYSTEM
16:50790891	*CASC16*	REACTOME_INNATE_IMMUNE_SYSTEM
17:39883520	*RETREG3*	REACTOME_INNATE_IMMUNE_SYSTEM
17:41845031	*FAM171A2*	REACTOME_INNATE_IMMUNE_SYSTEM
17:42423844	*FAM171A2*	REACTOME_INNATE_IMMUNE_SYSTEM
17:42426940	*FAM171A2*	REACTOME_INNATE_IMMUNE_SYSTEM
17:43390763	*CRHR1_2*	REACTOME_INNATE_IMMUNE_SYSTEM
17:76869878	*DNAH17*	REACTOME_INNATE_IMMUNE_SYSTEM
1:226907849	*ITPKB*	REACTOME_INOSITOL_PHOSPHATE_METABOLISM
1:226910340	*ITPKB*	REACTOME_INOSITOL_PHOSPHATE_METABOLISM
1:226916078	*ITPKB*	REACTOME_INOSITOL_PHOSPHATE_METABOLISM
3:48748989	*IP6K2*	REACTOME_INOSITOL_PHOSPHATE_METABOLISM
3:48766111	*IP6K2*	REACTOME_INOSITOL_PHOSPHATE_METABOLISM
8:17161374	*FGF20*	REACTOME_INOSITOL_PHOSPHATE_METABOLISM
8:17176046	*FGF20*	REACTOME_INOSITOL_PHOSPHATE_METABOLISM
8:17243260	*FGF20*	REACTOME_INOSITOL_PHOSPHATE_METABOLISM
8:17260937	*FGF20*	REACTOME_INOSITOL_PHOSPHATE_METABOLISM
17:43195981	*CRHR1_2*	REACTOME_INOSITOL_PHOSPHATE_METABOLISM
3:150298317	*MED12L*	REACTOME_INSERTION_OF_TAIL_ANCHORED_PROTEINS_INTO_THE_ENDOPLASMIC_RETICULUM_MEMBRANE
3:150303590	*MED12L*	REACTOME_INSERTION_OF_TAIL_ANCHORED_PROTEINS_INTO_THE_ENDOPLASMIC_RETICULUM_MEMBRANE
6:31607942	*HLA-DRB5*	REACTOME_INSERTION_OF_TAIL_ANCHORED_PROTEINS_INTO_THE_ENDOPLASMIC_RETICULUM_MEMBRANE
19:2771641	*SPPL2B*	REACTOME_INSERTION_OF_TAIL_ANCHORED_PROTEINS_INTO_THE_ENDOPLASMIC_RETICULUM_MEMBRANE
12:124219791	*HIP1R*	REACTOME_INSULIN_RECEPTOR_RECYCLING
2:18091890	*KCNS3*	REACTOME_INTEGRATION_OF_ENERGY_METABOLISM
2:18130187	*KCNS3*	REACTOME_INTEGRATION_OF_ENERGY_METABOLISM
2:18141542	*KCNS3*	REACTOME_INTEGRATION_OF_ENERGY_METABOLISM
2:18143082	*KCNS3*	REACTOME_INTEGRATION_OF_ENERGY_METABOLISM
2:18176120	*KCNS3*	REACTOME_INTEGRATION_OF_ENERGY_METABOLISM
2:18247777	*KCNS3*	REACTOME_INTEGRATION_OF_ENERGY_METABOLISM
2:18299564	*KCNS3*	REACTOME_INTEGRATION_OF_ENERGY_METABOLISM
2:18315537	*KCNS3*	REACTOME_INTEGRATION_OF_ENERGY_METABOLISM
2:18335515	*KCNS3*	REACTOME_INTEGRATION_OF_ENERGY_METABOLISM
2:18455049	*KCNS3*	REACTOME_INTEGRATION_OF_ENERGY_METABOLISM
2:18472861	*KCNS3*	REACTOME_INTEGRATION_OF_ENERGY_METABOLISM
3:48835245	*IP6K2*	REACTOME_INTEGRATION_OF_ENERGY_METABOLISM
3:123089448	*KPNA1*	REACTOME_INTEGRATION_OF_ENERGY_METABOLISM
5:133558885	*C5orf24*	REACTOME_INTEGRATION_OF_ENERGY_METABOLISM
8:131799767	*FAM49B*	REACTOME_INTEGRATION_OF_ENERGY_METABOLISM

8:131845512	*FAM49B*	REACTOME_INTEGRATION_OF_ENERGY_METABOLISM
8:131865425	*FAM49B*	REACTOME_INTEGRATION_OF_ENERGY_METABOLISM
8:131901170	*FAM49B*	REACTOME_INTEGRATION_OF_ENERGY_METABOLISM
16:50300511	*CASC16*	REACTOME_INTEGRATION_OF_ENERGY_METABOLISM
16:50317039	*CASC16*	REACTOME_INTEGRATION_OF_ENERGY_METABOLISM
19:2557113	*SPPL2B*	REACTOME_INTEGRATION_OF_ENERGY_METABOLISM
19:2563327	*SPPL2B*	REACTOME_INTEGRATION_OF_ENERGY_METABOLISM
19:2580057	*SPPL2B*	REACTOME_INTEGRATION_OF_ENERGY_METABOLISM
19:2660983	*SPPL2B*	REACTOME_INTEGRATION_OF_ENERGY_METABOLISM
19:3142784	*SPPL2B*	REACTOME_INTEGRATION_OF_ENERGY_METABOLISM
19:3147326	*SPPL2B*	REACTOME_INTEGRATION_OF_ENERGY_METABOLISM
3:122200170	*KPNA1*	REACTOME_INTEGRATION_OF_PROVIRUS
10:15562608	*ITGA8*	REACTOME_INTEGRIN_CELL_SURFACE_INTERACTIONS
10:15569598	*ITGA8*	REACTOME_INTEGRIN_CELL_SURFACE_INTERACTIONS
10:15652368	*ITGA8*	REACTOME_INTEGRIN_CELL_SURFACE_INTERACTIONS
11:133947732	*IGSF9B*	REACTOME_INTEGRIN_CELL_SURFACE_INTERACTIONS
16:30510571	*SETD1A*	REACTOME_INTEGRIN_CELL_SURFACE_INTERACTIONS
16:30516492	*SETD1A*	REACTOME_INTEGRIN_CELL_SURFACE_INTERACTIONS
16:30520856	*SETD1A*	REACTOME_INTEGRIN_CELL_SURFACE_INTERACTIONS
16:31370452	*SETD1A*	REACTOME_INTEGRIN_CELL_SURFACE_INTERACTIONS
17:42454243	*FAM171A2*	REACTOME_INTEGRIN_CELL_SURFACE_INTERACTIONS
17:42454243	*FAM171A2*	REACTOME_INTEGRIN_SIGNALING
1:204876966	*RAB29*	REACTOME_INTERACTION_BETWEEN_L1_AND_ANKYRINS
1:204940972	*RAB29*	REACTOME_INTERACTION_BETWEEN_L1_AND_ANKYRINS
4:113947327	*CAMK2D*	REACTOME_INTERACTION_BETWEEN_L1_AND_ANKYRINS
4:114015252	*CAMK2D*	REACTOME_INTERACTION_BETWEEN_L1_AND_ANKYRINS
4:114032570	*CAMK2D*	REACTOME_INTERACTION_BETWEEN_L1_AND_ANKYRINS
4:114067233	*CAMK2D*	REACTOME_INTERACTION_BETWEEN_L1_AND_ANKYRINS
4:114077386	*CAMK2D*	REACTOME_INTERACTION_BETWEEN_L1_AND_ANKYRINS
4:114113772	*CAMK2D*	REACTOME_INTERACTION_BETWEEN_L1_AND_ANKYRINS
4:114122398	*CAMK2D*	REACTOME_INTERACTION_BETWEEN_L1_AND_ANKYRINS
4:114172749	*CAMK2D*	REACTOME_INTERACTION_BETWEEN_L1_AND_ANKYRINS
4:114217997	*CAMK2D*	REACTOME_INTERACTION_BETWEEN_L1_AND_ANKYRINS
3:122200170	*KPNA1*	REACTOME_INTERACTIONS_OF_VPR_WITH_HOST_CELLULAR_PROTEINS
16:30438930	*SETD1A*	REACTOME_INTERCONVERSION_OF_NUCLEOTIDE_DI_AND_TRIPHOSPHATES
3:48748989	*IP6K2*	REACTOME_INTERFERON_ALPHA_BETA_SIGNALING
3:48766111	*IP6K2*	REACTOME_INTERFERON_ALPHA_BETA_SIGNALING
6:29798642	*TRIM40*	REACTOME_INTERFERON_ALPHA_BETA_SIGNALING
17:6667554	*V1NB1*	REACTOME_INTERFERON_ALPHA_BETA_SIGNALING
4:114424939	*CAMK2D*	REACTOME_INTERFERON_GAMMA_SIGNALING
4:114494052	*CAMK2D*	REACTOME_INTERFERON_GAMMA_SIGNALING
4:114529492	*CAMK2D*	REACTOME_INTERFERON_GAMMA_SIGNALING
4:114539964	*CAMK2D*	REACTOME_INTERFERON_GAMMA_SIGNALING
4:114597918	*CAMK2D*	REACTOME_INTERFERON_GAMMA_SIGNALING
6:29798642	*TRIM40*	REACTOME_INTERFERON_GAMMA_SIGNALING
6:30075864	*TRIM40*	REACTOME_INTERFERON_GAMMA_SIGNALING
6:32602372	*HLA-DRB5*	REACTOME_INTERFERON_GAMMA_SIGNALING
3:48748989	*IP6K2*	REACTOME_INTERFERON_SIGNALING
3:48766111	*IP6K2*	REACTOME_INTERFERON_SIGNALING
3:122200170	*KPNA1*	REACTOME_INTERFERON_SIGNALING
4:114424939	*CAMK2D*	REACTOME_INTERFERON_SIGNALING
4:114494052	*CAMK2D*	REACTOME_INTERFERON_SIGNALING
4:114529492	*CAMK2D*	REACTOME_INTERFERON_SIGNALING

4:114539964	*CAMK2D*	REACTOME_INTERFERON_SIGNALING
4:114597918	*CAMK2D*	REACTOME_INTERFERON_SIGNALING
6:29798642	*TRIM40*	REACTOME_INTERFERON_SIGNALING
6:30075864	*TRIM40*	REACTOME_INTERFERON_SIGNALING
6:32602372	*HLA-DRB5*	REACTOME_INTERFERON_SIGNALING
17:6667554	*V1NB1*	REACTOME_INTERFERON_SIGNALING
17:7479140	*V1NB1*	REACTOME_INTERFERON_SIGNALING
17:7480325	*V1NB1*	REACTOME_INTERFERON_SIGNALING
2:102612026	*MAP4K4*	REACTOME_INTERLEUKIN_10_SIGNALING
2:102621855	*MAP4K4*	REACTOME_INTERLEUKIN_10_SIGNALING
2:102631503	*MAP4K4*	REACTOME_INTERLEUKIN_10_SIGNALING
2:102633387	*MAP4K4*	REACTOME_INTERLEUKIN_10_SIGNALING
2:102720323	*MAP4K4*	REACTOME_INTERLEUKIN_10_SIGNALING
2:102783052	*MAP4K4*	REACTOME_INTERLEUKIN_10_SIGNALING
17:40392358	*RETREG3*	REACTOME_INTERLEUKIN_15_SIGNALING
5:133558885	*C5orf24*	REACTOME_INTERLEUKIN_17_SIGNALING
10:103185542	*GBF1*	REACTOME_INTERLEUKIN_17_SIGNALING
10:103305087	*GBF1*	REACTOME_INTERLEUKIN_17_SIGNALING
10:103308244	*GBF1*	REACTOME_INTERLEUKIN_17_SIGNALING
16:50730088	*CASC16*	REACTOME_INTERLEUKIN_17_SIGNALING
16:50730446	*CASC16*	REACTOME_INTERLEUKIN_17_SIGNALING
16:50745926	*CASC16*	REACTOME_INTERLEUKIN_17_SIGNALING
17:41845031	*FAM171A2*	REACTOME_INTERLEUKIN_17_SIGNALING
2:103047986	*MAP4K4*	REACTOME_INTERLEUKIN_18_SIGNALING
2:103063855	*MAP4K4*	REACTOME_INTERLEUKIN_18_SIGNALING
2:103066346	*MAP4K4*	REACTOME_INTERLEUKIN_18_SIGNALING
2:102612026	*MAP4K4*	REACTOME_INTERLEUKIN_1_FAMILY_SIGNALING
2:102621855	*MAP4K4*	REACTOME_INTERLEUKIN_1_FAMILY_SIGNALING
2:102631503	*MAP4K4*	REACTOME_INTERLEUKIN_1_FAMILY_SIGNALING
2:102633387	*MAP4K4*	REACTOME_INTERLEUKIN_1_FAMILY_SIGNALING
2:102720323	*MAP4K4*	REACTOME_INTERLEUKIN_1_FAMILY_SIGNALING
2:102783052	*MAP4K4*	REACTOME_INTERLEUKIN_1_FAMILY_SIGNALING
2:102826146	*MAP4K4*	REACTOME_INTERLEUKIN_1_FAMILY_SIGNALING
2:103047986	*MAP4K4*	REACTOME_INTERLEUKIN_1_FAMILY_SIGNALING
2:103063855	*MAP4K4*	REACTOME_INTERLEUKIN_1_FAMILY_SIGNALING
2:103066346	*MAP4K4*	REACTOME_INTERLEUKIN_1_FAMILY_SIGNALING
6:32151443	*HLA-DRB5*	REACTOME_INTERLEUKIN_1_FAMILY_SIGNALING
10:103185542	*GBF1*	REACTOME_INTERLEUKIN_1_FAMILY_SIGNALING
10:103305087	*GBF1*	REACTOME_INTERLEUKIN_1_FAMILY_SIGNALING
10:103308244	*GBF1*	REACTOME_INTERLEUKIN_1_FAMILY_SIGNALING
16:50730088	*CASC16*	REACTOME_INTERLEUKIN_1_FAMILY_SIGNALING
16:50730446	*CASC16*	REACTOME_INTERLEUKIN_1_FAMILY_SIGNALING
16:50745926	*CASC16*	REACTOME_INTERLEUKIN_1_FAMILY_SIGNALING
2:102612026	*MAP4K4*	REACTOME_INTERLEUKIN_1_SIGNALING
2:102621855	*MAP4K4*	REACTOME_INTERLEUKIN_1_SIGNALING
2:102631503	*MAP4K4*	REACTOME_INTERLEUKIN_1_SIGNALING
2:102633387	*MAP4K4*	REACTOME_INTERLEUKIN_1_SIGNALING
2:102720323	*MAP4K4*	REACTOME_INTERLEUKIN_1_SIGNALING
2:102783052	*MAP4K4*	REACTOME_INTERLEUKIN_1_SIGNALING
6:32151443	*HLA-DRB5*	REACTOME_INTERLEUKIN_1_SIGNALING
10:103185542	*GBF1*	REACTOME_INTERLEUKIN_1_SIGNALING
10:103305087	*GBF1*	REACTOME_INTERLEUKIN_1_SIGNALING
10:103308244	*GBF1*	REACTOME_INTERLEUKIN_1_SIGNALING

16:50730088	*CASC16*	REACTOME_INTERLEUKIN_1_SIGNALING
16:50730446	*CASC16*	REACTOME_INTERLEUKIN_1_SIGNALING
16:50745926	*CASC16*	REACTOME_INTERLEUKIN_1_SIGNALING
17:40392358	*RETREG3*	REACTOME_INTERLEUKIN_20_FAMILY_SIGNALING
17:40392358	*RETREG3*	REACTOME_INTERLEUKIN_21_SIGNALING
17:40392358	*RETREG3*	REACTOME_INTERLEUKIN_2_FAMILY_SIGNALING
17:40392358	*RETREG3*	REACTOME_INTERLEUKIN_2_SIGNALING
2:102826146	*MAP4K4*	REACTOME_INTERLEUKIN_36_PATHWAY
6:112137847	*FYN*	REACTOME_INTERLEUKIN_3_INTERLEUKIN_5_AND_GM_CSF_SIGNALING
6:112151452	*FYN*	REACTOME_INTERLEUKIN_3_INTERLEUKIN_5_AND_GM_CSF_SIGNALING
6:112175069	*FYN*	REACTOME_INTERLEUKIN_3_INTERLEUKIN_5_AND_GM_CSF_SIGNALING
17:40392358	*RETREG3*	REACTOME_INTERLEUKIN_3_INTERLEUKIN_5_AND_GM_CSF_SIGNALING
15:61016235	*VPS13C*	REACTOME_INTERLEUKIN_4_AND_INTERLEUKIN_13_SIGNALING
15:61065568	*VPS13C*	REACTOME_INTERLEUKIN_4_AND_INTERLEUKIN_13_SIGNALING
15:61094298	*VPS13C*	REACTOME_INTERLEUKIN_4_AND_INTERLEUKIN_13_SIGNALING
15:61140736	*VPS13C*	REACTOME_INTERLEUKIN_4_AND_INTERLEUKIN_13_SIGNALING
15:61142062	*VPS13C*	REACTOME_INTERLEUKIN_4_AND_INTERLEUKIN_13_SIGNALING
15:61162571	*VPS13C*	REACTOME_INTERLEUKIN_4_AND_INTERLEUKIN_13_SIGNALING
15:61191617	*VPS13C*	REACTOME_INTERLEUKIN_4_AND_INTERLEUKIN_13_SIGNALING
15:61195427	*VPS13C*	REACTOME_INTERLEUKIN_4_AND_INTERLEUKIN_13_SIGNALING
15:61221682	*VPS13C*	REACTOME_INTERLEUKIN_4_AND_INTERLEUKIN_13_SIGNALING
15:61298486	*VPS13C*	REACTOME_INTERLEUKIN_4_AND_INTERLEUKIN_13_SIGNALING
15:61375480	*VPS13C*	REACTOME_INTERLEUKIN_4_AND_INTERLEUKIN_13_SIGNALING
15:61434581	*VPS13C*	REACTOME_INTERLEUKIN_4_AND_INTERLEUKIN_13_SIGNALING
15:61467078	*VPS13C*	REACTOME_INTERLEUKIN_4_AND_INTERLEUKIN_13_SIGNALING
15:61470808	*VPS13C*	REACTOME_INTERLEUKIN_4_AND_INTERLEUKIN_13_SIGNALING
15:61500263	*VPS13C*	REACTOME_INTERLEUKIN_4_AND_INTERLEUKIN_13_SIGNALING
16:31370452	*SETD1A*	REACTOME_INTERLEUKIN_4_AND_INTERLEUKIN_13_SIGNALING
17:7578671	*V1NB1*	REACTOME_INTERLEUKIN_4_AND_INTERLEUKIN_13_SIGNALING
17:40392358	*RETREG3*	REACTOME_INTERLEUKIN_7_SIGNALING
17:40392358	*RETREG3*	REACTOME_INTERLEUKIN_9_SIGNALING
3:48835245	*IP6K2*	REACTOME_INTRACELLULAR_SIGNALING_BY_SECOND_MESSENGERS
3:123089448	*KPNA1*	REACTOME_INTRACELLULAR_SIGNALING_BY_SECOND_MESSENGERS
4:114424939	*CAMK2D*	REACTOME_INTRACELLULAR_SIGNALING_BY_SECOND_MESSENGERS
4:114494052	*CAMK2D*	REACTOME_INTRACELLULAR_SIGNALING_BY_SECOND_MESSENGERS
4:114529492	*CAMK2D*	REACTOME_INTRACELLULAR_SIGNALING_BY_SECOND_MESSENGERS
4:114539964	*CAMK2D*	REACTOME_INTRACELLULAR_SIGNALING_BY_SECOND_MESSENGERS
4:114597918	*CAMK2D*	REACTOME_INTRACELLULAR_SIGNALING_BY_SECOND_MESSENGERS
5:133558885	*C5orf24*	REACTOME_INTRACELLULAR_SIGNALING_BY_SECOND_MESSENGERS
6:112137847	*FYN*	REACTOME_INTRACELLULAR_SIGNALING_BY_SECOND_MESSENGERS
6:112151452	*FYN*	REACTOME_INTRACELLULAR_SIGNALING_BY_SECOND_MESSENGERS
6:112175069	*FYN*	REACTOME_INTRACELLULAR_SIGNALING_BY_SECOND_MESSENGERS
8:131799767	*FAM49B*	REACTOME_INTRACELLULAR_SIGNALING_BY_SECOND_MESSENGERS
8:131845512	*FAM49B*	REACTOME_INTRACELLULAR_SIGNALING_BY_SECOND_MESSENGERS
8:131865425	*FAM49B*	REACTOME_INTRACELLULAR_SIGNALING_BY_SECOND_MESSENGERS
8:131901170	*FAM49B*	REACTOME_INTRACELLULAR_SIGNALING_BY_SECOND_MESSENGERS
16:50300511	*CASC16*	REACTOME_INTRACELLULAR_SIGNALING_BY_SECOND_MESSENGERS
16:50317039	*CASC16*	REACTOME_INTRACELLULAR_SIGNALING_BY_SECOND_MESSENGERS
17:7578671	*V1NB1*	REACTOME_INTRACELLULAR_SIGNALING_BY_SECOND_MESSENGERS
1:171689486	*VAMP4*	REACTOME_INTRA_GOLGI_AND_RETROGRADE_GOLGI_TO_ER_TRAFFIC
2:135921205	*TMEM163*	REACTOME_INTRA_GOLGI_AND_RETROGRADE_GOLGI_TO_ER_TRAFFIC
10:104005410	*GBF1*	REACTOME_INTRA_GOLGI_AND_RETROGRADE_GOLGI_TO_ER_TRAFFIC
10:104034550	*GBF1*	REACTOME_INTRA_GOLGI_AND_RETROGRADE_GOLGI_TO_ER_TRAFFIC

10:104258303	*GBF1*	REACTOME_INTRA_GOLGI_AND_RETROGRADE_GOLGI_TO_ER_TRAFFIC
11:82719121	*DLG2*	REACTOME_INTRA_GOLGI_AND_RETROGRADE_GOLGI_TO_ER_TRAFFIC
12:39759090	*LRRK2_2*	REACTOME_INTRA_GOLGI_AND_RETROGRADE_GOLGI_TO_ER_TRAFFIC
12:39826709	*LRRK2_2*	REACTOME_INTRA_GOLGI_AND_RETROGRADE_GOLGI_TO_ER_TRAFFIC
17:43017194	*CRHR1_2*	REACTOME_INTRA_GOLGI_AND_RETROGRADE_GOLGI_TO_ER_TRAFFIC
17:43021962	*CRHR1_2*	REACTOME_INTRA_GOLGI_AND_RETROGRADE_GOLGI_TO_ER_TRAFFIC
17:44800946	*WNT3*	REACTOME_INTRA_GOLGI_AND_RETROGRADE_GOLGI_TO_ER_TRAFFIC
17:44833217	*WNT3*	REACTOME_INTRA_GOLGI_AND_RETROGRADE_GOLGI_TO_ER_TRAFFIC
17:45020426	*WNT3*	REACTOME_INTRA_GOLGI_AND_RETROGRADE_GOLGI_TO_ER_TRAFFIC
17:45059032	*WNT3*	REACTOME_INTRA_GOLGI_AND_RETROGRADE_GOLGI_TO_ER_TRAFFIC
17:45089680	*WNT3*	REACTOME_INTRA_GOLGI_AND_RETROGRADE_GOLGI_TO_ER_TRAFFIC
17:76727455	*DNAH17*	REACTOME_INTRA_GOLGI_AND_RETROGRADE_GOLGI_TO_ER_TRAFFIC
11:82719121	*DLG2*	REACTOME_INTRA_GOLGI_TRAFFIC
17:44800946	*WNT3*	REACTOME_INTRA_GOLGI_TRAFFIC
17:44833217	*WNT3*	REACTOME_INTRA_GOLGI_TRAFFIC
17:45020426	*WNT3*	REACTOME_INTRA_GOLGI_TRAFFIC
17:45059032	*WNT3*	REACTOME_INTRA_GOLGI_TRAFFIC
17:45089680	*WNT3*	REACTOME_INTRA_GOLGI_TRAFFIC
17:76727455	*DNAH17*	REACTOME_INTRA_GOLGI_TRAFFIC
17:7578671	*V1NB1*	REACTOME_INTRINSIC_PATHWAY_FOR_APOPTOSIS
17:43181287	*CRHR1_2*	REACTOME_INTRINSIC_PATHWAY_FOR_APOPTOSIS
17:43184627	*CRHR1_2*	REACTOME_INTRINSIC_PATHWAY_FOR_APOPTOSIS
11:82582134	*DLG2*	REACTOME_INTRINSIC_PATHWAY_OF_FIBRIN_CLOT_FORMATION
11:82635123	*DLG2*	REACTOME_INTRINSIC_PATHWAY_OF_FIBRIN_CLOT_FORMATION
11:82648357	*DLG2*	REACTOME_INTRINSIC_PATHWAY_OF_FIBRIN_CLOT_FORMATION
6:29565889	*TRIM40*	REACTOME_INWARDLY_RECTIFYING_KPLUS_CHANNELS
6:29590715	*TRIM40*	REACTOME_INWARDLY_RECTIFYING_KPLUS_CHANNELS
19:2557113	*SPPL2B*	REACTOME_INWARDLY_RECTIFYING_KPLUS_CHANNELS
19:2563327	*SPPL2B*	REACTOME_INWARDLY_RECTIFYING_KPLUS_CHANNELS
19:2580057	*SPPL2B*	REACTOME_INWARDLY_RECTIFYING_KPLUS_CHANNELS
19:2660983	*SPPL2B*	REACTOME_INWARDLY_RECTIFYING_KPLUS_CHANNELS
21:38984964	*DYRK1A*	REACTOME_INWARDLY_RECTIFYING_KPLUS_CHANNELS
21:39018136	*DYRK1A*	REACTOME_INWARDLY_RECTIFYING_KPLUS_CHANNELS
21:39021814	*DYRK1A*	REACTOME_INWARDLY_RECTIFYING_KPLUS_CHANNELS
21:39039956	*DYRK1A*	REACTOME_INWARDLY_RECTIFYING_KPLUS_CHANNELS
21:39088785	*DYRK1A*	REACTOME_INWARDLY_RECTIFYING_KPLUS_CHANNELS
21:39108160	*DYRK1A*	REACTOME_INWARDLY_RECTIFYING_KPLUS_CHANNELS
21:39120480	*DYRK1A*	REACTOME_INWARDLY_RECTIFYING_KPLUS_CHANNELS
21:39134980	*DYRK1A*	REACTOME_INWARDLY_RECTIFYING_KPLUS_CHANNELS
21:39197323	*DYRK1A*	REACTOME_INWARDLY_RECTIFYING_KPLUS_CHANNELS
21:39217472	*DYRK1A*	REACTOME_INWARDLY_RECTIFYING_KPLUS_CHANNELS
1:154306180	*GBAP1*	REACTOME_ION_CHANNEL_TRANSPORT
4:114424939	*CAMK2D*	REACTOME_ION_CHANNEL_TRANSPORT
4:114494052	*CAMK2D*	REACTOME_ION_CHANNEL_TRANSPORT
4:114529492	*CAMK2D*	REACTOME_ION_CHANNEL_TRANSPORT
4:114539964	*CAMK2D*	REACTOME_ION_CHANNEL_TRANSPORT
4:114597918	*CAMK2D*	REACTOME_ION_CHANNEL_TRANSPORT
4:170563153	*CLCN3*	REACTOME_ION_CHANNEL_TRANSPORT
4:170569172	*CLCN3*	REACTOME_ION_CHANNEL_TRANSPORT
4:170620812	*CLCN3*	REACTOME_ION_CHANNEL_TRANSPORT
12:45755099	*SCAF11*	REACTOME_ION_CHANNEL_TRANSPORT
12:124219791	*HIP1R*	REACTOME_ION_CHANNEL_TRANSPORT
17:7560294	*V1NB1*	REACTOME_ION_CHANNEL_TRANSPORT

17:40938261	*RETREG3*	REACTOME_ION_CHANNEL_TRANSPORT
19:1811130	*SPPL2B*	REACTOME_ION_CHANNEL_TRANSPORT
4:114424939	*CAMK2D*	REACTOME_ION_HOMEOSTASIS
4:114494052	*CAMK2D*	REACTOME_ION_HOMEOSTASIS
4:114529492	*CAMK2D*	REACTOME_ION_HOMEOSTASIS
4:114539964	*CAMK2D*	REACTOME_ION_HOMEOSTASIS
4:114597918	*CAMK2D*	REACTOME_ION_HOMEOSTASIS
17:7560294	*V1NB1*	REACTOME_ION_HOMEOSTASIS
17:7101771	*V1NB1*	REACTOME_IONOTROPIC_ACTIVITY_OF_KAINATE_RECEPTORS
1:154306180	*GBAP1*	REACTOME_ION_TRANSPORT_BY_P_TYPE_ATPASES
4:114424939	*CAMK2D*	REACTOME_ION_TRANSPORT_BY_P_TYPE_ATPASES
4:114494052	*CAMK2D*	REACTOME_ION_TRANSPORT_BY_P_TYPE_ATPASES
4:114529492	*CAMK2D*	REACTOME_ION_TRANSPORT_BY_P_TYPE_ATPASES
4:114539964	*CAMK2D*	REACTOME_ION_TRANSPORT_BY_P_TYPE_ATPASES
4:114597918	*CAMK2D*	REACTOME_ION_TRANSPORT_BY_P_TYPE_ATPASES
17:7560294	*V1NB1*	REACTOME_ION_TRANSPORT_BY_P_TYPE_ATPASES
19:1811130	*SPPL2B*	REACTOME_ION_TRANSPORT_BY_P_TYPE_ATPASES
1:156059276	*GBAP1*	REACTOME_IRE1ALPHA_ACTIVATES_CHAPERONES
3:122841099	*KPNA1*	REACTOME_IRE1ALPHA_ACTIVATES_CHAPERONES
3:122859868	*KPNA1*	REACTOME_IRE1ALPHA_ACTIVATES_CHAPERONES
3:122898729	*KPNA1*	REACTOME_IRE1ALPHA_ACTIVATES_CHAPERONES
3:150298317	*MED12L*	REACTOME_IRE1ALPHA_ACTIVATES_CHAPERONES
3:150303590	*MED12L*	REACTOME_IRE1ALPHA_ACTIVATES_CHAPERONES
17:45020426	*WNT3*	REACTOME_IRE1ALPHA_ACTIVATES_CHAPERONES
17:45059032	*WNT3*	REACTOME_IRE1ALPHA_ACTIVATES_CHAPERONES
17:45089680	*WNT3*	REACTOME_IRE1ALPHA_ACTIVATES_CHAPERONES
4:15608677	*BST1*	REACTOME_IRON_UPTAKE_AND_TRANSPORT
4:15618898	*BST1*	REACTOME_IRON_UPTAKE_AND_TRANSPORT
12:124219791	*HIP1R*	REACTOME_IRON_UPTAKE_AND_TRANSPORT
16:50730088	*CASC16*	REACTOME_JNK_C_JUN_KINASES_PHOSPHORYLATION_AND_ACTIVATION_MEDIATED_BY_ACTIVATED_HUMAN_TAK1
16:50730446	*CASC16*	REACTOME_JNK_C_JUN_KINASES_PHOSPHORYLATION_AND_ACTIVATION_MEDIATED_BY_ACTIVATED_HUMAN_TAK1
16:50745926	*CASC16*	REACTOME_JNK_C_JUN_KINASES_PHOSPHORYLATION_AND_ACTIVATION_MEDIATED_BY_ACTIVATED_HUMAN_TAK1
17:39883520	*RETREG3*	REACTOME_KERATINIZATION
12:39759090	*LRRK2_2*	REACTOME_KINESINS
12:39826709	*LRRK2_2*	REACTOME_KINESINS
17:43017194	*CRHR1_2*	REACTOME_KINESINS
17:43021962	*CRHR1_2*	REACTOME_KINESINS
1:171837985	*VAMP4*	REACTOME_L1CAM_INTERACTIONS
1:171945488	*VAMP4*	REACTOME_L1CAM_INTERACTIONS
1:172137119	*VAMP4*	REACTOME_L1CAM_INTERACTIONS
1:172386261	*VAMP4*	REACTOME_L1CAM_INTERACTIONS
1:204876966	*RAB29*	REACTOME_L1CAM_INTERACTIONS
1:204940972	*RAB29*	REACTOME_L1CAM_INTERACTIONS
4:113947327	*CAMK2D*	REACTOME_L1CAM_INTERACTIONS
4:114015252	*CAMK2D*	REACTOME_L1CAM_INTERACTIONS
4:114032570	*CAMK2D*	REACTOME_L1CAM_INTERACTIONS
4:114067233	*CAMK2D*	REACTOME_L1CAM_INTERACTIONS
4:114077386	*CAMK2D*	REACTOME_L1CAM_INTERACTIONS
4:114113772	*CAMK2D*	REACTOME_L1CAM_INTERACTIONS
4:114122398	*CAMK2D*	REACTOME_L1CAM_INTERACTIONS
4:114172749	*CAMK2D*	REACTOME_L1CAM_INTERACTIONS
4:114217997	*CAMK2D*	REACTOME_L1CAM_INTERACTIONS
9:17579690	*SH3GL2_2*	REACTOME_L1CAM_INTERACTIONS

9:17587160	*SH3GL2_2*	REACTOME_L1CAM_INTERACTIONS
9:17609302	*SH3GL2_2*	REACTOME_L1CAM_INTERACTIONS
9:17626762	*SH3GL2_2*	REACTOME_L1CAM_INTERACTIONS
9:17627444	*SH3GL2_2*	REACTOME_L1CAM_INTERACTIONS
9:17642664	*SH3GL2_2*	REACTOME_L1CAM_INTERACTIONS
9:17710876	*SH3GL2_2*	REACTOME_L1CAM_INTERACTIONS
9:17732590	*SH3GL2_2*	REACTOME_L1CAM_INTERACTIONS
9:17737514	*SH3GL2_2*	REACTOME_L1CAM_INTERACTIONS
9:17758945	*SH3GL2_2*	REACTOME_L1CAM_INTERACTIONS
12:41144387	*LRRK2_2*	REACTOME_L1CAM_INTERACTIONS
12:41147295	*LRRK2_2*	REACTOME_L1CAM_INTERACTIONS
12:41195135	*LRRK2_2*	REACTOME_L1CAM_INTERACTIONS
12:41251554	*LRRK2_2*	REACTOME_L1CAM_INTERACTIONS
12:41255129	*LRRK2_2*	REACTOME_L1CAM_INTERACTIONS
12:41297969	*LRRK2_2*	REACTOME_L1CAM_INTERACTIONS
12:41407403	*LRRK2_2*	REACTOME_L1CAM_INTERACTIONS
17:7101771	*V1NB1*	REACTOME_L1CAM_INTERACTIONS
17:42454243	*FAM171A2*	REACTOME_L1CAM_INTERACTIONS
3:49162284	*IP6K2*	REACTOME_LAMININ_INTERACTIONS
3:49162583	*IP6K2*	REACTOME_LAMININ_INTERACTIONS
6:112494762	*FYN*	REACTOME_LAMININ_INTERACTIONS
6:112531401	*FYN*	REACTOME_LAMININ_INTERACTIONS
6:112561154	*FYN*	REACTOME_LAMININ_INTERACTIONS
6:112561430	*FYN*	REACTOME_LAMININ_INTERACTIONS
8:22007293	*BIN3*	REACTOME_LGI_ADAM_INTERACTIONS
16:31004169	*SETD1A*	REACTOME_LGI_ADAM_INTERACTIONS
17:7101771	*V1NB1*	REACTOME_LGI_ADAM_INTERACTIONS
17:42855931	*CRHR1_2*	REACTOME_LGI_ADAM_INTERACTIONS
9:17579690	*SH3GL2_2*	REACTOME_LISTERIA_MONOCYTOGENES_ENTRY_INTO_HOST_CELLS
9:17587160	*SH3GL2_2*	REACTOME_LISTERIA_MONOCYTOGENES_ENTRY_INTO_HOST_CELLS
9:17609302	*SH3GL2_2*	REACTOME_LISTERIA_MONOCYTOGENES_ENTRY_INTO_HOST_CELLS
9:17626762	*SH3GL2_2*	REACTOME_LISTERIA_MONOCYTOGENES_ENTRY_INTO_HOST_CELLS
9:17627444	*SH3GL2_2*	REACTOME_LISTERIA_MONOCYTOGENES_ENTRY_INTO_HOST_CELLS
9:17642664	*SH3GL2_2*	REACTOME_LISTERIA_MONOCYTOGENES_ENTRY_INTO_HOST_CELLS
9:17710876	*SH3GL2_2*	REACTOME_LISTERIA_MONOCYTOGENES_ENTRY_INTO_HOST_CELLS
9:17732590	*SH3GL2_2*	REACTOME_LISTERIA_MONOCYTOGENES_ENTRY_INTO_HOST_CELLS
9:17737514	*SH3GL2_2*	REACTOME_LISTERIA_MONOCYTOGENES_ENTRY_INTO_HOST_CELLS
9:17758945	*SH3GL2_2*	REACTOME_LISTERIA_MONOCYTOGENES_ENTRY_INTO_HOST_CELLS
4:114424939	*CAMK2D*	REACTOME_LONG_TERM_POTENTIATION
4:114494052	*CAMK2D*	REACTOME_LONG_TERM_POTENTIATION
4:114529492	*CAMK2D*	REACTOME_LONG_TERM_POTENTIATION
4:114539964	*CAMK2D*	REACTOME_LONG_TERM_POTENTIATION
4:114597918	*CAMK2D*	REACTOME_LONG_TERM_POTENTIATION
11:83219006	*DLG2*	REACTOME_LONG_TERM_POTENTIATION
11:83232915	*DLG2*	REACTOME_LONG_TERM_POTENTIATION
11:83282740	*DLG2*	REACTOME_LONG_TERM_POTENTIATION
11:83387273	*DLG2*	REACTOME_LONG_TERM_POTENTIATION
11:83538733	*DLG2*	REACTOME_LONG_TERM_POTENTIATION
11:83544472	*DLG2*	REACTOME_LONG_TERM_POTENTIATION
11:83578152	*DLG2*	REACTOME_LONG_TERM_POTENTIATION
11:83647657	*DLG2*	REACTOME_LONG_TERM_POTENTIATION
11:83669903	*DLG2*	REACTOME_LONG_TERM_POTENTIATION
11:83736246	*DLG2*	REACTOME_LONG_TERM_POTENTIATION

11:83762918	*DLG2*	REACTOME_LONG_TERM_POTENTIATION
11:83823752	*DLG2*	REACTOME_LONG_TERM_POTENTIATION
11:83839992	*DLG2*	REACTOME_LONG_TERM_POTENTIATION
11:84104763	*DLG2*	REACTOME_LONG_TERM_POTENTIATION
11:84169039	*DLG2*	REACTOME_LONG_TERM_POTENTIATION
11:84221628	*DLG2*	REACTOME_LONG_TERM_POTENTIATION
11:84312008	*DLG2*	REACTOME_LONG_TERM_POTENTIATION
11:84324361	*DLG2*	REACTOME_LONG_TERM_POTENTIATION
11:84360346	*DLG2*	REACTOME_LONG_TERM_POTENTIATION
11:84403409	*DLG2*	REACTOME_LONG_TERM_POTENTIATION
17:7101771	*V1NB1*	REACTOME_LONG_TERM_POTENTIATION
14:37176333	*MIPOL1*	REACTOME_LYSINE_CATABOLISM
14:37176342	*MIPOL1*	REACTOME_LYSINE_CATABOLISM
14:37226055	*MIPOL1*	REACTOME_LYSINE_CATABOLISM
14:37268679	*MIPOL1*	REACTOME_LYSINE_CATABOLISM
14:37354184	*MIPOL1*	REACTOME_LYSINE_CATABOLISM
14:37385108	*MIPOL1*	REACTOME_LYSINE_CATABOLISM
14:37445399	*MIPOL1*	REACTOME_LYSINE_CATABOLISM
14:37472311	*MIPOL1*	REACTOME_LYSINE_CATABOLISM
9:17579690	*SH3GL2_2*	REACTOME_LYSOSOME_VESICLE_BIOGENESIS
9:17587160	*SH3GL2_2*	REACTOME_LYSOSOME_VESICLE_BIOGENESIS
9:17609302	*SH3GL2_2*	REACTOME_LYSOSOME_VESICLE_BIOGENESIS
9:17626762	*SH3GL2_2*	REACTOME_LYSOSOME_VESICLE_BIOGENESIS
9:17627444	*SH3GL2_2*	REACTOME_LYSOSOME_VESICLE_BIOGENESIS
9:17642664	*SH3GL2_2*	REACTOME_LYSOSOME_VESICLE_BIOGENESIS
9:17710876	*SH3GL2_2*	REACTOME_LYSOSOME_VESICLE_BIOGENESIS
9:17732590	*SH3GL2_2*	REACTOME_LYSOSOME_VESICLE_BIOGENESIS
9:17737514	*SH3GL2_2*	REACTOME_LYSOSOME_VESICLE_BIOGENESIS
9:17758945	*SH3GL2_2*	REACTOME_LYSOSOME_VESICLE_BIOGENESIS
12:133295501	*FBRSL1*	REACTOME_MACROAUTOPHAGY
14:55871532	*GCH1*	REACTOME_MACROAUTOPHAGY
17:42454243	*FAM171A2*	REACTOME_MAP2K_AND_MAPK_ACTIVATION
10:103185542	*GBF1*	REACTOME_MAP3K8_TPL2_DEPENDENT_MAPK1_3_ACTIVATION
10:103305087	*GBF1*	REACTOME_MAP3K8_TPL2_DEPENDENT_MAPK1_3_ACTIVATION
10:103308244	*GBF1*	REACTOME_MAP3K8_TPL2_DEPENDENT_MAPK1_3_ACTIVATION
18:48228968	*MEX3C*	REACTOME_MAPK6_MAPK4_SIGNALING
4:114424939	*CAMK2D*	REACTOME_MAPK_FAMILY_SIGNALING_CASCADES
4:114494052	*CAMK2D*	REACTOME_MAPK_FAMILY_SIGNALING_CASCADES
4:114529492	*CAMK2D*	REACTOME_MAPK_FAMILY_SIGNALING_CASCADES
4:114539964	*CAMK2D*	REACTOME_MAPK_FAMILY_SIGNALING_CASCADES
4:114597918	*CAMK2D*	REACTOME_MAPK_FAMILY_SIGNALING_CASCADES
5:133558885	*C5orf24*	REACTOME_MAPK_FAMILY_SIGNALING_CASCADES
6:112137847	*FYN*	REACTOME_MAPK_FAMILY_SIGNALING_CASCADES
6:112151452	*FYN*	REACTOME_MAPK_FAMILY_SIGNALING_CASCADES
6:112175069	*FYN*	REACTOME_MAPK_FAMILY_SIGNALING_CASCADES
8:21565088	*BIN3*	REACTOME_MAPK_FAMILY_SIGNALING_CASCADES
8:21582921	*BIN3*	REACTOME_MAPK_FAMILY_SIGNALING_CASCADES
8:21665306	*BIN3*	REACTOME_MAPK_FAMILY_SIGNALING_CASCADES
8:21668901	*BIN3*	REACTOME_MAPK_FAMILY_SIGNALING_CASCADES
11:83219006	*DLG2*	REACTOME_MAPK_FAMILY_SIGNALING_CASCADES
11:83232915	*DLG2*	REACTOME_MAPK_FAMILY_SIGNALING_CASCADES
11:83282740	*DLG2*	REACTOME_MAPK_FAMILY_SIGNALING_CASCADES
11:83387273	*DLG2*	REACTOME_MAPK_FAMILY_SIGNALING_CASCADES

11:83538733	*DLG2*	REACTOME_MAPK_FAMILY_SIGNALING_CASCADES
11:83544472	*DLG2*	REACTOME_MAPK_FAMILY_SIGNALING_CASCADES
11:83578152	*DLG2*	REACTOME_MAPK_FAMILY_SIGNALING_CASCADES
11:83647657	*DLG2*	REACTOME_MAPK_FAMILY_SIGNALING_CASCADES
11:83669903	*DLG2*	REACTOME_MAPK_FAMILY_SIGNALING_CASCADES
11:83736246	*DLG2*	REACTOME_MAPK_FAMILY_SIGNALING_CASCADES
11:83762918	*DLG2*	REACTOME_MAPK_FAMILY_SIGNALING_CASCADES
11:83823752	*DLG2*	REACTOME_MAPK_FAMILY_SIGNALING_CASCADES
11:83839992	*DLG2*	REACTOME_MAPK_FAMILY_SIGNALING_CASCADES
11:84104763	*DLG2*	REACTOME_MAPK_FAMILY_SIGNALING_CASCADES
11:84169039	*DLG2*	REACTOME_MAPK_FAMILY_SIGNALING_CASCADES
11:84221628	*DLG2*	REACTOME_MAPK_FAMILY_SIGNALING_CASCADES
11:84312008	*DLG2*	REACTOME_MAPK_FAMILY_SIGNALING_CASCADES
11:84324361	*DLG2*	REACTOME_MAPK_FAMILY_SIGNALING_CASCADES
11:84360346	*DLG2*	REACTOME_MAPK_FAMILY_SIGNALING_CASCADES
11:84403409	*DLG2*	REACTOME_MAPK_FAMILY_SIGNALING_CASCADES
17:7101771	*V1NB1*	REACTOME_MAPK_FAMILY_SIGNALING_CASCADES
17:42454243	*FAM171A2*	REACTOME_MAPK_FAMILY_SIGNALING_CASCADES
18:48228968	*MEX3C*	REACTOME_MAPK_FAMILY_SIGNALING_CASCADES
5:133558885	*C5orf24*	REACTOME_MAPK_TARGETS_NUCLEAR_EVENTS_MEDIATED_BY_MAP_KINASES
17:41845031	*FAM171A2*	REACTOME_MAPK_TARGETS_NUCLEAR_EVENTS_MEDIATED_BY_MAP_KINASES
5:133558885	*C5orf24*	REACTOME_MASTL_FACILITATES_MITOTIC_PROGRESSION
1:156059276	*GBAP1*	REACTOME_MEIOSIS
6:31716436	*HLA-DRB5*	REACTOME_MEIOSIS
14:75495059	*RPS6KL1*	REACTOME_MEIOSIS
6:31716436	*HLA-DRB5*	REACTOME_MEIOTIC_RECOMBINATION
14:75495059	*RPS6KL1*	REACTOME_MEIOTIC_RECOMBINATION
1:156059276	*GBAP1*	REACTOME_MEIOTIC_SYNAPSIS
10:104591639	*GBF1*	REACTOME_METABOLIC_DISORDERS_OF_BIOLOGICAL_OXIDATION_ENZYMES
1:226108200	*ITPKB*	REACTOME_METABOLISM_OF_AMINO_ACIDS_AND_DERIVATIVES
3:182760073	*MCCC1*	REACTOME_METABOLISM_OF_AMINO_ACIDS_AND_DERIVATIVES
4:17465704	*LCORL*	REACTOME_METABOLISM_OF_AMINO_ACIDS_AND_DERIVATIVES
4:17503839	*LCORL*	REACTOME_METABOLISM_OF_AMINO_ACIDS_AND_DERIVATIVES
11:134130174	*IGSF9B*	REACTOME_METABOLISM_OF_AMINO_ACIDS_AND_DERIVATIVES
12:133295501	*FBRSL1*	REACTOME_METABOLISM_OF_AMINO_ACIDS_AND_DERIVATIVES
14:37176333	*MIPOL1*	REACTOME_METABOLISM_OF_AMINO_ACIDS_AND_DERIVATIVES
14:37176342	*MIPOL1*	REACTOME_METABOLISM_OF_AMINO_ACIDS_AND_DERIVATIVES
14:37226055	*MIPOL1*	REACTOME_METABOLISM_OF_AMINO_ACIDS_AND_DERIVATIVES
14:37268679	*MIPOL1*	REACTOME_METABOLISM_OF_AMINO_ACIDS_AND_DERIVATIVES
14:37354184	*MIPOL1*	REACTOME_METABOLISM_OF_AMINO_ACIDS_AND_DERIVATIVES
14:37385108	*MIPOL1*	REACTOME_METABOLISM_OF_AMINO_ACIDS_AND_DERIVATIVES
14:37445399	*MIPOL1*	REACTOME_METABOLISM_OF_AMINO_ACIDS_AND_DERIVATIVES
14:37472311	*MIPOL1*	REACTOME_METABOLISM_OF_AMINO_ACIDS_AND_DERIVATIVES
2:168682052	*STK39*	REACTOME_METABOLISM_OF_CARBOHYDRATES
5:133558885	*C5orf24*	REACTOME_METABOLISM_OF_CARBOHYDRATES
6:71594785	*RIMS1*	REACTOME_METABOLISM_OF_CARBOHYDRATES
7:65425894	*GS1-124K5.11*	REACTOME_METABOLISM_OF_CARBOHYDRATES
11:134255325	*IGSF9B*	REACTOME_METABOLISM_OF_CARBOHYDRATES
13:97182312	*MBNL2*	REACTOME_METABOLISM_OF_CARBOHYDRATES
13:97200682	*MBNL2*	REACTOME_METABOLISM_OF_CARBOHYDRATES
13:97278310	*MBNL2*	REACTOME_METABOLISM_OF_CARBOHYDRATES
13:97441830	*MBNL2*	REACTOME_METABOLISM_OF_CARBOHYDRATES
17:41056283	*RETREG3*	REACTOME_METABOLISM_OF_CARBOHYDRATES

14:55348869	*GCH1*	REACTOME_METABOLISM_OF_COFACTORS
16:19085298	*SYT17*	REACTOME_METABOLISM_OF_COFACTORS
16:19090194	*SYT17*	REACTOME_METABOLISM_OF_COFACTORS
2:170012307	*STK39*	REACTOME_METABOLISM_OF_FAT_SOLUBLE_VITAMINS
2:170028024	*STK39*	REACTOME_METABOLISM_OF_FAT_SOLUBLE_VITAMINS
2:170066424	*STK39*	REACTOME_METABOLISM_OF_FAT_SOLUBLE_VITAMINS
1:155205634	*GBAP1*	REACTOME_METABOLISM_OF_LIPIDS
2:96858914	*KCNIP3*	REACTOME_METABOLISM_OF_LIPIDS
2:101439596	*MAP4K4*	REACTOME_METABOLISM_OF_LIPIDS
2:101464631	*MAP4K4*	REACTOME_METABOLISM_OF_LIPIDS
2:101547064	*MAP4K4*	REACTOME_METABOLISM_OF_LIPIDS
2:101550181	*MAP4K4*	REACTOME_METABOLISM_OF_LIPIDS
2:101578010	*MAP4K4*	REACTOME_METABOLISM_OF_LIPIDS
2:101603812	*MAP4K4*	REACTOME_METABOLISM_OF_LIPIDS
2:169389091	*STK39*	REACTOME_METABOLISM_OF_LIPIDS
2:169518974	*STK39*	REACTOME_METABOLISM_OF_LIPIDS
2:169519063	*STK39*	REACTOME_METABOLISM_OF_LIPIDS
2:169520026	*STK39*	REACTOME_METABOLISM_OF_LIPIDS
2:169550660	*STK39*	REACTOME_METABOLISM_OF_LIPIDS
2:169561027	*STK39*	REACTOME_METABOLISM_OF_LIPIDS
2:169597744	*STK39*	REACTOME_METABOLISM_OF_LIPIDS
2:170012307	*STK39*	REACTOME_METABOLISM_OF_LIPIDS
2:170028024	*STK39*	REACTOME_METABOLISM_OF_LIPIDS
2:170066424	*STK39*	REACTOME_METABOLISM_OF_LIPIDS
3:160668220	*SPTSSB*	REACTOME_METABOLISM_OF_LIPIDS
3:160817877	*SPTSSB*	REACTOME_METABOLISM_OF_LIPIDS
3:161068566	*SPTSSB*	REACTOME_METABOLISM_OF_LIPIDS
3:161075189	*SPTSSB*	REACTOME_METABOLISM_OF_LIPIDS
4:17622910	*LCORL*	REACTOME_METABOLISM_OF_LIPIDS
4:114899005	*CAMK2D*	REACTOME_METABOLISM_OF_LIPIDS
4:169806784	*CLCN3*	REACTOME_METABOLISM_OF_LIPIDS
5:60053220	*ELOVL7*	REACTOME_METABOLISM_OF_LIPIDS
5:60085489	*ELOVL7*	REACTOME_METABOLISM_OF_LIPIDS
5:60093890	*ELOVL7*	REACTOME_METABOLISM_OF_LIPIDS
6:31846234	*HLA-DRB5*	REACTOME_METABOLISM_OF_LIPIDS
6:32126002	*HLA-DRB5*	REACTOME_METABOLISM_OF_LIPIDS
8:11679472	*CTSB*	REACTOME_METABOLISM_OF_LIPIDS
8:17161374	*FGF20*	REACTOME_METABOLISM_OF_LIPIDS
8:17176046	*FGF20*	REACTOME_METABOLISM_OF_LIPIDS
8:17243260	*FGF20*	REACTOME_METABOLISM_OF_LIPIDS
8:17260937	*FGF20*	REACTOME_METABOLISM_OF_LIPIDS
10:104591639	*GBF1*	REACTOME_METABOLISM_OF_LIPIDS
10:121505277	*INPP5F*	REACTOME_METABOLISM_OF_LIPIDS
10:121538407	*INPP5F*	REACTOME_METABOLISM_OF_LIPIDS
10:121560563	*INPP5F*	REACTOME_METABOLISM_OF_LIPIDS
12:123561638	*HIP1R*	REACTOME_METABOLISM_OF_LIPIDS
12:123585705	*HIP1R*	REACTOME_METABOLISM_OF_LIPIDS
14:88372366	*GALC*	REACTOME_METABOLISM_OF_LIPIDS
14:88393900	*GALC*	REACTOME_METABOLISM_OF_LIPIDS
14:88415750	*GALC*	REACTOME_METABOLISM_OF_LIPIDS
14:88443553	*GALC*	REACTOME_METABOLISM_OF_LIPIDS
15:61016235	*VPS13C*	REACTOME_METABOLISM_OF_LIPIDS
15:61065568	*VPS13C*	REACTOME_METABOLISM_OF_LIPIDS

15:61094298	*VPS13C*	REACTOME_METABOLISM_OF_LIPIDS
15:61140736	*VPS13C*	REACTOME_METABOLISM_OF_LIPIDS
15:61142062	*VPS13C*	REACTOME_METABOLISM_OF_LIPIDS
15:61162571	*VPS13C*	REACTOME_METABOLISM_OF_LIPIDS
15:61191617	*VPS13C*	REACTOME_METABOLISM_OF_LIPIDS
15:61195427	*VPS13C*	REACTOME_METABOLISM_OF_LIPIDS
15:61221682	*VPS13C*	REACTOME_METABOLISM_OF_LIPIDS
15:61298486	*VPS13C*	REACTOME_METABOLISM_OF_LIPIDS
15:61375480	*VPS13C*	REACTOME_METABOLISM_OF_LIPIDS
15:61434581	*VPS13C*	REACTOME_METABOLISM_OF_LIPIDS
15:61467078	*VPS13C*	REACTOME_METABOLISM_OF_LIPIDS
15:61470808	*VPS13C*	REACTOME_METABOLISM_OF_LIPIDS
15:61500263	*VPS13C*	REACTOME_METABOLISM_OF_LIPIDS
16:29871730	*CD19*	REACTOME_METABOLISM_OF_LIPIDS
16:30999462	*SETD1A*	REACTOME_METABOLISM_OF_LIPIDS
17:6370241	*V1NB1*	REACTOME_METABOLISM_OF_LIPIDS
17:6406333	*V1NB1*	REACTOME_METABOLISM_OF_LIPIDS
17:7983799	*V1NB1*	REACTOME_METABOLISM_OF_LIPIDS
17:8019125	*V1NB1*	REACTOME_METABOLISM_OF_LIPIDS
17:60124582	*BRIP1*	REACTOME_METABOLISM_OF_LIPIDS
20:5575860	*CRLS1*	REACTOME_METABOLISM_OF_LIPIDS
20:6011934	*CRLS1*	REACTOME_METABOLISM_OF_LIPIDS
2:169659620	*STK39*	REACTOME_METABOLISM_OF_NITRIC_OXIDE:_ENOS_ACTIVATION_AND_REGULATION
10:104847713	*GBF1*	REACTOME_METABOLISM_OF_NUCLEOTIDES
10:104861736	*GBF1*	REACTOME_METABOLISM_OF_NUCLEOTIDES
10:104926485	*GBF1*	REACTOME_METABOLISM_OF_NUCLEOTIDES
10:104929716	*GBF1*	REACTOME_METABOLISM_OF_NUCLEOTIDES
11:10392432	*RNF141*	REACTOME_METABOLISM_OF_NUCLEOTIDES
11:10429998	*RNF141*	REACTOME_METABOLISM_OF_NUCLEOTIDES
11:10458098	*RNF141*	REACTOME_METABOLISM_OF_NUCLEOTIDES
11:10471738	*RNF141*	REACTOME_METABOLISM_OF_NUCLEOTIDES
11:10487997	*RNF141*	REACTOME_METABOLISM_OF_NUCLEOTIDES
11:10494132	*RNF141*	REACTOME_METABOLISM_OF_NUCLEOTIDES
14:74477206	*RPS6KL1*	REACTOME_METABOLISM_OF_NUCLEOTIDES
16:30438930	*SETD1A*	REACTOME_METABOLISM_OF_NUCLEOTIDES
17:76177567	*DNAH17*	REACTOME_METABOLISM_OF_NUCLEOTIDES
5:133558885	*C5orf24*	REACTOME_METABOLISM_OF_RNA
5:134132269	*C5orf24*	REACTOME_METABOLISM_OF_RNA
6:30628082	*TRIM40*	REACTOME_METABOLISM_OF_RNA
6:30631063	*TRIM40*	REACTOME_METABOLISM_OF_RNA
6:30639369	*TRIM40*	REACTOME_METABOLISM_OF_RNA
7:23382980	*GPNMB*	REACTOME_METABOLISM_OF_RNA
7:23385251	*GPNMB*	REACTOME_METABOLISM_OF_RNA
7:23387110	*GPNMB*	REACTOME_METABOLISM_OF_RNA
7:23446436	*GPNMB*	REACTOME_METABOLISM_OF_RNA
7:23481701	*GPNMB*	REACTOME_METABOLISM_OF_RNA
7:23495961	*GPNMB*	REACTOME_METABOLISM_OF_RNA
7:66487445	*GS1-124K5.11*	REACTOME_METABOLISM_OF_RNA
7:66543959	*GS1-124K5.11*	REACTOME_METABOLISM_OF_RNA
7:66638082	*GS1-124K5.11*	REACTOME_METABOLISM_OF_RNA
9:33467577	*UBAP2*	REACTOME_METABOLISM_OF_RNA
17:7479140	*V1NB1*	REACTOME_METABOLISM_OF_RNA
17:7480325	*V1NB1*	REACTOME_METABOLISM_OF_RNA

17:42929985	*CRHR1_2*	REACTOME_METABOLISM_OF_RNA
10:104591639	*GBF1*	REACTOME_METABOLISM_OF_STEROID_HORMONES
2:170012307	*STK39*	REACTOME_METABOLISM_OF_STEROIDS
2:170028024	*STK39*	REACTOME_METABOLISM_OF_STEROIDS
2:170066424	*STK39*	REACTOME_METABOLISM_OF_STEROIDS
8:11679472	*CTSB*	REACTOME_METABOLISM_OF_STEROIDS
10:104591639	*GBF1*	REACTOME_METABOLISM_OF_STEROIDS
16:30999462	*SETD1A*	REACTOME_METABOLISM_OF_STEROIDS
2:170012307	*STK39*	REACTOME_METABOLISM_OF_VITAMINS_AND_COFACTORS
2:170028024	*STK39*	REACTOME_METABOLISM_OF_VITAMINS_AND_COFACTORS
2:170066424	*STK39*	REACTOME_METABOLISM_OF_VITAMINS_AND_COFACTORS
3:122259640	*KPNA1*	REACTOME_METABOLISM_OF_VITAMINS_AND_COFACTORS
3:182760073	*MCCC1*	REACTOME_METABOLISM_OF_VITAMINS_AND_COFACTORS
4:15709252	*BST1*	REACTOME_METABOLISM_OF_VITAMINS_AND_COFACTORS
4:15713608	*BST1*	REACTOME_METABOLISM_OF_VITAMINS_AND_COFACTORS
4:15724372	*BST1*	REACTOME_METABOLISM_OF_VITAMINS_AND_COFACTORS
4:15737882	*BST1*	REACTOME_METABOLISM_OF_VITAMINS_AND_COFACTORS
4:15789288	*BST1*	REACTOME_METABOLISM_OF_VITAMINS_AND_COFACTORS
4:15816670	*BST1*	REACTOME_METABOLISM_OF_VITAMINS_AND_COFACTORS
4:15829612	*BST1*	REACTOME_METABOLISM_OF_VITAMINS_AND_COFACTORS
5:102889674	*PAM*	REACTOME_METABOLISM_OF_VITAMINS_AND_COFACTORS
6:133025715	*RPS12*	REACTOME_METABOLISM_OF_VITAMINS_AND_COFACTORS
6:133028870	*RPS12*	REACTOME_METABOLISM_OF_VITAMINS_AND_COFACTORS
6:133075265	*RPS12*	REACTOME_METABOLISM_OF_VITAMINS_AND_COFACTORS
9:33791164	*UBAP2*	REACTOME_METABOLISM_OF_VITAMINS_AND_COFACTORS
14:55348869	*GCH1*	REACTOME_METABOLISM_OF_VITAMINS_AND_COFACTORS
14:74764925	*RPS6KL1*	REACTOME_METABOLISM_OF_VITAMINS_AND_COFACTORS
16:19085298	*SYT17*	REACTOME_METABOLISM_OF_VITAMINS_AND_COFACTORS
16:19090194	*SYT17*	REACTOME_METABOLISM_OF_VITAMINS_AND_COFACTORS
21:38136802	*DYRK1A*	REACTOME_METABOLISM_OF_VITAMINS_AND_COFACTORS
21:38168749	*DYRK1A*	REACTOME_METABOLISM_OF_VITAMINS_AND_COFACTORS
21:38210057	*DYRK1A*	REACTOME_METABOLISM_OF_VITAMINS_AND_COFACTORS
21:38238679	*DYRK1A*	REACTOME_METABOLISM_OF_VITAMINS_AND_COFACTORS
21:38328886	*DYRK1A*	REACTOME_METABOLISM_OF_VITAMINS_AND_COFACTORS
21:38332144	*DYRK1A*	REACTOME_METABOLISM_OF_VITAMINS_AND_COFACTORS
21:38356061	*DYRK1A*	REACTOME_METABOLISM_OF_VITAMINS_AND_COFACTORS
3:122259640	*KPNA1*	REACTOME_METABOLISM_OF_WATER_SOLUBLE_VITAMINS_AND_COFACTORS
3:182760073	*MCCC1*	REACTOME_METABOLISM_OF_WATER_SOLUBLE_VITAMINS_AND_COFACTORS
4:15709252	*BST1*	REACTOME_METABOLISM_OF_WATER_SOLUBLE_VITAMINS_AND_COFACTORS
4:15713608	*BST1*	REACTOME_METABOLISM_OF_WATER_SOLUBLE_VITAMINS_AND_COFACTORS
4:15724372	*BST1*	REACTOME_METABOLISM_OF_WATER_SOLUBLE_VITAMINS_AND_COFACTORS
4:15737882	*BST1*	REACTOME_METABOLISM_OF_WATER_SOLUBLE_VITAMINS_AND_COFACTORS
4:15789288	*BST1*	REACTOME_METABOLISM_OF_WATER_SOLUBLE_VITAMINS_AND_COFACTORS
4:15816670	*BST1*	REACTOME_METABOLISM_OF_WATER_SOLUBLE_VITAMINS_AND_COFACTORS
4:15829612	*BST1*	REACTOME_METABOLISM_OF_WATER_SOLUBLE_VITAMINS_AND_COFACTORS
5:102889674	*PAM*	REACTOME_METABOLISM_OF_WATER_SOLUBLE_VITAMINS_AND_COFACTORS
6:133025715	*RPS12*	REACTOME_METABOLISM_OF_WATER_SOLUBLE_VITAMINS_AND_COFACTORS
6:133028870	*RPS12*	REACTOME_METABOLISM_OF_WATER_SOLUBLE_VITAMINS_AND_COFACTORS
6:133075265	*RPS12*	REACTOME_METABOLISM_OF_WATER_SOLUBLE_VITAMINS_AND_COFACTORS
9:33791164	*UBAP2*	REACTOME_METABOLISM_OF_WATER_SOLUBLE_VITAMINS_AND_COFACTORS
14:74764925	*RPS6KL1*	REACTOME_METABOLISM_OF_WATER_SOLUBLE_VITAMINS_AND_COFACTORS
21:38136802	*DYRK1A*	REACTOME_METABOLISM_OF_WATER_SOLUBLE_VITAMINS_AND_COFACTORS
21:38168749	*DYRK1A*	REACTOME_METABOLISM_OF_WATER_SOLUBLE_VITAMINS_AND_COFACTORS

21:38210057	*DYRK1A*	REACTOME_METABOLISM_OF_WATER_SOLUBLE_VITAMINS_AND_COFACTORS
21:38238679	*DYRK1A*	REACTOME_METABOLISM_OF_WATER_SOLUBLE_VITAMINS_AND_COFACTORS
21:38328886	*DYRK1A*	REACTOME_METABOLISM_OF_WATER_SOLUBLE_VITAMINS_AND_COFACTORS
21:38332144	*DYRK1A*	REACTOME_METABOLISM_OF_WATER_SOLUBLE_VITAMINS_AND_COFACTORS
21:38356061	*DYRK1A*	REACTOME_METABOLISM_OF_WATER_SOLUBLE_VITAMINS_AND_COFACTORS
3:49162284	*IP6K2*	REACTOME_MET_ACTIVATES_PTK2_SIGNALING
3:49162583	*IP6K2*	REACTOME_MET_ACTIVATES_PTK2_SIGNALING
6:33153528	*HLA-DRB5*	REACTOME_MET_ACTIVATES_PTK2_SIGNALING
6:112494762	*FYN*	REACTOME_MET_ACTIVATES_PTK2_SIGNALING
6:112531401	*FYN*	REACTOME_MET_ACTIVATES_PTK2_SIGNALING
6:112561154	*FYN*	REACTOME_MET_ACTIVATES_PTK2_SIGNALING
6:112561430	*FYN*	REACTOME_MET_ACTIVATES_PTK2_SIGNALING
10:104635620	*GBF1*	REACTOME_METHYLATION
3:49162284	*IP6K2*	REACTOME_MET_PROMOTES_CELL_MOTILITY
3:49162583	*IP6K2*	REACTOME_MET_PROMOTES_CELL_MOTILITY
6:33153528	*HLA-DRB5*	REACTOME_MET_PROMOTES_CELL_MOTILITY
6:112494762	*FYN*	REACTOME_MET_PROMOTES_CELL_MOTILITY
6:112531401	*FYN*	REACTOME_MET_PROMOTES_CELL_MOTILITY
6:112561154	*FYN*	REACTOME_MET_PROMOTES_CELL_MOTILITY
6:112561430	*FYN*	REACTOME_MET_PROMOTES_CELL_MOTILITY
1:171837985	*VAMP4*	REACTOME_MHC_CLASS_II_ANTIGEN_PRESENTATION
1:171945488	*VAMP4*	REACTOME_MHC_CLASS_II_ANTIGEN_PRESENTATION
1:172137119	*VAMP4*	REACTOME_MHC_CLASS_II_ANTIGEN_PRESENTATION
1:172386261	*VAMP4*	REACTOME_MHC_CLASS_II_ANTIGEN_PRESENTATION
6:32602372	*HLA-DRB5*	REACTOME_MHC_CLASS_II_ANTIGEN_PRESENTATION
6:32930679	*HLA-DRB5*	REACTOME_MHC_CLASS_II_ANTIGEN_PRESENTATION
6:32934409	*HLA-DRB5*	REACTOME_MHC_CLASS_II_ANTIGEN_PRESENTATION
6:32973303	*HLA-DRB5*	REACTOME_MHC_CLASS_II_ANTIGEN_PRESENTATION
6:32974268	*HLA-DRB5*	REACTOME_MHC_CLASS_II_ANTIGEN_PRESENTATION
8:11712443	*CTSB*	REACTOME_MHC_CLASS_II_ANTIGEN_PRESENTATION
9:17579690	*SH3GL2_2*	REACTOME_MHC_CLASS_II_ANTIGEN_PRESENTATION
9:17587160	*SH3GL2_2*	REACTOME_MHC_CLASS_II_ANTIGEN_PRESENTATION
9:17609302	*SH3GL2_2*	REACTOME_MHC_CLASS_II_ANTIGEN_PRESENTATION
9:17626762	*SH3GL2_2*	REACTOME_MHC_CLASS_II_ANTIGEN_PRESENTATION
9:17627444	*SH3GL2_2*	REACTOME_MHC_CLASS_II_ANTIGEN_PRESENTATION
9:17642664	*SH3GL2_2*	REACTOME_MHC_CLASS_II_ANTIGEN_PRESENTATION
9:17710876	*SH3GL2_2*	REACTOME_MHC_CLASS_II_ANTIGEN_PRESENTATION
9:17732590	*SH3GL2_2*	REACTOME_MHC_CLASS_II_ANTIGEN_PRESENTATION
9:17737514	*SH3GL2_2*	REACTOME_MHC_CLASS_II_ANTIGEN_PRESENTATION
9:17758945	*SH3GL2_2*	REACTOME_MHC_CLASS_II_ANTIGEN_PRESENTATION
10:104258303	*GBF1*	REACTOME_MHC_CLASS_II_ANTIGEN_PRESENTATION
8:17125768	*FGF20*	REACTOME_MICROAUTOPHAGY
1:155181232	*GBAP1*	REACTOME_MITOCHONDRIAL_BIOGENESIS
8:16929685	*FGF20*	REACTOME_MITOCHONDRIAL_CALCIUM_ION_TRANSPORT
8:16967392	*FGF20*	REACTOME_MITOCHONDRIAL_CALCIUM_ION_TRANSPORT
8:16971257	*FGF20*	REACTOME_MITOCHONDRIAL_CALCIUM_ION_TRANSPORT
8:23415524	*BIN3*	REACTOME_MITOCHONDRIAL_IRON_SULFUR_CLUSTER_BIOGENESIS
1:155181232	*GBAP1*	REACTOME_MITOCHONDRIAL_PROTEIN_IMPORT
1:155698425	*GBAP1*	REACTOME_MITOCHONDRIAL_TRANSLATION
12:133295501	*FBRSL1*	REACTOME_MITOPHAGY
1:226497419	*ITPKB*	REACTOME_MITOTIC_G1_G1_S_PHASES
3:48199695	*IP6K2*	REACTOME_MITOTIC_G1_G1_S_PHASES
3:48214438	*IP6K2*	REACTOME_MITOTIC_G1_G1_S_PHASES

5:133558885	*C5orf24*	REACTOME_MITOTIC_G1_G1_S_PHASES
12:133206648	*FBRSL1*	REACTOME_MITOTIC_G1_G1_S_PHASES
12:133223685	*FBRSL1*	REACTOME_MITOTIC_G1_G1_S_PHASES
14:74640180	*RPS6KL1*	REACTOME_MITOTIC_G1_G1_S_PHASES
17:76177567	*DNAH17*	REACTOME_MITOTIC_G1_G1_S_PHASES
20:5934513	*CRLS1*	REACTOME_MITOTIC_G1_G1_S_PHASES
20:5935522	*CRLS1*	REACTOME_MITOTIC_G1_G1_S_PHASES
20:5968737	*CRLS1*	REACTOME_MITOTIC_G1_G1_S_PHASES
20:5968964	*CRLS1*	REACTOME_MITOTIC_G1_G1_S_PHASES
21:38740824	*DYRK1A*	REACTOME_MITOTIC_G1_G1_S_PHASES
21:38753354	*DYRK1A*	REACTOME_MITOTIC_G1_G1_S_PHASES
21:38770530	*DYRK1A*	REACTOME_MITOTIC_G1_G1_S_PHASES
21:38783614	*DYRK1A*	REACTOME_MITOTIC_G1_G1_S_PHASES
21:38814867	*DYRK1A*	REACTOME_MITOTIC_G1_G1_S_PHASES
1:226497419	*ITPKB*	REACTOME_MITOTIC_G2_G2_M_PHASES
3:48199695	*IP6K2*	REACTOME_MITOTIC_G2_G2_M_PHASES
3:48214438	*IP6K2*	REACTOME_MITOTIC_G2_G2_M_PHASES
5:133558885	*C5orf24*	REACTOME_MITOTIC_G2_G2_M_PHASES
10:103185542	*GBF1*	REACTOME_MITOTIC_G2_G2_M_PHASES
10:103305087	*GBF1*	REACTOME_MITOTIC_G2_G2_M_PHASES
10:103308244	*GBF1*	REACTOME_MITOTIC_G2_G2_M_PHASES
10:104258303	*GBF1*	REACTOME_MITOTIC_G2_G2_M_PHASES
14:74640180	*RPS6KL1*	REACTOME_MITOTIC_G2_G2_M_PHASES
16:19562008	*SYT17*	REACTOME_MITOTIC_G2_G2_M_PHASES
17:7578671	*V1NB1*	REACTOME_MITOTIC_G2_G2_M_PHASES
17:40818584	*RETREG3*	REACTOME_MITOTIC_G2_G2_M_PHASES
1:156059276	*GBAP1*	REACTOME_MITOTIC_METAPHASE_AND_ANAPHASE
2:169747216	*STK39*	REACTOME_MITOTIC_METAPHASE_AND_ANAPHASE
5:133558885	*C5orf24*	REACTOME_MITOTIC_METAPHASE_AND_ANAPHASE
12:122816734	*HIP1R*	REACTOME_MITOTIC_METAPHASE_AND_ANAPHASE
12:122817580	*HIP1R*	REACTOME_MITOTIC_METAPHASE_AND_ANAPHASE
12:122853197	*HIP1R*	REACTOME_MITOTIC_METAPHASE_AND_ANAPHASE
12:123057792	*HIP1R*	REACTOME_MITOTIC_METAPHASE_AND_ANAPHASE
12:123110365	*HIP1R*	REACTOME_MITOTIC_METAPHASE_AND_ANAPHASE
12:123110785	*HIP1R*	REACTOME_MITOTIC_METAPHASE_AND_ANAPHASE
17:45215865	*WNT3*	REACTOME_MITOTIC_METAPHASE_AND_ANAPHASE
17:45244822	*WNT3*	REACTOME_MITOTIC_METAPHASE_AND_ANAPHASE
2:169747216	*STK39*	REACTOME_MITOTIC_PROMETAPHASE
5:133558885	*C5orf24*	REACTOME_MITOTIC_PROMETAPHASE
10:104258303	*GBF1*	REACTOME_MITOTIC_PROMETAPHASE
12:122816734	*HIP1R*	REACTOME_MITOTIC_PROMETAPHASE
12:122817580	*HIP1R*	REACTOME_MITOTIC_PROMETAPHASE
12:122853197	*HIP1R*	REACTOME_MITOTIC_PROMETAPHASE
12:123057792	*HIP1R*	REACTOME_MITOTIC_PROMETAPHASE
12:123110365	*HIP1R*	REACTOME_MITOTIC_PROMETAPHASE
12:123110785	*HIP1R*	REACTOME_MITOTIC_PROMETAPHASE
16:19562008	*SYT17*	REACTOME_MITOTIC_PROMETAPHASE
17:40818584	*RETREG3*	REACTOME_MITOTIC_PROMETAPHASE
1:156059276	*GBAP1*	REACTOME_MITOTIC_PROPHASE
4:76695927	*FAM47E-STBD1*	REACTOME_MITOTIC_PROPHASE
5:133558885	*C5orf24*	REACTOME_MITOTIC_PROPHASE
2:169747216	*STK39*	REACTOME_MITOTIC_SPINDLE_CHECKPOINT
5:133558885	*C5orf24*	REACTOME_MITOTIC_SPINDLE_CHECKPOINT

12:122816734	*HIP1R*	REACTOME_MITOTIC_SPINDLE_CHECKPOINT
12:122817580	*HIP1R*	REACTOME_MITOTIC_SPINDLE_CHECKPOINT
12:122853197	*HIP1R*	REACTOME_MITOTIC_SPINDLE_CHECKPOINT
12:123057792	*HIP1R*	REACTOME_MITOTIC_SPINDLE_CHECKPOINT
12:123110365	*HIP1R*	REACTOME_MITOTIC_SPINDLE_CHECKPOINT
12:123110785	*HIP1R*	REACTOME_MITOTIC_SPINDLE_CHECKPOINT
17:45215865	*WNT3*	REACTOME_MITOTIC_SPINDLE_CHECKPOINT
17:45244822	*WNT3*	REACTOME_MITOTIC_SPINDLE_CHECKPOINT
10:15562608	*ITGA8*	REACTOME_MOLECULES_ASSOCIATED_WITH_ELASTIC_FIBRES
10:15569598	*ITGA8*	REACTOME_MOLECULES_ASSOCIATED_WITH_ELASTIC_FIBRES
10:15652368	*ITGA8*	REACTOME_MOLECULES_ASSOCIATED_WITH_ELASTIC_FIBRES
14:75057014	*RPS6KL1*	REACTOME_MOLECULES_ASSOCIATED_WITH_ELASTIC_FIBRES
1:156059276	*GBAP1*	REACTOME_M_PHASE
2:169747216	*STK39*	REACTOME_M_PHASE
4:76695927	*FAM47E-STBD1*	REACTOME_M_PHASE
5:133558885	*C5orf24*	REACTOME_M_PHASE
10:104258303	*GBF1*	REACTOME_M_PHASE
12:122816734	*HIP1R*	REACTOME_M_PHASE
12:122817580	*HIP1R*	REACTOME_M_PHASE
12:122853197	*HIP1R*	REACTOME_M_PHASE
12:123057792	*HIP1R*	REACTOME_M_PHASE
12:123110365	*HIP1R*	REACTOME_M_PHASE
12:123110785	*HIP1R*	REACTOME_M_PHASE
16:19562008	*SYT17*	REACTOME_M_PHASE
17:40818584	*RETREG3*	REACTOME_M_PHASE
17:45215865	*WNT3*	REACTOME_M_PHASE
17:45244822	*WNT3*	REACTOME_M_PHASE
17:42929985	*CRHR1_2*	REACTOME_MRNA_SPLICING_MINOR_PATHWAY
5:134132269	*C5orf24*	REACTOME_MRNA_SPLICING
6:30628082	*TRIM40*	REACTOME_MRNA_SPLICING
6:30631063	*TRIM40*	REACTOME_MRNA_SPLICING
6:30639369	*TRIM40*	REACTOME_MRNA_SPLICING
17:42929985	*CRHR1_2*	REACTOME_MRNA_SPLICING
13:49890086	*CAB39L*	REACTOME_MTOR_SIGNALLING
13:49897815	*CAB39L*	REACTOME_MTOR_SIGNALLING
13:49914395	*CAB39L*	REACTOME_MTOR_SIGNALLING
13:49927732	*CAB39L*	REACTOME_MTOR_SIGNALLING
13:49944031	*CAB39L*	REACTOME_MTOR_SIGNALLING
13:49960054	*CAB39L*	REACTOME_MTOR_SIGNALLING
13:49976904	*CAB39L*	REACTOME_MTOR_SIGNALLING
13:50013818	*CAB39L*	REACTOME_MTOR_SIGNALLING
7:65425894	*GS1-124K5.11*	REACTOME_MUCOPOLYSACCHARIDOSES
1:205884274	*RAB29*	REACTOME_MULTIFUNCTIONAL_ANION_EXCHANGERS
2:95967628	*KCNIP3*	REACTOME_MUSCLE_CONTRACTION
4:114424939	*CAMK2D*	REACTOME_MUSCLE_CONTRACTION
4:114494052	*CAMK2D*	REACTOME_MUSCLE_CONTRACTION
4:114529492	*CAMK2D*	REACTOME_MUSCLE_CONTRACTION
4:114539964	*CAMK2D*	REACTOME_MUSCLE_CONTRACTION
4:114597918	*CAMK2D*	REACTOME_MUSCLE_CONTRACTION
8:11558567	*CTSB*	REACTOME_MUSCLE_CONTRACTION
8:11599269	*CTSB*	REACTOME_MUSCLE_CONTRACTION
8:22421355	*BIN3*	REACTOME_MUSCLE_CONTRACTION
14:88681675	*GALC*	REACTOME_MUSCLE_CONTRACTION

14:88709038	*GALC*	REACTOME_MUSCLE_CONTRACTION
14:88738350	*GALC*	REACTOME_MUSCLE_CONTRACTION
14:88769689	*GALC*	REACTOME_MUSCLE_CONTRACTION
14:88778263	*GALC*	REACTOME_MUSCLE_CONTRACTION
17:7560294	*V1NB1*	REACTOME_MUSCLE_CONTRACTION
5:133558885	*C5orf24*	REACTOME_MYD88_CASCADE_INITIATED_ON_PLASMA_MEMBRANE
6:32151443	*HLA-DRB5*	REACTOME_MYD88_CASCADE_INITIATED_ON_PLASMA_MEMBRANE
10:103185542	*GBF1*	REACTOME_MYD88_CASCADE_INITIATED_ON_PLASMA_MEMBRANE
10:103305087	*GBF1*	REACTOME_MYD88_CASCADE_INITIATED_ON_PLASMA_MEMBRANE
10:103308244	*GBF1*	REACTOME_MYD88_CASCADE_INITIATED_ON_PLASMA_MEMBRANE
16:50730088	*CASC16*	REACTOME_MYD88_CASCADE_INITIATED_ON_PLASMA_MEMBRANE
16:50730446	*CASC16*	REACTOME_MYD88_CASCADE_INITIATED_ON_PLASMA_MEMBRANE
16:50745926	*CASC16*	REACTOME_MYD88_CASCADE_INITIATED_ON_PLASMA_MEMBRANE
17:41845031	*FAM171A2*	REACTOME_MYD88_CASCADE_INITIATED_ON_PLASMA_MEMBRANE
19:1611856	*SPPL2B*	REACTOME_MYOGENESIS
8:21565088	*BIN3*	REACTOME_NCAM1_INTERACTIONS
8:21582921	*BIN3*	REACTOME_NCAM1_INTERACTIONS
8:21665306	*BIN3*	REACTOME_NCAM1_INTERACTIONS
8:21668901	*BIN3*	REACTOME_NCAM1_INTERACTIONS
6:112137847	*FYN*	REACTOME_NCAM_SIGNALING_FOR_NEURITE_OUT_GROWTH
6:112151452	*FYN*	REACTOME_NCAM_SIGNALING_FOR_NEURITE_OUT_GROWTH
6:112175069	*FYN*	REACTOME_NCAM_SIGNALING_FOR_NEURITE_OUT_GROWTH
8:21565088	*BIN3*	REACTOME_NCAM_SIGNALING_FOR_NEURITE_OUT_GROWTH
8:21582921	*BIN3*	REACTOME_NCAM_SIGNALING_FOR_NEURITE_OUT_GROWTH
8:21665306	*BIN3*	REACTOME_NCAM_SIGNALING_FOR_NEURITE_OUT_GROWTH
8:21668901	*BIN3*	REACTOME_NCAM_SIGNALING_FOR_NEURITE_OUT_GROWTH
3:121319489	*KPNA1*	REACTOME_NEDDYLATION
3:121324643	*KPNA1*	REACTOME_NEDDYLATION
3:121340955	*KPNA1*	REACTOME_NEDDYLATION
3:182656610	*MCCC1*	REACTOME_NEDDYLATION
4:15608677	*BST1*	REACTOME_NEDDYLATION
4:15618898	*BST1*	REACTOME_NEDDYLATION
7:66096028	*GS1-124K5.11*	REACTOME_NEDDYLATION
7:66136281	*GS1-124K5.11*	REACTOME_NEDDYLATION
10:103185542	*GBF1*	REACTOME_NEDDYLATION
10:103305087	*GBF1*	REACTOME_NEDDYLATION
10:103308244	*GBF1*	REACTOME_NEDDYLATION
6:112137847	*FYN*	REACTOME_NEF_AND_SIGNAL_TRANSDUCTION
6:112151452	*FYN*	REACTOME_NEF_AND_SIGNAL_TRANSDUCTION
6:112175069	*FYN*	REACTOME_NEF_AND_SIGNAL_TRANSDUCTION
5:133558885	*C5orf24*	REACTOME_NEGATIVE_REGULATION_OF_FGFR1_SIGNALING
5:133558885	*C5orf24*	REACTOME_NEGATIVE_REGULATION_OF_FGFR2_SIGNALING
5:133558885	*C5orf24*	REACTOME_NEGATIVE_REGULATION_OF_FGFR3_SIGNALING
5:133558885	*C5orf24*	REACTOME_NEGATIVE_REGULATION_OF_FGFR4_SIGNALING
5:133558885	*C5orf24*	REACTOME_NEGATIVE_REGULATION_OF_MAPK_PATHWAY
9:17579690	*SH3GL2_2*	REACTOME_NEGATIVE_REGULATION_OF_MET_ACTIVITY
9:17587160	*SH3GL2_2*	REACTOME_NEGATIVE_REGULATION_OF_MET_ACTIVITY
9:17609302	*SH3GL2_2*	REACTOME_NEGATIVE_REGULATION_OF_MET_ACTIVITY
9:17626762	*SH3GL2_2*	REACTOME_NEGATIVE_REGULATION_OF_MET_ACTIVITY
9:17627444	*SH3GL2_2*	REACTOME_NEGATIVE_REGULATION_OF_MET_ACTIVITY
9:17642664	*SH3GL2_2*	REACTOME_NEGATIVE_REGULATION_OF_MET_ACTIVITY
9:17710876	*SH3GL2_2*	REACTOME_NEGATIVE_REGULATION_OF_MET_ACTIVITY
9:17732590	*SH3GL2_2*	REACTOME_NEGATIVE_REGULATION_OF_MET_ACTIVITY

9:17737514	*SH3GL2_2*	REACTOME_NEGATIVE_REGULATION_OF_MET_ACTIVITY
9:17758945	*SH3GL2_2*	REACTOME_NEGATIVE_REGULATION_OF_MET_ACTIVITY
4:114424939	*CAMK2D*	REACTOME_NEGATIVE_REGULATION_OF_NMDA_RECEPTOR_MEDIATED_NEURONAL_TRANSMISSION
4:114494052	*CAMK2D*	REACTOME_NEGATIVE_REGULATION_OF_NMDA_RECEPTOR_MEDIATED_NEURONAL_TRANSMISSION
4:114529492	*CAMK2D*	REACTOME_NEGATIVE_REGULATION_OF_NMDA_RECEPTOR_MEDIATED_NEURONAL_TRANSMISSION
4:114539964	*CAMK2D*	REACTOME_NEGATIVE_REGULATION_OF_NMDA_RECEPTOR_MEDIATED_NEURONAL_TRANSMISSION
4:114597918	*CAMK2D*	REACTOME_NEGATIVE_REGULATION_OF_NMDA_RECEPTOR_MEDIATED_NEURONAL_TRANSMISSION
11:83219006	*DLG2*	REACTOME_NEGATIVE_REGULATION_OF_NMDA_RECEPTOR_MEDIATED_NEURONAL_TRANSMISSION
11:83232915	*DLG2*	REACTOME_NEGATIVE_REGULATION_OF_NMDA_RECEPTOR_MEDIATED_NEURONAL_TRANSMISSION
11:83282740	*DLG2*	REACTOME_NEGATIVE_REGULATION_OF_NMDA_RECEPTOR_MEDIATED_NEURONAL_TRANSMISSION
11:83387273	*DLG2*	REACTOME_NEGATIVE_REGULATION_OF_NMDA_RECEPTOR_MEDIATED_NEURONAL_TRANSMISSION
11:83538733	*DLG2*	REACTOME_NEGATIVE_REGULATION_OF_NMDA_RECEPTOR_MEDIATED_NEURONAL_TRANSMISSION
11:83544472	*DLG2*	REACTOME_NEGATIVE_REGULATION_OF_NMDA_RECEPTOR_MEDIATED_NEURONAL_TRANSMISSION
11:83578152	*DLG2*	REACTOME_NEGATIVE_REGULATION_OF_NMDA_RECEPTOR_MEDIATED_NEURONAL_TRANSMISSION
11:83647657	*DLG2*	REACTOME_NEGATIVE_REGULATION_OF_NMDA_RECEPTOR_MEDIATED_NEURONAL_TRANSMISSION
11:83669903	*DLG2*	REACTOME_NEGATIVE_REGULATION_OF_NMDA_RECEPTOR_MEDIATED_NEURONAL_TRANSMISSION
11:83736246	*DLG2*	REACTOME_NEGATIVE_REGULATION_OF_NMDA_RECEPTOR_MEDIATED_NEURONAL_TRANSMISSION
11:83762918	*DLG2*	REACTOME_NEGATIVE_REGULATION_OF_NMDA_RECEPTOR_MEDIATED_NEURONAL_TRANSMISSION
11:83823752	*DLG2*	REACTOME_NEGATIVE_REGULATION_OF_NMDA_RECEPTOR_MEDIATED_NEURONAL_TRANSMISSION
11:83839992	*DLG2*	REACTOME_NEGATIVE_REGULATION_OF_NMDA_RECEPTOR_MEDIATED_NEURONAL_TRANSMISSION
11:84104763	*DLG2*	REACTOME_NEGATIVE_REGULATION_OF_NMDA_RECEPTOR_MEDIATED_NEURONAL_TRANSMISSION
11:84169039	*DLG2*	REACTOME_NEGATIVE_REGULATION_OF_NMDA_RECEPTOR_MEDIATED_NEURONAL_TRANSMISSION
11:84221628	*DLG2*	REACTOME_NEGATIVE_REGULATION_OF_NMDA_RECEPTOR_MEDIATED_NEURONAL_TRANSMISSION
11:84312008	*DLG2*	REACTOME_NEGATIVE_REGULATION_OF_NMDA_RECEPTOR_MEDIATED_NEURONAL_TRANSMISSION
11:84324361	*DLG2*	REACTOME_NEGATIVE_REGULATION_OF_NMDA_RECEPTOR_MEDIATED_NEURONAL_TRANSMISSION
11:84360346	*DLG2*	REACTOME_NEGATIVE_REGULATION_OF_NMDA_RECEPTOR_MEDIATED_NEURONAL_TRANSMISSION
11:84403409	*DLG2*	REACTOME_NEGATIVE_REGULATION_OF_NMDA_RECEPTOR_MEDIATED_NEURONAL_TRANSMISSION
17:7101771	*V1NB1*	REACTOME_NEGATIVE_REGULATION_OF_NMDA_RECEPTOR_MEDIATED_NEURONAL_TRANSMISSION
6:32179319	*HLA-DRB5*	REACTOME_NEGATIVE_REGULATION_OF_NOTCH4_SIGNALING
6:32186193	*HLA-DRB5*	REACTOME_NEGATIVE_REGULATION_OF_NOTCH4_SIGNALING
17:7129974	*V1NB1*	REACTOME_NEGATIVE_REGULATION_OF_TCF_DEPENDENT_SIGNALING_BY_DVL_INTERACTING_PROTEINS
5:133558885	*C5orf24*	REACTOME_NEGATIVE_REGULATION_OF_THE_PI3K_AKT_NETWORK
6:112137847	*FYN*	REACTOME_NEGATIVE_REGULATION_OF_THE_PI3K_AKT_NETWORK
6:112151452	*FYN*	REACTOME_NEGATIVE_REGULATION_OF_THE_PI3K_AKT_NETWORK
6:112175069	*FYN*	REACTOME_NEGATIVE_REGULATION_OF_THE_PI3K_AKT_NETWORK
16:50790891	*CASC16*	REACTOME_NEGATIVE_REGULATORS_OF_DDX58_IFIH1_SIGNALING
6:112137847	*FYN*	REACTOME_NEPHRIN_FAMILY_INTERACTIONS
6:112151452	*FYN*	REACTOME_NEPHRIN_FAMILY_INTERACTIONS
6:112175069	*FYN*	REACTOME_NEPHRIN_FAMILY_INTERACTIONS
6:112137847	*FYN*	REACTOME_NETRIN_1_SIGNALING
6:112151452	*FYN*	REACTOME_NETRIN_1_SIGNALING
6:112175069	*FYN*	REACTOME_NETRIN_1_SIGNALING
11:83219006	*DLG2*	REACTOME_NEUREXINS_AND_NEUROLIGINS
11:83232915	*DLG2*	REACTOME_NEUREXINS_AND_NEUROLIGINS
11:83282740	*DLG2*	REACTOME_NEUREXINS_AND_NEUROLIGINS
11:83387273	*DLG2*	REACTOME_NEUREXINS_AND_NEUROLIGINS
11:83538733	*DLG2*	REACTOME_NEUREXINS_AND_NEUROLIGINS
11:83544472	*DLG2*	REACTOME_NEUREXINS_AND_NEUROLIGINS
11:83578152	*DLG2*	REACTOME_NEUREXINS_AND_NEUROLIGINS
11:83647657	*DLG2*	REACTOME_NEUREXINS_AND_NEUROLIGINS
11:83669903	*DLG2*	REACTOME_NEUREXINS_AND_NEUROLIGINS
11:83736246	*DLG2*	REACTOME_NEUREXINS_AND_NEUROLIGINS
11:83762918	*DLG2*	REACTOME_NEUREXINS_AND_NEUROLIGINS

11:83823752	*DLG2*	REACTOME_NEUREXINS_AND_NEUROLIGINS
11:83839992	*DLG2*	REACTOME_NEUREXINS_AND_NEUROLIGINS
11:84104763	*DLG2*	REACTOME_NEUREXINS_AND_NEUROLIGINS
11:84169039	*DLG2*	REACTOME_NEUREXINS_AND_NEUROLIGINS
11:84221628	*DLG2*	REACTOME_NEUREXINS_AND_NEUROLIGINS
11:84312008	*DLG2*	REACTOME_NEUREXINS_AND_NEUROLIGINS
11:84324361	*DLG2*	REACTOME_NEUREXINS_AND_NEUROLIGINS
11:84360346	*DLG2*	REACTOME_NEUREXINS_AND_NEUROLIGINS
11:84403409	*DLG2*	REACTOME_NEUREXINS_AND_NEUROLIGINS
17:7101771	*V1NB1*	REACTOME_NEUREXINS_AND_NEUROLIGINS
1:156059276	*GBAP1*	REACTOME_NEURODEGENERATIVE_DISEASES
3:48199695	*IP6K2*	REACTOME_NEURODEGENERATIVE_DISEASES
3:48214438	*IP6K2*	REACTOME_NEURODEGENERATIVE_DISEASES
1:204876966	*RAB29*	REACTOME_NEUROFASCIN_INTERACTIONS
1:204940972	*RAB29*	REACTOME_NEUROFASCIN_INTERACTIONS
12:41144387	*LRRK2_2*	REACTOME_NEUROFASCIN_INTERACTIONS
12:41147295	*LRRK2_2*	REACTOME_NEUROFASCIN_INTERACTIONS
12:41195135	*LRRK2_2*	REACTOME_NEUROFASCIN_INTERACTIONS
12:41251554	*LRRK2_2*	REACTOME_NEUROFASCIN_INTERACTIONS
12:41255129	*LRRK2_2*	REACTOME_NEUROFASCIN_INTERACTIONS
12:41297969	*LRRK2_2*	REACTOME_NEUROFASCIN_INTERACTIONS
12:41407403	*LRRK2_2*	REACTOME_NEUROFASCIN_INTERACTIONS
1:154701406	*GBAP1*	REACTOME_NEURONAL_SYSTEM
1:154776412	*GBAP1*	REACTOME_NEURONAL_SYSTEM
1:154801670	*GBAP1*	REACTOME_NEURONAL_SYSTEM
1:154804988	*GBAP1*	REACTOME_NEURONAL_SYSTEM
2:18091890	*KCNS3*	REACTOME_NEURONAL_SYSTEM
2:18130187	*KCNS3*	REACTOME_NEURONAL_SYSTEM
2:18141542	*KCNS3*	REACTOME_NEURONAL_SYSTEM
2:18143082	*KCNS3*	REACTOME_NEURONAL_SYSTEM
2:18176120	*KCNS3*	REACTOME_NEURONAL_SYSTEM
2:18247777	*KCNS3*	REACTOME_NEURONAL_SYSTEM
2:18299564	*KCNS3*	REACTOME_NEURONAL_SYSTEM
2:18315537	*KCNS3*	REACTOME_NEURONAL_SYSTEM
2:18335515	*KCNS3*	REACTOME_NEURONAL_SYSTEM
2:18455049	*KCNS3*	REACTOME_NEURONAL_SYSTEM
2:18472861	*KCNS3*	REACTOME_NEURONAL_SYSTEM
3:19236100	*SATB1*	REACTOME_NEURONAL_SYSTEM
3:19312697	*SATB1*	REACTOME_NEURONAL_SYSTEM
3:19329968	*SATB1*	REACTOME_NEURONAL_SYSTEM
3:19334895	*SATB1*	REACTOME_NEURONAL_SYSTEM
3:19352476	*SATB1*	REACTOME_NEURONAL_SYSTEM
3:48835245	*IP6K2*	REACTOME_NEURONAL_SYSTEM
3:123089448	*KPNA1*	REACTOME_NEURONAL_SYSTEM
4:114424939	*CAMK2D*	REACTOME_NEURONAL_SYSTEM
4:114494052	*CAMK2D*	REACTOME_NEURONAL_SYSTEM
4:114529492	*CAMK2D*	REACTOME_NEURONAL_SYSTEM
4:114539964	*CAMK2D*	REACTOME_NEURONAL_SYSTEM
4:114597918	*CAMK2D*	REACTOME_NEURONAL_SYSTEM
6:29565889	*TRIM40*	REACTOME_NEURONAL_SYSTEM
6:29590715	*TRIM40*	REACTOME_NEURONAL_SYSTEM
6:72646790	*RIMS1*	REACTOME_NEURONAL_SYSTEM
6:72648340	*RIMS1*	REACTOME_NEURONAL_SYSTEM

6:72686444	*RIMS1*	REACTOME_NEURONAL_SYSTEM
6:72716358	*RIMS1*	REACTOME_NEURONAL_SYSTEM
6:72904599	*RIMS1*	REACTOME_NEURONAL_SYSTEM
6:72913435	*RIMS1*	REACTOME_NEURONAL_SYSTEM
6:72930929	*RIMS1*	REACTOME_NEURONAL_SYSTEM
6:72982289	*RIMS1*	REACTOME_NEURONAL_SYSTEM
6:72986711	*RIMS1*	REACTOME_NEURONAL_SYSTEM
6:73008061	*RIMS1*	REACTOME_NEURONAL_SYSTEM
6:73060492	*RIMS1*	REACTOME_NEURONAL_SYSTEM
6:73383348	*RIMS1*	REACTOME_NEURONAL_SYSTEM
6:73448086	*RIMS1*	REACTOME_NEURONAL_SYSTEM
8:131799767	*FAM49B*	REACTOME_NEURONAL_SYSTEM
8:131845512	*FAM49B*	REACTOME_NEURONAL_SYSTEM
8:131865425	*FAM49B*	REACTOME_NEURONAL_SYSTEM
8:131901170	*FAM49B*	REACTOME_NEURONAL_SYSTEM
11:83219006	*DLG2*	REACTOME_NEURONAL_SYSTEM
11:83232915	*DLG2*	REACTOME_NEURONAL_SYSTEM
11:83282740	*DLG2*	REACTOME_NEURONAL_SYSTEM
11:83387273	*DLG2*	REACTOME_NEURONAL_SYSTEM
11:83538733	*DLG2*	REACTOME_NEURONAL_SYSTEM
11:83544472	*DLG2*	REACTOME_NEURONAL_SYSTEM
11:83578152	*DLG2*	REACTOME_NEURONAL_SYSTEM
11:83647657	*DLG2*	REACTOME_NEURONAL_SYSTEM
11:83669903	*DLG2*	REACTOME_NEURONAL_SYSTEM
11:83736246	*DLG2*	REACTOME_NEURONAL_SYSTEM
11:83762918	*DLG2*	REACTOME_NEURONAL_SYSTEM
11:83823752	*DLG2*	REACTOME_NEURONAL_SYSTEM
11:83839992	*DLG2*	REACTOME_NEURONAL_SYSTEM
11:84104763	*DLG2*	REACTOME_NEURONAL_SYSTEM
11:84169039	*DLG2*	REACTOME_NEURONAL_SYSTEM
11:84221628	*DLG2*	REACTOME_NEURONAL_SYSTEM
11:84312008	*DLG2*	REACTOME_NEURONAL_SYSTEM
11:84324361	*DLG2*	REACTOME_NEURONAL_SYSTEM
11:84360346	*DLG2*	REACTOME_NEURONAL_SYSTEM
11:84403409	*DLG2*	REACTOME_NEURONAL_SYSTEM
12:46618863	*SCAF11*	REACTOME_NEURONAL_SYSTEM
14:88681675	*GALC*	REACTOME_NEURONAL_SYSTEM
14:88709038	*GALC*	REACTOME_NEURONAL_SYSTEM
14:88738350	*GALC*	REACTOME_NEURONAL_SYSTEM
14:88769689	*GALC*	REACTOME_NEURONAL_SYSTEM
14:88778263	*GALC*	REACTOME_NEURONAL_SYSTEM
16:50300511	*CASC16*	REACTOME_NEURONAL_SYSTEM
16:50317039	*CASC16*	REACTOME_NEURONAL_SYSTEM
17:7101771	*V1NB1*	REACTOME_NEURONAL_SYSTEM
17:7832034	*V1NB1*	REACTOME_NEURONAL_SYSTEM
17:43999203	*WNT3*	REACTOME_NEURONAL_SYSTEM
17:44005297	*WNT3*	REACTOME_NEURONAL_SYSTEM
17:44019712	*WNT3*	REACTOME_NEURONAL_SYSTEM
17:44060492	*WNT3*	REACTOME_NEURONAL_SYSTEM
17:44065263	*WNT3*	REACTOME_NEURONAL_SYSTEM
17:44081178	*WNT3*	REACTOME_NEURONAL_SYSTEM
17:44083297	*WNT3*	REACTOME_NEURONAL_SYSTEM
17:44800946	*WNT3*	REACTOME_NEURONAL_SYSTEM

17:44833217	*WNT3*	REACTOME_NEURONAL_SYSTEM
19:2557113	*SPPL2B*	REACTOME_NEURONAL_SYSTEM
19:2563327	*SPPL2B*	REACTOME_NEURONAL_SYSTEM
19:2580057	*SPPL2B*	REACTOME_NEURONAL_SYSTEM
19:2660983	*SPPL2B*	REACTOME_NEURONAL_SYSTEM
21:38984964	*DYRK1A*	REACTOME_NEURONAL_SYSTEM
21:39018136	*DYRK1A*	REACTOME_NEURONAL_SYSTEM
21:39021814	*DYRK1A*	REACTOME_NEURONAL_SYSTEM
21:39039956	*DYRK1A*	REACTOME_NEURONAL_SYSTEM
21:39088785	*DYRK1A*	REACTOME_NEURONAL_SYSTEM
21:39108160	*DYRK1A*	REACTOME_NEURONAL_SYSTEM
21:39120480	*DYRK1A*	REACTOME_NEURONAL_SYSTEM
21:39134980	*DYRK1A*	REACTOME_NEURONAL_SYSTEM
21:39197323	*DYRK1A*	REACTOME_NEURONAL_SYSTEM
21:39217472	*DYRK1A*	REACTOME_NEURONAL_SYSTEM
16:31004169	*SETD1A*	REACTOME_NEUROTOXICITY_OF_CLOSTRIDIUM_TOXINS
3:48835245	*IP6K2*	REACTOME_NEUROTRANSMITTER_RECEPTORS_AND_POSTSYNAPTIC_SIGNAL_TRANSMISSION
3:123089448	*KPNA1*	REACTOME_NEUROTRANSMITTER_RECEPTORS_AND_POSTSYNAPTIC_SIGNAL_TRANSMISSION
4:114424939	*CAMK2D*	REACTOME_NEUROTRANSMITTER_RECEPTORS_AND_POSTSYNAPTIC_SIGNAL_TRANSMISSION
4:114494052	*CAMK2D*	REACTOME_NEUROTRANSMITTER_RECEPTORS_AND_POSTSYNAPTIC_SIGNAL_TRANSMISSION
4:114529492	*CAMK2D*	REACTOME_NEUROTRANSMITTER_RECEPTORS_AND_POSTSYNAPTIC_SIGNAL_TRANSMISSION
4:114539964	*CAMK2D*	REACTOME_NEUROTRANSMITTER_RECEPTORS_AND_POSTSYNAPTIC_SIGNAL_TRANSMISSION
4:114597918	*CAMK2D*	REACTOME_NEUROTRANSMITTER_RECEPTORS_AND_POSTSYNAPTIC_SIGNAL_TRANSMISSION
6:29565889	*TRIM40*	REACTOME_NEUROTRANSMITTER_RECEPTORS_AND_POSTSYNAPTIC_SIGNAL_TRANSMISSION
6:29590715	*TRIM40*	REACTOME_NEUROTRANSMITTER_RECEPTORS_AND_POSTSYNAPTIC_SIGNAL_TRANSMISSION
8:131799767	*FAM49B*	REACTOME_NEUROTRANSMITTER_RECEPTORS_AND_POSTSYNAPTIC_SIGNAL_TRANSMISSION
8:131845512	*FAM49B*	REACTOME_NEUROTRANSMITTER_RECEPTORS_AND_POSTSYNAPTIC_SIGNAL_TRANSMISSION
8:131865425	*FAM49B*	REACTOME_NEUROTRANSMITTER_RECEPTORS_AND_POSTSYNAPTIC_SIGNAL_TRANSMISSION
8:131901170	*FAM49B*	REACTOME_NEUROTRANSMITTER_RECEPTORS_AND_POSTSYNAPTIC_SIGNAL_TRANSMISSION
11:83219006	*DLG2*	REACTOME_NEUROTRANSMITTER_RECEPTORS_AND_POSTSYNAPTIC_SIGNAL_TRANSMISSION
11:83232915	*DLG2*	REACTOME_NEUROTRANSMITTER_RECEPTORS_AND_POSTSYNAPTIC_SIGNAL_TRANSMISSION
11:83282740	*DLG2*	REACTOME_NEUROTRANSMITTER_RECEPTORS_AND_POSTSYNAPTIC_SIGNAL_TRANSMISSION
11:83387273	*DLG2*	REACTOME_NEUROTRANSMITTER_RECEPTORS_AND_POSTSYNAPTIC_SIGNAL_TRANSMISSION
11:83538733	*DLG2*	REACTOME_NEUROTRANSMITTER_RECEPTORS_AND_POSTSYNAPTIC_SIGNAL_TRANSMISSION
11:83544472	*DLG2*	REACTOME_NEUROTRANSMITTER_RECEPTORS_AND_POSTSYNAPTIC_SIGNAL_TRANSMISSION
11:83578152	*DLG2*	REACTOME_NEUROTRANSMITTER_RECEPTORS_AND_POSTSYNAPTIC_SIGNAL_TRANSMISSION
11:83647657	*DLG2*	REACTOME_NEUROTRANSMITTER_RECEPTORS_AND_POSTSYNAPTIC_SIGNAL_TRANSMISSION
11:83669903	*DLG2*	REACTOME_NEUROTRANSMITTER_RECEPTORS_AND_POSTSYNAPTIC_SIGNAL_TRANSMISSION
11:83736246	*DLG2*	REACTOME_NEUROTRANSMITTER_RECEPTORS_AND_POSTSYNAPTIC_SIGNAL_TRANSMISSION
11:83762918	*DLG2*	REACTOME_NEUROTRANSMITTER_RECEPTORS_AND_POSTSYNAPTIC_SIGNAL_TRANSMISSION
11:83823752	*DLG2*	REACTOME_NEUROTRANSMITTER_RECEPTORS_AND_POSTSYNAPTIC_SIGNAL_TRANSMISSION
11:83839992	*DLG2*	REACTOME_NEUROTRANSMITTER_RECEPTORS_AND_POSTSYNAPTIC_SIGNAL_TRANSMISSION
11:84104763	*DLG2*	REACTOME_NEUROTRANSMITTER_RECEPTORS_AND_POSTSYNAPTIC_SIGNAL_TRANSMISSION
11:84169039	*DLG2*	REACTOME_NEUROTRANSMITTER_RECEPTORS_AND_POSTSYNAPTIC_SIGNAL_TRANSMISSION
11:84221628	*DLG2*	REACTOME_NEUROTRANSMITTER_RECEPTORS_AND_POSTSYNAPTIC_SIGNAL_TRANSMISSION
11:84312008	*DLG2*	REACTOME_NEUROTRANSMITTER_RECEPTORS_AND_POSTSYNAPTIC_SIGNAL_TRANSMISSION
11:84324361	*DLG2*	REACTOME_NEUROTRANSMITTER_RECEPTORS_AND_POSTSYNAPTIC_SIGNAL_TRANSMISSION
11:84360346	*DLG2*	REACTOME_NEUROTRANSMITTER_RECEPTORS_AND_POSTSYNAPTIC_SIGNAL_TRANSMISSION
11:84403409	*DLG2*	REACTOME_NEUROTRANSMITTER_RECEPTORS_AND_POSTSYNAPTIC_SIGNAL_TRANSMISSION
16:50300511	*CASC16*	REACTOME_NEUROTRANSMITTER_RECEPTORS_AND_POSTSYNAPTIC_SIGNAL_TRANSMISSION
16:50317039	*CASC16*	REACTOME_NEUROTRANSMITTER_RECEPTORS_AND_POSTSYNAPTIC_SIGNAL_TRANSMISSION
17:7101771	*V1NB1*	REACTOME_NEUROTRANSMITTER_RECEPTORS_AND_POSTSYNAPTIC_SIGNAL_TRANSMISSION
17:43999203	*WNT3*	REACTOME_NEUROTRANSMITTER_RECEPTORS_AND_POSTSYNAPTIC_SIGNAL_TRANSMISSION

17:44005297	*WNT3*	REACTOME_NEUROTRANSMITTER_RECEPTORS_AND_POSTSYNAPTIC_SIGNAL_TRANSMISSION
17:44019712	*WNT3*	REACTOME_NEUROTRANSMITTER_RECEPTORS_AND_POSTSYNAPTIC_SIGNAL_TRANSMISSION
17:44060492	*WNT3*	REACTOME_NEUROTRANSMITTER_RECEPTORS_AND_POSTSYNAPTIC_SIGNAL_TRANSMISSION
17:44065263	*WNT3*	REACTOME_NEUROTRANSMITTER_RECEPTORS_AND_POSTSYNAPTIC_SIGNAL_TRANSMISSION
17:44081178	*WNT3*	REACTOME_NEUROTRANSMITTER_RECEPTORS_AND_POSTSYNAPTIC_SIGNAL_TRANSMISSION
17:44083297	*WNT3*	REACTOME_NEUROTRANSMITTER_RECEPTORS_AND_POSTSYNAPTIC_SIGNAL_TRANSMISSION
17:44800946	*WNT3*	REACTOME_NEUROTRANSMITTER_RECEPTORS_AND_POSTSYNAPTIC_SIGNAL_TRANSMISSION
17:44833217	*WNT3*	REACTOME_NEUROTRANSMITTER_RECEPTORS_AND_POSTSYNAPTIC_SIGNAL_TRANSMISSION
19:2557113	*SPPL2B*	REACTOME_NEUROTRANSMITTER_RECEPTORS_AND_POSTSYNAPTIC_SIGNAL_TRANSMISSION
19:2563327	*SPPL2B*	REACTOME_NEUROTRANSMITTER_RECEPTORS_AND_POSTSYNAPTIC_SIGNAL_TRANSMISSION
19:2580057	*SPPL2B*	REACTOME_NEUROTRANSMITTER_RECEPTORS_AND_POSTSYNAPTIC_SIGNAL_TRANSMISSION
19:2660983	*SPPL2B*	REACTOME_NEUROTRANSMITTER_RECEPTORS_AND_POSTSYNAPTIC_SIGNAL_TRANSMISSION
21:38984964	*DYRK1A*	REACTOME_NEUROTRANSMITTER_RECEPTORS_AND_POSTSYNAPTIC_SIGNAL_TRANSMISSION
21:39018136	*DYRK1A*	REACTOME_NEUROTRANSMITTER_RECEPTORS_AND_POSTSYNAPTIC_SIGNAL_TRANSMISSION
21:39021814	*DYRK1A*	REACTOME_NEUROTRANSMITTER_RECEPTORS_AND_POSTSYNAPTIC_SIGNAL_TRANSMISSION
21:39039956	*DYRK1A*	REACTOME_NEUROTRANSMITTER_RECEPTORS_AND_POSTSYNAPTIC_SIGNAL_TRANSMISSION
21:39088785	*DYRK1A*	REACTOME_NEUROTRANSMITTER_RECEPTORS_AND_POSTSYNAPTIC_SIGNAL_TRANSMISSION
21:39108160	*DYRK1A*	REACTOME_NEUROTRANSMITTER_RECEPTORS_AND_POSTSYNAPTIC_SIGNAL_TRANSMISSION
21:39120480	*DYRK1A*	REACTOME_NEUROTRANSMITTER_RECEPTORS_AND_POSTSYNAPTIC_SIGNAL_TRANSMISSION
21:39134980	*DYRK1A*	REACTOME_NEUROTRANSMITTER_RECEPTORS_AND_POSTSYNAPTIC_SIGNAL_TRANSMISSION
21:39197323	*DYRK1A*	REACTOME_NEUROTRANSMITTER_RECEPTORS_AND_POSTSYNAPTIC_SIGNAL_TRANSMISSION
21:39217472	*DYRK1A*	REACTOME_NEUROTRANSMITTER_RECEPTORS_AND_POSTSYNAPTIC_SIGNAL_TRANSMISSION
6:72646790	*RIMS1*	REACTOME_NEUROTRANSMITTER_RELEASE_CYCLE
6:72648340	*RIMS1*	REACTOME_NEUROTRANSMITTER_RELEASE_CYCLE
6:72686444	*RIMS1*	REACTOME_NEUROTRANSMITTER_RELEASE_CYCLE
6:72716358	*RIMS1*	REACTOME_NEUROTRANSMITTER_RELEASE_CYCLE
6:72904599	*RIMS1*	REACTOME_NEUROTRANSMITTER_RELEASE_CYCLE
6:72913435	*RIMS1*	REACTOME_NEUROTRANSMITTER_RELEASE_CYCLE
6:72930929	*RIMS1*	REACTOME_NEUROTRANSMITTER_RELEASE_CYCLE
6:72982289	*RIMS1*	REACTOME_NEUROTRANSMITTER_RELEASE_CYCLE
6:72986711	*RIMS1*	REACTOME_NEUROTRANSMITTER_RELEASE_CYCLE
6:73008061	*RIMS1*	REACTOME_NEUROTRANSMITTER_RELEASE_CYCLE
6:73060492	*RIMS1*	REACTOME_NEUROTRANSMITTER_RELEASE_CYCLE
1:204876966	*RAB29*	REACTOME_NEUTROPHIL_DEGRANULATION
1:204940972	*RAB29*	REACTOME_NEUTROPHIL_DEGRANULATION
1:226044391	*ITPKB*	REACTOME_NEUTROPHIL_DEGRANULATION
1:226052830	*ITPKB*	REACTOME_NEUTROPHIL_DEGRANULATION
3:49406095	*IP6K2*	REACTOME_NEUTROPHIL_DEGRANULATION
3:49424231	*IP6K2*	REACTOME_NEUTROPHIL_DEGRANULATION
3:49450137	*IP6K2*	REACTOME_NEUTROPHIL_DEGRANULATION
4:15709252	*BST1*	REACTOME_NEUTROPHIL_DEGRANULATION
4:15713608	*BST1*	REACTOME_NEUTROPHIL_DEGRANULATION
4:15724372	*BST1*	REACTOME_NEUTROPHIL_DEGRANULATION
4:15737882	*BST1*	REACTOME_NEUTROPHIL_DEGRANULATION
5:101579003	*PAM*	REACTOME_NEUTROPHIL_DEGRANULATION
6:133025715	*RPS12*	REACTOME_NEUTROPHIL_DEGRANULATION
6:133028870	*RPS12*	REACTOME_NEUTROPHIL_DEGRANULATION
7:65425894	*GS1-124K5.11*	REACTOME_NEUTROPHIL_DEGRANULATION
8:11712443	*CTSB*	REACTOME_NEUTROPHIL_DEGRANULATION
9:33791164	*UBAP2*	REACTOME_NEUTROPHIL_DEGRANULATION
11:10392432	*RNF141*	REACTOME_NEUTROPHIL_DEGRANULATION
11:10429998	*RNF141*	REACTOME_NEUTROPHIL_DEGRANULATION
11:10458098	*RNF141*	REACTOME_NEUTROPHIL_DEGRANULATION

11:10471738	*RNF141*	REACTOME_NEUTROPHIL_DEGRANULATION
11:10487997	*RNF141*	REACTOME_NEUTROPHIL_DEGRANULATION
11:10494132	*RNF141*	REACTOME_NEUTROPHIL_DEGRANULATION
11:82582134	*DLG2*	REACTOME_NEUTROPHIL_DEGRANULATION
11:82635123	*DLG2*	REACTOME_NEUTROPHIL_DEGRANULATION
11:82648357	*DLG2*	REACTOME_NEUTROPHIL_DEGRANULATION
12:45755099	*SCAF11*	REACTOME_NEUTROPHIL_DEGRANULATION
16:30510571	*SETD1A*	REACTOME_NEUTROPHIL_DEGRANULATION
16:30516492	*SETD1A*	REACTOME_NEUTROPHIL_DEGRANULATION
16:30520856	*SETD1A*	REACTOME_NEUTROPHIL_DEGRANULATION
16:31370452	*SETD1A*	REACTOME_NEUTROPHIL_DEGRANULATION
17:39883520	*RETREG3*	REACTOME_NEUTROPHIL_DEGRANULATION
17:42423844	*FAM171A2*	REACTOME_NEUTROPHIL_DEGRANULATION
17:42426940	*FAM171A2*	REACTOME_NEUTROPHIL_DEGRANULATION
17:76869878	*DNAH17*	REACTOME_NEUTROPHIL_DEGRANULATION
2:134902070	*TMEM163*	REACTOME_N_GLYCAN_ANTENNAE_ELONGATION_IN_THE_MEDIAL_TRANS_GOLGI
2:134932210	*TMEM163*	REACTOME_N_GLYCAN_ANTENNAE_ELONGATION_IN_THE_MEDIAL_TRANS_GOLGI
2:134956835	*TMEM163*	REACTOME_N_GLYCAN_ANTENNAE_ELONGATION_IN_THE_MEDIAL_TRANS_GOLGI
2:134986578	*TMEM163*	REACTOME_N_GLYCAN_ANTENNAE_ELONGATION_IN_THE_MEDIAL_TRANS_GOLGI
2:134993021	*TMEM163*	REACTOME_N_GLYCAN_ANTENNAE_ELONGATION_IN_THE_MEDIAL_TRANS_GOLGI
2:135035038	*TMEM163*	REACTOME_N_GLYCAN_ANTENNAE_ELONGATION_IN_THE_MEDIAL_TRANS_GOLGI
2:135040234	*TMEM163*	REACTOME_N_GLYCAN_ANTENNAE_ELONGATION_IN_THE_MEDIAL_TRANS_GOLGI
2:135165078	*TMEM163*	REACTOME_N_GLYCAN_ANTENNAE_ELONGATION_IN_THE_MEDIAL_TRANS_GOLGI
2:135166385	*TMEM163*	REACTOME_N_GLYCAN_ANTENNAE_ELONGATION_IN_THE_MEDIAL_TRANS_GOLGI
2:134902070	*TMEM163*	REACTOME_N_GLYCAN_ANTENNAE_ELONGATION
2:134932210	*TMEM163*	REACTOME_N_GLYCAN_ANTENNAE_ELONGATION
2:134956835	*TMEM163*	REACTOME_N_GLYCAN_ANTENNAE_ELONGATION
2:134986578	*TMEM163*	REACTOME_N_GLYCAN_ANTENNAE_ELONGATION
2:134993021	*TMEM163*	REACTOME_N_GLYCAN_ANTENNAE_ELONGATION
2:135035038	*TMEM163*	REACTOME_N_GLYCAN_ANTENNAE_ELONGATION
2:135040234	*TMEM163*	REACTOME_N_GLYCAN_ANTENNAE_ELONGATION
2:135165078	*TMEM163*	REACTOME_N_GLYCAN_ANTENNAE_ELONGATION
2:135166385	*TMEM163*	REACTOME_N_GLYCAN_ANTENNAE_ELONGATION
6:32151443	*HLA-DRB5*	REACTOME_N_GLYCAN_TRIMMING_IN_THE_ER_AND_CALNEXIN_CALRETICULIN_CYCLE
3:122259640	*KPNA1*	REACTOME_NICOTINAMIDE_SALVAGING
5:102889674	*PAM*	REACTOME_NICOTINAMIDE_SALVAGING
3:122259640	*KPNA1*	REACTOME_NICOTINATE_METABOLISM
4:15709252	*BST1*	REACTOME_NICOTINATE_METABOLISM
4:15713608	*BST1*	REACTOME_NICOTINATE_METABOLISM
4:15724372	*BST1*	REACTOME_NICOTINATE_METABOLISM
4:15737882	*BST1*	REACTOME_NICOTINATE_METABOLISM
4:15789288	*BST1*	REACTOME_NICOTINATE_METABOLISM
4:15816670	*BST1*	REACTOME_NICOTINATE_METABOLISM
4:15829612	*BST1*	REACTOME_NICOTINATE_METABOLISM
5:102889674	*PAM*	REACTOME_NICOTINATE_METABOLISM
11:10647977	*RNF141*	REACTOME_NITRIC_OXIDE_STIMULATES_GUANYLATE_CYCLASE
11:10648481	*RNF141*	REACTOME_NITRIC_OXIDE_STIMULATES_GUANYLATE_CYCLASE
11:10660840	*RNF141*	REACTOME_NITRIC_OXIDE_STIMULATES_GUANYLATE_CYCLASE
16:50730088	*CASC16*	REACTOME_NOD1_2_SIGNALING_PATHWAY
16:50730446	*CASC16*	REACTOME_NOD1_2_SIGNALING_PATHWAY
16:50745926	*CASC16*	REACTOME_NOD1_2_SIGNALING_PATHWAY
16:50790891	*CASC16*	REACTOME_NOD1_2_SIGNALING_PATHWAY
1:227067656	*ITPKB*	REACTOME_NONCANONICAL_ACTIVATION_OF_NOTCH3

3:49162284	*IP6K2*	REACTOME_NON_INTEGRIN_MEMBRANE_ECM_INTERACTIONS
3:49162583	*IP6K2*	REACTOME_NON_INTEGRIN_MEMBRANE_ECM_INTERACTIONS
3:49561208	*IP6K2*	REACTOME_NON_INTEGRIN_MEMBRANE_ECM_INTERACTIONS
6:30850582	*TRIM40*	REACTOME_NON_INTEGRIN_MEMBRANE_ECM_INTERACTIONS
6:33153528	*HLA-DRB5*	REACTOME_NON_INTEGRIN_MEMBRANE_ECM_INTERACTIONS
6:112494762	*FYN*	REACTOME_NON_INTEGRIN_MEMBRANE_ECM_INTERACTIONS
6:112531401	*FYN*	REACTOME_NON_INTEGRIN_MEMBRANE_ECM_INTERACTIONS
6:112561154	*FYN*	REACTOME_NON_INTEGRIN_MEMBRANE_ECM_INTERACTIONS
6:112561430	*FYN*	REACTOME_NON_INTEGRIN_MEMBRANE_ECM_INTERACTIONS
5:133558885	*C5orf24*	REACTOME_NONSENSE_MEDIATED_DECAY_NMD
6:72646790	*RIMS1*	REACTOME_NOREPINEPHRINE_NEUROTRANSMITTER_RELEASE_CYCLE
6:72648340	*RIMS1*	REACTOME_NOREPINEPHRINE_NEUROTRANSMITTER_RELEASE_CYCLE
6:72686444	*RIMS1*	REACTOME_NOREPINEPHRINE_NEUROTRANSMITTER_RELEASE_CYCLE
6:72716358	*RIMS1*	REACTOME_NOREPINEPHRINE_NEUROTRANSMITTER_RELEASE_CYCLE
6:72904599	*RIMS1*	REACTOME_NOREPINEPHRINE_NEUROTRANSMITTER_RELEASE_CYCLE
6:72913435	*RIMS1*	REACTOME_NOREPINEPHRINE_NEUROTRANSMITTER_RELEASE_CYCLE
6:72930929	*RIMS1*	REACTOME_NOREPINEPHRINE_NEUROTRANSMITTER_RELEASE_CYCLE
6:72982289	*RIMS1*	REACTOME_NOREPINEPHRINE_NEUROTRANSMITTER_RELEASE_CYCLE
6:72986711	*RIMS1*	REACTOME_NOREPINEPHRINE_NEUROTRANSMITTER_RELEASE_CYCLE
6:73008061	*RIMS1*	REACTOME_NOREPINEPHRINE_NEUROTRANSMITTER_RELEASE_CYCLE
6:73060492	*RIMS1*	REACTOME_NOREPINEPHRINE_NEUROTRANSMITTER_RELEASE_CYCLE
2:169659620	*STK39*	REACTOME_NOSTRIN_MEDIATED_ENOS_TRAFFICKING
19:3008151	*SPPL2B*	REACTOME_NOTCH1_INTRACELLULAR_DOMAIN_REGULATES_TRANSCRIPTION
19:3024077	*SPPL2B*	REACTOME_NOTCH1_INTRACELLULAR_DOMAIN_REGULATES_TRANSCRIPTION
1:227067656	*ITPKB*	REACTOME_NOTCH2_ACTIVATION_AND_TRANSMISSION_OF_SIGNAL_TO_THE_NUCLEUS
12:41144387	*LRRK2_2*	REACTOME_NOTCH2_ACTIVATION_AND_TRANSMISSION_OF_SIGNAL_TO_THE_NUCLEUS
12:41147295	*LRRK2_2*	REACTOME_NOTCH2_ACTIVATION_AND_TRANSMISSION_OF_SIGNAL_TO_THE_NUCLEUS
12:41195135	*LRRK2_2*	REACTOME_NOTCH2_ACTIVATION_AND_TRANSMISSION_OF_SIGNAL_TO_THE_NUCLEUS
12:41251554	*LRRK2_2*	REACTOME_NOTCH2_ACTIVATION_AND_TRANSMISSION_OF_SIGNAL_TO_THE_NUCLEUS
12:41255129	*LRRK2_2*	REACTOME_NOTCH2_ACTIVATION_AND_TRANSMISSION_OF_SIGNAL_TO_THE_NUCLEUS
12:41297969	*LRRK2_2*	REACTOME_NOTCH2_ACTIVATION_AND_TRANSMISSION_OF_SIGNAL_TO_THE_NUCLEUS
12:41407403	*LRRK2_2*	REACTOME_NOTCH2_ACTIVATION_AND_TRANSMISSION_OF_SIGNAL_TO_THE_NUCLEUS
1:227067656	*ITPKB*	REACTOME_NOTCH3_ACTIVATION_AND_TRANSMISSION_OF_SIGNAL_TO_THE_NUCLEUS
1:227067656	*ITPKB*	REACTOME_NOTCH4_ACTIVATION_AND_TRANSMISSION_OF_SIGNAL_TO_THE_NUCLEUS
6:32179319	*HLA-DRB5*	REACTOME_NOTCH4_ACTIVATION_AND_TRANSMISSION_OF_SIGNAL_TO_THE_NUCLEUS
6:32186193	*HLA-DRB5*	REACTOME_NOTCH4_ACTIVATION_AND_TRANSMISSION_OF_SIGNAL_TO_THE_NUCLEUS
6:32179319	*HLA-DRB5*	REACTOME_NOTCH4_INTRACELLULAR_DOMAIN_REGULATES_TRANSCRIPTION
6:32186193	*HLA-DRB5*	REACTOME_NOTCH4_INTRACELLULAR_DOMAIN_REGULATES_TRANSCRIPTION
6:32179319	*HLA-DRB5*	REACTOME_NOTCH_HLH_TRANSCRIPTION_PATHWAY
6:32186193	*HLA-DRB5*	REACTOME_NOTCH_HLH_TRANSCRIPTION_PATHWAY
1:155975202	*GBAP1*	REACTOME_NRAGE_SIGNALS_DEATH_THROUGH_JNK
17:7101771	*V1NB1*	REACTOME_NRCAM_INTERACTIONS
1:227067656	*ITPKB*	REACTOME_NRIF_SIGNALS_CELL_DEATH_FROM_THE_NUCLEUS
6:112137847	*FYN*	REACTOME_NTRK2_ACTIVATES_RAC1
6:112151452	*FYN*	REACTOME_NTRK2_ACTIVATES_RAC1
6:112175069	*FYN*	REACTOME_NTRK2_ACTIVATES_RAC1
1:156059276	*GBAP1*	REACTOME_NUCLEAR_ENVELOPE_BREAKDOWN
1:156059276	*GBAP1*	REACTOME_NUCLEAR_ENVELOPE_REASSEMBLY
5:133558885	*C5orf24*	REACTOME_NUCLEAR_ENVELOPE_REASSEMBLY
5:133558885	*C5orf24*	REACTOME_NUCLEAR_EVENTS_KINASE_AND_TRANSCRIPTION_FACTOR_ACTIVATION
17:41845031	*FAM171A2*	REACTOME_NUCLEAR_EVENTS_KINASE_AND_TRANSCRIPTION_FACTOR_ACTIVATION
15:61016235	*VPS13C*	REACTOME_NUCLEAR_RECEPTOR_TRANSCRIPTION_PATHWAY
15:61065568	*VPS13C*	REACTOME_NUCLEAR_RECEPTOR_TRANSCRIPTION_PATHWAY

15:61094298	*VPS13C*	REACTOME_NUCLEAR_RECEPTOR_TRANSCRIPTION_PATHWAY
15:61140736	*VPS13C*	REACTOME_NUCLEAR_RECEPTOR_TRANSCRIPTION_PATHWAY
15:61142062	*VPS13C*	REACTOME_NUCLEAR_RECEPTOR_TRANSCRIPTION_PATHWAY
15:61162571	*VPS13C*	REACTOME_NUCLEAR_RECEPTOR_TRANSCRIPTION_PATHWAY
15:61191617	*VPS13C*	REACTOME_NUCLEAR_RECEPTOR_TRANSCRIPTION_PATHWAY
15:61195427	*VPS13C*	REACTOME_NUCLEAR_RECEPTOR_TRANSCRIPTION_PATHWAY
15:61221682	*VPS13C*	REACTOME_NUCLEAR_RECEPTOR_TRANSCRIPTION_PATHWAY
15:61298486	*VPS13C*	REACTOME_NUCLEAR_RECEPTOR_TRANSCRIPTION_PATHWAY
15:61375480	*VPS13C*	REACTOME_NUCLEAR_RECEPTOR_TRANSCRIPTION_PATHWAY
15:61434581	*VPS13C*	REACTOME_NUCLEAR_RECEPTOR_TRANSCRIPTION_PATHWAY
15:61467078	*VPS13C*	REACTOME_NUCLEAR_RECEPTOR_TRANSCRIPTION_PATHWAY
15:61470808	*VPS13C*	REACTOME_NUCLEAR_RECEPTOR_TRANSCRIPTION_PATHWAY
15:61500263	*VPS13C*	REACTOME_NUCLEAR_RECEPTOR_TRANSCRIPTION_PATHWAY
1:227067656	*ITPKB*	REACTOME_NUCLEAR_SIGNALING_BY_ERBB4
17:42990894	*CRHR1_2*	REACTOME_NUCLEAR_SIGNALING_BY_ERBB4
10:104847713	*GBF1*	REACTOME_NUCLEOBASE_CATABOLISM
10:104861736	*GBF1*	REACTOME_NUCLEOBASE_CATABOLISM
10:104926485	*GBF1*	REACTOME_NUCLEOBASE_CATABOLISM
10:104929716	*GBF1*	REACTOME_NUCLEOBASE_CATABOLISM
14:74477206	*RPS6KL1*	REACTOME_NUCLEOBASE_CATABOLISM
16:50730088	*CASC16*	REACTOME_NUCLEOTIDE_BINDING_DOMAIN_LEUCINE_RICH_REPEAT_CONTAINING_RECEPTOR_NLR_SIGNALING_PATHWAYS
16:50730446	*CASC16*	REACTOME_NUCLEOTIDE_BINDING_DOMAIN_LEUCINE_RICH_REPEAT_CONTAINING_RECEPTOR_NLR_SIGNALING_PATHWAYS
16:50745926	*CASC16*	REACTOME_NUCLEOTIDE_BINDING_DOMAIN_LEUCINE_RICH_REPEAT_CONTAINING_RECEPTOR_NLR_SIGNALING_PATHWAYS
16:50790891	*CASC16*	REACTOME_NUCLEOTIDE_BINDING_DOMAIN_LEUCINE_RICH_REPEAT_CONTAINING_RECEPTOR_NLR_SIGNALING_PATHWAYS
1:226568665	*ITPKB*	REACTOME_NUCLEOTIDE_EXCISION_REPAIR
12:133206648	*FBRSL1*	REACTOME_NUCLEOTIDE_EXCISION_REPAIR
12:133223685	*FBRSL1*	REACTOME_NUCLEOTIDE_EXCISION_REPAIR
3:150935203	*MED12L*	REACTOME_NUCLEOTIDE_LIKE_PURINERGIC_RECEPTORS
3:151090424	*MED12L*	REACTOME_NUCLEOTIDE_LIKE_PURINERGIC_RECEPTORS
3:151099733	*MED12L*	REACTOME_NUCLEOTIDE_LIKE_PURINERGIC_RECEPTORS
11:10392432	*RNF141*	REACTOME_NUCLEOTIDE_SALVAGE
11:10429998	*RNF141*	REACTOME_NUCLEOTIDE_SALVAGE
11:10458098	*RNF141*	REACTOME_NUCLEOTIDE_SALVAGE
11:10471738	*RNF141*	REACTOME_NUCLEOTIDE_SALVAGE
11:10487997	*RNF141*	REACTOME_NUCLEOTIDE_SALVAGE
11:10494132	*RNF141*	REACTOME_NUCLEOTIDE_SALVAGE
17:76177567	*DNAH17*	REACTOME_NUCLEOTIDE_SALVAGE
17:42332803	*FAM171A2*	REACTOME_O2_CO2_EXCHANGE_IN_ERYTHROCYTES
17:42338993	*FAM171A2*	REACTOME_O2_CO2_EXCHANGE_IN_ERYTHROCYTES
3:122645304	*KPNA1*	REACTOME_O_GLYCOSYLATION_OF_TSR_DOMAIN_CONTAINING_PROTEINS
3:122720608	*KPNA1*	REACTOME_O_GLYCOSYLATION_OF_TSR_DOMAIN_CONTAINING_PROTEINS
9:18502815	*SH3GL2_2*	REACTOME_O_GLYCOSYLATION_OF_TSR_DOMAIN_CONTAINING_PROTEINS
9:18522254	*SH3GL2_2*	REACTOME_O_GLYCOSYLATION_OF_TSR_DOMAIN_CONTAINING_PROTEINS
9:18583014	*SH3GL2_2*	REACTOME_O_GLYCOSYLATION_OF_TSR_DOMAIN_CONTAINING_PROTEINS
9:18623302	*SH3GL2_2*	REACTOME_O_GLYCOSYLATION_OF_TSR_DOMAIN_CONTAINING_PROTEINS
9:18633417	*SH3GL2_2*	REACTOME_O_GLYCOSYLATION_OF_TSR_DOMAIN_CONTAINING_PROTEINS
9:18663540	*SH3GL2_2*	REACTOME_O_GLYCOSYLATION_OF_TSR_DOMAIN_CONTAINING_PROTEINS
9:18721847	*SH3GL2_2*	REACTOME_O_GLYCOSYLATION_OF_TSR_DOMAIN_CONTAINING_PROTEINS
9:18725820	*SH3GL2_2*	REACTOME_O_GLYCOSYLATION_OF_TSR_DOMAIN_CONTAINING_PROTEINS
6:29373213	*TRIM40*	REACTOME_OLFACTORY_SIGNALING_PATHWAY
6:29407378	*TRIM40*	REACTOME_OLFACTORY_SIGNALING_PATHWAY
6:29412222	*TRIM40*	REACTOME_OLFACTORY_SIGNALING_PATHWAY
3:182976382	*MCCC1*	REACTOME_O_LINKED_GLYCOSYLATION_OF_MUCINS

3:182979912	*MCCC1*	REACTOME_O_LINKED_GLYCOSYLATION_OF_MUCINS
3:183005320	*MCCC1*	REACTOME_O_LINKED_GLYCOSYLATION_OF_MUCINS
11:11299050	*RNF141*	REACTOME_O_LINKED_GLYCOSYLATION_OF_MUCINS
11:11316049	*RNF141*	REACTOME_O_LINKED_GLYCOSYLATION_OF_MUCINS
11:11364660	*RNF141*	REACTOME_O_LINKED_GLYCOSYLATION_OF_MUCINS
11:11399582	*RNF141*	REACTOME_O_LINKED_GLYCOSYLATION_OF_MUCINS
11:11412180	*RNF141*	REACTOME_O_LINKED_GLYCOSYLATION_OF_MUCINS
11:11494181	*RNF141*	REACTOME_O_LINKED_GLYCOSYLATION_OF_MUCINS
11:11504200	*RNF141*	REACTOME_O_LINKED_GLYCOSYLATION_OF_MUCINS
11:11508780	*RNF141*	REACTOME_O_LINKED_GLYCOSYLATION_OF_MUCINS
11:11519365	*RNF141*	REACTOME_O_LINKED_GLYCOSYLATION_OF_MUCINS
12:132687184	*FBRSL1*	REACTOME_O_LINKED_GLYCOSYLATION_OF_MUCINS
3:49561208	*IP6K2*	REACTOME_O_LINKED_GLYCOSYLATION
3:122645304	*KPNA1*	REACTOME_O_LINKED_GLYCOSYLATION
3:122720608	*KPNA1*	REACTOME_O_LINKED_GLYCOSYLATION
3:182976382	*MCCC1*	REACTOME_O_LINKED_GLYCOSYLATION
3:182979912	*MCCC1*	REACTOME_O_LINKED_GLYCOSYLATION
3:183005320	*MCCC1*	REACTOME_O_LINKED_GLYCOSYLATION
9:18502815	*SH3GL2_2*	REACTOME_O_LINKED_GLYCOSYLATION
9:18522254	*SH3GL2_2*	REACTOME_O_LINKED_GLYCOSYLATION
9:18583014	*SH3GL2_2*	REACTOME_O_LINKED_GLYCOSYLATION
9:18623302	*SH3GL2_2*	REACTOME_O_LINKED_GLYCOSYLATION
9:18633417	*SH3GL2_2*	REACTOME_O_LINKED_GLYCOSYLATION
9:18663540	*SH3GL2_2*	REACTOME_O_LINKED_GLYCOSYLATION
9:18721847	*SH3GL2_2*	REACTOME_O_LINKED_GLYCOSYLATION
9:18725820	*SH3GL2_2*	REACTOME_O_LINKED_GLYCOSYLATION
11:11299050	*RNF141*	REACTOME_O_LINKED_GLYCOSYLATION
11:11316049	*RNF141*	REACTOME_O_LINKED_GLYCOSYLATION
11:11364660	*RNF141*	REACTOME_O_LINKED_GLYCOSYLATION
11:11399582	*RNF141*	REACTOME_O_LINKED_GLYCOSYLATION
11:11412180	*RNF141*	REACTOME_O_LINKED_GLYCOSYLATION
11:11494181	*RNF141*	REACTOME_O_LINKED_GLYCOSYLATION
11:11504200	*RNF141*	REACTOME_O_LINKED_GLYCOSYLATION
11:11508780	*RNF141*	REACTOME_O_LINKED_GLYCOSYLATION
11:11519365	*RNF141*	REACTOME_O_LINKED_GLYCOSYLATION
12:132687184	*FBRSL1*	REACTOME_O_LINKED_GLYCOSYLATION
17:7578671	*V1NB1*	REACTOME_ONCOGENE_INDUCED_SENESCENCE
1:156059276	*GBAP1*	REACTOME_ONCOGENIC_MAPK_SIGNALING
4:114424939	*CAMK2D*	REACTOME_ONCOGENIC_MAPK_SIGNALING
4:114494052	*CAMK2D*	REACTOME_ONCOGENIC_MAPK_SIGNALING
4:114529492	*CAMK2D*	REACTOME_ONCOGENIC_MAPK_SIGNALING
4:114539964	*CAMK2D*	REACTOME_ONCOGENIC_MAPK_SIGNALING
4:114597918	*CAMK2D*	REACTOME_ONCOGENIC_MAPK_SIGNALING
17:42454243	*FAM171A2*	REACTOME_ONCOGENIC_MAPK_SIGNALING
3:48835245	*IP6K2*	REACTOME_OPIOID_SIGNALLING
3:123089448	*KPNA1*	REACTOME_OPIOID_SIGNALLING
4:114424939	*CAMK2D*	REACTOME_OPIOID_SIGNALLING
4:114494052	*CAMK2D*	REACTOME_OPIOID_SIGNALLING
4:114529492	*CAMK2D*	REACTOME_OPIOID_SIGNALLING
4:114539964	*CAMK2D*	REACTOME_OPIOID_SIGNALLING
4:114597918	*CAMK2D*	REACTOME_OPIOID_SIGNALLING
5:59236972	*ELOVL7*	REACTOME_OPIOID_SIGNALLING
5:59490965	*ELOVL7*	REACTOME_OPIOID_SIGNALLING

5:59554719	*ELOVL7*	REACTOME_OPIOID_SIGNALLING
5:59705316	*ELOVL7*	REACTOME_OPIOID_SIGNALLING
5:59736085	*ELOVL7*	REACTOME_OPIOID_SIGNALLING
5:59812853	*ELOVL7*	REACTOME_OPIOID_SIGNALLING
5:133558885	*C5orf24*	REACTOME_OPIOID_SIGNALLING
8:131799767	*FAM49B*	REACTOME_OPIOID_SIGNALLING
8:131845512	*FAM49B*	REACTOME_OPIOID_SIGNALLING
8:131865425	*FAM49B*	REACTOME_OPIOID_SIGNALLING
8:131901170	*FAM49B*	REACTOME_OPIOID_SIGNALLING
16:50300511	*CASC16*	REACTOME_OPIOID_SIGNALLING
16:50317039	*CASC16*	REACTOME_OPIOID_SIGNALLING
19:2557113	*SPPL2B*	REACTOME_OPIOID_SIGNALLING
19:2563327	*SPPL2B*	REACTOME_OPIOID_SIGNALLING
19:2580057	*SPPL2B*	REACTOME_OPIOID_SIGNALLING
19:2660983	*SPPL2B*	REACTOME_OPIOID_SIGNALLING
20:5934513	*CRLS1*	REACTOME_ORC1_REMOVAL_FROM_CHROMATIN
20:5935522	*CRLS1*	REACTOME_ORC1_REMOVAL_FROM_CHROMATIN
20:5968737	*CRLS1*	REACTOME_ORC1_REMOVAL_FROM_CHROMATIN
20:5968964	*CRLS1*	REACTOME_ORC1_REMOVAL_FROM_CHROMATIN
1:155181232	*GBAP1*	REACTOME_ORGANELLE_BIOGENESIS_AND_MAINTENANCE
3:121553904	*KPNA1*	REACTOME_ORGANELLE_BIOGENESIS_AND_MAINTENANCE
4:15492268	*BST1*	REACTOME_ORGANELLE_BIOGENESIS_AND_MAINTENANCE
4:15547513	*BST1*	REACTOME_ORGANELLE_BIOGENESIS_AND_MAINTENANCE
6:30601232	*TRIM40*	REACTOME_ORGANELLE_BIOGENESIS_AND_MAINTENANCE
6:30612107	*TRIM40*	REACTOME_ORGANELLE_BIOGENESIS_AND_MAINTENANCE
8:131145058	*FAM49B*	REACTOME_ORGANELLE_BIOGENESIS_AND_MAINTENANCE
8:131153067	*FAM49B*	REACTOME_ORGANELLE_BIOGENESIS_AND_MAINTENANCE
8:131245821	*FAM49B*	REACTOME_ORGANELLE_BIOGENESIS_AND_MAINTENANCE
8:131328260	*FAM49B*	REACTOME_ORGANELLE_BIOGENESIS_AND_MAINTENANCE
8:131404984	*FAM49B*	REACTOME_ORGANELLE_BIOGENESIS_AND_MAINTENANCE
8:131408202	*FAM49B*	REACTOME_ORGANELLE_BIOGENESIS_AND_MAINTENANCE
8:131455469	*FAM49B*	REACTOME_ORGANELLE_BIOGENESIS_AND_MAINTENANCE
9:34300212	*UBAP2*	REACTOME_ORGANELLE_BIOGENESIS_AND_MAINTENANCE
10:104005410	*GBF1*	REACTOME_ORGANELLE_BIOGENESIS_AND_MAINTENANCE
10:104034550	*GBF1*	REACTOME_ORGANELLE_BIOGENESIS_AND_MAINTENANCE
10:104258303	*GBF1*	REACTOME_ORGANELLE_BIOGENESIS_AND_MAINTENANCE
10:104443875	*GBF1*	REACTOME_ORGANELLE_BIOGENESIS_AND_MAINTENANCE
10:104452818	*GBF1*	REACTOME_ORGANELLE_BIOGENESIS_AND_MAINTENANCE
16:19562008	*SYT17*	REACTOME_ORGANELLE_BIOGENESIS_AND_MAINTENANCE
4:114424939	*CAMK2D*	REACTOME_OTHER_INTERLEUKIN_SIGNALING
4:114494052	*CAMK2D*	REACTOME_OTHER_INTERLEUKIN_SIGNALING
4:114529492	*CAMK2D*	REACTOME_OTHER_INTERLEUKIN_SIGNALING
4:114539964	*CAMK2D*	REACTOME_OTHER_INTERLEUKIN_SIGNALING
4:114597918	*CAMK2D*	REACTOME_OTHER_INTERLEUKIN_SIGNALING
5:133558885	*C5orf24*	REACTOME_OTHER_INTERLEUKIN_SIGNALING
6:112137847	*FYN*	REACTOME_OTHER_INTERLEUKIN_SIGNALING
6:112151452	*FYN*	REACTOME_OTHER_INTERLEUKIN_SIGNALING
6:112175069	*FYN*	REACTOME_OTHER_INTERLEUKIN_SIGNALING
8:21565088	*BIN3*	REACTOME_OTHER_INTERLEUKIN_SIGNALING
8:21582921	*BIN3*	REACTOME_OTHER_INTERLEUKIN_SIGNALING
8:21665306	*BIN3*	REACTOME_OTHER_INTERLEUKIN_SIGNALING
8:21668901	*BIN3*	REACTOME_OTHER_INTERLEUKIN_SIGNALING
11:83219006	*DLG2*	REACTOME_OTHER_INTERLEUKIN_SIGNALING

11:83232915	*DLG2*	REACTOME_OTHER_INTERLEUKIN_SIGNALING
11:83282740	*DLG2*	REACTOME_OTHER_INTERLEUKIN_SIGNALING
11:83387273	*DLG2*	REACTOME_OTHER_INTERLEUKIN_SIGNALING
11:83538733	*DLG2*	REACTOME_OTHER_INTERLEUKIN_SIGNALING
11:83544472	*DLG2*	REACTOME_OTHER_INTERLEUKIN_SIGNALING
11:83578152	*DLG2*	REACTOME_OTHER_INTERLEUKIN_SIGNALING
11:83647657	*DLG2*	REACTOME_OTHER_INTERLEUKIN_SIGNALING
11:83669903	*DLG2*	REACTOME_OTHER_INTERLEUKIN_SIGNALING
11:83736246	*DLG2*	REACTOME_OTHER_INTERLEUKIN_SIGNALING
11:83762918	*DLG2*	REACTOME_OTHER_INTERLEUKIN_SIGNALING
11:83823752	*DLG2*	REACTOME_OTHER_INTERLEUKIN_SIGNALING
11:83839992	*DLG2*	REACTOME_OTHER_INTERLEUKIN_SIGNALING
11:84104763	*DLG2*	REACTOME_OTHER_INTERLEUKIN_SIGNALING
11:84169039	*DLG2*	REACTOME_OTHER_INTERLEUKIN_SIGNALING
11:84221628	*DLG2*	REACTOME_OTHER_INTERLEUKIN_SIGNALING
11:84312008	*DLG2*	REACTOME_OTHER_INTERLEUKIN_SIGNALING
11:84324361	*DLG2*	REACTOME_OTHER_INTERLEUKIN_SIGNALING
11:84360346	*DLG2*	REACTOME_OTHER_INTERLEUKIN_SIGNALING
11:84403409	*DLG2*	REACTOME_OTHER_INTERLEUKIN_SIGNALING
17:7101771	*V1NB1*	REACTOME_OTHER_INTERLEUKIN_SIGNALING
17:40392358	*RETREG3*	REACTOME_OTHER_INTERLEUKIN_SIGNALING
17:42454243	*FAM171A2*	REACTOME_OTHER_INTERLEUKIN_SIGNALING
3:49406095	*IP6K2*	REACTOME_OVARIAN_TUMOR_DOMAIN_PROTEASES
3:49424231	*IP6K2*	REACTOME_OVARIAN_TUMOR_DOMAIN_PROTEASES
3:49450137	*IP6K2*	REACTOME_OVARIAN_TUMOR_DOMAIN_PROTEASES
16:50730088	*CASC16*	REACTOME_OVARIAN_TUMOR_DOMAIN_PROTEASES
16:50730446	*CASC16*	REACTOME_OVARIAN_TUMOR_DOMAIN_PROTEASES
16:50745926	*CASC16*	REACTOME_OVARIAN_TUMOR_DOMAIN_PROTEASES
17:7578671	*V1NB1*	REACTOME_OVARIAN_TUMOR_DOMAIN_PROTEASES
2:102357447	*MAP4K4*	REACTOME_OXIDATIVE_STRESS_INDUCED_SENESCENCE
2:102379173	*MAP4K4*	REACTOME_OXIDATIVE_STRESS_INDUCED_SENESCENCE
2:102413116	*MAP4K4*	REACTOME_OXIDATIVE_STRESS_INDUCED_SENESCENCE
17:7578671	*V1NB1*	REACTOME_OXIDATIVE_STRESS_INDUCED_SENESCENCE
17:42454243	*FAM171A2*	REACTOME_P130CAS_LINKAGE_TO_MAPK_SIGNALING_FOR_INTEGRINS
3:150935203	*MED12L*	REACTOME_P2Y_RECEPTORS
3:151090424	*MED12L*	REACTOME_P2Y_RECEPTORS
3:151099733	*MED12L*	REACTOME_P2Y_RECEPTORS
1:155975202	*GBAP1*	REACTOME_P75_NTR_RECEPTOR_MEDIATED_SIGNALLING
1:227067656	*ITPKB*	REACTOME_P75_NTR_RECEPTOR_MEDIATED_SIGNALLING
3:49406095	*IP6K2*	REACTOME_P75_NTR_RECEPTOR_MEDIATED_SIGNALLING
3:49424231	*IP6K2*	REACTOME_P75_NTR_RECEPTOR_MEDIATED_SIGNALLING
3:49450137	*IP6K2*	REACTOME_P75_NTR_RECEPTOR_MEDIATED_SIGNALLING
3:49406095	*IP6K2*	REACTOME_P75NTR_REGULATES_AXONOGENESIS
3:49424231	*IP6K2*	REACTOME_P75NTR_REGULATES_AXONOGENESIS
3:49450137	*IP6K2*	REACTOME_P75NTR_REGULATES_AXONOGENESIS
9:33447547	*UBAP2*	REACTOME_PASSIVE_TRANSPORT_BY_AQUAPORINS
12:133206648	*FBRSL1*	REACTOME_PCNA_DEPENDENT_LONG_PATCH_BASE_EXCISION_REPAIR
12:133223685	*FBRSL1*	REACTOME_PCNA_DEPENDENT_LONG_PATCH_BASE_EXCISION_REPAIR
3:49406095	*IP6K2*	REACTOME_PCP_CE_PATHWAY
3:49424231	*IP6K2*	REACTOME_PCP_CE_PATHWAY
3:49450137	*IP6K2*	REACTOME_PCP_CE_PATHWAY
17:7129974	*V1NB1*	REACTOME_PCP_CE_PATHWAY
6:32602372	*HLA-DRB5*	REACTOME_PD_1_SIGNALING

6:112137847	*FYN*	REACTOME_PECAM1_INTERACTIONS
6:112151452	*FYN*	REACTOME_PECAM1_INTERACTIONS
6:112175069	*FYN*	REACTOME_PECAM1_INTERACTIONS
8:11558567	*CTSB*	REACTOME_PEPTIDE_HORMONE_METABOLISM
8:11599269	*CTSB*	REACTOME_PEPTIDE_HORMONE_METABOLISM
4:76924571	*FAM47E-STBD1*	REACTOME_PEPTIDE_LIGAND_BINDING_RECEPTORS
20:5290081	*CRLS1*	REACTOME_PEPTIDE_LIGAND_BINDING_RECEPTORS
1:161875645	*FCGR2A*	REACTOME_PERK_REGULATES_GENE_EXPRESSION
1:161930954	*FCGR2A*	REACTOME_PERK_REGULATES_GENE_EXPRESSION
4:114424939	*CAMK2D*	REACTOME_PHASE_0_RAPID_DEPOLARISATION
4:114494052	*CAMK2D*	REACTOME_PHASE_0_RAPID_DEPOLARISATION
4:114529492	*CAMK2D*	REACTOME_PHASE_0_RAPID_DEPOLARISATION
4:114539964	*CAMK2D*	REACTOME_PHASE_0_RAPID_DEPOLARISATION
4:114597918	*CAMK2D*	REACTOME_PHASE_0_RAPID_DEPOLARISATION
2:95967628	*KCNIP3*	REACTOME_PHASE_1_INACTIVATION_OF_FAST_NAPLUS_CHANNELS
14:88681675	*GALC*	REACTOME_PHASE_4_RESTING_MEMBRANE_POTENTIAL
14:88709038	*GALC*	REACTOME_PHASE_4_RESTING_MEMBRANE_POTENTIAL
14:88738350	*GALC*	REACTOME_PHASE_4_RESTING_MEMBRANE_POTENTIAL
14:88769689	*GALC*	REACTOME_PHASE_4_RESTING_MEMBRANE_POTENTIAL
14:88778263	*GALC*	REACTOME_PHASE_4_RESTING_MEMBRANE_POTENTIAL
7:65705034	*GS1-124K5.11*	REACTOME_PHASE_II_CONJUGATION_OF_COMPOUNDS
7:65824287	*GS1-124K5.11*	REACTOME_PHASE_II_CONJUGATION_OF_COMPOUNDS
10:104635620	*GBF1*	REACTOME_PHASE_II_CONJUGATION_OF_COMPOUNDS
4:17465704	*LCORL*	REACTOME_PHENYLALANINE_AND_TYROSINE_METABOLISM
4:17503839	*LCORL*	REACTOME_PHENYLALANINE_AND_TYROSINE_METABOLISM
4:17465704	*LCORL*	REACTOME_PHENYLALANINE_METABOLISM
4:17503839	*LCORL*	REACTOME_PHENYLALANINE_METABOLISM
14:74477206	*RPS6KL1*	REACTOME_PHOSPHATE_BOND_HYDROLYSIS_BY_NTPDASE_PROTEINS
2:96858914	*KCNIP3*	REACTOME_PHOSPHOLIPID_METABOLISM
6:31846234	*HLA-DRB5*	REACTOME_PHOSPHOLIPID_METABOLISM
8:17161374	*FGF20*	REACTOME_PHOSPHOLIPID_METABOLISM
8:17176046	*FGF20*	REACTOME_PHOSPHOLIPID_METABOLISM
8:17243260	*FGF20*	REACTOME_PHOSPHOLIPID_METABOLISM
8:17260937	*FGF20*	REACTOME_PHOSPHOLIPID_METABOLISM
10:121505277	*INPP5F*	REACTOME_PHOSPHOLIPID_METABOLISM
10:121538407	*INPP5F*	REACTOME_PHOSPHOLIPID_METABOLISM
10:121560563	*INPP5F*	REACTOME_PHOSPHOLIPID_METABOLISM
12:123561638	*HIP1R*	REACTOME_PHOSPHOLIPID_METABOLISM
12:123585705	*HIP1R*	REACTOME_PHOSPHOLIPID_METABOLISM
16:29871730	*CD19*	REACTOME_PHOSPHOLIPID_METABOLISM
17:6370241	*V1NB1*	REACTOME_PHOSPHOLIPID_METABOLISM
17:6406333	*V1NB1*	REACTOME_PHOSPHOLIPID_METABOLISM
20:5575860	*CRLS1*	REACTOME_PHOSPHOLIPID_METABOLISM
20:6011934	*CRLS1*	REACTOME_PHOSPHOLIPID_METABOLISM
6:32602372	*HLA-DRB5*	REACTOME_PHOSPHORYLATION_OF_CD3_AND_TCR_ZETA_CHAINS
17:45215865	*WNT3*	REACTOME_PHOSPHORYLATION_OF_THE_APC_C
17:45244822	*WNT3*	REACTOME_PHOSPHORYLATION_OF_THE_APC_C
5:133558885	*C5orf24*	ME_PHOSPHORYLATION_SITE_MUTANTS_OF_CTNNB1_ARE_NOT_TARGETED_TO_THE_PROTEASOME_BY_THE_DESTRUCTION_
8:11558567	*CTSB*	REACTOME_PHYSIOLOGICAL_FACTORS
8:11599269	*CTSB*	REACTOME_PHYSIOLOGICAL_FACTORS
3:49406095	*IP6K2*	REACTOME_PI3K_AKT_ACTIVATION
3:49424231	*IP6K2*	REACTOME_PI3K_AKT_ACTIVATION
3:49450137	*IP6K2*	REACTOME_PI3K_AKT_ACTIVATION

6:112137847	*FYN*	REACTOME_PI3K_AKT_SIGNALING_IN_CANCER
6:112151452	*FYN*	REACTOME_PI3K_AKT_SIGNALING_IN_CANCER
6:112175069	*FYN*	REACTOME_PI3K_AKT_SIGNALING_IN_CANCER
17:7578671	*V1NB1*	REACTOME_PI5P_REGULATES_TP53_ACETYLATION
8:17161374	*FGF20*	REACTOME_PI_METABOLISM
8:17176046	*FGF20*	REACTOME_PI_METABOLISM
8:17243260	*FGF20*	REACTOME_PI_METABOLISM
8:17260937	*FGF20*	REACTOME_PI_METABOLISM
10:121505277	*INPP5F*	REACTOME_PI_METABOLISM
10:121538407	*INPP5F*	REACTOME_PI_METABOLISM
10:121560563	*INPP5F*	REACTOME_PI_METABOLISM
3:48835245	*IP6K2*	REACTOME_PKA_ACTIVATION_IN_GLUCAGON_SIGNALLING
3:123089448	*KPNA1*	REACTOME_PKA_ACTIVATION_IN_GLUCAGON_SIGNALLING
8:131799767	*FAM49B*	REACTOME_PKA_ACTIVATION_IN_GLUCAGON_SIGNALLING
8:131845512	*FAM49B*	REACTOME_PKA_ACTIVATION_IN_GLUCAGON_SIGNALLING
8:131865425	*FAM49B*	REACTOME_PKA_ACTIVATION_IN_GLUCAGON_SIGNALLING
8:131901170	*FAM49B*	REACTOME_PKA_ACTIVATION_IN_GLUCAGON_SIGNALLING
16:50300511	*CASC16*	REACTOME_PKA_ACTIVATION_IN_GLUCAGON_SIGNALLING
16:50317039	*CASC16*	REACTOME_PKA_ACTIVATION_IN_GLUCAGON_SIGNALLING
3:48835245	*IP6K2*	REACTOME_PKA_MEDIATED_PHOSPHORYLATION_OF_CREB
3:123089448	*KPNA1*	REACTOME_PKA_MEDIATED_PHOSPHORYLATION_OF_CREB
8:131799767	*FAM49B*	REACTOME_PKA_MEDIATED_PHOSPHORYLATION_OF_CREB
8:131845512	*FAM49B*	REACTOME_PKA_MEDIATED_PHOSPHORYLATION_OF_CREB
8:131865425	*FAM49B*	REACTOME_PKA_MEDIATED_PHOSPHORYLATION_OF_CREB
8:131901170	*FAM49B*	REACTOME_PKA_MEDIATED_PHOSPHORYLATION_OF_CREB
16:50300511	*CASC16*	REACTOME_PKA_MEDIATED_PHOSPHORYLATION_OF_CREB
16:50317039	*CASC16*	REACTOME_PKA_MEDIATED_PHOSPHORYLATION_OF_CREB
1:155359992	*GBAP1*	REACTOME_PKMTS_METHYLATE_HISTONE_LYSINES
6:31862876	*HLA-DRB5*	REACTOME_PKMTS_METHYLATE_HISTONE_LYSINES
19:2172266	*SPPL2B*	REACTOME_PKMTS_METHYLATE_HISTONE_LYSINES
19:2202252	*SPPL2B*	REACTOME_PKMTS_METHYLATE_HISTONE_LYSINES
3:49406095	*IP6K2*	REACTOME_PLATELET_ACTIVATION_SIGNALING_AND_AGGREGATION
3:49424231	*IP6K2*	REACTOME_PLATELET_ACTIVATION_SIGNALING_AND_AGGREGATION
3:49450137	*IP6K2*	REACTOME_PLATELET_ACTIVATION_SIGNALING_AND_AGGREGATION
3:151090424	*MED12L*	REACTOME_PLATELET_ACTIVATION_SIGNALING_AND_AGGREGATION
3:151099733	*MED12L*	REACTOME_PLATELET_ACTIVATION_SIGNALING_AND_AGGREGATION
4:90804532	*SNCA_2*	REACTOME_PLATELET_ACTIVATION_SIGNALING_AND_AGGREGATION
4:90868355	*SNCA_2*	REACTOME_PLATELET_ACTIVATION_SIGNALING_AND_AGGREGATION
6:31682029	*HLA-DRB5*	REACTOME_PLATELET_ACTIVATION_SIGNALING_AND_AGGREGATION
6:112137847	*FYN*	REACTOME_PLATELET_ACTIVATION_SIGNALING_AND_AGGREGATION
6:112151452	*FYN*	REACTOME_PLATELET_ACTIVATION_SIGNALING_AND_AGGREGATION
6:112175069	*FYN*	REACTOME_PLATELET_ACTIVATION_SIGNALING_AND_AGGREGATION
8:130914130	*FAM49B*	REACTOME_PLATELET_ACTIVATION_SIGNALING_AND_AGGREGATION
8:131002284	*FAM49B*	REACTOME_PLATELET_ACTIVATION_SIGNALING_AND_AGGREGATION
17:42454243	*FAM171A2*	REACTOME_PLATELET_ACTIVATION_SIGNALING_AND_AGGREGATION
17:76969183	*DNAH17*	REACTOME_PLATELET_ACTIVATION_SIGNALING_AND_AGGREGATION
19:2557113	*SPPL2B*	REACTOME_PLATELET_ACTIVATION_SIGNALING_AND_AGGREGATION
19:2563327	*SPPL2B*	REACTOME_PLATELET_ACTIVATION_SIGNALING_AND_AGGREGATION
19:2580057	*SPPL2B*	REACTOME_PLATELET_ACTIVATION_SIGNALING_AND_AGGREGATION
19:2660983	*SPPL2B*	REACTOME_PLATELET_ACTIVATION_SIGNALING_AND_AGGREGATION
19:3142784	*SPPL2B*	REACTOME_PLATELET_ACTIVATION_SIGNALING_AND_AGGREGATION
19:3147326	*SPPL2B*	REACTOME_PLATELET_ACTIVATION_SIGNALING_AND_AGGREGATION
6:112137847	*FYN*	REACTOME_PLATELET_ADHESION_TO_EXPOSED_COLLAGEN

6:112151452	*FYN*	REACTOME_PLATELET_ADHESION_TO_EXPOSED_COLLAGEN
6:112175069	*FYN*	REACTOME_PLATELET_ADHESION_TO_EXPOSED_COLLAGEN
17:42454243	*FAM171A2*	REACTOME_PLATELET_AGGREGATION_PLUG_FORMATION
5:133558885	*C5orf24*	REACTOME_PLATELET_HOMEOSTASIS
11:10647977	*RNF141*	REACTOME_PLATELET_HOMEOSTASIS
11:10648481	*RNF141*	REACTOME_PLATELET_HOMEOSTASIS
11:10660840	*RNF141*	REACTOME_PLATELET_HOMEOSTASIS
19:2557113	*SPPL2B*	REACTOME_PLATELET_HOMEOSTASIS
19:2563327	*SPPL2B*	REACTOME_PLATELET_HOMEOSTASIS
19:2580057	*SPPL2B*	REACTOME_PLATELET_HOMEOSTASIS
19:2660983	*SPPL2B*	REACTOME_PLATELET_HOMEOSTASIS
5:133558885	*C5orf24*	REACTOME_PLATELET_SENSITIZATION_BY_LDL
1:226568665	*ITPKB*	REACTOME_POLB_DEPENDENT_LONG_PATCH_BASE_EXCISION_REPAIR
1:226497419	*ITPKB*	REACTOME_POLO_LIKE_KINASE_MEDIATED_EVENTS
3:48199695	*IP6K2*	REACTOME_POLO_LIKE_KINASE_MEDIATED_EVENTS
3:48214438	*IP6K2*	REACTOME_POLO_LIKE_KINASE_MEDIATED_EVENTS
14:74640180	*RPS6KL1*	REACTOME_POLO_LIKE_KINASE_MEDIATED_EVENTS
6:31862876	*HLA-DRB5*	REACTOME_POSITIVE_EPIGENETIC_REGULATION_OF_RRNA_EXPRESSION
1:172386261	*VAMP4*	REACTOME_POST_TRANSLATIONAL_MODIFICATION:_SYNTHESIS_OF_GPI_ANCHORED_PROTEINS
1:172388441	*VAMP4*	REACTOME_POST_TRANSLATIONAL_MODIFICATION:_SYNTHESIS_OF_GPI_ANCHORED_PROTEINS
1:172389198	*VAMP4*	REACTOME_POST_TRANSLATIONAL_MODIFICATION:_SYNTHESIS_OF_GPI_ANCHORED_PROTEINS
4:15709252	*BST1*	REACTOME_POST_TRANSLATIONAL_MODIFICATION:_SYNTHESIS_OF_GPI_ANCHORED_PROTEINS
4:15713608	*BST1*	REACTOME_POST_TRANSLATIONAL_MODIFICATION:_SYNTHESIS_OF_GPI_ANCHORED_PROTEINS
4:15724372	*BST1*	REACTOME_POST_TRANSLATIONAL_MODIFICATION:_SYNTHESIS_OF_GPI_ANCHORED_PROTEINS
4:15737882	*BST1*	REACTOME_POST_TRANSLATIONAL_MODIFICATION:_SYNTHESIS_OF_GPI_ANCHORED_PROTEINS
4:76986566	*FAM47E-STBD1*	REACTOME_POST_TRANSLATIONAL_MODIFICATION:_SYNTHESIS_OF_GPI_ANCHORED_PROTEINS
4:77021433	*FAM47E-STBD1*	REACTOME_POST_TRANSLATIONAL_MODIFICATION:_SYNTHESIS_OF_GPI_ANCHORED_PROTEINS
6:133025715	*RPS12*	REACTOME_POST_TRANSLATIONAL_MODIFICATION:_SYNTHESIS_OF_GPI_ANCHORED_PROTEINS
6:133028870	*RPS12*	REACTOME_POST_TRANSLATIONAL_MODIFICATION:_SYNTHESIS_OF_GPI_ANCHORED_PROTEINS
6:133075265	*RPS12*	REACTOME_POST_TRANSLATIONAL_MODIFICATION:_SYNTHESIS_OF_GPI_ANCHORED_PROTEINS
11:132796757	*IGSF9B*	REACTOME_POST_TRANSLATIONAL_MODIFICATION:_SYNTHESIS_OF_GPI_ANCHORED_PROTEINS
11:132803872	*IGSF9B*	REACTOME_POST_TRANSLATIONAL_MODIFICATION:_SYNTHESIS_OF_GPI_ANCHORED_PROTEINS
11:132894570	*IGSF9B*	REACTOME_POST_TRANSLATIONAL_MODIFICATION:_SYNTHESIS_OF_GPI_ANCHORED_PROTEINS
11:132905763	*IGSF9B*	REACTOME_POST_TRANSLATIONAL_MODIFICATION:_SYNTHESIS_OF_GPI_ANCHORED_PROTEINS
11:132911017	*IGSF9B*	REACTOME_POST_TRANSLATIONAL_MODIFICATION:_SYNTHESIS_OF_GPI_ANCHORED_PROTEINS
11:132913867	*IGSF9B*	REACTOME_POST_TRANSLATIONAL_MODIFICATION:_SYNTHESIS_OF_GPI_ANCHORED_PROTEINS
11:132920409	*IGSF9B*	REACTOME_POST_TRANSLATIONAL_MODIFICATION:_SYNTHESIS_OF_GPI_ANCHORED_PROTEINS
11:132922730	*IGSF9B*	REACTOME_POST_TRANSLATIONAL_MODIFICATION:_SYNTHESIS_OF_GPI_ANCHORED_PROTEINS
11:132941181	*IGSF9B*	REACTOME_POST_TRANSLATIONAL_MODIFICATION:_SYNTHESIS_OF_GPI_ANCHORED_PROTEINS
11:133012999	*IGSF9B*	REACTOME_POST_TRANSLATIONAL_MODIFICATION:_SYNTHESIS_OF_GPI_ANCHORED_PROTEINS
11:133022507	*IGSF9B*	REACTOME_POST_TRANSLATIONAL_MODIFICATION:_SYNTHESIS_OF_GPI_ANCHORED_PROTEINS
11:133088981	*IGSF9B*	REACTOME_POST_TRANSLATIONAL_MODIFICATION:_SYNTHESIS_OF_GPI_ANCHORED_PROTEINS
11:133095792	*IGSF9B*	REACTOME_POST_TRANSLATIONAL_MODIFICATION:_SYNTHESIS_OF_GPI_ANCHORED_PROTEINS
11:133147495	*IGSF9B*	REACTOME_POST_TRANSLATIONAL_MODIFICATION:_SYNTHESIS_OF_GPI_ANCHORED_PROTEINS
11:133341504	*IGSF9B*	REACTOME_POST_TRANSLATIONAL_MODIFICATION:_SYNTHESIS_OF_GPI_ANCHORED_PROTEINS
1:156037357	*GBAP1*	REACTOME_POST_TRANSLATIONAL_PROTEIN_MODIFICATION
1:172386261	*VAMP4*	REACTOME_POST_TRANSLATIONAL_PROTEIN_MODIFICATION
1:172388441	*VAMP4*	REACTOME_POST_TRANSLATIONAL_PROTEIN_MODIFICATION
1:172389198	*VAMP4*	REACTOME_POST_TRANSLATIONAL_PROTEIN_MODIFICATION
1:226568665	*ITPKB*	REACTOME_POST_TRANSLATIONAL_PROTEIN_MODIFICATION
2:17941653	*KCNS3*	REACTOME_POST_TRANSLATIONAL_PROTEIN_MODIFICATION
2:17963171	*KCNS3*	REACTOME_POST_TRANSLATIONAL_PROTEIN_MODIFICATION
2:134902070	*TMEM163*	REACTOME_POST_TRANSLATIONAL_PROTEIN_MODIFICATION

2:134932210	*TMEM163*	REACTOME_POST_TRANSLATIONAL_PROTEIN_MODIFICATION
2:134956835	*TMEM163*	REACTOME_POST_TRANSLATIONAL_PROTEIN_MODIFICATION
2:134986578	*TMEM163*	REACTOME_POST_TRANSLATIONAL_PROTEIN_MODIFICATION
2:134993021	*TMEM163*	REACTOME_POST_TRANSLATIONAL_PROTEIN_MODIFICATION
2:135035038	*TMEM163*	REACTOME_POST_TRANSLATIONAL_PROTEIN_MODIFICATION
2:135040234	*TMEM163*	REACTOME_POST_TRANSLATIONAL_PROTEIN_MODIFICATION
2:135165078	*TMEM163*	REACTOME_POST_TRANSLATIONAL_PROTEIN_MODIFICATION
2:135166385	*TMEM163*	REACTOME_POST_TRANSLATIONAL_PROTEIN_MODIFICATION
3:18395010	*SATB1*	REACTOME_POST_TRANSLATIONAL_PROTEIN_MODIFICATION
3:18472577	*SATB1*	REACTOME_POST_TRANSLATIONAL_PROTEIN_MODIFICATION
3:48199695	*IP6K2*	REACTOME_POST_TRANSLATIONAL_PROTEIN_MODIFICATION
3:48214438	*IP6K2*	REACTOME_POST_TRANSLATIONAL_PROTEIN_MODIFICATION
3:49162284	*IP6K2*	REACTOME_POST_TRANSLATIONAL_PROTEIN_MODIFICATION
3:49162583	*IP6K2*	REACTOME_POST_TRANSLATIONAL_PROTEIN_MODIFICATION
3:49406095	*IP6K2*	REACTOME_POST_TRANSLATIONAL_PROTEIN_MODIFICATION
3:49424231	*IP6K2*	REACTOME_POST_TRANSLATIONAL_PROTEIN_MODIFICATION
3:49450137	*IP6K2*	REACTOME_POST_TRANSLATIONAL_PROTEIN_MODIFICATION
3:49462410	*IP6K2*	REACTOME_POST_TRANSLATIONAL_PROTEIN_MODIFICATION
3:49561208	*IP6K2*	REACTOME_POST_TRANSLATIONAL_PROTEIN_MODIFICATION
3:49740895	*IP6K2*	REACTOME_POST_TRANSLATIONAL_PROTEIN_MODIFICATION
3:121319489	*KPNA1*	REACTOME_POST_TRANSLATIONAL_PROTEIN_MODIFICATION
3:121324643	*KPNA1*	REACTOME_POST_TRANSLATIONAL_PROTEIN_MODIFICATION
3:121340955	*KPNA1*	REACTOME_POST_TRANSLATIONAL_PROTEIN_MODIFICATION
3:121411876	*KPNA1*	REACTOME_POST_TRANSLATIONAL_PROTEIN_MODIFICATION
3:121412702	*KPNA1*	REACTOME_POST_TRANSLATIONAL_PROTEIN_MODIFICATION
3:122645304	*KPNA1*	REACTOME_POST_TRANSLATIONAL_PROTEIN_MODIFICATION
3:122720608	*KPNA1*	REACTOME_POST_TRANSLATIONAL_PROTEIN_MODIFICATION
3:122986429	*KPNA1*	REACTOME_POST_TRANSLATIONAL_PROTEIN_MODIFICATION
3:182656610	*MCCC1*	REACTOME_POST_TRANSLATIONAL_PROTEIN_MODIFICATION
3:182976382	*MCCC1*	REACTOME_POST_TRANSLATIONAL_PROTEIN_MODIFICATION
3:182979912	*MCCC1*	REACTOME_POST_TRANSLATIONAL_PROTEIN_MODIFICATION
3:183005320	*MCCC1*	REACTOME_POST_TRANSLATIONAL_PROTEIN_MODIFICATION
4:15608677	*BST1*	REACTOME_POST_TRANSLATIONAL_PROTEIN_MODIFICATION
4:15618898	*BST1*	REACTOME_POST_TRANSLATIONAL_PROTEIN_MODIFICATION
4:15709252	*BST1*	REACTOME_POST_TRANSLATIONAL_PROTEIN_MODIFICATION
4:15713608	*BST1*	REACTOME_POST_TRANSLATIONAL_PROTEIN_MODIFICATION
4:15724372	*BST1*	REACTOME_POST_TRANSLATIONAL_PROTEIN_MODIFICATION
4:15737882	*BST1*	REACTOME_POST_TRANSLATIONAL_PROTEIN_MODIFICATION
4:76695927	*FAM47E-STBD1*	REACTOME_POST_TRANSLATIONAL_PROTEIN_MODIFICATION
4:76986566	*FAM47E-STBD1*	REACTOME_POST_TRANSLATIONAL_PROTEIN_MODIFICATION
4:77021433	*FAM47E-STBD1*	REACTOME_POST_TRANSLATIONAL_PROTEIN_MODIFICATION
4:113947327	*CAMK2D*	REACTOME_POST_TRANSLATIONAL_PROTEIN_MODIFICATION
4:114015252	*CAMK2D*	REACTOME_POST_TRANSLATIONAL_PROTEIN_MODIFICATION
4:114032570	*CAMK2D*	REACTOME_POST_TRANSLATIONAL_PROTEIN_MODIFICATION
4:114067233	*CAMK2D*	REACTOME_POST_TRANSLATIONAL_PROTEIN_MODIFICATION
4:114077386	*CAMK2D*	REACTOME_POST_TRANSLATIONAL_PROTEIN_MODIFICATION
4:114113772	*CAMK2D*	REACTOME_POST_TRANSLATIONAL_PROTEIN_MODIFICATION
4:114122398	*CAMK2D*	REACTOME_POST_TRANSLATIONAL_PROTEIN_MODIFICATION
4:114172749	*CAMK2D*	REACTOME_POST_TRANSLATIONAL_PROTEIN_MODIFICATION
4:114217997	*CAMK2D*	REACTOME_POST_TRANSLATIONAL_PROTEIN_MODIFICATION
4:114899005	*CAMK2D*	REACTOME_POST_TRANSLATIONAL_PROTEIN_MODIFICATION
5:133710339	*C5orf24*	REACTOME_POST_TRANSLATIONAL_PROTEIN_MODIFICATION
6:32151443	*HLA-DRB5*	REACTOME_POST_TRANSLATIONAL_PROTEIN_MODIFICATION

6:33287183	*HLA-DRB5*	REACTOME_POST_TRANSLATIONAL_PROTEIN_MODIFICATION
6:133025715	*RPS12*	REACTOME_POST_TRANSLATIONAL_PROTEIN_MODIFICATION
6:133028870	*RPS12*	REACTOME_POST_TRANSLATIONAL_PROTEIN_MODIFICATION
6:133075265	*RPS12*	REACTOME_POST_TRANSLATIONAL_PROTEIN_MODIFICATION
7:66096028	*GS1-124K5.11*	REACTOME_POST_TRANSLATIONAL_PROTEIN_MODIFICATION
7:66136281	*GS1-124K5.11*	REACTOME_POST_TRANSLATIONAL_PROTEIN_MODIFICATION
9:18502815	*SH3GL2_2*	REACTOME_POST_TRANSLATIONAL_PROTEIN_MODIFICATION
9:18522254	*SH3GL2_2*	REACTOME_POST_TRANSLATIONAL_PROTEIN_MODIFICATION
9:18583014	*SH3GL2_2*	REACTOME_POST_TRANSLATIONAL_PROTEIN_MODIFICATION
9:18623302	*SH3GL2_2*	REACTOME_POST_TRANSLATIONAL_PROTEIN_MODIFICATION
9:18633417	*SH3GL2_2*	REACTOME_POST_TRANSLATIONAL_PROTEIN_MODIFICATION
9:18663540	*SH3GL2_2*	REACTOME_POST_TRANSLATIONAL_PROTEIN_MODIFICATION
9:18721847	*SH3GL2_2*	REACTOME_POST_TRANSLATIONAL_PROTEIN_MODIFICATION
9:18725820	*SH3GL2_2*	REACTOME_POST_TRANSLATIONAL_PROTEIN_MODIFICATION
9:33845203	*UBAP2*	REACTOME_POST_TRANSLATIONAL_PROTEIN_MODIFICATION
10:103185542	*GBF1*	REACTOME_POST_TRANSLATIONAL_PROTEIN_MODIFICATION
10:103305087	*GBF1*	REACTOME_POST_TRANSLATIONAL_PROTEIN_MODIFICATION
10:103308244	*GBF1*	REACTOME_POST_TRANSLATIONAL_PROTEIN_MODIFICATION
10:104005410	*GBF1*	REACTOME_POST_TRANSLATIONAL_PROTEIN_MODIFICATION
10:104034550	*GBF1*	REACTOME_POST_TRANSLATIONAL_PROTEIN_MODIFICATION
10:104258303	*GBF1*	REACTOME_POST_TRANSLATIONAL_PROTEIN_MODIFICATION
10:121669571	*INPP5F*	REACTOME_POST_TRANSLATIONAL_PROTEIN_MODIFICATION
10:121683206	*INPP5F*	REACTOME_POST_TRANSLATIONAL_PROTEIN_MODIFICATION
10:121686714	*INPP5F*	REACTOME_POST_TRANSLATIONAL_PROTEIN_MODIFICATION
10:121689030	*INPP5F*	REACTOME_POST_TRANSLATIONAL_PROTEIN_MODIFICATION
11:11299050	*RNF141*	REACTOME_POST_TRANSLATIONAL_PROTEIN_MODIFICATION
11:11316049	*RNF141*	REACTOME_POST_TRANSLATIONAL_PROTEIN_MODIFICATION
11:11364660	*RNF141*	REACTOME_POST_TRANSLATIONAL_PROTEIN_MODIFICATION
11:11399582	*RNF141*	REACTOME_POST_TRANSLATIONAL_PROTEIN_MODIFICATION
11:11412180	*RNF141*	REACTOME_POST_TRANSLATIONAL_PROTEIN_MODIFICATION
11:11494181	*RNF141*	REACTOME_POST_TRANSLATIONAL_PROTEIN_MODIFICATION
11:11504200	*RNF141*	REACTOME_POST_TRANSLATIONAL_PROTEIN_MODIFICATION
11:11508780	*RNF141*	REACTOME_POST_TRANSLATIONAL_PROTEIN_MODIFICATION
11:11519365	*RNF141*	REACTOME_POST_TRANSLATIONAL_PROTEIN_MODIFICATION
11:82719121	*DLG2*	REACTOME_POST_TRANSLATIONAL_PROTEIN_MODIFICATION
11:132796757	*IGSF9B*	REACTOME_POST_TRANSLATIONAL_PROTEIN_MODIFICATION
11:132803872	*IGSF9B*	REACTOME_POST_TRANSLATIONAL_PROTEIN_MODIFICATION
11:132894570	*IGSF9B*	REACTOME_POST_TRANSLATIONAL_PROTEIN_MODIFICATION
11:132905763	*IGSF9B*	REACTOME_POST_TRANSLATIONAL_PROTEIN_MODIFICATION
11:132911017	*IGSF9B*	REACTOME_POST_TRANSLATIONAL_PROTEIN_MODIFICATION
11:132913867	*IGSF9B*	REACTOME_POST_TRANSLATIONAL_PROTEIN_MODIFICATION
11:132920409	*IGSF9B*	REACTOME_POST_TRANSLATIONAL_PROTEIN_MODIFICATION
11:132922730	*IGSF9B*	REACTOME_POST_TRANSLATIONAL_PROTEIN_MODIFICATION
11:132941181	*IGSF9B*	REACTOME_POST_TRANSLATIONAL_PROTEIN_MODIFICATION
11:133012999	*IGSF9B*	REACTOME_POST_TRANSLATIONAL_PROTEIN_MODIFICATION
11:133022507	*IGSF9B*	REACTOME_POST_TRANSLATIONAL_PROTEIN_MODIFICATION
11:133088981	*IGSF9B*	REACTOME_POST_TRANSLATIONAL_PROTEIN_MODIFICATION
11:133095792	*IGSF9B*	REACTOME_POST_TRANSLATIONAL_PROTEIN_MODIFICATION
11:133147495	*IGSF9B*	REACTOME_POST_TRANSLATIONAL_PROTEIN_MODIFICATION
11:133341504	*IGSF9B*	REACTOME_POST_TRANSLATIONAL_PROTEIN_MODIFICATION
12:132687184	*FBRSL1*	REACTOME_POST_TRANSLATIONAL_PROTEIN_MODIFICATION
14:56110265	*GCH1*	REACTOME_POST_TRANSLATIONAL_PROTEIN_MODIFICATION
14:76135546	*RPS6KL1*	REACTOME_POST_TRANSLATIONAL_PROTEIN_MODIFICATION

14:76159192	*RPS6KL1*	REACTOME_POST_TRANSLATIONAL_PROTEIN_MODIFICATION
14:76169229	*RPS6KL1*	REACTOME_POST_TRANSLATIONAL_PROTEIN_MODIFICATION
14:76170371	*RPS6KL1*	REACTOME_POST_TRANSLATIONAL_PROTEIN_MODIFICATION
14:76244271	*RPS6KL1*	REACTOME_POST_TRANSLATIONAL_PROTEIN_MODIFICATION
15:61016235	*VPS13C*	REACTOME_POST_TRANSLATIONAL_PROTEIN_MODIFICATION
15:61065568	*VPS13C*	REACTOME_POST_TRANSLATIONAL_PROTEIN_MODIFICATION
15:61094298	*VPS13C*	REACTOME_POST_TRANSLATIONAL_PROTEIN_MODIFICATION
15:61140736	*VPS13C*	REACTOME_POST_TRANSLATIONAL_PROTEIN_MODIFICATION
15:61142062	*VPS13C*	REACTOME_POST_TRANSLATIONAL_PROTEIN_MODIFICATION
15:61162571	*VPS13C*	REACTOME_POST_TRANSLATIONAL_PROTEIN_MODIFICATION
15:61191617	*VPS13C*	REACTOME_POST_TRANSLATIONAL_PROTEIN_MODIFICATION
15:61195427	*VPS13C*	REACTOME_POST_TRANSLATIONAL_PROTEIN_MODIFICATION
15:61221682	*VPS13C*	REACTOME_POST_TRANSLATIONAL_PROTEIN_MODIFICATION
15:61298486	*VPS13C*	REACTOME_POST_TRANSLATIONAL_PROTEIN_MODIFICATION
15:61375480	*VPS13C*	REACTOME_POST_TRANSLATIONAL_PROTEIN_MODIFICATION
15:61434581	*VPS13C*	REACTOME_POST_TRANSLATIONAL_PROTEIN_MODIFICATION
15:61467078	*VPS13C*	REACTOME_POST_TRANSLATIONAL_PROTEIN_MODIFICATION
15:61470808	*VPS13C*	REACTOME_POST_TRANSLATIONAL_PROTEIN_MODIFICATION
15:61500263	*VPS13C*	REACTOME_POST_TRANSLATIONAL_PROTEIN_MODIFICATION
16:19562008	*SYT17*	REACTOME_POST_TRANSLATIONAL_PROTEIN_MODIFICATION
16:50730088	*CASC16*	REACTOME_POST_TRANSLATIONAL_PROTEIN_MODIFICATION
16:50730446	*CASC16*	REACTOME_POST_TRANSLATIONAL_PROTEIN_MODIFICATION
16:50745926	*CASC16*	REACTOME_POST_TRANSLATIONAL_PROTEIN_MODIFICATION
16:50790891	*CASC16*	REACTOME_POST_TRANSLATIONAL_PROTEIN_MODIFICATION
17:7491546	*V1NB1*	REACTOME_POST_TRANSLATIONAL_PROTEIN_MODIFICATION
17:7578671	*V1NB1*	REACTOME_POST_TRANSLATIONAL_PROTEIN_MODIFICATION
17:44800946	*WNT3*	REACTOME_POST_TRANSLATIONAL_PROTEIN_MODIFICATION
17:44833217	*WNT3*	REACTOME_POST_TRANSLATIONAL_PROTEIN_MODIFICATION
17:45020426	*WNT3*	REACTOME_POST_TRANSLATIONAL_PROTEIN_MODIFICATION
17:45059032	*WNT3*	REACTOME_POST_TRANSLATIONAL_PROTEIN_MODIFICATION
17:45089680	*WNT3*	REACTOME_POST_TRANSLATIONAL_PROTEIN_MODIFICATION
1:154701406	*GBAP1*	REACTOME_POTASSIUM_CHANNELS
1:154776412	*GBAP1*	REACTOME_POTASSIUM_CHANNELS
1:154801670	*GBAP1*	REACTOME_POTASSIUM_CHANNELS
1:154804988	*GBAP1*	REACTOME_POTASSIUM_CHANNELS
2:18091890	*KCNS3*	REACTOME_POTASSIUM_CHANNELS
2:18130187	*KCNS3*	REACTOME_POTASSIUM_CHANNELS
2:18141542	*KCNS3*	REACTOME_POTASSIUM_CHANNELS
2:18143082	*KCNS3*	REACTOME_POTASSIUM_CHANNELS
2:18176120	*KCNS3*	REACTOME_POTASSIUM_CHANNELS
2:18247777	*KCNS3*	REACTOME_POTASSIUM_CHANNELS
2:18299564	*KCNS3*	REACTOME_POTASSIUM_CHANNELS
2:18315537	*KCNS3*	REACTOME_POTASSIUM_CHANNELS
2:18335515	*KCNS3*	REACTOME_POTASSIUM_CHANNELS
2:18455049	*KCNS3*	REACTOME_POTASSIUM_CHANNELS
2:18472861	*KCNS3*	REACTOME_POTASSIUM_CHANNELS
3:19236100	*SATB1*	REACTOME_POTASSIUM_CHANNELS
3:19312697	*SATB1*	REACTOME_POTASSIUM_CHANNELS
3:19329968	*SATB1*	REACTOME_POTASSIUM_CHANNELS
3:19334895	*SATB1*	REACTOME_POTASSIUM_CHANNELS
3:19352476	*SATB1*	REACTOME_POTASSIUM_CHANNELS
6:29565889	*TRIM40*	REACTOME_POTASSIUM_CHANNELS
6:29590715	*TRIM40*	REACTOME_POTASSIUM_CHANNELS

6:73383348	*RIMS1*	REACTOME_POTASSIUM_CHANNELS
6:73448086	*RIMS1*	REACTOME_POTASSIUM_CHANNELS
14:88681675	*GALC*	REACTOME_POTASSIUM_CHANNELS
14:88709038	*GALC*	REACTOME_POTASSIUM_CHANNELS
14:88738350	*GALC*	REACTOME_POTASSIUM_CHANNELS
14:88769689	*GALC*	REACTOME_POTASSIUM_CHANNELS
14:88778263	*GALC*	REACTOME_POTASSIUM_CHANNELS
17:7832034	*V1NB1*	REACTOME_POTASSIUM_CHANNELS
19:2557113	*SPPL2B*	REACTOME_POTASSIUM_CHANNELS
19:2563327	*SPPL2B*	REACTOME_POTASSIUM_CHANNELS
19:2580057	*SPPL2B*	REACTOME_POTASSIUM_CHANNELS
19:2660983	*SPPL2B*	REACTOME_POTASSIUM_CHANNELS
21:38984964	*DYRK1A*	REACTOME_POTASSIUM_CHANNELS
21:39018136	*DYRK1A*	REACTOME_POTASSIUM_CHANNELS
21:39021814	*DYRK1A*	REACTOME_POTASSIUM_CHANNELS
21:39039956	*DYRK1A*	REACTOME_POTASSIUM_CHANNELS
21:39088785	*DYRK1A*	REACTOME_POTASSIUM_CHANNELS
21:39108160	*DYRK1A*	REACTOME_POTASSIUM_CHANNELS
21:39120480	*DYRK1A*	REACTOME_POTASSIUM_CHANNELS
21:39134980	*DYRK1A*	REACTOME_POTASSIUM_CHANNELS
21:39197323	*DYRK1A*	REACTOME_POTASSIUM_CHANNELS
21:39217472	*DYRK1A*	REACTOME_POTASSIUM_CHANNELS
5:133558885	*C5orf24*	REACTOME_PP2A_MEDIATED_DEPHOSPHORYLATION_OF_KEY_METABOLIC_FACTORS
6:33381459	*HLA-DRB5*	REACTOME_PRC2_METHYLATES_HISTONES_AND_DNA
6:32179319	*HLA-DRB5*	REACTOME_PRE_NOTCH_EXPRESSION_AND_PROCESSING
6:32186193	*HLA-DRB5*	REACTOME_PRE_NOTCH_EXPRESSION_AND_PROCESSING
17:7578671	*V1NB1*	REACTOME_PRE_NOTCH_EXPRESSION_AND_PROCESSING
6:32179319	*HLA-DRB5*	REACTOME_PRE_NOTCH_PROCESSING_IN_GOLGI
6:32186193	*HLA-DRB5*	REACTOME_PRE_NOTCH_PROCESSING_IN_GOLGI
6:32179319	*HLA-DRB5*	REACTOME_PRE_NOTCH_PROCESSING_IN_THE_ENDOPLASMIC_RETICULUM
6:32186193	*HLA-DRB5*	REACTOME_PRE_NOTCH_PROCESSING_IN_THE_ENDOPLASMIC_RETICULUM
19:2557113	*SPPL2B*	REACTOME_PRESYNAPTIC_FUNCTION_OF_KAINATE_RECEPTORS
19:2563327	*SPPL2B*	REACTOME_PRESYNAPTIC_FUNCTION_OF_KAINATE_RECEPTORS
19:2580057	*SPPL2B*	REACTOME_PRESYNAPTIC_FUNCTION_OF_KAINATE_RECEPTORS
19:2660983	*SPPL2B*	REACTOME_PRESYNAPTIC_FUNCTION_OF_KAINATE_RECEPTORS
5:134132269	*C5orf24*	REACTOME_PROCESSING_OF_CAPPED_INTRON_CONTAINING_PRE_MRNA
6:30628082	*TRIM40*	REACTOME_PROCESSING_OF_CAPPED_INTRON_CONTAINING_PRE_MRNA
6:30631063	*TRIM40*	REACTOME_PROCESSING_OF_CAPPED_INTRON_CONTAINING_PRE_MRNA
6:30639369	*TRIM40*	REACTOME_PROCESSING_OF_CAPPED_INTRON_CONTAINING_PRE_MRNA
17:42929985	*CRHR1_2*	REACTOME_PROCESSING_OF_CAPPED_INTRON_CONTAINING_PRE_MRNA
17:59791813	*BRIP1*	REACTOME_PROCESSING_OF_DNA_DOUBLE_STRAND_BREAK_ENDS
17:59922925	*BRIP1*	REACTOME_PROCESSING_OF_DNA_DOUBLE_STRAND_BREAK_ENDS
8:16929685	*FGF20*	REACTOME_PROCESSING_OF_SMDT1
8:16967392	*FGF20*	REACTOME_PROCESSING_OF_SMDT1
8:16971257	*FGF20*	REACTOME_PROCESSING_OF_SMDT1
1:156059276	*GBAP1*	REACTOME_PROGRAMMED_CELL_DEATH
3:18395010	*SATB1*	REACTOME_PROGRAMMED_CELL_DEATH
3:18472577	*SATB1*	REACTOME_PROGRAMMED_CELL_DEATH
3:122200170	*KPNA1*	REACTOME_PROGRAMMED_CELL_DEATH
8:23050400	*BIN3*	REACTOME_PROGRAMMED_CELL_DEATH
17:7578671	*V1NB1*	REACTOME_PROGRAMMED_CELL_DEATH
17:43181287	*CRHR1_2*	REACTOME_PROGRAMMED_CELL_DEATH
17:43184627	*CRHR1_2*	REACTOME_PROGRAMMED_CELL_DEATH

17:43999203	*WNT3*	REACTOME_PROGRAMMED_CELL_DEATH
17:44005297	*WNT3*	REACTOME_PROGRAMMED_CELL_DEATH
17:44019712	*WNT3*	REACTOME_PROGRAMMED_CELL_DEATH
17:44060492	*WNT3*	REACTOME_PROGRAMMED_CELL_DEATH
17:44065263	*WNT3*	REACTOME_PROGRAMMED_CELL_DEATH
17:44081178	*WNT3*	REACTOME_PROGRAMMED_CELL_DEATH
17:44083297	*WNT3*	REACTOME_PROGRAMMED_CELL_DEATH
10:103185542	*GBF1*	REACTOME_PROLACTIN_RECEPTOR_SIGNALING
10:103305087	*GBF1*	REACTOME_PROLACTIN_RECEPTOR_SIGNALING
10:103308244	*GBF1*	REACTOME_PROLACTIN_RECEPTOR_SIGNALING
17:40392358	*RETREG3*	REACTOME_PROLACTIN_RECEPTOR_SIGNALING
19:2557113	*SPPL2B*	REACTOME_PROSTACYCLIN_SIGNALLING_THROUGH_PROSTACYCLIN_RECEPTOR
19:2563327	*SPPL2B*	REACTOME_PROSTACYCLIN_SIGNALLING_THROUGH_PROSTACYCLIN_RECEPTOR
19:2580057	*SPPL2B*	REACTOME_PROSTACYCLIN_SIGNALLING_THROUGH_PROSTACYCLIN_RECEPTOR
19:2660983	*SPPL2B*	REACTOME_PROSTACYCLIN_SIGNALLING_THROUGH_PROSTACYCLIN_RECEPTOR
1:155205634	*GBAP1*	REACTOME_PROTEIN_FOLDING
4:15608677	*BST1*	REACTOME_PROTEIN_FOLDING
4:15618898	*BST1*	REACTOME_PROTEIN_FOLDING
19:2557113	*SPPL2B*	REACTOME_PROTEIN_FOLDING
19:2563327	*SPPL2B*	REACTOME_PROTEIN_FOLDING
19:2580057	*SPPL2B*	REACTOME_PROTEIN_FOLDING
19:2660983	*SPPL2B*	REACTOME_PROTEIN_FOLDING
1:155181232	*GBAP1*	REACTOME_PROTEIN_LOCALIZATION
3:150298317	*MED12L*	REACTOME_PROTEIN_LOCALIZATION
3:150303590	*MED12L*	REACTOME_PROTEIN_LOCALIZATION
6:31607942	*HLA-DRB5*	REACTOME_PROTEIN_LOCALIZATION
12:39960237	*LRRK2_2*	REACTOME_PROTEIN_LOCALIZATION
12:133295501	*FBRSL1*	REACTOME_PROTEIN_LOCALIZATION
19:2771641	*SPPL2B*	REACTOME_PROTEIN_LOCALIZATION
11:83219006	*DLG2*	REACTOME_PROTEIN_PROTEIN_INTERACTIONS_AT_SYNAPSES
11:83232915	*DLG2*	REACTOME_PROTEIN_PROTEIN_INTERACTIONS_AT_SYNAPSES
11:83282740	*DLG2*	REACTOME_PROTEIN_PROTEIN_INTERACTIONS_AT_SYNAPSES
11:83387273	*DLG2*	REACTOME_PROTEIN_PROTEIN_INTERACTIONS_AT_SYNAPSES
11:83538733	*DLG2*	REACTOME_PROTEIN_PROTEIN_INTERACTIONS_AT_SYNAPSES
11:83544472	*DLG2*	REACTOME_PROTEIN_PROTEIN_INTERACTIONS_AT_SYNAPSES
11:83578152	*DLG2*	REACTOME_PROTEIN_PROTEIN_INTERACTIONS_AT_SYNAPSES
11:83647657	*DLG2*	REACTOME_PROTEIN_PROTEIN_INTERACTIONS_AT_SYNAPSES
11:83669903	*DLG2*	REACTOME_PROTEIN_PROTEIN_INTERACTIONS_AT_SYNAPSES
11:83736246	*DLG2*	REACTOME_PROTEIN_PROTEIN_INTERACTIONS_AT_SYNAPSES
11:83762918	*DLG2*	REACTOME_PROTEIN_PROTEIN_INTERACTIONS_AT_SYNAPSES
11:83823752	*DLG2*	REACTOME_PROTEIN_PROTEIN_INTERACTIONS_AT_SYNAPSES
11:83839992	*DLG2*	REACTOME_PROTEIN_PROTEIN_INTERACTIONS_AT_SYNAPSES
11:84104763	*DLG2*	REACTOME_PROTEIN_PROTEIN_INTERACTIONS_AT_SYNAPSES
11:84169039	*DLG2*	REACTOME_PROTEIN_PROTEIN_INTERACTIONS_AT_SYNAPSES
11:84221628	*DLG2*	REACTOME_PROTEIN_PROTEIN_INTERACTIONS_AT_SYNAPSES
11:84312008	*DLG2*	REACTOME_PROTEIN_PROTEIN_INTERACTIONS_AT_SYNAPSES
11:84324361	*DLG2*	REACTOME_PROTEIN_PROTEIN_INTERACTIONS_AT_SYNAPSES
11:84360346	*DLG2*	REACTOME_PROTEIN_PROTEIN_INTERACTIONS_AT_SYNAPSES
11:84403409	*DLG2*	REACTOME_PROTEIN_PROTEIN_INTERACTIONS_AT_SYNAPSES
17:7101771	*V1NB1*	REACTOME_PROTEIN_PROTEIN_INTERACTIONS_AT_SYNAPSES
5:133710339	*C5orf24*	REACTOME_PROTEIN_UBIQUITINATION
9:33845203	*UBAP2*	REACTOME_PROTEIN_UBIQUITINATION
17:7578671	*V1NB1*	REACTOME_PTEN_REGULATION

7:23277372	*GPNMB*	REACTOME_PTK6_PROMOTES_HIF1A_STABILIZATION
7:23282960	*GPNMB*	REACTOME_PTK6_PROMOTES_HIF1A_STABILIZATION
7:23293746	*GPNMB*	REACTOME_PTK6_PROMOTES_HIF1A_STABILIZATION
7:23313171	*GPNMB*	REACTOME_PTK6_PROMOTES_HIF1A_STABILIZATION
12:40614434	*LRRK2_2*	REACTOME_PTK6_PROMOTES_HIF1A_STABILIZATION
12:40614656	*LRRK2_2*	REACTOME_PTK6_PROMOTES_HIF1A_STABILIZATION
12:40617231	*LRRK2_2*	REACTOME_PTK6_PROMOTES_HIF1A_STABILIZATION
12:40653993	*LRRK2_2*	REACTOME_PTK6_PROMOTES_HIF1A_STABILIZATION
12:40698422	*LRRK2_2*	REACTOME_PTK6_PROMOTES_HIF1A_STABILIZATION
12:40707861	*LRRK2_2*	REACTOME_PTK6_PROMOTES_HIF1A_STABILIZATION
12:40734202	*LRRK2_2*	REACTOME_PTK6_PROMOTES_HIF1A_STABILIZATION
12:40753796	*LRRK2_2*	REACTOME_PTK6_PROMOTES_HIF1A_STABILIZATION
3:49406095	*IP6K2*	REACTOME_PTK6_REGULATES_RHO_GTPASES_RAS_GTPASE_AND_MAP_KINASES
3:49424231	*IP6K2*	REACTOME_PTK6_REGULATES_RHO_GTPASES_RAS_GTPASE_AND_MAP_KINASES
3:49450137	*IP6K2*	REACTOME_PTK6_REGULATES_RHO_GTPASES_RAS_GTPASE_AND_MAP_KINASES
10:104847713	*GBF1*	REACTOME_PURINE_CATABOLISM
10:104861736	*GBF1*	REACTOME_PURINE_CATABOLISM
10:104926485	*GBF1*	REACTOME_PURINE_CATABOLISM
10:104929716	*GBF1*	REACTOME_PURINE_CATABOLISM
11:10392432	*RNF141*	REACTOME_PURINE_SALVAGE
11:10429998	*RNF141*	REACTOME_PURINE_SALVAGE
11:10458098	*RNF141*	REACTOME_PURINE_SALVAGE
11:10471738	*RNF141*	REACTOME_PURINE_SALVAGE
11:10487997	*RNF141*	REACTOME_PURINE_SALVAGE
11:10494132	*RNF141*	REACTOME_PURINE_SALVAGE
17:76177567	*DNAH17*	REACTOME_PYRIMIDINE_SALVAGE
2:135921205	*TMEM163*	REACTOME_RAB_GEFS_EXCHANGE_GTP_FOR_GDP_ON_RABS
1:156037357	*GBAP1*	REACTOME_RAB_GERANYLGERANYLATION
11:82719121	*DLG2*	REACTOME_RAB_GERANYLGERANYLATION
2:135921205	*TMEM163*	REACTOME_RAB_REGULATION_OF_TRAFFICKING
4:114424939	*CAMK2D*	REACTOME_RAF_ACTIVATION
4:114494052	*CAMK2D*	REACTOME_RAF_ACTIVATION
4:114529492	*CAMK2D*	REACTOME_RAF_ACTIVATION
4:114539964	*CAMK2D*	REACTOME_RAF_ACTIVATION
4:114597918	*CAMK2D*	REACTOME_RAF_ACTIVATION
5:133558885	*C5orf24*	REACTOME_RAF_ACTIVATION
4:114424939	*CAMK2D*	REACTOME_RAS_ACTIVATION_UPON_CA2PLUS_INFLUX_THROUGH_NMDA_RECEPTOR
4:114494052	*CAMK2D*	REACTOME_RAS_ACTIVATION_UPON_CA2PLUS_INFLUX_THROUGH_NMDA_RECEPTOR
4:114529492	*CAMK2D*	REACTOME_RAS_ACTIVATION_UPON_CA2PLUS_INFLUX_THROUGH_NMDA_RECEPTOR
4:114539964	*CAMK2D*	REACTOME_RAS_ACTIVATION_UPON_CA2PLUS_INFLUX_THROUGH_NMDA_RECEPTOR
4:114597918	*CAMK2D*	REACTOME_RAS_ACTIVATION_UPON_CA2PLUS_INFLUX_THROUGH_NMDA_RECEPTOR
11:83219006	*DLG2*	REACTOME_RAS_ACTIVATION_UPON_CA2PLUS_INFLUX_THROUGH_NMDA_RECEPTOR
11:83232915	*DLG2*	REACTOME_RAS_ACTIVATION_UPON_CA2PLUS_INFLUX_THROUGH_NMDA_RECEPTOR
11:83282740	*DLG2*	REACTOME_RAS_ACTIVATION_UPON_CA2PLUS_INFLUX_THROUGH_NMDA_RECEPTOR
11:83387273	*DLG2*	REACTOME_RAS_ACTIVATION_UPON_CA2PLUS_INFLUX_THROUGH_NMDA_RECEPTOR
11:83538733	*DLG2*	REACTOME_RAS_ACTIVATION_UPON_CA2PLUS_INFLUX_THROUGH_NMDA_RECEPTOR
11:83544472	*DLG2*	REACTOME_RAS_ACTIVATION_UPON_CA2PLUS_INFLUX_THROUGH_NMDA_RECEPTOR
11:83578152	*DLG2*	REACTOME_RAS_ACTIVATION_UPON_CA2PLUS_INFLUX_THROUGH_NMDA_RECEPTOR
11:83647657	*DLG2*	REACTOME_RAS_ACTIVATION_UPON_CA2PLUS_INFLUX_THROUGH_NMDA_RECEPTOR
11:83669903	*DLG2*	REACTOME_RAS_ACTIVATION_UPON_CA2PLUS_INFLUX_THROUGH_NMDA_RECEPTOR
11:83736246	*DLG2*	REACTOME_RAS_ACTIVATION_UPON_CA2PLUS_INFLUX_THROUGH_NMDA_RECEPTOR
11:83762918	*DLG2*	REACTOME_RAS_ACTIVATION_UPON_CA2PLUS_INFLUX_THROUGH_NMDA_RECEPTOR
11:83823752	*DLG2*	REACTOME_RAS_ACTIVATION_UPON_CA2PLUS_INFLUX_THROUGH_NMDA_RECEPTOR

11:83839992	*DLG2*	REACTOME_RAS_ACTIVATION_UPON_CA2PLUS_INFLUX_THROUGH_NMDA_RECEPTOR
11:84104763	*DLG2*	REACTOME_RAS_ACTIVATION_UPON_CA2PLUS_INFLUX_THROUGH_NMDA_RECEPTOR
11:84169039	*DLG2*	REACTOME_RAS_ACTIVATION_UPON_CA2PLUS_INFLUX_THROUGH_NMDA_RECEPTOR
11:84221628	*DLG2*	REACTOME_RAS_ACTIVATION_UPON_CA2PLUS_INFLUX_THROUGH_NMDA_RECEPTOR
11:84312008	*DLG2*	REACTOME_RAS_ACTIVATION_UPON_CA2PLUS_INFLUX_THROUGH_NMDA_RECEPTOR
11:84324361	*DLG2*	REACTOME_RAS_ACTIVATION_UPON_CA2PLUS_INFLUX_THROUGH_NMDA_RECEPTOR
11:84360346	*DLG2*	REACTOME_RAS_ACTIVATION_UPON_CA2PLUS_INFLUX_THROUGH_NMDA_RECEPTOR
11:84403409	*DLG2*	REACTOME_RAS_ACTIVATION_UPON_CA2PLUS_INFLUX_THROUGH_NMDA_RECEPTOR
17:7101771	*V1NB1*	REACTOME_RAS_ACTIVATION_UPON_CA2PLUS_INFLUX_THROUGH_NMDA_RECEPTOR
12:133295501	*FBRSL1*	REACTOME_RECEPTOR_MEDIATED_MITOPHAGY
5:133710339	*C5orf24*	REACTOME_RECOGNITION_OF_DNA_DAMAGE_BY_PCNA_CONTAINING_REPLICATION_COMPLEX
12:133206648	*FBRSL1*	REACTOME_RECOGNITION_OF_DNA_DAMAGE_BY_PCNA_CONTAINING_REPLICATION_COMPLEX
12:133223685	*FBRSL1*	REACTOME_RECOGNITION_OF_DNA_DAMAGE_BY_PCNA_CONTAINING_REPLICATION_COMPLEX
10:104258303	*GBF1*	REACTOME_RECRUITMENT_OF_NUMA_TO_MITOTIC_CENTROSOMES
16:19562008	*SYT17*	REACTOME_RECRUITMENT_OF_NUMA_TO_MITOTIC_CENTROSOMES
17:40818584	*RETREG3*	REACTOME_RECRUITMENT_OF_NUMA_TO_MITOTIC_CENTROSOMES
1:171837985	*VAMP4*	REACTOME_RECYCLING_PATHWAY_OF_L1
1:171945488	*VAMP4*	REACTOME_RECYCLING_PATHWAY_OF_L1
1:172137119	*VAMP4*	REACTOME_RECYCLING_PATHWAY_OF_L1
1:172386261	*VAMP4*	REACTOME_RECYCLING_PATHWAY_OF_L1
9:17579690	*SH3GL2_2*	REACTOME_RECYCLING_PATHWAY_OF_L1
9:17587160	*SH3GL2_2*	REACTOME_RECYCLING_PATHWAY_OF_L1
9:17609302	*SH3GL2_2*	REACTOME_RECYCLING_PATHWAY_OF_L1
9:17626762	*SH3GL2_2*	REACTOME_RECYCLING_PATHWAY_OF_L1
9:17627444	*SH3GL2_2*	REACTOME_RECYCLING_PATHWAY_OF_L1
9:17642664	*SH3GL2_2*	REACTOME_RECYCLING_PATHWAY_OF_L1
9:17710876	*SH3GL2_2*	REACTOME_RECYCLING_PATHWAY_OF_L1
9:17732590	*SH3GL2_2*	REACTOME_RECYCLING_PATHWAY_OF_L1
9:17737514	*SH3GL2_2*	REACTOME_RECYCLING_PATHWAY_OF_L1
9:17758945	*SH3GL2_2*	REACTOME_RECYCLING_PATHWAY_OF_L1
6:112137847	*FYN*	REACTOME_REELIN_SIGNALLING_PATHWAY
6:112151452	*FYN*	REACTOME_REELIN_SIGNALLING_PATHWAY
6:112175069	*FYN*	REACTOME_REELIN_SIGNALLING_PATHWAY
8:23050400	*BIN3*	REACTOME_REGULATED_NECROSIS
1:227067656	*ITPKB*	REACTOME_REGULATED_PROTEOLYSIS_OF_P75NTR
8:23050400	*BIN3*	REACTOME_REGULATION_BY_C_FLIP
8:11679472	*CTSB*	REACTOME_REGULATION_OF_CHOLESTEROL_BIOSYNTHESIS_BY_SREBP_SREBF
3:49561208	*IP6K2*	REACTOME_REGULATION_OF_EXPRESSION_OF_SLITS_AND_ROBOS
5:133558885	*C5orf24*	REACTOME_REGULATION_OF_GLYCOLYSIS_BY_FRUCTOSE_2_6_BISPHOSPHATE_METABOLISM
10:14908849	*ITGA8*	REACTOME_REGULATION_OF_HSF1_MEDIATED_HEAT_SHOCK_RESPONSE
10:121429633	*INPP5F*	REACTOME_REGULATION_OF_HSF1_MEDIATED_HEAT_SHOCK_RESPONSE
10:121437329	*INPP5F*	REACTOME_REGULATION_OF_HSF1_MEDIATED_HEAT_SHOCK_RESPONSE
17:40132591	*RETREG3*	REACTOME_REGULATION_OF_HSF1_MEDIATED_HEAT_SHOCK_RESPONSE
3:49162284	*IP6K2*	ULATION_OF_INSULIN_LIKE_GROWTH_FACTOR_IGF_TRANSPORT_AND_UPTAKE_BY_INSULIN_LIKE_GROWTH_FACTOR_BINDIN
3:49162583	*IP6K2*	ULATION_OF_INSULIN_LIKE_GROWTH_FACTOR_IGF_TRANSPORT_AND_UPTAKE_BY_INSULIN_LIKE_GROWTH_FACTOR_BINDIN
14:56110265	*GCH1*	ULATION_OF_INSULIN_LIKE_GROWTH_FACTOR_IGF_TRANSPORT_AND_UPTAKE_BY_INSULIN_LIKE_GROWTH_FACTOR_BINDIN
2:18091890	*KCNS3*	REACTOME_REGULATION_OF_INSULIN_SECRETION
2:18130187	*KCNS3*	REACTOME_REGULATION_OF_INSULIN_SECRETION
2:18141542	*KCNS3*	REACTOME_REGULATION_OF_INSULIN_SECRETION
2:18143082	*KCNS3*	REACTOME_REGULATION_OF_INSULIN_SECRETION
2:18176120	*KCNS3*	REACTOME_REGULATION_OF_INSULIN_SECRETION
2:18247777	*KCNS3*	REACTOME_REGULATION_OF_INSULIN_SECRETION
2:18299564	*KCNS3*	REACTOME_REGULATION_OF_INSULIN_SECRETION

2:18315537	*KCNS3*	REACTOME_REGULATION_OF_INSULIN_SECRETION
2:18335515	*KCNS3*	REACTOME_REGULATION_OF_INSULIN_SECRETION
2:18455049	*KCNS3*	REACTOME_REGULATION_OF_INSULIN_SECRETION
2:18472861	*KCNS3*	REACTOME_REGULATION_OF_INSULIN_SECRETION
3:48835245	*IP6K2*	REACTOME_REGULATION_OF_INSULIN_SECRETION
3:123089448	*KPNA1*	REACTOME_REGULATION_OF_INSULIN_SECRETION
8:131799767	*FAM49B*	REACTOME_REGULATION_OF_INSULIN_SECRETION
8:131845512	*FAM49B*	REACTOME_REGULATION_OF_INSULIN_SECRETION
8:131865425	*FAM49B*	REACTOME_REGULATION_OF_INSULIN_SECRETION
8:131901170	*FAM49B*	REACTOME_REGULATION_OF_INSULIN_SECRETION
19:2557113	*SPPL2B*	REACTOME_REGULATION_OF_INSULIN_SECRETION
19:2563327	*SPPL2B*	REACTOME_REGULATION_OF_INSULIN_SECRETION
19:2580057	*SPPL2B*	REACTOME_REGULATION_OF_INSULIN_SECRETION
19:2660983	*SPPL2B*	REACTOME_REGULATION_OF_INSULIN_SECRETION
19:3142784	*SPPL2B*	REACTOME_REGULATION_OF_INSULIN_SECRETION
19:3147326	*SPPL2B*	REACTOME_REGULATION_OF_INSULIN_SECRETION
6:112137847	*FYN*	REACTOME_REGULATION_OF_KIT_SIGNALING
6:112151452	*FYN*	REACTOME_REGULATION_OF_KIT_SIGNALING
6:112175069	*FYN*	REACTOME_REGULATION_OF_KIT_SIGNALING
2:101439596	*MAP4K4*	EACTOME_REGULATION_OF_LIPID_METABOLISM_BY_PEROXISOME_PROLIFERATOR_ACTIVATED_RECEPTOR_ALPHA_PPARALP
2:101464631	*MAP4K4*	EACTOME_REGULATION_OF_LIPID_METABOLISM_BY_PEROXISOME_PROLIFERATOR_ACTIVATED_RECEPTOR_ALPHA_PPARALP
2:101547064	*MAP4K4*	EACTOME_REGULATION_OF_LIPID_METABOLISM_BY_PEROXISOME_PROLIFERATOR_ACTIVATED_RECEPTOR_ALPHA_PPARALP
2:101550181	*MAP4K4*	EACTOME_REGULATION_OF_LIPID_METABOLISM_BY_PEROXISOME_PROLIFERATOR_ACTIVATED_RECEPTOR_ALPHA_PPARALP
2:101578010	*MAP4K4*	EACTOME_REGULATION_OF_LIPID_METABOLISM_BY_PEROXISOME_PROLIFERATOR_ACTIVATED_RECEPTOR_ALPHA_PPARALP
2:101603812	*MAP4K4*	EACTOME_REGULATION_OF_LIPID_METABOLISM_BY_PEROXISOME_PROLIFERATOR_ACTIVATED_RECEPTOR_ALPHA_PPARALP
4:17622910	*LCORL*	EACTOME_REGULATION_OF_LIPID_METABOLISM_BY_PEROXISOME_PROLIFERATOR_ACTIVATED_RECEPTOR_ALPHA_PPARALP
8:11679472	*CTSB*	EACTOME_REGULATION_OF_LIPID_METABOLISM_BY_PEROXISOME_PROLIFERATOR_ACTIVATED_RECEPTOR_ALPHA_PPARALP
15:61016235	*VPS13C*	EACTOME_REGULATION_OF_LIPID_METABOLISM_BY_PEROXISOME_PROLIFERATOR_ACTIVATED_RECEPTOR_ALPHA_PPARALP
15:61065568	*VPS13C*	EACTOME_REGULATION_OF_LIPID_METABOLISM_BY_PEROXISOME_PROLIFERATOR_ACTIVATED_RECEPTOR_ALPHA_PPARALP
15:61094298	*VPS13C*	EACTOME_REGULATION_OF_LIPID_METABOLISM_BY_PEROXISOME_PROLIFERATOR_ACTIVATED_RECEPTOR_ALPHA_PPARALP
15:61140736	*VPS13C*	EACTOME_REGULATION_OF_LIPID_METABOLISM_BY_PEROXISOME_PROLIFERATOR_ACTIVATED_RECEPTOR_ALPHA_PPARALP
15:61142062	*VPS13C*	EACTOME_REGULATION_OF_LIPID_METABOLISM_BY_PEROXISOME_PROLIFERATOR_ACTIVATED_RECEPTOR_ALPHA_PPARALP
15:61162571	*VPS13C*	EACTOME_REGULATION_OF_LIPID_METABOLISM_BY_PEROXISOME_PROLIFERATOR_ACTIVATED_RECEPTOR_ALPHA_PPARALP
15:61191617	*VPS13C*	EACTOME_REGULATION_OF_LIPID_METABOLISM_BY_PEROXISOME_PROLIFERATOR_ACTIVATED_RECEPTOR_ALPHA_PPARALP
15:61195427	*VPS13C*	EACTOME_REGULATION_OF_LIPID_METABOLISM_BY_PEROXISOME_PROLIFERATOR_ACTIVATED_RECEPTOR_ALPHA_PPARALP
15:61221682	*VPS13C*	EACTOME_REGULATION_OF_LIPID_METABOLISM_BY_PEROXISOME_PROLIFERATOR_ACTIVATED_RECEPTOR_ALPHA_PPARALP
15:61298486	*VPS13C*	EACTOME_REGULATION_OF_LIPID_METABOLISM_BY_PEROXISOME_PROLIFERATOR_ACTIVATED_RECEPTOR_ALPHA_PPARALP
15:61375480	*VPS13C*	EACTOME_REGULATION_OF_LIPID_METABOLISM_BY_PEROXISOME_PROLIFERATOR_ACTIVATED_RECEPTOR_ALPHA_PPARALP
15:61434581	*VPS13C*	EACTOME_REGULATION_OF_LIPID_METABOLISM_BY_PEROXISOME_PROLIFERATOR_ACTIVATED_RECEPTOR_ALPHA_PPARALP
15:61467078	*VPS13C*	EACTOME_REGULATION_OF_LIPID_METABOLISM_BY_PEROXISOME_PROLIFERATOR_ACTIVATED_RECEPTOR_ALPHA_PPARALP
15:61470808	*VPS13C*	EACTOME_REGULATION_OF_LIPID_METABOLISM_BY_PEROXISOME_PROLIFERATOR_ACTIVATED_RECEPTOR_ALPHA_PPARALP
15:61500263	*VPS13C*	EACTOME_REGULATION_OF_LIPID_METABOLISM_BY_PEROXISOME_PROLIFERATOR_ACTIVATED_RECEPTOR_ALPHA_PPARALP
17:60124582	*BRIP1*	EACTOME_REGULATION_OF_LIPID_METABOLISM_BY_PEROXISOME_PROLIFERATOR_ACTIVATED_RECEPTOR_ALPHA_PPARALP
4:114424939	*CAMK2D*	REACTOME_REGULATION_OF_MECP2_EXPRESSION_AND_ACTIVITY
4:114494052	*CAMK2D*	REACTOME_REGULATION_OF_MECP2_EXPRESSION_AND_ACTIVITY
4:114529492	*CAMK2D*	REACTOME_REGULATION_OF_MECP2_EXPRESSION_AND_ACTIVITY
4:114539964	*CAMK2D*	REACTOME_REGULATION_OF_MECP2_EXPRESSION_AND_ACTIVITY
4:114597918	*CAMK2D*	REACTOME_REGULATION_OF_MECP2_EXPRESSION_AND_ACTIVITY
10:103185542	*GBF1*	REACTOME_REGULATION_OF_MITOTIC_CELL_CYCLE
10:103305087	*GBF1*	REACTOME_REGULATION_OF_MITOTIC_CELL_CYCLE
10:103308244	*GBF1*	REACTOME_REGULATION_OF_MITOTIC_CELL_CYCLE
17:45215865	*WNT3*	REACTOME_REGULATION_OF_MITOTIC_CELL_CYCLE
17:45244822	*WNT3*	REACTOME_REGULATION_OF_MITOTIC_CELL_CYCLE

10:103185542	*GBF1*	REACTOME_REGULATION_OF_PLK1_ACTIVITY_AT_G2_M_TRANSITION
10:103305087	*GBF1*	REACTOME_REGULATION_OF_PLK1_ACTIVITY_AT_G2_M_TRANSITION
10:103308244	*GBF1*	REACTOME_REGULATION_OF_PLK1_ACTIVITY_AT_G2_M_TRANSITION
10:104258303	*GBF1*	REACTOME_REGULATION_OF_PLK1_ACTIVITY_AT_G2_M_TRANSITION
16:19562008	*SYT17*	REACTOME_REGULATION_OF_PLK1_ACTIVITY_AT_G2_M_TRANSITION
17:7578671	*V1NB1*	REACTOME_REGULATION_OF_PTEN_GENE_TRANSCRIPTION
6:112137847	*FYN*	REACTOME_REGULATION_OF_SIGNALING_BY_CBL
6:112151452	*FYN*	REACTOME_REGULATION_OF_SIGNALING_BY_CBL
6:112175069	*FYN*	REACTOME_REGULATION_OF_SIGNALING_BY_CBL
1:226074321	*ITPKB*	REACTOME_REGULATION_OF_SIGNALING_BY_NODAL
16:50790891	*CASC16*	REACTOME_REGULATION_OF_TNFR1_SIGNALING
19:2335872	*SPPL2B*	REACTOME_REGULATION_OF_TNFR1_SIGNALING
19:2346990	*SPPL2B*	REACTOME_REGULATION_OF_TNFR1_SIGNALING
19:2352757	*SPPL2B*	REACTOME_REGULATION_OF_TNFR1_SIGNALING
5:133558885	*C5orf24*	REACTOME_REGULATION_OF_TP53_ACTIVITY
6:31862876	*HLA-DRB5*	REACTOME_REGULATION_OF_TP53_ACTIVITY
6:33287183	*HLA-DRB5*	REACTOME_REGULATION_OF_TP53_ACTIVITY
17:7578671	*V1NB1*	REACTOME_REGULATION_OF_TP53_ACTIVITY
17:59791813	*BRIP1*	REACTOME_REGULATION_OF_TP53_ACTIVITY
17:59922925	*BRIP1*	REACTOME_REGULATION_OF_TP53_ACTIVITY
17:7578671	*V1NB1*	REACTOME_REGULATION_OF_TP53_ACTIVITY_THROUGH_ACETYLATION
17:7578671	*V1NB1*	REACTOME_REGULATION_OF_TP53_ACTIVITY_THROUGH_ASSOCIATION_WITH_CO_FACTORS
6:31862876	*HLA-DRB5*	REACTOME_REGULATION_OF_TP53_ACTIVITY_THROUGH_METHYLATION
17:7578671	*V1NB1*	REACTOME_REGULATION_OF_TP53_ACTIVITY_THROUGH_METHYLATION
17:7578671	*V1NB1*	REACTOME_REGULATION_OF_TP53_ACTIVITY_THROUGH_PHOSPHORYLATION
17:59791813	*BRIP1*	REACTOME_REGULATION_OF_TP53_ACTIVITY_THROUGH_PHOSPHORYLATION
17:59922925	*BRIP1*	REACTOME_REGULATION_OF_TP53_ACTIVITY_THROUGH_PHOSPHORYLATION
5:133558885	*C5orf24*	REACTOME_REGULATION_OF_TP53_EXPRESSION_AND_DEGRADATION
6:33287183	*HLA-DRB5*	REACTOME_REGULATION_OF_TP53_EXPRESSION_AND_DEGRADATION
17:7578671	*V1NB1*	REACTOME_REGULATION_OF_TP53_EXPRESSION_AND_DEGRADATION
19:3008151	*SPPL2B*	REACTOME_REPRESSION_OF_WNT_TARGET_GENES
19:3024077	*SPPL2B*	REACTOME_REPRESSION_OF_WNT_TARGET_GENES
1:156059276	*GBAP1*	REACTOME_REPRODUCTION
5:134307319	*C5orf24*	REACTOME_REPRODUCTION
5:134315853	*C5orf24*	REACTOME_REPRODUCTION
6:31716436	*HLA-DRB5*	REACTOME_REPRODUCTION
14:75495059	*RPS6KL1*	REACTOME_REPRODUCTION
1:226568665	*ITPKB*	REACTOME_RESOLUTION_OF_ABASIC_SITES_AP_SITES
12:133206648	*FBRSL1*	REACTOME_RESOLUTION_OF_ABASIC_SITES_AP_SITES
12:133223685	*FBRSL1*	REACTOME_RESOLUTION_OF_ABASIC_SITES_AP_SITES
1:226568665	*ITPKB*	REACTOME_RESOLUTION_OF_AP_SITES_VIA_THE_MULTIPLE_NUCLEOTIDE_PATCH_REPLACEMENT_PATHWAY
12:133206648	*FBRSL1*	REACTOME_RESOLUTION_OF_AP_SITES_VIA_THE_MULTIPLE_NUCLEOTIDE_PATCH_REPLACEMENT_PATHWAY
12:133223685	*FBRSL1*	REACTOME_RESOLUTION_OF_AP_SITES_VIA_THE_MULTIPLE_NUCLEOTIDE_PATCH_REPLACEMENT_PATHWAY
2:17941653	*KCNS3*	REACTOME_RESOLUTION_OF_D_LOOP_STRUCTURES
2:17963171	*KCNS3*	REACTOME_RESOLUTION_OF_D_LOOP_STRUCTURES
17:59791813	*BRIP1*	REACTOME_RESOLUTION_OF_D_LOOP_STRUCTURES
17:59922925	*BRIP1*	REACTOME_RESOLUTION_OF_D_LOOP_STRUCTURES
17:59791813	*BRIP1*	REACTOME_RESOLUTION_OF_D_LOOP_STRUCTURES_THROUGH_SYNTHESIS_DEPENDENT_STRAND_ANNEALING_SDSA
17:59922925	*BRIP1*	REACTOME_RESOLUTION_OF_D_LOOP_STRUCTURES_THROUGH_SYNTHESIS_DEPENDENT_STRAND_ANNEALING_SDSA
2:169747216	*STK39*	REACTOME_RESOLUTION_OF_SISTER_CHROMATID_COHESION
5:133558885	*C5orf24*	REACTOME_RESOLUTION_OF_SISTER_CHROMATID_COHESION
12:122816734	*HIP1R*	REACTOME_RESOLUTION_OF_SISTER_CHROMATID_COHESION
12:122817580	*HIP1R*	REACTOME_RESOLUTION_OF_SISTER_CHROMATID_COHESION

12:122853197	*HIP1R*	REACTOME_RESOLUTION_OF_SISTER_CHROMATID_COHESION
12:123057792	*HIP1R*	REACTOME_RESOLUTION_OF_SISTER_CHROMATID_COHESION
12:123110365	*HIP1R*	REACTOME_RESOLUTION_OF_SISTER_CHROMATID_COHESION
12:123110785	*HIP1R*	REACTOME_RESOLUTION_OF_SISTER_CHROMATID_COHESION
1:161171736	*FCGR2A*	ESPIRATORY_ELECTRON_TRANSPORT_ATP_SYNTHESIS_BY_CHEMIOSMOTIC_COUPLING_AND_HEAT_PRODUCTION_BY_UNCOUI
5:60273923	*ELOVL7*	ESPIRATORY_ELECTRON_TRANSPORT_ATP_SYNTHESIS_BY_CHEMIOSMOTIC_COUPLING_AND_HEAT_PRODUCTION_BY_UNCOUI
5:60288288	*ELOVL7*	ESPIRATORY_ELECTRON_TRANSPORT_ATP_SYNTHESIS_BY_CHEMIOSMOTIC_COUPLING_AND_HEAT_PRODUCTION_BY_UNCOUI
1:161171736	*FCGR2A*	REACTOME_RESPIRATORY_ELECTRON_TRANSPORT
5:60273923	*ELOVL7*	REACTOME_RESPIRATORY_ELECTRON_TRANSPORT
5:60288288	*ELOVL7*	REACTOME_RESPIRATORY_ELECTRON_TRANSPORT
4:90804532	*SNCA_2*	REACTOME_RESPONSE_TO_ELEVATED_PLATELET_CYTOSOLIC_CA2PLUS
4:90868355	*SNCA_2*	REACTOME_RESPONSE_TO_ELEVATED_PLATELET_CYTOSOLIC_CA2PLUS
6:31682029	*HLA-DRB5*	REACTOME_RESPONSE_TO_ELEVATED_PLATELET_CYTOSOLIC_CA2PLUS
8:130914130	*FAM49B*	REACTOME_RESPONSE_TO_ELEVATED_PLATELET_CYTOSOLIC_CA2PLUS
8:131002284	*FAM49B*	REACTOME_RESPONSE_TO_ELEVATED_PLATELET_CYTOSOLIC_CA2PLUS
17:42454243	*FAM171A2*	REACTOME_RESPONSE_TO_ELEVATED_PLATELET_CYTOSOLIC_CA2PLUS
17:76969183	*DNAH17*	REACTOME_RESPONSE_TO_ELEVATED_PLATELET_CYTOSOLIC_CA2PLUS
1:171837985	*VAMP4*	REACTOME_RETROGRADE_NEUROTROPHIN_SIGNALLING
1:171945488	*VAMP4*	REACTOME_RETROGRADE_NEUROTROPHIN_SIGNALLING
1:172137119	*VAMP4*	REACTOME_RETROGRADE_NEUROTROPHIN_SIGNALLING
1:172386261	*VAMP4*	REACTOME_RETROGRADE_NEUROTROPHIN_SIGNALLING
9:17579690	*SH3GL2_2*	REACTOME_RETROGRADE_NEUROTROPHIN_SIGNALLING
9:17587160	*SH3GL2_2*	REACTOME_RETROGRADE_NEUROTROPHIN_SIGNALLING
9:17609302	*SH3GL2_2*	REACTOME_RETROGRADE_NEUROTROPHIN_SIGNALLING
9:17626762	*SH3GL2_2*	REACTOME_RETROGRADE_NEUROTROPHIN_SIGNALLING
9:17627444	*SH3GL2_2*	REACTOME_RETROGRADE_NEUROTROPHIN_SIGNALLING
9:17642664	*SH3GL2_2*	REACTOME_RETROGRADE_NEUROTROPHIN_SIGNALLING
9:17710876	*SH3GL2_2*	REACTOME_RETROGRADE_NEUROTROPHIN_SIGNALLING
9:17732590	*SH3GL2_2*	REACTOME_RETROGRADE_NEUROTROPHIN_SIGNALLING
9:17737514	*SH3GL2_2*	REACTOME_RETROGRADE_NEUROTROPHIN_SIGNALLING
9:17758945	*SH3GL2_2*	REACTOME_RETROGRADE_NEUROTROPHIN_SIGNALLING
1:171689486	*VAMP4*	REACTOME_RETROGRADE_TRANSPORT_AT_THE_TRANS_GOLGI_NETWORK
17:44800946	*WNT3*	REACTOME_RETROGRADE_TRANSPORT_AT_THE_TRANS_GOLGI_NETWORK
17:44833217	*WNT3*	REACTOME_RETROGRADE_TRANSPORT_AT_THE_TRANS_GOLGI_NETWORK
8:21565088	*BIN3*	REACTOME_RET_SIGNALING
8:21582921	*BIN3*	REACTOME_RET_SIGNALING
8:21665306	*BIN3*	REACTOME_RET_SIGNALING
8:21668901	*BIN3*	REACTOME_RET_SIGNALING
1:155975202	*GBAP1*	REACTOME_RHO_GTPASE_CYCLE
1:161035780	*FCGR2A*	REACTOME_RHO_GTPASE_CYCLE
3:49406095	*IP6K2*	REACTOME_RHO_GTPASE_CYCLE
3:49424231	*IP6K2*	REACTOME_RHO_GTPASE_CYCLE
3:49450137	*IP6K2*	REACTOME_RHO_GTPASE_CYCLE
4:89711319	*SNCA_2*	REACTOME_RHO_GTPASE_CYCLE
4:89740128	*SNCA_2*	REACTOME_RHO_GTPASE_CYCLE
4:90002389	*SNCA_2*	REACTOME_RHO_GTPASE_CYCLE
5:59958372	*ELOVL7*	REACTOME_RHO_GTPASE_CYCLE
17:43472507	*CRHR1_2*	REACTOME_RHO_GTPASE_CYCLE
2:169747216	*STK39*	REACTOME_RHO_GTPASE_EFFECTORS
3:49406095	*IP6K2*	REACTOME_RHO_GTPASE_EFFECTORS
3:49424231	*IP6K2*	REACTOME_RHO_GTPASE_EFFECTORS
3:49450137	*IP6K2*	REACTOME_RHO_GTPASE_EFFECTORS
5:133558885	*C5orf24*	REACTOME_RHO_GTPASE_EFFECTORS

12:122816734	*HIP1R*	REACTOME_RHO_GTPASE_EFFECTORS
12:122817580	*HIP1R*	REACTOME_RHO_GTPASE_EFFECTORS
12:122853197	*HIP1R*	REACTOME_RHO_GTPASE_EFFECTORS
12:123057792	*HIP1R*	REACTOME_RHO_GTPASE_EFFECTORS
12:123110365	*HIP1R*	REACTOME_RHO_GTPASE_EFFECTORS
12:123110785	*HIP1R*	REACTOME_RHO_GTPASE_EFFECTORS
14:56110265	*GCH1*	REACTOME_RHO_GTPASE_EFFECTORS
17:7101771	*V1NB1*	REACTOME_RHO_GTPASE_EFFECTORS
17:7129974	*V1NB1*	REACTOME_RHO_GTPASE_EFFECTORS
17:43302259	*CRHR1_2*	REACTOME_RHO_GTPASE_EFFECTORS
3:49406095	*IP6K2*	REACTOME_RHO_GTPASES_ACTIVATE_CIT
3:49424231	*IP6K2*	REACTOME_RHO_GTPASES_ACTIVATE_CIT
3:49450137	*IP6K2*	REACTOME_RHO_GTPASES_ACTIVATE_CIT
17:7101771	*V1NB1*	REACTOME_RHO_GTPASES_ACTIVATE_CIT
2:169747216	*STK39*	REACTOME_RHO_GTPASES_ACTIVATE_FORMINS
3:49406095	*IP6K2*	REACTOME_RHO_GTPASES_ACTIVATE_FORMINS
3:49424231	*IP6K2*	REACTOME_RHO_GTPASES_ACTIVATE_FORMINS
3:49450137	*IP6K2*	REACTOME_RHO_GTPASES_ACTIVATE_FORMINS
5:133558885	*C5orf24*	REACTOME_RHO_GTPASES_ACTIVATE_FORMINS
12:122816734	*HIP1R*	REACTOME_RHO_GTPASES_ACTIVATE_FORMINS
12:122817580	*HIP1R*	REACTOME_RHO_GTPASES_ACTIVATE_FORMINS
12:122853197	*HIP1R*	REACTOME_RHO_GTPASES_ACTIVATE_FORMINS
12:123057792	*HIP1R*	REACTOME_RHO_GTPASES_ACTIVATE_FORMINS
12:123110365	*HIP1R*	REACTOME_RHO_GTPASES_ACTIVATE_FORMINS
12:123110785	*HIP1R*	REACTOME_RHO_GTPASES_ACTIVATE_FORMINS
17:7129974	*V1NB1*	REACTOME_RHO_GTPASES_ACTIVATE_FORMINS
17:43302259	*CRHR1_2*	REACTOME_RHO_GTPASES_ACTIVATE_FORMINS
12:122816734	*HIP1R*	REACTOME_RHO_GTPASES_ACTIVATE_IQGAPS
12:122817580	*HIP1R*	REACTOME_RHO_GTPASES_ACTIVATE_IQGAPS
12:122853197	*HIP1R*	REACTOME_RHO_GTPASES_ACTIVATE_IQGAPS
3:49406095	*IP6K2*	REACTOME_RHO_GTPASES_ACTIVATE_KTN1
3:49424231	*IP6K2*	REACTOME_RHO_GTPASES_ACTIVATE_KTN1
3:49450137	*IP6K2*	REACTOME_RHO_GTPASES_ACTIVATE_KTN1
14:56110265	*GCH1*	REACTOME_RHO_GTPASES_ACTIVATE_KTN1
3:49406095	*IP6K2*	REACTOME_RHO_GTPASES_ACTIVATE_PKNS
3:49424231	*IP6K2*	REACTOME_RHO_GTPASES_ACTIVATE_PKNS
3:49450137	*IP6K2*	REACTOME_RHO_GTPASES_ACTIVATE_PKNS
3:49406095	*IP6K2*	REACTOME_RHO_GTPASES_ACTIVATE_RHOTEKIN_AND_RHOPHILINS
3:49424231	*IP6K2*	REACTOME_RHO_GTPASES_ACTIVATE_RHOTEKIN_AND_RHOPHILINS
3:49450137	*IP6K2*	REACTOME_RHO_GTPASES_ACTIVATE_RHOTEKIN_AND_RHOPHILINS
3:49406095	*IP6K2*	REACTOME_RHO_GTPASES_ACTIVATE_ROCKS
3:49424231	*IP6K2*	REACTOME_RHO_GTPASES_ACTIVATE_ROCKS
3:49450137	*IP6K2*	REACTOME_RHO_GTPASES_ACTIVATE_ROCKS
12:46134812	*SCAF11*	REACTOME_RMTS_METHYLATE_HISTONE_ARGININES
12:46206268	*SCAF11*	REACTOME_RMTS_METHYLATE_HISTONE_ARGININES
8:22108784	*BIN3*	REACTOME_RNA_POLYMERASE_III_CHAIN_ELONGATION
8:22108784	*BIN3*	REACTOME_RNA_POLYMERASE_III_TRANSCRIPTION_INITIATION_FROM_TYPE_1_PROMOTER
8:22108784	*BIN3*	REACTOME_RNA_POLYMERASE_III_TRANSCRIPTION_INITIATION_FROM_TYPE_3_PROMOTER
8:22108784	*BIN3*	REACTOME_RNA_POLYMERASE_III_TRANSCRIPTION
8:22108784	*BIN3*	REACTOME_RNA_POLYMERASE_III_TRANSCRIPTION_TERMINATION
2:135708115	*TMEM163*	REACTOME_RNA_POLYMERASE_II_PRE_TRANSCRIPTION_EVENTS
6:31925679	*HLA-DRB5*	REACTOME_RNA_POLYMERASE_II_PRE_TRANSCRIPTION_EVENTS
2:135708115	*TMEM163*	REACTOME_RNA_POLYMERASE_II_TRANSCRIBES_SNRNA_GENES

17:59967940	*BRIP1*	REACTOME_RNA_POLYMERASE_II_TRANSCRIBES_SNRNA_GENES
2:135708115	*TMEM163*	REACTOME_RNA_POLYMERASE_II_TRANSCRIPTION_ELONGATION
6:31925679	*HLA-DRB5*	REACTOME_RNA_POLYMERASE_II_TRANSCRIPTION_ELONGATION
6:31862876	*HLA-DRB5*	REACTOME_RNA_POLYMERASE_I_TRANSCRIPTION_INITIATION
6:31862876	*HLA-DRB5*	REACTOME_RNA_POLYMERASE_I_TRANSCRIPTION
3:48835245	*IP6K2*	REACTOME_ROBO_RECEPTORS_BIND_AKAP5
6:112137847	*FYN*	REACTOME_ROLE_OF_LAT2_NTAL_LAB_ON_CALCIUM_MOBILIZATION
6:112151452	*FYN*	REACTOME_ROLE_OF_LAT2_NTAL_LAB_ON_CALCIUM_MOBILIZATION
6:112175069	*FYN*	REACTOME_ROLE_OF_LAT2_NTAL_LAB_ON_CALCIUM_MOBILIZATION
15:61016235	*VPS13C*	REACTOME_RORA_ACTIVATES_GENE_EXPRESSION
15:61065568	*VPS13C*	REACTOME_RORA_ACTIVATES_GENE_EXPRESSION
15:61094298	*VPS13C*	REACTOME_RORA_ACTIVATES_GENE_EXPRESSION
15:61140736	*VPS13C*	REACTOME_RORA_ACTIVATES_GENE_EXPRESSION
15:61142062	*VPS13C*	REACTOME_RORA_ACTIVATES_GENE_EXPRESSION
15:61162571	*VPS13C*	REACTOME_RORA_ACTIVATES_GENE_EXPRESSION
15:61191617	*VPS13C*	REACTOME_RORA_ACTIVATES_GENE_EXPRESSION
15:61195427	*VPS13C*	REACTOME_RORA_ACTIVATES_GENE_EXPRESSION
15:61221682	*VPS13C*	REACTOME_RORA_ACTIVATES_GENE_EXPRESSION
15:61298486	*VPS13C*	REACTOME_RORA_ACTIVATES_GENE_EXPRESSION
15:61375480	*VPS13C*	REACTOME_RORA_ACTIVATES_GENE_EXPRESSION
15:61434581	*VPS13C*	REACTOME_RORA_ACTIVATES_GENE_EXPRESSION
15:61467078	*VPS13C*	REACTOME_RORA_ACTIVATES_GENE_EXPRESSION
15:61470808	*VPS13C*	REACTOME_RORA_ACTIVATES_GENE_EXPRESSION
15:61500263	*VPS13C*	REACTOME_RORA_ACTIVATES_GENE_EXPRESSION
12:124219791	*HIP1R*	REACTOME_ROS_AND_RNS_PRODUCTION_IN_PHAGOCYTES
9:33467577	*UBAP2*	REACTOME_RRNA_MODIFICATION_IN_THE_NUCLEUS_AND_CYTOSOL
9:33467577	*UBAP2*	REACTOME_RRNA_PROCESSING_IN_THE_NUCLEUS_AND_CYTOSOL
9:33467577	*UBAP2*	REACTOME_RRNA_PROCESSING
12:46134812	*SCAF11*	REACTOME_RUNX1_INTERACTS_WITH_CO_FACTORS_WHOSE_PRECISE_EFFECT_ON_RUNX1_TARGETS_IS_NOT_KNOWN
12:46206268	*SCAF11*	REACTOME_RUNX1_INTERACTS_WITH_CO_FACTORS_WHOSE_PRECISE_EFFECT_ON_RUNX1_TARGETS_IS_NOT_KNOWN
17:42454243	*FAM171A2*	REACTOME_RUNX1_REGULATES_GENES_INVOLVED_IN_MEGAKARYOCYTE_DIFFERENTIATION_AND_PLATELET_FUNCTION
8:11353586	*CTSB*	REACTOME_RUNX1_REGULATES_TRANSCRIPTION_OF_GENES_INVOLVED_IN_BCR_SIGNALING
8:11363667	*CTSB*	REACTOME_RUNX1_REGULATES_TRANSCRIPTION_OF_GENES_INVOLVED_IN_BCR_SIGNALING
8:11381148	*CTSB*	REACTOME_RUNX1_REGULATES_TRANSCRIPTION_OF_GENES_INVOLVED_IN_BCR_SIGNALING
19:1611856	*SPPL2B*	REACTOME_RUNX1_REGULATES_TRANSCRIPTION_OF_GENES_INVOLVED_IN_DIFFERENTIATION_OF_HSCS
14:55515304	*GCH1*	REACTOME_RUNX1_REGULATES_TRANSCRIPTION_OF_GENES_INVOLVED_IN_DIFFERENTIATION_OF_KERATINOCYTES
17:7578671	*V1NB1*	REACTOME_RUNX3_REGULATES_CDKN1A_TRANSCRIPTION
16:30510571	*SETD1A*	REACTOME_RUNX3_REGULATES_IMMUNE_RESPONSE_AND_CELL_MIGRATION
16:30516492	*SETD1A*	REACTOME_RUNX3_REGULATES_IMMUNE_RESPONSE_AND_CELL_MIGRATION
16:30520856	*SETD1A*	REACTOME_RUNX3_REGULATES_IMMUNE_RESPONSE_AND_CELL_MIGRATION
6:32941506	*HLA-DRB5*	REACTOME_RUNX3_REGULATES_P14_ARF
8:16086683	*FGF20*	REACTOME_SCAVENGING_BY_CLASS_A_RECEPTORS
8:16135246	*FGF20*	REACTOME_SCAVENGING_BY_CLASS_A_RECEPTORS
6:112137847	*FYN*	REACTOME_SEMA3A_PAK_DEPENDENT_AXON_REPULSION
6:112151452	*FYN*	REACTOME_SEMA3A_PAK_DEPENDENT_AXON_REPULSION
6:112175069	*FYN*	REACTOME_SEMA3A_PAK_DEPENDENT_AXON_REPULSION
6:112137847	*FYN*	REACTOME_SEMA3A_PLEXIN_REPULSION_SIGNALING_BY_INHIBITING_INTEGRIN_ADHESION
6:112151452	*FYN*	REACTOME_SEMA3A_PLEXIN_REPULSION_SIGNALING_BY_INHIBITING_INTEGRIN_ADHESION
6:112175069	*FYN*	REACTOME_SEMA3A_PLEXIN_REPULSION_SIGNALING_BY_INHIBITING_INTEGRIN_ADHESION
3:49406095	*IP6K2*	REACTOME_SEMA4D_INDUCED_CELL_MIGRATION_AND_GROWTH_CONE_COLLAPSE
3:49424231	*IP6K2*	REACTOME_SEMA4D_INDUCED_CELL_MIGRATION_AND_GROWTH_CONE_COLLAPSE
3:49450137	*IP6K2*	REACTOME_SEMA4D_INDUCED_CELL_MIGRATION_AND_GROWTH_CONE_COLLAPSE
3:49406095	*IP6K2*	REACTOME_SEMA4D_IN_SEMAPHORIN_SIGNALING

3:49424231	*IP6K2*	REACTOME_SEMA4D_IN_SEMAPHORIN_SIGNALING
3:49450137	*IP6K2*	REACTOME_SEMA4D_IN_SEMAPHORIN_SIGNALING
3:49406095	*IP6K2*	REACTOME_SEMA4D_MEDIATED_INHIBITION_OF_CELL_ATTACHMENT_AND_MIGRATION
3:49424231	*IP6K2*	REACTOME_SEMA4D_MEDIATED_INHIBITION_OF_CELL_ATTACHMENT_AND_MIGRATION
3:49450137	*IP6K2*	REACTOME_SEMA4D_MEDIATED_INHIBITION_OF_CELL_ATTACHMENT_AND_MIGRATION
3:49406095	*IP6K2*	REACTOME_SEMAPHORIN_INTERACTIONS
3:49424231	*IP6K2*	REACTOME_SEMAPHORIN_INTERACTIONS
3:49450137	*IP6K2*	REACTOME_SEMAPHORIN_INTERACTIONS
6:112137847	*FYN*	REACTOME_SEMAPHORIN_INTERACTIONS
6:112151452	*FYN*	REACTOME_SEMAPHORIN_INTERACTIONS
6:112175069	*FYN*	REACTOME_SEMAPHORIN_INTERACTIONS
6:31862876	*HLA-DRB5*	REACTOME_SENESCENCE_ASSOCIATED_SECRETORY_PHENOTYPE_SASP
17:45215865	*WNT3*	REACTOME_SENESCENCE_ASSOCIATED_SECRETORY_PHENOTYPE_SASP
17:45244822	*WNT3*	REACTOME_SENESCENCE_ASSOCIATED_SECRETORY_PHENOTYPE_SASP
6:72646790	*RIMS1*	REACTOME_SEROTONIN_NEUROTRANSMITTER_RELEASE_CYCLE
6:72648340	*RIMS1*	REACTOME_SEROTONIN_NEUROTRANSMITTER_RELEASE_CYCLE
6:72686444	*RIMS1*	REACTOME_SEROTONIN_NEUROTRANSMITTER_RELEASE_CYCLE
6:72716358	*RIMS1*	REACTOME_SEROTONIN_NEUROTRANSMITTER_RELEASE_CYCLE
6:72904599	*RIMS1*	REACTOME_SEROTONIN_NEUROTRANSMITTER_RELEASE_CYCLE
6:72913435	*RIMS1*	REACTOME_SEROTONIN_NEUROTRANSMITTER_RELEASE_CYCLE
6:72930929	*RIMS1*	REACTOME_SEROTONIN_NEUROTRANSMITTER_RELEASE_CYCLE
6:72982289	*RIMS1*	REACTOME_SEROTONIN_NEUROTRANSMITTER_RELEASE_CYCLE
6:72986711	*RIMS1*	REACTOME_SEROTONIN_NEUROTRANSMITTER_RELEASE_CYCLE
6:73008061	*RIMS1*	REACTOME_SEROTONIN_NEUROTRANSMITTER_RELEASE_CYCLE
6:73060492	*RIMS1*	REACTOME_SEROTONIN_NEUROTRANSMITTER_RELEASE_CYCLE
3:151090424	*MED12L*	REACTOME_SIGNAL_AMPLIFICATION
3:151099733	*MED12L*	REACTOME_SIGNAL_AMPLIFICATION
19:2557113	*SPPL2B*	REACTOME_SIGNAL_AMPLIFICATION
19:2563327	*SPPL2B*	REACTOME_SIGNAL_AMPLIFICATION
19:2580057	*SPPL2B*	REACTOME_SIGNAL_AMPLIFICATION
19:2660983	*SPPL2B*	REACTOME_SIGNAL_AMPLIFICATION
19:3142784	*SPPL2B*	REACTOME_SIGNAL_AMPLIFICATION
19:3147326	*SPPL2B*	REACTOME_SIGNAL_AMPLIFICATION
19:2251817	*SPPL2B*	REACTOME_SIGNALING_BY_BMP
1:156059276	*GBAP1*	REACTOME_SIGNALING_BY_BRAF_AND_RAF_FUSIONS
4:114424939	*CAMK2D*	REACTOME_SIGNALING_BY_BRAF_AND_RAF_FUSIONS
4:114494052	*CAMK2D*	REACTOME_SIGNALING_BY_BRAF_AND_RAF_FUSIONS
4:114529492	*CAMK2D*	REACTOME_SIGNALING_BY_BRAF_AND_RAF_FUSIONS
4:114539964	*CAMK2D*	REACTOME_SIGNALING_BY_BRAF_AND_RAF_FUSIONS
4:114597918	*CAMK2D*	REACTOME_SIGNALING_BY_BRAF_AND_RAF_FUSIONS
17:42454243	*FAM171A2*	REACTOME_SIGNALING_BY_BRAF_AND_RAF_FUSIONS
17:40392358	*RETREG3*	REACTOME_SIGNALING_BY_CYTOSOLIC_FGFR1_FUSION_MUTANTS
9:17579690	*SH3GL2_2*	REACTOME_SIGNALING_BY_EGFR
9:17587160	*SH3GL2_2*	REACTOME_SIGNALING_BY_EGFR
9:17609302	*SH3GL2_2*	REACTOME_SIGNALING_BY_EGFR
9:17626762	*SH3GL2_2*	REACTOME_SIGNALING_BY_EGFR
9:17627444	*SH3GL2_2*	REACTOME_SIGNALING_BY_EGFR
9:17642664	*SH3GL2_2*	REACTOME_SIGNALING_BY_EGFR
9:17710876	*SH3GL2_2*	REACTOME_SIGNALING_BY_EGFR
9:17732590	*SH3GL2_2*	REACTOME_SIGNALING_BY_EGFR
9:17737514	*SH3GL2_2*	REACTOME_SIGNALING_BY_EGFR
9:17758945	*SH3GL2_2*	REACTOME_SIGNALING_BY_EGFR
3:49406095	*IP6K2*	REACTOME_SIGNALING_BY_ERBB2

3:49424231	*IP6K2*	REACTOME_SIGNALING_BY_ERBB2
3:49450137	*IP6K2*	REACTOME_SIGNALING_BY_ERBB2
6:112137847	*FYN*	REACTOME_SIGNALING_BY_ERBB2
6:112151452	*FYN*	REACTOME_SIGNALING_BY_ERBB2
6:112175069	*FYN*	REACTOME_SIGNALING_BY_ERBB2
1:227067656	*ITPKB*	REACTOME_SIGNALING_BY_ERBB4
17:7101771	*V1NB1*	REACTOME_SIGNALING_BY_ERBB4
17:42990894	*CRHR1_2*	REACTOME_SIGNALING_BY_ERBB4
17:40392358	*RETREG3*	REACTOME_SIGNALING_BY_ERYTHROPOIETIN
17:40392358	*RETREG3*	REACTOME_SIGNALING_BY_FGFR1_IN_DISEASE
5:133558885	*C5orf24*	REACTOME_SIGNALING_BY_FGFR1
5:133558885	*C5orf24*	REACTOME_SIGNALING_BY_FGFR2
5:133558885	*C5orf24*	REACTOME_SIGNALING_BY_FGFR3
5:133558885	*C5orf24*	REACTOME_SIGNALING_BY_FGFR4
17:40392358	*RETREG3*	REACTOME_SIGNALING_BY_FGFR_IN_DISEASE
5:133558885	*C5orf24*	REACTOME_SIGNALING_BY_FGFR
1:155975202	*GBAP1*	REACTOME_SIGNALING_BY_GPCR
2:170012307	*STK39*	REACTOME_SIGNALING_BY_GPCR
2:170028024	*STK39*	REACTOME_SIGNALING_BY_GPCR
2:170066424	*STK39*	REACTOME_SIGNALING_BY_GPCR
3:48835245	*IP6K2*	REACTOME_SIGNALING_BY_GPCR
3:49406095	*IP6K2*	REACTOME_SIGNALING_BY_GPCR
3:49424231	*IP6K2*	REACTOME_SIGNALING_BY_GPCR
3:49450137	*IP6K2*	REACTOME_SIGNALING_BY_GPCR
3:123089448	*KPNA1*	REACTOME_SIGNALING_BY_GPCR
3:150935203	*MED12L*	REACTOME_SIGNALING_BY_GPCR
3:151090424	*MED12L*	REACTOME_SIGNALING_BY_GPCR
3:151099733	*MED12L*	REACTOME_SIGNALING_BY_GPCR
4:76924571	*FAM47E-STBD1*	REACTOME_SIGNALING_BY_GPCR
4:114424939	*CAMK2D*	REACTOME_SIGNALING_BY_GPCR
4:114494052	*CAMK2D*	REACTOME_SIGNALING_BY_GPCR
4:114529492	*CAMK2D*	REACTOME_SIGNALING_BY_GPCR
4:114539964	*CAMK2D*	REACTOME_SIGNALING_BY_GPCR
4:114597918	*CAMK2D*	REACTOME_SIGNALING_BY_GPCR
5:59236972	*ELOVL7*	REACTOME_SIGNALING_BY_GPCR
5:59490965	*ELOVL7*	REACTOME_SIGNALING_BY_GPCR
5:59554719	*ELOVL7*	REACTOME_SIGNALING_BY_GPCR
5:59705316	*ELOVL7*	REACTOME_SIGNALING_BY_GPCR
5:59736085	*ELOVL7*	REACTOME_SIGNALING_BY_GPCR
5:59812853	*ELOVL7*	REACTOME_SIGNALING_BY_GPCR
5:133558885	*C5orf24*	REACTOME_SIGNALING_BY_GPCR
6:29407378	*TRIM40*	REACTOME_SIGNALING_BY_GPCR
6:29412222	*TRIM40*	REACTOME_SIGNALING_BY_GPCR
6:29565889	*TRIM40*	REACTOME_SIGNALING_BY_GPCR
6:29590715	*TRIM40*	REACTOME_SIGNALING_BY_GPCR
8:131799767	*FAM49B*	REACTOME_SIGNALING_BY_GPCR
8:131845512	*FAM49B*	REACTOME_SIGNALING_BY_GPCR
8:131865425	*FAM49B*	REACTOME_SIGNALING_BY_GPCR
8:131901170	*FAM49B*	REACTOME_SIGNALING_BY_GPCR
10:15199347	*ITGA8*	REACTOME_SIGNALING_BY_GPCR
10:121054578	*INPP5F*	REACTOME_SIGNALING_BY_GPCR
10:121077191	*INPP5F*	REACTOME_SIGNALING_BY_GPCR
10:121085648	*INPP5F*	REACTOME_SIGNALING_BY_GPCR

10:121104943	*INPP5F*	REACTOME_SIGNALING_BY_GPCR
10:121114292	*INPP5F*	REACTOME_SIGNALING_BY_GPCR
10:121191186	*INPP5F*	REACTOME_SIGNALING_BY_GPCR
10:121201427	*INPP5F*	REACTOME_SIGNALING_BY_GPCR
10:121295884	*INPP5F*	REACTOME_SIGNALING_BY_GPCR
14:88472612	*GALC*	REACTOME_SIGNALING_BY_GPCR
16:50300511	*CASC16*	REACTOME_SIGNALING_BY_GPCR
16:50317039	*CASC16*	REACTOME_SIGNALING_BY_GPCR
17:43181287	*CRHR1_2*	REACTOME_SIGNALING_BY_GPCR
17:43184627	*CRHR1_2*	REACTOME_SIGNALING_BY_GPCR
17:43744203	*CRHR1_2*	REACTOME_SIGNALING_BY_GPCR
17:43748712	*CRHR1_2*	REACTOME_SIGNALING_BY_GPCR
17:43784228	*CRHR1_2*	REACTOME_SIGNALING_BY_GPCR
17:43791502	*CRHR1_2*	REACTOME_SIGNALING_BY_GPCR
17:43796210	*CRHR1_2*	REACTOME_SIGNALING_BY_GPCR
17:43843861	*CRHR1_2*	REACTOME_SIGNALING_BY_GPCR
17:43847503	*CRHR1_2*	REACTOME_SIGNALING_BY_GPCR
17:43849514	*CRHR1_2*	REACTOME_SIGNALING_BY_GPCR
17:44843651	*WNT3*	REACTOME_SIGNALING_BY_GPCR
17:44870978	*WNT3*	REACTOME_SIGNALING_BY_GPCR
17:44871422	*WNT3*	REACTOME_SIGNALING_BY_GPCR
17:44888209	*WNT3*	REACTOME_SIGNALING_BY_GPCR
17:44888715	*WNT3*	REACTOME_SIGNALING_BY_GPCR
17:44906949	*WNT3*	REACTOME_SIGNALING_BY_GPCR
17:44912901	*WNT3*	REACTOME_SIGNALING_BY_GPCR
17:44931399	*WNT3*	REACTOME_SIGNALING_BY_GPCR
17:44947756	*WNT3*	REACTOME_SIGNALING_BY_GPCR
19:2557113	*SPPL2B*	REACTOME_SIGNALING_BY_GPCR
19:2563327	*SPPL2B*	REACTOME_SIGNALING_BY_GPCR
19:2580057	*SPPL2B*	REACTOME_SIGNALING_BY_GPCR
19:2660983	*SPPL2B*	REACTOME_SIGNALING_BY_GPCR
19:3142784	*SPPL2B*	REACTOME_SIGNALING_BY_GPCR
19:3147326	*SPPL2B*	REACTOME_SIGNALING_BY_GPCR
20:5290081	*CRLS1*	REACTOME_SIGNALING_BY_GPCR
3:48835245	*IP6K2*	REACTOME_SIGNALING_BY_HEDGEHOG
3:123089448	*KPNA1*	REACTOME_SIGNALING_BY_HEDGEHOG
8:131799767	*FAM49B*	REACTOME_SIGNALING_BY_HEDGEHOG
8:131845512	*FAM49B*	REACTOME_SIGNALING_BY_HEDGEHOG
8:131865425	*FAM49B*	REACTOME_SIGNALING_BY_HEDGEHOG
8:131901170	*FAM49B*	REACTOME_SIGNALING_BY_HEDGEHOG
10:103185542	*GBF1*	REACTOME_SIGNALING_BY_HEDGEHOG
10:103305087	*GBF1*	REACTOME_SIGNALING_BY_HEDGEHOG
10:103308244	*GBF1*	REACTOME_SIGNALING_BY_HEDGEHOG
10:104278055	*GBF1*	REACTOME_SIGNALING_BY_HEDGEHOG
10:104305501	*GBF1*	REACTOME_SIGNALING_BY_HEDGEHOG
16:50300511	*CASC16*	REACTOME_SIGNALING_BY_HEDGEHOG
16:50317039	*CASC16*	REACTOME_SIGNALING_BY_HEDGEHOG
17:7129974	*V1NB1*	REACTOME_SIGNALING_BY_HIPPO
12:124219791	*HIP1R*	REACTOME_SIGNALING_BY_INSULIN_RECEPTOR
2:102612026	*MAP4K4*	REACTOME_SIGNALING_BY_INTERLEUKINS
2:102621855	*MAP4K4*	REACTOME_SIGNALING_BY_INTERLEUKINS
2:102631503	*MAP4K4*	REACTOME_SIGNALING_BY_INTERLEUKINS
2:102633387	*MAP4K4*	REACTOME_SIGNALING_BY_INTERLEUKINS

2:102720323	*MAP4K4*	REACTOME_SIGNALING_BY_INTERLEUKINS
2:102783052	*MAP4K4*	REACTOME_SIGNALING_BY_INTERLEUKINS
2:102826146	*MAP4K4*	REACTOME_SIGNALING_BY_INTERLEUKINS
2:103047986	*MAP4K4*	REACTOME_SIGNALING_BY_INTERLEUKINS
2:103063855	*MAP4K4*	REACTOME_SIGNALING_BY_INTERLEUKINS
2:103066346	*MAP4K4*	REACTOME_SIGNALING_BY_INTERLEUKINS
4:114424939	*CAMK2D*	REACTOME_SIGNALING_BY_INTERLEUKINS
4:114494052	*CAMK2D*	REACTOME_SIGNALING_BY_INTERLEUKINS
4:114529492	*CAMK2D*	REACTOME_SIGNALING_BY_INTERLEUKINS
4:114539964	*CAMK2D*	REACTOME_SIGNALING_BY_INTERLEUKINS
4:114597918	*CAMK2D*	REACTOME_SIGNALING_BY_INTERLEUKINS
5:133558885	*C5orf24*	REACTOME_SIGNALING_BY_INTERLEUKINS
6:32151443	*HLA-DRB5*	REACTOME_SIGNALING_BY_INTERLEUKINS
6:112137847	*FYN*	REACTOME_SIGNALING_BY_INTERLEUKINS
6:112151452	*FYN*	REACTOME_SIGNALING_BY_INTERLEUKINS
6:112175069	*FYN*	REACTOME_SIGNALING_BY_INTERLEUKINS
8:21565088	*BIN3*	REACTOME_SIGNALING_BY_INTERLEUKINS
8:21582921	*BIN3*	REACTOME_SIGNALING_BY_INTERLEUKINS
8:21665306	*BIN3*	REACTOME_SIGNALING_BY_INTERLEUKINS
8:21668901	*BIN3*	REACTOME_SIGNALING_BY_INTERLEUKINS
10:103185542	*GBF1*	REACTOME_SIGNALING_BY_INTERLEUKINS
10:103305087	*GBF1*	REACTOME_SIGNALING_BY_INTERLEUKINS
10:103308244	*GBF1*	REACTOME_SIGNALING_BY_INTERLEUKINS
11:83219006	*DLG2*	REACTOME_SIGNALING_BY_INTERLEUKINS
11:83232915	*DLG2*	REACTOME_SIGNALING_BY_INTERLEUKINS
11:83282740	*DLG2*	REACTOME_SIGNALING_BY_INTERLEUKINS
11:83387273	*DLG2*	REACTOME_SIGNALING_BY_INTERLEUKINS
11:83538733	*DLG2*	REACTOME_SIGNALING_BY_INTERLEUKINS
11:83544472	*DLG2*	REACTOME_SIGNALING_BY_INTERLEUKINS
11:83578152	*DLG2*	REACTOME_SIGNALING_BY_INTERLEUKINS
11:83647657	*DLG2*	REACTOME_SIGNALING_BY_INTERLEUKINS
11:83669903	*DLG2*	REACTOME_SIGNALING_BY_INTERLEUKINS
11:83736246	*DLG2*	REACTOME_SIGNALING_BY_INTERLEUKINS
11:83762918	*DLG2*	REACTOME_SIGNALING_BY_INTERLEUKINS
11:83823752	*DLG2*	REACTOME_SIGNALING_BY_INTERLEUKINS
11:83839992	*DLG2*	REACTOME_SIGNALING_BY_INTERLEUKINS
11:84104763	*DLG2*	REACTOME_SIGNALING_BY_INTERLEUKINS
11:84169039	*DLG2*	REACTOME_SIGNALING_BY_INTERLEUKINS
11:84221628	*DLG2*	REACTOME_SIGNALING_BY_INTERLEUKINS
11:84312008	*DLG2*	REACTOME_SIGNALING_BY_INTERLEUKINS
11:84324361	*DLG2*	REACTOME_SIGNALING_BY_INTERLEUKINS
11:84360346	*DLG2*	REACTOME_SIGNALING_BY_INTERLEUKINS
11:84403409	*DLG2*	REACTOME_SIGNALING_BY_INTERLEUKINS
15:61016235	*VPS13C*	REACTOME_SIGNALING_BY_INTERLEUKINS
15:61065568	*VPS13C*	REACTOME_SIGNALING_BY_INTERLEUKINS
15:61094298	*VPS13C*	REACTOME_SIGNALING_BY_INTERLEUKINS
15:61140736	*VPS13C*	REACTOME_SIGNALING_BY_INTERLEUKINS
15:61142062	*VPS13C*	REACTOME_SIGNALING_BY_INTERLEUKINS
15:61162571	*VPS13C*	REACTOME_SIGNALING_BY_INTERLEUKINS
15:61191617	*VPS13C*	REACTOME_SIGNALING_BY_INTERLEUKINS
15:61195427	*VPS13C*	REACTOME_SIGNALING_BY_INTERLEUKINS
15:61221682	*VPS13C*	REACTOME_SIGNALING_BY_INTERLEUKINS
15:61298486	*VPS13C*	REACTOME_SIGNALING_BY_INTERLEUKINS

15:61375480	*VPS13C*	REACTOME_SIGNALING_BY_INTERLEUKINS
15:61434581	*VPS13C*	REACTOME_SIGNALING_BY_INTERLEUKINS
15:61467078	*VPS13C*	REACTOME_SIGNALING_BY_INTERLEUKINS
15:61470808	*VPS13C*	REACTOME_SIGNALING_BY_INTERLEUKINS
15:61500263	*VPS13C*	REACTOME_SIGNALING_BY_INTERLEUKINS
16:31370452	*SETD1A*	REACTOME_SIGNALING_BY_INTERLEUKINS
16:50730088	*CASC16*	REACTOME_SIGNALING_BY_INTERLEUKINS
16:50730446	*CASC16*	REACTOME_SIGNALING_BY_INTERLEUKINS
16:50745926	*CASC16*	REACTOME_SIGNALING_BY_INTERLEUKINS
17:7101771	*V1NB1*	REACTOME_SIGNALING_BY_INTERLEUKINS
17:7578671	*V1NB1*	REACTOME_SIGNALING_BY_INTERLEUKINS
17:40392358	*RETREG3*	REACTOME_SIGNALING_BY_INTERLEUKINS
17:41845031	*FAM171A2*	REACTOME_SIGNALING_BY_INTERLEUKINS
17:42454243	*FAM171A2*	REACTOME_SIGNALING_BY_INTERLEUKINS
17:40392358	*RETREG3*	REACTOME_SIGNALING_BY_LEPTIN
3:49162284	*IP6K2*	REACTOME_SIGNALING_BY_MET
3:49162583	*IP6K2*	REACTOME_SIGNALING_BY_MET
6:33153528	*HLA-DRB5*	REACTOME_SIGNALING_BY_MET
6:112494762	*FYN*	REACTOME_SIGNALING_BY_MET
6:112531401	*FYN*	REACTOME_SIGNALING_BY_MET
6:112561154	*FYN*	REACTOME_SIGNALING_BY_MET
6:112561430	*FYN*	REACTOME_SIGNALING_BY_MET
9:17579690	*SH3GL2_2*	REACTOME_SIGNALING_BY_MET
9:17587160	*SH3GL2_2*	REACTOME_SIGNALING_BY_MET
9:17609302	*SH3GL2_2*	REACTOME_SIGNALING_BY_MET
9:17626762	*SH3GL2_2*	REACTOME_SIGNALING_BY_MET
9:17627444	*SH3GL2_2*	REACTOME_SIGNALING_BY_MET
9:17642664	*SH3GL2_2*	REACTOME_SIGNALING_BY_MET
9:17710876	*SH3GL2_2*	REACTOME_SIGNALING_BY_MET
9:17732590	*SH3GL2_2*	REACTOME_SIGNALING_BY_MET
9:17737514	*SH3GL2_2*	REACTOME_SIGNALING_BY_MET
9:17758945	*SH3GL2_2*	REACTOME_SIGNALING_BY_MET
4:114424939	*CAMK2D*	REACTOME_SIGNALING_BY_MODERATE_KINASE_ACTIVITY_BRAF_MUTANTS
4:114494052	*CAMK2D*	REACTOME_SIGNALING_BY_MODERATE_KINASE_ACTIVITY_BRAF_MUTANTS
4:114529492	*CAMK2D*	REACTOME_SIGNALING_BY_MODERATE_KINASE_ACTIVITY_BRAF_MUTANTS
4:114539964	*CAMK2D*	REACTOME_SIGNALING_BY_MODERATE_KINASE_ACTIVITY_BRAF_MUTANTS
4:114597918	*CAMK2D*	REACTOME_SIGNALING_BY_MODERATE_KINASE_ACTIVITY_BRAF_MUTANTS
17:42454243	*FAM171A2*	REACTOME_SIGNALING_BY_MODERATE_KINASE_ACTIVITY_BRAF_MUTANTS
1:226074321	*ITPKB*	REACTOME_SIGNALING_BY_NODAL
3:49406095	*IP6K2*	REACTOME_SIGNALING_BY_NON_RECEPTOR_TYROSINE_KINASES
3:49424231	*IP6K2*	REACTOME_SIGNALING_BY_NON_RECEPTOR_TYROSINE_KINASES
3:49450137	*IP6K2*	REACTOME_SIGNALING_BY_NON_RECEPTOR_TYROSINE_KINASES
7:23277372	*GPNMB*	REACTOME_SIGNALING_BY_NON_RECEPTOR_TYROSINE_KINASES
7:23282960	*GPNMB*	REACTOME_SIGNALING_BY_NON_RECEPTOR_TYROSINE_KINASES
7:23293746	*GPNMB*	REACTOME_SIGNALING_BY_NON_RECEPTOR_TYROSINE_KINASES
7:23313171	*GPNMB*	REACTOME_SIGNALING_BY_NON_RECEPTOR_TYROSINE_KINASES
12:40614434	*LRRK2_2*	REACTOME_SIGNALING_BY_NON_RECEPTOR_TYROSINE_KINASES
12:40614656	*LRRK2_2*	REACTOME_SIGNALING_BY_NON_RECEPTOR_TYROSINE_KINASES
12:40617231	*LRRK2_2*	REACTOME_SIGNALING_BY_NON_RECEPTOR_TYROSINE_KINASES
12:40653993	*LRRK2_2*	REACTOME_SIGNALING_BY_NON_RECEPTOR_TYROSINE_KINASES
12:40698422	*LRRK2_2*	REACTOME_SIGNALING_BY_NON_RECEPTOR_TYROSINE_KINASES
12:40707861	*LRRK2_2*	REACTOME_SIGNALING_BY_NON_RECEPTOR_TYROSINE_KINASES
12:40734202	*LRRK2_2*	REACTOME_SIGNALING_BY_NON_RECEPTOR_TYROSINE_KINASES

12:40753796	*LRRK2_2*	REACTOME_SIGNALING_BY_NON_RECEPTOR_TYROSINE_KINASES
1:227067656	*ITPKB*	REACTOME_SIGNALING_BY_NOTCH1_IN_CANCER
1:227067656	*ITPKB*	REACTOME_SIGNALING_BY_NOTCH1
12:41144387	*LRRK2_2*	REACTOME_SIGNALING_BY_NOTCH1
12:41147295	*LRRK2_2*	REACTOME_SIGNALING_BY_NOTCH1
12:41195135	*LRRK2_2*	REACTOME_SIGNALING_BY_NOTCH1
12:41251554	*LRRK2_2*	REACTOME_SIGNALING_BY_NOTCH1
12:41255129	*LRRK2_2*	REACTOME_SIGNALING_BY_NOTCH1
12:41297969	*LRRK2_2*	REACTOME_SIGNALING_BY_NOTCH1
12:41407403	*LRRK2_2*	REACTOME_SIGNALING_BY_NOTCH1
19:3008151	*SPPL2B*	REACTOME_SIGNALING_BY_NOTCH1
19:3024077	*SPPL2B*	REACTOME_SIGNALING_BY_NOTCH1
1:227067656	*ITPKB*	REACTOME_SIGNALING_BY_NOTCH2
12:41144387	*LRRK2_2*	REACTOME_SIGNALING_BY_NOTCH2
12:41147295	*LRRK2_2*	REACTOME_SIGNALING_BY_NOTCH2
12:41195135	*LRRK2_2*	REACTOME_SIGNALING_BY_NOTCH2
12:41251554	*LRRK2_2*	REACTOME_SIGNALING_BY_NOTCH2
12:41255129	*LRRK2_2*	REACTOME_SIGNALING_BY_NOTCH2
12:41297969	*LRRK2_2*	REACTOME_SIGNALING_BY_NOTCH2
12:41407403	*LRRK2_2*	REACTOME_SIGNALING_BY_NOTCH2
1:227067656	*ITPKB*	REACTOME_SIGNALING_BY_NOTCH3
1:227067656	*ITPKB*	REACTOME_SIGNALING_BY_NOTCH4
6:32179319	*HLA-DRB5*	REACTOME_SIGNALING_BY_NOTCH4
6:32186193	*HLA-DRB5*	REACTOME_SIGNALING_BY_NOTCH4
1:227067656	*ITPKB*	REACTOME_SIGNALING_BY_NOTCH
6:32179319	*HLA-DRB5*	REACTOME_SIGNALING_BY_NOTCH
6:32186193	*HLA-DRB5*	REACTOME_SIGNALING_BY_NOTCH
12:41144387	*LRRK2_2*	REACTOME_SIGNALING_BY_NOTCH
12:41147295	*LRRK2_2*	REACTOME_SIGNALING_BY_NOTCH
12:41195135	*LRRK2_2*	REACTOME_SIGNALING_BY_NOTCH
12:41251554	*LRRK2_2*	REACTOME_SIGNALING_BY_NOTCH
12:41255129	*LRRK2_2*	REACTOME_SIGNALING_BY_NOTCH
12:41297969	*LRRK2_2*	REACTOME_SIGNALING_BY_NOTCH
12:41407403	*LRRK2_2*	REACTOME_SIGNALING_BY_NOTCH
17:7578671	*V1NB1*	REACTOME_SIGNALING_BY_NOTCH
19:3008151	*SPPL2B*	REACTOME_SIGNALING_BY_NOTCH
19:3024077	*SPPL2B*	REACTOME_SIGNALING_BY_NOTCH
1:171837985	*VAMP4*	REACTOME_SIGNALING_BY_NTRK1_TRKA
1:171945488	*VAMP4*	REACTOME_SIGNALING_BY_NTRK1_TRKA
1:172137119	*VAMP4*	REACTOME_SIGNALING_BY_NTRK1_TRKA
1:172386261	*VAMP4*	REACTOME_SIGNALING_BY_NTRK1_TRKA
3:49406095	*IP6K2*	REACTOME_SIGNALING_BY_NTRK1_TRKA
3:49424231	*IP6K2*	REACTOME_SIGNALING_BY_NTRK1_TRKA
3:49450137	*IP6K2*	REACTOME_SIGNALING_BY_NTRK1_TRKA
5:133558885	*C5orf24*	REACTOME_SIGNALING_BY_NTRK1_TRKA
9:17579690	*SH3GL2_2*	REACTOME_SIGNALING_BY_NTRK1_TRKA
9:17587160	*SH3GL2_2*	REACTOME_SIGNALING_BY_NTRK1_TRKA
9:17609302	*SH3GL2_2*	REACTOME_SIGNALING_BY_NTRK1_TRKA
9:17626762	*SH3GL2_2*	REACTOME_SIGNALING_BY_NTRK1_TRKA
9:17627444	*SH3GL2_2*	REACTOME_SIGNALING_BY_NTRK1_TRKA
9:17642664	*SH3GL2_2*	REACTOME_SIGNALING_BY_NTRK1_TRKA
9:17710876	*SH3GL2_2*	REACTOME_SIGNALING_BY_NTRK1_TRKA
9:17732590	*SH3GL2_2*	REACTOME_SIGNALING_BY_NTRK1_TRKA

9:17737514	*SH3GL2_2*	REACTOME_SIGNALING_BY_NTRK1_TRKA
9:17758945	*SH3GL2_2*	REACTOME_SIGNALING_BY_NTRK1_TRKA
17:41845031	*FAM171A2*	REACTOME_SIGNALING_BY_NTRK1_TRKA
18:40409806	*RIT2*	REACTOME_SIGNALING_BY_NTRK1_TRKA
18:40494893	*RIT2*	REACTOME_SIGNALING_BY_NTRK1_TRKA
18:40543738	*RIT2*	REACTOME_SIGNALING_BY_NTRK1_TRKA
18:40612823	*RIT2*	REACTOME_SIGNALING_BY_NTRK1_TRKA
18:40649778	*RIT2*	REACTOME_SIGNALING_BY_NTRK1_TRKA
18:40671720	*RIT2*	REACTOME_SIGNALING_BY_NTRK1_TRKA
18:40673380	*RIT2*	REACTOME_SIGNALING_BY_NTRK1_TRKA
6:112137847	*FYN*	REACTOME_SIGNALING_BY_NTRK2_TRKB
6:112151452	*FYN*	REACTOME_SIGNALING_BY_NTRK2_TRKB
6:112175069	*FYN*	REACTOME_SIGNALING_BY_NTRK2_TRKB
1:171837985	*VAMP4*	REACTOME_SIGNALING_BY_NTRKS
1:171945488	*VAMP4*	REACTOME_SIGNALING_BY_NTRKS
1:172137119	*VAMP4*	REACTOME_SIGNALING_BY_NTRKS
1:172386261	*VAMP4*	REACTOME_SIGNALING_BY_NTRKS
3:49406095	*IP6K2*	REACTOME_SIGNALING_BY_NTRKS
3:49424231	*IP6K2*	REACTOME_SIGNALING_BY_NTRKS
3:49450137	*IP6K2*	REACTOME_SIGNALING_BY_NTRKS
5:133558885	*C5orf24*	REACTOME_SIGNALING_BY_NTRKS
6:112137847	*FYN*	REACTOME_SIGNALING_BY_NTRKS
6:112151452	*FYN*	REACTOME_SIGNALING_BY_NTRKS
6:112175069	*FYN*	REACTOME_SIGNALING_BY_NTRKS
9:17579690	*SH3GL2_2*	REACTOME_SIGNALING_BY_NTRKS
9:17587160	*SH3GL2_2*	REACTOME_SIGNALING_BY_NTRKS
9:17609302	*SH3GL2_2*	REACTOME_SIGNALING_BY_NTRKS
9:17626762	*SH3GL2_2*	REACTOME_SIGNALING_BY_NTRKS
9:17627444	*SH3GL2_2*	REACTOME_SIGNALING_BY_NTRKS
9:17642664	*SH3GL2_2*	REACTOME_SIGNALING_BY_NTRKS
9:17710876	*SH3GL2_2*	REACTOME_SIGNALING_BY_NTRKS
9:17732590	*SH3GL2_2*	REACTOME_SIGNALING_BY_NTRKS
9:17737514	*SH3GL2_2*	REACTOME_SIGNALING_BY_NTRKS
9:17758945	*SH3GL2_2*	REACTOME_SIGNALING_BY_NTRKS
17:41845031	*FAM171A2*	REACTOME_SIGNALING_BY_NTRKS
18:40409806	*RIT2*	REACTOME_SIGNALING_BY_NTRKS
18:40494893	*RIT2*	REACTOME_SIGNALING_BY_NTRKS
18:40543738	*RIT2*	REACTOME_SIGNALING_BY_NTRKS
18:40612823	*RIT2*	REACTOME_SIGNALING_BY_NTRKS
18:40649778	*RIT2*	REACTOME_SIGNALING_BY_NTRKS
18:40671720	*RIT2*	REACTOME_SIGNALING_BY_NTRKS
18:40673380	*RIT2*	REACTOME_SIGNALING_BY_NTRKS
14:38061742	*MIPOL1*	REACTOME_SIGNALING_BY_NUCLEAR_RECEPTORS
19:2557113	*SPPL2B*	REACTOME_SIGNALING_BY_NUCLEAR_RECEPTORS
19:2563327	*SPPL2B*	REACTOME_SIGNALING_BY_NUCLEAR_RECEPTORS
19:2580057	*SPPL2B*	REACTOME_SIGNALING_BY_NUCLEAR_RECEPTORS
19:2660983	*SPPL2B*	REACTOME_SIGNALING_BY_NUCLEAR_RECEPTORS
17:40392358	*RETREG3*	REACTOME_SIGNALING_BY_PDGF
4:114424939	*CAMK2D*	REACTOME_SIGNALING_BY_RAS_MUTANTS
4:114494052	*CAMK2D*	REACTOME_SIGNALING_BY_RAS_MUTANTS
4:114529492	*CAMK2D*	REACTOME_SIGNALING_BY_RAS_MUTANTS
4:114539964	*CAMK2D*	REACTOME_SIGNALING_BY_RAS_MUTANTS
4:114597918	*CAMK2D*	REACTOME_SIGNALING_BY_RAS_MUTANTS

17:42454243	*FAM171A2*	REACTOME_SIGNALING_BY_RAS_MUTANTS
1:171837985	*VAMP4*	REACTOME_SIGNALING_BY_RECEPTOR_TYROSINE_KINASES
1:171945488	*VAMP4*	REACTOME_SIGNALING_BY_RECEPTOR_TYROSINE_KINASES
1:172137119	*VAMP4*	REACTOME_SIGNALING_BY_RECEPTOR_TYROSINE_KINASES
1:172386261	*VAMP4*	REACTOME_SIGNALING_BY_RECEPTOR_TYROSINE_KINASES
1:227067656	*ITPKB*	REACTOME_SIGNALING_BY_RECEPTOR_TYROSINE_KINASES
3:49162284	*IP6K2*	REACTOME_SIGNALING_BY_RECEPTOR_TYROSINE_KINASES
3:49162583	*IP6K2*	REACTOME_SIGNALING_BY_RECEPTOR_TYROSINE_KINASES
3:49406095	*IP6K2*	REACTOME_SIGNALING_BY_RECEPTOR_TYROSINE_KINASES
3:49424231	*IP6K2*	REACTOME_SIGNALING_BY_RECEPTOR_TYROSINE_KINASES
3:49450137	*IP6K2*	REACTOME_SIGNALING_BY_RECEPTOR_TYROSINE_KINASES
5:133558885	*C5orf24*	REACTOME_SIGNALING_BY_RECEPTOR_TYROSINE_KINASES
6:33153528	*HLA-DRB5*	REACTOME_SIGNALING_BY_RECEPTOR_TYROSINE_KINASES
6:112137847	*FYN*	REACTOME_SIGNALING_BY_RECEPTOR_TYROSINE_KINASES
6:112151452	*FYN*	REACTOME_SIGNALING_BY_RECEPTOR_TYROSINE_KINASES
6:112175069	*FYN*	REACTOME_SIGNALING_BY_RECEPTOR_TYROSINE_KINASES
6:112494762	*FYN*	REACTOME_SIGNALING_BY_RECEPTOR_TYROSINE_KINASES
6:112531401	*FYN*	REACTOME_SIGNALING_BY_RECEPTOR_TYROSINE_KINASES
6:112561154	*FYN*	REACTOME_SIGNALING_BY_RECEPTOR_TYROSINE_KINASES
6:112561430	*FYN*	REACTOME_SIGNALING_BY_RECEPTOR_TYROSINE_KINASES
9:17579690	*SH3GL2_2*	REACTOME_SIGNALING_BY_RECEPTOR_TYROSINE_KINASES
9:17587160	*SH3GL2_2*	REACTOME_SIGNALING_BY_RECEPTOR_TYROSINE_KINASES
9:17609302	*SH3GL2_2*	REACTOME_SIGNALING_BY_RECEPTOR_TYROSINE_KINASES
9:17626762	*SH3GL2_2*	REACTOME_SIGNALING_BY_RECEPTOR_TYROSINE_KINASES
9:17627444	*SH3GL2_2*	REACTOME_SIGNALING_BY_RECEPTOR_TYROSINE_KINASES
9:17642664	*SH3GL2_2*	REACTOME_SIGNALING_BY_RECEPTOR_TYROSINE_KINASES
9:17710876	*SH3GL2_2*	REACTOME_SIGNALING_BY_RECEPTOR_TYROSINE_KINASES
9:17732590	*SH3GL2_2*	REACTOME_SIGNALING_BY_RECEPTOR_TYROSINE_KINASES
9:17737514	*SH3GL2_2*	REACTOME_SIGNALING_BY_RECEPTOR_TYROSINE_KINASES
9:17758945	*SH3GL2_2*	REACTOME_SIGNALING_BY_RECEPTOR_TYROSINE_KINASES
12:124219791	*HIP1R*	REACTOME_SIGNALING_BY_RECEPTOR_TYROSINE_KINASES
17:7101771	*V1NB1*	REACTOME_SIGNALING_BY_RECEPTOR_TYROSINE_KINASES
17:39883520	*RETREG3*	REACTOME_SIGNALING_BY_RECEPTOR_TYROSINE_KINASES
17:40392358	*RETREG3*	REACTOME_SIGNALING_BY_RECEPTOR_TYROSINE_KINASES
17:41845031	*FAM171A2*	REACTOME_SIGNALING_BY_RECEPTOR_TYROSINE_KINASES
17:42990894	*CRHR1_2*	REACTOME_SIGNALING_BY_RECEPTOR_TYROSINE_KINASES
18:40409806	*RIT2*	REACTOME_SIGNALING_BY_RECEPTOR_TYROSINE_KINASES
18:40494893	*RIT2*	REACTOME_SIGNALING_BY_RECEPTOR_TYROSINE_KINASES
18:40543738	*RIT2*	REACTOME_SIGNALING_BY_RECEPTOR_TYROSINE_KINASES
18:40612823	*RIT2*	REACTOME_SIGNALING_BY_RECEPTOR_TYROSINE_KINASES
18:40649778	*RIT2*	REACTOME_SIGNALING_BY_RECEPTOR_TYROSINE_KINASES
18:40671720	*RIT2*	REACTOME_SIGNALING_BY_RECEPTOR_TYROSINE_KINASES
18:40673380	*RIT2*	REACTOME_SIGNALING_BY_RECEPTOR_TYROSINE_KINASES
1:155975202	*GBAP1*	REACTOME_SIGNALING_BY_RHO_GTPASES
1:161035780	*FCGR2A*	REACTOME_SIGNALING_BY_RHO_GTPASES
2:169747216	*STK39*	REACTOME_SIGNALING_BY_RHO_GTPASES
3:49406095	*IP6K2*	REACTOME_SIGNALING_BY_RHO_GTPASES
3:49424231	*IP6K2*	REACTOME_SIGNALING_BY_RHO_GTPASES
3:49450137	*IP6K2*	REACTOME_SIGNALING_BY_RHO_GTPASES
4:89711319	*SNCA_2*	REACTOME_SIGNALING_BY_RHO_GTPASES
4:89740128	*SNCA_2*	REACTOME_SIGNALING_BY_RHO_GTPASES
4:90002389	*SNCA_2*	REACTOME_SIGNALING_BY_RHO_GTPASES
5:59958372	*ELOVL7*	REACTOME_SIGNALING_BY_RHO_GTPASES

5:133558885	*C5orf24*	REACTOME_SIGNALING_BY_RHO_GTPASES
12:122816734	*HIP1R*	REACTOME_SIGNALING_BY_RHO_GTPASES
12:122817580	*HIP1R*	REACTOME_SIGNALING_BY_RHO_GTPASES
12:122853197	*HIP1R*	REACTOME_SIGNALING_BY_RHO_GTPASES
12:123057792	*HIP1R*	REACTOME_SIGNALING_BY_RHO_GTPASES
12:123110365	*HIP1R*	REACTOME_SIGNALING_BY_RHO_GTPASES
12:123110785	*HIP1R*	REACTOME_SIGNALING_BY_RHO_GTPASES
14:56110265	*GCH1*	REACTOME_SIGNALING_BY_RHO_GTPASES
17:7101771	*V1NB1*	REACTOME_SIGNALING_BY_RHO_GTPASES
17:7129974	*V1NB1*	REACTOME_SIGNALING_BY_RHO_GTPASES
17:43302259	*CRHR1_2*	REACTOME_SIGNALING_BY_RHO_GTPASES
17:43472507	*CRHR1_2*	REACTOME_SIGNALING_BY_RHO_GTPASES
3:48835245	*IP6K2*	REACTOME_SIGNALING_BY_ROBO_RECEPTORS
3:49406095	*IP6K2*	REACTOME_SIGNALING_BY_ROBO_RECEPTORS
3:49424231	*IP6K2*	REACTOME_SIGNALING_BY_ROBO_RECEPTORS
3:49450137	*IP6K2*	REACTOME_SIGNALING_BY_ROBO_RECEPTORS
3:49561208	*IP6K2*	REACTOME_SIGNALING_BY_ROBO_RECEPTORS
6:112137847	*FYN*	REACTOME_SIGNALING_BY_SCF_KIT
6:112151452	*FYN*	REACTOME_SIGNALING_BY_SCF_KIT
6:112175069	*FYN*	REACTOME_SIGNALING_BY_SCF_KIT
17:40392358	*RETREG3*	REACTOME_SIGNALING_BY_SCF_KIT
1:226568665	*ITPKB*	REACTOME_SIGNALING_BY_TGF_BETA_FAMILY_MEMBERS
2:135708115	*TMEM163*	REACTOME_SIGNALING_BY_TGF_BETA_FAMILY_MEMBERS
3:49406095	*IP6K2*	REACTOME_SIGNALING_BY_TGF_BETA_FAMILY_MEMBERS
3:49424231	*IP6K2*	REACTOME_SIGNALING_BY_TGF_BETA_FAMILY_MEMBERS
3:49450137	*IP6K2*	REACTOME_SIGNALING_BY_TGF_BETA_FAMILY_MEMBERS
19:2251817	*SPPL2B*	REACTOME_SIGNALING_BY_TGF_BETA_FAMILY_MEMBERS
1:226568665	*ITPKB*	REACTOME_SIGNALING_BY_TGF_BETA_RECEPTOR_COMPLEX
2:135708115	*TMEM163*	REACTOME_SIGNALING_BY_TGF_BETA_RECEPTOR_COMPLEX
3:49406095	*IP6K2*	REACTOME_SIGNALING_BY_TGF_BETA_RECEPTOR_COMPLEX
3:49424231	*IP6K2*	REACTOME_SIGNALING_BY_TGF_BETA_RECEPTOR_COMPLEX
3:49450137	*IP6K2*	REACTOME_SIGNALING_BY_TGF_BETA_RECEPTOR_COMPLEX
6:112137847	*FYN*	REACTOME_SIGNALING_BY_THE_B_CELL_RECEPTOR_BCR
6:112151452	*FYN*	REACTOME_SIGNALING_BY_THE_B_CELL_RECEPTOR_BCR
6:112175069	*FYN*	REACTOME_SIGNALING_BY_THE_B_CELL_RECEPTOR_BCR
8:11353586	*CTSB*	REACTOME_SIGNALING_BY_THE_B_CELL_RECEPTOR_BCR
8:11363667	*CTSB*	REACTOME_SIGNALING_BY_THE_B_CELL_RECEPTOR_BCR
8:11381148	*CTSB*	REACTOME_SIGNALING_BY_THE_B_CELL_RECEPTOR_BCR
10:103185542	*GBF1*	REACTOME_SIGNALING_BY_THE_B_CELL_RECEPTOR_BCR
10:103305087	*GBF1*	REACTOME_SIGNALING_BY_THE_B_CELL_RECEPTOR_BCR
10:103308244	*GBF1*	REACTOME_SIGNALING_BY_THE_B_CELL_RECEPTOR_BCR
3:49406095	*IP6K2*	REACTOME_SIGNALING_BY_VEGF
3:49424231	*IP6K2*	REACTOME_SIGNALING_BY_VEGF
3:49450137	*IP6K2*	REACTOME_SIGNALING_BY_VEGF
6:112137847	*FYN*	REACTOME_SIGNALING_BY_VEGF
6:112151452	*FYN*	REACTOME_SIGNALING_BY_VEGF
6:112175069	*FYN*	REACTOME_SIGNALING_BY_VEGF
17:39883520	*RETREG3*	REACTOME_SIGNALING_BY_VEGF
5:133558885	*C5orf24*	REACTOME_SIGNALING_BY_WNT_IN_CANCER
3:49406095	*IP6K2*	REACTOME_SIGNALING_BY_WNT
3:49424231	*IP6K2*	REACTOME_SIGNALING_BY_WNT
3:49450137	*IP6K2*	REACTOME_SIGNALING_BY_WNT
5:133558885	*C5orf24*	REACTOME_SIGNALING_BY_WNT

10:103185542	*GBF1*	REACTOME_SIGNALING_BY_WNT
10:103305087	*GBF1*	REACTOME_SIGNALING_BY_WNT
10:103308244	*GBF1*	REACTOME_SIGNALING_BY_WNT
17:7129974	*V1NB1*	REACTOME_SIGNALING_BY_WNT
17:44843651	*WNT3*	REACTOME_SIGNALING_BY_WNT
17:44870978	*WNT3*	REACTOME_SIGNALING_BY_WNT
17:44871422	*WNT3*	REACTOME_SIGNALING_BY_WNT
17:44888209	*WNT3*	REACTOME_SIGNALING_BY_WNT
17:44888715	*WNT3*	REACTOME_SIGNALING_BY_WNT
17:44906949	*WNT3*	REACTOME_SIGNALING_BY_WNT
17:44912901	*WNT3*	REACTOME_SIGNALING_BY_WNT
17:44931399	*WNT3*	REACTOME_SIGNALING_BY_WNT
17:44947756	*WNT3*	REACTOME_SIGNALING_BY_WNT
19:2557113	*SPPL2B*	REACTOME_SIGNALING_BY_WNT
19:2563327	*SPPL2B*	REACTOME_SIGNALING_BY_WNT
19:2580057	*SPPL2B*	REACTOME_SIGNALING_BY_WNT
19:2660983	*SPPL2B*	REACTOME_SIGNALING_BY_WNT
19:3008151	*SPPL2B*	REACTOME_SIGNALING_BY_WNT
19:3024077	*SPPL2B*	REACTOME_SIGNALING_BY_WNT
18:40409806	*RIT2*	REACTOME_SIGNALLING_TO_ERKS
18:40494893	*RIT2*	REACTOME_SIGNALLING_TO_ERKS
18:40543738	*RIT2*	REACTOME_SIGNALLING_TO_ERKS
18:40612823	*RIT2*	REACTOME_SIGNALLING_TO_ERKS
18:40649778	*RIT2*	REACTOME_SIGNALLING_TO_ERKS
18:40671720	*RIT2*	REACTOME_SIGNALLING_TO_ERKS
18:40673380	*RIT2*	REACTOME_SIGNALLING_TO_ERKS
18:40409806	*RIT2*	REACTOME_SIGNALLING_TO_P38_VIA_RIT_AND_RIN
18:40494893	*RIT2*	REACTOME_SIGNALLING_TO_P38_VIA_RIT_AND_RIN
18:40543738	*RIT2*	REACTOME_SIGNALLING_TO_P38_VIA_RIT_AND_RIN
18:40612823	*RIT2*	REACTOME_SIGNALLING_TO_P38_VIA_RIT_AND_RIN
18:40649778	*RIT2*	REACTOME_SIGNALLING_TO_P38_VIA_RIT_AND_RIN
18:40671720	*RIT2*	REACTOME_SIGNALLING_TO_P38_VIA_RIT_AND_RIN
18:40673380	*RIT2*	REACTOME_SIGNALLING_TO_P38_VIA_RIT_AND_RIN
17:42454243	*FAM171A2*	REACTOME_SIGNAL_TRANSDUCTION_BY_L1
1:205884274	*RAB29*	REACTOME_SLC_MEDIATED_TRANSMEMBRANE_TRANSPORT
2:103267601	*MAP4K4*	REACTOME_SLC_MEDIATED_TRANSMEMBRANE_TRANSPORT
2:103282717	*MAP4K4*	REACTOME_SLC_MEDIATED_TRANSMEMBRANE_TRANSPORT
2:103317676	*MAP4K4*	REACTOME_SLC_MEDIATED_TRANSMEMBRANE_TRANSPORT
5:101579003	*PAM*	REACTOME_SLC_MEDIATED_TRANSMEMBRANE_TRANSPORT
6:31846234	*HLA-DRB5*	REACTOME_SLC_MEDIATED_TRANSMEMBRANE_TRANSPORT
8:17390689	*FGF20*	REACTOME_SLC_MEDIATED_TRANSMEMBRANE_TRANSPORT
8:17402274	*FGF20*	REACTOME_SLC_MEDIATED_TRANSMEMBRANE_TRANSPORT
8:17427186	*FGF20*	REACTOME_SLC_MEDIATED_TRANSMEMBRANE_TRANSPORT
12:40240262	*LRRK2_2*	REACTOME_SLC_MEDIATED_TRANSMEMBRANE_TRANSPORT
12:40252360	*LRRK2_2*	REACTOME_SLC_MEDIATED_TRANSMEMBRANE_TRANSPORT
12:40267539	*LRRK2_2*	REACTOME_SLC_MEDIATED_TRANSMEMBRANE_TRANSPORT
12:40302599	*LRRK2_2*	REACTOME_SLC_MEDIATED_TRANSMEMBRANE_TRANSPORT
12:40348056	*LRRK2_2*	REACTOME_SLC_MEDIATED_TRANSMEMBRANE_TRANSPORT
12:40349236	*LRRK2_2*	REACTOME_SLC_MEDIATED_TRANSMEMBRANE_TRANSPORT
12:40378773	*LRRK2_2*	REACTOME_SLC_MEDIATED_TRANSMEMBRANE_TRANSPORT
12:40393852	*LRRK2_2*	REACTOME_SLC_MEDIATED_TRANSMEMBRANE_TRANSPORT
12:40405478	*LRRK2_2*	REACTOME_SLC_MEDIATED_TRANSMEMBRANE_TRANSPORT
12:40413305	*LRRK2_2*	REACTOME_SLC_MEDIATED_TRANSMEMBRANE_TRANSPORT

12:40414626	*LRRK2_2*	REACTOME_SLC_MEDIATED_TRANSMEMBRANE_TRANSPORT
12:40437969	*LRRK2_2*	REACTOME_SLC_MEDIATED_TRANSMEMBRANE_TRANSPORT
12:46618863	*SCAF11*	REACTOME_SLC_MEDIATED_TRANSMEMBRANE_TRANSPORT
12:47192323	*SCAF11*	REACTOME_SLC_MEDIATED_TRANSMEMBRANE_TRANSPORT
12:47222402	*SCAF11*	REACTOME_SLC_MEDIATED_TRANSMEMBRANE_TRANSPORT
17:6594869	*V1NB1*	REACTOME_SLC_MEDIATED_TRANSMEMBRANE_TRANSPORT
17:6612244	*V1NB1*	REACTOME_SLC_MEDIATED_TRANSMEMBRANE_TRANSPORT
17:42332803	*FAM171A2*	REACTOME_SLC_MEDIATED_TRANSMEMBRANE_TRANSPORT
17:42338993	*FAM171A2*	REACTOME_SLC_MEDIATED_TRANSMEMBRANE_TRANSPORT
17:42332803	*FAM171A2*	REACTOME_SLC_TRANSPORTER_DISORDERS
17:42338993	*FAM171A2*	REACTOME_SLC_TRANSPORTER_DISORDERS
2:135708115	*TMEM163*	REACTOME_SMAD2_SMAD3:SMAD4_HETEROTRIMER_REGULATES_TRANSCRIPTION
8:22421355	*BIN3*	REACTOME_SMOOTH_MUSCLE_CONTRACTION
17:6594869	*V1NB1*	REACTOME_SODIUM_COUPLED_SULPHATE_DI_AND_TRI_CARBOXYLATE_TRANSPORTERS
17:6612244	*V1NB1*	REACTOME_SODIUM_COUPLED_SULPHATE_DI_AND_TRI_CARBOXYLATE_TRANSPORTERS
2:103267601	*MAP4K4*	REACTOME_SODIUM_PROTON_EXCHANGERS
2:103282717	*MAP4K4*	REACTOME_SODIUM_PROTON_EXCHANGERS
2:103317676	*MAP4K4*	REACTOME_SODIUM_PROTON_EXCHANGERS
5:134307319	*C5orf24*	REACTOME_SPERM_MOTILITY_AND_TAXES
5:134315853	*C5orf24*	REACTOME_SPERM_MOTILITY_AND_TAXES
1:226497419	*ITPKB*	REACTOME_S_PHASE
3:48199695	*IP6K2*	REACTOME_S_PHASE
3:48214438	*IP6K2*	REACTOME_S_PHASE
12:133206648	*FBRSL1*	REACTOME_S_PHASE
12:133223685	*FBRSL1*	REACTOME_S_PHASE
14:74640180	*RPS6KL1*	REACTOME_S_PHASE
17:45215865	*WNT3*	REACTOME_S_PHASE
17:45244822	*WNT3*	REACTOME_S_PHASE
20:5934513	*CRLS1*	REACTOME_S_PHASE
20:5935522	*CRLS1*	REACTOME_S_PHASE
20:5968737	*CRLS1*	REACTOME_S_PHASE
20:5968964	*CRLS1*	REACTOME_S_PHASE
2:169389091	*STK39*	REACTOME_SPHINGOLIPID_DE_NOVO_BIOSYNTHESIS
2:169518974	*STK39*	REACTOME_SPHINGOLIPID_DE_NOVO_BIOSYNTHESIS
2:169519063	*STK39*	REACTOME_SPHINGOLIPID_DE_NOVO_BIOSYNTHESIS
2:169520026	*STK39*	REACTOME_SPHINGOLIPID_DE_NOVO_BIOSYNTHESIS
2:169550660	*STK39*	REACTOME_SPHINGOLIPID_DE_NOVO_BIOSYNTHESIS
2:169561027	*STK39*	REACTOME_SPHINGOLIPID_DE_NOVO_BIOSYNTHESIS
2:169597744	*STK39*	REACTOME_SPHINGOLIPID_DE_NOVO_BIOSYNTHESIS
3:160668220	*SPTSSB*	REACTOME_SPHINGOLIPID_DE_NOVO_BIOSYNTHESIS
3:161068566	*SPTSSB*	REACTOME_SPHINGOLIPID_DE_NOVO_BIOSYNTHESIS
3:161075189	*SPTSSB*	REACTOME_SPHINGOLIPID_DE_NOVO_BIOSYNTHESIS
1:155205634	*GBAP1*	REACTOME_SPHINGOLIPID_METABOLISM
2:169389091	*STK39*	REACTOME_SPHINGOLIPID_METABOLISM
2:169518974	*STK39*	REACTOME_SPHINGOLIPID_METABOLISM
2:169519063	*STK39*	REACTOME_SPHINGOLIPID_METABOLISM
2:169520026	*STK39*	REACTOME_SPHINGOLIPID_METABOLISM
2:169550660	*STK39*	REACTOME_SPHINGOLIPID_METABOLISM
2:169561027	*STK39*	REACTOME_SPHINGOLIPID_METABOLISM
2:169597744	*STK39*	REACTOME_SPHINGOLIPID_METABOLISM
3:160668220	*SPTSSB*	REACTOME_SPHINGOLIPID_METABOLISM
3:160817877	*SPTSSB*	REACTOME_SPHINGOLIPID_METABOLISM
3:161068566	*SPTSSB*	REACTOME_SPHINGOLIPID_METABOLISM

3:161075189	*SPTSSB*	REACTOME_SPHINGOLIPID_METABOLISM
4:114899005	*CAMK2D*	REACTOME_SPHINGOLIPID_METABOLISM
14:88372366	*GALC*	REACTOME_SPHINGOLIPID_METABOLISM
14:88393900	*GALC*	REACTOME_SPHINGOLIPID_METABOLISM
14:88415750	*GALC*	REACTOME_SPHINGOLIPID_METABOLISM
14:88443553	*GALC*	REACTOME_SPHINGOLIPID_METABOLISM
5:133558885	*C5orf24*	REACTOME_SPRY_REGULATION_OF_FGF_SIGNALING
17:7578671	*V1NB1*	REACTOME_STABILIZATION_OF_P53
17:40392358	*RETREG3*	REACTOME_STAT5_ACTIVATION
4:170563153	*CLCN3*	REACTOME_STIMULI_SENSING_CHANNELS
4:170569172	*CLCN3*	REACTOME_STIMULI_SENSING_CHANNELS
4:170620812	*CLCN3*	REACTOME_STIMULI_SENSING_CHANNELS
12:45755099	*SCAF11*	REACTOME_STIMULI_SENSING_CHANNELS
17:40938261	*RETREG3*	REACTOME_STIMULI_SENSING_CHANNELS
3:18395010	*SATB1*	REACTOME_SUMOYLATION_OF_CHROMATIN_ORGANIZATION_PROTEINS
3:18472577	*SATB1*	REACTOME_SUMOYLATION_OF_CHROMATIN_ORGANIZATION_PROTEINS
1:226568665	*ITPKB*	REACTOME_SUMOYLATION_OF_DNA_DAMAGE_RESPONSE_AND_REPAIR_PROTEINS
2:17941653	*KCNS3*	REACTOME_SUMOYLATION_OF_DNA_DAMAGE_RESPONSE_AND_REPAIR_PROTEINS
2:17963171	*KCNS3*	REACTOME_SUMOYLATION_OF_DNA_DAMAGE_RESPONSE_AND_REPAIR_PROTEINS
15:61016235	*VPS13C*	REACTOME_SUMOYLATION_OF_INTRACELLULAR_RECEPTORS
15:61065568	*VPS13C*	REACTOME_SUMOYLATION_OF_INTRACELLULAR_RECEPTORS
15:61094298	*VPS13C*	REACTOME_SUMOYLATION_OF_INTRACELLULAR_RECEPTORS
15:61140736	*VPS13C*	REACTOME_SUMOYLATION_OF_INTRACELLULAR_RECEPTORS
15:61142062	*VPS13C*	REACTOME_SUMOYLATION_OF_INTRACELLULAR_RECEPTORS
15:61162571	*VPS13C*	REACTOME_SUMOYLATION_OF_INTRACELLULAR_RECEPTORS
15:61191617	*VPS13C*	REACTOME_SUMOYLATION_OF_INTRACELLULAR_RECEPTORS
15:61195427	*VPS13C*	REACTOME_SUMOYLATION_OF_INTRACELLULAR_RECEPTORS
15:61221682	*VPS13C*	REACTOME_SUMOYLATION_OF_INTRACELLULAR_RECEPTORS
15:61298486	*VPS13C*	REACTOME_SUMOYLATION_OF_INTRACELLULAR_RECEPTORS
15:61375480	*VPS13C*	REACTOME_SUMOYLATION_OF_INTRACELLULAR_RECEPTORS
15:61434581	*VPS13C*	REACTOME_SUMOYLATION_OF_INTRACELLULAR_RECEPTORS
15:61467078	*VPS13C*	REACTOME_SUMOYLATION_OF_INTRACELLULAR_RECEPTORS
15:61470808	*VPS13C*	REACTOME_SUMOYLATION_OF_INTRACELLULAR_RECEPTORS
15:61500263	*VPS13C*	REACTOME_SUMOYLATION_OF_INTRACELLULAR_RECEPTORS
6:33287183	*HLA-DRB5*	REACTOME_SUMOYLATION_OF_TRANSCRIPTION_COFACTORS
17:7578671	*V1NB1*	REACTOME_SUMOYLATION_OF_TRANSCRIPTION_FACTORS
1:226568665	*ITPKB*	REACTOME_SUMOYLATION
2:17941653	*KCNS3*	REACTOME_SUMOYLATION
2:17963171	*KCNS3*	REACTOME_SUMOYLATION
3:18395010	*SATB1*	REACTOME_SUMOYLATION
3:18472577	*SATB1*	REACTOME_SUMOYLATION
6:33287183	*HLA-DRB5*	REACTOME_SUMOYLATION
15:61016235	*VPS13C*	REACTOME_SUMOYLATION
15:61065568	*VPS13C*	REACTOME_SUMOYLATION
15:61094298	*VPS13C*	REACTOME_SUMOYLATION
15:61140736	*VPS13C*	REACTOME_SUMOYLATION
15:61142062	*VPS13C*	REACTOME_SUMOYLATION
15:61162571	*VPS13C*	REACTOME_SUMOYLATION
15:61191617	*VPS13C*	REACTOME_SUMOYLATION
15:61195427	*VPS13C*	REACTOME_SUMOYLATION
15:61221682	*VPS13C*	REACTOME_SUMOYLATION
15:61298486	*VPS13C*	REACTOME_SUMOYLATION
15:61375480	*VPS13C*	REACTOME_SUMOYLATION

15:61434581	*VPS13C*	REACTOME_SUMOYLATION
15:61467078	*VPS13C*	REACTOME_SUMOYLATION
15:61470808	*VPS13C*	REACTOME_SUMOYLATION
15:61500263	*VPS13C*	REACTOME_SUMOYLATION
17:7578671	*V1NB1*	REACTOME_SUMOYLATION
8:17014585	*FGF20*	REACTOME_SURFACTANT_METABOLISM
8:17030852	*FGF20*	REACTOME_SURFACTANT_METABOLISM
8:17053322	*FGF20*	REACTOME_SURFACTANT_METABOLISM
17:45215865	*WNT3*	REACTOME_SWITCHING_OF_ORIGINS_TO_A_POST_REPLICATIVE_STATE
17:45244822	*WNT3*	REACTOME_SWITCHING_OF_ORIGINS_TO_A_POST_REPLICATIVE_STATE
20:5934513	*CRLS1*	REACTOME_SWITCHING_OF_ORIGINS_TO_A_POST_REPLICATIVE_STATE
20:5935522	*CRLS1*	REACTOME_SWITCHING_OF_ORIGINS_TO_A_POST_REPLICATIVE_STATE
20:5968737	*CRLS1*	REACTOME_SWITCHING_OF_ORIGINS_TO_A_POST_REPLICATIVE_STATE
20:5968964	*CRLS1*	REACTOME_SWITCHING_OF_ORIGINS_TO_A_POST_REPLICATIVE_STATE
17:7101771	*V1NB1*	REACTOME_SYNAPTIC_ADHESION_LIKE_MOLECULES
17:7983799	*V1NB1*	REACTOME_SYNTHESIS_OF_12_EICOSATETRAENOIC_ACID_DERIVATIVES
17:8019125	*V1NB1*	REACTOME_SYNTHESIS_OF_12_EICOSATETRAENOIC_ACID_DERIVATIVES
5:133710339	*C5orf24*	REACTOME_SYNTHESIS_OF_ACTIVE_UBIQUITIN:_ROLES_OF_E1_AND_E2_ENZYMES
9:33845203	*UBAP2*	REACTOME_SYNTHESIS_OF_ACTIVE_UBIQUITIN:_ROLES_OF_E1_AND_E2_ENZYMES
16:30999462	*SETD1A*	REACTOME_SYNTHESIS_OF_BILE_ACIDS_AND_BILE_SALTS
16:30999462	*SETD1A*	REACTOME_SYNTHESIS_OF_BILE_ACIDS_AND_BILE_SALTS_VIA_24_HYDROXYCHOLESTEROL
16:30999462	*SETD1A*	REACTOME_SYNTHESIS_OF_BILE_ACIDS_AND_BILE_SALTS_VIA_27_HYDROXYCHOLESTEROL
16:30999462	*SETD1A*	REACTOME_SYNTHESIS_OF_BILE_ACIDS_AND_BILE_SALTS_VIA_7ALPHA_HYDROXYCHOLESTEROL
1:172386261	*VAMP4*	REACTOME_SYNTHESIS_OF_GLYCOSYLPHOSPHATIDYLINOSITOL_GPI
1:172388441	*VAMP4*	REACTOME_SYNTHESIS_OF_GLYCOSYLPHOSPHATIDYLINOSITOL_GPI
1:172389198	*VAMP4*	REACTOME_SYNTHESIS_OF_GLYCOSYLPHOSPHATIDYLINOSITOL_GPI
8:17161374	*FGF20*	REACTOME_SYNTHESIS_OF_IP2_IP_AND_INS_IN_THE_CYTOSOL
8:17176046	*FGF20*	REACTOME_SYNTHESIS_OF_IP2_IP_AND_INS_IN_THE_CYTOSOL
8:17243260	*FGF20*	REACTOME_SYNTHESIS_OF_IP2_IP_AND_INS_IN_THE_CYTOSOL
8:17260937	*FGF20*	REACTOME_SYNTHESIS_OF_IP2_IP_AND_INS_IN_THE_CYTOSOL
1:226907849	*ITPKB*	REACTOME_SYNTHESIS_OF_IP3_AND_IP4_IN_THE_CYTOSOL
1:226910340	*ITPKB*	REACTOME_SYNTHESIS_OF_IP3_AND_IP4_IN_THE_CYTOSOL
1:226916078	*ITPKB*	REACTOME_SYNTHESIS_OF_IP3_AND_IP4_IN_THE_CYTOSOL
17:43195981	*CRHR1_2*	REACTOME_SYNTHESIS_OF_IP3_AND_IP4_IN_THE_CYTOSOL
2:96858914	*KCNIP3*	REACTOME_SYNTHESIS_OF_PC
6:31846234	*HLA-DRB5*	REACTOME_SYNTHESIS_OF_PC
10:121505277	*INPP5F*	REACTOME_SYNTHESIS_OF_PIPS_AT_THE_EARLY_ENDOSOME_MEMBRANE
10:121538407	*INPP5F*	REACTOME_SYNTHESIS_OF_PIPS_AT_THE_EARLY_ENDOSOME_MEMBRANE
10:121560563	*INPP5F*	REACTOME_SYNTHESIS_OF_PIPS_AT_THE_EARLY_ENDOSOME_MEMBRANE
8:17161374	*FGF20*	REACTOME_SYNTHESIS_OF_PIPS_AT_THE_LATE_ENDOSOME_MEMBRANE
8:17176046	*FGF20*	REACTOME_SYNTHESIS_OF_PIPS_AT_THE_LATE_ENDOSOME_MEMBRANE
8:17243260	*FGF20*	REACTOME_SYNTHESIS_OF_PIPS_AT_THE_LATE_ENDOSOME_MEMBRANE
8:17260937	*FGF20*	REACTOME_SYNTHESIS_OF_PIPS_AT_THE_LATE_ENDOSOME_MEMBRANE
12:123561638	*HIP1R*	REACTOME_SYNTHESIS_OF_PI
12:123585705	*HIP1R*	REACTOME_SYNTHESIS_OF_PI
16:29871730	*CD19*	REACTOME_SYNTHESIS_OF_PI
17:6370241	*V1NB1*	REACTOME_SYNTHESIS_OF_PI
17:6406333	*V1NB1*	REACTOME_SYNTHESIS_OF_PI
5:60053220	*ELOVL7*	REACTOME_SYNTHESIS_OF_VERY_LONG_CHAIN_FATTY_ACYL_COAS
5:60085489	*ELOVL7*	REACTOME_SYNTHESIS_OF_VERY_LONG_CHAIN_FATTY_ACYL_COAS
5:60093890	*ELOVL7*	REACTOME_SYNTHESIS_OF_VERY_LONG_CHAIN_FATTY_ACYL_COAS
7:66487445	*GS1-124K5.11*	REACTOME_SYNTHESIS_OF_WYBUTOSINE_AT_G37_OF_TRNA_PHE
7:66543959	*GS1-124K5.11*	REACTOME_SYNTHESIS_OF_WYBUTOSINE_AT_G37_OF_TRNA_PHE

7:66638082	*GS1-124K5.11*	REACTOME_SYNTHESIS_OF_WYBUTOSINE_AT_G37_OF_TRNA_PHE
8:11558567	*CTSB*	REACTOME_SYNTHESIS_SECRETION_AND_INACTIVATION_OF_GLUCOSE_DEPENDENT_INSULINOTROPIC_POLYPEPTIDE_GIP
8:11599269	*CTSB*	REACTOME_SYNTHESIS_SECRETION_AND_INACTIVATION_OF_GLUCOSE_DEPENDENT_INSULINOTROPIC_POLYPEPTIDE_GIP
6:32151443	*HLA-DRB5*	REACTOME_TAK1_ACTIVATES_NFKB_BY_PHOSPHORYLATION_AND_ACTIVATION_OF_IKKS_COMPLEX
16:50730088	*CASC16*	REACTOME_TAK1_ACTIVATES_NFKB_BY_PHOSPHORYLATION_AND_ACTIVATION_OF_IKKS_COMPLEX
16:50730446	*CASC16*	REACTOME_TAK1_ACTIVATES_NFKB_BY_PHOSPHORYLATION_AND_ACTIVATION_OF_IKKS_COMPLEX
16:50745926	*CASC16*	REACTOME_TAK1_ACTIVATES_NFKB_BY_PHOSPHORYLATION_AND_ACTIVATION_OF_IKKS_COMPLEX
14:88681675	*GALC*	REACTOME_TANDEM_PORE_DOMAIN_POTASSIUM_CHANNELS
14:88709038	*GALC*	REACTOME_TANDEM_PORE_DOMAIN_POTASSIUM_CHANNELS
14:88738350	*GALC*	REACTOME_TANDEM_PORE_DOMAIN_POTASSIUM_CHANNELS
14:88769689	*GALC*	REACTOME_TANDEM_PORE_DOMAIN_POTASSIUM_CHANNELS
14:88778263	*GALC*	REACTOME_TANDEM_PORE_DOMAIN_POTASSIUM_CHANNELS
5:133558885	*C5orf24*	REACTOME_TCF_DEPENDENT_SIGNALING_IN_RESPONSE_TO_WNT
10:103185542	*GBF1*	REACTOME_TCF_DEPENDENT_SIGNALING_IN_RESPONSE_TO_WNT
10:103305087	*GBF1*	REACTOME_TCF_DEPENDENT_SIGNALING_IN_RESPONSE_TO_WNT
10:103308244	*GBF1*	REACTOME_TCF_DEPENDENT_SIGNALING_IN_RESPONSE_TO_WNT
17:7129974	*V1NB1*	REACTOME_TCF_DEPENDENT_SIGNALING_IN_RESPONSE_TO_WNT
17:44843651	*WNT3*	REACTOME_TCF_DEPENDENT_SIGNALING_IN_RESPONSE_TO_WNT
17:44870978	*WNT3*	REACTOME_TCF_DEPENDENT_SIGNALING_IN_RESPONSE_TO_WNT
17:44871422	*WNT3*	REACTOME_TCF_DEPENDENT_SIGNALING_IN_RESPONSE_TO_WNT
17:44888209	*WNT3*	REACTOME_TCF_DEPENDENT_SIGNALING_IN_RESPONSE_TO_WNT
17:44888715	*WNT3*	REACTOME_TCF_DEPENDENT_SIGNALING_IN_RESPONSE_TO_WNT
17:44906949	*WNT3*	REACTOME_TCF_DEPENDENT_SIGNALING_IN_RESPONSE_TO_WNT
19:3008151	*SPPL2B*	REACTOME_TCF_DEPENDENT_SIGNALING_IN_RESPONSE_TO_WNT
19:3024077	*SPPL2B*	REACTOME_TCF_DEPENDENT_SIGNALING_IN_RESPONSE_TO_WNT
6:32602372	*HLA-DRB5*	REACTOME_TCR_SIGNALING
10:103185542	*GBF1*	REACTOME_TCR_SIGNALING
10:103305087	*GBF1*	REACTOME_TCR_SIGNALING
10:103308244	*GBF1*	REACTOME_TCR_SIGNALING
12:133206648	*FBRSL1*	REACTOME_TELOMERE_C_STRAND_LAGGING_STRAND_SYNTHESIS
12:133223685	*FBRSL1*	REACTOME_TELOMERE_C_STRAND_LAGGING_STRAND_SYNTHESIS
12:133206648	*FBRSL1*	REACTOME_TELOMERE_MAINTENANCE
12:133223685	*FBRSL1*	REACTOME_TELOMERE_MAINTENANCE
12:133206648	*FBRSL1*	REACTOME_TERMINATION_OF_TRANSLESION_DNA_SYNTHESIS
12:133223685	*FBRSL1*	REACTOME_TERMINATION_OF_TRANSLESION_DNA_SYNTHESIS
14:55348869	*GCH1*	REACTOME_TETRAHYDROBIOPTERIN_BH4_SYNTHESIS_RECYCLING_SALVAGE_AND_REGULATION
3:49406095	*IP6K2*	REACTOME_TGF_BETA_RECEPTOR_SIGNALING_IN_EMT_EPITHELIAL_TO_MESENCHYMAL_TRANSITION
3:49424231	*IP6K2*	REACTOME_TGF_BETA_RECEPTOR_SIGNALING_IN_EMT_EPITHELIAL_TO_MESENCHYMAL_TRANSITION
3:49450137	*IP6K2*	REACTOME_TGF_BETA_RECEPTOR_SIGNALING_IN_EMT_EPITHELIAL_TO_MESENCHYMAL_TRANSITION
4:114899005	*CAMK2D*	REACTOME_THE_ACTIVATION_OF_ARYLSULFATASES
1:161171736	*FCGR2A*	REACTOME_THE_CITRIC_ACID_TCA_CYCLE_AND_RESPIRATORY_ELECTRON_TRANSPORT
5:60273923	*ELOVL7*	REACTOME_THE_CITRIC_ACID_TCA_CYCLE_AND_RESPIRATORY_ELECTRON_TRANSPORT
5:60288288	*ELOVL7*	REACTOME_THE_CITRIC_ACID_TCA_CYCLE_AND_RESPIRATORY_ELECTRON_TRANSPORT
10:15199347	*ITGA8*	REACTOME_THE_PHOTOTRANSDUCTION_CASCADE
17:43181287	*CRHR1_2*	REACTOME_THE_PHOTOTRANSDUCTION_CASCADE
17:43184627	*CRHR1_2*	REACTOME_THE_PHOTOTRANSDUCTION_CASCADE
17:7578671	*V1NB1*	REACTOME_THE_ROLE_OF_GTSE1_IN_G2_M_PROGRESSION_AFTER_G2_CHECKPOINT
6:112137847	*FYN*	REACTOME_THE_ROLE_OF_NEF_IN_HIV_1_REPLICATION_AND_DISEASE_PATHOGENESIS
6:112151452	*FYN*	REACTOME_THE_ROLE_OF_NEF_IN_HIV_1_REPLICATION_AND_DISEASE_PATHOGENESIS
6:112175069	*FYN*	REACTOME_THE_ROLE_OF_NEF_IN_HIV_1_REPLICATION_AND_DISEASE_PATHOGENESIS
19:2557113	*SPPL2B*	REACTOME_THROMBIN_SIGNALLING_THROUGH_PROTEINASE_ACTIVATED_RECEPTORS_PARS
19:2563327	*SPPL2B*	REACTOME_THROMBIN_SIGNALLING_THROUGH_PROTEINASE_ACTIVATED_RECEPTORS_PARS
19:2580057	*SPPL2B*	REACTOME_THROMBIN_SIGNALLING_THROUGH_PROTEINASE_ACTIVATED_RECEPTORS_PARS

19:2660983	*SPPL2B*	REACTOME_THROMBIN_SIGNALLING_THROUGH_PROTEINASE_ACTIVATED_RECEPTORS_PARS
19:3142784	*SPPL2B*	REACTOME_THROMBIN_SIGNALLING_THROUGH_PROTEINASE_ACTIVATED_RECEPTORS_PARS
19:3147326	*SPPL2B*	REACTOME_THROMBIN_SIGNALLING_THROUGH_PROTEINASE_ACTIVATED_RECEPTORS_PARS
19:2557113	*SPPL2B*	REACTOME_THROMBOXANE_SIGNALLING_THROUGH_TP_RECEPTOR
19:2563327	*SPPL2B*	REACTOME_THROMBOXANE_SIGNALLING_THROUGH_TP_RECEPTOR
19:2580057	*SPPL2B*	REACTOME_THROMBOXANE_SIGNALLING_THROUGH_TP_RECEPTOR
19:2660983	*SPPL2B*	REACTOME_THROMBOXANE_SIGNALLING_THROUGH_TP_RECEPTOR
19:3142784	*SPPL2B*	REACTOME_THROMBOXANE_SIGNALLING_THROUGH_TP_RECEPTOR
19:3147326	*SPPL2B*	REACTOME_THROMBOXANE_SIGNALLING_THROUGH_TP_RECEPTOR
21:37854286	*DYRK1A*	REACTOME_TIGHT_JUNCTION_INTERACTIONS
21:37859449	*DYRK1A*	REACTOME_TIGHT_JUNCTION_INTERACTIONS
21:37860785	*DYRK1A*	REACTOME_TIGHT_JUNCTION_INTERACTIONS
21:37894089	*DYRK1A*	REACTOME_TIGHT_JUNCTION_INTERACTIONS
21:37899076	*DYRK1A*	REACTOME_TIGHT_JUNCTION_INTERACTIONS
16:50790891	*CASC16*	REACTOME_TNFR1_INDUCED_NFKAPPAB_SIGNALING_PATHWAY
16:50790891	*CASC16*	REACTOME_TNFR1_INDUCED_PROAPOPTOTIC_SIGNALING
10:103185542	*GBF1*	REACTOME_TNFR2_NON_CANONICAL_NF_KB_PATHWAY
10:103305087	*GBF1*	REACTOME_TNFR2_NON_CANONICAL_NF_KB_PATHWAY
10:103308244	*GBF1*	REACTOME_TNFR2_NON_CANONICAL_NF_KB_PATHWAY
17:7455536	*V1NB1*	REACTOME_TNFR2_NON_CANONICAL_NF_KB_PATHWAY
17:43390763	*CRHR1_2*	REACTOME_TNFR2_NON_CANONICAL_NF_KB_PATHWAY
17:7455536	*V1NB1*	REACTOME_TNF_RECEPTOR_SUPERFAMILY_TNFSF_MEMBERS_MEDIATING_NON_CANONICAL_NF_KB_PATHWAY
17:43390763	*CRHR1_2*	REACTOME_TNF_RECEPTOR_SUPERFAMILY_TNFSF_MEMBERS_MEDIATING_NON_CANONICAL_NF_KB_PATHWAY
16:50790891	*CASC16*	REACTOME_TNF_SIGNALING
19:2335872	*SPPL2B*	REACTOME_TNF_SIGNALING
19:2346990	*SPPL2B*	REACTOME_TNF_SIGNALING
19:2352757	*SPPL2B*	REACTOME_TNF_SIGNALING
1:171837985	*VAMP4*	REACTOME_TOLL_LIKE_RECEPTOR_4_TLR4_CASCADE
1:171945488	*VAMP4*	REACTOME_TOLL_LIKE_RECEPTOR_4_TLR4_CASCADE
1:172137119	*VAMP4*	REACTOME_TOLL_LIKE_RECEPTOR_4_TLR4_CASCADE
1:172386261	*VAMP4*	REACTOME_TOLL_LIKE_RECEPTOR_4_TLR4_CASCADE
5:133558885	*C5orf24*	REACTOME_TOLL_LIKE_RECEPTOR_4_TLR4_CASCADE
6:32151443	*HLA-DRB5*	REACTOME_TOLL_LIKE_RECEPTOR_4_TLR4_CASCADE
10:103185542	*GBF1*	REACTOME_TOLL_LIKE_RECEPTOR_4_TLR4_CASCADE
10:103305087	*GBF1*	REACTOME_TOLL_LIKE_RECEPTOR_4_TLR4_CASCADE
10:103308244	*GBF1*	REACTOME_TOLL_LIKE_RECEPTOR_4_TLR4_CASCADE
16:50730088	*CASC16*	REACTOME_TOLL_LIKE_RECEPTOR_4_TLR4_CASCADE
16:50730446	*CASC16*	REACTOME_TOLL_LIKE_RECEPTOR_4_TLR4_CASCADE
16:50745926	*CASC16*	REACTOME_TOLL_LIKE_RECEPTOR_4_TLR4_CASCADE
17:41845031	*FAM171A2*	REACTOME_TOLL_LIKE_RECEPTOR_4_TLR4_CASCADE
5:133558885	*C5orf24*	REACTOME_TOLL_LIKE_RECEPTOR_9_TLR9_CASCADE
6:32151443	*HLA-DRB5*	REACTOME_TOLL_LIKE_RECEPTOR_9_TLR9_CASCADE
10:103185542	*GBF1*	REACTOME_TOLL_LIKE_RECEPTOR_9_TLR9_CASCADE
10:103305087	*GBF1*	REACTOME_TOLL_LIKE_RECEPTOR_9_TLR9_CASCADE
10:103308244	*GBF1*	REACTOME_TOLL_LIKE_RECEPTOR_9_TLR9_CASCADE
16:50730088	*CASC16*	REACTOME_TOLL_LIKE_RECEPTOR_9_TLR9_CASCADE
16:50730446	*CASC16*	REACTOME_TOLL_LIKE_RECEPTOR_9_TLR9_CASCADE
16:50745926	*CASC16*	REACTOME_TOLL_LIKE_RECEPTOR_9_TLR9_CASCADE
17:41845031	*FAM171A2*	REACTOME_TOLL_LIKE_RECEPTOR_9_TLR9_CASCADE
1:171837985	*VAMP4*	REACTOME_TOLL_LIKE_RECEPTOR_CASCADES
1:171945488	*VAMP4*	REACTOME_TOLL_LIKE_RECEPTOR_CASCADES
1:172137119	*VAMP4*	REACTOME_TOLL_LIKE_RECEPTOR_CASCADES
1:172386261	*VAMP4*	REACTOME_TOLL_LIKE_RECEPTOR_CASCADES

5:133558885	*C5orf24*	REACTOME_TOLL_LIKE_RECEPTOR_CASCADES
6:32151443	*HLA-DRB5*	REACTOME_TOLL_LIKE_RECEPTOR_CASCADES
8:11712443	*CTSB*	REACTOME_TOLL_LIKE_RECEPTOR_CASCADES
10:103185542	*GBF1*	REACTOME_TOLL_LIKE_RECEPTOR_CASCADES
10:103305087	*GBF1*	REACTOME_TOLL_LIKE_RECEPTOR_CASCADES
10:103308244	*GBF1*	REACTOME_TOLL_LIKE_RECEPTOR_CASCADES
16:50730088	*CASC16*	REACTOME_TOLL_LIKE_RECEPTOR_CASCADES
16:50730446	*CASC16*	REACTOME_TOLL_LIKE_RECEPTOR_CASCADES
16:50745926	*CASC16*	REACTOME_TOLL_LIKE_RECEPTOR_CASCADES
17:41845031	*FAM171A2*	REACTOME_TOLL_LIKE_RECEPTOR_CASCADES
5:133558885	*C5orf24*	REACTOME_TOLL_LIKE_RECEPTOR_TLR1:TLR2_CASCADE
6:32151443	*HLA-DRB5*	REACTOME_TOLL_LIKE_RECEPTOR_TLR1:TLR2_CASCADE
10:103185542	*GBF1*	REACTOME_TOLL_LIKE_RECEPTOR_TLR1:TLR2_CASCADE
10:103305087	*GBF1*	REACTOME_TOLL_LIKE_RECEPTOR_TLR1:TLR2_CASCADE
10:103308244	*GBF1*	REACTOME_TOLL_LIKE_RECEPTOR_TLR1:TLR2_CASCADE
16:50730088	*CASC16*	REACTOME_TOLL_LIKE_RECEPTOR_TLR1:TLR2_CASCADE
16:50730446	*CASC16*	REACTOME_TOLL_LIKE_RECEPTOR_TLR1:TLR2_CASCADE
16:50745926	*CASC16*	REACTOME_TOLL_LIKE_RECEPTOR_TLR1:TLR2_CASCADE
17:41845031	*FAM171A2*	REACTOME_TOLL_LIKE_RECEPTOR_TLR1:TLR2_CASCADE
17:7578671	*V1NB1*	REACTOME_TP53_REGULATES_METABOLIC_GENES
17:7578671	*V1NB1*	P53_REGULATES_TRANSCRIPTION_OF_ADDITIONAL_CELL_CYCLE_GENES_WHOSE_EXACT_ROLE_IN_THE_P53_PATHWAY_REM
17:7578671	*V1NB1*	REACTOME_TP53_REGULATES_TRANSCRIPTION_OF_CASPASE_ACTIVATORS_AND_CASPASES
17:7578671	*V1NB1*	REACTOME_TP53_REGULATES_TRANSCRIPTION_OF_CELL_CYCLE_GENES
8:22974278	*BIN3*	REACTOME_TP53_REGULATES_TRANSCRIPTION_OF_CELL_DEATH_GENES
8:23050400	*BIN3*	REACTOME_TP53_REGULATES_TRANSCRIPTION_OF_CELL_DEATH_GENES
17:7578671	*V1NB1*	REACTOME_TP53_REGULATES_TRANSCRIPTION_OF_CELL_DEATH_GENES
8:22974278	*BIN3*	REACTOME_TP53_REGULATES_TRANSCRIPTION_OF_DEATH_RECEPTORS_AND_LIGANDS
8:23050400	*BIN3*	REACTOME_TP53_REGULATES_TRANSCRIPTION_OF_DEATH_RECEPTORS_AND_LIGANDS
17:7578671	*V1NB1*	REACTOME_TP53_REGULATES_TRANSCRIPTION_OF_DEATH_RECEPTORS_AND_LIGANDS
2:135708115	*TMEM163*	REACTOME_TP53_REGULATES_TRANSCRIPTION_OF_DNA_REPAIR_GENES
6:31925679	*HLA-DRB5*	REACTOME_TP53_REGULATES_TRANSCRIPTION_OF_DNA_REPAIR_GENES
17:7578671	*V1NB1*	REACTOME_TP53_REGULATES_TRANSCRIPTION_OF_DNA_REPAIR_GENES
17:7578671	*V1NB1*	REACTOME_TP53_REGULATES_TRANSCRIPTION_OF_GENES_INVOLVED_IN_CYTOCHROME_C_RELEASE
17:7578671	*V1NB1*	REACTOME_TP53_REGULATES_TRANSCRIPTION_OF_GENES_INVOLVED_IN_G1_CELL_CYCLE_ARREST
17:7578671	*V1NB1*	REACTOME_TP53_REGULATES_TRANSCRIPTION_OF_GENES_INVOLVED_IN_G2_CELL_CYCLE_ARREST
17:7578671	*V1NB1*	ATES_TRANSCRIPTION_OF_SEVERAL_ADDITIONAL_CELL_DEATH_GENES_WHOSE_SPECIFIC_ROLES_IN_P53_DEPENDENT_APOP
6:32151443	*HLA-DRB5*	REACTOME_TRAF6_MEDIATED_NF_KB_ACTIVATION
8:11712443	*CTSB*	REACTOME_TRAFFICKING_AND_PROCESSING_OF_ENDOSOMAL_TLR
17:44800946	*WNT3*	REACTOME_TRAFFICKING_OF_GLUR2_CONTAINING_AMPA_RECEPTORS
17:44833217	*WNT3*	REACTOME_TRAFFICKING_OF_GLUR2_CONTAINING_AMPA_RECEPTORS
10:104443875	*GBF1*	REACTOME_TRAFFICKING_OF_MYRISTOYLATED_PROTEINS_TO_THE_CILIUM
10:104452818	*GBF1*	REACTOME_TRAFFICKING_OF_MYRISTOYLATED_PROTEINS_TO_THE_CILIUM
8:23050400	*BIN3*	REACTOME_TRAIL_SIGNALING
1:226568665	*ITPKB*	REACTOME_TRANSCRIPTIONAL_ACTIVITY_OF_SMAD2_SMAD3:SMAD4_HETEROTRIMER
2:135708115	*TMEM163*	REACTOME_TRANSCRIPTIONAL_ACTIVITY_OF_SMAD2_SMAD3:SMAD4_HETEROTRIMER
6:31862876	*HLA-DRB5*	REACTOME_TRANSCRIPTIONAL_REGULATION_BY_E2F6
4:114424939	*CAMK2D*	REACTOME_TRANSCRIPTIONAL_REGULATION_BY_MECP2
4:114494052	*CAMK2D*	REACTOME_TRANSCRIPTIONAL_REGULATION_BY_MECP2
4:114529492	*CAMK2D*	REACTOME_TRANSCRIPTIONAL_REGULATION_BY_MECP2
4:114539964	*CAMK2D*	REACTOME_TRANSCRIPTIONAL_REGULATION_BY_MECP2
4:114597918	*CAMK2D*	REACTOME_TRANSCRIPTIONAL_REGULATION_BY_MECP2
8:11353586	*CTSB*	REACTOME_TRANSCRIPTIONAL_REGULATION_BY_RUNX1
8:11363667	*CTSB*	REACTOME_TRANSCRIPTIONAL_REGULATION_BY_RUNX1

8:11381148	*CTSB*	REACTOME_TRANSCRIPTIONAL_REGULATION_BY_RUNX1
12:46134812	*SCAF11*	REACTOME_TRANSCRIPTIONAL_REGULATION_BY_RUNX1
12:46206268	*SCAF11*	REACTOME_TRANSCRIPTIONAL_REGULATION_BY_RUNX1
14:55515304	*GCH1*	REACTOME_TRANSCRIPTIONAL_REGULATION_BY_RUNX1
17:42454243	*FAM171A2*	REACTOME_TRANSCRIPTIONAL_REGULATION_BY_RUNX1
19:1611856	*SPPL2B*	REACTOME_TRANSCRIPTIONAL_REGULATION_BY_RUNX1
6:32941506	*HLA-DRB5*	REACTOME_TRANSCRIPTIONAL_REGULATION_BY_RUNX3
16:30510571	*SETD1A*	REACTOME_TRANSCRIPTIONAL_REGULATION_BY_RUNX3
16:30516492	*SETD1A*	REACTOME_TRANSCRIPTIONAL_REGULATION_BY_RUNX3
16:30520856	*SETD1A*	REACTOME_TRANSCRIPTIONAL_REGULATION_BY_RUNX3
17:7578671	*V1NB1*	REACTOME_TRANSCRIPTIONAL_REGULATION_BY_RUNX3
2:135708115	*TMEM163*	REACTOME_TRANSCRIPTIONAL_REGULATION_BY_TP53
5:133558885	*C5orf24*	REACTOME_TRANSCRIPTIONAL_REGULATION_BY_TP53
6:31862876	*HLA-DRB5*	REACTOME_TRANSCRIPTIONAL_REGULATION_BY_TP53
6:31925679	*HLA-DRB5*	REACTOME_TRANSCRIPTIONAL_REGULATION_BY_TP53
6:33287183	*HLA-DRB5*	REACTOME_TRANSCRIPTIONAL_REGULATION_BY_TP53
8:22974278	*BIN3*	REACTOME_TRANSCRIPTIONAL_REGULATION_BY_TP53
8:23050400	*BIN3*	REACTOME_TRANSCRIPTIONAL_REGULATION_BY_TP53
17:7578671	*V1NB1*	REACTOME_TRANSCRIPTIONAL_REGULATION_BY_TP53
17:59791813	*BRIP1*	REACTOME_TRANSCRIPTIONAL_REGULATION_BY_TP53
17:59922925	*BRIP1*	REACTOME_TRANSCRIPTIONAL_REGULATION_BY_TP53
4:17622910	*LCORL*	REACTOME_TRANSCRIPTIONAL_REGULATION_OF_WHITE_ADIPOCYTE_DIFFERENTIATION
17:60124582	*BRIP1*	REACTOME_TRANSCRIPTIONAL_REGULATION_OF_WHITE_ADIPOCYTE_DIFFERENTIATION
12:133206648	*FBRSL1*	REACTOME_TRANSCRIPTION_COUPLED_NUCLEOTIDE_EXCISION_REPAIR_TC_NER
12:133223685	*FBRSL1*	REACTOME_TRANSCRIPTION_COUPLED_NUCLEOTIDE_EXCISION_REPAIR_TC_NER
1:226497419	*ITPKB*	REACTOME_TRANSCRIPTION_OF_E2F_TARGETS_UNDER_NEGATIVE_CONTROL_BY_DREAM_COMPLEX
3:48199695	*IP6K2*	REACTOME_TRANSCRIPTION_OF_E2F_TARGETS_UNDER_NEGATIVE_CONTROL_BY_DREAM_COMPLEX
3:48214438	*IP6K2*	REACTOME_TRANSCRIPTION_OF_E2F_TARGETS_UNDER_NEGATIVE_CONTROL_BY_DREAM_COMPLEX
14:74640180	*RPS6KL1*	REACTOME_TRANSCRIPTION_OF_E2F_TARGETS_UNDER_NEGATIVE_CONTROL_BY_DREAM_COMPLEX
1:226497419	*ITPKB*	OME_TRANSCRIPTION_OF_E2F_TARGETS_UNDER_NEGATIVE_CONTROL_BY_P107_RBL1_AND_P130_RBL2_IN_COMPLEX_WITH_
14:74640180	*RPS6KL1*	OME_TRANSCRIPTION_OF_E2F_TARGETS_UNDER_NEGATIVE_CONTROL_BY_P107_RBL1_AND_P130_RBL2_IN_COMPLEX_WITH_
2:135708115	*TMEM163*	REACTOME_TRANSCRIPTION_OF_THE_HIV_GENOME
6:31925679	*HLA-DRB5*	REACTOME_TRANSCRIPTION_OF_THE_HIV_GENOME
12:124219791	*HIP1R*	REACTOME_TRANSFERRIN_ENDOCYTOSIS_AND_RECYCLING
1:226351254	*ITPKB*	REACTOME_TRANS_GOLGI_NETWORK_VESICLE_BUDDING
1:226372766	*ITPKB*	REACTOME_TRANS_GOLGI_NETWORK_VESICLE_BUDDING
3:121411876	*KPNA1*	REACTOME_TRANS_GOLGI_NETWORK_VESICLE_BUDDING
3:121412702	*KPNA1*	REACTOME_TRANS_GOLGI_NETWORK_VESICLE_BUDDING
4:925376	*GAK*	REACTOME_TRANS_GOLGI_NETWORK_VESICLE_BUDDING
9:17579690	*SH3GL2_2*	REACTOME_TRANS_GOLGI_NETWORK_VESICLE_BUDDING
9:17587160	*SH3GL2_2*	REACTOME_TRANS_GOLGI_NETWORK_VESICLE_BUDDING
9:17609302	*SH3GL2_2*	REACTOME_TRANS_GOLGI_NETWORK_VESICLE_BUDDING
9:17626762	*SH3GL2_2*	REACTOME_TRANS_GOLGI_NETWORK_VESICLE_BUDDING
9:17627444	*SH3GL2_2*	REACTOME_TRANS_GOLGI_NETWORK_VESICLE_BUDDING
9:17642664	*SH3GL2_2*	REACTOME_TRANS_GOLGI_NETWORK_VESICLE_BUDDING
9:17710876	*SH3GL2_2*	REACTOME_TRANS_GOLGI_NETWORK_VESICLE_BUDDING
9:17732590	*SH3GL2_2*	REACTOME_TRANS_GOLGI_NETWORK_VESICLE_BUDDING
9:17737514	*SH3GL2_2*	REACTOME_TRANS_GOLGI_NETWORK_VESICLE_BUDDING
9:17758945	*SH3GL2_2*	REACTOME_TRANS_GOLGI_NETWORK_VESICLE_BUDDING
10:104005410	*GBF1*	REACTOME_TRANS_GOLGI_NETWORK_VESICLE_BUDDING
10:104034550	*GBF1*	REACTOME_TRANS_GOLGI_NETWORK_VESICLE_BUDDING
12:123326598	*HIP1R*	REACTOME_TRANS_GOLGI_NETWORK_VESICLE_BUDDING
1:155698425	*GBAP1*	REACTOME_TRANSLATION

6:31758240	*HLA-DRB5*	REACTOME_TRANSLATION
6:31762311	*HLA-DRB5*	REACTOME_TRANSLATION
17:7479140	*V1NB1*	REACTOME_TRANSLATION
17:7480325	*V1NB1*	REACTOME_TRANSLATION
4:76407998	*FAM47E-STBD1*	REACTOME_TRANSLESION_SYNTHESIS_BY_POLH
6:111628626	*FYN*	REACTOME_TRANSLESION_SYNTHESIS_BY_POLK
4:76407998	*FAM47E-STBD1*	REACTOME_TRANSLESION_SYNTHESIS_BY_Y_FAMILY_DNA_POLYMERASES_BYPASSES_LESIONS_ON_DNA_TEMPLATE
6:111628626	*FYN*	REACTOME_TRANSLESION_SYNTHESIS_BY_Y_FAMILY_DNA_POLYMERASES_BYPASSES_LESIONS_ON_DNA_TEMPLATE
12:133206648	*FBRSL1*	REACTOME_TRANSLESION_SYNTHESIS_BY_Y_FAMILY_DNA_POLYMERASES_BYPASSES_LESIONS_ON_DNA_TEMPLATE
12:133223685	*FBRSL1*	REACTOME_TRANSLESION_SYNTHESIS_BY_Y_FAMILY_DNA_POLYMERASES_BYPASSES_LESIONS_ON_DNA_TEMPLATE
6:32602372	*HLA-DRB5*	REACTOME_TRANSLOCATION_OF_ZAP_70_TO_IMMUNOLOGICAL_SYNAPSE
3:48835245	*IP6K2*	REACTOME_TRANSMISSION_ACROSS_CHEMICAL_SYNAPSES
3:123089448	*KPNA1*	REACTOME_TRANSMISSION_ACROSS_CHEMICAL_SYNAPSES
4:114424939	*CAMK2D*	REACTOME_TRANSMISSION_ACROSS_CHEMICAL_SYNAPSES
4:114494052	*CAMK2D*	REACTOME_TRANSMISSION_ACROSS_CHEMICAL_SYNAPSES
4:114529492	*CAMK2D*	REACTOME_TRANSMISSION_ACROSS_CHEMICAL_SYNAPSES
4:114539964	*CAMK2D*	REACTOME_TRANSMISSION_ACROSS_CHEMICAL_SYNAPSES
4:114597918	*CAMK2D*	REACTOME_TRANSMISSION_ACROSS_CHEMICAL_SYNAPSES
6:29565889	*TRIM40*	REACTOME_TRANSMISSION_ACROSS_CHEMICAL_SYNAPSES
6:29590715	*TRIM40*	REACTOME_TRANSMISSION_ACROSS_CHEMICAL_SYNAPSES
6:72646790	*RIMS1*	REACTOME_TRANSMISSION_ACROSS_CHEMICAL_SYNAPSES
6:72648340	*RIMS1*	REACTOME_TRANSMISSION_ACROSS_CHEMICAL_SYNAPSES
6:72686444	*RIMS1*	REACTOME_TRANSMISSION_ACROSS_CHEMICAL_SYNAPSES
6:72716358	*RIMS1*	REACTOME_TRANSMISSION_ACROSS_CHEMICAL_SYNAPSES
6:72904599	*RIMS1*	REACTOME_TRANSMISSION_ACROSS_CHEMICAL_SYNAPSES
6:72913435	*RIMS1*	REACTOME_TRANSMISSION_ACROSS_CHEMICAL_SYNAPSES
6:72930929	*RIMS1*	REACTOME_TRANSMISSION_ACROSS_CHEMICAL_SYNAPSES
6:72982289	*RIMS1*	REACTOME_TRANSMISSION_ACROSS_CHEMICAL_SYNAPSES
6:72986711	*RIMS1*	REACTOME_TRANSMISSION_ACROSS_CHEMICAL_SYNAPSES
6:73008061	*RIMS1*	REACTOME_TRANSMISSION_ACROSS_CHEMICAL_SYNAPSES
6:73060492	*RIMS1*	REACTOME_TRANSMISSION_ACROSS_CHEMICAL_SYNAPSES
8:131799767	*FAM49B*	REACTOME_TRANSMISSION_ACROSS_CHEMICAL_SYNAPSES
8:131845512	*FAM49B*	REACTOME_TRANSMISSION_ACROSS_CHEMICAL_SYNAPSES
8:131865425	*FAM49B*	REACTOME_TRANSMISSION_ACROSS_CHEMICAL_SYNAPSES
8:131901170	*FAM49B*	REACTOME_TRANSMISSION_ACROSS_CHEMICAL_SYNAPSES
11:83219006	*DLG2*	REACTOME_TRANSMISSION_ACROSS_CHEMICAL_SYNAPSES
11:83232915	*DLG2*	REACTOME_TRANSMISSION_ACROSS_CHEMICAL_SYNAPSES
11:83282740	*DLG2*	REACTOME_TRANSMISSION_ACROSS_CHEMICAL_SYNAPSES
11:83387273	*DLG2*	REACTOME_TRANSMISSION_ACROSS_CHEMICAL_SYNAPSES
11:83538733	*DLG2*	REACTOME_TRANSMISSION_ACROSS_CHEMICAL_SYNAPSES
11:83544472	*DLG2*	REACTOME_TRANSMISSION_ACROSS_CHEMICAL_SYNAPSES
11:83578152	*DLG2*	REACTOME_TRANSMISSION_ACROSS_CHEMICAL_SYNAPSES
11:83647657	*DLG2*	REACTOME_TRANSMISSION_ACROSS_CHEMICAL_SYNAPSES
11:83669903	*DLG2*	REACTOME_TRANSMISSION_ACROSS_CHEMICAL_SYNAPSES
11:83736246	*DLG2*	REACTOME_TRANSMISSION_ACROSS_CHEMICAL_SYNAPSES
11:83762918	*DLG2*	REACTOME_TRANSMISSION_ACROSS_CHEMICAL_SYNAPSES
11:83823752	*DLG2*	REACTOME_TRANSMISSION_ACROSS_CHEMICAL_SYNAPSES
11:83839992	*DLG2*	REACTOME_TRANSMISSION_ACROSS_CHEMICAL_SYNAPSES
11:84104763	*DLG2*	REACTOME_TRANSMISSION_ACROSS_CHEMICAL_SYNAPSES
11:84169039	*DLG2*	REACTOME_TRANSMISSION_ACROSS_CHEMICAL_SYNAPSES
11:84221628	*DLG2*	REACTOME_TRANSMISSION_ACROSS_CHEMICAL_SYNAPSES
11:84312008	*DLG2*	REACTOME_TRANSMISSION_ACROSS_CHEMICAL_SYNAPSES
11:84324361	*DLG2*	REACTOME_TRANSMISSION_ACROSS_CHEMICAL_SYNAPSES

11:84360346	*DLG2*	REACTOME_TRANSMISSION_ACROSS_CHEMICAL_SYNAPSES
11:84403409	*DLG2*	REACTOME_TRANSMISSION_ACROSS_CHEMICAL_SYNAPSES
12:46618863	*SCAF11*	REACTOME_TRANSMISSION_ACROSS_CHEMICAL_SYNAPSES
16:50300511	*CASC16*	REACTOME_TRANSMISSION_ACROSS_CHEMICAL_SYNAPSES
16:50317039	*CASC16*	REACTOME_TRANSMISSION_ACROSS_CHEMICAL_SYNAPSES
17:7101771	*V1NB1*	REACTOME_TRANSMISSION_ACROSS_CHEMICAL_SYNAPSES
17:43999203	*WNT3*	REACTOME_TRANSMISSION_ACROSS_CHEMICAL_SYNAPSES
17:44005297	*WNT3*	REACTOME_TRANSMISSION_ACROSS_CHEMICAL_SYNAPSES
17:44019712	*WNT3*	REACTOME_TRANSMISSION_ACROSS_CHEMICAL_SYNAPSES
17:44060492	*WNT3*	REACTOME_TRANSMISSION_ACROSS_CHEMICAL_SYNAPSES
17:44065263	*WNT3*	REACTOME_TRANSMISSION_ACROSS_CHEMICAL_SYNAPSES
17:44081178	*WNT3*	REACTOME_TRANSMISSION_ACROSS_CHEMICAL_SYNAPSES
17:44083297	*WNT3*	REACTOME_TRANSMISSION_ACROSS_CHEMICAL_SYNAPSES
17:44800946	*WNT3*	REACTOME_TRANSMISSION_ACROSS_CHEMICAL_SYNAPSES
17:44833217	*WNT3*	REACTOME_TRANSMISSION_ACROSS_CHEMICAL_SYNAPSES
19:2557113	*SPPL2B*	REACTOME_TRANSMISSION_ACROSS_CHEMICAL_SYNAPSES
19:2563327	*SPPL2B*	REACTOME_TRANSMISSION_ACROSS_CHEMICAL_SYNAPSES
19:2580057	*SPPL2B*	REACTOME_TRANSMISSION_ACROSS_CHEMICAL_SYNAPSES
19:2660983	*SPPL2B*	REACTOME_TRANSMISSION_ACROSS_CHEMICAL_SYNAPSES
21:38984964	*DYRK1A*	REACTOME_TRANSMISSION_ACROSS_CHEMICAL_SYNAPSES
21:39018136	*DYRK1A*	REACTOME_TRANSMISSION_ACROSS_CHEMICAL_SYNAPSES
21:39021814	*DYRK1A*	REACTOME_TRANSMISSION_ACROSS_CHEMICAL_SYNAPSES
21:39039956	*DYRK1A*	REACTOME_TRANSMISSION_ACROSS_CHEMICAL_SYNAPSES
21:39088785	*DYRK1A*	REACTOME_TRANSMISSION_ACROSS_CHEMICAL_SYNAPSES
21:39108160	*DYRK1A*	REACTOME_TRANSMISSION_ACROSS_CHEMICAL_SYNAPSES
21:39120480	*DYRK1A*	REACTOME_TRANSMISSION_ACROSS_CHEMICAL_SYNAPSES
21:39134980	*DYRK1A*	REACTOME_TRANSMISSION_ACROSS_CHEMICAL_SYNAPSES
21:39197323	*DYRK1A*	REACTOME_TRANSMISSION_ACROSS_CHEMICAL_SYNAPSES
21:39217472	*DYRK1A*	REACTOME_TRANSMISSION_ACROSS_CHEMICAL_SYNAPSES
6:31846234	*HLA-DRB5*	REACTOME_TRANSPORT_OF_BILE_SALTS_AND_ORGANIC_ACIDS_METAL_IONS_AND_AMINE_COMPOUNDS
12:40240262	*LRRK2_2*	REACTOME_TRANSPORT_OF_BILE_SALTS_AND_ORGANIC_ACIDS_METAL_IONS_AND_AMINE_COMPOUNDS
12:40252360	*LRRK2_2*	REACTOME_TRANSPORT_OF_BILE_SALTS_AND_ORGANIC_ACIDS_METAL_IONS_AND_AMINE_COMPOUNDS
12:40267539	*LRRK2_2*	REACTOME_TRANSPORT_OF_BILE_SALTS_AND_ORGANIC_ACIDS_METAL_IONS_AND_AMINE_COMPOUNDS
12:40302599	*LRRK2_2*	REACTOME_TRANSPORT_OF_BILE_SALTS_AND_ORGANIC_ACIDS_METAL_IONS_AND_AMINE_COMPOUNDS
12:40348056	*LRRK2_2*	REACTOME_TRANSPORT_OF_BILE_SALTS_AND_ORGANIC_ACIDS_METAL_IONS_AND_AMINE_COMPOUNDS
12:40349236	*LRRK2_2*	REACTOME_TRANSPORT_OF_BILE_SALTS_AND_ORGANIC_ACIDS_METAL_IONS_AND_AMINE_COMPOUNDS
12:40378773	*LRRK2_2*	REACTOME_TRANSPORT_OF_BILE_SALTS_AND_ORGANIC_ACIDS_METAL_IONS_AND_AMINE_COMPOUNDS
12:40393852	*LRRK2_2*	REACTOME_TRANSPORT_OF_BILE_SALTS_AND_ORGANIC_ACIDS_METAL_IONS_AND_AMINE_COMPOUNDS
12:40405478	*LRRK2_2*	REACTOME_TRANSPORT_OF_BILE_SALTS_AND_ORGANIC_ACIDS_METAL_IONS_AND_AMINE_COMPOUNDS
12:40413305	*LRRK2_2*	REACTOME_TRANSPORT_OF_BILE_SALTS_AND_ORGANIC_ACIDS_METAL_IONS_AND_AMINE_COMPOUNDS
12:40414626	*LRRK2_2*	REACTOME_TRANSPORT_OF_BILE_SALTS_AND_ORGANIC_ACIDS_METAL_IONS_AND_AMINE_COMPOUNDS
12:40437969	*LRRK2_2*	REACTOME_TRANSPORT_OF_BILE_SALTS_AND_ORGANIC_ACIDS_METAL_IONS_AND_AMINE_COMPOUNDS
17:6594869	*V1NB1*	REACTOME_TRANSPORT_OF_BILE_SALTS_AND_ORGANIC_ACIDS_METAL_IONS_AND_AMINE_COMPOUNDS
17:6612244	*V1NB1*	REACTOME_TRANSPORT_OF_BILE_SALTS_AND_ORGANIC_ACIDS_METAL_IONS_AND_AMINE_COMPOUNDS
1:205884274	*RAB29*	REACTOME_TRANSPORT_OF_INORGANIC_CATIONS_ANIONS_AND_AMINO_ACIDS_OLIGOPEPTIDES
2:103267601	*MAP4K4*	REACTOME_TRANSPORT_OF_INORGANIC_CATIONS_ANIONS_AND_AMINO_ACIDS_OLIGOPEPTIDES
2:103282717	*MAP4K4*	REACTOME_TRANSPORT_OF_INORGANIC_CATIONS_ANIONS_AND_AMINO_ACIDS_OLIGOPEPTIDES
2:103317676	*MAP4K4*	REACTOME_TRANSPORT_OF_INORGANIC_CATIONS_ANIONS_AND_AMINO_ACIDS_OLIGOPEPTIDES
8:17390689	*FGF20*	REACTOME_TRANSPORT_OF_INORGANIC_CATIONS_ANIONS_AND_AMINO_ACIDS_OLIGOPEPTIDES
8:17402274	*FGF20*	REACTOME_TRANSPORT_OF_INORGANIC_CATIONS_ANIONS_AND_AMINO_ACIDS_OLIGOPEPTIDES
8:17427186	*FGF20*	REACTOME_TRANSPORT_OF_INORGANIC_CATIONS_ANIONS_AND_AMINO_ACIDS_OLIGOPEPTIDES
12:46618863	*SCAF11*	REACTOME_TRANSPORT_OF_INORGANIC_CATIONS_ANIONS_AND_AMINO_ACIDS_OLIGOPEPTIDES
12:47192323	*SCAF11*	REACTOME_TRANSPORT_OF_INORGANIC_CATIONS_ANIONS_AND_AMINO_ACIDS_OLIGOPEPTIDES

12:47222402	*SCAF11*	REACTOME_TRANSPORT_OF_INORGANIC_CATIONS_ANIONS_AND_AMINO_ACIDS_OLIGOPEPTIDES
17:42332803	*FAM171A2*	REACTOME_TRANSPORT_OF_INORGANIC_CATIONS_ANIONS_AND_AMINO_ACIDS_OLIGOPEPTIDES
17:42338993	*FAM171A2*	REACTOME_TRANSPORT_OF_INORGANIC_CATIONS_ANIONS_AND_AMINO_ACIDS_OLIGOPEPTIDES
5:101579003	*PAM*	REACTOME_TRANSPORT_OF_ORGANIC_ANIONS
1:154306180	*GBAP1*	REACTOME_TRANSPORT_OF_SMALL_MOLECULES
1:205884274	*RAB29*	REACTOME_TRANSPORT_OF_SMALL_MOLECULES
2:103267601	*MAP4K4*	REACTOME_TRANSPORT_OF_SMALL_MOLECULES
2:103282717	*MAP4K4*	REACTOME_TRANSPORT_OF_SMALL_MOLECULES
2:103317676	*MAP4K4*	REACTOME_TRANSPORT_OF_SMALL_MOLECULES
3:48835245	*IP6K2*	REACTOME_TRANSPORT_OF_SMALL_MOLECULES
3:123089448	*KPNA1*	REACTOME_TRANSPORT_OF_SMALL_MOLECULES
4:15608677	*BST1*	REACTOME_TRANSPORT_OF_SMALL_MOLECULES
4:15618898	*BST1*	REACTOME_TRANSPORT_OF_SMALL_MOLECULES
4:114424939	*CAMK2D*	REACTOME_TRANSPORT_OF_SMALL_MOLECULES
4:114494052	*CAMK2D*	REACTOME_TRANSPORT_OF_SMALL_MOLECULES
4:114529492	*CAMK2D*	REACTOME_TRANSPORT_OF_SMALL_MOLECULES
4:114539964	*CAMK2D*	REACTOME_TRANSPORT_OF_SMALL_MOLECULES
4:114597918	*CAMK2D*	REACTOME_TRANSPORT_OF_SMALL_MOLECULES
4:170563153	*CLCN3*	REACTOME_TRANSPORT_OF_SMALL_MOLECULES
4:170569172	*CLCN3*	REACTOME_TRANSPORT_OF_SMALL_MOLECULES
4:170620812	*CLCN3*	REACTOME_TRANSPORT_OF_SMALL_MOLECULES
5:101579003	*PAM*	REACTOME_TRANSPORT_OF_SMALL_MOLECULES
6:31846234	*HLA-DRB5*	REACTOME_TRANSPORT_OF_SMALL_MOLECULES
6:32151443	*HLA-DRB5*	REACTOME_TRANSPORT_OF_SMALL_MOLECULES
8:16929685	*FGF20*	REACTOME_TRANSPORT_OF_SMALL_MOLECULES
8:16967392	*FGF20*	REACTOME_TRANSPORT_OF_SMALL_MOLECULES
8:16971257	*FGF20*	REACTOME_TRANSPORT_OF_SMALL_MOLECULES
8:17390689	*FGF20*	REACTOME_TRANSPORT_OF_SMALL_MOLECULES
8:17402274	*FGF20*	REACTOME_TRANSPORT_OF_SMALL_MOLECULES
8:17427186	*FGF20*	REACTOME_TRANSPORT_OF_SMALL_MOLECULES
8:131799767	*FAM49B*	REACTOME_TRANSPORT_OF_SMALL_MOLECULES
8:131845512	*FAM49B*	REACTOME_TRANSPORT_OF_SMALL_MOLECULES
8:131865425	*FAM49B*	REACTOME_TRANSPORT_OF_SMALL_MOLECULES
8:131901170	*FAM49B*	REACTOME_TRANSPORT_OF_SMALL_MOLECULES
9:33447547	*UBAP2*	REACTOME_TRANSPORT_OF_SMALL_MOLECULES
12:39960237	*LRRK2_2*	REACTOME_TRANSPORT_OF_SMALL_MOLECULES
12:40240262	*LRRK2_2*	REACTOME_TRANSPORT_OF_SMALL_MOLECULES
12:40252360	*LRRK2_2*	REACTOME_TRANSPORT_OF_SMALL_MOLECULES
12:40267539	*LRRK2_2*	REACTOME_TRANSPORT_OF_SMALL_MOLECULES
12:40302599	*LRRK2_2*	REACTOME_TRANSPORT_OF_SMALL_MOLECULES
12:40348056	*LRRK2_2*	REACTOME_TRANSPORT_OF_SMALL_MOLECULES
12:40349236	*LRRK2_2*	REACTOME_TRANSPORT_OF_SMALL_MOLECULES
12:40378773	*LRRK2_2*	REACTOME_TRANSPORT_OF_SMALL_MOLECULES
12:40393852	*LRRK2_2*	REACTOME_TRANSPORT_OF_SMALL_MOLECULES
12:40405478	*LRRK2_2*	REACTOME_TRANSPORT_OF_SMALL_MOLECULES
12:40413305	*LRRK2_2*	REACTOME_TRANSPORT_OF_SMALL_MOLECULES
12:40414626	*LRRK2_2*	REACTOME_TRANSPORT_OF_SMALL_MOLECULES
12:40437969	*LRRK2_2*	REACTOME_TRANSPORT_OF_SMALL_MOLECULES
12:45755099	*SCAF11*	REACTOME_TRANSPORT_OF_SMALL_MOLECULES
12:46618863	*SCAF11*	REACTOME_TRANSPORT_OF_SMALL_MOLECULES
12:47192323	*SCAF11*	REACTOME_TRANSPORT_OF_SMALL_MOLECULES
12:47222402	*SCAF11*	REACTOME_TRANSPORT_OF_SMALL_MOLECULES
12:123453034	*HIP1R*	REACTOME_TRANSPORT_OF_SMALL_MOLECULES

12:124219791	*HIP1R*	REACTOME_TRANSPORT_OF_SMALL_MOLECULES
16:50300511	*CASC16*	REACTOME_TRANSPORT_OF_SMALL_MOLECULES
16:50317039	*CASC16*	REACTOME_TRANSPORT_OF_SMALL_MOLECULES
17:6594869	*V1NB1*	REACTOME_TRANSPORT_OF_SMALL_MOLECULES
17:6612244	*V1NB1*	REACTOME_TRANSPORT_OF_SMALL_MOLECULES
17:7560294	*V1NB1*	REACTOME_TRANSPORT_OF_SMALL_MOLECULES
17:40938261	*RETREG3*	REACTOME_TRANSPORT_OF_SMALL_MOLECULES
17:42332803	*FAM171A2*	REACTOME_TRANSPORT_OF_SMALL_MOLECULES
17:42338993	*FAM171A2*	REACTOME_TRANSPORT_OF_SMALL_MOLECULES
19:1811130	*SPPL2B*	REACTOME_TRANSPORT_OF_SMALL_MOLECULES
19:2557113	*SPPL2B*	REACTOME_TRANSPORT_OF_SMALL_MOLECULES
19:2563327	*SPPL2B*	REACTOME_TRANSPORT_OF_SMALL_MOLECULES
19:2580057	*SPPL2B*	REACTOME_TRANSPORT_OF_SMALL_MOLECULES
19:2660983	*SPPL2B*	REACTOME_TRANSPORT_OF_SMALL_MOLECULES
5:101579003	*PAM*	REACTOME_TRANSPORT_OF_VITAMINS_NUCLEOSIDES_AND_RELATED_MOLECULES
2:134902070	*TMEM163*	REACTOME_TRANSPORT_TO_THE_GOLGI_AND_SUBSEQUENT_MODIFICATION
2:134932210	*TMEM163*	REACTOME_TRANSPORT_TO_THE_GOLGI_AND_SUBSEQUENT_MODIFICATION
2:134956835	*TMEM163*	REACTOME_TRANSPORT_TO_THE_GOLGI_AND_SUBSEQUENT_MODIFICATION
2:134986578	*TMEM163*	REACTOME_TRANSPORT_TO_THE_GOLGI_AND_SUBSEQUENT_MODIFICATION
2:134993021	*TMEM163*	REACTOME_TRANSPORT_TO_THE_GOLGI_AND_SUBSEQUENT_MODIFICATION
2:135035038	*TMEM163*	REACTOME_TRANSPORT_TO_THE_GOLGI_AND_SUBSEQUENT_MODIFICATION
2:135040234	*TMEM163*	REACTOME_TRANSPORT_TO_THE_GOLGI_AND_SUBSEQUENT_MODIFICATION
2:135165078	*TMEM163*	REACTOME_TRANSPORT_TO_THE_GOLGI_AND_SUBSEQUENT_MODIFICATION
2:135166385	*TMEM163*	REACTOME_TRANSPORT_TO_THE_GOLGI_AND_SUBSEQUENT_MODIFICATION
3:121411876	*KPNA1*	REACTOME_TRANSPORT_TO_THE_GOLGI_AND_SUBSEQUENT_MODIFICATION
3:121412702	*KPNA1*	REACTOME_TRANSPORT_TO_THE_GOLGI_AND_SUBSEQUENT_MODIFICATION
3:122986429	*KPNA1*	REACTOME_TRANSPORT_TO_THE_GOLGI_AND_SUBSEQUENT_MODIFICATION
4:76695927	*FAM47E-STBD1*	REACTOME_TRANSPORT_TO_THE_GOLGI_AND_SUBSEQUENT_MODIFICATION
4:113947327	*CAMK2D*	REACTOME_TRANSPORT_TO_THE_GOLGI_AND_SUBSEQUENT_MODIFICATION
4:114015252	*CAMK2D*	REACTOME_TRANSPORT_TO_THE_GOLGI_AND_SUBSEQUENT_MODIFICATION
4:114032570	*CAMK2D*	REACTOME_TRANSPORT_TO_THE_GOLGI_AND_SUBSEQUENT_MODIFICATION
4:114067233	*CAMK2D*	REACTOME_TRANSPORT_TO_THE_GOLGI_AND_SUBSEQUENT_MODIFICATION
4:114077386	*CAMK2D*	REACTOME_TRANSPORT_TO_THE_GOLGI_AND_SUBSEQUENT_MODIFICATION
4:114113772	*CAMK2D*	REACTOME_TRANSPORT_TO_THE_GOLGI_AND_SUBSEQUENT_MODIFICATION
4:114122398	*CAMK2D*	REACTOME_TRANSPORT_TO_THE_GOLGI_AND_SUBSEQUENT_MODIFICATION
4:114172749	*CAMK2D*	REACTOME_TRANSPORT_TO_THE_GOLGI_AND_SUBSEQUENT_MODIFICATION
4:114217997	*CAMK2D*	REACTOME_TRANSPORT_TO_THE_GOLGI_AND_SUBSEQUENT_MODIFICATION
10:104005410	*GBF1*	REACTOME_TRANSPORT_TO_THE_GOLGI_AND_SUBSEQUENT_MODIFICATION
10:104034550	*GBF1*	REACTOME_TRANSPORT_TO_THE_GOLGI_AND_SUBSEQUENT_MODIFICATION
10:104258303	*GBF1*	REACTOME_TRANSPORT_TO_THE_GOLGI_AND_SUBSEQUENT_MODIFICATION
10:121669571	*INPP5F*	REACTOME_TRANSPORT_TO_THE_GOLGI_AND_SUBSEQUENT_MODIFICATION
10:121683206	*INPP5F*	REACTOME_TRANSPORT_TO_THE_GOLGI_AND_SUBSEQUENT_MODIFICATION
10:121686714	*INPP5F*	REACTOME_TRANSPORT_TO_THE_GOLGI_AND_SUBSEQUENT_MODIFICATION
10:121689030	*INPP5F*	REACTOME_TRANSPORT_TO_THE_GOLGI_AND_SUBSEQUENT_MODIFICATION
17:44800946	*WNT3*	REACTOME_TRANSPORT_TO_THE_GOLGI_AND_SUBSEQUENT_MODIFICATION
17:44833217	*WNT3*	REACTOME_TRANSPORT_TO_THE_GOLGI_AND_SUBSEQUENT_MODIFICATION
17:45020426	*WNT3*	REACTOME_TRANSPORT_TO_THE_GOLGI_AND_SUBSEQUENT_MODIFICATION
17:45059032	*WNT3*	REACTOME_TRANSPORT_TO_THE_GOLGI_AND_SUBSEQUENT_MODIFICATION
17:45089680	*WNT3*	REACTOME_TRANSPORT_TO_THE_GOLGI_AND_SUBSEQUENT_MODIFICATION
5:133558885	*C5orf24*	REACTOME_TRIF_TICAM1_MEDIATED_TLR4_SIGNALING
6:32151443	*HLA-DRB5*	REACTOME_TRIF_TICAM1_MEDIATED_TLR4_SIGNALING
10:103185542	*GBF1*	REACTOME_TRIF_TICAM1_MEDIATED_TLR4_SIGNALING
10:103305087	*GBF1*	REACTOME_TRIF_TICAM1_MEDIATED_TLR4_SIGNALING

10:103308244	*GBF1*	REACTOME_TRIF_TICAM1_MEDIATED_TLR4_SIGNALING
16:50730088	*CASC16*	REACTOME_TRIF_TICAM1_MEDIATED_TLR4_SIGNALING
16:50730446	*CASC16*	REACTOME_TRIF_TICAM1_MEDIATED_TLR4_SIGNALING
16:50745926	*CASC16*	REACTOME_TRIF_TICAM1_MEDIATED_TLR4_SIGNALING
17:41845031	*FAM171A2*	REACTOME_TRIF_TICAM1_MEDIATED_TLR4_SIGNALING
6:31758240	*HLA-DRB5*	REACTOME_TRNA_AMINOACYLATION
6:31762311	*HLA-DRB5*	REACTOME_TRNA_AMINOACYLATION
7:66487445	*GS1-124K5.11*	REACTOME_TRNA_MODIFICATION_IN_THE_NUCLEUS_AND_CYTOSOL
7:66543959	*GS1-124K5.11*	REACTOME_TRNA_MODIFICATION_IN_THE_NUCLEUS_AND_CYTOSOL
7:66638082	*GS1-124K5.11*	REACTOME_TRNA_MODIFICATION_IN_THE_NUCLEUS_AND_CYTOSOL
7:66487445	*GS1-124K5.11*	REACTOME_TRNA_PROCESSING
7:66543959	*GS1-124K5.11*	REACTOME_TRNA_PROCESSING
7:66638082	*GS1-124K5.11*	REACTOME_TRNA_PROCESSING
16:19085298	*SYT17*	REACTOME_UBIQUINOL_BIOSYNTHESIS
16:19090194	*SYT17*	REACTOME_UBIQUINOL_BIOSYNTHESIS
3:48199695	*IP6K2*	REACTOME_UB_SPECIFIC_PROCESSING_PROTEASES
3:48214438	*IP6K2*	REACTOME_UB_SPECIFIC_PROCESSING_PROTEASES
3:49740895	*IP6K2*	REACTOME_UB_SPECIFIC_PROCESSING_PROTEASES
16:19562008	*SYT17*	REACTOME_UB_SPECIFIC_PROCESSING_PROTEASES
16:50790891	*CASC16*	REACTOME_UB_SPECIFIC_PROCESSING_PROTEASES
17:7578671	*V1NB1*	REACTOME_UB_SPECIFIC_PROCESSING_PROTEASES
4:114424939	*CAMK2D*	REACTOME_UNBLOCKING_OF_NMDA_RECEPTORS_GLUTAMATE_BINDING_AND_ACTIVATION
4:114494052	*CAMK2D*	REACTOME_UNBLOCKING_OF_NMDA_RECEPTORS_GLUTAMATE_BINDING_AND_ACTIVATION
4:114529492	*CAMK2D*	REACTOME_UNBLOCKING_OF_NMDA_RECEPTORS_GLUTAMATE_BINDING_AND_ACTIVATION
4:114539964	*CAMK2D*	REACTOME_UNBLOCKING_OF_NMDA_RECEPTORS_GLUTAMATE_BINDING_AND_ACTIVATION
4:114597918	*CAMK2D*	REACTOME_UNBLOCKING_OF_NMDA_RECEPTORS_GLUTAMATE_BINDING_AND_ACTIVATION
11:83219006	*DLG2*	REACTOME_UNBLOCKING_OF_NMDA_RECEPTORS_GLUTAMATE_BINDING_AND_ACTIVATION
11:83232915	*DLG2*	REACTOME_UNBLOCKING_OF_NMDA_RECEPTORS_GLUTAMATE_BINDING_AND_ACTIVATION
11:83282740	*DLG2*	REACTOME_UNBLOCKING_OF_NMDA_RECEPTORS_GLUTAMATE_BINDING_AND_ACTIVATION
11:83387273	*DLG2*	REACTOME_UNBLOCKING_OF_NMDA_RECEPTORS_GLUTAMATE_BINDING_AND_ACTIVATION
11:83538733	*DLG2*	REACTOME_UNBLOCKING_OF_NMDA_RECEPTORS_GLUTAMATE_BINDING_AND_ACTIVATION
11:83544472	*DLG2*	REACTOME_UNBLOCKING_OF_NMDA_RECEPTORS_GLUTAMATE_BINDING_AND_ACTIVATION
11:83578152	*DLG2*	REACTOME_UNBLOCKING_OF_NMDA_RECEPTORS_GLUTAMATE_BINDING_AND_ACTIVATION
11:83647657	*DLG2*	REACTOME_UNBLOCKING_OF_NMDA_RECEPTORS_GLUTAMATE_BINDING_AND_ACTIVATION
11:83669903	*DLG2*	REACTOME_UNBLOCKING_OF_NMDA_RECEPTORS_GLUTAMATE_BINDING_AND_ACTIVATION
11:83736246	*DLG2*	REACTOME_UNBLOCKING_OF_NMDA_RECEPTORS_GLUTAMATE_BINDING_AND_ACTIVATION
11:83762918	*DLG2*	REACTOME_UNBLOCKING_OF_NMDA_RECEPTORS_GLUTAMATE_BINDING_AND_ACTIVATION
11:83823752	*DLG2*	REACTOME_UNBLOCKING_OF_NMDA_RECEPTORS_GLUTAMATE_BINDING_AND_ACTIVATION
11:83839992	*DLG2*	REACTOME_UNBLOCKING_OF_NMDA_RECEPTORS_GLUTAMATE_BINDING_AND_ACTIVATION
11:84104763	*DLG2*	REACTOME_UNBLOCKING_OF_NMDA_RECEPTORS_GLUTAMATE_BINDING_AND_ACTIVATION
11:84169039	*DLG2*	REACTOME_UNBLOCKING_OF_NMDA_RECEPTORS_GLUTAMATE_BINDING_AND_ACTIVATION
11:84221628	*DLG2*	REACTOME_UNBLOCKING_OF_NMDA_RECEPTORS_GLUTAMATE_BINDING_AND_ACTIVATION
11:84312008	*DLG2*	REACTOME_UNBLOCKING_OF_NMDA_RECEPTORS_GLUTAMATE_BINDING_AND_ACTIVATION
11:84324361	*DLG2*	REACTOME_UNBLOCKING_OF_NMDA_RECEPTORS_GLUTAMATE_BINDING_AND_ACTIVATION
11:84360346	*DLG2*	REACTOME_UNBLOCKING_OF_NMDA_RECEPTORS_GLUTAMATE_BINDING_AND_ACTIVATION
11:84403409	*DLG2*	REACTOME_UNBLOCKING_OF_NMDA_RECEPTORS_GLUTAMATE_BINDING_AND_ACTIVATION
17:7101771	*V1NB1*	REACTOME_UNBLOCKING_OF_NMDA_RECEPTORS_GLUTAMATE_BINDING_AND_ACTIVATION
1:156059276	*GBAP1*	REACTOME_UNFOLDED_PROTEIN_RESPONSE_UPR
1:161875645	*FCGR2A*	REACTOME_UNFOLDED_PROTEIN_RESPONSE_UPR
1:161930954	*FCGR2A*	REACTOME_UNFOLDED_PROTEIN_RESPONSE_UPR
3:122841099	*KPNA1*	REACTOME_UNFOLDED_PROTEIN_RESPONSE_UPR
3:122859868	*KPNA1*	REACTOME_UNFOLDED_PROTEIN_RESPONSE_UPR
3:122898729	*KPNA1*	REACTOME_UNFOLDED_PROTEIN_RESPONSE_UPR

3:150298317	*MED12L*	REACTOME_UNFOLDED_PROTEIN_RESPONSE_UPR
3:150303590	*MED12L*	REACTOME_UNFOLDED_PROTEIN_RESPONSE_UPR
17:45020426	*WNT3*	REACTOME_UNFOLDED_PROTEIN_RESPONSE_UPR
17:45059032	*WNT3*	REACTOME_UNFOLDED_PROTEIN_RESPONSE_UPR
17:45089680	*WNT3*	REACTOME_UNFOLDED_PROTEIN_RESPONSE_UPR
20:5934513	*CRLS1*	REACTOME_UNWINDING_OF_DNA
20:5935522	*CRLS1*	REACTOME_UNWINDING_OF_DNA
20:5968737	*CRLS1*	REACTOME_UNWINDING_OF_DNA
20:5968964	*CRLS1*	REACTOME_UNWINDING_OF_DNA
16:31004169	*SETD1A*	REACTOME_UPTAKE_AND_ACTIONS_OF_BACTERIAL_TOXINS
3:48835245	*IP6K2*	REACTOME_VASOPRESSIN_REGULATES_RENAL_WATER_HOMEOSTASIS_VIA_AQUAPORINS
3:123089448	*KPNA1*	REACTOME_VASOPRESSIN_REGULATES_RENAL_WATER_HOMEOSTASIS_VIA_AQUAPORINS
8:131799767	*FAM49B*	REACTOME_VASOPRESSIN_REGULATES_RENAL_WATER_HOMEOSTASIS_VIA_AQUAPORINS
8:131845512	*FAM49B*	REACTOME_VASOPRESSIN_REGULATES_RENAL_WATER_HOMEOSTASIS_VIA_AQUAPORINS
8:131865425	*FAM49B*	REACTOME_VASOPRESSIN_REGULATES_RENAL_WATER_HOMEOSTASIS_VIA_AQUAPORINS
8:131901170	*FAM49B*	REACTOME_VASOPRESSIN_REGULATES_RENAL_WATER_HOMEOSTASIS_VIA_AQUAPORINS
9:33447547	*UBAP2*	REACTOME_VASOPRESSIN_REGULATES_RENAL_WATER_HOMEOSTASIS_VIA_AQUAPORINS
16:50300511	*CASC16*	REACTOME_VASOPRESSIN_REGULATES_RENAL_WATER_HOMEOSTASIS_VIA_AQUAPORINS
16:50317039	*CASC16*	REACTOME_VASOPRESSIN_REGULATES_RENAL_WATER_HOMEOSTASIS_VIA_AQUAPORINS
19:2557113	*SPPL2B*	REACTOME_VASOPRESSIN_REGULATES_RENAL_WATER_HOMEOSTASIS_VIA_AQUAPORINS
19:2563327	*SPPL2B*	REACTOME_VASOPRESSIN_REGULATES_RENAL_WATER_HOMEOSTASIS_VIA_AQUAPORINS
19:2580057	*SPPL2B*	REACTOME_VASOPRESSIN_REGULATES_RENAL_WATER_HOMEOSTASIS_VIA_AQUAPORINS
19:2660983	*SPPL2B*	REACTOME_VASOPRESSIN_REGULATES_RENAL_WATER_HOMEOSTASIS_VIA_AQUAPORINS
17:39883520	*RETREG3*	REACTOME_VEGFR2_MEDIATED_VASCULAR_PERMEABILITY
1:155847427	*GBAP1*	REACTOME_VESICLE_MEDIATED_TRANSPORT
1:171689486	*VAMP4*	REACTOME_VESICLE_MEDIATED_TRANSPORT
1:171837985	*VAMP4*	REACTOME_VESICLE_MEDIATED_TRANSPORT
1:171945488	*VAMP4*	REACTOME_VESICLE_MEDIATED_TRANSPORT
1:172137119	*VAMP4*	REACTOME_VESICLE_MEDIATED_TRANSPORT
1:172386261	*VAMP4*	REACTOME_VESICLE_MEDIATED_TRANSPORT
1:226351254	*ITPKB*	REACTOME_VESICLE_MEDIATED_TRANSPORT
1:226372766	*ITPKB*	REACTOME_VESICLE_MEDIATED_TRANSPORT
2:135921205	*TMEM163*	REACTOME_VESICLE_MEDIATED_TRANSPORT
2:170012307	*STK39*	REACTOME_VESICLE_MEDIATED_TRANSPORT
2:170028024	*STK39*	REACTOME_VESICLE_MEDIATED_TRANSPORT
2:170066424	*STK39*	REACTOME_VESICLE_MEDIATED_TRANSPORT
3:121411876	*KPNA1*	REACTOME_VESICLE_MEDIATED_TRANSPORT
3:121412702	*KPNA1*	REACTOME_VESICLE_MEDIATED_TRANSPORT
3:122986429	*KPNA1*	REACTOME_VESICLE_MEDIATED_TRANSPORT
4:925376	*GAK*	REACTOME_VESICLE_MEDIATED_TRANSPORT
4:76695927	*FAM47E-STBD1*	REACTOME_VESICLE_MEDIATED_TRANSPORT
4:77104152	*FAM47E-STBD1*	REACTOME_VESICLE_MEDIATED_TRANSPORT
4:77124196	*FAM47E-STBD1*	REACTOME_VESICLE_MEDIATED_TRANSPORT
4:77134786	*FAM47E-STBD1*	REACTOME_VESICLE_MEDIATED_TRANSPORT
4:113947327	*CAMK2D*	REACTOME_VESICLE_MEDIATED_TRANSPORT
4:114015252	*CAMK2D*	REACTOME_VESICLE_MEDIATED_TRANSPORT
4:114032570	*CAMK2D*	REACTOME_VESICLE_MEDIATED_TRANSPORT
4:114067233	*CAMK2D*	REACTOME_VESICLE_MEDIATED_TRANSPORT
4:114077386	*CAMK2D*	REACTOME_VESICLE_MEDIATED_TRANSPORT
4:114113772	*CAMK2D*	REACTOME_VESICLE_MEDIATED_TRANSPORT
4:114122398	*CAMK2D*	REACTOME_VESICLE_MEDIATED_TRANSPORT
4:114172749	*CAMK2D*	REACTOME_VESICLE_MEDIATED_TRANSPORT
4:114217997	*CAMK2D*	REACTOME_VESICLE_MEDIATED_TRANSPORT

8:16086683	*FGF20*	REACTOME_VESICLE_MEDIATED_TRANSPORT
8:16135246	*FGF20*	REACTOME_VESICLE_MEDIATED_TRANSPORT
8:17125768	*FGF20*	REACTOME_VESICLE_MEDIATED_TRANSPORT
9:17579690	*SH3GL2_2*	REACTOME_VESICLE_MEDIATED_TRANSPORT
9:17587160	*SH3GL2_2*	REACTOME_VESICLE_MEDIATED_TRANSPORT
9:17609302	*SH3GL2_2*	REACTOME_VESICLE_MEDIATED_TRANSPORT
9:17626762	*SH3GL2_2*	REACTOME_VESICLE_MEDIATED_TRANSPORT
9:17627444	*SH3GL2_2*	REACTOME_VESICLE_MEDIATED_TRANSPORT
9:17642664	*SH3GL2_2*	REACTOME_VESICLE_MEDIATED_TRANSPORT
9:17710876	*SH3GL2_2*	REACTOME_VESICLE_MEDIATED_TRANSPORT
9:17732590	*SH3GL2_2*	REACTOME_VESICLE_MEDIATED_TRANSPORT
9:17737514	*SH3GL2_2*	REACTOME_VESICLE_MEDIATED_TRANSPORT
9:17758945	*SH3GL2_2*	REACTOME_VESICLE_MEDIATED_TRANSPORT
10:104005410	*GBF1*	REACTOME_VESICLE_MEDIATED_TRANSPORT
10:104034550	*GBF1*	REACTOME_VESICLE_MEDIATED_TRANSPORT
10:104258303	*GBF1*	REACTOME_VESICLE_MEDIATED_TRANSPORT
10:121669571	*INPP5F*	REACTOME_VESICLE_MEDIATED_TRANSPORT
10:121683206	*INPP5F*	REACTOME_VESICLE_MEDIATED_TRANSPORT
10:121686714	*INPP5F*	REACTOME_VESICLE_MEDIATED_TRANSPORT
10:121689030	*INPP5F*	REACTOME_VESICLE_MEDIATED_TRANSPORT
11:82719121	*DLG2*	REACTOME_VESICLE_MEDIATED_TRANSPORT
12:39759090	*LRRK2_2*	REACTOME_VESICLE_MEDIATED_TRANSPORT
12:39826709	*LRRK2_2*	REACTOME_VESICLE_MEDIATED_TRANSPORT
12:123326598	*HIP1R*	REACTOME_VESICLE_MEDIATED_TRANSPORT
17:7129974	*V1NB1*	REACTOME_VESICLE_MEDIATED_TRANSPORT
17:43017194	*CRHR1_2*	REACTOME_VESICLE_MEDIATED_TRANSPORT
17:43021962	*CRHR1_2*	REACTOME_VESICLE_MEDIATED_TRANSPORT
17:44800946	*WNT3*	REACTOME_VESICLE_MEDIATED_TRANSPORT
17:44833217	*WNT3*	REACTOME_VESICLE_MEDIATED_TRANSPORT
17:45020426	*WNT3*	REACTOME_VESICLE_MEDIATED_TRANSPORT
17:45059032	*WNT3*	REACTOME_VESICLE_MEDIATED_TRANSPORT
17:45089680	*WNT3*	REACTOME_VESICLE_MEDIATED_TRANSPORT
17:76727455	*DNAH17*	REACTOME_VESICLE_MEDIATED_TRANSPORT
2:170012307	*STK39*	REACTOME_VISUAL_PHOTOTRANSDUCTION
2:170028024	*STK39*	REACTOME_VISUAL_PHOTOTRANSDUCTION
2:170066424	*STK39*	REACTOME_VISUAL_PHOTOTRANSDUCTION
10:15199347	*ITGA8*	REACTOME_VISUAL_PHOTOTRANSDUCTION
17:43181287	*CRHR1_2*	REACTOME_VISUAL_PHOTOTRANSDUCTION
17:43184627	*CRHR1_2*	REACTOME_VISUAL_PHOTOTRANSDUCTION
6:133025715	*RPS12*	REACTOME_VITAMIN_B5_PANTOTHENATE_METABOLISM
6:133028870	*RPS12*	REACTOME_VITAMIN_B5_PANTOTHENATE_METABOLISM
6:133075265	*RPS12*	REACTOME_VITAMIN_B5_PANTOTHENATE_METABOLISM
2:170012307	*STK39*	REACTOME_VITAMIN_D_CALCIFEROL_METABOLISM
2:170028024	*STK39*	REACTOME_VITAMIN_D_CALCIFEROL_METABOLISM
2:170066424	*STK39*	REACTOME_VITAMIN_D_CALCIFEROL_METABOLISM
2:18091890	*KCNS3*	REACTOME_VOLTAGE_GATED_POTASSIUM_CHANNELS
2:18130187	*KCNS3*	REACTOME_VOLTAGE_GATED_POTASSIUM_CHANNELS
2:18141542	*KCNS3*	REACTOME_VOLTAGE_GATED_POTASSIUM_CHANNELS
2:18143082	*KCNS3*	REACTOME_VOLTAGE_GATED_POTASSIUM_CHANNELS
2:18176120	*KCNS3*	REACTOME_VOLTAGE_GATED_POTASSIUM_CHANNELS
2:18247777	*KCNS3*	REACTOME_VOLTAGE_GATED_POTASSIUM_CHANNELS
2:18299564	*KCNS3*	REACTOME_VOLTAGE_GATED_POTASSIUM_CHANNELS
2:18315537	*KCNS3*	REACTOME_VOLTAGE_GATED_POTASSIUM_CHANNELS

2:18335515	*KCNS3*	REACTOME_VOLTAGE_GATED_POTASSIUM_CHANNELS
2:18455049	*KCNS3*	REACTOME_VOLTAGE_GATED_POTASSIUM_CHANNELS
2:18472861	*KCNS3*	REACTOME_VOLTAGE_GATED_POTASSIUM_CHANNELS
3:19236100	*SATB1*	REACTOME_VOLTAGE_GATED_POTASSIUM_CHANNELS
3:19312697	*SATB1*	REACTOME_VOLTAGE_GATED_POTASSIUM_CHANNELS
3:19329968	*SATB1*	REACTOME_VOLTAGE_GATED_POTASSIUM_CHANNELS
3:19334895	*SATB1*	REACTOME_VOLTAGE_GATED_POTASSIUM_CHANNELS
3:19352476	*SATB1*	REACTOME_VOLTAGE_GATED_POTASSIUM_CHANNELS
6:73383348	*RIMS1*	REACTOME_VOLTAGE_GATED_POTASSIUM_CHANNELS
6:73448086	*RIMS1*	REACTOME_VOLTAGE_GATED_POTASSIUM_CHANNELS
17:7832034	*V1NB1*	REACTOME_VOLTAGE_GATED_POTASSIUM_CHANNELS
8:131145058	*FAM49B*	REACTOME_VXPX_CARGO_TARGETING_TO_CILIUM
8:131153067	*FAM49B*	REACTOME_VXPX_CARGO_TARGETING_TO_CILIUM
8:131245821	*FAM49B*	REACTOME_VXPX_CARGO_TARGETING_TO_CILIUM
8:131328260	*FAM49B*	REACTOME_VXPX_CARGO_TARGETING_TO_CILIUM
8:131404984	*FAM49B*	REACTOME_VXPX_CARGO_TARGETING_TO_CILIUM
8:131408202	*FAM49B*	REACTOME_VXPX_CARGO_TARGETING_TO_CILIUM
8:131455469	*FAM49B*	REACTOME_VXPX_CARGO_TARGETING_TO_CILIUM
10:104005410	*GBF1*	REACTOME_VXPX_CARGO_TARGETING_TO_CILIUM
10:104034550	*GBF1*	REACTOME_VXPX_CARGO_TARGETING_TO_CILIUM
17:7129974	*V1NB1*	REACTOME_WNT5A_DEPENDENT_INTERNALIZATION_OF_FZD4
17:44843651	*WNT3*	REACTOME_WNT_LIGAND_BIOGENESIS_AND_TRAFFICKING
17:44870978	*WNT3*	REACTOME_WNT_LIGAND_BIOGENESIS_AND_TRAFFICKING
17:44871422	*WNT3*	REACTOME_WNT_LIGAND_BIOGENESIS_AND_TRAFFICKING
17:44888209	*WNT3*	REACTOME_WNT_LIGAND_BIOGENESIS_AND_TRAFFICKING
17:44888715	*WNT3*	REACTOME_WNT_LIGAND_BIOGENESIS_AND_TRAFFICKING
17:44906949	*WNT3*	REACTOME_WNT_LIGAND_BIOGENESIS_AND_TRAFFICKING
17:44912901	*WNT3*	REACTOME_WNT_LIGAND_BIOGENESIS_AND_TRAFFICKING
17:44931399	*WNT3*	REACTOME_WNT_LIGAND_BIOGENESIS_AND_TRAFFICKING
17:44947756	*WNT3*	REACTOME_WNT_LIGAND_BIOGENESIS_AND_TRAFFICKING
17:7129974	*V1NB1*	REACTOME_WNT_MEDIATED_ACTIVATION_OF_DVL
8:11558567	*CTSB*	REACTOME_YAP1_AND_WWTR1_TAZ_STIMULATED_GENE_EXPRESSION
8:11599269	*CTSB*	REACTOME_YAP1_AND_WWTR1_TAZ_STIMULATED_GENE_EXPRESSION
1:226568665	*ITPKB*	SA_CASPASE_CASCADE
17:7578671	*V1NB1*	SA_G1_AND_S_PHASES
3:48199695	*IP6K2*	SA_G2_AND_M_PHASES
3:48214438	*IP6K2*	SA_G2_AND_M_PHASES
4:170477125	*CLCN3*	SA_G2_AND_M_PHASES
3:49406095	*IP6K2*	SA_TRKA_RECEPTOR
3:49424231	*IP6K2*	SA_TRKA_RECEPTOR
3:49450137	*IP6K2*	SA_TRKA_RECEPTOR
3:49561208	*IP6K2*	SIG_BCR_SIGNALING_PATHWAY
5:133558885	*C5orf24*	WNT_SIGNALING
10:103185542	*GBF1*	WNT_SIGNALING
10:103305087	*GBF1*	WNT_SIGNALING
10:103308244	*GBF1*	WNT_SIGNALING
16:50612930	*CASC16*	WNT_SIGNALING
16:50643086	*CASC16*	WNT_SIGNALING
17:7129974	*V1NB1*	WNT_SIGNALING
17:44843651	*WNT3*	WNT_SIGNALING
17:44870978	*WNT3*	WNT_SIGNALING
17:44871422	*WNT3*	WNT_SIGNALING
17:44888209	*WNT3*	WNT_SIGNALING

17:44888715	*WNT3*	WNT_SIGNALING
17:44906949	*WNT3*	WNT_SIGNALING
19:3008151	*SPPL2B*	WNT_SIGNALING
19:3024077	*SPPL2B*	WNT_SIGNALING

b) TESTING PHASE

SNP	Gene	Gene-set
17:7352976	*V1NB1*	BIOCARTA_ACH_PATHWAY
3:48835245	*IP6K2*	BIOCARTA_AGPCR_PATHWAY
3:49561208	*IP6K2*	BIOCARTA_AGR_PATHWAY
6:112561154	*FYN*	BIOCARTA_AGR_PATHWAY
3:48835245	*IP6K2*	BIOCARTA_AKAP13_PATHWAY
3:49406095	*IP6K2*	BIOCARTA_AKAP13_PATHWAY
3:49424231	*IP6K2*	BIOCARTA_AKAP13_PATHWAY
3:49450137	*IP6K2*	BIOCARTA_AKAP13_PATHWAY
3:48835245	*IP6K2*	BIOCARTA_AKAP95_PATHWAY
3:48835245	*IP6K2*	BIOCARTA_AKAPCENTROSOME_PATHWAY
3:49406095	*IP6K2*	BIOCARTA_AKAPCENTROSOME_PATHWAY
3:49424231	*IP6K2*	BIOCARTA_AKAPCENTROSOME_PATHWAY
3:49450137	*IP6K2*	BIOCARTA_AKAPCENTROSOME_PATHWAY
6:31897219	*HLA-DRB5*	BIOCARTA_ALTERNATIVE_PATHWAY
8:131153067	*FAM49B*	BIOCARTA_ARAP_PATHWAY
8:131190319	*FAM49B*	BIOCARTA_ARAP_PATHWAY
8:131427864	*FAM49B*	BIOCARTA_ARAP_PATHWAY
10:104005410	*GBF1*	BIOCARTA_ARAP_PATHWAY
10:104034550	*GBF1*	BIOCARTA_ARAP_PATHWAY
17:76727455	*DNAH17*	BIOCARTA_ARAP_PATHWAY
17:7578671	*V1NB1*	BIOCARTA_ARF_PATHWAY
17:7578671	*V1NB1*	BIOCARTA_ATM_PATHWAY
17:7578671	*V1NB1*	BIOCARTA_ATRBRCA_PATHWAY
3:48835245	*IP6K2*	BIOCARTA_BAD_PATHWAY
6:112009427	*FYN*	BIOCARTA_BIOPEPTIDES_PATHWAY
6:112056759	*FYN*	BIOCARTA_BIOPEPTIDES_PATHWAY
6:112151452	*FYN*	BIOCARTA_BIOPEPTIDES_PATHWAY
16:30510571	*SETD1A*	BIOCARTA_BLYMPHOCYTE_PATHWAY
16:30516492	*SETD1A*	BIOCARTA_BLYMPHOCYTE_PATHWAY
16:30520856	*SETD1A*	BIOCARTA_BLYMPHOCYTE_PATHWAY
17:7578671	*V1NB1*	BIOCARTA_BTG2_PATHWAY
3:49406095	*IP6K2*	BIOCARTA_CARDIACEGF_PATHWAY
3:49424231	*IP6K2*	BIOCARTA_CARDIACEGF_PATHWAY
3:49450137	*IP6K2*	BIOCARTA_CARDIACEGF_PATHWAY
3:48835245	*IP6K2*	BIOCARTA_CARM1_PATHWAY
1:156059276	*GBAP1*	BIOCARTA_CASPASE_PATHWAY
3:49406095	*IP6K2*	BIOCARTA_CCR3_PATHWAY
3:49424231	*IP6K2*	BIOCARTA_CCR3_PATHWAY
3:49450137	*IP6K2*	BIOCARTA_CCR3_PATHWAY
17:43390763	*CRHR1_2*	BIOCARTA_CD40_PATHWAY
3:48199695	*IP6K2*	BIOCARTA_CDC25_PATHWAY
3:48214438	*IP6K2*	BIOCARTA_CDC25_PATHWAY
3:49406095	*IP6K2*	BIOCARTA_CDC42RAC_PATHWAY

3:49424231	*IP6K2*	BIOCARTA_CDC42RAC_PATHWAY
3:49450137	*IP6K2*	BIOCARTA_CDC42RAC_PATHWAY
3:48199695	*IP6K2*	BIOCARTA_CELLCYCLE_PATHWAY
3:48214438	*IP6K2*	BIOCARTA_CELLCYCLE_PATHWAY
3:48835245	*IP6K2*	BIOCARTA_CFTR_PATHWAY
17:7578671	*V1NB1*	BIOCARTA_CHEMICAL_PATHWAY
3:48835245	*IP6K2*	BIOCARTA_CHREBP_PATHWAY
3:48835245	*IP6K2*	BIOCARTA_CK1_PATHWAY
6:31873292	*HLA-DRB5*	BIOCARTA_CLASSIC_PATHWAY
6:31879377	*HLA-DRB5*	BIOCARTA_CLASSIC_PATHWAY
6:31895092	*HLA-DRB5*	BIOCARTA_CLASSIC_PATHWAY
6:31897219	*HLA-DRB5*	BIOCARTA_CLASSIC_PATHWAY
6:31873292	*HLA-DRB5*	BIOCARTA_COMP_PATHWAY
6:31879377	*HLA-DRB5*	BIOCARTA_COMP_PATHWAY
6:31895092	*HLA-DRB5*	BIOCARTA_COMP_PATHWAY
6:31897219	*HLA-DRB5*	BIOCARTA_COMP_PATHWAY
3:48835245	*IP6K2*	BIOCARTA_CREB_PATHWAY
3:48835245	*IP6K2*	BIOCARTA_CSK_PATHWAY
17:7578671	*V1NB1*	BIOCARTA_CTCF_PATHWAY
16:30510571	*SETD1A*	BIOCARTA_CTL_PATHWAY
16:30516492	*SETD1A*	BIOCARTA_CTL_PATHWAY
16:30520856	*SETD1A*	BIOCARTA_CTL_PATHWAY
16:31370452	*SETD1A*	BIOCARTA_DC_PATHWAY
1:156059276	*GBAP1*	BIOCARTA_DEATH_PATHWAY
17:43390763	*CRHR1_2*	BIOCARTA_DEATH_PATHWAY
3:48835245	*IP6K2*	BIOCARTA_DREAM_PATHWAY
3:49406095	*IP6K2*	BIOCARTA_ECM_PATHWAY
3:49424231	*IP6K2*	BIOCARTA_ECM_PATHWAY
3:49450137	*IP6K2*	BIOCARTA_ECM_PATHWAY
6:112009427	*FYN*	BIOCARTA_ECM_PATHWAY
6:112056759	*FYN*	BIOCARTA_ECM_PATHWAY
6:112151452	*FYN*	BIOCARTA_ECM_PATHWAY
3:49406095	*IP6K2*	BIOCARTA_EDG1_PATHWAY
3:49424231	*IP6K2*	BIOCARTA_EDG1_PATHWAY
3:49450137	*IP6K2*	BIOCARTA_EDG1_PATHWAY
17:7578671	*V1NB1*	BIOCARTA_EFP_PATHWAY
17:7479140	*V1NB1*	BIOCARTA_EIF4_PATHWAY
17:7480325	*V1NB1*	BIOCARTA_EIF4_PATHWAY
17:7479140	*V1NB1*	BIOCARTA_EIF_PATHWAY
17:7480325	*V1NB1*	BIOCARTA_EIF_PATHWAY
6:112009427	*FYN*	BIOCARTA_EPHA4_PATHWAY
6:112056759	*FYN*	BIOCARTA_EPHA4_PATHWAY
6:112151452	*FYN*	BIOCARTA_EPHA4_PATHWAY
17:40392358	*RETREG3*	BIOCARTA_EPO_PATHWAY
1:156059276	*GBAP1*	BIOCARTA_FAS_PATHWAY
19:3156505	*SPPL2B*	BIOCARTA_FMLP_PATHWAY
3:48199695	*IP6K2*	BIOCARTA_G1_PATHWAY
3:48214438	*IP6K2*	BIOCARTA_G1_PATHWAY
17:7578671	*V1NB1*	BIOCARTA_G1_PATHWAY
3:48199695	*IP6K2*	BIOCARTA_G2_PATHWAY
3:48214438	*IP6K2*	BIOCARTA_G2_PATHWAY
17:7578671	*V1NB1*	BIOCARTA_G2_PATHWAY
17:44800946	*WNT3*	BIOCARTA_GABA_PATHWAY

17:44833217	*WNT3*	BIOCARTA_GABA_PATHWAY
3:48835245	*IP6K2*	BIOCARTA_GATA3_PATHWAY
17:40392358	*RETREG3*	BIOCARTA_GH_PATHWAY
7:22311894	*GPNMB*	BIOCARTA_GHRELIN_PATHWAY
17:40392358	*RETREG3*	BIOCARTA_GLEEVEC_PATHWAY
3:48835245	*IP6K2*	BIOCARTA_GPCR_PATHWAY
16:30510571	*SETD1A*	BIOCARTA_GRANULOCYTES_PATHWAY
16:30516492	*SETD1A*	BIOCARTA_GRANULOCYTES_PATHWAY
16:30520856	*SETD1A*	BIOCARTA_GRANULOCYTES_PATHWAY
17:8025979	*V1NB1*	BIOCARTA_HES_PATHWAY
1:156059276	*GBAP1*	BIOCARTA_HIVNEF_PATHWAY
1:227067656	*ITPKB*	BIOCARTA_HIVNEF_PATHWAY
17:43390763	*CRHR1_2*	BIOCARTA_HIVNEF_PATHWAY
3:48835245	*IP6K2*	BIOCARTA_IGF1R_PATHWAY
17:43390763	*CRHR1_2*	BIOCARTA_IL1R_PATHWAY
17:40392358	*RETREG3*	BIOCARTA_IL22BP_PATHWAY
17:40392358	*RETREG3*	BIOCARTA_IL2_PATHWAY
17:40392358	*RETREG3*	BIOCARTA_IL2RB_PATHWAY
17:40392358	*RETREG3*	BIOCARTA_IL3_PATHWAY
6:112009427	*FYN*	BIOCARTA_IL7_PATHWAY
6:112056759	*FYN*	BIOCARTA_IL7_PATHWAY
6:112151452	*FYN*	BIOCARTA_IL7_PATHWAY
17:40392358	*RETREG3*	BIOCARTA_IL7_PATHWAY
3:49406095	*IP6K2*	BIOCARTA_INTEGRIN_PATHWAY
3:49424231	*IP6K2*	BIOCARTA_INTEGRIN_PATHWAY
3:49450137	*IP6K2*	BIOCARTA_INTEGRIN_PATHWAY
6:112009427	*FYN*	BIOCARTA_INTEGRIN_PATHWAY
6:112056759	*FYN*	BIOCARTA_INTEGRIN_PATHWAY
6:112151452	*FYN*	BIOCARTA_INTEGRIN_PATHWAY
17:7479140	*V1NB1*	BIOCARTA_IRES_PATHWAY
17:7480325	*V1NB1*	BIOCARTA_IRES_PATHWAY
17:43390763	*CRHR1_2*	BIOCARTA_KERATINOCYTE_PATHWAY
16:30510571	*SETD1A*	BIOCARTA_LAIR_PATHWAY
16:30516492	*SETD1A*	BIOCARTA_LAIR_PATHWAY
16:30520856	*SETD1A*	BIOCARTA_LAIR_PATHWAY
6:31873292	*HLA-DRB5*	BIOCARTA_LECTIN_PATHWAY
6:31879377	*HLA-DRB5*	BIOCARTA_LECTIN_PATHWAY
6:31895092	*HLA-DRB5*	BIOCARTA_LECTIN_PATHWAY
6:31897219	*HLA-DRB5*	BIOCARTA_LECTIN_PATHWAY
12:122816734	*HIP1R*	BIOCARTA_LIS1_PATHWAY
12:122817580	*HIP1R*	BIOCARTA_LIS1_PATHWAY
12:122853197	*HIP1R*	BIOCARTA_LIS1_PATHWAY
17:7631694	*V1NB1*	BIOCARTA_LIS1_PATHWAY
17:7682605	*V1NB1*	BIOCARTA_LIS1_PATHWAY
16:30510571	*SETD1A*	BIOCARTA_LYM_PATHWAY
16:30516492	*SETD1A*	BIOCARTA_LYM_PATHWAY
16:30520856	*SETD1A*	BIOCARTA_LYM_PATHWAY
16:30510571	*SETD1A*	BIOCARTA_LYMPHOCYTE_PATHWAY
16:30516492	*SETD1A*	BIOCARTA_LYMPHOCYTE_PATHWAY
16:30520856	*SETD1A*	BIOCARTA_LYMPHOCYTE_PATHWAY
3:49406095	*IP6K2*	BIOCARTA_MAL_PATHWAY
3:49424231	*IP6K2*	BIOCARTA_MAL_PATHWAY
3:49450137	*IP6K2*	BIOCARTA_MAL_PATHWAY

2:102357447	*MAP4K4*	BIOCARTA_MAPK_PATHWAY
2:102413116	*MAP4K4*	BIOCARTA_MAPK_PATHWAY
17:43390763	*CRHR1_2*	BIOCARTA_MAPK_PATHWAY
3:48835245	*IP6K2*	BIOCARTA_MCALPAIN_PATHWAY
6:32789400	*HLA-DRB5*	BIOCARTA_MHC_PATHWAY
6:32791831	*HLA-DRB5*	BIOCARTA_MHC_PATHWAY
6:32797361	*HLA-DRB5*	BIOCARTA_MHC_PATHWAY
6:32802462	*HLA-DRB5*	BIOCARTA_MHC_PATHWAY
6:32814942	*HLA-DRB5*	BIOCARTA_MHC_PATHWAY
16:30510571	*SETD1A*	BIOCARTA_MONOCYTE_PATHWAY
16:30516492	*SETD1A*	BIOCARTA_MONOCYTE_PATHWAY
16:30520856	*SETD1A*	BIOCARTA_MONOCYTE_PATHWAY
3:48835245	*IP6K2*	BIOCARTA_MPR_PATHWAY
17:7479140	*V1NB1*	BIOCARTA_MTOR_PATHWAY
17:7480325	*V1NB1*	BIOCARTA_MTOR_PATHWAY
16:30510571	*SETD1A*	BIOCARTA_NEUTROPHIL_PATHWAY
16:30516492	*SETD1A*	BIOCARTA_NEUTROPHIL_PATHWAY
16:30520856	*SETD1A*	BIOCARTA_NEUTROPHIL_PATHWAY
3:48835245	*IP6K2*	BIOCARTA_NFAT_PATHWAY
17:43390763	*CRHR1_2*	BIOCARTA_NFKB_PATHWAY
3:48835245	*IP6K2*	BIOCARTA_NO1_PATHWAY
3:48835245	*IP6K2*	BIOCARTA_NOS1_PATHWAY
17:7101771	*V1NB1*	BIOCARTA_NOS1_PATHWAY
17:43390763	*CRHR1_2*	BIOCARTA_NTHI_PATHWAY
12:39960237	*LRRK2_2*	BIOCARTA_NUCLEARRS_PATHWAY
17:7578671	*V1NB1*	BIOCARTA_P53HYPOXIA_PATHWAY
17:7578671	*V1NB1*	BIOCARTA_P53_PATHWAY
3:49406095	*IP6K2*	BIOCARTA_PAR1_PATHWAY
3:49424231	*IP6K2*	BIOCARTA_PAR1_PATHWAY
3:49450137	*IP6K2*	BIOCARTA_PAR1_PATHWAY
4:90677243	*SNCA_2*	BIOCARTA_PARKIN_PATHWAY
4:90700329	*SNCA_2*	BIOCARTA_PARKIN_PATHWAY
4:90727315	*SNCA_2*	BIOCARTA_PARKIN_PATHWAY
4:90738839	*SNCA_2*	BIOCARTA_PARKIN_PATHWAY
4:90757294	*SNCA_2*	BIOCARTA_PARKIN_PATHWAY
11:83188607	*DLG2*	BIOCARTA_PDZS_PATHWAY
11:83219006	*DLG2*	BIOCARTA_PDZS_PATHWAY
11:83538733	*DLG2*	BIOCARTA_PDZS_PATHWAY
11:83544472	*DLG2*	BIOCARTA_PDZS_PATHWAY
11:83656201	*DLG2*	BIOCARTA_PDZS_PATHWAY
11:83700228	*DLG2*	BIOCARTA_PDZS_PATHWAY
11:83762918	*DLG2*	BIOCARTA_PDZS_PATHWAY
11:83894247	*DLG2*	BIOCARTA_PDZS_PATHWAY
11:84021037	*DLG2*	BIOCARTA_PDZS_PATHWAY
11:84324361	*DLG2*	BIOCARTA_PDZS_PATHWAY
11:84373496	*DLG2*	BIOCARTA_PDZS_PATHWAY
11:84403409	*DLG2*	BIOCARTA_PDZS_PATHWAY
17:7101771	*V1NB1*	BIOCARTA_PDZS_PATHWAY
17:42423844	*FAM171A2*	BIOCARTA_PEPI_PATHWAY
3:48835245	*IP6K2*	BIOCARTA_PLCE_PATHWAY
17:7578671	*V1NB1*	BIOCARTA_PLK3_PATHWAY
17:7578671	*V1NB1*	BIOCARTA_PML_PATHWAY
3:48835245	*IP6K2*	BIOCARTA_PPARA_PATHWAY

17:40392358	*RETREG3*	BIOCARTA_PPARA_PATHWAY
10:103185542	*GBF1*	BIOCARTA_PS1_PATHWAY
10:103308244	*GBF1*	BIOCARTA_PS1_PATHWAY
3:48199695	*IP6K2*	BIOCARTA_PTC1_PATHWAY
3:48214438	*IP6K2*	BIOCARTA_PTC1_PATHWAY
1:155975202	*GBAP1*	BIOCARTA_PTDINS_PATHWAY
3:49406095	*IP6K2*	BIOCARTA_RACCYCD_PATHWAY
3:49424231	*IP6K2*	BIOCARTA_RACCYCD_PATHWAY
3:49450137	*IP6K2*	BIOCARTA_RACCYCD_PATHWAY
3:49406095	*IP6K2*	BIOCARTA_RAS_PATHWAY
3:49424231	*IP6K2*	BIOCARTA_RAS_PATHWAY
3:49450137	*IP6K2*	BIOCARTA_RAS_PATHWAY
3:48199695	*IP6K2*	BIOCARTA_RB_PATHWAY
3:48214438	*IP6K2*	BIOCARTA_RB_PATHWAY
17:7578671	*V1NB1*	BIOCARTA_RB_PATHWAY
6:112009427	*FYN*	BIOCARTA_REELIN_PATHWAY
6:112056759	*FYN*	BIOCARTA_REELIN_PATHWAY
6:112151452	*FYN*	BIOCARTA_REELIN_PATHWAY
3:49406095	*IP6K2*	BIOCARTA_RHO_PATHWAY
3:49424231	*IP6K2*	BIOCARTA_RHO_PATHWAY
3:49450137	*IP6K2*	BIOCARTA_RHO_PATHWAY
17:7578671	*V1NB1*	BIOCARTA_RNA_PATHWAY
17:43390763	*CRHR1_2*	BIOCARTA_RNA_PATHWAY
3:48835245	*IP6K2*	BIOCARTA_SHH_PATHWAY
10:104285824	*GBF1*	BIOCARTA_SHH_PATHWAY
21:38740824	*DYRK1A*	BIOCARTA_SHH_PATHWAY
21:38770530	*DYRK1A*	BIOCARTA_SHH_PATHWAY
3:48199695	*IP6K2*	BIOCARTA_SRCRPTP_PATHWAY
3:48214438	*IP6K2*	BIOCARTA_SRCRPTP_PATHWAY
3:48835245	*IP6K2*	BIOCARTA_STATHMIN_PATHWAY
17:43390763	*CRHR1_2*	BIOCARTA_STRESS_PATHWAY
17:7455536	*V1NB1*	BIOCARTA_TALL1_PATHWAY
17:43390763	*CRHR1_2*	BIOCARTA_TALL1_PATHWAY
6:112009427	*FYN*	BIOCARTA_TCRA_PATHWAY
6:112056759	*FYN*	BIOCARTA_TCRA_PATHWAY
6:112151452	*FYN*	BIOCARTA_TCRA_PATHWAY
6:112009427	*FYN*	BIOCARTA_TCR_PATHWAY
6:112056759	*FYN*	BIOCARTA_TCR_PATHWAY
6:112151452	*FYN*	BIOCARTA_TCR_PATHWAY
16:30510571	*SETD1A*	BIOCARTA_TCYTOTOXIC_PATHWAY
16:30516492	*SETD1A*	BIOCARTA_TCYTOTOXIC_PATHWAY
16:30520856	*SETD1A*	BIOCARTA_TCYTOTOXIC_PATHWAY
17:7578671	*V1NB1*	BIOCARTA_TEL_PATHWAY
17:7578671	*V1NB1*	BIOCARTA_TERT_PATHWAY
3:49406095	*IP6K2*	BIOCARTA_TFF_PATHWAY
3:49424231	*IP6K2*	BIOCARTA_TFF_PATHWAY
3:49450137	*IP6K2*	BIOCARTA_TFF_PATHWAY
16:30510571	*SETD1A*	BIOCARTA_THELPER_PATHWAY
16:30516492	*SETD1A*	BIOCARTA_THELPER_PATHWAY
16:30520856	*SETD1A*	BIOCARTA_THELPER_PATHWAY
17:7578671	*V1NB1*	BIOCARTA_TID_PATHWAY
1:156059276	*GBAP1*	BIOCARTA_TNFR1_PATHWAY
17:43390763	*CRHR1_2*	BIOCARTA_TNFR2_PATHWAY

17:43390763	*CRHR1_2*	BIOCARTA_TOLL_PATHWAY
17:40392358	*RETREG3*	BIOCARTA_TPO_PATHWAY
6:112009427	*FYN*	BIOCARTA_TSP1_PATHWAY
6:112056759	*FYN*	BIOCARTA_TSP1_PATHWAY
6:112151452	*FYN*	BIOCARTA_TSP1_PATHWAY
3:49406095	*IP6K2*	BIOCARTA_UCALPAIN_PATHWAY
3:49424231	*IP6K2*	BIOCARTA_UCALPAIN_PATHWAY
3:49450137	*IP6K2*	BIOCARTA_UCALPAIN_PATHWAY
3:48835245	*IP6K2*	BIOCARTA_VIP_PATHWAY
10:103185542	*GBF1*	BIOCARTA_WNT_PATHWAY
10:103308244	*GBF1*	BIOCARTA_WNT_PATHWAY
6:32789400	*HLA-DRB5*	KEGG_ABC_TRANSPORTERS
6:32791831	*HLA-DRB5*	KEGG_ABC_TRANSPORTERS
6:32797361	*HLA-DRB5*	KEGG_ABC_TRANSPORTERS
6:32802462	*HLA-DRB5*	KEGG_ABC_TRANSPORTERS
6:32814942	*HLA-DRB5*	KEGG_ABC_TRANSPORTERS
12:39960237	*LRRK2_2*	KEGG_ABC_TRANSPORTERS
12:123453034	*HIP1R*	KEGG_ABC_TRANSPORTERS
17:39883520	*RETREG3*	KEGG_ACUTE_MYELOID_LEUKEMIA
17:40392358	*RETREG3*	KEGG_ACUTE_MYELOID_LEUKEMIA
3:49406095	*IP6K2*	KEGG_ADHERENS_JUNCTION
3:49424231	*IP6K2*	KEGG_ADHERENS_JUNCTION
3:49450137	*IP6K2*	KEGG_ADHERENS_JUNCTION
6:112009427	*FYN*	KEGG_ADHERENS_JUNCTION
6:112056759	*FYN*	KEGG_ADHERENS_JUNCTION
6:112151452	*FYN*	KEGG_ADHERENS_JUNCTION
17:41056283	*RETREG3*	KEGG_ADIPOCYTOKINE_SIGNALING_PATHWAY
17:7560294	*V1NB1*	KEGG_ALDOSTERONE_REGULATED_SODIUM_REABSORPTION
6:29798642	*TRIM40*	KEGG_ALLOGRAFT_REJECTION
6:32602372	*HLA-DRB5*	KEGG_ALLOGRAFT_REJECTION
6:32930679	*HLA-DRB5*	KEGG_ALLOGRAFT_REJECTION
6:32934409	*HLA-DRB5*	KEGG_ALLOGRAFT_REJECTION
6:32973303	*HLA-DRB5*	KEGG_ALLOGRAFT_REJECTION
6:32974268	*HLA-DRB5*	KEGG_ALLOGRAFT_REJECTION
1:161171736	*FCGR2A*	KEGG_ALZHEIMERS_DISEASE
1:161875645	*FCGR2A*	KEGG_ALZHEIMERS_DISEASE
1:161930954	*FCGR2A*	KEGG_ALZHEIMERS_DISEASE
1:227067656	*ITPKB*	KEGG_ALZHEIMERS_DISEASE
4:90677243	*SNCA_2*	KEGG_ALZHEIMERS_DISEASE
4:90700329	*SNCA_2*	KEGG_ALZHEIMERS_DISEASE
4:90727315	*SNCA_2*	KEGG_ALZHEIMERS_DISEASE
4:90738839	*SNCA_2*	KEGG_ALZHEIMERS_DISEASE
4:90757294	*SNCA_2*	KEGG_ALZHEIMERS_DISEASE
17:43999203	*WNT3*	KEGG_ALZHEIMERS_DISEASE
17:44005297	*WNT3*	KEGG_ALZHEIMERS_DISEASE
17:44019712	*WNT3*	KEGG_ALZHEIMERS_DISEASE
17:44060492	*WNT3*	KEGG_ALZHEIMERS_DISEASE
17:44065263	*WNT3*	KEGG_ALZHEIMERS_DISEASE
17:44081178	*WNT3*	KEGG_ALZHEIMERS_DISEASE
17:44083297	*WNT3*	KEGG_ALZHEIMERS_DISEASE
6:31758240	*HLA-DRB5*	KEGG_AMINOACYL_TRNA_BIOSYNTHESIS
6:31762311	*HLA-DRB5*	KEGG_AMINOACYL_TRNA_BIOSYNTHESIS
17:7578671	*V1NB1*	KEGG_AMYOTROPHIC_LATERAL_SCLEROSIS_ALS

6:29798642	*TRIM40*	KEGG_ANTIGEN_PROCESSING_AND_PRESENTATION
6:32602372	*HLA-DRB5*	KEGG_ANTIGEN_PROCESSING_AND_PRESENTATION
6:32789400	*HLA-DRB5*	KEGG_ANTIGEN_PROCESSING_AND_PRESENTATION
6:32791831	*HLA-DRB5*	KEGG_ANTIGEN_PROCESSING_AND_PRESENTATION
6:32797361	*HLA-DRB5*	KEGG_ANTIGEN_PROCESSING_AND_PRESENTATION
6:32802462	*HLA-DRB5*	KEGG_ANTIGEN_PROCESSING_AND_PRESENTATION
6:32814942	*HLA-DRB5*	KEGG_ANTIGEN_PROCESSING_AND_PRESENTATION
6:32930679	*HLA-DRB5*	KEGG_ANTIGEN_PROCESSING_AND_PRESENTATION
6:32934409	*HLA-DRB5*	KEGG_ANTIGEN_PROCESSING_AND_PRESENTATION
6:32973303	*HLA-DRB5*	KEGG_ANTIGEN_PROCESSING_AND_PRESENTATION
6:32974268	*HLA-DRB5*	KEGG_ANTIGEN_PROCESSING_AND_PRESENTATION
8:11712443	*CTSB*	KEGG_ANTIGEN_PROCESSING_AND_PRESENTATION
3:48835245	*IP6K2*	KEGG_APOPTOSIS
8:23050400	*BIN3*	KEGG_APOPTOSIS
17:7578671	*V1NB1*	KEGG_APOPTOSIS
17:43390763	*CRHR1_2*	KEGG_APOPTOSIS
17:7983799	*V1NB1*	KEGG_ARACHIDONIC_ACID_METABOLISM
1:226108200	*ITPKB*	KEGG_ARGININE_AND_PROLINE_METABOLISM
1:156059276	*GBAP1*	KEGG_ARRHYTHMOGENIC_RIGHT_VENTRICULAR_CARDIOMYOPATHY_ARVC
3:49561208	*IP6K2*	KEGG_ARRHYTHMOGENIC_RIGHT_VENTRICULAR_CARDIOMYOPATHY_ARVC
10:15569598	*ITGA8*	KEGG_ARRHYTHMOGENIC_RIGHT_VENTRICULAR_CARDIOMYOPATHY_ARVC
10:15652368	*ITGA8*	KEGG_ARRHYTHMOGENIC_RIGHT_VENTRICULAR_CARDIOMYOPATHY_ARVC
17:39883520	*RETREG3*	KEGG_ARRHYTHMOGENIC_RIGHT_VENTRICULAR_CARDIOMYOPATHY_ARVC
6:32602372	*HLA-DRB5*	KEGG_ASTHMA
6:32930679	*HLA-DRB5*	KEGG_ASTHMA
6:32934409	*HLA-DRB5*	KEGG_ASTHMA
6:32973303	*HLA-DRB5*	KEGG_ASTHMA
6:32974268	*HLA-DRB5*	KEGG_ASTHMA
6:29798642	*TRIM40*	KEGG_AUTOIMMUNE_THYROID_DISEASE
6:32602372	*HLA-DRB5*	KEGG_AUTOIMMUNE_THYROID_DISEASE
6:32930679	*HLA-DRB5*	KEGG_AUTOIMMUNE_THYROID_DISEASE
6:32934409	*HLA-DRB5*	KEGG_AUTOIMMUNE_THYROID_DISEASE
6:32973303	*HLA-DRB5*	KEGG_AUTOIMMUNE_THYROID_DISEASE
6:32974268	*HLA-DRB5*	KEGG_AUTOIMMUNE_THYROID_DISEASE
3:49406095	*IP6K2*	KEGG_AXON_GUIDANCE
3:49424231	*IP6K2*	KEGG_AXON_GUIDANCE
3:49450137	*IP6K2*	KEGG_AXON_GUIDANCE
3:122645304	*KPNA1*	KEGG_AXON_GUIDANCE
6:112009427	*FYN*	KEGG_AXON_GUIDANCE
6:112056759	*FYN*	KEGG_AXON_GUIDANCE
6:112151452	*FYN*	KEGG_AXON_GUIDANCE
10:104285824	*GBF1*	KEGG_BASAL_CELL_CARCINOMA
17:7129974	*V1NB1*	KEGG_BASAL_CELL_CARCINOMA
17:7578671	*V1NB1*	KEGG_BASAL_CELL_CARCINOMA
17:44843651	*WNT3*	KEGG_BASAL_CELL_CARCINOMA
17:44888209	*WNT3*	KEGG_BASAL_CELL_CARCINOMA
17:44888715	*WNT3*	KEGG_BASAL_CELL_CARCINOMA
17:44906949	*WNT3*	KEGG_BASAL_CELL_CARCINOMA
17:44912901	*WNT3*	KEGG_BASAL_CELL_CARCINOMA
17:44931399	*WNT3*	KEGG_BASAL_CELL_CARCINOMA
17:44947756	*WNT3*	KEGG_BASAL_CELL_CARCINOMA
14:55902186	*GCH1*	KEGG_BASAL_TRANSCRIPTION_FACTORS
12:133206648	*FBRSL1*	KEGG_BASE_EXCISION_REPAIR

12:133223685	*FBRSL1*	KEGG_BASE_EXCISION_REPAIR
12:133225045	*FBRSL1*	KEGG_BASE_EXCISION_REPAIR
17:7578671	*V1NB1*	KEGG_BLADDER_CANCER
1:226910340	*ITPKB*	KEGG_CALCIUM_SIGNALING_PATHWAY
1:226916078	*ITPKB*	KEGG_CALCIUM_SIGNALING_PATHWAY
4:15709252	*BST1*	KEGG_CALCIUM_SIGNALING_PATHWAY
4:15713608	*BST1*	KEGG_CALCIUM_SIGNALING_PATHWAY
4:15724372	*BST1*	KEGG_CALCIUM_SIGNALING_PATHWAY
4:15737882	*BST1*	KEGG_CALCIUM_SIGNALING_PATHWAY
4:15789288	*BST1*	KEGG_CALCIUM_SIGNALING_PATHWAY
4:15816670	*BST1*	KEGG_CALCIUM_SIGNALING_PATHWAY
4:15829612	*BST1*	KEGG_CALCIUM_SIGNALING_PATHWAY
4:114424939	*CAMK2D*	KEGG_CALCIUM_SIGNALING_PATHWAY
17:43195981	*CRHR1_2*	KEGG_CALCIUM_SIGNALING_PATHWAY
19:3156505	*SPPL2B*	KEGG_CALCIUM_SIGNALING_PATHWAY
17:7560294	*V1NB1*	KEGG_CARDIAC_MUSCLE_CONTRACTION
6:29798642	*TRIM40*	KEGG_CELL_ADHESION_MOLECULES_CAMS
6:32602372	*HLA-DRB5*	KEGG_CELL_ADHESION_MOLECULES_CAMS
6:32930679	*HLA-DRB5*	KEGG_CELL_ADHESION_MOLECULES_CAMS
6:32934409	*HLA-DRB5*	KEGG_CELL_ADHESION_MOLECULES_CAMS
6:32973303	*HLA-DRB5*	KEGG_CELL_ADHESION_MOLECULES_CAMS
6:32974268	*HLA-DRB5*	KEGG_CELL_ADHESION_MOLECULES_CAMS
10:15569598	*ITGA8*	KEGG_CELL_ADHESION_MOLECULES_CAMS
10:15652368	*ITGA8*	KEGG_CELL_ADHESION_MOLECULES_CAMS
11:133947732	*IGSF9B*	KEGG_CELL_ADHESION_MOLECULES_CAMS
12:41147295	*LRRK2_2*	KEGG_CELL_ADHESION_MOLECULES_CAMS
12:41251554	*LRRK2_2*	KEGG_CELL_ADHESION_MOLECULES_CAMS
12:41407403	*LRRK2_2*	KEGG_CELL_ADHESION_MOLECULES_CAMS
16:30510571	*SETD1A*	KEGG_CELL_ADHESION_MOLECULES_CAMS
16:30516492	*SETD1A*	KEGG_CELL_ADHESION_MOLECULES_CAMS
16:30520856	*SETD1A*	KEGG_CELL_ADHESION_MOLECULES_CAMS
3:48199695	*IP6K2*	KEGG_CELL_CYCLE
3:48214438	*IP6K2*	KEGG_CELL_CYCLE
17:7578671	*V1NB1*	KEGG_CELL_CYCLE
17:45215865	*WNT3*	KEGG_CELL_CYCLE
17:45244822	*WNT3*	KEGG_CELL_CYCLE
3:49406095	*IP6K2*	KEGG_CHEMOKINE_SIGNALING_PATHWAY
3:49424231	*IP6K2*	KEGG_CHEMOKINE_SIGNALING_PATHWAY
3:49450137	*IP6K2*	KEGG_CHEMOKINE_SIGNALING_PATHWAY
3:123057397	*KPNA1*	KEGG_CHEMOKINE_SIGNALING_PATHWAY
10:121114292	*INPP5F*	KEGG_CHEMOKINE_SIGNALING_PATHWAY
10:121138071	*INPP5F*	KEGG_CHEMOKINE_SIGNALING_PATHWAY
10:121191186	*INPP5F*	KEGG_CHEMOKINE_SIGNALING_PATHWAY
17:40392358	*RETREG3*	KEGG_CHEMOKINE_SIGNALING_PATHWAY
17:7578671	*V1NB1*	KEGG_CHRONIC_MYELOID_LEUKEMIA
17:40392358	*RETREG3*	KEGG_CHRONIC_MYELOID_LEUKEMIA
2:101439596	*MAP4K4*	KEGG_CIRCADIAN_RHYTHM_MAMMAL
2:101550181	*MAP4K4*	KEGG_CIRCADIAN_RHYTHM_MAMMAL
2:101603812	*MAP4K4*	KEGG_CIRCADIAN_RHYTHM_MAMMAL
3:49406095	*IP6K2*	KEGG_COLORECTAL_CANCER
3:49424231	*IP6K2*	KEGG_COLORECTAL_CANCER
3:49450137	*IP6K2*	KEGG_COLORECTAL_CANCER
17:7578671	*V1NB1*	KEGG_COLORECTAL_CANCER

6:31897219	*HLA-DRB5*	KEGG_COMPLEMENT_AND_COAGULATION_CASCADES
2:102609356	*MAP4K4*	KEGG_CYTOKINE_CYTOKINE_RECEPTOR_INTERACTION
2:102636759	*MAP4K4*	KEGG_CYTOKINE_CYTOKINE_RECEPTOR_INTERACTION
2:103063855	*MAP4K4*	KEGG_CYTOKINE_CYTOKINE_RECEPTOR_INTERACTION
8:23050400	*BIN3*	KEGG_CYTOKINE_CYTOKINE_RECEPTOR_INTERACTION
19:2251817	*SPPL2B*	KEGG_CYTOKINE_CYTOKINE_RECEPTOR_INTERACTION
1:156059276	*GBAP1*	KEGG_DILATED_CARDIOMYOPATHY
3:49561208	*IP6K2*	KEGG_DILATED_CARDIOMYOPATHY
3:123057397	*KPNA1*	KEGG_DILATED_CARDIOMYOPATHY
10:15569598	*ITGA8*	KEGG_DILATED_CARDIOMYOPATHY
10:15652368	*ITGA8*	KEGG_DILATED_CARDIOMYOPATHY
12:133206648	*FBRSL1*	KEGG_DNA_REPLICATION
12:133223685	*FBRSL1*	KEGG_DNA_REPLICATION
12:133225045	*FBRSL1*	KEGG_DNA_REPLICATION
6:32186193	*HLA-DRB5*	KEGG_DORSO_VENTRAL_AXIS_FORMATION
7:65425894	*GS1-124K5.11*	KEGG_DRUG_METABOLISM_OTHER_ENZYMES
17:76177567	*DNAH17*	KEGG_DRUG_METABOLISM_OTHER_ENZYMES
3:49162284	*IP6K2*	KEGG_ECM_RECEPTOR_INTERACTION
3:49162583	*IP6K2*	KEGG_ECM_RECEPTOR_INTERACTION
3:49561208	*IP6K2*	KEGG_ECM_RECEPTOR_INTERACTION
6:32031904	*HLA-DRB5*	KEGG_ECM_RECEPTOR_INTERACTION
6:32062687	*HLA-DRB5*	KEGG_ECM_RECEPTOR_INTERACTION
6:33153528	*HLA-DRB5*	KEGG_ECM_RECEPTOR_INTERACTION
6:112561154	*FYN*	KEGG_ECM_RECEPTOR_INTERACTION
10:15569598	*ITGA8*	KEGG_ECM_RECEPTOR_INTERACTION
10:15652368	*ITGA8*	KEGG_ECM_RECEPTOR_INTERACTION
1:171945488	*VAMP4*	KEGG_ENDOCYTOSIS
1:172137119	*VAMP4*	KEGG_ENDOCYTOSIS
1:172386261	*VAMP4*	KEGG_ENDOCYTOSIS
6:29798642	*TRIM40*	KEGG_ENDOCYTOSIS
8:17125768	*FGF20*	KEGG_ENDOCYTOSIS
8:131153067	*FAM49B*	KEGG_ENDOCYTOSIS
8:131190319	*FAM49B*	KEGG_ENDOCYTOSIS
8:131427864	*FAM49B*	KEGG_ENDOCYTOSIS
9:17579690	*SH3GL2_2*	KEGG_ENDOCYTOSIS
9:17609302	*SH3GL2_2*	KEGG_ENDOCYTOSIS
9:17627444	*SH3GL2_2*	KEGG_ENDOCYTOSIS
9:17710876	*SH3GL2_2*	KEGG_ENDOCYTOSIS
9:17732590	*SH3GL2_2*	KEGG_ENDOCYTOSIS
9:17737514	*SH3GL2_2*	KEGG_ENDOCYTOSIS
9:17758945	*SH3GL2_2*	KEGG_ENDOCYTOSIS
10:121114292	*INPP5F*	KEGG_ENDOCYTOSIS
10:121138071	*INPP5F*	KEGG_ENDOCYTOSIS
10:121191186	*INPP5F*	KEGG_ENDOCYTOSIS
17:7578671	*V1NB1*	KEGG_ENDOMETRIAL_CANCER
11:133947732	*IGSF9B*	KEGG_EPITHELIAL_CELL_SIGNALING_IN_HELICOBACTER_PYLORI_INFECTION
12:124219791	*HIP1R*	KEGG_EPITHELIAL_CELL_SIGNALING_IN_HELICOBACTER_PYLORI_INFECTION
17:43390763	*CRHR1_2*	KEGG_EPITHELIAL_CELL_SIGNALING_IN_HELICOBACTER_PYLORI_INFECTION
4:114424939	*CAMK2D*	KEGG_ERBB_SIGNALING_PATHWAY
17:40392358	*RETREG3*	KEGG_ERBB_SIGNALING_PATHWAY
6:112009427	*FYN*	KEGG_FC_EPSILON_RI_SIGNALING_PATHWAY
6:112056759	*FYN*	KEGG_FC_EPSILON_RI_SIGNALING_PATHWAY
6:112151452	*FYN*	KEGG_FC_EPSILON_RI_SIGNALING_PATHWAY

1:171945488	*VAMP4*	KEGG_FC_GAMMA_R_MEDIATED_PHAGOCYTOSIS
1:172137119	*VAMP4*	KEGG_FC_GAMMA_R_MEDIATED_PHAGOCYTOSIS
1:172386261	*VAMP4*	KEGG_FC_GAMMA_R_MEDIATED_PHAGOCYTOSIS
8:131153067	*FAM49B*	KEGG_FC_GAMMA_R_MEDIATED_PHAGOCYTOSIS
8:131190319	*FAM49B*	KEGG_FC_GAMMA_R_MEDIATED_PHAGOCYTOSIS
8:131427864	*FAM49B*	KEGG_FC_GAMMA_R_MEDIATED_PHAGOCYTOSIS
3:49162284	*IP6K2*	KEGG_FOCAL_ADHESION
3:49162583	*IP6K2*	KEGG_FOCAL_ADHESION
3:49406095	*IP6K2*	KEGG_FOCAL_ADHESION
3:49424231	*IP6K2*	KEGG_FOCAL_ADHESION
3:49450137	*IP6K2*	KEGG_FOCAL_ADHESION
6:32031904	*HLA-DRB5*	KEGG_FOCAL_ADHESION
6:32062687	*HLA-DRB5*	KEGG_FOCAL_ADHESION
6:33153528	*HLA-DRB5*	KEGG_FOCAL_ADHESION
6:112009427	*FYN*	KEGG_FOCAL_ADHESION
6:112056759	*FYN*	KEGG_FOCAL_ADHESION
6:112151452	*FYN*	KEGG_FOCAL_ADHESION
6:112561154	*FYN*	KEGG_FOCAL_ADHESION
10:15569598	*ITGA8*	KEGG_FOCAL_ADHESION
10:15652368	*ITGA8*	KEGG_FOCAL_ADHESION
15:62817568	*VPS13C*	KEGG_FOCAL_ADHESION
14:55348869	*GCH1*	KEGG_FOLATE_BIOSYNTHESIS
17:41056283	*RETREG3*	KEGG_GALACTOSE_METABOLISM
3:123057397	*KPNA1*	KEGG_GAP_JUNCTION
4:114424939	*CAMK2D*	KEGG_GLIOMA
17:7578671	*V1NB1*	KEGG_GLIOMA
20:6011934	*CRLS1*	KEGG_GLYCEROPHOSPHOLIPID_METABOLISM
17:41056283	*RETREG3*	KEGG_GLYCOLYSIS_GLUCONEOGENESIS
6:71594785	*RIMS1*	KEGG_GLYCOSAMINOGLYCAN_BIOSYNTHESIS_CHONDROITIN_SULFATE
6:71594785	*RIMS1*	KEGG_GLYCOSAMINOGLYCAN_BIOSYNTHESIS_HEPARAN_SULFATE
7:65425894	*GS1-124K5.11*	KEGG_GLYCOSAMINOGLYCAN_DEGRADATION
6:33252561	*HLA-DRB5*	KEGG_GLYCOSPHINGOLIPID_BIOSYNTHESIS_GANGLIO_SERIES
3:160817877	*SPTSSB*	KEGG_GLYCOSPHINGOLIPID_BIOSYNTHESIS_GLOBO_SERIES
3:182976382	*MCCC1*	KEGG_GLYCOSPHINGOLIPID_BIOSYNTHESIS_LACTO_AND_NEOLACTO_SERIES
3:182979912	*MCCC1*	KEGG_GLYCOSPHINGOLIPID_BIOSYNTHESIS_LACTO_AND_NEOLACTO_SERIES
3:183005320	*MCCC1*	KEGG_GLYCOSPHINGOLIPID_BIOSYNTHESIS_LACTO_AND_NEOLACTO_SERIES
1:172386261	*VAMP4*	KEGG_GLYCOSYLPHOSPHATIDYLINOSITOL_GPI_ANCHOR_BIOSYNTHESIS
1:172388441	*VAMP4*	KEGG_GLYCOSYLPHOSPHATIDYLINOSITOL_GPI_ANCHOR_BIOSYNTHESIS
1:172389198	*VAMP4*	KEGG_GLYCOSYLPHOSPHATIDYLINOSITOL_GPI_ANCHOR_BIOSYNTHESIS
3:123057397	*KPNA1*	KEGG_GNRH_SIGNALING_PATHWAY
4:114424939	*CAMK2D*	KEGG_GNRH_SIGNALING_PATHWAY
6:29798642	*TRIM40*	KEGG_GRAFT_VERSUS_HOST_DISEASE
6:32602372	*HLA-DRB5*	KEGG_GRAFT_VERSUS_HOST_DISEASE
6:32930679	*HLA-DRB5*	KEGG_GRAFT_VERSUS_HOST_DISEASE
6:32934409	*HLA-DRB5*	KEGG_GRAFT_VERSUS_HOST_DISEASE
6:32973303	*HLA-DRB5*	KEGG_GRAFT_VERSUS_HOST_DISEASE
6:32974268	*HLA-DRB5*	KEGG_GRAFT_VERSUS_HOST_DISEASE
2:169999639	*STK39*	KEGG_HEDGEHOG_SIGNALING_PATHWAY
2:170028024	*STK39*	KEGG_HEDGEHOG_SIGNALING_PATHWAY
10:103185542	*GBF1*	KEGG_HEDGEHOG_SIGNALING_PATHWAY
10:103308244	*GBF1*	KEGG_HEDGEHOG_SIGNALING_PATHWAY
10:104285824	*GBF1*	KEGG_HEDGEHOG_SIGNALING_PATHWAY
17:44843651	*WNT3*	KEGG_HEDGEHOG_SIGNALING_PATHWAY

17:44888209	*WNT3*	KEGG_HEDGEHOG_SIGNALING_PATHWAY
17:44888715	*WNT3*	KEGG_HEDGEHOG_SIGNALING_PATHWAY
17:44906949	*WNT3*	KEGG_HEDGEHOG_SIGNALING_PATHWAY
17:44912901	*WNT3*	KEGG_HEDGEHOG_SIGNALING_PATHWAY
17:44931399	*WNT3*	KEGG_HEDGEHOG_SIGNALING_PATHWAY
17:44947756	*WNT3*	KEGG_HEDGEHOG_SIGNALING_PATHWAY
2:102609356	*MAP4K4*	KEGG_HEMATOPOIETIC_CELL_LINEAGE
2:102636759	*MAP4K4*	KEGG_HEMATOPOIETIC_CELL_LINEAGE
4:15789288	*BST1*	KEGG_HEMATOPOIETIC_CELL_LINEAGE
4:15816670	*BST1*	KEGG_HEMATOPOIETIC_CELL_LINEAGE
4:15829612	*BST1*	KEGG_HEMATOPOIETIC_CELL_LINEAGE
1:161171736	*FCGR2A*	KEGG_HUNTINGTONS_DISEASE
14:55902186	*GCH1*	KEGG_HUNTINGTONS_DISEASE
17:7101771	*V1NB1*	KEGG_HUNTINGTONS_DISEASE
17:7578671	*V1NB1*	KEGG_HUNTINGTONS_DISEASE
17:7631694	*V1NB1*	KEGG_HUNTINGTONS_DISEASE
17:7682605	*V1NB1*	KEGG_HUNTINGTONS_DISEASE
17:39883520	*RETREG3*	KEGG_HUNTINGTONS_DISEASE
1:156059276	*GBAP1*	KEGG_HYPERTROPHIC_CARDIOMYOPATHY_HCM
3:49561208	*IP6K2*	KEGG_HYPERTROPHIC_CARDIOMYOPATHY_HCM
10:15569598	*ITGA8*	KEGG_HYPERTROPHIC_CARDIOMYOPATHY_HCM
10:15652368	*ITGA8*	KEGG_HYPERTROPHIC_CARDIOMYOPATHY_HCM
1:226910340	*ITPKB*	KEGG_INOSITOL_PHOSPHATE_METABOLISM
1:226916078	*ITPKB*	KEGG_INOSITOL_PHOSPHATE_METABOLISM
17:43195981	*CRHR1_2*	KEGG_INOSITOL_PHOSPHATE_METABOLISM
3:48835245	*IP6K2*	KEGG_INSULIN_SIGNALING_PATHWAY
14:55515304	*GCH1*	KEGG_INSULIN_SIGNALING_PATHWAY
17:41056283	*RETREG3*	KEGG_INSULIN_SIGNALING_PATHWAY
6:32602372	*HLA-DRB5*	KEGG_INTESTINAL_IMMUNE_NETWORK_FOR_IGA_PRODUCTION
6:32930679	*HLA-DRB5*	KEGG_INTESTINAL_IMMUNE_NETWORK_FOR_IGA_PRODUCTION
6:32934409	*HLA-DRB5*	KEGG_INTESTINAL_IMMUNE_NETWORK_FOR_IGA_PRODUCTION
6:32973303	*HLA-DRB5*	KEGG_INTESTINAL_IMMUNE_NETWORK_FOR_IGA_PRODUCTION
6:32974268	*HLA-DRB5*	KEGG_INTESTINAL_IMMUNE_NETWORK_FOR_IGA_PRODUCTION
17:43390763	*CRHR1_2*	KEGG_INTESTINAL_IMMUNE_NETWORK_FOR_IGA_PRODUCTION
14:55515304	*GCH1*	KEGG_JAK_STAT_SIGNALING_PATHWAY
17:40392358	*RETREG3*	KEGG_JAK_STAT_SIGNALING_PATHWAY
6:32602372	*HLA-DRB5*	KEGG_LEISHMANIA_INFECTION
6:32930679	*HLA-DRB5*	KEGG_LEISHMANIA_INFECTION
6:32934409	*HLA-DRB5*	KEGG_LEISHMANIA_INFECTION
6:32973303	*HLA-DRB5*	KEGG_LEISHMANIA_INFECTION
6:32974268	*HLA-DRB5*	KEGG_LEISHMANIA_INFECTION
3:49406095	*IP6K2*	KEGG_LEUKOCYTE_TRANSENDOTHELIAL_MIGRATION
3:49424231	*IP6K2*	KEGG_LEUKOCYTE_TRANSENDOTHELIAL_MIGRATION
3:49450137	*IP6K2*	KEGG_LEUKOCYTE_TRANSENDOTHELIAL_MIGRATION
11:133947732	*IGSF9B*	KEGG_LEUKOCYTE_TRANSENDOTHELIAL_MIGRATION
16:30510571	*SETD1A*	KEGG_LEUKOCYTE_TRANSENDOTHELIAL_MIGRATION
16:30516492	*SETD1A*	KEGG_LEUKOCYTE_TRANSENDOTHELIAL_MIGRATION
16:30520856	*SETD1A*	KEGG_LEUKOCYTE_TRANSENDOTHELIAL_MIGRATION
17:43744203	*CRHR1_2*	KEGG_LONG_TERM_DEPRESSION
17:43748712	*CRHR1_2*	KEGG_LONG_TERM_DEPRESSION
17:43767804	*CRHR1_2*	KEGG_LONG_TERM_DEPRESSION
17:43784228	*CRHR1_2*	KEGG_LONG_TERM_DEPRESSION
17:43791502	*CRHR1_2*	KEGG_LONG_TERM_DEPRESSION

17:43843861	*CRHR1_2*	KEGG_LONG_TERM_DEPRESSION
17:43847503	*CRHR1_2*	KEGG_LONG_TERM_DEPRESSION
4:114424939	*CAMK2D*	KEGG_LONG_TERM_POTENTIATION
1:155359992	*GBAP1*	KEGG_LYSINE_DEGRADATION
6:31862876	*HLA-DRB5*	KEGG_LYSINE_DEGRADATION
19:2172266	*SPPL2B*	KEGG_LYSINE_DEGRADATION
3:182872860	*MCCC1*	KEGG_LYSOSOME
4:77100807	*FAM47E-STBD1*	KEGG_LYSOSOME
4:77104152	*FAM47E-STBD1*	KEGG_LYSOSOME
4:77124196	*FAM47E-STBD1*	KEGG_LYSOSOME
4:77134786	*FAM47E-STBD1*	KEGG_LYSOSOME
6:32126002	*HLA-DRB5*	KEGG_LYSOSOME
7:65425894	*GS1-124K5.11*	KEGG_LYSOSOME
8:11712443	*CTSB*	KEGG_LYSOSOME
12:123453034	*HIP1R*	KEGG_LYSOSOME
12:124219791	*HIP1R*	KEGG_LYSOSOME
14:88393900	*GALC*	KEGG_LYSOSOME
14:88443553	*GALC*	KEGG_LYSOSOME
19:2114694	*SPPL2B*	KEGG_LYSOSOME
2:102357447	*MAP4K4*	KEGG_MAPK_SIGNALING_PATHWAY
2:102413116	*MAP4K4*	KEGG_MAPK_SIGNALING_PATHWAY
2:102609356	*MAP4K4*	KEGG_MAPK_SIGNALING_PATHWAY
2:102636759	*MAP4K4*	KEGG_MAPK_SIGNALING_PATHWAY
17:7578671	*V1NB1*	KEGG_MAPK_SIGNALING_PATHWAY
17:43390763	*CRHR1_2*	KEGG_MAPK_SIGNALING_PATHWAY
17:43999203	*WNT3*	KEGG_MAPK_SIGNALING_PATHWAY
17:44005297	*WNT3*	KEGG_MAPK_SIGNALING_PATHWAY
17:44019712	*WNT3*	KEGG_MAPK_SIGNALING_PATHWAY
17:44060492	*WNT3*	KEGG_MAPK_SIGNALING_PATHWAY
17:44065263	*WNT3*	KEGG_MAPK_SIGNALING_PATHWAY
17:44081178	*WNT3*	KEGG_MAPK_SIGNALING_PATHWAY
17:44083297	*WNT3*	KEGG_MAPK_SIGNALING_PATHWAY
3:123057397	*KPNA1*	KEGG_MELANOGENESIS
4:114424939	*CAMK2D*	KEGG_MELANOGENESIS
17:7129974	*V1NB1*	KEGG_MELANOGENESIS
17:44843651	*WNT3*	KEGG_MELANOGENESIS
17:44888209	*WNT3*	KEGG_MELANOGENESIS
17:44888715	*WNT3*	KEGG_MELANOGENESIS
17:44906949	*WNT3*	KEGG_MELANOGENESIS
17:44912901	*WNT3*	KEGG_MELANOGENESIS
17:44931399	*WNT3*	KEGG_MELANOGENESIS
17:44947756	*WNT3*	KEGG_MELANOGENESIS
17:7578671	*V1NB1*	KEGG_MELANOMA
14:75495059	*RPS6KL1*	KEGG_MISMATCH_REPAIR
13:49914395	*CAB39L*	KEGG_MTOR_SIGNALING_PATHWAY
13:49927732	*CAB39L*	KEGG_MTOR_SIGNALING_PATHWAY
13:49948786	*CAB39L*	KEGG_MTOR_SIGNALING_PATHWAY
13:49960054	*CAB39L*	KEGG_MTOR_SIGNALING_PATHWAY
13:49977462	*CAB39L*	KEGG_MTOR_SIGNALING_PATHWAY
6:29798642	*TRIM40*	KEGG_NATURAL_KILLER_CELL_MEDIATED_CYTOTOXICITY
6:112009427	*FYN*	KEGG_NATURAL_KILLER_CELL_MEDIATED_CYTOTOXICITY
6:112056759	*FYN*	KEGG_NATURAL_KILLER_CELL_MEDIATED_CYTOTOXICITY
6:112151452	*FYN*	KEGG_NATURAL_KILLER_CELL_MEDIATED_CYTOTOXICITY

8:23050400	*BIN3*	KEGG_NATURAL_KILLER_CELL_MEDIATED_CYTOTOXICITY
16:30510571	*SETD1A*	KEGG_NATURAL_KILLER_CELL_MEDIATED_CYTOTOXICITY
16:30516492	*SETD1A*	KEGG_NATURAL_KILLER_CELL_MEDIATED_CYTOTOXICITY
16:30520856	*SETD1A*	KEGG_NATURAL_KILLER_CELL_MEDIATED_CYTOTOXICITY
6:29579544	*TRIM40*	KEGG_NEUROACTIVE_LIGAND_RECEPTOR_INTERACTION
9:33791164	*UBAP2*	KEGG_NEUROACTIVE_LIGAND_RECEPTOR_INTERACTION
17:7352976	*V1NB1*	KEGG_NEUROACTIVE_LIGAND_RECEPTOR_INTERACTION
17:43744203	*CRHR1_2*	KEGG_NEUROACTIVE_LIGAND_RECEPTOR_INTERACTION
17:43748712	*CRHR1_2*	KEGG_NEUROACTIVE_LIGAND_RECEPTOR_INTERACTION
17:43767804	*CRHR1_2*	KEGG_NEUROACTIVE_LIGAND_RECEPTOR_INTERACTION
17:43784228	*CRHR1_2*	KEGG_NEUROACTIVE_LIGAND_RECEPTOR_INTERACTION
17:43791502	*CRHR1_2*	KEGG_NEUROACTIVE_LIGAND_RECEPTOR_INTERACTION
17:43843861	*CRHR1_2*	KEGG_NEUROACTIVE_LIGAND_RECEPTOR_INTERACTION
17:43847503	*CRHR1_2*	KEGG_NEUROACTIVE_LIGAND_RECEPTOR_INTERACTION
3:49406095	*IP6K2*	KEGG_NEUROTROPHIN_SIGNALING_PATHWAY
3:49424231	*IP6K2*	KEGG_NEUROTROPHIN_SIGNALING_PATHWAY
3:49450137	*IP6K2*	KEGG_NEUROTROPHIN_SIGNALING_PATHWAY
4:114424939	*CAMK2D*	KEGG_NEUROTROPHIN_SIGNALING_PATHWAY
17:7578671	*V1NB1*	KEGG_NEUROTROPHIN_SIGNALING_PATHWAY
2:134977261	*TMEM163*	KEGG_N_GLYCAN_BIOSYNTHESIS
2:135194840	*TMEM163*	KEGG_N_GLYCAN_BIOSYNTHESIS
2:135202455	*TMEM163*	KEGG_N_GLYCAN_BIOSYNTHESIS
4:15709252	*BST1*	KEGG_NICOTINATE_AND_NICOTINAMIDE_METABOLISM
4:15713608	*BST1*	KEGG_NICOTINATE_AND_NICOTINAMIDE_METABOLISM
4:15724372	*BST1*	KEGG_NICOTINATE_AND_NICOTINAMIDE_METABOLISM
4:15737882	*BST1*	KEGG_NICOTINATE_AND_NICOTINAMIDE_METABOLISM
4:15789288	*BST1*	KEGG_NICOTINATE_AND_NICOTINAMIDE_METABOLISM
4:15816670	*BST1*	KEGG_NICOTINATE_AND_NICOTINAMIDE_METABOLISM
4:15829612	*BST1*	KEGG_NICOTINATE_AND_NICOTINAMIDE_METABOLISM
10:104847713	*GBF1*	KEGG_NICOTINATE_AND_NICOTINAMIDE_METABOLISM
10:104861736	*GBF1*	KEGG_NICOTINATE_AND_NICOTINAMIDE_METABOLISM
10:104926485	*GBF1*	KEGG_NICOTINATE_AND_NICOTINAMIDE_METABOLISM
10:104929716	*GBF1*	KEGG_NICOTINATE_AND_NICOTINAMIDE_METABOLISM
16:50730088	*CASC16*	KEGG_NOD_LIKE_RECEPTOR_SIGNALING_PATHWAY
16:50730446	*CASC16*	KEGG_NOD_LIKE_RECEPTOR_SIGNALING_PATHWAY
16:50745926	*CASC16*	KEGG_NOD_LIKE_RECEPTOR_SIGNALING_PATHWAY
17:7578671	*V1NB1*	KEGG_NON_SMALL_CELL_LUNG_CANCER
1:227067656	*ITPKB*	KEGG_NOTCH_SIGNALING_PATHWAY
3:122289921	*KPNA1*	KEGG_NOTCH_SIGNALING_PATHWAY
6:32186193	*HLA-DRB5*	KEGG_NOTCH_SIGNALING_PATHWAY
17:7129974	*V1NB1*	KEGG_NOTCH_SIGNALING_PATHWAY
12:133206648	*FBRSL1*	KEGG_NUCLEOTIDE_EXCISION_REPAIR
12:133223685	*FBRSL1*	KEGG_NUCLEOTIDE_EXCISION_REPAIR
12:133225045	*FBRSL1*	KEGG_NUCLEOTIDE_EXCISION_REPAIR
12:132687184	*FBRSL1*	KEGG_O_GLYCAN_BIOSYNTHESIS
4:114424939	*CAMK2D*	KEGG_OLFACTORY_TRANSDUCTION
6:29351969	*TRIM40*	KEGG_OLFACTORY_TRANSDUCTION
6:29373213	*TRIM40*	KEGG_OLFACTORY_TRANSDUCTION
6:29407378	*TRIM40*	KEGG_OLFACTORY_TRANSDUCTION
6:29412222	*TRIM40*	KEGG_OLFACTORY_TRANSDUCTION
3:123057397	*KPNA1*	KEGG_OOCYTE_MEIOSIS
4:114424939	*CAMK2D*	KEGG_OOCYTE_MEIOSIS
10:103185542	*GBF1*	KEGG_OOCYTE_MEIOSIS

10:103308244	*GBF1*	KEGG_OOCYTE_MEIOSIS
17:45215865	*WNT3*	KEGG_OOCYTE_MEIOSIS
17:45244822	*WNT3*	KEGG_OOCYTE_MEIOSIS
1:161171736	*FCGR2A*	KEGG_OXIDATIVE_PHOSPHORYLATION
12:124219791	*HIP1R*	KEGG_OXIDATIVE_PHOSPHORYLATION
4:78175384	*FAM47E-STBD1*	KEGG_P53_SIGNALING_PATHWAY
17:7578671	*V1NB1*	KEGG_P53_SIGNALING_PATHWAY
17:7578671	*V1NB1*	KEGG_PANCREATIC_CANCER
6:133025715	*RPS12*	KEGG_PANTOTHENATE_AND_COA_BIOSYNTHESIS
6:133028870	*RPS12*	KEGG_PANTOTHENATE_AND_COA_BIOSYNTHESIS
6:133034076	*RPS12*	KEGG_PANTOTHENATE_AND_COA_BIOSYNTHESIS
1:161171736	*FCGR2A*	KEGG_PARKINSONS_DISEASE
4:90677243	*SNCA_2*	KEGG_PARKINSONS_DISEASE
4:90700329	*SNCA_2*	KEGG_PARKINSONS_DISEASE
4:90727315	*SNCA_2*	KEGG_PARKINSONS_DISEASE
4:90738839	*SNCA_2*	KEGG_PARKINSONS_DISEASE
4:90757294	*SNCA_2*	KEGG_PARKINSONS_DISEASE
12:40614434	*LRRK2_2*	KEGG_PARKINSONS_DISEASE
12:40614656	*LRRK2_2*	KEGG_PARKINSONS_DISEASE
12:40617231	*LRRK2_2*	KEGG_PARKINSONS_DISEASE
12:40653993	*LRRK2_2*	KEGG_PARKINSONS_DISEASE
12:40698422	*LRRK2_2*	KEGG_PARKINSONS_DISEASE
12:40707861	*LRRK2_2*	KEGG_PARKINSONS_DISEASE
12:40753796	*LRRK2_2*	KEGG_PARKINSONS_DISEASE
1:155975202	*GBAP1*	KEGG_PATHOGENIC_ESCHERICHIA_COLI_INFECTION
3:49406095	*IP6K2*	KEGG_PATHOGENIC_ESCHERICHIA_COLI_INFECTION
3:49424231	*IP6K2*	KEGG_PATHOGENIC_ESCHERICHIA_COLI_INFECTION
3:49450137	*IP6K2*	KEGG_PATHOGENIC_ESCHERICHIA_COLI_INFECTION
6:112009427	*FYN*	KEGG_PATHOGENIC_ESCHERICHIA_COLI_INFECTION
6:112056759	*FYN*	KEGG_PATHOGENIC_ESCHERICHIA_COLI_INFECTION
6:112151452	*FYN*	KEGG_PATHOGENIC_ESCHERICHIA_COLI_INFECTION
3:49162284	*IP6K2*	KEGG_PATHWAYS_IN_CANCER
3:49162583	*IP6K2*	KEGG_PATHWAYS_IN_CANCER
3:49406095	*IP6K2*	KEGG_PATHWAYS_IN_CANCER
3:49424231	*IP6K2*	KEGG_PATHWAYS_IN_CANCER
3:49450137	*IP6K2*	KEGG_PATHWAYS_IN_CANCER
6:112561154	*FYN*	KEGG_PATHWAYS_IN_CANCER
10:104285824	*GBF1*	KEGG_PATHWAYS_IN_CANCER
17:7129974	*V1NB1*	KEGG_PATHWAYS_IN_CANCER
17:7578671	*V1NB1*	KEGG_PATHWAYS_IN_CANCER
17:39883520	*RETREG3*	KEGG_PATHWAYS_IN_CANCER
17:40392358	*RETREG3*	KEGG_PATHWAYS_IN_CANCER
17:44843651	*WNT3*	KEGG_PATHWAYS_IN_CANCER
17:44888209	*WNT3*	KEGG_PATHWAYS_IN_CANCER
17:44888715	*WNT3*	KEGG_PATHWAYS_IN_CANCER
17:44906949	*WNT3*	KEGG_PATHWAYS_IN_CANCER
17:44912901	*WNT3*	KEGG_PATHWAYS_IN_CANCER
17:44931399	*WNT3*	KEGG_PATHWAYS_IN_CANCER
17:44947756	*WNT3*	KEGG_PATHWAYS_IN_CANCER
7:65425894	*GS1-124K5.11*	KEGG_PENTOSE_AND_GLUCURONATE_INTERCONVERSIONS
12:39960237	*LRRK2_2*	KEGG_PEROXISOME
12:133295501	*FBRSL1*	KEGG_PEROXISOME
1:226910340	*ITPKB*	KEGG_PHOSPHATIDYLINOSITOL_SIGNALING_SYSTEM

1:226916078	*ITPKB*	KEGG_PHOSPHATIDYLINOSITOL_SIGNALING_SYSTEM
17:43195981	*CRHR1_2*	KEGG_PHOSPHATIDYLINOSITOL_SIGNALING_SYSTEM
7:65425894	*GS1-124K5.11*	KEGG_PORPHYRIN_AND_CHLOROPHYLL_METABOLISM
16:30999462	*SETD1A*	KEGG_PRIMARY_BILE_ACID_BIOSYNTHESIS
6:32789400	*HLA-DRB5*	KEGG_PRIMARY_IMMUNODEFICIENCY
6:32791831	*HLA-DRB5*	KEGG_PRIMARY_IMMUNODEFICIENCY
6:32797361	*HLA-DRB5*	KEGG_PRIMARY_IMMUNODEFICIENCY
6:32802462	*HLA-DRB5*	KEGG_PRIMARY_IMMUNODEFICIENCY
6:32814942	*HLA-DRB5*	KEGG_PRIMARY_IMMUNODEFICIENCY
6:112009427	*FYN*	KEGG_PRION_DISEASES
6:112056759	*FYN*	KEGG_PRION_DISEASES
6:112151452	*FYN*	KEGG_PRION_DISEASES
3:48199695	*IP6K2*	KEGG_PROGESTERONE_MEDIATED_OOCYTE_MATURATION
3:48214438	*IP6K2*	KEGG_PROGESTERONE_MEDIATED_OOCYTE_MATURATION
3:123057397	*KPNA1*	KEGG_PROGESTERONE_MEDIATED_OOCYTE_MATURATION
17:45215865	*WNT3*	KEGG_PROGESTERONE_MEDIATED_OOCYTE_MATURATION
17:45244822	*WNT3*	KEGG_PROGESTERONE_MEDIATED_OOCYTE_MATURATION
17:7578671	*V1NB1*	KEGG_PROSTATE_CANCER
6:32814942	*HLA-DRB5*	KEGG_PROTEASOME
17:7560294	*V1NB1*	KEGG_PROXIMAL_TUBULE_BICARBONATE_RECLAMATION
3:123057397	*KPNA1*	KEGG_PURINE_METABOLISM
5:59236972	*ELOVL7*	KEGG_PURINE_METABOLISM
5:59665659	*ELOVL7*	KEGG_PURINE_METABOLISM
5:59705316	*ELOVL7*	KEGG_PURINE_METABOLISM
5:59736085	*ELOVL7*	KEGG_PURINE_METABOLISM
9:34342881	*UBAP2*	KEGG_PURINE_METABOLISM
10:104847713	*GBF1*	KEGG_PURINE_METABOLISM
10:104861736	*GBF1*	KEGG_PURINE_METABOLISM
10:104926485	*GBF1*	KEGG_PURINE_METABOLISM
10:104929716	*GBF1*	KEGG_PURINE_METABOLISM
11:10392432	*RNF141*	KEGG_PURINE_METABOLISM
11:10487997	*RNF141*	KEGG_PURINE_METABOLISM
12:133206648	*FBRSL1*	KEGG_PURINE_METABOLISM
12:133223685	*FBRSL1*	KEGG_PURINE_METABOLISM
12:133225045	*FBRSL1*	KEGG_PURINE_METABOLISM
14:74477206	*RPS6KL1*	KEGG_PURINE_METABOLISM
9:34342881	*UBAP2*	KEGG_PYRIMIDINE_METABOLISM
10:104847713	*GBF1*	KEGG_PYRIMIDINE_METABOLISM
10:104861736	*GBF1*	KEGG_PYRIMIDINE_METABOLISM
10:104926485	*GBF1*	KEGG_PYRIMIDINE_METABOLISM
10:104929716	*GBF1*	KEGG_PYRIMIDINE_METABOLISM
12:133206648	*FBRSL1*	KEGG_PYRIMIDINE_METABOLISM
12:133223685	*FBRSL1*	KEGG_PYRIMIDINE_METABOLISM
12:133225045	*FBRSL1*	KEGG_PYRIMIDINE_METABOLISM
14:74477206	*RPS6KL1*	KEGG_PYRIMIDINE_METABOLISM
17:76177567	*DNAH17*	KEGG_PYRIMIDINE_METABOLISM
3:49406095	*IP6K2*	KEGG_REGULATION_OF_ACTIN_CYTOSKELETON
3:49424231	*IP6K2*	KEGG_REGULATION_OF_ACTIN_CYTOSKELETON
3:49450137	*IP6K2*	KEGG_REGULATION_OF_ACTIN_CYTOSKELETON
10:15569598	*ITGA8*	KEGG_REGULATION_OF_ACTIN_CYTOSKELETON
10:15652368	*ITGA8*	KEGG_REGULATION_OF_ACTIN_CYTOSKELETON
16:30510571	*SETD1A*	KEGG_REGULATION_OF_ACTIN_CYTOSKELETON
16:30516492	*SETD1A*	KEGG_REGULATION_OF_ACTIN_CYTOSKELETON

16:30520856	*SETD1A*	KEGG_REGULATION_OF_ACTIN_CYTOSKELETON
16:31370452	*SETD1A*	KEGG_REGULATION_OF_ACTIN_CYTOSKELETON
3:49162284	*IP6K2*	KEGG_SMALL_CELL_LUNG_CANCER
3:49162583	*IP6K2*	KEGG_SMALL_CELL_LUNG_CANCER
6:112561154	*FYN*	KEGG_SMALL_CELL_LUNG_CANCER
17:7578671	*V1NB1*	KEGG_SMALL_CELL_LUNG_CANCER
1:171689486	*VAMP4*	KEGG_SNARE_INTERACTIONS_IN_VESICULAR_TRANSPORT
16:31004169	*SETD1A*	KEGG_SNARE_INTERACTIONS_IN_VESICULAR_TRANSPORT
17:45059032	*WNT3*	KEGG_SNARE_INTERACTIONS_IN_VESICULAR_TRANSPORT
17:45089680	*WNT3*	KEGG_SNARE_INTERACTIONS_IN_VESICULAR_TRANSPORT
14:88393900	*GALC*	KEGG_SPHINGOLIPID_METABOLISM
14:88443553	*GALC*	KEGG_SPHINGOLIPID_METABOLISM
6:30628082	*TRIM40*	KEGG_SPLICEOSOME
6:30631063	*TRIM40*	KEGG_SPLICEOSOME
6:30639369	*TRIM40*	KEGG_SPLICEOSOME
17:42929985	*CRHR1_2*	KEGG_SPLICEOSOME
7:65425894	*GS1-124K5.11*	KEGG_STARCH_AND_SUCROSE_METABOLISM
17:41056283	*RETREG3*	KEGG_STARCH_AND_SUCROSE_METABOLISM
10:104591639	*GBF1*	KEGG_STEROID_HORMONE_BIOSYNTHESIS
6:31873292	*HLA-DRB5*	KEGG_SYSTEMIC_LUPUS_ERYTHEMATOSUS
6:31879377	*HLA-DRB5*	KEGG_SYSTEMIC_LUPUS_ERYTHEMATOSUS
6:31895092	*HLA-DRB5*	KEGG_SYSTEMIC_LUPUS_ERYTHEMATOSUS
6:31897219	*HLA-DRB5*	KEGG_SYSTEMIC_LUPUS_ERYTHEMATOSUS
6:32602372	*HLA-DRB5*	KEGG_SYSTEMIC_LUPUS_ERYTHEMATOSUS
6:32930679	*HLA-DRB5*	KEGG_SYSTEMIC_LUPUS_ERYTHEMATOSUS
6:32934409	*HLA-DRB5*	KEGG_SYSTEMIC_LUPUS_ERYTHEMATOSUS
6:32973303	*HLA-DRB5*	KEGG_SYSTEMIC_LUPUS_ERYTHEMATOSUS
6:32974268	*HLA-DRB5*	KEGG_SYSTEMIC_LUPUS_ERYTHEMATOSUS
3:49406095	*IP6K2*	KEGG_T_CELL_RECEPTOR_SIGNALING_PATHWAY
3:49424231	*IP6K2*	KEGG_T_CELL_RECEPTOR_SIGNALING_PATHWAY
3:49450137	*IP6K2*	KEGG_T_CELL_RECEPTOR_SIGNALING_PATHWAY
6:112009427	*FYN*	KEGG_T_CELL_RECEPTOR_SIGNALING_PATHWAY
6:112056759	*FYN*	KEGG_T_CELL_RECEPTOR_SIGNALING_PATHWAY
6:112151452	*FYN*	KEGG_T_CELL_RECEPTOR_SIGNALING_PATHWAY
17:43390763	*CRHR1_2*	KEGG_T_CELL_RECEPTOR_SIGNALING_PATHWAY
3:49406095	*IP6K2*	KEGG_TGF_BETA_SIGNALING_PATHWAY
3:49424231	*IP6K2*	KEGG_TGF_BETA_SIGNALING_PATHWAY
3:49450137	*IP6K2*	KEGG_TGF_BETA_SIGNALING_PATHWAY
19:2251817	*SPPL2B*	KEGG_TGF_BETA_SIGNALING_PATHWAY
17:7578671	*V1NB1*	KEGG_THYROID_CANCER
1:155359992	*GBAP1*	KEGG_TIGHT_JUNCTION
3:49406095	*IP6K2*	KEGG_TIGHT_JUNCTION
3:49424231	*IP6K2*	KEGG_TIGHT_JUNCTION
3:49450137	*IP6K2*	KEGG_TIGHT_JUNCTION
11:133947732	*IGSF9B*	KEGG_TIGHT_JUNCTION
6:29798642	*TRIM40*	KEGG_TYPE_I_DIABETES_MELLITUS
6:32602372	*HLA-DRB5*	KEGG_TYPE_I_DIABETES_MELLITUS
6:32930679	*HLA-DRB5*	KEGG_TYPE_I_DIABETES_MELLITUS
6:32934409	*HLA-DRB5*	KEGG_TYPE_I_DIABETES_MELLITUS
6:32973303	*HLA-DRB5*	KEGG_TYPE_I_DIABETES_MELLITUS
6:32974268	*HLA-DRB5*	KEGG_TYPE_I_DIABETES_MELLITUS
14:55515304	*GCH1*	KEGG_TYPE_II_DIABETES_MELLITUS
5:133710339	*C5orf24*	KEGG_UBIQUITIN_MEDIATED_PROTEOLYSIS

9:33845203	*UBAP2*	KEGG_UBIQUITIN_MEDIATED_PROTEOLYSIS
10:103185542	*GBF1*	KEGG_UBIQUITIN_MEDIATED_PROTEOLYSIS
10:103308244	*GBF1*	KEGG_UBIQUITIN_MEDIATED_PROTEOLYSIS
17:45215865	*WNT3*	KEGG_UBIQUITIN_MEDIATED_PROTEOLYSIS
17:45244822	*WNT3*	KEGG_UBIQUITIN_MEDIATED_PROTEOLYSIS
6:31758240	*HLA-DRB5*	KEGG_VALINE_LEUCINE_AND_ISOLEUCINE_BIOSYNTHESIS
6:31762311	*HLA-DRB5*	KEGG_VALINE_LEUCINE_AND_ISOLEUCINE_BIOSYNTHESIS
3:182760073	*MCCC1*	KEGG_VALINE_LEUCINE_AND_ISOLEUCINE_DEGRADATION
3:49406095	*IP6K2*	KEGG_VASCULAR_SMOOTH_MUSCLE_CONTRACTION
3:49424231	*IP6K2*	KEGG_VASCULAR_SMOOTH_MUSCLE_CONTRACTION
3:49450137	*IP6K2*	KEGG_VASCULAR_SMOOTH_MUSCLE_CONTRACTION
3:123057397	*KPNA1*	KEGG_VASCULAR_SMOOTH_MUSCLE_CONTRACTION
11:10647977	*RNF141*	KEGG_VASCULAR_SMOOTH_MUSCLE_CONTRACTION
11:10648481	*RNF141*	KEGG_VASCULAR_SMOOTH_MUSCLE_CONTRACTION
11:10660840	*RNF141*	KEGG_VASCULAR_SMOOTH_MUSCLE_CONTRACTION
17:44800946	*WNT3*	KEGG_VASOPRESSIN_REGULATED_WATER_REABSORPTION
17:44833217	*WNT3*	KEGG_VASOPRESSIN_REGULATED_WATER_REABSORPTION
12:124219791	*HIP1R*	KEGG_VIBRIO_CHOLERAE_INFECTION
3:49561208	*IP6K2*	KEGG_VIRAL_MYOCARDITIS
6:29798642	*TRIM40*	KEGG_VIRAL_MYOCARDITIS
6:32602372	*HLA-DRB5*	KEGG_VIRAL_MYOCARDITIS
6:32930679	*HLA-DRB5*	KEGG_VIRAL_MYOCARDITIS
6:32934409	*HLA-DRB5*	KEGG_VIRAL_MYOCARDITIS
6:32973303	*HLA-DRB5*	KEGG_VIRAL_MYOCARDITIS
6:32974268	*HLA-DRB5*	KEGG_VIRAL_MYOCARDITIS
6:112009427	*FYN*	KEGG_VIRAL_MYOCARDITIS
6:112056759	*FYN*	KEGG_VIRAL_MYOCARDITIS
6:112151452	*FYN*	KEGG_VIRAL_MYOCARDITIS
16:30510571	*SETD1A*	KEGG_VIRAL_MYOCARDITIS
16:30516492	*SETD1A*	KEGG_VIRAL_MYOCARDITIS
16:30520856	*SETD1A*	KEGG_VIRAL_MYOCARDITIS
3:49406095	*IP6K2*	KEGG_WNT_SIGNALING_PATHWAY
3:49424231	*IP6K2*	KEGG_WNT_SIGNALING_PATHWAY
3:49450137	*IP6K2*	KEGG_WNT_SIGNALING_PATHWAY
4:114424939	*CAMK2D*	KEGG_WNT_SIGNALING_PATHWAY
10:103185542	*GBF1*	KEGG_WNT_SIGNALING_PATHWAY
10:103308244	*GBF1*	KEGG_WNT_SIGNALING_PATHWAY
17:7129974	*V1NB1*	KEGG_WNT_SIGNALING_PATHWAY
17:7578671	*V1NB1*	KEGG_WNT_SIGNALING_PATHWAY
17:44843651	*WNT3*	KEGG_WNT_SIGNALING_PATHWAY
17:44888209	*WNT3*	KEGG_WNT_SIGNALING_PATHWAY
17:44888715	*WNT3*	KEGG_WNT_SIGNALING_PATHWAY
17:44906949	*WNT3*	KEGG_WNT_SIGNALING_PATHWAY
17:44912901	*WNT3*	KEGG_WNT_SIGNALING_PATHWAY
17:44931399	*WNT3*	KEGG_WNT_SIGNALING_PATHWAY
17:44947756	*WNT3*	KEGG_WNT_SIGNALING_PATHWAY
3:49162284	*IP6K2*	NABA_BASEMENT_MEMBRANES
3:49162583	*IP6K2*	NABA_BASEMENT_MEMBRANES
6:112561154	*FYN*	NABA_BASEMENT_MEMBRANES
6:33153528	*HLA-DRB5*	NABA_COLLAGENS
3:49162284	*IP6K2*	NABA_CORE_MATRISOME
3:49162583	*IP6K2*	NABA_CORE_MATRISOME
4:90804532	*SNCA_2*	NABA_CORE_MATRISOME

4:90868355	*SNCA_2*	NABA_CORE_MATRISOME
6:32031904	*HLA-DRB5*	NABA_CORE_MATRISOME
6:32062687	*HLA-DRB5*	NABA_CORE_MATRISOME
6:33153528	*HLA-DRB5*	NABA_CORE_MATRISOME
6:112561154	*FYN*	NABA_CORE_MATRISOME
14:75057014	*RPS6KL1*	NABA_CORE_MATRISOME
3:122645304	*KPNA1*	NABA_ECM_AFFILIATED
4:15354235	*BST1*	NABA_ECM_AFFILIATED
6:31002527	*TRIM40*	NABA_ECM_AFFILIATED
12:40798608	*LRRK2_2*	NABA_ECM_AFFILIATED
12:40800267	*LRRK2_2*	NABA_ECM_AFFILIATED
12:40837264	*LRRK2_2*	NABA_ECM_AFFILIATED
12:40837331	*LRRK2_2*	NABA_ECM_AFFILIATED
12:40872410	*LRRK2_2*	NABA_ECM_AFFILIATED
12:40918443	*LRRK2_2*	NABA_ECM_AFFILIATED
3:49162284	*IP6K2*	NABA_ECM_GLYCOPROTEINS
3:49162583	*IP6K2*	NABA_ECM_GLYCOPROTEINS
4:90804532	*SNCA_2*	NABA_ECM_GLYCOPROTEINS
4:90868355	*SNCA_2*	NABA_ECM_GLYCOPROTEINS
6:32031904	*HLA-DRB5*	NABA_ECM_GLYCOPROTEINS
6:32062687	*HLA-DRB5*	NABA_ECM_GLYCOPROTEINS
6:112561154	*FYN*	NABA_ECM_GLYCOPROTEINS
14:75057014	*RPS6KL1*	NABA_ECM_GLYCOPROTEINS
1:155027082	*GBAP1*	NABA_ECM_REGULATORS
1:155033317	*GBAP1*	NABA_ECM_REGULATORS
8:11712443	*CTSB*	NABA_ECM_REGULATORS
8:23190941	*BIN3*	NABA_ECM_REGULATORS
8:23212542	*BIN3*	NABA_ECM_REGULATORS
8:23223713	*BIN3*	NABA_ECM_REGULATORS
9:18650547	*SH3GL2_2*	NABA_ECM_REGULATORS
9:33791164	*UBAP2*	NABA_ECM_REGULATORS
1:155027082	*GBAP1*	NABA_MATRISOME_ASSOCIATED
1:155033317	*GBAP1*	NABA_MATRISOME_ASSOCIATED
3:122645304	*KPNA1*	NABA_MATRISOME_ASSOCIATED
4:15354235	*BST1*	NABA_MATRISOME_ASSOCIATED
6:31002527	*TRIM40*	NABA_MATRISOME_ASSOCIATED
8:11712443	*CTSB*	NABA_MATRISOME_ASSOCIATED
8:23190941	*BIN3*	NABA_MATRISOME_ASSOCIATED
8:23212542	*BIN3*	NABA_MATRISOME_ASSOCIATED
8:23223713	*BIN3*	NABA_MATRISOME_ASSOCIATED
9:18650547	*SH3GL2_2*	NABA_MATRISOME_ASSOCIATED
9:33791164	*UBAP2*	NABA_MATRISOME_ASSOCIATED
12:40798608	*LRRK2_2*	NABA_MATRISOME_ASSOCIATED
12:40800267	*LRRK2_2*	NABA_MATRISOME_ASSOCIATED
12:40837264	*LRRK2_2*	NABA_MATRISOME_ASSOCIATED
12:40837331	*LRRK2_2*	NABA_MATRISOME_ASSOCIATED
12:40872410	*LRRK2_2*	NABA_MATRISOME_ASSOCIATED
12:40918443	*LRRK2_2*	NABA_MATRISOME_ASSOCIATED
17:44843651	*WNT3*	NABA_MATRISOME_ASSOCIATED
17:44888209	*WNT3*	NABA_MATRISOME_ASSOCIATED
17:44888715	*WNT3*	NABA_MATRISOME_ASSOCIATED
17:44906949	*WNT3*	NABA_MATRISOME_ASSOCIATED
17:44912901	*WNT3*	NABA_MATRISOME_ASSOCIATED

17:44931399	*WNT3*	NABA_MATRISOME_ASSOCIATED
17:44947756	*WNT3*	NABA_MATRISOME_ASSOCIATED
19:2251817	*SPPL2B*	NABA_MATRISOME_ASSOCIATED
1:155027082	*GBAP1*	NABA_MATRISOME
1:155033317	*GBAP1*	NABA_MATRISOME
3:49162284	*IP6K2*	NABA_MATRISOME
3:49162583	*IP6K2*	NABA_MATRISOME
3:122645304	*KPNA1*	NABA_MATRISOME
4:15354235	*BST1*	NABA_MATRISOME
4:90804532	*SNCA_2*	NABA_MATRISOME
4:90868355	*SNCA_2*	NABA_MATRISOME
6:31002527	*TRIM40*	NABA_MATRISOME
6:32031904	*HLA-DRB5*	NABA_MATRISOME
6:32062687	*HLA-DRB5*	NABA_MATRISOME
6:33153528	*HLA-DRB5*	NABA_MATRISOME
6:112561154	*FYN*	NABA_MATRISOME
8:11712443	*CTSB*	NABA_MATRISOME
8:23190941	*BIN3*	NABA_MATRISOME
8:23212542	*BIN3*	NABA_MATRISOME
8:23223713	*BIN3*	NABA_MATRISOME
9:18650547	*SH3GL2_2*	NABA_MATRISOME
9:33791164	*UBAP2*	NABA_MATRISOME
12:40798608	*LRRK2_2*	NABA_MATRISOME
12:40800267	*LRRK2_2*	NABA_MATRISOME
12:40837264	*LRRK2_2*	NABA_MATRISOME
12:40837331	*LRRK2_2*	NABA_MATRISOME
12:40872410	*LRRK2_2*	NABA_MATRISOME
12:40918443	*LRRK2_2*	NABA_MATRISOME
14:75057014	*RPS6KL1*	NABA_MATRISOME
17:44843651	*WNT3*	NABA_MATRISOME
17:44888209	*WNT3*	NABA_MATRISOME
17:44888715	*WNT3*	NABA_MATRISOME
17:44906949	*WNT3*	NABA_MATRISOME
17:44912901	*WNT3*	NABA_MATRISOME
17:44931399	*WNT3*	NABA_MATRISOME
17:44947756	*WNT3*	NABA_MATRISOME
19:2251817	*SPPL2B*	NABA_MATRISOME
17:7455536	*V1NB1*	NABA_SECRETED_FACTORS
17:44843651	*WNT3*	NABA_SECRETED_FACTORS
17:44888209	*WNT3*	NABA_SECRETED_FACTORS
17:44888715	*WNT3*	NABA_SECRETED_FACTORS
17:44906949	*WNT3*	NABA_SECRETED_FACTORS
17:44912901	*WNT3*	NABA_SECRETED_FACTORS
17:44931399	*WNT3*	NABA_SECRETED_FACTORS
17:44947756	*WNT3*	NABA_SECRETED_FACTORS
19:2251817	*SPPL2B*	NABA_SECRETED_FACTORS
3:49162284	*IP6K2*	PID_A6B1_A6B4_INTEGRIN_PATHWAY
3:49162583	*IP6K2*	PID_A6B1_A6B4_INTEGRIN_PATHWAY
6:112561154	*FYN*	PID_A6B1_A6B4_INTEGRIN_PATHWAY
6:112009427	*FYN*	PID_AJDISS_2PATHWAY
6:112056759	*FYN*	PID_AJDISS_2PATHWAY
6:112151452	*FYN*	PID_AJDISS_2PATHWAY
17:39883520	*RETREG3*	PID_AJDISS_2PATHWAY

19:2251817	*SPPL2B*	PID_ALK2_PATHWAY
4:90677243	*SNCA_2*	PID_ALPHA_SYNUCLEIN_PATHWAY
4:90700329	*SNCA_2*	PID_ALPHA_SYNUCLEIN_PATHWAY
4:90727315	*SNCA_2*	PID_ALPHA_SYNUCLEIN_PATHWAY
4:90738839	*SNCA_2*	PID_ALPHA_SYNUCLEIN_PATHWAY
4:90757294	*SNCA_2*	PID_ALPHA_SYNUCLEIN_PATHWAY
6:112009427	*FYN*	PID_ALPHA_SYNUCLEIN_PATHWAY
6:112056759	*FYN*	PID_ALPHA_SYNUCLEIN_PATHWAY
6:112151452	*FYN*	PID_ALPHA_SYNUCLEIN_PATHWAY
8:11381148	*CTSB*	PID_ALPHA_SYNUCLEIN_PATHWAY
10:121114292	*INPP5F*	PID_ALPHA_SYNUCLEIN_PATHWAY
10:121138071	*INPP5F*	PID_ALPHA_SYNUCLEIN_PATHWAY
10:121191186	*INPP5F*	PID_ALPHA_SYNUCLEIN_PATHWAY
3:49406095	*IP6K2*	PID_AMB2_NEUTROPHILS_PATHWAY
3:49424231	*IP6K2*	PID_AMB2_NEUTROPHILS_PATHWAY
3:49450137	*IP6K2*	PID_AMB2_NEUTROPHILS_PATHWAY
6:32151443	*HLA-DRB5*	PID_AMB2_NEUTROPHILS_PATHWAY
6:112009427	*FYN*	PID_AMB2_NEUTROPHILS_PATHWAY
6:112056759	*FYN*	PID_AMB2_NEUTROPHILS_PATHWAY
6:112151452	*FYN*	PID_AMB2_NEUTROPHILS_PATHWAY
8:11381148	*CTSB*	PID_AMB2_NEUTROPHILS_PATHWAY
11:133947732	*IGSF9B*	PID_AMB2_NEUTROPHILS_PATHWAY
6:112009427	*FYN*	PID_ANGIOPOIETIN_RECEPTOR_PATHWAY
6:112056759	*FYN*	PID_ANGIOPOIETIN_RECEPTOR_PATHWAY
6:112151452	*FYN*	PID_ANGIOPOIETIN_RECEPTOR_PATHWAY
17:40392358	*RETREG3*	PID_ANGIOPOIETIN_RECEPTOR_PATHWAY
17:7578671	*V1NB1*	PID_AP1_PATHWAY
4:76695927	*FAM47E-STBD1*	PID_ARF_3PATHWAY
8:131153067	*FAM49B*	PID_ARF_3PATHWAY
8:131190319	*FAM49B*	PID_ARF_3PATHWAY
8:131427864	*FAM49B*	PID_ARF_3PATHWAY
10:104005410	*GBF1*	PID_ARF_3PATHWAY
10:104034550	*GBF1*	PID_ARF_3PATHWAY
17:45059032	*WNT3*	PID_ARF_3PATHWAY
17:45089680	*WNT3*	PID_ARF_3PATHWAY
3:49406095	*IP6K2*	PID_ARF6_DOWNSTREAM_PATHWAY
3:49424231	*IP6K2*	PID_ARF6_DOWNSTREAM_PATHWAY
3:49450137	*IP6K2*	PID_ARF6_DOWNSTREAM_PATHWAY
19:3156505	*SPPL2B*	PID_ARF6_PATHWAY
10:15569598	*ITGA8*	PID_ARF6_TRAFFICKING_PATHWAY
10:15652368	*ITGA8*	PID_ARF6_TRAFFICKING_PATHWAY
6:31862876	*HLA-DRB5*	PID_AR_TF_PATHWAY
3:48199695	*IP6K2*	PID_ATM_PATHWAY
3:48214438	*IP6K2*	PID_ATM_PATHWAY
3:48199695	*IP6K2*	PID_ATR_PATHWAY
3:48214438	*IP6K2*	PID_ATR_PATHWAY
17:7578671	*V1NB1*	PID_AURORA_A_PATHWAY
3:49406095	*IP6K2*	PID_AURORA_B_PATHWAY
3:49424231	*IP6K2*	PID_AURORA_B_PATHWAY
3:49450137	*IP6K2*	PID_AURORA_B_PATHWAY
3:49406095	*IP6K2*	PID_AVB3_INTEGRIN_PATHWAY
3:49424231	*IP6K2*	PID_AVB3_INTEGRIN_PATHWAY
3:49450137	*IP6K2*	PID_AVB3_INTEGRIN_PATHWAY

6:33153528	*HLA-DRB5*	PID_AVB3_INTEGRIN_PATHWAY
3:49406095	*IP6K2*	PID_AVB3_OPN_PATHWAY
3:49424231	*IP6K2*	PID_AVB3_OPN_PATHWAY
3:49450137	*IP6K2*	PID_AVB3_OPN_PATHWAY
17:43390763	*CRHR1_2*	PID_AVB3_OPN_PATHWAY
17:7578671	*V1NB1*	PID_BARD1_PATHWAY
17:7129974	*V1NB1*	PID_BETA_CATENIN_DEG_PATHWAY
19:3022354	*SPPL2B*	PID_BETA_CATENIN_NUC_PATHWAY
19:3036350	*SPPL2B*	PID_BETA_CATENIN_NUC_PATHWAY
1:156059276	*GBAP1*	PID_CASPASE_PATHWAY
17:43390763	*CRHR1_2*	PID_CD40_PATHWAY
6:112009427	*FYN*	PID_CD8_TCR_PATHWAY
6:112056759	*FYN*	PID_CD8_TCR_PATHWAY
6:112151452	*FYN*	PID_CD8_TCR_PATHWAY
17:43390763	*CRHR1_2*	PID_CD8_TCR_PATHWAY
1:227182033	*ITPKB*	PID_CDC42_PATHWAY
1:227270318	*ITPKB*	PID_CDC42_PATHWAY
2:102357447	*MAP4K4*	PID_CERAMIDE_PATHWAY
2:102413116	*MAP4K4*	PID_CERAMIDE_PATHWAY
2:101439596	*MAP4K4*	PID_CIRCADIAN_PATHWAY
2:101550181	*MAP4K4*	PID_CIRCADIAN_PATHWAY
2:101603812	*MAP4K4*	PID_CIRCADIAN_PATHWAY
16:30510571	*SETD1A*	PID_CXCR3_PATHWAY
16:30516492	*SETD1A*	PID_CXCR3_PATHWAY
16:30520856	*SETD1A*	PID_CXCR3_PATHWAY
3:49406095	*IP6K2*	PID_CXCR4_PATHWAY
3:49424231	*IP6K2*	PID_CXCR4_PATHWAY
3:49450137	*IP6K2*	PID_CXCR4_PATHWAY
6:112009427	*FYN*	PID_CXCR4_PATHWAY
6:112056759	*FYN*	PID_CXCR4_PATHWAY
6:112151452	*FYN*	PID_CXCR4_PATHWAY
8:11381148	*CTSB*	PID_CXCR4_PATHWAY
10:15569598	*ITGA8*	PID_CXCR4_PATHWAY
10:15652368	*ITGA8*	PID_CXCR4_PATHWAY
17:40392358	*RETREG3*	PID_CXCR4_PATHWAY
3:48199695	*IP6K2*	PID_E2F_PATHWAY
3:48214438	*IP6K2*	PID_E2F_PATHWAY
17:76177567	*DNAH17*	PID_E2F_PATHWAY
3:49406095	*IP6K2*	PID_ECADHERIN_KERATINOCYTE_PATHWAY
3:49424231	*IP6K2*	PID_ECADHERIN_KERATINOCYTE_PATHWAY
3:49450137	*IP6K2*	PID_ECADHERIN_KERATINOCYTE_PATHWAY
6:112009427	*FYN*	PID_ECADHERIN_KERATINOCYTE_PATHWAY
6:112056759	*FYN*	PID_ECADHERIN_KERATINOCYTE_PATHWAY
6:112151452	*FYN*	PID_ECADHERIN_KERATINOCYTE_PATHWAY
17:39883520	*RETREG3*	PID_ECADHERIN_KERATINOCYTE_PATHWAY
3:49406095	*IP6K2*	PID_ECADHERIN_NASCENT_AJ_PATHWAY
3:49424231	*IP6K2*	PID_ECADHERIN_NASCENT_AJ_PATHWAY
3:49450137	*IP6K2*	PID_ECADHERIN_NASCENT_AJ_PATHWAY
17:39883520	*RETREG3*	PID_ECADHERIN_NASCENT_AJ_PATHWAY
3:49406095	*IP6K2*	PID_ECADHERIN_STABILIZATION_PATHWAY
3:49424231	*IP6K2*	PID_ECADHERIN_STABILIZATION_PATHWAY
3:49450137	*IP6K2*	PID_ECADHERIN_STABILIZATION_PATHWAY
3:49406095	*IP6K2*	PID_ENDOTHELIN_PATHWAY

3:49424231	*IP6K2*	PID_ENDOTHELIN_PATHWAY
3:49450137	*IP6K2*	PID_ENDOTHELIN_PATHWAY
3:123057397	*KPNA1*	PID_ENDOTHELIN_PATHWAY
19:3156505	*SPPL2B*	PID_ENDOTHELIN_PATHWAY
3:49406095	*IP6K2*	PID_EPHA2_FWD_PATHWAY
3:49424231	*IP6K2*	PID_EPHA2_FWD_PATHWAY
3:49450137	*IP6K2*	PID_EPHA2_FWD_PATHWAY
3:49406095	*IP6K2*	PID_EPHA_FWDPATHWAY
3:49424231	*IP6K2*	PID_EPHA_FWDPATHWAY
3:49450137	*IP6K2*	PID_EPHA_FWDPATHWAY
6:112009427	*FYN*	PID_EPHA_FWDPATHWAY
6:112056759	*FYN*	PID_EPHA_FWDPATHWAY
6:112151452	*FYN*	PID_EPHA_FWDPATHWAY
8:11381148	*CTSB*	PID_EPHA_FWDPATHWAY
2:102357447	*MAP4K4*	PID_EPHB_FWD_PATHWAY
2:102413116	*MAP4K4*	PID_EPHB_FWD_PATHWAY
6:112009427	*FYN*	PID_EPHRINB_REV_PATHWAY
6:112056759	*FYN*	PID_EPHRINB_REV_PATHWAY
6:112151452	*FYN*	PID_EPHRINB_REV_PATHWAY
8:11381148	*CTSB*	PID_EPHRINB_REV_PATHWAY
17:40392358	*RETREG3*	PID_EPO_PATHWAY
9:17579690	*SH3GL2_2*	PID_ERBB1_INTERNALIZATION_PATHWAY
9:17609302	*SH3GL2_2*	PID_ERBB1_INTERNALIZATION_PATHWAY
9:17627444	*SH3GL2_2*	PID_ERBB1_INTERNALIZATION_PATHWAY
9:17710876	*SH3GL2_2*	PID_ERBB1_INTERNALIZATION_PATHWAY
9:17732590	*SH3GL2_2*	PID_ERBB1_INTERNALIZATION_PATHWAY
9:17737514	*SH3GL2_2*	PID_ERBB1_INTERNALIZATION_PATHWAY
9:17758945	*SH3GL2_2*	PID_ERBB1_INTERNALIZATION_PATHWAY
6:112009427	*FYN*	PID_ERBB4_PATHWAY
6:112056759	*FYN*	PID_ERBB4_PATHWAY
6:112151452	*FYN*	PID_ERBB4_PATHWAY
17:7101771	*V1NB1*	PID_ERBB4_PATHWAY
17:40392358	*RETREG3*	PID_ERBB4_PATHWAY
3:49406095	*IP6K2*	PID_ER_NONGENOMIC_PATHWAY
3:49424231	*IP6K2*	PID_ER_NONGENOMIC_PATHWAY
3:49450137	*IP6K2*	PID_ER_NONGENOMIC_PATHWAY
19:3156505	*SPPL2B*	PID_ER_NONGENOMIC_PATHWAY
3:49406095	*IP6K2*	PID_FAK_PATHWAY
3:49424231	*IP6K2*	PID_FAK_PATHWAY
3:49450137	*IP6K2*	PID_FAK_PATHWAY
6:112009427	*FYN*	PID_FAK_PATHWAY
6:112056759	*FYN*	PID_FAK_PATHWAY
6:112151452	*FYN*	PID_FAK_PATHWAY
8:131153067	*FAM49B*	PID_FAK_PATHWAY
8:131190319	*FAM49B*	PID_FAK_PATHWAY
8:131427864	*FAM49B*	PID_FAK_PATHWAY
17:59922925	*BRIP1*	PID_FANCONI_PATHWAY
6:112009427	*FYN*	PID_FCER1_PATHWAY
6:112056759	*FYN*	PID_FCER1_PATHWAY
6:112151452	*FYN*	PID_FCER1_PATHWAY
17:40392358	*RETREG3*	PID_FGF_PATHWAY
6:112561154	*FYN*	PID_FOXM1_PATHWAY
17:41056283	*RETREG3*	PID_FOXO_PATHWAY

6:112009427	*FYN*	PID_GLYPICAN_1PATHWAY
6:112056759	*FYN*	PID_GLYPICAN_1PATHWAY
6:112151452	*FYN*	PID_GLYPICAN_1PATHWAY
8:11381148	*CTSB*	PID_GLYPICAN_1PATHWAY
17:40392358	*RETREG3*	PID_GMCSF_PATHWAY
17:7578671	*V1NB1*	PID_HDAC_CLASSIII_PATHWAY
2:169999639	*STK39*	PID_HEDGEHOG_2PATHWAY
2:170028024	*STK39*	PID_HEDGEHOG_2PATHWAY
10:104285824	*GBF1*	PID_HEDGEHOG_GLI_PATHWAY
4:114424939	*CAMK2D*	PID_HES_HEY_PATHWAY
17:7578671	*V1NB1*	PID_HIF1A_PATHWAY
15:61293467	*VPS13C*	PID_HIF1_TFPATHWAY
17:43390763	*CRHR1_2*	PID_HIV_NEF_PATHWAY
14:38061742	*MIPOL1*	PID_HNF3A_PATHWAY
14:38061742	*MIPOL1*	PID_HNF3B_PATHWAY
17:41056283	*RETREG3*	PID_HNF3B_PATHWAY
4:114424939	*CAMK2D*	PID_IFNG_PATHWAY
2:103063855	*MAP4K4*	PID_IL12_2PATHWAY
2:103063855	*MAP4K4*	PID_IL12_STAT4_PATHWAY
2:102609356	*MAP4K4*	PID_IL1_PATHWAY
2:102636759	*MAP4K4*	PID_IL1_PATHWAY
3:49406095	*IP6K2*	PID_IL2_1PATHWAY
3:49424231	*IP6K2*	PID_IL2_1PATHWAY
3:49450137	*IP6K2*	PID_IL2_1PATHWAY
6:112009427	*FYN*	PID_IL2_1PATHWAY
6:112056759	*FYN*	PID_IL2_1PATHWAY
6:112151452	*FYN*	PID_IL2_1PATHWAY
17:40392358	*RETREG3*	PID_IL2_1PATHWAY
2:103063855	*MAP4K4*	PID_IL23_PATHWAY
17:7983799	*V1NB1*	PID_IL23_PATHWAY
17:40392358	*RETREG3*	PID_IL2_STAT5_PATHWAY
17:40392358	*RETREG3*	PID_IL3_PATHWAY
17:40392358	*RETREG3*	PID_IL4_2PATHWAY
17:40392358	*RETREG3*	PID_IL5_PATHWAY
19:3156505	*SPPL2B*	PID_IL8_CXCR1_PATHWAY
19:3156505	*SPPL2B*	PID_IL8_CXCR2_PATHWAY
3:49162284	*IP6K2*	PID_INTEGRIN1_PATHWAY
3:49162583	*IP6K2*	PID_INTEGRIN1_PATHWAY
6:33153528	*HLA-DRB5*	PID_INTEGRIN1_PATHWAY
6:112561154	*FYN*	PID_INTEGRIN1_PATHWAY
10:15569598	*ITGA8*	PID_INTEGRIN1_PATHWAY
10:15652368	*ITGA8*	PID_INTEGRIN1_PATHWAY
11:133947732	*IGSF9B*	PID_INTEGRIN2_PATHWAY
16:30510571	*SETD1A*	PID_INTEGRIN2_PATHWAY
16:30516492	*SETD1A*	PID_INTEGRIN2_PATHWAY
16:30520856	*SETD1A*	PID_INTEGRIN2_PATHWAY
16:31370452	*SETD1A*	PID_INTEGRIN2_PATHWAY
6:112561154	*FYN*	PID_INTEGRIN3_PATHWAY
3:49162284	*IP6K2*	PID_INTEGRIN4_PATHWAY
3:49162583	*IP6K2*	PID_INTEGRIN4_PATHWAY
1:155027082	*GBAP1*	PID_INTEGRIN_A9B1_PATHWAY
1:155033317	*GBAP1*	PID_INTEGRIN_A9B1_PATHWAY
10:15569598	*ITGA8*	PID_INTEGRIN_CS_PATHWAY

10:15652368	*ITGA8*	PID_INTEGRIN_CS_PATHWAY
16:30510571	*SETD1A*	PID_INTEGRIN_CS_PATHWAY
16:30516492	*SETD1A*	PID_INTEGRIN_CS_PATHWAY
16:30520856	*SETD1A*	PID_INTEGRIN_CS_PATHWAY
16:31370452	*SETD1A*	PID_INTEGRIN_CS_PATHWAY
3:49406095	*IP6K2*	PID_LIS1_PATHWAY
3:49424231	*IP6K2*	PID_LIS1_PATHWAY
3:49450137	*IP6K2*	PID_LIS1_PATHWAY
12:122816734	*HIP1R*	PID_LIS1_PATHWAY
12:122817580	*HIP1R*	PID_LIS1_PATHWAY
12:122853197	*HIP1R*	PID_LIS1_PATHWAY
1:227067656	*ITPKB*	PID_LKB1_PATHWAY
17:7578671	*V1NB1*	PID_LKB1_PATHWAY
17:43999203	*WNT3*	PID_LKB1_PATHWAY
17:44005297	*WNT3*	PID_LKB1_PATHWAY
17:44019712	*WNT3*	PID_LKB1_PATHWAY
17:44060492	*WNT3*	PID_LKB1_PATHWAY
17:44065263	*WNT3*	PID_LKB1_PATHWAY
17:44081178	*WNT3*	PID_LKB1_PATHWAY
17:44083297	*WNT3*	PID_LKB1_PATHWAY
3:123057397	*KPNA1*	PID_LPA4_PATHWAY
3:49406095	*IP6K2*	PID_LYSOPHOSPHOLIPID_PATHWAY
3:49424231	*IP6K2*	PID_LYSOPHOSPHOLIPID_PATHWAY
3:49450137	*IP6K2*	PID_LYSOPHOSPHOLIPID_PATHWAY
3:123057397	*KPNA1*	PID_LYSOPHOSPHOLIPID_PATHWAY
17:43999203	*WNT3*	PID_LYSOPHOSPHOLIPID_PATHWAY
17:44005297	*WNT3*	PID_LYSOPHOSPHOLIPID_PATHWAY
17:44019712	*WNT3*	PID_LYSOPHOSPHOLIPID_PATHWAY
17:44060492	*WNT3*	PID_LYSOPHOSPHOLIPID_PATHWAY
17:44065263	*WNT3*	PID_LYSOPHOSPHOLIPID_PATHWAY
17:44081178	*WNT3*	PID_LYSOPHOSPHOLIPID_PATHWAY
17:44083297	*WNT3*	PID_LYSOPHOSPHOLIPID_PATHWAY
19:3156505	*SPPL2B*	PID_LYSOPHOSPHOLIPID_PATHWAY
18:40543738	*RIT2*	PID_MAPK_TRK_PATHWAY
18:40596271	*RIT2*	PID_MAPK_TRK_PATHWAY
18:40612823	*RIT2*	PID_MAPK_TRK_PATHWAY
18:40649778	*RIT2*	PID_MAPK_TRK_PATHWAY
18:40673380	*RIT2*	PID_MAPK_TRK_PATHWAY
3:49406095	*IP6K2*	PID_MET_PATHWAY
3:49424231	*IP6K2*	PID_MET_PATHWAY
3:49450137	*IP6K2*	PID_MET_PATHWAY
9:17579690	*SH3GL2_2*	PID_MET_PATHWAY
9:17609302	*SH3GL2_2*	PID_MET_PATHWAY
9:17627444	*SH3GL2_2*	PID_MET_PATHWAY
9:17710876	*SH3GL2_2*	PID_MET_PATHWAY
9:17732590	*SH3GL2_2*	PID_MET_PATHWAY
9:17737514	*SH3GL2_2*	PID_MET_PATHWAY
9:17758945	*SH3GL2_2*	PID_MET_PATHWAY
3:49406095	*IP6K2*	PID_MTOR_4PATHWAY
3:49424231	*IP6K2*	PID_MTOR_4PATHWAY
3:49450137	*IP6K2*	PID_MTOR_4PATHWAY
12:122816734	*HIP1R*	PID_MTOR_4PATHWAY
12:122817580	*HIP1R*	PID_MTOR_4PATHWAY

12:122853197	*HIP1R*	PID_MTOR_4PATHWAY
17:7479140	*V1NB1*	PID_MTOR_4PATHWAY
17:7480325	*V1NB1*	PID_MTOR_4PATHWAY
3:48199695	*IP6K2*	PID_MYC_ACTIV_PATHWAY
3:48214438	*IP6K2*	PID_MYC_ACTIV_PATHWAY
5:60273923	*ELOVL7*	PID_MYC_ACTIV_PATHWAY
17:7479140	*V1NB1*	PID_MYC_ACTIV_PATHWAY
17:7480325	*V1NB1*	PID_MYC_ACTIV_PATHWAY
17:7578671	*V1NB1*	PID_MYC_ACTIV_PATHWAY
17:76177567	*DNAH17*	PID_MYC_ACTIV_PATHWAY
3:49406095	*IP6K2*	PID_NCADHERIN_PATHWAY
3:49424231	*IP6K2*	PID_NCADHERIN_PATHWAY
3:49450137	*IP6K2*	PID_NCADHERIN_PATHWAY
17:39883520	*RETREG3*	PID_NCADHERIN_PATHWAY
6:112009427	*FYN*	PID_NEPHRIN_NEPH1_PATHWAY
6:112056759	*FYN*	PID_NEPHRIN_NEPH1_PATHWAY
6:112151452	*FYN*	PID_NEPHRIN_NEPH1_PATHWAY
3:49406095	*IP6K2*	PID_NETRIN_PATHWAY
3:49424231	*IP6K2*	PID_NETRIN_PATHWAY
3:49450137	*IP6K2*	PID_NETRIN_PATHWAY
6:112009427	*FYN*	PID_NETRIN_PATHWAY
6:112056759	*FYN*	PID_NETRIN_PATHWAY
6:112151452	*FYN*	PID_NETRIN_PATHWAY
16:50730088	*CASC16*	PID_NFKAPPAB_CANONICAL_PATHWAY
16:50730446	*CASC16*	PID_NFKAPPAB_CANONICAL_PATHWAY
16:50745926	*CASC16*	PID_NFKAPPAB_CANONICAL_PATHWAY
6:32186193	*HLA-DRB5*	PID_NOTCH_PATHWAY
12:41147295	*LRRK2_2*	PID_NOTCH_PATHWAY
12:41251554	*LRRK2_2*	PID_NOTCH_PATHWAY
12:41407403	*LRRK2_2*	PID_NOTCH_PATHWAY
1:161875645	*FCGR2A*	PID_P38_ALPHA_BETA_DOWNSTREAM_PATHWAY
1:161930954	*FCGR2A*	PID_P38_ALPHA_BETA_DOWNSTREAM_PATHWAY
17:7578671	*V1NB1*	PID_P38_ALPHA_BETA_DOWNSTREAM_PATHWAY
6:112009427	*FYN*	PID_P38_ALPHA_BETA_PATHWAY
6:112056759	*FYN*	PID_P38_ALPHA_BETA_PATHWAY
6:112151452	*FYN*	PID_P38_ALPHA_BETA_PATHWAY
8:11381148	*CTSB*	PID_P38_ALPHA_BETA_PATHWAY
17:43999203	*WNT3*	PID_P38_GAMMA_DELTA_PATHWAY
17:44005297	*WNT3*	PID_P38_GAMMA_DELTA_PATHWAY
17:44019712	*WNT3*	PID_P38_GAMMA_DELTA_PATHWAY
17:44060492	*WNT3*	PID_P38_GAMMA_DELTA_PATHWAY
17:44065263	*WNT3*	PID_P38_GAMMA_DELTA_PATHWAY
17:44081178	*WNT3*	PID_P38_GAMMA_DELTA_PATHWAY
17:44083297	*WNT3*	PID_P38_GAMMA_DELTA_PATHWAY
2:102357447	*MAP4K4*	PID_P53_DOWNSTREAM_PATHWAY
2:102413116	*MAP4K4*	PID_P53_DOWNSTREAM_PATHWAY
6:32814942	*HLA-DRB5*	PID_P53_DOWNSTREAM_PATHWAY
8:23050400	*BIN3*	PID_P53_DOWNSTREAM_PATHWAY
14:38061742	*MIPOL1*	PID_P53_DOWNSTREAM_PATHWAY
17:7578671	*V1NB1*	PID_P53_DOWNSTREAM_PATHWAY
17:7578671	*V1NB1*	PID_P53_REGULATION_PATHWAY
1:161171736	*FCGR2A*	PID_P73PATHWAY
3:49406095	*IP6K2*	PID_P75_NTR_PATHWAY

3:49424231	*IP6K2*	PID_P75_NTR_PATHWAY
3:49450137	*IP6K2*	PID_P75_NTR_PATHWAY
17:7578671	*V1NB1*	PID_P75_NTR_PATHWAY
3:49406095	*IP6K2*	PID_PDGFRB_PATHWAY
3:49424231	*IP6K2*	PID_PDGFRB_PATHWAY
3:49450137	*IP6K2*	PID_PDGFRB_PATHWAY
6:112009427	*FYN*	PID_PDGFRB_PATHWAY
6:112056759	*FYN*	PID_PDGFRB_PATHWAY
6:112151452	*FYN*	PID_PDGFRB_PATHWAY
8:11381148	*CTSB*	PID_PDGFRB_PATHWAY
17:40392358	*RETREG3*	PID_PDGFRB_PATHWAY
3:49406095	*IP6K2*	PID_PI3KCI_PATHWAY
3:49424231	*IP6K2*	PID_PI3KCI_PATHWAY
3:49450137	*IP6K2*	PID_PI3KCI_PATHWAY
6:112009427	*FYN*	PID_PI3KCI_PATHWAY
6:112056759	*FYN*	PID_PI3KCI_PATHWAY
6:112151452	*FYN*	PID_PI3KCI_PATHWAY
8:11381148	*CTSB*	PID_PI3KCI_PATHWAY
17:76727455	*DNAH17*	PID_PI3KCI_PATHWAY
3:49406095	*IP6K2*	PID_PLK1_PATHWAY
3:49424231	*IP6K2*	PID_PLK1_PATHWAY
3:49450137	*IP6K2*	PID_PLK1_PATHWAY
3:49406095	*IP6K2*	PID_PRL_SIGNALING_EVENTS_PATHWAY
3:49424231	*IP6K2*	PID_PRL_SIGNALING_EVENTS_PATHWAY
3:49450137	*IP6K2*	PID_PRL_SIGNALING_EVENTS_PATHWAY
3:49406095	*IP6K2*	PID_PTP1B_PATHWAY
3:49424231	*IP6K2*	PID_PTP1B_PATHWAY
3:49450137	*IP6K2*	PID_PTP1B_PATHWAY
6:112009427	*FYN*	PID_PTP1B_PATHWAY
6:112056759	*FYN*	PID_PTP1B_PATHWAY
6:112151452	*FYN*	PID_PTP1B_PATHWAY
8:11381148	*CTSB*	PID_PTP1B_PATHWAY
17:40392358	*RETREG3*	PID_PTP1B_PATHWAY
1:155975202	*GBAP1*	PID_RAC1_REG_PATHWAY
6:32941506	*HLA-DRB5*	PID_RB_1PATHWAY
1:155975202	*GBAP1*	PID_REELIN_PATHWAY
6:112009427	*FYN*	PID_REELIN_PATHWAY
6:112056759	*FYN*	PID_REELIN_PATHWAY
6:112151452	*FYN*	PID_REELIN_PATHWAY
17:43999203	*WNT3*	PID_REELIN_PATHWAY
17:44005297	*WNT3*	PID_REELIN_PATHWAY
17:44019712	*WNT3*	PID_REELIN_PATHWAY
17:44060492	*WNT3*	PID_REELIN_PATHWAY
17:44065263	*WNT3*	PID_REELIN_PATHWAY
17:44081178	*WNT3*	PID_REELIN_PATHWAY
17:44083297	*WNT3*	PID_REELIN_PATHWAY
17:7578671	*V1NB1*	PID_REG_GR_PATHWAY
17:40392358	*RETREG3*	PID_REG_GR_PATHWAY
3:49406095	*IP6K2*	PID_RET_PATHWAY
3:49424231	*IP6K2*	PID_RET_PATHWAY
3:49450137	*IP6K2*	PID_RET_PATHWAY
3:49406095	*IP6K2*	PID_RHOA_PATHWAY
3:49424231	*IP6K2*	PID_RHOA_PATHWAY

3:49450137	*IP6K2*	PID_RHOA_PATHWAY
9:17579690	*SH3GL2_2*	PID_RHOA_PATHWAY
9:17609302	*SH3GL2_2*	PID_RHOA_PATHWAY
9:17627444	*SH3GL2_2*	PID_RHOA_PATHWAY
9:17710876	*SH3GL2_2*	PID_RHOA_PATHWAY
9:17732590	*SH3GL2_2*	PID_RHOA_PATHWAY
9:17737514	*SH3GL2_2*	PID_RHOA_PATHWAY
9:17758945	*SH3GL2_2*	PID_RHOA_PATHWAY
1:155975202	*GBAP1*	PID_RHOA_REG_PATHWAY
3:49406095	*IP6K2*	PID_RHOA_REG_PATHWAY
3:49424231	*IP6K2*	PID_RHOA_REG_PATHWAY
3:49450137	*IP6K2*	PID_RHOA_REG_PATHWAY
19:3156505	*SPPL2B*	PID_S1P_META_PATHWAY
3:49406095	*IP6K2*	PID_S1P_S1P1_PATHWAY
3:49424231	*IP6K2*	PID_S1P_S1P1_PATHWAY
3:49450137	*IP6K2*	PID_S1P_S1P1_PATHWAY
3:49406095	*IP6K2*	PID_S1P_S1P2_PATHWAY
3:49424231	*IP6K2*	PID_S1P_S1P2_PATHWAY
3:49450137	*IP6K2*	PID_S1P_S1P2_PATHWAY
19:3156505	*SPPL2B*	PID_S1P_S1P2_PATHWAY
3:49406095	*IP6K2*	PID_S1P_S1P3_PATHWAY
3:49424231	*IP6K2*	PID_S1P_S1P3_PATHWAY
3:49450137	*IP6K2*	PID_S1P_S1P3_PATHWAY
19:3156505	*SPPL2B*	PID_S1P_S1P3_PATHWAY
3:49406095	*IP6K2*	PID_S1P_S1P4_PATHWAY
3:49424231	*IP6K2*	PID_S1P_S1P4_PATHWAY
3:49450137	*IP6K2*	PID_S1P_S1P4_PATHWAY
3:49406095	*IP6K2*	PID_SHP2_PATHWAY
3:49424231	*IP6K2*	PID_SHP2_PATHWAY
3:49450137	*IP6K2*	PID_SHP2_PATHWAY
6:33153528	*HLA-DRB5*	PID_SYNDECAN_1_PATHWAY
3:49406095	*IP6K2*	PID_SYNDECAN_2_PATHWAY
3:49424231	*IP6K2*	PID_SYNDECAN_2_PATHWAY
3:49450137	*IP6K2*	PID_SYNDECAN_2_PATHWAY
6:112009427	*FYN*	PID_SYNDECAN_3_PATHWAY
6:112056759	*FYN*	PID_SYNDECAN_3_PATHWAY
6:112151452	*FYN*	PID_SYNDECAN_3_PATHWAY
3:49406095	*IP6K2*	PID_SYNDECAN_4_PATHWAY
3:49424231	*IP6K2*	PID_SYNDECAN_4_PATHWAY
3:49450137	*IP6K2*	PID_SYNDECAN_4_PATHWAY
17:40392358	*RETREG3*	PID_TCPTP_PATHWAY
2:168931636	*STK39*	PID_TCR_PATHWAY
2:168979290	*STK39*	PID_TCR_PATHWAY
2:169016304	*STK39*	PID_TCR_PATHWAY
2:169023263	*STK39*	PID_TCR_PATHWAY
6:112009427	*FYN*	PID_TCR_PATHWAY
6:112056759	*FYN*	PID_TCR_PATHWAY
6:112151452	*FYN*	PID_TCR_PATHWAY
17:43390763	*CRHR1_2*	PID_TCR_PATHWAY
3:49406095	*IP6K2*	PID_TGFBR_PATHWAY
3:49424231	*IP6K2*	PID_TGFBR_PATHWAY
3:49450137	*IP6K2*	PID_TGFBR_PATHWAY
3:150298317	*MED12L*	PID_TGFBR_PATHWAY

3:49406095	*IP6K2*	PID_THROMBIN_PAR1_PATHWAY
3:49424231	*IP6K2*	PID_THROMBIN_PAR1_PATHWAY
3:49450137	*IP6K2*	PID_THROMBIN_PAR1_PATHWAY
19:3156505	*SPPL2B*	PID_THROMBIN_PAR1_PATHWAY
3:49406095	*IP6K2*	PID_THROMBIN_PAR4_PATHWAY
3:49424231	*IP6K2*	PID_THROMBIN_PAR4_PATHWAY
3:49450137	*IP6K2*	PID_THROMBIN_PAR4_PATHWAY
19:3156505	*SPPL2B*	PID_THROMBIN_PAR4_PATHWAY
2:102357447	*MAP4K4*	PID_TNF_PATHWAY
2:102413116	*MAP4K4*	PID_TNF_PATHWAY
3:49406095	*IP6K2*	PID_TOLL_ENDOGENOUS_PATHWAY
3:49424231	*IP6K2*	PID_TOLL_ENDOGENOUS_PATHWAY
3:49450137	*IP6K2*	PID_TOLL_ENDOGENOUS_PATHWAY
1:155698425	*GBAP1*	PID_TRAIL_PATHWAY
8:23050400	*BIN3*	PID_TRAIL_PATHWAY
3:49406095	*IP6K2*	PID_TRKR_PATHWAY
3:49424231	*IP6K2*	PID_TRKR_PATHWAY
3:49450137	*IP6K2*	PID_TRKR_PATHWAY
18:40543738	*RIT2*	PID_TRKR_PATHWAY
18:40596271	*RIT2*	PID_TRKR_PATHWAY
18:40612823	*RIT2*	PID_TRKR_PATHWAY
18:40649778	*RIT2*	PID_TRKR_PATHWAY
18:40673380	*RIT2*	PID_TRKR_PATHWAY
3:49406095	*IP6K2*	PID_TXA2PATHWAY
3:49424231	*IP6K2*	PID_TXA2PATHWAY
3:49450137	*IP6K2*	PID_TXA2PATHWAY
6:112009427	*FYN*	PID_TXA2PATHWAY
6:112056759	*FYN*	PID_TXA2PATHWAY
6:112151452	*FYN*	PID_TXA2PATHWAY
8:11381148	*CTSB*	PID_TXA2PATHWAY
19:3156505	*SPPL2B*	PID_TXA2PATHWAY
3:49406095	*IP6K2*	PID_VEGFR1_2_PATHWAY
3:49424231	*IP6K2*	PID_VEGFR1_2_PATHWAY
3:49450137	*IP6K2*	PID_VEGFR1_2_PATHWAY
6:112009427	*FYN*	PID_VEGFR1_2_PATHWAY
6:112056759	*FYN*	PID_VEGFR1_2_PATHWAY
6:112151452	*FYN*	PID_VEGFR1_2_PATHWAY
17:7129974	*V1NB1*	PID_WNT_CANONICAL_PATHWAY
3:49406095	*IP6K2*	PID_WNT_NONCANONICAL_PATHWAY
3:49424231	*IP6K2*	PID_WNT_NONCANONICAL_PATHWAY
3:49450137	*IP6K2*	PID_WNT_NONCANONICAL_PATHWAY
17:7129974	*V1NB1*	PID_WNT_NONCANONICAL_PATHWAY
17:44843651	*WNT3*	PID_WNT_SIGNALING_PATHWAY
17:44888209	*WNT3*	PID_WNT_SIGNALING_PATHWAY
17:44888715	*WNT3*	PID_WNT_SIGNALING_PATHWAY
17:44906949	*WNT3*	PID_WNT_SIGNALING_PATHWAY
10:104847713	*GBF1*	REACTOME_ABACAVIR_METABOLISM
10:104861736	*GBF1*	REACTOME_ABACAVIR_METABOLISM
10:104926485	*GBF1*	REACTOME_ABACAVIR_METABOLISM
10:104929716	*GBF1*	REACTOME_ABACAVIR_METABOLISM
10:104847713	*GBF1*	REACTOME_ABACAVIR_TRANSPORT_AND_METABOLISM
10:104861736	*GBF1*	REACTOME_ABACAVIR_TRANSPORT_AND_METABOLISM
10:104926485	*GBF1*	REACTOME_ABACAVIR_TRANSPORT_AND_METABOLISM

10:104929716	*GBF1*	REACTOME_ABACAVIR_TRANSPORT_AND_METABOLISM
6:32151443	*HLA-DRB5*	REACTOME_ABC_FAMILY_PROTEINS_MEDIATED_TRANSPORT
6:32814942	*HLA-DRB5*	REACTOME_ABC_FAMILY_PROTEINS_MEDIATED_TRANSPORT
12:39960237	*LRRK2_2*	REACTOME_ABC_FAMILY_PROTEINS_MEDIATED_TRANSPORT
12:123453034	*HIP1R*	REACTOME_ABC_FAMILY_PROTEINS_MEDIATED_TRANSPORT
6:32151443	*HLA-DRB5*	REACTOME_ABC_TRANSPORTER_DISORDERS
6:32814942	*HLA-DRB5*	REACTOME_ABC_TRANSPORTER_DISORDERS
12:39960237	*LRRK2_2*	REACTOME_ABC_TRANSPORTERS_IN_LIPID_HOMEOSTASIS
6:31925679	*HLA-DRB5*	REACTOME_ABORTIVE_ELONGATION_OF_HIV_1_TRANSCRIPT_IN_THE_ABSENCE_OF_TAT
19:3156505	*SPPL2B*	REACTOME_ACETYLCHOLINE_REGULATES_INSULIN_SECRETION
1:227067656	*ITPKB*	REACTOME_ACTIVATED_NOTCH1_TRANSMITS_SIGNAL_TO_THE_NUCLEUS
12:41147295	*LRRK2_2*	REACTOME_ACTIVATED_NOTCH1_TRANSMITS_SIGNAL_TO_THE_NUCLEUS
12:41251554	*LRRK2_2*	REACTOME_ACTIVATED_NOTCH1_TRANSMITS_SIGNAL_TO_THE_NUCLEUS
12:41407403	*LRRK2_2*	REACTOME_ACTIVATED_NOTCH1_TRANSMITS_SIGNAL_TO_THE_NUCLEUS
6:112009427	*FYN*	REACTOME_ACTIVATED_NTRK2_SIGNALS_THROUGH_FYN
6:112056759	*FYN*	REACTOME_ACTIVATED_NTRK2_SIGNALS_THROUGH_FYN
6:112151452	*FYN*	REACTOME_ACTIVATED_NTRK2_SIGNALS_THROUGH_FYN
16:50730088	*CASC16*	REACTOME_ACTIVATED_TAK1_MEDIATES_P38_MAPK_ACTIVATION
16:50730446	*CASC16*	REACTOME_ACTIVATED_TAK1_MEDIATES_P38_MAPK_ACTIVATION
16:50745926	*CASC16*	REACTOME_ACTIVATED_TAK1_MEDIATES_P38_MAPK_ACTIVATION
17:43999203	*WNT3*	REACTOME_ACTIVATION_OF_AMPK_DOWNSTREAM_OF_NMDARS
17:44005297	*WNT3*	REACTOME_ACTIVATION_OF_AMPK_DOWNSTREAM_OF_NMDARS
17:44019712	*WNT3*	REACTOME_ACTIVATION_OF_AMPK_DOWNSTREAM_OF_NMDARS
17:44060492	*WNT3*	REACTOME_ACTIVATION_OF_AMPK_DOWNSTREAM_OF_NMDARS
17:44065263	*WNT3*	REACTOME_ACTIVATION_OF_AMPK_DOWNSTREAM_OF_NMDARS
17:44081178	*WNT3*	REACTOME_ACTIVATION_OF_AMPK_DOWNSTREAM_OF_NMDARS
17:44083297	*WNT3*	REACTOME_ACTIVATION_OF_AMPK_DOWNSTREAM_OF_NMDARS
6:32814942	*HLA-DRB5*	REACTOME_ACTIVATION_OF_APC_C_AND_APC_C:CDC20_MEDIATED_DEGRADATION_OF_MITOTIC_PROTEINS
17:45215865	*WNT3*	REACTOME_ACTIVATION_OF_APC_C_AND_APC_C:CDC20_MEDIATED_DEGRADATION_OF_MITOTIC_PROTEINS
17:45244822	*WNT3*	REACTOME_ACTIVATION_OF_APC_C_AND_APC_C:CDC20_MEDIATED_DEGRADATION_OF_MITOTIC_PROTEINS
3:48199695	*IP6K2*	REACTOME_ACTIVATION_OF_ATR_IN_RESPONSE_TO_REPLICATION_STRESS
3:48214438	*IP6K2*	REACTOME_ACTIVATION_OF_ATR_IN_RESPONSE_TO_REPLICATION_STRESS
20:5934513	*CRLS1*	REACTOME_ACTIVATION_OF_ATR_IN_RESPONSE_TO_REPLICATION_STRESS
20:5968737	*CRLS1*	REACTOME_ACTIVATION_OF_ATR_IN_RESPONSE_TO_REPLICATION_STRESS
20:5968964	*CRLS1*	REACTOME_ACTIVATION_OF_ATR_IN_RESPONSE_TO_REPLICATION_STRESS
17:7578671	*V1NB1*	REACTOME_ACTIVATION_OF_BH3_ONLY_PROTEINS
6:31897219	*HLA-DRB5*	REACTOME_ACTIVATION_OF_C3_AND_C5
17:7101771	*V1NB1*	REACTOME_ACTIVATION_OF_KAINATE_RECEPTORS_UPON_GLUTAMATE_BINDING
6:32814942	*HLA-DRB5*	REACTOME_ACTIVATION_OF_NF_KAPPAB_IN_B_CELLS
10:103185542	*GBF1*	REACTOME_ACTIVATION_OF_NF_KAPPAB_IN_B_CELLS
10:103308244	*GBF1*	REACTOME_ACTIVATION_OF_NF_KAPPAB_IN_B_CELLS
3:48835245	*IP6K2*	REACTOME_ACTIVATION_OF_NMDA_RECEPTORS_AND_POSTSYNAPTIC_EVENTS
4:114424939	*CAMK2D*	REACTOME_ACTIVATION_OF_NMDA_RECEPTORS_AND_POSTSYNAPTIC_EVENTS
11:83188607	*DLG2*	REACTOME_ACTIVATION_OF_NMDA_RECEPTORS_AND_POSTSYNAPTIC_EVENTS
11:83219006	*DLG2*	REACTOME_ACTIVATION_OF_NMDA_RECEPTORS_AND_POSTSYNAPTIC_EVENTS
11:83538733	*DLG2*	REACTOME_ACTIVATION_OF_NMDA_RECEPTORS_AND_POSTSYNAPTIC_EVENTS
11:83544472	*DLG2*	REACTOME_ACTIVATION_OF_NMDA_RECEPTORS_AND_POSTSYNAPTIC_EVENTS
11:83656201	*DLG2*	REACTOME_ACTIVATION_OF_NMDA_RECEPTORS_AND_POSTSYNAPTIC_EVENTS
11:83700228	*DLG2*	REACTOME_ACTIVATION_OF_NMDA_RECEPTORS_AND_POSTSYNAPTIC_EVENTS
11:83762918	*DLG2*	REACTOME_ACTIVATION_OF_NMDA_RECEPTORS_AND_POSTSYNAPTIC_EVENTS
11:83894247	*DLG2*	REACTOME_ACTIVATION_OF_NMDA_RECEPTORS_AND_POSTSYNAPTIC_EVENTS
11:84021037	*DLG2*	REACTOME_ACTIVATION_OF_NMDA_RECEPTORS_AND_POSTSYNAPTIC_EVENTS
11:84324361	*DLG2*	REACTOME_ACTIVATION_OF_NMDA_RECEPTORS_AND_POSTSYNAPTIC_EVENTS

11:84373496	*DLG2*	REACTOME_ACTIVATION_OF_NMDA_RECEPTORS_AND_POSTSYNAPTIC_EVENTS
11:84403409	*DLG2*	REACTOME_ACTIVATION_OF_NMDA_RECEPTORS_AND_POSTSYNAPTIC_EVENTS
17:7101771	*V1NB1*	REACTOME_ACTIVATION_OF_NMDA_RECEPTORS_AND_POSTSYNAPTIC_EVENTS
17:43999203	*WNT3*	REACTOME_ACTIVATION_OF_NMDA_RECEPTORS_AND_POSTSYNAPTIC_EVENTS
17:44005297	*WNT3*	REACTOME_ACTIVATION_OF_NMDA_RECEPTORS_AND_POSTSYNAPTIC_EVENTS
17:44019712	*WNT3*	REACTOME_ACTIVATION_OF_NMDA_RECEPTORS_AND_POSTSYNAPTIC_EVENTS
17:44060492	*WNT3*	REACTOME_ACTIVATION_OF_NMDA_RECEPTORS_AND_POSTSYNAPTIC_EVENTS
17:44065263	*WNT3*	REACTOME_ACTIVATION_OF_NMDA_RECEPTORS_AND_POSTSYNAPTIC_EVENTS
17:44081178	*WNT3*	REACTOME_ACTIVATION_OF_NMDA_RECEPTORS_AND_POSTSYNAPTIC_EVENTS
17:44083297	*WNT3*	REACTOME_ACTIVATION_OF_NMDA_RECEPTORS_AND_POSTSYNAPTIC_EVENTS
17:7578671	*V1NB1*	REACTOME_ACTIVATION_OF_NOXA_AND_TRANSLOCATION_TO_MITOCHONDRIA
17:7578671	*V1NB1*	REACTOME_ACTIVATION_OF_PUMA_AND_TRANSLOCATION_TO_MITOCHONDRIA
17:7479140	*V1NB1*	ME_ACTIVATION_OF_THE_MRNA_UPON_BINDING_OF_THE_CAP_BINDING_COMPLEX_AND_EIFS_AND_SUBSEQUENT_BINDIN
17:7480325	*V1NB1*	ME_ACTIVATION_OF_THE_MRNA_UPON_BINDING_OF_THE_CAP_BINDING_COMPLEX_AND_EIFS_AND_SUBSEQUENT_BINDIN
12:133206648	*FBRSL1*	REACTOME_ACTIVATION_OF_THE_PRE_REPLICATIVE_COMPLEX
12:133223685	*FBRSL1*	REACTOME_ACTIVATION_OF_THE_PRE_REPLICATIVE_COMPLEX
12:133225045	*FBRSL1*	REACTOME_ACTIVATION_OF_THE_PRE_REPLICATIVE_COMPLEX
20:5934513	*CRLS1*	REACTOME_ACTIVATION_OF_THE_PRE_REPLICATIVE_COMPLEX
20:5968737	*CRLS1*	REACTOME_ACTIVATION_OF_THE_PRE_REPLICATIVE_COMPLEX
20:5968964	*CRLS1*	REACTOME_ACTIVATION_OF_THE_PRE_REPLICATIVE_COMPLEX
20:6011934	*CRLS1*	REACTOME_ACYL_CHAIN_REMODELLING_OF_PG
1:171945488	*VAMP4*	REACTOME_ADAPTIVE_IMMUNE_SYSTEM
1:172137119	*VAMP4*	REACTOME_ADAPTIVE_IMMUNE_SYSTEM
1:172386261	*VAMP4*	REACTOME_ADAPTIVE_IMMUNE_SYSTEM
3:49740895	*IP6K2*	REACTOME_ADAPTIVE_IMMUNE_SYSTEM
3:121324643	*KPNA1*	REACTOME_ADAPTIVE_IMMUNE_SYSTEM
3:121340955	*KPNA1*	REACTOME_ADAPTIVE_IMMUNE_SYSTEM
3:122289921	*KPNA1*	REACTOME_ADAPTIVE_IMMUNE_SYSTEM
4:15608677	*BST1*	REACTOME_ADAPTIVE_IMMUNE_SYSTEM
4:170015977	*CLCN3*	REACTOME_ADAPTIVE_IMMUNE_SYSTEM
5:133710339	*C5orf24*	REACTOME_ADAPTIVE_IMMUNE_SYSTEM
6:29798642	*TRIM40*	REACTOME_ADAPTIVE_IMMUNE_SYSTEM
6:32362318	*HLA-DRB5*	REACTOME_ADAPTIVE_IMMUNE_SYSTEM
6:32602372	*HLA-DRB5*	REACTOME_ADAPTIVE_IMMUNE_SYSTEM
6:32789400	*HLA-DRB5*	REACTOME_ADAPTIVE_IMMUNE_SYSTEM
6:32791831	*HLA-DRB5*	REACTOME_ADAPTIVE_IMMUNE_SYSTEM
6:32797361	*HLA-DRB5*	REACTOME_ADAPTIVE_IMMUNE_SYSTEM
6:32802462	*HLA-DRB5*	REACTOME_ADAPTIVE_IMMUNE_SYSTEM
6:32814942	*HLA-DRB5*	REACTOME_ADAPTIVE_IMMUNE_SYSTEM
6:32930679	*HLA-DRB5*	REACTOME_ADAPTIVE_IMMUNE_SYSTEM
6:32934409	*HLA-DRB5*	REACTOME_ADAPTIVE_IMMUNE_SYSTEM
6:32973303	*HLA-DRB5*	REACTOME_ADAPTIVE_IMMUNE_SYSTEM
6:32974268	*HLA-DRB5*	REACTOME_ADAPTIVE_IMMUNE_SYSTEM
6:112009427	*FYN*	REACTOME_ADAPTIVE_IMMUNE_SYSTEM
6:112056759	*FYN*	REACTOME_ADAPTIVE_IMMUNE_SYSTEM
6:112151452	*FYN*	REACTOME_ADAPTIVE_IMMUNE_SYSTEM
7:66096028	*GS1-124K5.11*	REACTOME_ADAPTIVE_IMMUNE_SYSTEM
8:11381148	*CTSB*	REACTOME_ADAPTIVE_IMMUNE_SYSTEM
8:11712443	*CTSB*	REACTOME_ADAPTIVE_IMMUNE_SYSTEM
8:12580949	*CTSB*	REACTOME_ADAPTIVE_IMMUNE_SYSTEM
9:17579690	*SH3GL2_2*	REACTOME_ADAPTIVE_IMMUNE_SYSTEM
9:17609302	*SH3GL2_2*	REACTOME_ADAPTIVE_IMMUNE_SYSTEM
9:17627444	*SH3GL2_2*	REACTOME_ADAPTIVE_IMMUNE_SYSTEM

9:17710876	*SH3GL2_2*	REACTOME_ADAPTIVE_IMMUNE_SYSTEM
9:17732590	*SH3GL2_2*	REACTOME_ADAPTIVE_IMMUNE_SYSTEM
9:17737514	*SH3GL2_2*	REACTOME_ADAPTIVE_IMMUNE_SYSTEM
9:17758945	*SH3GL2_2*	REACTOME_ADAPTIVE_IMMUNE_SYSTEM
9:33845203	*UBAP2*	REACTOME_ADAPTIVE_IMMUNE_SYSTEM
10:103185542	*GBF1*	REACTOME_ADAPTIVE_IMMUNE_SYSTEM
10:103308244	*GBF1*	REACTOME_ADAPTIVE_IMMUNE_SYSTEM
10:104258303	*GBF1*	REACTOME_ADAPTIVE_IMMUNE_SYSTEM
16:30510571	*SETD1A*	REACTOME_ADAPTIVE_IMMUNE_SYSTEM
16:30516492	*SETD1A*	REACTOME_ADAPTIVE_IMMUNE_SYSTEM
16:30520856	*SETD1A*	REACTOME_ADAPTIVE_IMMUNE_SYSTEM
17:43390763	*CRHR1_2*	REACTOME_ADAPTIVE_IMMUNE_SYSTEM
17:45215865	*WNT3*	REACTOME_ADAPTIVE_IMMUNE_SYSTEM
17:45244822	*WNT3*	REACTOME_ADAPTIVE_IMMUNE_SYSTEM
3:123057397	*KPNA1*	REACTOME_ADENYLATE_CYCLASE_ACTIVATING_PATHWAY
3:123057397	*KPNA1*	REACTOME_ADENYLATE_CYCLASE_INHIBITORY_PATHWAY
17:39883520	*RETREG3*	REACTOME_ADHERENS_JUNCTIONS_INTERACTIONS
3:151090424	*MED12L*	REACTOME_ADP_SIGNALLING_THROUGH_P2Y_PURINOCEPTOR_12
3:151099733	*MED12L*	REACTOME_ADP_SIGNALLING_THROUGH_P2Y_PURINOCEPTOR_12
19:3156505	*SPPL2B*	REACTOME_ADP_SIGNALLING_THROUGH_P2Y_PURINOCEPTOR_1
3:123057397	*KPNA1*	REACTOME_ADRENALINE_NORADRENALINE_INHIBITS_INSULIN_SECRETION
6:32151443	*HLA-DRB5*	REACTOME_ADVANCED_GLYCOSYLATION_ENDPRODUCT_RECEPTOR_SIGNALING
9:33791164	*UBAP2*	REACTOME_ALPHA_DEFENSINS
6:31897219	*HLA-DRB5*	REACTOME_ALTERNATIVE_COMPLEMENT_ACTIVATION
8:17427186	*FGF20*	REACTOME_AMINO_ACID_TRANSPORT_ACROSS_THE_PLASMA_MEMBRANE
12:47192323	*SCAF11*	REACTOME_AMINO_ACID_TRANSPORT_ACROSS_THE_PLASMA_MEMBRANE
4:90677243	*SNCA_2*	REACTOME_AMYLOID_FIBER_FORMATION
4:90700329	*SNCA_2*	REACTOME_AMYLOID_FIBER_FORMATION
4:90727315	*SNCA_2*	REACTOME_AMYLOID_FIBER_FORMATION
4:90738839	*SNCA_2*	REACTOME_AMYLOID_FIBER_FORMATION
4:90757294	*SNCA_2*	REACTOME_AMYLOID_FIBER_FORMATION
3:121553904	*KPNA1*	REACTOME_ANCHORING_OF_THE_BASAL_BODY_TO_THE_PLASMA_MEMBRANE
4:15492268	*BST1*	REACTOME_ANCHORING_OF_THE_BASAL_BODY_TO_THE_PLASMA_MEMBRANE
4:15547513	*BST1*	REACTOME_ANCHORING_OF_THE_BASAL_BODY_TO_THE_PLASMA_MEMBRANE
4:15554219	*BST1*	REACTOME_ANCHORING_OF_THE_BASAL_BODY_TO_THE_PLASMA_MEMBRANE
10:104258303	*GBF1*	REACTOME_ANCHORING_OF_THE_BASAL_BODY_TO_THE_PLASMA_MEMBRANE
10:104591639	*GBF1*	REACTOME_ANDROGEN_BIOSYNTHESIS
6:112009427	*FYN*	REACTOME_ANTIGEN_ACTIVATES_B_CELL_RECEPTOR_BCR_LEADING_TO_GENERATION_OF_SECOND_MESSENGERS
6:112056759	*FYN*	REACTOME_ANTIGEN_ACTIVATES_B_CELL_RECEPTOR_BCR_LEADING_TO_GENERATION_OF_SECOND_MESSENGERS
6:112151452	*FYN*	REACTOME_ANTIGEN_ACTIVATES_B_CELL_RECEPTOR_BCR_LEADING_TO_GENERATION_OF_SECOND_MESSENGERS
8:11381148	*CTSB*	REACTOME_ANTIGEN_ACTIVATES_B_CELL_RECEPTOR_BCR_LEADING_TO_GENERATION_OF_SECOND_MESSENGERS
6:29798642	*TRIM40*	REACTOME_ANTIGEN_PRESENTATION:_FOLDING_ASSEMBLY_AND_PEPTIDE_LOADING_OF_CLASS_I_MHC
6:32789400	*HLA-DRB5*	REACTOME_ANTIGEN_PRESENTATION:_FOLDING_ASSEMBLY_AND_PEPTIDE_LOADING_OF_CLASS_I_MHC
6:32791831	*HLA-DRB5*	REACTOME_ANTIGEN_PRESENTATION:_FOLDING_ASSEMBLY_AND_PEPTIDE_LOADING_OF_CLASS_I_MHC
6:32797361	*HLA-DRB5*	REACTOME_ANTIGEN_PRESENTATION:_FOLDING_ASSEMBLY_AND_PEPTIDE_LOADING_OF_CLASS_I_MHC
6:32802462	*HLA-DRB5*	REACTOME_ANTIGEN_PRESENTATION:_FOLDING_ASSEMBLY_AND_PEPTIDE_LOADING_OF_CLASS_I_MHC
6:32814942	*HLA-DRB5*	REACTOME_ANTIGEN_PRESENTATION:_FOLDING_ASSEMBLY_AND_PEPTIDE_LOADING_OF_CLASS_I_MHC
6:29798642	*TRIM40*	REACTOME_ANTIGEN_PROCESSING_CROSS_PRESENTATION
6:32789400	*HLA-DRB5*	REACTOME_ANTIGEN_PROCESSING_CROSS_PRESENTATION
6:32791831	*HLA-DRB5*	REACTOME_ANTIGEN_PROCESSING_CROSS_PRESENTATION
6:32797361	*HLA-DRB5*	REACTOME_ANTIGEN_PROCESSING_CROSS_PRESENTATION
6:32802462	*HLA-DRB5*	REACTOME_ANTIGEN_PROCESSING_CROSS_PRESENTATION
6:32814942	*HLA-DRB5*	REACTOME_ANTIGEN_PROCESSING_CROSS_PRESENTATION

3:49740895	*IP6K2*	REACTOME_ANTIGEN_PROCESSING:_UBIQUITINATION_PROTEASOME_DEGRADATION
3:121324643	*KPNA1*	REACTOME_ANTIGEN_PROCESSING:_UBIQUITINATION_PROTEASOME_DEGRADATION
3:121340955	*KPNA1*	REACTOME_ANTIGEN_PROCESSING:_UBIQUITINATION_PROTEASOME_DEGRADATION
3:122289921	*KPNA1*	REACTOME_ANTIGEN_PROCESSING:_UBIQUITINATION_PROTEASOME_DEGRADATION
4:15608677	*BST1*	REACTOME_ANTIGEN_PROCESSING:_UBIQUITINATION_PROTEASOME_DEGRADATION
4:170015977	*CLCN3*	REACTOME_ANTIGEN_PROCESSING:_UBIQUITINATION_PROTEASOME_DEGRADATION
5:133710339	*C5orf24*	REACTOME_ANTIGEN_PROCESSING:_UBIQUITINATION_PROTEASOME_DEGRADATION
6:32814942	*HLA-DRB5*	REACTOME_ANTIGEN_PROCESSING:_UBIQUITINATION_PROTEASOME_DEGRADATION
7:66096028	*GS1-124K5.11*	REACTOME_ANTIGEN_PROCESSING:_UBIQUITINATION_PROTEASOME_DEGRADATION
8:12580949	*CTSB*	REACTOME_ANTIGEN_PROCESSING:_UBIQUITINATION_PROTEASOME_DEGRADATION
9:33845203	*UBAP2*	REACTOME_ANTIGEN_PROCESSING:_UBIQUITINATION_PROTEASOME_DEGRADATION
10:103185542	*GBF1*	REACTOME_ANTIGEN_PROCESSING:_UBIQUITINATION_PROTEASOME_DEGRADATION
10:103308244	*GBF1*	REACTOME_ANTIGEN_PROCESSING:_UBIQUITINATION_PROTEASOME_DEGRADATION
17:45215865	*WNT3*	REACTOME_ANTIGEN_PROCESSING:_UBIQUITINATION_PROTEASOME_DEGRADATION
17:45244822	*WNT3*	REACTOME_ANTIGEN_PROCESSING:_UBIQUITINATION_PROTEASOME_DEGRADATION
9:33791164	*UBAP2*	REACTOME_ANTIMICROBIAL_PEPTIDES
17:7479140	*V1NB1*	REACTOME_ANTIVIRAL_MECHANISM_BY_IFN_STIMULATED_GENES
17:7480325	*V1NB1*	REACTOME_ANTIVIRAL_MECHANISM_BY_IFN_STIMULATED_GENES
17:45215865	*WNT3*	REACTOME_APC_C:CDC20_MEDIATED_DEGRADATION_OF_CYCLIN_B
17:45244822	*WNT3*	REACTOME_APC_C:CDC20_MEDIATED_DEGRADATION_OF_CYCLIN_B
6:32814942	*HLA-DRB5*	ME_APC_C:CDH1_MEDIATED_DEGRADATION_OF_CDC20_AND_OTHER_APC_C:CDH1_TARGETED_PROTEINS_IN_LATE_MITOSIS_
17:45215865	*WNT3*	ME_APC_C:CDH1_MEDIATED_DEGRADATION_OF_CDC20_AND_OTHER_APC_C:CDH1_TARGETED_PROTEINS_IN_LATE_MITOSIS_
17:45244822	*WNT3*	ME_APC_C:CDH1_MEDIATED_DEGRADATION_OF_CDC20_AND_OTHER_APC_C:CDH1_TARGETED_PROTEINS_IN_LATE_MITOSIS_
17:45215865	*WNT3*	REACTOME_APC_CDC20_MEDIATED_DEGRADATION_OF_NEK2A
17:45244822	*WNT3*	REACTOME_APC_CDC20_MEDIATED_DEGRADATION_OF_NEK2A
1:156059276	*GBAP1*	REACTOME_APOPTOTIC_CLEAVAGE_OF_CELLULAR_PROTEINS
17:43999203	*WNT3*	REACTOME_APOPTOTIC_CLEAVAGE_OF_CELLULAR_PROTEINS
17:44005297	*WNT3*	REACTOME_APOPTOTIC_CLEAVAGE_OF_CELLULAR_PROTEINS
17:44019712	*WNT3*	REACTOME_APOPTOTIC_CLEAVAGE_OF_CELLULAR_PROTEINS
17:44060492	*WNT3*	REACTOME_APOPTOTIC_CLEAVAGE_OF_CELLULAR_PROTEINS
17:44065263	*WNT3*	REACTOME_APOPTOTIC_CLEAVAGE_OF_CELLULAR_PROTEINS
17:44081178	*WNT3*	REACTOME_APOPTOTIC_CLEAVAGE_OF_CELLULAR_PROTEINS
17:44083297	*WNT3*	REACTOME_APOPTOTIC_CLEAVAGE_OF_CELLULAR_PROTEINS
1:156059276	*GBAP1*	REACTOME_APOPTOTIC_EXECUTION_PHASE
17:43999203	*WNT3*	REACTOME_APOPTOTIC_EXECUTION_PHASE
17:44005297	*WNT3*	REACTOME_APOPTOTIC_EXECUTION_PHASE
17:44019712	*WNT3*	REACTOME_APOPTOTIC_EXECUTION_PHASE
17:44060492	*WNT3*	REACTOME_APOPTOTIC_EXECUTION_PHASE
17:44065263	*WNT3*	REACTOME_APOPTOTIC_EXECUTION_PHASE
17:44081178	*WNT3*	REACTOME_APOPTOTIC_EXECUTION_PHASE
17:44083297	*WNT3*	REACTOME_APOPTOTIC_EXECUTION_PHASE
3:48835245	*IP6K2*	REACTOME_AQUAPORIN_MEDIATED_TRANSPORT
3:123057397	*KPNA1*	REACTOME_AQUAPORIN_MEDIATED_TRANSPORT
17:7983799	*V1NB1*	REACTOME_ARACHIDONIC_ACID_METABOLISM
17:8019125	*V1NB1*	REACTOME_ARACHIDONIC_ACID_METABOLISM
2:134977261	*TMEM163*	REACTOME_ASPARAGINE_N_LINKED_GLYCOSYLATION
2:135194840	*TMEM163*	REACTOME_ASPARAGINE_N_LINKED_GLYCOSYLATION
2:135202455	*TMEM163*	REACTOME_ASPARAGINE_N_LINKED_GLYCOSYLATION
3:121411876	*KPNA1*	REACTOME_ASPARAGINE_N_LINKED_GLYCOSYLATION
3:121412702	*KPNA1*	REACTOME_ASPARAGINE_N_LINKED_GLYCOSYLATION
4:76695927	*FAM47E-STBD1*	REACTOME_ASPARAGINE_N_LINKED_GLYCOSYLATION
4:113938109	*CAMK2D*	REACTOME_ASPARAGINE_N_LINKED_GLYCOSYLATION
4:114077386	*CAMK2D*	REACTOME_ASPARAGINE_N_LINKED_GLYCOSYLATION

4:114217997	*CAMK2D*	REACTOME_ASPARAGINE_N_LINKED_GLYCOSYLATION
6:32151443	*HLA-DRB5*	REACTOME_ASPARAGINE_N_LINKED_GLYCOSYLATION
10:104005410	*GBF1*	REACTOME_ASPARAGINE_N_LINKED_GLYCOSYLATION
10:104034550	*GBF1*	REACTOME_ASPARAGINE_N_LINKED_GLYCOSYLATION
10:104258303	*GBF1*	REACTOME_ASPARAGINE_N_LINKED_GLYCOSYLATION
10:121683206	*INPP5F*	REACTOME_ASPARAGINE_N_LINKED_GLYCOSYLATION
10:121686714	*INPP5F*	REACTOME_ASPARAGINE_N_LINKED_GLYCOSYLATION
10:121689030	*INPP5F*	REACTOME_ASPARAGINE_N_LINKED_GLYCOSYLATION
17:7491546	*V1NB1*	REACTOME_ASPARAGINE_N_LINKED_GLYCOSYLATION
17:44800946	*WNT3*	REACTOME_ASPARAGINE_N_LINKED_GLYCOSYLATION
17:44833217	*WNT3*	REACTOME_ASPARAGINE_N_LINKED_GLYCOSYLATION
17:45059032	*WNT3*	REACTOME_ASPARAGINE_N_LINKED_GLYCOSYLATION
17:45089680	*WNT3*	REACTOME_ASPARAGINE_N_LINKED_GLYCOSYLATION
4:114424939	*CAMK2D*	REACTOME_ASSEMBLY_AND_CELL_SURFACE_PRESENTATION_OF_NMDA_RECEPTORS
11:83188607	*DLG2*	REACTOME_ASSEMBLY_AND_CELL_SURFACE_PRESENTATION_OF_NMDA_RECEPTORS
11:83219006	*DLG2*	REACTOME_ASSEMBLY_AND_CELL_SURFACE_PRESENTATION_OF_NMDA_RECEPTORS
11:83538733	*DLG2*	REACTOME_ASSEMBLY_AND_CELL_SURFACE_PRESENTATION_OF_NMDA_RECEPTORS
11:83544472	*DLG2*	REACTOME_ASSEMBLY_AND_CELL_SURFACE_PRESENTATION_OF_NMDA_RECEPTORS
11:83656201	*DLG2*	REACTOME_ASSEMBLY_AND_CELL_SURFACE_PRESENTATION_OF_NMDA_RECEPTORS
11:83700228	*DLG2*	REACTOME_ASSEMBLY_AND_CELL_SURFACE_PRESENTATION_OF_NMDA_RECEPTORS
11:83762918	*DLG2*	REACTOME_ASSEMBLY_AND_CELL_SURFACE_PRESENTATION_OF_NMDA_RECEPTORS
11:83894247	*DLG2*	REACTOME_ASSEMBLY_AND_CELL_SURFACE_PRESENTATION_OF_NMDA_RECEPTORS
11:84021037	*DLG2*	REACTOME_ASSEMBLY_AND_CELL_SURFACE_PRESENTATION_OF_NMDA_RECEPTORS
11:84324361	*DLG2*	REACTOME_ASSEMBLY_AND_CELL_SURFACE_PRESENTATION_OF_NMDA_RECEPTORS
11:84373496	*DLG2*	REACTOME_ASSEMBLY_AND_CELL_SURFACE_PRESENTATION_OF_NMDA_RECEPTORS
11:84403409	*DLG2*	REACTOME_ASSEMBLY_AND_CELL_SURFACE_PRESENTATION_OF_NMDA_RECEPTORS
17:7101771	*V1NB1*	REACTOME_ASSEMBLY_AND_CELL_SURFACE_PRESENTATION_OF_NMDA_RECEPTORS
6:33153528	*HLA-DRB5*	REACTOME_ASSEMBLY_OF_COLLAGEN_FIBRILS_AND_OTHER_MULTIMERIC_STRUCTURES
8:11712443	*CTSB*	REACTOME_ASSEMBLY_OF_COLLAGEN_FIBRILS_AND_OTHER_MULTIMERIC_STRUCTURES
8:23190941	*BIN3*	REACTOME_ASSEMBLY_OF_COLLAGEN_FIBRILS_AND_OTHER_MULTIMERIC_STRUCTURES
8:23212542	*BIN3*	REACTOME_ASSEMBLY_OF_COLLAGEN_FIBRILS_AND_OTHER_MULTIMERIC_STRUCTURES
8:23223713	*BIN3*	REACTOME_ASSEMBLY_OF_COLLAGEN_FIBRILS_AND_OTHER_MULTIMERIC_STRUCTURES
8:17125768	*FGF20*	REACTOME_ASSEMBLY_OF_THE_HIV_VIRION
10:15199347	*ITGA8*	REACTOME_ASSEMBLY_OF_THE_HIV_VIRION
6:32814942	*HLA-DRB5*	REACTOME_ASSEMBLY_OF_THE_PRE_REPLICATIVE_COMPLEX
20:5934513	*CRLS1*	REACTOME_ASSEMBLY_OF_THE_PRE_REPLICATIVE_COMPLEX
20:5968737	*CRLS1*	REACTOME_ASSEMBLY_OF_THE_PRE_REPLICATIVE_COMPLEX
20:5968964	*CRLS1*	REACTOME_ASSEMBLY_OF_THE_PRE_REPLICATIVE_COMPLEX
4:15608677	*BST1*	REACTOME_ASSOCIATION_OF_TRIC_CCT_WITH_TARGET_PROTEINS_DURING_BIOSYNTHESIS
6:32814942	*HLA-DRB5*	REACTOME_ASYMMETRIC_LOCALIZATION_OF_PCP_PROTEINS
17:7129974	*V1NB1*	REACTOME_ASYMMETRIC_LOCALIZATION_OF_PCP_PROTEINS
6:71594785	*RIMS1*	REACTOME_A_TETRASACCHARIDE_LINKER_SEQUENCE_IS_REQUIRED_FOR_GAG_SYNTHESIS
1:161875645	*FCGR2A*	REACTOME_ATF4_ACTIVATES_GENES_IN_RESPONSE_TO_ENDOPLASMIC_RETICULUM_STRESS
1:161930954	*FCGR2A*	REACTOME_ATF4_ACTIVATES_GENES_IN_RESPONSE_TO_ENDOPLASMIC_RETICULUM_STRESS
1:161875645	*FCGR2A*	REACTOME_ATF6_ATF6_ALPHA_ACTIVATES_CHAPERONE_GENES
1:161930954	*FCGR2A*	REACTOME_ATF6_ATF6_ALPHA_ACTIVATES_CHAPERONE_GENES
1:161875645	*FCGR2A*	REACTOME_ATF6_ATF6_ALPHA_ACTIVATES_CHAPERONES
1:161930954	*FCGR2A*	REACTOME_ATF6_ATF6_ALPHA_ACTIVATES_CHAPERONES
6:32814942	*HLA-DRB5*	REACTOME_AUF1_HNRNP_D0_BINDS_AND_DESTABILIZES_MRNA
10:104258303	*GBF1*	REACTOME_AURKA_ACTIVATION_BY_TPX2
8:17125768	*FGF20*	REACTOME_AUTOPHAGY
12:133295501	*FBRSL1*	REACTOME_AUTOPHAGY
1:171945488	*VAMP4*	REACTOME_AXON_GUIDANCE

1:172137119	*VAMP4*	REACTOME_AXON_GUIDANCE
1:172386261	*VAMP4*	REACTOME_AXON_GUIDANCE
1:227067656	*ITPKB*	REACTOME_AXON_GUIDANCE
3:48835245	*IP6K2*	REACTOME_AXON_GUIDANCE
3:49406095	*IP6K2*	REACTOME_AXON_GUIDANCE
3:49424231	*IP6K2*	REACTOME_AXON_GUIDANCE
3:49450137	*IP6K2*	REACTOME_AXON_GUIDANCE
3:49561208	*IP6K2*	REACTOME_AXON_GUIDANCE
4:113938109	*CAMK2D*	REACTOME_AXON_GUIDANCE
4:114077386	*CAMK2D*	REACTOME_AXON_GUIDANCE
4:114217997	*CAMK2D*	REACTOME_AXON_GUIDANCE
6:32814942	*HLA-DRB5*	REACTOME_AXON_GUIDANCE
6:112009427	*FYN*	REACTOME_AXON_GUIDANCE
6:112056759	*FYN*	REACTOME_AXON_GUIDANCE
6:112151452	*FYN*	REACTOME_AXON_GUIDANCE
8:21665306	*BIN3*	REACTOME_AXON_GUIDANCE
8:21668901	*BIN3*	REACTOME_AXON_GUIDANCE
9:17579690	*SH3GL2_2*	REACTOME_AXON_GUIDANCE
9:17609302	*SH3GL2_2*	REACTOME_AXON_GUIDANCE
9:17627444	*SH3GL2_2*	REACTOME_AXON_GUIDANCE
9:17710876	*SH3GL2_2*	REACTOME_AXON_GUIDANCE
9:17732590	*SH3GL2_2*	REACTOME_AXON_GUIDANCE
9:17737514	*SH3GL2_2*	REACTOME_AXON_GUIDANCE
9:17758945	*SH3GL2_2*	REACTOME_AXON_GUIDANCE
12:41147295	*LRRK2_2*	REACTOME_AXON_GUIDANCE
12:41251554	*LRRK2_2*	REACTOME_AXON_GUIDANCE
12:41407403	*LRRK2_2*	REACTOME_AXON_GUIDANCE
17:7101771	*V1NB1*	REACTOME_AXON_GUIDANCE
12:133206648	*FBRSL1*	REACTOME_BASE_EXCISION_REPAIR
12:133223685	*FBRSL1*	REACTOME_BASE_EXCISION_REPAIR
12:133225045	*FBRSL1*	REACTOME_BASE_EXCISION_REPAIR
17:7560294	*V1NB1*	REACTOME_BASIGIN_INTERACTIONS
3:49406095	*IP6K2*	REACTOME_BETA_CATENIN_INDEPENDENT_WNT_SIGNALING
3:49424231	*IP6K2*	REACTOME_BETA_CATENIN_INDEPENDENT_WNT_SIGNALING
3:49450137	*IP6K2*	REACTOME_BETA_CATENIN_INDEPENDENT_WNT_SIGNALING
6:32814942	*HLA-DRB5*	REACTOME_BETA_CATENIN_INDEPENDENT_WNT_SIGNALING
17:7129974	*V1NB1*	REACTOME_BETA_CATENIN_INDEPENDENT_WNT_SIGNALING
17:42338993	*FAM171A2*	REACTOME_BICARBONATE_TRANSPORTERS
16:30999462	*SETD1A*	REACTOME_BILE_ACID_AND_BILE_SALT_METABOLISM
7:65705034	*GS1-124K5.11*	REACTOME_BIOLOGICAL_OXIDATIONS
10:104635620	*GBF1*	REACTOME_BIOLOGICAL_OXIDATIONS
17:7491546	*V1NB1*	OSYNTHESIS_OF_THE_N_GLYCAN_PRECURSOR_DOLICHOL_LIPID_LINKED_OLIGOSACCHARIDE_LLO_AND_TRANSFER_TO_A_NA
3:182760073	*MCCC1*	REACTOME_BIOTIN_TRANSPORT_AND_METABOLISM
21:38328886	*DYRK1A*	REACTOME_BIOTIN_TRANSPORT_AND_METABOLISM
6:33252561	*HLA-DRB5*	REACTOME_BLOOD_GROUP_SYSTEMS_BIOSYNTHESIS
2:101439596	*MAP4K4*	REACTOME_BMAL1:CLOCK_NPAS2_ACTIVATES_CIRCADIAN_GENE_EXPRESSION
2:101550181	*MAP4K4*	REACTOME_BMAL1:CLOCK_NPAS2_ACTIVATES_CIRCADIAN_GENE_EXPRESSION
2:101603812	*MAP4K4*	REACTOME_BMAL1:CLOCK_NPAS2_ACTIVATES_CIRCADIAN_GENE_EXPRESSION
3:182760073	*MCCC1*	REACTOME_BRANCHED_CHAIN_AMINO_ACID_CATABOLISM
8:17125768	*FGF20*	REACTOME_BUDDING_AND_MATURATION_OF_HIV_VIRION
6:32362318	*HLA-DRB5*	REACTOME_BUTYROPHILIN_BTN_FAMILY_INTERACTIONS
1:154804988	*GBAP1*	REACTOME_CA2PLUS_ACTIVATED_KPLUS_CHANNELS
3:48835245	*IP6K2*	REACTOME_CA_DEPENDENT_EVENTS

3:123057397	*KPNA1*	REACTOME_CA_DEPENDENT_EVENTS
4:114424939	*CAMK2D*	REACTOME_CA_DEPENDENT_EVENTS
6:32151443	*HLA-DRB5*	REACTOME_CALNEXIN_CALRETICULIN_CYCLE
4:114424939	*CAMK2D*	REACTOME_CAMK_IV_MEDIATED_PHOSPHORYLATION_OF_CREB
3:49462410	*IP6K2*	REACTOME_CARBOXYTERMINAL_POST_TRANSLATIONAL_MODIFICATIONS_OF_TUBULIN
14:76159192	*RPS6KL1*	REACTOME_CARBOXYTERMINAL_POST_TRANSLATIONAL_MODIFICATIONS_OF_TUBULIN
4:114424939	*CAMK2D*	REACTOME_CARDIAC_CONDUCTION
17:7560294	*V1NB1*	REACTOME_CARDIAC_CONDUCTION
17:45059032	*WNT3*	REACTOME_CARGO_CONCENTRATION_IN_THE_ER
17:45089680	*WNT3*	REACTOME_CARGO_CONCENTRATION_IN_THE_ER
1:155847427	*GBAP1*	REACTOME_CARGO_RECOGNITION_FOR_CLATHRIN_MEDIATED_ENDOCYTOSIS
1:171689486	*VAMP4*	REACTOME_CARGO_RECOGNITION_FOR_CLATHRIN_MEDIATED_ENDOCYTOSIS
2:169999639	*STK39*	REACTOME_CARGO_RECOGNITION_FOR_CLATHRIN_MEDIATED_ENDOCYTOSIS
2:170028024	*STK39*	REACTOME_CARGO_RECOGNITION_FOR_CLATHRIN_MEDIATED_ENDOCYTOSIS
4:77100807	*FAM47E-STBD1*	REACTOME_CARGO_RECOGNITION_FOR_CLATHRIN_MEDIATED_ENDOCYTOSIS
4:77104152	*FAM47E-STBD1*	REACTOME_CARGO_RECOGNITION_FOR_CLATHRIN_MEDIATED_ENDOCYTOSIS
4:77124196	*FAM47E-STBD1*	REACTOME_CARGO_RECOGNITION_FOR_CLATHRIN_MEDIATED_ENDOCYTOSIS
4:77134786	*FAM47E-STBD1*	REACTOME_CARGO_RECOGNITION_FOR_CLATHRIN_MEDIATED_ENDOCYTOSIS
9:17579690	*SH3GL2_2*	REACTOME_CARGO_RECOGNITION_FOR_CLATHRIN_MEDIATED_ENDOCYTOSIS
9:17609302	*SH3GL2_2*	REACTOME_CARGO_RECOGNITION_FOR_CLATHRIN_MEDIATED_ENDOCYTOSIS
9:17627444	*SH3GL2_2*	REACTOME_CARGO_RECOGNITION_FOR_CLATHRIN_MEDIATED_ENDOCYTOSIS
9:17710876	*SH3GL2_2*	REACTOME_CARGO_RECOGNITION_FOR_CLATHRIN_MEDIATED_ENDOCYTOSIS
9:17732590	*SH3GL2_2*	REACTOME_CARGO_RECOGNITION_FOR_CLATHRIN_MEDIATED_ENDOCYTOSIS
9:17737514	*SH3GL2_2*	REACTOME_CARGO_RECOGNITION_FOR_CLATHRIN_MEDIATED_ENDOCYTOSIS
9:17758945	*SH3GL2_2*	REACTOME_CARGO_RECOGNITION_FOR_CLATHRIN_MEDIATED_ENDOCYTOSIS
17:7129974	*V1NB1*	REACTOME_CARGO_RECOGNITION_FOR_CLATHRIN_MEDIATED_ENDOCYTOSIS
8:131153067	*FAM49B*	REACTOME_CARGO_TRAFFICKING_TO_THE_PERICILIARY_MEMBRANE
8:131190319	*FAM49B*	REACTOME_CARGO_TRAFFICKING_TO_THE_PERICILIARY_MEMBRANE
8:131427864	*FAM49B*	REACTOME_CARGO_TRAFFICKING_TO_THE_PERICILIARY_MEMBRANE
10:104005410	*GBF1*	REACTOME_CARGO_TRAFFICKING_TO_THE_PERICILIARY_MEMBRANE
10:104034550	*GBF1*	REACTOME_CARGO_TRAFFICKING_TO_THE_PERICILIARY_MEMBRANE
10:104443875	*GBF1*	REACTOME_CARGO_TRAFFICKING_TO_THE_PERICILIARY_MEMBRANE
10:104452818	*GBF1*	REACTOME_CARGO_TRAFFICKING_TO_THE_PERICILIARY_MEMBRANE
8:23050400	*BIN3*	REACTOME_CASPASE_ACTIVATION_VIA_DEATH_RECEPTORS_IN_THE_PRESENCE_OF_LIGAND
8:23050400	*BIN3*	REACTOME_CASPASE_ACTIVATION_VIA_EXTRINSIC_APOPTOTIC_SIGNALLING_PATHWAY
17:43999203	*WNT3*	REACTOME_CASPASE_MEDIATED_CLEAVAGE_OF_CYTOSKELETAL_PROTEINS
17:44005297	*WNT3*	REACTOME_CASPASE_MEDIATED_CLEAVAGE_OF_CYTOSKELETAL_PROTEINS
17:44019712	*WNT3*	REACTOME_CASPASE_MEDIATED_CLEAVAGE_OF_CYTOSKELETAL_PROTEINS
17:44060492	*WNT3*	REACTOME_CASPASE_MEDIATED_CLEAVAGE_OF_CYTOSKELETAL_PROTEINS
17:44065263	*WNT3*	REACTOME_CASPASE_MEDIATED_CLEAVAGE_OF_CYTOSKELETAL_PROTEINS
17:44081178	*WNT3*	REACTOME_CASPASE_MEDIATED_CLEAVAGE_OF_CYTOSKELETAL_PROTEINS
17:44083297	*WNT3*	REACTOME_CASPASE_MEDIATED_CLEAVAGE_OF_CYTOSKELETAL_PROTEINS
6:112009427	*FYN*	REACTOME_CD209_DC_SIGN_SIGNALING
6:112056759	*FYN*	REACTOME_CD209_DC_SIGN_SIGNALING
6:112151452	*FYN*	REACTOME_CD209_DC_SIGN_SIGNALING
6:112009427	*FYN*	REACTOME_CD28_CO_STIMULATION
6:112056759	*FYN*	REACTOME_CD28_CO_STIMULATION
6:112151452	*FYN*	REACTOME_CD28_CO_STIMULATION
17:43390763	*CRHR1_2*	REACTOME_CD28_CO_STIMULATION
6:112009427	*FYN*	REACTOME_CD28_DEPENDENT_PI3K_AKT_SIGNALING
6:112056759	*FYN*	REACTOME_CD28_DEPENDENT_PI3K_AKT_SIGNALING
6:112151452	*FYN*	REACTOME_CD28_DEPENDENT_PI3K_AKT_SIGNALING
17:43390763	*CRHR1_2*	REACTOME_CD28_DEPENDENT_PI3K_AKT_SIGNALING

6:112009427	*FYN*	REACTOME_CD28_DEPENDENT_VAV1_PATHWAY
6:112056759	*FYN*	REACTOME_CD28_DEPENDENT_VAV1_PATHWAY
6:112151452	*FYN*	REACTOME_CD28_DEPENDENT_VAV1_PATHWAY
20:5934513	*CRLS1*	REACTOME_CDC6_ASSOCIATION_WITH_THE_ORC:ORIGIN_COMPLEX
20:5968737	*CRLS1*	REACTOME_CDC6_ASSOCIATION_WITH_THE_ORC:ORIGIN_COMPLEX
20:5968964	*CRLS1*	REACTOME_CDC6_ASSOCIATION_WITH_THE_ORC:ORIGIN_COMPLEX
6:32814942	*HLA-DRB5*	REACTOME_CDK_MEDIATED_PHOSPHORYLATION_AND_REMOVAL_OF_CDC6
17:45215865	*WNT3*	REACTOME_CDK_MEDIATED_PHOSPHORYLATION_AND_REMOVAL_OF_CDC6
17:45244822	*WNT3*	REACTOME_CDK_MEDIATED_PHOSPHORYLATION_AND_REMOVAL_OF_CDC6
6:112009427	*FYN*	REACTOME_CELL_CELL_COMMUNICATION
6:112056759	*FYN*	REACTOME_CELL_CELL_COMMUNICATION
6:112151452	*FYN*	REACTOME_CELL_CELL_COMMUNICATION
17:39883520	*RETREG3*	REACTOME_CELL_CELL_COMMUNICATION
17:39883520	*RETREG3*	REACTOME_CELL_CELL_JUNCTION_ORGANIZATION
3:48199695	*IP6K2*	REACTOME_CELL_CYCLE_CHECKPOINTS
3:48214438	*IP6K2*	REACTOME_CELL_CYCLE_CHECKPOINTS
6:32814942	*HLA-DRB5*	REACTOME_CELL_CYCLE_CHECKPOINTS
12:122816734	*HIP1R*	REACTOME_CELL_CYCLE_CHECKPOINTS
12:122817580	*HIP1R*	REACTOME_CELL_CYCLE_CHECKPOINTS
12:122853197	*HIP1R*	REACTOME_CELL_CYCLE_CHECKPOINTS
12:123057792	*HIP1R*	REACTOME_CELL_CYCLE_CHECKPOINTS
12:123110365	*HIP1R*	REACTOME_CELL_CYCLE_CHECKPOINTS
12:123110785	*HIP1R*	REACTOME_CELL_CYCLE_CHECKPOINTS
17:7578671	*V1NB1*	REACTOME_CELL_CYCLE_CHECKPOINTS
17:45215865	*WNT3*	REACTOME_CELL_CYCLE_CHECKPOINTS
17:45244822	*WNT3*	REACTOME_CELL_CYCLE_CHECKPOINTS
17:59922925	*BRIP1*	REACTOME_CELL_CYCLE_CHECKPOINTS
20:5934513	*CRLS1*	REACTOME_CELL_CYCLE_CHECKPOINTS
20:5968737	*CRLS1*	REACTOME_CELL_CYCLE_CHECKPOINTS
20:5968964	*CRLS1*	REACTOME_CELL_CYCLE_CHECKPOINTS
1:156059276	*GBAP1*	REACTOME_CELL_CYCLE_MITOTIC
3:48199695	*IP6K2*	REACTOME_CELL_CYCLE_MITOTIC
3:48214438	*IP6K2*	REACTOME_CELL_CYCLE_MITOTIC
4:76695927	*FAM47E-STBD1*	REACTOME_CELL_CYCLE_MITOTIC
6:32814942	*HLA-DRB5*	REACTOME_CELL_CYCLE_MITOTIC
10:103185542	*GBF1*	REACTOME_CELL_CYCLE_MITOTIC
10:103308244	*GBF1*	REACTOME_CELL_CYCLE_MITOTIC
10:104258303	*GBF1*	REACTOME_CELL_CYCLE_MITOTIC
12:122816734	*HIP1R*	REACTOME_CELL_CYCLE_MITOTIC
12:122817580	*HIP1R*	REACTOME_CELL_CYCLE_MITOTIC
12:122853197	*HIP1R*	REACTOME_CELL_CYCLE_MITOTIC
12:123057792	*HIP1R*	REACTOME_CELL_CYCLE_MITOTIC
12:123110365	*HIP1R*	REACTOME_CELL_CYCLE_MITOTIC
12:123110785	*HIP1R*	REACTOME_CELL_CYCLE_MITOTIC
12:133206648	*FBRSL1*	REACTOME_CELL_CYCLE_MITOTIC
12:133223685	*FBRSL1*	REACTOME_CELL_CYCLE_MITOTIC
12:133225045	*FBRSL1*	REACTOME_CELL_CYCLE_MITOTIC
17:7578671	*V1NB1*	REACTOME_CELL_CYCLE_MITOTIC
17:40818584	*RETREG3*	REACTOME_CELL_CYCLE_MITOTIC
17:45215865	*WNT3*	REACTOME_CELL_CYCLE_MITOTIC
17:45244822	*WNT3*	REACTOME_CELL_CYCLE_MITOTIC
17:76177567	*DNAH17*	REACTOME_CELL_CYCLE_MITOTIC
20:5934513	*CRLS1*	REACTOME_CELL_CYCLE_MITOTIC

20:5968737	*CRLS1*	REACTOME_CELL_CYCLE_MITOTIC
20:5968964	*CRLS1*	REACTOME_CELL_CYCLE_MITOTIC
21:38740824	*DYRK1A*	REACTOME_CELL_CYCLE_MITOTIC
21:38770530	*DYRK1A*	REACTOME_CELL_CYCLE_MITOTIC
1:156059276	*GBAP1*	REACTOME_CELL_CYCLE
3:48199695	*IP6K2*	REACTOME_CELL_CYCLE
3:48214438	*IP6K2*	REACTOME_CELL_CYCLE
4:76695927	*FAM47E-STBD1*	REACTOME_CELL_CYCLE
6:32814942	*HLA-DRB5*	REACTOME_CELL_CYCLE
10:103185542	*GBF1*	REACTOME_CELL_CYCLE
10:103308244	*GBF1*	REACTOME_CELL_CYCLE
10:104258303	*GBF1*	REACTOME_CELL_CYCLE
12:122816734	*HIP1R*	REACTOME_CELL_CYCLE
12:122817580	*HIP1R*	REACTOME_CELL_CYCLE
12:122853197	*HIP1R*	REACTOME_CELL_CYCLE
12:123057792	*HIP1R*	REACTOME_CELL_CYCLE
12:123110365	*HIP1R*	REACTOME_CELL_CYCLE
12:123110785	*HIP1R*	REACTOME_CELL_CYCLE
12:133206648	*FBRSL1*	REACTOME_CELL_CYCLE
12:133223685	*FBRSL1*	REACTOME_CELL_CYCLE
12:133225045	*FBRSL1*	REACTOME_CELL_CYCLE
14:75495059	*RPS6KL1*	REACTOME_CELL_CYCLE
17:40818584	*RETREG3*	REACTOME_CELL_CYCLE
17:45215865	*WNT3*	REACTOME_CELL_CYCLE
17:45244822	*WNT3*	REACTOME_CELL_CYCLE
17:59922925	*BRIP1*	REACTOME_CELL_CYCLE
17:76177567	*DNAH17*	REACTOME_CELL_CYCLE
20:5934513	*CRLS1*	REACTOME_CELL_CYCLE
20:5968737	*CRLS1*	REACTOME_CELL_CYCLE
20:5968964	*CRLS1*	REACTOME_CELL_CYCLE
21:38740824	*DYRK1A*	REACTOME_CELL_CYCLE
21:38770530	*DYRK1A*	REACTOME_CELL_CYCLE
1:155975202	*GBAP1*	REACTOME_CELL_DEATH_SIGNALLING_VIA_NRAGE_NRIF_AND_NADE
1:227067656	*ITPKB*	REACTOME_CELL_DEATH_SIGNALLING_VIA_NRAGE_NRIF_AND_NADE
17:39883520	*RETREG3*	REACTOME_CELL_JUNCTION_ORGANIZATION
6:112009427	*FYN*	REACTOME_CELL_SURFACE_INTERACTIONS_AT_THE_VASCULAR_WALL
6:112056759	*FYN*	REACTOME_CELL_SURFACE_INTERACTIONS_AT_THE_VASCULAR_WALL
6:112151452	*FYN*	REACTOME_CELL_SURFACE_INTERACTIONS_AT_THE_VASCULAR_WALL
8:23050400	*BIN3*	REACTOME_CELL_SURFACE_INTERACTIONS_AT_THE_VASCULAR_WALL
11:133947732	*IGSF9B*	REACTOME_CELL_SURFACE_INTERACTIONS_AT_THE_VASCULAR_WALL
16:30510571	*SETD1A*	REACTOME_CELL_SURFACE_INTERACTIONS_AT_THE_VASCULAR_WALL
16:30516492	*SETD1A*	REACTOME_CELL_SURFACE_INTERACTIONS_AT_THE_VASCULAR_WALL
16:30520856	*SETD1A*	REACTOME_CELL_SURFACE_INTERACTIONS_AT_THE_VASCULAR_WALL
16:31370452	*SETD1A*	REACTOME_CELL_SURFACE_INTERACTIONS_AT_THE_VASCULAR_WALL
17:7560294	*V1NB1*	REACTOME_CELL_SURFACE_INTERACTIONS_AT_THE_VASCULAR_WALL
1:205645310	*RAB29*	REACTOME_CELLULAR_HEXOSE_TRANSPORT
2:102357447	*MAP4K4*	REACTOME_CELLULAR_RESPONSES_TO_EXTERNAL_STIMULI
2:102413116	*MAP4K4*	REACTOME_CELLULAR_RESPONSES_TO_EXTERNAL_STIMULI
4:114424939	*CAMK2D*	REACTOME_CELLULAR_RESPONSES_TO_EXTERNAL_STIMULI
6:31862876	*HLA-DRB5*	REACTOME_CELLULAR_RESPONSES_TO_EXTERNAL_STIMULI
6:32814942	*HLA-DRB5*	REACTOME_CELLULAR_RESPONSES_TO_EXTERNAL_STIMULI
9:34342881	*UBAP2*	REACTOME_CELLULAR_RESPONSES_TO_EXTERNAL_STIMULI
10:104258303	*GBF1*	REACTOME_CELLULAR_RESPONSES_TO_EXTERNAL_STIMULI

10:121429633	*INPP5F*	REACTOME_CELLULAR_RESPONSES_TO_EXTERNAL_STIMULI
10:121437329	*INPP5F*	REACTOME_CELLULAR_RESPONSES_TO_EXTERNAL_STIMULI
12:133295501	*FBRSL1*	REACTOME_CELLULAR_RESPONSES_TO_EXTERNAL_STIMULI
17:7578671	*V1NB1*	REACTOME_CELLULAR_RESPONSES_TO_EXTERNAL_STIMULI
17:40132591	*RETREG3*	REACTOME_CELLULAR_RESPONSES_TO_EXTERNAL_STIMULI
17:45215865	*WNT3*	REACTOME_CELLULAR_RESPONSES_TO_EXTERNAL_STIMULI
17:45244822	*WNT3*	REACTOME_CELLULAR_RESPONSES_TO_EXTERNAL_STIMULI
2:102357447	*MAP4K4*	REACTOME_CELLULAR_RESPONSES_TO_STRESS
2:102413116	*MAP4K4*	REACTOME_CELLULAR_RESPONSES_TO_STRESS
4:114424939	*CAMK2D*	REACTOME_CELLULAR_RESPONSES_TO_STRESS
6:31862876	*HLA-DRB5*	REACTOME_CELLULAR_RESPONSES_TO_STRESS
6:32814942	*HLA-DRB5*	REACTOME_CELLULAR_RESPONSES_TO_STRESS
9:34342881	*UBAP2*	REACTOME_CELLULAR_RESPONSES_TO_STRESS
10:104258303	*GBF1*	REACTOME_CELLULAR_RESPONSES_TO_STRESS
10:121429633	*INPP5F*	REACTOME_CELLULAR_RESPONSES_TO_STRESS
10:121437329	*INPP5F*	REACTOME_CELLULAR_RESPONSES_TO_STRESS
17:7578671	*V1NB1*	REACTOME_CELLULAR_RESPONSES_TO_STRESS
17:40132591	*RETREG3*	REACTOME_CELLULAR_RESPONSES_TO_STRESS
17:45215865	*WNT3*	REACTOME_CELLULAR_RESPONSES_TO_STRESS
17:45244822	*WNT3*	REACTOME_CELLULAR_RESPONSES_TO_STRESS
4:114424939	*CAMK2D*	REACTOME_CELLULAR_RESPONSE_TO_HEAT_STRESS
10:121429633	*INPP5F*	REACTOME_CELLULAR_RESPONSE_TO_HEAT_STRESS
10:121437329	*INPP5F*	REACTOME_CELLULAR_RESPONSE_TO_HEAT_STRESS
17:40132591	*RETREG3*	REACTOME_CELLULAR_RESPONSE_TO_HEAT_STRESS
6:32814942	*HLA-DRB5*	REACTOME_CELLULAR_RESPONSE_TO_HYPOXIA
2:102357447	*MAP4K4*	REACTOME_CELLULAR_SENESCENCE
2:102413116	*MAP4K4*	REACTOME_CELLULAR_SENESCENCE
6:31862876	*HLA-DRB5*	REACTOME_CELLULAR_SENESCENCE
17:7578671	*V1NB1*	REACTOME_CELLULAR_SENESCENCE
17:45215865	*WNT3*	REACTOME_CELLULAR_SENESCENCE
17:45244822	*WNT3*	REACTOME_CELLULAR_SENESCENCE
10:104258303	*GBF1*	REACTOME_CENTROSOME_MATURATION
17:40818584	*RETREG3*	REACTOME_CENTROSOME_MATURATION
11:10647977	*RNF141*	REACTOME_CGMP_EFFECTS
11:10648481	*RNF141*	REACTOME_CGMP_EFFECTS
11:10660840	*RNF141*	REACTOME_CGMP_EFFECTS
6:71594785	*RIMS1*	REACTOME_CHONDROITIN_SULFATE_DERMATAN_SULFATE_METABOLISM
1:155359992	*GBAP1*	REACTOME_CHROMATIN_ORGANIZATION
3:183427301	*MCCC1*	REACTOME_CHROMATIN_ORGANIZATION
6:31862876	*HLA-DRB5*	REACTOME_CHROMATIN_ORGANIZATION
12:46134812	*SCAF11*	REACTOME_CHROMATIN_ORGANIZATION
17:7155861	*V1NB1*	REACTOME_CHROMATIN_ORGANIZATION
17:44160410	*WNT3*	REACTOME_CHROMATIN_ORGANIZATION
17:44189067	*WNT3*	REACTOME_CHROMATIN_ORGANIZATION
19:2172266	*SPPL2B*	REACTOME_CHROMATIN_ORGANIZATION
12:133206648	*FBRSL1*	REACTOME_CHROMOSOME_MAINTENANCE
12:133223685	*FBRSL1*	REACTOME_CHROMOSOME_MAINTENANCE
12:133225045	*FBRSL1*	REACTOME_CHROMOSOME_MAINTENANCE
3:121553904	*KPNA1*	REACTOME_CILIUM_ASSEMBLY
4:15492268	*BST1*	REACTOME_CILIUM_ASSEMBLY
4:15547513	*BST1*	REACTOME_CILIUM_ASSEMBLY
4:15554219	*BST1*	REACTOME_CILIUM_ASSEMBLY
6:30601232	*TRIM40*	REACTOME_CILIUM_ASSEMBLY

8:131153067	*FAM49B*	REACTOME_CILIUM_ASSEMBLY
8:131190319	*FAM49B*	REACTOME_CILIUM_ASSEMBLY
8:131427864	*FAM49B*	REACTOME_CILIUM_ASSEMBLY
10:104005410	*GBF1*	REACTOME_CILIUM_ASSEMBLY
10:104034550	*GBF1*	REACTOME_CILIUM_ASSEMBLY
10:104258303	*GBF1*	REACTOME_CILIUM_ASSEMBLY
10:104443875	*GBF1*	REACTOME_CILIUM_ASSEMBLY
10:104452818	*GBF1*	REACTOME_CILIUM_ASSEMBLY
2:101439596	*MAP4K4*	REACTOME_CIRCADIAN_CLOCK
2:101550181	*MAP4K4*	REACTOME_CIRCADIAN_CLOCK
2:101603812	*MAP4K4*	REACTOME_CIRCADIAN_CLOCK
10:103185542	*GBF1*	REACTOME_CIRCADIAN_CLOCK
10:103308244	*GBF1*	REACTOME_CIRCADIAN_CLOCK
15:61293467	*VPS13C*	REACTOME_CIRCADIAN_CLOCK
3:151090424	*MED12L*	REACTOME_CLASS_A_1_RHODOPSIN_LIKE_RECEPTORS
3:151099733	*MED12L*	REACTOME_CLASS_A_1_RHODOPSIN_LIKE_RECEPTORS
3:151599393	*MED12L*	REACTOME_CLASS_A_1_RHODOPSIN_LIKE_RECEPTORS
12:123110365	*HIP1R*	REACTOME_CLASS_A_1_RHODOPSIN_LIKE_RECEPTORS
12:123110785	*HIP1R*	REACTOME_CLASS_A_1_RHODOPSIN_LIKE_RECEPTORS
12:123139394	*HIP1R*	REACTOME_CLASS_A_1_RHODOPSIN_LIKE_RECEPTORS
12:123155263	*HIP1R*	REACTOME_CLASS_A_1_RHODOPSIN_LIKE_RECEPTORS
12:123194636	*HIP1R*	REACTOME_CLASS_A_1_RHODOPSIN_LIKE_RECEPTORS
14:88472612	*GALC*	REACTOME_CLASS_A_1_RHODOPSIN_LIKE_RECEPTORS
17:43744203	*CRHR1_2*	REACTOME_CLASS_B_2_SECRETIN_FAMILY_RECEPTORS
17:43748712	*CRHR1_2*	REACTOME_CLASS_B_2_SECRETIN_FAMILY_RECEPTORS
17:43767804	*CRHR1_2*	REACTOME_CLASS_B_2_SECRETIN_FAMILY_RECEPTORS
17:43784228	*CRHR1_2*	REACTOME_CLASS_B_2_SECRETIN_FAMILY_RECEPTORS
17:43791502	*CRHR1_2*	REACTOME_CLASS_B_2_SECRETIN_FAMILY_RECEPTORS
17:43843861	*CRHR1_2*	REACTOME_CLASS_B_2_SECRETIN_FAMILY_RECEPTORS
17:43847503	*CRHR1_2*	REACTOME_CLASS_B_2_SECRETIN_FAMILY_RECEPTORS
17:44843651	*WNT3*	REACTOME_CLASS_B_2_SECRETIN_FAMILY_RECEPTORS
17:44888209	*WNT3*	REACTOME_CLASS_B_2_SECRETIN_FAMILY_RECEPTORS
17:44888715	*WNT3*	REACTOME_CLASS_B_2_SECRETIN_FAMILY_RECEPTORS
17:44906949	*WNT3*	REACTOME_CLASS_B_2_SECRETIN_FAMILY_RECEPTORS
17:44912901	*WNT3*	REACTOME_CLASS_B_2_SECRETIN_FAMILY_RECEPTORS
17:44931399	*WNT3*	REACTOME_CLASS_B_2_SECRETIN_FAMILY_RECEPTORS
17:44947756	*WNT3*	REACTOME_CLASS_B_2_SECRETIN_FAMILY_RECEPTORS
6:29579544	*TRIM40*	REACTOME_CLASS_C_3_METABOTROPIC_GLUTAMATE_PHEROMONE_RECEPTORS
3:49740895	*IP6K2*	REACTOME_CLASS_I_MHC_MEDIATED_ANTIGEN_PROCESSING_PRESENTATION
3:121324643	*KPNA1*	REACTOME_CLASS_I_MHC_MEDIATED_ANTIGEN_PROCESSING_PRESENTATION
3:121340955	*KPNA1*	REACTOME_CLASS_I_MHC_MEDIATED_ANTIGEN_PROCESSING_PRESENTATION
3:122289921	*KPNA1*	REACTOME_CLASS_I_MHC_MEDIATED_ANTIGEN_PROCESSING_PRESENTATION
4:15608677	*BST1*	REACTOME_CLASS_I_MHC_MEDIATED_ANTIGEN_PROCESSING_PRESENTATION
4:170015977	*CLCN3*	REACTOME_CLASS_I_MHC_MEDIATED_ANTIGEN_PROCESSING_PRESENTATION
5:133710339	*C5orf24*	REACTOME_CLASS_I_MHC_MEDIATED_ANTIGEN_PROCESSING_PRESENTATION
6:29798642	*TRIM40*	REACTOME_CLASS_I_MHC_MEDIATED_ANTIGEN_PROCESSING_PRESENTATION
6:32789400	*HLA-DRB5*	REACTOME_CLASS_I_MHC_MEDIATED_ANTIGEN_PROCESSING_PRESENTATION
6:32791831	*HLA-DRB5*	REACTOME_CLASS_I_MHC_MEDIATED_ANTIGEN_PROCESSING_PRESENTATION
6:32797361	*HLA-DRB5*	REACTOME_CLASS_I_MHC_MEDIATED_ANTIGEN_PROCESSING_PRESENTATION
6:32802462	*HLA-DRB5*	REACTOME_CLASS_I_MHC_MEDIATED_ANTIGEN_PROCESSING_PRESENTATION
6:32814942	*HLA-DRB5*	REACTOME_CLASS_I_MHC_MEDIATED_ANTIGEN_PROCESSING_PRESENTATION
7:66096028	*GS1-124K5.11*	REACTOME_CLASS_I_MHC_MEDIATED_ANTIGEN_PROCESSING_PRESENTATION
8:12580949	*CTSB*	REACTOME_CLASS_I_MHC_MEDIATED_ANTIGEN_PROCESSING_PRESENTATION

9:33845203	*UBAP2*	REACTOME_CLASS_I_MHC_MEDIATED_ANTIGEN_PROCESSING_PRESENTATION
10:103185542	*GBF1*	REACTOME_CLASS_I_MHC_MEDIATED_ANTIGEN_PROCESSING_PRESENTATION
10:103308244	*GBF1*	REACTOME_CLASS_I_MHC_MEDIATED_ANTIGEN_PROCESSING_PRESENTATION
17:45215865	*WNT3*	REACTOME_CLASS_I_MHC_MEDIATED_ANTIGEN_PROCESSING_PRESENTATION
17:45244822	*WNT3*	REACTOME_CLASS_I_MHC_MEDIATED_ANTIGEN_PROCESSING_PRESENTATION
12:39960237	*LRRK2_2*	REACTOME_CLASS_I_PEROXISOMAL_MEMBRANE_PROTEIN_IMPORT
12:133295501	*FBRSL1*	REACTOME_CLASS_I_PEROXISOMAL_MEMBRANE_PROTEIN_IMPORT
1:155847427	*GBAP1*	REACTOME_CLATHRIN_MEDIATED_ENDOCYTOSIS
1:171689486	*VAMP4*	REACTOME_CLATHRIN_MEDIATED_ENDOCYTOSIS
1:171945488	*VAMP4*	REACTOME_CLATHRIN_MEDIATED_ENDOCYTOSIS
1:172137119	*VAMP4*	REACTOME_CLATHRIN_MEDIATED_ENDOCYTOSIS
1:172386261	*VAMP4*	REACTOME_CLATHRIN_MEDIATED_ENDOCYTOSIS
2:169999639	*STK39*	REACTOME_CLATHRIN_MEDIATED_ENDOCYTOSIS
2:170028024	*STK39*	REACTOME_CLATHRIN_MEDIATED_ENDOCYTOSIS
4:925376	*GAK*	REACTOME_CLATHRIN_MEDIATED_ENDOCYTOSIS
4:77100807	*FAM47E-STBD1*	REACTOME_CLATHRIN_MEDIATED_ENDOCYTOSIS
4:77104152	*FAM47E-STBD1*	REACTOME_CLATHRIN_MEDIATED_ENDOCYTOSIS
4:77124196	*FAM47E-STBD1*	REACTOME_CLATHRIN_MEDIATED_ENDOCYTOSIS
4:77134786	*FAM47E-STBD1*	REACTOME_CLATHRIN_MEDIATED_ENDOCYTOSIS
9:17579690	*SH3GL2_2*	REACTOME_CLATHRIN_MEDIATED_ENDOCYTOSIS
9:17609302	*SH3GL2_2*	REACTOME_CLATHRIN_MEDIATED_ENDOCYTOSIS
9:17627444	*SH3GL2_2*	REACTOME_CLATHRIN_MEDIATED_ENDOCYTOSIS
9:17710876	*SH3GL2_2*	REACTOME_CLATHRIN_MEDIATED_ENDOCYTOSIS
9:17732590	*SH3GL2_2*	REACTOME_CLATHRIN_MEDIATED_ENDOCYTOSIS
9:17737514	*SH3GL2_2*	REACTOME_CLATHRIN_MEDIATED_ENDOCYTOSIS
9:17758945	*SH3GL2_2*	REACTOME_CLATHRIN_MEDIATED_ENDOCYTOSIS
12:123326598	*HIP1R*	REACTOME_CLATHRIN_MEDIATED_ENDOCYTOSIS
17:7129974	*V1NB1*	REACTOME_CLATHRIN_MEDIATED_ENDOCYTOSIS
6:32814942	*HLA-DRB5*	REACTOME_CLEC7A_DECTIN_1_SIGNALING
10:103185542	*GBF1*	REACTOME_CLEC7A_DECTIN_1_SIGNALING
10:103308244	*GBF1*	REACTOME_CLEC7A_DECTIN_1_SIGNALING
17:43390763	*CRHR1_2*	REACTOME_CLEC7A_DECTIN_1_SIGNALING
9:33791164	*UBAP2*	REACTOME_COBALAMIN_CBL_VITAMIN_B12_TRANSPORT_AND_METABOLISM
6:33153528	*HLA-DRB5*	REACTOME_COLLAGEN_BIOSYNTHESIS_AND_MODIFYING_ENZYMES
6:33153528	*HLA-DRB5*	REACTOME_COLLAGEN_CHAIN_TRIMERIZATION
6:33153528	*HLA-DRB5*	REACTOME_COLLAGEN_DEGRADATION
8:11712443	*CTSB*	REACTOME_COLLAGEN_DEGRADATION
6:33153528	*HLA-DRB5*	REACTOME_COLLAGEN_FORMATION
8:11712443	*CTSB*	REACTOME_COLLAGEN_FORMATION
8:23190941	*BIN3*	REACTOME_COLLAGEN_FORMATION
8:23212542	*BIN3*	REACTOME_COLLAGEN_FORMATION
8:23223713	*BIN3*	REACTOME_COLLAGEN_FORMATION
6:31873292	*HLA-DRB5*	REACTOME_COMPLEMENT_CASCADE
6:31879377	*HLA-DRB5*	REACTOME_COMPLEMENT_CASCADE
6:31895092	*HLA-DRB5*	REACTOME_COMPLEMENT_CASCADE
6:31897219	*HLA-DRB5*	REACTOME_COMPLEMENT_CASCADE
1:161171736	*FCGR2A*	REACTOME_COMPLEX_I_BIOGENESIS
5:60273923	*ELOVL7*	REACTOME_COMPLEX_I_BIOGENESIS
6:112009427	*FYN*	REACTOME_CONSTITUTIVE_SIGNALING_BY_ABERRANT_PI3K_IN_CANCER
6:112056759	*FYN*	REACTOME_CONSTITUTIVE_SIGNALING_BY_ABERRANT_PI3K_IN_CANCER
6:112151452	*FYN*	REACTOME_CONSTITUTIVE_SIGNALING_BY_ABERRANT_PI3K_IN_CANCER
17:45215865	*WNT3*	REACTOME_CONVERSION_FROM_APC_C:CDC20_TO_APC_C:CDH1_IN_LATE_ANAPHASE
17:45244822	*WNT3*	REACTOME_CONVERSION_FROM_APC_C:CDC20_TO_APC_C:CDH1_IN_LATE_ANAPHASE

10:104005410	*GBF1*	REACTOME_COPI_DEPENDENT_GOLGI_TO_ER_RETROGRADE_TRAFFIC
10:104034550	*GBF1*	REACTOME_COPI_DEPENDENT_GOLGI_TO_ER_RETROGRADE_TRAFFIC
12:39759090	*LRRK2_2*	REACTOME_COPI_DEPENDENT_GOLGI_TO_ER_RETROGRADE_TRAFFIC
17:43021962	*CRHR1_2*	REACTOME_COPI_DEPENDENT_GOLGI_TO_ER_RETROGRADE_TRAFFIC
17:44800946	*WNT3*	REACTOME_COPI_DEPENDENT_GOLGI_TO_ER_RETROGRADE_TRAFFIC
17:44833217	*WNT3*	REACTOME_COPI_DEPENDENT_GOLGI_TO_ER_RETROGRADE_TRAFFIC
4:76695927	*FAM47E-STBD1*	REACTOME_COPII_MEDIATED_VESICLE_TRANSPORT
10:121683206	*INPP5F*	REACTOME_COPII_MEDIATED_VESICLE_TRANSPORT
10:121686714	*INPP5F*	REACTOME_COPII_MEDIATED_VESICLE_TRANSPORT
10:121689030	*INPP5F*	REACTOME_COPII_MEDIATED_VESICLE_TRANSPORT
17:44800946	*WNT3*	REACTOME_COPII_MEDIATED_VESICLE_TRANSPORT
17:44833217	*WNT3*	REACTOME_COPII_MEDIATED_VESICLE_TRANSPORT
17:45059032	*WNT3*	REACTOME_COPII_MEDIATED_VESICLE_TRANSPORT
17:45089680	*WNT3*	REACTOME_COPII_MEDIATED_VESICLE_TRANSPORT
2:135921205	*TMEM163*	REACTOME_COPI_INDEPENDENT_GOLGI_TO_ER_RETROGRADE_TRAFFIC
10:104258303	*GBF1*	REACTOME_COPI_INDEPENDENT_GOLGI_TO_ER_RETROGRADE_TRAFFIC
3:121411876	*KPNA1*	REACTOME_COPI_MEDIATED_ANTEROGRADE_TRANSPORT
3:121412702	*KPNA1*	REACTOME_COPI_MEDIATED_ANTEROGRADE_TRANSPORT
4:76695927	*FAM47E-STBD1*	REACTOME_COPI_MEDIATED_ANTEROGRADE_TRANSPORT
4:113938109	*CAMK2D*	REACTOME_COPI_MEDIATED_ANTEROGRADE_TRANSPORT
4:114077386	*CAMK2D*	REACTOME_COPI_MEDIATED_ANTEROGRADE_TRANSPORT
4:114217997	*CAMK2D*	REACTOME_COPI_MEDIATED_ANTEROGRADE_TRANSPORT
10:104005410	*GBF1*	REACTOME_COPI_MEDIATED_ANTEROGRADE_TRANSPORT
10:104034550	*GBF1*	REACTOME_COPI_MEDIATED_ANTEROGRADE_TRANSPORT
10:104258303	*GBF1*	REACTOME_COPI_MEDIATED_ANTEROGRADE_TRANSPORT
17:44800946	*WNT3*	REACTOME_COPI_MEDIATED_ANTEROGRADE_TRANSPORT
17:44833217	*WNT3*	REACTOME_COPI_MEDIATED_ANTEROGRADE_TRANSPORT
17:45059032	*WNT3*	REACTOME_COPI_MEDIATED_ANTEROGRADE_TRANSPORT
17:45089680	*WNT3*	REACTOME_COPI_MEDIATED_ANTEROGRADE_TRANSPORT
6:32602372	*HLA-DRB5*	REACTOME_COSTIMULATION_BY_THE_CD28_FAMILY
6:112009427	*FYN*	REACTOME_COSTIMULATION_BY_THE_CD28_FAMILY
6:112056759	*FYN*	REACTOME_COSTIMULATION_BY_THE_CD28_FAMILY
6:112151452	*FYN*	REACTOME_COSTIMULATION_BY_THE_CD28_FAMILY
17:43390763	*CRHR1_2*	REACTOME_COSTIMULATION_BY_THE_CD28_FAMILY
4:114424939	*CAMK2D*	REACTOME_CREB1_PHOSPHORYLATION_THROUGH_NMDA_RECEPTOR_MEDIATED_ACTIVATION_OF_RAS_SIGNALING
11:83188607	*DLG2*	REACTOME_CREB1_PHOSPHORYLATION_THROUGH_NMDA_RECEPTOR_MEDIATED_ACTIVATION_OF_RAS_SIGNALING
11:83219006	*DLG2*	REACTOME_CREB1_PHOSPHORYLATION_THROUGH_NMDA_RECEPTOR_MEDIATED_ACTIVATION_OF_RAS_SIGNALING
11:83538733	*DLG2*	REACTOME_CREB1_PHOSPHORYLATION_THROUGH_NMDA_RECEPTOR_MEDIATED_ACTIVATION_OF_RAS_SIGNALING
11:83544472	*DLG2*	REACTOME_CREB1_PHOSPHORYLATION_THROUGH_NMDA_RECEPTOR_MEDIATED_ACTIVATION_OF_RAS_SIGNALING
11:83656201	*DLG2*	REACTOME_CREB1_PHOSPHORYLATION_THROUGH_NMDA_RECEPTOR_MEDIATED_ACTIVATION_OF_RAS_SIGNALING
11:83700228	*DLG2*	REACTOME_CREB1_PHOSPHORYLATION_THROUGH_NMDA_RECEPTOR_MEDIATED_ACTIVATION_OF_RAS_SIGNALING
11:83762918	*DLG2*	REACTOME_CREB1_PHOSPHORYLATION_THROUGH_NMDA_RECEPTOR_MEDIATED_ACTIVATION_OF_RAS_SIGNALING
11:83894247	*DLG2*	REACTOME_CREB1_PHOSPHORYLATION_THROUGH_NMDA_RECEPTOR_MEDIATED_ACTIVATION_OF_RAS_SIGNALING
11:84021037	*DLG2*	REACTOME_CREB1_PHOSPHORYLATION_THROUGH_NMDA_RECEPTOR_MEDIATED_ACTIVATION_OF_RAS_SIGNALING
11:84324361	*DLG2*	REACTOME_CREB1_PHOSPHORYLATION_THROUGH_NMDA_RECEPTOR_MEDIATED_ACTIVATION_OF_RAS_SIGNALING
11:84373496	*DLG2*	REACTOME_CREB1_PHOSPHORYLATION_THROUGH_NMDA_RECEPTOR_MEDIATED_ACTIVATION_OF_RAS_SIGNALING
11:84403409	*DLG2*	REACTOME_CREB1_PHOSPHORYLATION_THROUGH_NMDA_RECEPTOR_MEDIATED_ACTIVATION_OF_RAS_SIGNALING
17:7101771	*V1NB1*	REACTOME_CREB1_PHOSPHORYLATION_THROUGH_NMDA_RECEPTOR_MEDIATED_ACTIVATION_OF_RAS_SIGNALING
3:48835245	*IP6K2*	REACTOME_CREB1_PHOSPHORYLATION_THROUGH_THE_ACTIVATION_OF_ADENYLATE_CYCLASE
1:155181232	*GBAP1*	REACTOME_CRISTAE_FORMATION
6:112009427	*FYN*	REACTOME_CRMPS_IN_SEMA3A_SIGNALING
6:112056759	*FYN*	REACTOME_CRMPS_IN_SEMA3A_SIGNALING
6:112151452	*FYN*	REACTOME_CRMPS_IN_SEMA3A_SIGNALING

8:23190941	*BIN3*	REACTOME_CROSSLINKING_OF_COLLAGEN_FIBRILS
8:23212542	*BIN3*	REACTOME_CROSSLINKING_OF_COLLAGEN_FIBRILS
8:23223713	*BIN3*	REACTOME_CROSSLINKING_OF_COLLAGEN_FIBRILS
6:32814942	*HLA-DRB5*	REACTOME_CROSS_PRESENTATION_OF_SOLUBLE_EXOGENOUS_ANTIGENS_ENDOSOMES
6:112009427	*FYN*	REACTOME_CTLA4_INHIBITORY_SIGNALING
6:112056759	*FYN*	REACTOME_CTLA4_INHIBITORY_SIGNALING
6:112151452	*FYN*	REACTOME_CTLA4_INHIBITORY_SIGNALING
6:32814942	*HLA-DRB5*	REACTOME_C_TYPE_LECTIN_RECEPTORS_CLRS
6:112009427	*FYN*	REACTOME_C_TYPE_LECTIN_RECEPTORS_CLRS
6:112056759	*FYN*	REACTOME_C_TYPE_LECTIN_RECEPTORS_CLRS
6:112151452	*FYN*	REACTOME_C_TYPE_LECTIN_RECEPTORS_CLRS
10:103185542	*GBF1*	REACTOME_C_TYPE_LECTIN_RECEPTORS_CLRS
10:103308244	*GBF1*	REACTOME_C_TYPE_LECTIN_RECEPTORS_CLRS
17:43390763	*CRHR1_2*	REACTOME_C_TYPE_LECTIN_RECEPTORS_CLRS
3:48199695	*IP6K2*	REACTOME_CYCLIN_A_B1_B2_ASSOCIATED_EVENTS_DURING_G2_M_TRANSITION
3:48214438	*IP6K2*	REACTOME_CYCLIN_A_B1_B2_ASSOCIATED_EVENTS_DURING_G2_M_TRANSITION
3:48199695	*IP6K2*	REACTOME_CYCLIN_A:CDK2_ASSOCIATED_EVENTS_AT_S_PHASE_ENTRY
3:48214438	*IP6K2*	REACTOME_CYCLIN_A:CDK2_ASSOCIATED_EVENTS_AT_S_PHASE_ENTRY
6:32814942	*HLA-DRB5*	REACTOME_CYCLIN_A:CDK2_ASSOCIATED_EVENTS_AT_S_PHASE_ENTRY
2:102609356	*MAP4K4*	REACTOME_CYTOKINE_SIGNALING_IN_IMMUNE_SYSTEM
2:102636759	*MAP4K4*	REACTOME_CYTOKINE_SIGNALING_IN_IMMUNE_SYSTEM
2:103063855	*MAP4K4*	REACTOME_CYTOKINE_SIGNALING_IN_IMMUNE_SYSTEM
3:48748989	*IP6K2*	REACTOME_CYTOKINE_SIGNALING_IN_IMMUNE_SYSTEM
3:48766111	*IP6K2*	REACTOME_CYTOKINE_SIGNALING_IN_IMMUNE_SYSTEM
4:114424939	*CAMK2D*	REACTOME_CYTOKINE_SIGNALING_IN_IMMUNE_SYSTEM
6:29798642	*TRIM40*	REACTOME_CYTOKINE_SIGNALING_IN_IMMUNE_SYSTEM
6:30075864	*TRIM40*	REACTOME_CYTOKINE_SIGNALING_IN_IMMUNE_SYSTEM
6:32151443	*HLA-DRB5*	REACTOME_CYTOKINE_SIGNALING_IN_IMMUNE_SYSTEM
6:32602372	*HLA-DRB5*	REACTOME_CYTOKINE_SIGNALING_IN_IMMUNE_SYSTEM
6:32814942	*HLA-DRB5*	REACTOME_CYTOKINE_SIGNALING_IN_IMMUNE_SYSTEM
6:112009427	*FYN*	REACTOME_CYTOKINE_SIGNALING_IN_IMMUNE_SYSTEM
6:112056759	*FYN*	REACTOME_CYTOKINE_SIGNALING_IN_IMMUNE_SYSTEM
6:112151452	*FYN*	REACTOME_CYTOKINE_SIGNALING_IN_IMMUNE_SYSTEM
8:21665306	*BIN3*	REACTOME_CYTOKINE_SIGNALING_IN_IMMUNE_SYSTEM
8:21668901	*BIN3*	REACTOME_CYTOKINE_SIGNALING_IN_IMMUNE_SYSTEM
10:103185542	*GBF1*	REACTOME_CYTOKINE_SIGNALING_IN_IMMUNE_SYSTEM
10:103308244	*GBF1*	REACTOME_CYTOKINE_SIGNALING_IN_IMMUNE_SYSTEM
11:83188607	*DLG2*	REACTOME_CYTOKINE_SIGNALING_IN_IMMUNE_SYSTEM
11:83219006	*DLG2*	REACTOME_CYTOKINE_SIGNALING_IN_IMMUNE_SYSTEM
11:83538733	*DLG2*	REACTOME_CYTOKINE_SIGNALING_IN_IMMUNE_SYSTEM
11:83544472	*DLG2*	REACTOME_CYTOKINE_SIGNALING_IN_IMMUNE_SYSTEM
11:83656201	*DLG2*	REACTOME_CYTOKINE_SIGNALING_IN_IMMUNE_SYSTEM
11:83700228	*DLG2*	REACTOME_CYTOKINE_SIGNALING_IN_IMMUNE_SYSTEM
11:83762918	*DLG2*	REACTOME_CYTOKINE_SIGNALING_IN_IMMUNE_SYSTEM
11:83894247	*DLG2*	REACTOME_CYTOKINE_SIGNALING_IN_IMMUNE_SYSTEM
11:84021037	*DLG2*	REACTOME_CYTOKINE_SIGNALING_IN_IMMUNE_SYSTEM
11:84324361	*DLG2*	REACTOME_CYTOKINE_SIGNALING_IN_IMMUNE_SYSTEM
11:84373496	*DLG2*	REACTOME_CYTOKINE_SIGNALING_IN_IMMUNE_SYSTEM
11:84403409	*DLG2*	REACTOME_CYTOKINE_SIGNALING_IN_IMMUNE_SYSTEM
15:61293467	*VPS13C*	REACTOME_CYTOKINE_SIGNALING_IN_IMMUNE_SYSTEM
16:31370452	*SETD1A*	REACTOME_CYTOKINE_SIGNALING_IN_IMMUNE_SYSTEM
16:50730088	*CASC16*	REACTOME_CYTOKINE_SIGNALING_IN_IMMUNE_SYSTEM
16:50730446	*CASC16*	REACTOME_CYTOKINE_SIGNALING_IN_IMMUNE_SYSTEM

16:50745926	*CASC16*	REACTOME_CYTOKINE_SIGNALING_IN_IMMUNE_SYSTEM
17:7101771	*V1NB1*	REACTOME_CYTOKINE_SIGNALING_IN_IMMUNE_SYSTEM
17:7479140	*V1NB1*	REACTOME_CYTOKINE_SIGNALING_IN_IMMUNE_SYSTEM
17:7480325	*V1NB1*	REACTOME_CYTOKINE_SIGNALING_IN_IMMUNE_SYSTEM
17:7578671	*V1NB1*	REACTOME_CYTOKINE_SIGNALING_IN_IMMUNE_SYSTEM
17:40392358	*RETREG3*	REACTOME_CYTOKINE_SIGNALING_IN_IMMUNE_SYSTEM
17:43390763	*CRHR1_2*	REACTOME_CYTOKINE_SIGNALING_IN_IMMUNE_SYSTEM
17:59922925	*BRIP1*	REACTOME_CYTOSOLIC_IRON_SULFUR_CLUSTER_ASSEMBLY
7:65705034	*GS1-124K5.11*	REACTOME_CYTOSOLIC_SULFONATION_OF_SMALL_MOLECULES
6:31758240	*HLA-DRB5*	REACTOME_CYTOSOLIC_TRNA_AMINOACYLATION
6:31762311	*HLA-DRB5*	REACTOME_CYTOSOLIC_TRNA_AMINOACYLATION
3:48835245	*IP6K2*	REACTOME_DAG_AND_IP3_SIGNALING
3:123057397	*KPNA1*	REACTOME_DAG_AND_IP3_SIGNALING
4:114424939	*CAMK2D*	REACTOME_DAG_AND_IP3_SIGNALING
6:112009427	*FYN*	REACTOME_DAP12_INTERACTIONS
6:112056759	*FYN*	REACTOME_DAP12_INTERACTIONS
6:112151452	*FYN*	REACTOME_DAP12_INTERACTIONS
6:112009427	*FYN*	REACTOME_DAP12_SIGNALING
6:112056759	*FYN*	REACTOME_DAP12_SIGNALING
6:112151452	*FYN*	REACTOME_DAP12_SIGNALING
3:48835245	*IP6K2*	REACTOME_DARPP_32_EVENTS
5:59236972	*ELOVL7*	REACTOME_DARPP_32_EVENTS
5:59665659	*ELOVL7*	REACTOME_DARPP_32_EVENTS
5:59705316	*ELOVL7*	REACTOME_DARPP_32_EVENTS
5:59736085	*ELOVL7*	REACTOME_DARPP_32_EVENTS
6:112009427	*FYN*	REACTOME_DCC_MEDIATED_ATTRACTIVE_SIGNALING
6:112056759	*FYN*	REACTOME_DCC_MEDIATED_ATTRACTIVE_SIGNALING
6:112151452	*FYN*	REACTOME_DCC_MEDIATED_ATTRACTIVE_SIGNALING
6:32151443	*HLA-DRB5*	REACTOME_DDX58_IFIH1_MEDIATED_INDUCTION_OF_INTERFERON_ALPHA_BETA
10:103185542	*GBF1*	REACTOME_DEACTIVATION_OF_THE_BETA_CATENIN_TRANSACTIVATING_COMPLEX
10:103308244	*GBF1*	REACTOME_DEACTIVATION_OF_THE_BETA_CATENIN_TRANSACTIVATING_COMPLEX
19:3022354	*SPPL2B*	REACTOME_DEACTIVATION_OF_THE_BETA_CATENIN_TRANSACTIVATING_COMPLEX
19:3036350	*SPPL2B*	REACTOME_DEACTIVATION_OF_THE_BETA_CATENIN_TRANSACTIVATING_COMPLEX
17:7479140	*V1NB1*	REACTOME_DEADENYLATION_DEPENDENT_MRNA_DECAY
17:7480325	*V1NB1*	REACTOME_DEADENYLATION_DEPENDENT_MRNA_DECAY
17:7479140	*V1NB1*	REACTOME_DEADENYLATION_OF_MRNA
17:7480325	*V1NB1*	REACTOME_DEADENYLATION_OF_MRNA
1:155975202	*GBAP1*	REACTOME_DEATH_RECEPTOR_SIGNALLING
1:227067656	*ITPKB*	REACTOME_DEATH_RECEPTOR_SIGNALLING
3:49406095	*IP6K2*	REACTOME_DEATH_RECEPTOR_SIGNALLING
3:49424231	*IP6K2*	REACTOME_DEATH_RECEPTOR_SIGNALLING
3:49450137	*IP6K2*	REACTOME_DEATH_RECEPTOR_SIGNALLING
8:23050400	*BIN3*	REACTOME_DEATH_RECEPTOR_SIGNALLING
19:2335872	*SPPL2B*	REACTOME_DEATH_RECEPTOR_SIGNALLING
19:2346990	*SPPL2B*	REACTOME_DEATH_RECEPTOR_SIGNALLING
19:2352757	*SPPL2B*	REACTOME_DEATH_RECEPTOR_SIGNALLING
6:32814942	*HLA-DRB5*	REACTOME_DECTIN_1_MEDIATED_NONCANONICAL_NF_KB_SIGNALING
10:103185542	*GBF1*	REACTOME_DECTIN_1_MEDIATED_NONCANONICAL_NF_KB_SIGNALING
10:103308244	*GBF1*	REACTOME_DECTIN_1_MEDIATED_NONCANONICAL_NF_KB_SIGNALING
17:43390763	*CRHR1_2*	REACTOME_DECTIN_1_MEDIATED_NONCANONICAL_NF_KB_SIGNALING
6:112009427	*FYN*	REACTOME_DECTIN_2_FAMILY
6:112056759	*FYN*	REACTOME_DECTIN_2_FAMILY
6:112151452	*FYN*	REACTOME_DECTIN_2_FAMILY

6:32151443	*HLA-DRB5*	REACTOME_DEFECTIVE_CFTR_CAUSES_CYSTIC_FIBROSIS
6:32814942	*HLA-DRB5*	REACTOME_DEFECTIVE_CFTR_CAUSES_CYSTIC_FIBROSIS
6:32186193	*HLA-DRB5*	REACTOME_DEFECTIVE_LFNG_CAUSES_SCDO3
3:182760073	*MCCC1*	REACTOME_DEFECTS_IN_BIOTIN_BTN_METABOLISM
21:38328886	*DYRK1A*	REACTOME_DEFECTS_IN_BIOTIN_BTN_METABOLISM
3:182760073	*MCCC1*	REACTOME_DEFECTS_IN_VITAMIN_AND_COFACTOR_METABOLISM
21:38328886	*DYRK1A*	REACTOME_DEFECTS_IN_VITAMIN_AND_COFACTOR_METABOLISM
9:33791164	*UBAP2*	REACTOME_DEFENSINS
6:32814942	*HLA-DRB5*	REACTOME_DEGRADATION_OF_AXIN
6:32814942	*HLA-DRB5*	REACTOME_DEGRADATION_OF_BETA_CATENIN_BY_THE_DESTRUCTION_COMPLEX
10:103185542	*GBF1*	REACTOME_DEGRADATION_OF_BETA_CATENIN_BY_THE_DESTRUCTION_COMPLEX
10:103308244	*GBF1*	REACTOME_DEGRADATION_OF_BETA_CATENIN_BY_THE_DESTRUCTION_COMPLEX
19:3022354	*SPPL2B*	REACTOME_DEGRADATION_OF_BETA_CATENIN_BY_THE_DESTRUCTION_COMPLEX
19:3036350	*SPPL2B*	REACTOME_DEGRADATION_OF_BETA_CATENIN_BY_THE_DESTRUCTION_COMPLEX
6:32814942	*HLA-DRB5*	REACTOME_DEGRADATION_OF_DVL
17:7129974	*V1NB1*	REACTOME_DEGRADATION_OF_DVL
6:32814942	*HLA-DRB5*	REACTOME_DEGRADATION_OF_GLI1_BY_THE_PROTEASOME
10:103185542	*GBF1*	REACTOME_DEGRADATION_OF_GLI1_BY_THE_PROTEASOME
10:103308244	*GBF1*	REACTOME_DEGRADATION_OF_GLI1_BY_THE_PROTEASOME
10:104285824	*GBF1*	REACTOME_DEGRADATION_OF_GLI1_BY_THE_PROTEASOME
1:155027082	*GBAP1*	REACTOME_DEGRADATION_OF_THE_EXTRACELLULAR_MATRIX
1:155033317	*GBAP1*	REACTOME_DEGRADATION_OF_THE_EXTRACELLULAR_MATRIX
6:33153528	*HLA-DRB5*	REACTOME_DEGRADATION_OF_THE_EXTRACELLULAR_MATRIX
8:11712443	*CTSB*	REACTOME_DEGRADATION_OF_THE_EXTRACELLULAR_MATRIX
1:156059276	*GBAP1*	REACTOME_DEPOLYMERISATION_OF_THE_NUCLEAR_LAMINA
9:34342881	*UBAP2*	REACTOME_DETOXIFICATION_OF_REACTIVE_OXYGEN_SPECIES
3:48199695	*IP6K2*	REACTOME_DEUBIQUITINATION
3:48214438	*IP6K2*	REACTOME_DEUBIQUITINATION
3:49406095	*IP6K2*	REACTOME_DEUBIQUITINATION
3:49424231	*IP6K2*	REACTOME_DEUBIQUITINATION
3:49450137	*IP6K2*	REACTOME_DEUBIQUITINATION
3:49740895	*IP6K2*	REACTOME_DEUBIQUITINATION
6:32814942	*HLA-DRB5*	REACTOME_DEUBIQUITINATION
16:50730088	*CASC16*	REACTOME_DEUBIQUITINATION
16:50730446	*CASC16*	REACTOME_DEUBIQUITINATION
16:50745926	*CASC16*	REACTOME_DEUBIQUITINATION
17:7578671	*V1NB1*	REACTOME_DEUBIQUITINATION
1:171945488	*VAMP4*	REACTOME_DEVELOPMENTAL_BIOLOGY
1:172137119	*VAMP4*	REACTOME_DEVELOPMENTAL_BIOLOGY
1:172386261	*VAMP4*	REACTOME_DEVELOPMENTAL_BIOLOGY
1:227067656	*ITPKB*	REACTOME_DEVELOPMENTAL_BIOLOGY
3:48835245	*IP6K2*	REACTOME_DEVELOPMENTAL_BIOLOGY
3:49406095	*IP6K2*	REACTOME_DEVELOPMENTAL_BIOLOGY
3:49424231	*IP6K2*	REACTOME_DEVELOPMENTAL_BIOLOGY
3:49450137	*IP6K2*	REACTOME_DEVELOPMENTAL_BIOLOGY
3:49561208	*IP6K2*	REACTOME_DEVELOPMENTAL_BIOLOGY
4:17622910	*LCORL*	REACTOME_DEVELOPMENTAL_BIOLOGY
4:17632603	*LCORL*	REACTOME_DEVELOPMENTAL_BIOLOGY
4:113938109	*CAMK2D*	REACTOME_DEVELOPMENTAL_BIOLOGY
4:114077386	*CAMK2D*	REACTOME_DEVELOPMENTAL_BIOLOGY
4:114217997	*CAMK2D*	REACTOME_DEVELOPMENTAL_BIOLOGY
6:32814942	*HLA-DRB5*	REACTOME_DEVELOPMENTAL_BIOLOGY
6:112009427	*FYN*	REACTOME_DEVELOPMENTAL_BIOLOGY

6:112056759	*FYN*	REACTOME_DEVELOPMENTAL_BIOLOGY
6:112151452	*FYN*	REACTOME_DEVELOPMENTAL_BIOLOGY
8:21665306	*BIN3*	REACTOME_DEVELOPMENTAL_BIOLOGY
8:21668901	*BIN3*	REACTOME_DEVELOPMENTAL_BIOLOGY
9:17579690	*SH3GL2_2*	REACTOME_DEVELOPMENTAL_BIOLOGY
9:17609302	*SH3GL2_2*	REACTOME_DEVELOPMENTAL_BIOLOGY
9:17627444	*SH3GL2_2*	REACTOME_DEVELOPMENTAL_BIOLOGY
9:17710876	*SH3GL2_2*	REACTOME_DEVELOPMENTAL_BIOLOGY
9:17732590	*SH3GL2_2*	REACTOME_DEVELOPMENTAL_BIOLOGY
9:17737514	*SH3GL2_2*	REACTOME_DEVELOPMENTAL_BIOLOGY
9:17758945	*SH3GL2_2*	REACTOME_DEVELOPMENTAL_BIOLOGY
12:41147295	*LRRK2_2*	REACTOME_DEVELOPMENTAL_BIOLOGY
12:41251554	*LRRK2_2*	REACTOME_DEVELOPMENTAL_BIOLOGY
12:41407403	*LRRK2_2*	REACTOME_DEVELOPMENTAL_BIOLOGY
16:31004169	*SETD1A*	REACTOME_DEVELOPMENTAL_BIOLOGY
17:7101771	*V1NB1*	REACTOME_DEVELOPMENTAL_BIOLOGY
17:39883520	*RETREG3*	REACTOME_DEVELOPMENTAL_BIOLOGY
17:7129974	*V1NB1*	REACTOME_DISASSEMBLY_OF_THE_DESTRUCTION_COMPLEX_AND_RECRUITMENT_OF_AXIN_TO_THE_MEMBRANE
17:7491546	*V1NB1*	REACTOME_DISEASES_ASSOCIATED_WITH_N_GLYCOSYLATION_OF_PROTEINS
3:49561208	*IP6K2*	REACTOME_DISEASES_ASSOCIATED_WITH_O_GLYCOSYLATION_OF_PROTEINS
3:122645304	*KPNA1*	REACTOME_DISEASES_ASSOCIATED_WITH_O_GLYCOSYLATION_OF_PROTEINS
6:32186193	*HLA-DRB5*	REACTOME_DISEASES_ASSOCIATED_WITH_O_GLYCOSYLATION_OF_PROTEINS
9:18650547	*SH3GL2_2*	REACTOME_DISEASES_ASSOCIATED_WITH_O_GLYCOSYLATION_OF_PROTEINS
1:156059276	*GBAP1*	REACTOME_DISEASE
1:227067656	*ITPKB*	REACTOME_DISEASE
2:135708115	*TMEM163*	REACTOME_DISEASE
3:48199695	*IP6K2*	REACTOME_DISEASE
3:48214438	*IP6K2*	REACTOME_DISEASE
3:49561208	*IP6K2*	REACTOME_DISEASE
3:122645304	*KPNA1*	REACTOME_DISEASE
3:182760073	*MCCC1*	REACTOME_DISEASE
4:114424939	*CAMK2D*	REACTOME_DISEASE
6:31925679	*HLA-DRB5*	REACTOME_DISEASE
6:32151443	*HLA-DRB5*	REACTOME_DISEASE
6:32186193	*HLA-DRB5*	REACTOME_DISEASE
6:32814942	*HLA-DRB5*	REACTOME_DISEASE
6:112009427	*FYN*	REACTOME_DISEASE
6:112056759	*FYN*	REACTOME_DISEASE
6:112151452	*FYN*	REACTOME_DISEASE
7:65425894	*GS1-124K5.11*	REACTOME_DISEASE
8:17125768	*FGF20*	REACTOME_DISEASE
9:17579690	*SH3GL2_2*	REACTOME_DISEASE
9:17609302	*SH3GL2_2*	REACTOME_DISEASE
9:17627444	*SH3GL2_2*	REACTOME_DISEASE
9:17710876	*SH3GL2_2*	REACTOME_DISEASE
9:17732590	*SH3GL2_2*	REACTOME_DISEASE
9:17737514	*SH3GL2_2*	REACTOME_DISEASE
9:17758945	*SH3GL2_2*	REACTOME_DISEASE
9:18650547	*SH3GL2_2*	REACTOME_DISEASE
10:15199347	*ITGA8*	REACTOME_DISEASE
10:103185542	*GBF1*	REACTOME_DISEASE
10:103308244	*GBF1*	REACTOME_DISEASE
10:104591639	*GBF1*	REACTOME_DISEASE

16:31004169	*SETD1A*	REACTOME_DISEASE
17:7491546	*V1NB1*	REACTOME_DISEASE
17:40392358	*RETREG3*	REACTOME_DISEASE
17:41056283	*RETREG3*	REACTOME_DISEASE
17:42338993	*FAM171A2*	REACTOME_DISEASE
17:43132290	*CRHR1_2*	REACTOME_DISEASE
21:38328886	*DYRK1A*	REACTOME_DISEASE
7:65425894	*GS1-124K5.11*	REACTOME_DISEASES_OF_CARBOHYDRATE_METABOLISM
17:41056283	*RETREG3*	REACTOME_DISEASES_OF_CARBOHYDRATE_METABOLISM
3:49561208	*IP6K2*	REACTOME_DISEASES_OF_GLYCOSYLATION
3:122645304	*KPNA1*	REACTOME_DISEASES_OF_GLYCOSYLATION
6:32186193	*HLA-DRB5*	REACTOME_DISEASES_OF_GLYCOSYLATION
9:18650547	*SH3GL2_2*	REACTOME_DISEASES_OF_GLYCOSYLATION
17:7491546	*V1NB1*	REACTOME_DISEASES_OF_GLYCOSYLATION
3:182760073	*MCCC1*	REACTOME_DISEASES_OF_METABOLISM
7:65425894	*GS1-124K5.11*	REACTOME_DISEASES_OF_METABOLISM
10:104591639	*GBF1*	REACTOME_DISEASES_OF_METABOLISM
17:41056283	*RETREG3*	REACTOME_DISEASES_OF_METABOLISM
21:38328886	*DYRK1A*	REACTOME_DISEASES_OF_METABOLISM
1:156059276	*GBAP1*	REACTOME_DISEASES_OF_SIGNAL_TRANSDUCTION
1:227067656	*ITPKB*	REACTOME_DISEASES_OF_SIGNAL_TRANSDUCTION
4:114424939	*CAMK2D*	REACTOME_DISEASES_OF_SIGNAL_TRANSDUCTION
6:32814942	*HLA-DRB5*	REACTOME_DISEASES_OF_SIGNAL_TRANSDUCTION
6:112009427	*FYN*	REACTOME_DISEASES_OF_SIGNAL_TRANSDUCTION
6:112056759	*FYN*	REACTOME_DISEASES_OF_SIGNAL_TRANSDUCTION
6:112151452	*FYN*	REACTOME_DISEASES_OF_SIGNAL_TRANSDUCTION
17:40392358	*RETREG3*	REACTOME_DISEASES_OF_SIGNAL_TRANSDUCTION
6:32151443	*HLA-DRB5*	REACTOME_DISORDERS_OF_TRANSMEMBRANE_TRANSPORTERS
6:32814942	*HLA-DRB5*	REACTOME_DISORDERS_OF_TRANSMEMBRANE_TRANSPORTERS
17:42338993	*FAM171A2*	REACTOME_DISORDERS_OF_TRANSMEMBRANE_TRANSPORTERS
5:133710339	*C5orf24*	REACTOME_DNA_DAMAGE_BYPASS
6:111628626	*FYN*	REACTOME_DNA_DAMAGE_BYPASS
12:133206648	*FBRSL1*	REACTOME_DNA_DAMAGE_BYPASS
12:133223685	*FBRSL1*	REACTOME_DNA_DAMAGE_BYPASS
12:133225045	*FBRSL1*	REACTOME_DNA_DAMAGE_BYPASS
16:53747395	*CHD9*	REACTOME_DNA_DAMAGE_REVERSAL
16:53842518	*CHD9*	REACTOME_DNA_DAMAGE_REVERSAL
17:7578671	*V1NB1*	REACTOME_DNA_DAMAGE_TELOMERE_STRESS_INDUCED_SENESCENCE
6:133619778	*RPS12*	REACTOME_DNA_DOUBLE_STRAND_BREAK_REPAIR
6:133646494	*RPS12*	REACTOME_DNA_DOUBLE_STRAND_BREAK_REPAIR
6:133788110	*RPS12*	REACTOME_DNA_DOUBLE_STRAND_BREAK_REPAIR
12:133206648	*FBRSL1*	REACTOME_DNA_DOUBLE_STRAND_BREAK_REPAIR
12:133223685	*FBRSL1*	REACTOME_DNA_DOUBLE_STRAND_BREAK_REPAIR
12:133225045	*FBRSL1*	REACTOME_DNA_DOUBLE_STRAND_BREAK_REPAIR
17:7578671	*V1NB1*	REACTOME_DNA_DOUBLE_STRAND_BREAK_REPAIR
17:59922925	*BRIP1*	REACTOME_DNA_DOUBLE_STRAND_BREAK_REPAIR
6:133619778	*RPS12*	REACTOME_DNA_DOUBLE_STRAND_BREAK_RESPONSE
6:133646494	*RPS12*	REACTOME_DNA_DOUBLE_STRAND_BREAK_RESPONSE
6:133788110	*RPS12*	REACTOME_DNA_DOUBLE_STRAND_BREAK_RESPONSE
17:7578671	*V1NB1*	REACTOME_DNA_DOUBLE_STRAND_BREAK_RESPONSE
5:133710339	*C5orf24*	REACTOME_DNA_REPAIR
6:111628626	*FYN*	REACTOME_DNA_REPAIR
6:133619778	*RPS12*	REACTOME_DNA_REPAIR

6:133646494	*RPS12*	REACTOME_DNA_REPAIR
6:133788110	*RPS12*	REACTOME_DNA_REPAIR
12:133206648	*FBRSL1*	REACTOME_DNA_REPAIR
12:133223685	*FBRSL1*	REACTOME_DNA_REPAIR
12:133225045	*FBRSL1*	REACTOME_DNA_REPAIR
16:53747395	*CHD9*	REACTOME_DNA_REPAIR
16:53842518	*CHD9*	REACTOME_DNA_REPAIR
17:7578671	*V1NB1*	REACTOME_DNA_REPAIR
17:59922925	*BRIP1*	REACTOME_DNA_REPAIR
12:133206648	*FBRSL1*	REACTOME_DNA_REPLICATION_INITIATION
12:133223685	*FBRSL1*	REACTOME_DNA_REPLICATION_INITIATION
12:133225045	*FBRSL1*	REACTOME_DNA_REPLICATION_INITIATION
6:32814942	*HLA-DRB5*	REACTOME_DNA_REPLICATION_PRE_INITIATION
12:133206648	*FBRSL1*	REACTOME_DNA_REPLICATION_PRE_INITIATION
12:133223685	*FBRSL1*	REACTOME_DNA_REPLICATION_PRE_INITIATION
12:133225045	*FBRSL1*	REACTOME_DNA_REPLICATION_PRE_INITIATION
20:5934513	*CRLS1*	REACTOME_DNA_REPLICATION_PRE_INITIATION
20:5968737	*CRLS1*	REACTOME_DNA_REPLICATION_PRE_INITIATION
20:5968964	*CRLS1*	REACTOME_DNA_REPLICATION_PRE_INITIATION
6:32814942	*HLA-DRB5*	REACTOME_DNA_REPLICATION
12:133206648	*FBRSL1*	REACTOME_DNA_REPLICATION
12:133223685	*FBRSL1*	REACTOME_DNA_REPLICATION
12:133225045	*FBRSL1*	REACTOME_DNA_REPLICATION
17:45215865	*WNT3*	REACTOME_DNA_REPLICATION
17:45244822	*WNT3*	REACTOME_DNA_REPLICATION
20:5934513	*CRLS1*	REACTOME_DNA_REPLICATION
20:5968737	*CRLS1*	REACTOME_DNA_REPLICATION
20:5968964	*CRLS1*	REACTOME_DNA_REPLICATION
20:5934513	*CRLS1*	REACTOME_DNA_STRAND_ELONGATION
20:5968737	*CRLS1*	REACTOME_DNA_STRAND_ELONGATION
20:5968964	*CRLS1*	REACTOME_DNA_STRAND_ELONGATION
6:32814942	*HLA-DRB5*	REACTOME_DOWNSTREAM_SIGNALING_EVENTS_OF_B_CELL_RECEPTOR_BCR
10:103185542	*GBF1*	REACTOME_DOWNSTREAM_SIGNALING_EVENTS_OF_B_CELL_RECEPTOR_BCR
10:103308244	*GBF1*	REACTOME_DOWNSTREAM_SIGNALING_EVENTS_OF_B_CELL_RECEPTOR_BCR
17:40392358	*RETREG3*	REACTOME_DOWNSTREAM_SIGNAL_TRANSDUCTION
6:32602372	*HLA-DRB5*	REACTOME_DOWNSTREAM_TCR_SIGNALING
6:32814942	*HLA-DRB5*	REACTOME_DOWNSTREAM_TCR_SIGNALING
10:103185542	*GBF1*	REACTOME_DOWNSTREAM_TCR_SIGNALING
10:103308244	*GBF1*	REACTOME_DOWNSTREAM_TCR_SIGNALING
12:133206648	*FBRSL1*	REACTOME_DUAL_INCISION_IN_GG_NER
12:133223685	*FBRSL1*	REACTOME_DUAL_INCISION_IN_GG_NER
12:133225045	*FBRSL1*	REACTOME_DUAL_INCISION_IN_GG_NER
12:133206648	*FBRSL1*	REACTOME_DUAL_INCISION_IN_TC_NER
12:133223685	*FBRSL1*	REACTOME_DUAL_INCISION_IN_TC_NER
12:133225045	*FBRSL1*	REACTOME_DUAL_INCISION_IN_TC_NER
20:5934513	*CRLS1*	REACTOME_E2F_ENABLED_INHIBITION_OF_PRE_REPLICATION_COMPLEX_FORMATION
20:5968737	*CRLS1*	REACTOME_E2F_ENABLED_INHIBITION_OF_PRE_REPLICATION_COMPLEX_FORMATION
20:5968964	*CRLS1*	REACTOME_E2F_ENABLED_INHIBITION_OF_PRE_REPLICATION_COMPLEX_FORMATION
20:5934513	*CRLS1*	REACTOME_E2F_MEDIATED_REGULATION_OF_DNA_REPLICATION
20:5968737	*CRLS1*	REACTOME_E2F_MEDIATED_REGULATION_OF_DNA_REPLICATION
20:5968964	*CRLS1*	REACTOME_E2F_MEDIATED_REGULATION_OF_DNA_REPLICATION
5:133710339	*C5orf24*	REACTOME_E3_UBIQUITIN_LIGASES_UBIQUITINATE_TARGET_PROTEINS
3:49162284	*IP6K2*	REACTOME_ECM_PROTEOGLYCANS

3:49162583	*IP6K2*	REACTOME_ECM_PROTEOGLYCANS
3:49561208	*IP6K2*	REACTOME_ECM_PROTEOGLYCANS
6:32031904	*HLA-DRB5*	REACTOME_ECM_PROTEOGLYCANS
6:32062687	*HLA-DRB5*	REACTOME_ECM_PROTEOGLYCANS
6:112561154	*FYN*	REACTOME_ECM_PROTEOGLYCANS
10:15569598	*ITGA8*	REACTOME_ECM_PROTEOGLYCANS
10:15652368	*ITGA8*	REACTOME_ECM_PROTEOGLYCANS
16:31370452	*SETD1A*	REACTOME_ECM_PROTEOGLYCANS
9:17579690	*SH3GL2_2*	REACTOME_EGFR_DOWNREGULATION
9:17609302	*SH3GL2_2*	REACTOME_EGFR_DOWNREGULATION
9:17627444	*SH3GL2_2*	REACTOME_EGFR_DOWNREGULATION
9:17710876	*SH3GL2_2*	REACTOME_EGFR_DOWNREGULATION
9:17732590	*SH3GL2_2*	REACTOME_EGFR_DOWNREGULATION
9:17737514	*SH3GL2_2*	REACTOME_EGFR_DOWNREGULATION
9:17758945	*SH3GL2_2*	REACTOME_EGFR_DOWNREGULATION
8:23190941	*BIN3*	REACTOME_ELASTIC_FIBRE_FORMATION
8:23212542	*BIN3*	REACTOME_ELASTIC_FIBRE_FORMATION
8:23223713	*BIN3*	REACTOME_ELASTIC_FIBRE_FORMATION
10:15569598	*ITGA8*	REACTOME_ELASTIC_FIBRE_FORMATION
10:15652368	*ITGA8*	REACTOME_ELASTIC_FIBRE_FORMATION
14:75057014	*RPS6KL1*	REACTOME_ELASTIC_FIBRE_FORMATION
8:17125768	*FGF20*	REACTOME_ENDOSOMAL_SORTING_COMPLEX_REQUIRED_FOR_TRANSPORT_ESCRT
6:29798642	*TRIM40*	REACTOME_ENDOSOMAL_VACUOLAR_PATHWAY
13:49914395	*CAB39L*	REACTOME_ENERGY_DEPENDENT_REGULATION_OF_MTOR_BY_LKB1_AMPK
13:49927732	*CAB39L*	REACTOME_ENERGY_DEPENDENT_REGULATION_OF_MTOR_BY_LKB1_AMPK
13:49948786	*CAB39L*	REACTOME_ENERGY_DEPENDENT_REGULATION_OF_MTOR_BY_LKB1_AMPK
13:49960054	*CAB39L*	REACTOME_ENERGY_DEPENDENT_REGULATION_OF_MTOR_BY_LKB1_AMPK
13:49977462	*CAB39L*	REACTOME_ENERGY_DEPENDENT_REGULATION_OF_MTOR_BY_LKB1_AMPK
3:49406095	*IP6K2*	REACTOME_EPHA_MEDIATED_GROWTH_CONE_COLLAPSE
3:49424231	*IP6K2*	REACTOME_EPHA_MEDIATED_GROWTH_CONE_COLLAPSE
3:49450137	*IP6K2*	REACTOME_EPHA_MEDIATED_GROWTH_CONE_COLLAPSE
6:112009427	*FYN*	REACTOME_EPHA_MEDIATED_GROWTH_CONE_COLLAPSE
6:112056759	*FYN*	REACTOME_EPHA_MEDIATED_GROWTH_CONE_COLLAPSE
6:112151452	*FYN*	REACTOME_EPHA_MEDIATED_GROWTH_CONE_COLLAPSE
3:49406095	*IP6K2*	REACTOME_EPHB_MEDIATED_FORWARD_SIGNALING
3:49424231	*IP6K2*	REACTOME_EPHB_MEDIATED_FORWARD_SIGNALING
3:49450137	*IP6K2*	REACTOME_EPHB_MEDIATED_FORWARD_SIGNALING
6:112009427	*FYN*	REACTOME_EPHB_MEDIATED_FORWARD_SIGNALING
6:112056759	*FYN*	REACTOME_EPHB_MEDIATED_FORWARD_SIGNALING
6:112151452	*FYN*	REACTOME_EPHB_MEDIATED_FORWARD_SIGNALING
1:227067656	*ITPKB*	REACTOME_EPH_EPHRIN_MEDIATED_REPULSION_OF_CELLS
6:112009427	*FYN*	REACTOME_EPH_EPHRIN_MEDIATED_REPULSION_OF_CELLS
6:112056759	*FYN*	REACTOME_EPH_EPHRIN_MEDIATED_REPULSION_OF_CELLS
6:112151452	*FYN*	REACTOME_EPH_EPHRIN_MEDIATED_REPULSION_OF_CELLS
1:227067656	*ITPKB*	REACTOME_EPH_EPHRIN_SIGNALING
3:49406095	*IP6K2*	REACTOME_EPH_EPHRIN_SIGNALING
3:49424231	*IP6K2*	REACTOME_EPH_EPHRIN_SIGNALING
3:49450137	*IP6K2*	REACTOME_EPH_EPHRIN_SIGNALING
6:112009427	*FYN*	REACTOME_EPH_EPHRIN_SIGNALING
6:112056759	*FYN*	REACTOME_EPH_EPHRIN_SIGNALING
6:112151452	*FYN*	REACTOME_EPH_EPHRIN_SIGNALING
6:112009427	*FYN*	REACTOME_EPHRIN_SIGNALING
6:112056759	*FYN*	REACTOME_EPHRIN_SIGNALING

6:112151452	*FYN*	REACTOME_EPHRIN_SIGNALING
6:31862876	*HLA-DRB5*	REACTOME_EPIGENETIC_REGULATION_OF_GENE_EXPRESSION
6:33381459	*HLA-DRB5*	REACTOME_EPIGENETIC_REGULATION_OF_GENE_EXPRESSION
3:49406095	*IP6K2*	REACTOME_ERBB2_REGULATES_CELL_MOTILITY
3:49424231	*IP6K2*	REACTOME_ERBB2_REGULATES_CELL_MOTILITY
3:49450137	*IP6K2*	REACTOME_ERBB2_REGULATES_CELL_MOTILITY
6:31862876	*HLA-DRB5*	REACTOME_ERCC6_CSB_AND_EHMT2_G9A_POSITIVELY_REGULATE_RRNA_EXPRESSION
6:32151443	*HLA-DRB5*	REACTOME_ER_QUALITY_CONTROL_COMPARTMENT_ERQC
3:121411876	*KPNA1*	REACTOME_ER_TO_GOLGI_ANTEROGRADE_TRANSPORT
3:121412702	*KPNA1*	REACTOME_ER_TO_GOLGI_ANTEROGRADE_TRANSPORT
4:76695927	*FAM47E-STBD1*	REACTOME_ER_TO_GOLGI_ANTEROGRADE_TRANSPORT
4:113938109	*CAMK2D*	REACTOME_ER_TO_GOLGI_ANTEROGRADE_TRANSPORT
4:114077386	*CAMK2D*	REACTOME_ER_TO_GOLGI_ANTEROGRADE_TRANSPORT
4:114217997	*CAMK2D*	REACTOME_ER_TO_GOLGI_ANTEROGRADE_TRANSPORT
10:104005410	*GBF1*	REACTOME_ER_TO_GOLGI_ANTEROGRADE_TRANSPORT
10:104034550	*GBF1*	REACTOME_ER_TO_GOLGI_ANTEROGRADE_TRANSPORT
10:104258303	*GBF1*	REACTOME_ER_TO_GOLGI_ANTEROGRADE_TRANSPORT
10:121683206	*INPP5F*	REACTOME_ER_TO_GOLGI_ANTEROGRADE_TRANSPORT
10:121686714	*INPP5F*	REACTOME_ER_TO_GOLGI_ANTEROGRADE_TRANSPORT
10:121689030	*INPP5F*	REACTOME_ER_TO_GOLGI_ANTEROGRADE_TRANSPORT
17:44800946	*WNT3*	REACTOME_ER_TO_GOLGI_ANTEROGRADE_TRANSPORT
17:44833217	*WNT3*	REACTOME_ER_TO_GOLGI_ANTEROGRADE_TRANSPORT
17:45059032	*WNT3*	REACTOME_ER_TO_GOLGI_ANTEROGRADE_TRANSPORT
17:45089680	*WNT3*	REACTOME_ER_TO_GOLGI_ANTEROGRADE_TRANSPORT
17:42338993	*FAM171A2*	REACTOME_ERYTHROCYTES_TAKE_UP_OXYGEN_AND_RELEASE_CARBON_DIOXIDE
17:40392358	*RETREG3*	REACTOME_ERYTHROPOIETIN_ACTIVATES_STAT5
14:38061742	*MIPOL1*	REACTOME_ESR_MEDIATED_SIGNALING
14:38061742	*MIPOL1*	REACTOME_ESTROGEN_DEPENDENT_GENE_EXPRESSION
17:7479140	*V1NB1*	REACTOME_EUKARYOTIC_TRANSLATION_INITIATION
17:7480325	*V1NB1*	REACTOME_EUKARYOTIC_TRANSLATION_INITIATION
12:133206648	*FBRSL1*	REACTOME_EXTENSION_OF_TELOMERES
12:133223685	*FBRSL1*	REACTOME_EXTENSION_OF_TELOMERES
12:133225045	*FBRSL1*	REACTOME_EXTENSION_OF_TELOMERES
1:155027082	*GBAP1*	REACTOME_EXTRACELLULAR_MATRIX_ORGANIZATION
1:155033317	*GBAP1*	REACTOME_EXTRACELLULAR_MATRIX_ORGANIZATION
3:49162284	*IP6K2*	REACTOME_EXTRACELLULAR_MATRIX_ORGANIZATION
3:49162583	*IP6K2*	REACTOME_EXTRACELLULAR_MATRIX_ORGANIZATION
3:49561208	*IP6K2*	REACTOME_EXTRACELLULAR_MATRIX_ORGANIZATION
6:30850582	*TRIM40*	REACTOME_EXTRACELLULAR_MATRIX_ORGANIZATION
6:32031904	*HLA-DRB5*	REACTOME_EXTRACELLULAR_MATRIX_ORGANIZATION
6:32062687	*HLA-DRB5*	REACTOME_EXTRACELLULAR_MATRIX_ORGANIZATION
6:33153528	*HLA-DRB5*	REACTOME_EXTRACELLULAR_MATRIX_ORGANIZATION
6:112561154	*FYN*	REACTOME_EXTRACELLULAR_MATRIX_ORGANIZATION
8:11712443	*CTSB*	REACTOME_EXTRACELLULAR_MATRIX_ORGANIZATION
8:23190941	*BIN3*	REACTOME_EXTRACELLULAR_MATRIX_ORGANIZATION
8:23212542	*BIN3*	REACTOME_EXTRACELLULAR_MATRIX_ORGANIZATION
8:23223713	*BIN3*	REACTOME_EXTRACELLULAR_MATRIX_ORGANIZATION
10:15569598	*ITGA8*	REACTOME_EXTRACELLULAR_MATRIX_ORGANIZATION
10:15652368	*ITGA8*	REACTOME_EXTRACELLULAR_MATRIX_ORGANIZATION
11:133947732	*IGSF9B*	REACTOME_EXTRACELLULAR_MATRIX_ORGANIZATION
14:75057014	*RPS6KL1*	REACTOME_EXTRACELLULAR_MATRIX_ORGANIZATION
16:30510571	*SETD1A*	REACTOME_EXTRACELLULAR_MATRIX_ORGANIZATION
16:30516492	*SETD1A*	REACTOME_EXTRACELLULAR_MATRIX_ORGANIZATION

16:30520856	*SETD1A*	REACTOME_EXTRACELLULAR_MATRIX_ORGANIZATION
16:31370452	*SETD1A*	REACTOME_EXTRACELLULAR_MATRIX_ORGANIZATION
3:48835245	*IP6K2*	REACTOME_FACTORS_INVOLVED_IN_MEGAKARYOCYTE_DEVELOPMENT_AND_PLATELET_PRODUCTION
12:39759090	*LRRK2_2*	REACTOME_FACTORS_INVOLVED_IN_MEGAKARYOCYTE_DEVELOPMENT_AND_PLATELET_PRODUCTION
17:7578671	*V1NB1*	REACTOME_FACTORS_INVOLVED_IN_MEGAKARYOCYTE_DEVELOPMENT_AND_PLATELET_PRODUCTION
17:43021962	*CRHR1_2*	REACTOME_FACTORS_INVOLVED_IN_MEGAKARYOCYTE_DEVELOPMENT_AND_PLATELET_PRODUCTION
5:60053220	*ELOVL7*	REACTOME_FATTY_ACID_METABOLISM
6:32126002	*HLA-DRB5*	REACTOME_FATTY_ACID_METABOLISM
17:7983799	*V1NB1*	REACTOME_FATTY_ACID_METABOLISM
17:8019125	*V1NB1*	REACTOME_FATTY_ACID_METABOLISM
19:3156505	*SPPL2B*	REACTOME_FATTY_ACIDS_BOUND_TO_GPR40_FFAR1_REGULATE_INSULIN_SECRETION
5:60053220	*ELOVL7*	REACTOME_FATTY_ACYL_COA_BIOSYNTHESIS
6:32126002	*HLA-DRB5*	REACTOME_FATTY_ACYL_COA_BIOSYNTHESIS
6:32814942	*HLA-DRB5*	REACTOME_FBXL7_DOWN_REGULATES_AURKA_DURING_MITOTIC_ENTRY_AND_IN_EARLY_MITOSIS
6:32814942	*HLA-DRB5*	REACTOME_FC_EPSILON_RECEPTOR_FCERI_SIGNALING
6:112009427	*FYN*	REACTOME_FC_EPSILON_RECEPTOR_FCERI_SIGNALING
6:112056759	*FYN*	REACTOME_FC_EPSILON_RECEPTOR_FCERI_SIGNALING
6:112151452	*FYN*	REACTOME_FC_EPSILON_RECEPTOR_FCERI_SIGNALING
10:103185542	*GBF1*	REACTOME_FC_EPSILON_RECEPTOR_FCERI_SIGNALING
10:103308244	*GBF1*	REACTOME_FC_EPSILON_RECEPTOR_FCERI_SIGNALING
6:32814942	*HLA-DRB5*	REACTOME_FCERI_MEDIATED_NF_KB_ACTIVATION
10:103185542	*GBF1*	REACTOME_FCERI_MEDIATED_NF_KB_ACTIVATION
10:103308244	*GBF1*	REACTOME_FCERI_MEDIATED_NF_KB_ACTIVATION
6:112009427	*FYN*	REACTOME_FCGAMMA_RECEPTOR_FCGR_DEPENDENT_PHAGOCYTOSIS
6:112056759	*FYN*	REACTOME_FCGAMMA_RECEPTOR_FCGR_DEPENDENT_PHAGOCYTOSIS
6:112151452	*FYN*	REACTOME_FCGAMMA_RECEPTOR_FCGR_DEPENDENT_PHAGOCYTOSIS
6:112009427	*FYN*	REACTOME_FCGR_ACTIVATION
6:112056759	*FYN*	REACTOME_FCGR_ACTIVATION
6:112151452	*FYN*	REACTOME_FCGR_ACTIVATION
5:134315853	*C5orf24*	REACTOME_FERTILIZATION
17:40392358	*RETREG3*	REACTOME_FGFR1_MUTANT_RECEPTOR_ACTIVATION
11:82582134	*DLG2*	REACTOME_FORMATION_OF_FIBRIN_CLOT_CLOTTING_CASCADE
11:82648357	*DLG2*	REACTOME_FORMATION_OF_FIBRIN_CLOT_CLOTTING_CASCADE
2:135708115	*TMEM163*	REACTOME_FORMATION_OF_HIV_ELONGATION_COMPLEX_IN_THE_ABSENCE_OF_HIV_TAT
6:31925679	*HLA-DRB5*	REACTOME_FORMATION_OF_HIV_ELONGATION_COMPLEX_IN_THE_ABSENCE_OF_HIV_TAT
17:7578671	*V1NB1*	REACTOME_FORMATION_OF_SENESCENCE_ASSOCIATED_HETEROCHROMATIN_FOCI_SAHF
19:3022354	*SPPL2B*	REACTOME_FORMATION_OF_THE_BETA_CATENIN:TCF_TRANSACTIVATING_COMPLEX
19:3036350	*SPPL2B*	REACTOME_FORMATION_OF_THE_BETA_CATENIN:TCF_TRANSACTIVATING_COMPLEX
17:39883520	*RETREG3*	REACTOME_FORMATION_OF_THE_CORNIFIED_ENVELOPE
6:31925679	*HLA-DRB5*	REACTOME_FORMATION_OF_THE_EARLY_ELONGATION_COMPLEX
4:78175384	*FAM47E-STBD1*	REACTOME_FOXO_MEDIATED_TRANSCRIPTION_OF_CELL_CYCLE_GENES
17:41056283	*RETREG3*	REACTOME_FOXO_MEDIATED_TRANSCRIPTION_OF_OXIDATIVE_STRESS_METABOLIC_AND_NEURONAL_GENES
4:78175384	*FAM47E-STBD1*	REACTOME_FOXO_MEDIATED_TRANSCRIPTION
17:41056283	*RETREG3*	REACTOME_FOXO_MEDIATED_TRANSCRIPTION
19:3156505	*SPPL2B*	REACTOME_FREE_FATTY_ACIDS_REGULATE_INSULIN_SECRETION
3:48199695	*IP6K2*	REACTOME_G0_AND_EARLY_G1
3:48214438	*IP6K2*	REACTOME_G0_AND_EARLY_G1
21:38740824	*DYRK1A*	REACTOME_G0_AND_EARLY_G1
21:38770530	*DYRK1A*	REACTOME_G0_AND_EARLY_G1
3:48199695	*IP6K2*	REACTOME_G1_S_DNA_DAMAGE_CHECKPOINTS
3:48214438	*IP6K2*	REACTOME_G1_S_DNA_DAMAGE_CHECKPOINTS
6:32814942	*HLA-DRB5*	REACTOME_G1_S_DNA_DAMAGE_CHECKPOINTS
17:7578671	*V1NB1*	REACTOME_G1_S_DNA_DAMAGE_CHECKPOINTS

3:48199695	*IP6K2*	REACTOME_G1_S_SPECIFIC_TRANSCRIPTION
3:48214438	*IP6K2*	REACTOME_G1_S_SPECIFIC_TRANSCRIPTION
17:76177567	*DNAH17*	REACTOME_G1_S_SPECIFIC_TRANSCRIPTION
3:48199695	*IP6K2*	REACTOME_G2_M_CHECKPOINTS
3:48214438	*IP6K2*	REACTOME_G2_M_CHECKPOINTS
6:32814942	*HLA-DRB5*	REACTOME_G2_M_CHECKPOINTS
17:7578671	*V1NB1*	REACTOME_G2_M_CHECKPOINTS
17:59922925	*BRIP1*	REACTOME_G2_M_CHECKPOINTS
20:5934513	*CRLS1*	REACTOME_G2_M_CHECKPOINTS
20:5968737	*CRLS1*	REACTOME_G2_M_CHECKPOINTS
20:5968964	*CRLS1*	REACTOME_G2_M_CHECKPOINTS
17:7578671	*V1NB1*	REACTOME_G2_M_DNA_DAMAGE_CHECKPOINT
17:59922925	*BRIP1*	REACTOME_G2_M_DNA_DAMAGE_CHECKPOINT
3:123057397	*KPNA1*	REACTOME_GABA_B_RECEPTOR_ACTIVATION
6:29579544	*TRIM40*	REACTOME_GABA_B_RECEPTOR_ACTIVATION
21:39197323	*DYRK1A*	REACTOME_GABA_B_RECEPTOR_ACTIVATION
3:123057397	*KPNA1*	REACTOME_GABA_RECEPTOR_ACTIVATION
6:29579544	*TRIM40*	REACTOME_GABA_RECEPTOR_ACTIVATION
21:39197323	*DYRK1A*	REACTOME_GABA_RECEPTOR_ACTIVATION
1:155975202	*GBAP1*	REACTOME_G_ALPHA_12_13_SIGNALLING_EVENTS
3:49406095	*IP6K2*	REACTOME_G_ALPHA_12_13_SIGNALLING_EVENTS
3:49424231	*IP6K2*	REACTOME_G_ALPHA_12_13_SIGNALLING_EVENTS
3:49450137	*IP6K2*	REACTOME_G_ALPHA_12_13_SIGNALLING_EVENTS
2:169999639	*STK39*	REACTOME_G_ALPHA_I_SIGNALLING_EVENTS
2:170028024	*STK39*	REACTOME_G_ALPHA_I_SIGNALLING_EVENTS
3:48835245	*IP6K2*	REACTOME_G_ALPHA_I_SIGNALLING_EVENTS
3:123057397	*KPNA1*	REACTOME_G_ALPHA_I_SIGNALLING_EVENTS
3:151090424	*MED12L*	REACTOME_G_ALPHA_I_SIGNALLING_EVENTS
3:151099733	*MED12L*	REACTOME_G_ALPHA_I_SIGNALLING_EVENTS
3:151599393	*MED12L*	REACTOME_G_ALPHA_I_SIGNALLING_EVENTS
4:114424939	*CAMK2D*	REACTOME_G_ALPHA_I_SIGNALLING_EVENTS
5:59236972	*ELOVL7*	REACTOME_G_ALPHA_I_SIGNALLING_EVENTS
5:59665659	*ELOVL7*	REACTOME_G_ALPHA_I_SIGNALLING_EVENTS
5:59705316	*ELOVL7*	REACTOME_G_ALPHA_I_SIGNALLING_EVENTS
5:59736085	*ELOVL7*	REACTOME_G_ALPHA_I_SIGNALLING_EVENTS
6:29579544	*TRIM40*	REACTOME_G_ALPHA_I_SIGNALLING_EVENTS
10:15199347	*ITGA8*	REACTOME_G_ALPHA_I_SIGNALLING_EVENTS
10:121295884	*INPP5F*	REACTOME_G_ALPHA_I_SIGNALLING_EVENTS
12:123110365	*HIP1R*	REACTOME_G_ALPHA_I_SIGNALLING_EVENTS
12:123110785	*HIP1R*	REACTOME_G_ALPHA_I_SIGNALLING_EVENTS
12:123139394	*HIP1R*	REACTOME_G_ALPHA_I_SIGNALLING_EVENTS
12:123155263	*HIP1R*	REACTOME_G_ALPHA_I_SIGNALLING_EVENTS
12:123194636	*HIP1R*	REACTOME_G_ALPHA_I_SIGNALLING_EVENTS
17:43132290	*CRHR1_2*	REACTOME_G_ALPHA_I_SIGNALLING_EVENTS
10:121114292	*INPP5F*	REACTOME_G_ALPHA_Q_SIGNALLING_EVENTS
10:121138071	*INPP5F*	REACTOME_G_ALPHA_Q_SIGNALLING_EVENTS
10:121191186	*INPP5F*	REACTOME_G_ALPHA_Q_SIGNALLING_EVENTS
14:88472612	*GALC*	REACTOME_G_ALPHA_Q_SIGNALLING_EVENTS
19:3156505	*SPPL2B*	REACTOME_G_ALPHA_Q_SIGNALLING_EVENTS
3:123057397	*KPNA1*	REACTOME_G_ALPHA_S_SIGNALLING_EVENTS
5:59236972	*ELOVL7*	REACTOME_G_ALPHA_S_SIGNALLING_EVENTS
5:59665659	*ELOVL7*	REACTOME_G_ALPHA_S_SIGNALLING_EVENTS
5:59705316	*ELOVL7*	REACTOME_G_ALPHA_S_SIGNALLING_EVENTS

5:59736085	*ELOVL7*	REACTOME_G_ALPHA_S_SIGNALLING_EVENTS
6:29351969	*TRIM40*	REACTOME_G_ALPHA_S_SIGNALLING_EVENTS
6:29373213	*TRIM40*	REACTOME_G_ALPHA_S_SIGNALLING_EVENTS
6:29407378	*TRIM40*	REACTOME_G_ALPHA_S_SIGNALLING_EVENTS
6:29412222	*TRIM40*	REACTOME_G_ALPHA_S_SIGNALLING_EVENTS
10:121114292	*INPP5F*	REACTOME_G_ALPHA_S_SIGNALLING_EVENTS
10:121138071	*INPP5F*	REACTOME_G_ALPHA_S_SIGNALLING_EVENTS
10:121191186	*INPP5F*	REACTOME_G_ALPHA_S_SIGNALLING_EVENTS
17:43744203	*CRHR1_2*	REACTOME_G_ALPHA_S_SIGNALLING_EVENTS
17:43748712	*CRHR1_2*	REACTOME_G_ALPHA_S_SIGNALLING_EVENTS
17:43767804	*CRHR1_2*	REACTOME_G_ALPHA_S_SIGNALLING_EVENTS
17:43784228	*CRHR1_2*	REACTOME_G_ALPHA_S_SIGNALLING_EVENTS
17:43791502	*CRHR1_2*	REACTOME_G_ALPHA_S_SIGNALLING_EVENTS
17:43843861	*CRHR1_2*	REACTOME_G_ALPHA_S_SIGNALLING_EVENTS
17:43847503	*CRHR1_2*	REACTOME_G_ALPHA_S_SIGNALLING_EVENTS
3:123057397	*KPNA1*	REACTOME_G_ALPHA_Z_SIGNALLING_EVENTS
12:133206648	*FBRSL1*	REACTOME_GAP_FILLING_DNA_REPAIR_SYNTHESIS_AND_LIGATION_IN_GG_NER
12:133223685	*FBRSL1*	REACTOME_GAP_FILLING_DNA_REPAIR_SYNTHESIS_AND_LIGATION_IN_GG_NER
12:133225045	*FBRSL1*	REACTOME_GAP_FILLING_DNA_REPAIR_SYNTHESIS_AND_LIGATION_IN_GG_NER
3:49406095	*IP6K2*	REACTOME_G_BETA:GAMMA_SIGNALLING_THROUGH_PI3KGAMMA
3:49424231	*IP6K2*	REACTOME_G_BETA:GAMMA_SIGNALLING_THROUGH_PI3KGAMMA
3:49450137	*IP6K2*	REACTOME_G_BETA:GAMMA_SIGNALLING_THROUGH_PI3KGAMMA
1:227754686	*ITPKB*	REACTOME_GENE_EXPRESSION_TRANSCRIPTION
1:227804041	*ITPKB*	REACTOME_GENE_EXPRESSION_TRANSCRIPTION
1:231698854	*SIPA1L2*	REACTOME_GENE_EXPRESSION_TRANSCRIPTION
2:135708115	*TMEM163*	REACTOME_GENE_EXPRESSION_TRANSCRIPTION
4:78175384	*FAM47E-STBD1*	REACTOME_GENE_EXPRESSION_TRANSCRIPTION
4:114424939	*CAMK2D*	REACTOME_GENE_EXPRESSION_TRANSCRIPTION
6:28117331	*LOC100131289*	REACTOME_GENE_EXPRESSION_TRANSCRIPTION
6:28223680	*LOC100131289*	REACTOME_GENE_EXPRESSION_TRANSCRIPTION
6:31862876	*HLA-DRB5*	REACTOME_GENE_EXPRESSION_TRANSCRIPTION
6:31925679	*HLA-DRB5*	REACTOME_GENE_EXPRESSION_TRANSCRIPTION
6:32186193	*HLA-DRB5*	REACTOME_GENE_EXPRESSION_TRANSCRIPTION
6:32814942	*HLA-DRB5*	REACTOME_GENE_EXPRESSION_TRANSCRIPTION
6:32941506	*HLA-DRB5*	REACTOME_GENE_EXPRESSION_TRANSCRIPTION
6:33381459	*HLA-DRB5*	REACTOME_GENE_EXPRESSION_TRANSCRIPTION
8:11381148	*CTSB*	REACTOME_GENE_EXPRESSION_TRANSCRIPTION
8:23050400	*BIN3*	REACTOME_GENE_EXPRESSION_TRANSCRIPTION
11:82886475	*DLG2*	REACTOME_GENE_EXPRESSION_TRANSCRIPTION
12:46134812	*SCAF11*	REACTOME_GENE_EXPRESSION_TRANSCRIPTION
14:55515304	*GCH1*	REACTOME_GENE_EXPRESSION_TRANSCRIPTION
15:61293467	*VPS13C*	REACTOME_GENE_EXPRESSION_TRANSCRIPTION
16:30438930	*SETD1A*	REACTOME_GENE_EXPRESSION_TRANSCRIPTION
16:30510571	*SETD1A*	REACTOME_GENE_EXPRESSION_TRANSCRIPTION
16:30516492	*SETD1A*	REACTOME_GENE_EXPRESSION_TRANSCRIPTION
16:30520856	*SETD1A*	REACTOME_GENE_EXPRESSION_TRANSCRIPTION
17:7578671	*V1NB1*	REACTOME_GENE_EXPRESSION_TRANSCRIPTION
17:41056283	*RETREG3*	REACTOME_GENE_EXPRESSION_TRANSCRIPTION
17:59922925	*BRIP1*	REACTOME_GENE_EXPRESSION_TRANSCRIPTION
6:32602372	*HLA-DRB5*	REACTOME_GENERATION_OF_SECOND_MESSENGER_MOLECULES
1:227754686	*ITPKB*	REACTOME_GENERIC_TRANSCRIPTION_PATHWAY
1:227804041	*ITPKB*	REACTOME_GENERIC_TRANSCRIPTION_PATHWAY
2:135708115	*TMEM163*	REACTOME_GENERIC_TRANSCRIPTION_PATHWAY

4:78175384	*FAM47E-STBD1*	REACTOME_GENERIC_TRANSCRIPTION_PATHWAY
4:114424939	*CAMK2D*	REACTOME_GENERIC_TRANSCRIPTION_PATHWAY
6:28117331	*LOC100131289*	REACTOME_GENERIC_TRANSCRIPTION_PATHWAY
6:28223680	*LOC100131289*	REACTOME_GENERIC_TRANSCRIPTION_PATHWAY
6:31862876	*HLA-DRB5*	REACTOME_GENERIC_TRANSCRIPTION_PATHWAY
6:31925679	*HLA-DRB5*	REACTOME_GENERIC_TRANSCRIPTION_PATHWAY
6:32186193	*HLA-DRB5*	REACTOME_GENERIC_TRANSCRIPTION_PATHWAY
6:32814942	*HLA-DRB5*	REACTOME_GENERIC_TRANSCRIPTION_PATHWAY
6:32941506	*HLA-DRB5*	REACTOME_GENERIC_TRANSCRIPTION_PATHWAY
8:11381148	*CTSB*	REACTOME_GENERIC_TRANSCRIPTION_PATHWAY
8:23050400	*BIN3*	REACTOME_GENERIC_TRANSCRIPTION_PATHWAY
12:46134812	*SCAF11*	REACTOME_GENERIC_TRANSCRIPTION_PATHWAY
14:55515304	*GCH1*	REACTOME_GENERIC_TRANSCRIPTION_PATHWAY
15:61293467	*VPS13C*	REACTOME_GENERIC_TRANSCRIPTION_PATHWAY
16:30438930	*SETD1A*	REACTOME_GENERIC_TRANSCRIPTION_PATHWAY
16:30510571	*SETD1A*	REACTOME_GENERIC_TRANSCRIPTION_PATHWAY
16:30516492	*SETD1A*	REACTOME_GENERIC_TRANSCRIPTION_PATHWAY
16:30520856	*SETD1A*	REACTOME_GENERIC_TRANSCRIPTION_PATHWAY
17:7578671	*V1NB1*	REACTOME_GENERIC_TRANSCRIPTION_PATHWAY
17:41056283	*RETREG3*	REACTOME_GENERIC_TRANSCRIPTION_PATHWAY
17:59922925	*BRIP1*	REACTOME_GENERIC_TRANSCRIPTION_PATHWAY
1:231698854	*SIPA1L2*	REACTOME_GENE_SILENCING_BY_RNA
12:133206648	*FBRSL1*	REACTOME_GLOBAL_GENOME_NUCLEOTIDE_EXCISION_REPAIR_GG_NER
12:133223685	*FBRSL1*	REACTOME_GLOBAL_GENOME_NUCLEOTIDE_EXCISION_REPAIR_GG_NER
12:133225045	*FBRSL1*	REACTOME_GLOBAL_GENOME_NUCLEOTIDE_EXCISION_REPAIR_GG_NER
2:18143082	*KCNS3*	REACTOME_GLUCAGON_LIKE_PEPTIDE_1_GLP1_REGULATES_INSULIN_SECRETION
2:18176120	*KCNS3*	REACTOME_GLUCAGON_LIKE_PEPTIDE_1_GLP1_REGULATES_INSULIN_SECRETION
2:18247777	*KCNS3*	REACTOME_GLUCAGON_LIKE_PEPTIDE_1_GLP1_REGULATES_INSULIN_SECRETION
2:18296870	*KCNS3*	REACTOME_GLUCAGON_LIKE_PEPTIDE_1_GLP1_REGULATES_INSULIN_SECRETION
2:18335515	*KCNS3*	REACTOME_GLUCAGON_LIKE_PEPTIDE_1_GLP1_REGULATES_INSULIN_SECRETION
3:48835245	*IP6K2*	REACTOME_GLUCAGON_LIKE_PEPTIDE_1_GLP1_REGULATES_INSULIN_SECRETION
3:123057397	*KPNA1*	REACTOME_GLUCAGON_LIKE_PEPTIDE_1_GLP1_REGULATES_INSULIN_SECRETION
3:48835245	*IP6K2*	REACTOME_GLUCAGON_SIGNALING_IN_METABOLIC_REGULATION
3:123057397	*KPNA1*	REACTOME_GLUCAGON_SIGNALING_IN_METABOLIC_REGULATION
10:104591639	*GBF1*	REACTOME_GLUCOCORTICOID_BIOSYNTHESIS
17:41056283	*RETREG3*	REACTOME_GLUCONEOGENESIS
17:41056283	*RETREG3*	REACTOME_GLUCOSE_METABOLISM
1:226108200	*ITPKB*	REACTOME_GLUTAMATE_AND_GLUTAMINE_METABOLISM
4:114424939	*CAMK2D*	REACTOME_GLUTAMATE_BINDING_ACTIVATION_OF_AMPA_RECEPTORS_AND_SYNAPTIC_PLASTICITY
17:7101771	*V1NB1*	REACTOME_GLUTAMATE_BINDING_ACTIVATION_OF_AMPA_RECEPTORS_AND_SYNAPTIC_PLASTICITY
17:44800946	*WNT3*	REACTOME_GLUTAMATE_BINDING_ACTIVATION_OF_AMPA_RECEPTORS_AND_SYNAPTIC_PLASTICITY
17:44833217	*WNT3*	REACTOME_GLUTAMATE_BINDING_ACTIVATION_OF_AMPA_RECEPTORS_AND_SYNAPTIC_PLASTICITY
2:96858914	*KCNIP3*	REACTOME_GLYCEROPHOSPHOLIPID_BIOSYNTHESIS
6:31846234	*HLA-DRB5*	REACTOME_GLYCEROPHOSPHOLIPID_BIOSYNTHESIS
12:123561638	*HIP1R*	REACTOME_GLYCEROPHOSPHOLIPID_BIOSYNTHESIS
12:123585705	*HIP1R*	REACTOME_GLYCEROPHOSPHOLIPID_BIOSYNTHESIS
20:6011934	*CRLS1*	REACTOME_GLYCEROPHOSPHOLIPID_BIOSYNTHESIS
17:41056283	*RETREG3*	REACTOME_GLYCOGEN_STORAGE_DISEASES
6:71594785	*RIMS1*	REACTOME_GLYCOSAMINOGLYCAN_METABOLISM
7:65425894	*GS1-124K5.11*	REACTOME_GLYCOSAMINOGLYCAN_METABOLISM
3:160817877	*SPTSSB*	REACTOME_GLYCOSPHINGOLIPID_METABOLISM
14:88393900	*GALC*	REACTOME_GLYCOSPHINGOLIPID_METABOLISM
14:88443553	*GALC*	REACTOME_GLYCOSPHINGOLIPID_METABOLISM

12:133295501	*FBRSL1*	REACTOME_GLYOXYLATE_METABOLISM_AND_GLYCINE_DEGRADATION
3:121411876	*KPNA1*	REACTOME_GOLGI_ASSOCIATED_VESICLE_BIOGENESIS
3:121412702	*KPNA1*	REACTOME_GOLGI_ASSOCIATED_VESICLE_BIOGENESIS
4:925376	*GAK*	REACTOME_GOLGI_ASSOCIATED_VESICLE_BIOGENESIS
9:17579690	*SH3GL2_2*	REACTOME_GOLGI_ASSOCIATED_VESICLE_BIOGENESIS
9:17609302	*SH3GL2_2*	REACTOME_GOLGI_ASSOCIATED_VESICLE_BIOGENESIS
9:17627444	*SH3GL2_2*	REACTOME_GOLGI_ASSOCIATED_VESICLE_BIOGENESIS
9:17710876	*SH3GL2_2*	REACTOME_GOLGI_ASSOCIATED_VESICLE_BIOGENESIS
9:17732590	*SH3GL2_2*	REACTOME_GOLGI_ASSOCIATED_VESICLE_BIOGENESIS
9:17737514	*SH3GL2_2*	REACTOME_GOLGI_ASSOCIATED_VESICLE_BIOGENESIS
9:17758945	*SH3GL2_2*	REACTOME_GOLGI_ASSOCIATED_VESICLE_BIOGENESIS
12:123326598	*HIP1R*	REACTOME_GOLGI_ASSOCIATED_VESICLE_BIOGENESIS
4:76695927	*FAM47E-STBD1*	REACTOME_GOLGI_CISTERNAE_PERICENTRIOLAR_STACK_REORGANIZATION
2:135921205	*TMEM163*	REACTOME_GOLGI_TO_ER_RETROGRADE_TRANSPORT
10:104005410	*GBF1*	REACTOME_GOLGI_TO_ER_RETROGRADE_TRANSPORT
10:104034550	*GBF1*	REACTOME_GOLGI_TO_ER_RETROGRADE_TRANSPORT
10:104258303	*GBF1*	REACTOME_GOLGI_TO_ER_RETROGRADE_TRANSPORT
12:39759090	*LRRK2_2*	REACTOME_GOLGI_TO_ER_RETROGRADE_TRANSPORT
17:43021962	*CRHR1_2*	REACTOME_GOLGI_TO_ER_RETROGRADE_TRANSPORT
17:44800946	*WNT3*	REACTOME_GOLGI_TO_ER_RETROGRADE_TRANSPORT
17:44833217	*WNT3*	REACTOME_GOLGI_TO_ER_RETROGRADE_TRANSPORT
3:151090424	*MED12L*	REACTOME_GPCR_LIGAND_BINDING
3:151099733	*MED12L*	REACTOME_GPCR_LIGAND_BINDING
3:151599393	*MED12L*	REACTOME_GPCR_LIGAND_BINDING
6:29579544	*TRIM40*	REACTOME_GPCR_LIGAND_BINDING
14:88472612	*GALC*	REACTOME_GPCR_LIGAND_BINDING
17:43744203	*CRHR1_2*	REACTOME_GPCR_LIGAND_BINDING
17:43748712	*CRHR1_2*	REACTOME_GPCR_LIGAND_BINDING
17:43767804	*CRHR1_2*	REACTOME_GPCR_LIGAND_BINDING
17:43784228	*CRHR1_2*	REACTOME_GPCR_LIGAND_BINDING
17:43791502	*CRHR1_2*	REACTOME_GPCR_LIGAND_BINDING
17:43843861	*CRHR1_2*	REACTOME_GPCR_LIGAND_BINDING
17:43847503	*CRHR1_2*	REACTOME_GPCR_LIGAND_BINDING
17:44843651	*WNT3*	REACTOME_GPCR_LIGAND_BINDING
17:44888209	*WNT3*	REACTOME_GPCR_LIGAND_BINDING
17:44888715	*WNT3*	REACTOME_GPCR_LIGAND_BINDING
17:44906949	*WNT3*	REACTOME_GPCR_LIGAND_BINDING
17:44912901	*WNT3*	REACTOME_GPCR_LIGAND_BINDING
17:44931399	*WNT3*	REACTOME_GPCR_LIGAND_BINDING
17:44947756	*WNT3*	REACTOME_GPCR_LIGAND_BINDING
3:49406095	*IP6K2*	REACTOME_G_PROTEIN_BETA:GAMMA_SIGNALLING
3:49424231	*IP6K2*	REACTOME_G_PROTEIN_BETA:GAMMA_SIGNALLING
3:49450137	*IP6K2*	REACTOME_G_PROTEIN_BETA:GAMMA_SIGNALLING
6:29579544	*TRIM40*	REACTOME_G_PROTEIN_GATED_POTASSIUM_CHANNELS
21:39197323	*DYRK1A*	REACTOME_G_PROTEIN_GATED_POTASSIUM_CHANNELS
3:48835245	*IP6K2*	REACTOME_G_PROTEIN_MEDIATED_EVENTS
3:123057397	*KPNA1*	REACTOME_G_PROTEIN_MEDIATED_EVENTS
4:114424939	*CAMK2D*	REACTOME_G_PROTEIN_MEDIATED_EVENTS
3:49406095	*IP6K2*	REACTOME_GPVI_MEDIATED_ACTIVATION_CASCADE
3:49424231	*IP6K2*	REACTOME_GPVI_MEDIATED_ACTIVATION_CASCADE
3:49450137	*IP6K2*	REACTOME_GPVI_MEDIATED_ACTIVATION_CASCADE
6:112009427	*FYN*	REACTOME_GPVI_MEDIATED_ACTIVATION_CASCADE
6:112056759	*FYN*	REACTOME_GPVI_MEDIATED_ACTIVATION_CASCADE

6:112151452	*FYN*	REACTOME_GPVI_MEDIATED_ACTIVATION_CASCADE
17:40392358	*RETREG3*	REACTOME_GROWTH_HORMONE_RECEPTOR_SIGNALING
3:183427301	*MCCC1*	REACTOME_HATS_ACETYLATE_HISTONES
17:7155861	*V1NB1*	REACTOME_HATS_ACETYLATE_HISTONES
17:44160410	*WNT3*	REACTOME_HATS_ACETYLATE_HISTONES
17:44189067	*WNT3*	REACTOME_HATS_ACETYLATE_HISTONES
12:133206648	*FBRSL1*	REACTOME_HDR_THROUGH_HOMOLOGOUS_RECOMBINATION_HRR
12:133223685	*FBRSL1*	REACTOME_HDR_THROUGH_HOMOLOGOUS_RECOMBINATION_HRR
12:133225045	*FBRSL1*	REACTOME_HDR_THROUGH_HOMOLOGOUS_RECOMBINATION_HRR
17:59922925	*BRIP1*	REACTOME_HDR_THROUGH_HOMOLOGOUS_RECOMBINATION_HRR
17:59922925	*BRIP1*	REACTOME_HDR_THROUGH_SINGLE_STRAND_ANNEALING_SSA
6:32814942	*HLA-DRB5*	REACTOME_HEDGEHOG_LIGAND_BIOGENESIS
3:48835245	*IP6K2*	REACTOME_HEDGEHOG_OFF_STATE
3:123057397	*KPNA1*	REACTOME_HEDGEHOG_OFF_STATE
6:32814942	*HLA-DRB5*	REACTOME_HEDGEHOG_OFF_STATE
10:103185542	*GBF1*	REACTOME_HEDGEHOG_OFF_STATE
10:103308244	*GBF1*	REACTOME_HEDGEHOG_OFF_STATE
10:104285824	*GBF1*	REACTOME_HEDGEHOG_OFF_STATE
6:32814942	*HLA-DRB5*	REACTOME_HEDGEHOG_ON_STATE
10:104285824	*GBF1*	REACTOME_HEDGEHOG_ON_STATE
3:48835245	*IP6K2*	REACTOME_HEMOSTASIS
3:49406095	*IP6K2*	REACTOME_HEMOSTASIS
3:49424231	*IP6K2*	REACTOME_HEMOSTASIS
3:49450137	*IP6K2*	REACTOME_HEMOSTASIS
3:151090424	*MED12L*	REACTOME_HEMOSTASIS
3:151099733	*MED12L*	REACTOME_HEMOSTASIS
4:90804532	*SNCA_2*	REACTOME_HEMOSTASIS
4:90868355	*SNCA_2*	REACTOME_HEMOSTASIS
6:112009427	*FYN*	REACTOME_HEMOSTASIS
6:112056759	*FYN*	REACTOME_HEMOSTASIS
6:112151452	*FYN*	REACTOME_HEMOSTASIS
8:23050400	*BIN3*	REACTOME_HEMOSTASIS
8:130914130	*FAM49B*	REACTOME_HEMOSTASIS
11:10647977	*RNF141*	REACTOME_HEMOSTASIS
11:10648481	*RNF141*	REACTOME_HEMOSTASIS
11:10660840	*RNF141*	REACTOME_HEMOSTASIS
11:82582134	*DLG2*	REACTOME_HEMOSTASIS
11:82648357	*DLG2*	REACTOME_HEMOSTASIS
11:133947732	*IGSF9B*	REACTOME_HEMOSTASIS
12:39759090	*LRRK2_2*	REACTOME_HEMOSTASIS
16:30510571	*SETD1A*	REACTOME_HEMOSTASIS
16:30516492	*SETD1A*	REACTOME_HEMOSTASIS
16:30520856	*SETD1A*	REACTOME_HEMOSTASIS
16:31370452	*SETD1A*	REACTOME_HEMOSTASIS
17:7560294	*V1NB1*	REACTOME_HEMOSTASIS
17:7578671	*V1NB1*	REACTOME_HEMOSTASIS
17:43021962	*CRHR1_2*	REACTOME_HEMOSTASIS
19:3156505	*SPPL2B*	REACTOME_HEMOSTASIS
6:71594785	*RIMS1*	REACTOME_HEPARAN_SULFATE_HEPARIN_HS_GAG_METABOLISM
7:65425894	*GS1-124K5.11*	REACTOME_HEPARAN_SULFATE_HEPARIN_HS_GAG_METABOLISM
2:135708115	*TMEM163*	REACTOME_HIV_ELONGATION_ARREST_AND_RECOVERY
6:31925679	*HLA-DRB5*	REACTOME_HIV_ELONGATION_ARREST_AND_RECOVERY
2:135708115	*TMEM163*	REACTOME_HIV_INFECTION

6:31925679	*HLA-DRB5*	REACTOME_HIV_INFECTION
6:32814942	*HLA-DRB5*	REACTOME_HIV_INFECTION
6:112009427	*FYN*	REACTOME_HIV_INFECTION
6:112056759	*FYN*	REACTOME_HIV_INFECTION
6:112151452	*FYN*	REACTOME_HIV_INFECTION
8:17125768	*FGF20*	REACTOME_HIV_INFECTION
10:15199347	*ITGA8*	REACTOME_HIV_INFECTION
10:103185542	*GBF1*	REACTOME_HIV_INFECTION
10:103308244	*GBF1*	REACTOME_HIV_INFECTION
17:43132290	*CRHR1_2*	REACTOME_HIV_INFECTION
2:135708115	*TMEM163*	REACTOME_HIV_LIFE_CYCLE
6:31925679	*HLA-DRB5*	REACTOME_HIV_LIFE_CYCLE
8:17125768	*FGF20*	REACTOME_HIV_LIFE_CYCLE
10:15199347	*ITGA8*	REACTOME_HIV_LIFE_CYCLE
17:43132290	*CRHR1_2*	REACTOME_HIV_LIFE_CYCLE
17:59922925	*BRIP1*	REACTOME_HOMOLOGOUS_DNA_PAIRING_AND_STRAND_EXCHANGE
12:133206648	*FBRSL1*	REACTOME_HOMOLOGY_DIRECTED_REPAIR
12:133223685	*FBRSL1*	REACTOME_HOMOLOGY_DIRECTED_REPAIR
12:133225045	*FBRSL1*	REACTOME_HOMOLOGY_DIRECTED_REPAIR
17:59922925	*BRIP1*	REACTOME_HOMOLOGY_DIRECTED_REPAIR
6:32814942	*HLA-DRB5*	REACTOME_HOST_INTERACTIONS_OF_HIV_FACTORS
6:112009427	*FYN*	REACTOME_HOST_INTERACTIONS_OF_HIV_FACTORS
6:112056759	*FYN*	REACTOME_HOST_INTERACTIONS_OF_HIV_FACTORS
6:112151452	*FYN*	REACTOME_HOST_INTERACTIONS_OF_HIV_FACTORS
10:103185542	*GBF1*	REACTOME_HOST_INTERACTIONS_OF_HIV_FACTORS
10:103308244	*GBF1*	REACTOME_HOST_INTERACTIONS_OF_HIV_FACTORS
4:114424939	*CAMK2D*	REACTOME_HSF1_DEPENDENT_TRANSACTIVATION
7:65425894	*GS1-124K5.11*	REACTOME_HS_GAG_DEGRADATION
10:104258303	*GBF1*	REACTOME_HSP90_CHAPERONE_CYCLE_FOR_STEROID_HORMONE_RECEPTORS_SHR
7:65425894	*GS1-124K5.11*	REACTOME_HYALURONAN_METABOLISM
7:65425894	*GS1-124K5.11*	REACTOME_HYALURONAN_UPTAKE_AND_DEGRADATION
6:29798642	*TRIM40*	REACTOME_IMMUNOREGULATORY_INTERACTIONS_BETWEEN_A_LYMPHOID_AND_A_NON_LYMPHOID_CELL
16:30510571	*SETD1A*	REACTOME_IMMUNOREGULATORY_INTERACTIONS_BETWEEN_A_LYMPHOID_AND_A_NON_LYMPHOID_CELL
16:30516492	*SETD1A*	REACTOME_IMMUNOREGULATORY_INTERACTIONS_BETWEEN_A_LYMPHOID_AND_A_NON_LYMPHOID_CELL
16:30520856	*SETD1A*	REACTOME_IMMUNOREGULATORY_INTERACTIONS_BETWEEN_A_LYMPHOID_AND_A_NON_LYMPHOID_CELL
2:135708115	*TMEM163*	REACTOME_INFECTIOUS_DISEASE
6:31925679	*HLA-DRB5*	REACTOME_INFECTIOUS_DISEASE
6:32814942	*HLA-DRB5*	REACTOME_INFECTIOUS_DISEASE
6:112009427	*FYN*	REACTOME_INFECTIOUS_DISEASE
6:112056759	*FYN*	REACTOME_INFECTIOUS_DISEASE
6:112151452	*FYN*	REACTOME_INFECTIOUS_DISEASE
8:17125768	*FGF20*	REACTOME_INFECTIOUS_DISEASE
9:17579690	*SH3GL2_2*	REACTOME_INFECTIOUS_DISEASE
9:17609302	*SH3GL2_2*	REACTOME_INFECTIOUS_DISEASE
9:17627444	*SH3GL2_2*	REACTOME_INFECTIOUS_DISEASE
9:17710876	*SH3GL2_2*	REACTOME_INFECTIOUS_DISEASE
9:17732590	*SH3GL2_2*	REACTOME_INFECTIOUS_DISEASE
9:17737514	*SH3GL2_2*	REACTOME_INFECTIOUS_DISEASE
9:17758945	*SH3GL2_2*	REACTOME_INFECTIOUS_DISEASE
10:15199347	*ITGA8*	REACTOME_INFECTIOUS_DISEASE
10:103185542	*GBF1*	REACTOME_INFECTIOUS_DISEASE
10:103308244	*GBF1*	REACTOME_INFECTIOUS_DISEASE
16:31004169	*SETD1A*	REACTOME_INFECTIOUS_DISEASE

17:43132290	*CRHR1_2*	REACTOME_INFECTIOUS_DISEASE
17:45215865	*WNT3*	TION_OF_THE_PROTEOLYTIC_ACTIVITY_OF_APC_C_REQUIRED_FOR_THE_ONSET_OF_ANAPHASE_BY_MITOTIC_SPINDLE_CHECI
17:45244822	*WNT3*	TION_OF_THE_PROTEOLYTIC_ACTIVITY_OF_APC_C_REQUIRED_FOR_THE_ONSET_OF_ANAPHASE_BY_MITOTIC_SPINDLE_CHECI
6:31897219	*HLA-DRB5*	REACTOME_INITIAL_TRIGGERING_OF_COMPLEMENT
9:17579690	*SH3GL2_2*	REACTOME_INLB_MEDIATED_ENTRY_OF_LISTERIA_MONOCYTOGENES_INTO_HOST_CELL
9:17609302	*SH3GL2_2*	REACTOME_INLB_MEDIATED_ENTRY_OF_LISTERIA_MONOCYTOGENES_INTO_HOST_CELL
9:17627444	*SH3GL2_2*	REACTOME_INLB_MEDIATED_ENTRY_OF_LISTERIA_MONOCYTOGENES_INTO_HOST_CELL
9:17710876	*SH3GL2_2*	REACTOME_INLB_MEDIATED_ENTRY_OF_LISTERIA_MONOCYTOGENES_INTO_HOST_CELL
9:17732590	*SH3GL2_2*	REACTOME_INLB_MEDIATED_ENTRY_OF_LISTERIA_MONOCYTOGENES_INTO_HOST_CELL
9:17737514	*SH3GL2_2*	REACTOME_INLB_MEDIATED_ENTRY_OF_LISTERIA_MONOCYTOGENES_INTO_HOST_CELL
9:17758945	*SH3GL2_2*	REACTOME_INLB_MEDIATED_ENTRY_OF_LISTERIA_MONOCYTOGENES_INTO_HOST_CELL
1:171945488	*VAMP4*	REACTOME_INNATE_IMMUNE_SYSTEM
1:172137119	*VAMP4*	REACTOME_INNATE_IMMUNE_SYSTEM
1:172386261	*VAMP4*	REACTOME_INNATE_IMMUNE_SYSTEM
1:226052830	*ITPKB*	REACTOME_INNATE_IMMUNE_SYSTEM
3:49406095	*IP6K2*	REACTOME_INNATE_IMMUNE_SYSTEM
3:49424231	*IP6K2*	REACTOME_INNATE_IMMUNE_SYSTEM
3:49450137	*IP6K2*	REACTOME_INNATE_IMMUNE_SYSTEM
4:15709252	*BST1*	REACTOME_INNATE_IMMUNE_SYSTEM
4:15713608	*BST1*	REACTOME_INNATE_IMMUNE_SYSTEM
4:15724372	*BST1*	REACTOME_INNATE_IMMUNE_SYSTEM
4:15737882	*BST1*	REACTOME_INNATE_IMMUNE_SYSTEM
6:31873292	*HLA-DRB5*	REACTOME_INNATE_IMMUNE_SYSTEM
6:31879377	*HLA-DRB5*	REACTOME_INNATE_IMMUNE_SYSTEM
6:31895092	*HLA-DRB5*	REACTOME_INNATE_IMMUNE_SYSTEM
6:31897219	*HLA-DRB5*	REACTOME_INNATE_IMMUNE_SYSTEM
6:32151443	*HLA-DRB5*	REACTOME_INNATE_IMMUNE_SYSTEM
6:32814942	*HLA-DRB5*	REACTOME_INNATE_IMMUNE_SYSTEM
6:112009427	*FYN*	REACTOME_INNATE_IMMUNE_SYSTEM
6:112056759	*FYN*	REACTOME_INNATE_IMMUNE_SYSTEM
6:112151452	*FYN*	REACTOME_INNATE_IMMUNE_SYSTEM
6:133025715	*RPS12*	REACTOME_INNATE_IMMUNE_SYSTEM
6:133028870	*RPS12*	REACTOME_INNATE_IMMUNE_SYSTEM
6:133034076	*RPS12*	REACTOME_INNATE_IMMUNE_SYSTEM
7:65425894	*GS1-124K5.11*	REACTOME_INNATE_IMMUNE_SYSTEM
8:11712443	*CTSB*	REACTOME_INNATE_IMMUNE_SYSTEM
9:33791164	*UBAP2*	REACTOME_INNATE_IMMUNE_SYSTEM
10:103185542	*GBF1*	REACTOME_INNATE_IMMUNE_SYSTEM
10:103308244	*GBF1*	REACTOME_INNATE_IMMUNE_SYSTEM
11:10392432	*RNF141*	REACTOME_INNATE_IMMUNE_SYSTEM
11:10487997	*RNF141*	REACTOME_INNATE_IMMUNE_SYSTEM
11:82582134	*DLG2*	REACTOME_INNATE_IMMUNE_SYSTEM
11:82648357	*DLG2*	REACTOME_INNATE_IMMUNE_SYSTEM
12:45755099	*SCAF11*	REACTOME_INNATE_IMMUNE_SYSTEM
12:124219791	*HIP1R*	REACTOME_INNATE_IMMUNE_SYSTEM
16:30510571	*SETD1A*	REACTOME_INNATE_IMMUNE_SYSTEM
16:30516492	*SETD1A*	REACTOME_INNATE_IMMUNE_SYSTEM
16:30520856	*SETD1A*	REACTOME_INNATE_IMMUNE_SYSTEM
16:31370452	*SETD1A*	REACTOME_INNATE_IMMUNE_SYSTEM
16:50730088	*CASC16*	REACTOME_INNATE_IMMUNE_SYSTEM
16:50730446	*CASC16*	REACTOME_INNATE_IMMUNE_SYSTEM
16:50745926	*CASC16*	REACTOME_INNATE_IMMUNE_SYSTEM
17:39883520	*RETREG3*	REACTOME_INNATE_IMMUNE_SYSTEM

17:42423844	*FAM171A2*	REACTOME_INNATE_IMMUNE_SYSTEM
17:43390763	*CRHR1_2*	REACTOME_INNATE_IMMUNE_SYSTEM
1:226910340	*ITPKB*	REACTOME_INOSITOL_PHOSPHATE_METABOLISM
1:226916078	*ITPKB*	REACTOME_INOSITOL_PHOSPHATE_METABOLISM
3:48748989	*IP6K2*	REACTOME_INOSITOL_PHOSPHATE_METABOLISM
3:48766111	*IP6K2*	REACTOME_INOSITOL_PHOSPHATE_METABOLISM
17:43195981	*CRHR1_2*	REACTOME_INOSITOL_PHOSPHATE_METABOLISM
3:150298317	*MED12L*	REACTOME_INSERTION_OF_TAIL_ANCHORED_PROTEINS_INTO_THE_ENDOPLASMIC_RETICULUM_MEMBRANE
6:31607942	*HLA-DRB5*	REACTOME_INSERTION_OF_TAIL_ANCHORED_PROTEINS_INTO_THE_ENDOPLASMIC_RETICULUM_MEMBRANE
12:124219791	*HIP1R*	REACTOME_INSULIN_RECEPTOR_RECYCLING
2:18143082	*KCNS3*	REACTOME_INTEGRATION_OF_ENERGY_METABOLISM
2:18176120	*KCNS3*	REACTOME_INTEGRATION_OF_ENERGY_METABOLISM
2:18247777	*KCNS3*	REACTOME_INTEGRATION_OF_ENERGY_METABOLISM
2:18296870	*KCNS3*	REACTOME_INTEGRATION_OF_ENERGY_METABOLISM
2:18335515	*KCNS3*	REACTOME_INTEGRATION_OF_ENERGY_METABOLISM
3:48835245	*IP6K2*	REACTOME_INTEGRATION_OF_ENERGY_METABOLISM
3:123057397	*KPNA1*	REACTOME_INTEGRATION_OF_ENERGY_METABOLISM
19:3156505	*SPPL2B*	REACTOME_INTEGRATION_OF_ENERGY_METABOLISM
10:15569598	*ITGA8*	REACTOME_INTEGRIN_CELL_SURFACE_INTERACTIONS
10:15652368	*ITGA8*	REACTOME_INTEGRIN_CELL_SURFACE_INTERACTIONS
11:133947732	*IGSF9B*	REACTOME_INTEGRIN_CELL_SURFACE_INTERACTIONS
16:30510571	*SETD1A*	REACTOME_INTEGRIN_CELL_SURFACE_INTERACTIONS
16:30516492	*SETD1A*	REACTOME_INTEGRIN_CELL_SURFACE_INTERACTIONS
16:30520856	*SETD1A*	REACTOME_INTEGRIN_CELL_SURFACE_INTERACTIONS
16:31370452	*SETD1A*	REACTOME_INTEGRIN_CELL_SURFACE_INTERACTIONS
4:113938109	*CAMK2D*	REACTOME_INTERACTION_BETWEEN_L1_AND_ANKYRINS
4:114077386	*CAMK2D*	REACTOME_INTERACTION_BETWEEN_L1_AND_ANKYRINS
4:114217997	*CAMK2D*	REACTOME_INTERACTION_BETWEEN_L1_AND_ANKYRINS
16:30438930	*SETD1A*	REACTOME_INTERCONVERSION_OF_NUCLEOTIDE_DI_AND_TRIPHOSPHATES
3:48748989	*IP6K2*	REACTOME_INTERFERON_ALPHA_BETA_SIGNALING
3:48766111	*IP6K2*	REACTOME_INTERFERON_ALPHA_BETA_SIGNALING
6:29798642	*TRIM40*	REACTOME_INTERFERON_ALPHA_BETA_SIGNALING
4:114424939	*CAMK2D*	REACTOME_INTERFERON_GAMMA_SIGNALING
6:29798642	*TRIM40*	REACTOME_INTERFERON_GAMMA_SIGNALING
6:30075864	*TRIM40*	REACTOME_INTERFERON_GAMMA_SIGNALING
6:32602372	*HLA-DRB5*	REACTOME_INTERFERON_GAMMA_SIGNALING
3:48748989	*IP6K2*	REACTOME_INTERFERON_SIGNALING
3:48766111	*IP6K2*	REACTOME_INTERFERON_SIGNALING
4:114424939	*CAMK2D*	REACTOME_INTERFERON_SIGNALING
6:29798642	*TRIM40*	REACTOME_INTERFERON_SIGNALING
6:30075864	*TRIM40*	REACTOME_INTERFERON_SIGNALING
6:32602372	*HLA-DRB5*	REACTOME_INTERFERON_SIGNALING
17:7479140	*V1NB1*	REACTOME_INTERFERON_SIGNALING
17:7480325	*V1NB1*	REACTOME_INTERFERON_SIGNALING
2:102609356	*MAP4K4*	REACTOME_INTERLEUKIN_10_SIGNALING
2:102636759	*MAP4K4*	REACTOME_INTERLEUKIN_10_SIGNALING
17:40392358	*RETREG3*	REACTOME_INTERLEUKIN_15_SIGNALING
10:103185542	*GBF1*	REACTOME_INTERLEUKIN_17_SIGNALING
10:103308244	*GBF1*	REACTOME_INTERLEUKIN_17_SIGNALING
16:50730088	*CASC16*	REACTOME_INTERLEUKIN_17_SIGNALING
16:50730446	*CASC16*	REACTOME_INTERLEUKIN_17_SIGNALING
16:50745926	*CASC16*	REACTOME_INTERLEUKIN_17_SIGNALING
2:103063855	*MAP4K4*	REACTOME_INTERLEUKIN_18_SIGNALING

2:102609356	*MAP4K4*	REACTOME_INTERLEUKIN_1_FAMILY_SIGNALING
2:102636759	*MAP4K4*	REACTOME_INTERLEUKIN_1_FAMILY_SIGNALING
2:103063855	*MAP4K4*	REACTOME_INTERLEUKIN_1_FAMILY_SIGNALING
6:32151443	*HLA-DRB5*	REACTOME_INTERLEUKIN_1_FAMILY_SIGNALING
6:32814942	*HLA-DRB5*	REACTOME_INTERLEUKIN_1_FAMILY_SIGNALING
10:103185542	*GBF1*	REACTOME_INTERLEUKIN_1_FAMILY_SIGNALING
10:103308244	*GBF1*	REACTOME_INTERLEUKIN_1_FAMILY_SIGNALING
16:50730088	*CASC16*	REACTOME_INTERLEUKIN_1_FAMILY_SIGNALING
16:50730446	*CASC16*	REACTOME_INTERLEUKIN_1_FAMILY_SIGNALING
16:50745926	*CASC16*	REACTOME_INTERLEUKIN_1_FAMILY_SIGNALING
2:102609356	*MAP4K4*	REACTOME_INTERLEUKIN_1_SIGNALING
2:102636759	*MAP4K4*	REACTOME_INTERLEUKIN_1_SIGNALING
6:32151443	*HLA-DRB5*	REACTOME_INTERLEUKIN_1_SIGNALING
6:32814942	*HLA-DRB5*	REACTOME_INTERLEUKIN_1_SIGNALING
10:103185542	*GBF1*	REACTOME_INTERLEUKIN_1_SIGNALING
10:103308244	*GBF1*	REACTOME_INTERLEUKIN_1_SIGNALING
16:50730088	*CASC16*	REACTOME_INTERLEUKIN_1_SIGNALING
16:50730446	*CASC16*	REACTOME_INTERLEUKIN_1_SIGNALING
16:50745926	*CASC16*	REACTOME_INTERLEUKIN_1_SIGNALING
17:40392358	*RETREG3*	REACTOME_INTERLEUKIN_20_FAMILY_SIGNALING
17:40392358	*RETREG3*	REACTOME_INTERLEUKIN_21_SIGNALING
17:40392358	*RETREG3*	REACTOME_INTERLEUKIN_2_FAMILY_SIGNALING
17:40392358	*RETREG3*	REACTOME_INTERLEUKIN_2_SIGNALING
6:112009427	*FYN*	REACTOME_INTERLEUKIN_3_INTERLEUKIN_5_AND_GM_CSF_SIGNALING
6:112056759	*FYN*	REACTOME_INTERLEUKIN_3_INTERLEUKIN_5_AND_GM_CSF_SIGNALING
6:112151452	*FYN*	REACTOME_INTERLEUKIN_3_INTERLEUKIN_5_AND_GM_CSF_SIGNALING
17:40392358	*RETREG3*	REACTOME_INTERLEUKIN_3_INTERLEUKIN_5_AND_GM_CSF_SIGNALING
15:61293467	*VPS13C*	REACTOME_INTERLEUKIN_4_AND_INTERLEUKIN_13_SIGNALING
16:31370452	*SETD1A*	REACTOME_INTERLEUKIN_4_AND_INTERLEUKIN_13_SIGNALING
17:7578671	*V1NB1*	REACTOME_INTERLEUKIN_4_AND_INTERLEUKIN_13_SIGNALING
17:40392358	*RETREG3*	REACTOME_INTERLEUKIN_7_SIGNALING
17:40392358	*RETREG3*	REACTOME_INTERLEUKIN_9_SIGNALING
3:48835245	*IP6K2*	REACTOME_INTRACELLULAR_SIGNALING_BY_SECOND_MESSENGERS
3:123057397	*KPNA1*	REACTOME_INTRACELLULAR_SIGNALING_BY_SECOND_MESSENGERS
4:114424939	*CAMK2D*	REACTOME_INTRACELLULAR_SIGNALING_BY_SECOND_MESSENGERS
6:32814942	*HLA-DRB5*	REACTOME_INTRACELLULAR_SIGNALING_BY_SECOND_MESSENGERS
6:112009427	*FYN*	REACTOME_INTRACELLULAR_SIGNALING_BY_SECOND_MESSENGERS
6:112056759	*FYN*	REACTOME_INTRACELLULAR_SIGNALING_BY_SECOND_MESSENGERS
6:112151452	*FYN*	REACTOME_INTRACELLULAR_SIGNALING_BY_SECOND_MESSENGERS
17:7578671	*V1NB1*	REACTOME_INTRACELLULAR_SIGNALING_BY_SECOND_MESSENGERS
1:171689486	*VAMP4*	REACTOME_INTRA_GOLGI_AND_RETROGRADE_GOLGI_TO_ER_TRAFFIC
2:135921205	*TMEM163*	REACTOME_INTRA_GOLGI_AND_RETROGRADE_GOLGI_TO_ER_TRAFFIC
10:104005410	*GBF1*	REACTOME_INTRA_GOLGI_AND_RETROGRADE_GOLGI_TO_ER_TRAFFIC
10:104034550	*GBF1*	REACTOME_INTRA_GOLGI_AND_RETROGRADE_GOLGI_TO_ER_TRAFFIC
10:104258303	*GBF1*	REACTOME_INTRA_GOLGI_AND_RETROGRADE_GOLGI_TO_ER_TRAFFIC
12:39759090	*LRRK2_2*	REACTOME_INTRA_GOLGI_AND_RETROGRADE_GOLGI_TO_ER_TRAFFIC
17:43021962	*CRHR1_2*	REACTOME_INTRA_GOLGI_AND_RETROGRADE_GOLGI_TO_ER_TRAFFIC
17:44800946	*WNT3*	REACTOME_INTRA_GOLGI_AND_RETROGRADE_GOLGI_TO_ER_TRAFFIC
17:44833217	*WNT3*	REACTOME_INTRA_GOLGI_AND_RETROGRADE_GOLGI_TO_ER_TRAFFIC
17:45059032	*WNT3*	REACTOME_INTRA_GOLGI_AND_RETROGRADE_GOLGI_TO_ER_TRAFFIC
17:45089680	*WNT3*	REACTOME_INTRA_GOLGI_AND_RETROGRADE_GOLGI_TO_ER_TRAFFIC
17:76727455	*DNAH17*	REACTOME_INTRA_GOLGI_AND_RETROGRADE_GOLGI_TO_ER_TRAFFIC
17:44800946	*WNT3*	REACTOME_INTRA_GOLGI_TRAFFIC

17:44833217	*WNT3*	REACTOME_INTRA_GOLGI_TRAFFIC
17:45059032	*WNT3*	REACTOME_INTRA_GOLGI_TRAFFIC
17:45089680	*WNT3*	REACTOME_INTRA_GOLGI_TRAFFIC
17:76727455	*DNAH17*	REACTOME_INTRA_GOLGI_TRAFFIC
17:7578671	*V1NB1*	REACTOME_INTRINSIC_PATHWAY_FOR_APOPTOSIS
17:43132290	*CRHR1_2*	REACTOME_INTRINSIC_PATHWAY_FOR_APOPTOSIS
11:82582134	*DLG2*	REACTOME_INTRINSIC_PATHWAY_OF_FIBRIN_CLOT_FORMATION
11:82648357	*DLG2*	REACTOME_INTRINSIC_PATHWAY_OF_FIBRIN_CLOT_FORMATION
6:29579544	*TRIM40*	REACTOME_INWARDLY_RECTIFYING_KPLUS_CHANNELS
21:39197323	*DYRK1A*	REACTOME_INWARDLY_RECTIFYING_KPLUS_CHANNELS
1:154306180	*GBAP1*	REACTOME_ION_CHANNEL_TRANSPORT
4:114424939	*CAMK2D*	REACTOME_ION_CHANNEL_TRANSPORT
4:170563153	*CLCN3*	REACTOME_ION_CHANNEL_TRANSPORT
4:170569172	*CLCN3*	REACTOME_ION_CHANNEL_TRANSPORT
4:170620812	*CLCN3*	REACTOME_ION_CHANNEL_TRANSPORT
12:45755099	*SCAF11*	REACTOME_ION_CHANNEL_TRANSPORT
12:124219791	*HIP1R*	REACTOME_ION_CHANNEL_TRANSPORT
17:7560294	*V1NB1*	REACTOME_ION_CHANNEL_TRANSPORT
17:40938261	*RETREG3*	REACTOME_ION_CHANNEL_TRANSPORT
19:1811130	*SPPL2B*	REACTOME_ION_CHANNEL_TRANSPORT
4:114424939	*CAMK2D*	REACTOME_ION_HOMEOSTASIS
17:7560294	*V1NB1*	REACTOME_ION_HOMEOSTASIS
17:7101771	*V1NB1*	REACTOME_IONOTROPIC_ACTIVITY_OF_KAINATE_RECEPTORS
1:154306180	*GBAP1*	REACTOME_ION_TRANSPORT_BY_P_TYPE_ATPASES
4:114424939	*CAMK2D*	REACTOME_ION_TRANSPORT_BY_P_TYPE_ATPASES
17:7560294	*V1NB1*	REACTOME_ION_TRANSPORT_BY_P_TYPE_ATPASES
19:1811130	*SPPL2B*	REACTOME_ION_TRANSPORT_BY_P_TYPE_ATPASES
1:156059276	*GBAP1*	REACTOME_IRE1ALPHA_ACTIVATES_CHAPERONES
3:150298317	*MED12L*	REACTOME_IRE1ALPHA_ACTIVATES_CHAPERONES
17:45059032	*WNT3*	REACTOME_IRE1ALPHA_ACTIVATES_CHAPERONES
17:45089680	*WNT3*	REACTOME_IRE1ALPHA_ACTIVATES_CHAPERONES
4:15608677	*BST1*	REACTOME_IRON_UPTAKE_AND_TRANSPORT
12:124219791	*HIP1R*	REACTOME_IRON_UPTAKE_AND_TRANSPORT
16:50730088	*CASC16*	REACTOME_JNK_C_JUN_KINASES_PHOSPHORYLATION_AND_ACTIVATION_MEDIATED_BY_ACTIVATED_HUMAN_TAK1
16:50730446	*CASC16*	REACTOME_JNK_C_JUN_KINASES_PHOSPHORYLATION_AND_ACTIVATION_MEDIATED_BY_ACTIVATED_HUMAN_TAK1
16:50745926	*CASC16*	REACTOME_JNK_C_JUN_KINASES_PHOSPHORYLATION_AND_ACTIVATION_MEDIATED_BY_ACTIVATED_HUMAN_TAK1
17:39883520	*RETREG3*	REACTOME_KERATINIZATION
12:39759090	*LRRK2_2*	REACTOME_KINESINS
17:43021962	*CRHR1_2*	REACTOME_KINESINS
1:171945488	*VAMP4*	REACTOME_L1CAM_INTERACTIONS
1:172137119	*VAMP4*	REACTOME_L1CAM_INTERACTIONS
1:172386261	*VAMP4*	REACTOME_L1CAM_INTERACTIONS
4:113938109	*CAMK2D*	REACTOME_L1CAM_INTERACTIONS
4:114077386	*CAMK2D*	REACTOME_L1CAM_INTERACTIONS
4:114217997	*CAMK2D*	REACTOME_L1CAM_INTERACTIONS
9:17579690	*SH3GL2_2*	REACTOME_L1CAM_INTERACTIONS
9:17609302	*SH3GL2_2*	REACTOME_L1CAM_INTERACTIONS
9:17627444	*SH3GL2_2*	REACTOME_L1CAM_INTERACTIONS
9:17710876	*SH3GL2_2*	REACTOME_L1CAM_INTERACTIONS
9:17732590	*SH3GL2_2*	REACTOME_L1CAM_INTERACTIONS
9:17737514	*SH3GL2_2*	REACTOME_L1CAM_INTERACTIONS
9:17758945	*SH3GL2_2*	REACTOME_L1CAM_INTERACTIONS
12:41147295	*LRRK2_2*	REACTOME_L1CAM_INTERACTIONS

12:41251554	*LRRK2_2*	REACTOME_L1CAM_INTERACTIONS
12:41407403	*LRRK2_2*	REACTOME_L1CAM_INTERACTIONS
17:7101771	*V1NB1*	REACTOME_L1CAM_INTERACTIONS
3:49162284	*IP6K2*	REACTOME_LAMININ_INTERACTIONS
3:49162583	*IP6K2*	REACTOME_LAMININ_INTERACTIONS
6:112561154	*FYN*	REACTOME_LAMININ_INTERACTIONS
16:31004169	*SETD1A*	REACTOME_LGI_ADAM_INTERACTIONS
17:7101771	*V1NB1*	REACTOME_LGI_ADAM_INTERACTIONS
9:17579690	*SH3GL2_2*	REACTOME_LISTERIA_MONOCYTOGENES_ENTRY_INTO_HOST_CELLS
9:17609302	*SH3GL2_2*	REACTOME_LISTERIA_MONOCYTOGENES_ENTRY_INTO_HOST_CELLS
9:17627444	*SH3GL2_2*	REACTOME_LISTERIA_MONOCYTOGENES_ENTRY_INTO_HOST_CELLS
9:17710876	*SH3GL2_2*	REACTOME_LISTERIA_MONOCYTOGENES_ENTRY_INTO_HOST_CELLS
9:17732590	*SH3GL2_2*	REACTOME_LISTERIA_MONOCYTOGENES_ENTRY_INTO_HOST_CELLS
9:17737514	*SH3GL2_2*	REACTOME_LISTERIA_MONOCYTOGENES_ENTRY_INTO_HOST_CELLS
9:17758945	*SH3GL2_2*	REACTOME_LISTERIA_MONOCYTOGENES_ENTRY_INTO_HOST_CELLS
4:114424939	*CAMK2D*	REACTOME_LONG_TERM_POTENTIATION
11:83188607	*DLG2*	REACTOME_LONG_TERM_POTENTIATION
11:83219006	*DLG2*	REACTOME_LONG_TERM_POTENTIATION
11:83538733	*DLG2*	REACTOME_LONG_TERM_POTENTIATION
11:83544472	*DLG2*	REACTOME_LONG_TERM_POTENTIATION
11:83656201	*DLG2*	REACTOME_LONG_TERM_POTENTIATION
11:83700228	*DLG2*	REACTOME_LONG_TERM_POTENTIATION
11:83762918	*DLG2*	REACTOME_LONG_TERM_POTENTIATION
11:83894247	*DLG2*	REACTOME_LONG_TERM_POTENTIATION
11:84021037	*DLG2*	REACTOME_LONG_TERM_POTENTIATION
11:84324361	*DLG2*	REACTOME_LONG_TERM_POTENTIATION
11:84373496	*DLG2*	REACTOME_LONG_TERM_POTENTIATION
11:84403409	*DLG2*	REACTOME_LONG_TERM_POTENTIATION
17:7101771	*V1NB1*	REACTOME_LONG_TERM_POTENTIATION
9:17579690	*SH3GL2_2*	REACTOME_LYSOSOME_VESICLE_BIOGENESIS
9:17609302	*SH3GL2_2*	REACTOME_LYSOSOME_VESICLE_BIOGENESIS
9:17627444	*SH3GL2_2*	REACTOME_LYSOSOME_VESICLE_BIOGENESIS
9:17710876	*SH3GL2_2*	REACTOME_LYSOSOME_VESICLE_BIOGENESIS
9:17732590	*SH3GL2_2*	REACTOME_LYSOSOME_VESICLE_BIOGENESIS
9:17737514	*SH3GL2_2*	REACTOME_LYSOSOME_VESICLE_BIOGENESIS
9:17758945	*SH3GL2_2*	REACTOME_LYSOSOME_VESICLE_BIOGENESIS
12:133295501	*FBRSL1*	REACTOME_MACROAUTOPHAGY
10:103185542	*GBF1*	REACTOME_MAP3K8_TPL2_DEPENDENT_MAPK1_3_ACTIVATION
10:103308244	*GBF1*	REACTOME_MAP3K8_TPL2_DEPENDENT_MAPK1_3_ACTIVATION
6:32814942	*HLA-DRB5*	REACTOME_MAPK6_MAPK4_SIGNALING
4:114424939	*CAMK2D*	REACTOME_MAPK_FAMILY_SIGNALING_CASCADES
6:32814942	*HLA-DRB5*	REACTOME_MAPK_FAMILY_SIGNALING_CASCADES
6:112009427	*FYN*	REACTOME_MAPK_FAMILY_SIGNALING_CASCADES
6:112056759	*FYN*	REACTOME_MAPK_FAMILY_SIGNALING_CASCADES
6:112151452	*FYN*	REACTOME_MAPK_FAMILY_SIGNALING_CASCADES
8:21665306	*BIN3*	REACTOME_MAPK_FAMILY_SIGNALING_CASCADES
8:21668901	*BIN3*	REACTOME_MAPK_FAMILY_SIGNALING_CASCADES
11:83188607	*DLG2*	REACTOME_MAPK_FAMILY_SIGNALING_CASCADES
11:83219006	*DLG2*	REACTOME_MAPK_FAMILY_SIGNALING_CASCADES
11:83538733	*DLG2*	REACTOME_MAPK_FAMILY_SIGNALING_CASCADES
11:83544472	*DLG2*	REACTOME_MAPK_FAMILY_SIGNALING_CASCADES
11:83656201	*DLG2*	REACTOME_MAPK_FAMILY_SIGNALING_CASCADES
11:83700228	*DLG2*	REACTOME_MAPK_FAMILY_SIGNALING_CASCADES

11:83762918	*DLG2*	REACTOME_MAPK_FAMILY_SIGNALING_CASCADES
11:83894247	*DLG2*	REACTOME_MAPK_FAMILY_SIGNALING_CASCADES
11:84021037	*DLG2*	REACTOME_MAPK_FAMILY_SIGNALING_CASCADES
11:84324361	*DLG2*	REACTOME_MAPK_FAMILY_SIGNALING_CASCADES
11:84373496	*DLG2*	REACTOME_MAPK_FAMILY_SIGNALING_CASCADES
11:84403409	*DLG2*	REACTOME_MAPK_FAMILY_SIGNALING_CASCADES
17:7101771	*V1NB1*	REACTOME_MAPK_FAMILY_SIGNALING_CASCADES
1:156059276	*GBAP1*	REACTOME_MEIOSIS
14:75495059	*RPS6KL1*	REACTOME_MEIOSIS
14:75495059	*RPS6KL1*	REACTOME_MEIOTIC_RECOMBINATION
1:156059276	*GBAP1*	REACTOME_MEIOTIC_SYNAPSIS
10:104591639	*GBF1*	REACTOME_METABOLIC_DISORDERS_OF_BIOLOGICAL_OXIDATION_ENZYMES
1:226108200	*ITPKB*	REACTOME_METABOLISM_OF_AMINO_ACIDS_AND_DERIVATIVES
3:182760073	*MCCC1*	REACTOME_METABOLISM_OF_AMINO_ACIDS_AND_DERIVATIVES
6:32814942	*HLA-DRB5*	REACTOME_METABOLISM_OF_AMINO_ACIDS_AND_DERIVATIVES
12:133295501	*FBRSL1*	REACTOME_METABOLISM_OF_AMINO_ACIDS_AND_DERIVATIVES
6:33252561	*HLA-DRB5*	REACTOME_METABOLISM_OF_CARBOHYDRATES
6:71594785	*RIMS1*	REACTOME_METABOLISM_OF_CARBOHYDRATES
7:65425894	*GS1-124K5.11*	REACTOME_METABOLISM_OF_CARBOHYDRATES
17:41056283	*RETREG3*	REACTOME_METABOLISM_OF_CARBOHYDRATES
14:55348869	*GCH1*	REACTOME_METABOLISM_OF_COFACTORS
16:19085298	*SYT17*	REACTOME_METABOLISM_OF_COFACTORS
2:169999639	*STK39*	REACTOME_METABOLISM_OF_FAT_SOLUBLE_VITAMINS
2:170028024	*STK39*	REACTOME_METABOLISM_OF_FAT_SOLUBLE_VITAMINS
2:96858914	*KCNIP3*	REACTOME_METABOLISM_OF_LIPIDS
2:101439596	*MAP4K4*	REACTOME_METABOLISM_OF_LIPIDS
2:101550181	*MAP4K4*	REACTOME_METABOLISM_OF_LIPIDS
2:101603812	*MAP4K4*	REACTOME_METABOLISM_OF_LIPIDS
2:169389091	*STK39*	REACTOME_METABOLISM_OF_LIPIDS
2:169999639	*STK39*	REACTOME_METABOLISM_OF_LIPIDS
2:170028024	*STK39*	REACTOME_METABOLISM_OF_LIPIDS
3:160668220	*SPTSSB*	REACTOME_METABOLISM_OF_LIPIDS
3:160817877	*SPTSSB*	REACTOME_METABOLISM_OF_LIPIDS
3:161068566	*SPTSSB*	REACTOME_METABOLISM_OF_LIPIDS
3:161075189	*SPTSSB*	REACTOME_METABOLISM_OF_LIPIDS
4:17622910	*LCORL*	REACTOME_METABOLISM_OF_LIPIDS
4:17632603	*LCORL*	REACTOME_METABOLISM_OF_LIPIDS
5:60053220	*ELOVL7*	REACTOME_METABOLISM_OF_LIPIDS
6:31846234	*HLA-DRB5*	REACTOME_METABOLISM_OF_LIPIDS
6:32126002	*HLA-DRB5*	REACTOME_METABOLISM_OF_LIPIDS
10:104591639	*GBF1*	REACTOME_METABOLISM_OF_LIPIDS
10:121505277	*INPP5F*	REACTOME_METABOLISM_OF_LIPIDS
10:121538407	*INPP5F*	REACTOME_METABOLISM_OF_LIPIDS
12:123561638	*HIP1R*	REACTOME_METABOLISM_OF_LIPIDS
12:123585705	*HIP1R*	REACTOME_METABOLISM_OF_LIPIDS
14:88393900	*GALC*	REACTOME_METABOLISM_OF_LIPIDS
14:88443553	*GALC*	REACTOME_METABOLISM_OF_LIPIDS
15:61293467	*VPS13C*	REACTOME_METABOLISM_OF_LIPIDS
16:30999462	*SETD1A*	REACTOME_METABOLISM_OF_LIPIDS
17:7983799	*V1NB1*	REACTOME_METABOLISM_OF_LIPIDS
17:8019125	*V1NB1*	REACTOME_METABOLISM_OF_LIPIDS
20:6011934	*CRLS1*	REACTOME_METABOLISM_OF_LIPIDS
10:104847713	*GBF1*	REACTOME_METABOLISM_OF_NUCLEOTIDES

10:104861736	*GBF1*	REACTOME_METABOLISM_OF_NUCLEOTIDES
10:104926485	*GBF1*	REACTOME_METABOLISM_OF_NUCLEOTIDES
10:104929716	*GBF1*	REACTOME_METABOLISM_OF_NUCLEOTIDES
11:10392432	*RNF141*	REACTOME_METABOLISM_OF_NUCLEOTIDES
11:10487997	*RNF141*	REACTOME_METABOLISM_OF_NUCLEOTIDES
14:74477206	*RPS6KL1*	REACTOME_METABOLISM_OF_NUCLEOTIDES
16:30438930	*SETD1A*	REACTOME_METABOLISM_OF_NUCLEOTIDES
17:76177567	*DNAH17*	REACTOME_METABOLISM_OF_NUCLEOTIDES
6:32814942	*HLA-DRB5*	REACTOME_METABOLISM_OF_POLYAMINES
6:30628082	*TRIM40*	REACTOME_METABOLISM_OF_RNA
6:30631063	*TRIM40*	REACTOME_METABOLISM_OF_RNA
6:30639369	*TRIM40*	REACTOME_METABOLISM_OF_RNA
6:32814942	*HLA-DRB5*	REACTOME_METABOLISM_OF_RNA
6:33252561	*HLA-DRB5*	REACTOME_METABOLISM_OF_RNA
7:23382980	*GPNMB*	REACTOME_METABOLISM_OF_RNA
7:23385251	*GPNMB*	REACTOME_METABOLISM_OF_RNA
7:23387110	*GPNMB*	REACTOME_METABOLISM_OF_RNA
7:23446436	*GPNMB*	REACTOME_METABOLISM_OF_RNA
7:23495961	*GPNMB*	REACTOME_METABOLISM_OF_RNA
11:82886475	*DLG2*	REACTOME_METABOLISM_OF_RNA
17:7479140	*V1NB1*	REACTOME_METABOLISM_OF_RNA
17:7480325	*V1NB1*	REACTOME_METABOLISM_OF_RNA
17:42929985	*CRHR1_2*	REACTOME_METABOLISM_OF_RNA
10:104591639	*GBF1*	REACTOME_METABOLISM_OF_STEROID_HORMONES
2:169999639	*STK39*	REACTOME_METABOLISM_OF_STEROIDS
2:170028024	*STK39*	REACTOME_METABOLISM_OF_STEROIDS
10:104591639	*GBF1*	REACTOME_METABOLISM_OF_STEROIDS
16:30999462	*SETD1A*	REACTOME_METABOLISM_OF_STEROIDS
2:169999639	*STK39*	REACTOME_METABOLISM_OF_VITAMINS_AND_COFACTORS
2:170028024	*STK39*	REACTOME_METABOLISM_OF_VITAMINS_AND_COFACTORS
3:122259640	*KPNA1*	REACTOME_METABOLISM_OF_VITAMINS_AND_COFACTORS
3:182760073	*MCCC1*	REACTOME_METABOLISM_OF_VITAMINS_AND_COFACTORS
4:15709252	*BST1*	REACTOME_METABOLISM_OF_VITAMINS_AND_COFACTORS
4:15713608	*BST1*	REACTOME_METABOLISM_OF_VITAMINS_AND_COFACTORS
4:15724372	*BST1*	REACTOME_METABOLISM_OF_VITAMINS_AND_COFACTORS
4:15737882	*BST1*	REACTOME_METABOLISM_OF_VITAMINS_AND_COFACTORS
4:15789288	*BST1*	REACTOME_METABOLISM_OF_VITAMINS_AND_COFACTORS
4:15816670	*BST1*	REACTOME_METABOLISM_OF_VITAMINS_AND_COFACTORS
4:15829612	*BST1*	REACTOME_METABOLISM_OF_VITAMINS_AND_COFACTORS
6:133025715	*RPS12*	REACTOME_METABOLISM_OF_VITAMINS_AND_COFACTORS
6:133028870	*RPS12*	REACTOME_METABOLISM_OF_VITAMINS_AND_COFACTORS
6:133034076	*RPS12*	REACTOME_METABOLISM_OF_VITAMINS_AND_COFACTORS
9:33791164	*UBAP2*	REACTOME_METABOLISM_OF_VITAMINS_AND_COFACTORS
14:55348869	*GCH1*	REACTOME_METABOLISM_OF_VITAMINS_AND_COFACTORS
16:19085298	*SYT17*	REACTOME_METABOLISM_OF_VITAMINS_AND_COFACTORS
21:38328886	*DYRK1A*	REACTOME_METABOLISM_OF_VITAMINS_AND_COFACTORS
3:122259640	*KPNA1*	REACTOME_METABOLISM_OF_WATER_SOLUBLE_VITAMINS_AND_COFACTORS
3:182760073	*MCCC1*	REACTOME_METABOLISM_OF_WATER_SOLUBLE_VITAMINS_AND_COFACTORS
4:15709252	*BST1*	REACTOME_METABOLISM_OF_WATER_SOLUBLE_VITAMINS_AND_COFACTORS
4:15713608	*BST1*	REACTOME_METABOLISM_OF_WATER_SOLUBLE_VITAMINS_AND_COFACTORS
4:15724372	*BST1*	REACTOME_METABOLISM_OF_WATER_SOLUBLE_VITAMINS_AND_COFACTORS
4:15737882	*BST1*	REACTOME_METABOLISM_OF_WATER_SOLUBLE_VITAMINS_AND_COFACTORS
4:15789288	*BST1*	REACTOME_METABOLISM_OF_WATER_SOLUBLE_VITAMINS_AND_COFACTORS

4:15816670	*BST1*	REACTOME_METABOLISM_OF_WATER_SOLUBLE_VITAMINS_AND_COFACTORS
4:15829612	*BST1*	REACTOME_METABOLISM_OF_WATER_SOLUBLE_VITAMINS_AND_COFACTORS
6:133025715	*RPS12*	REACTOME_METABOLISM_OF_WATER_SOLUBLE_VITAMINS_AND_COFACTORS
6:133028870	*RPS12*	REACTOME_METABOLISM_OF_WATER_SOLUBLE_VITAMINS_AND_COFACTORS
6:133034076	*RPS12*	REACTOME_METABOLISM_OF_WATER_SOLUBLE_VITAMINS_AND_COFACTORS
9:33791164	*UBAP2*	REACTOME_METABOLISM_OF_WATER_SOLUBLE_VITAMINS_AND_COFACTORS
21:38328886	*DYRK1A*	REACTOME_METABOLISM_OF_WATER_SOLUBLE_VITAMINS_AND_COFACTORS
3:49162284	*IP6K2*	REACTOME_MET_ACTIVATES_PTK2_SIGNALING
3:49162583	*IP6K2*	REACTOME_MET_ACTIVATES_PTK2_SIGNALING
6:33153528	*HLA-DRB5*	REACTOME_MET_ACTIVATES_PTK2_SIGNALING
6:112561154	*FYN*	REACTOME_MET_ACTIVATES_PTK2_SIGNALING
10:104635620	*GBF1*	REACTOME_METHYLATION
3:49162284	*IP6K2*	REACTOME_MET_PROMOTES_CELL_MOTILITY
3:49162583	*IP6K2*	REACTOME_MET_PROMOTES_CELL_MOTILITY
6:33153528	*HLA-DRB5*	REACTOME_MET_PROMOTES_CELL_MOTILITY
6:112561154	*FYN*	REACTOME_MET_PROMOTES_CELL_MOTILITY
1:171945488	*VAMP4*	REACTOME_MHC_CLASS_II_ANTIGEN_PRESENTATION
1:172137119	*VAMP4*	REACTOME_MHC_CLASS_II_ANTIGEN_PRESENTATION
1:172386261	*VAMP4*	REACTOME_MHC_CLASS_II_ANTIGEN_PRESENTATION
6:32602372	*HLA-DRB5*	REACTOME_MHC_CLASS_II_ANTIGEN_PRESENTATION
6:32930679	*HLA-DRB5*	REACTOME_MHC_CLASS_II_ANTIGEN_PRESENTATION
6:32934409	*HLA-DRB5*	REACTOME_MHC_CLASS_II_ANTIGEN_PRESENTATION
6:32973303	*HLA-DRB5*	REACTOME_MHC_CLASS_II_ANTIGEN_PRESENTATION
6:32974268	*HLA-DRB5*	REACTOME_MHC_CLASS_II_ANTIGEN_PRESENTATION
8:11712443	*CTSB*	REACTOME_MHC_CLASS_II_ANTIGEN_PRESENTATION
9:17579690	*SH3GL2_2*	REACTOME_MHC_CLASS_II_ANTIGEN_PRESENTATION
9:17609302	*SH3GL2_2*	REACTOME_MHC_CLASS_II_ANTIGEN_PRESENTATION
9:17627444	*SH3GL2_2*	REACTOME_MHC_CLASS_II_ANTIGEN_PRESENTATION
9:17710876	*SH3GL2_2*	REACTOME_MHC_CLASS_II_ANTIGEN_PRESENTATION
9:17732590	*SH3GL2_2*	REACTOME_MHC_CLASS_II_ANTIGEN_PRESENTATION
9:17737514	*SH3GL2_2*	REACTOME_MHC_CLASS_II_ANTIGEN_PRESENTATION
9:17758945	*SH3GL2_2*	REACTOME_MHC_CLASS_II_ANTIGEN_PRESENTATION
10:104258303	*GBF1*	REACTOME_MHC_CLASS_II_ANTIGEN_PRESENTATION
8:17125768	*FGF20*	REACTOME_MICROAUTOPHAGY
1:155181232	*GBAP1*	REACTOME_MITOCHONDRIAL_BIOGENESIS
8:23415524	*BIN3*	REACTOME_MITOCHONDRIAL_IRON_SULFUR_CLUSTER_BIOGENESIS
1:155181232	*GBAP1*	REACTOME_MITOCHONDRIAL_PROTEIN_IMPORT
1:155698425	*GBAP1*	REACTOME_MITOCHONDRIAL_TRANSLATION
12:133295501	*FBRSL1*	REACTOME_MITOPHAGY
3:48199695	*IP6K2*	REACTOME_MITOTIC_G1_G1_S_PHASES
3:48214438	*IP6K2*	REACTOME_MITOTIC_G1_G1_S_PHASES
6:32814942	*HLA-DRB5*	REACTOME_MITOTIC_G1_G1_S_PHASES
12:133206648	*FBRSL1*	REACTOME_MITOTIC_G1_G1_S_PHASES
12:133223685	*FBRSL1*	REACTOME_MITOTIC_G1_G1_S_PHASES
12:133225045	*FBRSL1*	REACTOME_MITOTIC_G1_G1_S_PHASES
17:76177567	*DNAH17*	REACTOME_MITOTIC_G1_G1_S_PHASES
20:5934513	*CRLS1*	REACTOME_MITOTIC_G1_G1_S_PHASES
20:5968737	*CRLS1*	REACTOME_MITOTIC_G1_G1_S_PHASES
20:5968964	*CRLS1*	REACTOME_MITOTIC_G1_G1_S_PHASES
21:38740824	*DYRK1A*	REACTOME_MITOTIC_G1_G1_S_PHASES
21:38770530	*DYRK1A*	REACTOME_MITOTIC_G1_G1_S_PHASES
3:48199695	*IP6K2*	REACTOME_MITOTIC_G2_G2_M_PHASES
3:48214438	*IP6K2*	REACTOME_MITOTIC_G2_G2_M_PHASES

6:32814942	*HLA-DRB5*	REACTOME_MITOTIC_G2_G2_M_PHASES
10:103185542	*GBF1*	REACTOME_MITOTIC_G2_G2_M_PHASES
10:103308244	*GBF1*	REACTOME_MITOTIC_G2_G2_M_PHASES
10:104258303	*GBF1*	REACTOME_MITOTIC_G2_G2_M_PHASES
17:7578671	*V1NB1*	REACTOME_MITOTIC_G2_G2_M_PHASES
17:40818584	*RETREG3*	REACTOME_MITOTIC_G2_G2_M_PHASES
1:156059276	*GBAP1*	REACTOME_MITOTIC_METAPHASE_AND_ANAPHASE
6:32814942	*HLA-DRB5*	REACTOME_MITOTIC_METAPHASE_AND_ANAPHASE
12:122816734	*HIP1R*	REACTOME_MITOTIC_METAPHASE_AND_ANAPHASE
12:122817580	*HIP1R*	REACTOME_MITOTIC_METAPHASE_AND_ANAPHASE
12:122853197	*HIP1R*	REACTOME_MITOTIC_METAPHASE_AND_ANAPHASE
12:123057792	*HIP1R*	REACTOME_MITOTIC_METAPHASE_AND_ANAPHASE
12:123110365	*HIP1R*	REACTOME_MITOTIC_METAPHASE_AND_ANAPHASE
12:123110785	*HIP1R*	REACTOME_MITOTIC_METAPHASE_AND_ANAPHASE
17:45215865	*WNT3*	REACTOME_MITOTIC_METAPHASE_AND_ANAPHASE
17:45244822	*WNT3*	REACTOME_MITOTIC_METAPHASE_AND_ANAPHASE
10:104258303	*GBF1*	REACTOME_MITOTIC_PROMETAPHASE
12:122816734	*HIP1R*	REACTOME_MITOTIC_PROMETAPHASE
12:122817580	*HIP1R*	REACTOME_MITOTIC_PROMETAPHASE
12:122853197	*HIP1R*	REACTOME_MITOTIC_PROMETAPHASE
12:123057792	*HIP1R*	REACTOME_MITOTIC_PROMETAPHASE
12:123110365	*HIP1R*	REACTOME_MITOTIC_PROMETAPHASE
12:123110785	*HIP1R*	REACTOME_MITOTIC_PROMETAPHASE
17:40818584	*RETREG3*	REACTOME_MITOTIC_PROMETAPHASE
1:156059276	*GBAP1*	REACTOME_MITOTIC_PROPHASE
4:76695927	*FAM47E-STBD1*	REACTOME_MITOTIC_PROPHASE
12:122816734	*HIP1R*	REACTOME_MITOTIC_SPINDLE_CHECKPOINT
12:122817580	*HIP1R*	REACTOME_MITOTIC_SPINDLE_CHECKPOINT
12:122853197	*HIP1R*	REACTOME_MITOTIC_SPINDLE_CHECKPOINT
12:123057792	*HIP1R*	REACTOME_MITOTIC_SPINDLE_CHECKPOINT
12:123110365	*HIP1R*	REACTOME_MITOTIC_SPINDLE_CHECKPOINT
12:123110785	*HIP1R*	REACTOME_MITOTIC_SPINDLE_CHECKPOINT
17:45215865	*WNT3*	REACTOME_MITOTIC_SPINDLE_CHECKPOINT
17:45244822	*WNT3*	REACTOME_MITOTIC_SPINDLE_CHECKPOINT
10:15569598	*ITGA8*	REACTOME_MOLECULES_ASSOCIATED_WITH_ELASTIC_FIBRES
10:15652368	*ITGA8*	REACTOME_MOLECULES_ASSOCIATED_WITH_ELASTIC_FIBRES
14:75057014	*RPS6KL1*	REACTOME_MOLECULES_ASSOCIATED_WITH_ELASTIC_FIBRES
1:156059276	*GBAP1*	REACTOME_M_PHASE
4:76695927	*FAM47E-STBD1*	REACTOME_M_PHASE
6:32814942	*HLA-DRB5*	REACTOME_M_PHASE
10:104258303	*GBF1*	REACTOME_M_PHASE
12:122816734	*HIP1R*	REACTOME_M_PHASE
12:122817580	*HIP1R*	REACTOME_M_PHASE
12:122853197	*HIP1R*	REACTOME_M_PHASE
12:123057792	*HIP1R*	REACTOME_M_PHASE
12:123110365	*HIP1R*	REACTOME_M_PHASE
12:123110785	*HIP1R*	REACTOME_M_PHASE
17:40818584	*RETREG3*	REACTOME_M_PHASE
17:45215865	*WNT3*	REACTOME_M_PHASE
17:45244822	*WNT3*	REACTOME_M_PHASE
17:42929985	*CRHR1_2*	REACTOME_MRNA_SPLICING_MINOR_PATHWAY
6:30628082	*TRIM40*	REACTOME_MRNA_SPLICING
6:30631063	*TRIM40*	REACTOME_MRNA_SPLICING

6:30639369	*TRIM40*	REACTOME_MRNA_SPLICING
11:82886475	*DLG2*	REACTOME_MRNA_SPLICING
17:42929985	*CRHR1_2*	REACTOME_MRNA_SPLICING
13:49914395	*CAB39L*	REACTOME_MTOR_SIGNALLING
13:49927732	*CAB39L*	REACTOME_MTOR_SIGNALLING
13:49948786	*CAB39L*	REACTOME_MTOR_SIGNALLING
13:49960054	*CAB39L*	REACTOME_MTOR_SIGNALLING
13:49977462	*CAB39L*	REACTOME_MTOR_SIGNALLING
7:65425894	*GS1-124K5.11*	REACTOME_MUCOPOLYSACCHARIDOSES
1:205884274	*RAB29*	REACTOME_MULTIFUNCTIONAL_ANION_EXCHANGERS
4:114424939	*CAMK2D*	REACTOME_MUSCLE_CONTRACTION
17:7560294	*V1NB1*	REACTOME_MUSCLE_CONTRACTION
6:32151443	*HLA-DRB5*	REACTOME_MYD88_CASCADE_INITIATED_ON_PLASMA_MEMBRANE
10:103185542	*GBF1*	REACTOME_MYD88_CASCADE_INITIATED_ON_PLASMA_MEMBRANE
10:103308244	*GBF1*	REACTOME_MYD88_CASCADE_INITIATED_ON_PLASMA_MEMBRANE
16:50730088	*CASC16*	REACTOME_MYD88_CASCADE_INITIATED_ON_PLASMA_MEMBRANE
16:50730446	*CASC16*	REACTOME_MYD88_CASCADE_INITIATED_ON_PLASMA_MEMBRANE
16:50745926	*CASC16*	REACTOME_MYD88_CASCADE_INITIATED_ON_PLASMA_MEMBRANE
8:21665306	*BIN3*	REACTOME_NCAM1_INTERACTIONS
8:21668901	*BIN3*	REACTOME_NCAM1_INTERACTIONS
6:112009427	*FYN*	REACTOME_NCAM_SIGNALING_FOR_NEURITE_OUT_GROWTH
6:112056759	*FYN*	REACTOME_NCAM_SIGNALING_FOR_NEURITE_OUT_GROWTH
6:112151452	*FYN*	REACTOME_NCAM_SIGNALING_FOR_NEURITE_OUT_GROWTH
8:21665306	*BIN3*	REACTOME_NCAM_SIGNALING_FOR_NEURITE_OUT_GROWTH
8:21668901	*BIN3*	REACTOME_NCAM_SIGNALING_FOR_NEURITE_OUT_GROWTH
3:121324643	*KPNA1*	REACTOME_NEDDYLATION
3:121340955	*KPNA1*	REACTOME_NEDDYLATION
3:182656610	*MCCC1*	REACTOME_NEDDYLATION
4:15608677	*BST1*	REACTOME_NEDDYLATION
6:32814942	*HLA-DRB5*	REACTOME_NEDDYLATION
7:66096028	*GS1-124K5.11*	REACTOME_NEDDYLATION
10:103185542	*GBF1*	REACTOME_NEDDYLATION
10:103308244	*GBF1*	REACTOME_NEDDYLATION
6:112009427	*FYN*	REACTOME_NEF_AND_SIGNAL_TRANSDUCTION
6:112056759	*FYN*	REACTOME_NEF_AND_SIGNAL_TRANSDUCTION
6:112151452	*FYN*	REACTOME_NEF_AND_SIGNAL_TRANSDUCTION
9:17579690	*SH3GL2_2*	REACTOME_NEGATIVE_REGULATION_OF_MET_ACTIVITY
9:17609302	*SH3GL2_2*	REACTOME_NEGATIVE_REGULATION_OF_MET_ACTIVITY
9:17627444	*SH3GL2_2*	REACTOME_NEGATIVE_REGULATION_OF_MET_ACTIVITY
9:17710876	*SH3GL2_2*	REACTOME_NEGATIVE_REGULATION_OF_MET_ACTIVITY
9:17732590	*SH3GL2_2*	REACTOME_NEGATIVE_REGULATION_OF_MET_ACTIVITY
9:17737514	*SH3GL2_2*	REACTOME_NEGATIVE_REGULATION_OF_MET_ACTIVITY
9:17758945	*SH3GL2_2*	REACTOME_NEGATIVE_REGULATION_OF_MET_ACTIVITY
4:114424939	*CAMK2D*	REACTOME_NEGATIVE_REGULATION_OF_NMDA_RECEPTOR_MEDIATED_NEURONAL_TRANSMISSION
11:83188607	*DLG2*	REACTOME_NEGATIVE_REGULATION_OF_NMDA_RECEPTOR_MEDIATED_NEURONAL_TRANSMISSION
11:83219006	*DLG2*	REACTOME_NEGATIVE_REGULATION_OF_NMDA_RECEPTOR_MEDIATED_NEURONAL_TRANSMISSION
11:83538733	*DLG2*	REACTOME_NEGATIVE_REGULATION_OF_NMDA_RECEPTOR_MEDIATED_NEURONAL_TRANSMISSION
11:83544472	*DLG2*	REACTOME_NEGATIVE_REGULATION_OF_NMDA_RECEPTOR_MEDIATED_NEURONAL_TRANSMISSION
11:83656201	*DLG2*	REACTOME_NEGATIVE_REGULATION_OF_NMDA_RECEPTOR_MEDIATED_NEURONAL_TRANSMISSION
11:83700228	*DLG2*	REACTOME_NEGATIVE_REGULATION_OF_NMDA_RECEPTOR_MEDIATED_NEURONAL_TRANSMISSION
11:83762918	*DLG2*	REACTOME_NEGATIVE_REGULATION_OF_NMDA_RECEPTOR_MEDIATED_NEURONAL_TRANSMISSION
11:83894247	*DLG2*	REACTOME_NEGATIVE_REGULATION_OF_NMDA_RECEPTOR_MEDIATED_NEURONAL_TRANSMISSION
11:84021037	*DLG2*	REACTOME_NEGATIVE_REGULATION_OF_NMDA_RECEPTOR_MEDIATED_NEURONAL_TRANSMISSION

11:84324361	*DLG2*	REACTOME_NEGATIVE_REGULATION_OF_NMDA_RECEPTOR_MEDIATED_NEURONAL_TRANSMISSION
11:84373496	*DLG2*	REACTOME_NEGATIVE_REGULATION_OF_NMDA_RECEPTOR_MEDIATED_NEURONAL_TRANSMISSION
11:84403409	*DLG2*	REACTOME_NEGATIVE_REGULATION_OF_NMDA_RECEPTOR_MEDIATED_NEURONAL_TRANSMISSION
17:7101771	*V1NB1*	REACTOME_NEGATIVE_REGULATION_OF_NMDA_RECEPTOR_MEDIATED_NEURONAL_TRANSMISSION
6:32186193	*HLA-DRB5*	REACTOME_NEGATIVE_REGULATION_OF_NOTCH4_SIGNALING
6:32814942	*HLA-DRB5*	REACTOME_NEGATIVE_REGULATION_OF_NOTCH4_SIGNALING
17:7129974	*V1NB1*	REACTOME_NEGATIVE_REGULATION_OF_TCF_DEPENDENT_SIGNALING_BY_DVL_INTERACTING_PROTEINS
6:112009427	*FYN*	REACTOME_NEGATIVE_REGULATION_OF_THE_PI3K_AKT_NETWORK
6:112056759	*FYN*	REACTOME_NEGATIVE_REGULATION_OF_THE_PI3K_AKT_NETWORK
6:112151452	*FYN*	REACTOME_NEGATIVE_REGULATION_OF_THE_PI3K_AKT_NETWORK
6:112009427	*FYN*	REACTOME_NEPHRIN_FAMILY_INTERACTIONS
6:112056759	*FYN*	REACTOME_NEPHRIN_FAMILY_INTERACTIONS
6:112151452	*FYN*	REACTOME_NEPHRIN_FAMILY_INTERACTIONS
6:112009427	*FYN*	REACTOME_NETRIN_1_SIGNALING
6:112056759	*FYN*	REACTOME_NETRIN_1_SIGNALING
6:112151452	*FYN*	REACTOME_NETRIN_1_SIGNALING
11:83188607	*DLG2*	REACTOME_NEUREXINS_AND_NEUROLIGINS
11:83219006	*DLG2*	REACTOME_NEUREXINS_AND_NEUROLIGINS
11:83538733	*DLG2*	REACTOME_NEUREXINS_AND_NEUROLIGINS
11:83544472	*DLG2*	REACTOME_NEUREXINS_AND_NEUROLIGINS
11:83656201	*DLG2*	REACTOME_NEUREXINS_AND_NEUROLIGINS
11:83700228	*DLG2*	REACTOME_NEUREXINS_AND_NEUROLIGINS
11:83762918	*DLG2*	REACTOME_NEUREXINS_AND_NEUROLIGINS
11:83894247	*DLG2*	REACTOME_NEUREXINS_AND_NEUROLIGINS
11:84021037	*DLG2*	REACTOME_NEUREXINS_AND_NEUROLIGINS
11:84324361	*DLG2*	REACTOME_NEUREXINS_AND_NEUROLIGINS
11:84373496	*DLG2*	REACTOME_NEUREXINS_AND_NEUROLIGINS
11:84403409	*DLG2*	REACTOME_NEUREXINS_AND_NEUROLIGINS
17:7101771	*V1NB1*	REACTOME_NEUREXINS_AND_NEUROLIGINS
1:156059276	*GBAP1*	REACTOME_NEURODEGENERATIVE_DISEASES
3:48199695	*IP6K2*	REACTOME_NEURODEGENERATIVE_DISEASES
3:48214438	*IP6K2*	REACTOME_NEURODEGENERATIVE_DISEASES
12:41147295	*LRRK2_2*	REACTOME_NEUROFASCIN_INTERACTIONS
12:41251554	*LRRK2_2*	REACTOME_NEUROFASCIN_INTERACTIONS
12:41407403	*LRRK2_2*	REACTOME_NEUROFASCIN_INTERACTIONS
1:154804988	*GBAP1*	REACTOME_NEURONAL_SYSTEM
2:18143082	*KCNS3*	REACTOME_NEURONAL_SYSTEM
2:18176120	*KCNS3*	REACTOME_NEURONAL_SYSTEM
2:18247777	*KCNS3*	REACTOME_NEURONAL_SYSTEM
2:18296870	*KCNS3*	REACTOME_NEURONAL_SYSTEM
2:18335515	*KCNS3*	REACTOME_NEURONAL_SYSTEM
3:19312697	*SATB1*	REACTOME_NEURONAL_SYSTEM
3:19334895	*SATB1*	REACTOME_NEURONAL_SYSTEM
3:48835245	*IP6K2*	REACTOME_NEURONAL_SYSTEM
3:123057397	*KPNA1*	REACTOME_NEURONAL_SYSTEM
4:114424939	*CAMK2D*	REACTOME_NEURONAL_SYSTEM
6:29579544	*TRIM40*	REACTOME_NEURONAL_SYSTEM
11:83188607	*DLG2*	REACTOME_NEURONAL_SYSTEM
11:83219006	*DLG2*	REACTOME_NEURONAL_SYSTEM
11:83538733	*DLG2*	REACTOME_NEURONAL_SYSTEM
11:83544472	*DLG2*	REACTOME_NEURONAL_SYSTEM
11:83656201	*DLG2*	REACTOME_NEURONAL_SYSTEM
11:83700228	*DLG2*	REACTOME_NEURONAL_SYSTEM

11:83762918	*DLG2*	REACTOME_NEURONAL_SYSTEM
11:83894247	*DLG2*	REACTOME_NEURONAL_SYSTEM
11:84021037	*DLG2*	REACTOME_NEURONAL_SYSTEM
11:84324361	*DLG2*	REACTOME_NEURONAL_SYSTEM
11:84373496	*DLG2*	REACTOME_NEURONAL_SYSTEM
11:84403409	*DLG2*	REACTOME_NEURONAL_SYSTEM
17:7101771	*V1NB1*	REACTOME_NEURONAL_SYSTEM
17:7832034	*V1NB1*	REACTOME_NEURONAL_SYSTEM
17:43999203	*WNT3*	REACTOME_NEURONAL_SYSTEM
17:44005297	*WNT3*	REACTOME_NEURONAL_SYSTEM
17:44019712	*WNT3*	REACTOME_NEURONAL_SYSTEM
17:44060492	*WNT3*	REACTOME_NEURONAL_SYSTEM
17:44065263	*WNT3*	REACTOME_NEURONAL_SYSTEM
17:44081178	*WNT3*	REACTOME_NEURONAL_SYSTEM
17:44083297	*WNT3*	REACTOME_NEURONAL_SYSTEM
17:44800946	*WNT3*	REACTOME_NEURONAL_SYSTEM
17:44833217	*WNT3*	REACTOME_NEURONAL_SYSTEM
21:39197323	*DYRK1A*	REACTOME_NEURONAL_SYSTEM
16:31004169	*SETD1A*	REACTOME_NEUROTOXICITY_OF_CLOSTRIDIUM_TOXINS
3:48835245	*IP6K2*	REACTOME_NEUROTRANSMITTER_RECEPTORS_AND_POSTSYNAPTIC_SIGNAL_TRANSMISSION
3:123057397	*KPNA1*	REACTOME_NEUROTRANSMITTER_RECEPTORS_AND_POSTSYNAPTIC_SIGNAL_TRANSMISSION
4:114424939	*CAMK2D*	REACTOME_NEUROTRANSMITTER_RECEPTORS_AND_POSTSYNAPTIC_SIGNAL_TRANSMISSION
6:29579544	*TRIM40*	REACTOME_NEUROTRANSMITTER_RECEPTORS_AND_POSTSYNAPTIC_SIGNAL_TRANSMISSION
11:83188607	*DLG2*	REACTOME_NEUROTRANSMITTER_RECEPTORS_AND_POSTSYNAPTIC_SIGNAL_TRANSMISSION
11:83219006	*DLG2*	REACTOME_NEUROTRANSMITTER_RECEPTORS_AND_POSTSYNAPTIC_SIGNAL_TRANSMISSION
11:83538733	*DLG2*	REACTOME_NEUROTRANSMITTER_RECEPTORS_AND_POSTSYNAPTIC_SIGNAL_TRANSMISSION
11:83544472	*DLG2*	REACTOME_NEUROTRANSMITTER_RECEPTORS_AND_POSTSYNAPTIC_SIGNAL_TRANSMISSION
11:83656201	*DLG2*	REACTOME_NEUROTRANSMITTER_RECEPTORS_AND_POSTSYNAPTIC_SIGNAL_TRANSMISSION
11:83700228	*DLG2*	REACTOME_NEUROTRANSMITTER_RECEPTORS_AND_POSTSYNAPTIC_SIGNAL_TRANSMISSION
11:83762918	*DLG2*	REACTOME_NEUROTRANSMITTER_RECEPTORS_AND_POSTSYNAPTIC_SIGNAL_TRANSMISSION
11:83894247	*DLG2*	REACTOME_NEUROTRANSMITTER_RECEPTORS_AND_POSTSYNAPTIC_SIGNAL_TRANSMISSION
11:84021037	*DLG2*	REACTOME_NEUROTRANSMITTER_RECEPTORS_AND_POSTSYNAPTIC_SIGNAL_TRANSMISSION
11:84324361	*DLG2*	REACTOME_NEUROTRANSMITTER_RECEPTORS_AND_POSTSYNAPTIC_SIGNAL_TRANSMISSION
11:84373496	*DLG2*	REACTOME_NEUROTRANSMITTER_RECEPTORS_AND_POSTSYNAPTIC_SIGNAL_TRANSMISSION
11:84403409	*DLG2*	REACTOME_NEUROTRANSMITTER_RECEPTORS_AND_POSTSYNAPTIC_SIGNAL_TRANSMISSION
17:7101771	*V1NB1*	REACTOME_NEUROTRANSMITTER_RECEPTORS_AND_POSTSYNAPTIC_SIGNAL_TRANSMISSION
17:43999203	*WNT3*	REACTOME_NEUROTRANSMITTER_RECEPTORS_AND_POSTSYNAPTIC_SIGNAL_TRANSMISSION
17:44005297	*WNT3*	REACTOME_NEUROTRANSMITTER_RECEPTORS_AND_POSTSYNAPTIC_SIGNAL_TRANSMISSION
17:44019712	*WNT3*	REACTOME_NEUROTRANSMITTER_RECEPTORS_AND_POSTSYNAPTIC_SIGNAL_TRANSMISSION
17:44060492	*WNT3*	REACTOME_NEUROTRANSMITTER_RECEPTORS_AND_POSTSYNAPTIC_SIGNAL_TRANSMISSION
17:44065263	*WNT3*	REACTOME_NEUROTRANSMITTER_RECEPTORS_AND_POSTSYNAPTIC_SIGNAL_TRANSMISSION
17:44081178	*WNT3*	REACTOME_NEUROTRANSMITTER_RECEPTORS_AND_POSTSYNAPTIC_SIGNAL_TRANSMISSION
17:44083297	*WNT3*	REACTOME_NEUROTRANSMITTER_RECEPTORS_AND_POSTSYNAPTIC_SIGNAL_TRANSMISSION
17:44800946	*WNT3*	REACTOME_NEUROTRANSMITTER_RECEPTORS_AND_POSTSYNAPTIC_SIGNAL_TRANSMISSION
17:44833217	*WNT3*	REACTOME_NEUROTRANSMITTER_RECEPTORS_AND_POSTSYNAPTIC_SIGNAL_TRANSMISSION
21:39197323	*DYRK1A*	REACTOME_NEUROTRANSMITTER_RECEPTORS_AND_POSTSYNAPTIC_SIGNAL_TRANSMISSION
1:226052830	*ITPKB*	REACTOME_NEUTROPHIL_DEGRANULATION
3:49406095	*IP6K2*	REACTOME_NEUTROPHIL_DEGRANULATION
3:49424231	*IP6K2*	REACTOME_NEUTROPHIL_DEGRANULATION
3:49450137	*IP6K2*	REACTOME_NEUTROPHIL_DEGRANULATION
4:15709252	*BST1*	REACTOME_NEUTROPHIL_DEGRANULATION
4:15713608	*BST1*	REACTOME_NEUTROPHIL_DEGRANULATION
4:15724372	*BST1*	REACTOME_NEUTROPHIL_DEGRANULATION

4:15737882	*BST1*	REACTOME_NEUTROPHIL_DEGRANULATION
6:133025715	*RPS12*	REACTOME_NEUTROPHIL_DEGRANULATION
6:133028870	*RPS12*	REACTOME_NEUTROPHIL_DEGRANULATION
6:133034076	*RPS12*	REACTOME_NEUTROPHIL_DEGRANULATION
7:65425894	*GS1-124K5.11*	REACTOME_NEUTROPHIL_DEGRANULATION
8:11712443	*CTSB*	REACTOME_NEUTROPHIL_DEGRANULATION
9:33791164	*UBAP2*	REACTOME_NEUTROPHIL_DEGRANULATION
11:10392432	*RNF141*	REACTOME_NEUTROPHIL_DEGRANULATION
11:10487997	*RNF141*	REACTOME_NEUTROPHIL_DEGRANULATION
11:82582134	*DLG2*	REACTOME_NEUTROPHIL_DEGRANULATION
11:82648357	*DLG2*	REACTOME_NEUTROPHIL_DEGRANULATION
12:45755099	*SCAF11*	REACTOME_NEUTROPHIL_DEGRANULATION
16:30510571	*SETD1A*	REACTOME_NEUTROPHIL_DEGRANULATION
16:30516492	*SETD1A*	REACTOME_NEUTROPHIL_DEGRANULATION
16:30520856	*SETD1A*	REACTOME_NEUTROPHIL_DEGRANULATION
16:31370452	*SETD1A*	REACTOME_NEUTROPHIL_DEGRANULATION
17:39883520	*RETREG3*	REACTOME_NEUTROPHIL_DEGRANULATION
17:42423844	*FAM171A2*	REACTOME_NEUTROPHIL_DEGRANULATION
2:134977261	*TMEM163*	REACTOME_N_GLYCAN_ANTENNAE_ELONGATION_IN_THE_MEDIAL_TRANS_GOLGI
2:135194840	*TMEM163*	REACTOME_N_GLYCAN_ANTENNAE_ELONGATION_IN_THE_MEDIAL_TRANS_GOLGI
2:135202455	*TMEM163*	REACTOME_N_GLYCAN_ANTENNAE_ELONGATION_IN_THE_MEDIAL_TRANS_GOLGI
2:134977261	*TMEM163*	REACTOME_N_GLYCAN_ANTENNAE_ELONGATION
2:135194840	*TMEM163*	REACTOME_N_GLYCAN_ANTENNAE_ELONGATION
2:135202455	*TMEM163*	REACTOME_N_GLYCAN_ANTENNAE_ELONGATION
6:32151443	*HLA-DRB5*	REACTOME_N_GLYCAN_TRIMMING_IN_THE_ER_AND_CALNEXIN_CALRETICULIN_CYCLE
3:122259640	*KPNA1*	REACTOME_NICOTINAMIDE_SALVAGING
3:122259640	*KPNA1*	REACTOME_NICOTINATE_METABOLISM
4:15709252	*BST1*	REACTOME_NICOTINATE_METABOLISM
4:15713608	*BST1*	REACTOME_NICOTINATE_METABOLISM
4:15724372	*BST1*	REACTOME_NICOTINATE_METABOLISM
4:15737882	*BST1*	REACTOME_NICOTINATE_METABOLISM
4:15789288	*BST1*	REACTOME_NICOTINATE_METABOLISM
4:15816670	*BST1*	REACTOME_NICOTINATE_METABOLISM
4:15829612	*BST1*	REACTOME_NICOTINATE_METABOLISM
11:10647977	*RNF141*	REACTOME_NITRIC_OXIDE_STIMULATES_GUANYLATE_CYCLASE
11:10648481	*RNF141*	REACTOME_NITRIC_OXIDE_STIMULATES_GUANYLATE_CYCLASE
11:10660840	*RNF141*	REACTOME_NITRIC_OXIDE_STIMULATES_GUANYLATE_CYCLASE
16:50730088	*CASC16*	REACTOME_NOD1_2_SIGNALING_PATHWAY
16:50730446	*CASC16*	REACTOME_NOD1_2_SIGNALING_PATHWAY
16:50745926	*CASC16*	REACTOME_NOD1_2_SIGNALING_PATHWAY
1:227067656	*ITPKB*	REACTOME_NONCANONICAL_ACTIVATION_OF_NOTCH3
3:49162284	*IP6K2*	REACTOME_NON_INTEGRIN_MEMBRANE_ECM_INTERACTIONS
3:49162583	*IP6K2*	REACTOME_NON_INTEGRIN_MEMBRANE_ECM_INTERACTIONS
3:49561208	*IP6K2*	REACTOME_NON_INTEGRIN_MEMBRANE_ECM_INTERACTIONS
6:30850582	*TRIM40*	REACTOME_NON_INTEGRIN_MEMBRANE_ECM_INTERACTIONS
6:33153528	*HLA-DRB5*	REACTOME_NON_INTEGRIN_MEMBRANE_ECM_INTERACTIONS
6:112561154	*FYN*	REACTOME_NON_INTEGRIN_MEMBRANE_ECM_INTERACTIONS
19:3022354	*SPPL2B*	REACTOME_NOTCH1_INTRACELLULAR_DOMAIN_REGULATES_TRANSCRIPTION
19:3036350	*SPPL2B*	REACTOME_NOTCH1_INTRACELLULAR_DOMAIN_REGULATES_TRANSCRIPTION
1:227067656	*ITPKB*	REACTOME_NOTCH2_ACTIVATION_AND_TRANSMISSION_OF_SIGNAL_TO_THE_NUCLEUS
12:41147295	*LRRK2_2*	REACTOME_NOTCH2_ACTIVATION_AND_TRANSMISSION_OF_SIGNAL_TO_THE_NUCLEUS
12:41251554	*LRRK2_2*	REACTOME_NOTCH2_ACTIVATION_AND_TRANSMISSION_OF_SIGNAL_TO_THE_NUCLEUS
12:41407403	*LRRK2_2*	REACTOME_NOTCH2_ACTIVATION_AND_TRANSMISSION_OF_SIGNAL_TO_THE_NUCLEUS

1:227067656	*ITPKB*	REACTOME_NOTCH3_ACTIVATION_AND_TRANSMISSION_OF_SIGNAL_TO_THE_NUCLEUS
1:227067656	*ITPKB*	REACTOME_NOTCH4_ACTIVATION_AND_TRANSMISSION_OF_SIGNAL_TO_THE_NUCLEUS
6:32186193	*HLA-DRB5*	REACTOME_NOTCH4_ACTIVATION_AND_TRANSMISSION_OF_SIGNAL_TO_THE_NUCLEUS
6:32186193	*HLA-DRB5*	REACTOME_NOTCH4_INTRACELLULAR_DOMAIN_REGULATES_TRANSCRIPTION
6:32186193	*HLA-DRB5*	REACTOME_NOTCH_HLH_TRANSCRIPTION_PATHWAY
1:155975202	*GBAP1*	REACTOME_NRAGE_SIGNALS_DEATH_THROUGH_JNK
17:7101771	*V1NB1*	REACTOME_NRCAM_INTERACTIONS
1:227067656	*ITPKB*	REACTOME_NRIF_SIGNALS_CELL_DEATH_FROM_THE_NUCLEUS
6:112009427	*FYN*	REACTOME_NTRK2_ACTIVATES_RAC1
6:112056759	*FYN*	REACTOME_NTRK2_ACTIVATES_RAC1
6:112151452	*FYN*	REACTOME_NTRK2_ACTIVATES_RAC1
1:156059276	*GBAP1*	REACTOME_NUCLEAR_ENVELOPE_BREAKDOWN
1:156059276	*GBAP1*	REACTOME_NUCLEAR_ENVELOPE_REASSEMBLY
15:61293467	*VPS13C*	REACTOME_NUCLEAR_RECEPTOR_TRANSCRIPTION_PATHWAY
1:227067656	*ITPKB*	REACTOME_NUCLEAR_SIGNALING_BY_ERBB4
10:104847713	*GBF1*	REACTOME_NUCLEOBASE_CATABOLISM
10:104861736	*GBF1*	REACTOME_NUCLEOBASE_CATABOLISM
10:104926485	*GBF1*	REACTOME_NUCLEOBASE_CATABOLISM
10:104929716	*GBF1*	REACTOME_NUCLEOBASE_CATABOLISM
14:74477206	*RPS6KL1*	REACTOME_NUCLEOBASE_CATABOLISM
16:50730088	*CASC16*	REACTOME_NUCLEOTIDE_BINDING_DOMAIN_LEUCINE_RICH_REPEAT_CONTAINING_RECEPTOR_NLR_SIGNALING_PATHWAYS
16:50730446	*CASC16*	REACTOME_NUCLEOTIDE_BINDING_DOMAIN_LEUCINE_RICH_REPEAT_CONTAINING_RECEPTOR_NLR_SIGNALING_PATHWAYS
16:50745926	*CASC16*	REACTOME_NUCLEOTIDE_BINDING_DOMAIN_LEUCINE_RICH_REPEAT_CONTAINING_RECEPTOR_NLR_SIGNALING_PATHWAYS
12:133206648	*FBRSL1*	REACTOME_NUCLEOTIDE_EXCISION_REPAIR
12:133223685	*FBRSL1*	REACTOME_NUCLEOTIDE_EXCISION_REPAIR
12:133225045	*FBRSL1*	REACTOME_NUCLEOTIDE_EXCISION_REPAIR
3:151090424	*MED12L*	REACTOME_NUCLEOTIDE_LIKE_PURINERGIC_RECEPTORS
3:151099733	*MED12L*	REACTOME_NUCLEOTIDE_LIKE_PURINERGIC_RECEPTORS
11:10392432	*RNF141*	REACTOME_NUCLEOTIDE_SALVAGE
11:10487997	*RNF141*	REACTOME_NUCLEOTIDE_SALVAGE
17:76177567	*DNAH17*	REACTOME_NUCLEOTIDE_SALVAGE
17:42338993	*FAM171A2*	REACTOME_O2_CO2_EXCHANGE_IN_ERYTHROCYTES
3:122645304	*KPNA1*	REACTOME_O_GLYCOSYLATION_OF_TSR_DOMAIN_CONTAINING_PROTEINS
9:18650547	*SH3GL2_2*	REACTOME_O_GLYCOSYLATION_OF_TSR_DOMAIN_CONTAINING_PROTEINS
6:29351969	*TRIM40*	REACTOME_OLFACTORY_SIGNALING_PATHWAY
6:29373213	*TRIM40*	REACTOME_OLFACTORY_SIGNALING_PATHWAY
6:29407378	*TRIM40*	REACTOME_OLFACTORY_SIGNALING_PATHWAY
6:29412222	*TRIM40*	REACTOME_OLFACTORY_SIGNALING_PATHWAY
3:182976382	*MCCC1*	REACTOME_O_LINKED_GLYCOSYLATION_OF_MUCINS
3:182979912	*MCCC1*	REACTOME_O_LINKED_GLYCOSYLATION_OF_MUCINS
3:183005320	*MCCC1*	REACTOME_O_LINKED_GLYCOSYLATION_OF_MUCINS
12:132687184	*FBRSL1*	REACTOME_O_LINKED_GLYCOSYLATION_OF_MUCINS
3:49561208	*IP6K2*	REACTOME_O_LINKED_GLYCOSYLATION
3:122645304	*KPNA1*	REACTOME_O_LINKED_GLYCOSYLATION
3:182976382	*MCCC1*	REACTOME_O_LINKED_GLYCOSYLATION
3:182979912	*MCCC1*	REACTOME_O_LINKED_GLYCOSYLATION
3:183005320	*MCCC1*	REACTOME_O_LINKED_GLYCOSYLATION
9:18650547	*SH3GL2_2*	REACTOME_O_LINKED_GLYCOSYLATION
12:132687184	*FBRSL1*	REACTOME_O_LINKED_GLYCOSYLATION
17:7578671	*V1NB1*	REACTOME_ONCOGENE_INDUCED_SENESCENCE
1:156059276	*GBAP1*	REACTOME_ONCOGENIC_MAPK_SIGNALING
4:114424939	*CAMK2D*	REACTOME_ONCOGENIC_MAPK_SIGNALING
3:48835245	*IP6K2*	REACTOME_OPIOID_SIGNALLING

3:123057397	*KPNA1*	REACTOME_OPIOID_SIGNALLING
4:114424939	*CAMK2D*	REACTOME_OPIOID_SIGNALLING
5:59236972	*ELOVL7*	REACTOME_OPIOID_SIGNALLING
5:59665659	*ELOVL7*	REACTOME_OPIOID_SIGNALLING
5:59705316	*ELOVL7*	REACTOME_OPIOID_SIGNALLING
5:59736085	*ELOVL7*	REACTOME_OPIOID_SIGNALLING
6:32814942	*HLA-DRB5*	REACTOME_ORC1_REMOVAL_FROM_CHROMATIN
20:5934513	*CRLS1*	REACTOME_ORC1_REMOVAL_FROM_CHROMATIN
20:5968737	*CRLS1*	REACTOME_ORC1_REMOVAL_FROM_CHROMATIN
20:5968964	*CRLS1*	REACTOME_ORC1_REMOVAL_FROM_CHROMATIN
1:155181232	*GBAP1*	REACTOME_ORGANELLE_BIOGENESIS_AND_MAINTENANCE
3:121553904	*KPNA1*	REACTOME_ORGANELLE_BIOGENESIS_AND_MAINTENANCE
4:15492268	*BST1*	REACTOME_ORGANELLE_BIOGENESIS_AND_MAINTENANCE
4:15547513	*BST1*	REACTOME_ORGANELLE_BIOGENESIS_AND_MAINTENANCE
4:15554219	*BST1*	REACTOME_ORGANELLE_BIOGENESIS_AND_MAINTENANCE
6:30601232	*TRIM40*	REACTOME_ORGANELLE_BIOGENESIS_AND_MAINTENANCE
8:131153067	*FAM49B*	REACTOME_ORGANELLE_BIOGENESIS_AND_MAINTENANCE
8:131190319	*FAM49B*	REACTOME_ORGANELLE_BIOGENESIS_AND_MAINTENANCE
8:131427864	*FAM49B*	REACTOME_ORGANELLE_BIOGENESIS_AND_MAINTENANCE
10:104005410	*GBF1*	REACTOME_ORGANELLE_BIOGENESIS_AND_MAINTENANCE
10:104034550	*GBF1*	REACTOME_ORGANELLE_BIOGENESIS_AND_MAINTENANCE
10:104258303	*GBF1*	REACTOME_ORGANELLE_BIOGENESIS_AND_MAINTENANCE
10:104443875	*GBF1*	REACTOME_ORGANELLE_BIOGENESIS_AND_MAINTENANCE
10:104452818	*GBF1*	REACTOME_ORGANELLE_BIOGENESIS_AND_MAINTENANCE
4:114424939	*CAMK2D*	REACTOME_OTHER_INTERLEUKIN_SIGNALING
6:32814942	*HLA-DRB5*	REACTOME_OTHER_INTERLEUKIN_SIGNALING
6:112009427	*FYN*	REACTOME_OTHER_INTERLEUKIN_SIGNALING
6:112056759	*FYN*	REACTOME_OTHER_INTERLEUKIN_SIGNALING
6:112151452	*FYN*	REACTOME_OTHER_INTERLEUKIN_SIGNALING
8:21665306	*BIN3*	REACTOME_OTHER_INTERLEUKIN_SIGNALING
8:21668901	*BIN3*	REACTOME_OTHER_INTERLEUKIN_SIGNALING
11:83188607	*DLG2*	REACTOME_OTHER_INTERLEUKIN_SIGNALING
11:83219006	*DLG2*	REACTOME_OTHER_INTERLEUKIN_SIGNALING
11:83538733	*DLG2*	REACTOME_OTHER_INTERLEUKIN_SIGNALING
11:83544472	*DLG2*	REACTOME_OTHER_INTERLEUKIN_SIGNALING
11:83656201	*DLG2*	REACTOME_OTHER_INTERLEUKIN_SIGNALING
11:83700228	*DLG2*	REACTOME_OTHER_INTERLEUKIN_SIGNALING
11:83762918	*DLG2*	REACTOME_OTHER_INTERLEUKIN_SIGNALING
11:83894247	*DLG2*	REACTOME_OTHER_INTERLEUKIN_SIGNALING
11:84021037	*DLG2*	REACTOME_OTHER_INTERLEUKIN_SIGNALING
11:84324361	*DLG2*	REACTOME_OTHER_INTERLEUKIN_SIGNALING
11:84373496	*DLG2*	REACTOME_OTHER_INTERLEUKIN_SIGNALING
11:84403409	*DLG2*	REACTOME_OTHER_INTERLEUKIN_SIGNALING
17:7101771	*V1NB1*	REACTOME_OTHER_INTERLEUKIN_SIGNALING
17:40392358	*RETREG3*	REACTOME_OTHER_INTERLEUKIN_SIGNALING
3:49406095	*IP6K2*	REACTOME_OVARIAN_TUMOR_DOMAIN_PROTEASES
3:49424231	*IP6K2*	REACTOME_OVARIAN_TUMOR_DOMAIN_PROTEASES
3:49450137	*IP6K2*	REACTOME_OVARIAN_TUMOR_DOMAIN_PROTEASES
16:50730088	*CASC16*	REACTOME_OVARIAN_TUMOR_DOMAIN_PROTEASES
16:50730446	*CASC16*	REACTOME_OVARIAN_TUMOR_DOMAIN_PROTEASES
16:50745926	*CASC16*	REACTOME_OVARIAN_TUMOR_DOMAIN_PROTEASES
17:7578671	*V1NB1*	REACTOME_OVARIAN_TUMOR_DOMAIN_PROTEASES
2:102357447	*MAP4K4*	REACTOME_OXIDATIVE_STRESS_INDUCED_SENESCENCE

2:102413116	*MAP4K4*	REACTOME_OXIDATIVE_STRESS_INDUCED_SENESCENCE
17:7578671	*V1NB1*	REACTOME_OXIDATIVE_STRESS_INDUCED_SENESCENCE
3:151090424	*MED12L*	REACTOME_P2Y_RECEPTORS
3:151099733	*MED12L*	REACTOME_P2Y_RECEPTORS
1:155975202	*GBAP1*	REACTOME_P75_NTR_RECEPTOR_MEDIATED_SIGNALLING
1:227067656	*ITPKB*	REACTOME_P75_NTR_RECEPTOR_MEDIATED_SIGNALLING
3:49406095	*IP6K2*	REACTOME_P75_NTR_RECEPTOR_MEDIATED_SIGNALLING
3:49424231	*IP6K2*	REACTOME_P75_NTR_RECEPTOR_MEDIATED_SIGNALLING
3:49450137	*IP6K2*	REACTOME_P75_NTR_RECEPTOR_MEDIATED_SIGNALLING
3:49406095	*IP6K2*	REACTOME_P75NTR_REGULATES_AXONOGENESIS
3:49424231	*IP6K2*	REACTOME_P75NTR_REGULATES_AXONOGENESIS
3:49450137	*IP6K2*	REACTOME_P75NTR_REGULATES_AXONOGENESIS
12:133206648	*FBRSL1*	REACTOME_PCNA_DEPENDENT_LONG_PATCH_BASE_EXCISION_REPAIR
12:133223685	*FBRSL1*	REACTOME_PCNA_DEPENDENT_LONG_PATCH_BASE_EXCISION_REPAIR
12:133225045	*FBRSL1*	REACTOME_PCNA_DEPENDENT_LONG_PATCH_BASE_EXCISION_REPAIR
3:49406095	*IP6K2*	REACTOME_PCP_CE_PATHWAY
3:49424231	*IP6K2*	REACTOME_PCP_CE_PATHWAY
3:49450137	*IP6K2*	REACTOME_PCP_CE_PATHWAY
6:32814942	*HLA-DRB5*	REACTOME_PCP_CE_PATHWAY
17:7129974	*V1NB1*	REACTOME_PCP_CE_PATHWAY
6:32602372	*HLA-DRB5*	REACTOME_PD_1_SIGNALING
6:112009427	*FYN*	REACTOME_PECAM1_INTERACTIONS
6:112056759	*FYN*	REACTOME_PECAM1_INTERACTIONS
6:112151452	*FYN*	REACTOME_PECAM1_INTERACTIONS
1:161875645	*FCGR2A*	REACTOME_PERK_REGULATES_GENE_EXPRESSION
1:161930954	*FCGR2A*	REACTOME_PERK_REGULATES_GENE_EXPRESSION
4:114424939	*CAMK2D*	REACTOME_PHASE_0_RAPID_DEPOLARISATION
7:65705034	*GS1-124K5.11*	REACTOME_PHASE_II_CONJUGATION_OF_COMPOUNDS
10:104635620	*GBF1*	REACTOME_PHASE_II_CONJUGATION_OF_COMPOUNDS
14:74477206	*RPS6KL1*	REACTOME_PHOSPHATE_BOND_HYDROLYSIS_BY_NTPDASE_PROTEINS
2:96858914	*KCNIP3*	REACTOME_PHOSPHOLIPID_METABOLISM
6:31846234	*HLA-DRB5*	REACTOME_PHOSPHOLIPID_METABOLISM
10:121505277	*INPP5F*	REACTOME_PHOSPHOLIPID_METABOLISM
10:121538407	*INPP5F*	REACTOME_PHOSPHOLIPID_METABOLISM
12:123561638	*HIP1R*	REACTOME_PHOSPHOLIPID_METABOLISM
12:123585705	*HIP1R*	REACTOME_PHOSPHOLIPID_METABOLISM
20:6011934	*CRLS1*	REACTOME_PHOSPHOLIPID_METABOLISM
6:32602372	*HLA-DRB5*	REACTOME_PHOSPHORYLATION_OF_CD3_AND_TCR_ZETA_CHAINS
17:45215865	*WNT3*	REACTOME_PHOSPHORYLATION_OF_THE_APC_C
17:45244822	*WNT3*	REACTOME_PHOSPHORYLATION_OF_THE_APC_C
3:49406095	*IP6K2*	REACTOME_PI3K_AKT_ACTIVATION
3:49424231	*IP6K2*	REACTOME_PI3K_AKT_ACTIVATION
3:49450137	*IP6K2*	REACTOME_PI3K_AKT_ACTIVATION
6:112009427	*FYN*	REACTOME_PI3K_AKT_SIGNALING_IN_CANCER
6:112056759	*FYN*	REACTOME_PI3K_AKT_SIGNALING_IN_CANCER
6:112151452	*FYN*	REACTOME_PI3K_AKT_SIGNALING_IN_CANCER
17:7578671	*V1NB1*	REACTOME_PI5P_REGULATES_TP53_ACETYLATION
10:121505277	*INPP5F*	REACTOME_PI_METABOLISM
10:121538407	*INPP5F*	REACTOME_PI_METABOLISM
3:48835245	*IP6K2*	REACTOME_PKA_ACTIVATION_IN_GLUCAGON_SIGNALLING
3:123057397	*KPNA1*	REACTOME_PKA_ACTIVATION_IN_GLUCAGON_SIGNALLING
3:48835245	*IP6K2*	REACTOME_PKA_MEDIATED_PHOSPHORYLATION_OF_CREB
3:123057397	*KPNA1*	REACTOME_PKA_MEDIATED_PHOSPHORYLATION_OF_CREB

1:155359992	*GBAP1*	REACTOME_PKMTS_METHYLATE_HISTONE_LYSINES
6:31862876	*HLA-DRB5*	REACTOME_PKMTS_METHYLATE_HISTONE_LYSINES
19:2172266	*SPPL2B*	REACTOME_PKMTS_METHYLATE_HISTONE_LYSINES
3:49406095	*IP6K2*	REACTOME_PLATELET_ACTIVATION_SIGNALING_AND_AGGREGATION
3:49424231	*IP6K2*	REACTOME_PLATELET_ACTIVATION_SIGNALING_AND_AGGREGATION
3:49450137	*IP6K2*	REACTOME_PLATELET_ACTIVATION_SIGNALING_AND_AGGREGATION
3:151090424	*MED12L*	REACTOME_PLATELET_ACTIVATION_SIGNALING_AND_AGGREGATION
3:151099733	*MED12L*	REACTOME_PLATELET_ACTIVATION_SIGNALING_AND_AGGREGATION
4:90804532	*SNCA_2*	REACTOME_PLATELET_ACTIVATION_SIGNALING_AND_AGGREGATION
4:90868355	*SNCA_2*	REACTOME_PLATELET_ACTIVATION_SIGNALING_AND_AGGREGATION
6:112009427	*FYN*	REACTOME_PLATELET_ACTIVATION_SIGNALING_AND_AGGREGATION
6:112056759	*FYN*	REACTOME_PLATELET_ACTIVATION_SIGNALING_AND_AGGREGATION
6:112151452	*FYN*	REACTOME_PLATELET_ACTIVATION_SIGNALING_AND_AGGREGATION
8:130914130	*FAM49B*	REACTOME_PLATELET_ACTIVATION_SIGNALING_AND_AGGREGATION
19:3156505	*SPPL2B*	REACTOME_PLATELET_ACTIVATION_SIGNALING_AND_AGGREGATION
6:112009427	*FYN*	REACTOME_PLATELET_ADHESION_TO_EXPOSED_COLLAGEN
6:112056759	*FYN*	REACTOME_PLATELET_ADHESION_TO_EXPOSED_COLLAGEN
6:112151452	*FYN*	REACTOME_PLATELET_ADHESION_TO_EXPOSED_COLLAGEN
11:10647977	*RNF141*	REACTOME_PLATELET_HOMEOSTASIS
11:10648481	*RNF141*	REACTOME_PLATELET_HOMEOSTASIS
11:10660840	*RNF141*	REACTOME_PLATELET_HOMEOSTASIS
3:48199695	*IP6K2*	REACTOME_POLO_LIKE_KINASE_MEDIATED_EVENTS
3:48214438	*IP6K2*	REACTOME_POLO_LIKE_KINASE_MEDIATED_EVENTS
6:31862876	*HLA-DRB5*	REACTOME_POSITIVE_EPIGENETIC_REGULATION_OF_RRNA_EXPRESSION
1:172386261	*VAMP4*	REACTOME_POST_TRANSLATIONAL_MODIFICATION:_SYNTHESIS_OF_GPI_ANCHORED_PROTEINS
1:172388441	*VAMP4*	REACTOME_POST_TRANSLATIONAL_MODIFICATION:_SYNTHESIS_OF_GPI_ANCHORED_PROTEINS
1:172389198	*VAMP4*	REACTOME_POST_TRANSLATIONAL_MODIFICATION:_SYNTHESIS_OF_GPI_ANCHORED_PROTEINS
4:15709252	*BST1*	REACTOME_POST_TRANSLATIONAL_MODIFICATION:_SYNTHESIS_OF_GPI_ANCHORED_PROTEINS
4:15713608	*BST1*	REACTOME_POST_TRANSLATIONAL_MODIFICATION:_SYNTHESIS_OF_GPI_ANCHORED_PROTEINS
4:15724372	*BST1*	REACTOME_POST_TRANSLATIONAL_MODIFICATION:_SYNTHESIS_OF_GPI_ANCHORED_PROTEINS
4:15737882	*BST1*	REACTOME_POST_TRANSLATIONAL_MODIFICATION:_SYNTHESIS_OF_GPI_ANCHORED_PROTEINS
4:76986566	*FAM47E-STBD1*	REACTOME_POST_TRANSLATIONAL_MODIFICATION:_SYNTHESIS_OF_GPI_ANCHORED_PROTEINS
4:77021433	*FAM47E-STBD1*	REACTOME_POST_TRANSLATIONAL_MODIFICATION:_SYNTHESIS_OF_GPI_ANCHORED_PROTEINS
6:133025715	*RPS12*	REACTOME_POST_TRANSLATIONAL_MODIFICATION:_SYNTHESIS_OF_GPI_ANCHORED_PROTEINS
6:133028870	*RPS12*	REACTOME_POST_TRANSLATIONAL_MODIFICATION:_SYNTHESIS_OF_GPI_ANCHORED_PROTEINS
6:133034076	*RPS12*	REACTOME_POST_TRANSLATIONAL_MODIFICATION:_SYNTHESIS_OF_GPI_ANCHORED_PROTEINS
1:172386261	*VAMP4*	REACTOME_POST_TRANSLATIONAL_PROTEIN_MODIFICATION
1:172388441	*VAMP4*	REACTOME_POST_TRANSLATIONAL_PROTEIN_MODIFICATION
1:172389198	*VAMP4*	REACTOME_POST_TRANSLATIONAL_PROTEIN_MODIFICATION
2:134977261	*TMEM163*	REACTOME_POST_TRANSLATIONAL_PROTEIN_MODIFICATION
2:135194840	*TMEM163*	REACTOME_POST_TRANSLATIONAL_PROTEIN_MODIFICATION
2:135202455	*TMEM163*	REACTOME_POST_TRANSLATIONAL_PROTEIN_MODIFICATION
3:48199695	*IP6K2*	REACTOME_POST_TRANSLATIONAL_PROTEIN_MODIFICATION
3:48214438	*IP6K2*	REACTOME_POST_TRANSLATIONAL_PROTEIN_MODIFICATION
3:49162284	*IP6K2*	REACTOME_POST_TRANSLATIONAL_PROTEIN_MODIFICATION
3:49162583	*IP6K2*	REACTOME_POST_TRANSLATIONAL_PROTEIN_MODIFICATION
3:49406095	*IP6K2*	REACTOME_POST_TRANSLATIONAL_PROTEIN_MODIFICATION
3:49424231	*IP6K2*	REACTOME_POST_TRANSLATIONAL_PROTEIN_MODIFICATION
3:49450137	*IP6K2*	REACTOME_POST_TRANSLATIONAL_PROTEIN_MODIFICATION
3:49462410	*IP6K2*	REACTOME_POST_TRANSLATIONAL_PROTEIN_MODIFICATION
3:49561208	*IP6K2*	REACTOME_POST_TRANSLATIONAL_PROTEIN_MODIFICATION
3:49740895	*IP6K2*	REACTOME_POST_TRANSLATIONAL_PROTEIN_MODIFICATION
3:121324643	*KPNA1*	REACTOME_POST_TRANSLATIONAL_PROTEIN_MODIFICATION

3:121340955	*KPNA1*	REACTOME_POST_TRANSLATIONAL_PROTEIN_MODIFICATION
3:121411876	*KPNA1*	REACTOME_POST_TRANSLATIONAL_PROTEIN_MODIFICATION
3:121412702	*KPNA1*	REACTOME_POST_TRANSLATIONAL_PROTEIN_MODIFICATION
3:122645304	*KPNA1*	REACTOME_POST_TRANSLATIONAL_PROTEIN_MODIFICATION
3:182656610	*MCCC1*	REACTOME_POST_TRANSLATIONAL_PROTEIN_MODIFICATION
3:182976382	*MCCC1*	REACTOME_POST_TRANSLATIONAL_PROTEIN_MODIFICATION
3:182979912	*MCCC1*	REACTOME_POST_TRANSLATIONAL_PROTEIN_MODIFICATION
3:183005320	*MCCC1*	REACTOME_POST_TRANSLATIONAL_PROTEIN_MODIFICATION
4:15608677	*BST1*	REACTOME_POST_TRANSLATIONAL_PROTEIN_MODIFICATION
4:15709252	*BST1*	REACTOME_POST_TRANSLATIONAL_PROTEIN_MODIFICATION
4:15713608	*BST1*	REACTOME_POST_TRANSLATIONAL_PROTEIN_MODIFICATION
4:15724372	*BST1*	REACTOME_POST_TRANSLATIONAL_PROTEIN_MODIFICATION
4:15737882	*BST1*	REACTOME_POST_TRANSLATIONAL_PROTEIN_MODIFICATION
4:76695927	*FAM47E-STBD1*	REACTOME_POST_TRANSLATIONAL_PROTEIN_MODIFICATION
4:76986566	*FAM47E-STBD1*	REACTOME_POST_TRANSLATIONAL_PROTEIN_MODIFICATION
4:77021433	*FAM47E-STBD1*	REACTOME_POST_TRANSLATIONAL_PROTEIN_MODIFICATION
4:113938109	*CAMK2D*	REACTOME_POST_TRANSLATIONAL_PROTEIN_MODIFICATION
4:114077386	*CAMK2D*	REACTOME_POST_TRANSLATIONAL_PROTEIN_MODIFICATION
4:114217997	*CAMK2D*	REACTOME_POST_TRANSLATIONAL_PROTEIN_MODIFICATION
5:133710339	*C5orf24*	REACTOME_POST_TRANSLATIONAL_PROTEIN_MODIFICATION
6:32151443	*HLA-DRB5*	REACTOME_POST_TRANSLATIONAL_PROTEIN_MODIFICATION
6:32814942	*HLA-DRB5*	REACTOME_POST_TRANSLATIONAL_PROTEIN_MODIFICATION
6:133025715	*RPS12*	REACTOME_POST_TRANSLATIONAL_PROTEIN_MODIFICATION
6:133028870	*RPS12*	REACTOME_POST_TRANSLATIONAL_PROTEIN_MODIFICATION
6:133034076	*RPS12*	REACTOME_POST_TRANSLATIONAL_PROTEIN_MODIFICATION
7:66096028	*GS1-124K5.11*	REACTOME_POST_TRANSLATIONAL_PROTEIN_MODIFICATION
9:18650547	*SH3GL2_2*	REACTOME_POST_TRANSLATIONAL_PROTEIN_MODIFICATION
9:33845203	*UBAP2*	REACTOME_POST_TRANSLATIONAL_PROTEIN_MODIFICATION
10:103185542	*GBF1*	REACTOME_POST_TRANSLATIONAL_PROTEIN_MODIFICATION
10:103308244	*GBF1*	REACTOME_POST_TRANSLATIONAL_PROTEIN_MODIFICATION
10:104005410	*GBF1*	REACTOME_POST_TRANSLATIONAL_PROTEIN_MODIFICATION
10:104034550	*GBF1*	REACTOME_POST_TRANSLATIONAL_PROTEIN_MODIFICATION
10:104258303	*GBF1*	REACTOME_POST_TRANSLATIONAL_PROTEIN_MODIFICATION
10:121683206	*INPP5F*	REACTOME_POST_TRANSLATIONAL_PROTEIN_MODIFICATION
10:121686714	*INPP5F*	REACTOME_POST_TRANSLATIONAL_PROTEIN_MODIFICATION
10:121689030	*INPP5F*	REACTOME_POST_TRANSLATIONAL_PROTEIN_MODIFICATION
12:132687184	*FBRSL1*	REACTOME_POST_TRANSLATIONAL_PROTEIN_MODIFICATION
14:76159192	*RPS6KL1*	REACTOME_POST_TRANSLATIONAL_PROTEIN_MODIFICATION
15:61293467	*VPS13C*	REACTOME_POST_TRANSLATIONAL_PROTEIN_MODIFICATION
16:50730088	*CASC16*	REACTOME_POST_TRANSLATIONAL_PROTEIN_MODIFICATION
16:50730446	*CASC16*	REACTOME_POST_TRANSLATIONAL_PROTEIN_MODIFICATION
16:50745926	*CASC16*	REACTOME_POST_TRANSLATIONAL_PROTEIN_MODIFICATION
17:7491546	*V1NB1*	REACTOME_POST_TRANSLATIONAL_PROTEIN_MODIFICATION
17:7578671	*V1NB1*	REACTOME_POST_TRANSLATIONAL_PROTEIN_MODIFICATION
17:44800946	*WNT3*	REACTOME_POST_TRANSLATIONAL_PROTEIN_MODIFICATION
17:44833217	*WNT3*	REACTOME_POST_TRANSLATIONAL_PROTEIN_MODIFICATION
17:45059032	*WNT3*	REACTOME_POST_TRANSLATIONAL_PROTEIN_MODIFICATION
17:45089680	*WNT3*	REACTOME_POST_TRANSLATIONAL_PROTEIN_MODIFICATION
1:154804988	*GBAP1*	REACTOME_POTASSIUM_CHANNELS
2:18143082	*KCNS3*	REACTOME_POTASSIUM_CHANNELS
2:18176120	*KCNS3*	REACTOME_POTASSIUM_CHANNELS
2:18247777	*KCNS3*	REACTOME_POTASSIUM_CHANNELS
2:18296870	*KCNS3*	REACTOME_POTASSIUM_CHANNELS

2:18335515	*KCNS3*	REACTOME_POTASSIUM_CHANNELS
3:19312697	*SATB1*	REACTOME_POTASSIUM_CHANNELS
3:19334895	*SATB1*	REACTOME_POTASSIUM_CHANNELS
6:29579544	*TRIM40*	REACTOME_POTASSIUM_CHANNELS
17:7832034	*V1NB1*	REACTOME_POTASSIUM_CHANNELS
21:39197323	*DYRK1A*	REACTOME_POTASSIUM_CHANNELS
6:33381459	*HLA-DRB5*	REACTOME_PRC2_METHYLATES_HISTONES_AND_DNA
6:32186193	*HLA-DRB5*	REACTOME_PRE_NOTCH_EXPRESSION_AND_PROCESSING
17:7578671	*V1NB1*	REACTOME_PRE_NOTCH_EXPRESSION_AND_PROCESSING
6:32186193	*HLA-DRB5*	REACTOME_PRE_NOTCH_PROCESSING_IN_GOLGI
6:32186193	*HLA-DRB5*	REACTOME_PRE_NOTCH_PROCESSING_IN_THE_ENDOPLASMIC_RETICULUM
6:30628082	*TRIM40*	REACTOME_PROCESSING_OF_CAPPED_INTRON_CONTAINING_PRE_MRNA
6:30631063	*TRIM40*	REACTOME_PROCESSING_OF_CAPPED_INTRON_CONTAINING_PRE_MRNA
6:30639369	*TRIM40*	REACTOME_PROCESSING_OF_CAPPED_INTRON_CONTAINING_PRE_MRNA
11:82886475	*DLG2*	REACTOME_PROCESSING_OF_CAPPED_INTRON_CONTAINING_PRE_MRNA
17:42929985	*CRHR1_2*	REACTOME_PROCESSING_OF_CAPPED_INTRON_CONTAINING_PRE_MRNA
11:82886475	*DLG2*	REACTOME_PROCESSING_OF_CAPPED_INTRONLESS_PRE_MRNA
17:59922925	*BRIP1*	REACTOME_PROCESSING_OF_DNA_DOUBLE_STRAND_BREAK_ENDS
11:82886475	*DLG2*	REACTOME_PROCESSING_OF_INTRONLESS_PRE_MRNAS
1:156059276	*GBAP1*	REACTOME_PROGRAMMED_CELL_DEATH
6:32814942	*HLA-DRB5*	REACTOME_PROGRAMMED_CELL_DEATH
8:23050400	*BIN3*	REACTOME_PROGRAMMED_CELL_DEATH
17:7578671	*V1NB1*	REACTOME_PROGRAMMED_CELL_DEATH
17:43132290	*CRHR1_2*	REACTOME_PROGRAMMED_CELL_DEATH
17:43999203	*WNT3*	REACTOME_PROGRAMMED_CELL_DEATH
17:44005297	*WNT3*	REACTOME_PROGRAMMED_CELL_DEATH
17:44019712	*WNT3*	REACTOME_PROGRAMMED_CELL_DEATH
17:44060492	*WNT3*	REACTOME_PROGRAMMED_CELL_DEATH
17:44065263	*WNT3*	REACTOME_PROGRAMMED_CELL_DEATH
17:44081178	*WNT3*	REACTOME_PROGRAMMED_CELL_DEATH
17:44083297	*WNT3*	REACTOME_PROGRAMMED_CELL_DEATH
10:103185542	*GBF1*	REACTOME_PROLACTIN_RECEPTOR_SIGNALING
10:103308244	*GBF1*	REACTOME_PROLACTIN_RECEPTOR_SIGNALING
17:40392358	*RETREG3*	REACTOME_PROLACTIN_RECEPTOR_SIGNALING
4:15608677	*BST1*	REACTOME_PROTEIN_FOLDING
1:155181232	*GBAP1*	REACTOME_PROTEIN_LOCALIZATION
3:150298317	*MED12L*	REACTOME_PROTEIN_LOCALIZATION
6:31607942	*HLA-DRB5*	REACTOME_PROTEIN_LOCALIZATION
12:39960237	*LRRK2_2*	REACTOME_PROTEIN_LOCALIZATION
12:133295501	*FBRSL1*	REACTOME_PROTEIN_LOCALIZATION
11:83188607	*DLG2*	REACTOME_PROTEIN_PROTEIN_INTERACTIONS_AT_SYNAPSES
11:83219006	*DLG2*	REACTOME_PROTEIN_PROTEIN_INTERACTIONS_AT_SYNAPSES
11:83538733	*DLG2*	REACTOME_PROTEIN_PROTEIN_INTERACTIONS_AT_SYNAPSES
11:83544472	*DLG2*	REACTOME_PROTEIN_PROTEIN_INTERACTIONS_AT_SYNAPSES
11:83656201	*DLG2*	REACTOME_PROTEIN_PROTEIN_INTERACTIONS_AT_SYNAPSES
11:83700228	*DLG2*	REACTOME_PROTEIN_PROTEIN_INTERACTIONS_AT_SYNAPSES
11:83762918	*DLG2*	REACTOME_PROTEIN_PROTEIN_INTERACTIONS_AT_SYNAPSES
11:83894247	*DLG2*	REACTOME_PROTEIN_PROTEIN_INTERACTIONS_AT_SYNAPSES
11:84021037	*DLG2*	REACTOME_PROTEIN_PROTEIN_INTERACTIONS_AT_SYNAPSES
11:84324361	*DLG2*	REACTOME_PROTEIN_PROTEIN_INTERACTIONS_AT_SYNAPSES
11:84373496	*DLG2*	REACTOME_PROTEIN_PROTEIN_INTERACTIONS_AT_SYNAPSES
11:84403409	*DLG2*	REACTOME_PROTEIN_PROTEIN_INTERACTIONS_AT_SYNAPSES
17:7101771	*V1NB1*	REACTOME_PROTEIN_PROTEIN_INTERACTIONS_AT_SYNAPSES

5:133710339	*C5orf24*	REACTOME_PROTEIN_UBIQUITINATION
9:33845203	*UBAP2*	REACTOME_PROTEIN_UBIQUITINATION
6:32814942	*HLA-DRB5*	REACTOME_PTEN_REGULATION
17:7578671	*V1NB1*	REACTOME_PTEN_REGULATION
7:23277372	*GPNMB*	REACTOME_PTK6_PROMOTES_HIF1A_STABILIZATION
7:23293746	*GPNMB*	REACTOME_PTK6_PROMOTES_HIF1A_STABILIZATION
7:23313171	*GPNMB*	REACTOME_PTK6_PROMOTES_HIF1A_STABILIZATION
12:40614434	*LRRK2_2*	REACTOME_PTK6_PROMOTES_HIF1A_STABILIZATION
12:40614656	*LRRK2_2*	REACTOME_PTK6_PROMOTES_HIF1A_STABILIZATION
12:40617231	*LRRK2_2*	REACTOME_PTK6_PROMOTES_HIF1A_STABILIZATION
12:40653993	*LRRK2_2*	REACTOME_PTK6_PROMOTES_HIF1A_STABILIZATION
12:40698422	*LRRK2_2*	REACTOME_PTK6_PROMOTES_HIF1A_STABILIZATION
12:40707861	*LRRK2_2*	REACTOME_PTK6_PROMOTES_HIF1A_STABILIZATION
12:40753796	*LRRK2_2*	REACTOME_PTK6_PROMOTES_HIF1A_STABILIZATION
3:49406095	*IP6K2*	REACTOME_PTK6_REGULATES_RHO_GTPASES_RAS_GTPASE_AND_MAP_KINASES
3:49424231	*IP6K2*	REACTOME_PTK6_REGULATES_RHO_GTPASES_RAS_GTPASE_AND_MAP_KINASES
3:49450137	*IP6K2*	REACTOME_PTK6_REGULATES_RHO_GTPASES_RAS_GTPASE_AND_MAP_KINASES
10:104847713	*GBF1*	REACTOME_PURINE_CATABOLISM
10:104861736	*GBF1*	REACTOME_PURINE_CATABOLISM
10:104926485	*GBF1*	REACTOME_PURINE_CATABOLISM
10:104929716	*GBF1*	REACTOME_PURINE_CATABOLISM
11:10392432	*RNF141*	REACTOME_PURINE_SALVAGE
11:10487997	*RNF141*	REACTOME_PURINE_SALVAGE
17:76177567	*DNAH17*	REACTOME_PYRIMIDINE_SALVAGE
2:135921205	*TMEM163*	REACTOME_RAB_GEFS_EXCHANGE_GTP_FOR_GDP_ON_RABS
2:135921205	*TMEM163*	REACTOME_RAB_REGULATION_OF_TRAFFICKING
4:114424939	*CAMK2D*	REACTOME_RAF_ACTIVATION
4:114424939	*CAMK2D*	REACTOME_RAS_ACTIVATION_UPON_CA2PLUS_INFLUX_THROUGH_NMDA_RECEPTOR
11:83188607	*DLG2*	REACTOME_RAS_ACTIVATION_UPON_CA2PLUS_INFLUX_THROUGH_NMDA_RECEPTOR
11:83219006	*DLG2*	REACTOME_RAS_ACTIVATION_UPON_CA2PLUS_INFLUX_THROUGH_NMDA_RECEPTOR
11:83538733	*DLG2*	REACTOME_RAS_ACTIVATION_UPON_CA2PLUS_INFLUX_THROUGH_NMDA_RECEPTOR
11:83544472	*DLG2*	REACTOME_RAS_ACTIVATION_UPON_CA2PLUS_INFLUX_THROUGH_NMDA_RECEPTOR
11:83656201	*DLG2*	REACTOME_RAS_ACTIVATION_UPON_CA2PLUS_INFLUX_THROUGH_NMDA_RECEPTOR
11:83700228	*DLG2*	REACTOME_RAS_ACTIVATION_UPON_CA2PLUS_INFLUX_THROUGH_NMDA_RECEPTOR
11:83762918	*DLG2*	REACTOME_RAS_ACTIVATION_UPON_CA2PLUS_INFLUX_THROUGH_NMDA_RECEPTOR
11:83894247	*DLG2*	REACTOME_RAS_ACTIVATION_UPON_CA2PLUS_INFLUX_THROUGH_NMDA_RECEPTOR
11:84021037	*DLG2*	REACTOME_RAS_ACTIVATION_UPON_CA2PLUS_INFLUX_THROUGH_NMDA_RECEPTOR
11:84324361	*DLG2*	REACTOME_RAS_ACTIVATION_UPON_CA2PLUS_INFLUX_THROUGH_NMDA_RECEPTOR
11:84373496	*DLG2*	REACTOME_RAS_ACTIVATION_UPON_CA2PLUS_INFLUX_THROUGH_NMDA_RECEPTOR
11:84403409	*DLG2*	REACTOME_RAS_ACTIVATION_UPON_CA2PLUS_INFLUX_THROUGH_NMDA_RECEPTOR
17:7101771	*V1NB1*	REACTOME_RAS_ACTIVATION_UPON_CA2PLUS_INFLUX_THROUGH_NMDA_RECEPTOR
12:133295501	*FBRSL1*	REACTOME_RECEPTOR_MEDIATED_MITOPHAGY
5:133710339	*C5orf24*	REACTOME_RECOGNITION_OF_DNA_DAMAGE_BY_PCNA_CONTAINING_REPLICATION_COMPLEX
12:133206648	*FBRSL1*	REACTOME_RECOGNITION_OF_DNA_DAMAGE_BY_PCNA_CONTAINING_REPLICATION_COMPLEX
12:133223685	*FBRSL1*	REACTOME_RECOGNITION_OF_DNA_DAMAGE_BY_PCNA_CONTAINING_REPLICATION_COMPLEX
12:133225045	*FBRSL1*	REACTOME_RECOGNITION_OF_DNA_DAMAGE_BY_PCNA_CONTAINING_REPLICATION_COMPLEX
10:104258303	*GBF1*	REACTOME_RECRUITMENT_OF_NUMA_TO_MITOTIC_CENTROSOMES
17:40818584	*RETREG3*	REACTOME_RECRUITMENT_OF_NUMA_TO_MITOTIC_CENTROSOMES
1:171945488	*VAMP4*	REACTOME_RECYCLING_PATHWAY_OF_L1
1:172137119	*VAMP4*	REACTOME_RECYCLING_PATHWAY_OF_L1
1:172386261	*VAMP4*	REACTOME_RECYCLING_PATHWAY_OF_L1
9:17579690	*SH3GL2_2*	REACTOME_RECYCLING_PATHWAY_OF_L1
9:17609302	*SH3GL2_2*	REACTOME_RECYCLING_PATHWAY_OF_L1

9:17627444	*SH3GL2_2*	REACTOME_RECYCLING_PATHWAY_OF_L1
9:17710876	*SH3GL2_2*	REACTOME_RECYCLING_PATHWAY_OF_L1
9:17732590	*SH3GL2_2*	REACTOME_RECYCLING_PATHWAY_OF_L1
9:17737514	*SH3GL2_2*	REACTOME_RECYCLING_PATHWAY_OF_L1
9:17758945	*SH3GL2_2*	REACTOME_RECYCLING_PATHWAY_OF_L1
6:112009427	*FYN*	REACTOME_REELIN_SIGNALLING_PATHWAY
6:112056759	*FYN*	REACTOME_REELIN_SIGNALLING_PATHWAY
6:112151452	*FYN*	REACTOME_REELIN_SIGNALLING_PATHWAY
8:23050400	*BIN3*	REACTOME_REGULATED_NECROSIS
1:227067656	*ITPKB*	REACTOME_REGULATED_PROTEOLYSIS_OF_P75NTR
8:23050400	*BIN3*	REACTOME_REGULATION_BY_C_FLIP
6:32814942	*HLA-DRB5*	REACTOME_REGULATION_OF_APOPTOSIS
3:49561208	*IP6K2*	REACTOME_REGULATION_OF_EXPRESSION_OF_SLITS_AND_ROBOS
6:32814942	*HLA-DRB5*	REACTOME_REGULATION_OF_EXPRESSION_OF_SLITS_AND_ROBOS
10:121429633	*INPP5F*	REACTOME_REGULATION_OF_HSF1_MEDIATED_HEAT_SHOCK_RESPONSE
10:121437329	*INPP5F*	REACTOME_REGULATION_OF_HSF1_MEDIATED_HEAT_SHOCK_RESPONSE
17:40132591	*RETREG3*	REACTOME_REGULATION_OF_HSF1_MEDIATED_HEAT_SHOCK_RESPONSE
3:49162284	*IP6K2*	JLATION_OF_INSULIN_LIKE_GROWTH_FACTOR_IGF_TRANSPORT_AND_UPTAKE_BY_INSULIN_LIKE_GROWTH_FACTOR_BINDINC
3:49162583	*IP6K2*	JLATION_OF_INSULIN_LIKE_GROWTH_FACTOR_IGF_TRANSPORT_AND_UPTAKE_BY_INSULIN_LIKE_GROWTH_FACTOR_BINDINC
2:18143082	*KCNS3*	REACTOME_REGULATION_OF_INSULIN_SECRETION
2:18176120	*KCNS3*	REACTOME_REGULATION_OF_INSULIN_SECRETION
2:18247777	*KCNS3*	REACTOME_REGULATION_OF_INSULIN_SECRETION
2:18296870	*KCNS3*	REACTOME_REGULATION_OF_INSULIN_SECRETION
2:18335515	*KCNS3*	REACTOME_REGULATION_OF_INSULIN_SECRETION
3:48835245	*IP6K2*	REACTOME_REGULATION_OF_INSULIN_SECRETION
3:123057397	*KPNA1*	REACTOME_REGULATION_OF_INSULIN_SECRETION
19:3156505	*SPPL2B*	REACTOME_REGULATION_OF_INSULIN_SECRETION
6:112009427	*FYN*	REACTOME_REGULATION_OF_KIT_SIGNALING
6:112056759	*FYN*	REACTOME_REGULATION_OF_KIT_SIGNALING
6:112151452	*FYN*	REACTOME_REGULATION_OF_KIT_SIGNALING
2:101439596	*MAP4K4*	EACTOME_REGULATION_OF_LIPID_METABOLISM_BY_PEROXISOME_PROLIFERATOR_ACTIVATED_RECEPTOR_ALPHA_PPARALP
2:101550181	*MAP4K4*	EACTOME_REGULATION_OF_LIPID_METABOLISM_BY_PEROXISOME_PROLIFERATOR_ACTIVATED_RECEPTOR_ALPHA_PPARALP
2:101603812	*MAP4K4*	EACTOME_REGULATION_OF_LIPID_METABOLISM_BY_PEROXISOME_PROLIFERATOR_ACTIVATED_RECEPTOR_ALPHA_PPARALP
4:17622910	*LCORL*	EACTOME_REGULATION_OF_LIPID_METABOLISM_BY_PEROXISOME_PROLIFERATOR_ACTIVATED_RECEPTOR_ALPHA_PPARALP
4:17632603	*LCORL*	EACTOME_REGULATION_OF_LIPID_METABOLISM_BY_PEROXISOME_PROLIFERATOR_ACTIVATED_RECEPTOR_ALPHA_PPARALP
15:61293467	*VPS13C*	EACTOME_REGULATION_OF_LIPID_METABOLISM_BY_PEROXISOME_PROLIFERATOR_ACTIVATED_RECEPTOR_ALPHA_PPARALP
4:114424939	*CAMK2D*	REACTOME_REGULATION_OF_MECP2_EXPRESSION_AND_ACTIVITY
6:32814942	*HLA-DRB5*	REACTOME_REGULATION_OF_MITOTIC_CELL_CYCLE
10:103185542	*GBF1*	REACTOME_REGULATION_OF_MITOTIC_CELL_CYCLE
10:103308244	*GBF1*	REACTOME_REGULATION_OF_MITOTIC_CELL_CYCLE
17:45215865	*WNT3*	REACTOME_REGULATION_OF_MITOTIC_CELL_CYCLE
17:45244822	*WNT3*	REACTOME_REGULATION_OF_MITOTIC_CELL_CYCLE
6:32814942	*HLA-DRB5*	REACTOME_REGULATION_OF_MRNA_STABILITY_BY_PROTEINS_THAT_BIND_AU_RICH_ELEMENTS
10:103185542	*GBF1*	REACTOME_REGULATION_OF_PLK1_ACTIVITY_AT_G2_M_TRANSITION
10:103308244	*GBF1*	REACTOME_REGULATION_OF_PLK1_ACTIVITY_AT_G2_M_TRANSITION
10:104258303	*GBF1*	REACTOME_REGULATION_OF_PLK1_ACTIVITY_AT_G2_M_TRANSITION
17:7578671	*V1NB1*	REACTOME_REGULATION_OF_PTEN_GENE_TRANSCRIPTION
6:32814942	*HLA-DRB5*	REACTOME_REGULATION_OF_PTEN_STABILITY_AND_ACTIVITY
6:32814942	*HLA-DRB5*	REACTOME_REGULATION_OF_RAS_BY_GAPS
6:32814942	*HLA-DRB5*	REACTOME_REGULATION_OF_RUNX2_EXPRESSION_AND_ACTIVITY
6:32814942	*HLA-DRB5*	REACTOME_REGULATION_OF_RUNX3_EXPRESSION_AND_ACTIVITY
6:112009427	*FYN*	REACTOME_REGULATION_OF_SIGNALING_BY_CBL
6:112056759	*FYN*	REACTOME_REGULATION_OF_SIGNALING_BY_CBL

6:112151452	*FYN*	REACTOME_REGULATION_OF_SIGNALING_BY_CBL
19:2335872	*SPPL2B*	REACTOME_REGULATION_OF_TNFR1_SIGNALING
19:2346990	*SPPL2B*	REACTOME_REGULATION_OF_TNFR1_SIGNALING
19:2352757	*SPPL2B*	REACTOME_REGULATION_OF_TNFR1_SIGNALING
6:31862876	*HLA-DRB5*	REACTOME_REGULATION_OF_TP53_ACTIVITY
17:7578671	*V1NB1*	REACTOME_REGULATION_OF_TP53_ACTIVITY
17:59922925	*BRIP1*	REACTOME_REGULATION_OF_TP53_ACTIVITY
17:7578671	*V1NB1*	REACTOME_REGULATION_OF_TP53_ACTIVITY_THROUGH_ACETYLATION
17:7578671	*V1NB1*	REACTOME_REGULATION_OF_TP53_ACTIVITY_THROUGH_ASSOCIATION_WITH_CO_FACTORS
6:31862876	*HLA-DRB5*	REACTOME_REGULATION_OF_TP53_ACTIVITY_THROUGH_METHYLATION
17:7578671	*V1NB1*	REACTOME_REGULATION_OF_TP53_ACTIVITY_THROUGH_METHYLATION
17:7578671	*V1NB1*	REACTOME_REGULATION_OF_TP53_ACTIVITY_THROUGH_PHOSPHORYLATION
17:59922925	*BRIP1*	REACTOME_REGULATION_OF_TP53_ACTIVITY_THROUGH_PHOSPHORYLATION
17:7578671	*V1NB1*	REACTOME_REGULATION_OF_TP53_EXPRESSION_AND_DEGRADATION
19:3022354	*SPPL2B*	REACTOME_REPRESSION_OF_WNT_TARGET_GENES
19:3036350	*SPPL2B*	REACTOME_REPRESSION_OF_WNT_TARGET_GENES
1:156059276	*GBAP1*	REACTOME_REPRODUCTION
5:134315853	*C5orf24*	REACTOME_REPRODUCTION
14:75495059	*RPS6KL1*	REACTOME_REPRODUCTION
12:133206648	*FBRSL1*	REACTOME_RESOLUTION_OF_ABASIC_SITES_AP_SITES
12:133223685	*FBRSL1*	REACTOME_RESOLUTION_OF_ABASIC_SITES_AP_SITES
12:133225045	*FBRSL1*	REACTOME_RESOLUTION_OF_ABASIC_SITES_AP_SITES
12:133206648	*FBRSL1*	REACTOME_RESOLUTION_OF_AP_SITES_VIA_THE_MULTIPLE_NUCLEOTIDE_PATCH_REPLACEMENT_PATHWAY
12:133223685	*FBRSL1*	REACTOME_RESOLUTION_OF_AP_SITES_VIA_THE_MULTIPLE_NUCLEOTIDE_PATCH_REPLACEMENT_PATHWAY
12:133225045	*FBRSL1*	REACTOME_RESOLUTION_OF_AP_SITES_VIA_THE_MULTIPLE_NUCLEOTIDE_PATCH_REPLACEMENT_PATHWAY
17:59922925	*BRIP1*	REACTOME_RESOLUTION_OF_D_LOOP_STRUCTURES
17:59922925	*BRIP1*	REACTOME_RESOLUTION_OF_D_LOOP_STRUCTURES_THROUGH_SYNTHESIS_DEPENDENT_STRAND_ANNEALING_SDSA
12:122816734	*HIP1R*	REACTOME_RESOLUTION_OF_SISTER_CHROMATID_COHESION
12:122817580	*HIP1R*	REACTOME_RESOLUTION_OF_SISTER_CHROMATID_COHESION
12:122853197	*HIP1R*	REACTOME_RESOLUTION_OF_SISTER_CHROMATID_COHESION
12:123057792	*HIP1R*	REACTOME_RESOLUTION_OF_SISTER_CHROMATID_COHESION
12:123110365	*HIP1R*	REACTOME_RESOLUTION_OF_SISTER_CHROMATID_COHESION
12:123110785	*HIP1R*	REACTOME_RESOLUTION_OF_SISTER_CHROMATID_COHESION
1:161171736	*FCGR2A*	ESPIRATORY_ELECTRON_TRANSPORT_ATP_SYNTHESIS_BY_CHEMIOSMOTIC_COUPLING_AND_HEAT_PRODUCTION_BY_UNCOU
5:60273923	*ELOVL7*	ESPIRATORY_ELECTRON_TRANSPORT_ATP_SYNTHESIS_BY_CHEMIOSMOTIC_COUPLING_AND_HEAT_PRODUCTION_BY_UNCOU
1:161171736	*FCGR2A*	REACTOME_RESPIRATORY_ELECTRON_TRANSPORT
5:60273923	*ELOVL7*	REACTOME_RESPIRATORY_ELECTRON_TRANSPORT
4:90804532	*SNCA_2*	REACTOME_RESPONSE_TO_ELEVATED_PLATELET_CYTOSOLIC_CA2PLUS
4:90868355	*SNCA_2*	REACTOME_RESPONSE_TO_ELEVATED_PLATELET_CYTOSOLIC_CA2PLUS
8:130914130	*FAM49B*	REACTOME_RESPONSE_TO_ELEVATED_PLATELET_CYTOSOLIC_CA2PLUS
1:171945488	*VAMP4*	REACTOME_RETROGRADE_NEUROTROPHIN_SIGNALLING
1:172137119	*VAMP4*	REACTOME_RETROGRADE_NEUROTROPHIN_SIGNALLING
1:172386261	*VAMP4*	REACTOME_RETROGRADE_NEUROTROPHIN_SIGNALLING
9:17579690	*SH3GL2_2*	REACTOME_RETROGRADE_NEUROTROPHIN_SIGNALLING
9:17609302	*SH3GL2_2*	REACTOME_RETROGRADE_NEUROTROPHIN_SIGNALLING
9:17627444	*SH3GL2_2*	REACTOME_RETROGRADE_NEUROTROPHIN_SIGNALLING
9:17710876	*SH3GL2_2*	REACTOME_RETROGRADE_NEUROTROPHIN_SIGNALLING
9:17732590	*SH3GL2_2*	REACTOME_RETROGRADE_NEUROTROPHIN_SIGNALLING
9:17737514	*SH3GL2_2*	REACTOME_RETROGRADE_NEUROTROPHIN_SIGNALLING
9:17758945	*SH3GL2_2*	REACTOME_RETROGRADE_NEUROTROPHIN_SIGNALLING
1:171689486	*VAMP4*	REACTOME_RETROGRADE_TRANSPORT_AT_THE_TRANS_GOLGI_NETWORK
17:44800946	*WNT3*	REACTOME_RETROGRADE_TRANSPORT_AT_THE_TRANS_GOLGI_NETWORK
17:44833217	*WNT3*	REACTOME_RETROGRADE_TRANSPORT_AT_THE_TRANS_GOLGI_NETWORK

8:21665306	*BIN3*	REACTOME_RET_SIGNALING
8:21668901	*BIN3*	REACTOME_RET_SIGNALING
1:155975202	*GBAP1*	REACTOME_RHO_GTPASE_CYCLE
1:161035780	*FCGR2A*	REACTOME_RHO_GTPASE_CYCLE
3:49406095	*IP6K2*	REACTOME_RHO_GTPASE_CYCLE
3:49424231	*IP6K2*	REACTOME_RHO_GTPASE_CYCLE
3:49450137	*IP6K2*	REACTOME_RHO_GTPASE_CYCLE
4:89711319	*SNCA_2*	REACTOME_RHO_GTPASE_CYCLE
4:89740128	*SNCA_2*	REACTOME_RHO_GTPASE_CYCLE
4:90002389	*SNCA_2*	REACTOME_RHO_GTPASE_CYCLE
5:59958372	*ELOVL7*	REACTOME_RHO_GTPASE_CYCLE
17:43472507	*CRHR1_2*	REACTOME_RHO_GTPASE_CYCLE
3:49406095	*IP6K2*	REACTOME_RHO_GTPASE_EFFECTORS
3:49424231	*IP6K2*	REACTOME_RHO_GTPASE_EFFECTORS
3:49450137	*IP6K2*	REACTOME_RHO_GTPASE_EFFECTORS
12:122816734	*HIP1R*	REACTOME_RHO_GTPASE_EFFECTORS
12:122817580	*HIP1R*	REACTOME_RHO_GTPASE_EFFECTORS
12:122853197	*HIP1R*	REACTOME_RHO_GTPASE_EFFECTORS
12:123057792	*HIP1R*	REACTOME_RHO_GTPASE_EFFECTORS
12:123110365	*HIP1R*	REACTOME_RHO_GTPASE_EFFECTORS
12:123110785	*HIP1R*	REACTOME_RHO_GTPASE_EFFECTORS
17:7101771	*V1NB1*	REACTOME_RHO_GTPASE_EFFECTORS
17:7129974	*V1NB1*	REACTOME_RHO_GTPASE_EFFECTORS
17:43302259	*CRHR1_2*	REACTOME_RHO_GTPASE_EFFECTORS
3:49406095	*IP6K2*	REACTOME_RHO_GTPASES_ACTIVATE_CIT
3:49424231	*IP6K2*	REACTOME_RHO_GTPASES_ACTIVATE_CIT
3:49450137	*IP6K2*	REACTOME_RHO_GTPASES_ACTIVATE_CIT
17:7101771	*V1NB1*	REACTOME_RHO_GTPASES_ACTIVATE_CIT
3:49406095	*IP6K2*	REACTOME_RHO_GTPASES_ACTIVATE_FORMINS
3:49424231	*IP6K2*	REACTOME_RHO_GTPASES_ACTIVATE_FORMINS
3:49450137	*IP6K2*	REACTOME_RHO_GTPASES_ACTIVATE_FORMINS
12:122816734	*HIP1R*	REACTOME_RHO_GTPASES_ACTIVATE_FORMINS
12:122817580	*HIP1R*	REACTOME_RHO_GTPASES_ACTIVATE_FORMINS
12:122853197	*HIP1R*	REACTOME_RHO_GTPASES_ACTIVATE_FORMINS
12:123057792	*HIP1R*	REACTOME_RHO_GTPASES_ACTIVATE_FORMINS
12:123110365	*HIP1R*	REACTOME_RHO_GTPASES_ACTIVATE_FORMINS
12:123110785	*HIP1R*	REACTOME_RHO_GTPASES_ACTIVATE_FORMINS
17:7129974	*V1NB1*	REACTOME_RHO_GTPASES_ACTIVATE_FORMINS
17:43302259	*CRHR1_2*	REACTOME_RHO_GTPASES_ACTIVATE_FORMINS
12:122816734	*HIP1R*	REACTOME_RHO_GTPASES_ACTIVATE_IQGAPS
12:122817580	*HIP1R*	REACTOME_RHO_GTPASES_ACTIVATE_IQGAPS
12:122853197	*HIP1R*	REACTOME_RHO_GTPASES_ACTIVATE_IQGAPS
3:49406095	*IP6K2*	REACTOME_RHO_GTPASES_ACTIVATE_KTN1
3:49424231	*IP6K2*	REACTOME_RHO_GTPASES_ACTIVATE_KTN1
3:49450137	*IP6K2*	REACTOME_RHO_GTPASES_ACTIVATE_KTN1
3:49406095	*IP6K2*	REACTOME_RHO_GTPASES_ACTIVATE_PKNS
3:49424231	*IP6K2*	REACTOME_RHO_GTPASES_ACTIVATE_PKNS
3:49450137	*IP6K2*	REACTOME_RHO_GTPASES_ACTIVATE_PKNS
3:49406095	*IP6K2*	REACTOME_RHO_GTPASES_ACTIVATE_RHOTEKIN_AND_RHOPHILINS
3:49424231	*IP6K2*	REACTOME_RHO_GTPASES_ACTIVATE_RHOTEKIN_AND_RHOPHILINS
3:49450137	*IP6K2*	REACTOME_RHO_GTPASES_ACTIVATE_RHOTEKIN_AND_RHOPHILINS
3:49406095	*IP6K2*	REACTOME_RHO_GTPASES_ACTIVATE_ROCKS
3:49424231	*IP6K2*	REACTOME_RHO_GTPASES_ACTIVATE_ROCKS

3:49450137	*IP6K2*	REACTOME_RHO_GTPASES_ACTIVATE_ROCKS
12:46134812	*SCAF11*	REACTOME_RMTS_METHYLATE_HISTONE_ARGININES
2:135708115	*TMEM163*	REACTOME_RNA_POLYMERASE_II_PRE_TRANSCRIPTION_EVENTS
6:31925679	*HLA-DRB5*	REACTOME_RNA_POLYMERASE_II_PRE_TRANSCRIPTION_EVENTS
2:135708115	*TMEM163*	REACTOME_RNA_POLYMERASE_II_TRANSCRIBES_SNRNA_GENES
11:82886475	*DLG2*	REACTOME_RNA_POLYMERASE_II_TRANSCRIBES_SNRNA_GENES
2:135708115	*TMEM163*	REACTOME_RNA_POLYMERASE_II_TRANSCRIPTION_ELONGATION
6:31925679	*HLA-DRB5*	REACTOME_RNA_POLYMERASE_II_TRANSCRIPTION_ELONGATION
11:82886475	*DLG2*	REACTOME_RNA_POLYMERASE_II_TRANSCRIPTION_TERMINATION
6:31862876	*HLA-DRB5*	REACTOME_RNA_POLYMERASE_I_TRANSCRIPTION_INITIATION
6:31862876	*HLA-DRB5*	REACTOME_RNA_POLYMERASE_I_TRANSCRIPTION
3:48835245	*IP6K2*	REACTOME_ROBO_RECEPTORS_BIND_AKAP5
6:112009427	*FYN*	REACTOME_ROLE_OF_LAT2_NTAL_LAB_ON_CALCIUM_MOBILIZATION
6:112056759	*FYN*	REACTOME_ROLE_OF_LAT2_NTAL_LAB_ON_CALCIUM_MOBILIZATION
6:112151452	*FYN*	REACTOME_ROLE_OF_LAT2_NTAL_LAB_ON_CALCIUM_MOBILIZATION
15:61293467	*VPS13C*	REACTOME_RORA_ACTIVATES_GENE_EXPRESSION
12:124219791	*HIP1R*	REACTOME_ROS_AND_RNS_PRODUCTION_IN_PHAGOCYTES
6:33252561	*HLA-DRB5*	REACTOME_RRNA_MODIFICATION_IN_THE_NUCLEUS_AND_CYTOSOL
6:33252561	*HLA-DRB5*	REACTOME_RRNA_PROCESSING_IN_THE_NUCLEUS_AND_CYTOSOL
6:33252561	*HLA-DRB5*	REACTOME_RRNA_PROCESSING
12:46134812	*SCAF11*	REACTOME_RUNX1_INTERACTS_WITH_CO_FACTORS_WHOSE_PRECISE_EFFECT_ON_RUNX1_TARGETS_IS_NOT_KNOWN
8:11381148	*CTSB*	REACTOME_RUNX1_REGULATES_TRANSCRIPTION_OF_GENES_INVOLVED_IN_BCR_SIGNALING
6:32814942	*HLA-DRB5*	REACTOME_RUNX1_REGULATES_TRANSCRIPTION_OF_GENES_INVOLVED_IN_DIFFERENTIATION_OF_HSCS
14:55515304	*GCH1*	REACTOME_RUNX1_REGULATES_TRANSCRIPTION_OF_GENES_INVOLVED_IN_DIFFERENTIATION_OF_KERATINOCYTES
17:7578671	*V1NB1*	REACTOME_RUNX3_REGULATES_CDKN1A_TRANSCRIPTION
16:30510571	*SETD1A*	REACTOME_RUNX3_REGULATES_IMMUNE_RESPONSE_AND_CELL_MIGRATION
16:30516492	*SETD1A*	REACTOME_RUNX3_REGULATES_IMMUNE_RESPONSE_AND_CELL_MIGRATION
16:30520856	*SETD1A*	REACTOME_RUNX3_REGULATES_IMMUNE_RESPONSE_AND_CELL_MIGRATION
6:32941506	*HLA-DRB5*	REACTOME_RUNX3_REGULATES_P14_ARF
6:32814942	*HLA-DRB5*	REACTOME_SCF_SKP2_MEDIATED_DEGRADATION_OF_P27_P21
6:112009427	*FYN*	REACTOME_SEMA3A_PAK_DEPENDENT_AXON_REPULSION
6:112056759	*FYN*	REACTOME_SEMA3A_PAK_DEPENDENT_AXON_REPULSION
6:112151452	*FYN*	REACTOME_SEMA3A_PAK_DEPENDENT_AXON_REPULSION
6:112009427	*FYN*	REACTOME_SEMA3A_PLEXIN_REPULSION_SIGNALING_BY_INHIBITING_INTEGRIN_ADHESION
6:112056759	*FYN*	REACTOME_SEMA3A_PLEXIN_REPULSION_SIGNALING_BY_INHIBITING_INTEGRIN_ADHESION
6:112151452	*FYN*	REACTOME_SEMA3A_PLEXIN_REPULSION_SIGNALING_BY_INHIBITING_INTEGRIN_ADHESION
3:49406095	*IP6K2*	REACTOME_SEMA4D_INDUCED_CELL_MIGRATION_AND_GROWTH_CONE_COLLAPSE
3:49424231	*IP6K2*	REACTOME_SEMA4D_INDUCED_CELL_MIGRATION_AND_GROWTH_CONE_COLLAPSE
3:49450137	*IP6K2*	REACTOME_SEMA4D_INDUCED_CELL_MIGRATION_AND_GROWTH_CONE_COLLAPSE
3:49406095	*IP6K2*	REACTOME_SEMA4D_IN_SEMAPHORIN_SIGNALING
3:49424231	*IP6K2*	REACTOME_SEMA4D_IN_SEMAPHORIN_SIGNALING
3:49450137	*IP6K2*	REACTOME_SEMA4D_IN_SEMAPHORIN_SIGNALING
3:49406095	*IP6K2*	REACTOME_SEMA4D_MEDIATED_INHIBITION_OF_CELL_ATTACHMENT_AND_MIGRATION
3:49424231	*IP6K2*	REACTOME_SEMA4D_MEDIATED_INHIBITION_OF_CELL_ATTACHMENT_AND_MIGRATION
3:49450137	*IP6K2*	REACTOME_SEMA4D_MEDIATED_INHIBITION_OF_CELL_ATTACHMENT_AND_MIGRATION
3:49406095	*IP6K2*	REACTOME_SEMAPHORIN_INTERACTIONS
3:49424231	*IP6K2*	REACTOME_SEMAPHORIN_INTERACTIONS
3:49450137	*IP6K2*	REACTOME_SEMAPHORIN_INTERACTIONS
6:112009427	*FYN*	REACTOME_SEMAPHORIN_INTERACTIONS
6:112056759	*FYN*	REACTOME_SEMAPHORIN_INTERACTIONS
6:112151452	*FYN*	REACTOME_SEMAPHORIN_INTERACTIONS
6:31862876	*HLA-DRB5*	REACTOME_SENESCENCE_ASSOCIATED_SECRETORY_PHENOTYPE_SASP
17:45215865	*WNT3*	REACTOME_SENESCENCE_ASSOCIATED_SECRETORY_PHENOTYPE_SASP

17:45244822	*WNT3*	REACTOME_SENESCENCE_ASSOCIATED_SECRETORY_PHENOTYPE_SASP
3:151090424	*MED12L*	REACTOME_SIGNAL_AMPLIFICATION
3:151099733	*MED12L*	REACTOME_SIGNAL_AMPLIFICATION
19:3156505	*SPPL2B*	REACTOME_SIGNAL_AMPLIFICATION
19:2251817	*SPPL2B*	REACTOME_SIGNALING_BY_BMP
1:156059276	*GBAP1*	REACTOME_SIGNALING_BY_BRAF_AND_RAF_FUSIONS
4:114424939	*CAMK2D*	REACTOME_SIGNALING_BY_BRAF_AND_RAF_FUSIONS
17:40392358	*RETREG3*	REACTOME_SIGNALING_BY_CYTOSOLIC_FGFR1_FUSION_MUTANTS
9:17579690	*SH3GL2_2*	REACTOME_SIGNALING_BY_EGFR
9:17609302	*SH3GL2_2*	REACTOME_SIGNALING_BY_EGFR
9:17627444	*SH3GL2_2*	REACTOME_SIGNALING_BY_EGFR
9:17710876	*SH3GL2_2*	REACTOME_SIGNALING_BY_EGFR
9:17732590	*SH3GL2_2*	REACTOME_SIGNALING_BY_EGFR
9:17737514	*SH3GL2_2*	REACTOME_SIGNALING_BY_EGFR
9:17758945	*SH3GL2_2*	REACTOME_SIGNALING_BY_EGFR
3:49406095	*IP6K2*	REACTOME_SIGNALING_BY_ERBB2
3:49424231	*IP6K2*	REACTOME_SIGNALING_BY_ERBB2
3:49450137	*IP6K2*	REACTOME_SIGNALING_BY_ERBB2
6:112009427	*FYN*	REACTOME_SIGNALING_BY_ERBB2
6:112056759	*FYN*	REACTOME_SIGNALING_BY_ERBB2
6:112151452	*FYN*	REACTOME_SIGNALING_BY_ERBB2
1:227067656	*ITPKB*	REACTOME_SIGNALING_BY_ERBB4
17:7101771	*V1NB1*	REACTOME_SIGNALING_BY_ERBB4
17:40392358	*RETREG3*	REACTOME_SIGNALING_BY_ERYTHROPOIETIN
17:40392358	*RETREG3*	REACTOME_SIGNALING_BY_FGFR1_IN_DISEASE
17:40392358	*RETREG3*	REACTOME_SIGNALING_BY_FGFR_IN_DISEASE
1:155975202	*GBAP1*	REACTOME_SIGNALING_BY_GPCR
2:169999639	*STK39*	REACTOME_SIGNALING_BY_GPCR
2:170028024	*STK39*	REACTOME_SIGNALING_BY_GPCR
3:48835245	*IP6K2*	REACTOME_SIGNALING_BY_GPCR
3:49406095	*IP6K2*	REACTOME_SIGNALING_BY_GPCR
3:49424231	*IP6K2*	REACTOME_SIGNALING_BY_GPCR
3:49450137	*IP6K2*	REACTOME_SIGNALING_BY_GPCR
3:123057397	*KPNA1*	REACTOME_SIGNALING_BY_GPCR
3:151090424	*MED12L*	REACTOME_SIGNALING_BY_GPCR
3:151099733	*MED12L*	REACTOME_SIGNALING_BY_GPCR
3:151599393	*MED12L*	REACTOME_SIGNALING_BY_GPCR
4:114424939	*CAMK2D*	REACTOME_SIGNALING_BY_GPCR
5:59236972	*ELOVL7*	REACTOME_SIGNALING_BY_GPCR
5:59665659	*ELOVL7*	REACTOME_SIGNALING_BY_GPCR
5:59705316	*ELOVL7*	REACTOME_SIGNALING_BY_GPCR
5:59736085	*ELOVL7*	REACTOME_SIGNALING_BY_GPCR
6:29407378	*TRIM40*	REACTOME_SIGNALING_BY_GPCR
6:29412222	*TRIM40*	REACTOME_SIGNALING_BY_GPCR
6:29579544	*TRIM40*	REACTOME_SIGNALING_BY_GPCR
10:15199347	*ITGA8*	REACTOME_SIGNALING_BY_GPCR
10:121114292	*INPP5F*	REACTOME_SIGNALING_BY_GPCR
10:121138071	*INPP5F*	REACTOME_SIGNALING_BY_GPCR
10:121191186	*INPP5F*	REACTOME_SIGNALING_BY_GPCR
10:121295884	*INPP5F*	REACTOME_SIGNALING_BY_GPCR
14:88472612	*GALC*	REACTOME_SIGNALING_BY_GPCR
17:43132290	*CRHR1_2*	REACTOME_SIGNALING_BY_GPCR
17:43744203	*CRHR1_2*	REACTOME_SIGNALING_BY_GPCR

17:43748712	*CRHR1_2*	REACTOME_SIGNALING_BY_GPCR
17:43767804	*CRHR1_2*	REACTOME_SIGNALING_BY_GPCR
17:43784228	*CRHR1_2*	REACTOME_SIGNALING_BY_GPCR
17:43791502	*CRHR1_2*	REACTOME_SIGNALING_BY_GPCR
17:43843861	*CRHR1_2*	REACTOME_SIGNALING_BY_GPCR
17:43847503	*CRHR1_2*	REACTOME_SIGNALING_BY_GPCR
17:44843651	*WNT3*	REACTOME_SIGNALING_BY_GPCR
17:44888209	*WNT3*	REACTOME_SIGNALING_BY_GPCR
17:44888715	*WNT3*	REACTOME_SIGNALING_BY_GPCR
17:44906949	*WNT3*	REACTOME_SIGNALING_BY_GPCR
17:44912901	*WNT3*	REACTOME_SIGNALING_BY_GPCR
17:44931399	*WNT3*	REACTOME_SIGNALING_BY_GPCR
17:44947756	*WNT3*	REACTOME_SIGNALING_BY_GPCR
19:3156505	*SPPL2B*	REACTOME_SIGNALING_BY_GPCR
3:48835245	*IP6K2*	REACTOME_SIGNALING_BY_HEDGEHOG
3:123057397	*KPNA1*	REACTOME_SIGNALING_BY_HEDGEHOG
6:32814942	*HLA-DRB5*	REACTOME_SIGNALING_BY_HEDGEHOG
10:103185542	*GBF1*	REACTOME_SIGNALING_BY_HEDGEHOG
10:103308244	*GBF1*	REACTOME_SIGNALING_BY_HEDGEHOG
10:104285824	*GBF1*	REACTOME_SIGNALING_BY_HEDGEHOG
17:7129974	*V1NB1*	REACTOME_SIGNALING_BY_HIPPO
12:124219791	*HIP1R*	REACTOME_SIGNALING_BY_INSULIN_RECEPTOR
2:102609356	*MAP4K4*	REACTOME_SIGNALING_BY_INTERLEUKINS
2:102636759	*MAP4K4*	REACTOME_SIGNALING_BY_INTERLEUKINS
2:103063855	*MAP4K4*	REACTOME_SIGNALING_BY_INTERLEUKINS
4:114424939	*CAMK2D*	REACTOME_SIGNALING_BY_INTERLEUKINS
6:32151443	*HLA-DRB5*	REACTOME_SIGNALING_BY_INTERLEUKINS
6:32814942	*HLA-DRB5*	REACTOME_SIGNALING_BY_INTERLEUKINS
6:112009427	*FYN*	REACTOME_SIGNALING_BY_INTERLEUKINS
6:112056759	*FYN*	REACTOME_SIGNALING_BY_INTERLEUKINS
6:112151452	*FYN*	REACTOME_SIGNALING_BY_INTERLEUKINS
8:21665306	*BIN3*	REACTOME_SIGNALING_BY_INTERLEUKINS
8:21668901	*BIN3*	REACTOME_SIGNALING_BY_INTERLEUKINS
10:103185542	*GBF1*	REACTOME_SIGNALING_BY_INTERLEUKINS
10:103308244	*GBF1*	REACTOME_SIGNALING_BY_INTERLEUKINS
11:83188607	*DLG2*	REACTOME_SIGNALING_BY_INTERLEUKINS
11:83219006	*DLG2*	REACTOME_SIGNALING_BY_INTERLEUKINS
11:83538733	*DLG2*	REACTOME_SIGNALING_BY_INTERLEUKINS
11:83544472	*DLG2*	REACTOME_SIGNALING_BY_INTERLEUKINS
11:83656201	*DLG2*	REACTOME_SIGNALING_BY_INTERLEUKINS
11:83700228	*DLG2*	REACTOME_SIGNALING_BY_INTERLEUKINS
11:83762918	*DLG2*	REACTOME_SIGNALING_BY_INTERLEUKINS
11:83894247	*DLG2*	REACTOME_SIGNALING_BY_INTERLEUKINS
11:84021037	*DLG2*	REACTOME_SIGNALING_BY_INTERLEUKINS
11:84324361	*DLG2*	REACTOME_SIGNALING_BY_INTERLEUKINS
11:84373496	*DLG2*	REACTOME_SIGNALING_BY_INTERLEUKINS
11:84403409	*DLG2*	REACTOME_SIGNALING_BY_INTERLEUKINS
15:61293467	*VPS13C*	REACTOME_SIGNALING_BY_INTERLEUKINS
16:31370452	*SETD1A*	REACTOME_SIGNALING_BY_INTERLEUKINS
16:50730088	*CASC16*	REACTOME_SIGNALING_BY_INTERLEUKINS
16:50730446	*CASC16*	REACTOME_SIGNALING_BY_INTERLEUKINS
16:50745926	*CASC16*	REACTOME_SIGNALING_BY_INTERLEUKINS
17:7101771	*V1NB1*	REACTOME_SIGNALING_BY_INTERLEUKINS

17:7578671	*V1NB1*	REACTOME_SIGNALING_BY_INTERLEUKINS
17:40392358	*RETREG3*	REACTOME_SIGNALING_BY_INTERLEUKINS
17:40392358	*RETREG3*	REACTOME_SIGNALING_BY_LEPTIN
3:49162284	*IP6K2*	REACTOME_SIGNALING_BY_MET
3:49162583	*IP6K2*	REACTOME_SIGNALING_BY_MET
6:33153528	*HLA-DRB5*	REACTOME_SIGNALING_BY_MET
6:112561154	*FYN*	REACTOME_SIGNALING_BY_MET
9:17579690	*SH3GL2_2*	REACTOME_SIGNALING_BY_MET
9:17609302	*SH3GL2_2*	REACTOME_SIGNALING_BY_MET
9:17627444	*SH3GL2_2*	REACTOME_SIGNALING_BY_MET
9:17710876	*SH3GL2_2*	REACTOME_SIGNALING_BY_MET
9:17732590	*SH3GL2_2*	REACTOME_SIGNALING_BY_MET
9:17737514	*SH3GL2_2*	REACTOME_SIGNALING_BY_MET
9:17758945	*SH3GL2_2*	REACTOME_SIGNALING_BY_MET
4:114424939	*CAMK2D*	REACTOME_SIGNALING_BY_MODERATE_KINASE_ACTIVITY_BRAF_MUTANTS
3:49406095	*IP6K2*	REACTOME_SIGNALING_BY_NON_RECEPTOR_TYROSINE_KINASES
3:49424231	*IP6K2*	REACTOME_SIGNALING_BY_NON_RECEPTOR_TYROSINE_KINASES
3:49450137	*IP6K2*	REACTOME_SIGNALING_BY_NON_RECEPTOR_TYROSINE_KINASES
7:23277372	*GPNMB*	REACTOME_SIGNALING_BY_NON_RECEPTOR_TYROSINE_KINASES
7:23293746	*GPNMB*	REACTOME_SIGNALING_BY_NON_RECEPTOR_TYROSINE_KINASES
7:23313171	*GPNMB*	REACTOME_SIGNALING_BY_NON_RECEPTOR_TYROSINE_KINASES
12:40614434	*LRRK2_2*	REACTOME_SIGNALING_BY_NON_RECEPTOR_TYROSINE_KINASES
12:40614656	*LRRK2_2*	REACTOME_SIGNALING_BY_NON_RECEPTOR_TYROSINE_KINASES
12:40617231	*LRRK2_2*	REACTOME_SIGNALING_BY_NON_RECEPTOR_TYROSINE_KINASES
12:40653993	*LRRK2_2*	REACTOME_SIGNALING_BY_NON_RECEPTOR_TYROSINE_KINASES
12:40698422	*LRRK2_2*	REACTOME_SIGNALING_BY_NON_RECEPTOR_TYROSINE_KINASES
12:40707861	*LRRK2_2*	REACTOME_SIGNALING_BY_NON_RECEPTOR_TYROSINE_KINASES
12:40753796	*LRRK2_2*	REACTOME_SIGNALING_BY_NON_RECEPTOR_TYROSINE_KINASES
1:227067656	*ITPKB*	REACTOME_SIGNALING_BY_NOTCH1_IN_CANCER
1:227067656	*ITPKB*	REACTOME_SIGNALING_BY_NOTCH1
12:41147295	*LRRK2_2*	REACTOME_SIGNALING_BY_NOTCH1
12:41251554	*LRRK2_2*	REACTOME_SIGNALING_BY_NOTCH1
12:41407403	*LRRK2_2*	REACTOME_SIGNALING_BY_NOTCH1
19:3022354	*SPPL2B*	REACTOME_SIGNALING_BY_NOTCH1
19:3036350	*SPPL2B*	REACTOME_SIGNALING_BY_NOTCH1
1:227067656	*ITPKB*	REACTOME_SIGNALING_BY_NOTCH2
12:41147295	*LRRK2_2*	REACTOME_SIGNALING_BY_NOTCH2
12:41251554	*LRRK2_2*	REACTOME_SIGNALING_BY_NOTCH2
12:41407403	*LRRK2_2*	REACTOME_SIGNALING_BY_NOTCH2
1:227067656	*ITPKB*	REACTOME_SIGNALING_BY_NOTCH3
1:227067656	*ITPKB*	REACTOME_SIGNALING_BY_NOTCH4
6:32186193	*HLA-DRB5*	REACTOME_SIGNALING_BY_NOTCH4
6:32814942	*HLA-DRB5*	REACTOME_SIGNALING_BY_NOTCH4
1:227067656	*ITPKB*	REACTOME_SIGNALING_BY_NOTCH
6:32186193	*HLA-DRB5*	REACTOME_SIGNALING_BY_NOTCH
6:32814942	*HLA-DRB5*	REACTOME_SIGNALING_BY_NOTCH
12:41147295	*LRRK2_2*	REACTOME_SIGNALING_BY_NOTCH
12:41251554	*LRRK2_2*	REACTOME_SIGNALING_BY_NOTCH
12:41407403	*LRRK2_2*	REACTOME_SIGNALING_BY_NOTCH
17:7578671	*V1NB1*	REACTOME_SIGNALING_BY_NOTCH
19:3022354	*SPPL2B*	REACTOME_SIGNALING_BY_NOTCH
19:3036350	*SPPL2B*	REACTOME_SIGNALING_BY_NOTCH
1:171945488	*VAMP4*	REACTOME_SIGNALING_BY_NTRK1_TRKA

1:172137119	*VAMP4*	REACTOME_SIGNALING_BY_NTRK1_TRKA
1:172386261	*VAMP4*	REACTOME_SIGNALING_BY_NTRK1_TRKA
3:49406095	*IP6K2*	REACTOME_SIGNALING_BY_NTRK1_TRKA
3:49424231	*IP6K2*	REACTOME_SIGNALING_BY_NTRK1_TRKA
3:49450137	*IP6K2*	REACTOME_SIGNALING_BY_NTRK1_TRKA
9:17579690	*SH3GL2_2*	REACTOME_SIGNALING_BY_NTRK1_TRKA
9:17609302	*SH3GL2_2*	REACTOME_SIGNALING_BY_NTRK1_TRKA
9:17627444	*SH3GL2_2*	REACTOME_SIGNALING_BY_NTRK1_TRKA
9:17710876	*SH3GL2_2*	REACTOME_SIGNALING_BY_NTRK1_TRKA
9:17732590	*SH3GL2_2*	REACTOME_SIGNALING_BY_NTRK1_TRKA
9:17737514	*SH3GL2_2*	REACTOME_SIGNALING_BY_NTRK1_TRKA
9:17758945	*SH3GL2_2*	REACTOME_SIGNALING_BY_NTRK1_TRKA
18:40543738	*RIT2*	REACTOME_SIGNALING_BY_NTRK1_TRKA
18:40596271	*RIT2*	REACTOME_SIGNALING_BY_NTRK1_TRKA
18:40612823	*RIT2*	REACTOME_SIGNALING_BY_NTRK1_TRKA
18:40649778	*RIT2*	REACTOME_SIGNALING_BY_NTRK1_TRKA
18:40673380	*RIT2*	REACTOME_SIGNALING_BY_NTRK1_TRKA
6:112009427	*FYN*	REACTOME_SIGNALING_BY_NTRK2_TRKB
6:112056759	*FYN*	REACTOME_SIGNALING_BY_NTRK2_TRKB
6:112151452	*FYN*	REACTOME_SIGNALING_BY_NTRK2_TRKB
1:171945488	*VAMP4*	REACTOME_SIGNALING_BY_NTRKS
1:172137119	*VAMP4*	REACTOME_SIGNALING_BY_NTRKS
1:172386261	*VAMP4*	REACTOME_SIGNALING_BY_NTRKS
3:49406095	*IP6K2*	REACTOME_SIGNALING_BY_NTRKS
3:49424231	*IP6K2*	REACTOME_SIGNALING_BY_NTRKS
3:49450137	*IP6K2*	REACTOME_SIGNALING_BY_NTRKS
6:112009427	*FYN*	REACTOME_SIGNALING_BY_NTRKS
6:112056759	*FYN*	REACTOME_SIGNALING_BY_NTRKS
6:112151452	*FYN*	REACTOME_SIGNALING_BY_NTRKS
9:17579690	*SH3GL2_2*	REACTOME_SIGNALING_BY_NTRKS
9:17609302	*SH3GL2_2*	REACTOME_SIGNALING_BY_NTRKS
9:17627444	*SH3GL2_2*	REACTOME_SIGNALING_BY_NTRKS
9:17710876	*SH3GL2_2*	REACTOME_SIGNALING_BY_NTRKS
9:17732590	*SH3GL2_2*	REACTOME_SIGNALING_BY_NTRKS
9:17737514	*SH3GL2_2*	REACTOME_SIGNALING_BY_NTRKS
9:17758945	*SH3GL2_2*	REACTOME_SIGNALING_BY_NTRKS
18:40543738	*RIT2*	REACTOME_SIGNALING_BY_NTRKS
18:40596271	*RIT2*	REACTOME_SIGNALING_BY_NTRKS
18:40612823	*RIT2*	REACTOME_SIGNALING_BY_NTRKS
18:40649778	*RIT2*	REACTOME_SIGNALING_BY_NTRKS
18:40673380	*RIT2*	REACTOME_SIGNALING_BY_NTRKS
14:38061742	*MIPOL1*	REACTOME_SIGNALING_BY_NUCLEAR_RECEPTORS
17:40392358	*RETREG3*	REACTOME_SIGNALING_BY_PDGF
4:114424939	*CAMK2D*	REACTOME_SIGNALING_BY_RAS_MUTANTS
1:171945488	*VAMP4*	REACTOME_SIGNALING_BY_RECEPTOR_TYROSINE_KINASES
1:172137119	*VAMP4*	REACTOME_SIGNALING_BY_RECEPTOR_TYROSINE_KINASES
1:172386261	*VAMP4*	REACTOME_SIGNALING_BY_RECEPTOR_TYROSINE_KINASES
1:227067656	*ITPKB*	REACTOME_SIGNALING_BY_RECEPTOR_TYROSINE_KINASES
3:49162284	*IP6K2*	REACTOME_SIGNALING_BY_RECEPTOR_TYROSINE_KINASES
3:49162583	*IP6K2*	REACTOME_SIGNALING_BY_RECEPTOR_TYROSINE_KINASES
3:49406095	*IP6K2*	REACTOME_SIGNALING_BY_RECEPTOR_TYROSINE_KINASES
3:49424231	*IP6K2*	REACTOME_SIGNALING_BY_RECEPTOR_TYROSINE_KINASES
3:49450137	*IP6K2*	REACTOME_SIGNALING_BY_RECEPTOR_TYROSINE_KINASES

6:33153528	*HLA-DRB5*	REACTOME_SIGNALING_BY_RECEPTOR_TYROSINE_KINASES
6:112009427	*FYN*	REACTOME_SIGNALING_BY_RECEPTOR_TYROSINE_KINASES
6:112056759	*FYN*	REACTOME_SIGNALING_BY_RECEPTOR_TYROSINE_KINASES
6:112151452	*FYN*	REACTOME_SIGNALING_BY_RECEPTOR_TYROSINE_KINASES
6:112561154	*FYN*	REACTOME_SIGNALING_BY_RECEPTOR_TYROSINE_KINASES
9:17579690	*SH3GL2_2*	REACTOME_SIGNALING_BY_RECEPTOR_TYROSINE_KINASES
9:17609302	*SH3GL2_2*	REACTOME_SIGNALING_BY_RECEPTOR_TYROSINE_KINASES
9:17627444	*SH3GL2_2*	REACTOME_SIGNALING_BY_RECEPTOR_TYROSINE_KINASES
9:17710876	*SH3GL2_2*	REACTOME_SIGNALING_BY_RECEPTOR_TYROSINE_KINASES
9:17732590	*SH3GL2_2*	REACTOME_SIGNALING_BY_RECEPTOR_TYROSINE_KINASES
9:17737514	*SH3GL2_2*	REACTOME_SIGNALING_BY_RECEPTOR_TYROSINE_KINASES
9:17758945	*SH3GL2_2*	REACTOME_SIGNALING_BY_RECEPTOR_TYROSINE_KINASES
12:124219791	*HIP1R*	REACTOME_SIGNALING_BY_RECEPTOR_TYROSINE_KINASES
17:7101771	*V1NB1*	REACTOME_SIGNALING_BY_RECEPTOR_TYROSINE_KINASES
17:39883520	*RETREG3*	REACTOME_SIGNALING_BY_RECEPTOR_TYROSINE_KINASES
17:40392358	*RETREG3*	REACTOME_SIGNALING_BY_RECEPTOR_TYROSINE_KINASES
18:40543738	*RIT2*	REACTOME_SIGNALING_BY_RECEPTOR_TYROSINE_KINASES
18:40596271	*RIT2*	REACTOME_SIGNALING_BY_RECEPTOR_TYROSINE_KINASES
18:40612823	*RIT2*	REACTOME_SIGNALING_BY_RECEPTOR_TYROSINE_KINASES
18:40649778	*RIT2*	REACTOME_SIGNALING_BY_RECEPTOR_TYROSINE_KINASES
18:40673380	*RIT2*	REACTOME_SIGNALING_BY_RECEPTOR_TYROSINE_KINASES
1:155975202	*GBAP1*	REACTOME_SIGNALING_BY_RHO_GTPASES
1:161035780	*FCGR2A*	REACTOME_SIGNALING_BY_RHO_GTPASES
3:49406095	*IP6K2*	REACTOME_SIGNALING_BY_RHO_GTPASES
3:49424231	*IP6K2*	REACTOME_SIGNALING_BY_RHO_GTPASES
3:49450137	*IP6K2*	REACTOME_SIGNALING_BY_RHO_GTPASES
4:89711319	*SNCA_2*	REACTOME_SIGNALING_BY_RHO_GTPASES
4:89740128	*SNCA_2*	REACTOME_SIGNALING_BY_RHO_GTPASES
4:90002389	*SNCA_2*	REACTOME_SIGNALING_BY_RHO_GTPASES
5:59958372	*ELOVL7*	REACTOME_SIGNALING_BY_RHO_GTPASES
12:122816734	*HIP1R*	REACTOME_SIGNALING_BY_RHO_GTPASES
12:122817580	*HIP1R*	REACTOME_SIGNALING_BY_RHO_GTPASES
12:122853197	*HIP1R*	REACTOME_SIGNALING_BY_RHO_GTPASES
12:123057792	*HIP1R*	REACTOME_SIGNALING_BY_RHO_GTPASES
12:123110365	*HIP1R*	REACTOME_SIGNALING_BY_RHO_GTPASES
12:123110785	*HIP1R*	REACTOME_SIGNALING_BY_RHO_GTPASES
17:7101771	*V1NB1*	REACTOME_SIGNALING_BY_RHO_GTPASES
17:7129974	*V1NB1*	REACTOME_SIGNALING_BY_RHO_GTPASES
17:43302259	*CRHR1_2*	REACTOME_SIGNALING_BY_RHO_GTPASES
17:43472507	*CRHR1_2*	REACTOME_SIGNALING_BY_RHO_GTPASES
3:48835245	*IP6K2*	REACTOME_SIGNALING_BY_ROBO_RECEPTORS
3:49406095	*IP6K2*	REACTOME_SIGNALING_BY_ROBO_RECEPTORS
3:49424231	*IP6K2*	REACTOME_SIGNALING_BY_ROBO_RECEPTORS
3:49450137	*IP6K2*	REACTOME_SIGNALING_BY_ROBO_RECEPTORS
3:49561208	*IP6K2*	REACTOME_SIGNALING_BY_ROBO_RECEPTORS
6:32814942	*HLA-DRB5*	REACTOME_SIGNALING_BY_ROBO_RECEPTORS
6:112009427	*FYN*	REACTOME_SIGNALING_BY_SCF_KIT
6:112056759	*FYN*	REACTOME_SIGNALING_BY_SCF_KIT
6:112151452	*FYN*	REACTOME_SIGNALING_BY_SCF_KIT
17:40392358	*RETREG3*	REACTOME_SIGNALING_BY_SCF_KIT
2:135708115	*TMEM163*	REACTOME_SIGNALING_BY_TGF_BETA_FAMILY_MEMBERS
3:49406095	*IP6K2*	REACTOME_SIGNALING_BY_TGF_BETA_FAMILY_MEMBERS
3:49424231	*IP6K2*	REACTOME_SIGNALING_BY_TGF_BETA_FAMILY_MEMBERS

3:49450137	*IP6K2*	REACTOME_SIGNALING_BY_TGF_BETA_FAMILY_MEMBERS
19:2251817	*SPPL2B*	REACTOME_SIGNALING_BY_TGF_BETA_FAMILY_MEMBERS
2:135708115	*TMEM163*	REACTOME_SIGNALING_BY_TGF_BETA_RECEPTOR_COMPLEX
3:49406095	*IP6K2*	REACTOME_SIGNALING_BY_TGF_BETA_RECEPTOR_COMPLEX
3:49424231	*IP6K2*	REACTOME_SIGNALING_BY_TGF_BETA_RECEPTOR_COMPLEX
3:49450137	*IP6K2*	REACTOME_SIGNALING_BY_TGF_BETA_RECEPTOR_COMPLEX
6:32814942	*HLA-DRB5*	REACTOME_SIGNALING_BY_THE_B_CELL_RECEPTOR_BCR
6:112009427	*FYN*	REACTOME_SIGNALING_BY_THE_B_CELL_RECEPTOR_BCR
6:112056759	*FYN*	REACTOME_SIGNALING_BY_THE_B_CELL_RECEPTOR_BCR
6:112151452	*FYN*	REACTOME_SIGNALING_BY_THE_B_CELL_RECEPTOR_BCR
8:11381148	*CTSB*	REACTOME_SIGNALING_BY_THE_B_CELL_RECEPTOR_BCR
10:103185542	*GBF1*	REACTOME_SIGNALING_BY_THE_B_CELL_RECEPTOR_BCR
10:103308244	*GBF1*	REACTOME_SIGNALING_BY_THE_B_CELL_RECEPTOR_BCR
3:49406095	*IP6K2*	REACTOME_SIGNALING_BY_VEGF
3:49424231	*IP6K2*	REACTOME_SIGNALING_BY_VEGF
3:49450137	*IP6K2*	REACTOME_SIGNALING_BY_VEGF
6:112009427	*FYN*	REACTOME_SIGNALING_BY_VEGF
6:112056759	*FYN*	REACTOME_SIGNALING_BY_VEGF
6:112151452	*FYN*	REACTOME_SIGNALING_BY_VEGF
17:39883520	*RETREG3*	REACTOME_SIGNALING_BY_VEGF
3:49406095	*IP6K2*	REACTOME_SIGNALING_BY_WNT
3:49424231	*IP6K2*	REACTOME_SIGNALING_BY_WNT
3:49450137	*IP6K2*	REACTOME_SIGNALING_BY_WNT
6:32814942	*HLA-DRB5*	REACTOME_SIGNALING_BY_WNT
10:103185542	*GBF1*	REACTOME_SIGNALING_BY_WNT
10:103308244	*GBF1*	REACTOME_SIGNALING_BY_WNT
17:7129974	*V1NB1*	REACTOME_SIGNALING_BY_WNT
17:44843651	*WNT3*	REACTOME_SIGNALING_BY_WNT
17:44888209	*WNT3*	REACTOME_SIGNALING_BY_WNT
17:44888715	*WNT3*	REACTOME_SIGNALING_BY_WNT
17:44906949	*WNT3*	REACTOME_SIGNALING_BY_WNT
17:44912901	*WNT3*	REACTOME_SIGNALING_BY_WNT
17:44931399	*WNT3*	REACTOME_SIGNALING_BY_WNT
17:44947756	*WNT3*	REACTOME_SIGNALING_BY_WNT
19:3022354	*SPPL2B*	REACTOME_SIGNALING_BY_WNT
19:3036350	*SPPL2B*	REACTOME_SIGNALING_BY_WNT
18:40543738	*RIT2*	REACTOME_SIGNALLING_TO_ERKS
18:40596271	*RIT2*	REACTOME_SIGNALLING_TO_ERKS
18:40612823	*RIT2*	REACTOME_SIGNALLING_TO_ERKS
18:40649778	*RIT2*	REACTOME_SIGNALLING_TO_ERKS
18:40673380	*RIT2*	REACTOME_SIGNALLING_TO_ERKS
18:40543738	*RIT2*	REACTOME_SIGNALLING_TO_P38_VIA_RIT_AND_RIN
18:40596271	*RIT2*	REACTOME_SIGNALLING_TO_P38_VIA_RIT_AND_RIN
18:40612823	*RIT2*	REACTOME_SIGNALLING_TO_P38_VIA_RIT_AND_RIN
18:40649778	*RIT2*	REACTOME_SIGNALLING_TO_P38_VIA_RIT_AND_RIN
18:40673380	*RIT2*	REACTOME_SIGNALLING_TO_P38_VIA_RIT_AND_RIN
1:205645310	*RAB29*	REACTOME_SLC_MEDIATED_TRANSMEMBRANE_TRANSPORT
1:205884274	*RAB29*	REACTOME_SLC_MEDIATED_TRANSMEMBRANE_TRANSPORT
6:31846234	*HLA-DRB5*	REACTOME_SLC_MEDIATED_TRANSMEMBRANE_TRANSPORT
8:17427186	*FGF20*	REACTOME_SLC_MEDIATED_TRANSMEMBRANE_TRANSPORT
12:40240262	*LRRK2_2*	REACTOME_SLC_MEDIATED_TRANSMEMBRANE_TRANSPORT
12:40252360	*LRRK2_2*	REACTOME_SLC_MEDIATED_TRANSMEMBRANE_TRANSPORT
12:40267539	*LRRK2_2*	REACTOME_SLC_MEDIATED_TRANSMEMBRANE_TRANSPORT

12:40302599	*LRRK2_2*	REACTOME_SLC_MEDIATED_TRANSMEMBRANE_TRANSPORT
12:40348056	*LRRK2_2*	REACTOME_SLC_MEDIATED_TRANSMEMBRANE_TRANSPORT
12:40349236	*LRRK2_2*	REACTOME_SLC_MEDIATED_TRANSMEMBRANE_TRANSPORT
12:40378773	*LRRK2_2*	REACTOME_SLC_MEDIATED_TRANSMEMBRANE_TRANSPORT
12:40393852	*LRRK2_2*	REACTOME_SLC_MEDIATED_TRANSMEMBRANE_TRANSPORT
12:40405478	*LRRK2_2*	REACTOME_SLC_MEDIATED_TRANSMEMBRANE_TRANSPORT
12:40413305	*LRRK2_2*	REACTOME_SLC_MEDIATED_TRANSMEMBRANE_TRANSPORT
12:40414626	*LRRK2_2*	REACTOME_SLC_MEDIATED_TRANSMEMBRANE_TRANSPORT
12:40437969	*LRRK2_2*	REACTOME_SLC_MEDIATED_TRANSMEMBRANE_TRANSPORT
12:47192323	*SCAF11*	REACTOME_SLC_MEDIATED_TRANSMEMBRANE_TRANSPORT
17:42338993	*FAM171A2*	REACTOME_SLC_MEDIATED_TRANSMEMBRANE_TRANSPORT
17:42338993	*FAM171A2*	REACTOME_SLC_TRANSPORTER_DISORDERS
2:135708115	*TMEM163*	REACTOME_SMAD2_SMAD3:SMAD4_HETEROTRIMER_REGULATES_TRANSCRIPTION
1:231698854	*SIPA1L2*	REACTOME_SMALL_INTERFERING_RNA_SIRNA_BIOGENESIS
5:134315853	*C5orf24*	REACTOME_SPERM_MOTILITY_AND_TAXES
3:48199695	*IP6K2*	REACTOME_S_PHASE
3:48214438	*IP6K2*	REACTOME_S_PHASE
6:32814942	*HLA-DRB5*	REACTOME_S_PHASE
12:133206648	*FBRSL1*	REACTOME_S_PHASE
12:133223685	*FBRSL1*	REACTOME_S_PHASE
12:133225045	*FBRSL1*	REACTOME_S_PHASE
17:45215865	*WNT3*	REACTOME_S_PHASE
17:45244822	*WNT3*	REACTOME_S_PHASE
20:5934513	*CRLS1*	REACTOME_S_PHASE
20:5968737	*CRLS1*	REACTOME_S_PHASE
20:5968964	*CRLS1*	REACTOME_S_PHASE
2:169389091	*STK39*	REACTOME_SPHINGOLIPID_DE_NOVO_BIOSYNTHESIS
3:160668220	*SPTSSB*	REACTOME_SPHINGOLIPID_DE_NOVO_BIOSYNTHESIS
3:161068566	*SPTSSB*	REACTOME_SPHINGOLIPID_DE_NOVO_BIOSYNTHESIS
3:161075189	*SPTSSB*	REACTOME_SPHINGOLIPID_DE_NOVO_BIOSYNTHESIS
2:169389091	*STK39*	REACTOME_SPHINGOLIPID_METABOLISM
3:160668220	*SPTSSB*	REACTOME_SPHINGOLIPID_METABOLISM
3:160817877	*SPTSSB*	REACTOME_SPHINGOLIPID_METABOLISM
3:161068566	*SPTSSB*	REACTOME_SPHINGOLIPID_METABOLISM
3:161075189	*SPTSSB*	REACTOME_SPHINGOLIPID_METABOLISM
14:88393900	*GALC*	REACTOME_SPHINGOLIPID_METABOLISM
14:88443553	*GALC*	REACTOME_SPHINGOLIPID_METABOLISM
6:32814942	*HLA-DRB5*	REACTOME_STABILIZATION_OF_P53
17:7578671	*V1NB1*	REACTOME_STABILIZATION_OF_P53
17:40392358	*RETREG3*	REACTOME_STAT5_ACTIVATION
4:170563153	*CLCN3*	REACTOME_STIMULI_SENSING_CHANNELS
4:170569172	*CLCN3*	REACTOME_STIMULI_SENSING_CHANNELS
4:170620812	*CLCN3*	REACTOME_STIMULI_SENSING_CHANNELS
12:45755099	*SCAF11*	REACTOME_STIMULI_SENSING_CHANNELS
17:40938261	*RETREG3*	REACTOME_STIMULI_SENSING_CHANNELS
15:61293467	*VPS13C*	REACTOME_SUMOYLATION_OF_INTRACELLULAR_RECEPTORS
17:7578671	*V1NB1*	REACTOME_SUMOYLATION_OF_TRANSCRIPTION_FACTORS
15:61293467	*VPS13C*	REACTOME_SUMOYLATION
17:7578671	*V1NB1*	REACTOME_SUMOYLATION
6:32814942	*HLA-DRB5*	REACTOME_SWITCHING_OF_ORIGINS_TO_A_POST_REPLICATIVE_STATE
17:45215865	*WNT3*	REACTOME_SWITCHING_OF_ORIGINS_TO_A_POST_REPLICATIVE_STATE
17:45244822	*WNT3*	REACTOME_SWITCHING_OF_ORIGINS_TO_A_POST_REPLICATIVE_STATE
20:5934513	*CRLS1*	REACTOME_SWITCHING_OF_ORIGINS_TO_A_POST_REPLICATIVE_STATE

20:5968737	*CRLS1*	REACTOME_SWITCHING_OF_ORIGINS_TO_A_POST_REPLICATIVE_STATE
20:5968964	*CRLS1*	REACTOME_SWITCHING_OF_ORIGINS_TO_A_POST_REPLICATIVE_STATE
17:7101771	*V1NB1*	REACTOME_SYNAPTIC_ADHESION_LIKE_MOLECULES
17:7983799	*V1NB1*	REACTOME_SYNTHESIS_OF_12_EICOSATETRAENOIC_ACID_DERIVATIVES
17:8019125	*V1NB1*	REACTOME_SYNTHESIS_OF_12_EICOSATETRAENOIC_ACID_DERIVATIVES
5:133710339	*C5orf24*	REACTOME_SYNTHESIS_OF_ACTIVE_UBIQUITIN:_ROLES_OF_E1_AND_E2_ENZYMES
9:33845203	*UBAP2*	REACTOME_SYNTHESIS_OF_ACTIVE_UBIQUITIN:_ROLES_OF_E1_AND_E2_ENZYMES
16:30999462	*SETD1A*	REACTOME_SYNTHESIS_OF_BILE_ACIDS_AND_BILE_SALTS
16:30999462	*SETD1A*	REACTOME_SYNTHESIS_OF_BILE_ACIDS_AND_BILE_SALTS_VIA_24_HYDROXYCHOLESTEROL
16:30999462	*SETD1A*	REACTOME_SYNTHESIS_OF_BILE_ACIDS_AND_BILE_SALTS_VIA_27_HYDROXYCHOLESTEROL
16:30999462	*SETD1A*	REACTOME_SYNTHESIS_OF_BILE_ACIDS_AND_BILE_SALTS_VIA_7ALPHA_HYDROXYCHOLESTEROL
1:172386261	*VAMP4*	REACTOME_SYNTHESIS_OF_GLYCOSYLPHOSPHATIDYLINOSITOL_GPI
1:172388441	*VAMP4*	REACTOME_SYNTHESIS_OF_GLYCOSYLPHOSPHATIDYLINOSITOL_GPI
1:172389198	*VAMP4*	REACTOME_SYNTHESIS_OF_GLYCOSYLPHOSPHATIDYLINOSITOL_GPI
1:226910340	*ITPKB*	REACTOME_SYNTHESIS_OF_IP3_AND_IP4_IN_THE_CYTOSOL
1:226916078	*ITPKB*	REACTOME_SYNTHESIS_OF_IP3_AND_IP4_IN_THE_CYTOSOL
17:43195981	*CRHR1_2*	REACTOME_SYNTHESIS_OF_IP3_AND_IP4_IN_THE_CYTOSOL
2:96858914	*KCNIP3*	REACTOME_SYNTHESIS_OF_PC
6:31846234	*HLA-DRB5*	REACTOME_SYNTHESIS_OF_PC
10:121505277	*INPP5F*	REACTOME_SYNTHESIS_OF_PIPS_AT_THE_EARLY_ENDOSOME_MEMBRANE
10:121538407	*INPP5F*	REACTOME_SYNTHESIS_OF_PIPS_AT_THE_EARLY_ENDOSOME_MEMBRANE
12:123561638	*HIP1R*	REACTOME_SYNTHESIS_OF_PI
12:123585705	*HIP1R*	REACTOME_SYNTHESIS_OF_PI
5:60053220	*ELOVL7*	REACTOME_SYNTHESIS_OF_VERY_LONG_CHAIN_FATTY_ACYL_COAS
6:32151443	*HLA-DRB5*	REACTOME_TAK1_ACTIVATES_NFKB_BY_PHOSPHORYLATION_AND_ACTIVATION_OF_IKKS_COMPLEX
16:50730088	*CASC16*	REACTOME_TAK1_ACTIVATES_NFKB_BY_PHOSPHORYLATION_AND_ACTIVATION_OF_IKKS_COMPLEX
16:50730446	*CASC16*	REACTOME_TAK1_ACTIVATES_NFKB_BY_PHOSPHORYLATION_AND_ACTIVATION_OF_IKKS_COMPLEX
16:50745926	*CASC16*	REACTOME_TAK1_ACTIVATES_NFKB_BY_PHOSPHORYLATION_AND_ACTIVATION_OF_IKKS_COMPLEX
6:32814942	*HLA-DRB5*	REACTOME_TCF_DEPENDENT_SIGNALING_IN_RESPONSE_TO_WNT
10:103185542	*GBF1*	REACTOME_TCF_DEPENDENT_SIGNALING_IN_RESPONSE_TO_WNT
10:103308244	*GBF1*	REACTOME_TCF_DEPENDENT_SIGNALING_IN_RESPONSE_TO_WNT
17:7129974	*V1NB1*	REACTOME_TCF_DEPENDENT_SIGNALING_IN_RESPONSE_TO_WNT
17:44843651	*WNT3*	REACTOME_TCF_DEPENDENT_SIGNALING_IN_RESPONSE_TO_WNT
17:44888209	*WNT3*	REACTOME_TCF_DEPENDENT_SIGNALING_IN_RESPONSE_TO_WNT
17:44888715	*WNT3*	REACTOME_TCF_DEPENDENT_SIGNALING_IN_RESPONSE_TO_WNT
17:44906949	*WNT3*	REACTOME_TCF_DEPENDENT_SIGNALING_IN_RESPONSE_TO_WNT
19:3022354	*SPPL2B*	REACTOME_TCF_DEPENDENT_SIGNALING_IN_RESPONSE_TO_WNT
19:3036350	*SPPL2B*	REACTOME_TCF_DEPENDENT_SIGNALING_IN_RESPONSE_TO_WNT
6:32602372	*HLA-DRB5*	REACTOME_TCR_SIGNALING
6:32814942	*HLA-DRB5*	REACTOME_TCR_SIGNALING
10:103185542	*GBF1*	REACTOME_TCR_SIGNALING
10:103308244	*GBF1*	REACTOME_TCR_SIGNALING
12:133206648	*FBRSL1*	REACTOME_TELOMERE_C_STRAND_LAGGING_STRAND_SYNTHESIS
12:133223685	*FBRSL1*	REACTOME_TELOMERE_C_STRAND_LAGGING_STRAND_SYNTHESIS
12:133225045	*FBRSL1*	REACTOME_TELOMERE_C_STRAND_LAGGING_STRAND_SYNTHESIS
12:133206648	*FBRSL1*	REACTOME_TELOMERE_MAINTENANCE
12:133223685	*FBRSL1*	REACTOME_TELOMERE_MAINTENANCE
12:133225045	*FBRSL1*	REACTOME_TELOMERE_MAINTENANCE
12:133206648	*FBRSL1*	REACTOME_TERMINATION_OF_TRANSLESION_DNA_SYNTHESIS
12:133223685	*FBRSL1*	REACTOME_TERMINATION_OF_TRANSLESION_DNA_SYNTHESIS
12:133225045	*FBRSL1*	REACTOME_TERMINATION_OF_TRANSLESION_DNA_SYNTHESIS
14:55348869	*GCH1*	REACTOME_TETRAHYDROBIOPTERIN_BH4_SYNTHESIS_RECYCLING_SALVAGE_AND_REGULATION
3:49406095	*IP6K2*	REACTOME_TGF_BETA_RECEPTOR_SIGNALING_IN_EMT_EPITHELIAL_TO_MESENCHYMAL_TRANSITION

3:49424231	*IP6K2*	REACTOME_TGF_BETA_RECEPTOR_SIGNALING_IN_EMT_EPITHELIAL_TO_MESENCHYMAL_TRANSITION
3:49450137	*IP6K2*	REACTOME_TGF_BETA_RECEPTOR_SIGNALING_IN_EMT_EPITHELIAL_TO_MESENCHYMAL_TRANSITION
1:161171736	*FCGR2A*	REACTOME_THE_CITRIC_ACID_TCA_CYCLE_AND_RESPIRATORY_ELECTRON_TRANSPORT
5:60273923	*ELOVL7*	REACTOME_THE_CITRIC_ACID_TCA_CYCLE_AND_RESPIRATORY_ELECTRON_TRANSPORT
10:15199347	*ITGA8*	REACTOME_THE_PHOTOTRANSDUCTION_CASCADE
17:43132290	*CRHR1_2*	REACTOME_THE_PHOTOTRANSDUCTION_CASCADE
6:32814942	*HLA-DRB5*	REACTOME_THE_ROLE_OF_GTSE1_IN_G2_M_PROGRESSION_AFTER_G2_CHECKPOINT
17:7578671	*V1NB1*	REACTOME_THE_ROLE_OF_GTSE1_IN_G2_M_PROGRESSION_AFTER_G2_CHECKPOINT
6:112009427	*FYN*	REACTOME_THE_ROLE_OF_NEF_IN_HIV_1_REPLICATION_AND_DISEASE_PATHOGENESIS
6:112056759	*FYN*	REACTOME_THE_ROLE_OF_NEF_IN_HIV_1_REPLICATION_AND_DISEASE_PATHOGENESIS
6:112151452	*FYN*	REACTOME_THE_ROLE_OF_NEF_IN_HIV_1_REPLICATION_AND_DISEASE_PATHOGENESIS
19:3156505	*SPPL2B*	REACTOME_THROMBIN_SIGNALLING_THROUGH_PROTEINASE_ACTIVATED_RECEPTORS_PARS
19:3156505	*SPPL2B*	REACTOME_THROMBOXANE_SIGNALLING_THROUGH_TP_RECEPTOR
6:32814942	*HLA-DRB5*	REACTOME_TNFR2_NON_CANONICAL_NF_KB_PATHWAY
10:103185542	*GBF1*	REACTOME_TNFR2_NON_CANONICAL_NF_KB_PATHWAY
10:103308244	*GBF1*	REACTOME_TNFR2_NON_CANONICAL_NF_KB_PATHWAY
17:7455536	*V1NB1*	REACTOME_TNFR2_NON_CANONICAL_NF_KB_PATHWAY
17:43390763	*CRHR1_2*	REACTOME_TNFR2_NON_CANONICAL_NF_KB_PATHWAY
17:7455536	*V1NB1*	REACTOME_TNF_RECEPTOR_SUPERFAMILY_TNFSF_MEMBERS_MEDIATING_NON_CANONICAL_NF_KB_PATHWAY
17:43390763	*CRHR1_2*	REACTOME_TNF_RECEPTOR_SUPERFAMILY_TNFSF_MEMBERS_MEDIATING_NON_CANONICAL_NF_KB_PATHWAY
19:2335872	*SPPL2B*	REACTOME_TNF_SIGNALING
19:2346990	*SPPL2B*	REACTOME_TNF_SIGNALING
19:2352757	*SPPL2B*	REACTOME_TNF_SIGNALING
1:171945488	*VAMP4*	REACTOME_TOLL_LIKE_RECEPTOR_4_TLR4_CASCADE
1:172137119	*VAMP4*	REACTOME_TOLL_LIKE_RECEPTOR_4_TLR4_CASCADE
1:172386261	*VAMP4*	REACTOME_TOLL_LIKE_RECEPTOR_4_TLR4_CASCADE
6:32151443	*HLA-DRB5*	REACTOME_TOLL_LIKE_RECEPTOR_4_TLR4_CASCADE
10:103185542	*GBF1*	REACTOME_TOLL_LIKE_RECEPTOR_4_TLR4_CASCADE
10:103308244	*GBF1*	REACTOME_TOLL_LIKE_RECEPTOR_4_TLR4_CASCADE
16:50730088	*CASC16*	REACTOME_TOLL_LIKE_RECEPTOR_4_TLR4_CASCADE
16:50730446	*CASC16*	REACTOME_TOLL_LIKE_RECEPTOR_4_TLR4_CASCADE
16:50745926	*CASC16*	REACTOME_TOLL_LIKE_RECEPTOR_4_TLR4_CASCADE
6:32151443	*HLA-DRB5*	REACTOME_TOLL_LIKE_RECEPTOR_9_TLR9_CASCADE
10:103185542	*GBF1*	REACTOME_TOLL_LIKE_RECEPTOR_9_TLR9_CASCADE
10:103308244	*GBF1*	REACTOME_TOLL_LIKE_RECEPTOR_9_TLR9_CASCADE
16:50730088	*CASC16*	REACTOME_TOLL_LIKE_RECEPTOR_9_TLR9_CASCADE
16:50730446	*CASC16*	REACTOME_TOLL_LIKE_RECEPTOR_9_TLR9_CASCADE
16:50745926	*CASC16*	REACTOME_TOLL_LIKE_RECEPTOR_9_TLR9_CASCADE
1:171945488	*VAMP4*	REACTOME_TOLL_LIKE_RECEPTOR_CASCADES
1:172137119	*VAMP4*	REACTOME_TOLL_LIKE_RECEPTOR_CASCADES
1:172386261	*VAMP4*	REACTOME_TOLL_LIKE_RECEPTOR_CASCADES
6:32151443	*HLA-DRB5*	REACTOME_TOLL_LIKE_RECEPTOR_CASCADES
8:11712443	*CTSB*	REACTOME_TOLL_LIKE_RECEPTOR_CASCADES
10:103185542	*GBF1*	REACTOME_TOLL_LIKE_RECEPTOR_CASCADES
10:103308244	*GBF1*	REACTOME_TOLL_LIKE_RECEPTOR_CASCADES
16:50730088	*CASC16*	REACTOME_TOLL_LIKE_RECEPTOR_CASCADES
16:50730446	*CASC16*	REACTOME_TOLL_LIKE_RECEPTOR_CASCADES
16:50745926	*CASC16*	REACTOME_TOLL_LIKE_RECEPTOR_CASCADES
6:32151443	*HLA-DRB5*	REACTOME_TOLL_LIKE_RECEPTOR_TLR1:TLR2_CASCADE
10:103185542	*GBF1*	REACTOME_TOLL_LIKE_RECEPTOR_TLR1:TLR2_CASCADE
10:103308244	*GBF1*	REACTOME_TOLL_LIKE_RECEPTOR_TLR1:TLR2_CASCADE
16:50730088	*CASC16*	REACTOME_TOLL_LIKE_RECEPTOR_TLR1:TLR2_CASCADE
16:50730446	*CASC16*	REACTOME_TOLL_LIKE_RECEPTOR_TLR1:TLR2_CASCADE

16:50745926	*CASC16*	REACTOME_TOLL_LIKE_RECEPTOR_TLR1:TLR2_CASCADE
17:7578671	*V1NB1*	REACTOME_TP53_REGULATES_METABOLIC_GENES
17:7578671	*V1NB1*	'P53_REGULATES_TRANSCRIPTION_OF_ADDITIONAL_CELL_CYCLE_GENES_WHOSE_EXACT_ROLE_IN_THE_P53_PATHWAY_REM/
17:7578671	*V1NB1*	REACTOME_TP53_REGULATES_TRANSCRIPTION_OF_CASPASE_ACTIVATORS_AND_CASPASES
17:7578671	*V1NB1*	REACTOME_TP53_REGULATES_TRANSCRIPTION_OF_CELL_CYCLE_GENES
8:23050400	*BIN3*	REACTOME_TP53_REGULATES_TRANSCRIPTION_OF_CELL_DEATH_GENES
17:7578671	*V1NB1*	REACTOME_TP53_REGULATES_TRANSCRIPTION_OF_CELL_DEATH_GENES
8:23050400	*BIN3*	REACTOME_TP53_REGULATES_TRANSCRIPTION_OF_DEATH_RECEPTORS_AND_LIGANDS
17:7578671	*V1NB1*	REACTOME_TP53_REGULATES_TRANSCRIPTION_OF_DEATH_RECEPTORS_AND_LIGANDS
2:135708115	*TMEM163*	REACTOME_TP53_REGULATES_TRANSCRIPTION_OF_DNA_REPAIR_GENES
6:31925679	*HLA-DRB5*	REACTOME_TP53_REGULATES_TRANSCRIPTION_OF_DNA_REPAIR_GENES
17:7578671	*V1NB1*	REACTOME_TP53_REGULATES_TRANSCRIPTION_OF_DNA_REPAIR_GENES
17:7578671	*V1NB1*	REACTOME_TP53_REGULATES_TRANSCRIPTION_OF_GENES_INVOLVED_IN_CYTOCHROME_C_RELEASE
17:7578671	*V1NB1*	REACTOME_TP53_REGULATES_TRANSCRIPTION_OF_GENES_INVOLVED_IN_G1_CELL_CYCLE_ARREST
17:7578671	*V1NB1*	REACTOME_TP53_REGULATES_TRANSCRIPTION_OF_GENES_INVOLVED_IN_G2_CELL_CYCLE_ARREST
17:7578671	*V1NB1*	ATES_TRANSCRIPTION_OF_SEVERAL_ADDITIONAL_CELL_DEATH_GENES_WHOSE_SPECIFIC_ROLES_IN_P53_DEPENDENT_APOP
6:32151443	*HLA-DRB5*	REACTOME_TRAF6_MEDIATED_NF_KB_ACTIVATION
8:11712443	*CTSB*	REACTOME_TRAFFICKING_AND_PROCESSING_OF_ENDOSOMAL_TLR
17:44800946	*WNT3*	REACTOME_TRAFFICKING_OF_GLUR2_CONTAINING_AMPA_RECEPTORS
17:44833217	*WNT3*	REACTOME_TRAFFICKING_OF_GLUR2_CONTAINING_AMPA_RECEPTORS
10:104443875	*GBF1*	REACTOME_TRAFFICKING_OF_MYRISTOYLATED_PROTEINS_TO_THE_CILIUM
10:104452818	*GBF1*	REACTOME_TRAFFICKING_OF_MYRISTOYLATED_PROTEINS_TO_THE_CILIUM
8:23050400	*BIN3*	REACTOME_TRAIL_SIGNALING
2:135708115	*TMEM163*	REACTOME_TRANSCRIPTIONAL_ACTIVITY_OF_SMAD2_SMAD3:SMAD4_HETEROTRIMER
6:31862876	*HLA-DRB5*	REACTOME_TRANSCRIPTIONAL_REGULATION_BY_E2F6
4:114424939	*CAMK2D*	REACTOME_TRANSCRIPTIONAL_REGULATION_BY_MECP2
6:32814942	*HLA-DRB5*	REACTOME_TRANSCRIPTIONAL_REGULATION_BY_RUNX1
8:11381148	*CTSB*	REACTOME_TRANSCRIPTIONAL_REGULATION_BY_RUNX1
12:46134812	*SCAF11*	REACTOME_TRANSCRIPTIONAL_REGULATION_BY_RUNX1
14:55515304	*GCH1*	REACTOME_TRANSCRIPTIONAL_REGULATION_BY_RUNX1
6:32814942	*HLA-DRB5*	REACTOME_TRANSCRIPTIONAL_REGULATION_BY_RUNX2
6:32814942	*HLA-DRB5*	REACTOME_TRANSCRIPTIONAL_REGULATION_BY_RUNX3
6:32941506	*HLA-DRB5*	REACTOME_TRANSCRIPTIONAL_REGULATION_BY_RUNX3
16:30510571	*SETD1A*	REACTOME_TRANSCRIPTIONAL_REGULATION_BY_RUNX3
16:30516492	*SETD1A*	REACTOME_TRANSCRIPTIONAL_REGULATION_BY_RUNX3
16:30520856	*SETD1A*	REACTOME_TRANSCRIPTIONAL_REGULATION_BY_RUNX3
17:7578671	*V1NB1*	REACTOME_TRANSCRIPTIONAL_REGULATION_BY_RUNX3
2:135708115	*TMEM163*	REACTOME_TRANSCRIPTIONAL_REGULATION_BY_TP53
6:31862876	*HLA-DRB5*	REACTOME_TRANSCRIPTIONAL_REGULATION_BY_TP53
6:31925679	*HLA-DRB5*	REACTOME_TRANSCRIPTIONAL_REGULATION_BY_TP53
8:23050400	*BIN3*	REACTOME_TRANSCRIPTIONAL_REGULATION_BY_TP53
17:7578671	*V1NB1*	REACTOME_TRANSCRIPTIONAL_REGULATION_BY_TP53
17:59922925	*BRIP1*	REACTOME_TRANSCRIPTIONAL_REGULATION_BY_TP53
4:17622910	*LCORL*	REACTOME_TRANSCRIPTIONAL_REGULATION_OF_WHITE_ADIPOCYTE_DIFFERENTIATION
4:17632603	*LCORL*	REACTOME_TRANSCRIPTIONAL_REGULATION_OF_WHITE_ADIPOCYTE_DIFFERENTIATION
12:133206648	*FBRSL1*	REACTOME_TRANSCRIPTION_COUPLED_NUCLEOTIDE_EXCISION_REPAIR_TC_NER
12:133223685	*FBRSL1*	REACTOME_TRANSCRIPTION_COUPLED_NUCLEOTIDE_EXCISION_REPAIR_TC_NER
12:133225045	*FBRSL1*	REACTOME_TRANSCRIPTION_COUPLED_NUCLEOTIDE_EXCISION_REPAIR_TC_NER
3:48199695	*IP6K2*	REACTOME_TRANSCRIPTION_OF_E2F_TARGETS_UNDER_NEGATIVE_CONTROL_BY_DREAM_COMPLEX
3:48214438	*IP6K2*	REACTOME_TRANSCRIPTION_OF_E2F_TARGETS_UNDER_NEGATIVE_CONTROL_BY_DREAM_COMPLEX
2:135708115	*TMEM163*	REACTOME_TRANSCRIPTION_OF_THE_HIV_GENOME
6:31925679	*HLA-DRB5*	REACTOME_TRANSCRIPTION_OF_THE_HIV_GENOME
12:124219791	*HIP1R*	REACTOME_TRANSFERRIN_ENDOCYTOSIS_AND_RECYCLING

3:121411876	*KPNA1*	REACTOME_TRANS_GOLGI_NETWORK_VESICLE_BUDDING
3:121412702	*KPNA1*	REACTOME_TRANS_GOLGI_NETWORK_VESICLE_BUDDING
4:925376	*GAK*	REACTOME_TRANS_GOLGI_NETWORK_VESICLE_BUDDING
9:17579690	*SH3GL2_2*	REACTOME_TRANS_GOLGI_NETWORK_VESICLE_BUDDING
9:17609302	*SH3GL2_2*	REACTOME_TRANS_GOLGI_NETWORK_VESICLE_BUDDING
9:17627444	*SH3GL2_2*	REACTOME_TRANS_GOLGI_NETWORK_VESICLE_BUDDING
9:17710876	*SH3GL2_2*	REACTOME_TRANS_GOLGI_NETWORK_VESICLE_BUDDING
9:17732590	*SH3GL2_2*	REACTOME_TRANS_GOLGI_NETWORK_VESICLE_BUDDING
9:17737514	*SH3GL2_2*	REACTOME_TRANS_GOLGI_NETWORK_VESICLE_BUDDING
9:17758945	*SH3GL2_2*	REACTOME_TRANS_GOLGI_NETWORK_VESICLE_BUDDING
10:104005410	*GBF1*	REACTOME_TRANS_GOLGI_NETWORK_VESICLE_BUDDING
10:104034550	*GBF1*	REACTOME_TRANS_GOLGI_NETWORK_VESICLE_BUDDING
12:123326598	*HIP1R*	REACTOME_TRANS_GOLGI_NETWORK_VESICLE_BUDDING
1:155698425	*GBAP1*	REACTOME_TRANSLATION
6:31758240	*HLA-DRB5*	REACTOME_TRANSLATION
6:31762311	*HLA-DRB5*	REACTOME_TRANSLATION
17:7479140	*V1NB1*	REACTOME_TRANSLATION
17:7480325	*V1NB1*	REACTOME_TRANSLATION
6:111628626	*FYN*	REACTOME_TRANSLESION_SYNTHESIS_BY_POLK
6:111628626	*FYN*	REACTOME_TRANSLESION_SYNTHESIS_BY_Y_FAMILY_DNA_POLYMERASES_BYPASSES_LESIONS_ON_DNA_TEMPLATE
12:133206648	*FBRSL1*	REACTOME_TRANSLESION_SYNTHESIS_BY_Y_FAMILY_DNA_POLYMERASES_BYPASSES_LESIONS_ON_DNA_TEMPLATE
12:133223685	*FBRSL1*	REACTOME_TRANSLESION_SYNTHESIS_BY_Y_FAMILY_DNA_POLYMERASES_BYPASSES_LESIONS_ON_DNA_TEMPLATE
12:133225045	*FBRSL1*	REACTOME_TRANSLESION_SYNTHESIS_BY_Y_FAMILY_DNA_POLYMERASES_BYPASSES_LESIONS_ON_DNA_TEMPLATE
6:32602372	*HLA-DRB5*	REACTOME_TRANSLOCATION_OF_ZAP_70_TO_IMMUNOLOGICAL_SYNAPSE
3:48835245	*IP6K2*	REACTOME_TRANSMISSION_ACROSS_CHEMICAL_SYNAPSES
3:123057397	*KPNA1*	REACTOME_TRANSMISSION_ACROSS_CHEMICAL_SYNAPSES
4:114424939	*CAMK2D*	REACTOME_TRANSMISSION_ACROSS_CHEMICAL_SYNAPSES
6:29579544	*TRIM40*	REACTOME_TRANSMISSION_ACROSS_CHEMICAL_SYNAPSES
11:83188607	*DLG2*	REACTOME_TRANSMISSION_ACROSS_CHEMICAL_SYNAPSES
11:83219006	*DLG2*	REACTOME_TRANSMISSION_ACROSS_CHEMICAL_SYNAPSES
11:83538733	*DLG2*	REACTOME_TRANSMISSION_ACROSS_CHEMICAL_SYNAPSES
11:83544472	*DLG2*	REACTOME_TRANSMISSION_ACROSS_CHEMICAL_SYNAPSES
11:83656201	*DLG2*	REACTOME_TRANSMISSION_ACROSS_CHEMICAL_SYNAPSES
11:83700228	*DLG2*	REACTOME_TRANSMISSION_ACROSS_CHEMICAL_SYNAPSES
11:83762918	*DLG2*	REACTOME_TRANSMISSION_ACROSS_CHEMICAL_SYNAPSES
11:83894247	*DLG2*	REACTOME_TRANSMISSION_ACROSS_CHEMICAL_SYNAPSES
11:84021037	*DLG2*	REACTOME_TRANSMISSION_ACROSS_CHEMICAL_SYNAPSES
11:84324361	*DLG2*	REACTOME_TRANSMISSION_ACROSS_CHEMICAL_SYNAPSES
11:84373496	*DLG2*	REACTOME_TRANSMISSION_ACROSS_CHEMICAL_SYNAPSES
11:84403409	*DLG2*	REACTOME_TRANSMISSION_ACROSS_CHEMICAL_SYNAPSES
17:7101771	*V1NB1*	REACTOME_TRANSMISSION_ACROSS_CHEMICAL_SYNAPSES
17:43999203	*WNT3*	REACTOME_TRANSMISSION_ACROSS_CHEMICAL_SYNAPSES
17:44005297	*WNT3*	REACTOME_TRANSMISSION_ACROSS_CHEMICAL_SYNAPSES
17:44019712	*WNT3*	REACTOME_TRANSMISSION_ACROSS_CHEMICAL_SYNAPSES
17:44060492	*WNT3*	REACTOME_TRANSMISSION_ACROSS_CHEMICAL_SYNAPSES
17:44065263	*WNT3*	REACTOME_TRANSMISSION_ACROSS_CHEMICAL_SYNAPSES
17:44081178	*WNT3*	REACTOME_TRANSMISSION_ACROSS_CHEMICAL_SYNAPSES
17:44083297	*WNT3*	REACTOME_TRANSMISSION_ACROSS_CHEMICAL_SYNAPSES
17:44800946	*WNT3*	REACTOME_TRANSMISSION_ACROSS_CHEMICAL_SYNAPSES
17:44833217	*WNT3*	REACTOME_TRANSMISSION_ACROSS_CHEMICAL_SYNAPSES
21:39197323	*DYRK1A*	REACTOME_TRANSMISSION_ACROSS_CHEMICAL_SYNAPSES
6:31846234	*HLA-DRB5*	REACTOME_TRANSPORT_OF_BILE_SALTS_AND_ORGANIC_ACIDS_METAL_IONS_AND_AMINE_COMPOUNDS
12:40240262	*LRRK2_2*	REACTOME_TRANSPORT_OF_BILE_SALTS_AND_ORGANIC_ACIDS_METAL_IONS_AND_AMINE_COMPOUNDS

12:40252360	*LRRK2_2*	REACTOME_TRANSPORT_OF_BILE_SALTS_AND_ORGANIC_ACIDS_METAL_IONS_AND_AMINE_COMPOUNDS
12:40267539	*LRRK2_2*	REACTOME_TRANSPORT_OF_BILE_SALTS_AND_ORGANIC_ACIDS_METAL_IONS_AND_AMINE_COMPOUNDS
12:40302599	*LRRK2_2*	REACTOME_TRANSPORT_OF_BILE_SALTS_AND_ORGANIC_ACIDS_METAL_IONS_AND_AMINE_COMPOUNDS
12:40348056	*LRRK2_2*	REACTOME_TRANSPORT_OF_BILE_SALTS_AND_ORGANIC_ACIDS_METAL_IONS_AND_AMINE_COMPOUNDS
12:40349236	*LRRK2_2*	REACTOME_TRANSPORT_OF_BILE_SALTS_AND_ORGANIC_ACIDS_METAL_IONS_AND_AMINE_COMPOUNDS
12:40378773	*LRRK2_2*	REACTOME_TRANSPORT_OF_BILE_SALTS_AND_ORGANIC_ACIDS_METAL_IONS_AND_AMINE_COMPOUNDS
12:40393852	*LRRK2_2*	REACTOME_TRANSPORT_OF_BILE_SALTS_AND_ORGANIC_ACIDS_METAL_IONS_AND_AMINE_COMPOUNDS
12:40405478	*LRRK2_2*	REACTOME_TRANSPORT_OF_BILE_SALTS_AND_ORGANIC_ACIDS_METAL_IONS_AND_AMINE_COMPOUNDS
12:40413305	*LRRK2_2*	REACTOME_TRANSPORT_OF_BILE_SALTS_AND_ORGANIC_ACIDS_METAL_IONS_AND_AMINE_COMPOUNDS
12:40414626	*LRRK2_2*	REACTOME_TRANSPORT_OF_BILE_SALTS_AND_ORGANIC_ACIDS_METAL_IONS_AND_AMINE_COMPOUNDS
12:40437969	*LRRK2_2*	REACTOME_TRANSPORT_OF_BILE_SALTS_AND_ORGANIC_ACIDS_METAL_IONS_AND_AMINE_COMPOUNDS
1:205884274	*RAB29*	REACTOME_TRANSPORT_OF_INORGANIC_CATIONS_ANIONS_AND_AMINO_ACIDS_OLIGOPEPTIDES
8:17427186	*FGF20*	REACTOME_TRANSPORT_OF_INORGANIC_CATIONS_ANIONS_AND_AMINO_ACIDS_OLIGOPEPTIDES
12:47192323	*SCAF11*	REACTOME_TRANSPORT_OF_INORGANIC_CATIONS_ANIONS_AND_AMINO_ACIDS_OLIGOPEPTIDES
17:42338993	*FAM171A2*	REACTOME_TRANSPORT_OF_INORGANIC_CATIONS_ANIONS_AND_AMINO_ACIDS_OLIGOPEPTIDES
1:154306180	*GBAP1*	REACTOME_TRANSPORT_OF_SMALL_MOLECULES
1:205645310	*RAB29*	REACTOME_TRANSPORT_OF_SMALL_MOLECULES
1:205884274	*RAB29*	REACTOME_TRANSPORT_OF_SMALL_MOLECULES
3:48835245	*IP6K2*	REACTOME_TRANSPORT_OF_SMALL_MOLECULES
3:123057397	*KPNA1*	REACTOME_TRANSPORT_OF_SMALL_MOLECULES
4:15608677	*BST1*	REACTOME_TRANSPORT_OF_SMALL_MOLECULES
4:114424939	*CAMK2D*	REACTOME_TRANSPORT_OF_SMALL_MOLECULES
4:170563153	*CLCN3*	REACTOME_TRANSPORT_OF_SMALL_MOLECULES
4:170569172	*CLCN3*	REACTOME_TRANSPORT_OF_SMALL_MOLECULES
4:170620812	*CLCN3*	REACTOME_TRANSPORT_OF_SMALL_MOLECULES
6:31846234	*HLA-DRB5*	REACTOME_TRANSPORT_OF_SMALL_MOLECULES
6:32151443	*HLA-DRB5*	REACTOME_TRANSPORT_OF_SMALL_MOLECULES
6:32814942	*HLA-DRB5*	REACTOME_TRANSPORT_OF_SMALL_MOLECULES
8:17427186	*FGF20*	REACTOME_TRANSPORT_OF_SMALL_MOLECULES
12:39960237	*LRRK2_2*	REACTOME_TRANSPORT_OF_SMALL_MOLECULES
12:40240262	*LRRK2_2*	REACTOME_TRANSPORT_OF_SMALL_MOLECULES
12:40252360	*LRRK2_2*	REACTOME_TRANSPORT_OF_SMALL_MOLECULES
12:40267539	*LRRK2_2*	REACTOME_TRANSPORT_OF_SMALL_MOLECULES
12:40302599	*LRRK2_2*	REACTOME_TRANSPORT_OF_SMALL_MOLECULES
12:40348056	*LRRK2_2*	REACTOME_TRANSPORT_OF_SMALL_MOLECULES
12:40349236	*LRRK2_2*	REACTOME_TRANSPORT_OF_SMALL_MOLECULES
12:40378773	*LRRK2_2*	REACTOME_TRANSPORT_OF_SMALL_MOLECULES
12:40393852	*LRRK2_2*	REACTOME_TRANSPORT_OF_SMALL_MOLECULES
12:40405478	*LRRK2_2*	REACTOME_TRANSPORT_OF_SMALL_MOLECULES
12:40413305	*LRRK2_2*	REACTOME_TRANSPORT_OF_SMALL_MOLECULES
12:40414626	*LRRK2_2*	REACTOME_TRANSPORT_OF_SMALL_MOLECULES
12:40437969	*LRRK2_2*	REACTOME_TRANSPORT_OF_SMALL_MOLECULES
12:45755099	*SCAF11*	REACTOME_TRANSPORT_OF_SMALL_MOLECULES
12:47192323	*SCAF11*	REACTOME_TRANSPORT_OF_SMALL_MOLECULES
12:123453034	*HIP1R*	REACTOME_TRANSPORT_OF_SMALL_MOLECULES
12:124219791	*HIP1R*	REACTOME_TRANSPORT_OF_SMALL_MOLECULES
17:7560294	*V1NB1*	REACTOME_TRANSPORT_OF_SMALL_MOLECULES
17:40938261	*RETREG3*	REACTOME_TRANSPORT_OF_SMALL_MOLECULES
17:42338993	*FAM171A2*	REACTOME_TRANSPORT_OF_SMALL_MOLECULES
19:1811130	*SPPL2B*	REACTOME_TRANSPORT_OF_SMALL_MOLECULES
2:134977261	*TMEM163*	REACTOME_TRANSPORT_TO_THE_GOLGI_AND_SUBSEQUENT_MODIFICATION
2:135194840	*TMEM163*	REACTOME_TRANSPORT_TO_THE_GOLGI_AND_SUBSEQUENT_MODIFICATION
2:135202455	*TMEM163*	REACTOME_TRANSPORT_TO_THE_GOLGI_AND_SUBSEQUENT_MODIFICATION

3:121411876	*KPNA1*	REACTOME_TRANSPORT_TO_THE_GOLGI_AND_SUBSEQUENT_MODIFICATION
3:121412702	*KPNA1*	REACTOME_TRANSPORT_TO_THE_GOLGI_AND_SUBSEQUENT_MODIFICATION
4:76695927	*FAM47E-STBD1*	REACTOME_TRANSPORT_TO_THE_GOLGI_AND_SUBSEQUENT_MODIFICATION
4:113938109	*CAMK2D*	REACTOME_TRANSPORT_TO_THE_GOLGI_AND_SUBSEQUENT_MODIFICATION
4:114077386	*CAMK2D*	REACTOME_TRANSPORT_TO_THE_GOLGI_AND_SUBSEQUENT_MODIFICATION
4:114217997	*CAMK2D*	REACTOME_TRANSPORT_TO_THE_GOLGI_AND_SUBSEQUENT_MODIFICATION
10:104005410	*GBF1*	REACTOME_TRANSPORT_TO_THE_GOLGI_AND_SUBSEQUENT_MODIFICATION
10:104034550	*GBF1*	REACTOME_TRANSPORT_TO_THE_GOLGI_AND_SUBSEQUENT_MODIFICATION
10:104258303	*GBF1*	REACTOME_TRANSPORT_TO_THE_GOLGI_AND_SUBSEQUENT_MODIFICATION
10:121683206	*INPP5F*	REACTOME_TRANSPORT_TO_THE_GOLGI_AND_SUBSEQUENT_MODIFICATION
10:121686714	*INPP5F*	REACTOME_TRANSPORT_TO_THE_GOLGI_AND_SUBSEQUENT_MODIFICATION
10:121689030	*INPP5F*	REACTOME_TRANSPORT_TO_THE_GOLGI_AND_SUBSEQUENT_MODIFICATION
17:44800946	*WNT3*	REACTOME_TRANSPORT_TO_THE_GOLGI_AND_SUBSEQUENT_MODIFICATION
17:44833217	*WNT3*	REACTOME_TRANSPORT_TO_THE_GOLGI_AND_SUBSEQUENT_MODIFICATION
17:45059032	*WNT3*	REACTOME_TRANSPORT_TO_THE_GOLGI_AND_SUBSEQUENT_MODIFICATION
17:45089680	*WNT3*	REACTOME_TRANSPORT_TO_THE_GOLGI_AND_SUBSEQUENT_MODIFICATION
6:32151443	*HLA-DRB5*	REACTOME_TRIF_TICAM1_MEDIATED_TLR4_SIGNALING
10:103185542	*GBF1*	REACTOME_TRIF_TICAM1_MEDIATED_TLR4_SIGNALING
10:103308244	*GBF1*	REACTOME_TRIF_TICAM1_MEDIATED_TLR4_SIGNALING
16:50730088	*CASC16*	REACTOME_TRIF_TICAM1_MEDIATED_TLR4_SIGNALING
16:50730446	*CASC16*	REACTOME_TRIF_TICAM1_MEDIATED_TLR4_SIGNALING
16:50745926	*CASC16*	REACTOME_TRIF_TICAM1_MEDIATED_TLR4_SIGNALING
6:31758240	*HLA-DRB5*	REACTOME_TRNA_AMINOACYLATION
6:31762311	*HLA-DRB5*	REACTOME_TRNA_AMINOACYLATION
16:19085298	*SYT17*	REACTOME_UBIQUINOL_BIOSYNTHESIS
3:48199695	*IP6K2*	REACTOME_UB_SPECIFIC_PROCESSING_PROTEASES
3:48214438	*IP6K2*	REACTOME_UB_SPECIFIC_PROCESSING_PROTEASES
3:49740895	*IP6K2*	REACTOME_UB_SPECIFIC_PROCESSING_PROTEASES
6:32814942	*HLA-DRB5*	REACTOME_UB_SPECIFIC_PROCESSING_PROTEASES
17:7578671	*V1NB1*	REACTOME_UB_SPECIFIC_PROCESSING_PROTEASES
6:32814942	*HLA-DRB5*	REACTOME_UCH_PROTEINASES
4:114424939	*CAMK2D*	REACTOME_UNBLOCKING_OF_NMDA_RECEPTORS_GLUTAMATE_BINDING_AND_ACTIVATION
11:83188607	*DLG2*	REACTOME_UNBLOCKING_OF_NMDA_RECEPTORS_GLUTAMATE_BINDING_AND_ACTIVATION
11:83219006	*DLG2*	REACTOME_UNBLOCKING_OF_NMDA_RECEPTORS_GLUTAMATE_BINDING_AND_ACTIVATION
11:83538733	*DLG2*	REACTOME_UNBLOCKING_OF_NMDA_RECEPTORS_GLUTAMATE_BINDING_AND_ACTIVATION
11:83544472	*DLG2*	REACTOME_UNBLOCKING_OF_NMDA_RECEPTORS_GLUTAMATE_BINDING_AND_ACTIVATION
11:83656201	*DLG2*	REACTOME_UNBLOCKING_OF_NMDA_RECEPTORS_GLUTAMATE_BINDING_AND_ACTIVATION
11:83700228	*DLG2*	REACTOME_UNBLOCKING_OF_NMDA_RECEPTORS_GLUTAMATE_BINDING_AND_ACTIVATION
11:83762918	*DLG2*	REACTOME_UNBLOCKING_OF_NMDA_RECEPTORS_GLUTAMATE_BINDING_AND_ACTIVATION
11:83894247	*DLG2*	REACTOME_UNBLOCKING_OF_NMDA_RECEPTORS_GLUTAMATE_BINDING_AND_ACTIVATION
11:84021037	*DLG2*	REACTOME_UNBLOCKING_OF_NMDA_RECEPTORS_GLUTAMATE_BINDING_AND_ACTIVATION
11:84324361	*DLG2*	REACTOME_UNBLOCKING_OF_NMDA_RECEPTORS_GLUTAMATE_BINDING_AND_ACTIVATION
11:84373496	*DLG2*	REACTOME_UNBLOCKING_OF_NMDA_RECEPTORS_GLUTAMATE_BINDING_AND_ACTIVATION
11:84403409	*DLG2*	REACTOME_UNBLOCKING_OF_NMDA_RECEPTORS_GLUTAMATE_BINDING_AND_ACTIVATION
17:7101771	*V1NB1*	REACTOME_UNBLOCKING_OF_NMDA_RECEPTORS_GLUTAMATE_BINDING_AND_ACTIVATION
1:156059276	*GBAP1*	REACTOME_UNFOLDED_PROTEIN_RESPONSE_UPR
1:161875645	*FCGR2A*	REACTOME_UNFOLDED_PROTEIN_RESPONSE_UPR
1:161930954	*FCGR2A*	REACTOME_UNFOLDED_PROTEIN_RESPONSE_UPR
3:150298317	*MED12L*	REACTOME_UNFOLDED_PROTEIN_RESPONSE_UPR
17:45059032	*WNT3*	REACTOME_UNFOLDED_PROTEIN_RESPONSE_UPR
17:45089680	*WNT3*	REACTOME_UNFOLDED_PROTEIN_RESPONSE_UPR
20:5934513	*CRLS1*	REACTOME_UNWINDING_OF_DNA
20:5968737	*CRLS1*	REACTOME_UNWINDING_OF_DNA

20:5968964	*CRLS1*	REACTOME_UNWINDING_OF_DNA
16:31004169	*SETD1A*	REACTOME_UPTAKE_AND_ACTIONS_OF_BACTERIAL_TOXINS
3:48835245	*IP6K2*	REACTOME_VASOPRESSIN_REGULATES_RENAL_WATER_HOMEOSTASIS_VIA_AQUAPORINS
3:123057397	*KPNA1*	REACTOME_VASOPRESSIN_REGULATES_RENAL_WATER_HOMEOSTASIS_VIA_AQUAPORINS
17:39883520	*RETREG3*	REACTOME_VEGFR2_MEDIATED_VASCULAR_PERMEABILITY
1:155847427	*GBAP1*	REACTOME_VESICLE_MEDIATED_TRANSPORT
1:171689486	*VAMP4*	REACTOME_VESICLE_MEDIATED_TRANSPORT
1:171945488	*VAMP4*	REACTOME_VESICLE_MEDIATED_TRANSPORT
1:172137119	*VAMP4*	REACTOME_VESICLE_MEDIATED_TRANSPORT
1:172386261	*VAMP4*	REACTOME_VESICLE_MEDIATED_TRANSPORT
2:135921205	*TMEM163*	REACTOME_VESICLE_MEDIATED_TRANSPORT
2:169999639	*STK39*	REACTOME_VESICLE_MEDIATED_TRANSPORT
2:170028024	*STK39*	REACTOME_VESICLE_MEDIATED_TRANSPORT
3:121411876	*KPNA1*	REACTOME_VESICLE_MEDIATED_TRANSPORT
3:121412702	*KPNA1*	REACTOME_VESICLE_MEDIATED_TRANSPORT
4:925376	*GAK*	REACTOME_VESICLE_MEDIATED_TRANSPORT
4:76695927	*FAM47E-STBD1*	REACTOME_VESICLE_MEDIATED_TRANSPORT
4:77100807	*FAM47E-STBD1*	REACTOME_VESICLE_MEDIATED_TRANSPORT
4:77104152	*FAM47E-STBD1*	REACTOME_VESICLE_MEDIATED_TRANSPORT
4:77124196	*FAM47E-STBD1*	REACTOME_VESICLE_MEDIATED_TRANSPORT
4:77134786	*FAM47E-STBD1*	REACTOME_VESICLE_MEDIATED_TRANSPORT
4:113938109	*CAMK2D*	REACTOME_VESICLE_MEDIATED_TRANSPORT
4:114077386	*CAMK2D*	REACTOME_VESICLE_MEDIATED_TRANSPORT
4:114217997	*CAMK2D*	REACTOME_VESICLE_MEDIATED_TRANSPORT
8:17125768	*FGF20*	REACTOME_VESICLE_MEDIATED_TRANSPORT
9:17579690	*SH3GL2_2*	REACTOME_VESICLE_MEDIATED_TRANSPORT
9:17609302	*SH3GL2_2*	REACTOME_VESICLE_MEDIATED_TRANSPORT
9:17627444	*SH3GL2_2*	REACTOME_VESICLE_MEDIATED_TRANSPORT
9:17710876	*SH3GL2_2*	REACTOME_VESICLE_MEDIATED_TRANSPORT
9:17732590	*SH3GL2_2*	REACTOME_VESICLE_MEDIATED_TRANSPORT
9:17737514	*SH3GL2_2*	REACTOME_VESICLE_MEDIATED_TRANSPORT
9:17758945	*SH3GL2_2*	REACTOME_VESICLE_MEDIATED_TRANSPORT
10:104005410	*GBF1*	REACTOME_VESICLE_MEDIATED_TRANSPORT
10:104034550	*GBF1*	REACTOME_VESICLE_MEDIATED_TRANSPORT
10:104258303	*GBF1*	REACTOME_VESICLE_MEDIATED_TRANSPORT
10:121683206	*INPP5F*	REACTOME_VESICLE_MEDIATED_TRANSPORT
10:121686714	*INPP5F*	REACTOME_VESICLE_MEDIATED_TRANSPORT
10:121689030	*INPP5F*	REACTOME_VESICLE_MEDIATED_TRANSPORT
12:39759090	*LRRK2_2*	REACTOME_VESICLE_MEDIATED_TRANSPORT
12:123326598	*HIP1R*	REACTOME_VESICLE_MEDIATED_TRANSPORT
17:7129974	*V1NB1*	REACTOME_VESICLE_MEDIATED_TRANSPORT
17:43021962	*CRHR1_2*	REACTOME_VESICLE_MEDIATED_TRANSPORT
17:44800946	*WNT3*	REACTOME_VESICLE_MEDIATED_TRANSPORT
17:44833217	*WNT3*	REACTOME_VESICLE_MEDIATED_TRANSPORT
17:45059032	*WNT3*	REACTOME_VESICLE_MEDIATED_TRANSPORT
17:45089680	*WNT3*	REACTOME_VESICLE_MEDIATED_TRANSPORT
17:76727455	*DNAH17*	REACTOME_VESICLE_MEDIATED_TRANSPORT
6:32814942	*HLA-DRB5*	REACTOME_VIF_MEDIATED_DEGRADATION_OF_APOBEC3G
2:169999639	*STK39*	REACTOME_VISUAL_PHOTOTRANSDUCTION
2:170028024	*STK39*	REACTOME_VISUAL_PHOTOTRANSDUCTION
10:15199347	*ITGA8*	REACTOME_VISUAL_PHOTOTRANSDUCTION
17:43132290	*CRHR1_2*	REACTOME_VISUAL_PHOTOTRANSDUCTION
6:133025715	*RPS12*	REACTOME_VITAMIN_B5_PANTOTHENATE_METABOLISM

6:133028870	*RPS12*	REACTOME_VITAMIN_B5_PANTOTHENATE_METABOLISM
6:133034076	*RPS12*	REACTOME_VITAMIN_B5_PANTOTHENATE_METABOLISM
2:169999639	*STK39*	REACTOME_VITAMIN_D_CALCIFEROL_METABOLISM
2:170028024	*STK39*	REACTOME_VITAMIN_D_CALCIFEROL_METABOLISM
2:18143082	*KCNS3*	REACTOME_VOLTAGE_GATED_POTASSIUM_CHANNELS
2:18176120	*KCNS3*	REACTOME_VOLTAGE_GATED_POTASSIUM_CHANNELS
2:18247777	*KCNS3*	REACTOME_VOLTAGE_GATED_POTASSIUM_CHANNELS
2:18296870	*KCNS3*	REACTOME_VOLTAGE_GATED_POTASSIUM_CHANNELS
2:18335515	*KCNS3*	REACTOME_VOLTAGE_GATED_POTASSIUM_CHANNELS
3:19312697	*SATB1*	REACTOME_VOLTAGE_GATED_POTASSIUM_CHANNELS
3:19334895	*SATB1*	REACTOME_VOLTAGE_GATED_POTASSIUM_CHANNELS
17:7832034	*V1NB1*	REACTOME_VOLTAGE_GATED_POTASSIUM_CHANNELS
8:131153067	*FAM49B*	REACTOME_VXPX_CARGO_TARGETING_TO_CILIUM
8:131190319	*FAM49B*	REACTOME_VXPX_CARGO_TARGETING_TO_CILIUM
8:131427864	*FAM49B*	REACTOME_VXPX_CARGO_TARGETING_TO_CILIUM
10:104005410	*GBF1*	REACTOME_VXPX_CARGO_TARGETING_TO_CILIUM
10:104034550	*GBF1*	REACTOME_VXPX_CARGO_TARGETING_TO_CILIUM
17:7129974	*V1NB1*	REACTOME_WNT5A_DEPENDENT_INTERNALIZATION_OF_FZD4
17:44843651	*WNT3*	REACTOME_WNT_LIGAND_BIOGENESIS_AND_TRAFFICKING
17:44888209	*WNT3*	REACTOME_WNT_LIGAND_BIOGENESIS_AND_TRAFFICKING
17:44888715	*WNT3*	REACTOME_WNT_LIGAND_BIOGENESIS_AND_TRAFFICKING
17:44906949	*WNT3*	REACTOME_WNT_LIGAND_BIOGENESIS_AND_TRAFFICKING
17:44912901	*WNT3*	REACTOME_WNT_LIGAND_BIOGENESIS_AND_TRAFFICKING
17:44931399	*WNT3*	REACTOME_WNT_LIGAND_BIOGENESIS_AND_TRAFFICKING
17:44947756	*WNT3*	REACTOME_WNT_LIGAND_BIOGENESIS_AND_TRAFFICKING
17:7129974	*V1NB1*	REACTOME_WNT_MEDIATED_ACTIVATION_OF_DVL
17:7578671	*V1NB1*	SA_G1_AND_S_PHASES
3:48199695	*IP6K2*	SA_G2_AND_M_PHASES
3:48214438	*IP6K2*	SA_G2_AND_M_PHASES
4:170477125	*CLCN3*	SA_G2_AND_M_PHASES
3:49406095	*IP6K2*	SA_TRKA_RECEPTOR
3:49424231	*IP6K2*	SA_TRKA_RECEPTOR
3:49450137	*IP6K2*	SA_TRKA_RECEPTOR
3:49561208	*IP6K2*	SIG_BCR_SIGNALING_PATHWAY
10:103185542	*GBF1*	WNT_SIGNALING
10:103308244	*GBF1*	WNT_SIGNALING
17:7129974	*V1NB1*	WNT_SIGNALING
17:44843651	*WNT3*	WNT_SIGNALING
17:44888209	*WNT3*	WNT_SIGNALING
17:44888715	*WNT3*	WNT_SIGNALING
17:44906949	*WNT3*	WNT_SIGNALING
19:3022354	*SPPL2B*	WNT_SIGNALING
19:3036350	*SPPL2B*	WNT_SIGNALING

Supplementary Table 5. Gene prioritization based on pleiotropy across significant gene-sets associated with Parkinson's disease

Gene	N Gene sets	Gene sets
ACTR10	10	ADAPTIVE IMMUNE SYSTEM,ASPARAGINE N LINKED GLYCOSYLATION,COPI MEDIATED ANTEROGRADE TRANSPORT,ER TO GOLGI ANTEROGRADE TRANSPORT,INNATE IMMUNE SYSTEM,INTRA GOLGI AND RETROGRADE GOLGI TO ER TRAFFIC,NEUTROPHIL DEGRANULATION,POST TRANSLATIONAL PROTEIN MODIFICATION,TRANSPORT TO THE GOLGI AND SUBSEQUENT MODIFICATION,VESICLE MEDIATED TRANSPORT
AP2A2	10	ADAPTIVE IMMUNE SYSTEM,CLATHRIN MEDIATED ENDOCYTOSIS,GLUTAMATE BINDING ACTIVATION OF AMPA RECEPTORS AND SYNAPTIC PLASTICITY,INNATE IMMUNE SYSTEM,NEURONAL SYSTEM,NEUROTRANSMITTER RECEPTORS AND POSTSYNAPTIC SIGNAL TRANSMISSION, NEUTROPHIL DEGRANULATION,TRAFFICKING OF GLUR2 CONTAINING AMPA RECEPTORS,TRANSMISSION ACROSS CHEMICAL SYNAPSES,VESICLE MEDIATED TRANSPORT
ARF1	13	ADAPTIVE IMMUNE SYSTEM,ASPARAGINE N LINKED GLYCOSYLATION,COPI DEPENDENT GOLGI TO ER RETROGRADE TRAFFIC,COPI MEDIATED ANTEROGRADE TRANSPORT,ER TO GOLGI ANTEROGRADE TRANSPORT,GOLGI ASSOCIATED VESICLE BIOGENESIS,INTRA GOLGI AND RETROGRADE GOLGI TO ER TRAFFIC,INTRA GOLGI TRAFFIC,METABOLISM OF LIPIDS,POST TRANSLATIONAL PROTEIN MODIFICATION, TRANSPORT TO THE GOLGI AND SUBSEQUENT MODIFICATION, TRANS GOLGI NETWORK VESICLE BUDDING,VESICLE MEDIATED TRANSPORT
ARF3	9	ASPARAGINE N LINKED GLYCOSYLATION,COPI DEPENDENT GOLGI TO ER RETROGRADE TRAFFIC,COPI MEDIATED ANTEROGRADE TRANSPORT,ER TO GOLGI ANTEROGRADE TRANSPORT,INTRA GOLGI AND RETROGRADE GOLGI TO ER TRAFFIC,METABOLISM OF LIPIDS,POST TRANSLATIONAL PROTEIN MODIFICATION,TRANSPORT TO THE GOLGI AND SUBSEQUENT MODIFICATION,VESICLE MEDIATED TRANSPORT
ARFGAP1	9	ASPARAGINE N LINKED GLYCOSYLATION,CLATHRIN MEDIATED ENDOCYTOSIS,COPI DEPENDENT GOLGI TO ER RETROGRADE TRAFFIC,COPI MEDIATED ANTEROGRADE TRANSPORT,ER TO GOLGI ANTEROGRADE TRANSPORT,INTRA GOLGI AND RETROGRADE GOLGI TO ER TRAFFIC,POST TRANSLATIONAL PROTEIN MODIFICATION,TRANSPORT TO THE GOLGI AND SUBSEQUENT MODIFICATION,VESICLE MEDIATED TRANSPORT
BET1L	9	ASPARAGINE N LINKED GLYCOSYLATION,COPI MEDIATED ANTEROGRADE TRANSPORT,ER TO GOLGI ANTEROGRADE TRANSPORT,INTRA GOLGI AND RETROGRADE GOLGI TO ER TRAFFIC,INTRA GOLGI TRAFFIC,POST TRANSLATIONAL PROTEIN MODIFICATION,TRANSPORT TO THE GOLGI AND SUBSEQUENT MODIFICATION,VESICLE MEDIATED TRANSPORT
CALM1	10	ALZHEIMERS DISEASE,ADAPTIVE IMMUNE SYSTEM,INNATE IMMUNE SYSTEM,METABOLISM OF VITAMINS AND COFACTORS,NEURONAL SYSTEM,NEUROTRANSMITTER RECEPTORS AND POSTSYNAPTIC SIGNAL TRANSMISSION,POST TRANSLATIONAL PROTEIN MODIFICATION,SIGNALING BY GPCR,TRANSMISSION ACROSS CHEMICAL SYNAPSES,VESICLE MEDIATED TRANSPORT
CAPZA1	9	ADAPTIVE IMMUNE SYSTEM,ASPARAGINE N LINKED GLYCOSYLATION,COPI MEDIATED ANTEROGRADE TRANSPORT,ER TO GOLGI ANTEROGRADE TRANSPORT,INNATE IMMUNE SYSTEM,INTRA GOLGI AND RETROGRADE GOLGI TO ER TRAFFIC,POST TRANSLATIONAL PROTEIN MODIFICATION,TRANSPORT TO THE GOLGI AND SUBSEQUENT MODIFICATION,VESICLE MEDIATED TRANSPORT
CAPZA2	9	ADAPTIVE IMMUNE SYSTEM,ASPARAGINE N LINKED GLYCOSYLATION,COPI MEDIATED ANTEROGRADE TRANSPORT,ER TO GOLGI ANTEROGRADE TRANSPORT,INNATE IMMUNE SYSTEM,INTRA GOLGI AND RETROGRADE GOLGI TO ER TRAFFIC,POST TRANSLATIONAL PROTEIN MODIFICATION,TRANSPORT TO THE GOLGI AND SUBSEQUENT MODIFICATION,VESICLE MEDIATED TRANSPORT
CD55	11	ASPARAGINE N LINKED GLYCOSYLATION,CLASS B 2 SECRETIN FAMILY RECEPTORS,COPI MEDIATED ANTEROGRADE TRANSPORT,ER TO GOLGI ANTEROGRADE TRANSPORT,GPCR LIGAND BINDING,INNATE IMMUNE SYSTEM,NEUTROPHIL DEGRANULATION,POST TRANSLATIONAL PROTEIN MODIFICATION,SIGNALING BY GPCR,TRANSPORT TO THE GOLGI AND SUBSEQUENT MODIFICATION,VESICLE MEDIATED TRANSPORT
CD59	9	ASPARAGINE N LINKED GLYCOSYLATION,COPII MEDIATED VESICLE TRANSPORT,COPI MEDIATED ANTEROGRADE TRANSPORT,ER TO GOLGI ANTEROGRADE TRANSPORT,INNATE IMMUNE SYSTEM,NEUTROPHIL DEGRANULATION,POST TRANSLATIONAL PROTEIN MODIFICATION,TRANSPORT TO THE GOLGI AND SUBSEQUENT MODIFICATION,VESICLE MEDIATED TRANSPORT
COG1	9	ASPARAGINE N LINKED GLYCOSYLATION,COPI MEDIATED ANTEROGRADE TRANSPORT,ER TO GOLGI ANTEROGRADE TRANSPORT,INTRA GOLGI AND RETROGRADE GOLGI TO ER TRAFFIC,INTRA GOLGI TRAFFIC,POST TRANSLATIONAL PROTEIN MODIFICATION,RETROGRADE TRANSPORT AT THE TRANS GOLGI NETWORK,TRANSPORT TO THE GOLGI AND SUBSEQUENT MODIFICATION,VESICLE MEDIATED TRANSPORT
COG2	9	ASPARAGINE N LINKED GLYCOSYLATION,COPI MEDIATED ANTEROGRADE TRANSPORT,ER TO GOLGI ANTEROGRADE TRANSPORT,INTRA GOLGI AND RETROGRADE GOLGI TO ER TRAFFIC,INTRA GOLGI TRAFFIC,POST TRANSLATIONAL PROTEIN MODIFICATION,RETROGRADE TRANSPORT AT THE TRANS GOLGI NETWORK,TRANSPORT TO THE GOLGI AND SUBSEQUENT MODIFICATION,VESICLE MEDIATED TRANSPORT
COG3	9	ASPARAGINE N LINKED GLYCOSYLATION,COPI MEDIATED ANTEROGRADE TRANSPORT,ER TO GOLGI ANTEROGRADE TRANSPORT,INTRA GOLGI AND RETROGRADE GOLGI TO ER TRAFFIC,INTRA GOLGI TRAFFIC,POST TRANSLATIONAL PROTEIN MODIFICATION,RETROGRADE TRANSPORT AT THE TRANS GOLGI NETWORK,TRANSPORT TO THE GOLGI AND SUBSEQUENT MODIFICATION,VESICLE MEDIATED TRANSPORT
COG4	9	ASPARAGINE N LINKED GLYCOSYLATION,COPI MEDIATED ANTEROGRADE TRANSPORT,ER TO GOLGI ANTEROGRADE TRANSPORT,INTRA GOLGI AND RETROGRADE GOLGI TO ER TRAFFIC,INTRA GOLGI TRAFFIC,POST TRANSLATIONAL PROTEIN MODIFICATION,RETROGRADE TRANSPORT AT THE TRANS GOLGI NETWORK,TRANSPORT TO THE GOLGI AND SUBSEQUENT MODIFICATION,VESICLE MEDIATED TRANSPORT
COG5	9	ASPARAGINE N LINKED GLYCOSYLATION,COPI MEDIATED ANTEROGRADE TRANSPORT,ER TO GOLGI ANTEROGRADE TRANSPORT,INTRA GOLGI AND RETROGRADE GOLGI TO ER TRAFFIC,INTRA GOLGI TRAFFIC,POST TRANSLATIONAL PROTEIN MODIFICATION,RETROGRADE TRANSPORT AT THE TRANS GOLGI NETWORK,TRANSPORT TO THE GOLGI AND SUBSEQUENT MODIFICATION,VESICLE MEDIATED TRANSPORT
COG6	9	ASPARAGINE N LINKED GLYCOSYLATION,COPI MEDIATED ANTEROGRADE TRANSPORT,ER TO GOLGI ANTEROGRADE TRANSPORT,INTRA GOLGI AND RETROGRADE GOLGI TO ER TRAFFIC,INTRA GOLGI TRAFFIC,POST TRANSLATIONAL PROTEIN MODIFICATION,RETROGRADE TRANSPORT AT THE TRANS GOLGI NETWORK,TRANSPORT TO THE GOLGI AND SUBSEQUENT MODIFICATION,VESICLE MEDIATED TRANSPORT
COG7	9	ASPARAGINE N LINKED GLYCOSYLATION,COPI MEDIATED ANTEROGRADE TRANSPORT,ER TO GOLGI ANTEROGRADE TRANSPORT,INTRA GOLGI AND RETROGRADE GOLGI TO ER TRAFFIC,INTRA GOLGI TRAFFIC,POST TRANSLATIONAL PROTEIN MODIFICATION,RETROGRADE TRANSPORT AT THE TRANS GOLGI NETWORK,TRANSPORT TO THE GOLGI AND SUBSEQUENT MODIFICATION,VESICLE MEDIATED TRANSPORT
COG8	9	ASPARAGINE N LINKED GLYCOSYLATION,COPI MEDIATED ANTEROGRADE TRANSPORT,ER TO GOLGI ANTEROGRADE TRANSPORT,INTRA GOLGI AND RETROGRADE GOLGI TO ER TRAFFIC,INTRA GOLGI TRAFFIC,POST TRANSLATIONAL PROTEIN MODIFICATION,RETROGRADE TRANSPORT AT THE TRANS GOLGI NETWORK,TRANSPORT TO THE GOLGI AND SUBSEQUENT MODIFICATION,VESICLE MEDIATED TRANSPORT
COPB1	10	ASPARAGINE N LINKED GLYCOSYLATION,COPI DEPENDENT GOLGI TO ER RETROGRADE TRAFFIC,COPI MEDIATED ANTEROGRADE TRANSPORT,ER TO GOLGI ANTEROGRADE TRANSPORT,INNATE IMMUNE SYSTEM, INTRA GOLGI AND RETROGRADE GOLGI TO ER TRAFFIC,NEUTROPHIL DEGRANULATION,POST TRANSLATIONAL PROTEIN MODIFICATION,TRANSPORT TO THE GOLGI AND SUBSEQUENT MODIFICATION,VESICLE MEDIATED TRANSPORT
CTSC	10	LYSOSOME,ADAPTIVE IMMUNE SYSTEM,ASPARAGINE N LINKED GLYCOSYLATION,COPII MEDIATED VESICLE TRANSPORT,ER TO GOLGI ANTEROGRADE TRANSPORT,INNATE IMMUNE SYSTEM,NEUTROPHIL DEGRANULATION,POST TRANSLATIONAL PROTEIN MODIFICATION,TRANSPORT TO THE GOLGI AND SUBSEQUENT MODIFICATION,VESICLE MEDIATED TRANSPORT
CTSZ	10	LYSOSOME,ASPARAGINE N LINKED GLYCOSYLATION,COPII MEDIATED VESICLE TRANSPORT,ER TO GOLGI ANTEROGRADE TRANSPORT,INNATE IMMUNE SYSTEM,NEUTROPHIL DEGRANULATION,POST TRANSLATIONAL PROTEIN MODIFICATION,TRANSPORT TO THE GOLGI AND SUBSEQUENT MODIFICATION,TRANS GOLGI NETWORK VESICLE BUDDING,VESICLE MEDIATED TRANSPORT
DCTN1	9	VASOPRESSIN REGULATED WATER REABSORPTION,ADAPTIVE IMMUNE SYSTEM,ASPARAGINE N LINKED GLYCOSYLATION,COPI MEDIATED ANTEROGRADE TRANSPORT,ER TO GOLGI ANTEROGRADE TRANSPORT,INTRA GOLGI AND RETROGRADE GOLGI TO ER TRAFFIC,POST TRANSLATIONAL PROTEIN MODIFICATION,TRANSPORT TO THE GOLGI AND SUBSEQUENT MODIFICATION,VESICLE MEDIATED TRANSPORT
DCTN2	9	VASOPRESSIN REGULATED WATER REABSORPTION,ADAPTIVE IMMUNE SYSTEM,ASPARAGINE N LINKED GLYCOSYLATION,COPI MEDIATED ANTEROGRADE TRANSPORT,ER TO GOLGI ANTEROGRADE TRANSPORT,INTRA GOLGI AND RETROGRADE GOLGI TO ER TRAFFIC,POST TRANSLATIONAL PROTEIN MODIFICATION,TRANSPORT TO THE GOLGI AND SUBSEQUENT MODIFICATION,VESICLE MEDIATED TRANSPORT
DCTN4	9	VASOPRESSIN REGULATED WATER REABSORPTION,ADAPTIVE IMMUNE SYSTEM,ASPARAGINE N LINKED GLYCOSYLATION,COPI MEDIATED ANTEROGRADE TRANSPORT,ER TO GOLGI ANTEROGRADE TRANSPORT,INTRA GOLGI AND RETROGRADE GOLGI TO ER TRAFFIC,POST TRANSLATIONAL PROTEIN MODIFICATION,TRANSPORT TO THE GOLGI AND SUBSEQUENT MODIFICATION,VESICLE MEDIATED TRANSPORT
DCTN5	9	VASOPRESSIN REGULATED WATER REABSORPTION,ADAPTIVE IMMUNE SYSTEM,ASPARAGINE N LINKED GLYCOSYLATION,COPI MEDIATED ANTEROGRADE TRANSPORT,ER TO GOLGI ANTEROGRADE TRANSPORT,INTRA GOLGI AND RETROGRADE GOLGI TO ER TRAFFIC,POST TRANSLATIONAL PROTEIN MODIFICATION,TRANSPORT TO THE GOLGI AND SUBSEQUENT MODIFICATION,VESICLE MEDIATED TRANSPORT
DCTN6	9	VASOPRESSIN REGULATED WATER REABSORPTION,ADAPTIVE IMMUNE SYSTEM,ASPARAGINE N LINKED GLYCOSYLATION,COPI MEDIATED ANTEROGRADE TRANSPORT,ER TO GOLGI ANTEROGRADE TRANSPORT,INTRA GOLGI AND RETROGRADE GOLGI TO ER TRAFFIC,POST TRANSLATIONAL PROTEIN MODIFICATION,TRANSPORT TO THE GOLGI AND SUBSEQUENT MODIFICATION,VESICLE MEDIATED TRANSPORT
DYNC1H1	11	VASOPRESSIN REGULATED WATER REABSORPTION,ADAPTIVE IMMUNE SYSTEM,ASPARAGINE N LINKED GLYCOSYLATION,COPI MEDIATED ANTEROGRADE TRANSPORT,ER TO GOLGI ANTEROGRADE TRANSPORT,INNATE IMMUNE SYSTEM, INTRA GOLGI AND RETROGRADE GOLGI TO ER TRAFFIC,NEUTROPHIL DEGRANULATION,POST TRANSLATIONAL PROTEIN MODIFICATION,TRANSPORT TO THE GOLGI AND SUBSEQUENT MODIFICATION,VESICLE MEDIATED TRANSPORT
DYNC1I1	9	VASOPRESSIN REGULATED WATER REABSORPTION,ADAPTIVE IMMUNE SYSTEM,ASPARAGINE N LINKED GLYCOSYLATION,COPI MEDIATED ANTEROGRADE TRANSPORT,ER TO GOLGI ANTEROGRADE TRANSPORT,INTRA GOLGI AND RETROGRADE GOLGI TO ER TRAFFIC,POST TRANSLATIONAL PROTEIN MODIFICATION,TRANSPORT TO THE GOLGI AND SUBSEQUENT MODIFICATION,VESICLE MEDIATED TRANSPORT
DYNC1I2	9	VASOPRESSIN REGULATED WATER REABSORPTION,ADAPTIVE IMMUNE SYSTEM,ASPARAGINE N LINKED GLYCOSYLATION,COPI MEDIATED ANTEROGRADE TRANSPORT,ER TO GOLGI ANTEROGRADE TRANSPORT,INTRA GOLGI AND RETROGRADE GOLGI TO ER TRAFFIC,POST TRANSLATIONAL PROTEIN MODIFICATION,TRANSPORT TO THE GOLGI AND SUBSEQUENT MODIFICATION,VESICLE MEDIATED TRANSPORT
DYNC1LI1	11	VASOPRESSIN REGULATED WATER REABSORPTION,ADAPTIVE IMMUNE SYSTEM,ASPARAGINE N LINKED GLYCOSYLATION,COPI MEDIATED ANTEROGRADE TRANSPORT,ER TO GOLGI ANTEROGRADE TRANSPORT,INNATE IMMUNE SYSTEM,INTRA GOLGI AND RETROGRADE GOLGI TO ER TRAFFIC,NEUTROPHIL DEGRANULATION, POST TRANSLATIONAL PROTEIN MODIFICATION,TRANSPORT TO THE GOLGI AND SUBSEQUENT MODIFICATION,VESICLE MEDIATED TRANSPORT
DYNC1LI2	9	VASOPRESSIN REGULATED WATER REABSORPTION,ADAPTIVE IMMUNE SYSTEM,ASPARAGINE N LINKED GLYCOSYLATION,COPI MEDIATED ANTEROGRADE TRANSPORT,ER TO GOLGI ANTEROGRADE TRANSPORT,INTRA GOLGI AND RETROGRADE GOLGI TO ER TRAFFIC,POST TRANSLATIONAL PROTEIN MODIFICATION,TRANSPORT TO THE GOLGI AND SUBSEQUENT MODIFICATION,VESICLE MEDIATED TRANSPORT
DYNLL1	11	VASOPRESSIN REGULATED WATER REABSORPTION,ADAPTIVE IMMUNE SYSTEM,ASPARAGINE N LINKED GLYCOSYLATION,COPI MEDIATED ANTEROGRADE TRANSPORT,ER TO GOLGI ANTEROGRADE TRANSPORT, INNATE IMMUNE SYSTEM,INTRA GOLGI AND RETROGRADE GOLGI TO ER TRAFFIC,NEUTROPHIL DEGRANULATION,POST TRANSLATIONAL PROTEIN MODIFICATION,TRANSPORT TO THE GOLGI AND SUBSEQUENT MODIFICATION,VESICLE MEDIATED TRANSPORT
GBF1	9	ASPARAGINE N LINKED GLYCOSYLATION,COPI DEPENDENT GOLGI TO ER RETROGRADE TRAFFIC,COPI MEDIATED ANTEROGRADE TRANSPORT,ER TO GOLGI ANTEROGRADE TRANSPORT,INTRA GOLGI AND RETROGRADE GOLGI TO ER TRAFFIC,POST TRANSLATIONAL PROTEIN MODIFICATION,TRANSPORT TO THE GOLGI AND SUBSEQUENT MODIFICATION,TRANS GOLGI NETWORK VESICLE BUDDING, VESICLE MEDIATED TRANSPORT
GOSR1	9	ASPARAGINE N LINKED GLYCOSYLATION,COPI MEDIATED ANTEROGRADE TRANSPORT,ER TO GOLGI ANTEROGRADE TRANSPORT,INTRA GOLGI AND RETROGRADE GOLGI TO ER TRAFFIC,INTRA GOLGI TRAFFIC,POST TRANSLATIONAL PROTEIN MODIFICATION,TRANSPORT TO THE GOLGI AND SUBSEQUENT MODIFICATION,VESICLE MEDIATED TRANSPORT
GOSR2	10	ASPARAGINE N LINKED GLYCOSYLATION,COPII MEDIATED VESICLE TRANSPORT,COPI MEDIATED ANTEROGRADE TRANSPORT,ER TO GOLGI ANTEROGRADE TRANSPORT,INTRA GOLGI AND RETROGRADE GOLGI TO ER TRAFFIC,INTRA GOLGI TRAFFIC,POST TRANSLATIONAL PROTEIN MODIFICATION,TRANSPORT TO THE GOLGI AND SUBSEQUENT MODIFICATION,VESICLE MEDIATED TRANSPORT
GRIA1	13	LONG TERM DEPRESSION,NEUROACTIVE LIGAND RECEPTOR INTERACTION,ASPARAGINE N LINKED GLYCOSYLATION,COPII MEDIATED VESICLE TRANSPORT,ER TO GOLGI ANTEROGRADE TRANSPORT,GLUTAMATE BINDING ACTIVATION OF AMPA RECEPTORS AND SYNAPTIC PLASTICITY,NEURONAL SYSTEM,NEUROTRANSMITTER RECEPTORS AND POSTSYNAPTIC SIGNAL TRANSMISSION, POST TRANSLATIONAL PROTEIN MODIFICATION,TRAFFICKING OF GLUR2 CONTAINING AMPA RECEPTORS,TRANSMISSION ACROSS CHEMICAL SYNAPSES,TRANSPORT TO THE GOLGI AND SUBSEQUENT MODIFICATION,VESICLE MEDIATED TRANSPORT
HSPA8	10	MAPK SIGNALING PATHWAY,CLATHRIN MEDIATED ENDOCYTOSIS,GOLGI ASSOCIATED VESICLE BIOGENESIS,INNATE IMMUNE SYSTEM,NEURONAL SYSTEM,NEUTROPHIL DEGRANULATION,POST TRANSLATIONAL PROTEIN MODIFICATION,TRANSMISSION ACROSS CHEMICAL SYNAPSES,TRANS GOLGI NETWORK VESICLE BUDDING,VESICLE MEDIATED TRANSPORT
IGF2R	9	LYSOSOME,CLATHRIN MEDIATED ENDOCYTOSIS,GOLGI ASSOCIATED VESICLE BIOGENESIS,INNATE IMMUNE SYSTEM,INTRA GOLGI AND RETROGRADE GOLGI TO ER TRAFFIC,NEUTROPHIL DEGRANULATION,RETROGRADE TRANSPORT AT THE TRANS GOLGI NETWORK,TRANS GOLGI NETWORK VESICLE BUDDING,VESICLE MEDIATED TRANSPORT
MAPK1	10	ALZHEIMERS DISEASE,LONG TERM DEPRESSION,MAPK SIGNALING PATHWAY,ALPHA SYNUCLEIN PATHWAY,INNATE IMMUNE SYSTEM,NEURONAL SYSTEM,NEUROTRANSMITTER RECEPTORS AND POSTSYNAPTIC SIGNAL TRANSMISSION,NEUTROPHIL DEGRANULATION,SIGNALING BY GPCR,TRANSMISSION ACROSS CHEMICAL SYNAPSES
MAPK3	9	ALZHEIMERS DISEASE,LONG TERM DEPRESSION,MAPK SIGNALING PATHWAY,ALPHA SYNUCLEIN PATHWAY,INNATE IMMUNE SYSTEM,NEURONAL SYSTEM,NEUROTRANSMITTER RECEPTORS AND POSTSYNAPTIC SIGNAL TRANSMISSION,SIGNALING BY GPCR,TRANSMISSION ACROSS CHEMICAL SYNAPSES
MAPT	11	ALZHEIMERS DISEASE,MAPK SIGNALING PATHWAY,LKB1 PATHWAY,P38 GAMMA DELTA PATHWAY,APOPTOTIC CLEAVAGE OF CELLULAR PROTEINS,APOPTOTIC EXECUTION PHASE,CASPASE MEDIATED CLEAVAGE OF CYTOSKELETAL PROTEINS,NEURONAL SYSTEM,NEUROTRANSMITTER RECEPTORS AND POSTSYNAPTIC SIGNAL TRANSMISSION,TRANSMISSION ACROSS CHEMICAL SYNAPSES
NAPA	13	ASPARAGINE N LINKED GLYCOSYLATION,COPII MEDIATED VESICLE TRANSPORT,COPI DEPENDENT GOLGI TO ER RETROGRADE TRAFFIC,COPI MEDIATED ANTEROGRADE TRANSPORT,ER TO GOLGI ANTEROGRADE TRANSPORT,GOLGI ASSOCIATED VESICLE BIOGENESIS,INTRA GOLGI AND RETROGRADE GOLGI TO ER TRAFFIC,INTRA GOLGI TRAFFIC, POST TRANSLATIONAL PROTEIN MODIFICATION,RETROGRADE TRANSPORT AT THE TRANS GOLGI NETWORK, TRANSPORT TO THE GOLGI AND SUBSEQUENT MODIFICATION,TRANS GOLGI NETWORK VESICLE BUDDING,VESICLE MEDIATED TRANSPORT
NAPB	11	ASPARAGINE N LINKED GLYCOSYLATION,COPII MEDIATED VESICLE TRANSPORT,COPI DEPENDENT GOLGI TO ER RETROGRADE TRAFFIC,COPI MEDIATED ANTEROGRADE TRANSPORT,ER TO GOLGI ANTEROGRADE TRANSPORT,INTRA GOLGI AND RETROGRADE GOLGI TO ER TRAFFIC,INTRA GOLGI TRAFFIC,POST TRANSLATIONAL PROTEIN MODIFICATION, RETROGRADE TRANSPORT AT THE TRANS GOLGI NETWORK,TRANSPORT TO THE GOLGI AND SUBSEQUENT MODIFICATION,VESICLE MEDIATED TRANSPORT
NAPG	11	ASPARAGINE N LINKED GLYCOSYLATION,COPII MEDIATED VESICLE TRANSPORT,COPI DEPENDENT GOLGI TO ER RETROGRADE TRAFFIC,COPI MEDIATED ANTEROGRADE TRANSPORT,ER TO GOLGI ANTEROGRADE TRANSPORT,INTRA GOLGI AND RETROGRADE GOLGI TO ER TRAFFIC,INTRA GOLGI TRAFFIC,POST TRANSLATIONAL PROTEIN MODIFICATION, RETROGRADE TRANSPORT AT THE TRANS GOLGI NETWORK,TRANSPORT TO THE GOLGI AND SUBSEQUENT MODIFICATION,VESICLE MEDIATED TRANSPORT
NRAS	9	LONG TERM DEPRESSION,MAPK SIGNALING PATHWAY,ADAPTIVE IMMUNE SYSTEM,INNATE IMMUNE SYSTEM,NEURONAL SYSTEM,NEUROTRANSMITTER RECEPTORS AND POSTSYNAPTIC SIGNAL TRANSMISSION,NEUTROPHIL DEGRANULATION,SIGNALING BY GPCR,TRANSMISSION ACROSS CHEMICAL SYNAPSES,,
NSF	17	VASOPRESSIN REGULATED WATER REABSORPTION,ASPARAGINE N LINKED GLYCOSYLATION,COPII MEDIATED VESICLE TRANSPORT,COPI DEPENDENT GOLGI TO ER RETROGRADE TRAFFIC,COPI MEDIATED ANTEROGRADE TRANSPORT,ER TO GOLGI ANTEROGRADE TRANSPORT, TRANSMISSION,POST TRANSLATIONAL PROTEIN MODIFICATION,RETROGRADE TRANSPORT AT THE TRANS GOLGI NETWORK, TRAFFICKING OF GLUR2 CONTAINING AMPA RECEPTORS,TRANSMISSION ACROSS CHEMICAL SYNAPSES,TRANSPORT TO THE GOLGI AND SUBSEQUENT MODIFICATION,REACTOME_VESICLE_MEDIATED_TRANSPORT, GLUTAMATE BINDING ACTIVATION OF AMPA RECEPTORS AND SYNAPTIC PLASTICITY,INTRA GOLGI AND RETROGRADE GOLGI TO ER TRAFFIC,INTRA GOLGI TRAFFIC,NEURONAL SYSTEM, NEUROTRANSMITTER RECEPTORS AND POSTSYNAPTIC SIGNAL
PRKACA	10	MAPK SIGNALING PATHWAY,VASOPRESSIN REGULATED WATER REABSORPTION,LKB1 PATHWAY,ADAPTIVE IMMUNE SYSTEM,INNATE IMMUNE SYSTEM,METABOLISM OF LIPIDS,NEURONAL SYSTEM, NEUROTRANSMITTER RECEPTORS AND POSTSYNAPTIC SIGNAL TRANSMISSION,SIGNALING BY GPCR,TRANSMISSION ACROSS CHEMICAL SYNAPSES
PRKACB	9	MAPK SIGNALING PATHWAY,VASOPRESSIN REGULATED WATER REABSORPTION,ADAPTIVE IMMUNE SYSTEM,INNATE IMMUNE SYSTEM,METABOLISM OF LIPIDS,NEURONAL SYSTEM,NEUROTRANSMITTER RECEPTORS AND POSTSYNAPTIC SIGNAL TRANSMISSION,SIGNALING BY GPCR,TRANSMISSION ACROSS CHEMICAL SYNAPSES
PRKACG	9	MAPK SIGNALING PATHWAY,VASOPRESSIN REGULATED WATER REABSORPTION,ADAPTIVE IMMUNE SYSTEM,INNATE IMMUNE SYSTEM,METABOLISM OF LIPIDS,NEURONAL SYSTEM,NEUROTRANSMITTER RECEPTORS AND POSTSYNAPTIC SIGNAL TRANSMISSION,SIGNALING BY GPCR,TRANSMISSION ACROSS CHEMICAL SYNAPSES
PRKCB	9	LONG TERM DEPRESSION,MAPK SIGNALING PATHWAY,ADAPTIVE IMMUNE SYSTEM,GLUTAMATE BINDING ACTIVATION OF AMPA RECEPTORS AND SYNAPTIC PLASTICITY,NEURONAL SYSTEM,NEUROTRANSMITTER RECEPTORS AND POSTSYNAPTIC SIGNAL TRANSMISSION,SIGNALING BY GPCR,TRAFFICKING OF GLUR2 CONTAINING AMPA RECEPTORS,TRANSMISSION ACROSS CHEMICAL SYNAPSES
RAB1A	9	ASPARAGINE N LINKED GLYCOSYLATION,COPII MEDIATED VESICLE TRANSPORT,COPI DEPENDENT GOLGI TO ER RETROGRADE TRAFFIC,COPI MEDIATED ANTEROGRADE TRANSPORT,ER TO GOLGI ANTEROGRADE TRANSPORT,INTRA GOLGI AND RETROGRADE GOLGI TO ER TRAFFIC,POST TRANSLATIONAL PROTEIN MODIFICATION,TRANSPORT TO THE GOLGI AND SUBSEQUENT MODIFICATION,VESICLE MEDIATED TRANSPORT
RAB1B	9	ASPARAGINE N LINKED GLYCOSYLATION,COPII MEDIATED VESICLE TRANSPORT,COPI DEPENDENT GOLGI TO ER RETROGRADE TRAFFIC,COPI MEDIATED ANTEROGRADE TRANSPORT,ER TO GOLGI ANTEROGRADE TRANSPORT,INTRA GOLGI AND RETROGRADE GOLGI TO ER TRAFFIC,POST TRANSLATIONAL PROTEIN MODIFICATION,TRANSPORT TO THE GOLGI AND SUBSEQUENT MODIFICATION,VESICLE MEDIATED TRANSPORT
SPTAN1	10	APOPTOTIC CLEAVAGE OF CELLULAR PROTEINS,APOPTOTIC EXECUTION PHASE,ASPARAGINE N LINKED GLYCOSYLATION,CASPASE MEDIATED CLEAVAGE OF CYTOSKELETAL PROTEINS,COPI MEDIATED ANTEROGRADE TRANSPORT,ER TO GOLGI ANTEROGRADE TRANSPORT,INNATE IMMUNE SYSTEM,NEUTROPHIL DEGRANULATION,POST TRANSLATIONAL PROTEIN MODIFICATION,TRANSPORT TO THE GOLGI AND SUBSEQUENT MODIFICATION,VESICLE MEDIATED TRANSPORT
STX5	10	ASPARAGINE N LINKED GLYCOSYLATION,COPII MEDIATED VESICLE TRANSPORT,COPI MEDIATED ANTEROGRADE TRANSPORT,ER TO GOLGI ANTEROGRADE TRANSPORT,INTRA GOLGI AND RETROGRADE GOLGI TO ER TRAFFIC,INTRA GOLGI TRAFFIC,POST TRANSLATIONAL PROTEIN MODIFICATION,TRANSPORT TO THE GOLGI AND SUBSEQUENT MODIFICATION,VESICLE MEDIATED TRANSPORT
TMED10	9	ASPARAGINE N LINKED GLYCOSYLATION,COPII MEDIATED VESICLE TRANSPORT,COPI DEPENDENT GOLGI TO ER RETROGRADE TRAFFIC,COPI MEDIATED ANTEROGRADE TRANSPORT,ER TO GOLGI ANTEROGRADE TRANSPORT,INTRA GOLGI AND RETROGRADE GOLGI TO ER TRAFFIC,POST TRANSLATIONAL PROTEIN MODIFICATION,TRANSPORT TO THE GOLGI AND SUBSEQUENT MODIFICATION,VESICLE MEDIATED TRANSPORT
TMED2	9	ASPARAGINE N LINKED GLYCOSYLATION,COPII MEDIATED VESICLE TRANSPORT,COPI DEPENDENT GOLGI TO ER RETROGRADE TRAFFIC,COPI MEDIATED ANTEROGRADE TRANSPORT,ER TO GOLGI ANTEROGRADE TRANSPORT,INTRA GOLGI AND RETROGRADE GOLGI TO ER TRAFFIC,POST TRANSLATIONAL PROTEIN MODIFICATION,TRANSPORT TO THE GOLGI AND SUBSEQUENT MODIFICATION,VESICLE MEDIATED TRANSPORT
TUBA1A	13	ADAPTIVE IMMUNE SYSTEM,ASPARAGINE N LINKED GLYCOSYLATION,COPI DEPENDENT GOLGI TO ER RETROGRADE TRAFFIC,COPI MEDIATED ANTEROGRADE TRANSPORT,ER TO GOLGI ANTEROGRADE TRANSPORT,INTRA GOLGI AND RETROGRADE GOLGI TO ER TRAFFIC,NEURONAL SYSTEM, NEUROTRANSMITTER RECEPTORS AND POSTSYNAPTIC SIGNAL TRANSMISSION,POST TRANSLATIONAL PROTEIN MODIFICATION,TRANSMISSION ACROSS CHEMICAL SYNAPSES,TRANSPORT TO THE GOLGI AND SUBSEQUENT MODIFICATION,VESICLE MEDIATED TRANSPORT
TUBA1B	13	ADAPTIVE IMMUNE SYSTEM,ASPARAGINE N LINKED GLYCOSYLATION,COPI DEPENDENT GOLGI TO ER RETROGRADE TRAFFIC,COPI MEDIATED ANTEROGRADE TRANSPORT,ER TO GOLGI ANTEROGRADE TRANSPORT,INTRA GOLGI AND RETROGRADE GOLGI TO ER TRAFFIC,NEURONAL SYSTEM, NEUROTRANSMITTER RECEPTORS AND POSTSYNAPTIC SIGNAL TRANSMISSION,POST TRANSLATIONAL PROTEIN MODIFICATION,TRANSMISSION ACROSS CHEMICAL SYNAPSES,TRANSPORT TO THE GOLGI AND SUBSEQUENT MODIFICATION,VESICLE MEDIATED TRANSPORT
TUBA1C	13	ADAPTIVE IMMUNE SYSTEM,ASPARAGINE N LINKED GLYCOSYLATION,COPI DEPENDENT GOLGI TO ER RETROGRADE TRAFFIC,COPI MEDIATED ANTEROGRADE TRANSPORT,ER TO GOLGI ANTEROGRADE TRANSPORT,INTRA GOLGI AND RETROGRADE GOLGI TO ER TRAFFIC,NEURONAL SYSTEM, NEUROTRANSMITTER RECEPTORS AND POSTSYNAPTIC SIGNAL TRANSMISSION,POST TRANSLATIONAL PROTEIN MODIFICATION,TRANSMISSION ACROSS CHEMICAL SYNAPSES,TRANSPORT TO THE GOLGI AND SUBSEQUENT MODIFICATION,VESICLE MEDIATED TRANSPORT
TUBA3C	13	ADAPTIVE IMMUNE SYSTEM,ASPARAGINE N LINKED GLYCOSYLATION,COPI DEPENDENT GOLGI TO ER RETROGRADE TRAFFIC,COPI MEDIATED ANTEROGRADE TRANSPORT,ER TO GOLGI ANTEROGRADE TRANSPORT,INTRA GOLGI AND RETROGRADE GOLGI TO ER TRAFFIC,NEURONAL SYSTEM, NEUROTRANSMITTER RECEPTORS AND POSTSYNAPTIC SIGNAL TRANSMISSION,POST TRANSLATIONAL PROTEIN MODIFICATION,TRANSMISSION ACROSS CHEMICAL SYNAPSES,TRANSPORT TO THE GOLGI AND SUBSEQUENT MODIFICATION,VESICLE MEDIATED TRANSPORT
TUBA3D	13	ADAPTIVE IMMUNE SYSTEM,ASPARAGINE N LINKED GLYCOSYLATION,COPI DEPENDENT GOLGI TO ER RETROGRADE TRAFFIC,COPI MEDIATED ANTEROGRADE TRANSPORT,ER TO GOLGI ANTEROGRADE TRANSPORT,INTRA GOLGI AND RETROGRADE GOLGI TO ER TRAFFIC,NEURONAL SYSTEM, NEUROTRANSMITTER RECEPTORS AND POSTSYNAPTIC SIGNAL TRANSMISSION,POST TRANSLATIONAL PROTEIN MODIFICATION,TRANSMISSION ACROSS CHEMICAL SYNAPSES,TRANSPORT TO THE GOLGI AND SUBSEQUENT MODIFICATION,VESICLE MEDIATED TRANSPORT
TUBA3E	13	ADAPTIVE IMMUNE SYSTEM,ASPARAGINE N LINKED GLYCOSYLATION,COPI DEPENDENT GOLGI TO ER RETROGRADE TRAFFIC,COPI MEDIATED ANTEROGRADE TRANSPORT,ER TO GOLGI ANTEROGRADE TRANSPORT,INTRA GOLGI AND RETROGRADE GOLGI TO ER TRAFFIC,NEURONAL SYSTEM, NEUROTRANSMITTER RECEPTORS AND POSTSYNAPTIC SIGNAL TRANSMISSION,POST TRANSLATIONAL PROTEIN MODIFICATION,TRANSMISSION ACROSS CHEMICAL SYNAPSES,TRANSPORT TO THE GOLGI AND SUBSEQUENT MODIFICATION,VESICLE MEDIATED TRANSPORT
TUBA4A	13	ADAPTIVE IMMUNE SYSTEM,ASPARAGINE N LINKED GLYCOSYLATION,COPI DEPENDENT GOLGI TO ER RETROGRADE TRAFFIC,COPI MEDIATED ANTEROGRADE TRANSPORT,ER TO GOLGI ANTEROGRADE TRANSPORT,INTRA GOLGI AND RETROGRADE GOLGI TO ER TRAFFIC,NEURONAL SYSTEM, NEUROTRANSMITTER RECEPTORS AND POSTSYNAPTIC SIGNAL TRANSMISSION,POST TRANSLATIONAL PROTEIN MODIFICATION,TRANSMISSION ACROSS CHEMICAL SYNAPSES,TRANSPORT TO THE GOLGI AND SUBSEQUENT MODIFICATION,VESICLE MEDIATED TRANSPORT
TUBA4B	13	ADAPTIVE IMMUNE SYSTEM,ASPARAGINE N LINKED GLYCOSYLATION,COPI DEPENDENT GOLGI TO ER RETROGRADE TRAFFIC,COPI MEDIATED ANTEROGRADE TRANSPORT,ER TO GOLGI ANTEROGRADE TRANSPORT,INTRA GOLGI AND RETROGRADE GOLGI TO ER TRAFFIC,NEURONAL SYSTEM ,NEUROTRANSMITTER RECEPTORS AND POSTSYNAPTIC SIGNAL TRANSMISSION,POST TRANSLATIONAL PROTEIN MODIFICATION,TRANSMISSION ACROSS CHEMICAL SYNAPSES,TRANSPORT TO THE GOLGI AND SUBSEQUENT MODIFICATION,VESICLE MEDIATED TRANSPORT
TUBA8	13	ADAPTIVE IMMUNE SYSTEM,ASPARAGINE N LINKED GLYCOSYLATION,COPI DEPENDENT GOLGI TO ER RETROGRADE TRAFFIC,COPI MEDIATED ANTEROGRADE TRANSPORT,ER TO GOLGI ANTEROGRADE TRANSPORT,INTRA GOLGI AND RETROGRADE GOLGI TO ER TRAFFIC,NEURONAL SYSTEM, NEUROTRANSMITTER RECEPTORS AND POSTSYNAPTIC SIGNAL TRANSMISSION,POST TRANSLATIONAL PROTEIN MODIFICATION,TRANSMISSION ACROSS CHEMICAL SYNAPSES,TRANSPORT TO THE GOLGI AND SUBSEQUENT MODIFICATION,VESICLE MEDIATED TRANSPORT
TUBAL3	13	ADAPTIVE IMMUNE SYSTEM,ASPARAGINE N LINKED GLYCOSYLATION,COPI DEPENDENT GOLGI TO ER RETROGRADE TRAFFIC,COPI MEDIATED ANTEROGRADE TRANSPORT,ER TO GOLGI ANTEROGRADE TRANSPORT,INTRA GOLGI AND RETROGRADE GOLGI TO ER TRAFFIC,NEURONAL SYSTEM, NEUROTRANSMITTER RECEPTORS AND POSTSYNAPTIC SIGNAL TRANSMISSION,POST TRANSLATIONAL PROTEIN MODIFICATION,TRANSMISSION ACROSS CHEMICAL SYNAPSES,TRANSPORT TO THE GOLGI AND SUBSEQUENT MODIFICATION,VESICLE MEDIATED TRANSPORT
TUBB1	13	ADAPTIVE IMMUNE SYSTEM,ASPARAGINE N LINKED GLYCOSYLATION,COPI DEPENDENT GOLGI TO ER RETROGRADE TRAFFIC,COPI MEDIATED ANTEROGRADE TRANSPORT,ER TO GOLGI ANTEROGRADE TRANSPORT,INTRA GOLGI AND RETROGRADE GOLGI TO ER TRAFFIC,NEURONAL SYSTEM, NEUROTRANSMITTER RECEPTORS AND POSTSYNAPTIC SIGNAL TRANSMISSION,POST TRANSLATIONAL PROTEIN MODIFICATION,TRANSMISSION ACROSS CHEMICAL SYNAPSES,TRANSPORT TO THE GOLGI AND SUBSEQUENT MODIFICATION,VESICLE MEDIATED TRANSPORT
TUBB2A	13	ADAPTIVE IMMUNE SYSTEM,ASPARAGINE N LINKED GLYCOSYLATION,COPI DEPENDENT GOLGI TO ER RETROGRADE TRAFFIC,COPI MEDIATED ANTEROGRADE TRANSPORT,ER TO GOLGI ANTEROGRADE TRANSPORT,INTRA GOLGI AND RETROGRADE GOLGI TO ER TRAFFIC,NEURONAL SYSTEM, NEUROTRANSMITTER RECEPTORS AND POSTSYNAPTIC SIGNAL TRANSMISSION,POST TRANSLATIONAL PROTEIN MODIFICATION,TRANSMISSION ACROSS CHEMICAL SYNAPSES,TRANSPORT TO THE GOLGI AND SUBSEQUENT MODIFICATION,VESICLE MEDIATED TRANSPORT
TUBB2B	13	ADAPTIVE IMMUNE SYSTEM,ASPARAGINE N LINKED GLYCOSYLATION,COPI DEPENDENT GOLGI TO ER RETROGRADE TRAFFIC,COPI MEDIATED ANTEROGRADE TRANSPORT,ER TO GOLGI ANTEROGRADE TRANSPORT,INTRA GOLGI AND RETROGRADE GOLGI TO ER TRAFFIC,NEURONAL SYSTEM, NEUROTRANSMITTER RECEPTORS AND POSTSYNAPTIC SIGNAL TRANSMISSION,POST TRANSLATIONAL PROTEIN MODIFICATION,TRANSMISSION ACROSS CHEMICAL SYNAPSES,TRANSPORT TO THE GOLGI AND SUBSEQUENT MODIFICATION,VESICLE MEDIATED TRANSPORT
TUBB3	13	ADAPTIVE IMMUNE SYSTEM,ASPARAGINE N LINKED GLYCOSYLATION,COPI DEPENDENT GOLGI TO ER RETROGRADE TRAFFIC,COPI MEDIATED ANTEROGRADE TRANSPORT,ER TO GOLGI ANTEROGRADE TRANSPORT,INTRA GOLGI AND RETROGRADE GOLGI TO ER TRAFFIC,NEURONAL SYSTEM, NEUROTRANSMITTER RECEPTORS AND POSTSYNAPTIC SIGNAL TRANSMISSION,POST TRANSLATIONAL PROTEIN MODIFICATION,TRANSMISSION ACROSS CHEMICAL SYNAPSES,TRANSPORT TO THE GOLGI AND SUBSEQUENT MODIFICATION,VESICLE MEDIATED TRANSPORT
TUBB4A	13	ADAPTIVE IMMUNE SYSTEM,ASPARAGINE N LINKED GLYCOSYLATION,COPI DEPENDENT GOLGI TO ER RETROGRADE TRAFFIC,COPI MEDIATED ANTEROGRADE TRANSPORT,ER TO GOLGI ANTEROGRADE TRANSPORT,INTRA GOLGI AND RETROGRADE GOLGI TO ER TRAFFIC,NEURONAL SYSTEM, NEUROTRANSMITTER RECEPTORS AND POSTSYNAPTIC SIGNAL TRANSMISSION,POST TRANSLATIONAL PROTEIN MODIFICATION,TRANSMISSION ACROSS CHEMICAL SYNAPSES,TRANSPORT TO THE GOLGI AND SUBSEQUENT MODIFICATION,VESICLE MEDIATED TRANSPORT
TUBB4B	15	ADAPTIVE IMMUNE SYSTEM,ASPARAGINE N LINKED GLYCOSYLATION,COPI DEPENDENT GOLGI TO ER RETROGRADE TRAFFIC,COPI MEDIATED ANTEROGRADE TRANSPORT,ER TO GOLGI ANTEROGRADE TRANSPORT,INNATE IMMUNE SYSTEM,INTRA GOLGI AND RETROGRADE GOLGI TO ER TRAFFIC,NEURONAL SYSTEM, NEUROTRANSMITTER RECEPTORS AND POSTSYNAPTIC SIGNAL TRANSMISSION,NEUTROPHIL DEGRANULATION,POST TRANSLATIONAL PROTEIN MODIFICATION,TRANSMISSION ACROSS CHEMICAL SYNAPSES,TRANSPORT TO THE GOLGI AND SUBSEQUENT MODIFICATION,VESICLE MEDIATED TRANSPORT
TUBB6	13	ADAPTIVE IMMUNE SYSTEM,ASPARAGINE N LINKED GLYCOSYLATION,COPI DEPENDENT GOLGI TO ER RETROGRADE TRAFFIC,COPI MEDIATED ANTEROGRADE TRANSPORT,ER TO GOLGI ANTEROGRADE TRANSPORT,INTRA GOLGI AND RETROGRADE GOLGI TO ER TRAFFIC,NEURONAL SYSTEM, NEUROTRANSMITTER RECEPTORS AND POSTSYNAPTIC SIGNAL TRANSMISSION,POST TRANSLATIONAL PROTEIN MODIFICATION,TRANSMISSION ACROSS CHEMICAL SYNAPSES,TRANSPORT TO THE GOLGI AND SUBSEQUENT MODIFICATION,VESICLE MEDIATED TRANSPORT
TUBB8	13	ADAPTIVE IMMUNE SYSTEM,ASPARAGINE N LINKED GLYCOSYLATION,COPI DEPENDENT GOLGI TO ER RETROGRADE TRAFFIC,COPI MEDIATED ANTEROGRADE TRANSPORT,ER TO GOLGI ANTEROGRADE TRANSPORT,INTRA GOLGI AND RETROGRADE GOLGI TO ER TRAFFIC,NEURONAL SYSTEM, NEUROTRANSMITTER RECEPTORS AND POSTSYNAPTIC SIGNAL TRANSMISSION,POST TRANSLATIONAL PROTEIN MODIFICATION,TRANSMISSION ACROSS CHEMICAL SYNAPSES,TRANSPORT TO THE GOLGI AND SUBSEQUENT MODIFICATION,VESICLE MEDIATED TRANSPORT
YKT6	9	ASPARAGINE N LINKED GLYCOSYLATION,COPII MEDIATED VESICLE TRANSPORT,COPI MEDIATED ANTEROGRADE TRANSPORT,ER TO GOLGI ANTEROGRADE TRANSPORT,INTRA GOLGI AND RETROGRADE GOLGI TO ER TRAFFIC,INTRA GOLGI TRAFFIC,POST TRANSLATIONAL PROTEIN MODIFICATION,TRANSPORT TO THE GOLGI AND SUBSEQUENT MODIFICATION,VESICLE MEDIATED TRANSPORT

Supplementary Table 6. Canonical pathways significantly associated with PD risk through rare variation excluding PD known risk loci +/- 1Mb upstream and downstream

		MAF < 3		MAF < 1	
**Gene Set**	**Funtional subcategory**	**Num SNP**	**SKAT-O P**	**Num SNP**	**SKAT-O P**
ADAPTIVE IMMUNE SYSTEM (REACTOME)	**Missense**	3075	0.163	2871	0.123
	**Loss of function**	1559	0.010	1438	1.23E-02
	**CADD > 12 %**	86	0.847	80	1
INNATE IMMUNE SYSTEM (REACTOME)	**Missense**	6587	0.002	6123	6.72E-04
	**Loss of function**	2672	0.030	2445	0.107
	**CADD > 12 %**	169	0.899	160	0.807
METABOLISM OF LIPIDS (REACTOME)	**Missense**	3648	0.815	3398	0.399
	**Loss of function**	1709	0.743	1552	0.822
	**CADD > 12 %**	141	0.765	130	0.698
NEUTROPHIL DEGRANULATION (REACTOME)	**Missense**	2417	0.237	2243	0.080
	**Loss of function**	1049	0.454	944	1
	**CADD > 12 %**	91	0.730	84	0.790
SIGNALING BY GPCR (REACTOME)	**Missense**	5406	3.86E-04	4963	1.08E-06
	**Loss of function**	1467	0.360	1333	0.699
	**CADD > 12 %**	129	0.075	120	0.182
VESICLE MEDIATED TRANSPORT (REACTOME)	**Missense**	3300	0.909	3060	0.268
	**Loss of function**	1628	0.851	1468	0.060
	**CADD > 12 %**	81	1	77	1

Supplementary Table 7. Gene prioritization per functionality category based on Sequence Kernel Association Test analysis

a) MISSENSE VARIANTS

Gene	NumVar	Q	Pvalue	Gene-set
NDUFA10	3	3.11E+04	0.001	ALZHEIMERS DISEASE
TNFRSF1A	5	3.60E+04	0.003	ALZHEIMERS DISEASE
NDUFS8	9	2.11E+04	0.008	ALZHEIMERS DISEASE
APAF1	13	3.41E+04	0.020	ALZHEIMERS DISEASE
APP	2	3.42E+03	0.021	ALZHEIMERS DISEASE
UQCR10	1	7.97E+03	0.037	ALZHEIMERS DISEASE
UQCR11	1	6.97E+02	0.047	ALZHEIMERS DISEASE
NDUFS7	10	4.16E+04	0.058	ALZHEIMERS DISEASE
COX7A1	2	1.95E+03	0.075	ALZHEIMERS DISEASE
ATF6	6	2.58E+04	0.077	ALZHEIMERS DISEASE
NDUFA7	2	1.24E+04	0.077	ALZHEIMERS DISEASE
ITPR1	13	3.91E+04	0.079	ALZHEIMERS DISEASE
PSEN2	4	1.17E+04	0.080	ALZHEIMERS DISEASE
COX6B2	1	2.21E+03	0.107	ALZHEIMERS DISEASE
NDUFS5	1	4.64E+02	0.109	ALZHEIMERS DISEASE
CASP7	8	1.19E+04	0.134	ALZHEIMERS DISEASE
NDUFS6	1	4.30E+02	0.135	ALZHEIMERS DISEASE
NDUFB5	1	6.02E+03	0.159	ALZHEIMERS DISEASE
PPP3R2	3	4.96E+03	0.163	ALZHEIMERS DISEASE
GAPDH	3	1.29E+03	0.165	ALZHEIMERS DISEASE
COX6A2	2	4.59E+03	0.168	ALZHEIMERS DISEASE
PLCB2	12	1.50E+04	0.196	ALZHEIMERS DISEASE
CALM2	1	3.38E+02	0.198	ALZHEIMERS DISEASE
ADAM10	2	1.51E+03	0.202	ALZHEIMERS DISEASE
PSEN1	1	5.96E+03	0.209	ALZHEIMERS DISEASE
CACNA1D	16	1.44E+04	0.211	ALZHEIMERS DISEASE
CHP2	3	2.91E+03	0.217	ALZHEIMERS DISEASE
BID	1	5.61E+03	0.220	ALZHEIMERS DISEASE
ATP2A3	13	2.29E+04	0.229	ALZHEIMERS DISEASE
NDUFA9	5	8.13E+03	0.240	ALZHEIMERS DISEASE
APBB1	9	1.26E+04	0.243	ALZHEIMERS DISEASE
PPP3CA	2	8.30E+02	0.249	ALZHEIMERS DISEASE
NDUFA2	2	1.54E+03	0.275	ALZHEIMERS DISEASE
GRIN2D	3	4.55E+03	0.287	ALZHEIMERS DISEASE
UQCRB	1	3.58E+02	0.292	ALZHEIMERS DISEASE
SDHB	2	6.63E+03	0.299	ALZHEIMERS DISEASE
PPP3CC	1	3.03E+03	0.303	ALZHEIMERS DISEASE
NDUFA4L2	1	3.40E+02	0.307	ALZHEIMERS DISEASE
LPL	4	1.26E+04	0.316	ALZHEIMERS DISEASE
NDUFB3	4	2.55E+03	0.350	ALZHEIMERS DISEASE
COX6A1	3	1.24E+03	0.358	ALZHEIMERS DISEASE
NAE1	2	6.58E+02	0.369	ALZHEIMERS DISEASE
PLCB1	8	6.14E+03	0.392	ALZHEIMERS DISEASE
NOS1	17	1.54E+04	0.393	ALZHEIMERS DISEASE
ITPR2	10	9.65E+03	0.393	ALZHEIMERS DISEASE
CAPN2	10	1.01E+04	0.395	ALZHEIMERS DISEASE
NDUFS4	2	4.76E+02	0.396	ALZHEIMERS DISEASE
FAS	2	2.34E+03	0.420	ALZHEIMERS DISEASE
BAD	2	4.85E+02	0.424	ALZHEIMERS DISEASE
NDUFS3	1	9.32E+02	0.436	ALZHEIMERS DISEASE
BACE2	2	2.87E+03	0.439	ALZHEIMERS DISEASE
NDUFB6	2	6.88E+02	0.440	ALZHEIMERS DISEASE
NDUFB9	1	4.30E+02	0.443	ALZHEIMERS DISEASE
CYC1	5	3.74E+03	0.448	ALZHEIMERS DISEASE
COX4I1	2	1.10E+03	0.451	ALZHEIMERS DISEASE
NDUFA3	1	1.99E+03	0.452	ALZHEIMERS DISEASE
SDHD	4	9.99E+03	0.452	ALZHEIMERS DISEASE
UQCRC1	6	6.87E+03	0.454	ALZHEIMERS DISEASE
COX8C	1	1.25E+03	0.465	ALZHEIMERS DISEASE
COX7A2L	2	6.42E+02	0.468	ALZHEIMERS DISEASE
IDE	3	1.10E+03	0.469	ALZHEIMERS DISEASE
GRIN2A	9	9.26E+03	0.475	ALZHEIMERS DISEASE
ADAM17	6	7.75E+03	0.505	ALZHEIMERS DISEASE
NDUFS2	3	2.06E+03	0.544	ALZHEIMERS DISEASE
PLCB4	7	4.27E+03	0.544	ALZHEIMERS DISEASE
COX5B	1	1.23E+02	0.550	ALZHEIMERS DISEASE
GRIN2C	12	8.47E+03	0.564	ALZHEIMERS DISEASE
NCSTN	5	2.13E+03	0.575	ALZHEIMERS DISEASE

NDUFB10	1	4.50E+01	0.589	ALZHEIMERS DISEASE
CASP9	8	6.15E+03	0.602	ALZHEIMERS DISEASE
SDHA	2	4.17E+02	0.608	ALZHEIMERS DISEASE
PLCB3	7	6.74E+03	0.611	ALZHEIMERS DISEASE
CAPN1	7	2.70E+03	0.618	ALZHEIMERS DISEASE
ITPR3	15	4.94E+03	0.623	ALZHEIMERS DISEASE
NDUFA6	1	3.99E+02	0.625	ALZHEIMERS DISEASE
CALML3	1	3.54E+01	0.670	ALZHEIMERS DISEASE
NDUFV1	1	4.58E+01	0.671	ALZHEIMERS DISEASE
CALML5	2	7.99E+02	0.680	ALZHEIMERS DISEASE
CACNA1C	14	4.13E+03	0.694	ALZHEIMERS DISEASE
ERN1	5	3.81E+03	0.707	ALZHEIMERS DISEASE
CACNA1S	22	1.57E+04	0.707	ALZHEIMERS DISEASE
SDHC	3	2.73E+03	0.715	ALZHEIMERS DISEASE
EIF2AK3	7	1.67E+03	0.733	ALZHEIMERS DISEASE
TNF	1	1.53E+02	0.736	ALZHEIMERS DISEASE
COX5A	4	6.19E+02	0.750	ALZHEIMERS DISEASE
MAPT	10	5.37E+03	0.751	ALZHEIMERS DISEASE
COX4I2	3	3.59E+02	0.754	ALZHEIMERS DISEASE
CALML6	1	3.16E+01	0.765	ALZHEIMERS DISEASE
ATP2A1	2	1.28E+02	0.790	ALZHEIMERS DISEASE
COX7A2	1	4.51E+01	0.796	ALZHEIMERS DISEASE
RYR3	37	1.84E+04	0.815	ALZHEIMERS DISEASE
APOE	4	4.35E+02	0.821	ALZHEIMERS DISEASE
UQCRC2	3	3.68E+02	0.823	ALZHEIMERS DISEASE
CDK5R1	1	8.77E+00	0.839	ALZHEIMERS DISEASE
NDUFA5	3	2.42E+02	0.857	ALZHEIMERS DISEASE
BACE1	3	5.41E+02	0.873	ALZHEIMERS DISEASE
NDUFV3	7	7.46E+02	0.884	ALZHEIMERS DISEASE
COX6B1	1	2.55E+00	0.911	ALZHEIMERS DISEASE
UQCRHL	1	2.88E+00	0.915	ALZHEIMERS DISEASE
COX6C	1	1.03E+01	0.939	ALZHEIMERS DISEASE
IL1B	1	7.49E-01	0.950	ALZHEIMERS DISEASE
CASP3	2	2.05E+02	1	ALZHEIMERS DISEASE
NDUFS1	9	1.78E+01	1	ALZHEIMERS DISEASE
CASP8	3	4.03E+02	1	ALZHEIMERS DISEASE
MME	9	2.49E+03	1	ALZHEIMERS DISEASE
ATP2A2	3	4.04E+00	1	ALZHEIMERS DISEASE
LRP1	26	1.45E+04	1	ALZHEIMERS DISEASE
MAPK3	5	2.78E+01	1	ALZHEIMERS DISEASE
NDUFB1	3	6.84E+02	1	ALZHEIMERS DISEASE

Gene	NumVar	Q	Pvalue	Gene-set
HIST1H2BA	3	6.50E+03	0.003	AMYLOID FIBER FORMATION
HIST2H2BE	1	5.20E+03	0.009	AMYLOID FIBER FORMATION
HIST1H2BE	2	1.05E+04	0.019	AMYLOID FIBER FORMATION
APH1B	5	2.03E+04	0.021	AMYLOID FIBER FORMATION
HIST1H2BO	1	8.61E+02	0.021	AMYLOID FIBER FORMATION
APP	2	3.42E+03	0.021	AMYLOID FIBER FORMATION
APOA4	9	2.92E+04	0.057	AMYLOID FIBER FORMATION
HSPG2	81	1.01E+05	0.064	AMYLOID FIBER FORMATION
HIST1H2AJ	1	8.79E+02	0.065	AMYLOID FIBER FORMATION
CALB1	1	5.50E+02	0.069	AMYLOID FIBER FORMATION
HIST1H2BG	2	1.64E+03	0.076	AMYLOID FIBER FORMATION
RPS27A	1	2.69E+03	0.087	AMYLOID FIBER FORMATION
PRKN	10	2.32E+04	0.093	AMYLOID FIBER FORMATION
HIST2H3D	1	2.46E+03	0.095	AMYLOID FIBER FORMATION
SORL1	23	7.24E+04	0.097	AMYLOID FIBER FORMATION
HIST1H2BH	1	7.15E+02	0.109	AMYLOID FIBER FORMATION
HIST1H4F	1	3.78E+03	0.118	AMYLOID FIBER FORMATION
SIAH2	3	1.50E+03	0.130	AMYLOID FIBER FORMATION
PRL	2	1.18E+03	0.138	AMYLOID FIBER FORMATION
APOA1	3	2.38E+03	0.160	AMYLOID FIBER FORMATION
NAT8	10	1.75E+04	0.166	AMYLOID FIBER FORMATION
ADAM10	2	1.51E+03	0.202	AMYLOID FIBER FORMATION
CST3	1	4.78E+02	0.251	AMYLOID FIBER FORMATION
FURIN	8	1.42E+04	0.262	AMYLOID FIBER FORMATION
HIST1H2BI	2	1.64E+03	0.267	AMYLOID FIBER FORMATION
HIST1H4L	1	3.15E+03	0.268	AMYLOID FIBER FORMATION
APCS	1	2.26E+03	0.276	AMYLOID FIBER FORMATION
HIST1H2BB	1	1.81E+02	0.305	AMYLOID FIBER FORMATION
HIST1H4C	1	3.67E+02	0.307	AMYLOID FIBER FORMATION
SEMG1	12	2.89E+04	0.317	AMYLOID FIBER FORMATION
GGA3	15	2.69E+04	0.339	AMYLOID FIBER FORMATION

HIST1H3A	2	7.92E+02	0.346	AMYLOID FIBER FORMATION
TGFBI	7	4.29E+03	0.360	AMYLOID FIBER FORMATION
ITM2B	2	4.91E+02	0.433	AMYLOID FIBER FORMATION
SNCAIP	10	1.58E+04	0.434	AMYLOID FIBER FORMATION
GSN	14	1.59E+04	0.440	AMYLOID FIBER FORMATION
NPPA	2	1.63E+03	0.491	AMYLOID FIBER FORMATION
FGA	7	1.83E+03	0.515	AMYLOID FIBER FORMATION
GGA2	7	2.27E+03	0.541	AMYLOID FIBER FORMATION
ODAM	2	1.31E+03	0.549	AMYLOID FIBER FORMATION
UBE2L6	1	6.07E+01	0.557	AMYLOID FIBER FORMATION
HIST1H2AB	1	6.33E+01	0.573	AMYLOID FIBER FORMATION
NCSTN	5	2.13E+03	0.575	AMYLOID FIBER FORMATION
SAA1	6	3.52E+03	0.597	AMYLOID FIBER FORMATION
HIST1H2AD	2	1.86E+02	0.665	AMYLOID FIBER FORMATION
GGA1	3	1.89E+03	0.671	AMYLOID FIBER FORMATION
HIST1H3G	1	3.66E+01	0.702	AMYLOID FIBER FORMATION
MFGE8	7	1.42E+03	0.707	AMYLOID FIBER FORMATION
CALCA	3	5.33E+03	0.716	AMYLOID FIBER FORMATION
TTR	1	1.45E+02	0.724	AMYLOID FIBER FORMATION
BACE1	3	5.41E+02	0.873	AMYLOID FIBER FORMATION
LTF	8	5.90E+02	0.900	AMYLOID FIBER FORMATION
B2M	1	4.62E+00	0.913	AMYLOID FIBER FORMATION
TSPAN15	4	2.55E+01	1	AMYLOID FIBER FORMATION

Gene	NumVar	Q	Pvalue	Gene-set
CSNK1D	4	1.32E+04	0.002	ASPARAGINE N LINKED GLYCOSYLATION
MAN1B1	15	3.58E+04	0.010	ASPARAGINE N LINKED GLYCOSYLATION
COG5	10	3.93E+04	0.011	ASPARAGINE N LINKED GLYCOSYLATION
ARFGAP1	8	2.88E+04	0.013	ASPARAGINE N LINKED GLYCOSYLATION
ENGASE	15	4.17E+04	0.015	ASPARAGINE N LINKED GLYCOSYLATION
TUBB1	8	4.79E+04	0.016	ASPARAGINE N LINKED GLYCOSYLATION
ST6GALNAC6	4	2.75E+04	0.016	ASPARAGINE N LINKED GLYCOSYLATION
COPG1	3	5.26E+03	0.020	ASPARAGINE N LINKED GLYCOSYLATION
ST8SIA3	2	2.68E+03	0.021	ASPARAGINE N LINKED GLYCOSYLATION
B4GALT1	1	1.34E+03	0.022	ASPARAGINE N LINKED GLYCOSYLATION
VCP	2	3.84E+03	0.022	ASPARAGINE N LINKED GLYCOSYLATION
ASGR1	1	8.46E+02	0.023	ASPARAGINE N LINKED GLYCOSYLATION
ACTR10	2	4.89E+03	0.029	ASPARAGINE N LINKED GLYCOSYLATION
EDEM1	8	1.37E+04	0.030	ASPARAGINE N LINKED GLYCOSYLATION
DYNC1LI2	2	2.58E+03	0.030	ASPARAGINE N LINKED GLYCOSYLATION
STT3A	2	2.10E+03	0.031	ASPARAGINE N LINKED GLYCOSYLATION
MGAT1	1	1.98E+03	0.036	ASPARAGINE N LINKED GLYCOSYLATION
DYNC1LI1	5	2.20E+04	0.038	ASPARAGINE N LINKED GLYCOSYLATION
SLC17A5	5	1.63E+04	0.054	ASPARAGINE N LINKED GLYCOSYLATION
TUBB8	5	1.83E+04	0.058	ASPARAGINE N LINKED GLYCOSYLATION
NPL	6	4.06E+03	0.060	ASPARAGINE N LINKED GLYCOSYLATION
DOLK	6	1.71E+04	0.060	ASPARAGINE N LINKED GLYCOSYLATION
ALG6	1	1.24E+04	0.068	ASPARAGINE N LINKED GLYCOSYLATION
COG4	5	1.41E+04	0.072	ASPARAGINE N LINKED GLYCOSYLATION
NEU2	3	9.10E+03	0.076	ASPARAGINE N LINKED GLYCOSYLATION
RPS27A	1	2.69E+03	0.087	ASPARAGINE N LINKED GLYCOSYLATION
EDEM2	4	9.43E+03	0.091	ASPARAGINE N LINKED GLYCOSYLATION
ARFGAP2	3	1.43E+04	0.092	ASPARAGINE N LINKED GLYCOSYLATION
STX5	6	1.12E+04	0.094	ASPARAGINE N LINKED GLYCOSYLATION
COPB2	2	1.95E+03	0.096	ASPARAGINE N LINKED GLYCOSYLATION
PPP6R3	5	2.60E+04	0.096	ASPARAGINE N LINKED GLYCOSYLATION
SPTBN2	17	3.45E+04	0.097	ASPARAGINE N LINKED GLYCOSYLATION
ST6GAL2	10	3.49E+04	0.099	ASPARAGINE N LINKED GLYCOSYLATION
RPN1	8	7.20E+03	0.100	ASPARAGINE N LINKED GLYCOSYLATION
TRAPPC6A	4	5.10E+03	0.102	ASPARAGINE N LINKED GLYCOSYLATION
GBF1	5	5.83E+03	0.116	ASPARAGINE N LINKED GLYCOSYLATION
STX17	2	6.90E+03	0.126	ASPARAGINE N LINKED GLYCOSYLATION
TMED9	2	2.02E+03	0.132	ASPARAGINE N LINKED GLYCOSYLATION
ST3GAL1	4	1.96E+04	0.142	ASPARAGINE N LINKED GLYCOSYLATION
ST6GALNAC1	2	8.30E+03	0.147	ASPARAGINE N LINKED GLYCOSYLATION
SYVN1	3	1.88E+03	0.148	ASPARAGINE N LINKED GLYCOSYLATION
TUBA8	4	1.16E+04	0.157	ASPARAGINE N LINKED GLYCOSYLATION
DCTN4	5	1.08E+04	0.159	ASPARAGINE N LINKED GLYCOSYLATION
TFG	1	1.70E+03	0.163	ASPARAGINE N LINKED GLYCOSYLATION
RNF185	2	3.37E+03	0.164	ASPARAGINE N LINKED GLYCOSYLATION
ANKRD28	11	1.70E+04	0.167	ASPARAGINE N LINKED GLYCOSYLATION
MGAT4B	6	2.03E+04	0.171	ASPARAGINE N LINKED GLYCOSYLATION
TRAPPC4	1	8.59E+02	0.171	ASPARAGINE N LINKED GLYCOSYLATION
COPA	5	2.91E+03	0.178	ASPARAGINE N LINKED GLYCOSYLATION

COG7	6	6.85E+03	0.179	ASPARAGINE N LINKED GLYCOSYLATION
SPTAN1	7	9.73E+03	0.184	ASPARAGINE N LINKED GLYCOSYLATION
ALG12	2	4.93E+03	0.190	ASPARAGINE N LINKED GLYCOSYLATION
B4GALT5	1	6.10E+03	0.198	ASPARAGINE N LINKED GLYCOSYLATION
TRAPPC6B	1	7.57E+02	0.203	ASPARAGINE N LINKED GLYCOSYLATION
ALG14	4	9.85E+03	0.206	ASPARAGINE N LINKED GLYCOSYLATION
DCTN6	1	9.42E+02	0.212	ASPARAGINE N LINKED GLYCOSYLATION
GOLGB1	31	3.79E+04	0.219	ASPARAGINE N LINKED GLYCOSYLATION
MGAT3	6	6.56E+03	0.227	ASPARAGINE N LINKED GLYCOSYLATION
DCTN5	1	2.97E+02	0.228	ASPARAGINE N LINKED GLYCOSYLATION
DYNC1I2	4	9.48E+02	0.261	ASPARAGINE N LINKED GLYCOSYLATION
F5	40	4.85E+04	0.263	ASPARAGINE N LINKED GLYCOSYLATION
ST8SIA5	2	1.29E+03	0.267	ASPARAGINE N LINKED GLYCOSYLATION
TUBA1C	2	6.49E+03	0.276	ASPARAGINE N LINKED GLYCOSYLATION
GORASP1	8	2.49E+04	0.282	ASPARAGINE N LINKED GLYCOSYLATION
DHDDS	2	1.39E+03	0.287	ASPARAGINE N LINKED GLYCOSYLATION
TMEM115	2	7.51E+02	0.292	ASPARAGINE N LINKED GLYCOSYLATION
SEC22B	1	1.94E+02	0.292	ASPARAGINE N LINKED GLYCOSYLATION
TUBB2B	2	1.47E+03	0.301	ASPARAGINE N LINKED GLYCOSYLATION
SEC24D	9	1.62E+04	0.302	ASPARAGINE N LINKED GLYCOSYLATION
DYNC1I1	2	1.00E+03	0.308	ASPARAGINE N LINKED GLYCOSYLATION
SEC23IP	9	1.31E+04	0.310	ASPARAGINE N LINKED GLYCOSYLATION
PGM3	4	1.48E+03	0.315	ASPARAGINE N LINKED GLYCOSYLATION
SLC35C1	1	2.24E+03	0.318	ASPARAGINE N LINKED GLYCOSYLATION
SEC16A	27	3.63E+04	0.330	ASPARAGINE N LINKED GLYCOSYLATION
CTSZ	3	3.74E+03	0.331	ASPARAGINE N LINKED GLYCOSYLATION
SEC24A	7	5.44E+03	0.334	ASPARAGINE N LINKED GLYCOSYLATION
ST8SIA6	3	2.83E+03	0.339	ASPARAGINE N LINKED GLYCOSYLATION
ST3GAL4	6	6.28E+03	0.341	ASPARAGINE N LINKED GLYCOSYLATION
SEC24B	6	2.88E+03	0.343	ASPARAGINE N LINKED GLYCOSYLATION
SPTBN4	18	2.07E+04	0.348	ASPARAGINE N LINKED GLYCOSYLATION
TMED2	1	2.34E+02	0.351	ASPARAGINE N LINKED GLYCOSYLATION
PDIA3	4	2.46E+03	0.362	ASPARAGINE N LINKED GLYCOSYLATION
FPGT	5	4.58E+03	0.365	ASPARAGINE N LINKED GLYCOSYLATION
TSTA3	5	1.74E+03	0.365	ASPARAGINE N LINKED GLYCOSYLATION
UBXN1	2	4.55E+03	0.387	ASPARAGINE N LINKED GLYCOSYLATION
RFT1	7	4.00E+03	0.395	ASPARAGINE N LINKED GLYCOSYLATION
ST6GALNAC4	1	5.47E+02	0.399	ASPARAGINE N LINKED GLYCOSYLATION
GANAB	5	6.35E+03	0.403	ASPARAGINE N LINKED GLYCOSYLATION
B4GALT4	3	3.04E+03	0.405	ASPARAGINE N LINKED GLYCOSYLATION
NSF	1	2.81E+02	0.406	ASPARAGINE N LINKED GLYCOSYLATION
CAPZB	2	3.66E+03	0.406	ASPARAGINE N LINKED GLYCOSYLATION
SLC35A1	2	1.90E+03	0.407	ASPARAGINE N LINKED GLYCOSYLATION
COPG2	3	6.16E+03	0.414	ASPARAGINE N LINKED GLYCOSYLATION
KDELR3	2	1.72E+03	0.416	ASPARAGINE N LINKED GLYCOSYLATION
TUBA3C	4	1.05E+04	0.420	ASPARAGINE N LINKED GLYCOSYLATION
DPM1	2	7.93E+02	0.426	ASPARAGINE N LINKED GLYCOSYLATION
B4GALT2	4	2.53E+03	0.433	ASPARAGINE N LINKED GLYCOSYLATION
CANX	4	3.11E+03	0.436	ASPARAGINE N LINKED GLYCOSYLATION
CHST10	3	1.44E+03	0.437	ASPARAGINE N LINKED GLYCOSYLATION
MANEA	5	2.17E+03	0.445	ASPARAGINE N LINKED GLYCOSYLATION
DOLPP1	1	1.69E+02	0.446	ASPARAGINE N LINKED GLYCOSYLATION
ANK3	34	3.47E+04	0.447	ASPARAGINE N LINKED GLYCOSYLATION
ST6GALNAC2	4	2.46E+03	0.449	ASPARAGINE N LINKED GLYCOSYLATION
NUDT14	6	4.09E+03	0.456	ASPARAGINE N LINKED GLYCOSYLATION
GRIA1	2	1.90E+03	0.459	ASPARAGINE N LINKED GLYCOSYLATION
MAN2A2	9	8.13E+03	0.459	ASPARAGINE N LINKED GLYCOSYLATION
ARCN1	1	5.55E+01	0.466	ASPARAGINE N LINKED GLYCOSYLATION
TUBB4A	1	4.61E+02	0.476	ASPARAGINE N LINKED GLYCOSYLATION
UGGT1	7	3.83E+03	0.479	ASPARAGINE N LINKED GLYCOSYLATION
RAD23B	1	2.06E+02	0.481	ASPARAGINE N LINKED GLYCOSYLATION
ALG10	3	1.24E+03	0.483	ASPARAGINE N LINKED GLYCOSYLATION
NAPG	3	1.57E+03	0.491	ASPARAGINE N LINKED GLYCOSYLATION
MOGS	10	1.44E+04	0.494	ASPARAGINE N LINKED GLYCOSYLATION
SCFD1	4	4.76E+03	0.504	ASPARAGINE N LINKED GLYCOSYLATION
MPDU1	2	6.44E+02	0.506	ASPARAGINE N LINKED GLYCOSYLATION
CAPZA2	2	4.45E+02	0.506	ASPARAGINE N LINKED GLYCOSYLATION
MIA3	19	1.60E+04	0.512	ASPARAGINE N LINKED GLYCOSYLATION
MAN2A1	6	1.54E+03	0.513	ASPARAGINE N LINKED GLYCOSYLATION
GNE	3	8.75E+02	0.516	ASPARAGINE N LINKED GLYCOSYLATION
MGAT4A	5	3.88E+03	0.520	ASPARAGINE N LINKED GLYCOSYLATION
RAB1A	1	1.38E+03	0.521	ASPARAGINE N LINKED GLYCOSYLATION
LMAN1L	6	2.86E+03	0.524	ASPARAGINE N LINKED GLYCOSYLATION
TRAPPC10	7	3.67E+03	0.527	ASPARAGINE N LINKED GLYCOSYLATION

SPTBN1	13	8.86E+03	0.555	ASPARAGINE N LINKED GLYCOSYLATION
AREG	1	1.33E+03	0.563	ASPARAGINE N LINKED GLYCOSYLATION
PMM2	7	4.86E+03	0.567	ASPARAGINE N LINKED GLYCOSYLATION
ST6GALNAC5	2	1.51E+03	0.569	ASPARAGINE N LINKED GLYCOSYLATION
ALG2	4	2.48E+03	0.571	ASPARAGINE N LINKED GLYCOSYLATION
ARFGAP3	8	1.41E+04	0.571	ASPARAGINE N LINKED GLYCOSYLATION
TBC1D20	2	1.78E+03	0.573	ASPARAGINE N LINKED GLYCOSYLATION
PRKCSH	12	7.49E+03	0.576	ASPARAGINE N LINKED GLYCOSYLATION
MAN1A1	3	2.12E+03	0.590	ASPARAGINE N LINKED GLYCOSYLATION
B4GALNT2	5	6.61E+03	0.595	ASPARAGINE N LINKED GLYCOSYLATION
TRAPPC1	1	1.99E+01	0.595	ASPARAGINE N LINKED GLYCOSYLATION
USO1	7	1.69E+03	0.595	ASPARAGINE N LINKED GLYCOSYLATION
B4GALT6	1	7.97E+01	0.597	ASPARAGINE N LINKED GLYCOSYLATION
AMFR	2	4.94E+02	0.603	ASPARAGINE N LINKED GLYCOSYLATION
SEL1L	9	8.63E+03	0.603	ASPARAGINE N LINKED GLYCOSYLATION
ANK2	31	1.51E+04	0.612	ASPARAGINE N LINKED GLYCOSYLATION
COG2	6	3.53E+03	0.615	ASPARAGINE N LINKED GLYCOSYLATION
COPZ2	2	7.01E+02	0.623	ASPARAGINE N LINKED GLYCOSYLATION
GOSR2	3	2.58E+03	0.625	ASPARAGINE N LINKED GLYCOSYLATION
LMAN2	3	1.56E+03	0.627	ASPARAGINE N LINKED GLYCOSYLATION
FUT8	4	2.19E+03	0.627	ASPARAGINE N LINKED GLYCOSYLATION
ALG1	9	3.80E+03	0.628	ASPARAGINE N LINKED GLYCOSYLATION
CHST8	6	2.75E+03	0.628	ASPARAGINE N LINKED GLYCOSYLATION
OS9	7	7.48E+02	0.629	ASPARAGINE N LINKED GLYCOSYLATION
CALR	5	1.10E+03	0.637	ASPARAGINE N LINKED GLYCOSYLATION
TUBA3D	1	6.84E+01	0.638	ASPARAGINE N LINKED GLYCOSYLATION
CTSC	5	8.46E+02	0.639	ASPARAGINE N LINKED GLYCOSYLATION
COPE	5	1.64E+03	0.639	ASPARAGINE N LINKED GLYCOSYLATION
SEC23A	3	5.92E+02	0.640	ASPARAGINE N LINKED GLYCOSYLATION
DAD1	1	1.27E+02	0.641	ASPARAGINE N LINKED GLYCOSYLATION
GOLGA2	13	5.05E+03	0.643	ASPARAGINE N LINKED GLYCOSYLATION
EDEM3	6	1.02E+04	0.645	ASPARAGINE N LINKED GLYCOSYLATION
GMPPA	7	2.55E+03	0.652	ASPARAGINE N LINKED GLYCOSYLATION
TUBB6	5	2.34E+03	0.653	ASPARAGINE N LINKED GLYCOSYLATION
B4GALT3	3	1.10E+03	0.655	ASPARAGINE N LINKED GLYCOSYLATION
SEC24C	11	7.28E+03	0.658	ASPARAGINE N LINKED GLYCOSYLATION
NEU4	11	7.75E+03	0.668	ASPARAGINE N LINKED GLYCOSYLATION
CAPZA3	9	9.04E+03	0.673	ASPARAGINE N LINKED GLYCOSYLATION
ST8SIA2	1	3.24E+01	0.676	ASPARAGINE N LINKED GLYCOSYLATION
DCTN2	3	2.90E+02	0.676	ASPARAGINE N LINKED GLYCOSYLATION
NAPA	2	4.76E+02	0.681	ASPARAGINE N LINKED GLYCOSYLATION
MLEC	1	3.56E+01	0.683	ASPARAGINE N LINKED GLYCOSYLATION
ALG5	1	1.19E+02	0.688	ASPARAGINE N LINKED GLYCOSYLATION
TRAPPC9	11	4.40E+03	0.691	ASPARAGINE N LINKED GLYCOSYLATION
ST3GAL5	3	1.80E+03	0.694	ASPARAGINE N LINKED GLYCOSYLATION
NGLY1	5	6.36E+02	0.697	ASPARAGINE N LINKED GLYCOSYLATION
SEC31A	6	2.22E+03	0.697	ASPARAGINE N LINKED GLYCOSYLATION
CAPZA1	4	2.33E+03	0.706	ASPARAGINE N LINKED GLYCOSYLATION
TRIM13	1	4.83E+02	0.716	ASPARAGINE N LINKED GLYCOSYLATION
CTSA	3	6.21E+02	0.717	ASPARAGINE N LINKED GLYCOSYLATION
MGAT4C	3	1.21E+03	0.721	ASPARAGINE N LINKED GLYCOSYLATION
RNF103	9	4.69E+03	0.722	ASPARAGINE N LINKED GLYCOSYLATION
NEU3	6	2.67E+03	0.726	ASPARAGINE N LINKED GLYCOSYLATION
UGGT2	18	6.41E+03	0.731	ASPARAGINE N LINKED GLYCOSYLATION
ALG9	6	1.10E+03	0.734	ASPARAGINE N LINKED GLYCOSYLATION
ALG3	4	1.36E+03	0.734	ASPARAGINE N LINKED GLYCOSYLATION
GMPPB	2	1.41E+03	0.737	ASPARAGINE N LINKED GLYCOSYLATION
SERPINA1	11	4.48E+03	0.737	ASPARAGINE N LINKED GLYCOSYLATION
MPI	4	1.30E+03	0.738	ASPARAGINE N LINKED GLYCOSYLATION
COG1	11	9.35E+03	0.745	ASPARAGINE N LINKED GLYCOSYLATION
UMOD	8	2.08E+03	0.749	ASPARAGINE N LINKED GLYCOSYLATION
YKT6	3	3.95E+02	0.751	ASPARAGINE N LINKED GLYCOSYLATION
NUS1	2	2.10E+02	0.754	ASPARAGINE N LINKED GLYCOSYLATION
NAGK	4	3.71E+02	0.757	ASPARAGINE N LINKED GLYCOSYLATION
DCTN1	14	4.29E+03	0.758	ASPARAGINE N LINKED GLYCOSYLATION
DYNC1H1	7	1.68E+03	0.763	ASPARAGINE N LINKED GLYCOSYLATION
TUBA4A	1	2.05E+01	0.764	ASPARAGINE N LINKED GLYCOSYLATION
LMAN2L	1	1.50E+01	0.764	ASPARAGINE N LINKED GLYCOSYLATION
SPTB	27	4.35E+03	0.768	ASPARAGINE N LINKED GLYCOSYLATION
PREB	1	3.27E+01	0.770	ASPARAGINE N LINKED GLYCOSYLATION
DERL1	1	3.33E+01	0.772	ASPARAGINE N LINKED GLYCOSYLATION
GFPT2	5	4.66E+02	0.774	ASPARAGINE N LINKED GLYCOSYLATION
LMAN1	2	3.58E+02	0.777	ASPARAGINE N LINKED GLYCOSYLATION
LHB	3	1.75E+03	0.780	ASPARAGINE N LINKED GLYCOSYLATION

MGAT5	5	4.79E+02	0.785	ASPARAGINE N LINKED GLYCOSYLATION
GOSR1	1	2.71E+01	0.789	ASPARAGINE N LINKED GLYCOSYLATION
NANS	6	2.21E+03	0.791	ASPARAGINE N LINKED GLYCOSYLATION
ST8SIA1	3	5.17E+02	0.794	ASPARAGINE N LINKED GLYCOSYLATION
COG6	2	2.38E+02	0.802	ASPARAGINE N LINKED GLYCOSYLATION
TUBA3E	6	1.15E+03	0.814	ASPARAGINE N LINKED GLYCOSYLATION
SEC22C	4	7.97E+02	0.816	ASPARAGINE N LINKED GLYCOSYLATION
DDOST	5	1.20E+03	0.818	ASPARAGINE N LINKED GLYCOSYLATION
COG3	5	1.20E+03	0.822	ASPARAGINE N LINKED GLYCOSYLATION
TUSC3	3	9.12E+02	0.823	ASPARAGINE N LINKED GLYCOSYLATION
COL7A1	24	1.24E+04	0.824	ASPARAGINE N LINKED GLYCOSYLATION
TUBAL3	7	6.78E+02	0.831	ASPARAGINE N LINKED GLYCOSYLATION
MGAT2	3	3.43E+02	0.838	ASPARAGINE N LINKED GLYCOSYLATION
MAN1A2	2	3.68E+02	0.841	ASPARAGINE N LINKED GLYCOSYLATION
TRAPPC2L	4	3.36E+03	0.843	ASPARAGINE N LINKED GLYCOSYLATION
ST6GALNAC3	4	1.22E+03	0.846	ASPARAGINE N LINKED GLYCOSYLATION
TMED3	3	2.98E+02	0.858	ASPARAGINE N LINKED GLYCOSYLATION
SEC16B	14	3.71E+03	0.860	ASPARAGINE N LINKED GLYCOSYLATION
PSMC1	1	4.42E+00	0.860	ASPARAGINE N LINKED GLYCOSYLATION
NAPB	1	2.77E+01	0.861	ASPARAGINE N LINKED GLYCOSYLATION
FUT3	16	2.16E+03	0.867	ASPARAGINE N LINKED GLYCOSYLATION
SPTA1	44	1.36E+04	0.869	ASPARAGINE N LINKED GLYCOSYLATION
MVD	10	5.04E+02	0.876	ASPARAGINE N LINKED GLYCOSYLATION
AMDHD2	10	1.37E+03	0.877	ASPARAGINE N LINKED GLYCOSYLATION
SEC13	1	5.26E+00	0.889	ASPARAGINE N LINKED GLYCOSYLATION
ALG10B	10	6.55E+03	0.889	ASPARAGINE N LINKED GLYCOSYLATION
COG8	4	5.00E+02	0.891	ASPARAGINE N LINKED GLYCOSYLATION
FUCA1	4	5.41E+02	0.896	ASPARAGINE N LINKED GLYCOSYLATION
FOLR1	1	1.99E+01	0.899	ASPARAGINE N LINKED GLYCOSYLATION
CD55	5	1.70E+02	0.906	ASPARAGINE N LINKED GLYCOSYLATION
CNIH3	1	3.48E+00	0.907	ASPARAGINE N LINKED GLYCOSYLATION
ANK1	20	1.01E+04	0.917	ASPARAGINE N LINKED GLYCOSYLATION
DCTN3	1	1.52E+00	0.925	ASPARAGINE N LINKED GLYCOSYLATION
SPTBN5	57	3.18E+04	0.928	ASPARAGINE N LINKED GLYCOSYLATION
TRAPPC5	1	1.18E+00	0.935	ASPARAGINE N LINKED GLYCOSYLATION
SEC22A	1	2.38E-01	0.967	ASPARAGINE N LINKED GLYCOSYLATION
MCFD2	2	2.75E+02	1	ASPARAGINE N LINKED GLYCOSYLATION
ST3GAL3	4	5.29E+02	1	ASPARAGINE N LINKED GLYCOSYLATION
UAP1	7	1.03E+03	1	ASPARAGINE N LINKED GLYCOSYLATION
PMM1	5	1.29E+03	1	ASPARAGINE N LINKED GLYCOSYLATION
FUOM	4	6.20E+00	1	ASPARAGINE N LINKED GLYCOSYLATION
NANP	2	1.18E+02	1	ASPARAGINE N LINKED GLYCOSYLATION
RPN2	6	5.08E+01	1	ASPARAGINE N LINKED GLYCOSYLATION
GLB1	7	7.02E+02	1	ASPARAGINE N LINKED GLYCOSYLATION
ALG11	10	3.49E+01	1	ASPARAGINE N LINKED GLYCOSYLATION
ST3GAL6	3	1.19E+01	1	ASPARAGINE N LINKED GLYCOSYLATION
ALG8	5	1.32E+03	1	ASPARAGINE N LINKED GLYCOSYLATION
GFPT1	2	1.41E+01	1	ASPARAGINE N LINKED GLYCOSYLATION
DPAGT1	2	1.12E+02	1	ASPARAGINE N LINKED GLYCOSYLATION
COPB1	6	1.14E+02	1	ASPARAGINE N LINKED GLYCOSYLATION
MAN1C1	3	2.76E+02	1	ASPARAGINE N LINKED GLYCOSYLATION
PPP6R1	11	6.29E+03	1	ASPARAGINE N LINKED GLYCOSYLATION
TUBA4B	5	2.49E+02	1	ASPARAGINE N LINKED GLYCOSYLATION

Gene	NumVar	Q	Pvalue	Gene-set
DRD2	13	5.66E+06	2.15E-10	GPCR LIGAND BINDING
FFAR3	14	3.28E+05	0.001	GPCR LIGAND BINDING
RRH	5	3.58E+04	0.001	GPCR LIGAND BINDING
QRFPR	8	1.58E+05	0.010	GPCR LIGAND BINDING
WNT2	4	2.90E+04	0.012	GPCR LIGAND BINDING
C5AR1	7	4.11E+04	0.018	GPCR LIGAND BINDING
ADRA1D	8	4.79E+04	0.019	GPCR LIGAND BINDING
APP	2	3.42E+03	0.021	GPCR LIGAND BINDING
SSTR4	6	1.98E+04	0.026	GPCR LIGAND BINDING
FZD7	1	1.47E+04	0.028	GPCR LIGAND BINDING
GNG4	1	4.00E+03	0.028	GPCR LIGAND BINDING
PROK2	2	1.68E+03	0.028	GPCR LIGAND BINDING
TAS2R13	1	2.03E+03	0.031	GPCR LIGAND BINDING
ADM2	4	6.37E+03	0.034	GPCR LIGAND BINDING
MC4R	5	4.32E+04	0.034	GPCR LIGAND BINDING
NMBR	7	3.42E+04	0.037	GPCR LIGAND BINDING
CXCL13	1	5.44E+02	0.040	GPCR LIGAND BINDING
MLNR	1	1.22E+04	0.042	GPCR LIGAND BINDING
F2	5	7.45E+03	0.047	GPCR LIGAND BINDING

CCL25	2	1.51E+04	0.055	GPCR LIGAND BINDING
TAS2R39	4	1.10E+04	0.058	GPCR LIGAND BINDING
INSL5	3	7.77E+03	0.060	GPCR LIGAND BINDING
GNG10	1	4.84E+03	0.060	GPCR LIGAND BINDING
RLN2	6	1.71E+04	0.062	GPCR LIGAND BINDING
APLNR	4	6.90E+03	0.065	GPCR LIGAND BINDING
GNAS	16	3.79E+04	0.066	GPCR LIGAND BINDING
GPHB5	3	1.53E+04	0.067	GPCR LIGAND BINDING
P2RY6	4	5.25E+03	0.068	GPCR LIGAND BINDING
CRHBP	3	5.16E+03	0.071	GPCR LIGAND BINDING
LPAR5	1	4.80E+02	0.082	GPCR LIGAND BINDING
NPBWR1	3	5.56E+03	0.083	GPCR LIGAND BINDING
KNG1	2	3.00E+03	0.084	GPCR LIGAND BINDING
TAAR9	5	8.59E+03	0.085	GPCR LIGAND BINDING
GHRHR	2	1.23E+04	0.088	GPCR LIGAND BINDING
CX3CL1	5	1.21E+04	0.088	GPCR LIGAND BINDING
CCR2	3	1.69E+03	0.088	GPCR LIGAND BINDING
CCL27	2	2.45E+03	0.090	GPCR LIGAND BINDING
GRP	2	8.99E+02	0.092	GPCR LIGAND BINDING
PSAP	3	3.19E+03	0.097	GPCR LIGAND BINDING
ADRB1	1	1.27E+03	0.110	GPCR LIGAND BINDING
PTH1R	4	2.07E+04	0.116	GPCR LIGAND BINDING
GIPR	9	1.08E+04	0.116	GPCR LIGAND BINDING
TAS1R1	19	5.90E+04	0.117	GPCR LIGAND BINDING
PLPPR4	5	1.65E+04	0.117	GPCR LIGAND BINDING
SMO	11	2.18E+04	0.118	GPCR LIGAND BINDING
WNT7B	1	6.05E+02	0.118	GPCR LIGAND BINDING
F2RL1	4	1.12E+04	0.121	GPCR LIGAND BINDING
WNT9A	3	3.40E+03	0.125	GPCR LIGAND BINDING
HTR5A	1	9.91E+02	0.126	GPCR LIGAND BINDING
CNR1	1	5.34E+02	0.126	GPCR LIGAND BINDING
TBXA2R	12	2.12E+04	0.133	GPCR LIGAND BINDING
CXCR4	3	5.43E+03	0.136	GPCR LIGAND BINDING
WNT10B	4	1.61E+04	0.136	GPCR LIGAND BINDING
NPB	4	5.38E+03	0.141	GPCR LIGAND BINDING
ADRB2	2	8.42E+03	0.146	GPCR LIGAND BINDING
HTR1A	3	1.64E+04	0.147	GPCR LIGAND BINDING
CHRM2	3	8.45E+03	0.148	GPCR LIGAND BINDING
TAS2R50	1	4.83E+02	0.149	GPCR LIGAND BINDING
PTGFR	6	1.78E+04	0.150	GPCR LIGAND BINDING
GCG	3	3.55E+03	0.151	GPCR LIGAND BINDING
S1PR2	4	1.54E+04	0.156	GPCR LIGAND BINDING
CCR3	7	2.09E+04	0.161	GPCR LIGAND BINDING
PTGDR	6	1.47E+04	0.161	GPCR LIGAND BINDING
HTR2B	1	2.85E+02	0.171	GPCR LIGAND BINDING
OPN4	8	2.53E+04	0.177	GPCR LIGAND BINDING
ANXA1	2	2.11E+03	0.179	GPCR LIGAND BINDING
GCGR	6	1.09E+04	0.184	GPCR LIGAND BINDING
CXCR1	8	1.15E+04	0.187	GPCR LIGAND BINDING
ACKR3	4	9.26E+03	0.188	GPCR LIGAND BINDING
HTR1B	1	6.22E+03	0.191	GPCR LIGAND BINDING
CXCL3	2	6.60E+03	0.191	GPCR LIGAND BINDING
GRM7	1	4.46E+02	0.193	GPCR LIGAND BINDING
CORT	1	3.50E+02	0.202	GPCR LIGAND BINDING
FZD4	5	2.95E+04	0.203	GPCR LIGAND BINDING
CYSLTR2	7	5.38E+04	0.204	GPCR LIGAND BINDING
GPR37	7	1.15E+04	0.206	GPCR LIGAND BINDING
FFAR4	2	6.99E+03	0.210	GPCR LIGAND BINDING
CCL5	1	3.12E+02	0.211	GPCR LIGAND BINDING
CRHR2	11	2.24E+04	0.214	GPCR LIGAND BINDING
AVPR1B	9	1.01E+04	0.228	GPCR LIGAND BINDING
ADRA2A	4	3.12E+03	0.228	GPCR LIGAND BINDING
POMC	7	1.57E+04	0.229	GPCR LIGAND BINDING
GPR17	11	9.27E+03	0.232	GPCR LIGAND BINDING
GRM6	17	3.20E+04	0.234	GPCR LIGAND BINDING
CALCB	1	3.69E+02	0.237	GPCR LIGAND BINDING
NMUR2	7	1.75E+04	0.244	GPCR LIGAND BINDING
GALR1	4	2.03E+03	0.250	GPCR LIGAND BINDING
CXCL2	3	1.19E+04	0.258	GPCR LIGAND BINDING
GNRH1	1	1.34E+03	0.266	GPCR LIGAND BINDING
PTGER1	6	6.37E+03	0.267	GPCR LIGAND BINDING
NLN	7	5.29E+03	0.275	GPCR LIGAND BINDING
KEL	8	9.16E+03	0.278	GPCR LIGAND BINDING
CCR9	4	8.16E+03	0.279	GPCR LIGAND BINDING

TAS2R43	4	2.08E+04	0.282	GPCR LIGAND BINDING
CXCL9	1	5.36E+02	0.289	GPCR LIGAND BINDING
RXFP3	5	2.24E+03	0.290	GPCR LIGAND BINDING
TAS2R14	4	1.25E+04	0.293	GPCR LIGAND BINDING
C5	12	1.23E+04	0.294	GPCR LIGAND BINDING
GPER1	5	9.33E+03	0.297	GPCR LIGAND BINDING
CNR2	5	6.09E+03	0.298	GPCR LIGAND BINDING
UCN3	1	2.38E+02	0.298	GPCR LIGAND BINDING
WNT8A	2	1.07E+03	0.300	GPCR LIGAND BINDING
FSHR	5	4.26E+03	0.301	GPCR LIGAND BINDING
S1PR4	11	1.41E+04	0.301	GPCR LIGAND BINDING
GPR18	2	3.08E+03	0.301	GPCR LIGAND BINDING
VIPR2	7	5.04E+03	0.302	GPCR LIGAND BINDING
HRH3	2	8.34E+02	0.304	GPCR LIGAND BINDING
TRHR	4	2.37E+03	0.307	GPCR LIGAND BINDING
CCR6	1	3.00E+03	0.307	GPCR LIGAND BINDING
PTGIR	4	1.04E+04	0.308	GPCR LIGAND BINDING
CXCL1	1	7.24E+02	0.308	GPCR LIGAND BINDING
MC2R	5	5.79E+03	0.313	GPCR LIGAND BINDING
WNT11	1	3.04E+02	0.315	GPCR LIGAND BINDING
TACR1	2	9.74E+02	0.318	GPCR LIGAND BINDING
IHH	5	1.99E+03	0.326	GPCR LIGAND BINDING
LTB4R	4	1.86E+03	0.327	GPCR LIGAND BINDING
GRM5	4	1.84E+03	0.328	GPCR LIGAND BINDING
HEBP1	3	1.16E+03	0.337	GPCR LIGAND BINDING
PTAFR	3	2.22E+03	0.341	GPCR LIGAND BINDING
RAMP1	1	1.35E+02	0.342	GPCR LIGAND BINDING
NPFFR1	8	5.71E+03	0.347	GPCR LIGAND BINDING
CXCR5	4	5.22E+03	0.349	GPCR LIGAND BINDING
ACKR4	5	3.86E+03	0.361	GPCR LIGAND BINDING
TAC3	1	1.28E+02	0.362	GPCR LIGAND BINDING
HRH2	3	1.80E+03	0.362	GPCR LIGAND BINDING
GPRC6A	9	1.86E+04	0.362	GPCR LIGAND BINDING
APLF	5	4.98E+03	0.366	GPCR LIGAND BINDING
CCL23	2	3.99E+03	0.367	GPCR LIGAND BINDING
OPN1SW	3	1.34E+03	0.371	GPCR LIGAND BINDING
GHRH	1	2.90E+03	0.373	GPCR LIGAND BINDING
OPRM1	16	1.52E+04	0.374	GPCR LIGAND BINDING
DRD5	6	5.99E+03	0.376	GPCR LIGAND BINDING
CALCR	6	4.50E+03	0.378	GPCR LIGAND BINDING
GRM4	2	8.27E+02	0.380	GPCR LIGAND BINDING
GNRHR	5	5.57E+03	0.387	GPCR LIGAND BINDING
TAAR8	2	1.09E+03	0.390	GPCR LIGAND BINDING
GPR31	4	2.12E+03	0.393	GPCR LIGAND BINDING
FZD3	3	1.34E+03	0.393	GPCR LIGAND BINDING
TAS2R46	2	9.42E+02	0.394	GPCR LIGAND BINDING
PROKR2	7	5.79E+03	0.396	GPCR LIGAND BINDING
GPR132	2	2.70E+03	0.398	GPCR LIGAND BINDING
DRD4	25	3.81E+04	0.398	GPCR LIGAND BINDING
SST	4	9.12E+03	0.401	GPCR LIGAND BINDING
NTS	3	1.62E+03	0.404	GPCR LIGAND BINDING
TAS2R30	23	4.50E+04	0.410	GPCR LIGAND BINDING
WNT3A	2	4.57E+02	0.412	GPCR LIGAND BINDING
NMB	1	3.51E+02	0.417	GPCR LIGAND BINDING
RAMP3	2	5.45E+02	0.427	GPCR LIGAND BINDING
FSHB	3	2.34E+03	0.432	GPCR LIGAND BINDING
TACR2	9	6.30E+03	0.433	GPCR LIGAND BINDING
ADGRE5	16	1.21E+04	0.437	GPCR LIGAND BINDING
C3AR1	2	3.22E+03	0.439	GPCR LIGAND BINDING
PLPPR1	2	3.09E+03	0.443	GPCR LIGAND BINDING
TAS2R16	4	1.56E+03	0.445	GPCR LIGAND BINDING
QRFP	3	7.08E+03	0.446	GPCR LIGAND BINDING
PENK	4	1.99E+03	0.448	GPCR LIGAND BINDING
CCR4	1	1.22E+02	0.449	GPCR LIGAND BINDING
CCRL2	5	8.35E+03	0.456	GPCR LIGAND BINDING
GABBR1	3	1.77E+03	0.462	GPCR LIGAND BINDING
PLPPR2	5	4.02E+03	0.464	GPCR LIGAND BINDING
TAS2R38	3	1.69E+03	0.465	GPCR LIGAND BINDING
LHCGR	2	1.24E+03	0.466	GPCR LIGAND BINDING
S1PR5	4	6.22E+03	0.470	GPCR LIGAND BINDING
SSTR3	13	6.32E+03	0.471	GPCR LIGAND BINDING
GPBAR1	5	2.77E+03	0.475	GPCR LIGAND BINDING
MC5R	3	2.21E+03	0.475	GPCR LIGAND BINDING
MC3R	2	9.04E+02	0.477	GPCR LIGAND BINDING

TAS1R3	16	1.18E+04	0.479	GPCR LIGAND BINDING
GPR39	7	5.36E+03	0.481	GPCR LIGAND BINDING
NPY2R	1	2.28E+02	0.482	GPCR LIGAND BINDING
PTCH1	17	9.47E+03	0.483	GPCR LIGAND BINDING
F2R	2	6.48E+02	0.484	GPCR LIGAND BINDING
SSTR5	14	5.90E+03	0.484	GPCR LIGAND BINDING
RXFP4	8	4.86E+03	0.485	GPCR LIGAND BINDING
UCN2	2	1.05E+03	0.488	GPCR LIGAND BINDING
KISS1	1	9.08E+01	0.488	GPCR LIGAND BINDING
PTH2	4	1.75E+03	0.499	GPCR LIGAND BINDING
ADRA1B	6	2.10E+03	0.501	GPCR LIGAND BINDING
GPR55	2	4.16E+02	0.503	GPCR LIGAND BINDING
CXCR6	2	1.13E+03	0.505	GPCR LIGAND BINDING
GALR2	4	6.54E+03	0.506	GPCR LIGAND BINDING
CCL16	1	1.49E+03	0.506	GPCR LIGAND BINDING
OPRK1	3	5.16E+02	0.507	GPCR LIGAND BINDING
NPW	3	7.17E+02	0.508	GPCR LIGAND BINDING
NPSR1	4	3.46E+03	0.509	GPCR LIGAND BINDING
UTS2	2	3.07E+03	0.510	GPCR LIGAND BINDING
CMKLR1	5	8.10E+03	0.511	GPCR LIGAND BINDING
NMU	3	2.95E+03	0.514	GPCR LIGAND BINDING
GRM3	4	3.25E+03	0.520	GPCR LIGAND BINDING
GHRL	3	4.65E+03	0.524	GPCR LIGAND BINDING
PLPPR3	10	7.52E+03	0.526	GPCR LIGAND BINDING
LTB4R2	4	1.01E+03	0.527	GPCR LIGAND BINDING
FFAR1	3	1.69E+03	0.530	GPCR LIGAND BINDING
FPR2	3	1.80E+03	0.531	GPCR LIGAND BINDING
F2RL2	4	8.44E+03	0.534	GPCR LIGAND BINDING
CCL13	2	3.88E+02	0.535	GPCR LIGAND BINDING
S1PR1	2	2.46E+03	0.537	GPCR LIGAND BINDING
PMCH	1	9.44E+01	0.539	GPCR LIGAND BINDING
WNT4	1	9.13E+01	0.540	GPCR LIGAND BINDING
FFAR2	6	2.79E+03	0.541	GPCR LIGAND BINDING
NPY5R	1	7.21E+01	0.542	GPCR LIGAND BINDING
ADCYAP1R1	8	3.48E+03	0.544	GPCR LIGAND BINDING
UCN	1	2.24E+02	0.549	GPCR LIGAND BINDING
TACR3	4	3.29E+03	0.549	GPCR LIGAND BINDING
TAS2R42	1	8.11E+01	0.550	GPCR LIGAND BINDING
PRLH	2	7.62E+02	0.552	GPCR LIGAND BINDING
CCKBR	2	5.36E+02	0.553	GPCR LIGAND BINDING
GPR35	9	1.59E+04	0.553	GPCR LIGAND BINDING
LPAR2	2	1.59E+03	0.554	GPCR LIGAND BINDING
CCR1	3	1.03E+03	0.554	GPCR LIGAND BINDING
HTR1D	5	1.45E+03	0.558	GPCR LIGAND BINDING
MCHR2	1	1.26E+03	0.564	GPCR LIGAND BINDING
GHSR	3	8.21E+02	0.565	GPCR LIGAND BINDING
ADRB3	5	1.22E+03	0.565	GPCR LIGAND BINDING
ADORA1	4	1.27E+03	0.566	GPCR LIGAND BINDING
TAAR5	11	7.90E+03	0.567	GPCR LIGAND BINDING
ADORA2A	4	7.09E+02	0.579	GPCR LIGAND BINDING
TAS2R31	9	2.08E+04	0.579	GPCR LIGAND BINDING
EDN2	2	8.08E+02	0.582	GPCR LIGAND BINDING
TAAR6	8	6.24E+03	0.583	GPCR LIGAND BINDING
HCRTR1	6	2.57E+03	0.584	GPCR LIGAND BINDING
ADRA1A	11	1.61E+04	0.586	GPCR LIGAND BINDING
OPRD1	2	4.06E+02	0.587	GPCR LIGAND BINDING
GPR68	3	6.43E+02	0.588	GPCR LIGAND BINDING
SCTR	6	6.15E+03	0.589	GPCR LIGAND BINDING
TRH	1	4.51E+02	0.591	GPCR LIGAND BINDING
FPR1	5	2.74E+03	0.591	GPCR LIGAND BINDING
SAA1	6	3.52E+03	0.597	GPCR LIGAND BINDING
CRHR1	4	7.95E+03	0.597	GPCR LIGAND BINDING
ADM	2	1.17E+03	0.598	GPCR LIGAND BINDING
P2RY13	2	3.58E+03	0.602	GPCR LIGAND BINDING
PROK1	1	3.60E+01	0.608	GPCR LIGAND BINDING
P2RY11	9	8.60E+03	0.608	GPCR LIGAND BINDING
PNOC	2	2.92E+02	0.610	GPCR LIGAND BINDING
TAAR1	1	5.50E+01	0.616	GPCR LIGAND BINDING
TAS2R40	4	7.39E+02	0.618	GPCR LIGAND BINDING
NPFF	2	4.01E+02	0.626	GPCR LIGAND BINDING
GNG12	2	4.72E+02	0.627	GPCR LIGAND BINDING
BDKRB1	6	2.20E+03	0.629	GPCR LIGAND BINDING
GNB5	1	3.69E+02	0.629	GPCR LIGAND BINDING
GLP2R	5	3.84E+03	0.631	GPCR LIGAND BINDING

FZD5	3	1.22E+03	0.633	GPCR LIGAND BINDING
GALR3	11	8.22E+03	0.634	GPCR LIGAND BINDING
KISS1R	2	5.28E+02	0.635	GPCR LIGAND BINDING
GABBR2	1	5.62E+01	0.635	GPCR LIGAND BINDING
PTHLH	1	4.74E+01	0.637	GPCR LIGAND BINDING
SCT	2	4.99E+02	0.646	GPCR LIGAND BINDING
TAS2R19	5	7.01E+03	0.647	GPCR LIGAND BINDING
PRLHR	3	3.97E+02	0.648	GPCR LIGAND BINDING
TAAR2	4	5.91E+02	0.650	GPCR LIGAND BINDING
TAS2R1	2	7.80E+02	0.653	GPCR LIGAND BINDING
CCKAR	3	1.40E+03	0.653	GPCR LIGAND BINDING
CASR	6	2.50E+03	0.656	GPCR LIGAND BINDING
OPN5	2	3.82E+02	0.658	GPCR LIGAND BINDING
MTNR1A	7	5.22E+03	0.662	GPCR LIGAND BINDING
P2RY12	2	2.55E+02	0.662	GPCR LIGAND BINDING
GRM8	3	4.28E+02	0.664	GPCR LIGAND BINDING
HCAR3	7	1.20E+03	0.665	GPCR LIGAND BINDING
OPRL1	12	7.90E+03	0.665	GPCR LIGAND BINDING
NPFFR2	4	6.97E+02	0.666	GPCR LIGAND BINDING
PTCH2	8	6.06E+03	0.668	GPCR LIGAND BINDING
HCRT	1	2.83E+01	0.670	GPCR LIGAND BINDING
FPR3	1	7.26E+01	0.671	GPCR LIGAND BINDING
S1PR3	8	6.21E+03	0.682	GPCR LIGAND BINDING
MCHR1	7	6.51E+03	0.683	GPCR LIGAND BINDING
CXCL16	5	1.84E+03	0.684	GPCR LIGAND BINDING
FZD10	4	5.13E+02	0.686	GPCR LIGAND BINDING
FZD6	4	1.31E+03	0.687	GPCR LIGAND BINDING
CCL28	1	5.41E+01	0.688	GPCR LIGAND BINDING
P2RY14	2	2.63E+02	0.689	GPCR LIGAND BINDING
F2RL3	9	2.92E+03	0.689	GPCR LIGAND BINDING
CCR5	10	4.76E+03	0.692	GPCR LIGAND BINDING
RLN3	1	2.75E+02	0.693	GPCR LIGAND BINDING
C3	10	2.84E+03	0.694	GPCR LIGAND BINDING
NPBWR2	7	2.09E+03	0.695	GPCR LIGAND BINDING
NPY1R	4	1.41E+03	0.698	GPCR LIGAND BINDING
CCR8	2	5.78E+02	0.698	GPCR LIGAND BINDING
TAS2R9	5	2.99E+03	0.701	GPCR LIGAND BINDING
TAS2R4	4	9.02E+02	0.703	GPCR LIGAND BINDING
HTR4	4	9.27E+02	0.703	GPCR LIGAND BINDING
CCR10	1	1.64E+02	0.705	GPCR LIGAND BINDING
EDN1	7	9.31E+02	0.706	GPCR LIGAND BINDING
ADGRE1	15	1.47E+04	0.709	GPCR LIGAND BINDING
MLN	4	7.34E+02	0.710	GPCR LIGAND BINDING
UTS2R	5	2.45E+03	0.712	GPCR LIGAND BINDING
HTR7	3	2.25E+03	0.713	GPCR LIGAND BINDING
CALCA	3	5.33E+03	0.716	GPCR LIGAND BINDING
ADORA3	3	1.40E+03	0.716	GPCR LIGAND BINDING
GRM1	9	1.10E+04	0.716	GPCR LIGAND BINDING
TAS2R20	1	2.15E+01	0.717	GPCR LIGAND BINDING
XCR1	2	6.45E+02	0.718	GPCR LIGAND BINDING
GNB2	1	3.27E+01	0.721	GPCR LIGAND BINDING
CXCR2	5	9.70E+02	0.723	GPCR LIGAND BINDING
CXCL10	1	3.16E+02	0.723	GPCR LIGAND BINDING
SSTR2	2	1.37E+02	0.730	GPCR LIGAND BINDING
NTSR1	15	7.46E+03	0.732	GPCR LIGAND BINDING
PYY	1	3.07E+01	0.741	GPCR LIGAND BINDING
GRM2	10	2.77E+03	0.744	GPCR LIGAND BINDING
HCAR2	4	7.46E+02	0.746	GPCR LIGAND BINDING
EDNRB	4	4.16E+03	0.746	GPCR LIGAND BINDING
GAL	2	9.09E+02	0.754	GPCR LIGAND BINDING
LPAR6	3	1.01E+03	0.761	GPCR LIGAND BINDING
FZD8	4	4.99E+02	0.762	GPCR LIGAND BINDING
OPN3	12	3.26E+03	0.770	GPCR LIGAND BINDING
TAS2R41	4	5.80E+02	0.773	GPCR LIGAND BINDING
TAS1R2	10	2.99E+03	0.773	GPCR LIGAND BINDING
NTSR2	7	2.75E+03	0.775	GPCR LIGAND BINDING
WNT2B	6	1.22E+03	0.777	GPCR LIGAND BINDING
LHB	3	1.75E+03	0.780	GPCR LIGAND BINDING
FZD2	4	1.70E+03	0.783	GPCR LIGAND BINDING
VIPR1	6	9.78E+02	0.785	GPCR LIGAND BINDING
ADORA2B	2	8.40E+01	0.789	GPCR LIGAND BINDING
RXFP1	3	2.71E+02	0.792	GPCR LIGAND BINDING
CX3CR1	7	2.48E+03	0.795	GPCR LIGAND BINDING
CXCL12	2	1.32E+02	0.797	GPCR LIGAND BINDING

WNT9B	4	6.78E+02	0.797	GPCR LIGAND BINDING
PDYN	5	1.17E+03	0.806	GPCR LIGAND BINDING
HCRTR2	4	1.35E+03	0.809	GPCR LIGAND BINDING
PTGDR2	4	1.53E+02	0.809	GPCR LIGAND BINDING
SHH	2	1.20E+02	0.810	GPCR LIGAND BINDING
AGTR1	4	5.93E+02	0.816	GPCR LIGAND BINDING
NPY4R	6	8.62E+02	0.818	GPCR LIGAND BINDING
OXER1	9	7.23E+03	0.819	GPCR LIGAND BINDING
RXFP2	8	4.25E+03	0.819	GPCR LIGAND BINDING
RHO	1	1.37E+01	0.821	GPCR LIGAND BINDING
ADGRE2	14	7.81E+03	0.823	GPCR LIGAND BINDING
AGT	12	1.42E+03	0.831	GPCR LIGAND BINDING
OXTR	5	1.39E+03	0.831	GPCR LIGAND BINDING
TAS2R60	2	3.80E+02	0.833	GPCR LIGAND BINDING
GLP1R	8	1.62E+03	0.834	GPCR LIGAND BINDING
HTR2A	4	1.62E+03	0.843	GPCR LIGAND BINDING
RGR	5	3.68E+02	0.844	GPCR LIGAND BINDING
SSTR1	1	5.80E+00	0.848	GPCR LIGAND BINDING
TAS2R5	7	2.55E+03	0.849	GPCR LIGAND BINDING
GNRH2	3	4.53E+02	0.850	GPCR LIGAND BINDING
ECE1	3	6.76E+02	0.852	GPCR LIGAND BINDING
EDN3	1	7.52E+00	0.854	GPCR LIGAND BINDING
HRH1	6	6.30E+02	0.857	GPCR LIGAND BINDING
HTR1F	1	4.98E+01	0.857	GPCR LIGAND BINDING
FZD1	3	2.57E+02	0.863	GPCR LIGAND BINDING
CALCRL	1	2.34E+01	0.864	GPCR LIGAND BINDING
GNG13	1	3.52E+00	0.869	GPCR LIGAND BINDING
GPR65	1	6.27E+00	0.870	GPCR LIGAND BINDING
PTGER2	2	1.14E+03	0.871	GPCR LIGAND BINDING
AVPR1A	4	1.37E+03	0.875	GPCR LIGAND BINDING
GNB4	1	2.78E+01	0.877	GPCR LIGAND BINDING
MTNR1B	6	4.84E+02	0.878	GPCR LIGAND BINDING
CHRM5	6	2.85E+02	0.885	GPCR LIGAND BINDING
MC1R	17	2.05E+03	0.891	GPCR LIGAND BINDING
GNG2	1	3.16E+00	0.894	GPCR LIGAND BINDING
TSHR	5	4.59E+02	0.895	GPCR LIGAND BINDING
INSL3	6	1.27E+03	0.899	GPCR LIGAND BINDING
PTGER3	1	1.97E+01	0.901	GPCR LIGAND BINDING
CD55	5	1.70E+02	0.906	GPCR LIGAND BINDING
HTR1E	1	7.94E+00	0.909	GPCR LIGAND BINDING
TAS2R8	7	9.41E+02	0.911	GPCR LIGAND BINDING
CHRM4	1	2.02E+00	0.914	GPCR LIGAND BINDING
AVP	1	7.11E-01	0.976	GPCR LIGAND BINDING
DRD3	1	1.26E-01	0.982	GPCR LIGAND BINDING
ADRA2C	1	5.32E-02	0.983	GPCR LIGAND BINDING
WNT6	1	1.78E-01	0.987	GPCR LIGAND BINDING
PTGER4	1	2.47E-01	0.993	GPCR LIGAND BINDING
CHRM3	3	2.38E+02	1	GPCR LIGAND BINDING
C5AR2	2	3.91E+01	1	GPCR LIGAND BINDING
TAS2R3	2	5.83E+01	1	GPCR LIGAND BINDING
HTR6	6	5.49E+01	1	GPCR LIGAND BINDING
OXGR1	2	1.50E+02	1	GPCR LIGAND BINDING
PTH2R	7	1.16E+02	1	GPCR LIGAND BINDING
P2RY2	6	1.00E+01	1	GPCR LIGAND BINDING
NMUR1	8	8.72E+01	1	GPCR LIGAND BINDING
ACKR1	5	1.40E+03	1	GPCR LIGAND BINDING
LPAR3	2	2.37E+01	1	GPCR LIGAND BINDING
WNT10A	9	2.00E+03	1	GPCR LIGAND BINDING
DRD1	2	6.12E-01	1	GPCR LIGAND BINDING
HRH4	2	5.02E+01	1	GPCR LIGAND BINDING
HCAR1	4	4.89E+01	1	GPCR LIGAND BINDING
ADGRE3	8	4.95E+02	1	GPCR LIGAND BINDING
GNGT1	2	9.38E-01	1	GPCR LIGAND BINDING
WNT1	3	7.98E+01	1	GPCR LIGAND BINDING
XCL2	3	6.32E+00	1	GPCR LIGAND BINDING
PPY	2	8.53E+00	1	GPCR LIGAND BINDING
WNT16	2	4.00E+01	1	GPCR LIGAND BINDING
ADRA2B	3	7.47E+01	1	GPCR LIGAND BINDING
ECE2	4	4.03E+01	1	GPCR LIGAND BINDING
TAS2R7	5	2.20E+03	1	GPCR LIGAND BINDING
XCL1	3	4.48E+02	1	GPCR LIGAND BINDING
BDKRB2	2	6.28E+00	1	GPCR LIGAND BINDING
TAS2R10	2	2.87E+02	1	GPCR LIGAND BINDING
GPR37L1	5	1.85E+02	1	GPCR LIGAND BINDING

NMS	2	1.15E+00	1	GPCR LIGAND BINDING
FZD9	2	1.43E+01	1	GPCR LIGAND BINDING

Gene	NumVar	Q	Pvalue	Gene-set
ITK	5	2.47E+04	9.38E-05	INNATE IMMUNE SYSTEM
PTPRB	25	8.07E+04	0.001	INNATE IMMUNE SYSTEM
TLR1	14	6.77E+04	0.001	INNATE IMMUNE SYSTEM
FLG2	121	2.38E+05	0.003	INNATE IMMUNE SYSTEM
CEACAM8	9	4.74E+04	0.004	INNATE IMMUNE SYSTEM
MUC4	870	1.68E+07	0.006	INNATE IMMUNE SYSTEM
PRKCE	3	2.09E+04	0.008	INNATE IMMUNE SYSTEM
SERPINB12	4	3.11E+04	0.008	INNATE IMMUNE SYSTEM
SFTPA1	6	3.50E+04	0.009	INNATE IMMUNE SYSTEM
BST1	5	4.91E+04	0.010	INNATE IMMUNE SYSTEM
DSC1	4	1.17E+04	0.010	INNATE IMMUNE SYSTEM
VAMP8	2	3.94E+03	0.012	INNATE IMMUNE SYSTEM
LCN2	3	8.67E+03	0.012	INNATE IMMUNE SYSTEM
PKP1	11	5.12E+04	0.013	INNATE IMMUNE SYSTEM
LRG1	5	1.13E+04	0.013	INNATE IMMUNE SYSTEM
IRAK3	6	3.71E+04	0.015	INNATE IMMUNE SYSTEM
RNASE3	4	2.06E+04	0.015	INNATE IMMUNE SYSTEM
ATP11B	7	2.55E+04	0.016	INNATE IMMUNE SYSTEM
PRG2	4	7.11E+03	0.016	INNATE IMMUNE SYSTEM
C5AR1	7	4.11E+04	0.018	INNATE IMMUNE SYSTEM
NOD1	8	1.04E+04	0.018	INNATE IMMUNE SYSTEM
CAMP	3	2.50E+04	0.019	INNATE IMMUNE SYSTEM
MGST1	4	9.99E+03	0.019	INNATE IMMUNE SYSTEM
APAF1	13	3.41E+04	0.020	INNATE IMMUNE SYSTEM
RAB44	19	9.95E+04	0.021	INNATE IMMUNE SYSTEM
APP	2	3.42E+03	0.021	INNATE IMMUNE SYSTEM
CASP10	5	1.53E+04	0.022	INNATE IMMUNE SYSTEM
B4GALT1	1	1.34E+03	0.022	INNATE IMMUNE SYSTEM
VCP	2	3.84E+03	0.022	INNATE IMMUNE SYSTEM
POLR3GL	2	5.78E+03	0.022	INNATE IMMUNE SYSTEM
KCNAB2	3	1.01E+04	0.023	INNATE IMMUNE SYSTEM
GSDMD	4	8.67E+03	0.023	INNATE IMMUNE SYSTEM
RNASET2	2	1.25E+04	0.024	INNATE IMMUNE SYSTEM
ACTR10	2	4.89E+03	0.029	INNATE IMMUNE SYSTEM
PRTN3	4	1.84E+04	0.030	INNATE IMMUNE SYSTEM
ITLN1	3	9.72E+03	0.033	INNATE IMMUNE SYSTEM
MIF	2	3.91E+03	0.035	INNATE IMMUNE SYSTEM
DYNC1LI1	5	2.20E+04	0.038	INNATE IMMUNE SYSTEM
YPEL5	1	7.55E+02	0.040	INNATE IMMUNE SYSTEM
TBK1	4	1.84E+04	0.040	INNATE IMMUNE SYSTEM
POLR2F	3	2.15E+04	0.041	INNATE IMMUNE SYSTEM
ARG1	1	2.49E+03	0.043	INNATE IMMUNE SYSTEM
NF2	1	7.04E+02	0.043	INNATE IMMUNE SYSTEM
NLRP3	5	1.80E+04	0.044	INNATE IMMUNE SYSTEM
TXK	2	1.13E+04	0.045	INNATE IMMUNE SYSTEM
TRIM21	6	1.46E+04	0.047	INNATE IMMUNE SYSTEM
F2	5	7.45E+03	0.047	INNATE IMMUNE SYSTEM
TRIM32	4	1.53E+04	0.049	INNATE IMMUNE SYSTEM
MUC17	222	2.02E+06	0.049	INNATE IMMUNE SYSTEM
DEFA6	2	4.27E+03	0.049	INNATE IMMUNE SYSTEM
FCAR	1	7.05E+02	0.050	INNATE IMMUNE SYSTEM
CLEC12A	3	4.36E+03	0.051	INNATE IMMUNE SYSTEM
DEFB112	1	1.36E+03	0.056	INNATE IMMUNE SYSTEM
IFNA21	3	1.61E+04	0.058	INNATE IMMUNE SYSTEM
KPNB1	2	1.18E+04	0.058	INNATE IMMUNE SYSTEM
MYO10	14	1.11E+05	0.058	INNATE IMMUNE SYSTEM
MEF2A	4	9.97E+03	0.061	INNATE IMMUNE SYSTEM
HLA-E	12	7.25E+04	0.061	INNATE IMMUNE SYSTEM
IFNB1	3	2.18E+03	0.062	INNATE IMMUNE SYSTEM
DEFA1	1	6.46E+02	0.062	INNATE IMMUNE SYSTEM
DEFA3	1	6.46E+02	0.062	INNATE IMMUNE SYSTEM
DEFA1B	1	6.46E+02	0.062	INNATE IMMUNE SYSTEM
STOM	2	4.86E+03	0.063	INNATE IMMUNE SYSTEM
MAVS	5	5.63E+03	0.064	INNATE IMMUNE SYSTEM
GRAP2	3	1.21E+04	0.066	INNATE IMMUNE SYSTEM
DEFB116	2	1.52E+04	0.067	INNATE IMMUNE SYSTEM
OLFM4	4	1.68E+04	0.068	INNATE IMMUNE SYSTEM
C1S	4	1.10E+04	0.068	INNATE IMMUNE SYSTEM
MAP2K3	1	3.09E+03	0.072	INNATE IMMUNE SYSTEM
DEFB124	2	7.70E+03	0.072	INNATE IMMUNE SYSTEM

CDK13	9	3.51E+04	0.074	INNATE IMMUNE SYSTEM
TREM2	7	1.71E+04	0.074	INNATE IMMUNE SYSTEM
CREBBP	19	3.85E+04	0.075	INNATE IMMUNE SYSTEM
MAN2B1	13	2.40E+04	0.077	INNATE IMMUNE SYSTEM
ITPR1	13	3.91E+04	0.079	INNATE IMMUNE SYSTEM
GPI	3	1.32E+04	0.081	INNATE IMMUNE SYSTEM
CSTB	1	2.44E+03	0.082	INNATE IMMUNE SYSTEM
RPS27A	1	2.69E+03	0.087	INNATE IMMUNE SYSTEM
ADGRG3	8	6.29E+03	0.087	INNATE IMMUNE SYSTEM
ITCH	11	1.79E+04	0.088	INNATE IMMUNE SYSTEM
CCR2	3	1.69E+03	0.088	INNATE IMMUNE SYSTEM
COTL1	1	1.42E+03	0.089	INNATE IMMUNE SYSTEM
CD4	5	1.48E+04	0.089	INNATE IMMUNE SYSTEM
PRCP	7	1.10E+04	0.089	INNATE IMMUNE SYSTEM
CAND1	4	3.76E+03	0.090	INNATE IMMUNE SYSTEM
RNF135	10	3.45E+04	0.091	INNATE IMMUNE SYSTEM
TMBIM1	4	1.63E+04	0.093	INNATE IMMUNE SYSTEM
HSPA1A	2	1.72E+03	0.094	INNATE IMMUNE SYSTEM
CYFIP1	12	3.83E+04	0.094	INNATE IMMUNE SYSTEM
GAB2	3	3.48E+03	0.096	INNATE IMMUNE SYSTEM
RPS6KA1	4	6.96E+03	0.097	INNATE IMMUNE SYSTEM
POLR1D	1	9.24E+03	0.097	INNATE IMMUNE SYSTEM
PSAP	3	3.19E+03	0.097	INNATE IMMUNE SYSTEM
ITGAV	8	6.33E+03	0.100	INNATE IMMUNE SYSTEM
RELA	3	8.06E+03	0.100	INNATE IMMUNE SYSTEM
TAX1BP1	5	1.69E+04	0.100	INNATE IMMUNE SYSTEM
CD33	7	2.10E+04	0.101	INNATE IMMUNE SYSTEM
FCER1G	1	1.28E+03	0.103	INNATE IMMUNE SYSTEM
MUC3A	58	4.50E+05	0.104	INNATE IMMUNE SYSTEM
APOB	42	8.05E+04	0.105	INNATE IMMUNE SYSTEM
TKFC	8	1.43E+04	0.106	INNATE IMMUNE SYSTEM
TAB1	3	2.23E+03	0.109	INNATE IMMUNE SYSTEM
PIK3CA	2	7.43E+03	0.109	INNATE IMMUNE SYSTEM
POLR3C	4	1.15E+04	0.109	INNATE IMMUNE SYSTEM
SOS1	5	1.70E+04	0.112	INNATE IMMUNE SYSTEM
MMP8	6	8.71E+03	0.114	INNATE IMMUNE SYSTEM
DNM3	3	2.15E+03	0.118	INNATE IMMUNE SYSTEM
SYNGR1	4	6.52E+03	0.120	INNATE IMMUNE SYSTEM
PRKACB	2	2.25E+03	0.121	INNATE IMMUNE SYSTEM
MUC16	190	2.15E+05	0.122	INNATE IMMUNE SYSTEM
CREG1	2	1.15E+03	0.123	INNATE IMMUNE SYSTEM
METTL7A	2	2.28E+03	0.129	INNATE IMMUNE SYSTEM
DEFB105A	1	7.53E+02	0.131	INNATE IMMUNE SYSTEM
DEFB105B	1	7.53E+02	0.131	INNATE IMMUNE SYSTEM
ZBP1	8	2.83E+04	0.134	INNATE IMMUNE SYSTEM
PLD1	14	3.67E+04	0.136	INNATE IMMUNE SYSTEM
MAP3K14	6	1.35E+04	0.137	INNATE IMMUNE SYSTEM
RAB3D	3	3.42E+03	0.137	INNATE IMMUNE SYSTEM
AHCYL1	1	6.32E+02	0.138	INNATE IMMUNE SYSTEM
CD44	6	1.28E+04	0.138	INNATE IMMUNE SYSTEM
PAFAH1B2	2	8.57E+03	0.139	INNATE IMMUNE SYSTEM
CASP4	4	4.33E+03	0.141	INNATE IMMUNE SYSTEM
HERC5	7	9.12E+03	0.143	INNATE IMMUNE SYSTEM
QPCT	5	1.33E+04	0.144	INNATE IMMUNE SYSTEM
HSPA1B	3	4.65E+03	0.148	INNATE IMMUNE SYSTEM
ABI1	3	1.85E+03	0.148	INNATE IMMUNE SYSTEM
TMEM179B	1	3.76E+02	0.149	INNATE IMMUNE SYSTEM
ATP6V0D2	4	2.48E+03	0.149	INNATE IMMUNE SYSTEM
IMPDH1	9	1.35E+04	0.150	INNATE IMMUNE SYSTEM
DNAJC3	2	1.63E+03	0.151	INNATE IMMUNE SYSTEM
CFHR4	11	1.63E+04	0.152	INNATE IMMUNE SYSTEM
C6	15	4.10E+04	0.152	INNATE IMMUNE SYSTEM
RNF125	2	2.12E+03	0.153	INNATE IMMUNE SYSTEM
MS4A2	4	9.39E+03	0.155	INNATE IMMUNE SYSTEM
CRCP	2	1.30E+03	0.156	INNATE IMMUNE SYSTEM
LIMK1	4	4.10E+03	0.157	INNATE IMMUNE SYSTEM
CCT2	1	3.77E+02	0.158	INNATE IMMUNE SYSTEM
IFNA16	3	9.57E+03	0.159	INNATE IMMUNE SYSTEM
ATP6V1G1	1	3.06E+03	0.160	INNATE IMMUNE SYSTEM
CYB5R3	4	4.99E+03	0.160	INNATE IMMUNE SYSTEM
TLR10	11	2.53E+04	0.161	INNATE IMMUNE SYSTEM
P2RX1	2	1.55E+03	0.162	INNATE IMMUNE SYSTEM
IFNA4	5	6.09E+03	0.163	INNATE IMMUNE SYSTEM
PDAP1	2	2.73E+03	0.164	INNATE IMMUNE SYSTEM

CRACR2A	10	1.53E+04	0.164	INNATE IMMUNE SYSTEM
DEGS1	3	1.05E+04	0.165	INNATE IMMUNE SYSTEM
ASAH1	10	1.65E+04	0.167	INNATE IMMUNE SYSTEM
HEXB	4	9.65E+03	0.169	INNATE IMMUNE SYSTEM
CMTM6	2	1.50E+04	0.170	INNATE IMMUNE SYSTEM
CPB2	1	3.61E+02	0.171	INNATE IMMUNE SYSTEM
SDCBP	2	6.14E+02	0.176	INNATE IMMUNE SYSTEM
NCKAP1L	7	8.43E+03	0.179	INNATE IMMUNE SYSTEM
HPSE	4	8.42E+03	0.180	INNATE IMMUNE SYSTEM
MASP2	13	2.01E+04	0.183	INNATE IMMUNE SYSTEM
PLD2	12	2.39E+04	0.183	INNATE IMMUNE SYSTEM
SPTAN1	7	9.73E+03	0.184	INNATE IMMUNE SYSTEM
PIK3R1	1	3.44E+02	0.185	INNATE IMMUNE SYSTEM
CXCR1	8	1.15E+04	0.187	INNATE IMMUNE SYSTEM
IFIH1	18	3.25E+04	0.190	INNATE IMMUNE SYSTEM
MAP2K7	1	1.09E+03	0.190	INNATE IMMUNE SYSTEM
SIGLEC15	7	8.60E+03	0.192	INNATE IMMUNE SYSTEM
CHIT1	4	9.19E+03	0.193	INNATE IMMUNE SYSTEM
SERPING1	6	4.88E+03	0.193	INNATE IMMUNE SYSTEM
PDXK	4	3.27E+03	0.195	INNATE IMMUNE SYSTEM
CR2	12	1.96E+04	0.195	INNATE IMMUNE SYSTEM
COLEC10	3	2.89E+03	0.196	INNATE IMMUNE SYSTEM
LILRB2	32	2.02E+05	0.196	INNATE IMMUNE SYSTEM
CRISPLD2	8	2.20E+04	0.197	INNATE IMMUNE SYSTEM
LPO	7	4.56E+03	0.198	INNATE IMMUNE SYSTEM
NAPRT	7	7.44E+03	0.199	INNATE IMMUNE SYSTEM
CEACAM1	10	2.58E+04	0.199	INNATE IMMUNE SYSTEM
ROCK1	3	1.09E+04	0.202	INNATE IMMUNE SYSTEM
CAT	2	3.89E+03	0.202	INNATE IMMUNE SYSTEM
LAT2	4	9.49E+03	0.202	INNATE IMMUNE SYSTEM
ADAM10	2	1.51E+03	0.202	INNATE IMMUNE SYSTEM
NFKBIB	6	8.27E+03	0.203	INNATE IMMUNE SYSTEM
TLR2	9	1.23E+04	0.205	INNATE IMMUNE SYSTEM
HMOX2	4	4.91E+03	0.205	INNATE IMMUNE SYSTEM
PSEN1	1	5.96E+03	0.209	INNATE IMMUNE SYSTEM
HSP90AB1	2	8.68E+02	0.211	INNATE IMMUNE SYSTEM
PRKCD	5	4.92E+03	0.211	INNATE IMMUNE SYSTEM
TMEM63A	9	1.25E+04	0.213	INNATE IMMUNE SYSTEM
NLRC4	3	1.14E+04	0.214	INNATE IMMUNE SYSTEM
WIPF1	6	4.34E+03	0.214	INNATE IMMUNE SYSTEM
PLCG1	4	2.21E+03	0.215	INNATE IMMUNE SYSTEM
BIRC2	1	1.56E+03	0.217	INNATE IMMUNE SYSTEM
MUC5B	234	3.54E+05	0.221	INNATE IMMUNE SYSTEM
WIPF2	2	4.10E+03	0.221	INNATE IMMUNE SYSTEM
FOLR3	3	2.23E+03	0.223	INNATE IMMUNE SYSTEM
IQGAP1	8	1.26E+04	0.226	INNATE IMMUNE SYSTEM
LEAP2	1	2.81E+02	0.227	INNATE IMMUNE SYSTEM
RAB27A	6	1.83E+03	0.227	INNATE IMMUNE SYSTEM
NCF1	3	8.53E+03	0.229	INNATE IMMUNE SYSTEM
DEFB118	3	4.08E+03	0.230	INNATE IMMUNE SYSTEM
CD14	1	5.36E+02	0.232	INNATE IMMUNE SYSTEM
PRSS2	15	4.17E+04	0.232	INNATE IMMUNE SYSTEM
XRCC6	1	4.10E+02	0.232	INNATE IMMUNE SYSTEM
NFATC2	4	7.85E+03	0.232	INNATE IMMUNE SYSTEM
MUC6	40	7.95E+04	0.234	INNATE IMMUNE SYSTEM
IKBKB	6	3.11E+03	0.234	INNATE IMMUNE SYSTEM
WIPF3	9	1.33E+04	0.236	INNATE IMMUNE SYSTEM
SIRPA	5	1.11E+04	0.237	INNATE IMMUNE SYSTEM
IFI16	12	3.80E+04	0.239	INNATE IMMUNE SYSTEM
POLR2H	1	2.97E+03	0.240	INNATE IMMUNE SYSTEM
OLR1	1	3.17E+02	0.240	INNATE IMMUNE SYSTEM
TNFRSF1B	3	1.83E+03	0.242	INNATE IMMUNE SYSTEM
NFASC	15	4.25E+04	0.244	INNATE IMMUNE SYSTEM
SLC11A1	11	1.91E+04	0.244	INNATE IMMUNE SYSTEM
DEFB128	3	7.02E+03	0.244	INNATE IMMUNE SYSTEM
CHUK	4	4.16E+03	0.245	INNATE IMMUNE SYSTEM
DBNL	6	1.02E+04	0.246	INNATE IMMUNE SYSTEM
SFTPD	1	1.40E+03	0.247	INNATE IMMUNE SYSTEM
PPP3CA	2	8.30E+02	0.249	INNATE IMMUNE SYSTEM
CST3	1	4.78E+02	0.251	INNATE IMMUNE SYSTEM
AOC1	5	3.53E+03	0.252	INNATE IMMUNE SYSTEM
FABP5	1	1.56E+03	0.254	INNATE IMMUNE SYSTEM
AIM2	3	3.46E+03	0.254	INNATE IMMUNE SYSTEM
RNASE7	2	1.62E+03	0.255	INNATE IMMUNE SYSTEM

CD180	9	1.30E+04	0.256	INNATE IMMUNE SYSTEM
ARPC2	2	7.09E+02	0.260	INNATE IMMUNE SYSTEM
C6orf120	6	7.47E+03	0.261	INNATE IMMUNE SYSTEM
ELMO1	7	9.12E+03	0.264	INNATE IMMUNE SYSTEM
EEF2	1	2.17E+02	0.267	INNATE IMMUNE SYSTEM
TLR6	11	1.91E+04	0.268	INNATE IMMUNE SYSTEM
MUC21	84	6.98E+05	0.268	INNATE IMMUNE SYSTEM
PSMB6	1	3.10E+03	0.270	INNATE IMMUNE SYSTEM
ALDH3B1	7	4.36E+03	0.271	INNATE IMMUNE SYSTEM
ATP6V0E2	6	2.91E+03	0.273	INNATE IMMUNE SYSTEM
LRRC7	4	6.65E+03	0.279	INNATE IMMUNE SYSTEM
RAB4B	3	8.82E+02	0.281	INNATE IMMUNE SYSTEM
ATP6V1E2	2	2.78E+03	0.281	INNATE IMMUNE SYSTEM
MANBA	4	6.91E+03	0.283	INNATE IMMUNE SYSTEM
MBL2	2	5.35E+03	0.283	INNATE IMMUNE SYSTEM
HP	1	1.98E+03	0.284	INNATE IMMUNE SYSTEM
CFB	5	1.19E+04	0.285	INNATE IMMUNE SYSTEM
BTRC	3	1.17E+03	0.285	INNATE IMMUNE SYSTEM
TIRAP	3	8.35E+03	0.288	INNATE IMMUNE SYSTEM
IFNA8	2	6.26E+02	0.288	INNATE IMMUNE SYSTEM
RASGRP4	5	1.20E+04	0.292	INNATE IMMUNE SYSTEM
DEFB135	2	6.17E+02	0.294	INNATE IMMUNE SYSTEM
C5	12	1.23E+04	0.294	INNATE IMMUNE SYSTEM
LAT	2	4.60E+02	0.296	INNATE IMMUNE SYSTEM
RNASE2	1	6.38E+02	0.298	INNATE IMMUNE SYSTEM
ACLY	9	4.56E+03	0.298	INNATE IMMUNE SYSTEM
SLCO4C1	5	2.16E+03	0.299	INNATE IMMUNE SYSTEM
PIK3R2	7	5.87E+03	0.300	INNATE IMMUNE SYSTEM
C1R	7	5.30E+03	0.301	INNATE IMMUNE SYSTEM
DEFB108B	1	7.13E+02	0.301	INNATE IMMUNE SYSTEM
PRKDC	32	3.16E+04	0.301	INNATE IMMUNE SYSTEM
ATP6V1C2	5	2.72E+03	0.303	INNATE IMMUNE SYSTEM
PLA2G6	13	1.27E+04	0.304	INNATE IMMUNE SYSTEM
NCKIPSD	8	1.58E+04	0.304	INNATE IMMUNE SYSTEM
MAPK7	5	3.72E+03	0.306	INNATE IMMUNE SYSTEM
CCR6	1	3.00E+03	0.307	INNATE IMMUNE SYSTEM
MUC5AC	201	2.34E+05	0.307	INNATE IMMUNE SYSTEM
CXCL1	1	7.24E+02	0.308	INNATE IMMUNE SYSTEM
NCKAP1	2	5.37E+02	0.312	INNATE IMMUNE SYSTEM
ATP6V1G3	3	1.44E+03	0.313	INNATE IMMUNE SYSTEM
JUN	1	4.28E+02	0.314	INNATE IMMUNE SYSTEM
MUC20	14	2.38E+04	0.315	INNATE IMMUNE SYSTEM
ATP8A1	6	1.28E+04	0.315	INNATE IMMUNE SYSTEM
CEACAM3	10	4.68E+04	0.316	INNATE IMMUNE SYSTEM
SEMG1	12	2.89E+04	0.317	INNATE IMMUNE SYSTEM
MUC13	5	7.88E+03	0.317	INNATE IMMUNE SYSTEM
TCN1	4	5.72E+03	0.320	INNATE IMMUNE SYSTEM
EEA1	14	1.83E+04	0.322	INNATE IMMUNE SYSTEM
NOD2	32	2.88E+04	0.323	INNATE IMMUNE SYSTEM
MYD88	2	5.52E+02	0.323	INNATE IMMUNE SYSTEM
TRAF2	2	1.30E+03	0.323	INNATE IMMUNE SYSTEM
CNPY3	1	1.92E+02	0.324	INNATE IMMUNE SYSTEM
CLEC6A	4	6.22E+03	0.325	INNATE IMMUNE SYSTEM
GSTP1	5	1.88E+03	0.329	INNATE IMMUNE SYSTEM
CYBA	2	2.95E+03	0.329	INNATE IMMUNE SYSTEM
NFATC1	14	1.41E+04	0.329	INNATE IMMUNE SYSTEM
LGALS3	4	2.30E+03	0.330	INNATE IMMUNE SYSTEM
CD300LB	1	1.51E+02	0.330	INNATE IMMUNE SYSTEM
MAP3K8	2	8.52E+02	0.330	INNATE IMMUNE SYSTEM
CTSZ	3	3.74E+03	0.331	INNATE IMMUNE SYSTEM
MGAM	37	3.44E+04	0.333	INNATE IMMUNE SYSTEM
EPPIN	2	3.62E+03	0.336	INNATE IMMUNE SYSTEM
EPPIN-WFDC6	2	3.62E+03	0.336	INNATE IMMUNE SYSTEM
IFNA10	8	1.60E+04	0.337	INNATE IMMUNE SYSTEM
CNN2	2	6.90E+02	0.337	INNATE IMMUNE SYSTEM
DSP	23	2.38E+04	0.339	INNATE IMMUNE SYSTEM
MYO9B	22	2.45E+04	0.339	INNATE IMMUNE SYSTEM
CPNE1	8	1.01E+04	0.340	INNATE IMMUNE SYSTEM
PTAFR	3	2.22E+03	0.341	INNATE IMMUNE SYSTEM
GMFG	2	1.06E+03	0.341	INNATE IMMUNE SYSTEM
GM2A	3	4.62E+03	0.343	INNATE IMMUNE SYSTEM
HLA-C	4	3.98E+03	0.343	INNATE IMMUNE SYSTEM
FGL2	1	4.56E+02	0.344	INNATE IMMUNE SYSTEM
C1QA	3	2.79E+03	0.345	INNATE IMMUNE SYSTEM

UBR4	22	1.96E+04	0.346	INNATE IMMUNE SYSTEM
EPX	8	1.51E+04	0.346	INNATE IMMUNE SYSTEM
GNS	3	1.13E+03	0.346	INNATE IMMUNE SYSTEM
NPC2	4	6.14E+03	0.348	INNATE IMMUNE SYSTEM
PSMC5	1	2.08E+02	0.350	INNATE IMMUNE SYSTEM
TCIRG1	12	1.37E+04	0.354	INNATE IMMUNE SYSTEM
WASF3	7	1.35E+04	0.355	INNATE IMMUNE SYSTEM
AP2A2	5	3.59E+03	0.360	INNATE IMMUNE SYSTEM
MVP	6	4.23E+03	0.360	INNATE IMMUNE SYSTEM
CHI3L1	5	2.95E+03	0.360	INNATE IMMUNE SYSTEM
PKM	6	1.19E+04	0.361	INNATE IMMUNE SYSTEM
PECAM1	4	3.54E+03	0.361	INNATE IMMUNE SYSTEM
MYH2	11	1.60E+04	0.361	INNATE IMMUNE SYSTEM
C1orf35	2	9.06E+03	0.362	INNATE IMMUNE SYSTEM
APEH	4	9.74E+02	0.363	INNATE IMMUNE SYSTEM
CARD9	6	7.94E+03	0.368	INNATE IMMUNE SYSTEM
TICAM2	2	5.57E+02	0.368	INNATE IMMUNE SYSTEM
RIPK3	6	2.66E+03	0.368	INNATE IMMUNE SYSTEM
HMGB1	3	1.96E+03	0.370	INNATE IMMUNE SYSTEM
TNIP2	7	8.26E+03	0.372	INNATE IMMUNE SYSTEM
TLR3	6	3.26E+03	0.373	INNATE IMMUNE SYSTEM
TXNIP	4	5.60E+03	0.373	INNATE IMMUNE SYSTEM
LY86	5	2.45E+03	0.374	INNATE IMMUNE SYSTEM
POLR1C	11	4.35E+03	0.375	INNATE IMMUNE SYSTEM
NLRP1	19	3.05E+04	0.376	INNATE IMMUNE SYSTEM
S100A11	1	6.98E+02	0.376	INNATE IMMUNE SYSTEM
OSCAR	4	9.72E+03	0.376	INNATE IMMUNE SYSTEM
MYO5A	10	1.11E+04	0.378	INNATE IMMUNE SYSTEM
CLEC4A	1	2.61E+03	0.380	INNATE IMMUNE SYSTEM
QSOX1	7	1.18E+04	0.383	INNATE IMMUNE SYSTEM
LY96	2	3.84E+03	0.384	INNATE IMMUNE SYSTEM
CTSS	1	2.34E+02	0.384	INNATE IMMUNE SYSTEM
ANXA2	2	8.14E+02	0.386	INNATE IMMUNE SYSTEM
DEFB119	4	2.67E+03	0.387	INNATE IMMUNE SYSTEM
IGF2R	28	1.97E+04	0.387	INNATE IMMUNE SYSTEM
PLAUR	7	7.17E+03	0.389	INNATE IMMUNE SYSTEM
DHX58	8	1.48E+04	0.389	INNATE IMMUNE SYSTEM
MALT1	5	1.87E+03	0.391	INNATE IMMUNE SYSTEM
LGMN	4	6.40E+03	0.392	INNATE IMMUNE SYSTEM
MUC15	4	4.94E+03	0.392	INNATE IMMUNE SYSTEM
FCN2	4	6.43E+03	0.392	INNATE IMMUNE SYSTEM
BAIAP2	1	2.86E+02	0.393	INNATE IMMUNE SYSTEM
FCER1A	6	7.08E+03	0.393	INNATE IMMUNE SYSTEM
NOS1	17	1.54E+04	0.393	INNATE IMMUNE SYSTEM
ITPR2	10	9.65E+03	0.393	INNATE IMMUNE SYSTEM
STBD1	4	1.70E+03	0.393	INNATE IMMUNE SYSTEM
SIRPB1	7	7.39E+03	0.393	INNATE IMMUNE SYSTEM
ATF2	2	8.12E+02	0.394	INNATE IMMUNE SYSTEM
FYN	5	5.77E+03	0.395	INNATE IMMUNE SYSTEM
SCAMP1	1	9.50E+01	0.396	INNATE IMMUNE SYSTEM
MAPKAPK3	1	4.60E+02	0.396	INNATE IMMUNE SYSTEM
PIK3C3	1	2.37E+02	0.396	INNATE IMMUNE SYSTEM
DEFB129	2	1.53E+03	0.396	INNATE IMMUNE SYSTEM
PPIE	5	1.05E+04	0.398	INNATE IMMUNE SYSTEM
HTN3	1	2.00E+03	0.401	INNATE IMMUNE SYSTEM
SERPINA3	4	3.96E+03	0.401	INNATE IMMUNE SYSTEM
FTH1	1	5.83E+02	0.409	INNATE IMMUNE SYSTEM
PLEKHO2	8	1.53E+04	0.409	INNATE IMMUNE SYSTEM
HTN1	2	1.21E+03	0.410	INNATE IMMUNE SYSTEM
DEFA5	3	7.11E+03	0.410	INNATE IMMUNE SYSTEM
GRN	7	4.24E+03	0.412	INNATE IMMUNE SYSTEM
DHX36	3	1.79E+03	0.413	INNATE IMMUNE SYSTEM
PSMB11	7	1.01E+04	0.414	INNATE IMMUNE SYSTEM
PSMB9	5	8.02E+03	0.415	INNATE IMMUNE SYSTEM
BPI	9	1.46E+04	0.415	INNATE IMMUNE SYSTEM
LAMTOR1	1	1.84E+02	0.415	INNATE IMMUNE SYSTEM
FCGR1A	2	5.09E+02	0.417	INNATE IMMUNE SYSTEM
DDX58	7	1.02E+04	0.417	INNATE IMMUNE SYSTEM
PTPN4	5	1.03E+04	0.418	INNATE IMMUNE SYSTEM
UBE2M	1	8.16E+02	0.418	INNATE IMMUNE SYSTEM
PSMB1	1	1.16E+02	0.419	INNATE IMMUNE SYSTEM
PGM2	7	6.37E+03	0.419	INNATE IMMUNE SYSTEM
CPN1	1	5.03E+02	0.420	INNATE IMMUNE SYSTEM
C4BPB	1	2.15E+02	0.421	INNATE IMMUNE SYSTEM

IFNA5	2	2.05E+03	0.423	INNATE IMMUNE SYSTEM
SYK	1	2.60E+02	0.423	INNATE IMMUNE SYSTEM
TXN	1	6.38E+02	0.423	INNATE IMMUNE SYSTEM
CEACAM6	7	4.46E+03	0.425	INNATE IMMUNE SYSTEM
GZMM	7	6.41E+03	0.427	INNATE IMMUNE SYSTEM
RBSN	5	4.24E+03	0.427	INNATE IMMUNE SYSTEM
CYFIP2	8	7.94E+03	0.427	INNATE IMMUNE SYSTEM
DDX41	1	2.28E+02	0.428	INNATE IMMUNE SYSTEM
SLC2A3	2	1.07E+03	0.430	INNATE IMMUNE SYSTEM
NCF4	7	3.52E+03	0.432	INNATE IMMUNE SYSTEM
SLC15A4	3	3.13E+03	0.432	INNATE IMMUNE SYSTEM
ADA2	5	4.39E+03	0.432	INNATE IMMUNE SYSTEM
SURF4	1	1.29E+02	0.432	INNATE IMMUNE SYSTEM
PSMB8	4	5.06E+03	0.432	INNATE IMMUNE SYSTEM
TLR5	7	3.74E+04	0.433	INNATE IMMUNE SYSTEM
CD177	12	1.10E+04	0.436	INNATE IMMUNE SYSTEM
DEFB1	3	8.11E+02	0.437	INNATE IMMUNE SYSTEM
ADGRE5	16	1.21E+04	0.437	INNATE IMMUNE SYSTEM
C3AR1	2	3.22E+03	0.439	INNATE IMMUNE SYSTEM
PTPRN2	17	2.07E+04	0.439	INNATE IMMUNE SYSTEM
GSN	14	1.59E+04	0.440	INNATE IMMUNE SYSTEM
CRISP3	3	6.83E+02	0.440	INNATE IMMUNE SYSTEM
PGM1	6	1.86E+03	0.441	INNATE IMMUNE SYSTEM
WASF2	4	4.25E+03	0.441	INNATE IMMUNE SYSTEM
LRMP	3	5.48E+02	0.443	INNATE IMMUNE SYSTEM
PLD4	4	1.23E+03	0.445	INNATE IMMUNE SYSTEM
C8G	4	2.08E+03	0.446	INNATE IMMUNE SYSTEM
PSMA6	1	3.60E+02	0.447	INNATE IMMUNE SYSTEM
SERPINB3	9	1.73E+04	0.448	INNATE IMMUNE SYSTEM
GAA	10	9.46E+03	0.449	INNATE IMMUNE SYSTEM
KLRD1	1	1.85E+02	0.450	INNATE IMMUNE SYSTEM
CPNE3	5	4.76E+03	0.451	INNATE IMMUNE SYSTEM
ICAM3	7	3.02E+03	0.453	INNATE IMMUNE SYSTEM
CPN2	9	4.83E+03	0.454	INNATE IMMUNE SYSTEM
CFHR1	1	9.01E+01	0.455	INNATE IMMUNE SYSTEM
IDH1	3	3.33E+03	0.458	INNATE IMMUNE SYSTEM
HLA-A	11	1.78E+04	0.458	INNATE IMMUNE SYSTEM
CLEC10A	7	9.98E+03	0.458	INNATE IMMUNE SYSTEM
MMP25	9	7.15E+03	0.460	INNATE IMMUNE SYSTEM
TNFAIP6	3	4.65E+03	0.461	INNATE IMMUNE SYSTEM
PROS1	8	5.86E+03	0.462	INNATE IMMUNE SYSTEM
PRKCQ	5	1.70E+03	0.464	INNATE IMMUNE SYSTEM
PSMD5	6	6.49E+03	0.464	INNATE IMMUNE SYSTEM
CLU	6	3.72E+03	0.464	INNATE IMMUNE SYSTEM
SIGLEC14	3	1.52E+03	0.465	INNATE IMMUNE SYSTEM
CTSH	6	4.16E+03	0.466	INNATE IMMUNE SYSTEM
RHOF	4	5.46E+03	0.466	INNATE IMMUNE SYSTEM
ATP11A	10	7.40E+03	0.466	INNATE IMMUNE SYSTEM
IFNA1	2	1.33E+03	0.469	INNATE IMMUNE SYSTEM
POLR3A	4	9.27E+03	0.470	INNATE IMMUNE SYSTEM
CD58	3	2.01E+03	0.471	INNATE IMMUNE SYSTEM
BPIFB1	11	9.61E+03	0.474	INNATE IMMUNE SYSTEM
CD93	12	8.71E+03	0.476	INNATE IMMUNE SYSTEM
BPIFB4	6	6.59E+03	0.481	INNATE IMMUNE SYSTEM
POLR3D	2	8.80E+02	0.483	INNATE IMMUNE SYSTEM
MAPK14	3	9.45E+02	0.483	INNATE IMMUNE SYSTEM
SLC44A2	8	6.58E+03	0.486	INNATE IMMUNE SYSTEM
PTX3	6	3.85E+03	0.487	INNATE IMMUNE SYSTEM
HK3	20	2.46E+04	0.489	INNATE IMMUNE SYSTEM
PI3	2	2.44E+03	0.491	INNATE IMMUNE SYSTEM
PSMD7	3	1.22E+03	0.491	INNATE IMMUNE SYSTEM
IFNA6	5	4.16E+03	0.492	INNATE IMMUNE SYSTEM
DTX4	3	1.02E+03	0.494	INNATE IMMUNE SYSTEM
TRIM25	8	3.12E+03	0.494	INNATE IMMUNE SYSTEM
ACPP	2	3.47E+02	0.495	INNATE IMMUNE SYSTEM
DEFB132	6	8.35E+03	0.496	INNATE IMMUNE SYSTEM
PLCG2	17	2.09E+04	0.497	INNATE IMMUNE SYSTEM
ATG12	2	2.56E+03	0.499	INNATE IMMUNE SYSTEM
POLR3H	3	6.05E+02	0.502	INNATE IMMUNE SYSTEM
HSPA8	1	7.27E+01	0.503	INNATE IMMUNE SYSTEM
CKAP4	7	5.12E+03	0.505	INNATE IMMUNE SYSTEM
RNF216	4	1.09E+03	0.506	INNATE IMMUNE SYSTEM
CAPZA2	2	4.45E+02	0.506	INNATE IMMUNE SYSTEM
AGER	10	9.69E+03	0.510	INNATE IMMUNE SYSTEM

SFTPA2	5	3.02E+03	0.511	INNATE IMMUNE SYSTEM
MAP3K1	8	6.80E+03	0.511	INNATE IMMUNE SYSTEM
PSMD8	3	5.62E+02	0.512	INNATE IMMUNE SYSTEM
FGA	7	1.83E+03	0.515	INNATE IMMUNE SYSTEM
SERPINB6	5	3.70E+03	0.516	INNATE IMMUNE SYSTEM
PIN1	2	9.30E+02	0.516	INNATE IMMUNE SYSTEM
RAB18	1	1.03E+02	0.518	INNATE IMMUNE SYSTEM
LRRFIP1	16	1.63E+04	0.519	INNATE IMMUNE SYSTEM
DNAJC13	18	2.96E+04	0.519	INNATE IMMUNE SYSTEM
VCL	6	2.59E+03	0.520	INNATE IMMUNE SYSTEM
LAIR1	5	7.60E+03	0.521	INNATE IMMUNE SYSTEM
AZU1	2	7.70E+02	0.521	INNATE IMMUNE SYSTEM
ARSA	4	1.13E+03	0.522	INNATE IMMUNE SYSTEM
PSMB10	3	7.89E+02	0.522	INNATE IMMUNE SYSTEM
ANPEP	11	1.08E+04	0.522	INNATE IMMUNE SYSTEM
WASF1	3	1.39E+03	0.522	INNATE IMMUNE SYSTEM
CANT1	5	5.04E+03	0.522	INNATE IMMUNE SYSTEM
RAB31	1	1.86E+02	0.523	INNATE IMMUNE SYSTEM
HLA-B	7	3.50E+03	0.524	INNATE IMMUNE SYSTEM
UBA3	1	7.33E+01	0.526	INNATE IMMUNE SYSTEM
COMMD3	2	3.24E+03	0.526	INNATE IMMUNE SYSTEM
RAB6A	1	1.01E+03	0.529	INNATE IMMUNE SYSTEM
C2	8	4.83E+03	0.530	INNATE IMMUNE SYSTEM
CTSG	1	1.61E+02	0.531	INNATE IMMUNE SYSTEM
FPR2	3	1.80E+03	0.531	INNATE IMMUNE SYSTEM
CCL17	2	2.70E+02	0.531	INNATE IMMUNE SYSTEM
TRPM2	29	2.10E+04	0.532	INNATE IMMUNE SYSTEM
CFI	7	5.99E+03	0.532	INNATE IMMUNE SYSTEM
MNDA	5	4.33E+03	0.534	INNATE IMMUNE SYSTEM
BIN2	6	6.34E+03	0.539	INNATE IMMUNE SYSTEM
PTGES2	1	1.05E+03	0.542	INNATE IMMUNE SYSTEM
MAP2K6	1	5.90E+01	0.544	INNATE IMMUNE SYSTEM
LBP	15	2.64E+04	0.545	INNATE IMMUNE SYSTEM
UNC13D	17	1.53E+04	0.546	INNATE IMMUNE SYSTEM
POLR3E	4	2.60E+03	0.546	INNATE IMMUNE SYSTEM
FCN1	6	3.99E+03	0.547	INNATE IMMUNE SYSTEM
TMC6	12	5.81E+03	0.548	INNATE IMMUNE SYSTEM
ATP6V1H	3	1.38E+03	0.549	INNATE IMMUNE SYSTEM
PSMD9	3	9.67E+02	0.549	INNATE IMMUNE SYSTEM
PELI1	1	6.37E+01	0.549	INNATE IMMUNE SYSTEM
IRF7	6	2.37E+03	0.550	INNATE IMMUNE SYSTEM
UBE2L6	1	6.07E+01	0.557	INNATE IMMUNE SYSTEM
NFKBIA	1	7.31E+01	0.561	INNATE IMMUNE SYSTEM
IFNA17	1	7.35E+01	0.562	INNATE IMMUNE SYSTEM
DNM2	4	1.37E+03	0.562	INNATE IMMUNE SYSTEM
GLIPR1	5	1.44E+03	0.563	INNATE IMMUNE SYSTEM
CFD	3	2.22E+03	0.568	INNATE IMMUNE SYSTEM
MAPK12	6	3.97E+03	0.571	INNATE IMMUNE SYSTEM
MYH9	16	1.39E+04	0.571	INNATE IMMUNE SYSTEM
NOS2	11	5.18E+03	0.574	INNATE IMMUNE SYSTEM
NCSTN	5	2.13E+03	0.575	INNATE IMMUNE SYSTEM
SIGIRR	5	3.40E+03	0.575	INNATE IMMUNE SYSTEM
IFNA14	9	5.87E+03	0.575	INNATE IMMUNE SYSTEM
ATP6V1E1	1	8.36E+01	0.576	INNATE IMMUNE SYSTEM
TRIM56	13	6.04E+03	0.576	INNATE IMMUNE SYSTEM
COLEC11	4	2.30E+03	0.576	INNATE IMMUNE SYSTEM
PRKCSH	12	7.49E+03	0.576	INNATE IMMUNE SYSTEM
NCF2	9	8.45E+03	0.577	INNATE IMMUNE SYSTEM
POLR2E	3	1.34E+03	0.577	INNATE IMMUNE SYSTEM
MUC1	19	1.78E+04	0.577	INNATE IMMUNE SYSTEM
PSMC3	1	3.75E+01	0.578	INNATE IMMUNE SYSTEM
DOK3	6	1.83E+03	0.578	INNATE IMMUNE SYSTEM
SLC27A2	4	9.15E+02	0.578	INNATE IMMUNE SYSTEM
CD36	6	1.30E+03	0.580	INNATE IMMUNE SYSTEM
PTPN6	5	3.25E+03	0.580	INNATE IMMUNE SYSTEM
A1BG	5	2.80E+03	0.580	INNATE IMMUNE SYSTEM
WASL	2	1.79E+02	0.582	INNATE IMMUNE SYSTEM
MAP2K4	1	1.17E+02	0.583	INNATE IMMUNE SYSTEM
VAV2	10	5.39E+03	0.583	INNATE IMMUNE SYSTEM
PGLYRP3	7	1.08E+04	0.584	INNATE IMMUNE SYSTEM
MUC12	169	2.73E+05	0.584	INNATE IMMUNE SYSTEM
KRT1	4	4.11E+03	0.585	INNATE IMMUNE SYSTEM
DERA	1	6.62E+01	0.586	INNATE IMMUNE SYSTEM
POLR3B	5	2.25E+03	0.586	INNATE IMMUNE SYSTEM

KRAS	2	3.83E+02	0.589	INNATE IMMUNE SYSTEM
DEFB104B	2	5.91E+02	0.589	INNATE IMMUNE SYSTEM
DEFB104A	2	5.91E+02	0.589	INNATE IMMUNE SYSTEM
IST1	7	2.89E+03	0.591	INNATE IMMUNE SYSTEM
FPR1	5	2.74E+03	0.591	INNATE IMMUNE SYSTEM
HGSNAT	5	5.87E+03	0.592	INNATE IMMUNE SYSTEM
PLPP4	2	4.52E+02	0.592	INNATE IMMUNE SYSTEM
TRAPPC1	1	1.99E+01	0.595	INNATE IMMUNE SYSTEM
NIT2	5	1.02E+03	0.596	INNATE IMMUNE SYSTEM
SAA1	6	3.52E+03	0.597	INNATE IMMUNE SYSTEM
ANO6	9	4.83E+03	0.597	INNATE IMMUNE SYSTEM
STK10	10	5.13E+03	0.598	INNATE IMMUNE SYSTEM
PSMD11	1	2.60E+01	0.598	INNATE IMMUNE SYSTEM
DPP7	13	1.17E+04	0.598	INNATE IMMUNE SYSTEM
SERPINB1	2	4.42E+02	0.599	INNATE IMMUNE SYSTEM
HSP90B1	4	3.90E+03	0.601	INNATE IMMUNE SYSTEM
ALDOC	1	1.29E+02	0.602	INNATE IMMUNE SYSTEM
CASP9	8	6.15E+03	0.602	INNATE IMMUNE SYSTEM
ARSB	4	2.85E+03	0.604	INNATE IMMUNE SYSTEM
IFNA13	2	5.43E+02	0.606	INNATE IMMUNE SYSTEM
C1QB	2	2.90E+02	0.606	INNATE IMMUNE SYSTEM
FRK	7	5.90E+03	0.607	INNATE IMMUNE SYSTEM
BST2	2	1.76E+02	0.609	INNATE IMMUNE SYSTEM
CRP	1	3.39E+02	0.609	INNATE IMMUNE SYSTEM
CLEC4D	3	4.68E+02	0.609	INNATE IMMUNE SYSTEM
EP300	8	5.82E+03	0.612	INNATE IMMUNE SYSTEM
NCK1	3	2.22E+03	0.613	INNATE IMMUNE SYSTEM
VAT1	3	6.66E+02	0.613	INNATE IMMUNE SYSTEM
TNFAIP3	10	1.95E+03	0.614	INNATE IMMUNE SYSTEM
ADAM8	20	1.34E+04	0.615	INNATE IMMUNE SYSTEM
TXNDC5	8	3.18E+03	0.616	INNATE IMMUNE SYSTEM
P2RX7	13	1.34E+04	0.616	INNATE IMMUNE SYSTEM
LCK	1	1.25E+02	0.617	INNATE IMMUNE SYSTEM
CAPN1	7	2.70E+03	0.618	INNATE IMMUNE SYSTEM
ITPR3	15	4.94E+03	0.623	INNATE IMMUNE SYSTEM
RASGRP1	2	2.64E+02	0.624	INNATE IMMUNE SYSTEM
GPR84	6	2.07E+03	0.625	INNATE IMMUNE SYSTEM
BCL10	1	7.88E+01	0.625	INNATE IMMUNE SYSTEM
CTNNB1	2	4.03E+02	0.627	INNATE IMMUNE SYSTEM
PSMB3	1	5.09E+01	0.629	INNATE IMMUNE SYSTEM
RPS6KA5	5	2.79E+03	0.629	INNATE IMMUNE SYSTEM
UBA7	11	6.11E+03	0.630	INNATE IMMUNE SYSTEM
CLEC7A	2	9.12E+02	0.633	INNATE IMMUNE SYSTEM
C4B	8	2.80E+03	0.637	INNATE IMMUNE SYSTEM
CTSC	5	8.46E+02	0.639	INNATE IMMUNE SYSTEM
CD3G	3	8.87E+02	0.640	INNATE IMMUNE SYSTEM
ELMO2	1	5.01E+01	0.643	INNATE IMMUNE SYSTEM
NLRC3	26	1.75E+04	0.644	INNATE IMMUNE SYSTEM
PELI2	3	2.18E+03	0.644	INNATE IMMUNE SYSTEM
JUP	9	3.23E+03	0.644	INNATE IMMUNE SYSTEM
ART1	8	6.94E+03	0.644	INNATE IMMUNE SYSTEM
ORM2	7	4.57E+03	0.645	INNATE IMMUNE SYSTEM
COMMD9	3	5.13E+02	0.646	INNATE IMMUNE SYSTEM
LCP2	7	5.46E+03	0.648	INNATE IMMUNE SYSTEM
SIGLEC5	2	3.78E+02	0.649	INNATE IMMUNE SYSTEM
PYGB	8	3.12E+03	0.649	INNATE IMMUNE SYSTEM
PSME1	2	1.21E+03	0.649	INNATE IMMUNE SYSTEM
APRT	2	2.17E+02	0.649	INNATE IMMUNE SYSTEM
CD63	1	4.79E+01	0.652	INNATE IMMUNE SYSTEM
ABCA13	73	2.08E+05	0.660	INNATE IMMUNE SYSTEM
VAPA	2	1.89E+03	0.661	INNATE IMMUNE SYSTEM
FUCA2	3	1.56E+03	0.661	INNATE IMMUNE SYSTEM
PFKL	14	7.71E+03	0.662	INNATE IMMUNE SYSTEM
ARPC3	2	4.55E+02	0.664	INNATE IMMUNE SYSTEM
PELI3	3	1.37E+03	0.665	INNATE IMMUNE SYSTEM
CTSB	8	1.92E+03	0.666	INNATE IMMUNE SYSTEM
KIR2DS5	112	1.05E+05	0.668	INNATE IMMUNE SYSTEM
DEFB126	3	2.15E+03	0.669	INNATE IMMUNE SYSTEM
LILRB3	18	1.24E+04	0.672	INNATE IMMUNE SYSTEM
PLA2G2A	2	1.14E+03	0.672	INNATE IMMUNE SYSTEM
PSMD1	6	1.26E+03	0.674	INNATE IMMUNE SYSTEM
IQGAP2	18	1.16E+04	0.675	INNATE IMMUNE SYSTEM
TAB2	2	5.46E+02	0.676	INNATE IMMUNE SYSTEM
GALNS	9	3.88E+03	0.677	INNATE IMMUNE SYSTEM

DSG1	14	9.70E+03	0.680	INNATE IMMUNE SYSTEM
CALML5	2	7.99E+02	0.680	INNATE IMMUNE SYSTEM
CTSV	1	4.52E+01	0.680	INNATE IMMUNE SYSTEM
CREB1	1	5.55E+01	0.681	INNATE IMMUNE SYSTEM
CTSD	2	6.73E+02	0.681	INNATE IMMUNE SYSTEM
FGR	2	2.04E+02	0.681	INNATE IMMUNE SYSTEM
TREX1	2	7.38E+02	0.683	INNATE IMMUNE SYSTEM
MLEC	1	3.56E+01	0.683	INNATE IMMUNE SYSTEM
VAV1	3	1.50E+03	0.684	INNATE IMMUNE SYSTEM
C8B	7	5.19E+03	0.686	INNATE IMMUNE SYSTEM
CASP2	1	2.96E+01	0.689	INNATE IMMUNE SYSTEM
ORM1	8	3.97E+03	0.690	INNATE IMMUNE SYSTEM
CEP290	27	1.14E+04	0.692	INNATE IMMUNE SYSTEM
AGA	6	5.47E+03	0.692	INNATE IMMUNE SYSTEM
S100A7A	4	3.78E+02	0.693	INNATE IMMUNE SYSTEM
C3	10	2.84E+03	0.694	INNATE IMMUNE SYSTEM
ARHGAP45	11	2.55E+03	0.694	INNATE IMMUNE SYSTEM
SIKE1	1	6.89E+01	0.694	INNATE IMMUNE SYSTEM
PLAU	6	6.50E+03	0.694	INNATE IMMUNE SYSTEM
MAP3K7	2	2.71E+02	0.694	INNATE IMMUNE SYSTEM
XRCC5	3	8.49E+02	0.697	INNATE IMMUNE SYSTEM
BPIFB2	9	2.92E+03	0.697	INNATE IMMUNE SYSTEM
PLD3	9	2.40E+03	0.697	INNATE IMMUNE SYSTEM
TBC1D10C	9	5.89E+03	0.699	INNATE IMMUNE SYSTEM
PPP2R1B	5	2.89E+03	0.702	INNATE IMMUNE SYSTEM
DIAPH1	8	2.98E+03	0.704	INNATE IMMUNE SYSTEM
PIGR	13	4.54E+03	0.706	INNATE IMMUNE SYSTEM
ATP6V0A2	7	5.59E+03	0.706	INNATE IMMUNE SYSTEM
CAPZA1	4	2.33E+03	0.706	INNATE IMMUNE SYSTEM
MAPKAPK2	3	2.53E+03	0.708	INNATE IMMUNE SYSTEM
MPO	14	1.47E+04	0.710	INNATE IMMUNE SYSTEM
ABI2	6	1.36E+03	0.711	INNATE IMMUNE SYSTEM
HSPA6	9	2.23E+03	0.714	INNATE IMMUNE SYSTEM
FGB	5	1.47E+03	0.715	INNATE IMMUNE SYSTEM
CASP1	6	2.39E+03	0.716	INNATE IMMUNE SYSTEM
STK11IP	15	8.80E+03	0.717	INNATE IMMUNE SYSTEM
CTSA	3	6.21E+02	0.717	INNATE IMMUNE SYSTEM
GDI2	1	2.56E+01	0.718	INNATE IMMUNE SYSTEM
BCL2	1	3.01E+01	0.719	INNATE IMMUNE SYSTEM
TOM1	5	1.19E+03	0.720	INNATE IMMUNE SYSTEM
ATP6V1A	1	2.04E+01	0.722	INNATE IMMUNE SYSTEM
TREM1	3	2.44E+02	0.722	INNATE IMMUNE SYSTEM
CXCR2	5	9.70E+02	0.723	INNATE IMMUNE SYSTEM
SVIP	2	1.08E+02	0.723	INNATE IMMUNE SYSTEM
PSME4	9	2.12E+03	0.724	INNATE IMMUNE SYSTEM
TTR	1	1.45E+02	0.724	INNATE IMMUNE SYSTEM
SUGT1	2	4.95E+02	0.724	INNATE IMMUNE SYSTEM
VAV3	11	3.52E+03	0.728	INNATE IMMUNE SYSTEM
DUSP6	2	3.34E+02	0.728	INNATE IMMUNE SYSTEM
ARPC1B	4	9.56E+02	0.729	INNATE IMMUNE SYSTEM
BPIFB6	5	3.21E+03	0.732	INNATE IMMUNE SYSTEM
SERPINB10	7	5.53E+03	0.735	INNATE IMMUNE SYSTEM
ATP6V1B2	2	8.54E+02	0.736	INNATE IMMUNE SYSTEM
SERPINA1	11	4.48E+03	0.737	INNATE IMMUNE SYSTEM
ATAD3B	22	2.81E+04	0.739	INNATE IMMUNE SYSTEM
PRKACG	1	3.70E+01	0.742	INNATE IMMUNE SYSTEM
NFKB1	5	1.50E+03	0.747	INNATE IMMUNE SYSTEM
CD300A	2	2.19E+02	0.747	INNATE IMMUNE SYSTEM
HBB	1	1.02E+02	0.747	INNATE IMMUNE SYSTEM
PPP2R1A	1	1.55E+02	0.748	INNATE IMMUNE SYSTEM
PADI2	7	4.22E+03	0.749	INNATE IMMUNE SYSTEM
TMEM173	1	8.95E+01	0.750	INNATE IMMUNE SYSTEM
LPCAT1	3	4.24E+02	0.752	INNATE IMMUNE SYSTEM
S100A7	3	1.00E+03	0.752	INNATE IMMUNE SYSTEM
RASGRP2	4	6.70E+02	0.753	INNATE IMMUNE SYSTEM
BCL2L1	2	1.12E+02	0.753	INNATE IMMUNE SYSTEM
DCD	1	2.36E+02	0.754	INNATE IMMUNE SYSTEM
ECSIT	4	4.61E+02	0.755	INNATE IMMUNE SYSTEM
PLPP5	4	4.38E+02	0.756	INNATE IMMUNE SYSTEM
CFHR3	2	2.45E+02	0.757	INNATE IMMUNE SYSTEM
PSMD6	5	5.04E+02	0.757	INNATE IMMUNE SYSTEM
CHRNB4	6	2.86E+03	0.758	INNATE IMMUNE SYSTEM
RIPK2	1	3.60E+01	0.760	INNATE IMMUNE SYSTEM
PSMD13	3	1.26E+03	0.761	INNATE IMMUNE SYSTEM

CR1	23	1.07E+04	0.761	INNATE IMMUNE SYSTEM
DYNC1H1	7	1.68E+03	0.763	INNATE IMMUNE SYSTEM
CFH	10	4.46E+03	0.765	INNATE IMMUNE SYSTEM
FCGR3A	4	4.13E+03	0.765	INNATE IMMUNE SYSTEM
DEFB125	3	1.06E+03	0.765	INNATE IMMUNE SYSTEM
SHC1	3	1.24E+03	0.765	INNATE IMMUNE SYSTEM
AGPAT2	4	6.49E+02	0.766	INNATE IMMUNE SYSTEM
STAT6	4	5.96E+02	0.767	INNATE IMMUNE SYSTEM
ACAA1	4	1.07E+03	0.769	INNATE IMMUNE SYSTEM
CCL22	2	1.53E+03	0.771	INNATE IMMUNE SYSTEM
SIGLEC9	9	5.60E+03	0.771	INNATE IMMUNE SYSTEM
TARM1	3	1.52E+03	0.771	INNATE IMMUNE SYSTEM
PDPK1	1	2.60E+01	0.773	INNATE IMMUNE SYSTEM
AGL	19	8.98E+03	0.773	INNATE IMMUNE SYSTEM
REG3G	5	1.53E+03	0.774	INNATE IMMUNE SYSTEM
SELL	2	9.28E+02	0.776	INNATE IMMUNE SYSTEM
CTSL	1	1.89E+02	0.776	INNATE IMMUNE SYSTEM
CCT8	4	2.84E+03	0.777	INNATE IMMUNE SYSTEM
ORMDL3	1	2.84E+01	0.778	INNATE IMMUNE SYSTEM
PRKACA	4	1.16E+03	0.781	INNATE IMMUNE SYSTEM
MMP9	17	7.94E+03	0.782	INNATE IMMUNE SYSTEM
NFATC3	3	1.58E+02	0.783	INNATE IMMUNE SYSTEM
DOCK2	12	7.40E+03	0.783	INNATE IMMUNE SYSTEM
BIRC3	3	8.71E+02	0.785	INNATE IMMUNE SYSTEM
DEFB136	1	1.70E+01	0.788	INNATE IMMUNE SYSTEM
ARHGAP9	7	3.75E+03	0.788	INNATE IMMUNE SYSTEM
GUSB	3	6.23E+02	0.789	INNATE IMMUNE SYSTEM
ATP6V1F	3	1.18E+03	0.789	INNATE IMMUNE SYSTEM
TLR9	6	6.29E+02	0.794	INNATE IMMUNE SYSTEM
DUSP4	1	8.74E+01	0.796	INNATE IMMUNE SYSTEM
C9	12	2.46E+03	0.797	INNATE IMMUNE SYSTEM
VTN	6	2.11E+03	0.797	INNATE IMMUNE SYSTEM
LAMP1	5	1.07E+03	0.798	INNATE IMMUNE SYSTEM
CFHR2	5	2.94E+03	0.800	INNATE IMMUNE SYSTEM
PTPRC	14	9.93E+03	0.807	INNATE IMMUNE SYSTEM
PSTPIP1	7	7.69E+02	0.807	INNATE IMMUNE SYSTEM
TRAF3	5	1.43E+03	0.807	INNATE IMMUNE SYSTEM
ALDOA	5	1.17E+03	0.809	INNATE IMMUNE SYSTEM
IRAK2	9	6.91E+03	0.810	INNATE IMMUNE SYSTEM
RAF1	3	1.61E+02	0.811	INNATE IMMUNE SYSTEM
NLRX1	16	8.03E+03	0.811	INNATE IMMUNE SYSTEM
MYO1C	20	4.63E+03	0.813	INNATE IMMUNE SYSTEM
MAPK13	3	9.28E+02	0.813	INNATE IMMUNE SYSTEM
AMPD3	10	4.52E+03	0.814	INNATE IMMUNE SYSTEM
MS4A3	3	1.87E+02	0.814	INNATE IMMUNE SYSTEM
C4BPA	4	3.19E+03	0.815	INNATE IMMUNE SYSTEM
POLR3G	2	1.16E+02	0.816	INNATE IMMUNE SYSTEM
DDOST	5	1.20E+03	0.818	INNATE IMMUNE SYSTEM
ARL8A	2	1.22E+02	0.819	INNATE IMMUNE SYSTEM
ACTR1B	2	6.12E+02	0.820	INNATE IMMUNE SYSTEM
NFKB2	8	1.88E+03	0.825	INNATE IMMUNE SYSTEM
FGG	5	8.31E+02	0.825	INNATE IMMUNE SYSTEM
ATP6V1D	2	3.12E+02	0.825	INNATE IMMUNE SYSTEM
ERP44	1	7.17E+01	0.825	INNATE IMMUNE SYSTEM
HRNR	93	2.92E+04	0.825	INNATE IMMUNE SYSTEM
RAB5C	3	5.58E+02	0.825	INNATE IMMUNE SYSTEM
RETN	1	1.72E+01	0.826	INNATE IMMUNE SYSTEM
ARPC1A	1	1.37E+01	0.831	INNATE IMMUNE SYSTEM
GHDC	6	2.11E+03	0.832	INNATE IMMUNE SYSTEM
DGAT1	4	2.10E+03	0.835	INNATE IMMUNE SYSTEM
KLRC2	2	2.16E+02	0.835	INNATE IMMUNE SYSTEM
ATP6V1B1	6	8.93E+02	0.835	INNATE IMMUNE SYSTEM
TOLLIP	4	1.77E+02	0.837	INNATE IMMUNE SYSTEM
DEFB113	1	8.42E+00	0.838	INNATE IMMUNE SYSTEM
TICAM1	13	1.50E+03	0.839	INNATE IMMUNE SYSTEM
OSTF1	3	1.24E+02	0.840	INNATE IMMUNE SYSTEM
CHGA	5	5.72E+03	0.841	INNATE IMMUNE SYSTEM
S100A9	1	7.66E+00	0.842	INNATE IMMUNE SYSTEM
MAPK11	1	1.55E+02	0.842	INNATE IMMUNE SYSTEM
RIPK1	3	3.52E+02	0.843	INNATE IMMUNE SYSTEM
PSME3	1	7.83E+00	0.846	INNATE IMMUNE SYSTEM
PSMD2	2	1.22E+02	0.847	INNATE IMMUNE SYSTEM
TLR4	9	4.02E+03	0.849	INNATE IMMUNE SYSTEM
CD46	3	4.23E+02	0.849	INNATE IMMUNE SYSTEM

ALOX5	4	4.61E+02	0.850	INNATE IMMUNE SYSTEM
ABL1	7	5.15E+03	0.851	INNATE IMMUNE SYSTEM
RPS6KA2	3	6.28E+02	0.851	INNATE IMMUNE SYSTEM
BPIFA2	6	1.13E+03	0.854	INNATE IMMUNE SYSTEM
S100A8	5	6.91E+02	0.859	INNATE IMMUNE SYSTEM
DYNLT1	1	1.00E+01	0.860	INNATE IMMUNE SYSTEM
PSMC1	1	4.42E+00	0.860	INNATE IMMUNE SYSTEM
C7	8	3.07E+03	0.860	INNATE IMMUNE SYSTEM
HCK	4	6.33E+02	0.863	INNATE IMMUNE SYSTEM
IKBKE	6	3.17E+03	0.868	INNATE IMMUNE SYSTEM
ITGAL	4	5.63E+02	0.869	INNATE IMMUNE SYSTEM
ISG15	1	5.03E+00	0.870	INNATE IMMUNE SYSTEM
MASP1	9	1.41E+03	0.870	INNATE IMMUNE SYSTEM
NLRP4	9	1.46E+03	0.872	INNATE IMMUNE SYSTEM
C1QC	3	1.63E+02	0.872	INNATE IMMUNE SYSTEM
HSP90AA1	4	3.16E+02	0.872	INNATE IMMUNE SYSTEM
GCA	2	1.33E+03	0.873	INNATE IMMUNE SYSTEM
ATG7	6	1.06E+03	0.873	INNATE IMMUNE SYSTEM
NFAM1	2	9.22E+01	0.878	INNATE IMMUNE SYSTEM
CD19	3	1.70E+02	0.880	INNATE IMMUNE SYSTEM
NHLRC3	7	1.13E+03	0.883	INNATE IMMUNE SYSTEM
MUCL1	1	6.17E+00	0.884	INNATE IMMUNE SYSTEM
PYGL	6	5.46E+03	0.888	INNATE IMMUNE SYSTEM
RAB37	13	1.37E+03	0.892	INNATE IMMUNE SYSTEM
FUCA1	4	5.41E+02	0.896	INNATE IMMUNE SYSTEM
CFHR5	11	1.62E+03	0.896	INNATE IMMUNE SYSTEM
FCN3	1	1.95E+01	0.897	INNATE IMMUNE SYSTEM
ALAD	4	3.98E+02	0.897	INNATE IMMUNE SYSTEM
LTF	8	5.90E+02	0.900	INNATE IMMUNE SYSTEM
PGLYRP2	11	3.38E+03	0.901	INNATE IMMUNE SYSTEM
UNC93B1	12	9.23E+03	0.901	INNATE IMMUNE SYSTEM
MUC7	16	8.56E+03	0.902	INNATE IMMUNE SYSTEM
DEFB110	1	3.39E+00	0.902	INNATE IMMUNE SYSTEM
DSN1	1	7.33E+00	0.904	INNATE IMMUNE SYSTEM
NOS3	14	1.04E+03	0.905	INNATE IMMUNE SYSTEM
CD55	5	1.70E+02	0.906	INNATE IMMUNE SYSTEM
PSMB2	1	1.34E+01	0.906	INNATE IMMUNE SYSTEM
CYSTM1	1	2.56E+00	0.909	INNATE IMMUNE SYSTEM
NKIRAS2	1	2.12E+00	0.909	INNATE IMMUNE SYSTEM
NBEAL2	20	9.38E+03	0.909	INNATE IMMUNE SYSTEM
PTK2	4	4.36E+02	0.910	INNATE IMMUNE SYSTEM
B2M	1	4.62E+00	0.913	INNATE IMMUNE SYSTEM
ITGAX	14	7.19E+02	0.915	INNATE IMMUNE SYSTEM
MAPK9	1	5.64E+00	0.915	INNATE IMMUNE SYSTEM
CARD11	10	2.89E+02	0.922	INNATE IMMUNE SYSTEM
DEFB121	1	2.65E+00	0.924	INNATE IMMUNE SYSTEM
RHOG	1	1.16E+01	0.925	INNATE IMMUNE SYSTEM
CD47	1	1.34E+00	0.929	INNATE IMMUNE SYSTEM
SNAP29	1	1.05E+01	0.942	INNATE IMMUNE SYSTEM
PSMA4	1	6.70E-01	0.947	INNATE IMMUNE SYSTEM
IL1B	1	7.49E-01	0.950	INNATE IMMUNE SYSTEM
CDA	1	8.52E-01	0.955	INNATE IMMUNE SYSTEM
RNASE6	1	1.08E+00	0.962	INNATE IMMUNE SYSTEM
PSME2	1	6.85E+00	0.964	INNATE IMMUNE SYSTEM
FAF2	1	4.24E-01	0.980	INNATE IMMUNE SYSTEM
PIK3CB	1	9.66E-02	0.991	INNATE IMMUNE SYSTEM
IRF3	5	3.05E+02	1	INNATE IMMUNE SYSTEM
AP1M1	2	4.33E-01	1	INNATE IMMUNE SYSTEM
C4A	5	1.22E+02	1	INNATE IMMUNE SYSTEM
NCR2	5	1.40E+01	1	INNATE IMMUNE SYSTEM
C5AR2	2	3.91E+01	1	INNATE IMMUNE SYSTEM
ATP6V0A1	7	9.40E+00	1	INNATE IMMUNE SYSTEM
IRAK4	3	5.17E+01	1	INNATE IMMUNE SYSTEM
DEFB131A	3	3.28E+02	1	INNATE IMMUNE SYSTEM
ARMC8	2	2.03E+00	1	INNATE IMMUNE SYSTEM
TYROBP	3	5.55E+01	1	INNATE IMMUNE SYSTEM
ATP8B4	14	7.32E+03	1	INNATE IMMUNE SYSTEM
SLC2A5	7	1.38E+02	1	INNATE IMMUNE SYSTEM
ITGB2	6	1.67E+03	1	INNATE IMMUNE SYSTEM
PRSS3	13	1.72E+03	1	INNATE IMMUNE SYSTEM
CD209	4	2.40E+02	1	INNATE IMMUNE SYSTEM
PSMF1	7	1.61E+03	1	INNATE IMMUNE SYSTEM
PTPN11	3	1.93E+02	1	INNATE IMMUNE SYSTEM
GNLY	3	1.39E+02	1	INNATE IMMUNE SYSTEM

PGLYRP4	6	1.19E+02	1	INNATE IMMUNE SYSTEM
SRP14	2	4.31E+01	1	INNATE IMMUNE SYSTEM
NLRC5	16	9.56E+01	1	INNATE IMMUNE SYSTEM
LTA4H	4	7.41E+02	1	INNATE IMMUNE SYSTEM
CAP1	3	1.23E+02	1	INNATE IMMUNE SYSTEM
PGLYRP1	3	2.96E+01	1	INNATE IMMUNE SYSTEM
CPPED1	12	5.72E+01	1	INNATE IMMUNE SYSTEM
GLB1	7	7.02E+02	1	INNATE IMMUNE SYSTEM
HVCN1	3	2.38E+01	1	INNATE IMMUNE SYSTEM
HRAS	3	3.32E+00	1	INNATE IMMUNE SYSTEM
MRE11	5	5.13E+02	1	INNATE IMMUNE SYSTEM
FCGR3B	2	2.55E+02	1	INNATE IMMUNE SYSTEM
CASP8	3	4.03E+02	1	INNATE IMMUNE SYSTEM
MME	9	2.49E+03	1	INNATE IMMUNE SYSTEM
AHSG	3	1.48E+03	1	INNATE IMMUNE SYSTEM
HEBP2	8	6.39E+02	1	INNATE IMMUNE SYSTEM
ADGRE3	8	4.95E+02	1	INNATE IMMUNE SYSTEM
PSMA8	4	5.16E+00	1	INNATE IMMUNE SYSTEM
VRK3	9	5.39E+02	1	INNATE IMMUNE SYSTEM
TIMP2	10	1.84E+02	1	INNATE IMMUNE SYSTEM
PRG3	3	3.42E+00	1	INNATE IMMUNE SYSTEM
C8A	9	6.37E+02	1	INNATE IMMUNE SYSTEM
PANX1	2	3.35E+01	1	INNATE IMMUNE SYSTEM
YES1	2	1.99E+02	1	INNATE IMMUNE SYSTEM
FCGR2A	3	1.18E+02	1	INNATE IMMUNE SYSTEM
TANK	6	2.98E+03	1	INNATE IMMUNE SYSTEM
MEFV	13	1.09E+04	1	INNATE IMMUNE SYSTEM
PSMD3	2	7.88E+00	1	INNATE IMMUNE SYSTEM
FOS	5	2.75E+02	1	INNATE IMMUNE SYSTEM
ELANE	3	5.41E+01	1	INNATE IMMUNE SYSTEM
ATP6V0A4	9	3.09E+03	1	INNATE IMMUNE SYSTEM
GYG1	5	1.46E+02	1	INNATE IMMUNE SYSTEM
PYCARD	2	2.66E+02	1	INNATE IMMUNE SYSTEM
DOCK1	20	7.33E+02	1	INNATE IMMUNE SYSTEM
SARM1	6	2.75E+03	1	INNATE IMMUNE SYSTEM
COPB1	6	1.14E+02	1	INNATE IMMUNE SYSTEM
VNN1	6	1.12E+02	1	INNATE IMMUNE SYSTEM
ITGAM	14	7.91E+02	1	INNATE IMMUNE SYSTEM
MAPK3	5	2.78E+01	1	INNATE IMMUNE SYSTEM
IFNA7	6	1.32E+03	1	INNATE IMMUNE SYSTEM
CD68	2	4.02E+01	1	INNATE IMMUNE SYSTEM
PIK3R4	4	5.87E+02	1	INNATE IMMUNE SYSTEM
TEC	2	1.07E+01	1	INNATE IMMUNE SYSTEM
PTPRJ	17	4.13E+03	1	INNATE IMMUNE SYSTEM
SOCS1	2	9.38E+01	1	INNATE IMMUNE SYSTEM
DEFA4	6	1.74E+03	1	INNATE IMMUNE SYSTEM

Gene	NumVar	Q	Pvalue	Gene-set
GBA	9	1.83E+05	1.95E-09	LYSOSOME
SORT1	10	5.19E+04	0.033	LYSOSOME
AP3B1	6	2.86E+04	0.041	LYSOSOME
CTNS	6	1.15E+04	0.041	LYSOSOME
SLC17A5	5	1.63E+04	0.054	LYSOSOME
LIPA	1	3.82E+03	0.075	LYSOSOME
MAN2B1	13	2.40E+04	0.077	LYSOSOME
MFSD8	5	1.95E+04	0.086	LYSOSOME
PSAP	3	3.19E+03	0.097	LYSOSOME
CTSW	7	8.78E+03	0.102	LYSOSOME
SUMF1	3	4.22E+03	0.119	LYSOSOME
SMPD1	14	2.23E+04	0.136	LYSOSOME
PPT1	2	1.76E+03	0.142	LYSOSOME
ARSG	16	3.16E+04	0.148	LYSOSOME
ATP6V0D2	4	2.48E+03	0.149	LYSOSOME
ASAH1	10	1.65E+04	0.167	LYSOSOME
HEXB	4	9.65E+03	0.169	LYSOSOME
AP1B1	6	6.56E+03	0.177	LYSOSOME
CLN3	4	2.76E+03	0.221	LYSOSOME
AP4B1	4	6.62E+03	0.229	LYSOSOME
HEXA	6	7.40E+03	0.236	LYSOSOME
SLC11A1	11	1.91E+04	0.244	LYSOSOME
AP3M1	2	3.39E+03	0.245	LYSOSOME
ABCA2	16	2.67E+04	0.266	LYSOSOME
CTSF	2	3.50E+03	0.269	LYSOSOME
DNASE2	1	3.32E+03	0.270	LYSOSOME

NAPSA	4	2.00E+03	0.276	LYSOSOME
AP3D1	6	2.29E+03	0.282	LYSOSOME
AP1M2	4	4.05E+03	0.283	LYSOSOME
MANBA	4	6.91E+03	0.283	LYSOSOME
AP3B2	3	1.24E+03	0.291	LYSOSOME
PLA2G15	4	4.12E+03	0.293	LYSOSOME
CTSZ	3	3.74E+03	0.331	LYSOSOME
CLTA	1	3.60E+02	0.337	LYSOSOME
GGA3	15	2.69E+04	0.339	LYSOSOME
GM2A	3	4.62E+03	0.343	LYSOSOME
GNS	3	1.13E+03	0.346	LYSOSOME
NPC2	4	6.14E+03	0.348	LYSOSOME
TCIRG1	12	1.37E+04	0.354	LYSOSOME
CTSS	1	2.34E+02	0.384	LYSOSOME
IGF2R	28	1.97E+04	0.387	LYSOSOME
NAGLU	4	6.07E+03	0.390	LYSOSOME
LGMN	4	6.40E+03	0.392	LYSOSOME
ABCB9	5	6.37E+03	0.403	LYSOSOME
GALC	5	3.68E+03	0.404	LYSOSOME
ENTPD4	10	5.74E+03	0.406	LYSOSOME
NAGA	8	6.03E+03	0.444	LYSOSOME
LAPTM4B	4	2.71E+03	0.449	LYSOSOME
GAA	10	9.46E+03	0.449	LYSOSOME
IDUA	28	2.92E+04	0.465	LYSOSOME
ACP2	4	6.23E+03	0.465	LYSOSOME
CTSH	6	4.16E+03	0.466	LYSOSOME
AP4E1	4	1.62E+03	0.467	LYSOSOME
CLTB	2	5.56E+02	0.485	LYSOSOME
ARSA	4	1.13E+03	0.522	LYSOSOME
AP1S3	4	7.36E+03	0.524	LYSOSOME
CTSG	1	1.61E+02	0.531	LYSOSOME
ACP5	5	3.13E+03	0.540	LYSOSOME
GGA2	7	2.27E+03	0.541	LYSOSOME
ATP6V1H	3	1.38E+03	0.549	LYSOSOME
GNPTAB	8	7.29E+03	0.569	LYSOSOME
GNPTG	2	2.08E+02	0.577	LYSOSOME
DNASE2B	2	9.76E+02	0.583	LYSOSOME
HGSNAT	5	5.87E+03	0.592	LYSOSOME
ARSB	4	2.85E+03	0.604	LYSOSOME
NPC1	9	4.72E+03	0.623	LYSOSOME
LAMP3	2	3.65E+02	0.634	LYSOSOME
CTSC	5	8.46E+02	0.639	LYSOSOME
AP4S1	5	1.77E+03	0.649	LYSOSOME
CD63	1	4.79E+01	0.652	LYSOSOME
AP3M2	4	5.51E+02	0.656	LYSOSOME
CTSB	8	1.92E+03	0.666	LYSOSOME
GGA1	3	1.89E+03	0.671	LYSOSOME
GALNS	9	3.88E+03	0.677	LYSOSOME
CTSV	1	4.52E+01	0.680	LYSOSOME
CTSD	2	6.73E+02	0.681	LYSOSOME
LAPTM5	4	1.66E+03	0.690	LYSOSOME
AGA	6	5.47E+03	0.692	LYSOSOME
NAGPA	6	1.15E+03	0.693	LYSOSOME
ATP6V0A2	7	5.59E+03	0.706	LYSOSOME
LAPTM4A	2	3.84E+02	0.714	LYSOSOME
TPP1	6	1.60E+03	0.714	LYSOSOME
CTSA	3	6.21E+02	0.717	LYSOSOME
CLTCL1	22	2.13E+04	0.740	LYSOSOME
CTSL	1	1.89E+02	0.776	LYSOSOME
PSAPL1	12	6.80E+03	0.781	LYSOSOME
GUSB	3	6.23E+02	0.789	LYSOSOME
LAMP1	5	1.07E+03	0.798	LYSOSOME
CTSE	9	5.51E+03	0.808	LYSOSOME
HYAL1	2	2.59E+02	0.842	LYSOSOME
CLN5	5	8.98E+02	0.843	LYSOSOME
SCARB2	3	4.93E+02	0.865	LYSOSOME
PPT2	1	8.38E+00	0.872	LYSOSOME
FUCA1	4	5.41E+02	0.896	LYSOSOME
AP1G1	1	5.08E-02	0.993	LYSOSOME
AP1M1	2	4.33E-01	1	LYSOSOME
CTSO	2	1.02E+02	1	LYSOSOME
ATP6V0A1	7	9.40E+00	1	LYSOSOME
SLC11A2	5	2.69E+01	1	LYSOSOME
SGSH	8	2.42E+02	1	LYSOSOME

GLB1	7	7.02E+02	1	LYSOSOME
CD164	2	3.60E+00	1	LYSOSOME
AP4M1	9	7.97E+01	1	LYSOSOME
ATP6V0A4	9	3.09E+03	1	LYSOSOME
MCOLN1	5	3.10E+01	1	LYSOSOME
CD68	2	4.02E+01	1	LYSOSOME

Gene	NumVar	Q	Pvalue	Gene-set
DRD2	13	5.66E+06	2.15E-10	NEUROACTIVE LIGAND RECEPTOR INTERACTION
GABRA5	2	8.16E+03	0.002	NEUROACTIVE LIGAND RECEPTOR INTERACTION
PRLR	7	4.00E+04	0.004	NEUROACTIVE LIGAND RECEPTOR INTERACTION
C5AR1	7	4.11E+04	0.018	NEUROACTIVE LIGAND RECEPTOR INTERACTION
ADRA1D	8	4.79E+04	0.019	NEUROACTIVE LIGAND RECEPTOR INTERACTION
SSTR4	6	1.98E+04	0.026	NEUROACTIVE LIGAND RECEPTOR INTERACTION
CHRNA5	5	2.11E+04	0.028	NEUROACTIVE LIGAND RECEPTOR INTERACTION
GABRB2	1	3.28E+03	0.028	NEUROACTIVE LIGAND RECEPTOR INTERACTION
MC4R	5	4.32E+04	0.034	NEUROACTIVE LIGAND RECEPTOR INTERACTION
NMBR	7	3.42E+04	0.037	NEUROACTIVE LIGAND RECEPTOR INTERACTION
MLNR	1	1.22E+04	0.042	NEUROACTIVE LIGAND RECEPTOR INTERACTION
F2	5	7.45E+03	0.047	NEUROACTIVE LIGAND RECEPTOR INTERACTION
LEP	1	1.02E+03	0.048	NEUROACTIVE LIGAND RECEPTOR INTERACTION
APLNR	4	6.90E+03	0.065	NEUROACTIVE LIGAND RECEPTOR INTERACTION
P2RY6	4	5.25E+03	0.068	NEUROACTIVE LIGAND RECEPTOR INTERACTION
NPBWR1	3	5.56E+03	0.083	NEUROACTIVE LIGAND RECEPTOR INTERACTION
TAAR9	5	8.59E+03	0.085	NEUROACTIVE LIGAND RECEPTOR INTERACTION
GRIA2	1	1.09E+03	0.087	NEUROACTIVE LIGAND RECEPTOR INTERACTION
GHRHR	2	1.23E+04	0.088	NEUROACTIVE LIGAND RECEPTOR INTERACTION
GLRB	1	6.41E+02	0.089	NEUROACTIVE LIGAND RECEPTOR INTERACTION
ADRB1	1	1.27E+03	0.110	NEUROACTIVE LIGAND RECEPTOR INTERACTION
PTH1R	4	2.07E+04	0.116	NEUROACTIVE LIGAND RECEPTOR INTERACTION
GIPR	9	1.08E+04	0.116	NEUROACTIVE LIGAND RECEPTOR INTERACTION
F2RL1	4	1.12E+04	0.121	NEUROACTIVE LIGAND RECEPTOR INTERACTION
HTR5A	1	9.91E+02	0.126	NEUROACTIVE LIGAND RECEPTOR INTERACTION
CNR1	1	5.34E+02	0.126	NEUROACTIVE LIGAND RECEPTOR INTERACTION
GH1	2	1.58E+04	0.129	NEUROACTIVE LIGAND RECEPTOR INTERACTION
TBXA2R	12	2.12E+04	0.133	NEUROACTIVE LIGAND RECEPTOR INTERACTION
PRL	2	1.18E+03	0.138	NEUROACTIVE LIGAND RECEPTOR INTERACTION
ADRB2	2	8.42E+03	0.146	NEUROACTIVE LIGAND RECEPTOR INTERACTION
HTR1A	3	1.64E+04	0.147	NEUROACTIVE LIGAND RECEPTOR INTERACTION
CHRM2	3	8.45E+03	0.148	NEUROACTIVE LIGAND RECEPTOR INTERACTION
CHRNA4	10	8.29E+03	0.150	NEUROACTIVE LIGAND RECEPTOR INTERACTION
PTGFR	6	1.78E+04	0.150	NEUROACTIVE LIGAND RECEPTOR INTERACTION
S1PR2	4	1.54E+04	0.156	NEUROACTIVE LIGAND RECEPTOR INTERACTION
GABRR2	9	1.94E+04	0.160	NEUROACTIVE LIGAND RECEPTOR INTERACTION
PTGDR	6	1.47E+04	0.161	NEUROACTIVE LIGAND RECEPTOR INTERACTION
P2RX1	2	1.55E+03	0.162	NEUROACTIVE LIGAND RECEPTOR INTERACTION
CHRNA3	5	5.17E+03	0.168	NEUROACTIVE LIGAND RECEPTOR INTERACTION
HTR2B	1	2.85E+02	0.171	NEUROACTIVE LIGAND RECEPTOR INTERACTION
GRIA4	2	1.03E+03	0.177	NEUROACTIVE LIGAND RECEPTOR INTERACTION
GCGR	6	1.09E+04	0.184	NEUROACTIVE LIGAND RECEPTOR INTERACTION
CHRNA2	4	6.64E+03	0.191	NEUROACTIVE LIGAND RECEPTOR INTERACTION
HTR1B	1	6.22E+03	0.191	NEUROACTIVE LIGAND RECEPTOR INTERACTION
GRM7	1	4.46E+02	0.193	NEUROACTIVE LIGAND RECEPTOR INTERACTION
GPR83	3	2.96E+03	0.197	NEUROACTIVE LIGAND RECEPTOR INTERACTION
GABRB1	2	2.54E+03	0.202	NEUROACTIVE LIGAND RECEPTOR INTERACTION
CYSLTR2	7	5.38E+04	0.204	NEUROACTIVE LIGAND RECEPTOR INTERACTION
PLG	15	3.52E+04	0.207	NEUROACTIVE LIGAND RECEPTOR INTERACTION
CRHR2	11	2.24E+04	0.214	NEUROACTIVE LIGAND RECEPTOR INTERACTION
GRIK4	7	8.59E+03	0.216	NEUROACTIVE LIGAND RECEPTOR INTERACTION
GABRA4	2	4.84E+03	0.226	NEUROACTIVE LIGAND RECEPTOR INTERACTION
AVPR1B	9	1.01E+04	0.228	NEUROACTIVE LIGAND RECEPTOR INTERACTION
ADRA2A	4	3.12E+03	0.228	NEUROACTIVE LIGAND RECEPTOR INTERACTION
PRSS2	15	4.17E+04	0.232	NEUROACTIVE LIGAND RECEPTOR INTERACTION
GRM6	17	3.20E+04	0.234	NEUROACTIVE LIGAND RECEPTOR INTERACTION
TRPV1	12	9.79E+03	0.242	NEUROACTIVE LIGAND RECEPTOR INTERACTION
NMUR2	7	1.75E+04	0.244	NEUROACTIVE LIGAND RECEPTOR INTERACTION
GALR1	4	2.03E+03	0.250	NEUROACTIVE LIGAND RECEPTOR INTERACTION
PTGER1	6	6.37E+03	0.267	NEUROACTIVE LIGAND RECEPTOR INTERACTION
GABRG1	3	2.83E+03	0.276	NEUROACTIVE LIGAND RECEPTOR INTERACTION
GRIN2D	3	4.55E+03	0.287	NEUROACTIVE LIGAND RECEPTOR INTERACTION
CNR2	5	6.09E+03	0.298	NEUROACTIVE LIGAND RECEPTOR INTERACTION
FSHR	5	4.26E+03	0.301	NEUROACTIVE LIGAND RECEPTOR INTERACTION
S1PR4	11	1.41E+04	0.301	NEUROACTIVE LIGAND RECEPTOR INTERACTION
VIPR2	7	5.04E+03	0.302	NEUROACTIVE LIGAND RECEPTOR INTERACTION

HRH3	2	8.34E+02	0.304	NEUROACTIVE LIGAND RECEPTOR INTERACTION
TRHR	4	2.37E+03	0.307	NEUROACTIVE LIGAND RECEPTOR INTERACTION
CHRNE	17	1.74E+04	0.308	NEUROACTIVE LIGAND RECEPTOR INTERACTION
PTGIR	4	1.04E+04	0.308	NEUROACTIVE LIGAND RECEPTOR INTERACTION
MC2R	5	5.79E+03	0.313	NEUROACTIVE LIGAND RECEPTOR INTERACTION
TACR1	2	9.74E+02	0.318	NEUROACTIVE LIGAND RECEPTOR INTERACTION
LTB4R	4	1.86E+03	0.327	NEUROACTIVE LIGAND RECEPTOR INTERACTION
GRM5	4	1.84E+03	0.328	NEUROACTIVE LIGAND RECEPTOR INTERACTION
P2RX5	9	1.61E+04	0.333	NEUROACTIVE LIGAND RECEPTOR INTERACTION
PTAFR	3	2.22E+03	0.341	NEUROACTIVE LIGAND RECEPTOR INTERACTION
NPFFR1	8	5.71E+03	0.347	NEUROACTIVE LIGAND RECEPTOR INTERACTION
HRH2	3	1.80E+03	0.362	NEUROACTIVE LIGAND RECEPTOR INTERACTION
CHRNA10	6	8.79E+03	0.368	NEUROACTIVE LIGAND RECEPTOR INTERACTION
OPRM1	16	1.52E+04	0.374	NEUROACTIVE LIGAND RECEPTOR INTERACTION
DRD5	6	5.99E+03	0.376	NEUROACTIVE LIGAND RECEPTOR INTERACTION
CALCR	6	4.50E+03	0.378	NEUROACTIVE LIGAND RECEPTOR INTERACTION
GRM4	2	8.27E+02	0.380	NEUROACTIVE LIGAND RECEPTOR INTERACTION
GZMA	3	4.60E+03	0.383	NEUROACTIVE LIGAND RECEPTOR INTERACTION
GNRHR	5	5.57E+03	0.387	NEUROACTIVE LIGAND RECEPTOR INTERACTION
TAAR8	2	1.09E+03	0.390	NEUROACTIVE LIGAND RECEPTOR INTERACTION
DRD4	25	3.81E+04	0.398	NEUROACTIVE LIGAND RECEPTOR INTERACTION
GABRP	4	5.07E+03	0.402	NEUROACTIVE LIGAND RECEPTOR INTERACTION
GRIK2	3	4.87E+03	0.419	NEUROACTIVE LIGAND RECEPTOR INTERACTION
PRSS1	8	3.98E+03	0.425	NEUROACTIVE LIGAND RECEPTOR INTERACTION
FSHB	3	2.34E+03	0.432	NEUROACTIVE LIGAND RECEPTOR INTERACTION
TACR2	9	6.30E+03	0.433	NEUROACTIVE LIGAND RECEPTOR INTERACTION
CHRNA9	7	3.21E+03	0.434	NEUROACTIVE LIGAND RECEPTOR INTERACTION
C3AR1	2	3.22E+03	0.439	NEUROACTIVE LIGAND RECEPTOR INTERACTION
CSH1	1	1.02E+02	0.445	NEUROACTIVE LIGAND RECEPTOR INTERACTION
GRIA1	2	1.90E+03	0.459	NEUROACTIVE LIGAND RECEPTOR INTERACTION
PARD3	9	7.23E+03	0.460	NEUROACTIVE LIGAND RECEPTOR INTERACTION
GABBR1	3	1.77E+03	0.462	NEUROACTIVE LIGAND RECEPTOR INTERACTION
GPR156	9	5.24E+03	0.464	NEUROACTIVE LIGAND RECEPTOR INTERACTION
LHCGR	2	1.24E+03	0.466	NEUROACTIVE LIGAND RECEPTOR INTERACTION
S1PR5	4	6.22E+03	0.470	NEUROACTIVE LIGAND RECEPTOR INTERACTION
SSTR3	13	6.32E+03	0.471	NEUROACTIVE LIGAND RECEPTOR INTERACTION
GRIN2A	9	9.26E+03	0.475	NEUROACTIVE LIGAND RECEPTOR INTERACTION
MC5R	3	2.21E+03	0.475	NEUROACTIVE LIGAND RECEPTOR INTERACTION
MC3R	2	9.04E+02	0.477	NEUROACTIVE LIGAND RECEPTOR INTERACTION
NPY2R	1	2.28E+02	0.482	NEUROACTIVE LIGAND RECEPTOR INTERACTION
F2R	2	6.48E+02	0.484	NEUROACTIVE LIGAND RECEPTOR INTERACTION
SSTR5	14	5.90E+03	0.484	NEUROACTIVE LIGAND RECEPTOR INTERACTION
GABRR1	3	1.04E+03	0.486	NEUROACTIVE LIGAND RECEPTOR INTERACTION
ADRA1B	6	2.10E+03	0.501	NEUROACTIVE LIGAND RECEPTOR INTERACTION
GABRA2	3	2.24E+03	0.502	NEUROACTIVE LIGAND RECEPTOR INTERACTION
CHRNB1	2	3.64E+02	0.505	NEUROACTIVE LIGAND RECEPTOR INTERACTION
GALR2	4	6.54E+03	0.506	NEUROACTIVE LIGAND RECEPTOR INTERACTION
OPRK1	3	5.16E+02	0.507	NEUROACTIVE LIGAND RECEPTOR INTERACTION
GRM3	4	3.25E+03	0.520	NEUROACTIVE LIGAND RECEPTOR INTERACTION
CHRNA6	5	1.38E+03	0.522	NEUROACTIVE LIGAND RECEPTOR INTERACTION
LTB4R2	4	1.01E+03	0.527	NEUROACTIVE LIGAND RECEPTOR INTERACTION
CTSG	1	1.61E+02	0.531	NEUROACTIVE LIGAND RECEPTOR INTERACTION
FPR2	3	1.80E+03	0.531	NEUROACTIVE LIGAND RECEPTOR INTERACTION
F2RL2	4	8.44E+03	0.534	NEUROACTIVE LIGAND RECEPTOR INTERACTION
S1PR1	2	2.46E+03	0.537	NEUROACTIVE LIGAND RECEPTOR INTERACTION
CHRNB2	4	3.61E+03	0.542	NEUROACTIVE LIGAND RECEPTOR INTERACTION
NPY5R	1	7.21E+01	0.542	NEUROACTIVE LIGAND RECEPTOR INTERACTION
ADCYAP1R1	8	3.48E+03	0.544	NEUROACTIVE LIGAND RECEPTOR INTERACTION
GRIN3A	17	1.94E+04	0.547	NEUROACTIVE LIGAND RECEPTOR INTERACTION
GRIN3B	28	3.11E+04	0.548	NEUROACTIVE LIGAND RECEPTOR INTERACTION
TACR3	4	3.29E+03	0.549	NEUROACTIVE LIGAND RECEPTOR INTERACTION
CCKBR	2	5.36E+02	0.553	NEUROACTIVE LIGAND RECEPTOR INTERACTION
GPR35	9	1.59E+04	0.553	NEUROACTIVE LIGAND RECEPTOR INTERACTION
LPAR2	2	1.59E+03	0.554	NEUROACTIVE LIGAND RECEPTOR INTERACTION
HTR1D	5	1.45E+03	0.558	NEUROACTIVE LIGAND RECEPTOR INTERACTION
GRIN2C	12	8.47E+03	0.564	NEUROACTIVE LIGAND RECEPTOR INTERACTION
MCHR2	1	1.26E+03	0.564	NEUROACTIVE LIGAND RECEPTOR INTERACTION
GHSR	3	8.21E+02	0.565	NEUROACTIVE LIGAND RECEPTOR INTERACTION
ADRB3	5	1.22E+03	0.565	NEUROACTIVE LIGAND RECEPTOR INTERACTION
ADORA1	4	1.27E+03	0.566	NEUROACTIVE LIGAND RECEPTOR INTERACTION
TAAR5	11	7.90E+03	0.567	NEUROACTIVE LIGAND RECEPTOR INTERACTION
GABRG3	3	3.31E+03	0.573	NEUROACTIVE LIGAND RECEPTOR INTERACTION
CHRND	10	5.11E+03	0.578	NEUROACTIVE LIGAND RECEPTOR INTERACTION
ADORA2A	4	7.09E+02	0.579	NEUROACTIVE LIGAND RECEPTOR INTERACTION

TAAR6	8	6.24E+03	0.583	NEUROACTIVE LIGAND RECEPTOR INTERACTION
HCRTR1	6	2.57E+03	0.584	NEUROACTIVE LIGAND RECEPTOR INTERACTION
ADRA1A	11	1.61E+04	0.586	NEUROACTIVE LIGAND RECEPTOR INTERACTION
OPRD1	2	4.06E+02	0.587	NEUROACTIVE LIGAND RECEPTOR INTERACTION
SCTR	6	6.15E+03	0.589	NEUROACTIVE LIGAND RECEPTOR INTERACTION
FPR1	5	2.74E+03	0.591	NEUROACTIVE LIGAND RECEPTOR INTERACTION
CRHR1	4	7.95E+03	0.597	NEUROACTIVE LIGAND RECEPTOR INTERACTION
P2RY13	2	3.58E+03	0.602	NEUROACTIVE LIGAND RECEPTOR INTERACTION
P2RY11	9	8.60E+03	0.608	NEUROACTIVE LIGAND RECEPTOR INTERACTION
TAAR1	1	5.50E+01	0.616	NEUROACTIVE LIGAND RECEPTOR INTERACTION
P2RX7	13	1.34E+04	0.616	NEUROACTIVE LIGAND RECEPTOR INTERACTION
BDKRB1	6	2.20E+03	0.629	NEUROACTIVE LIGAND RECEPTOR INTERACTION
GLP2R	5	3.84E+03	0.631	NEUROACTIVE LIGAND RECEPTOR INTERACTION
GALR3	11	8.22E+03	0.634	NEUROACTIVE LIGAND RECEPTOR INTERACTION
KISS1R	2	5.28E+02	0.635	NEUROACTIVE LIGAND RECEPTOR INTERACTION
GABBR2	1	5.62E+01	0.635	NEUROACTIVE LIGAND RECEPTOR INTERACTION
GABRB3	1	5.09E+02	0.640	NEUROACTIVE LIGAND RECEPTOR INTERACTION
CHRNA7	9	5.03E+03	0.642	NEUROACTIVE LIGAND RECEPTOR INTERACTION
GLRA3	2	7.52E+02	0.646	NEUROACTIVE LIGAND RECEPTOR INTERACTION
PRLHR	3	3.97E+02	0.648	NEUROACTIVE LIGAND RECEPTOR INTERACTION
TAAR2	4	5.91E+02	0.650	NEUROACTIVE LIGAND RECEPTOR INTERACTION
CCKAR	3	1.40E+03	0.653	NEUROACTIVE LIGAND RECEPTOR INTERACTION
MTNR1A	7	5.22E+03	0.662	NEUROACTIVE LIGAND RECEPTOR INTERACTION
GRM8	3	4.28E+02	0.664	NEUROACTIVE LIGAND RECEPTOR INTERACTION
OPRL1	12	7.90E+03	0.665	NEUROACTIVE LIGAND RECEPTOR INTERACTION
NPFFR2	4	6.97E+02	0.666	NEUROACTIVE LIGAND RECEPTOR INTERACTION
TSPO	3	1.62E+03	0.667	NEUROACTIVE LIGAND RECEPTOR INTERACTION
FPR3	1	7.26E+01	0.671	NEUROACTIVE LIGAND RECEPTOR INTERACTION
S1PR3	8	6.21E+03	0.682	NEUROACTIVE LIGAND RECEPTOR INTERACTION
MCHR1	7	6.51E+03	0.683	NEUROACTIVE LIGAND RECEPTOR INTERACTION
P2RY14	2	2.63E+02	0.689	NEUROACTIVE LIGAND RECEPTOR INTERACTION
F2RL3	9	2.92E+03	0.689	NEUROACTIVE LIGAND RECEPTOR INTERACTION
NPBWR2	7	2.09E+03	0.695	NEUROACTIVE LIGAND RECEPTOR INTERACTION
NPY1R	4	1.41E+03	0.698	NEUROACTIVE LIGAND RECEPTOR INTERACTION
HTR4	4	9.27E+02	0.703	NEUROACTIVE LIGAND RECEPTOR INTERACTION
GABRA6	4	3.70E+03	0.709	NEUROACTIVE LIGAND RECEPTOR INTERACTION
GRID2	3	6.35E+02	0.709	NEUROACTIVE LIGAND RECEPTOR INTERACTION
UTS2R	5	2.45E+03	0.712	NEUROACTIVE LIGAND RECEPTOR INTERACTION
HTR7	3	2.25E+03	0.713	NEUROACTIVE LIGAND RECEPTOR INTERACTION
ADORA3	3	1.40E+03	0.716	NEUROACTIVE LIGAND RECEPTOR INTERACTION
GRM1	9	1.10E+04	0.716	NEUROACTIVE LIGAND RECEPTOR INTERACTION
GABRD	3	1.53E+03	0.724	NEUROACTIVE LIGAND RECEPTOR INTERACTION
CHRNA1	1	9.62E+01	0.725	NEUROACTIVE LIGAND RECEPTOR INTERACTION
SSTR2	2	1.37E+02	0.730	NEUROACTIVE LIGAND RECEPTOR INTERACTION
NTSR1	15	7.46E+03	0.732	NEUROACTIVE LIGAND RECEPTOR INTERACTION
GRM2	10	2.77E+03	0.744	NEUROACTIVE LIGAND RECEPTOR INTERACTION
MAS1	5	1.91E+03	0.746	NEUROACTIVE LIGAND RECEPTOR INTERACTION
EDNRB	4	4.16E+03	0.746	NEUROACTIVE LIGAND RECEPTOR INTERACTION
P2RX3	3	1.58E+02	0.748	NEUROACTIVE LIGAND RECEPTOR INTERACTION
CHRNB3	2	3.62E+02	0.758	NEUROACTIVE LIGAND RECEPTOR INTERACTION
CHRNB4	6	2.86E+03	0.758	NEUROACTIVE LIGAND RECEPTOR INTERACTION
LPAR6	3	1.01E+03	0.761	NEUROACTIVE LIGAND RECEPTOR INTERACTION
NTSR2	7	2.75E+03	0.775	NEUROACTIVE LIGAND RECEPTOR INTERACTION
LHB	3	1.75E+03	0.780	NEUROACTIVE LIGAND RECEPTOR INTERACTION
GH2	4	5.26E+02	0.781	NEUROACTIVE LIGAND RECEPTOR INTERACTION
VIPR1	6	9.78E+02	0.785	NEUROACTIVE LIGAND RECEPTOR INTERACTION
ADORA2B	2	8.40E+01	0.789	NEUROACTIVE LIGAND RECEPTOR INTERACTION
RXFP1	3	2.71E+02	0.792	NEUROACTIVE LIGAND RECEPTOR INTERACTION
GRID1	3	1.34E+03	0.794	NEUROACTIVE LIGAND RECEPTOR INTERACTION
CHRNG	8	7.82E+03	0.797	NEUROACTIVE LIGAND RECEPTOR INTERACTION
HCRTR2	4	1.35E+03	0.809	NEUROACTIVE LIGAND RECEPTOR INTERACTION
GABRA1	1	1.48E+01	0.809	NEUROACTIVE LIGAND RECEPTOR INTERACTION
AGTR1	4	5.93E+02	0.816	NEUROACTIVE LIGAND RECEPTOR INTERACTION
NPY4R	6	8.62E+02	0.818	NEUROACTIVE LIGAND RECEPTOR INTERACTION
RXFP2	8	4.25E+03	0.819	NEUROACTIVE LIGAND RECEPTOR INTERACTION
OXTR	5	1.39E+03	0.831	NEUROACTIVE LIGAND RECEPTOR INTERACTION
GLP1R	8	1.62E+03	0.834	NEUROACTIVE LIGAND RECEPTOR INTERACTION
HTR2A	4	1.62E+03	0.843	NEUROACTIVE LIGAND RECEPTOR INTERACTION
SSTR1	1	5.80E+00	0.848	NEUROACTIVE LIGAND RECEPTOR INTERACTION
HRH1	6	6.30E+02	0.857	NEUROACTIVE LIGAND RECEPTOR INTERACTION
HTR1F	1	4.98E+01	0.857	NEUROACTIVE LIGAND RECEPTOR INTERACTION
CALCRL	1	2.34E+01	0.864	NEUROACTIVE LIGAND RECEPTOR INTERACTION
PTGER2	2	1.14E+03	0.871	NEUROACTIVE LIGAND RECEPTOR INTERACTION
LEPR	9	1.82E+03	0.874	NEUROACTIVE LIGAND RECEPTOR INTERACTION

GHR	8	1.46E+03	0.874	NEUROACTIVE LIGAND RECEPTOR INTERACTION
AVPR1A	4	1.37E+03	0.875	NEUROACTIVE LIGAND RECEPTOR INTERACTION
MTNR1B	6	4.84E+02	0.878	NEUROACTIVE LIGAND RECEPTOR INTERACTION
CHRM5	6	2.85E+02	0.885	NEUROACTIVE LIGAND RECEPTOR INTERACTION
MC1R	17	2.05E+03	0.891	NEUROACTIVE LIGAND RECEPTOR INTERACTION
TSHR	5	4.59E+02	0.895	NEUROACTIVE LIGAND RECEPTOR INTERACTION
PTGER3	1	1.97E+01	0.901	NEUROACTIVE LIGAND RECEPTOR INTERACTION
HTR1E	1	7.94E+00	0.909	NEUROACTIVE LIGAND RECEPTOR INTERACTION
CHRM4	1	2.02E+00	0.914	NEUROACTIVE LIGAND RECEPTOR INTERACTION
THRA	1	8.38E-01	0.948	NEUROACTIVE LIGAND RECEPTOR INTERACTION
DRD3	1	1.26E-01	0.982	NEUROACTIVE LIGAND RECEPTOR INTERACTION
ADRA2C	1	5.32E-02	0.983	NEUROACTIVE LIGAND RECEPTOR INTERACTION
PTGER4	1	2.47E-01	0.993	NEUROACTIVE LIGAND RECEPTOR INTERACTION
CHRM3	3	2.38E+02	1	NEUROACTIVE LIGAND RECEPTOR INTERACTION
GRIK1	4	7.47E+00	1	NEUROACTIVE LIGAND RECEPTOR INTERACTION
P2RX4	9	3.74E+02	1	NEUROACTIVE LIGAND RECEPTOR INTERACTION
HTR6	6	5.49E+01	1	NEUROACTIVE LIGAND RECEPTOR INTERACTION
THRB	3	5.72E+01	1	NEUROACTIVE LIGAND RECEPTOR INTERACTION
PRSS3	13	1.72E+03	1	NEUROACTIVE LIGAND RECEPTOR INTERACTION
NR3C1	2	2.23E+02	1	NEUROACTIVE LIGAND RECEPTOR INTERACTION
PTH2R	7	1.16E+02	1	NEUROACTIVE LIGAND RECEPTOR INTERACTION
P2RY2	6	1.00E+01	1	NEUROACTIVE LIGAND RECEPTOR INTERACTION
P2RX2	5	4.35E+02	1	NEUROACTIVE LIGAND RECEPTOR INTERACTION
P2RX6	2	1.55E+00	1	NEUROACTIVE LIGAND RECEPTOR INTERACTION
NMUR1	8	8.72E+01	1	NEUROACTIVE LIGAND RECEPTOR INTERACTION
LPAR3	2	2.37E+01	1	NEUROACTIVE LIGAND RECEPTOR INTERACTION
DRD1	2	6.12E-01	1	NEUROACTIVE LIGAND RECEPTOR INTERACTION
GRIK5	9	2.36E+02	1	NEUROACTIVE LIGAND RECEPTOR INTERACTION
HRH4	2	5.02E+01	1	NEUROACTIVE LIGAND RECEPTOR INTERACTION
GLRA1	3	2.52E+01	1	NEUROACTIVE LIGAND RECEPTOR INTERACTION
ADRA2B	3	7.47E+01	1	NEUROACTIVE LIGAND RECEPTOR INTERACTION
BDKRB2	2	6.28E+00	1	NEUROACTIVE LIGAND RECEPTOR INTERACTION
GRIK3	2	3.34E+01	1	NEUROACTIVE LIGAND RECEPTOR INTERACTION

Gene	NumVar	Q	Pvalue	Gene-set
NDUFA10	3	3.11E+04	0.001	PARKINSONS DISEASE
NDUFS8	9	2.11E+04	0.008	PARKINSONS DISEASE
LRRK2	18	5.07E+04	0.016	PARKINSONS DISEASE
APAF1	13	3.41E+04	0.020	PARKINSONS DISEASE
UQCR10	1	7.97E+03	0.037	PARKINSONS DISEASE
UQCR11	1	6.97E+02	0.047	PARKINSONS DISEASE
NDUFS7	10	4.16E+04	0.058	PARKINSONS DISEASE
COX7A1	2	1.95E+03	0.075	PARKINSONS DISEASE
NDUFA7	2	1.24E+04	0.077	PARKINSONS DISEASE
PRKN	10	2.32E+04	0.093	PARKINSONS DISEASE
COX6B2	1	2.21E+03	0.107	PARKINSONS DISEASE
NDUFS5	1	4.64E+02	0.109	PARKINSONS DISEASE
NDUFS6	1	4.30E+02	0.135	PARKINSONS DISEASE
NDUFB5	1	6.02E+03	0.159	PARKINSONS DISEASE
COX6A2	2	4.59E+03	0.168	PARKINSONS DISEASE
UCHL1	2	2.11E+03	0.174	PARKINSONS DISEASE
GPR37	7	1.15E+04	0.206	PARKINSONS DISEASE
VDAC3	3	3.66E+03	0.206	PARKINSONS DISEASE
NDUFA9	5	8.13E+03	0.240	PARKINSONS DISEASE
VDAC1	2	5.69E+02	0.247	PARKINSONS DISEASE
NDUFA2	2	1.54E+03	0.275	PARKINSONS DISEASE
UQCRB	1	3.58E+02	0.292	PARKINSONS DISEASE
SDHB	2	6.63E+03	0.299	PARKINSONS DISEASE
NDUFA4L2	1	3.40E+02	0.307	PARKINSONS DISEASE
SLC6A3	5	2.85E+03	0.338	PARKINSONS DISEASE
NDUFB3	4	2.55E+03	0.350	PARKINSONS DISEASE
COX6A1	3	1.24E+03	0.358	PARKINSONS DISEASE
NDUFS4	2	4.76E+02	0.396	PARKINSONS DISEASE
UBE2J1	1	8.39E+01	0.433	PARKINSONS DISEASE
SNCAIP	10	1.58E+04	0.434	PARKINSONS DISEASE
NDUFS3	1	9.32E+02	0.436	PARKINSONS DISEASE
NDUFB6	2	6.88E+02	0.440	PARKINSONS DISEASE
NDUFB9	1	4.30E+02	0.443	PARKINSONS DISEASE
CYC1	5	3.74E+03	0.448	PARKINSONS DISEASE
COX4I1	2	1.10E+03	0.451	PARKINSONS DISEASE
NDUFA3	1	1.99E+03	0.452	PARKINSONS DISEASE
SDHD	4	9.99E+03	0.452	PARKINSONS DISEASE
UQCRC1	6	6.87E+03	0.454	PARKINSONS DISEASE
COX8C	1	1.25E+03	0.465	PARKINSONS DISEASE

COX7A2L	2	6.42E+02	0.468	PARKINSONS DISEASE
SLC18A1	6	2.39E+03	0.532	PARKINSONS DISEASE
NDUFS2	3	2.06E+03	0.544	PARKINSONS DISEASE
SLC25A31	4	2.00E+03	0.547	PARKINSONS DISEASE
COX5B	1	1.23E+02	0.550	PARKINSONS DISEASE
UBE2L6	1	6.07E+01	0.557	PARKINSONS DISEASE
NDUFB10	1	4.50E+01	0.589	PARKINSONS DISEASE
CASP9	8	6.15E+03	0.602	PARKINSONS DISEASE
SDHA	2	4.17E+02	0.608	PARKINSONS DISEASE
NDUFA6	1	3.99E+02	0.625	PARKINSONS DISEASE
UBA7	11	6.11E+03	0.630	PARKINSONS DISEASE
VDAC2	1	4.55E+01	0.632	PARKINSONS DISEASE
NDUFV1	1	4.58E+01	0.671	PARKINSONS DISEASE
SDHC	3	2.73E+03	0.715	PARKINSONS DISEASE
COX5A	4	6.19E+02	0.750	PARKINSONS DISEASE
COX4I2	3	3.59E+02	0.754	PARKINSONS DISEASE
TH	4	1.08E+03	0.757	PARKINSONS DISEASE
PARK7	3	8.53E+02	0.765	PARKINSONS DISEASE
COX7A2	1	4.51E+01	0.796	PARKINSONS DISEASE
UQCRC2	3	3.68E+02	0.823	PARKINSONS DISEASE
NDUFA5	3	2.42E+02	0.857	PARKINSONS DISEASE
HTRA2	4	7.48E+02	0.871	PARKINSONS DISEASE
NDUFV3	7	7.46E+02	0.884	PARKINSONS DISEASE
COX6B1	1	2.55E+00	0.911	PARKINSONS DISEASE
UQCRHL	1	2.88E+00	0.915	PARKINSONS DISEASE
COX6C	1	1.03E+01	0.939	PARKINSONS DISEASE
UBE2J2	2	1.71E+01	1	PARKINSONS DISEASE
CASP3	2	2.05E+02	1	PARKINSONS DISEASE
NDUFS1	9	1.78E+01	1	PARKINSONS DISEASE
PPID	2	7.90E+01	1	PARKINSONS DISEASE
PINK1	4	5.35E+01	1	PARKINSONS DISEASE
NDUFB1	3	6.84E+02	1	PARKINSONS DISEASE

Gene	NumVar	Q	Pvalue	Gene-set
TOP2B	11	1.08E+05	2.88E-04	POST TRANSLATIONAL PROTEIN MODIFICATION
ABRAXAS1	3	3.26E+04	0.001	POST TRANSLATIONAL PROTEIN MODIFICATION
CSNK1D	4	1.32E+04	0.002	POST TRANSLATIONAL PROTEIN MODIFICATION
HIST1H2BA	3	6.50E+03	0.003	POST TRANSLATIONAL PROTEIN MODIFICATION
MUC4	870	1.68E+07	0.006	POST TRANSLATIONAL PROTEIN MODIFICATION
GAN	5	3.02E+04	0.006	POST TRANSLATIONAL PROTEIN MODIFICATION
HDAC4	9	1.96E+04	0.009	POST TRANSLATIONAL PROTEIN MODIFICATION
HIST2H2BE	1	5.20E+03	0.009	POST TRANSLATIONAL PROTEIN MODIFICATION
BST1	5	4.91E+04	0.010	POST TRANSLATIONAL PROTEIN MODIFICATION
ADAMTS19	8	3.14E+04	0.010	POST TRANSLATIONAL PROTEIN MODIFICATION
MAN1B1	15	3.58E+04	0.010	POST TRANSLATIONAL PROTEIN MODIFICATION
DCAF10	3	1.03E+04	0.010	POST TRANSLATIONAL PROTEIN MODIFICATION
COG5	10	3.93E+04	0.011	POST TRANSLATIONAL PROTEIN MODIFICATION
BRCA1	19	4.99E+04	0.012	POST TRANSLATIONAL PROTEIN MODIFICATION
CSF1	6	1.82E+04	0.012	POST TRANSLATIONAL PROTEIN MODIFICATION
NUCB1	5	3.87E+04	0.013	POST TRANSLATIONAL PROTEIN MODIFICATION
ARFGAP1	8	2.88E+04	0.013	POST TRANSLATIONAL PROTEIN MODIFICATION
PHC1	8	3.98E+04	0.014	POST TRANSLATIONAL PROTEIN MODIFICATION
SENP5	9	4.23E+04	0.015	POST TRANSLATIONAL PROTEIN MODIFICATION
ENGASE	15	4.17E+04	0.015	POST TRANSLATIONAL PROTEIN MODIFICATION
TUBB1	8	4.79E+04	0.016	POST TRANSLATIONAL PROTEIN MODIFICATION
RTN4RL1	5	3.58E+04	0.016	POST TRANSLATIONAL PROTEIN MODIFICATION
ST6GALNAC6	4	2.75E+04	0.016	POST TRANSLATIONAL PROTEIN MODIFICATION
FBXW12	7	3.12E+04	0.016	POST TRANSLATIONAL PROTEIN MODIFICATION
WDTC1	3	6.36E+03	0.018	POST TRANSLATIONAL PROTEIN MODIFICATION
CDC25A	2	2.36E+04	0.018	POST TRANSLATIONAL PROTEIN MODIFICATION
NOD1	8	1.04E+04	0.018	POST TRANSLATIONAL PROTEIN MODIFICATION
DDX17	4	6.54E+03	0.018	POST TRANSLATIONAL PROTEIN MODIFICATION
HIST1H2BE	2	1.05E+04	0.019	POST TRANSLATIONAL PROTEIN MODIFICATION
SATB1	3	4.50E+03	0.020	POST TRANSLATIONAL PROTEIN MODIFICATION
COPG1	3	5.26E+03	0.020	POST TRANSLATIONAL PROTEIN MODIFICATION
HIST1H2BO	1	8.61E+02	0.021	POST TRANSLATIONAL PROTEIN MODIFICATION
ST8SIA3	2	2.68E+03	0.021	POST TRANSLATIONAL PROTEIN MODIFICATION
RAB44	19	9.95E+04	0.021	POST TRANSLATIONAL PROTEIN MODIFICATION
APP	2	3.42E+03	0.021	POST TRANSLATIONAL PROTEIN MODIFICATION
B4GALT1	1	1.34E+03	0.022	POST TRANSLATIONAL PROTEIN MODIFICATION
VCP	2	3.84E+03	0.022	POST TRANSLATIONAL PROTEIN MODIFICATION
ASGR1	1	8.46E+02	0.023	POST TRANSLATIONAL PROTEIN MODIFICATION
USP17L17	3	1.95E+04	0.027	POST TRANSLATIONAL PROTEIN MODIFICATION
NUP35	2	4.09E+03	0.027	POST TRANSLATIONAL PROTEIN MODIFICATION

FBXW8	9	2.08E+04	0.029	POST TRANSLATIONAL PROTEIN MODIFICATION
ACTR10	2	4.89E+03	0.029	POST TRANSLATIONAL PROTEIN MODIFICATION
EDEM1	8	1.37E+04	0.030	POST TRANSLATIONAL PROTEIN MODIFICATION
SOCS3	1	7.91E+02	0.030	POST TRANSLATIONAL PROTEIN MODIFICATION
DYNC1LI2	2	2.58E+03	0.030	POST TRANSLATIONAL PROTEIN MODIFICATION
STT3A	2	2.10E+03	0.031	POST TRANSLATIONAL PROTEIN MODIFICATION
SPON2	5	3.66E+04	0.033	POST TRANSLATIONAL PROTEIN MODIFICATION
MGAT1	1	1.98E+03	0.036	POST TRANSLATIONAL PROTEIN MODIFICATION
ADAMTS4	14	4.21E+04	0.036	POST TRANSLATIONAL PROTEIN MODIFICATION
ITIH2	13	4.30E+04	0.037	POST TRANSLATIONAL PROTEIN MODIFICATION
DYNC1LI1	5	2.20E+04	0.038	POST TRANSLATIONAL PROTEIN MODIFICATION
FBXL12	5	8.45E+03	0.038	POST TRANSLATIONAL PROTEIN MODIFICATION
ABRAXAS2	2	2.64E+03	0.039	POST TRANSLATIONAL PROTEIN MODIFICATION
USP5	1	5.28E+02	0.042	POST TRANSLATIONAL PROTEIN MODIFICATION
NLRP3	5	1.80E+04	0.044	POST TRANSLATIONAL PROTEIN MODIFICATION
TAF10	5	7.04E+03	0.045	POST TRANSLATIONAL PROTEIN MODIFICATION
KLHL9	1	3.46E+03	0.046	POST TRANSLATIONAL PROTEIN MODIFICATION
F2	5	7.45E+03	0.047	POST TRANSLATIONAL PROTEIN MODIFICATION
FBXL8	2	5.90E+03	0.049	POST TRANSLATIONAL PROTEIN MODIFICATION
MUC17	222	2.02E+06	0.049	POST TRANSLATIONAL PROTEIN MODIFICATION
AGBL5	5	2.55E+04	0.051	POST TRANSLATIONAL PROTEIN MODIFICATION
DDB2	2	6.07E+03	0.053	POST TRANSLATIONAL PROTEIN MODIFICATION
PLET1	4	2.35E+04	0.054	POST TRANSLATIONAL PROTEIN MODIFICATION
SLC17A5	5	1.63E+04	0.054	POST TRANSLATIONAL PROTEIN MODIFICATION
ICMT	2	1.00E+04	0.057	POST TRANSLATIONAL PROTEIN MODIFICATION
USP17L3	12	4.50E+04	0.057	POST TRANSLATIONAL PROTEIN MODIFICATION
USP17L1	12	4.50E+04	0.057	POST TRANSLATIONAL PROTEIN MODIFICATION
NUP107	5	1.43E+04	0.058	POST TRANSLATIONAL PROTEIN MODIFICATION
TUBB8	5	1.83E+04	0.058	POST TRANSLATIONAL PROTEIN MODIFICATION
NUP93	5	9.66E+03	0.059	POST TRANSLATIONAL PROTEIN MODIFICATION
GOLM1	12	2.69E+04	0.059	POST TRANSLATIONAL PROTEIN MODIFICATION
NPL	6	4.06E+03	0.060	POST TRANSLATIONAL PROTEIN MODIFICATION
DOLK	6	1.71E+04	0.060	POST TRANSLATIONAL PROTEIN MODIFICATION
FBXL7	5	6.27E+03	0.061	POST TRANSLATIONAL PROTEIN MODIFICATION
USP7	3	5.36E+03	0.062	POST TRANSLATIONAL PROTEIN MODIFICATION
IL6	3	2.27E+04	0.062	POST TRANSLATIONAL PROTEIN MODIFICATION
RXRA	3	9.87E+03	0.064	POST TRANSLATIONAL PROTEIN MODIFICATION
MAVS	5	5.63E+03	0.064	POST TRANSLATIONAL PROTEIN MODIFICATION
HIST1H2AJ	1	8.79E+02	0.065	POST TRANSLATIONAL PROTEIN MODIFICATION
ANKRD9	1	1.75E+03	0.067	POST TRANSLATIONAL PROTEIN MODIFICATION
ALG6	1	1.24E+04	0.068	POST TRANSLATIONAL PROTEIN MODIFICATION
PIGK	1	9.37E+02	0.069	POST TRANSLATIONAL PROTEIN MODIFICATION
TECTA	15	6.03E+04	0.070	POST TRANSLATIONAL PROTEIN MODIFICATION
COG4	5	1.41E+04	0.072	POST TRANSLATIONAL PROTEIN MODIFICATION
CNTN3	11	4.49E+04	0.073	POST TRANSLATIONAL PROTEIN MODIFICATION
RUVBL1	3	4.18E+03	0.074	POST TRANSLATIONAL PROTEIN MODIFICATION
RAB13	1	6.28E+03	0.074	POST TRANSLATIONAL PROTEIN MODIFICATION
CREBBP	19	3.85E+04	0.075	POST TRANSLATIONAL PROTEIN MODIFICATION
NEU2	3	9.10E+03	0.076	POST TRANSLATIONAL PROTEIN MODIFICATION
HIST1H2BG	2	1.64E+03	0.076	POST TRANSLATIONAL PROTEIN MODIFICATION
TEX101	1	5.16E+02	0.080	POST TRANSLATIONAL PROTEIN MODIFICATION
CDK1	2	1.73E+03	0.081	POST TRANSLATIONAL PROTEIN MODIFICATION
USP24	13	1.74E+04	0.082	POST TRANSLATIONAL PROTEIN MODIFICATION
TPGS1	5	1.43E+04	0.082	POST TRANSLATIONAL PROTEIN MODIFICATION
HGS	8	9.40E+03	0.083	POST TRANSLATIONAL PROTEIN MODIFICATION
KNG1	2	3.00E+03	0.084	POST TRANSLATIONAL PROTEIN MODIFICATION
FBXW9	8	7.14E+04	0.085	POST TRANSLATIONAL PROTEIN MODIFICATION
ATXN7	13	2.42E+04	0.086	POST TRANSLATIONAL PROTEIN MODIFICATION
RPS27A	1	2.69E+03	0.087	POST TRANSLATIONAL PROTEIN MODIFICATION
GATA3	2	1.45E+03	0.087	POST TRANSLATIONAL PROTEIN MODIFICATION
CAND1	4	3.76E+03	0.090	POST TRANSLATIONAL PROTEIN MODIFICATION
AFP	7	2.60E+04	0.091	POST TRANSLATIONAL PROTEIN MODIFICATION
EDEM2	4	9.43E+03	0.091	POST TRANSLATIONAL PROTEIN MODIFICATION
RNF135	10	3.45E+04	0.091	POST TRANSLATIONAL PROTEIN MODIFICATION
UBE2T	1	8.78E+02	0.091	POST TRANSLATIONAL PROTEIN MODIFICATION
LARGE2	12	2.28E+04	0.091	POST TRANSLATIONAL PROTEIN MODIFICATION
ARFGAP2	3	1.43E+04	0.092	POST TRANSLATIONAL PROTEIN MODIFICATION
PRKN	10	2.32E+04	0.093	POST TRANSLATIONAL PROTEIN MODIFICATION
STX5	6	1.12E+04	0.094	POST TRANSLATIONAL PROTEIN MODIFICATION
LY6E	1	9.34E+02	0.095	POST TRANSLATIONAL PROTEIN MODIFICATION
MCRS1	1	7.50E+02	0.095	POST TRANSLATIONAL PROTEIN MODIFICATION
COPB2	2	1.95E+03	0.096	POST TRANSLATIONAL PROTEIN MODIFICATION
PPP6R3	5	2.60E+04	0.096	POST TRANSLATIONAL PROTEIN MODIFICATION
SEH1L	2	1.17E+04	0.097	POST TRANSLATIONAL PROTEIN MODIFICATION

SPTBN2	17	3.45E+04	0.097	POST TRANSLATIONAL PROTEIN MODIFICATION
PARP1	8	1.22E+04	0.098	POST TRANSLATIONAL PROTEIN MODIFICATION
ST6GAL2	10	3.49E+04	0.099	POST TRANSLATIONAL PROTEIN MODIFICATION
RPN1	8	7.20E+03	0.100	POST TRANSLATIONAL PROTEIN MODIFICATION
RELA	3	8.06E+03	0.100	POST TRANSLATIONAL PROTEIN MODIFICATION
KIN	2	1.30E+03	0.101	POST TRANSLATIONAL PROTEIN MODIFICATION
THSD7A	8	1.41E+04	0.102	POST TRANSLATIONAL PROTEIN MODIFICATION
TRAPPC6A	4	5.10E+03	0.102	POST TRANSLATIONAL PROTEIN MODIFICATION
COMMD2	3	6.47E+03	0.103	POST TRANSLATIONAL PROTEIN MODIFICATION
MUC3A	58	4.50E+05	0.104	POST TRANSLATIONAL PROTEIN MODIFICATION
APOB	42	8.05E+04	0.105	POST TRANSLATIONAL PROTEIN MODIFICATION
USP17L4	4	1.12E+04	0.105	POST TRANSLATIONAL PROTEIN MODIFICATION
ASB2	11	2.81E+04	0.105	POST TRANSLATIONAL PROTEIN MODIFICATION
WDR48	2	7.15E+03	0.106	POST TRANSLATIONAL PROTEIN MODIFICATION
KLHL25	4	2.00E+04	0.106	POST TRANSLATIONAL PROTEIN MODIFICATION
USP20	11	3.19E+04	0.107	POST TRANSLATIONAL PROTEIN MODIFICATION
DPH6	6	4.45E+03	0.107	POST TRANSLATIONAL PROTEIN MODIFICATION
HIST1H2BH	1	7.15E+02	0.109	POST TRANSLATIONAL PROTEIN MODIFICATION
TAB1	3	2.23E+03	0.109	POST TRANSLATIONAL PROTEIN MODIFICATION
APOL1	13	7.55E+03	0.112	POST TRANSLATIONAL PROTEIN MODIFICATION
SCG2	3	1.23E+04	0.113	POST TRANSLATIONAL PROTEIN MODIFICATION
GALNTL6	2	1.09E+03	0.113	POST TRANSLATIONAL PROTEIN MODIFICATION
NUP210	28	3.39E+04	0.114	POST TRANSLATIONAL PROTEIN MODIFICATION
TFAP2C	2	1.22E+03	0.114	POST TRANSLATIONAL PROTEIN MODIFICATION
VNN2	7	2.23E+04	0.116	POST TRANSLATIONAL PROTEIN MODIFICATION
NR2C1	4	5.86E+03	0.116	POST TRANSLATIONAL PROTEIN MODIFICATION
GBF1	5	5.83E+03	0.116	POST TRANSLATIONAL PROTEIN MODIFICATION
HIST1H4F	1	3.78E+03	0.118	POST TRANSLATIONAL PROTEIN MODIFICATION
CBX4	4	1.09E+04	0.118	POST TRANSLATIONAL PROTEIN MODIFICATION
SOCS6	2	2.26E+03	0.119	POST TRANSLATIONAL PROTEIN MODIFICATION
SUMF1	3	4.22E+03	0.119	POST TRANSLATIONAL PROTEIN MODIFICATION
TTLL9	7	1.33E+04	0.120	POST TRANSLATIONAL PROTEIN MODIFICATION
CCNF	5	1.49E+04	0.120	POST TRANSLATIONAL PROTEIN MODIFICATION
MUC16	190	2.15E+05	0.122	POST TRANSLATIONAL PROTEIN MODIFICATION
TPR	16	2.16E+04	0.123	POST TRANSLATIONAL PROTEIN MODIFICATION
KLHL11	2	4.89E+03	0.126	POST TRANSLATIONAL PROTEIN MODIFICATION
STX17	2	6.90E+03	0.126	POST TRANSLATIONAL PROTEIN MODIFICATION
CD52	1	4.15E+03	0.126	POST TRANSLATIONAL PROTEIN MODIFICATION
HERC3	5	8.36E+03	0.128	POST TRANSLATIONAL PROTEIN MODIFICATION
SIAH2	3	1.50E+03	0.130	POST TRANSLATIONAL PROTEIN MODIFICATION
RAB23	2	8.09E+03	0.130	POST TRANSLATIONAL PROTEIN MODIFICATION
GALNT13	7	4.99E+03	0.131	POST TRANSLATIONAL PROTEIN MODIFICATION
TMED9	2	2.02E+03	0.132	POST TRANSLATIONAL PROTEIN MODIFICATION
UBA2	3	1.30E+04	0.133	POST TRANSLATIONAL PROTEIN MODIFICATION
DCAF4	12	3.95E+04	0.134	POST TRANSLATIONAL PROTEIN MODIFICATION
RNF181	3	1.71E+04	0.135	POST TRANSLATIONAL PROTEIN MODIFICATION
RAB3D	3	3.42E+03	0.137	POST TRANSLATIONAL PROTEIN MODIFICATION
XPC	9	2.41E+04	0.139	POST TRANSLATIONAL PROTEIN MODIFICATION
PEX12	3	1.33E+04	0.139	POST TRANSLATIONAL PROTEIN MODIFICATION
DNMT1	17	1.99E+04	0.139	POST TRANSLATIONAL PROTEIN MODIFICATION
RAB21	2	1.08E+04	0.142	POST TRANSLATIONAL PROTEIN MODIFICATION
ST3GAL1	4	1.96E+04	0.142	POST TRANSLATIONAL PROTEIN MODIFICATION
B4GAT1	1	1.23E+03	0.145	POST TRANSLATIONAL PROTEIN MODIFICATION
ADRB2	2	8.42E+03	0.146	POST TRANSLATIONAL PROTEIN MODIFICATION
XRCC4	5	1.65E+04	0.147	POST TRANSLATIONAL PROTEIN MODIFICATION
ST6GALNAC1	2	8.30E+03	0.147	POST TRANSLATIONAL PROTEIN MODIFICATION
SYVN1	3	1.88E+03	0.148	POST TRANSLATIONAL PROTEIN MODIFICATION
ARSG	16	3.16E+04	0.148	POST TRANSLATIONAL PROTEIN MODIFICATION
FBXO6	4	9.69E+03	0.149	POST TRANSLATIONAL PROTEIN MODIFICATION
AGBL4	10	1.43E+04	0.149	POST TRANSLATIONAL PROTEIN MODIFICATION
DNAJC3	2	1.63E+03	0.151	POST TRANSLATIONAL PROTEIN MODIFICATION
SMC3	3	1.59E+03	0.152	POST TRANSLATIONAL PROTEIN MODIFICATION
BTBD6	4	8.20E+03	0.154	POST TRANSLATIONAL PROTEIN MODIFICATION
MDM2	1	8.78E+02	0.156	POST TRANSLATIONAL PROTEIN MODIFICATION
MDGA2	8	1.29E+04	0.157	POST TRANSLATIONAL PROTEIN MODIFICATION
TUBA8	4	1.16E+04	0.157	POST TRANSLATIONAL PROTEIN MODIFICATION
RAB3B	1	2.47E+02	0.157	POST TRANSLATIONAL PROTEIN MODIFICATION
DCTN4	5	1.08E+04	0.159	POST TRANSLATIONAL PROTEIN MODIFICATION
APOA1	3	2.38E+03	0.160	POST TRANSLATIONAL PROTEIN MODIFICATION
POMT2	3	3.09E+03	0.160	POST TRANSLATIONAL PROTEIN MODIFICATION
CHD3	9	1.46E+04	0.162	POST TRANSLATIONAL PROTEIN MODIFICATION
TFG	1	1.70E+03	0.163	POST TRANSLATIONAL PROTEIN MODIFICATION
FKBP8	2	7.87E+03	0.163	POST TRANSLATIONAL PROTEIN MODIFICATION
MBD1	9	1.11E+04	0.164	POST TRANSLATIONAL PROTEIN MODIFICATION

RNF185	2	3.37E+03	0.164	POST TRANSLATIONAL PROTEIN MODIFICATION
FBXW7	1	3.72E+02	0.166	POST TRANSLATIONAL PROTEIN MODIFICATION
ANKRD28	11	1.70E+04	0.167	POST TRANSLATIONAL PROTEIN MODIFICATION
KLHL5	6	1.00E+04	0.168	POST TRANSLATIONAL PROTEIN MODIFICATION
APOA5	1	8.75E+02	0.168	POST TRANSLATIONAL PROTEIN MODIFICATION
NUP88	7	1.35E+04	0.169	POST TRANSLATIONAL PROTEIN MODIFICATION
RAB4A	1	1.18E+03	0.169	POST TRANSLATIONAL PROTEIN MODIFICATION
B3GLCT	5	7.78E+03	0.171	POST TRANSLATIONAL PROTEIN MODIFICATION
MGAT4B	6	2.03E+04	0.171	POST TRANSLATIONAL PROTEIN MODIFICATION
TRAPPC4	1	8.59E+02	0.171	POST TRANSLATIONAL PROTEIN MODIFICATION
RARA	2	8.08E+02	0.174	POST TRANSLATIONAL PROTEIN MODIFICATION
RAB5A	1	4.72E+02	0.174	POST TRANSLATIONAL PROTEIN MODIFICATION
UCHL1	2	2.11E+03	0.174	POST TRANSLATIONAL PROTEIN MODIFICATION
COPA	5	2.91E+03	0.178	POST TRANSLATIONAL PROTEIN MODIFICATION
RAB32	4	2.02E+03	0.179	POST TRANSLATIONAL PROTEIN MODIFICATION
COG7	6	6.85E+03	0.179	POST TRANSLATIONAL PROTEIN MODIFICATION
INO80B	2	1.01E+03	0.179	POST TRANSLATIONAL PROTEIN MODIFICATION
PUM2	3	5.04E+03	0.180	POST TRANSLATIONAL PROTEIN MODIFICATION
SPTAN1	7	9.73E+03	0.184	POST TRANSLATIONAL PROTEIN MODIFICATION
RAE1	3	1.19E+04	0.185	POST TRANSLATIONAL PROTEIN MODIFICATION
COMMD1	1	6.58E+03	0.186	POST TRANSLATIONAL PROTEIN MODIFICATION
POM121C	24	4.47E+04	0.186	POST TRANSLATIONAL PROTEIN MODIFICATION
HIST1H2AH	1	4.43E+03	0.189	POST TRANSLATIONAL PROTEIN MODIFICATION
FOXL2	5	9.96E+03	0.189	POST TRANSLATIONAL PROTEIN MODIFICATION
IFIH1	18	3.25E+04	0.190	POST TRANSLATIONAL PROTEIN MODIFICATION
ALG12	2	4.93E+03	0.190	POST TRANSLATIONAL PROTEIN MODIFICATION
THSD1	6	1.44E+04	0.191	POST TRANSLATIONAL PROTEIN MODIFICATION
HIST1H2AI	1	7.34E+02	0.193	POST TRANSLATIONAL PROTEIN MODIFICATION
CTBP1	3	9.89E+02	0.194	POST TRANSLATIONAL PROTEIN MODIFICATION
ZRANB1	3	1.82E+03	0.194	POST TRANSLATIONAL PROTEIN MODIFICATION
LARGE1	5	9.48E+03	0.195	POST TRANSLATIONAL PROTEIN MODIFICATION
RAB3C	1	2.88E+02	0.196	POST TRANSLATIONAL PROTEIN MODIFICATION
METTL22	3	5.81E+03	0.196	POST TRANSLATIONAL PROTEIN MODIFICATION
HIST3H2A	1	4.51E+02	0.197	POST TRANSLATIONAL PROTEIN MODIFICATION
FBXO32	2	1.26E+03	0.197	POST TRANSLATIONAL PROTEIN MODIFICATION
B4GALT5	1	6.10E+03	0.198	POST TRANSLATIONAL PROTEIN MODIFICATION
NDC1	3	9.43E+03	0.200	POST TRANSLATIONAL PROTEIN MODIFICATION
PEX2	6	1.49E+04	0.201	POST TRANSLATIONAL PROTEIN MODIFICATION
ASB1	1	4.19E+02	0.201	POST TRANSLATIONAL PROTEIN MODIFICATION
SBSPON	2	2.70E+03	0.202	POST TRANSLATIONAL PROTEIN MODIFICATION
ADAM10	2	1.51E+03	0.202	POST TRANSLATIONAL PROTEIN MODIFICATION
TRAPPC6B	1	7.57E+02	0.203	POST TRANSLATIONAL PROTEIN MODIFICATION
INO80	11	2.11E+04	0.203	POST TRANSLATIONAL PROTEIN MODIFICATION
APLP2	6	3.94E+03	0.205	POST TRANSLATIONAL PROTEIN MODIFICATION
ALG14	4	9.85E+03	0.206	POST TRANSLATIONAL PROTEIN MODIFICATION
CNTN4	6	4.65E+03	0.206	POST TRANSLATIONAL PROTEIN MODIFICATION
FBXO15	2	8.31E+02	0.206	POST TRANSLATIONAL PROTEIN MODIFICATION
VDAC3	3	3.66E+03	0.206	POST TRANSLATIONAL PROTEIN MODIFICATION
HLTF	9	1.87E+04	0.209	POST TRANSLATIONAL PROTEIN MODIFICATION
USP10	8	1.30E+04	0.210	POST TRANSLATIONAL PROTEIN MODIFICATION
SMURF2	3	9.38E+03	0.212	POST TRANSLATIONAL PROTEIN MODIFICATION
ADAMTS14	20	1.77E+04	0.212	POST TRANSLATIONAL PROTEIN MODIFICATION
DCTN6	1	9.42E+02	0.212	POST TRANSLATIONAL PROTEIN MODIFICATION
UHRF2	3	6.42E+03	0.212	POST TRANSLATIONAL PROTEIN MODIFICATION
ADAMTSL3	23	3.19E+04	0.213	POST TRANSLATIONAL PROTEIN MODIFICATION
VCAN	28	5.06E+04	0.214	POST TRANSLATIONAL PROTEIN MODIFICATION
BIRC2	1	1.56E+03	0.217	POST TRANSLATIONAL PROTEIN MODIFICATION
USP21	5	1.18E+04	0.218	POST TRANSLATIONAL PROTEIN MODIFICATION
RHOT1	3	1.46E+03	0.219	POST TRANSLATIONAL PROTEIN MODIFICATION
POM121	22	3.63E+04	0.219	POST TRANSLATIONAL PROTEIN MODIFICATION
GOLGB1	31	3.79E+04	0.219	POST TRANSLATIONAL PROTEIN MODIFICATION
CHST4	5	1.40E+04	0.220	POST TRANSLATIONAL PROTEIN MODIFICATION
MUC5B	234	3.54E+05	0.221	POST TRANSLATIONAL PROTEIN MODIFICATION
ULBP2	3	1.85E+03	0.224	POST TRANSLATIONAL PROTEIN MODIFICATION
PML	14	1.70E+04	0.224	POST TRANSLATIONAL PROTEIN MODIFICATION
CTR9	5	5.64E+03	0.225	POST TRANSLATIONAL PROTEIN MODIFICATION
SMAD3	1	5.08E+02	0.227	POST TRANSLATIONAL PROTEIN MODIFICATION
MGAT3	6	6.56E+03	0.227	POST TRANSLATIONAL PROTEIN MODIFICATION
RAB27A	6	1.83E+03	0.227	POST TRANSLATIONAL PROTEIN MODIFICATION
DNMT3A	5	4.10E+03	0.228	POST TRANSLATIONAL PROTEIN MODIFICATION
DCTN5	1	2.97E+02	0.228	POST TRANSLATIONAL PROTEIN MODIFICATION
AGBL2	7	1.51E+04	0.229	POST TRANSLATIONAL PROTEIN MODIFICATION
RAB19	1	2.88E+02	0.231	POST TRANSLATIONAL PROTEIN MODIFICATION
ADAMTS2	17	3.35E+04	0.231	POST TRANSLATIONAL PROTEIN MODIFICATION

USP34	16	2.90E+04	0.232	POST TRANSLATIONAL PROTEIN MODIFICATION
ASB13	2	7.27E+02	0.232	POST TRANSLATIONAL PROTEIN MODIFICATION
KAT2A	5	3.10E+03	0.234	POST TRANSLATIONAL PROTEIN MODIFICATION
MUC6	40	7.95E+04	0.234	POST TRANSLATIONAL PROTEIN MODIFICATION
USP42	24	3.77E+04	0.237	POST TRANSLATIONAL PROTEIN MODIFICATION
SELENOS	4	2.36E+03	0.238	POST TRANSLATIONAL PROTEIN MODIFICATION
ADAMTS13	17	1.81E+04	0.240	POST TRANSLATIONAL PROTEIN MODIFICATION
TNIP1	6	1.05E+04	0.242	POST TRANSLATIONAL PROTEIN MODIFICATION
VCPIP1	2	1.10E+03	0.244	POST TRANSLATIONAL PROTEIN MODIFICATION
FBXO7	4	2.88E+03	0.245	POST TRANSLATIONAL PROTEIN MODIFICATION
ELOC	1	2.61E+02	0.246	POST TRANSLATIONAL PROTEIN MODIFICATION
TGFBR2	5	7.00E+03	0.247	POST TRANSLATIONAL PROTEIN MODIFICATION
VDAC1	2	5.69E+02	0.247	POST TRANSLATIONAL PROTEIN MODIFICATION
ARSJ	4	5.16E+03	0.247	POST TRANSLATIONAL PROTEIN MODIFICATION
KLHL2	1	1.59E+03	0.248	POST TRANSLATIONAL PROTEIN MODIFICATION
GPAA1	5	9.37E+03	0.248	POST TRANSLATIONAL PROTEIN MODIFICATION
PIGV	4	5.19E+03	0.249	POST TRANSLATIONAL PROTEIN MODIFICATION
PPARGC1A	3	4.03E+03	0.250	POST TRANSLATIONAL PROTEIN MODIFICATION
EPAS1	6	1.52E+04	0.250	POST TRANSLATIONAL PROTEIN MODIFICATION
CST3	1	4.78E+02	0.251	POST TRANSLATIONAL PROTEIN MODIFICATION
ADAMTSL1	18	2.13E+04	0.252	POST TRANSLATIONAL PROTEIN MODIFICATION
ASB4	3	1.16E+03	0.253	POST TRANSLATIONAL PROTEIN MODIFICATION
ASB10	20	2.29E+04	0.253	POST TRANSLATIONAL PROTEIN MODIFICATION
DPH2	3	1.36E+03	0.254	POST TRANSLATIONAL PROTEIN MODIFICATION
PEX13	5	4.41E+03	0.255	POST TRANSLATIONAL PROTEIN MODIFICATION
BGLAP	1	1.37E+03	0.256	POST TRANSLATIONAL PROTEIN MODIFICATION
METTL21A	1	6.13E+02	0.258	POST TRANSLATIONAL PROTEIN MODIFICATION
TTLL10	23	4.60E+04	0.260	POST TRANSLATIONAL PROTEIN MODIFICATION
PSCA	2	5.56E+03	0.261	POST TRANSLATIONAL PROTEIN MODIFICATION
DYNC1I2	4	9.48E+02	0.261	POST TRANSLATIONAL PROTEIN MODIFICATION
FURIN	8	1.42E+04	0.262	POST TRANSLATIONAL PROTEIN MODIFICATION
F5	40	4.85E+04	0.263	POST TRANSLATIONAL PROTEIN MODIFICATION
PIAS3	3	7.08E+03	0.264	POST TRANSLATIONAL PROTEIN MODIFICATION
HIST1H2BI	2	1.64E+03	0.267	POST TRANSLATIONAL PROTEIN MODIFICATION
EEF2	1	2.17E+02	0.267	POST TRANSLATIONAL PROTEIN MODIFICATION
ST8SIA5	2	1.29E+03	0.267	POST TRANSLATIONAL PROTEIN MODIFICATION
HIST1H4L	1	3.15E+03	0.268	POST TRANSLATIONAL PROTEIN MODIFICATION
TTLL1	1	2.22E+02	0.268	POST TRANSLATIONAL PROTEIN MODIFICATION
LY6G6C	3	8.31E+03	0.268	POST TRANSLATIONAL PROTEIN MODIFICATION
MUC21	84	6.98E+05	0.268	POST TRANSLATIONAL PROTEIN MODIFICATION
PSMB6	1	3.10E+03	0.270	POST TRANSLATIONAL PROTEIN MODIFICATION
LYPD6B	1	1.20E+03	0.272	POST TRANSLATIONAL PROTEIN MODIFICATION
UBE2Z	1	5.36E+02	0.272	POST TRANSLATIONAL PROTEIN MODIFICATION
NUP37	1	3.70E+02	0.273	POST TRANSLATIONAL PROTEIN MODIFICATION
SPP1	6	3.52E+03	0.274	POST TRANSLATIONAL PROTEIN MODIFICATION
FBXW5	16	1.87E+04	0.274	POST TRANSLATIONAL PROTEIN MODIFICATION
DCUN1D4	4	3.98E+03	0.276	POST TRANSLATIONAL PROTEIN MODIFICATION
TUBA1C	2	6.49E+03	0.276	POST TRANSLATIONAL PROTEIN MODIFICATION
A4GNT	5	3.94E+03	0.276	POST TRANSLATIONAL PROTEIN MODIFICATION
MEPE	7	1.02E+04	0.277	POST TRANSLATIONAL PROTEIN MODIFICATION
GALNT1	3	1.14E+03	0.277	POST TRANSLATIONAL PROTEIN MODIFICATION
FEM1B	1	6.25E+02	0.280	POST TRANSLATIONAL PROTEIN MODIFICATION
RAB4B	3	8.82E+02	0.281	POST TRANSLATIONAL PROTEIN MODIFICATION
MATN3	6	1.36E+04	0.281	POST TRANSLATIONAL PROTEIN MODIFICATION
FBXL22	7	1.05E+04	0.282	POST TRANSLATIONAL PROTEIN MODIFICATION
GORASP1	8	2.49E+04	0.282	POST TRANSLATIONAL PROTEIN MODIFICATION
PIGF	2	8.01E+03	0.282	POST TRANSLATIONAL PROTEIN MODIFICATION
AGBL1	16	3.45E+04	0.283	POST TRANSLATIONAL PROTEIN MODIFICATION
ELOB	1	3.42E+02	0.283	POST TRANSLATIONAL PROTEIN MODIFICATION
CAMKMT	2	6.22E+02	0.283	POST TRANSLATIONAL PROTEIN MODIFICATION
TP53	2	7.01E+02	0.284	POST TRANSLATIONAL PROTEIN MODIFICATION
DPH7	4	8.52E+03	0.285	POST TRANSLATIONAL PROTEIN MODIFICATION
F10	5	4.86E+03	0.285	POST TRANSLATIONAL PROTEIN MODIFICATION
BTRC	3	1.17E+03	0.285	POST TRANSLATIONAL PROTEIN MODIFICATION
DHDDS	2	1.39E+03	0.287	POST TRANSLATIONAL PROTEIN MODIFICATION
FEM1C	1	4.06E+02	0.287	POST TRANSLATIONAL PROTEIN MODIFICATION
AAAS	5	3.35E+03	0.287	POST TRANSLATIONAL PROTEIN MODIFICATION
ADAMTSL2	12	1.78E+04	0.288	POST TRANSLATIONAL PROTEIN MODIFICATION
DCUN1D5	1	6.90E+02	0.288	POST TRANSLATIONAL PROTEIN MODIFICATION
ASB5	3	1.38E+03	0.288	POST TRANSLATIONAL PROTEIN MODIFICATION
CEACAM7	3	4.25E+03	0.288	POST TRANSLATIONAL PROTEIN MODIFICATION
RRAGA	1	1.60E+02	0.289	POST TRANSLATIONAL PROTEIN MODIFICATION
OTUB1	1	3.02E+02	0.291	POST TRANSLATIONAL PROTEIN MODIFICATION
LY6G6D	2	5.83E+02	0.291	POST TRANSLATIONAL PROTEIN MODIFICATION

TMEM115	2	7.51E+02	0.292	POST TRANSLATIONAL PROTEIN MODIFICATION
SEC22B	1	1.94E+02	0.292	POST TRANSLATIONAL PROTEIN MODIFICATION
RING1	3	2.73E+03	0.294	POST TRANSLATIONAL PROTEIN MODIFICATION
YY1	3	3.08E+03	0.295	POST TRANSLATIONAL PROTEIN MODIFICATION
BARD1	9	2.12E+04	0.296	POST TRANSLATIONAL PROTEIN MODIFICATION
SKP2	2	4.24E+02	0.299	POST TRANSLATIONAL PROTEIN MODIFICATION
TUBB2B	2	1.47E+03	0.301	POST TRANSLATIONAL PROTEIN MODIFICATION
PRKDC	32	3.16E+04	0.301	POST TRANSLATIONAL PROTEIN MODIFICATION
SEC24D	9	1.62E+04	0.302	POST TRANSLATIONAL PROTEIN MODIFICATION
HIST1H2BB	1	1.81E+02	0.305	POST TRANSLATIONAL PROTEIN MODIFICATION
NCOA2	11	6.63E+03	0.305	POST TRANSLATIONAL PROTEIN MODIFICATION
ERCC8	7	5.20E+03	0.305	POST TRANSLATIONAL PROTEIN MODIFICATION
NTNG1	1	1.84E+02	0.305	POST TRANSLATIONAL PROTEIN MODIFICATION
RAB15	2	5.18E+03	0.306	POST TRANSLATIONAL PROTEIN MODIFICATION
FBXO10	6	4.16E+03	0.307	POST TRANSLATIONAL PROTEIN MODIFICATION
HIST1H4C	1	3.67E+02	0.307	POST TRANSLATIONAL PROTEIN MODIFICATION
MUC5AC	201	2.34E+05	0.307	POST TRANSLATIONAL PROTEIN MODIFICATION
DYNC1I1	2	1.00E+03	0.308	POST TRANSLATIONAL PROTEIN MODIFICATION
USP2	5	8.15E+03	0.308	POST TRANSLATIONAL PROTEIN MODIFICATION
LRR1	3	4.98E+03	0.309	POST TRANSLATIONAL PROTEIN MODIFICATION
SEC23IP	9	1.31E+04	0.310	POST TRANSLATIONAL PROTEIN MODIFICATION
CPM	3	7.55E+03	0.310	POST TRANSLATIONAL PROTEIN MODIFICATION
B3GNT6	7	6.21E+03	0.312	POST TRANSLATIONAL PROTEIN MODIFICATION
NR1H3	3	2.30E+03	0.312	POST TRANSLATIONAL PROTEIN MODIFICATION
TFAP2B	2	1.20E+03	0.313	POST TRANSLATIONAL PROTEIN MODIFICATION
FGF23	2	8.34E+02	0.314	POST TRANSLATIONAL PROTEIN MODIFICATION
DCUN1D2	5	2.09E+03	0.314	POST TRANSLATIONAL PROTEIN MODIFICATION
FOXK2	11	1.16E+04	0.314	POST TRANSLATIONAL PROTEIN MODIFICATION
PGM3	4	1.48E+03	0.315	POST TRANSLATIONAL PROTEIN MODIFICATION
MUC20	14	2.38E+04	0.315	POST TRANSLATIONAL PROTEIN MODIFICATION
GALNTL5	5	6.49E+03	0.317	POST TRANSLATIONAL PROTEIN MODIFICATION
C1GALT1	3	1.46E+03	0.317	POST TRANSLATIONAL PROTEIN MODIFICATION
MUC13	5	7.88E+03	0.317	POST TRANSLATIONAL PROTEIN MODIFICATION
SLC35C1	1	2.24E+03	0.318	POST TRANSLATIONAL PROTEIN MODIFICATION
TADA3	2	9.89E+02	0.318	POST TRANSLATIONAL PROTEIN MODIFICATION
FAM20A	3	1.75E+03	0.319	POST TRANSLATIONAL PROTEIN MODIFICATION
TTLL7	5	6.70E+03	0.320	POST TRANSLATIONAL PROTEIN MODIFICATION
PRMT3	7	3.22E+03	0.321	POST TRANSLATIONAL PROTEIN MODIFICATION
RAB12	1	5.41E+02	0.321	POST TRANSLATIONAL PROTEIN MODIFICATION
SMAD4	3	1.81E+03	0.322	POST TRANSLATIONAL PROTEIN MODIFICATION
TOPORS	10	2.12E+04	0.323	POST TRANSLATIONAL PROTEIN MODIFICATION
NOD2	32	2.88E+04	0.323	POST TRANSLATIONAL PROTEIN MODIFICATION
TRAF2	2	1.30E+03	0.323	POST TRANSLATIONAL PROTEIN MODIFICATION
LYPD8	4	4.11E+03	0.323	POST TRANSLATIONAL PROTEIN MODIFICATION
RAB38	4	8.72E+03	0.323	POST TRANSLATIONAL PROTEIN MODIFICATION
ASB15	5	2.79E+03	0.324	POST TRANSLATIONAL PROTEIN MODIFICATION
NUP153	21	2.59E+04	0.326	POST TRANSLATIONAL PROTEIN MODIFICATION
PIGG	11	8.27E+03	0.327	POST TRANSLATIONAL PROTEIN MODIFICATION
FBXO4	3	4.17E+03	0.329	POST TRANSLATIONAL PROTEIN MODIFICATION
KAT2B	10	1.04E+04	0.330	POST TRANSLATIONAL PROTEIN MODIFICATION
SEC16A	27	3.63E+04	0.330	POST TRANSLATIONAL PROTEIN MODIFICATION
CTSZ	3	3.74E+03	0.331	POST TRANSLATIONAL PROTEIN MODIFICATION
RAB25	4	4.99E+03	0.333	POST TRANSLATIONAL PROTEIN MODIFICATION
LAMB1	13	2.20E+04	0.333	POST TRANSLATIONAL PROTEIN MODIFICATION
COMMD8	2	4.33E+03	0.334	POST TRANSLATIONAL PROTEIN MODIFICATION
SEC24A	7	5.44E+03	0.334	POST TRANSLATIONAL PROTEIN MODIFICATION
NCOR2	31	3.07E+04	0.335	POST TRANSLATIONAL PROTEIN MODIFICATION
NTM	5	1.40E+04	0.335	POST TRANSLATIONAL PROTEIN MODIFICATION
LSAMP	1	1.81E+02	0.337	POST TRANSLATIONAL PROTEIN MODIFICATION
MDC1	90	1.53E+05	0.338	POST TRANSLATIONAL PROTEIN MODIFICATION
ST8SIA6	3	2.83E+03	0.339	POST TRANSLATIONAL PROTEIN MODIFICATION
ST3GAL4	6	6.28E+03	0.341	POST TRANSLATIONAL PROTEIN MODIFICATION
UBXN7	1	1.74E+03	0.341	POST TRANSLATIONAL PROTEIN MODIFICATION
TTLL11	5	8.31E+03	0.342	POST TRANSLATIONAL PROTEIN MODIFICATION
SEC24B	6	2.88E+03	0.343	POST TRANSLATIONAL PROTEIN MODIFICATION
RNF20	2	1.95E+03	0.345	POST TRANSLATIONAL PROTEIN MODIFICATION
CD109	19	3.65E+04	0.346	POST TRANSLATIONAL PROTEIN MODIFICATION
SPTBN4	18	2.07E+04	0.348	POST TRANSLATIONAL PROTEIN MODIFICATION
FBXW2	3	1.60E+03	0.348	POST TRANSLATIONAL PROTEIN MODIFICATION
NSMCE4A	2	1.84E+03	0.348	POST TRANSLATIONAL PROTEIN MODIFICATION
RAB8B	1	4.00E+02	0.349	POST TRANSLATIONAL PROTEIN MODIFICATION
TTL	2	4.02E+03	0.349	POST TRANSLATIONAL PROTEIN MODIFICATION
CUL9	26	5.49E+04	0.350	POST TRANSLATIONAL PROTEIN MODIFICATION
PSMC5	1	2.08E+02	0.350	POST TRANSLATIONAL PROTEIN MODIFICATION

TMED2	1	2.34E+02	0.351	POST TRANSLATIONAL PROTEIN MODIFICATION
LYPD1	6	7.61E+03	0.352	POST TRANSLATIONAL PROTEIN MODIFICATION
GALNT5	13	1.71E+04	0.355	POST TRANSLATIONAL PROTEIN MODIFICATION
GALNT16	7	4.41E+03	0.355	POST TRANSLATIONAL PROTEIN MODIFICATION
ADAMTS7	49	5.90E+04	0.356	POST TRANSLATIONAL PROTEIN MODIFICATION
AMBN	9	5.92E+03	0.357	POST TRANSLATIONAL PROTEIN MODIFICATION
TOMM70	3	1.16E+03	0.357	POST TRANSLATIONAL PROTEIN MODIFICATION
CBX8	4	8.69E+03	0.359	POST TRANSLATIONAL PROTEIN MODIFICATION
B3GNT2	1	1.31E+03	0.361	POST TRANSLATIONAL PROTEIN MODIFICATION
HIC1	8	1.56E+04	0.361	POST TRANSLATIONAL PROTEIN MODIFICATION
PDIA3	4	2.46E+03	0.362	POST TRANSLATIONAL PROTEIN MODIFICATION
B3GNT8	7	4.24E+03	0.364	POST TRANSLATIONAL PROTEIN MODIFICATION
FPGT	5	4.58E+03	0.365	POST TRANSLATIONAL PROTEIN MODIFICATION
TSTA3	5	1.74E+03	0.365	POST TRANSLATIONAL PROTEIN MODIFICATION
FBXL18	6	9.68E+03	0.366	POST TRANSLATIONAL PROTEIN MODIFICATION
IGFBP5	1	3.50E+02	0.368	POST TRANSLATIONAL PROTEIN MODIFICATION
LAMC1	12	1.22E+04	0.369	POST TRANSLATIONAL PROTEIN MODIFICATION
MYSM1	4	5.24E+03	0.369	POST TRANSLATIONAL PROTEIN MODIFICATION
NAE1	2	6.58E+02	0.369	POST TRANSLATIONAL PROTEIN MODIFICATION
TNIP2	7	8.26E+03	0.372	POST TRANSLATIONAL PROTEIN MODIFICATION
ADAMTS17	14	9.24E+03	0.374	POST TRANSLATIONAL PROTEIN MODIFICATION
NUP133	8	1.02E+04	0.375	POST TRANSLATIONAL PROTEIN MODIFICATION
DMP1	10	8.86E+03	0.375	POST TRANSLATIONAL PROTEIN MODIFICATION
USP3	1	2.80E+02	0.376	POST TRANSLATIONAL PROTEIN MODIFICATION
USP49	4	2.39E+03	0.376	POST TRANSLATIONAL PROTEIN MODIFICATION
TF	7	4.94E+03	0.377	POST TRANSLATIONAL PROTEIN MODIFICATION
DCAF5	5	4.62E+03	0.382	POST TRANSLATIONAL PROTEIN MODIFICATION
GALNT8	10	1.33E+04	0.382	POST TRANSLATIONAL PROTEIN MODIFICATION
SMC6	3	8.16E+02	0.383	POST TRANSLATIONAL PROTEIN MODIFICATION
QSOX1	7	1.18E+04	0.383	POST TRANSLATIONAL PROTEIN MODIFICATION
ADAMTS3	14	1.80E+04	0.384	POST TRANSLATIONAL PROTEIN MODIFICATION
DDB1	1	2.67E+02	0.384	POST TRANSLATIONAL PROTEIN MODIFICATION
DNMT3B	7	7.20E+03	0.384	POST TRANSLATIONAL PROTEIN MODIFICATION
MBD5	9	4.94E+03	0.385	POST TRANSLATIONAL PROTEIN MODIFICATION
UBXN1	2	4.55E+03	0.387	POST TRANSLATIONAL PROTEIN MODIFICATION
USP37	7	1.32E+04	0.387	POST TRANSLATIONAL PROTEIN MODIFICATION
PLAUR	7	7.17E+03	0.389	POST TRANSLATIONAL PROTEIN MODIFICATION
SMC5	7	3.04E+03	0.389	POST TRANSLATIONAL PROTEIN MODIFICATION
FN3K	4	5.51E+03	0.391	POST TRANSLATIONAL PROTEIN MODIFICATION
MUC15	4	4.94E+03	0.392	POST TRANSLATIONAL PROTEIN MODIFICATION
RFT1	7	4.00E+03	0.395	POST TRANSLATIONAL PROTEIN MODIFICATION
PIGT	7	1.21E+04	0.396	POST TRANSLATIONAL PROTEIN MODIFICATION
ST6GALNAC4	1	5.47E+02	0.399	POST TRANSLATIONAL PROTEIN MODIFICATION
SCG3	1	1.28E+02	0.400	POST TRANSLATIONAL PROTEIN MODIFICATION
ZNF350	6	4.58E+03	0.400	POST TRANSLATIONAL PROTEIN MODIFICATION
LYPD3	4	3.96E+03	0.402	POST TRANSLATIONAL PROTEIN MODIFICATION
GANAB	5	6.35E+03	0.403	POST TRANSLATIONAL PROTEIN MODIFICATION
CCDC8	8	5.75E+03	0.405	POST TRANSLATIONAL PROTEIN MODIFICATION
B4GALT4	3	3.04E+03	0.405	POST TRANSLATIONAL PROTEIN MODIFICATION
OPCML	2	5.81E+02	0.405	POST TRANSLATIONAL PROTEIN MODIFICATION
NSF	1	2.81E+02	0.406	POST TRANSLATIONAL PROTEIN MODIFICATION
HIPK2	7	2.35E+03	0.406	POST TRANSLATIONAL PROTEIN MODIFICATION
CAPZB	2	3.66E+03	0.406	POST TRANSLATIONAL PROTEIN MODIFICATION
SLC35A1	2	1.90E+03	0.407	POST TRANSLATIONAL PROTEIN MODIFICATION
FBXO30	2	4.60E+03	0.409	POST TRANSLATIONAL PROTEIN MODIFICATION
DHPS	10	7.58E+03	0.411	POST TRANSLATIONAL PROTEIN MODIFICATION
EVA1A	1	2.41E+02	0.412	POST TRANSLATIONAL PROTEIN MODIFICATION
POMGNT1	5	7.54E+03	0.412	POST TRANSLATIONAL PROTEIN MODIFICATION
THSD4	13	1.68E+04	0.413	POST TRANSLATIONAL PROTEIN MODIFICATION
PNPLA2	10	1.22E+04	0.413	POST TRANSLATIONAL PROTEIN MODIFICATION
PSMB11	7	1.01E+04	0.414	POST TRANSLATIONAL PROTEIN MODIFICATION
COPG2	3	6.16E+03	0.414	POST TRANSLATIONAL PROTEIN MODIFICATION
LYPD2	1	2.13E+02	0.415	POST TRANSLATIONAL PROTEIN MODIFICATION
PSMB9	5	8.02E+03	0.415	POST TRANSLATIONAL PROTEIN MODIFICATION
INO80D	8	1.13E+04	0.415	POST TRANSLATIONAL PROTEIN MODIFICATION
KDELR3	2	1.72E+03	0.416	POST TRANSLATIONAL PROTEIN MODIFICATION
DDX58	7	1.02E+04	0.417	POST TRANSLATIONAL PROTEIN MODIFICATION
NR1H2	1	1.08E+02	0.417	POST TRANSLATIONAL PROTEIN MODIFICATION
UBE2M	1	8.16E+02	0.418	POST TRANSLATIONAL PROTEIN MODIFICATION
PSMB1	1	1.16E+02	0.419	POST TRANSLATIONAL PROTEIN MODIFICATION
DPH5	2	6.78E+02	0.419	POST TRANSLATIONAL PROTEIN MODIFICATION
FBXW4	3	1.15E+04	0.420	POST TRANSLATIONAL PROTEIN MODIFICATION
TUBA3C	4	1.05E+04	0.420	POST TRANSLATIONAL PROTEIN MODIFICATION
CDKN2A	2	1.50E+03	0.421	POST TRANSLATIONAL PROTEIN MODIFICATION

RCN1	3	4.27E+03	0.422	POST TRANSLATIONAL PROTEIN MODIFICATION
CFTR	36	2.43E+04	0.422	POST TRANSLATIONAL PROTEIN MODIFICATION
PCGF2	1	9.00E+01	0.422	POST TRANSLATIONAL PROTEIN MODIFICATION
ADAMTS20	15	3.59E+04	0.423	POST TRANSLATIONAL PROTEIN MODIFICATION
DAG1	7	9.44E+03	0.423	POST TRANSLATIONAL PROTEIN MODIFICATION
EEF1AKMT1	5	2.07E+03	0.423	POST TRANSLATIONAL PROTEIN MODIFICATION
SHPRH	14	8.64E+03	0.423	POST TRANSLATIONAL PROTEIN MODIFICATION
COMMD4	2	5.55E+02	0.424	POST TRANSLATIONAL PROTEIN MODIFICATION
ADAMTS5	4	1.05E+04	0.426	POST TRANSLATIONAL PROTEIN MODIFICATION
DPM1	2	7.93E+02	0.426	POST TRANSLATIONAL PROTEIN MODIFICATION
B3GNT3	5	9.31E+03	0.426	POST TRANSLATIONAL PROTEIN MODIFICATION
KCTD7	1	3.17E+02	0.426	POST TRANSLATIONAL PROTEIN MODIFICATION
LTBP1	23	3.47E+04	0.428	POST TRANSLATIONAL PROTEIN MODIFICATION
JOSD1	2	5.11E+02	0.430	POST TRANSLATIONAL PROTEIN MODIFICATION
TNIP3	3	9.39E+03	0.431	POST TRANSLATIONAL PROTEIN MODIFICATION
PSMB8	4	5.06E+03	0.432	POST TRANSLATIONAL PROTEIN MODIFICATION
B4GALT2	4	2.53E+03	0.433	POST TRANSLATIONAL PROTEIN MODIFICATION
AMTN	3	4.48E+03	0.434	POST TRANSLATIONAL PROTEIN MODIFICATION
ART4	2	1.25E+03	0.435	POST TRANSLATIONAL PROTEIN MODIFICATION
CANX	4	3.11E+03	0.436	POST TRANSLATIONAL PROTEIN MODIFICATION
CHST10	3	1.44E+03	0.437	POST TRANSLATIONAL PROTEIN MODIFICATION
ASB3	10	1.05E+04	0.438	POST TRANSLATIONAL PROTEIN MODIFICATION
PIGH	1	4.28E+02	0.438	POST TRANSLATIONAL PROTEIN MODIFICATION
PCSK9	3	4.36E+03	0.439	POST TRANSLATIONAL PROTEIN MODIFICATION
SENP8	2	9.30E+02	0.439	POST TRANSLATIONAL PROTEIN MODIFICATION
RNF123	10	1.09E+04	0.440	POST TRANSLATIONAL PROTEIN MODIFICATION
NUP50	2	8.84E+02	0.441	POST TRANSLATIONAL PROTEIN MODIFICATION
RAB17	5	5.78E+03	0.441	POST TRANSLATIONAL PROTEIN MODIFICATION
GPLD1	14	1.87E+04	0.444	POST TRANSLATIONAL PROTEIN MODIFICATION
MANEA	5	2.17E+03	0.445	POST TRANSLATIONAL PROTEIN MODIFICATION
DOLPP1	1	1.69E+02	0.446	POST TRANSLATIONAL PROTEIN MODIFICATION
PSMA6	1	3.60E+02	0.447	POST TRANSLATIONAL PROTEIN MODIFICATION
ANK3	34	3.47E+04	0.447	POST TRANSLATIONAL PROTEIN MODIFICATION
ALPL	4	5.66E+03	0.447	POST TRANSLATIONAL PROTEIN MODIFICATION
IZUMO1R	2	2.99E+03	0.448	POST TRANSLATIONAL PROTEIN MODIFICATION
PENK	4	1.99E+03	0.448	POST TRANSLATIONAL PROTEIN MODIFICATION
ST6GALNAC2	4	2.46E+03	0.449	POST TRANSLATIONAL PROTEIN MODIFICATION
MBD6	7	2.86E+03	0.449	POST TRANSLATIONAL PROTEIN MODIFICATION
SERPINC1	4	3.08E+03	0.449	POST TRANSLATIONAL PROTEIN MODIFICATION
CBX2	11	8.68E+03	0.451	POST TRANSLATIONAL PROTEIN MODIFICATION
GALNT11	12	1.32E+04	0.452	POST TRANSLATIONAL PROTEIN MODIFICATION
SNX3	1	1.36E+02	0.452	POST TRANSLATIONAL PROTEIN MODIFICATION
FBXL5	3	7.38E+02	0.452	POST TRANSLATIONAL PROTEIN MODIFICATION
FBN1	12	6.39E+03	0.452	POST TRANSLATIONAL PROTEIN MODIFICATION
PDIA6	4	5.09E+03	0.453	POST TRANSLATIONAL PROTEIN MODIFICATION
RAD23A	4	9.15E+03	0.454	POST TRANSLATIONAL PROTEIN MODIFICATION
CDH2	4	3.76E+03	0.454	POST TRANSLATIONAL PROTEIN MODIFICATION
NUDT14	6	4.09E+03	0.456	POST TRANSLATIONAL PROTEIN MODIFICATION
UCHL5	5	5.41E+03	0.456	POST TRANSLATIONAL PROTEIN MODIFICATION
ADAMTS16	19	2.24E+04	0.456	POST TRANSLATIONAL PROTEIN MODIFICATION
TTLL12	8	6.07E+03	0.457	POST TRANSLATIONAL PROTEIN MODIFICATION
IGFBP1	5	1.75E+03	0.457	POST TRANSLATIONAL PROTEIN MODIFICATION
SERPIND1	6	7.11E+03	0.457	POST TRANSLATIONAL PROTEIN MODIFICATION
HLA-A	11	1.78E+04	0.458	POST TRANSLATIONAL PROTEIN MODIFICATION
GRIA1	2	1.90E+03	0.459	POST TRANSLATIONAL PROTEIN MODIFICATION
MAN2A2	9	8.13E+03	0.459	POST TRANSLATIONAL PROTEIN MODIFICATION
TNKS2	4	2.13E+03	0.460	POST TRANSLATIONAL PROTEIN MODIFICATION
RNF152	3	5.62E+03	0.460	POST TRANSLATIONAL PROTEIN MODIFICATION
RTF1	1	1.04E+02	0.460	POST TRANSLATIONAL PROTEIN MODIFICATION
B3GNT7	3	1.61E+03	0.460	POST TRANSLATIONAL PROTEIN MODIFICATION
PAF1	5	4.35E+03	0.461	POST TRANSLATIONAL PROTEIN MODIFICATION
PROS1	8	5.86E+03	0.462	POST TRANSLATIONAL PROTEIN MODIFICATION
MSLN	31	2.95E+04	0.462	POST TRANSLATIONAL PROTEIN MODIFICATION
PIGO	6	4.27E+03	0.463	POST TRANSLATIONAL PROTEIN MODIFICATION
ANO8	9	7.82E+03	0.463	POST TRANSLATIONAL PROTEIN MODIFICATION
ASB6	4	1.04E+03	0.464	POST TRANSLATIONAL PROTEIN MODIFICATION
PSMD5	6	6.49E+03	0.464	POST TRANSLATIONAL PROTEIN MODIFICATION
TOP1	2	2.51E+03	0.464	POST TRANSLATIONAL PROTEIN MODIFICATION
ARCN1	1	5.55E+01	0.466	POST TRANSLATIONAL PROTEIN MODIFICATION
POFUT2	9	5.95E+03	0.466	POST TRANSLATIONAL PROTEIN MODIFICATION
EEF1AKMT2	2	8.34E+02	0.466	POST TRANSLATIONAL PROTEIN MODIFICATION
RAB20	2	7.85E+02	0.467	POST TRANSLATIONAL PROTEIN MODIFICATION
GALNT9	3	5.46E+02	0.468	POST TRANSLATIONAL PROTEIN MODIFICATION
STAMBP	1	5.19E+02	0.468	POST TRANSLATIONAL PROTEIN MODIFICATION

DCUN1D3	2	3.84E+02	0.468	POST TRANSLATIONAL PROTEIN MODIFICATION
RNF168	5	3.34E+03	0.469	POST TRANSLATIONAL PROTEIN MODIFICATION
TFPT	2	3.43E+03	0.469	POST TRANSLATIONAL PROTEIN MODIFICATION
IDE	3	1.10E+03	0.469	POST TRANSLATIONAL PROTEIN MODIFICATION
SP3	3	2.56E+03	0.470	POST TRANSLATIONAL PROTEIN MODIFICATION
AURKB	5	1.98E+03	0.470	POST TRANSLATIONAL PROTEIN MODIFICATION
SCMH1	7	5.69E+03	0.471	POST TRANSLATIONAL PROTEIN MODIFICATION
MUL1	4	5.01E+03	0.474	POST TRANSLATIONAL PROTEIN MODIFICATION
B3GALNT2	5	2.84E+03	0.476	POST TRANSLATIONAL PROTEIN MODIFICATION
ESR1	11	1.34E+04	0.476	POST TRANSLATIONAL PROTEIN MODIFICATION
TUBB4A	1	4.61E+02	0.476	POST TRANSLATIONAL PROTEIN MODIFICATION
FAM20C	6	6.76E+03	0.479	POST TRANSLATIONAL PROTEIN MODIFICATION
UGGT1	7	3.83E+03	0.479	POST TRANSLATIONAL PROTEIN MODIFICATION
RAD23B	1	2.06E+02	0.481	POST TRANSLATIONAL PROTEIN MODIFICATION
RAB36	3	1.70E+03	0.481	POST TRANSLATIONAL PROTEIN MODIFICATION
CP	9	8.01E+03	0.483	POST TRANSLATIONAL PROTEIN MODIFICATION
ALG10	3	1.24E+03	0.483	POST TRANSLATIONAL PROTEIN MODIFICATION
GPIHBP1	5	1.86E+03	0.483	POST TRANSLATIONAL PROTEIN MODIFICATION
RAB33B	1	7.39E+02	0.483	POST TRANSLATIONAL PROTEIN MODIFICATION
RAD21	1	6.46E+01	0.486	POST TRANSLATIONAL PROTEIN MODIFICATION
ADAMTSL4	21	2.60E+04	0.487	POST TRANSLATIONAL PROTEIN MODIFICATION
ACTR8	3	3.64E+03	0.487	POST TRANSLATIONAL PROTEIN MODIFICATION
NFRKB	6	5.50E+03	0.487	POST TRANSLATIONAL PROTEIN MODIFICATION
TULP4	19	1.30E+04	0.488	POST TRANSLATIONAL PROTEIN MODIFICATION
B3GNT5	2	2.57E+03	0.491	POST TRANSLATIONAL PROTEIN MODIFICATION
NAPG	3	1.57E+03	0.491	POST TRANSLATIONAL PROTEIN MODIFICATION
PSMD7	3	1.22E+03	0.491	POST TRANSLATIONAL PROTEIN MODIFICATION
NUP85	5	4.02E+03	0.492	POST TRANSLATIONAL PROTEIN MODIFICATION
MYC	3	2.83E+03	0.492	POST TRANSLATIONAL PROTEIN MODIFICATION
MOGS	10	1.44E+04	0.494	POST TRANSLATIONAL PROTEIN MODIFICATION
TRIM25	8	3.12E+03	0.494	POST TRANSLATIONAL PROTEIN MODIFICATION
ZNF131	3	1.68E+03	0.496	POST TRANSLATIONAL PROTEIN MODIFICATION
FBXL14	4	3.54E+03	0.498	POST TRANSLATIONAL PROTEIN MODIFICATION
MITF	3	1.49E+03	0.498	POST TRANSLATIONAL PROTEIN MODIFICATION
RAET1G	5	7.11E+03	0.499	POST TRANSLATIONAL PROTEIN MODIFICATION
P4HB	2	5.14E+02	0.499	POST TRANSLATIONAL PROTEIN MODIFICATION
GAS6	9	3.88E+03	0.501	POST TRANSLATIONAL PROTEIN MODIFICATION
HSPA8	1	7.27E+01	0.503	POST TRANSLATIONAL PROTEIN MODIFICATION
SCFD1	4	4.76E+03	0.504	POST TRANSLATIONAL PROTEIN MODIFICATION
CKAP4	7	5.12E+03	0.505	POST TRANSLATIONAL PROTEIN MODIFICATION
RWDD3	3	2.73E+03	0.506	POST TRANSLATIONAL PROTEIN MODIFICATION
ADAMTS12	13	9.22E+03	0.506	POST TRANSLATIONAL PROTEIN MODIFICATION
MPDU1	2	6.44E+02	0.506	POST TRANSLATIONAL PROTEIN MODIFICATION
CAPZA2	2	4.45E+02	0.506	POST TRANSLATIONAL PROTEIN MODIFICATION
ETFB	4	3.83E+03	0.507	POST TRANSLATIONAL PROTEIN MODIFICATION
PTEN	2	4.47E+02	0.508	POST TRANSLATIONAL PROTEIN MODIFICATION
SPRN	2	8.51E+02	0.508	POST TRANSLATIONAL PROTEIN MODIFICATION
WDR20	5	5.93E+03	0.511	POST TRANSLATIONAL PROTEIN MODIFICATION
MIA3	19	1.60E+04	0.512	POST TRANSLATIONAL PROTEIN MODIFICATION
PSMD8	3	5.62E+02	0.512	POST TRANSLATIONAL PROTEIN MODIFICATION
FBXW10	13	1.84E+04	0.513	POST TRANSLATIONAL PROTEIN MODIFICATION
PPARA	4	2.19E+03	0.513	POST TRANSLATIONAL PROTEIN MODIFICATION
MAN2A1	6	1.54E+03	0.513	POST TRANSLATIONAL PROTEIN MODIFICATION
DCAF11	3	2.59E+03	0.514	POST TRANSLATIONAL PROTEIN MODIFICATION
FGA	7	1.83E+03	0.515	POST TRANSLATIONAL PROTEIN MODIFICATION
GNE	3	8.75E+02	0.516	POST TRANSLATIONAL PROTEIN MODIFICATION
RECK	8	6.71E+03	0.518	POST TRANSLATIONAL PROTEIN MODIFICATION
RAB18	1	1.03E+02	0.518	POST TRANSLATIONAL PROTEIN MODIFICATION
STAM	4	9.79E+02	0.518	POST TRANSLATIONAL PROTEIN MODIFICATION
MDGA1	11	3.71E+03	0.519	POST TRANSLATIONAL PROTEIN MODIFICATION
MGAT4A	5	3.88E+03	0.520	POST TRANSLATIONAL PROTEIN MODIFICATION
RAB1A	1	1.38E+03	0.521	POST TRANSLATIONAL PROTEIN MODIFICATION
ARSA	4	1.13E+03	0.522	POST TRANSLATIONAL PROTEIN MODIFICATION
PSMB10	3	7.89E+02	0.522	POST TRANSLATIONAL PROTEIN MODIFICATION
TGOLN2	16	2.38E+04	0.522	POST TRANSLATIONAL PROTEIN MODIFICATION
CDC20	1	9.20E+02	0.522	POST TRANSLATIONAL PROTEIN MODIFICATION
RAB31	1	1.86E+02	0.523	POST TRANSLATIONAL PROTEIN MODIFICATION
LMAN1L	6	2.86E+03	0.524	POST TRANSLATIONAL PROTEIN MODIFICATION
THSD7B	12	8.23E+03	0.525	POST TRANSLATIONAL PROTEIN MODIFICATION
UBA3	1	7.33E+01	0.526	POST TRANSLATIONAL PROTEIN MODIFICATION
COMMD3	2	3.24E+03	0.526	POST TRANSLATIONAL PROTEIN MODIFICATION
TRAPPC10	7	3.67E+03	0.527	POST TRANSLATIONAL PROTEIN MODIFICATION
RAB6A	1	1.01E+03	0.529	POST TRANSLATIONAL PROTEIN MODIFICATION
ALB	4	1.14E+03	0.533	POST TRANSLATIONAL PROTEIN MODIFICATION

PRSS23	11	1.17E+04	0.533	POST TRANSLATIONAL PROTEIN MODIFICATION
PTRH2	1	8.90E+02	0.534	POST TRANSLATIONAL PROTEIN MODIFICATION
STC2	2	8.54E+02	0.536	POST TRANSLATIONAL PROTEIN MODIFICATION
B3GNTL1	5	3.51E+03	0.536	POST TRANSLATIONAL PROTEIN MODIFICATION
RAB11B	1	9.50E+01	0.538	POST TRANSLATIONAL PROTEIN MODIFICATION
FBXO21	1	7.50E+01	0.539	POST TRANSLATIONAL PROTEIN MODIFICATION
OTULIN	4	2.78E+03	0.540	POST TRANSLATIONAL PROTEIN MODIFICATION
ART3	4	2.28E+03	0.540	POST TRANSLATIONAL PROTEIN MODIFICATION
ADAMTS10	15	9.74E+03	0.540	POST TRANSLATIONAL PROTEIN MODIFICATION
KLHL21	2	2.49E+03	0.542	POST TRANSLATIONAL PROTEIN MODIFICATION
CCNA2	2	2.97E+03	0.543	POST TRANSLATIONAL PROTEIN MODIFICATION
KTN1	17	7.60E+03	0.543	POST TRANSLATIONAL PROTEIN MODIFICATION
PIGC	5	2.98E+03	0.543	POST TRANSLATIONAL PROTEIN MODIFICATION
RANGAP1	3	8.17E+02	0.546	POST TRANSLATIONAL PROTEIN MODIFICATION
RAB7B	2	1.04E+03	0.546	POST TRANSLATIONAL PROTEIN MODIFICATION
MELTF	15	2.00E+04	0.546	POST TRANSLATIONAL PROTEIN MODIFICATION
GALNT4	7	5.31E+03	0.546	POST TRANSLATIONAL PROTEIN MODIFICATION
NUB1	6	3.67E+03	0.548	POST TRANSLATIONAL PROTEIN MODIFICATION
CCNA1	4	7.94E+02	0.548	POST TRANSLATIONAL PROTEIN MODIFICATION
PSMD9	3	9.67E+02	0.549	POST TRANSLATIONAL PROTEIN MODIFICATION
RAD52	3	1.14E+03	0.550	POST TRANSLATIONAL PROTEIN MODIFICATION
RAD18	5	9.03E+02	0.551	POST TRANSLATIONAL PROTEIN MODIFICATION
DPH1	9	1.11E+04	0.551	POST TRANSLATIONAL PROTEIN MODIFICATION
CLSPN	8	7.17E+03	0.552	POST TRANSLATIONAL PROTEIN MODIFICATION
GALNT3	3	2.64E+03	0.554	POST TRANSLATIONAL PROTEIN MODIFICATION
SPTBN1	13	8.86E+03	0.555	POST TRANSLATIONAL PROTEIN MODIFICATION
APOA2	1	1.53E+02	0.557	POST TRANSLATIONAL PROTEIN MODIFICATION
MBTPS1	14	1.18E+04	0.560	POST TRANSLATIONAL PROTEIN MODIFICATION
NFKBIA	1	7.31E+01	0.561	POST TRANSLATIONAL PROTEIN MODIFICATION
POMK	6	1.15E+03	0.561	POST TRANSLATIONAL PROTEIN MODIFICATION
LRRC49	10	4.54E+03	0.562	POST TRANSLATIONAL PROTEIN MODIFICATION
AREG	1	1.33E+03	0.563	POST TRANSLATIONAL PROTEIN MODIFICATION
USP19	10	4.29E+03	0.564	POST TRANSLATIONAL PROTEIN MODIFICATION
PMM2	7	4.86E+03	0.567	POST TRANSLATIONAL PROTEIN MODIFICATION
ST6GALNAC5	2	1.51E+03	0.569	POST TRANSLATIONAL PROTEIN MODIFICATION
PROZ	6	3.76E+03	0.569	POST TRANSLATIONAL PROTEIN MODIFICATION
ALG2	4	2.48E+03	0.571	POST TRANSLATIONAL PROTEIN MODIFICATION
ARFGAP3	8	1.41E+04	0.571	POST TRANSLATIONAL PROTEIN MODIFICATION
GCNT7	1	8.23E+01	0.572	POST TRANSLATIONAL PROTEIN MODIFICATION
SIN3A	1	3.38E+02	0.572	POST TRANSLATIONAL PROTEIN MODIFICATION
TBC1D20	2	1.78E+03	0.573	POST TRANSLATIONAL PROTEIN MODIFICATION
HIST1H2AB	1	6.33E+01	0.573	POST TRANSLATIONAL PROTEIN MODIFICATION
NUP98	15	7.18E+03	0.574	POST TRANSLATIONAL PROTEIN MODIFICATION
WAC	1	8.14E+01	0.574	POST TRANSLATIONAL PROTEIN MODIFICATION
PEX10	3	1.43E+03	0.575	POST TRANSLATIONAL PROTEIN MODIFICATION
TOP2A	10	9.53E+03	0.575	POST TRANSLATIONAL PROTEIN MODIFICATION
VHL	4	3.46E+03	0.576	POST TRANSLATIONAL PROTEIN MODIFICATION
PRKCSH	12	7.49E+03	0.576	POST TRANSLATIONAL PROTEIN MODIFICATION
MUC1	19	1.78E+04	0.577	POST TRANSLATIONAL PROTEIN MODIFICATION
PSMC3	1	3.75E+01	0.578	POST TRANSLATIONAL PROTEIN MODIFICATION
SAFB	3	1.29E+03	0.579	POST TRANSLATIONAL PROTEIN MODIFICATION
IGFBP4	2	2.32E+02	0.579	POST TRANSLATIONAL PROTEIN MODIFICATION
TMEM132A	11	1.13E+04	0.580	POST TRANSLATIONAL PROTEIN MODIFICATION
WDR61	1	1.01E+02	0.581	POST TRANSLATIONAL PROTEIN MODIFICATION
KBTBD13	6	5.87E+03	0.582	POST TRANSLATIONAL PROTEIN MODIFICATION
SERPINA10	7	6.11E+03	0.583	POST TRANSLATIONAL PROTEIN MODIFICATION
AGBL3	14	1.63E+04	0.583	POST TRANSLATIONAL PROTEIN MODIFICATION
DOHH	3	1.27E+03	0.584	POST TRANSLATIONAL PROTEIN MODIFICATION
MUC12	169	2.73E+05	0.584	POST TRANSLATIONAL PROTEIN MODIFICATION
NRN1L	3	7.04E+02	0.584	POST TRANSLATIONAL PROTEIN MODIFICATION
USP8	3	3.01E+03	0.588	POST TRANSLATIONAL PROTEIN MODIFICATION
TECTB	3	4.03E+03	0.589	POST TRANSLATIONAL PROTEIN MODIFICATION
MAN1A1	3	2.12E+03	0.590	POST TRANSLATIONAL PROTEIN MODIFICATION
FN3KRP	4	4.99E+03	0.590	POST TRANSLATIONAL PROTEIN MODIFICATION
FSTL3	2	2.46E+03	0.593	POST TRANSLATIONAL PROTEIN MODIFICATION
EEF2KMT	8	4.38E+03	0.594	POST TRANSLATIONAL PROTEIN MODIFICATION
TRIM27	1	9.29E+01	0.594	POST TRANSLATIONAL PROTEIN MODIFICATION
USP28	13	8.39E+03	0.594	POST TRANSLATIONAL PROTEIN MODIFICATION
B4GALNT2	5	6.61E+03	0.595	POST TRANSLATIONAL PROTEIN MODIFICATION
KLHL22	2	3.29E+02	0.595	POST TRANSLATIONAL PROTEIN MODIFICATION
TRAPPC1	1	1.99E+01	0.595	POST TRANSLATIONAL PROTEIN MODIFICATION
USO1	7	1.69E+03	0.595	POST TRANSLATIONAL PROTEIN MODIFICATION
CNTN5	14	1.02E+04	0.596	POST TRANSLATIONAL PROTEIN MODIFICATION
B4GALT6	1	7.97E+01	0.597	POST TRANSLATIONAL PROTEIN MODIFICATION

PSMD11	1	2.60E+01	0.598	POST TRANSLATIONAL PROTEIN MODIFICATION
SUMF2	10	1.24E+04	0.598	POST TRANSLATIONAL PROTEIN MODIFICATION
PRSS41	13	1.07E+04	0.600	POST TRANSLATIONAL PROTEIN MODIFICATION
HSP90B1	4	3.90E+03	0.601	POST TRANSLATIONAL PROTEIN MODIFICATION
STAM2	1	3.34E+02	0.601	POST TRANSLATIONAL PROTEIN MODIFICATION
AMFR	2	4.94E+02	0.603	POST TRANSLATIONAL PROTEIN MODIFICATION
OTUD7B	7	1.56E+03	0.603	POST TRANSLATIONAL PROTEIN MODIFICATION
SEL1L	9	8.63E+03	0.603	POST TRANSLATIONAL PROTEIN MODIFICATION
ARSB	4	2.85E+03	0.604	POST TRANSLATIONAL PROTEIN MODIFICATION
TP53BP1	14	1.24E+04	0.604	POST TRANSLATIONAL PROTEIN MODIFICATION
IGFBP3	3	3.62E+03	0.604	POST TRANSLATIONAL PROTEIN MODIFICATION
USP16	5	1.34E+03	0.606	POST TRANSLATIONAL PROTEIN MODIFICATION
CISH	4	1.26E+03	0.608	POST TRANSLATIONAL PROTEIN MODIFICATION
GALNT14	11	1.23E+04	0.609	POST TRANSLATIONAL PROTEIN MODIFICATION
SEMA5B	4	4.01E+03	0.610	POST TRANSLATIONAL PROTEIN MODIFICATION
VCPKMT	4	1.80E+03	0.610	POST TRANSLATIONAL PROTEIN MODIFICATION
ANK2	31	1.51E+04	0.612	POST TRANSLATIONAL PROTEIN MODIFICATION
EP300	8	5.82E+03	0.612	POST TRANSLATIONAL PROTEIN MODIFICATION
FBXL13	15	7.38E+03	0.613	POST TRANSLATIONAL PROTEIN MODIFICATION
TNFAIP3	10	1.95E+03	0.614	POST TRANSLATIONAL PROTEIN MODIFICATION
GALNT17	4	1.13E+03	0.615	POST TRANSLATIONAL PROTEIN MODIFICATION
COG2	6	3.53E+03	0.615	POST TRANSLATIONAL PROTEIN MODIFICATION
ARRB2	3	1.62E+03	0.616	POST TRANSLATIONAL PROTEIN MODIFICATION
PIGP	4	4.68E+02	0.618	POST TRANSLATIONAL PROTEIN MODIFICATION
NUP188	17	9.52E+03	0.618	POST TRANSLATIONAL PROTEIN MODIFICATION
IL33	2	2.63E+02	0.620	POST TRANSLATIONAL PROTEIN MODIFICATION
ADAMTS18	22	1.73E+04	0.620	POST TRANSLATIONAL PROTEIN MODIFICATION
UFD1	2	1.60E+03	0.622	POST TRANSLATIONAL PROTEIN MODIFICATION
COPZ2	2	7.01E+02	0.623	POST TRANSLATIONAL PROTEIN MODIFICATION
SP100	4	2.36E+03	0.624	POST TRANSLATIONAL PROTEIN MODIFICATION
HDAC2	1	9.10E+01	0.625	POST TRANSLATIONAL PROTEIN MODIFICATION
GOSR2	3	2.58E+03	0.625	POST TRANSLATIONAL PROTEIN MODIFICATION
BCL10	1	7.88E+01	0.625	POST TRANSLATIONAL PROTEIN MODIFICATION
NR1I2	3	2.07E+03	0.627	POST TRANSLATIONAL PROTEIN MODIFICATION
LMAN2	3	1.56E+03	0.627	POST TRANSLATIONAL PROTEIN MODIFICATION
FUT8	4	2.19E+03	0.627	POST TRANSLATIONAL PROTEIN MODIFICATION
ALG1	9	3.80E+03	0.628	POST TRANSLATIONAL PROTEIN MODIFICATION
NUP43	2	2.59E+02	0.628	POST TRANSLATIONAL PROTEIN MODIFICATION
CHST8	6	2.75E+03	0.628	POST TRANSLATIONAL PROTEIN MODIFICATION
OS9	7	7.48E+02	0.629	POST TRANSLATIONAL PROTEIN MODIFICATION
PSMB3	1	5.09E+01	0.629	POST TRANSLATIONAL PROTEIN MODIFICATION
SPSB3	4	3.38E+03	0.629	POST TRANSLATIONAL PROTEIN MODIFICATION
VDAC2	1	4.55E+01	0.632	POST TRANSLATIONAL PROTEIN MODIFICATION
RAET1L	7	3.94E+03	0.633	POST TRANSLATIONAL PROTEIN MODIFICATION
UBE2F	2	3.64E+02	0.636	POST TRANSLATIONAL PROTEIN MODIFICATION
CALR	5	1.10E+03	0.637	POST TRANSLATIONAL PROTEIN MODIFICATION
DNAJC24	1	4.26E+02	0.638	POST TRANSLATIONAL PROTEIN MODIFICATION
TUBA3D	1	6.84E+01	0.638	POST TRANSLATIONAL PROTEIN MODIFICATION
F7	4	5.85E+02	0.639	POST TRANSLATIONAL PROTEIN MODIFICATION
CTSC	5	8.46E+02	0.639	POST TRANSLATIONAL PROTEIN MODIFICATION
COPE	5	1.64E+03	0.639	POST TRANSLATIONAL PROTEIN MODIFICATION
UBE2Q2	3	1.37E+03	0.640	POST TRANSLATIONAL PROTEIN MODIFICATION
SEC23A	3	5.92E+02	0.640	POST TRANSLATIONAL PROTEIN MODIFICATION
PIGN	11	8.87E+03	0.641	POST TRANSLATIONAL PROTEIN MODIFICATION
DAD1	1	1.27E+02	0.641	POST TRANSLATIONAL PROTEIN MODIFICATION
TTLL4	14	6.59E+03	0.642	POST TRANSLATIONAL PROTEIN MODIFICATION
DCAF6	5	2.21E+03	0.642	POST TRANSLATIONAL PROTEIN MODIFICATION
GOLGA2	13	5.05E+03	0.643	POST TRANSLATIONAL PROTEIN MODIFICATION
ARSI	5	8.40E+02	0.645	POST TRANSLATIONAL PROTEIN MODIFICATION
EDEM3	6	1.02E+04	0.645	POST TRANSLATIONAL PROTEIN MODIFICATION
CUL4A	5	2.87E+03	0.645	POST TRANSLATIONAL PROTEIN MODIFICATION
COMMD9	3	5.13E+02	0.646	POST TRANSLATIONAL PROTEIN MODIFICATION
AURKA	1	8.14E+02	0.647	POST TRANSLATIONAL PROTEIN MODIFICATION
PSME1	2	1.21E+03	0.649	POST TRANSLATIONAL PROTEIN MODIFICATION
GALNT10	3	4.01E+02	0.650	POST TRANSLATIONAL PROTEIN MODIFICATION
RNF40	6	2.89E+03	0.651	POST TRANSLATIONAL PROTEIN MODIFICATION
CDC73	2	4.90E+02	0.651	POST TRANSLATIONAL PROTEIN MODIFICATION
GGCX	7	1.01E+03	0.651	POST TRANSLATIONAL PROTEIN MODIFICATION
CHML	6	3.16E+03	0.652	POST TRANSLATIONAL PROTEIN MODIFICATION
GMPPA	7	2.55E+03	0.652	POST TRANSLATIONAL PROTEIN MODIFICATION
TUBB6	5	2.34E+03	0.653	POST TRANSLATIONAL PROTEIN MODIFICATION
PIGW	4	2.30E+03	0.653	POST TRANSLATIONAL PROTEIN MODIFICATION
B4GALT3	3	1.10E+03	0.655	POST TRANSLATIONAL PROTEIN MODIFICATION
BTBD1	6	1.74E+03	0.658	POST TRANSLATIONAL PROTEIN MODIFICATION

SEC24C	11	7.28E+03	0.658	POST TRANSLATIONAL PROTEIN MODIFICATION
NICN1	2	1.26E+03	0.659	POST TRANSLATIONAL PROTEIN MODIFICATION
NTNG2	2	2.70E+03	0.659	POST TRANSLATIONAL PROTEIN MODIFICATION
FEM1A	4	1.23E+03	0.659	POST TRANSLATIONAL PROTEIN MODIFICATION
FBXL20	1	2.57E+02	0.660	POST TRANSLATIONAL PROTEIN MODIFICATION
UIMC1	11	4.79E+03	0.660	POST TRANSLATIONAL PROTEIN MODIFICATION
RNF144A	2	6.72E+02	0.661	POST TRANSLATIONAL PROTEIN MODIFICATION
USP15	2	3.04E+02	0.661	POST TRANSLATIONAL PROTEIN MODIFICATION
FUCA2	3	1.56E+03	0.661	POST TRANSLATIONAL PROTEIN MODIFICATION
GCNT3	5	3.09E+03	0.661	POST TRANSLATIONAL PROTEIN MODIFICATION
KDM1B	4	3.49E+03	0.661	POST TRANSLATIONAL PROTEIN MODIFICATION
MEN1	2	2.04E+03	0.662	POST TRANSLATIONAL PROTEIN MODIFICATION
POMT1	8	5.01E+03	0.663	POST TRANSLATIONAL PROTEIN MODIFICATION
BABAM2	6	2.78E+03	0.664	POST TRANSLATIONAL PROTEIN MODIFICATION
HIST1H2AD	2	1.86E+02	0.665	POST TRANSLATIONAL PROTEIN MODIFICATION
NEU4	11	7.75E+03	0.668	POST TRANSLATIONAL PROTEIN MODIFICATION
MAT2B	2	2.58E+02	0.669	POST TRANSLATIONAL PROTEIN MODIFICATION
GALNT18	13	7.30E+03	0.669	POST TRANSLATIONAL PROTEIN MODIFICATION
NRIP1	18	1.11E+04	0.670	POST TRANSLATIONAL PROTEIN MODIFICATION
CAPZA3	9	9.04E+03	0.673	POST TRANSLATIONAL PROTEIN MODIFICATION
TTLL8	13	7.16E+03	0.673	POST TRANSLATIONAL PROTEIN MODIFICATION
PSMD1	6	1.26E+03	0.674	POST TRANSLATIONAL PROTEIN MODIFICATION
RAB34	4	3.90E+03	0.674	POST TRANSLATIONAL PROTEIN MODIFICATION
ASXL2	15	6.19E+03	0.676	POST TRANSLATIONAL PROTEIN MODIFICATION
ST8SIA2	1	3.24E+01	0.676	POST TRANSLATIONAL PROTEIN MODIFICATION
DCTN2	3	2.90E+02	0.676	POST TRANSLATIONAL PROTEIN MODIFICATION
NAPA	2	4.76E+02	0.681	POST TRANSLATIONAL PROTEIN MODIFICATION
SPON1	4	1.24E+03	0.682	POST TRANSLATIONAL PROTEIN MODIFICATION
NSMCE1	1	5.50E+02	0.683	POST TRANSLATIONAL PROTEIN MODIFICATION
MLEC	1	3.56E+01	0.683	POST TRANSLATIONAL PROTEIN MODIFICATION
KBTBD8	2	4.03E+02	0.685	POST TRANSLATIONAL PROTEIN MODIFICATION
OTOA	9	9.23E+03	0.687	POST TRANSLATIONAL PROTEIN MODIFICATION
CUL2	2	1.01E+03	0.687	POST TRANSLATIONAL PROTEIN MODIFICATION
ALG5	1	1.19E+02	0.688	POST TRANSLATIONAL PROTEIN MODIFICATION
NR5A1	3	1.40E+03	0.688	POST TRANSLATIONAL PROTEIN MODIFICATION
BMP4	3	3.64E+02	0.691	POST TRANSLATIONAL PROTEIN MODIFICATION
NUP214	20	6.60E+03	0.691	POST TRANSLATIONAL PROTEIN MODIFICATION
TRAPPC9	11	4.40E+03	0.691	POST TRANSLATIONAL PROTEIN MODIFICATION
DCAF13	4	1.28E+03	0.692	POST TRANSLATIONAL PROTEIN MODIFICATION
C3	10	2.84E+03	0.694	POST TRANSLATIONAL PROTEIN MODIFICATION
ST3GAL5	3	1.80E+03	0.694	POST TRANSLATIONAL PROTEIN MODIFICATION
MAP3K7	2	2.71E+02	0.694	POST TRANSLATIONAL PROTEIN MODIFICATION
KBTBD7	2	4.72E+02	0.696	POST TRANSLATIONAL PROTEIN MODIFICATION
NGLY1	5	6.36E+02	0.697	POST TRANSLATIONAL PROTEIN MODIFICATION
BPIFB2	9	2.92E+03	0.697	POST TRANSLATIONAL PROTEIN MODIFICATION
SEC31A	6	2.22E+03	0.697	POST TRANSLATIONAL PROTEIN MODIFICATION
TPGS2	6	4.01E+03	0.699	POST TRANSLATIONAL PROTEIN MODIFICATION
TDG	3	2.38E+03	0.702	POST TRANSLATIONAL PROTEIN MODIFICATION
B3GNT4	4	1.39E+03	0.704	POST TRANSLATIONAL PROTEIN MODIFICATION
USP48	6	3.05E+03	0.704	POST TRANSLATIONAL PROTEIN MODIFICATION
CAPZA1	4	2.33E+03	0.706	POST TRANSLATIONAL PROTEIN MODIFICATION
PRSS21	4	1.25E+03	0.707	POST TRANSLATIONAL PROTEIN MODIFICATION
MFGE8	7	1.42E+03	0.707	POST TRANSLATIONAL PROTEIN MODIFICATION
TRIM28	5	1.51E+03	0.707	POST TRANSLATIONAL PROTEIN MODIFICATION
USP17L11	7	6.47E+03	0.708	POST TRANSLATIONAL PROTEIN MODIFICATION
USP17L18	7	6.47E+03	0.708	POST TRANSLATIONAL PROTEIN MODIFICATION
NSMCE3	7	4.64E+03	0.711	POST TRANSLATIONAL PROTEIN MODIFICATION
UBE2S	3	1.73E+03	0.712	POST TRANSLATIONAL PROTEIN MODIFICATION
INO80E	7	2.37E+03	0.716	POST TRANSLATIONAL PROTEIN MODIFICATION
TRIM13	1	4.83E+02	0.716	POST TRANSLATIONAL PROTEIN MODIFICATION
TMEM129	2	9.74E+02	0.717	POST TRANSLATIONAL PROTEIN MODIFICATION
CTSA	3	6.21E+02	0.717	POST TRANSLATIONAL PROTEIN MODIFICATION
RAB29	2	1.53E+03	0.718	POST TRANSLATIONAL PROTEIN MODIFICATION
FBXO40	13	4.54E+03	0.719	POST TRANSLATIONAL PROTEIN MODIFICATION
B3GNT9	2	3.86E+02	0.719	POST TRANSLATIONAL PROTEIN MODIFICATION
MGAT4C	3	1.21E+03	0.721	POST TRANSLATIONAL PROTEIN MODIFICATION
RNF103	9	4.69E+03	0.722	POST TRANSLATIONAL PROTEIN MODIFICATION
USP33	2	3.48E+02	0.723	POST TRANSLATIONAL PROTEIN MODIFICATION
ADAMTS15	9	3.59E+03	0.723	POST TRANSLATIONAL PROTEIN MODIFICATION
UBD	1	3.97E+02	0.723	POST TRANSLATIONAL PROTEIN MODIFICATION
PSME4	9	2.12E+03	0.724	POST TRANSLATIONAL PROTEIN MODIFICATION
SPSB1	1	2.18E+01	0.725	POST TRANSLATIONAL PROTEIN MODIFICATION
NEU3	6	2.67E+03	0.726	POST TRANSLATIONAL PROTEIN MODIFICATION
COMMD5	2	8.29E+02	0.727	POST TRANSLATIONAL PROTEIN MODIFICATION

NR5A2	1	6.90E+01	0.728	POST TRANSLATIONAL PROTEIN MODIFICATION
PROC	3	1.56E+03	0.729	POST TRANSLATIONAL PROTEIN MODIFICATION
UGGT2	18	6.41E+03	0.731	POST TRANSLATIONAL PROTEIN MODIFICATION
LYPD4	3	1.12E+03	0.731	POST TRANSLATIONAL PROTEIN MODIFICATION
ASB14	7	9.46E+02	0.732	POST TRANSLATIONAL PROTEIN MODIFICATION
ALG9	6	1.10E+03	0.734	POST TRANSLATIONAL PROTEIN MODIFICATION
ALG3	4	1.36E+03	0.734	POST TRANSLATIONAL PROTEIN MODIFICATION
INO80C	6	1.62E+03	0.735	POST TRANSLATIONAL PROTEIN MODIFICATION
GMPPB	2	1.41E+03	0.737	POST TRANSLATIONAL PROTEIN MODIFICATION
PIGU	5	1.41E+03	0.737	POST TRANSLATIONAL PROTEIN MODIFICATION
SERPINA1	11	4.48E+03	0.737	POST TRANSLATIONAL PROTEIN MODIFICATION
MPI	4	1.30E+03	0.738	POST TRANSLATIONAL PROTEIN MODIFICATION
RPA1	2	1.55E+02	0.740	POST TRANSLATIONAL PROTEIN MODIFICATION
CUL7	14	7.10E+03	0.741	POST TRANSLATIONAL PROTEIN MODIFICATION
PIGQ	10	2.83E+03	0.741	POST TRANSLATIONAL PROTEIN MODIFICATION
USP12	1	2.36E+01	0.741	POST TRANSLATIONAL PROTEIN MODIFICATION
COG1	11	9.35E+03	0.745	POST TRANSLATIONAL PROTEIN MODIFICATION
GALNT15	1	3.22E+01	0.747	POST TRANSLATIONAL PROTEIN MODIFICATION
ADAMTS6	9	3.14E+03	0.748	POST TRANSLATIONAL PROTEIN MODIFICATION
USP47	8	1.83E+03	0.749	POST TRANSLATIONAL PROTEIN MODIFICATION
UMOD	8	2.08E+03	0.749	POST TRANSLATIONAL PROTEIN MODIFICATION
YKT6	3	3.95E+02	0.751	POST TRANSLATIONAL PROTEIN MODIFICATION
GPS1	1	2.00E+01	0.752	POST TRANSLATIONAL PROTEIN MODIFICATION
BAP1	1	9.38E+00	0.753	POST TRANSLATIONAL PROTEIN MODIFICATION
GALNT12	6	2.82E+03	0.753	POST TRANSLATIONAL PROTEIN MODIFICATION
RAB30	1	2.86E+01	0.753	POST TRANSLATIONAL PROTEIN MODIFICATION
NUS1	2	2.10E+02	0.754	POST TRANSLATIONAL PROTEIN MODIFICATION
CCP110	8	1.83E+03	0.755	POST TRANSLATIONAL PROTEIN MODIFICATION
ACTR5	4	5.75E+03	0.755	POST TRANSLATIONAL PROTEIN MODIFICATION
SMAD1	1	2.35E+01	0.756	POST TRANSLATIONAL PROTEIN MODIFICATION
PIGL	4	1.48E+03	0.756	POST TRANSLATIONAL PROTEIN MODIFICATION
MXRA8	6	3.95E+03	0.757	POST TRANSLATIONAL PROTEIN MODIFICATION
NAGK	4	3.71E+02	0.757	POST TRANSLATIONAL PROTEIN MODIFICATION
PSMD6	5	5.04E+02	0.757	POST TRANSLATIONAL PROTEIN MODIFICATION
DCTN1	14	4.29E+03	0.758	POST TRANSLATIONAL PROTEIN MODIFICATION
NUP160	13	4.12E+03	0.759	POST TRANSLATIONAL PROTEIN MODIFICATION
DCAF8	1	1.62E+01	0.759	POST TRANSLATIONAL PROTEIN MODIFICATION
RIPK2	1	3.60E+01	0.760	POST TRANSLATIONAL PROTEIN MODIFICATION
PSMD13	3	1.26E+03	0.761	POST TRANSLATIONAL PROTEIN MODIFICATION
DYNC1H1	7	1.68E+03	0.763	POST TRANSLATIONAL PROTEIN MODIFICATION
TUBA4A	1	2.05E+01	0.764	POST TRANSLATIONAL PROTEIN MODIFICATION
USP14	2	2.22E+02	0.764	POST TRANSLATIONAL PROTEIN MODIFICATION
NUP62	2	6.58E+02	0.764	POST TRANSLATIONAL PROTEIN MODIFICATION
LMAN2L	1	1.50E+01	0.764	POST TRANSLATIONAL PROTEIN MODIFICATION
OTUD3	7	3.13E+03	0.765	POST TRANSLATIONAL PROTEIN MODIFICATION
PARK7	3	8.53E+02	0.765	POST TRANSLATIONAL PROTEIN MODIFICATION
GCNT1	3	1.57E+03	0.765	POST TRANSLATIONAL PROTEIN MODIFICATION
PIGM	3	9.91E+02	0.766	POST TRANSLATIONAL PROTEIN MODIFICATION
USP4	10	1.89E+03	0.768	POST TRANSLATIONAL PROTEIN MODIFICATION
SPTB	27	4.35E+03	0.768	POST TRANSLATIONAL PROTEIN MODIFICATION
MTA1	7	4.70E+03	0.769	POST TRANSLATIONAL PROTEIN MODIFICATION
NR1H4	3	4.52E+02	0.769	POST TRANSLATIONAL PROTEIN MODIFICATION
LAMB2	16	9.89E+03	0.770	POST TRANSLATIONAL PROTEIN MODIFICATION
PREB	1	3.27E+01	0.770	POST TRANSLATIONAL PROTEIN MODIFICATION
BABAM1	2	1.95E+02	0.771	POST TRANSLATIONAL PROTEIN MODIFICATION
RABGGTA	6	1.12E+03	0.771	POST TRANSLATIONAL PROTEIN MODIFICATION
DERL1	1	3.33E+01	0.772	POST TRANSLATIONAL PROTEIN MODIFICATION
SENP2	1	9.02E+00	0.772	POST TRANSLATIONAL PROTEIN MODIFICATION
CHGB	11	8.44E+03	0.773	POST TRANSLATIONAL PROTEIN MODIFICATION
KBTBD6	2	2.27E+02	0.773	POST TRANSLATIONAL PROTEIN MODIFICATION
GFPT2	5	4.66E+02	0.774	POST TRANSLATIONAL PROTEIN MODIFICATION
TTLL2	13	3.57E+03	0.774	POST TRANSLATIONAL PROTEIN MODIFICATION
RANBP2	36	1.23E+04	0.775	POST TRANSLATIONAL PROTEIN MODIFICATION
LMAN1	2	3.58E+02	0.777	POST TRANSLATIONAL PROTEIN MODIFICATION
HIF3A	8	4.06E+03	0.778	POST TRANSLATIONAL PROTEIN MODIFICATION
FOXK1	6	4.98E+03	0.779	POST TRANSLATIONAL PROTEIN MODIFICATION
OTUB2	2	1.09E+02	0.779	POST TRANSLATIONAL PROTEIN MODIFICATION
LHB	3	1.75E+03	0.780	POST TRANSLATIONAL PROTEIN MODIFICATION
VWA1	6	4.46E+03	0.780	POST TRANSLATIONAL PROTEIN MODIFICATION
PIAS4	4	4.08E+02	0.781	POST TRANSLATIONAL PROTEIN MODIFICATION
FBXO22	2	3.78E+02	0.782	POST TRANSLATIONAL PROTEIN MODIFICATION
FBXO31	3	8.98E+02	0.783	POST TRANSLATIONAL PROTEIN MODIFICATION
FN1	25	1.36E+04	0.783	POST TRANSLATIONAL PROTEIN MODIFICATION
ATXN3	3	4.34E+02	0.784	POST TRANSLATIONAL PROTEIN MODIFICATION

MGAT5	5	4.79E+02	0.785	POST TRANSLATIONAL PROTEIN MODIFICATION
BIRC3	3	8.71E+02	0.785	POST TRANSLATIONAL PROTEIN MODIFICATION
SPARCL1	9	6.32E+02	0.786	POST TRANSLATIONAL PROTEIN MODIFICATION
L3MBTL2	3	3.97E+02	0.787	POST TRANSLATIONAL PROTEIN MODIFICATION
GOSR1	1	2.71E+01	0.789	POST TRANSLATIONAL PROTEIN MODIFICATION
GALNT6	5	4.80E+02	0.790	POST TRANSLATIONAL PROTEIN MODIFICATION
NANS	6	2.21E+03	0.791	POST TRANSLATIONAL PROTEIN MODIFICATION
COPS4	1	1.40E+02	0.791	POST TRANSLATIONAL PROTEIN MODIFICATION
ADAMTSL5	14	9.21E+03	0.792	POST TRANSLATIONAL PROTEIN MODIFICATION
HERC2	29	4.79E+03	0.794	POST TRANSLATIONAL PROTEIN MODIFICATION
ST8SIA1	3	5.17E+02	0.794	POST TRANSLATIONAL PROTEIN MODIFICATION
KLHL20	3	2.74E+02	0.794	POST TRANSLATIONAL PROTEIN MODIFICATION
POMGNT2	4	6.30E+02	0.795	POST TRANSLATIONAL PROTEIN MODIFICATION
WFS1	34	6.87E+03	0.796	POST TRANSLATIONAL PROTEIN MODIFICATION
ASB16	9	8.85E+03	0.798	POST TRANSLATIONAL PROTEIN MODIFICATION
STAG1	4	7.47E+02	0.799	POST TRANSLATIONAL PROTEIN MODIFICATION
ZBTB16	1	3.89E+01	0.799	POST TRANSLATIONAL PROTEIN MODIFICATION
COPS7A	1	3.81E+01	0.800	POST TRANSLATIONAL PROTEIN MODIFICATION
COG6	2	2.38E+02	0.802	POST TRANSLATIONAL PROTEIN MODIFICATION
FOLR2	3	1.27E+02	0.803	POST TRANSLATIONAL PROTEIN MODIFICATION
FBXL4	5	5.14E+02	0.803	POST TRANSLATIONAL PROTEIN MODIFICATION
HIST1H2AK	1	8.43E+01	0.804	POST TRANSLATIONAL PROTEIN MODIFICATION
NEGR1	1	1.89E+02	0.804	POST TRANSLATIONAL PROTEIN MODIFICATION
DAXX	2	1.01E+03	0.804	POST TRANSLATIONAL PROTEIN MODIFICATION
OBSL1	20	1.79E+04	0.805	POST TRANSLATIONAL PROTEIN MODIFICATION
TNKS	8	2.95E+03	0.806	POST TRANSLATIONAL PROTEIN MODIFICATION
TRAF3	5	1.43E+03	0.807	POST TRANSLATIONAL PROTEIN MODIFICATION
PEX5	4	2.12E+03	0.808	POST TRANSLATIONAL PROTEIN MODIFICATION
TGFBR1	1	5.15E+01	0.809	POST TRANSLATIONAL PROTEIN MODIFICATION
FBXL16	3	3.71E+02	0.812	POST TRANSLATIONAL PROTEIN MODIFICATION
ASXL1	14	6.35E+03	0.812	POST TRANSLATIONAL PROTEIN MODIFICATION
BIRC5	2	6.58E+02	0.813	POST TRANSLATIONAL PROTEIN MODIFICATION
GCNT4	2	2.00E+02	0.814	POST TRANSLATIONAL PROTEIN MODIFICATION
TUBA3E	6	1.15E+03	0.814	POST TRANSLATIONAL PROTEIN MODIFICATION
RTN4RL2	3	1.29E+03	0.814	POST TRANSLATIONAL PROTEIN MODIFICATION
FBXO41	12	4.92E+03	0.814	POST TRANSLATIONAL PROTEIN MODIFICATION
SEC22C	4	7.97E+02	0.816	POST TRANSLATIONAL PROTEIN MODIFICATION
NUP54	3	1.59E+02	0.817	POST TRANSLATIONAL PROTEIN MODIFICATION
DDOST	5	1.20E+03	0.818	POST TRANSLATIONAL PROTEIN MODIFICATION
RPS2	1	9.05E+00	0.820	POST TRANSLATIONAL PROTEIN MODIFICATION
NUP205	10	5.21E+03	0.821	POST TRANSLATIONAL PROTEIN MODIFICATION
APOE	4	4.35E+02	0.821	POST TRANSLATIONAL PROTEIN MODIFICATION
ENAM	15	3.05E+03	0.822	POST TRANSLATIONAL PROTEIN MODIFICATION
COG3	5	1.20E+03	0.822	POST TRANSLATIONAL PROTEIN MODIFICATION
FBXL15	2	2.19E+02	0.822	POST TRANSLATIONAL PROTEIN MODIFICATION
ADRM1	3	2.02E+03	0.823	POST TRANSLATIONAL PROTEIN MODIFICATION
TUSC3	3	9.12E+02	0.823	POST TRANSLATIONAL PROTEIN MODIFICATION
SUMO3	2	2.22E+03	0.824	POST TRANSLATIONAL PROTEIN MODIFICATION
COL7A1	24	1.24E+04	0.824	POST TRANSLATIONAL PROTEIN MODIFICATION
NFKB2	8	1.88E+03	0.825	POST TRANSLATIONAL PROTEIN MODIFICATION
FGG	5	8.31E+02	0.825	POST TRANSLATIONAL PROTEIN MODIFICATION
RAB5C	3	5.58E+02	0.825	POST TRANSLATIONAL PROTEIN MODIFICATION
FBXO27	3	1.65E+02	0.828	POST TRANSLATIONAL PROTEIN MODIFICATION
KLHL3	2	1.56E+02	0.828	POST TRANSLATIONAL PROTEIN MODIFICATION
HRC	18	5.68E+03	0.828	POST TRANSLATIONAL PROTEIN MODIFICATION
VNN3	2	1.45E+02	0.829	POST TRANSLATIONAL PROTEIN MODIFICATION
TUBAL3	7	6.78E+02	0.831	POST TRANSLATIONAL PROTEIN MODIFICATION
NUP155	8	5.63E+02	0.831	POST TRANSLATIONAL PROTEIN MODIFICATION
NR4A2	1	7.56E+00	0.831	POST TRANSLATIONAL PROTEIN MODIFICATION
YOD1	5	2.67E+02	0.831	POST TRANSLATIONAL PROTEIN MODIFICATION
BLM	11	1.28E+03	0.832	POST TRANSLATIONAL PROTEIN MODIFICATION
NOP58	2	2.79E+02	0.833	POST TRANSLATIONAL PROTEIN MODIFICATION
MGAT2	3	3.43E+02	0.838	POST TRANSLATIONAL PROTEIN MODIFICATION
DTL	2	4.99E+02	0.841	POST TRANSLATIONAL PROTEIN MODIFICATION
SUZ12	5	1.86E+03	0.841	POST TRANSLATIONAL PROTEIN MODIFICATION
UBA6	6	2.01E+03	0.841	POST TRANSLATIONAL PROTEIN MODIFICATION
MAN1A2	2	3.68E+02	0.841	POST TRANSLATIONAL PROTEIN MODIFICATION
RAB26	4	3.30E+02	0.842	POST TRANSLATIONAL PROTEIN MODIFICATION
CEACAM5	9	3.12E+03	0.842	POST TRANSLATIONAL PROTEIN MODIFICATION
RIPK1	3	3.52E+02	0.843	POST TRANSLATIONAL PROTEIN MODIFICATION
SENP1	3	7.92E+02	0.843	POST TRANSLATIONAL PROTEIN MODIFICATION
MDM4	5	4.00E+02	0.843	POST TRANSLATIONAL PROTEIN MODIFICATION
TRAPPC2L	4	3.36E+03	0.843	POST TRANSLATIONAL PROTEIN MODIFICATION
SEMA5A	7	3.01E+03	0.844	POST TRANSLATIONAL PROTEIN MODIFICATION

LEO1	3	6.19E+02	0.844	POST TRANSLATIONAL PROTEIN MODIFICATION
FBXO17	3	1.31E+02	0.844	POST TRANSLATIONAL PROTEIN MODIFICATION
PSME3	1	7.83E+00	0.846	POST TRANSLATIONAL PROTEIN MODIFICATION
ST6GALNAC3	4	1.22E+03	0.846	POST TRANSLATIONAL PROTEIN MODIFICATION
SAE1	1	7.37E+00	0.847	POST TRANSLATIONAL PROTEIN MODIFICATION
PSMD2	2	1.22E+02	0.847	POST TRANSLATIONAL PROTEIN MODIFICATION
PHC2	6	5.22E+02	0.849	POST TRANSLATIONAL PROTEIN MODIFICATION
TADA2B	1	1.13E+01	0.850	POST TRANSLATIONAL PROTEIN MODIFICATION
NUP58	5	3.86E+02	0.850	POST TRANSLATIONAL PROTEIN MODIFICATION
RAB42	3	3.06E+03	0.851	POST TRANSLATIONAL PROTEIN MODIFICATION
CDCA8	1	3.18E+01	0.855	POST TRANSLATIONAL PROTEIN MODIFICATION
TMED3	3	2.98E+02	0.858	POST TRANSLATIONAL PROTEIN MODIFICATION
PIGB	1	5.52E+00	0.858	POST TRANSLATIONAL PROTEIN MODIFICATION
GALNT7	5	3.31E+03	0.858	POST TRANSLATIONAL PROTEIN MODIFICATION
SEC16B	14	3.71E+03	0.860	POST TRANSLATIONAL PROTEIN MODIFICATION
PSMC1	1	4.42E+00	0.860	POST TRANSLATIONAL PROTEIN MODIFICATION
BECN1	1	4.45E+01	0.861	POST TRANSLATIONAL PROTEIN MODIFICATION
NAPB	1	2.77E+01	0.861	POST TRANSLATIONAL PROTEIN MODIFICATION
VDR	2	1.41E+02	0.864	POST TRANSLATIONAL PROTEIN MODIFICATION
RCE1	1	2.82E+01	0.865	POST TRANSLATIONAL PROTEIN MODIFICATION
USP25	8	7.35E+02	0.866	POST TRANSLATIONAL PROTEIN MODIFICATION
DCAF17	8	3.01E+03	0.867	POST TRANSLATIONAL PROTEIN MODIFICATION
FUT3	16	2.16E+03	0.867	POST TRANSLATIONAL PROTEIN MODIFICATION
IKBKE	6	3.17E+03	0.868	POST TRANSLATIONAL PROTEIN MODIFICATION
PGAP1	8	9.41E+02	0.869	POST TRANSLATIONAL PROTEIN MODIFICATION
SPTA1	44	1.36E+04	0.869	POST TRANSLATIONAL PROTEIN MODIFICATION
PIGX	2	5.25E+01	0.872	POST TRANSLATIONAL PROTEIN MODIFICATION
VGF	7	3.65E+02	0.876	POST TRANSLATIONAL PROTEIN MODIFICATION
MVD	10	5.04E+02	0.876	POST TRANSLATIONAL PROTEIN MODIFICATION
APC	32	3.44E+03	0.876	POST TRANSLATIONAL PROTEIN MODIFICATION
PEX14	5	9.51E+02	0.876	POST TRANSLATIONAL PROTEIN MODIFICATION
AMDHD2	10	1.37E+03	0.877	POST TRANSLATIONAL PROTEIN MODIFICATION
ADAMTS8	9	1.66E+03	0.884	POST TRANSLATIONAL PROTEIN MODIFICATION
MUCL1	1	6.17E+00	0.884	POST TRANSLATIONAL PROTEIN MODIFICATION
ARSK	6	1.51E+03	0.887	POST TRANSLATIONAL PROTEIN MODIFICATION
SEC13	1	5.26E+00	0.889	POST TRANSLATIONAL PROTEIN MODIFICATION
HIST2H2BF	5	7.65E+02	0.889	POST TRANSLATIONAL PROTEIN MODIFICATION
ALG10B	10	6.55E+03	0.889	POST TRANSLATIONAL PROTEIN MODIFICATION
COG8	4	5.00E+02	0.891	POST TRANSLATIONAL PROTEIN MODIFICATION
TRRAP	3	2.10E+02	0.891	POST TRANSLATIONAL PROTEIN MODIFICATION
TTLL5	21	1.54E+03	0.891	POST TRANSLATIONAL PROTEIN MODIFICATION
AXIN1	16	8.80E+03	0.891	POST TRANSLATIONAL PROTEIN MODIFICATION
RAB37	13	1.37E+03	0.892	POST TRANSLATIONAL PROTEIN MODIFICATION
FUCA1	4	5.41E+02	0.896	POST TRANSLATIONAL PROTEIN MODIFICATION
FOLR1	1	1.99E+01	0.899	POST TRANSLATIONAL PROTEIN MODIFICATION
WSB1	5	5.21E+02	0.899	POST TRANSLATIONAL PROTEIN MODIFICATION
USP17L2	31	2.48E+03	0.901	POST TRANSLATIONAL PROTEIN MODIFICATION
HIF1A	8	4.69E+03	0.901	POST TRANSLATIONAL PROTEIN MODIFICATION
MUC7	16	8.56E+03	0.902	POST TRANSLATIONAL PROTEIN MODIFICATION
WRN	16	1.89E+03	0.903	POST TRANSLATIONAL PROTEIN MODIFICATION
INCENP	10	2.32E+03	0.904	POST TRANSLATIONAL PROTEIN MODIFICATION
CD55	5	1.70E+02	0.906	POST TRANSLATIONAL PROTEIN MODIFICATION
PSMB2	1	1.34E+01	0.906	POST TRANSLATIONAL PROTEIN MODIFICATION
CNIH3	1	3.48E+00	0.907	POST TRANSLATIONAL PROTEIN MODIFICATION
UCHL3	1	2.00E+00	0.917	POST TRANSLATIONAL PROTEIN MODIFICATION
ANK1	20	1.01E+04	0.917	POST TRANSLATIONAL PROTEIN MODIFICATION
TNC	24	2.05E+04	0.920	POST TRANSLATIONAL PROTEIN MODIFICATION
DCTN3	1	1.52E+00	0.925	POST TRANSLATIONAL PROTEIN MODIFICATION
PIGZ	14	5.61E+02	0.926	POST TRANSLATIONAL PROTEIN MODIFICATION
SPTBN5	57	3.18E+04	0.928	POST TRANSLATIONAL PROTEIN MODIFICATION
TRAPPC5	1	1.18E+00	0.935	POST TRANSLATIONAL PROTEIN MODIFICATION
LY6H	1	1.39E+00	0.941	POST TRANSLATIONAL PROTEIN MODIFICATION
PSMA4	1	6.70E-01	0.947	POST TRANSLATIONAL PROTEIN MODIFICATION
THRA	1	8.38E-01	0.948	POST TRANSLATIONAL PROTEIN MODIFICATION
FBXL19	1	6.26E+00	0.958	POST TRANSLATIONAL PROTEIN MODIFICATION
PSME2	1	6.85E+00	0.964	POST TRANSLATIONAL PROTEIN MODIFICATION
DPH3	1	3.33E-01	0.965	POST TRANSLATIONAL PROTEIN MODIFICATION
SEC22A	1	2.38E-01	0.967	POST TRANSLATIONAL PROTEIN MODIFICATION
ASB17	1	2.98E-01	0.970	POST TRANSLATIONAL PROTEIN MODIFICATION
RAB6B	1	4.41E-01	0.975	POST TRANSLATIONAL PROTEIN MODIFICATION
WDR5	1	7.75E-03	0.993	POST TRANSLATIONAL PROTEIN MODIFICATION
UBE2J2	2	1.71E+01	1	POST TRANSLATIONAL PROTEIN MODIFICATION
FBXO44	6	3.77E+03	1	POST TRANSLATIONAL PROTEIN MODIFICATION
C4A	5	1.22E+02	1	POST TRANSLATIONAL PROTEIN MODIFICATION

LMO7	25	3.03E+02	1	POST TRANSLATIONAL PROTEIN MODIFICATION
TTLL6	10	2.51E+03	1	POST TRANSLATIONAL PROTEIN MODIFICATION
MCFD2	2	2.75E+02	1	POST TRANSLATIONAL PROTEIN MODIFICATION
ST3GAL3	4	5.29E+02	1	POST TRANSLATIONAL PROTEIN MODIFICATION
FBXO11	3	4.97E+02	1	POST TRANSLATIONAL PROTEIN MODIFICATION
GALNT2	4	1.71E+02	1	POST TRANSLATIONAL PROTEIN MODIFICATION
ASB18	7	1.14E+03	1	POST TRANSLATIONAL PROTEIN MODIFICATION
UAP1	7	1.03E+03	1	POST TRANSLATIONAL PROTEIN MODIFICATION
SHISA5	4	1.62E+02	1	POST TRANSLATIONAL PROTEIN MODIFICATION
THRB	3	5.72E+01	1	POST TRANSLATIONAL PROTEIN MODIFICATION
PGR	10	3.83E+03	1	POST TRANSLATIONAL PROTEIN MODIFICATION
FSTL1	2	1.83E+01	1	POST TRANSLATIONAL PROTEIN MODIFICATION
PMM1	5	1.29E+03	1	POST TRANSLATIONAL PROTEIN MODIFICATION
USP44	5	2.90E+02	1	POST TRANSLATIONAL PROTEIN MODIFICATION
PSMF1	7	1.61E+03	1	POST TRANSLATIONAL PROTEIN MODIFICATION
NR3C2	2	1.91E+01	1	POST TRANSLATIONAL PROTEIN MODIFICATION
FUOM	4	6.20E+00	1	POST TRANSLATIONAL PROTEIN MODIFICATION
USP30	5	7.90E+02	1	POST TRANSLATIONAL PROTEIN MODIFICATION
NR3C1	2	2.23E+02	1	POST TRANSLATIONAL PROTEIN MODIFICATION
NCOA1	8	4.43E+02	1	POST TRANSLATIONAL PROTEIN MODIFICATION
PHC3	7	2.20E+03	1	POST TRANSLATIONAL PROTEIN MODIFICATION
NANP	2	1.18E+02	1	POST TRANSLATIONAL PROTEIN MODIFICATION
RPN2	6	5.08E+01	1	POST TRANSLATIONAL PROTEIN MODIFICATION
SOCS5	4	3.35E+02	1	POST TRANSLATIONAL PROTEIN MODIFICATION
ETFBKMT	3	8.56E+02	1	POST TRANSLATIONAL PROTEIN MODIFICATION
SMAD7	2	6.06E+00	1	POST TRANSLATIONAL PROTEIN MODIFICATION
NSMCE2	2	8.69E+01	1	POST TRANSLATIONAL PROTEIN MODIFICATION
WSB2	2	5.70E+02	1	POST TRANSLATIONAL PROTEIN MODIFICATION
GLB1	7	7.02E+02	1	POST TRANSLATIONAL PROTEIN MODIFICATION
ALG11	10	3.49E+01	1	POST TRANSLATIONAL PROTEIN MODIFICATION
PRND	3	1.80E+03	1	POST TRANSLATIONAL PROTEIN MODIFICATION
OTUD7A	7	1.08E+03	1	POST TRANSLATIONAL PROTEIN MODIFICATION
FCGR3B	2	2.55E+02	1	POST TRANSLATIONAL PROTEIN MODIFICATION
KLHL41	5	2.15E+03	1	POST TRANSLATIONAL PROTEIN MODIFICATION
GP2	7	1.28E+03	1	POST TRANSLATIONAL PROTEIN MODIFICATION
ST3GAL6	3	1.19E+01	1	POST TRANSLATIONAL PROTEIN MODIFICATION
SPP2	3	6.95E+01	1	POST TRANSLATIONAL PROTEIN MODIFICATION
LGALS1	3	2.82E+02	1	POST TRANSLATIONAL PROTEIN MODIFICATION
COPS7B	2	1.11E+02	1	POST TRANSLATIONAL PROTEIN MODIFICATION
AHSG	3	1.48E+03	1	POST TRANSLATIONAL PROTEIN MODIFICATION
ALG8	5	1.32E+03	1	POST TRANSLATIONAL PROTEIN MODIFICATION
ACTL6A	2	4.31E+01	1	POST TRANSLATIONAL PROTEIN MODIFICATION
PIAS2	3	5.78E+02	1	POST TRANSLATIONAL PROTEIN MODIFICATION
GFPT1	2	1.41E+01	1	POST TRANSLATIONAL PROTEIN MODIFICATION
AXIN2	10	1.64E+02	1	POST TRANSLATIONAL PROTEIN MODIFICATION
KEAP1	2	2.22E+02	1	POST TRANSLATIONAL PROTEIN MODIFICATION
PSMA8	4	5.16E+00	1	POST TRANSLATIONAL PROTEIN MODIFICATION
ADAMTS9	16	3.32E+02	1	POST TRANSLATIONAL PROTEIN MODIFICATION
DPAGT1	2	1.12E+02	1	POST TRANSLATIONAL PROTEIN MODIFICATION
IGFBP7	3	1.04E+01	1	POST TRANSLATIONAL PROTEIN MODIFICATION
UBE2C	2	3.77E+02	1	POST TRANSLATIONAL PROTEIN MODIFICATION
EID3	4	2.86E+02	1	POST TRANSLATIONAL PROTEIN MODIFICATION
USP13	9	2.93E+02	1	POST TRANSLATIONAL PROTEIN MODIFICATION
PSMD3	2	7.88E+00	1	POST TRANSLATIONAL PROTEIN MODIFICATION
USP18	3	1.07E+01	1	POST TRANSLATIONAL PROTEIN MODIFICATION
TTLL3	12	2.63E+03	1	POST TRANSLATIONAL PROTEIN MODIFICATION
THBS1	6	8.31E+02	1	POST TRANSLATIONAL PROTEIN MODIFICATION
COPB1	6	1.14E+02	1	POST TRANSLATIONAL PROTEIN MODIFICATION
AGTPBP1	6	3.63E+02	1	POST TRANSLATIONAL PROTEIN MODIFICATION
VNN1	6	1.12E+02	1	POST TRANSLATIONAL PROTEIN MODIFICATION
RAB40B	3	1.97E+02	1	POST TRANSLATIONAL PROTEIN MODIFICATION
NOTUM	4	7.22E+02	1	POST TRANSLATIONAL PROTEIN MODIFICATION
MAN1C1	3	2.76E+02	1	POST TRANSLATIONAL PROTEIN MODIFICATION
STAMBPL1	5	9.31E+01	1	POST TRANSLATIONAL PROTEIN MODIFICATION
PPP6R1	11	6.29E+03	1	POST TRANSLATIONAL PROTEIN MODIFICATION
THBS2	12	3.37E+03	1	POST TRANSLATIONAL PROTEIN MODIFICATION
LRRC41	6	2.88E+03	1	POST TRANSLATIONAL PROTEIN MODIFICATION
FBXO2	3	3.07E+02	1	POST TRANSLATIONAL PROTEIN MODIFICATION
HDAC7	15	2.61E+02	1	POST TRANSLATIONAL PROTEIN MODIFICATION
TGFB1	2	2.82E+01	1	POST TRANSLATIONAL PROTEIN MODIFICATION
TUBA4B	5	2.49E+02	1	POST TRANSLATIONAL PROTEIN MODIFICATION
ALPI	4	6.26E+02	1	POST TRANSLATIONAL PROTEIN MODIFICATION
LY6K	2	1.84E+01	1	POST TRANSLATIONAL PROTEIN MODIFICATION
PIGS	6	1.75E+03	1	POST TRANSLATIONAL PROTEIN MODIFICATION

SPACA4	2	1.75E+01	1	POST TRANSLATIONAL PROTEIN MODIFICATION
POLB	3	1.15E+02	1	POST TRANSLATIONAL PROTEIN MODIFICATION
ADAMTS1	9	7.96E+02	1	POST TRANSLATIONAL PROTEIN MODIFICATION

Gene	NumVar	Q	Pvalue	Gene-set
LRRK2	18	5.07E+04	0.016	PTK6 PROMOTES HIF1A STABILIZATION
HBEGF	1	3.11E+03	0.135	PTK6 PROMOTES HIF1A STABILIZATION
PTK6	1	2.59E+02	0.243	PTK6 PROMOTES HIF1A STABILIZATION
EGFR	6	1.87E+03	0.355	PTK6 PROMOTES HIF1A STABILIZATION
GPNMB	8	1.69E+04	0.370	PTK6 PROMOTES HIF1A STABILIZATION
HIF1A	8	4.69E+03	0.901	PTK6 PROMOTES HIF1A STABILIZATION

Gene	NumVar	Q	Pvalue	Gene-set
DRD2	13	5.66E+06	2.15E-10	SIGNALING BY GPCR
OR14C36	3	4.36E+04	3.88E-04	SIGNALING BY GPCR
OR1J4	2	4.62E+04	4.45E-04	SIGNALING BY GPCR
FFAR3	14	3.28E+05	0.001	SIGNALING BY GPCR
RRH	5	3.58E+04	0.001	SIGNALING BY GPCR
OR13F1	4	4.37E+04	0.002	SIGNALING BY GPCR
GRK4	9	5.41E+04	0.003	SIGNALING BY GPCR
OR52N2	5	2.07E+04	0.004	SIGNALING BY GPCR
PRKCE	3	2.09E+04	0.008	SIGNALING BY GPCR
OR1D5	2	8.92E+03	0.008	SIGNALING BY GPCR
QRFPR	8	1.58E+05	0.010	SIGNALING BY GPCR
OR2D3	2	2.11E+04	0.010	SIGNALING BY GPCR
RDH5	3	5.62E+03	0.011	SIGNALING BY GPCR
OR8B3	4	8.11E+03	0.012	SIGNALING BY GPCR
WNT2	4	2.90E+04	0.012	SIGNALING BY GPCR
OR6C76	4	1.04E+04	0.014	SIGNALING BY GPCR
OR2V2	4	2.33E+04	0.014	SIGNALING BY GPCR
OR51J1	9	4.34E+04	0.014	SIGNALING BY GPCR
OR10H4	8	3.90E+04	0.015	SIGNALING BY GPCR
OR51Q1	10	7.81E+04	0.015	SIGNALING BY GPCR
RGS21	3	2.74E+04	0.017	SIGNALING BY GPCR
OR10J5	8	8.93E+03	0.017	SIGNALING BY GPCR
C5AR1	7	4.11E+04	0.018	SIGNALING BY GPCR
OR56A4	8	1.95E+04	0.018	SIGNALING BY GPCR
ADRA1D	8	4.79E+04	0.019	SIGNALING BY GPCR
OR51L1	4	3.44E+04	0.021	SIGNALING BY GPCR
APP	2	3.42E+03	0.021	SIGNALING BY GPCR
GNAT2	6	4.14E+04	0.021	SIGNALING BY GPCR
OR5M8	7	3.13E+04	0.024	SIGNALING BY GPCR
OR1D2	5	7.82E+03	0.025	SIGNALING BY GPCR
SSTR4	6	1.98E+04	0.026	SIGNALING BY GPCR
FZD7	1	1.47E+04	0.028	SIGNALING BY GPCR
GNG4	1	4.00E+03	0.028	SIGNALING BY GPCR
OR4F17	5	2.11E+04	0.028	SIGNALING BY GPCR
OR52N4	6	2.73E+04	0.028	SIGNALING BY GPCR
PROK2	2	1.68E+03	0.028	SIGNALING BY GPCR
OR10AG1	4	1.30E+04	0.029	SIGNALING BY GPCR
TAS2R13	1	2.03E+03	0.031	SIGNALING BY GPCR
RBP4	1	3.06E+03	0.032	SIGNALING BY GPCR
ADM2	4	6.37E+03	0.034	SIGNALING BY GPCR
RDH11	2	1.79E+04	0.034	SIGNALING BY GPCR
MC4R	5	4.32E+04	0.034	SIGNALING BY GPCR
OR52E4	3	9.88E+03	0.035	SIGNALING BY GPCR
NMBR	7	3.42E+04	0.037	SIGNALING BY GPCR
OR5K1	7	2.14E+04	0.038	SIGNALING BY GPCR
OR2J2	6	3.51E+04	0.039	SIGNALING BY GPCR
CXCL13	1	5.44E+02	0.040	SIGNALING BY GPCR
OR2W1	2	1.44E+04	0.041	SIGNALING BY GPCR
MLNR	1	1.22E+04	0.042	SIGNALING BY GPCR
DAGLA	4	1.02E+04	0.044	SIGNALING BY GPCR
RETSAT	11	9.81E+04	0.045	SIGNALING BY GPCR
F2	5	7.45E+03	0.047	SIGNALING BY GPCR
ARHGEF38	3	3.75E+03	0.052	SIGNALING BY GPCR
RGS13	1	1.23E+03	0.052	SIGNALING BY GPCR
RGS4	1	5.99E+02	0.054	SIGNALING BY GPCR
RBP1	2	2.88E+03	0.055	SIGNALING BY GPCR
CCL25	2	1.51E+04	0.055	SIGNALING BY GPCR
OR2A25	5	9.09E+03	0.056	SIGNALING BY GPCR
OR2A12	2	4.76E+03	0.057	SIGNALING BY GPCR
OR2M3	7	1.98E+04	0.057	SIGNALING BY GPCR
APOA4	9	2.92E+04	0.057	SIGNALING BY GPCR

ARHGEF4	29	5.93E+04	0.058	SIGNALING BY GPCR
TAS2R39	4	1.10E+04	0.058	SIGNALING BY GPCR
OR2C3	4	1.03E+04	0.059	SIGNALING BY GPCR
INSL5	3	7.77E+03	0.060	SIGNALING BY GPCR
GNG10	1	4.84E+03	0.060	SIGNALING BY GPCR
AKR1C3	9	3.31E+04	0.060	SIGNALING BY GPCR
OR5B2	3	2.83E+03	0.061	SIGNALING BY GPCR
RLN2	6	1.71E+04	0.062	SIGNALING BY GPCR
HSPG2	81	1.01E+05	0.064	SIGNALING BY GPCR
APLNR	4	6.90E+03	0.065	SIGNALING BY GPCR
GNAS	16	3.79E+04	0.066	SIGNALING BY GPCR
GPHB5	3	1.53E+04	0.067	SIGNALING BY GPCR
P2RY6	4	5.25E+03	0.068	SIGNALING BY GPCR
OR5P3	5	3.51E+04	0.068	SIGNALING BY GPCR
OR2W3	8	1.36E+04	0.068	SIGNALING BY GPCR
CRHBP	3	5.16E+03	0.071	SIGNALING BY GPCR
OR5H1	11	1.97E+04	0.072	SIGNALING BY GPCR
OR52E5	4	1.80E+04	0.077	SIGNALING BY GPCR
FGD2	14	2.82E+04	0.077	SIGNALING BY GPCR
ITPR1	13	3.91E+04	0.079	SIGNALING BY GPCR
OR8J1	5	2.84E+04	0.079	SIGNALING BY GPCR
OR52B6	1	6.47E+02	0.080	SIGNALING BY GPCR
ADCY6	12	4.54E+04	0.080	SIGNALING BY GPCR
OR5T1	2	1.14E+03	0.081	SIGNALING BY GPCR
OR2T33	13	3.25E+04	0.081	SIGNALING BY GPCR
LPAR5	1	4.80E+02	0.082	SIGNALING BY GPCR
NPBWR1	3	5.56E+03	0.083	SIGNALING BY GPCR
OR4X1	8	3.09E+04	0.084	SIGNALING BY GPCR
KNG1	2	3.00E+03	0.084	SIGNALING BY GPCR
OR11H4	7	1.86E+04	0.085	SIGNALING BY GPCR
TAAR9	5	8.59E+03	0.085	SIGNALING BY GPCR
GHRHR	2	1.23E+04	0.088	SIGNALING BY GPCR
CX3CL1	5	1.21E+04	0.088	SIGNALING BY GPCR
CCR2	3	1.69E+03	0.088	SIGNALING BY GPCR
CAMK2B	3	2.35E+03	0.089	SIGNALING BY GPCR
OR51E2	4	1.60E+04	0.089	SIGNALING BY GPCR
CCL27	2	2.45E+03	0.090	SIGNALING BY GPCR
OR4Q2	2	2.23E+03	0.091	SIGNALING BY GPCR
GRP	2	8.99E+02	0.092	SIGNALING BY GPCR
RPS6KA1	4	6.96E+03	0.097	SIGNALING BY GPCR
PSAP	3	3.19E+03	0.097	SIGNALING BY GPCR
OR5J2	7	1.42E+04	0.099	SIGNALING BY GPCR
REEP4	3	1.65E+04	0.103	SIGNALING BY GPCR
SOS2	8	3.67E+04	0.105	SIGNALING BY GPCR
SDC4	2	9.86E+02	0.105	SIGNALING BY GPCR
APOB	42	8.05E+04	0.105	SIGNALING BY GPCR
OR2K2	2	1.21E+03	0.105	SIGNALING BY GPCR
PIK3CA	2	7.43E+03	0.109	SIGNALING BY GPCR
OR1N2	2	8.65E+03	0.110	SIGNALING BY GPCR
ADRB1	1	1.27E+03	0.110	SIGNALING BY GPCR
GAST	4	6.01E+03	0.110	SIGNALING BY GPCR
REEP5	2	1.02E+04	0.110	SIGNALING BY GPCR
OR10G3	6	2.43E+04	0.111	SIGNALING BY GPCR
SOS1	5	1.70E+04	0.112	SIGNALING BY GPCR
OR8D2	3	4.87E+03	0.112	SIGNALING BY GPCR
OR8I2	5	9.96E+03	0.114	SIGNALING BY GPCR
KPNA2	5	1.51E+04	0.115	SIGNALING BY GPCR
PTH1R	4	2.07E+04	0.116	SIGNALING BY GPCR
GIPR	9	1.08E+04	0.116	SIGNALING BY GPCR
TAS1R1	19	5.90E+04	0.117	SIGNALING BY GPCR
PLPPR4	5	1.65E+04	0.117	SIGNALING BY GPCR
SMO	11	2.18E+04	0.118	SIGNALING BY GPCR
BCO1	9	1.97E+04	0.118	SIGNALING BY GPCR
WNT7B	1	6.05E+02	0.118	SIGNALING BY GPCR
PRKACB	2	2.25E+03	0.121	SIGNALING BY GPCR
F2RL1	4	1.12E+04	0.121	SIGNALING BY GPCR
OR4N5	6	5.97E+03	0.124	SIGNALING BY GPCR
WNT9A	3	3.40E+03	0.125	SIGNALING BY GPCR
HTR5A	1	9.91E+02	0.126	SIGNALING BY GPCR
OR10T2	7	5.95E+03	0.126	SIGNALING BY GPCR
OR6C74	5	1.11E+04	0.126	SIGNALING BY GPCR
CNR1	1	5.34E+02	0.126	SIGNALING BY GPCR
OR2M5	14	1.77E+05	0.127	SIGNALING BY GPCR
OR8S1	2	4.49E+03	0.127	SIGNALING BY GPCR

TIAM1	17	5.29E+04	0.128	SIGNALING BY GPCR
OR2G2	1	8.43E+03	0.131	SIGNALING BY GPCR
TBXA2R	12	2.12E+04	0.133	SIGNALING BY GPCR
OR2D2	8	1.58E+04	0.133	SIGNALING BY GPCR
PIK3CG	7	1.67E+04	0.134	SIGNALING BY GPCR
OR2A14	5	2.94E+03	0.134	SIGNALING BY GPCR
HBEGF	1	3.11E+03	0.135	SIGNALING BY GPCR
OR52D1	4	4.01E+03	0.135	SIGNALING BY GPCR
CXCR4	3	5.43E+03	0.136	SIGNALING BY GPCR
WNT10B	4	1.61E+04	0.136	SIGNALING BY GPCR
AHCYL1	1	6.32E+02	0.138	SIGNALING BY GPCR
OR4B1	3	4.18E+03	0.139	SIGNALING BY GPCR
OR52E6	5	5.77E+03	0.139	SIGNALING BY GPCR
NPB	4	5.38E+03	0.141	SIGNALING BY GPCR
GPR20	5	9.44E+03	0.143	SIGNALING BY GPCR
MCF2L	22	4.31E+04	0.143	SIGNALING BY GPCR
OR8H1	2	1.22E+04	0.143	SIGNALING BY GPCR
ARHGEF17	24	3.81E+04	0.145	SIGNALING BY GPCR
ADRB2	2	8.42E+03	0.146	SIGNALING BY GPCR
HTR1A	3	1.64E+04	0.147	SIGNALING BY GPCR
PLEKHG2	15	2.36E+04	0.148	SIGNALING BY GPCR
CHRM2	3	8.45E+03	0.148	SIGNALING BY GPCR
TAS2R50	1	4.83E+02	0.149	SIGNALING BY GPCR
PTGFR	6	1.78E+04	0.150	SIGNALING BY GPCR
GCG	3	3.55E+03	0.151	SIGNALING BY GPCR
OR2T8	14	1.22E+05	0.155	SIGNALING BY GPCR
S1PR2	4	1.54E+04	0.156	SIGNALING BY GPCR
OR4D1	3	4.03E+03	0.159	SIGNALING BY GPCR
OR11H1	15	2.76E+04	0.159	SIGNALING BY GPCR
APOA1	3	2.38E+03	0.160	SIGNALING BY GPCR
OR52J3	4	5.45E+03	0.160	SIGNALING BY GPCR
ABHD12	3	3.28E+03	0.160	SIGNALING BY GPCR
OR5H6	9	4.43E+04	0.161	SIGNALING BY GPCR
CAMKK1	3	4.94E+03	0.161	SIGNALING BY GPCR
CCR3	7	2.09E+04	0.161	SIGNALING BY GPCR
PTGDR	6	1.47E+04	0.161	SIGNALING BY GPCR
GPR45	2	5.44E+02	0.162	SIGNALING BY GPCR
CNGA1	6	1.67E+04	0.162	SIGNALING BY GPCR
RGSL1	11	1.01E+04	0.167	SIGNALING BY GPCR
GNAI3	1	4.73E+02	0.168	SIGNALING BY GPCR
OR6B2	5	1.62E+04	0.168	SIGNALING BY GPCR
OR2AT4	4	8.75E+03	0.168	SIGNALING BY GPCR
OR10A3	6	9.71E+03	0.169	SIGNALING BY GPCR
OR5D16	1	4.21E+02	0.171	SIGNALING BY GPCR
HTR2B	1	2.85E+02	0.171	SIGNALING BY GPCR
DGKQ	5	7.90E+03	0.171	SIGNALING BY GPCR
OR2T11	6	8.16E+03	0.175	SIGNALING BY GPCR
OR6K3	4	2.56E+03	0.175	SIGNALING BY GPCR
OPN4	8	2.53E+04	0.177	SIGNALING BY GPCR
PRKAR1B	5	6.22E+03	0.178	SIGNALING BY GPCR
RGS18	1	2.84E+02	0.178	SIGNALING BY GPCR
ANXA1	2	2.11E+03	0.179	SIGNALING BY GPCR
OR5A1	3	8.33E+03	0.180	SIGNALING BY GPCR
OR7C1	10	1.38E+04	0.180	SIGNALING BY GPCR
OR6C70	1	2.81E+02	0.180	SIGNALING BY GPCR
DHRS3	4	4.99E+03	0.181	SIGNALING BY GPCR
OR5K3	8	1.75E+04	0.183	SIGNALING BY GPCR
GCGR	6	1.09E+04	0.184	SIGNALING BY GPCR
PIK3R1	1	3.44E+02	0.185	SIGNALING BY GPCR
CXCR1	8	1.15E+04	0.187	SIGNALING BY GPCR
OR8G5	8	5.30E+04	0.187	SIGNALING BY GPCR
ACKR3	4	9.26E+03	0.188	SIGNALING BY GPCR
HTR1B	1	6.22E+03	0.191	SIGNALING BY GPCR
CXCL3	2	6.60E+03	0.191	SIGNALING BY GPCR
OR7G3	6	8.76E+03	0.193	SIGNALING BY GPCR
GRM7	1	4.46E+02	0.193	SIGNALING BY GPCR
GNA13	5	6.93E+03	0.194	SIGNALING BY GPCR
ARHGEF5	14	1.89E+04	0.195	SIGNALING BY GPCR
PLCB2	12	1.50E+04	0.196	SIGNALING BY GPCR
GPR83	3	2.96E+03	0.197	SIGNALING BY GPCR
OR9Q2	2	6.45E+03	0.199	SIGNALING BY GPCR
RTP4	4	1.30E+04	0.201	SIGNALING BY GPCR
OR4K15	6	1.03E+04	0.201	SIGNALING BY GPCR
ROCK1	3	1.09E+04	0.202	SIGNALING BY GPCR

CORT	1	3.50E+02	0.202	SIGNALING BY GPCR
PDE4D	10	1.89E+04	0.202	SIGNALING BY GPCR
OR6K2	2	1.71E+03	0.203	SIGNALING BY GPCR
OR51I1	8	3.02E+04	0.203	SIGNALING BY GPCR
FZD4	5	2.95E+04	0.203	SIGNALING BY GPCR
OR1J1	7	2.21E+04	0.204	SIGNALING BY GPCR
CYSLTR2	7	5.38E+04	0.204	SIGNALING BY GPCR
GPR37	7	1.15E+04	0.206	SIGNALING BY GPCR
ARHGEF16	11	2.31E+04	0.206	SIGNALING BY GPCR
OR8D4	3	3.55E+03	0.207	SIGNALING BY GPCR
REEP1	2	7.67E+02	0.208	SIGNALING BY GPCR
OR13J1	3	1.61E+03	0.209	SIGNALING BY GPCR
OR6C3	4	5.11E+03	0.209	SIGNALING BY GPCR
FFAR4	2	6.99E+03	0.210	SIGNALING BY GPCR
OR5R1	5	1.43E+04	0.210	SIGNALING BY GPCR
OR13C4	5	1.43E+04	0.210	SIGNALING BY GPCR
OR2T4	8	1.38E+04	0.210	SIGNALING BY GPCR
OR6C2	4	5.06E+03	0.211	SIGNALING BY GPCR
CCL5	1	3.12E+02	0.211	SIGNALING BY GPCR
PRKCD	5	4.92E+03	0.211	SIGNALING BY GPCR
CRHR2	11	2.24E+04	0.214	SIGNALING BY GPCR
PDE1A	5	3.11E+03	0.215	SIGNALING BY GPCR
TIAM2	26	4.92E+04	0.215	SIGNALING BY GPCR
PDE10A	3	6.23E+03	0.217	SIGNALING BY GPCR
GPR27	2	9.06E+02	0.218	SIGNALING BY GPCR
OR1C1	3	1.95E+03	0.218	SIGNALING BY GPCR
OR4K14	2	9.56E+02	0.220	SIGNALING BY GPCR
OR2T27	12	2.92E+04	0.221	SIGNALING BY GPCR
RTP1	4	1.05E+04	0.221	SIGNALING BY GPCR
OR5AU1	2	9.77E+02	0.222	SIGNALING BY GPCR
OR51T1	4	9.52E+03	0.222	SIGNALING BY GPCR
OR7A5	7	9.76E+03	0.225	SIGNALING BY GPCR
AVPR1B	9	1.01E+04	0.228	SIGNALING BY GPCR
ADRA2A	4	3.12E+03	0.228	SIGNALING BY GPCR
POMC	7	1.57E+04	0.229	SIGNALING BY GPCR
RGS12	16	3.91E+04	0.230	SIGNALING BY GPCR
GPR17	11	9.27E+03	0.232	SIGNALING BY GPCR
GRM6	17	3.20E+04	0.234	SIGNALING BY GPCR
OR10R2	5	1.30E+04	0.235	SIGNALING BY GPCR
CALCB	1	3.69E+02	0.237	SIGNALING BY GPCR
PLA2G4A	3	1.33E+04	0.237	SIGNALING BY GPCR
ADCY7	10	1.36E+04	0.238	SIGNALING BY GPCR
OR4M1	7	5.46E+03	0.239	SIGNALING BY GPCR
RGS6	7	1.03E+04	0.240	SIGNALING BY GPCR
NMUR2	7	1.75E+04	0.244	SIGNALING BY GPCR
OR7C2	4	2.35E+04	0.245	SIGNALING BY GPCR
OR10K1	1	9.61E+02	0.247	SIGNALING BY GPCR
OR51G1	9	8.48E+03	0.248	SIGNALING BY GPCR
OR5D18	3	1.02E+04	0.248	SIGNALING BY GPCR
PPP3CA	2	8.30E+02	0.249	SIGNALING BY GPCR
OR1I1	4	2.91E+04	0.250	SIGNALING BY GPCR
OR10D3	3	1.60E+03	0.250	SIGNALING BY GPCR
GALR1	4	2.03E+03	0.250	SIGNALING BY GPCR
OR5L1	2	9.23E+02	0.251	SIGNALING BY GPCR
NMT1	1	2.00E+02	0.256	SIGNALING BY GPCR
CXCL2	3	1.19E+04	0.258	SIGNALING BY GPCR
ARHGEF26	9	1.74E+04	0.259	SIGNALING BY GPCR
OR1L1	6	9.04E+03	0.262	SIGNALING BY GPCR
GNRH1	1	1.34E+03	0.266	SIGNALING BY GPCR
PTGER1	6	6.37E+03	0.267	SIGNALING BY GPCR
OR4X2	2	1.81E+03	0.267	SIGNALING BY GPCR
OR51M1	10	1.46E+04	0.268	SIGNALING BY GPCR
OR2A2	1	4.29E+02	0.268	SIGNALING BY GPCR
OR1N1	7	8.38E+03	0.269	SIGNALING BY GPCR
OR10J1	8	1.03E+04	0.271	SIGNALING BY GPCR
AKR1C1	10	2.10E+04	0.271	SIGNALING BY GPCR
RDH10	1	3.13E+03	0.275	SIGNALING BY GPCR
NLN	7	5.29E+03	0.275	SIGNALING BY GPCR
OR13C2	3	7.28E+03	0.276	SIGNALING BY GPCR
KEL	8	9.16E+03	0.278	SIGNALING BY GPCR
CCR9	4	8.16E+03	0.279	SIGNALING BY GPCR
PIK3R6	11	1.39E+04	0.279	SIGNALING BY GPCR
TAS2R43	4	2.08E+04	0.282	SIGNALING BY GPCR
OR1L6	6	1.35E+04	0.282	SIGNALING BY GPCR

ECT2	7	4.29E+03	0.283	SIGNALING BY GPCR
CAMKMT	2	6.22E+02	0.283	SIGNALING BY GPCR
APOC2	2	1.85E+03	0.285	SIGNALING BY GPCR
DGKB	5	8.22E+03	0.285	SIGNALING BY GPCR
OR4C16	7	1.24E+04	0.286	SIGNALING BY GPCR
OR9A2	5	4.09E+03	0.286	SIGNALING BY GPCR
CXCL9	1	5.36E+02	0.289	SIGNALING BY GPCR
PLEKHG5	18	1.58E+04	0.290	SIGNALING BY GPCR
RXFP3	5	2.24E+03	0.290	SIGNALING BY GPCR
OR4Q3	6	1.01E+04	0.290	SIGNALING BY GPCR
TAS2R14	4	1.25E+04	0.293	SIGNALING BY GPCR
C5	12	1.23E+04	0.294	SIGNALING BY GPCR
RASGRF2	5	5.78E+03	0.295	SIGNALING BY GPCR
GPER1	5	9.33E+03	0.297	SIGNALING BY GPCR
CNR2	5	6.09E+03	0.298	SIGNALING BY GPCR
OR2AJ1	3	8.03E+03	0.298	SIGNALING BY GPCR
UCN3	1	2.38E+02	0.298	SIGNALING BY GPCR
WNT8A	2	1.07E+03	0.300	SIGNALING BY GPCR
PIK3R2	7	5.87E+03	0.300	SIGNALING BY GPCR
FSHR	5	4.26E+03	0.301	SIGNALING BY GPCR
S1PR4	11	1.41E+04	0.301	SIGNALING BY GPCR
GPR18	2	3.08E+03	0.301	SIGNALING BY GPCR
VIPR2	7	5.04E+03	0.302	SIGNALING BY GPCR
PPP3CC	1	3.03E+03	0.303	SIGNALING BY GPCR
OR6C75	3	1.84E+03	0.303	SIGNALING BY GPCR
HRH3	2	8.34E+02	0.304	SIGNALING BY GPCR
ARHGEF33	4	1.42E+04	0.306	SIGNALING BY GPCR
MAPK7	5	3.72E+03	0.306	SIGNALING BY GPCR
OR2Y1	5	4.00E+04	0.307	SIGNALING BY GPCR
TRHR	4	2.37E+03	0.307	SIGNALING BY GPCR
CCR6	1	3.00E+03	0.307	SIGNALING BY GPCR
OR5I1	4	2.61E+03	0.308	SIGNALING BY GPCR
PTGIR	4	1.04E+04	0.308	SIGNALING BY GPCR
CXCL1	1	7.24E+02	0.308	SIGNALING BY GPCR
OR10X1	5	8.70E+03	0.309	SIGNALING BY GPCR
OR2V1	4	2.85E+03	0.310	SIGNALING BY GPCR
MC2R	5	5.79E+03	0.313	SIGNALING BY GPCR
WNT11	1	3.04E+02	0.315	SIGNALING BY GPCR
LPL	4	1.26E+04	0.316	SIGNALING BY GPCR
OR2L3	3	5.62E+03	0.317	SIGNALING BY GPCR
OR14A2	7	1.80E+04	0.318	SIGNALING BY GPCR
TACR1	2	9.74E+02	0.318	SIGNALING BY GPCR
ARHGEF15	10	1.36E+04	0.318	SIGNALING BY GPCR
OR6N2	2	3.56E+03	0.320	SIGNALING BY GPCR
OR6V1	5	4.89E+03	0.324	SIGNALING BY GPCR
CNGB1	15	2.18E+04	0.325	SIGNALING BY GPCR
IHH	5	1.99E+03	0.326	SIGNALING BY GPCR
OR8K1	3	1.79E+03	0.326	SIGNALING BY GPCR
DGKZ	12	2.49E+04	0.327	SIGNALING BY GPCR
APOC3	1	5.25E+02	0.327	SIGNALING BY GPCR
LTB4R	4	1.86E+03	0.327	SIGNALING BY GPCR
GRM5	4	1.84E+03	0.328	SIGNALING BY GPCR
OR14J1	2	1.15E+03	0.329	SIGNALING BY GPCR
OR1S2	11	9.09E+03	0.330	SIGNALING BY GPCR
SDR9C7	4	2.09E+03	0.332	SIGNALING BY GPCR
RHOB	1	3.01E+02	0.336	SIGNALING BY GPCR
HEBP1	3	1.16E+03	0.337	SIGNALING BY GPCR
OR1L3	9	2.65E+04	0.339	SIGNALING BY GPCR
LRP2	49	9.97E+04	0.340	SIGNALING BY GPCR
PTAFR	3	2.22E+03	0.341	SIGNALING BY GPCR
RAMP1	1	1.35E+02	0.342	SIGNALING BY GPCR
PDE8A	3	1.81E+03	0.342	SIGNALING BY GPCR
GPR176	1	2.06E+02	0.343	SIGNALING BY GPCR
NPFFR1	8	5.71E+03	0.347	SIGNALING BY GPCR
OR6F1	1	2.99E+03	0.348	SIGNALING BY GPCR
OR4E2	5	5.57E+03	0.348	SIGNALING BY GPCR
CXCR5	4	5.22E+03	0.349	SIGNALING BY GPCR
PDE2A	5	2.09E+03	0.352	SIGNALING BY GPCR
PCP2	2	1.39E+03	0.353	SIGNALING BY GPCR
OR6C6	5	7.48E+03	0.354	SIGNALING BY GPCR
OR56A5	3	7.44E+03	0.354	SIGNALING BY GPCR
EGFR	6	1.87E+03	0.355	SIGNALING BY GPCR
OR9G4	8	6.12E+03	0.355	SIGNALING BY GPCR
ADCY3	11	4.81E+03	0.359	SIGNALING BY GPCR

ACKR4	5	3.86E+03	0.361	SIGNALING BY GPCR
TAC3	1	1.28E+02	0.362	SIGNALING BY GPCR
HRH2	3	1.80E+03	0.362	SIGNALING BY GPCR
GPRC6A	9	1.86E+04	0.362	SIGNALING BY GPCR
GUCA1B	1	2.76E+03	0.363	SIGNALING BY GPCR
OR2T3	11	1.15E+04	0.364	SIGNALING BY GPCR
PIK3R3	4	3.69E+03	0.365	SIGNALING BY GPCR
OR52R1	1	5.25E+02	0.366	SIGNALING BY GPCR
APLF	5	4.98E+03	0.366	SIGNALING BY GPCR
CCL23	2	3.99E+03	0.367	SIGNALING BY GPCR
OR8B8	4	4.30E+03	0.368	SIGNALING BY GPCR
OPN1SW	3	1.34E+03	0.371	SIGNALING BY GPCR
OR4C13	9	7.99E+03	0.372	SIGNALING BY GPCR
ABCA4	35	4.32E+04	0.373	SIGNALING BY GPCR
GHRH	1	2.90E+03	0.373	SIGNALING BY GPCR
OPRM1	16	1.52E+04	0.374	SIGNALING BY GPCR
OR14I1	6	5.64E+03	0.374	SIGNALING BY GPCR
OR2A1	3	2.81E+03	0.375	SIGNALING BY GPCR
OR2A42	3	2.81E+03	0.375	SIGNALING BY GPCR
TRPC7	3	2.24E+03	0.375	SIGNALING BY GPCR
OR6J1	2	3.83E+03	0.376	SIGNALING BY GPCR
DRD5	6	5.99E+03	0.376	SIGNALING BY GPCR
OR4D6	8	1.62E+04	0.378	SIGNALING BY GPCR
CALCR	6	4.50E+03	0.378	SIGNALING BY GPCR
GRM4	2	8.27E+02	0.380	SIGNALING BY GPCR
OR2L2	5	4.03E+03	0.380	SIGNALING BY GPCR
OR52W1	4	3.69E+03	0.382	SIGNALING BY GPCR
AKR1C4	2	5.51E+02	0.386	SIGNALING BY GPCR
OR56A1	4	6.35E+03	0.387	SIGNALING BY GPCR
GNRHR	5	5.57E+03	0.387	SIGNALING BY GPCR
OR11L1	3	2.39E+03	0.388	SIGNALING BY GPCR
OR10A2	3	1.97E+03	0.388	SIGNALING BY GPCR
ARHGEF3	5	3.60E+03	0.389	SIGNALING BY GPCR
TAAR8	2	1.09E+03	0.390	SIGNALING BY GPCR
ADCY4	12	1.52E+04	0.392	SIGNALING BY GPCR
GUCA1A	5	3.36E+03	0.392	SIGNALING BY GPCR
PLCB1	8	6.14E+03	0.392	SIGNALING BY GPCR
GPR31	4	2.12E+03	0.393	SIGNALING BY GPCR
ITPR2	10	9.65E+03	0.393	SIGNALING BY GPCR
OR5C1	4	2.46E+03	0.393	SIGNALING BY GPCR
FZD3	3	1.34E+03	0.393	SIGNALING BY GPCR
TAS2R46	2	9.42E+02	0.394	SIGNALING BY GPCR
OR14K1	2	3.31E+03	0.394	SIGNALING BY GPCR
PROKR2	7	5.79E+03	0.396	SIGNALING BY GPCR
GPR132	2	2.70E+03	0.398	SIGNALING BY GPCR
DRD4	25	3.81E+04	0.398	SIGNALING BY GPCR
OR2L8	9	4.83E+03	0.399	SIGNALING BY GPCR
OR2T10	2	1.05E+03	0.400	SIGNALING BY GPCR
SST	4	9.12E+03	0.401	SIGNALING BY GPCR
OR13D1	6	7.49E+03	0.402	SIGNALING BY GPCR
RGS7	4	1.46E+03	0.403	SIGNALING BY GPCR
OR2T2	13	1.01E+04	0.403	SIGNALING BY GPCR
NTS	3	1.62E+03	0.404	SIGNALING BY GPCR
OR5L2	4	3.59E+03	0.406	SIGNALING BY GPCR
TAS2R30	23	4.50E+04	0.410	SIGNALING BY GPCR
OR2C1	7	8.28E+03	0.411	SIGNALING BY GPCR
WNT3A	2	4.57E+02	0.412	SIGNALING BY GPCR
NMB	1	3.51E+02	0.417	SIGNALING BY GPCR
ARHGEF37	7	5.88E+03	0.417	SIGNALING BY GPCR
OR5AR1	3	1.18E+03	0.419	SIGNALING BY GPCR
OR3A3	3	5.12E+03	0.425	SIGNALING BY GPCR
RAMP3	2	5.45E+02	0.427	SIGNALING BY GPCR
OR4N2	8	6.46E+03	0.427	SIGNALING BY GPCR
OR51A7	4	3.59E+03	0.427	SIGNALING BY GPCR
GPC1	11	1.82E+04	0.430	SIGNALING BY GPCR
OR52K1	5	5.79E+03	0.431	SIGNALING BY GPCR
FSHB	3	2.34E+03	0.432	SIGNALING BY GPCR
OR52A5	4	3.54E+03	0.432	SIGNALING BY GPCR
OR10Z1	4	7.23E+03	0.432	SIGNALING BY GPCR
TACR2	9	6.30E+03	0.433	SIGNALING BY GPCR
OR1J2	4	5.21E+03	0.435	SIGNALING BY GPCR
LRP10	7	5.21E+03	0.435	SIGNALING BY GPCR
PRKCG	1	1.84E+02	0.435	SIGNALING BY GPCR
OR2T1	4	3.34E+03	0.436	SIGNALING BY GPCR

STRA6	8	8.72E+03	0.437	SIGNALING BY GPCR
ADGRE5	16	1.21E+04	0.437	SIGNALING BY GPCR
C3AR1	2	3.22E+03	0.439	SIGNALING BY GPCR
PDE4C	2	1.55E+03	0.440	SIGNALING BY GPCR
OR2J1	6	4.48E+03	0.440	SIGNALING BY GPCR
OR5P2	6	3.05E+03	0.441	SIGNALING BY GPCR
DGKH	13	1.25E+04	0.442	SIGNALING BY GPCR
OR2AK2	2	1.36E+03	0.443	SIGNALING BY GPCR
PLPPR1	2	3.09E+03	0.443	SIGNALING BY GPCR
FGD3	5	5.15E+03	0.443	SIGNALING BY GPCR
OR5M10	5	2.01E+03	0.444	SIGNALING BY GPCR
NGEF	6	5.07E+03	0.445	SIGNALING BY GPCR
TAS2R16	4	1.56E+03	0.445	SIGNALING BY GPCR
QRFP	3	7.08E+03	0.446	SIGNALING BY GPCR
PENK	4	1.99E+03	0.448	SIGNALING BY GPCR
OR4K2	4	3.71E+03	0.448	SIGNALING BY GPCR
MYO7A	24	1.92E+04	0.449	SIGNALING BY GPCR
CCR4	1	1.22E+02	0.449	SIGNALING BY GPCR
GPC2	5	1.59E+03	0.452	SIGNALING BY GPCR
GPR32	8	6.71E+03	0.454	SIGNALING BY GPCR
RDH16	5	1.98E+03	0.455	SIGNALING BY GPCR
CCRL2	5	8.35E+03	0.456	SIGNALING BY GPCR
OR13C8	1	1.91E+03	0.459	SIGNALING BY GPCR
OR10G9	15	4.72E+04	0.460	SIGNALING BY GPCR
OR5W2	1	1.29E+02	0.462	SIGNALING BY GPCR
GABBR1	3	1.77E+03	0.462	SIGNALING BY GPCR
ARHGEF40	10	1.19E+04	0.462	SIGNALING BY GPCR
PLPPR2	5	4.02E+03	0.464	SIGNALING BY GPCR
PRKCQ	5	1.70E+03	0.464	SIGNALING BY GPCR
TAS2R38	3	1.69E+03	0.465	SIGNALING BY GPCR
AKR1B10	10	7.59E+03	0.466	SIGNALING BY GPCR
LHCGR	2	1.24E+03	0.466	SIGNALING BY GPCR
OR6P1	7	7.75E+03	0.466	SIGNALING BY GPCR
S1PR5	4	6.22E+03	0.470	SIGNALING BY GPCR
SSTR3	13	6.32E+03	0.471	SIGNALING BY GPCR
OR4K13	1	1.43E+03	0.474	SIGNALING BY GPCR
GPBAR1	5	2.77E+03	0.475	SIGNALING BY GPCR
FNTA	3	1.61E+03	0.475	SIGNALING BY GPCR
MC5R	3	2.21E+03	0.475	SIGNALING BY GPCR
OR5T3	4	3.93E+03	0.477	SIGNALING BY GPCR
MC3R	2	9.04E+02	0.477	SIGNALING BY GPCR
OR51A4	5	6.20E+03	0.478	SIGNALING BY GPCR
OR10AD1	4	2.91E+03	0.478	SIGNALING BY GPCR
TAS1R3	16	1.18E+04	0.479	SIGNALING BY GPCR
GPR39	7	5.36E+03	0.481	SIGNALING BY GPCR
OR1K1	10	1.37E+04	0.481	SIGNALING BY GPCR
NPY2R	1	2.28E+02	0.482	SIGNALING BY GPCR
PTCH1	17	9.47E+03	0.483	SIGNALING BY GPCR
GPIHBP1	5	1.86E+03	0.483	SIGNALING BY GPCR
F2R	2	6.48E+02	0.484	SIGNALING BY GPCR
SSTR5	14	5.90E+03	0.484	SIGNALING BY GPCR
RXFP4	8	4.86E+03	0.485	SIGNALING BY GPCR
OR51B5	55	6.11E+04	0.486	SIGNALING BY GPCR
OR2J3	5	4.38E+03	0.488	SIGNALING BY GPCR
UCN2	2	1.05E+03	0.488	SIGNALING BY GPCR
KISS1	1	9.08E+01	0.488	SIGNALING BY GPCR
ADCY2	4	3.70E+03	0.499	SIGNALING BY GPCR
PTH2	4	1.75E+03	0.499	SIGNALING BY GPCR
OR1Q1	4	2.84E+03	0.500	SIGNALING BY GPCR
SDC1	6	3.25E+03	0.500	SIGNALING BY GPCR
ADRA1B	6	2.10E+03	0.501	SIGNALING BY GPCR
OR4A47	7	1.15E+04	0.502	SIGNALING BY GPCR
GPR55	2	4.16E+02	0.503	SIGNALING BY GPCR
CXCR6	2	1.13E+03	0.505	SIGNALING BY GPCR
OR1B1	4	4.11E+03	0.505	SIGNALING BY GPCR
GALR2	4	6.54E+03	0.506	SIGNALING BY GPCR
CCL16	1	1.49E+03	0.506	SIGNALING BY GPCR
OR4S1	3	6.20E+02	0.506	SIGNALING BY GPCR
OR8H3	3	1.37E+03	0.506	SIGNALING BY GPCR
OPRK1	3	5.16E+02	0.507	SIGNALING BY GPCR
NPW	3	7.17E+02	0.508	SIGNALING BY GPCR
NPSR1	4	3.46E+03	0.509	SIGNALING BY GPCR
UTS2	2	3.07E+03	0.510	SIGNALING BY GPCR
CMKLR1	5	8.10E+03	0.511	SIGNALING BY GPCR

OR9I1	7	6.83E+03	0.514	SIGNALING BY GPCR
NMU	3	2.95E+03	0.514	SIGNALING BY GPCR
OR10J3	1	6.74E+01	0.514	SIGNALING BY GPCR
OR4C5	7	2.55E+04	0.517	SIGNALING BY GPCR
OR1F1	8	4.28E+03	0.519	SIGNALING BY GPCR
ADCY5	11	4.50E+03	0.520	SIGNALING BY GPCR
GRM3	4	3.25E+03	0.520	SIGNALING BY GPCR
OR8H2	3	3.99E+03	0.522	SIGNALING BY GPCR
OR5B17	5	4.17E+03	0.523	SIGNALING BY GPCR
GHRL	3	4.65E+03	0.524	SIGNALING BY GPCR
PLPPR3	10	7.52E+03	0.526	SIGNALING BY GPCR
LTB4R2	4	1.01E+03	0.527	SIGNALING BY GPCR
FFAR1	3	1.69E+03	0.530	SIGNALING BY GPCR
FPR2	3	1.80E+03	0.531	SIGNALING BY GPCR
OR6C65	1	1.41E+02	0.531	SIGNALING BY GPCR
ITSN1	5	1.89E+03	0.533	SIGNALING BY GPCR
F2RL2	4	8.44E+03	0.534	SIGNALING BY GPCR
CCL13	2	3.88E+02	0.535	SIGNALING BY GPCR
S1PR1	2	2.46E+03	0.537	SIGNALING BY GPCR
PMCH	1	9.44E+01	0.539	SIGNALING BY GPCR
GNAO1	1	9.57E+01	0.539	SIGNALING BY GPCR
OR2S2	4	3.07E+03	0.539	SIGNALING BY GPCR
OR10V1	4	4.49E+03	0.539	SIGNALING BY GPCR
OR5V1	5	9.24E+03	0.539	SIGNALING BY GPCR
WNT4	1	9.13E+01	0.540	SIGNALING BY GPCR
OR6A2	6	2.75E+03	0.540	SIGNALING BY GPCR
OR10G6	5	4.34E+03	0.540	SIGNALING BY GPCR
PDE7B	1	9.04E+01	0.541	SIGNALING BY GPCR
FFAR2	6	2.79E+03	0.541	SIGNALING BY GPCR
OR51B6	3	2.87E+03	0.542	SIGNALING BY GPCR
NPY5R	1	7.21E+01	0.542	SIGNALING BY GPCR
ADCYAP1R1	8	3.48E+03	0.544	SIGNALING BY GPCR
PLCB4	7	4.27E+03	0.544	SIGNALING BY GPCR
RGS22	15	8.88E+03	0.545	SIGNALING BY GPCR
GPR25	6	1.04E+04	0.546	SIGNALING BY GPCR
OR2M7	3	3.14E+03	0.549	SIGNALING BY GPCR
UCN	1	2.24E+02	0.549	SIGNALING BY GPCR
TACR3	4	3.29E+03	0.549	SIGNALING BY GPCR
RPE65	3	1.39E+03	0.549	SIGNALING BY GPCR
TAS2R42	1	8.11E+01	0.550	SIGNALING BY GPCR
PRLH	2	7.62E+02	0.552	SIGNALING BY GPCR
CCKBR	2	5.36E+02	0.553	SIGNALING BY GPCR
PRKCA	2	2.58E+03	0.553	SIGNALING BY GPCR
GPR35	9	1.59E+04	0.553	SIGNALING BY GPCR
LPAR2	2	1.59E+03	0.554	SIGNALING BY GPCR
CCR1	3	1.03E+03	0.554	SIGNALING BY GPCR
LRP8	5	1.31E+03	0.555	SIGNALING BY GPCR
OR5M11	4	3.46E+03	0.555	SIGNALING BY GPCR
OR2A4	1	7.67E+01	0.556	SIGNALING BY GPCR
APOA2	1	1.53E+02	0.557	SIGNALING BY GPCR
PRKCH	5	4.63E+03	0.558	SIGNALING BY GPCR
HTR1D	5	1.45E+03	0.558	SIGNALING BY GPCR
GRK3	2	7.31E+02	0.559	SIGNALING BY GPCR
OR2L13	31	1.87E+04	0.559	SIGNALING BY GPCR
OR10G8	8	5.34E+03	0.562	SIGNALING BY GPCR
ARHGEF39	4	3.39E+03	0.563	SIGNALING BY GPCR
OR51B4	5	2.12E+03	0.563	SIGNALING BY GPCR
OR2Z1	6	1.35E+03	0.563	SIGNALING BY GPCR
MCHR2	1	1.26E+03	0.564	SIGNALING BY GPCR
GHSR	3	8.21E+02	0.565	SIGNALING BY GPCR
ADRB3	5	1.22E+03	0.565	SIGNALING BY GPCR
OR9K2	3	1.47E+03	0.566	SIGNALING BY GPCR
ADORA1	4	1.27E+03	0.566	SIGNALING BY GPCR
OR1S1	8	5.01E+03	0.566	SIGNALING BY GPCR
TAAR5	11	7.90E+03	0.567	SIGNALING BY GPCR
LRAT	2	8.27E+02	0.571	SIGNALING BY GPCR
RGS3	24	1.14E+04	0.571	SIGNALING BY GPCR
OR51D1	4	1.82E+03	0.571	SIGNALING BY GPCR
GNAT1	2	3.05E+02	0.572	SIGNALING BY GPCR
OR8B2	4	3.99E+03	0.572	SIGNALING BY GPCR
MGLL	4	2.44E+03	0.572	SIGNALING BY GPCR
OR4L1	6	5.15E+03	0.573	SIGNALING BY GPCR
OR56B1	3	8.91E+02	0.574	SIGNALING BY GPCR
ARHGEF18	28	2.48E+04	0.576	SIGNALING BY GPCR

OR2AG2	4	1.31E+03	0.578	SIGNALING BY GPCR
ADORA2A	4	7.09E+02	0.579	SIGNALING BY GPCR
TAS2R31	9	2.08E+04	0.579	SIGNALING BY GPCR
ARHGEF10L	16	7.68E+03	0.581	SIGNALING BY GPCR
EDN2	2	8.08E+02	0.582	SIGNALING BY GPCR
TAAR6	8	6.24E+03	0.583	SIGNALING BY GPCR
VAV2	10	5.39E+03	0.583	SIGNALING BY GPCR
HCRTR1	6	2.57E+03	0.584	SIGNALING BY GPCR
OR10Q1	4	2.47E+03	0.586	SIGNALING BY GPCR
ADRA1A	11	1.61E+04	0.586	SIGNALING BY GPCR
PRKAR1A	10	4.10E+03	0.586	SIGNALING BY GPCR
DGKE	5	2.87E+03	0.587	SIGNALING BY GPCR
OPRD1	2	4.06E+02	0.587	SIGNALING BY GPCR
OR9A4	2	4.51E+02	0.588	SIGNALING BY GPCR
OR2AP1	6	1.37E+03	0.588	SIGNALING BY GPCR
GPR68	3	6.43E+02	0.588	SIGNALING BY GPCR
OR7G2	4	6.27E+02	0.589	SIGNALING BY GPCR
KRAS	2	3.83E+02	0.589	SIGNALING BY GPCR
SCTR	6	6.15E+03	0.589	SIGNALING BY GPCR
OR5AP2	3	2.72E+03	0.590	SIGNALING BY GPCR
ADCY8	7	5.47E+03	0.591	SIGNALING BY GPCR
TRH	1	4.51E+02	0.591	SIGNALING BY GPCR
FPR1	5	2.74E+03	0.591	SIGNALING BY GPCR
GRK7	9	7.81E+03	0.591	SIGNALING BY GPCR
OR12D2	7	5.65E+03	0.594	SIGNALING BY GPCR
OR10H3	2	6.63E+02	0.596	SIGNALING BY GPCR
RGS9BP	3	2.62E+03	0.596	SIGNALING BY GPCR
SAA1	6	3.52E+03	0.597	SIGNALING BY GPCR
CRHR1	4	7.95E+03	0.597	SIGNALING BY GPCR
ADM	2	1.17E+03	0.598	SIGNALING BY GPCR
RLBP1	4	6.73E+02	0.598	SIGNALING BY GPCR
PDE7A	3	6.45E+02	0.599	SIGNALING BY GPCR
NET1	8	1.63E+03	0.601	SIGNALING BY GPCR
OR6X1	3	2.33E+03	0.602	SIGNALING BY GPCR
P2RY13	2	3.58E+03	0.602	SIGNALING BY GPCR
GPSM1	5	2.29E+03	0.602	SIGNALING BY GPCR
ABR	12	1.25E+04	0.602	SIGNALING BY GPCR
RGS9	6	6.67E+03	0.605	SIGNALING BY GPCR
OR10W1	7	1.02E+04	0.607	SIGNALING BY GPCR
KALRN	27	2.42E+04	0.608	SIGNALING BY GPCR
PROK1	1	3.60E+01	0.608	SIGNALING BY GPCR
P2RY11	9	8.60E+03	0.608	SIGNALING BY GPCR
OR7D4	3	1.16E+03	0.609	SIGNALING BY GPCR
OR7A10	7	1.99E+03	0.609	SIGNALING BY GPCR
OR8B12	6	2.08E+03	0.610	SIGNALING BY GPCR
PNOC	2	2.92E+02	0.610	SIGNALING BY GPCR
OR13C5	12	5.92E+03	0.610	SIGNALING BY GPCR
OR8U1	8	5.76E+03	0.610	SIGNALING BY GPCR
PLCB3	7	6.74E+03	0.611	SIGNALING BY GPCR
ARRB2	3	1.62E+03	0.616	SIGNALING BY GPCR
TAAR1	1	5.50E+01	0.616	SIGNALING BY GPCR
TAS2R40	4	7.39E+02	0.618	SIGNALING BY GPCR
DGKD	7	1.14E+03	0.618	SIGNALING BY GPCR
PDE8B	3	1.93E+03	0.618	SIGNALING BY GPCR
RGS16	3	2.35E+03	0.622	SIGNALING BY GPCR
ITPR3	15	4.94E+03	0.623	SIGNALING BY GPCR
AKT2	3	4.37E+02	0.624	SIGNALING BY GPCR
RASGRP1	2	2.64E+02	0.624	SIGNALING BY GPCR
GPR84	6	2.07E+03	0.625	SIGNALING BY GPCR
NPFF	2	4.01E+02	0.626	SIGNALING BY GPCR
GNG12	2	4.72E+02	0.627	SIGNALING BY GPCR
OR9G1	2	7.89E+02	0.628	SIGNALING BY GPCR
OR10G2	4	2.44E+03	0.628	SIGNALING BY GPCR
BDKRB1	6	2.20E+03	0.629	SIGNALING BY GPCR
GNB5	1	3.69E+02	0.629	SIGNALING BY GPCR
GLP2R	5	3.84E+03	0.631	SIGNALING BY GPCR
PREX1	9	5.11E+03	0.632	SIGNALING BY GPCR
FZD5	3	1.22E+03	0.633	SIGNALING BY GPCR
AKT1	2	2.60E+02	0.633	SIGNALING BY GPCR
GALR3	11	8.22E+03	0.634	SIGNALING BY GPCR
PPP1R1B	4	5.23E+03	0.634	SIGNALING BY GPCR
KISS1R	2	5.28E+02	0.635	SIGNALING BY GPCR
GABBR2	1	5.62E+01	0.635	SIGNALING BY GPCR
OR4C12	2	4.91E+02	0.636	SIGNALING BY GPCR

PTHLH	1	4.74E+01	0.637	SIGNALING BY GPCR
OR8J3	2	4.83E+02	0.638	SIGNALING BY GPCR
OR4C3	4	3.11E+02	0.638	SIGNALING BY GPCR
OR3A1	7	3.72E+03	0.638	SIGNALING BY GPCR
OR1M1	9	2.85E+03	0.640	SIGNALING BY GPCR
OR56B4	2	1.27E+03	0.641	SIGNALING BY GPCR
OR2T6	5	5.26E+03	0.641	SIGNALING BY GPCR
OR52I1	10	6.64E+03	0.641	SIGNALING BY GPCR
OR52L1	3	1.81E+03	0.645	SIGNALING BY GPCR
OR10S1	6	3.94E+03	0.645	SIGNALING BY GPCR
SCT	2	4.99E+02	0.646	SIGNALING BY GPCR
TAS2R19	5	7.01E+03	0.647	SIGNALING BY GPCR
PRLHR	3	3.97E+02	0.648	SIGNALING BY GPCR
GUCA1C	3	5.74E+02	0.649	SIGNALING BY GPCR
TAAR2	4	5.91E+02	0.650	SIGNALING BY GPCR
OR9Q1	15	9.51E+03	0.650	SIGNALING BY GPCR
OR1E1	3	2.54E+03	0.651	SIGNALING BY GPCR
PDE1B	4	1.13E+03	0.652	SIGNALING BY GPCR
TAS2R1	2	7.80E+02	0.653	SIGNALING BY GPCR
CCKAR	3	1.40E+03	0.653	SIGNALING BY GPCR
OR2T12	16	1.99E+04	0.654	SIGNALING BY GPCR
CASR	6	2.50E+03	0.656	SIGNALING BY GPCR
PRKCB	4	1.14E+03	0.657	SIGNALING BY GPCR
OR2F2	2	1.37E+03	0.658	SIGNALING BY GPCR
GRK6	5	1.20E+03	0.658	SIGNALING BY GPCR
OPN5	2	3.82E+02	0.658	SIGNALING BY GPCR
OR8K3	5	1.88E+03	0.661	SIGNALING BY GPCR
MTNR1A	7	5.22E+03	0.662	SIGNALING BY GPCR
P2RY12	2	2.55E+02	0.662	SIGNALING BY GPCR
OR10H1	3	1.30E+03	0.663	SIGNALING BY GPCR
GRM8	3	4.28E+02	0.664	SIGNALING BY GPCR
OR6C4	5	3.23E+03	0.664	SIGNALING BY GPCR
HCAR3	7	1.20E+03	0.665	SIGNALING BY GPCR
OPRL1	12	7.90E+03	0.665	SIGNALING BY GPCR
OR10C1	18	5.64E+04	0.665	SIGNALING BY GPCR
OR11H2	14	1.69E+04	0.666	SIGNALING BY GPCR
NPFFR2	4	6.97E+02	0.666	SIGNALING BY GPCR
OR4E1	4	2.47E+03	0.667	SIGNALING BY GPCR
PIK3R5	11	3.13E+03	0.668	SIGNALING BY GPCR
PTCH2	8	6.06E+03	0.668	SIGNALING BY GPCR
HCRT	1	2.83E+01	0.670	SIGNALING BY GPCR
ROCK2	8	3.46E+03	0.671	SIGNALING BY GPCR
FPR3	1	7.26E+01	0.671	SIGNALING BY GPCR
RDH12	3	4.19E+02	0.672	SIGNALING BY GPCR
OR52N1	1	3.48E+01	0.673	SIGNALING BY GPCR
OR8K5	5	1.54E+03	0.674	SIGNALING BY GPCR
OR1E2	2	3.85E+02	0.678	SIGNALING BY GPCR
GNAI2	2	6.18E+02	0.679	SIGNALING BY GPCR
OR5B21	5	1.02E+03	0.680	SIGNALING BY GPCR
CREB1	1	5.55E+01	0.681	SIGNALING BY GPCR
S1PR3	8	6.21E+03	0.682	SIGNALING BY GPCR
MCHR1	7	6.51E+03	0.683	SIGNALING BY GPCR
OR51G2	5	1.25E+03	0.684	SIGNALING BY GPCR
CXCL16	5	1.84E+03	0.684	SIGNALING BY GPCR
VAV1	3	1.50E+03	0.684	SIGNALING BY GPCR
OR6C68	5	1.94E+03	0.685	SIGNALING BY GPCR
OR2B11	4	1.71E+03	0.685	SIGNALING BY GPCR
FZD10	4	5.13E+02	0.686	SIGNALING BY GPCR
ARHGEF25	8	1.10E+03	0.687	SIGNALING BY GPCR
FZD6	4	1.31E+03	0.687	SIGNALING BY GPCR
OR52A1	3	1.98E+03	0.687	SIGNALING BY GPCR
OR4C6	8	2.31E+03	0.688	SIGNALING BY GPCR
CCL28	1	5.41E+01	0.688	SIGNALING BY GPCR
ARHGEF7	4	1.79E+03	0.689	SIGNALING BY GPCR
P2RY14	2	2.63E+02	0.689	SIGNALING BY GPCR
F2RL3	9	2.92E+03	0.689	SIGNALING BY GPCR
OR5D14	4	1.07E+03	0.689	SIGNALING BY GPCR
OR1L8	8	2.13E+03	0.689	SIGNALING BY GPCR
CCR5	10	4.76E+03	0.692	SIGNALING BY GPCR
RLN3	1	2.75E+02	0.693	SIGNALING BY GPCR
C3	10	2.84E+03	0.694	SIGNALING BY GPCR
OR52K2	8	7.60E+03	0.694	SIGNALING BY GPCR
CAMK2D	3	9.19E+02	0.695	SIGNALING BY GPCR
NPBWR2	7	2.09E+03	0.695	SIGNALING BY GPCR

OR5B3	4	1.97E+03	0.696	SIGNALING BY GPCR
GRK5	6	2.80E+03	0.696	SIGNALING BY GPCR
NPY1R	4	1.41E+03	0.698	SIGNALING BY GPCR
CCR8	2	5.78E+02	0.698	SIGNALING BY GPCR
TAS2R9	5	2.99E+03	0.701	SIGNALING BY GPCR
PPP2R1B	5	2.89E+03	0.702	SIGNALING BY GPCR
DAGLB	12	1.04E+04	0.703	SIGNALING BY GPCR
TAS2R4	4	9.02E+02	0.703	SIGNALING BY GPCR
HTR4	4	9.27E+02	0.703	SIGNALING BY GPCR
OR7E24	5	1.53E+03	0.705	SIGNALING BY GPCR
CCR10	1	1.64E+02	0.705	SIGNALING BY GPCR
EDN1	7	9.31E+02	0.706	SIGNALING BY GPCR
SDC3	5	2.33E+03	0.706	SIGNALING BY GPCR
PDE4A	6	1.16E+03	0.707	SIGNALING BY GPCR
PLXNB1	14	5.74E+03	0.709	SIGNALING BY GPCR
ADGRE1	15	1.47E+04	0.709	SIGNALING BY GPCR
MLN	4	7.34E+02	0.710	SIGNALING BY GPCR
UTS2R	5	2.45E+03	0.712	SIGNALING BY GPCR
OR4F4	4	1.89E+03	0.713	SIGNALING BY GPCR
OR2T35	1	4.10E+01	0.713	SIGNALING BY GPCR
HTR7	3	2.25E+03	0.713	SIGNALING BY GPCR
OR51B2	3	2.60E+02	0.714	SIGNALING BY GPCR
CALCA	3	5.33E+03	0.716	SIGNALING BY GPCR
ADORA3	3	1.40E+03	0.716	SIGNALING BY GPCR
GRM1	9	1.10E+04	0.716	SIGNALING BY GPCR
CCL4	3	1.12E+03	0.717	SIGNALING BY GPCR
TAS2R20	1	2.15E+01	0.717	SIGNALING BY GPCR
XCR1	2	6.45E+02	0.718	SIGNALING BY GPCR
OR51S1	4	2.83E+03	0.718	SIGNALING BY GPCR
GNB2	1	3.27E+01	0.721	SIGNALING BY GPCR
CXCR2	5	9.70E+02	0.723	SIGNALING BY GPCR
PDE3A	6	2.14E+03	0.723	SIGNALING BY GPCR
RGS14	2	2.20E+02	0.723	SIGNALING BY GPCR
CXCL10	1	3.16E+02	0.723	SIGNALING BY GPCR
OR5H2	3	2.08E+03	0.724	SIGNALING BY GPCR
TTR	1	1.45E+02	0.724	SIGNALING BY GPCR
CYP4V2	6	1.06E+03	0.727	SIGNALING BY GPCR
VAV3	11	3.52E+03	0.728	SIGNALING BY GPCR
OR11H6	4	2.54E+03	0.729	SIGNALING BY GPCR
SSTR2	2	1.37E+02	0.730	SIGNALING BY GPCR
OR6S1	3	3.18E+03	0.731	SIGNALING BY GPCR
OR10G4	12	7.39E+03	0.732	SIGNALING BY GPCR
NTSR1	15	7.46E+03	0.732	SIGNALING BY GPCR
OR52E2	4	2.37E+03	0.732	SIGNALING BY GPCR
MMP3	3	2.63E+02	0.734	SIGNALING BY GPCR
OR8B4	2	9.72E+02	0.737	SIGNALING BY GPCR
OR2F1	6	3.08E+03	0.738	SIGNALING BY GPCR
OR10A5	3	9.74E+02	0.739	SIGNALING BY GPCR
PYY	1	3.07E+01	0.741	SIGNALING BY GPCR
PRKACG	1	3.70E+01	0.742	SIGNALING BY GPCR
OR4D9	7	3.63E+03	0.743	SIGNALING BY GPCR
RGS11	9	5.23E+03	0.743	SIGNALING BY GPCR
GRM2	10	2.77E+03	0.744	SIGNALING BY GPCR
HCAR2	4	7.46E+02	0.746	SIGNALING BY GPCR
EDNRB	4	4.16E+03	0.746	SIGNALING BY GPCR
RGS5	3	4.25E+02	0.747	SIGNALING BY GPCR
RDH8	5	2.02E+03	0.748	SIGNALING BY GPCR
PPP2R1A	1	1.55E+02	0.748	SIGNALING BY GPCR
RASGRP2	4	6.70E+02	0.753	SIGNALING BY GPCR
OR2M2	6	1.85E+03	0.753	SIGNALING BY GPCR
GAL	2	9.09E+02	0.754	SIGNALING BY GPCR
OR4C46	6	6.25E+03	0.757	SIGNALING BY GPCR
OR6M1	3	1.56E+03	0.758	SIGNALING BY GPCR
OR5AS1	5	2.04E+03	0.761	SIGNALING BY GPCR
LPAR6	3	1.01E+03	0.761	SIGNALING BY GPCR
OR2H2	5	2.17E+03	0.761	SIGNALING BY GPCR
FZD8	4	4.99E+02	0.762	SIGNALING BY GPCR
CLPS	3	2.40E+03	0.764	SIGNALING BY GPCR
PNLIP	2	8.11E+01	0.766	SIGNALING BY GPCR
RGS8	1	2.17E+01	0.767	SIGNALING BY GPCR
PLB1	28	1.71E+04	0.768	SIGNALING BY GPCR
REEP6	3	3.03E+02	0.770	SIGNALING BY GPCR
GRK2	1	1.91E+01	0.770	SIGNALING BY GPCR
OPN3	12	3.26E+03	0.770	SIGNALING BY GPCR

ARHGEF19	11	4.36E+03	0.772	SIGNALING BY GPCR
PDPK1	1	2.60E+01	0.773	SIGNALING BY GPCR
TAS2R41	4	5.80E+02	0.773	SIGNALING BY GPCR
TAS1R2	10	2.99E+03	0.773	SIGNALING BY GPCR
NTSR2	7	2.75E+03	0.775	SIGNALING BY GPCR
OR56A3	4	3.64E+02	0.777	SIGNALING BY GPCR
WNT2B	6	1.22E+03	0.777	SIGNALING BY GPCR
OR4F6	4	1.83E+03	0.780	SIGNALING BY GPCR
OR6Q1	2	1.05E+03	0.780	SIGNALING BY GPCR
LHB	3	1.75E+03	0.780	SIGNALING BY GPCR
OR8G1	3	2.80E+02	0.780	SIGNALING BY GPCR
PRKACA	4	1.16E+03	0.781	SIGNALING BY GPCR
FZD2	4	1.70E+03	0.783	SIGNALING BY GPCR
OR4D5	6	1.25E+03	0.783	SIGNALING BY GPCR
GNAL	2	2.91E+02	0.784	SIGNALING BY GPCR
TRPC6	3	2.16E+02	0.784	SIGNALING BY GPCR
OR5A2	1	1.35E+02	0.785	SIGNALING BY GPCR
OR52Z1	11	7.37E+03	0.785	SIGNALING BY GPCR
VIPR1	6	9.78E+02	0.785	SIGNALING BY GPCR
GPC5	6	5.78E+02	0.787	SIGNALING BY GPCR
ADORA2B	2	8.40E+01	0.789	SIGNALING BY GPCR
RXFP1	3	2.71E+02	0.792	SIGNALING BY GPCR
DHRS9	3	5.98E+02	0.792	SIGNALING BY GPCR
ARHGEF10	13	5.70E+03	0.792	SIGNALING BY GPCR
OR6N1	4	7.18E+03	0.794	SIGNALING BY GPCR
ARHGEF11	13	5.95E+03	0.794	SIGNALING BY GPCR
CX3CR1	7	2.48E+03	0.795	SIGNALING BY GPCR
OR6B3	7	3.57E+03	0.796	SIGNALING BY GPCR
CXCL12	2	1.32E+02	0.797	SIGNALING BY GPCR
WNT9B	4	6.78E+02	0.797	SIGNALING BY GPCR
LDLR	6	1.07E+03	0.798	SIGNALING BY GPCR
GPR150	4	3.15E+02	0.800	SIGNALING BY GPCR
OR2G3	7	1.21E+03	0.800	SIGNALING BY GPCR
APOM	2	5.64E+02	0.800	SIGNALING BY GPCR
OR6B1	8	1.30E+03	0.802	SIGNALING BY GPCR
OR5M1	6	1.30E+03	0.804	SIGNALING BY GPCR
GNAT3	4	1.26E+03	0.804	SIGNALING BY GPCR
PDYN	5	1.17E+03	0.806	SIGNALING BY GPCR
OR2AG1	3	3.85E+02	0.806	SIGNALING BY GPCR
PDE3B	10	2.26E+03	0.807	SIGNALING BY GPCR
ARHGEF1	3	3.07E+02	0.808	SIGNALING BY GPCR
LRP12	6	9.60E+02	0.808	SIGNALING BY GPCR
GNA12	4	4.30E+03	0.809	SIGNALING BY GPCR
HCRTR2	4	1.35E+03	0.809	SIGNALING BY GPCR
OR1G1	2	2.31E+02	0.809	SIGNALING BY GPCR
PTGDR2	4	1.53E+02	0.809	SIGNALING BY GPCR
OR2B2	1	2.16E+02	0.809	SIGNALING BY GPCR
SHH	2	1.20E+02	0.810	SIGNALING BY GPCR
RGS2	4	8.86E+02	0.811	SIGNALING BY GPCR
OR4C15	7	2.26E+03	0.812	SIGNALING BY GPCR
RBP3	13	1.99E+03	0.812	SIGNALING BY GPCR
GPSM2	3	8.14E+02	0.813	SIGNALING BY GPCR
OR5B12	10	1.90E+03	0.813	SIGNALING BY GPCR
OR2A7	9	2.77E+03	0.814	SIGNALING BY GPCR
RTP2	3	1.49E+02	0.815	SIGNALING BY GPCR
AGTR1	4	5.93E+02	0.816	SIGNALING BY GPCR
PDE4B	1	2.08E+02	0.818	SIGNALING BY GPCR
NPY4R	6	8.62E+02	0.818	SIGNALING BY GPCR
OXER1	9	7.23E+03	0.819	SIGNALING BY GPCR
RXFP2	8	4.25E+03	0.819	SIGNALING BY GPCR
OR1A1	3	2.62E+02	0.820	SIGNALING BY GPCR
PDE6A	13	2.08E+03	0.820	SIGNALING BY GPCR
OR51H1	4	3.22E+02	0.820	SIGNALING BY GPCR
RHO	1	1.37E+01	0.821	SIGNALING BY GPCR
APOE	4	4.35E+02	0.821	SIGNALING BY GPCR
ARHGEF12	7	3.74E+03	0.821	SIGNALING BY GPCR
ADGRE2	14	7.81E+03	0.823	SIGNALING BY GPCR
OR13G1	4	8.32E+02	0.824	SIGNALING BY GPCR
OR4F15	5	3.05E+03	0.825	SIGNALING BY GPCR
OR2L5	7	1.61E+03	0.826	SIGNALING BY GPCR
OR12D3	6	2.67E+02	0.830	SIGNALING BY GPCR
OR5K2	5	1.70E+03	0.830	SIGNALING BY GPCR
AGT	12	1.42E+03	0.831	SIGNALING BY GPCR
OXTR	5	1.39E+03	0.831	SIGNALING BY GPCR

OR11G2	5	3.48E+02	0.832	SIGNALING BY GPCR
TAS2R60	2	3.80E+02	0.833	SIGNALING BY GPCR
PDE1C	7	2.41E+03	0.833	SIGNALING BY GPCR
GLP1R	8	1.62E+03	0.834	SIGNALING BY GPCR
OR4D10	4	7.66E+02	0.837	SIGNALING BY GPCR
DGKI	5	5.60E+02	0.837	SIGNALING BY GPCR
FNTB	3	1.89E+02	0.838	SIGNALING BY GPCR
METAP2	1	8.95E+00	0.838	SIGNALING BY GPCR
OR6T1	8	4.06E+03	0.838	SIGNALING BY GPCR
RGS20	5	8.07E+02	0.840	SIGNALING BY GPCR
NAPEPLD	2	4.20E+02	0.841	SIGNALING BY GPCR
BCO2	9	1.92E+03	0.841	SIGNALING BY GPCR
OR8A1	4	1.88E+02	0.841	SIGNALING BY GPCR
HTR2A	4	1.62E+03	0.843	SIGNALING BY GPCR
RGR	5	3.68E+02	0.844	SIGNALING BY GPCR
GUCY2D	10	2.53E+03	0.844	SIGNALING BY GPCR
ARHGEF2	1	9.02E+00	0.845	SIGNALING BY GPCR
NMT2	4	5.68E+02	0.847	SIGNALING BY GPCR
SSTR1	1	5.80E+00	0.848	SIGNALING BY GPCR
TAS2R5	7	2.55E+03	0.849	SIGNALING BY GPCR
GNRH2	3	4.53E+02	0.850	SIGNALING BY GPCR
AGRN	42	1.24E+04	0.851	SIGNALING BY GPCR
OR10P1	3	2.01E+02	0.851	SIGNALING BY GPCR
RPS6KA2	3	6.28E+02	0.851	SIGNALING BY GPCR
ECE1	3	6.76E+02	0.852	SIGNALING BY GPCR
EDN3	1	7.52E+00	0.854	SIGNALING BY GPCR
OR52H1	4	4.15E+03	0.856	SIGNALING BY GPCR
HRH1	6	6.30E+02	0.857	SIGNALING BY GPCR
HTR1F	1	4.98E+01	0.857	SIGNALING BY GPCR
RTP5	12	3.64E+03	0.857	SIGNALING BY GPCR
HSD17B6	4	2.70E+02	0.858	SIGNALING BY GPCR
OR4D11	1	5.81E+00	0.859	SIGNALING BY GPCR
OR7A17	4	9.63E+02	0.860	SIGNALING BY GPCR
OR7D2	2	4.06E+01	0.862	SIGNALING BY GPCR
FZD1	3	2.57E+02	0.863	SIGNALING BY GPCR
OR5H14	17	3.63E+03	0.864	SIGNALING BY GPCR
CALCRL	1	2.34E+01	0.864	SIGNALING BY GPCR
RHOC	1	1.42E+01	0.868	SIGNALING BY GPCR
OR2H1	13	1.94E+03	0.868	SIGNALING BY GPCR
GNG13	1	3.52E+00	0.869	SIGNALING BY GPCR
GPR65	1	6.27E+00	0.870	SIGNALING BY GPCR
PTGER2	2	1.14E+03	0.871	SIGNALING BY GPCR
OR10H2	6	3.67E+02	0.872	SIGNALING BY GPCR
OR5AK2	1	6.11E+00	0.873	SIGNALING BY GPCR
AVPR1A	4	1.37E+03	0.875	SIGNALING BY GPCR
OR51F2	5	4.28E+02	0.875	SIGNALING BY GPCR
ADCY1	6	8.93E+02	0.875	SIGNALING BY GPCR
GNB4	1	2.78E+01	0.877	SIGNALING BY GPCR
MTNR1B	6	4.84E+02	0.878	SIGNALING BY GPCR
OR4A16	3	1.70E+02	0.883	SIGNALING BY GPCR
OR52M1	5	2.28E+02	0.884	SIGNALING BY GPCR
OR4K1	6	9.51E+02	0.884	SIGNALING BY GPCR
CHRM5	6	2.85E+02	0.885	SIGNALING BY GPCR
OR51V1	10	6.66E+02	0.887	SIGNALING BY GPCR
CCL4L2	5	1.74E+03	0.890	SIGNALING BY GPCR
MC1R	17	2.05E+03	0.891	SIGNALING BY GPCR
GNG2	1	3.16E+00	0.894	SIGNALING BY GPCR
TSHR	5	4.59E+02	0.895	SIGNALING BY GPCR
INSL3	6	1.27E+03	0.899	SIGNALING BY GPCR
OR51A2	1	2.19E+00	0.900	SIGNALING BY GPCR
PTGER3	1	1.97E+01	0.901	SIGNALING BY GPCR
GRK1	9	1.78E+03	0.903	SIGNALING BY GPCR
CD55	5	1.70E+02	0.906	SIGNALING BY GPCR
HTR1E	1	7.94E+00	0.909	SIGNALING BY GPCR
OR3A2	10	1.09E+03	0.910	SIGNALING BY GPCR
TAS2R8	7	9.41E+02	0.911	SIGNALING BY GPCR
CHRM4	1	2.02E+00	0.914	SIGNALING BY GPCR
TRIO	15	4.56E+03	0.925	SIGNALING BY GPCR
AVP	1	7.11E-01	0.976	SIGNALING BY GPCR
ARHGEF35	1	3.27E-01	0.978	SIGNALING BY GPCR
OR13A1	1	1.40E+00	0.978	SIGNALING BY GPCR
DRD3	1	1.26E-01	0.982	SIGNALING BY GPCR
ADRA2C	1	5.32E-02	0.983	SIGNALING BY GPCR
WNT6	1	1.78E-01	0.987	SIGNALING BY GPCR

PTGER4	1	2.47E-01	0.993	SIGNALING BY GPCR
METAP1	1	7.27E-05	0.999652	SIGNALING BY GPCR
RCVRN	1	6.78E-06	0.999889	SIGNALING BY GPCR
FGD4	8	3.42E+03	1	SIGNALING BY GPCR
OR5F1	10	5.21E+03	1	SIGNALING BY GPCR
CHRM3	3	2.38E+02	1	SIGNALING BY GPCR
CAMKK2	3	1.82E+02	1	SIGNALING BY GPCR
C5AR2	2	3.91E+01	1	SIGNALING BY GPCR
GNAI1	2	3.43E+01	1	SIGNALING BY GPCR
OR1A2	2	2.06E+01	1	SIGNALING BY GPCR
PRKAR2A	3	1.38E+01	1	SIGNALING BY GPCR
TAS2R3	2	5.83E+01	1	SIGNALING BY GPCR
OR10A4	6	9.97E+01	1	SIGNALING BY GPCR
HTR6	6	5.49E+01	1	SIGNALING BY GPCR
OR2M4	3	1.60E+02	1	SIGNALING BY GPCR
DGKA	5	2.23E+02	1	SIGNALING BY GPCR
PDE6B	11	2.77E+02	1	SIGNALING BY GPCR
OBSCN	153	2.21E+04	1	SIGNALING BY GPCR
OR2T34	2	8.82E+01	1	SIGNALING BY GPCR
OXGR1	2	1.50E+02	1	SIGNALING BY GPCR
PTH2R	7	1.16E+02	1	SIGNALING BY GPCR
P2RY2	6	1.00E+01	1	SIGNALING BY GPCR
HSD17B1	4	1.43E+02	1	SIGNALING BY GPCR
ABHD6	4	4.47E+02	1	SIGNALING BY GPCR
SAG	4	2.27E+01	1	SIGNALING BY GPCR
OR13C9	3	5.02E+00	1	SIGNALING BY GPCR
AKAP13	28	2.50E+03	1	SIGNALING BY GPCR
PDE11A	13	7.28E+03	1	SIGNALING BY GPCR
PRKAR2B	2	5.58E+01	1	SIGNALING BY GPCR
HRAS	3	3.32E+00	1	SIGNALING BY GPCR
NMUR1	8	8.72E+01	1	SIGNALING BY GPCR
OR2B6	3	9.22E+02	1	SIGNALING BY GPCR
OR11A1	5	9.70E+02	1	SIGNALING BY GPCR
ACKR1	5	1.40E+03	1	SIGNALING BY GPCR
SLC24A1	9	2.41E+03	1	SIGNALING BY GPCR
OR5AC2	5	5.86E+02	1	SIGNALING BY GPCR
LPAR3	2	2.37E+01	1	SIGNALING BY GPCR
WNT10A	9	2.00E+03	1	SIGNALING BY GPCR
DRD1	2	6.12E-01	1	SIGNALING BY GPCR
HRH4	2	5.02E+01	1	SIGNALING BY GPCR
RGS19	2	2.49E+02	1	SIGNALING BY GPCR
GNA15	3	1.19E+02	1	SIGNALING BY GPCR
HCAR1	4	4.89E+01	1	SIGNALING BY GPCR
CAMK4	3	4.67E+01	1	SIGNALING BY GPCR
GNA14	6	2.07E+03	1	SIGNALING BY GPCR
ADGRE3	8	4.95E+02	1	SIGNALING BY GPCR
GPSM3	2	2.55E+02	1	SIGNALING BY GPCR
GNGT1	2	9.38E-01	1	SIGNALING BY GPCR
OR51E1	6	1.85E+03	1	SIGNALING BY GPCR
WNT1	3	7.98E+01	1	SIGNALING BY GPCR
TRPC3	4	3.31E+01	1	SIGNALING BY GPCR
XCL2	3	6.32E+00	1	SIGNALING BY GPCR
PPY	2	8.53E+00	1	SIGNALING BY GPCR
DGKG	6	3.85E+01	1	SIGNALING BY GPCR
WNT16	2	4.00E+01	1	SIGNALING BY GPCR
ADRA2B	3	7.47E+01	1	SIGNALING BY GPCR
ECE2	4	4.03E+01	1	SIGNALING BY GPCR
GPC6	5	6.15E+00	1	SIGNALING BY GPCR
LRP1	26	1.45E+04	1	SIGNALING BY GPCR
TAS2R7	5	2.20E+03	1	SIGNALING BY GPCR
OR1L4	5	1.47E+03	1	SIGNALING BY GPCR
ADCY9	10	5.10E+03	1	SIGNALING BY GPCR
XCL1	3	4.48E+02	1	SIGNALING BY GPCR
BDKRB2	2	6.28E+00	1	SIGNALING BY GPCR
MAPK3	5	2.78E+01	1	SIGNALING BY GPCR
TAS2R10	2	2.87E+02	1	SIGNALING BY GPCR
OR10A7	4	2.06E+00	1	SIGNALING BY GPCR
GPR37L1	5	1.85E+02	1	SIGNALING BY GPCR
OR2A5	3	8.65E+00	1	SIGNALING BY GPCR
GPR15	6	6.87E+02	1	SIGNALING BY GPCR
NMS	2	1.15E+00	1	SIGNALING BY GPCR
OR5K4	7	3.43E+02	1	SIGNALING BY GPCR
OR5H15	11	1.93E+02	1	SIGNALING BY GPCR
OR2AE1	6	7.13E+02	1	SIGNALING BY GPCR

OR4A15	5	4.98E+02	1	SIGNALING BY GPCR
OR4A5	8	1.49E+01	1	SIGNALING BY GPCR
OR2B3	3	7.22E+01	1	SIGNALING BY GPCR
OR7G1	7	1.87E+03	1	SIGNALING BY GPCR
OR6Y1	13	1.49E+02	1	SIGNALING BY GPCR
OR6K6	14	2.50E+03	1	SIGNALING BY GPCR
OR6C1	3	4.28E+02	1	SIGNALING BY GPCR
OR52E8	2	8.29E+01	1	SIGNALING BY GPCR
OR51I2	5	4.31E+02	1	SIGNALING BY GPCR
OR51F1	2	3.37E+00	1	SIGNALING BY GPCR
OR5T2	2	1.19E+03	1	SIGNALING BY GPCR
OR5M9	3	5.09E+00	1	SIGNALING BY GPCR
OR5M3	6	3.08E+01	1	SIGNALING BY GPCR
OR4K17	4	6.69E+01	1	SIGNALING BY GPCR
OR10H5	5	3.10E+03	1	SIGNALING BY GPCR
OR10G7	11	1.56E+03	1	SIGNALING BY GPCR
OR10A6	2	2.07E+00	1	SIGNALING BY GPCR
OR52B2	6	1.54E+02	1	SIGNALING BY GPCR
OR8D1	4	2.83E+03	1	SIGNALING BY GPCR
OR5D13	5	5.20E+01	1	SIGNALING BY GPCR
OR14A16	4	4.56E+01	1	SIGNALING BY GPCR
OR13C3	2	3.23E+01	1	SIGNALING BY GPCR
FZD9	2	1.43E+01	1	SIGNALING BY GPCR
OR4K5	2	NA	NA	SIGNALING BY GPCR

b) LOSS OF FUNCTION VARIANTS

Gene	NumVar	Q	Pvalue	Gene-set
PRKAA1	2	45240.8	4.48E-04	ACTIVATION OF AMPK DOWNSTREAM OF NMDARS
TUBA8	1	8840.23	0.121	ACTIVATION OF AMPK DOWNSTREAM OF NMDARS
CAMKK2	5	1881.68	0.294	ACTIVATION OF AMPK DOWNSTREAM OF NMDARS
MAPT	1	208.046	0.324	ACTIVATION OF AMPK DOWNSTREAM OF NMDARS
TUBA3E	1	188.084	0.335	ACTIVATION OF AMPK DOWNSTREAM OF NMDARS
PRKAG3	2	1665.57	0.362	ACTIVATION OF AMPK DOWNSTREAM OF NMDARS
TUBB8	1	847.717	0.390	ACTIVATION OF AMPK DOWNSTREAM OF NMDARS
TUBA1B	3	1949.44	0.451	ACTIVATION OF AMPK DOWNSTREAM OF NMDARS
TUBB4A	1	460.836	0.476	ACTIVATION OF AMPK DOWNSTREAM OF NMDARS
TUBA3C	1	144.803	0.574	ACTIVATION OF AMPK DOWNSTREAM OF NMDARS
PRKAA2	2	3136.84	0.604	ACTIVATION OF AMPK DOWNSTREAM OF NMDARS
TUBB3	1	81.5899	0.632	ACTIVATION OF AMPK DOWNSTREAM OF NMDARS
TUBB1	1	14.6338	0.777	ACTIVATION OF AMPK DOWNSTREAM OF NMDARS
PRKAG2	6	2036.23	0.888	ACTIVATION OF AMPK DOWNSTREAM OF NMDARS
TUBB4B	1	5.99978	0.899	ACTIVATION OF AMPK DOWNSTREAM OF NMDARS
TUBB6	1	2.31435	0.912	ACTIVATION OF AMPK DOWNSTREAM OF NMDARS

Gene	NumVar	Q	Pvalue	Gene-set
SIGLEC11	3	27478.2	4.82E-04	ADAPTIVE IMMUNE SYSTEM
TAB2	2	12215.6	0.003	ADAPTIVE IMMUNE SYSTEM
SMURF1	10	51528	0.003	ADAPTIVE IMMUNE SYSTEM
TRIM39	3	12377.6	0.005	ADAPTIVE IMMUNE SYSTEM
KIF22	1	28811.9	0.005	ADAPTIVE IMMUNE SYSTEM
KIR2DL3	5	22698.8	0.006	ADAPTIVE IMMUNE SYSTEM
PRKN	13	856826	0.008	ADAPTIVE IMMUNE SYSTEM
LILRB3	6	60487.5	0.008	ADAPTIVE IMMUNE SYSTEM
AREL1	2	4168.91	0.010	ADAPTIVE IMMUNE SYSTEM
HLA-G	3	34362.2	0.012	ADAPTIVE IMMUNE SYSTEM
CD300LD	1	13873.4	0.012	ADAPTIVE IMMUNE SYSTEM
PSMA1	7	40662.6	0.012	ADAPTIVE IMMUNE SYSTEM
UBE3B	13	35820.3	0.014	ADAPTIVE IMMUNE SYSTEM
TAP1	3	24355	0.014	ADAPTIVE IMMUNE SYSTEM
DCTN6	2	7054.79	0.014	ADAPTIVE IMMUNE SYSTEM
UBE2D2	1	1438.9	0.017	ADAPTIVE IMMUNE SYSTEM
RASGRP2	4	5952.39	0.019	ADAPTIVE IMMUNE SYSTEM
CDH1	4	10863	0.024	ADAPTIVE IMMUNE SYSTEM
ASB2	3	10446.4	0.024	ADAPTIVE IMMUNE SYSTEM
DYNC1I2	6	12462.7	0.027	ADAPTIVE IMMUNE SYSTEM
ITGA4	4	4310.77	0.029	ADAPTIVE IMMUNE SYSTEM
PIK3R1	4	4137.19	0.030	ADAPTIVE IMMUNE SYSTEM
SEC23A	1	5107.56	0.031	ADAPTIVE IMMUNE SYSTEM
ITK	3	68614.7	0.031	ADAPTIVE IMMUNE SYSTEM
ATG7	1	1291.91	0.033	ADAPTIVE IMMUNE SYSTEM
GRB2	1	709.939	0.034	ADAPTIVE IMMUNE SYSTEM
GAN	1	3918.47	0.039	ADAPTIVE IMMUNE SYSTEM
TYROBP	1	12608.2	0.043	ADAPTIVE IMMUNE SYSTEM

LILRB4	8	19013.6	0.045	ADAPTIVE IMMUNE SYSTEM
PAK1	1	851.571	0.045	ADAPTIVE IMMUNE SYSTEM
FBXO44	4	5488.08	0.047	ADAPTIVE IMMUNE SYSTEM
KIF26A	5	32234.4	0.050	ADAPTIVE IMMUNE SYSTEM
HECTD1	5	17292.1	0.052	ADAPTIVE IMMUNE SYSTEM
TRAIP	2	7966.87	0.054	ADAPTIVE IMMUNE SYSTEM
FYN	6	20441	0.055	ADAPTIVE IMMUNE SYSTEM
TREML1	1	10266.6	0.059	ADAPTIVE IMMUNE SYSTEM
PIANP	1	527.582	0.066	ADAPTIVE IMMUNE SYSTEM
UBE2E1	2	4393.88	0.069	ADAPTIVE IMMUNE SYSTEM
CTLA4	2	2154.34	0.069	ADAPTIVE IMMUNE SYSTEM
CALR	3	1504.66	0.070	ADAPTIVE IMMUNE SYSTEM
ICAM1	1	1383.2	0.072	ADAPTIVE IMMUNE SYSTEM
UBE2W	2	13253.9	0.079	ADAPTIVE IMMUNE SYSTEM
KIR2DL1	16	36404.7	0.080	ADAPTIVE IMMUNE SYSTEM
TIRAP	2	1954.4	0.085	ADAPTIVE IMMUNE SYSTEM
PSMC2	2	17171.8	0.085	ADAPTIVE IMMUNE SYSTEM
KBTBD13	1	473.625	0.086	ADAPTIVE IMMUNE SYSTEM
HERC6	4	9940.29	0.086	ADAPTIVE IMMUNE SYSTEM
YWHAB	1	2143.69	0.087	ADAPTIVE IMMUNE SYSTEM
NFKB1	2	3793.03	0.088	ADAPTIVE IMMUNE SYSTEM
LAT	2	4609.45	0.088	ADAPTIVE IMMUNE SYSTEM
FBXO10	2	12030.5	0.091	ADAPTIVE IMMUNE SYSTEM
LY96	1	318.213	0.096	ADAPTIVE IMMUNE SYSTEM
PSMD5	3	4639.89	0.099	ADAPTIVE IMMUNE SYSTEM
UBE2C	1	627.269	0.102	ADAPTIVE IMMUNE SYSTEM
HLA-DRB1	15	51575.9	0.103	ADAPTIVE IMMUNE SYSTEM
FBXO11	5	7796.53	0.103	ADAPTIVE IMMUNE SYSTEM
UBE2D3	4	2979.44	0.104	ADAPTIVE IMMUNE SYSTEM
VCAM1	2	14695.6	0.104	ADAPTIVE IMMUNE SYSTEM
RNF25	4	2827.98	0.109	ADAPTIVE IMMUNE SYSTEM
UBE2Q2	2	10988.4	0.113	ADAPTIVE IMMUNE SYSTEM
NCF4	4	14976.1	0.116	ADAPTIVE IMMUNE SYSTEM
PRKACG	1	608.885	0.116	ADAPTIVE IMMUNE SYSTEM
BTN1A1	1	460.012	0.116	ADAPTIVE IMMUNE SYSTEM
KLRG1	1	1002.56	0.119	ADAPTIVE IMMUNE SYSTEM
TUBA8	1	8840.23	0.121	ADAPTIVE IMMUNE SYSTEM
LCK	2	6622.49	0.122	ADAPTIVE IMMUNE SYSTEM
UBE2B	1	861.479	0.126	ADAPTIVE IMMUNE SYSTEM
GLMN	4	9496.62	0.127	ADAPTIVE IMMUNE SYSTEM
KIF3C	2	1216.39	0.128	ADAPTIVE IMMUNE SYSTEM
TLR4	1	1168.91	0.129	ADAPTIVE IMMUNE SYSTEM
HLA-DQB2	6	7984.49	0.131	ADAPTIVE IMMUNE SYSTEM
CDC26	1	2078.7	0.132	ADAPTIVE IMMUNE SYSTEM
HACE1	1	395.445	0.132	ADAPTIVE IMMUNE SYSTEM
KLRC1	1	3136.67	0.133	ADAPTIVE IMMUNE SYSTEM
MLST8	1	297.162	0.134	ADAPTIVE IMMUNE SYSTEM
SEM1	1	344.268	0.138	ADAPTIVE IMMUNE SYSTEM
MKRN1	2	6173.38	0.139	ADAPTIVE IMMUNE SYSTEM
KIF23	4	3631.87	0.140	ADAPTIVE IMMUNE SYSTEM
HLA-B	1	665.838	0.142	ADAPTIVE IMMUNE SYSTEM
PSMA5	1	2697.22	0.143	ADAPTIVE IMMUNE SYSTEM
SEC61A2	1	2496.09	0.146	ADAPTIVE IMMUNE SYSTEM
PSMB10	1	762.085	0.157	ADAPTIVE IMMUNE SYSTEM
MAP3K14	2	2230.89	0.158	ADAPTIVE IMMUNE SYSTEM
PTEN	1	365.055	0.159	ADAPTIVE IMMUNE SYSTEM
VAV1	3	3589.44	0.161	ADAPTIVE IMMUNE SYSTEM
AKT1	1	1750.51	0.163	ADAPTIVE IMMUNE SYSTEM
DYNC1H1	8	6937.38	0.166	ADAPTIVE IMMUNE SYSTEM
DNM3	5	17841.2	0.166	ADAPTIVE IMMUNE SYSTEM
KLHL3	1	520.044	0.167	ADAPTIVE IMMUNE SYSTEM
ORAI1	6	36859.9	0.167	ADAPTIVE IMMUNE SYSTEM
PSMC3	1	2022.38	0.169	ADAPTIVE IMMUNE SYSTEM
HLA-DOB	1	580.545	0.173	ADAPTIVE IMMUNE SYSTEM
CD300C	1	665.811	0.178	ADAPTIVE IMMUNE SYSTEM
ASB15	7	21416.8	0.180	ADAPTIVE IMMUNE SYSTEM
FBXO4	2	1224.9	0.181	ADAPTIVE IMMUNE SYSTEM
AKT2	5	5960.06	0.181	ADAPTIVE IMMUNE SYSTEM
SIGLEC12	1	702.08	0.183	ADAPTIVE IMMUNE SYSTEM
MGRN1	7	18471.9	0.184	ADAPTIVE IMMUNE SYSTEM
EVL	1	199.743	0.187	ADAPTIVE IMMUNE SYSTEM
LILRB5	4	14237.7	0.190	ADAPTIVE IMMUNE SYSTEM
MAP3K8	1	323.456	0.198	ADAPTIVE IMMUNE SYSTEM
VHL	2	1878.41	0.200	ADAPTIVE IMMUNE SYSTEM

MRC1	2	4975.08	0.201	ADAPTIVE IMMUNE SYSTEM
FBXW11	2	5669.36	0.202	ADAPTIVE IMMUNE SYSTEM
SEC13	2	2503.14	0.202	ADAPTIVE IMMUNE SYSTEM
PTPN22	3	5735.5	0.202	ADAPTIVE IMMUNE SYSTEM
PIK3R2	3	1064.11	0.209	ADAPTIVE IMMUNE SYSTEM
BTN3A3	5	14644.8	0.211	ADAPTIVE IMMUNE SYSTEM
ZAP70	3	3537.18	0.212	ADAPTIVE IMMUNE SYSTEM
PPP2R5A	2	5291.16	0.214	ADAPTIVE IMMUNE SYSTEM
HERC5	5	13472	0.215	ADAPTIVE IMMUNE SYSTEM
LILRB2	12	16496.5	0.216	ADAPTIVE IMMUNE SYSTEM
IFI30	1	407.801	0.217	ADAPTIVE IMMUNE SYSTEM
CD74	2	2864.99	0.221	ADAPTIVE IMMUNE SYSTEM
CDC23	4	8438.17	0.223	ADAPTIVE IMMUNE SYSTEM
ASB18	1	288.439	0.223	ADAPTIVE IMMUNE SYSTEM
DYNC1I1	3	2752.7	0.224	ADAPTIVE IMMUNE SYSTEM
NPEPPS	2	9045.28	0.226	ADAPTIVE IMMUNE SYSTEM
RNF14	1	393.384	0.226	ADAPTIVE IMMUNE SYSTEM
UBA7	3	1898.47	0.227	ADAPTIVE IMMUNE SYSTEM
HECW2	7	5298.35	0.228	ADAPTIVE IMMUNE SYSTEM
DYNC1LI1	2	824.49	0.228	ADAPTIVE IMMUNE SYSTEM
HLA-A	8	10184.8	0.232	ADAPTIVE IMMUNE SYSTEM
UBE2J1	1	1008.81	0.232	ADAPTIVE IMMUNE SYSTEM
ITPR2	8	15127.6	0.233	ADAPTIVE IMMUNE SYSTEM
LILRA2	18	19164.6	0.236	ADAPTIVE IMMUNE SYSTEM
RNF213	16	16560.2	0.236	ADAPTIVE IMMUNE SYSTEM
KLRK1	2	1692.73	0.237	ADAPTIVE IMMUNE SYSTEM
UBA6	2	631.763	0.238	ADAPTIVE IMMUNE SYSTEM
CLTC	5	18429.1	0.239	ADAPTIVE IMMUNE SYSTEM
BTNL8	1	316.022	0.239	ADAPTIVE IMMUNE SYSTEM
ENAH	3	8599.74	0.240	ADAPTIVE IMMUNE SYSTEM
TREML2	1	281.059	0.247	ADAPTIVE IMMUNE SYSTEM
TREML4	1	593.905	0.248	ADAPTIVE IMMUNE SYSTEM
FCGR3A	9	22926	0.250	ADAPTIVE IMMUNE SYSTEM
KIF2C	1	262.925	0.251	ADAPTIVE IMMUNE SYSTEM
RILP	3	1930.36	0.253	ADAPTIVE IMMUNE SYSTEM
RNF41	1	493.016	0.254	ADAPTIVE IMMUNE SYSTEM
KLHL21	1	175.92	0.256	ADAPTIVE IMMUNE SYSTEM
CD36	10	17247.1	0.259	ADAPTIVE IMMUNE SYSTEM
LILRA4	4	10247.6	0.259	ADAPTIVE IMMUNE SYSTEM
SYK	1	726.573	0.266	ADAPTIVE IMMUNE SYSTEM
FBXW4	1	296.089	0.266	ADAPTIVE IMMUNE SYSTEM
PRR5	3	2097.9	0.267	ADAPTIVE IMMUNE SYSTEM
SLAMF6	1	4388.54	0.268	ADAPTIVE IMMUNE SYSTEM
PPP2R1B	3	7370.56	0.268	ADAPTIVE IMMUNE SYSTEM
VASP	3	1729.17	0.269	ADAPTIVE IMMUNE SYSTEM
PIK3CD	3	6426.86	0.270	ADAPTIVE IMMUNE SYSTEM
UBA3	2	1330.05	0.272	ADAPTIVE IMMUNE SYSTEM
CTSF	2	1025.46	0.273	ADAPTIVE IMMUNE SYSTEM
FBXO31	3	1795.23	0.278	ADAPTIVE IMMUNE SYSTEM
NCF1	2	1307.88	0.278	ADAPTIVE IMMUNE SYSTEM
RNF114	1	591.841	0.279	ADAPTIVE IMMUNE SYSTEM
BTNL2	5	15371.4	0.279	ADAPTIVE IMMUNE SYSTEM
TNFRSF14	2	522.842	0.281	ADAPTIVE IMMUNE SYSTEM
UBE4A	2	813.169	0.282	ADAPTIVE IMMUNE SYSTEM
NEDD4L	8	11382.2	0.283	ADAPTIVE IMMUNE SYSTEM
HLA-DPA1	1	1115.05	0.287	ADAPTIVE IMMUNE SYSTEM
SEC31A	3	3258.58	0.288	ADAPTIVE IMMUNE SYSTEM
PRKCQ	4	8536.51	0.293	ADAPTIVE IMMUNE SYSTEM
AKT3	1	343.971	0.293	ADAPTIVE IMMUNE SYSTEM
COL17A1	8	14222.4	0.293	ADAPTIVE IMMUNE SYSTEM
CD300E	1	573.672	0.296	ADAPTIVE IMMUNE SYSTEM
TPP2	3	1495.69	0.299	ADAPTIVE IMMUNE SYSTEM
INPP5D	3	1437.75	0.300	ADAPTIVE IMMUNE SYSTEM
KLHL20	2	2770.28	0.302	ADAPTIVE IMMUNE SYSTEM
HLA-F	1	309.909	0.302	ADAPTIVE IMMUNE SYSTEM
RAPGEF4	4	1444.81	0.303	ADAPTIVE IMMUNE SYSTEM
CAPZA1	3	9675.02	0.306	ADAPTIVE IMMUNE SYSTEM
COL1A2	6	8984.13	0.307	ADAPTIVE IMMUNE SYSTEM
PSMD6	2	870.354	0.307	ADAPTIVE IMMUNE SYSTEM
CD19	2	2253.31	0.309	ADAPTIVE IMMUNE SYSTEM
UBE2O	5	2258.89	0.313	ADAPTIVE IMMUNE SYSTEM
AP2B1	6	17982.5	0.316	ADAPTIVE IMMUNE SYSTEM
DYNC1LI2	2	1120.25	0.319	ADAPTIVE IMMUNE SYSTEM
KIF3B	1	155.997	0.322	ADAPTIVE IMMUNE SYSTEM

SEC24D	1	2512.26	0.323	ADAPTIVE IMMUNE SYSTEM
ELOB	2	951.009	0.329	ADAPTIVE IMMUNE SYSTEM
NFKBIE	1	353.203	0.329	ADAPTIVE IMMUNE SYSTEM
KIF3A	1	707.204	0.330	ADAPTIVE IMMUNE SYSTEM
RASGRP3	2	2158.27	0.331	ADAPTIVE IMMUNE SYSTEM
CD300LF	1	591.933	0.332	ADAPTIVE IMMUNE SYSTEM
PSMD8	1	169.844	0.334	ADAPTIVE IMMUNE SYSTEM
TUBA3E	1	188.084	0.335	ADAPTIVE IMMUNE SYSTEM
BTN3A2	2	793.24	0.335	ADAPTIVE IMMUNE SYSTEM
KRAS	1	2146.89	0.338	ADAPTIVE IMMUNE SYSTEM
DNM1	5	3402.03	0.344	ADAPTIVE IMMUNE SYSTEM
PRKG1	1	190.014	0.348	ADAPTIVE IMMUNE SYSTEM
HRAS	1	169.558	0.353	ADAPTIVE IMMUNE SYSTEM
AP2A1	8	7691.74	0.357	ADAPTIVE IMMUNE SYSTEM
LAG3	4	4930.02	0.357	ADAPTIVE IMMUNE SYSTEM
CD8A	4	6102.45	0.357	ADAPTIVE IMMUNE SYSTEM
PSMD1	6	6058.95	0.361	ADAPTIVE IMMUNE SYSTEM
TLR2	1	384.039	0.367	ADAPTIVE IMMUNE SYSTEM
HLA-DQA2	6	19186.6	0.375	ADAPTIVE IMMUNE SYSTEM
HERC4	2	465.964	0.375	ADAPTIVE IMMUNE SYSTEM
FBXO2	2	2609.67	0.378	ADAPTIVE IMMUNE SYSTEM
CUL1	5	3951.13	0.381	ADAPTIVE IMMUNE SYSTEM
UBB	4	3950.69	0.383	ADAPTIVE IMMUNE SYSTEM
LILRB1	13	12247.9	0.383	ADAPTIVE IMMUNE SYSTEM
PILRA	2	1101.37	0.384	ADAPTIVE IMMUNE SYSTEM
UBE3A	9	94215.4	0.386	ADAPTIVE IMMUNE SYSTEM
AP1B1	5	1827.33	0.386	ADAPTIVE IMMUNE SYSTEM
PPP3CB	1	2800.61	0.387	ADAPTIVE IMMUNE SYSTEM
SIPA1	4	7860.68	0.388	ADAPTIVE IMMUNE SYSTEM
TUBB8	1	847.717	0.390	ADAPTIVE IMMUNE SYSTEM
KLC1	4	4732.77	0.391	ADAPTIVE IMMUNE SYSTEM
CD1D	1	193.878	0.395	ADAPTIVE IMMUNE SYSTEM
RIPK2	7	17650.9	0.396	ADAPTIVE IMMUNE SYSTEM
ICAM4	2	3772.84	0.398	ADAPTIVE IMMUNE SYSTEM
BTN3A1	1	723.088	0.400	ADAPTIVE IMMUNE SYSTEM
SNAP23	1	401.739	0.402	ADAPTIVE IMMUNE SYSTEM
STIM1	3	10449.8	0.404	ADAPTIVE IMMUNE SYSTEM
PPP2R1A	2	1519.99	0.404	ADAPTIVE IMMUNE SYSTEM
NECTIN2	2	3874.93	0.409	ADAPTIVE IMMUNE SYSTEM
FBXW10	5	2904.39	0.412	ADAPTIVE IMMUNE SYSTEM
FBXW8	7	11483	0.416	ADAPTIVE IMMUNE SYSTEM
RNF220	2	2101.58	0.421	ADAPTIVE IMMUNE SYSTEM
CDC42	1	813.477	0.422	ADAPTIVE IMMUNE SYSTEM
PSMD2	2	471.053	0.422	ADAPTIVE IMMUNE SYSTEM
UBE2U	3	5599.87	0.425	ADAPTIVE IMMUNE SYSTEM
PRKACA	1	490.158	0.427	ADAPTIVE IMMUNE SYSTEM
SEC61A1	2	3154.03	0.429	ADAPTIVE IMMUNE SYSTEM
LMO7	6	4334.12	0.431	ADAPTIVE IMMUNE SYSTEM
PSMB8	1	72.0835	0.435	ADAPTIVE IMMUNE SYSTEM
HLA-DRB5	8	10538.1	0.437	ADAPTIVE IMMUNE SYSTEM
ASB13	2	696.714	0.439	ADAPTIVE IMMUNE SYSTEM
MAP3K7	1	313.961	0.442	ADAPTIVE IMMUNE SYSTEM
NRAS	1	122.852	0.443	ADAPTIVE IMMUNE SYSTEM
ICAM5	2	630.971	0.447	ADAPTIVE IMMUNE SYSTEM
CTSB	4	3057	0.447	ADAPTIVE IMMUNE SYSTEM
PSMC6	1	279.949	0.449	ADAPTIVE IMMUNE SYSTEM
AP1M1	3	938.774	0.449	ADAPTIVE IMMUNE SYSTEM
WSB1	2	2496.25	0.449	ADAPTIVE IMMUNE SYSTEM
TUBA1B	3	1949.44	0.451	ADAPTIVE IMMUNE SYSTEM
COL3A1	4	5392.94	0.456	ADAPTIVE IMMUNE SYSTEM
C3	2	683.331	0.460	ADAPTIVE IMMUNE SYSTEM
JAML	1	234.486	0.460	ADAPTIVE IMMUNE SYSTEM
UBE2S	1	86.0259	0.462	ADAPTIVE IMMUNE SYSTEM
ULBP3	3	2769.71	0.463	ADAPTIVE IMMUNE SYSTEM
FCGR1A	1	1366.08	0.463	ADAPTIVE IMMUNE SYSTEM
ASB3	2	1407.83	0.465	ADAPTIVE IMMUNE SYSTEM
PTPN6	5	3205.8	0.465	ADAPTIVE IMMUNE SYSTEM
MALT1	3	2025.31	0.466	ADAPTIVE IMMUNE SYSTEM
FBXO32	2	390.045	0.466	ADAPTIVE IMMUNE SYSTEM
TRIM36	2	2816.36	0.469	ADAPTIVE IMMUNE SYSTEM
HERC1	12	21032.6	0.469	ADAPTIVE IMMUNE SYSTEM
ITGB2	6	8130.09	0.470	ADAPTIVE IMMUNE SYSTEM
NCR3	2	428.445	0.472	ADAPTIVE IMMUNE SYSTEM
SFTPD	2	1775.08	0.474	ADAPTIVE IMMUNE SYSTEM

KLRD1	2	2189.82	0.475	ADAPTIVE IMMUNE SYSTEM
ARIH2	4	3181.46	0.475	ADAPTIVE IMMUNE SYSTEM
TUBB4A	1	460.836	0.476	ADAPTIVE IMMUNE SYSTEM
TRAF6	2	2718.63	0.478	ADAPTIVE IMMUNE SYSTEM
KIF2A	1	196.963	0.479	ADAPTIVE IMMUNE SYSTEM
PPP2R5E	3	1887.63	0.479	ADAPTIVE IMMUNE SYSTEM
PTPN11	3	7344.79	0.484	ADAPTIVE IMMUNE SYSTEM
ASB6	3	3012.4	0.485	ADAPTIVE IMMUNE SYSTEM
FBXO7	3	2720.72	0.486	ADAPTIVE IMMUNE SYSTEM
HLA-C	3	1558.62	0.486	ADAPTIVE IMMUNE SYSTEM
PSMB2	1	941.808	0.487	ADAPTIVE IMMUNE SYSTEM
NFKBIA	3	2901.92	0.489	ADAPTIVE IMMUNE SYSTEM
CD33	2	3298.38	0.490	ADAPTIVE IMMUNE SYSTEM
CD160	1	106.209	0.493	ADAPTIVE IMMUNE SYSTEM
SEC24B	1	1018.83	0.500	ADAPTIVE IMMUNE SYSTEM
FBXO6	1	146.736	0.501	ADAPTIVE IMMUNE SYSTEM
FBXO17	2	297.499	0.501	ADAPTIVE IMMUNE SYSTEM
KIFAP3	2	607.756	0.511	ADAPTIVE IMMUNE SYSTEM
LGMN	3	749.241	0.513	ADAPTIVE IMMUNE SYSTEM
NFATC1	1	175.101	0.516	ADAPTIVE IMMUNE SYSTEM
FCGR2B	2	549.869	0.519	ADAPTIVE IMMUNE SYSTEM
NCR3LG1	1	176.935	0.522	ADAPTIVE IMMUNE SYSTEM
MYD88	2	281.088	0.523	ADAPTIVE IMMUNE SYSTEM
LRSAM1	2	2262.36	0.525	ADAPTIVE IMMUNE SYSTEM
CD207	2	253.491	0.526	ADAPTIVE IMMUNE SYSTEM
DCAF1	2	759.106	0.529	ADAPTIVE IMMUNE SYSTEM
SPSB1	1	372.81	0.530	ADAPTIVE IMMUNE SYSTEM
SIGLEC1	11	15147.2	0.534	ADAPTIVE IMMUNE SYSTEM
FBXL16	2	347.859	0.534	ADAPTIVE IMMUNE SYSTEM
CBLB	1	74.7706	0.537	ADAPTIVE IMMUNE SYSTEM
ANAPC4	2	1575.77	0.540	ADAPTIVE IMMUNE SYSTEM
SIGLEC6	4	1074.49	0.542	ADAPTIVE IMMUNE SYSTEM
DNM2	4	3870.44	0.548	ADAPTIVE IMMUNE SYSTEM
SIGLEC7	2	1028.77	0.549	ADAPTIVE IMMUNE SYSTEM
ANAPC2	3	336.362	0.551	ADAPTIVE IMMUNE SYSTEM
AP1S3	2	333.621	0.551	ADAPTIVE IMMUNE SYSTEM
PDCD1LG2	3	1614.08	0.552	ADAPTIVE IMMUNE SYSTEM
SIGLEC10	9	5889.27	0.552	ADAPTIVE IMMUNE SYSTEM
XDH	8	5421.65	0.554	ADAPTIVE IMMUNE SYSTEM
TRIM63	4	1384.2	0.556	ADAPTIVE IMMUNE SYSTEM
FBXL19	3	2082.44	0.559	ADAPTIVE IMMUNE SYSTEM
UBR1	2	2229.15	0.561	ADAPTIVE IMMUNE SYSTEM
ITPR1	11	11865.3	0.565	ADAPTIVE IMMUNE SYSTEM
KIF5A	3	2830.34	0.566	ADAPTIVE IMMUNE SYSTEM
CD247	1	667.704	0.568	ADAPTIVE IMMUNE SYSTEM
SMURF2	4	1216.43	0.569	ADAPTIVE IMMUNE SYSTEM
REL	2	1894.02	0.569	ADAPTIVE IMMUNE SYSTEM
UBOX5	1	200.549	0.569	ADAPTIVE IMMUNE SYSTEM
PSME4	9	5497.86	0.570	ADAPTIVE IMMUNE SYSTEM
COL1A1	12	9288.38	0.570	ADAPTIVE IMMUNE SYSTEM
PSMA3	2	320.475	0.571	ADAPTIVE IMMUNE SYSTEM
PPP2R5C	8	4610.24	0.574	ADAPTIVE IMMUNE SYSTEM
TUBA3C	1	144.803	0.574	ADAPTIVE IMMUNE SYSTEM
FYB1	4	1845.95	0.576	ADAPTIVE IMMUNE SYSTEM
ASB8	1	104.563	0.578	ADAPTIVE IMMUNE SYSTEM
PSMD3	2	704.58	0.578	ADAPTIVE IMMUNE SYSTEM
CLEC4G	3	1147.2	0.578	ADAPTIVE IMMUNE SYSTEM
PSMB7	2	274.928	0.579	ADAPTIVE IMMUNE SYSTEM
FBXL5	2	914.411	0.580	ADAPTIVE IMMUNE SYSTEM
RNF6	1	142.618	0.582	ADAPTIVE IMMUNE SYSTEM
FBXL12	3	2520.17	0.583	ADAPTIVE IMMUNE SYSTEM
ITGB7	2	527.458	0.586	ADAPTIVE IMMUNE SYSTEM
SIGLEC8	2	314.123	0.587	ADAPTIVE IMMUNE SYSTEM
PSME3	1	155.122	0.591	ADAPTIVE IMMUNE SYSTEM
FBXO9	2	2052.73	0.597	ADAPTIVE IMMUNE SYSTEM
UBE2K	1	68.5941	0.599	ADAPTIVE IMMUNE SYSTEM
ACTR1A	3	609.671	0.604	ADAPTIVE IMMUNE SYSTEM
CD22	3	2879.37	0.605	ADAPTIVE IMMUNE SYSTEM
CD3D	1	39.7199	0.610	ADAPTIVE IMMUNE SYSTEM
FBXW7	1	29.7113	0.612	ADAPTIVE IMMUNE SYSTEM
CTSE	2	293.238	0.613	ADAPTIVE IMMUNE SYSTEM
MICB	4	2075.89	0.614	ADAPTIVE IMMUNE SYSTEM
RAET1E	4	1842.42	0.614	ADAPTIVE IMMUNE SYSTEM
PSMD13	2	170.236	0.614	ADAPTIVE IMMUNE SYSTEM

UBR2	5	1739.4	0.615	ADAPTIVE IMMUNE SYSTEM
PLCG1	2	334.818	0.617	ADAPTIVE IMMUNE SYSTEM
FZR1	2	761.638	0.617	ADAPTIVE IMMUNE SYSTEM
LILRA5	1	30.3013	0.621	ADAPTIVE IMMUNE SYSTEM
PPP2R5B	2	307.618	0.622	ADAPTIVE IMMUNE SYSTEM
HLA-DRA	2	1470.33	0.625	ADAPTIVE IMMUNE SYSTEM
CTSC	1	668.917	0.629	ADAPTIVE IMMUNE SYSTEM
SIGLEC5	3	1433.86	0.632	ADAPTIVE IMMUNE SYSTEM
TUBB3	1	81.5899	0.632	ADAPTIVE IMMUNE SYSTEM
CD209	2	1436.44	0.633	ADAPTIVE IMMUNE SYSTEM
BTN2A2	3	410.419	0.634	ADAPTIVE IMMUNE SYSTEM
HLA-E	1	270.267	0.635	ADAPTIVE IMMUNE SYSTEM
CUL7	1	839.348	0.635	ADAPTIVE IMMUNE SYSTEM
PIK3CB	4	1998.2	0.640	ADAPTIVE IMMUNE SYSTEM
PSMD12	2	485.828	0.641	ADAPTIVE IMMUNE SYSTEM
KIR2DL4	7	4118.04	0.642	ADAPTIVE IMMUNE SYSTEM
ICAM3	2	752.316	0.642	ADAPTIVE IMMUNE SYSTEM
SH3RF1	3	1467.68	0.645	ADAPTIVE IMMUNE SYSTEM
PIK3R3	2	941.166	0.647	ADAPTIVE IMMUNE SYSTEM
SPTBN2	9	7168.18	0.647	ADAPTIVE IMMUNE SYSTEM
KIF5B	3	695.657	0.649	ADAPTIVE IMMUNE SYSTEM
KIF15	3	975.021	0.650	ADAPTIVE IMMUNE SYSTEM
NCK1	1	473.579	0.650	ADAPTIVE IMMUNE SYSTEM
COL2A1	6	4325.4	0.650	ADAPTIVE IMMUNE SYSTEM
HSPA5	2	313.111	0.651	ADAPTIVE IMMUNE SYSTEM
ERAP1	6	5856.87	0.657	ADAPTIVE IMMUNE SYSTEM
CTSA	5	2126.3	0.658	ADAPTIVE IMMUNE SYSTEM
CUL3	3	536.151	0.660	ADAPTIVE IMMUNE SYSTEM
RNF123	7	2371.82	0.662	ADAPTIVE IMMUNE SYSTEM
WWP1	4	1684.08	0.664	ADAPTIVE IMMUNE SYSTEM
NFATC3	1	51.401	0.665	ADAPTIVE IMMUNE SYSTEM
ITCH	4	1458.34	0.666	ADAPTIVE IMMUNE SYSTEM
UBE2J2	2	1085.1	0.666	ADAPTIVE IMMUNE SYSTEM
THOP1	14	17355.4	0.667	ADAPTIVE IMMUNE SYSTEM
TRIM21	2	348.007	0.667	ADAPTIVE IMMUNE SYSTEM
HERC3	6	1494.79	0.668	ADAPTIVE IMMUNE SYSTEM
RNF217	3	2004.68	0.673	ADAPTIVE IMMUNE SYSTEM
YES1	1	543.682	0.674	ADAPTIVE IMMUNE SYSTEM
UBE2V2	2	372.489	0.678	ADAPTIVE IMMUNE SYSTEM
CARD11	6	6999.49	0.679	ADAPTIVE IMMUNE SYSTEM
CD96	1	206.011	0.680	ADAPTIVE IMMUNE SYSTEM
RAP1GAP	9	3855.18	0.686	ADAPTIVE IMMUNE SYSTEM
UNKL	9	4215.78	0.687	ADAPTIVE IMMUNE SYSTEM
CTSH	3	396.427	0.687	ADAPTIVE IMMUNE SYSTEM
RNF182	2	749.402	0.689	ADAPTIVE IMMUNE SYSTEM
DZIP3	3	858.59	0.690	ADAPTIVE IMMUNE SYSTEM
FBXL8	1	24.8194	0.690	ADAPTIVE IMMUNE SYSTEM
FBXO40	1	37.4501	0.691	ADAPTIVE IMMUNE SYSTEM
ITGB5	4	4889.89	0.694	ADAPTIVE IMMUNE SYSTEM
ZBTB16	1	24.7821	0.698	ADAPTIVE IMMUNE SYSTEM
DCTN1	2	955.305	0.701	ADAPTIVE IMMUNE SYSTEM
NPDC1	4	3066.64	0.702	ADAPTIVE IMMUNE SYSTEM
KLC2	1	258.711	0.705	ADAPTIVE IMMUNE SYSTEM
PPP2CB	1	67.6145	0.706	ADAPTIVE IMMUNE SYSTEM
PIK3AP1	1	70.9865	0.707	ADAPTIVE IMMUNE SYSTEM
HECTD3	2	1014.73	0.711	ADAPTIVE IMMUNE SYSTEM
RICTOR	2	390.283	0.713	ADAPTIVE IMMUNE SYSTEM
MADCAM1	12	10773.6	0.713	ADAPTIVE IMMUNE SYSTEM
PSMD4	2	543.955	0.715	ADAPTIVE IMMUNE SYSTEM
YWHAZ	1	92.0227	0.722	ADAPTIVE IMMUNE SYSTEM
TRIM9	1	427.606	0.723	ADAPTIVE IMMUNE SYSTEM
MIB2	6	1948.95	0.726	ADAPTIVE IMMUNE SYSTEM
KLC3	4	805.974	0.726	ADAPTIVE IMMUNE SYSTEM
ACTR1B	3	1431.5	0.727	ADAPTIVE IMMUNE SYSTEM
PDCD1	2	640.72	0.732	ADAPTIVE IMMUNE SYSTEM
PJA2	4	1750.35	0.733	ADAPTIVE IMMUNE SYSTEM
AP2A2	10	2396.76	0.743	ADAPTIVE IMMUNE SYSTEM
SELL	1	40.3109	0.743	ADAPTIVE IMMUNE SYSTEM
KLHL25	2	436.639	0.743	ADAPTIVE IMMUNE SYSTEM
RACGAP1	4	1302.33	0.744	ADAPTIVE IMMUNE SYSTEM
TLR1	5	862.332	0.744	ADAPTIVE IMMUNE SYSTEM
ITGB1	4	1853.42	0.744	ADAPTIVE IMMUNE SYSTEM
CHUK	5	3253.31	0.747	ADAPTIVE IMMUNE SYSTEM
AP1G1	5	881.608	0.748	ADAPTIVE IMMUNE SYSTEM

KLHL5	1	295.136	0.749	ADAPTIVE IMMUNE SYSTEM
RNF130	1	7.11406	0.750	ADAPTIVE IMMUNE SYSTEM
SAR1B	3	351.721	0.751	ADAPTIVE IMMUNE SYSTEM
CD101	1	187.88	0.751	ADAPTIVE IMMUNE SYSTEM
CSK	2	318.829	0.752	ADAPTIVE IMMUNE SYSTEM
PSMC1	2	502.865	0.754	ADAPTIVE IMMUNE SYSTEM
LCP2	3	890.138	0.754	ADAPTIVE IMMUNE SYSTEM
FBXO15	3	548.728	0.761	ADAPTIVE IMMUNE SYSTEM
NCR2	2	464.024	0.766	ADAPTIVE IMMUNE SYSTEM
UBE3C	5	2845.14	0.766	ADAPTIVE IMMUNE SYSTEM
RELA	2	1125.35	0.768	ADAPTIVE IMMUNE SYSTEM
RPS27A	1	73.2875	0.770	ADAPTIVE IMMUNE SYSTEM
KIF18A	1	80.9047	0.771	ADAPTIVE IMMUNE SYSTEM
LILRA1	4	709.752	0.773	ADAPTIVE IMMUNE SYSTEM
RNF111	8	2735.43	0.774	ADAPTIVE IMMUNE SYSTEM
ITGAL	4	2599.38	0.777	ADAPTIVE IMMUNE SYSTEM
CTSK	1	56.6202	0.777	ADAPTIVE IMMUNE SYSTEM
TUBB1	1	14.6338	0.777	ADAPTIVE IMMUNE SYSTEM
LRRC41	1	80.6609	0.786	ADAPTIVE IMMUNE SYSTEM
TAPBP	2	508.714	0.790	ADAPTIVE IMMUNE SYSTEM
OSBPL1A	3	1240.88	0.791	ADAPTIVE IMMUNE SYSTEM
CTSL	2	351.799	0.794	ADAPTIVE IMMUNE SYSTEM
ASB16	1	252.209	0.795	ADAPTIVE IMMUNE SYSTEM
AP2M1	3	795.672	0.800	ADAPTIVE IMMUNE SYSTEM
BLNK	4	1348.57	0.804	ADAPTIVE IMMUNE SYSTEM
TRIP12	8	3679.29	0.804	ADAPTIVE IMMUNE SYSTEM
TAP2	8	5811.75	0.809	ADAPTIVE IMMUNE SYSTEM
PSMA4	2	361.252	0.809	ADAPTIVE IMMUNE SYSTEM
RBBP6	1	13.7942	0.812	ADAPTIVE IMMUNE SYSTEM
BTNL9	4	761.977	0.813	ADAPTIVE IMMUNE SYSTEM
AP1M2	3	501.252	0.816	ADAPTIVE IMMUNE SYSTEM
SIGLEC9	3	599.003	0.817	ADAPTIVE IMMUNE SYSTEM
NEDD4	8	1174.35	0.818	ADAPTIVE IMMUNE SYSTEM
KIF20A	1	15.5086	0.819	ADAPTIVE IMMUNE SYSTEM
SEC24C	4	2689.02	0.825	ADAPTIVE IMMUNE SYSTEM
CD300A	3	245.205	0.828	ADAPTIVE IMMUNE SYSTEM
ICAM2	1	8.4315	0.830	ADAPTIVE IMMUNE SYSTEM
NCR1	3	192.569	0.830	ADAPTIVE IMMUNE SYSTEM
KIR3DL2	3	221.42	0.832	ADAPTIVE IMMUNE SYSTEM
CD1B	1	37.4283	0.833	ADAPTIVE IMMUNE SYSTEM
PSMC5	2	227.826	0.834	ADAPTIVE IMMUNE SYSTEM
PVR	1	160.926	0.834	ADAPTIVE IMMUNE SYSTEM
IKBKB	3	132.16	0.836	ADAPTIVE IMMUNE SYSTEM
ANAPC1	2	417.09	0.836	ADAPTIVE IMMUNE SYSTEM
PAK2	1	6.97659	0.837	ADAPTIVE IMMUNE SYSTEM
LILRA6	6	1159.03	0.837	ADAPTIVE IMMUNE SYSTEM
KIR3DL1	7	2292.05	0.837	ADAPTIVE IMMUNE SYSTEM
UBE2E2	4	651.073	0.838	ADAPTIVE IMMUNE SYSTEM
RAP1B	3	398.375	0.843	ADAPTIVE IMMUNE SYSTEM
CTSD	4	843.091	0.844	ADAPTIVE IMMUNE SYSTEM
FBXL20	2	621.424	0.844	ADAPTIVE IMMUNE SYSTEM
PSMD14	2	91.0049	0.845	ADAPTIVE IMMUNE SYSTEM
BTN2A1	5	1308.67	0.849	ADAPTIVE IMMUNE SYSTEM
NCF2	3	392.542	0.850	ADAPTIVE IMMUNE SYSTEM
ELOC	1	36.5738	0.851	ADAPTIVE IMMUNE SYSTEM
PSMA7	2	118.382	0.856	ADAPTIVE IMMUNE SYSTEM
KLHL22	1	102.597	0.857	ADAPTIVE IMMUNE SYSTEM
SOS1	4	654.542	0.859	ADAPTIVE IMMUNE SYSTEM
LRR1	2	34.8516	0.859	ADAPTIVE IMMUNE SYSTEM
CYBA	3	81.8242	0.860	ADAPTIVE IMMUNE SYSTEM
FBXO41	2	34.4517	0.861	ADAPTIVE IMMUNE SYSTEM
SRC	3	468.232	0.862	ADAPTIVE IMMUNE SYSTEM
HCST	1	54.1105	0.864	ADAPTIVE IMMUNE SYSTEM
UBE3D	2	65.3062	0.864	ADAPTIVE IMMUNE SYSTEM
RNF144B	1	26.5772	0.865	ADAPTIVE IMMUNE SYSTEM
KLRB1	1	6.34327	0.869	ADAPTIVE IMMUNE SYSTEM
LTN1	4	694.955	0.871	ADAPTIVE IMMUNE SYSTEM
RNF126	13	2496.46	0.871	ADAPTIVE IMMUNE SYSTEM
CD226	1	76.0673	0.871	ADAPTIVE IMMUNE SYSTEM
TRIB3	1	11.5523	0.873	ADAPTIVE IMMUNE SYSTEM
CANX	1	11.7519	0.876	ADAPTIVE IMMUNE SYSTEM
PTPRJ	5	190.391	0.877	ADAPTIVE IMMUNE SYSTEM
TRIM4	1	20.8361	0.878	ADAPTIVE IMMUNE SYSTEM
LNX1	3	67.2203	0.878	ADAPTIVE IMMUNE SYSTEM

UBE2V1	1	4.44387	0.882	ADAPTIVE IMMUNE SYSTEM
KLHL2	1	3.99832	0.885	ADAPTIVE IMMUNE SYSTEM
RAPGEF3	6	1944.74	0.887	ADAPTIVE IMMUNE SYSTEM
ITPR3	20	3866.94	0.887	ADAPTIVE IMMUNE SYSTEM
TUBB4B	1	5.99978	0.899	ADAPTIVE IMMUNE SYSTEM
PLCG2	9	1388.68	0.905	ADAPTIVE IMMUNE SYSTEM
PPL	7	292.206	0.910	ADAPTIVE IMMUNE SYSTEM
TUBB6	1	2.31435	0.912	ADAPTIVE IMMUNE SYSTEM
CD200R1	1	2.03298	0.916	ADAPTIVE IMMUNE SYSTEM
ACTR10	1	5.51176	0.921	ADAPTIVE IMMUNE SYSTEM
FBXW12	1	10.1706	0.928	ADAPTIVE IMMUNE SYSTEM
TRIM69	1	4.11981	0.939	ADAPTIVE IMMUNE SYSTEM
MAPKAP1	1	0.424263	0.952	ADAPTIVE IMMUNE SYSTEM
CTSV	1	1.0437	0.961	ADAPTIVE IMMUNE SYSTEM
CD3E	1	0.486528	0.963	ADAPTIVE IMMUNE SYSTEM
KEAP1	1	0.768956	0.974	ADAPTIVE IMMUNE SYSTEM
HECTD2	1	0.461612	0.981	ADAPTIVE IMMUNE SYSTEM
CAPZB	1	0.914266	0.983	ADAPTIVE IMMUNE SYSTEM
UBE2N	1	0.0113746	0.990	ADAPTIVE IMMUNE SYSTEM
PSMB11	1	0.189101	0.992	ADAPTIVE IMMUNE SYSTEM
LAIR2	1	0.0107827	0.996	ADAPTIVE IMMUNE SYSTEM
TRPC1	4	1.54251	1	ADAPTIVE IMMUNE SYSTEM
LAIR1	2	128.947	1	ADAPTIVE IMMUNE SYSTEM
CCNF	3	0.139558	1	ADAPTIVE IMMUNE SYSTEM
HERC2	26	14501.7	1	ADAPTIVE IMMUNE SYSTEM
SKP1	2	769.732	1	ADAPTIVE IMMUNE SYSTEM
CUL2	3	230.546	1	ADAPTIVE IMMUNE SYSTEM
PIK3CA	6	151.583	1	ADAPTIVE IMMUNE SYSTEM
CDC16	3	89.734	1	ADAPTIVE IMMUNE SYSTEM
UBA52	2	4.03884	1	ADAPTIVE IMMUNE SYSTEM
KIF11	5	49.4843	1	ADAPTIVE IMMUNE SYSTEM
FBXL18	2	48.2339	1	ADAPTIVE IMMUNE SYSTEM
RNF19A	2	54.8618	1	ADAPTIVE IMMUNE SYSTEM
BLK	3	45.268	1	ADAPTIVE IMMUNE SYSTEM
TRIM37	5	111.595	1	ADAPTIVE IMMUNE SYSTEM
PTPRC	4	395.503	1	ADAPTIVE IMMUNE SYSTEM
SPSB2	4	930.443	1	ADAPTIVE IMMUNE SYSTEM
ANAPC5	3	186.101	1	ADAPTIVE IMMUNE SYSTEM
TRAF7	9	508.43	1	ADAPTIVE IMMUNE SYSTEM
ERAP2	3	45.3735	1	ADAPTIVE IMMUNE SYSTEM
DCTN4	3	110.702	1	ADAPTIVE IMMUNE SYSTEM
FBXO22	2	278.226	1	ADAPTIVE IMMUNE SYSTEM
CDC27	4	362.076	1	ADAPTIVE IMMUNE SYSTEM
SEC24A	6	294.403	1	ADAPTIVE IMMUNE SYSTEM
UBR4	10	370.752	1	ADAPTIVE IMMUNE SYSTEM
PPP3CA	3	12.3568	1	ADAPTIVE IMMUNE SYSTEM
FBXL4	3	569.843	1	ADAPTIVE IMMUNE SYSTEM
CD34	2	43.2682	1	ADAPTIVE IMMUNE SYSTEM
FBXW2	2	0.55473	1	ADAPTIVE IMMUNE SYSTEM
RAC1	2	325.8	1	ADAPTIVE IMMUNE SYSTEM
LNPEP	3	2303.67	1	ADAPTIVE IMMUNE SYSTEM
MTOR	9	726.593	1	ADAPTIVE IMMUNE SYSTEM
CD8B	2	395.846	1	ADAPTIVE IMMUNE SYSTEM
FBXW5	6	111.707	1	ADAPTIVE IMMUNE SYSTEM
ASB14	2	45.3978	1	ADAPTIVE IMMUNE SYSTEM
BTBD6	12	7871.17	1	ADAPTIVE IMMUNE SYSTEM
CENPE	3	530.457	1	ADAPTIVE IMMUNE SYSTEM
TRIM50	4	18.0716	1	ADAPTIVE IMMUNE SYSTEM
PDIA3	2	18.977	1	ADAPTIVE IMMUNE SYSTEM
ZNRF1	3	106.279	1	ADAPTIVE IMMUNE SYSTEM
MRC2	4	91.2349	1	ADAPTIVE IMMUNE SYSTEM
RAF1	2	NA	NA	ADAPTIVE IMMUNE SYSTEM
ANAPC11	2	NA	NA	ADAPTIVE IMMUNE SYSTEM

Gene	NumVar	Q	Pvalue	Gene-set
PRKN	13	856826	0.008	ALPHA SYNUCLEIN PATHWAY
FYN	6	20441	0.055	ALPHA SYNUCLEIN PATHWAY
BAD	1	185.522	0.082	ALPHA SYNUCLEIN PATHWAY
PLD1	3	11745.6	0.097	ALPHA SYNUCLEIN PATHWAY
LCK	2	6622.49	0.122	ALPHA SYNUCLEIN PATHWAY
PLD2	9	18632.6	0.165	ALPHA SYNUCLEIN PATHWAY
UCHL1	1	1327.34	0.184	ALPHA SYNUCLEIN PATHWAY
SYK	1	726.573	0.266	ALPHA SYNUCLEIN PATHWAY
PARK7	3	1643.8	0.307	ALPHA SYNUCLEIN PATHWAY

PLCB2	6	10676.9	0.448	ALPHA SYNUCLEIN PATHWAY
PTK2B	5	4269.52	0.476	ALPHA SYNUCLEIN PATHWAY
SNCA	3	4903.09	0.486	ALPHA SYNUCLEIN PATHWAY
MAPK3	1	54.5027	0.549	ALPHA SYNUCLEIN PATHWAY
FGR	1	268.01	0.573	ALPHA SYNUCLEIN PATHWAY
YES1	1	543.682	0.674	ALPHA SYNUCLEIN PATHWAY
PRKCD	4	470.582	0.788	ALPHA SYNUCLEIN PATHWAY
SLC6A3	4	790.553	0.805	ALPHA SYNUCLEIN PATHWAY
GRK5	1	18.7956	0.805	ALPHA SYNUCLEIN PATHWAY
TOR1A	1	164.337	0.828	ALPHA SYNUCLEIN PATHWAY
TH	5	3586.85	0.847	ALPHA SYNUCLEIN PATHWAY
SRC	3	468.232	0.862	ALPHA SYNUCLEIN PATHWAY
HCK	5	1661.83	0.875	ALPHA SYNUCLEIN PATHWAY
BLK	3	45.268	1	ALPHA SYNUCLEIN PATHWAY

Gene	NumVar	Q	Pvalue	Gene-set
PRKN	13	856826	0.008	PARKINSONS DISEASE
NDUFA10	3	8280.31	0.031	PARKINSONS DISEASE
NDUFS8	4	8311.64	0.053	PARKINSONS DISEASE
NDUFS2	7	18048	0.054	PARKINSONS DISEASE
CASP9	5	20632.8	0.069	PARKINSONS DISEASE
COX4I2	3	38887.7	0.082	PARKINSONS DISEASE
NDUFS7	10	54469.5	0.100	PARKINSONS DISEASE
COX5A	1	1412.9	0.160	PARKINSONS DISEASE
NDUFV1	2	2158.75	0.170	PARKINSONS DISEASE
UCHL1	1	1327.34	0.184	PARKINSONS DISEASE
UQCRB	1	325.945	0.199	PARKINSONS DISEASE
NDUFS6	1	5106.93	0.227	PARKINSONS DISEASE
UBA7	3	1898.47	0.227	PARKINSONS DISEASE
UBE2J1	1	1008.81	0.232	PARKINSONS DISEASE
COX4I1	1	330.989	0.252	PARKINSONS DISEASE
NDUFB10	1	4333.45	0.288	PARKINSONS DISEASE
SLC18A1	2	2088.33	0.290	PARKINSONS DISEASE
PARK7	3	1643.8	0.307	PARKINSONS DISEASE
COX7A1	6	10988.6	0.351	PARKINSONS DISEASE
NDUFB7	1	243.419	0.362	PARKINSONS DISEASE
NDUFB3	2	1371.69	0.368	PARKINSONS DISEASE
SDHA	6	3838.7	0.380	PARKINSONS DISEASE
UBB	4	3950.69	0.383	PARKINSONS DISEASE
APAF1	4	5645.14	0.389	PARKINSONS DISEASE
NDUFB2	2	321.324	0.402	PARKINSONS DISEASE
UQCRH	1	130.97	0.407	PARKINSONS DISEASE
SDHB	3	5386.95	0.435	PARKINSONS DISEASE
COX6B2	5	5402.37	0.466	PARKINSONS DISEASE
PPID	3	2148.08	0.480	PARKINSONS DISEASE
SNCA	3	4903.09	0.486	PARKINSONS DISEASE
SLC18A2	2	724.162	0.492	PARKINSONS DISEASE
NDUFS5	1	46.154	0.496	PARKINSONS DISEASE
UQCR10	4	1349.63	0.538	PARKINSONS DISEASE
COX6A2	4	655.78	0.595	PARKINSONS DISEASE
NDUFA3	2	1707.39	0.601	PARKINSONS DISEASE
NDUFC1	1	54.7771	0.610	PARKINSONS DISEASE
CYC1	3	2395.6	0.628	PARKINSONS DISEASE
NDUFAB1	1	47.299	0.631	PARKINSONS DISEASE
COX5B	1	179.531	0.656	PARKINSONS DISEASE
UBE2J2	2	1085.1	0.666	PARKINSONS DISEASE
NDUFV3	4	1441.93	0.693	PARKINSONS DISEASE
NDUFA5	4	4121.43	0.715	PARKINSONS DISEASE
NDUFA8	1	14.1714	0.750	PARKINSONS DISEASE
COX7A2	3	839.18	0.766	PARKINSONS DISEASE
NDUFV2	1	17.9868	0.771	PARKINSONS DISEASE
NDUFS4	1	123.047	0.802	PARKINSONS DISEASE
SLC6A3	4	790.553	0.805	PARKINSONS DISEASE
UQCR11	1	131.742	0.828	PARKINSONS DISEASE
NDUFA4	1	12.5069	0.829	PARKINSONS DISEASE
TH	5	3586.85	0.847	PARKINSONS DISEASE
NDUFA6	1	0.639585	0.960	PARKINSONS DISEASE
NDUFC2	1	0.83966	0.984	PARKINSONS DISEASE
NDUFS1	7	964.171	1	PARKINSONS DISEASE
LRRK2	6	171.441	1	PARKINSONS DISEASE

c) CADD score > 12

Gene	NumVar	Q	Pvalue	Gene-set

MAPK3	1	15775.9	0.005	LONG TERM DEPRESSION
CACNA1A	1	20671.1	0.013	LONG TERM DEPRESSION
PLA2G4E	1	1010.48	0.027	LONG TERM DEPRESSION
PLA2G2E	1	422.514	0.140	LONG TERM DEPRESSION
PPP2R1A	1	1061.75	0.259	LONG TERM DEPRESSION
PPP2R1B	1	71.1039	0.529	LONG TERM DEPRESSION
JMJD7-PLA2G4B	1	42.8439	0.662	LONG TERM DEPRESSION
PLA2G4B	1	42.8439	0.662	LONG TERM DEPRESSION
PLA2G3	1	75.937	0.741	LONG TERM DEPRESSION

Gene	NumVar	Q	Pvalue	Gene-set
VAMP5	1	2100.02	0.077	SNARE INTERACTIONS IN VESICULAR TRANSPORT
GOSR2	1	206.968	0.388	SNARE INTERACTIONS IN VESICULAR TRANSPORT

Gene	NumVar	Q	Pvalue	Gene-set
MAPK3	1	15775.9	0.005	ALPHA SYNUCLEIN PATHWAY
PLD2	1	572.968	0.280	ALPHA SYNUCLEIN PATHWAY
PTK2B	1	163.702	0.387	ALPHA SYNUCLEIN PATHWAY

Gene	NumVar	Q	Pvalue	Gene-set
MAPK3	1	15775.9	0.005	DTRANSMITTER RECEPTORS AND POSTSYNAPTIC SIGNAL TRANSMISSION
GRIK2	1	12645	0.055	DTRANSMITTER RECEPTORS AND POSTSYNAPTIC SIGNAL TRANSMISSION
EPB41L1	2	5224.43	0.072	DTRANSMITTER RECEPTORS AND POSTSYNAPTIC SIGNAL TRANSMISSION
GABRA5	1	3053.72	0.080	DTRANSMITTER RECEPTORS AND POSTSYNAPTIC SIGNAL TRANSMISSION
CHRNE	1	1105.55	0.280	DTRANSMITTER RECEPTORS AND POSTSYNAPTIC SIGNAL TRANSMISSION
TUBA3E	1	188.084	0.335	DTRANSMITTER RECEPTORS AND POSTSYNAPTIC SIGNAL TRANSMISSION
CHRNA9	1	139.31	0.378	DTRANSMITTER RECEPTORS AND POSTSYNAPTIC SIGNAL TRANSMISSION
CACNG4	1	86.5487	0.526	DTRANSMITTER RECEPTORS AND POSTSYNAPTIC SIGNAL TRANSMISSION
CAMK2B	2	581.525	0.643	DTRANSMITTER RECEPTORS AND POSTSYNAPTIC SIGNAL TRANSMISSION
KCNJ16	1	24.8988	0.756	DTRANSMITTER RECEPTORS AND POSTSYNAPTIC SIGNAL TRANSMISSION
GNAT3	2	128.133	0.774	DTRANSMITTER RECEPTORS AND POSTSYNAPTIC SIGNAL TRANSMISSION
TUBB1	1	14.6338	0.777	DTRANSMITTER RECEPTORS AND POSTSYNAPTIC SIGNAL TRANSMISSION

Gene	NumVar	Q	Pvalue	Gene-set
MAPK3	1	15775.9	0.005	TRANSMISSION ACROSS CHEMICAL SYNAPSES
CACNA1A	1	20671.1	0.013	TRANSMISSION ACROSS CHEMICAL SYNAPSES
LRTOMT	1	10049.2	0.051	TRANSMISSION ACROSS CHEMICAL SYNAPSES
GRIK2	1	12645	0.055	TRANSMISSION ACROSS CHEMICAL SYNAPSES
EPB41L1	2	5224.43	0.072	TRANSMISSION ACROSS CHEMICAL SYNAPSES
GABRA5	1	3053.72	0.080	TRANSMISSION ACROSS CHEMICAL SYNAPSES
SLC22A1	1	569.652	0.275	TRANSMISSION ACROSS CHEMICAL SYNAPSES
CHRNE	1	1105.55	0.280	TRANSMISSION ACROSS CHEMICAL SYNAPSES
TUBA3E	1	188.084	0.335	TRANSMISSION ACROSS CHEMICAL SYNAPSES
CHRNA9	1	139.31	0.378	TRANSMISSION ACROSS CHEMICAL SYNAPSES
CACNG4	1	86.5487	0.526	TRANSMISSION ACROSS CHEMICAL SYNAPSES
CACNA2D3	1	104.697	0.609	TRANSMISSION ACROSS CHEMICAL SYNAPSES
CAMK2B	2	581.525	0.643	TRANSMISSION ACROSS CHEMICAL SYNAPSES
TSPOAP1	1	61.194	0.667	TRANSMISSION ACROSS CHEMICAL SYNAPSES
KCNJ16	1	24.8988	0.756	TRANSMISSION ACROSS CHEMICAL SYNAPSES
GNAT3	2	128.133	0.774	TRANSMISSION ACROSS CHEMICAL SYNAPSES
TUBB1	1	14.6338	0.777	TRANSMISSION ACROSS CHEMICAL SYNAPSES
SYT1	1	51.8168	0.784	TRANSMISSION ACROSS CHEMICAL SYNAPSES
ALDH5A1	1	6.82984	0.846	TRANSMISSION ACROSS CHEMICAL SYNAPSES

Supplementary Table 8. Genes within expression community maps and modularity ranges

Gene	Community (0-53)	Page rank
FGR	0	0.000207389
GCLC	4	0.00013302
NIPAL3	2	0.000317792
CYP51A1	3	0.0000454
RAD52	2	0.000244755
LAP3	5	0.0000827
MAD1L1	2	0.000161232
LASP1	1	0.000212445
SNX11	0	0.000267431
TMEM176A	6	0.000154847
ICA1	2	0.0000241
ALS2	2	0.000272124
CFLAR	0	0.000319236
TFPI	4	0.0000774
ARF5	0	0.000179664
PLXND1	1	0.0000941
FKBP4	2	0.0001935
RBM6	2	0.000268769
CAMKK1	1	0.0000635
SLC25A13	2	0.00030961
SLC4A1	4	0.000194682
PRSS22	7	0.000151485
UPF1	1	0.000291971
SKAP2	0	0.0000695
SLC25A5	3	0.000200838
POLR2J	0	0.00021205
THSD7A	8	0.0002488
LIG3	2	0.000333005
ACSM3	9	0.000154847
FAM214B	1	0.0000864

MSL3	0	0.000256808
TSPOAP1	1	0.0000508
MPO	10	0.000174902
WDR54	3	0.000128426
ABCB4	11	0.000172024
SOX8	7	0.0000488
FBXL3	0	0.000304971
ZFX	3	0.000134248
LAMP2	0	0.000235402
ITGA2B	4	0.00011682
GDE1	0	0.000121638
TMEM98	7	0.000217256
CACNG3	7	0.000247931
TNFRSF12A	0	0.0000534
MAP3K9	2	0.0000461
KDM7A	0	0.00026457
ALDH3B1	1	0.000102723
TTC22	1	0.000136515
FARP2	2	0.000186044
TBXA2R	7	0.0000352
IFRD1	0	0.000165943
LGALS14	7	0.000249265
COX10	3	0.000378736
PAF1	0	0.000173608
SCIN	7	0.000256244
ARSD	0	0.000039
PNPLA4	3	0.0000566
CDKL3	3	0.0000317
CCDC124	4	0.0000516
CEACAM21	3	0.000248262
PAFAH1B1	2	0.0002325
AC005747.1	1	0.0000893
MATK	3	0.0000437

CD79B	11	0.00014503
RPUSD1	1	0.000117324
LUC7L	2	0.000288773
CACNA2D2	3	0.0000817
CRAMP1	1	0.00026435
MYLIP	0	0.000232629
E2F2	4	0.0000743
JARID2	0	0.000194723
CDKL5	0	0.000211663
CDK11A	1	0.0000239
NADK	1	0.000209487
DLEC1	1	0.0000627
CYTH3	2	0.0000744
ADAM22	3	0.000113891
CYB561	1	0.0000321
SPAG9	0	0.00028054
CELSR3	7	0.0000503
AASS	2	0.0000244
MPND	1	0.000159824
MGST1	0	0.0000256
NFIX	4	0.0000757
ST3GAL1	2	0.000286823
MMP25	1	0.0000341
IL32	2	0.0000269
ETV7	5	0.0000684
DYRK4	3	0.000228871
HHATL	7	0.000294653
PHF7	2	0.00022974
SEMA3G	7	0.000174522
STAB1	1	0.0000348
FUZ	0	0.0000727
SLC6A13	7	0.000238273
PRSS3	7	0.000216868

ZNF200	0	0.000269771
BTK	0	0.000234692
HFE	3	0.0000985
SCMH1	2	0.000258686
FYN	2	0.000300577
HIVEP2	2	0.000323938
ELOA	2	0.000286588
MRC2	4	0.0000933
TSPAN9	7	0.000113569
ABHD5	0	0.00016657
AKAP8L	0	0.000238825
TTC19	3	0.000374536
PLAUR	0	0.000113566
QPCTL	1	0.0000272
PPP5C	2	0.000122966
TYROBP	0	0.0000518
BRCA1	0	0.00013349
CD22	11	0.000152747
SEMA3B	7	0.0000548
MBTPS2	3	0.000291986
LTF	10	0.000141965
EXTL3	1	0.000216714
NR1H4	7	0.000280815
ELOVL5	0	0.000293953
ALOX5	0	0.000161268
KDM5D	12	0.00016609
CALCOCO1	1	0.000341552
EHD3	4	0.0000425
SLC25A39	4	0.000218644
MVP	0	0.000196289
RNF14	4	0.000167246
DNASE1L1	0	0.000148716
TACC3	1	0.00016904

POLA2	1	0.000156389
ZC3H3	1	0.000221106
CAPN1	1	0.000103134
ACPP	0	0.0000606
MTMR11	0	0.0000352
WAS	1	0.000163736
BID	0	0.000279838
CHDH	7	0.0000371
ATP2C1	3	0.000388389
SLC38A5	4	0.000053
RALBP1	0	0.000114588
SLC11A1	1	0.00010206
TTC27	3	0.000330157
VSIG2	7	0.000260815
CD74	1	0.0000636
HGF	0	0.0000905
ADGRA2	7	0.0000254
PLEKHB1	2	0.0000531
SERPINB1	0	0.000205618
SPAST	0	0.000270609
OSBPL5	1	0.0000648
CPS1	7	0.000299113
FHL1	4	0.000115153
RTF2	0	0.000256097
SLC45A4	1	0.000136039
RNF10	4	0.000204315
GRAMD1B	1	0.0000621
RNH1	3	0.000160298
RB1CC1	0	0.000204464
ALAS1	1	0.000260066
SNAPC1	3	0.0000694
ABCC2	2	0.0000685
PLEKHO1	0	0.000242621

UBR2	0	0.000271068
EHD2	7	0.000289873
DEPDC1	14	0.000163692
CCDC28A	0	0.000285226
RRAGD	0	0.000191856
HSF2	3	0.000237157
HSD17B6	7	0.000245035
NR1H3	1	0.0000416
TYMP	5	0.00005
TOMM34	3	0.000156203
VIM	0	0.0001262
RNASET2	0	0.0000522
CD44	2	0.000315823
KCNG1	7	0.0000239
AGPAT4	2	0.0000564
MIPEP	2	0.000290262
IFNGR1	0	0.000152249
SH2D2A	1	0.0000463
TNFRSF1B	1	0.000206832
POU2F2	1	0.000149645
ANK1	4	0.000148509
TFB1M	3	0.000345528
BAK1	0	0.0000946
IKZF2	3	0.0000341
GRN	1	0.000131126
EIPR1	2	0.000360759
IFT88	3	0.000193012
ABCF2	2	0.000141429
FUT8	3	0.00023938
GAB2	1	0.00019356
ATP6V0A1	1	0.000227978
APBA2	2	0.0000591
MAP2K3	1	0.0000895

EFCAB1	7	0.000198679
TMSB10	3	0.00008
GABARAPL2	4	0.000129478
SH3YL1	3	0.000246184
VCL	1	0.0000966
DAPK2	1	0.00011164
STAP1	11	0.000113431
AC118549.1	3	0.000317322
SLC18A1	7	0.0000256
TUBG2	2	0.0000573
RPL26L1	3	0.0000691
FLT4	7	0.0000244
FBXO42	2	0.000430859
METTL1	3	0.0000295
BOD1L1	0	0.000225039
EDC4	1	0.000269796
TRIO	2	0.000120119
VCAN	0	0.0000532
CLEC16A	2	0.0002545
ZFYVE16	0	0.000198456
PHLPP2	4	0.0000814
SPDL1	3	0.00016303
SLC66A1	1	0.000150759
RTN4R	7	0.00018445
PHF23	0	0.000285095
RAB27B	4	0.000132256
PARP3	1	0.0000403
THAP3	3	0.000291449
AIFM2	2	0.000125406
AL049834.1	3	0.000130012
CAPG	0	0.0000357
AP2S1	4	0.000105109
ADAM28	11	0.000137811

DCUN1D1	4	0.0000967
LCP2	0	0.000313922
CTNNA1	0	0.0000876
PHKA2	1	0.000319694
CNTLN	0	0.0000507
DSG2	7	0.000293443
GEMIN8	3	0.000309818
OFD1	3	0.00031567
TPR	0	0.000280801
KIAA0556	1	0.000252452
DTNBP1	2	0.000226856
XK	4	0.0000842
SCML1	3	0.0000639
ARHGAP6	4	0.000174548
USP28	3	0.000161595
HDAC9	3	0.00005
NOP16	3	0.000206987
RRM2B	0	0.000176526
TNFRSF17	15	0.000108549
SNX29	2	0.00023814
VPS13D	2	0.000258723
ADAMTS6	11	0.0000418
H6PD	1	0.000197477
PER3	2	0.0000304
LTBP1	4	0.000297584
RCN1	3	0.0000271
RFC2	0	0.000328576
ARID1B	2	0.000310793
FOXP3	1	0.0000316
HEXB	0	0.000295126
PTCD2	2	0.000310843
DKK3	7	0.000141368
MCUR1	3	0.00010081

LIMA1	3	0.000128945
LAMC3	7	0.000273491
HOMER3	0	0.0000435
RAD51	7	0.0000236
POLQ	14	0.000230782
THOC3	3	0.000171649
HEBP2	0	0.000221345
PLEKHA5	3	0.0000309
RRP12	1	0.0000513
FNIP2	2	0.000251565
FSTL4	7	0.000202626
USE1	1	0.0000585
THRAP3	0	0.00026408
PHPT1	3	0.00013054
ENTPD2	7	0.0000237
LY75	0	0.000191399
OPN3	3	0.000212625
SDCCAG8	2	0.000312865
PTPRN	7	0.000268994
HHAT	2	0.0000816
KIF1B	1	0.000117045
FOXC1	7	0.000200715
TBC1D22A	2	0.000322339
SYNE2	2	0.00020832
RELT	1	0.000244369
TAB2	0	0.000336399
EIF2AK2	5	0.000120206
KMT2C	2	0.000289519
ITIH4	1	0.0000263
DCBLD2	3	0.0000483
SOAT1	0	0.000337661
PKP2	7	0.0000314
PRDM1	0	0.0000886

TMCC3	0	0.000167076
PITHD1	4	0.000138211
MTA3	2	0.0002564
USP13	2	0.000264434
CROCC	1	0.0000825
ATP2B4	2	0.000173067
YIPF1	0	0.000249042
DGKG	1	0.0000867
FLYWCH1	1	0.0000692
UNKL	1	0.000251663
TBXAS1	0	0.000260724
ALDH18A1	2	0.000264842
TARBP1	3	0.000304668
MXD1	0	0.00020851
SLC2A3	0	0.00010851
CTDP1	0	0.000221439
YBX3	4	0.000219523
WNK1	4	0.0000892
OGFR	0	0.000136992
GNA15	0	0.0000728
RIMBP2	7	0.000206677
QSER1	2	0.000255635
LZTS1	7	0.000270157
NCKAP1	3	0.0000254
GUCY1B1	4	0.000330147
DGAT2	0	0.000126039
CS	2	0.000282163
LTK	16	0.000154847
MRPS24	3	0.0000245
VMP1	0	0.000227615
SEZ6	7	0.000274229
SLC6A16	2	0.000124725
BICRA	1	0.000129968

CA11	7	0.000046
U2AF2	1	0.000270239
EPN1	1	0.000246479
AHRR	7	0.000115958
ZNF275	2	0.000198093
HAGH	4	0.000212795
CASP8	0	0.000313957
ST3GAL6	0	0.00013936
CDON	7	0.000153173
HIPK2	1	0.000129152
TMEM161A	1	0.0000957
CTSA	1	0.000113379
CNN2	1	0.000147566
ABCA7	1	0.000106634
FAR2	0	0.0000987
BCAS1	7	0.0000568
SBNO2	1	0.000148819
CALCRL	3	0.0000249
ANKS1A	1	0.000260724
UHRF1BP1	2	0.000138531
GNAI3	0	0.000339391
OAT	0	0.000177815
WDR3	3	0.000271668
PKN2	0	0.000174361
WDR18	3	0.0000717
DGKA	2	0.000185178
PDIA5	2	0.000028
TBC1D22B	4	0.000106235
MYLK	4	0.000203983
MAP2K4	0	0.000240554
PACC1	3	0.000393609
SLK	0	0.000188501
CYB5R4	0	0.000248437

SEC61A2	2	0.000144108
TLE2	7	0.000157933
MTHFD2	3	0.000137072
SLC9A7	11	0.00025974
YBX1	4	0.000119579
PDE4A	1	0.0000491
PPP2R5A	0	0.00014532
KDM4A	2	0.000387279
ASPM	14	0.000215478
SPI1	1	0.000120841
ATXN3	3	0.00042134
TRMT11	3	0.000289985
ZFAT	2	0.000264611
MTFR1	3	0.000168437
FECH	4	0.00015351
DDX3Y	12	0.000165137
PFKP	2	0.00012951
IDI1	3	0.000131099
SP100	0	0.000171756
PLPP1	2	0.000109887
NEO1	2	0.0000376
TNFRSF1A	1	0.000227512
CACNB1	1	0.000151715
STOML1	3	0.0000253
TRO	7	0.000124708
RHOA	0	0.000318146
PRKCZ	1	0.000164992
ZFY	12	0.000165137
ROGDI	4	0.0000506
PDZD4	3	0.0000437
ATP2B3	7	0.00019313
ROCK1	0	0.000237936
HYAL2	0	0.000101768

PLEKHH3	7	0.000285341
MEF2A	0	0.000204574
TFE3	1	0.00025742
ACSL4	0	0.000193936
INPP5A	0	0.00024492
POLR1A	2	0.000257527
LAPTM4A	0	0.000209576
STON1-GTF2A1L	7	0.000172857
PPP2R5B	4	0.0000767
PYGM	1	0.000147238
MAST4	2	0.0000976
BCL3	1	0.0000779
KCNAB2	1	0.000197157
MAOB	7	0.0000476
RORA	3	0.000187978
DRD4	7	0.0000934
TGFBR3	3	0.000119437
PIGB	0	0.0000873
GNB5	3	0.000236805
RAB27A	0	0.000260622
LRP6	3	0.0000313
PHRF1	1	0.00024485
NUCB2	3	0.000130421
PFN2	3	0.0000239
SPTB	4	0.0000952
DAPP1	0	0.000153652
DGCR2	1	0.000262791
MNT	1	0.000195853
WIPI1	0	0.0000699
NDST1	1	0.0000994
ASNS	2	0.0000936
AP3M2	3	0.000231213
ST6GALNAC2	1	0.000114018

PABPC1	2	0.000195657
TESK2	0	0.000235453
SLC12A3	7	0.000237572
NCK2	2	0.00020224
WDR1	1	0.000375247
ARHGAP10	2	0.0000675
RPS6KA2	13	0.0000832
SEL1L	0	0.000342534
ATP6AP1	1	0.000304863
TCF3	4	0.0000593
TRIB2	3	0.000225076
FAM50A	3	0.000210442
FAM3A	1	0.0000985
CPSF1	1	0.000071
CYBRD1	0	0.0000235
ACTN1	1	0.000223852
ZFYVE26	2	0.000298451
RPS6KA6	7	0.0000254
EPN2	13	0.0001032
LIMS2	7	0.0000982
TFRC	4	0.0000902
UBE2D1	0	0.0000858
RHOBTB1	4	0.000189411
SMC1A	2	0.00014613
HSD17B10	3	0.000233918
HMMR	14	0.000176114
TRHDE	7	0.000147005
P4HA2	0	0.0000265
ACADVL	0	0.000197805
ACAP1	1	0.000200955
EVC	7	0.000115034
SIDT1	2	0.000247288
NDE1	1	0.000230673

MRVI1	0	0.00011179
PVR	1	0.0000236
SCARB1	1	0.0000422
MCM2	14	0.000113761
PANX2	1	0.0000699
SELENOO	1	0.000158735
LLGL2	1	0.000126201
PDE8A	2	0.000308396
NLE1	3	0.000155176
FNDC8	7	0.000255903
GSDMB	2	0.000108272
ADAM11	7	0.000332033
PPP2R3A	7	0.000117719
PTGS2	0	0.0000412
IGF2BP2	4	0.000130984
MAP3K13	17	0.000154847
ST6GAL1	2	0.000257796
TBX21	3	0.000128376
PICALM	0	0.000236574
MRPS34	3	0.000131414
CA12	7	0.000302205
MGLL	4	0.000100003
BCS1L	2	0.000284367
ZNF532	2	0.0000417
MYDGF	3	0.000200093
ARHGEF10L	1	0.0000707
TXK	3	0.000114775
EIF4G3	2	0.000203059
GTSE1	7	0.0000873
SEMA3C	0	0.0000733
TTC38	3	0.0000833
GRAMD4	0	0.000110112
CELSR1	11	0.000150857

TIMM21	3	0.000361544
VPS9D1	1	0.0000919
MARK3	0	0.000225198
FOSL2	1	0.000206673
FSCN1	1	0.0000299
ACTB	1	0.000128207
MOCOS	7	0.000276534
PLD1	0	0.000125267
SEC31B	1	0.000131493
ARHGAP15	0	0.000187458
EXOSC7	3	0.000369038
REXO2	3	0.000186485
PTPN23	1	0.000235391
FMO4	3	0.000237344
SLC46A1	2	0.0000557
PLXNA2	1	0.0000244
SPAG5	14	0.000191519
ANKRD13A	0	0.000309433
TPD52	3	0.00015357
ACACB	2	0.000154463
ICAM3	0	0.000160312
NT5C2	0	0.000249776
MCAM	7	0.00031064
GPC4	7	0.0000338
MBNL3	4	0.0000907
MAP2K7	4	0.000158481
NFKB2	1	0.000219791
UBE2T	14	0.000170649
PPP1R12B	1	0.000104692
IL4R	1	0.0000258
PAK3	7	0.0000999
EXOSC5	3	0.000201112
APBB1IP	0	0.000219242

LRCH4	1	0.000257201
GPR137B	2	0.000031
JADE1	0	0.000391243
SLC25A43	2	0.0000935
LAMP3	5	0.000106609
NEBL	18	0.000154847
ACER3	0	0.000237203
N4BP2	3	0.00022106
TIGAR	2	0.000243448
TULP3	2	0.000126252
SYNJ2	2	0.0000485
PPP2R5C	2	0.00038893
HOXA9	7	0.000188842
MLLT10	2	0.000369733
ZCWPW1	3	0.000216906
P2RY10	3	0.0000852
NRDC	0	0.000315198
TNRC6C	2	0.000261781
TP53INP2	1	0.0000433
OSBPL6	7	0.00012948
MKNK1	1	0.000136635
TNS1	4	0.000143116
CDC14A	3	0.000257631
RAPGEF3	11	0.000163673
LIPE	1	0.0000743
FDFT1	3	0.000337002
PGM1	0	0.00025272
DNM2	1	0.000303967
EPB41L2	2	0.0000942
MOXD1	7	0.0000259
RABL2B	1	0.000192563
PTPRH	7	0.000290076
DCT	7	0.000186295

RAB21	0	0.000372421
SLC4A4	3	0.0000818
SESN1	3	0.000205287
PSEN1	0	0.000327904
CLDND1	3	0.000285934
MOK	7	0.0000341
HSP90AA1	3	0.0000688
IGSF9B	1	0.0000321
NDC80	14	0.000144638
MAGI3	2	0.0000333
CXCL2	7	0.0000344
COL4A4	11	0.0000902
TCF7	2	0.0000769
IMPG2	3	0.000150641
EXD2	2	0.000312994
MEF2C	3	0.000118669
ZNF506	3	0.000330384
AACS	2	0.000190572
DELE1	2	0.000280399
PCDHGA2	7	0.000267329
PHLPP1	0	0.000117773
SMARCD3	0	0.0000445
STRADB	4	0.000245837
C1QTNF3	2	0.00017827
COL19A1	11	0.0000767
EPB41L3	0	0.0000355
COBLL1	11	0.000172515
DLG3	2	0.000065
TRAF5	3	0.00029218
MRPL22	3	0.000270109
NFE2L1	0	0.000320347
ITGB5	4	0.000305817
ERC1	2	0.000237908

RNF13	0	0.000219968
BCKDHB	3	0.000308742
PLOD1	1	0.0000759
P2RX5	11	0.0000507
OXCT1	3	0.000287483
SLC27A5	2	0.00013018
ZNF324	1	0.000343765
ZNF671	3	0.000316954
ZNF586	0	0.000301831
ZNF446	1	0.000148939
RPS5	3	0.0001063
FAT1	7	0.0000239
SMAP2	1	0.000147335
PPIE	3	0.0000789
NOA1	3	0.00034905
HAL	0	0.000158887
GSTP1	3	0.0000988
APLP2	0	0.000268529
MAPRE3	1	0.0000427
CD82	0	0.0001508
BCORL1	1	0.000179831
FCN1	0	0.0000767
OVGP1	2	0.0000565
MAP3K4	2	0.0003516
PILRA	1	0.000146794
ABCB1	3	0.0000722
ZNF213	1	0.000189198
CPNE3	0	0.000326107
CTTN	4	0.000347297
WNT11	7	0.000125984
MGST2	0	0.0000383
USP40	2	0.000257108
B4GALT1	1	0.000205627

CHMP5	3	0.0000605
IPO11	3	0.000316021
EIF2AK1	4	0.000210937
NME8	0	0.0000409
SNX10	0	0.000198443
MRPL28	0	0.000206116
ITPKC	0	0.000258571
CEACAM6	10	0.000225835
TXLNG	3	0.000171531
LAT2	0	0.000235145
ALG9	2	0.000289508
PPP1R15A	1	0.0000651
SRRT	1	0.000262637
BAX	3	0.000255466
PGS1	1	0.000150783
LPCAT2	0	0.000108722
GNAO1	7	0.000036
TXNDC16	3	0.000217798
NID2	7	0.000324159
GNAS	4	0.0000644
ERGIC2	3	0.000161033
AURKA	14	0.0000846
CASS4	0	0.000194048
AAMDC	3	0.000069
RFX2	1	0.0000922
SULT2B1	7	0.000260344
GP6	4	0.00013212
GNA11	7	0.0000244
TPX2	14	0.000218104
DOCK9	2	0.000228157
DOCK3	2	0.0000442
C3orf18	3	0.000247109
PPP1R13B	2	0.000168785

SMOX	4	0.0000453
SIGLEC1	5	0.000136027
NSFL1C	0	0.000190044
MAVS	1	0.000266125
F11	7	0.000288576
TESC	4	0.000166327
SIRPG	2	0.0000651
MAPKAPK5	2	0.00040635
P2RX7	2	0.0000453
TASP1	3	0.000332241
OAS1	5	0.000164191
KIF16B	2	0.0000934
CHGB	7	0.000290923
ERP29	2	0.0000374
FUS	2	0.000190287
GRAMD1A	1	0.000195498
HEPH	19	0.000154847
GMIP	1	0.000176947
BIRC5	14	0.000186684
MLF2	0	0.000197494
OTUB2	7	0.000297985
ZBTB25	2	0.000287478
ARHGAP4	1	0.000148045
DHX32	3	0.000199316
BLVRB	4	0.000137897
PCBP4	3	0.0000357
NUDC	3	0.000180096
MAEA	1	0.000259068
ICAM1	1	0.000097
IRAK3	0	0.000088
LYZ	0	0.0000285
PDCD7	3	0.00031444
FLT3LG	1	0.0000441

GOLGA3	2	0.000286151
CD209	7	0.000161192
MCOLN1	4	0.0001029
USP48	0	0.000411037
EFNB1	1	0.0000699
PDPR	1	0.0000379
AARS1	3	0.000178492
TNRC6A	2	0.000280527
DLL3	7	0.000284606
NAT14	7	0.0000521
POU4F3	7	0.000269255
NLRC4	0	0.000136718
PUS7	3	0.00010121
IL5RA	13	0.000217127
ABCC6	1	0.0000358
CMTM6	0	0.000186526
ITGA6	3	0.000159185
MAP3K20	0	0.0000518
SEL1L3	2	0.000210406
CDV3	0	0.000226522
ALKBH5	0	0.000234544
SPAG7	3	0.000082
ESR1	7	0.0000323
CD200	11	0.0000872
CMA1	7	0.000228117
PPP2R3C	0	0.000150471
HAUS4	0	0.000038
OSGEP	3	0.000350372
RNF31	1	0.000198371
RPGRIP1	0	0.0000334
TGM1	1	0.0000331
CAPN3	2	0.000120678
TBX15	7	0.00029792

PHGDH	7	0.0000494
TEKT2	7	0.000119937
RFFL	1	0.000224334
DPYSL2	0	0.0000895
NUP50	0	0.000285181
COMT	1	0.000229338
ADA2	1	0.0000815
VNN3	0	0.0000549
LRRFIP2	0	0.000335353
XYLB	2	0.0000236
HDAC6	2	0.00022256
CDC6	14	0.000185643
CBX5	2	0.000184104
FMO2	7	0.000246636
MAP3K1	0	0.000340407
DHPS	2	0.000179607
PTGS1	4	0.000199107
SH2D3C	1	0.000237002
NANS	0	0.000288692
TBC1D2	1	0.000204585
IKZF5	3	0.000182539
BLNK	11	0.000240193
CYP26A1	7	0.00025419
TDRD1	7	0.0000281
CREM	3	0.000249729
HIVEP1	0	0.000168849
TREM2	7	0.0000249
CRISP3	10	0.000115779
FKBP5	0	0.0000538
SRPK1	0	0.000121716
TMEM14A	3	0.000156897
HSP90AB1	2	0.000174587
HNRNPH3	3	0.0000311

ACOT7	7	0.000162464
SH3GLB1	0	0.000240058
CDC7	14	0.0000737
SYDE2	3	0.00004
PCSK5	2	0.000101156
ABLIM1	2	0.000199327
ERMP1	2	0.00023156
MACROH2A2	2	0.000032
MAST3	1	0.000205661
OCEL1	1	0.0000574
MYO9B	1	0.000248782
KCNK6	1	0.000176938
FBXL19	1	0.000192189
STX1B	7	0.000184371
ATP5F1D	1	0.0000496
PRKY	12	0.00016609
IGFALS	7	0.0000951
MARCHF2	4	0.000137204
TECR	2	0.000123923
CDC34	4	0.000248957
MTAP	3	0.000356103
CEP170B	7	0.000276003
POLR2E	1	0.000168134
POLRMT	1	0.00014731
RASSF7	3	0.000125786
GADD45B	0	0.0000383
MADCAM1	7	0.0000891
MKNK2	1	0.000168365
SNAP29	0	0.000275268
SMARCB1	1	0.0000823
P2RX6	7	0.000265754
BCL2L13	4	0.000139488
SUSD2	7	0.000170066

YPEL1	2	0.00013513
MAPK1	0	0.000323764
GGT1	1	0.0000244
PPM1F	1	0.000155115
SLC35E4	1	0.0000332
CRYBB3	7	0.000074
CYTH4	0	0.000237814
MFNG	1	0.000255711
LRP5L	2	0.000101546
GRK3	2	0.000200167
SH3BP1	1	0.000123847
SEZ6L	7	0.0000452
LGALS1	3	0.0000427
HPS4	2	0.000350814
PIK3IP1	3	0.000159229
SRRD	4	0.00032171
PATZ1	2	0.00026312
TRIOBP	1	0.000221507
TFIP11	0	0.000154841
CRYBB1	7	0.000217065
CCDC134	1	0.000175559
PICK1	1	0.000126228
DMC1	7	0.0000287
JOSD1	3	0.000287188
FBXO7	4	0.00017224
RAB36	1	0.0000427
CYB5R3	1	0.0000937
LMF2	1	0.000202354
RHBDD3	3	0.000180887
PACSIN2	0	0.000217594
RASL10A	7	0.000316005
TOM1	1	0.000178236
CHKB	1	0.0000496

THOC5	1	0.000253073
MCM5	2	0.000221306
APOBEC3H	14	0.0000634
TSPO	0	0.0000625
TTLL12	1	0.000204358
CBX7	1	0.000257258
ZMAT5	0	0.000254114
RBFOX2	7	0.000229331
SYNGR1	1	0.0000265
ASCC2	4	0.000213931
APOL1	5	0.000111038
MYH9	1	0.000164816
CACNA1I	1	0.000043
TXN2	1	0.0000286
TNRC6B	2	0.000304791
SGSM3	2	0.000138004
IFT27	2	0.000300776
KIAA0930	1	0.000206787
NCF4	1	0.000154704
CSF2RB	1	0.000176776
SLC25A17	2	0.000374101
KCTD17	3	0.000101055
ST13	4	0.000124679
CHADL	7	0.000147709
PHF5A	3	0.000168767
POLR3H	2	0.000251017
PMM1	1	0.000173861
DESI1	2	0.000227301
BRD1	2	0.000358999
MLC1	3	0.0000445
HDAC10	1	0.000117648
KCNK10	7	0.0003168
ABHD4	1	0.000182336

KHNYN	0	0.000344646
CTSG	10	0.00018894
GZMH	3	0.0000686
GZMB	3	0.0000419
RBM23	0	0.00030023
PRMT5	0	0.000125333
AP4S1	3	0.000292584
POLE2	14	0.000128125
VCPKMT	0	0.000189412
SOS2	0	0.000172909
NIN	0	0.000328063
PYGL	0	0.000147441
CDKN3	14	0.0000817
CGRRF1	3	0.000183565
PLEK2	4	0.0000875
VTI1B	4	0.00020332
TIMM9	3	0.0000925
DAAM1	3	0.000286758
SPTLC2	0	0.000272672
LGMN	7	0.0000344
ITPK1	1	0.000189814
DHRS7	0	0.000232107
ASB2	20	0.0000673
CEP128	3	0.000169064
HIF1A	0	0.000186275
SLC10A1	7	0.000226449
SLC8A3	7	0.000240332
DICER1	0	0.000267242
ZFYVE21	0	0.00021315
TCL1A	11	0.0000357
TELO2	1	0.000141575
PCNX1	0	0.000229882
PAPLN	7	0.000270424

PSMB5	3	0.000356379
YY1	0	0.000463792
CCNB1IP1	3	0.000247372
APEX1	3	0.000206954
CHD8	2	0.000361661
PCK2	1	0.000141744
PRORP	3	0.0000799
NFKBIA	0	0.000165854
EMC9	3	0.0000976
PSME2	5	0.0000886
TM9SF1	0	0.000244426
GMPR2	0	0.000335654
RABGGTA	1	0.000130206
PLTP	7	0.000146586
TRPC4AP	0	0.000456952
PYGB	0	0.0000968
ABHD12	2	0.000267017
CD40	11	0.0000883
UQCC1	2	0.000304375
ZMYND8	2	0.000302679
IFT52	3	0.000201587
NDRG3	0	0.000266089
RAB5IF	0	0.000163267
TPD52L2	0	0.000273162
TUBB1	4	0.000296501
MTG2	1	0.000218658
MRGBP	3	0.000294987
TCFL5	3	0.000137325
SLC17A9	1	0.0000648
BIRC7	7	0.0000615
ARFGAP1	1	0.000270424
RNF24	1	0.000126903
ARFRP1	2	0.000325928

RASSF2	0	0.000235393
CDS2	0	0.0002245
SNPH	2	0.0000568
SIRPB1	1	0.000129971
MYL9	4	0.0000392
HCK	0	0.000183345
TLDC2	7	0.000186393
NOP56	3	0.00030452
MANBAL	2	0.000314406
MAPRE1	0	0.000344364
OXT	7	0.0000783
CHMP4B	0	0.0000714
BPI	10	0.000225835
CST3	1	0.000027
ASIP	7	0.0000796
AHCY	1	0.0000804
DNTTIP1	0	0.000181577
PIGU	2	0.00031534
APMAP	0	0.000137202
ZNF516	1	0.000206812
RBFA	2	0.000296913
LPIN2	1	0.000154248
CEP76	3	0.000308908
SMAD7	3	0.000107299
POLI	3	0.000192723
RIOK3	4	0.000200603
CSTF2	3	0.000310557
VSIG1	3	0.000122188
STS	0	0.0000567
PGRMC1	4	0.000178371
POLA1	3	0.000288716
MID1	7	0.000216917
ALG13	2	0.000241464

TLR8	0	0.000171252
AMMECR1	3	0.000334199
WDR13	0	0.00018078
SRPX	7	0.000157753
STAG2	0	0.000191445
ABCD1	1	0.00017509
CCDC22	3	0.000187025
CACNA1F	7	0.000097
SYP	7	0.0000259
PLP2	0	0.0000236
BMX	0	0.000024
PLS3	7	0.0000275
NAA10	1	0.0000998
RENBP	1	0.00015227
SMARCA1	7	0.000039
MTMR8	2	0.000266987
ZC3H12B	2	0.0000257
KCND1	1	0.0000316
PIM2	1	0.000153653
SCML2	2	0.0000366
PQBP1	3	0.000294277
GATA1	4	0.000172412
SMS	0	0.000250446
CD99L2	2	0.000336835
RP2	0	0.000207367
JADE3	11	0.0000764
CDK16	1	0.000118083
USP11	2	0.000176499
PCYT1B	4	0.000109186
CD40LG	3	0.00011325
PIN4	3	0.000204921
KLF8	11	0.0000507
SYTL4	4	0.00010914

ZDHHC15	7	0.0000957
CENPI	14	0.0000306
DRP2	7	0.000170417
GLA	0	0.000157276
BEX4	3	0.000144437
RUBCNL	0	0.0000247
FGF14	7	0.000297104
HTR2A	7	0.000255968
TNFSF13B	0	0.0000752
MLNR	7	0.000226609
CAB39L	3	0.000187845
DNAJC3	0	0.000202554
UGGT2	3	0.0000292
ARHGEF7	2	0.000330713
SLC25A15	3	0.000287379
RGCC	4	0.0000335
VWA8	2	0.000326904
KATNAL1	3	0.0001797
DHRS12	0	0.0000402
MEDAG	7	0.00028476
TSC22D1	2	0.0000747
OLFM4	10	0.000102428
ZNF629	2	0.0000579
CORO1A	1	0.000215256
MAPK3	0	0.0000852
LYRM1	0	0.000306473
NUTF2	3	0.000230792
TSNAXIP1	7	0.0000526
NFAT5	2	0.000178159
LONP2	3	0.000354892
N4BP1	0	0.000293954
PLLP	7	0.000144724
DHODH	2	0.000278304

ACD	1	0.000134222
ZNF821	2	0.000118328
SETD6	3	0.000338482
SMPD3	13	0.000165437
SLC7A6	2	0.000234266
PLA2G15	1	0.0000953
FA2H	7	0.000278684
WDR59	2	0.00032051
MLYCD	2	0.00029523
SEC14L5	4	0.0000334
USP10	0	0.000183699
CRISPLD2	1	0.0000333
ABCC1	1	0.00019764
NOMO3	1	0.0000389
MTHFSD	2	0.000402264
HAGHL	2	0.0000905
SLC7A5	4	0.0000679
METRN	2	0.0000614
MEFV	1	0.000173918
CRYM	7	0.0000753
PIEZO1	1	0.000156504
GSPT1	4	0.000179104
ZNF174	3	0.000361642
CLUAP1	3	0.000181569
PRSS33	13	0.000146081
ELOB	4	0.000058
CPPED1	0	0.000155676
USP31	2	0.000250582
XYLT1	1	0.000230462
PYCARD	0	0.00013649
STX4	0	0.000249196
BCKDK	1	0.000218132
NOMO1	2	0.00015455

TMC5	21	0.000139022
RNF40	1	0.000276883
AQP9	0	0.000143545
CORO2B	7	0.0000262
TRIP4	2	0.000366958
MTFMT	0	0.000318932
ACSBG1	7	0.0000814
HOMER2	2	0.0000301
ZNF106	0	0.0003041
DMXL2	0	0.000169249
VPS18	0	0.000269177
CSPP1	3	0.000281295
ZDHHC2	3	0.0000672
NBN	0	0.000180228
CPQ	0	0.000235528
IMPAD1	3	0.000379279
NIPAL2	3	0.0000238
IKBKB	2	0.0002996
GDAP1	3	0.000179619
IL7	11	0.000041
TRPS1	0	0.000287985
SPAG1	0	0.0000249
TSTA3	4	0.0000274
EEF1D	3	0.000162895
SQLE	3	0.0000519
SLC39A14	2	0.0000394
GSR	0	0.000287319
TNFRSF10A	3	0.000271719
ERICH1	0	0.0000405
ASAH1	0	0.00018825
BNIP3L	4	0.000153521
MAN2B1	1	0.000203812
KCNN4	1	0.0000246

NUCB1	1	0.0000877
DHDH	7	0.000146216
ECH1	1	0.0000795
NFKBIB	0	0.000199148
SARS2	2	0.0000248
RELB	1	0.000057
CLASRP	1	0.000219638
FCGRT	0	0.000171751
ARHGEF18	1	0.000132089
PPP1R13L	7	0.000172885
ERCC2	1	0.000117274
KLC3	4	0.0000262
SF3A2	1	0.000204716
AMH	7	0.0000369
LYL1	4	0.000124469
OAZ1	4	0.000195776
STX10	0	0.000200248
FCER2	11	0.0000339
TLE6	7	0.000297042
PTOV1	1	0.000120535
TLE5	1	0.0000489
MED25	1	0.000149903
LILRA1	1	0.000188054
SNAPC2	1	0.0000276
C19orf53	3	0.000121258
TIMM44	3	0.000233545
IL27RA	3	0.000189537
ASF1B	1	0.000053
C19orf44	7	0.0000372
OLFM2	2	0.0000251
RPS16	3	0.0000345
FBL	3	0.000145548
CLC	22	0.000154847

PLD3	1	0.0000934
PIAS4	1	0.000156458
NUMBL	1	0.000175519
SHD	7	0.000161572
POLR2I	3	0.0000743
SLC1A5	4	0.000144913
TJP3	2	0.0000448
CCDC9	1	0.000100504
HNRNPUL1	1	0.000276774
FZR1	1	0.00011472
BBC3	0	0.0000672
CEACAM4	1	0.0000941
PLIN3	0	0.000146187
SIGLEC8	13	0.000243751
CD79A	11	0.0000761
ICAM4	4	0.0000343
NKG7	3	0.0000698
CD33	1	0.0000697
BABAM1	4	0.000166279
TYK2	1	0.000303448
CDC37	3	0.000239741
NAPA	1	0.0000459
RABAC1	3	0.0000385
PTPRS	7	0.0000318
CNFN	7	0.0000658
CYTH2	1	0.000296369
GRWD1	2	0.000169891
GRIN2D	7	0.000242265
CCDC114	7	0.000276028
LIG1	2	0.000154637
SIGLEC5	1	0.0000237
RAB3D	1	0.000180711
TMEM205	3	0.000184308

PLPPR2	1	0.000148692
LILRB5	7	0.000112016
KLF1	4	0.0000903
PRPF31	3	0.00025916
TFPT	3	0.000199031
RPL18A	3	0.0000831
ELL	1	0.000204826
UPK1A	7	0.0000246
ETV2	7	0.00008
HAMP	7	0.0000447
LSR	2	0.0000341
FKBP8	4	0.000166342
HPN	7	0.000282731
PBX4	1	0.0000636
ERF	1	0.000156153
ATP13A1	1	0.000212286
ZNF574	1	0.0000969
ZNF85	3	0.000155516
ETHE1	3	0.000166229
RUNDC3B	7	0.0000916
CFAP69	7	0.000324111
CDK6	3	0.000227335
NAMPT	0	0.000134504
PIK3CG	0	0.000327637
ITGB8	23	0.000207865
HBP1	0	0.000257711
DUS4L	3	0.000287754
MTPN	0	0.000199174
ZC3HAV1	0	0.0000912
ADAP1	1	0.0000936
CAV1	7	0.000125359
DNAJB6	0	0.00024342
HOXA6	7	0.0000803

BRAT1	1	0.000144645
IQCE	1	0.000140434
VIPR2	7	0.000152104
HIBADH	3	0.000349087
TAX1BP1	3	0.00018348
CPVL	0	0.0000522
CHN2	2	0.0000599
ABHD11	0	0.0000658
CHCHD2	3	0.000193166
CCL24	7	0.0000535
EIF3B	4	0.000124839
SNX8	1	0.0000594
NUDT1	3	0.0000578
TFR2	4	0.0000535
FSCN3	7	0.0000315
MOSPD3	1	0.0000294
PCOLCE	7	0.000152566
FBXO24	7	0.000100237
IMPDH1	1	0.00021707
AGFG2	2	0.000141158
SERPINE1	7	0.0000352
PLOD3	1	0.000172108
CLDN15	1	0.000190321
EZH2	2	0.0000362
CEP41	2	0.000128859
ZNF862	2	0.000326991
TSPAN13	11	0.0000311
TMEM176B	6	0.000154847
GLI3	7	0.000323583
BLVRA	3	0.0000863
AEBP1	7	0.0000416
POLD2	2	0.00016016
FSD1L	3	0.0000946

TMEM245	2	0.000273437
MEGF9	0	0.000154292
CORO2A	2	0.000043
TGFBR1	0	0.000229482
C5	3	0.0000267
TLE4	0	0.000333865
PTGR1	7	0.000292158
SUSD1	2	0.000133927
DNM1	7	0.000293623
RLN1	3	0.0000789
KDM4C	2	0.000342587
TESK1	1	0.000122666
DDX58	5	0.000177179
PIP5K1B	2	0.0000313
GLIS3	7	0.0000253
BAG1	4	0.000212533
DNTT	7	0.000245448
HPS1	4	0.000101969
PHYH	3	0.0000659
RASSF4	1	0.000073
DNMBP	2	0.0000318
CUBN	2	0.0000964
TRDMT1	2	0.000275394
ATE1	3	0.000400298
PLEKHA1	3	0.000241996
PALD1	7	0.000297493
UNC5B	7	0.000265116
CDH23	1	0.0000564
VSIR	1	0.000242464
LIPA	3	0.0000239
TWNK	3	0.000254798
SFXN3	2	0.00023556
FBXW4	2	0.000305233

FGF8	7	0.000266834
NPM3	3	0.000156511
ARHGAP21	2	0.000163566
CUEDC2	3	0.000270801
LHPP	1	0.00015741
SH3PXD2A	2	0.0000269
CCNY	0	0.000393776
UBE2S	0	0.0000634
RPL28	1	0.0000242
ZMIZ1	1	0.00022373
PPIF	1	0.000160847
PBLD	0	0.0000305
TBC1D12	3	0.0000243
FBXL20	1	0.000248278
UBTF	2	0.000350037
PSMD3	3	0.00022649
RAPGEFL1	1	0.000046
RGS9	3	0.000111517
RNF43	7	0.0000302
WNT3	11	0.0000662
P2RX1	1	0.0000252
TUBD1	3	0.000322863
KPNB1	0	0.000267555
GOSR2	2	0.000307955
PNPO	2	0.000284605
CBX1	0	0.000223774
GALK1	0	0.000126469
ENO3	3	0.0000984
PFN1	1	0.000118247
RAI1	1	0.000170152
NUP88	3	0.00028105
C1QBP	3	0.000258665
CPD	0	0.00017402

GOSR1	2	0.000369105
CCDC47	0	0.000375357
SMARCD2	0	0.000213193
ICAM2	3	0.000184566
B9D1	24	0.0000598
LGALS3BP	5	0.0000377
CCL2	5	0.00011371
CCL8	5	0.00010015
CCL1	7	0.000155379
DHX58	5	0.000209209
KAT2A	1	0.000181448
HSD17B1	7	0.000124391
EZH1	0	0.000329303
PPP1R9B	1	0.000168896
SGCA	7	0.00024516
VAT1	1	0.0000641
ALOX12	4	0.000123355
HDAC5	1	0.00022414
ABCC3	4	0.0000677
DUSP3	0	0.000114719
CACNG1	7	0.000101131
SLC16A6	0	0.00021246
PRKAR1A	0	0.000210532
MMD	4	0.000112791
DPH1	1	0.000181899
MAP2K6	0	0.000164228
SLC9A3R1	1	0.00018191
MYH3	2	0.000140097
NAT9	3	0.000307817
TMEM104	1	0.000243938
TMEM97	3	0.0000279
PMP22	20	0.0000673
UNC119	0	0.000225708

ALDOC	1	0.0000238
PHF12	1	0.000239116
USP46	3	0.000131471
LAMTOR3	0	0.000178182
ELF2	0	0.000288055
NDUFC1	3	0.000280437
TBC1D9	2	0.0000357
INPP4B	3	0.000155158
GAR1	3	0.0000515
SLC2A9	1	0.0000485
NEIL3	14	0.000117589
BST1	0	0.000185626
RAPGEF2	0	0.00020714
SNX25	3	0.000154505
KLHL5	3	0.000204687
FAM149A	7	0.000271504
NCAPG	14	0.000181412
PPARGC1A	7	0.0000713
HTATIP2	0	0.000236856
TECTA	7	0.000136067
CRTAM	3	0.0000246
B3GAT1	3	0.000052
VWA5A	3	0.0000848
SIAE	2	0.0000299
SNX15	1	0.0000904
DTX4	1	0.0000236
EHD1	1	0.000182887
PUS3	3	0.000218331
NRXN2	7	0.000303316
MS4A6A	0	0.000100418
ST3GAL4	1	0.0000759
CPT1A	1	0.0000255
CCDC86	1	0.0000515

HPX	7	0.000224668
ANAPC15	3	0.0000574
PANX1	3	0.000188091
ARHGEF17	7	0.0000307
RNF141	0	0.000176839
CEP126	3	0.0000639
EIF4G2	0	0.000315959
GALNT18	7	0.000270747
UPK2	7	0.000165067
NECTIN1	1	0.0000455
HIPK3	0	0.000224739
COMMD9	3	0.000250755
SLC15A3	0	0.000135112
CD5	2	0.000047
SLC22A18	1	0.0000236
C11orf21	1	0.000123778
SOX6	4	0.000054
NUP98	0	0.000302886
CHKA	2	0.0000968
EXPH5	3	0.0000267
POU2AF1	11	0.000177175
P3H3	7	0.0000291
PPFIBP1	2	0.0000912
CLEC2B	0	0.0000775
SELPLG	1	0.000161562
CORO1C	0	0.000213208
ASIC1	7	0.000293045
SLC11A2	2	0.000331456
MVK	2	0.000108806
CAMKK2	1	0.000228758
BIN2	0	0.000369069
IL23A	3	0.0000871
ATP5F1B	3	0.000185414

BCL7A	11	0.000029
CYP27B1	7	0.000252508
LIN7A	0	0.000129194
ACSS3	0	0.0000972
TNS2	7	0.000326307
PPM1H	1	0.000027
LTA4H	0	0.0000441
WNT5B	7	0.0000271
ITFG2	2	0.000309657
FOXM1	7	0.000140804
PRR4	2	0.0000257
PARP11	3	0.000252791
ARPC3	0	0.00015882
GPN3	3	0.0001429
MYL2	7	0.000254597
RAD51AP1	14	0.0000724
CUX2	7	0.000317873
MANSC1	0	0.0000328
DUSP16	2	0.000149624
ALDH2	1	0.0000315
CDKN1B	0	0.000305795
LTBR	1	0.000145815
OGFOD2	0	0.000106613
CDK2AP1	0	0.0000271
OAS3	5	0.000174972
OAS2	5	0.000197275
ART4	21	0.0000605
MGP	7	0.0000963
RASAL1	7	0.000326921
ARHGDIB	0	0.000239462
VDR	1	0.000161921
STX2	2	0.0000358
TBC1D30	0	0.0000254

RAB5B	1	0.000158785
MDM1	3	0.000282359
GAPDH	0	0.000111816
NOP2	2	0.000107752
CHD4	2	0.000302395
ACRBP	4	0.0000636
UHRF1BP1L	0	0.000226593
GNB3	7	0.000165631
CDCA3	7	0.000066
CHPT1	4	0.0000487
GNPTAB	3	0.000268148
ATN1	1	0.0000919
C12orf57	3	0.000067
LPCAT3	1	0.000201893
NT5DC3	2	0.0000247
NANOG	7	0.000274001
LDHB	3	0.000103262
AICDA	7	0.000210253
RAB35	0	0.000317234
PHC1	2	0.000206395
COX6A1	3	0.000102376
TDP2	0	0.000201634
DSE	0	0.000151767
MAK	0	0.000028
TMEM14C	3	0.0000845
GCNT2	2	0.0000239
NEDD9	0	0.000200061
FAM184A	3	0.0000454
MAN1A1	0	0.0000826
SERINC1	0	0.000155288
UST	2	0.0000378
RHAG	4	0.0000709
KCTD20	0	0.000340166

SOD2	0	0.000132369
MCM3	3	0.000140118
FBXO9	4	0.000135973
CD83	3	0.0000849
MDN1	2	0.000252826
BACH2	2	0.00011202
TREML2	0	0.000131823
TSPO2	4	0.0000679
KHDRBS2	11	0.000158044
FBXL4	2	0.000277617
PRDM13	7	0.00021446
E2F3	0	0.00024772
PTP4A1	0	0.0000271
ALDH5A1	4	0.000049
VNN2	0	0.0000576
RPS12	3	0.0000577
C6orf62	0	0.000321303
SOBP	7	0.00013212
SNX3	4	0.000261342
SLC16A10	3	0.0000711
ADGRG6	7	0.0000591
PHACTR2	2	0.000230786
EPM2A	2	0.000203252
SLC39A7	0	0.000263558
CCR6	2	0.0000343
CUTA	3	0.000142156
PACRG	7	0.000206469
TFEB	1	0.000239258
CCND3	1	0.000103403
BYSL	3	0.0000721
TBP	2	0.00035341
GUCA1B	2	0.0000292
BICRAL	2	0.000255143

PTK7	7	0.0000281
DNPH1	3	0.0000955
DUSP22	0	0.000351304
GMDS	2	0.000193186
TTK	14	0.000185415
SLC29A1	13	0.000202484
CLIC5	2	0.000027
ENPP5	3	0.0000748
LY86	3	0.0000735
TENT4A	2	0.000264946
DAP	1	0.000123146
GZMK	3	0.0000238
APBB3	1	0.000220828
SPARC	4	0.000188181
IK	0	0.000220652
HMGCR	0	0.000253579
PCDHB3	7	0.00025084
PDE8B	7	0.000191114
PCDHB15	7	0.00027543
HAVCR1	2	0.0000614
ITK	3	0.000262404
RNF130	0	0.000243305
BTNL8	0	0.000038
TTC1	3	0.000329403
MSH3	2	0.000245739
RASGRF2	2	0.000163029
DROSHA	2	0.000290293
LMNB1	0	0.0000825
ARRDC3	0	0.0000943
NPR3	7	0.000243373
SLC27A6	7	0.000270298
IL4	23	0.0000676
RAD50	2	0.000258551

ST8SIA4	0	0.00019314
GNPDA1	3	0.000289103
SEC24A	3	0.000257966
ERGIC1	1	0.000176296
CPEB4	0	0.000175182
HRH2	1	0.0001156
EHHADH	17	0.000154847
SELENOK	4	0.0000645
BCL6	1	0.000102979
ARL6	3	0.000161026
CD86	0	0.0000906
KPNA1	0	0.000354371
SLC25A36	3	0.000189811
GRK7	7	0.000265312
KAT2B	4	0.000122631
SERPINI2	0	0.0000401
PFKFB4	1	0.000215651
USP4	0	0.000285568
ACAP2	0	0.000265381
ECT2	3	0.0000605
GNAI2	1	0.000266144
USP9Y	12	0.00016609
HYAL1	7	0.000146827
TUSC2	0	0.000229539
NPRL2	3	0.000320592
CYB561D2	3	0.000363061
HHLA2	7	0.000154901
C3orf52	7	0.0000921
FRMD4B	0	0.00010395
SLC41A3	2	0.000224006
ATP6V1A	0	0.000233261
KIAA1257	1	0.000103305
NEK11	3	0.0000268

CISH	1	0.0000358
ACVR2B	2	0.00010547
ABCC5	0	0.0000883
ABHD14B	1	0.00013212
EIF1B	4	0.0000775
PLCH1	7	0.0000491
VIPR1	1	0.0000908
ZBTB47	0	0.0000698
FOXP1	2	0.000301159
KANSL3	2	0.000249777
IL1A	7	0.000134689
FAHD2A	2	0.000323665
EPB41L5	3	0.000176557
DNAJC27	2	0.000315218
OTOF	5	0.0000852
CENPA	14	0.0000605
ACVR1	2	0.000237112
ITGA4	3	0.000210328
PPM1G	3	0.000102912
PCSK4	7	0.000244725
IFIH1	5	0.000198296
RPS15	3	0.000031
GCA	0	0.000125958
INO80B	3	0.0000339
PCGF1	3	0.000167137
SPTBN1	2	0.000204351
RTN4	0	0.00022702
LOXL3	1	0.0000539
DOK1	0	0.000273673
GALNT3	0	0.000180059
WDR75	3	0.000337705
FANCL	3	0.0000289
GGCX	2	0.00032303

GNLY	3	0.0000269
CHST10	24	0.0000746
PDCL3	3	0.000154134
IL1R2	0	0.0000658
IL1R1	1	0.0000452
IL1RL1	13	0.0000419
IL18R1	0	0.0000363
CNPPD1	4	0.0000848
UXS1	2	0.000307463
HDLBP	0	0.00012765
PASK	2	0.000103506
TPO	7	0.000285977
ODC1	4	0.0000766
QPCT	0	0.000116201
LCT	7	0.000294255
PLCL1	3	0.000049
PLEK	1	0.000146585
AAK1	2	0.000228902
TRAK2	4	0.00019406
ARID3A	1	0.000190158
EPHA4	3	0.0000507
PARD3B	7	0.000112737
FARSB	3	0.000313251
BCL9	2	0.0000675
MORN1	2	0.000129588
CEP104	2	0.000256876
MRPL37	3	0.000281297
QSOX1	1	0.000182501
PHF13	2	0.000272367
AMPD2	1	0.000153149
MECR	2	0.000227062
WDR77	2	0.000318454
CAPZA1	0	0.000166982

RNF19B	1	0.000181358
TRIM62	1	0.000193223
ARHGEF2	1	0.000343211
MEF2D	1	0.000257035
DOCK7	3	0.000123141
SRM	1	0.0000282
FBXO2	7	0.0000319
FBXO6	5	0.000142697
C1orf21	3	0.000115811
SWT1	14	0.000122602
DNAJC6	14	0.000117589
MFN2	1	0.0000241
PRG4	7	0.000068
MIIP	0	0.0000892
SMG7	2	0.000285082
NCF2	0	0.000221995
PLA2G4A	0	0.000112421
RGS2	0	0.0000771
AGMAT	3	0.0000719
CRYZ	3	0.0000242
PHTF1	0	0.000216285
ZBTB17	0	0.000258647
CD58	0	0.000248196
CD2	3	0.0000973
TTF2	2	0.000216891
TMEM9	3	0.0000383
OSCP1	7	0.0000282
EXOC8	0	0.000293802
C1orf109	3	0.000323816
RRAGC	0	0.000301896
LGALS8	0	0.000363448
MTR	2	0.000341422
MYCL	1	0.0000735

SIPA1L2	0	0.0000398
RLF	0	0.000254272
KMO	11	0.000122832
RIMS3	2	0.0000468
SLAMF1	2	0.000205044
PADI2	1	0.0000251
KDM5B	0	0.000314178
UAP1	3	0.000253713
CTBS	0	0.000164098
SSX2IP	3	0.000135462
ZNHIT6	3	0.000263346
GBP3	5	0.0000267
GBP1	5	0.000207845
GPR89A	3	0.00025051
ECE1	1	0.00017289
ID3	11	0.0000292
CR2	11	0.000109096
CD46	0	0.000222703
APH1A	0	0.000348026
SLC2A1	4	0.000227591
CDC20	14	0.000182738
MPL	7	0.000058
ARTN	7	0.000275092
ATP6V0B	0	0.000215283
PTCH2	7	0.000187281
F3	7	0.000281667
FASLG	3	0.0000891
TNFSF4	4	0.0000956
PRDX6	4	0.0000367
RCAN3	2	0.000165307
MAN1C1	2	0.0000708
NEK2	14	0.000175231
RPS6KA1	1	0.000343722

NENF	3	0.000107958
CENPF	14	0.000245529
RPA2	3	0.000264321
2-Mar	7	0.0000464
SLC5A9	7	0.0000236
OSBPL9	0	0.000371698
TXNDC12	0	0.000240063
ESYT2	3	0.000329962
CTSD	1	0.0000755
STK11	4	0.000120604
KMT2A	2	0.000226411
IFT46	3	0.000227611
MMP8	10	0.000169992
APOA1	7	0.0000441
ZNF541	7	0.000281634
KIF14	14	0.0000585
ATF6	0	0.000250397
MREG	2	0.000253384
KLF7	1	0.000197165
C1orf54	7	0.0000239
LRMP	0	0.00024025
SPCS2	3	0.0000914
HMGN3	3	0.000221196
UBE3D	2	0.000264747
SGIP1	21	0.0000758
TNFAIP3	2	0.0000811
MYB	2	0.0000285
SGK1	0	0.000117759
FBXL5	0	0.000172088
VAMP8	3	0.00012629
FOXO3	4	0.000185395
ARMC2	2	0.000173067
ABCG2	4	0.0000529

CCNI	4	0.0000716
RARRES1	7	0.000261282
EEF2KMT	3	0.000217804
UBN1	1	0.00021309
UCHL3	3	0.000224609
HS1BP3	1	0.000250642
DNAH7	7	0.0000254
NDUFB3	3	0.000054
SATB2	7	0.000162736
TRPM6	0	0.0000392
KLF9	3	0.000138446
ITGB1BP1	3	0.000429226
C1orf198	4	0.000123523
HEATR1	3	0.000338127
PTBP3	0	0.000290728
RAD23B	0	0.000287475
CTNNAL1	4	0.0000468
ABITRAM	0	0.000271271
WDR34	11	0.0000353
SET	3	0.000177013
CNTRL	2	0.00031204
NEK6	1	0.000151504
NDUFA8	3	0.000271444
HDHD3	2	0.0000903
SLC46A2	1	0.000029
HSDL2	0	0.000190932
NR4A3	7	0.0000517
INVS	2	0.000362226
DENND1A	1	0.00018716
CSF3R	1	0.000166693
VPS4B	0	0.000327741
ZBTB45	3	0.000154682
YLPM1	2	0.000305261

DCAF4	2	0.0000366
IFI27L2	3	0.0000716
BBOF1	4	0.000182366
ACYP1	2	0.000236235
IRF2BPL	1	0.0000835
ACOT2	3	0.000119231
AREL1	0	0.000316259
TTLL5	2	0.000232928
FLVCR2	0	0.0000436
GPR68	3	0.0000655
ESRRB	7	0.000242563
RHOQ	0	0.00025024
KLHL29	7	0.000202799
FKBP1B	4	0.0000418
YPEL5	0	0.00019694
CNRIP1	7	0.000115297
BCL11A	11	0.000177017
EPCAM	7	0.000187874
OGFRL1	0	0.000150597
IFIT3	5	0.000168759
IFIT2	5	0.000155324
PYROXD2	2	0.000173143
CNNM1	7	0.000259377
MXI1	4	0.000192616
HELLS	3	0.0000342
TCTN3	2	0.000258726
DENND10	0	0.000251879
KCNIP2	1	0.0000503
CFAP58	0	0.0000279
C10orf95	7	0.000105088
SFRP5	7	0.000227262
GNA13	0	0.000292416
DUSP1	0	0.0000443

RCL1	3	0.000083
EQTN	7	0.000108622
MOB3B	2	0.0000707
CD274	5	0.000200529
NUP43	3	0.000355551
MTHFD1L	2	0.0000785
PLEKHG1	11	0.000176559
CYSTM1	0	0.0000276
ARAP3	1	0.000120312
PCDHB14	7	0.00028516
SNX19	2	0.000319115
MSANTD2	2	0.000332495
PDZD11	2	0.000181761
PLXDC2	0	0.000194347
CCDC77	3	0.000255548
TNFSF11	7	0.0000314
DNAJC15	3	0.000122528
HSPH1	3	0.000211696
KBTBD7	0	0.000118915
KDM3B	1	0.000272474
GLT8D2	7	0.000305953
MTERF2	3	0.000248915
SOCS2	3	0.0000253
APAF1	0	0.000243707
DUSP4	7	0.0000406
CLU	4	0.000185309
TNFRSF10B	0	0.000166154
SORBS3	1	0.000188754
PTK2B	1	0.000256631
PDLIM2	1	0.000231777
EPHX2	2	0.00009
UBIAD1	2	0.000307627
TNFRSF8	1	0.0000698

CRISPLD1	7	0.000251855
RDH10	3	0.000121968
AKAP1	2	0.000309779
TRIM25	1	0.000107737
SCPEP1	0	0.000192137
TEX14	7	0.000317489
FAM117A	4	0.000149818
NCAPH	14	0.0000456
TMEM131L	2	0.000330537
TRIM6	5	0.0000436
ADCY7	1	0.000348221
TSHZ3	1	0.0000386
ECHDC2	2	0.000191632
PLBD1	0	0.0000758
TAS2R10	3	0.0000289
KCNJ8	7	0.000260676
TAS2R7	7	0.0000399
BCL2L14	7	0.000129486
ZSCAN18	2	0.0000248
TRMT1L	3	0.000224004
CSTA	3	0.0000465
B4GALT4	3	0.000207422
GJA3	7	0.000219701
TBC1D15	3	0.000250321
CCRL2	0	0.0000274
CCR2	0	0.0000853
POLR3GL	3	0.000145772
GHSR	7	0.0000242
TNFSF10	0	0.000116591
TMEM156	3	0.000135832
CPXM2	7	0.00029534
TMEM54	7	0.000260247
LRIF1	0	0.000216344

GPSM2	0	0.000116924
SV2C	7	0.000343716
MTERF4	2	0.000288061
XPNPEP2	7	0.0000265
OCRL	2	0.000137003
MRPS2	3	0.000114615
FMOD	11	0.000242799
KIAA1191	3	0.000251639
CD244	3	0.000131681
LY9	2	0.000174651
SERAC1	0	0.000155296
NAA60	1	0.000259837
SLC66A2	4	0.0000329
ZMIZ2	1	0.000184594
EEPD1	1	0.000165585
FAM126A	3	0.000249721
INHBA	10	0.000130238
ARL4A	4	0.0000624
CCZ1	3	0.000104485
GLIPR2	0	0.000264048
SLC25A51	3	0.000253596
CALD1	4	0.000111246
NUDT10	7	0.0000345
SFTPA1	7	0.000280151
SRGN	0	0.000081
EGR2	7	0.000123189
ZWINT	14	0.000181618
RBM19	2	0.00033547
HVCN1	0	0.0000895
CDKN2C	3	0.0000267
RNF11	4	0.0000813
CCDC91	3	0.000312682
ACOT9	0	0.000155533

ADGRE5	1	0.000165867
WDR83	3	0.000288939
CCDC70	7	0.000198246
SPRYD7	3	0.000203602
EBPL	3	0.0000543
NLN	2	0.0000276
OPTN	4	0.00012602
NEUROD4	7	0.000246323
ARHGAP9	1	0.00016775
MMP19	7	0.000264945
PDE1B	1	0.0000522
NFE2	4	0.0000505
STIL	4	0.0000576
RAB9B	2	0.0000238
METTL8	11	0.0000919
TTC21B	3	0.000334142
TNFAIP6	0	0.000025
ACVR1C	3	0.0000499
SLC36A1	1	0.0000987
LPGAT1	0	0.000243312
G0S2	25	0.000129026
KCNJ2	0	0.0000789
PLA2G12A	3	0.000166778
PFKFB2	0	0.000097
C4BPB	7	0.000182797
GPR83	7	0.000259026
MXD4	1	0.000141967
CKS2	14	0.0000611
MOGAT1	7	0.000177368
FAM124B	13	0.0000405
ENKD1	0	0.0000991
FAM210B	4	0.000251621
PREX1	1	0.000222806

KCNS1	7	0.000269503
CHD6	2	0.000345998
PLCG1	2	0.000239633
TOX2	7	0.000226509
ZNFX1	0	0.000146991
ZNF831	2	0.000226754
EDN3	7	0.000153742
STAU1	1	0.000200799
CDH26	0	0.0000758
SNAI1	7	0.000132857
PMEPA1	26	0.000154847
ANKRD60	7	0.000241945
BCAS4	2	0.0000653
ZBP1	5	0.000211596
NEURL2	3	0.0000252
PEPD	3	0.000208947
CHST8	7	0.000155153
NAGK	0	0.0000859
CEACAM8	10	0.000240153
F13A1	4	0.000198786
PACSIN1	7	0.000276012
BTN1A1	7	0.000202817
SNRPC	3	0.000127654
SLC17A3	7	0.0000237
SERPINB6	2	0.000163082
H1-3	3	0.0000987
NQO2	0	0.0000243
UNC5CL	2	0.0000963
RPS10	3	0.000275326
H2BC11	14	0.000179884
GNMT	1	0.0000402
TREM1	0	0.000104799
MEA1	3	0.000233297

KLHL31	7	0.0000238
SOX4	0	0.0000247
CPNE5	11	0.0000755
KCNK17	7	0.0000659
RPP40	3	0.00015766
RUNX2	2	0.000168933
MYRF	7	0.000207087
EMC3	4	0.000200458
WNT1	7	0.0000822
SH3TC1	1	0.0000955
LRRC29	1	0.0000904
BBS2	2	0.000230234
GPR18	3	0.0000791
CLYBL	2	0.000201816
TMTC4	2	0.000133675
ABCC4	4	0.0000985
TM9SF2	0	0.000233176
HROB	7	0.000215701
UPF3B	3	0.000285492
RNF113A	3	0.000324524
SEPTIN6	2	0.000185994
NDUFA1	3	0.0000538
DMAC2L	2	0.000229355
PTGER2	3	0.000129793
ARMC7	1	0.000214362
NUP85	2	0.000257337
C1orf61	7	0.00020953
DDX31	2	0.000287631
MBOAT7	1	0.000161667
SRMS	7	0.000037
OPRL1	1	0.000142352
IL1B	0	0.0000539
PSD4	1	0.000239458

SLC25A23	1	0.0000481
PSPN	1	0.0000258
GTF2F1	0	0.000174139
ALKBH7	3	0.000111973
CLPP	3	0.00014866
GRIA3	7	0.000162393
C3	7	0.0000694
SH2D3A	1	0.000100604
TRIP10	7	0.0000255
GPR108	0	0.000246519
TNFSF14	0	0.000161689
FOSB	25	0.000175914
RTN2	1	0.0000241
VASP	0	0.000136341
CD93	0	0.0000749
PSMF1	4	0.000147339
TMX4	0	0.00020868
STK35	1	0.000308951
NRSN2	7	0.000292454
RRBP1	0	0.0000734
BMP2	7	0.000166746
BFSP1	7	0.0000377
MGME1	3	0.000234848
FAM110A	1	0.000106956
SIRPD	1	0.000120672
S1PR4	0	0.0000834
NCLN	1	0.000212844
CITED1	7	0.0000976
ARMCX5	3	0.000345342
NECAB3	1	0.0000587
DYNLRB1	2	0.0000854
ERGIC3	2	0.000220828
ROMO1	3	0.0000512

PLAGL2	1	0.000150231
MMP24OS	1	0.000251876
KDM5C	2	0.0000286
AMOT	2	0.0000427
TMEM115	1	0.000200627
UROD	3	0.000188657
ST3GAL3	2	0.000205338
HECTD3	1	0.000212558
TUBGCP3	2	0.000323899
PCID2	3	0.000368001
LRFN3	7	0.0000797
IGFLR1	3	0.0000269
CAPNS1	1	0.0000856
PDCD2L	3	0.000121202
FFAR2	0	0.0000838
HCST	3	0.0000264
FFAR1	7	0.000245099
COX6B1	3	0.0000728
THRA	1	0.000160314
CCR7	3	0.0000812
NR1D1	1	0.0000427
FRMD8	1	0.000267495
BCL2L12	2	0.0000305
IRF3	2	0.000238641
SCAF1	1	0.000143492
TSKS	7	0.0000278
ASL	0	0.000155288
CSN1S1	7	0.0000383
STAT5A	0	0.000205961
WNK4	7	0.000306889
TRAP1	2	0.000206618
AHDC1	1	0.000162298
DACH2	7	0.000175601

CFP	0	0.000101611
DLGAP5	14	0.000180912
L3HYPDH	3	0.000294975
HSPA2	7	0.0000244
PLEKHG3	1	0.00019398
RHOT1	0	0.000277727
EVI2A	3	0.0000621
OMG	0	0.0000258
NUP214	0	0.000314358
SLC10A3	1	0.0000912
MAP2K2	1	0.0000455
HNRNPH2	0	0.000128923
NXF5	7	0.000158484
TIMM8A	3	0.000256584
ZC4H2	2	0.000116204
CANX	3	0.000292945
PPCS	3	0.000321521
BCL11B	2	0.000247285
HELB	2	0.000308602
PTPRB	7	0.0000886
YEATS4	3	0.000132419
TAS2R3	2	0.00014701
TAS2R4	2	0.000143916
TAS2R5	2	0.0000974
LRRC61	1	0.000155608
TRPV5	7	0.000197556
IDUA	1	0.000153993
FGFRL1	1	0.000048
AUNIP	7	0.000119251
ADGRE2	1	0.0000375
SIN3B	1	0.000175593
EPS15L1	1	0.000218299
F2RL3	4	0.0000345

UQCR11	3	0.000074
GFER	3	0.000186106
KDM4B	1	0.00021407
EMC6	3	0.000171233
METTL16	2	0.000254396
TUBA4A	0	0.000167396
VIL1	4	0.0000683
AAMP	3	0.000210173
TNFRSF19	7	0.000248274
ECHS1	3	0.000209003
GNG11	4	0.0000952
PTPN12	0	0.000185393
POR	1	0.000121833
STYXL1	0	0.000237689
STEAP4	0	0.0000892
SGCE	27	0.000154847
LRFN1	1	0.000121005
ZFP36	1	0.0000979
PAICS	3	0.000161762
ADM2	7	0.000164431
DGCR6L	3	0.000176332
DGCR8	2	0.000207348
ASPHD2	5	0.0000278
SDF2L1	3	0.000131343
GAL3ST1	7	0.00029408
YWHAH	1	0.0000559
GNAZ	4	0.000247289
ADORA2A	2	0.0000508
ATF4	3	0.000233233
CDC42EP1	7	0.0000238
APOL3	3	0.000175216
TPST2	0	0.000146234
MPST	0	0.0000532

TST	0	0.0000938
IGLL1	7	0.0000446
APOL2	0	0.0000985
APOBEC3A	1	0.0000528
APOBEC3F	1	0.0000862
RIBC2	7	0.000222745
RNF112	7	0.000289124
SPECC1	4	0.000129579
DOCK4	5	0.0000263
ATP6V1F	3	0.0000806
CDHR3	2	0.000105057
STRIP2	7	0.000034
LRRC4	0	0.000120547
OPN1SW	7	0.0000236
HAT1	3	0.000107544
HERC2	2	0.000182943
SNRPN	2	0.000233041
WDFY4	1	0.0000698
MYO5C	7	0.0000303
TMOD2	0	0.000106995
CCDC32	2	0.000187835
KNSTRN	0	0.0000576
CHAC1	7	0.000163736
CLN6	2	0.0000302
VPS13C	3	0.000294421
CALML4	0	0.0000439
THAP10	7	0.0000638
ANAPC13	0	0.000227395
SUMF2	3	0.00030013
TPH1	3	0.0000674
E2F8	7	0.0000788
DCTD	3	0.000422317
USP6	7	0.0000356

ATP1B2	7	0.0000243
FXR2	0	0.000232027
KIF1C	1	0.000269974
KRI1	2	0.000165578
CDKN2D	0	0.0000354
SIGLEC9	1	0.00019699
RIPK3	1	0.000235683
ADCY4	1	0.000119369
RAB2B	4	0.000128349
HEATR5A	0	0.000242508
TEP1	1	0.000267167
QRICH2	2	0.0000772
FOXJ1	7	0.000149938
SEC14L1	1	0.000174144
AANAT	7	0.000257188
ASH2L	0	0.000100276
CHRNA10	1	0.000157614
RPS4Y1	12	0.000165137
DOHH	3	0.0000475
PLPPR3	7	0.000160219
CBFA2T3	1	0.00013873
GAMT	3	0.0000511
PUDP	2	0.0000719
ERMARD	2	0.000355715
GALNT8	7	0.0000511
KCNA5	7	0.000162855
FAM155B	7	0.000171866
SAT1	0	0.000132988
GNL3L	2	0.000165352
SH3BP4	7	0.000119725
MOSPD2	0	0.000143142
ECSIT	4	0.0000636
ELOF1	4	0.00014648

TSPAN16	7	0.0000854
APOC1	7	0.000111308
XPO7	4	0.000138379
BST2	5	0.000108289
SLC27A1	1	0.000146777
DDA1	1	0.000254848
MRPL34	3	0.000136525
PGLS	1	0.000163078
TULP4	2	0.000311868
SNX9	2	0.00022225
QRSL1	3	0.000205624
RSPH3	0	0.000136854
MLLT1	1	0.000127909
ACTN4	1	0.000120872
STK33	7	0.0000942
EPO	7	0.000227595
ARPC1B	0	0.000283466
FCHO1	1	0.00023211
UNC13A	7	0.000281375
MAP1S	1	0.000214734
PXDN	7	0.000276365
JUND	1	0.0000283
TRPM4	7	0.000275355
UBAC1	0	0.000185581
SAG	7	0.000280474
ZBTB46	7	0.0000635
SAMD10	1	0.0000558
LSP1	1	0.000108291
TNNI2	0	0.0000246
TUBGCP2	1	0.000289425
PNPLA7	7	0.0000376
ZNF337	3	0.000363325
TAF4	1	0.000203883

OSBPL2	0	0.000305561
CHMP2A	0	0.000156768
UBE2M	0	0.0000467
METTL26	1	0.0000631
EIF2S3	3	0.000224395
GMFG	0	0.0000908
SESN2	1	0.000144243
THEMIS2	0	0.000220492
CLIP1	0	0.000312813
HIP1R	2	0.0000712
PPAN	3	0.0000943
SLC6A8	4	0.000123357
MPP1	4	0.000272333
ZNF331	3	0.0002663
ZNF236	2	0.000348483
C12orf65	3	0.000302318
CASZ1	1	0.000105585
PKDREJ	7	0.000293277
HABP4	3	0.000185863
AKAP12	23	0.000204739
SYNE1	2	0.0000889
LILRB2	1	0.0000938
AAR2	2	0.000312237
GGT7	2	0.000179173
GSE1	1	0.0000575
GINS2	14	0.000213995
SLC34A1	7	0.000210571
PRR7	1	0.000146174
NFATC1	1	0.000139952
IDO1	5	0.0000639
CAP1	0	0.000253705
PPT1	0	0.0000642
RAB11FIP4	0	0.000241202

RLIM	0	0.000309671
TRAF3	2	0.000306432
HAUS8	3	0.000167212
ADGRE3	0	0.000141378
SH3BP5	3	0.000253295
RFTN1	2	0.000321489
SLC6A6	1	0.000234784
KCNC3	1	0.000056
NAPSA	7	0.0000249
MGAT1	0	0.000302788
TUBG1	2	0.0000255
RPL27	3	0.000061
PSMC3IP	2	0.0000235
AOC3	1	0.0000605
ACLY	0	0.000180398
AOC2	1	0.000119311
NDUFA2	3	0.000161856
DIAPH1	1	0.000240304
NDFIP1	3	0.000315703
PPFIA1	1	0.000215337
TMEM204	3	0.0000256
BARX1	7	0.000206584
NINJ1	1	0.000137357
MAP1B	7	0.000145202
IL13RA1	0	0.000138857
CKMT2	7	0.000285307
TOP2A	14	0.000219166
RARA	1	0.00020069
CHD1L	3	0.000379288
PEX11B	3	0.00039667
FMO5	0	0.00018959
RAI2	7	0.0000361
AC005261.1	3	0.000240056

ZSCAN5A	2	0.000229454
ZNF132	3	0.0000766
CHSY1	0	0.000102974
RHPN2	7	0.000153078
C19orf12	3	0.000339463
LGALS3	4	0.0000295
ZNF20	0	0.0000708
C19orf57	7	0.000147963
TRIM21	0	0.000185641
SPATA6	0	0.0000414
CCT6B	3	0.0000272
RAF1	0	0.000290332
PPARG	7	0.000188204
FCRLA	11	0.000157247
ENOSF1	2	0.000103236
EMILIN2	1	0.000117676
TRIM5	5	0.000162836
TRIM22	5	0.000183048
RRP8	2	0.000411298
ILKAP	2	0.000278121
PER2	1	0.000225569
PTPRE	0	0.000301085
CARD6	0	0.0000743
AC002316.1	1	0.000228926
RPA1	2	0.000154612
TBC1D14	0	0.000238106
COQ3	3	0.000118752
LANCL2	3	0.000350813
ENAM	7	0.000268591
JCHAIN	15	0.000201145
UTP3	3	0.0000882
WBP2	1	0.000124991
UNK	2	0.000273529

EIF5A	3	0.000146094
KDM6B	1	0.000144696
GPS2	1	0.0000838
XAF1	5	0.000198902
DLG4	1	0.000147471
RIDA	3	0.0000758
REEP2	7	0.00027247
PCBD2	2	0.000341214
SDF2	0	0.00032871
PRMT7	2	0.000340855
ANKEF1	3	0.000148247
SCP2D1	7	0.00013375
PCED1A	2	0.000199749
BTBD3	0	0.0000854
RAB25	7	0.000105134
FCRL2	11	0.000160188
SYT11	3	0.000103887
IGHMBP2	1	0.0000305
ACY3	7	0.0000746
TESMIN	0	0.0000959
MMACHC	2	0.0000409
DPH2	2	0.00017641
RBM38	4	0.000156065
VSTM2L	7	0.000195919
PPP1R3D	0	0.000241968
DMGDH	7	0.00029787
ZBED3	7	0.0000244
PATJ	3	0.000219669
FBXO44	2	0.0000463
CPLANE2	3	0.0000354
CASP9	0	0.000251649
ATP8A2	7	0.000140779
XPO4	3	0.000347892

CDK8	3	0.00025083
ALOX5AP	0	0.0000383
WASF3	7	0.0000582
CHRM3	7	0.000301098
PEMT	1	0.0000609
PIK3C2B	2	0.000109429
LGR6	3	0.0000933
TMCC2	4	0.0000545
SPART	0	0.000192093
EPSTI1	5	0.000160916
TPT1	3	0.0000592
RFC3	3	0.000233686
BEX2	3	0.0000432
TCEAL4	3	0.0000423
FAM104A	4	0.00032447
EPHB2	7	0.000111104
PRAM1	1	0.000108914
KMT5C	1	0.0000464
PDE6B	3	0.000135738
CSNK1G2	1	0.000204116
CNDP2	2	0.00023646
MACROD1	2	0.000100774
LGALS12	13	0.000135394
RTN3	0	0.000174192
MORC2	2	0.000328015
LARGE1	26	0.000154847
SLC2A11	2	0.000063
C1QTNF6	3	0.000168387
SEC14L4	4	0.0000447
MKRN1	4	0.000261229
E2F5	11	0.000229923
CA1	4	0.0000417
ARNTL	0	0.000273612

LYVE1	0	0.0000237
MICAL2	1	0.0000844
RNF122	1	0.000148078
ERG28	2	0.000276447
GSC	7	0.000131692
DGLUCY	1	0.000192793
NUMB	1	0.000185495
CATSPERB	2	0.0000238
ADAM20	2	0.0000815
LOXL2	2	0.0000742
CCNB1	14	0.000223091
CD180	11	0.0000944
IRAK2	0	0.0000447
CAMK1	13	0.000106422
VHL	3	0.000494772
BHLHE40	1	0.0000486
MEIS2	7	0.000302279
KATNBL1	0	0.000106044
REG4	7	0.000298242
TSPAN2	0	0.0000444
VAV3	0	0.000242973
PSRC1	0	0.0000253
SORT1	1	0.0000594
NOTCH2	1	0.00019899
TRIM45	7	0.00032627
FKBP11	1	0.000175571
RSAD2	5	0.000159648
MYCN	7	0.000225195
LPIN1	2	0.000317393
CMPK2	5	0.000189493
LDHA	3	0.000158809
IL6ST	3	0.000133827
NAV1	2	0.0000613

RPS15A	3	0.0000522
IL2RA	2	0.0000331
ANKRD16	2	0.000156622
ECHDC3	0	0.0000533
IL15RA	2	0.000042
HRH4	13	0.0000601
TMEM241	2	0.0000829
KCTD1	24	0.000174281
KLRD1	3	0.0000964
PRH2	7	0.000182244
MYBPC3	1	0.0000464
ACP2	1	0.0001517
RTL8C	1	0.0000504
PIWIL4	28	0.000154847
PHC2	1	0.000143347
AGO4	0	0.000273455
CYP2J2	7	0.0000501
TPGS2	2	0.000101574
DAGLA	7	0.000106861
DHX34	1	0.000194609
TMEM258	3	0.000155139
TCN1	10	0.0000585
C5AR2	0	0.000213193
TMEM165	0	0.000241861
GGACT	3	0.000108443
UBAC2	2	0.00038088
TPP2	3	0.000349815
ARHGAP32	2	0.000089
ETS1	2	0.000272601
SLC37A2	1	0.0000903
TMED7	3	0.0001964
APC	0	0.000248925
NREP	3	0.0000652

OSTF1	0	0.000267289
C9orf40	4	0.000100418
CTSL	3	0.0000239
ISCA1	4	0.000049
ADAM19	1	0.00017447
HAVCR2	3	0.0000818
CCNJL	1	0.0000976
USP30	2	0.000336756
SDS	7	0.000277196
OASL	5	0.000185827
RNFT2	7	0.0000572
BICDL1	2	0.000120911
TRAFD1	0	0.000158157
TMEM243	3	0.000323049
CCDC146	0	0.0000257
CD36	0	0.0000707
PNPLA8	3	0.000178068
HILPDA	3	0.0000494
ANKRD6	0	0.000101387
KHDC1	7	0.0000245
AKIRIN2	0	0.000235055
PRR5L	3	0.000102116
LMO2	0	0.0000948
PHF21A	0	0.000219399
NAT10	2	0.000311184
EHF	7	0.000262705
PRRG4	0	0.0000235
ATP5MC2	3	0.000150969
AVIL	1	0.000041
TESPA1	2	0.00020033
AGAP2	1	0.000257199
BLOC1S1	3	0.0000313
CDK4	2	0.000180736

TROAP	4	0.0000355
COQ10A	3	0.000287637
PAN2	2	0.000263528
KRT7	7	0.000298898
HNRNPA1	3	0.0000447
ACVR1B	1	0.00018921
OS9	1	0.000313601
LTV1	3	0.000275702
AFG1L	3	0.00032984
HEY2	7	0.000252417
SMPD2	1	0.000195612
TEC	2	0.000122912
SEMA4F	2	0.000277739
RAB11FIP5	3	0.0000586
SMYD5	2	0.000185638
DYSF	1	0.000124156
USP15	0	0.000155785
GNS	0	0.000200807
CPM	0	0.0000379
MPHOSPH6	3	0.00022299
KIAA0513	1	0.000215665
FBXL8	1	0.0000503
FHOD1	1	0.00023345
CCDC102A	3	0.000059
SLC9A5	7	0.000267393
PCNX2	2	0.000234237
URB2	2	0.000345122
EGLN1	0	0.000203637
ABCB10	3	0.000153762
NTPCR	2	0.000156536
GLUL	0	0.0000818
STX6	0	0.000387832
RNASEL	0	0.000287723

NPL	0	0.00019154
NIBAN1	0	0.00011497
LAMC1	11	0.000039
SP110	1	0.000141756
TTLL4	1	0.000150204
DNAJB2	4	0.000189005
TMBIM1	0	0.000270069
ARMC9	7	0.000372224
CAB39	0	0.000297074
COX5B	3	0.000188923
TSGA10	3	0.0000834
TMEM127	1	0.000256208
EDAR	2	0.0000492
ANKRD36	3	0.000194706
ISCU	0	0.000187028
USP44	3	0.0000267
SCYL2	0	0.000311791
CKAP4	0	0.0000563
PLXNC1	0	0.000217668
APPL2	2	0.000291097
WASHC4	0	0.000201253
SLC41A2	3	0.0000236
FLNB	2	0.000104734
NEK3	3	0.000131938
RNASEH2B	3	0.000309369
TBC1D4	3	0.00013473
THSD1	2	0.0000247
LMO7	2	0.000242102
ITM2B	0	0.000251206
EDNRB	7	0.000285392
RCBTB2	0	0.000265257
LCP1	0	0.000236925
SCRN1	2	0.0000508

C7orf25	0	0.000133833
CHST12	1	0.000115449
RAC1	0	0.000228742
IL6	7	0.0000265
AOAH	0	0.000131514
BZW2	3	0.000333905
DBNL	0	0.000338318
CCM2	0	0.00033599
CIDEB	1	0.00013209
MTHFS	0	0.00016018
ALPK3	7	0.000083
TM6SF1	0	0.000217739
CALCOCO2	0	0.0000856
MYCBPAP	7	0.0000812
TEX2	2	0.000302995
BRIP1	14	0.0000914
RTP4	5	0.000144047
MARCHF7	0	0.000251611
ERMN	2	0.000113818
SCN7A	7	0.000237644
BLK	11	0.000186469
HLX	1	0.0000742
KCTD3	3	0.000122702
IL1RN	1	0.0000438
IL36A	7	0.0000522
BIN1	2	0.000125439
HS6ST1	1	0.000168825
GYPC	4	0.000193266
LRRC8A	4	0.0000874
TXN	0	0.000054
TOR1B	5	0.000137893
C9orf78	4	0.0000259
KLF4	0	0.0000492

NIBAN2	1	0.000100526
ST6GALNAC4	4	0.000104769
TMOD1	4	0.000159389
DAB2IP	7	0.00025976
STXBP1	7	0.0000351
SLC2A8	1	0.000032
CDK5RAP2	2	0.000319735
ZFP37	3	0.0000267
SLC31A2	0	0.000108452
SLC31A1	0	0.000128435
TLR4	0	0.000152208
USP20	2	0.000289837
DPM2	4	0.000231071
HEMGN	4	0.0000269
RPL35	3	0.0000628
DSCC1	14	0.0000587
MYC	3	0.0000762
IL33	7	0.0001297
IL11RA	2	0.00014894
UBAP2	4	0.0000432
TLN1	1	0.000156195
SIT1	3	0.000147665
SPAG8	2	0.0000375
DCTN3	0	0.000297035
CD72	11	0.0000755
GRHPR	3	0.0000864
ALDH1B1	3	0.000101501
ARHGEF39	7	0.000321398
CNPY3	1	0.000188006
FOXP4	1	0.0000625
KIF13A	0	0.000213596
ZSCAN9	3	0.000197556
PIM1	4	0.000106396

GMPR	4	0.000211392
TMEM63B	4	0.000176267
FRS3	1	0.0000944
CAPN11	7	0.000180998
KIAA0319	1	0.0000236
IRF4	2	0.0000404
LRRC1	3	0.0000371
BPHL	2	0.000228714
UQCC2	3	0.000272328
FLOT1	0	0.000128443
MDC1	2	0.000238139
PGBD1	3	0.0000464
RNF144B	0	0.000177206
VARS2	1	0.0000769
TAF8	2	0.000239841
FAM8A1	0	0.000134414
FGFBP1	7	0.000167886
FGFBP2	3	0.0000584
TLR2	0	0.0000858
MYO7A	7	0.000244696
FCHSD2	2	0.00019601
SYTL2	3	0.0000814
RAB30	11	0.000174161
LRRC32	7	0.000248871
NARS2	3	0.000312872
GGH	14	0.0000272
SLCO5A1	7	0.000275237
SDCBP	0	0.000209998
DDX60	5	0.000175317
SORL1	0	0.000192452
RDX	3	0.000273309
C11orf1	3	0.0000728
FXYD6	7	0.000115607

ARHGAP20	7	0.0000367
CASP1	0	0.000145802
CASP5	5	0.0000474
SQOR	0	0.000212846
NUSAP1	14	0.000214724
KNL1	14	0.00016017
RTF1	0	0.000315684
PARP6	2	0.000296253
RPLP1	3	0.0000301
PAQR5	7	0.000259276
PAK6	7	0.000291967
SPTBN5	7	0.0000506
GCOM1	29	0.000154847
TTLL7	8	0.00010787
IFI44L	5	0.000151966
GIPC2	7	0.000173267
IFI44	5	0.00015709
IFT172	2	0.000217981
SLC5A6	2	0.000204956
PREPL	3	0.000334749
CENPO	2	0.000177098
DTNB	2	0.000226841
ACTR1A	0	0.000350568
MFSD13A	1	0.0000429
CYP2C8	7	0.0000662
MYOF	2	0.000026
STAMBPL1	3	0.000239055
KIF11	14	0.000241167
CUZD1	3	0.000100171
CALHM2	1	0.0000886
ARL3	0	0.0000928
CEP55	14	0.000189317
KIF20B	3	0.000092

ENTPD1	0	0.000217445
ZNF365	3	0.0000303
OIT3	7	0.000123605
ADAMTS14	7	0.000258487
TET1	3	0.000144079
DNA2	3	0.0000489
ATIC	2	0.000269765
MSTN	7	0.000115015
CDK15	7	0.0000316
HECW2	0	0.0000282
CIR1	0	0.00020036
ITPRID2	0	0.000263089
FAM117B	2	0.000135538
ABI2	2	0.000265018
SLC40A1	0	0.000092
SLC49A4	0	0.000171809
COX17	3	0.000320591
PARP9	5	0.000154227
GLCE	3	0.000295975
APH1B	0	0.000340031
CILP	7	0.0000311
PARP16	2	0.0000903
PPCDC	1	0.000209898
SEMA7A	13	0.0000953
UBL7	4	0.000130315
ARHGAP24	11	0.0000329
HERC6	5	0.000173138
HERC5	5	0.000161659
HNRNPD	3	0.000391338
GPAT3	0	0.000122595
MMRN1	4	0.000125656
PDE5A	4	0.000240644
SEPTIN11	2	0.000238557

CCNG2	0	0.000198039
CDKL2	7	0.000290118
ANXA3	0	0.0000239
CENPE	14	0.000184043
LEF1	2	0.0000939
HADH	2	0.000314379
EGF	4	0.000192229
PAPSS1	0	0.0000235
MAPK8IP3	1	0.00024777
RGS3	1	0.000136916
GUCD1	4	0.000252228
ERP27	2	0.000180839
GABARAPL1	0	0.000196542
KIF21A	3	0.000143574
FGD4	0	0.000149509
ETFBKMT	3	0.0000244
PRICKLE1	11	0.0000558
C1RL	1	0.000235388
CLSTN3	1	0.0000579
KLRG1	3	0.000062
VAMP1	2	0.000179842
CD27	2	0.0000691
RBP5	7	0.000198014
PEX5	2	0.000333372
AMIGO2	3	0.000204674
GLIPR1	0	0.000169491
DUSP6	0	0.0000446
POC1B	3	0.000127094
SLC15A4	0	0.000238519
RITA1	3	0.000223146
GLTP	0	0.000197307
TCHP	2	0.000419767
FAM222A	7	0.00029401

NUP58	0	0.00028237
MTMR6	0	0.000239759
SLC7A1	2	0.000109848
LNX2	3	0.000301568
TARBP2	3	0.000195019
RDH16	2	0.0000416
BRCA2	3	0.0000429
ITGB7	3	0.000104389
GALNT6	0	0.0000236
CSAD	1	0.00019803
C12orf10	3	0.000107942
TMBIM6	0	0.000340102
SMIM2	7	0.000197914
HNRNPA1L2	3	0.0000653
VPS33A	3	0.000403173
RHOF	1	0.000162756
DIAPH3	30	0.000154847
SLAIN1	3	0.000261094
GRTP1	7	0.000146976
CUL4A	3	0.000283894
CDH24	7	0.000239633
REM2	1	0.0000949
PELI2	0	0.000260416
RTN1	1	0.0000247
SLC38A6	3	0.000236223
DCAF5	2	0.00037589
ESR2	7	0.000120913
STON2	4	0.000144158
EFCAB11	2	0.0000577
JDP2	0	0.000194077
AK7	7	0.000299385
CLBA1	7	0.0000316
WARS1	5	0.000036

NIPA2	3	0.000447905
DUOXA1	7	0.000222021
SORD	13	0.000179039
SERF2	4	0.000103539
ZSCAN29	3	0.000386463
LYSMD2	0	0.0000888
SLC27A2	14	0.0000506
HDC	23	0.000289051
BAHD1	2	0.000253371
CDAN1	1	0.000321078
TLE3	1	0.000210568
ANP32A	0	0.000247346
PSTPIP1	1	0.000258851
BCL2A1	3	0.0000541
SCAPER	3	0.000264747
TSPAN3	3	0.0000974
MAN2C1	1	0.0000432
DNAJA4	4	0.000125974
TPM1	4	0.0000474
IGF1R	1	0.000146039
CYP1A1	7	0.000240747
ADAMTS17	2	0.0000363
LINS1	3	0.00027275
PCSK6	4	0.000239602
HAPLN3	2	0.0000542
ABHD2	1	0.000154929
DET1	2	0.000201907
MFGE8	2	0.0000925
ZNF710	1	0.000195456
MCTP2	0	0.000165201
IQGAP1	0	0.000196071
CRTC3	2	0.000296756
GLYR1	0	0.000429892

PMM2	2	0.00019381
ITGAX	1	0.000107601
FTO	2	0.000248149
CDR2	3	0.000193206
IGSF6	0	0.000160988
ARHGAP17	2	0.000161229
MYLK3	7	0.000311682
NKD1	7	0.000100131
DHX38	2	0.000335469
ZFHX3	1	0.0000609
CPNE2	1	0.0000768
NLRC5	1	0.000121502
KATNB1	1	0.000232943
CMTM3	0	0.00019324
CMTM2	0	0.0000447
MAP1LC3B	0	0.000158162
MBTPS1	2	0.000441144
MEAK7	2	0.00023614
IRF8	3	0.0000815
RPS2	3	0.000130227
DEF8	1	0.000260324
TCF25	1	0.000248602
GAS8	2	0.0000324
GID4	4	0.0001303
KSR1	1	0.000135371
UTP4	2	0.000308589
RANBP10	4	0.000193975
CTRL	1	0.0000272
DPEP3	1	0.0000689
GFOD2	4	0.0000715
NOB1	2	0.000116759
PCTP	0	0.0000424
TOB1	3	0.00020152

SPATA22	7	0.000108566
NPEPPS	0	0.000276995
SKAP1	2	0.000188666
LRRC46	7	0.000150051
SSH2	1	0.000176728
BCAS3	2	0.000282491
TAF4B	3	0.00020242
PRELID3A	7	0.00028225
IMPA2	0	0.000139659
GALNT1	3	0.000180295
MEP1B	7	0.000284537
OSBPL1A	0	0.0000504
GATA6	7	0.000190988
SLC14A1	4	0.000105873
ARRB2	1	0.00021464
ASGR1	0	0.0000352
SLC16A3	0	0.000152199
ANAPC11	3	0.000202907
FN3KRP	2	0.000257834
NARF	1	0.000220908
CBX8	1	0.0000322
SECTM1	1	0.0000338
RNF157	2	0.000071
CEP131	1	0.000109748
DYM	2	0.000423244
ZCCHC2	5	0.000148331
FBXO15	7	0.000238422
PMAIP1	3	0.0000462
P3H4	2	0.0000988
ERBB2	3	0.0000306
GRB7	7	0.000243093
MIEN1	1	0.000048
SLC39A3	3	0.0000842

TPGS1	3	0.000162678
PRDM15	2	0.000252143
FEM1A	0	0.000153596
VAV1	0	0.000304815
DMRTC2	7	0.0002262
TMEM91	0	0.000117808
PGGHG	1	0.000128536
COL6A1	7	0.000202524
IFNAR1	0	0.000236313
TRPM2	1	0.0000811
SCYL1	1	0.000242636
URB1	2	0.000273993
AKT1	1	0.000274567
EMP3	3	0.000137999
LMTK3	7	0.000102812
ADAMTS10	1	0.0000266
MYO1F	1	0.00021893
NLRP12	0	0.000179496
CACNG8	7	0.0000791
ZNF787	0	0.000106413
CARM1	4	0.000124714
ZNF473	3	0.000360691
RPS11	3	0.000128645
CTU1	3	0.0000883
NOSIP	3	0.000199476
RCN3	1	0.0000788
SLC2A5	10	0.000165057
MMEL1	7	0.0000442
EPHA2	7	0.000283852
EFHD2	1	0.000172766
PEX14	2	0.000219928
PGD	0	0.000138697
C1orf216	3	0.000150722

EVA1B	0	0.0000279
PLK4	14	0.000174365
MAP3K6	1	0.0000238
FCN3	7	0.000180294
ITGB3BP	3	0.000112956
BCL10	0	0.000325225
KIF2C	14	0.000173619
PTPRF	7	0.000206081
LMO4	0	0.000399037
SLC44A3	7	0.000144738
ZNF697	0	0.0000283
CTTNBP2NL	2	0.0000562
C1orf162	0	0.0000956
PROK1	7	0.000250174
ALDH9A1	0	0.000305712
NME7	3	0.000191326
POGK	2	0.000443174
CREG1	4	0.0000892
DCAF6	2	0.000186763
GPA33	2	0.0000534
MAEL	7	0.000284523
ADCY10	7	0.000103472
PPOX	3	0.000183729
FCGR2A	0	0.000109166
NUF2	14	0.000134211
RGS5	7	0.0000491
PRCC	2	0.000350458
FCRL5	11	0.0000286
CRABP2	7	0.0000324
HDGF	4	0.000213605
RGS16	7	0.0000294
PRUNE1	0	0.000136702
RORC	16	0.000154847

TUFT1	0	0.000025
SF3B4	1	0.000238758
SNX27	1	0.000217158
MCL1	0	0.000285685
CTSK	2	0.000100861
MINDY1	1	0.000209061
SELENBP1	4	0.000199119
CERS2	0	0.000312415
SEMA6C	7	0.000242144
ARNT	2	0.000353994
GABPB2	2	0.000296086
DTL	14	0.000194726
DYRK3	4	0.0000782
HHIPL2	7	0.000290717
ATP8B2	2	0.000232154
ADAM15	1	0.0000658
JTB	3	0.0000748
S100A8	3	0.0000505
TPM3	0	0.000313804
SNAPIN	3	0.000298614
S100A7	7	0.000146253
SLC39A1	3	0.0000954
CREB3L4	3	0.0000982
EFNA3	7	0.000083
RIT1	0	0.000228053
INTS3	1	0.000274503
ACTA1	7	0.0000628
SCCPDH	3	0.0000609
SNAP47	3	0.000393915
DEGS1	0	0.000285428
ARF1	1	0.000167937
CNIH4	0	0.00013213
ITPKB	2	0.000267663

GUK1	4	0.000178524
MBOAT2	0	0.0000802
PARP1	3	0.000216991
PSEN2	2	0.0000248
PYCR2	2	0.000186021
LBR	0	0.000197961
EPHX1	7	0.0000447
PPFIA4	7	0.0000539
PLEKHA6	7	0.000363798
PTPN7	1	0.000130257
RHOB	0	0.0000571
GALM	3	0.0000362
CAMKMT	2	0.000255403
EML4	3	0.000357789
WDPCP	2	0.000273545
ZNF514	3	0.000205022
ANKRD53	7	0.000286396
EXOC6B	2	0.000300633
SFXN5	1	0.000237455
DQX1	7	0.0000278
RALB	0	0.000204966
TMEM177	2	0.000214265
FBLN7	2	0.0000458
ZC3H8	3	0.000338915
FAHD2B	2	0.0000876
LYG1	2	0.0000235
AFF3	11	0.000160397
SPOPL	0	0.000175453
GPR17	7	0.000075
AMMECR1L	2	0.000367525
PKP4	0	0.000079
CDCA7	14	0.000187045
PHOSPHO2	3	0.00017341

CCDC150	7	0.000325668
CPO	7	0.000145866
NYAP2	7	0.0000972
RHBDD1	3	0.000175727
ANKMY1	2	0.000285195
COPS7B	2	0.00027743
FANCD2	3	0.000309067
TAMM41	2	0.000153681
VGLL4	2	0.000319163
RAB5A	0	0.000296298
RETREG2	4	0.000125067
STK11IP	1	0.000257712
GMPPA	1	0.00021124
DYNC1LI1	0	0.000199659
RBMS3	7	0.000278793
OSBPL10	11	0.000145918
POMGNT2	4	0.0000281
ACKR2	7	0.000169537
GASK1A	7	0.000297546
CSRNP1	0	0.0000347
SLC25A38	3	0.000149538
ITGA9	10	0.0000422
CTDSPL	4	0.000232797
STAC	7	0.000187189
TMF1	3	0.000280879
LRTM1	7	0.000247964
LIMD1	2	0.000219511
NFKBIZ	0	0.000216386
PHLDB2	2	0.0000712
ADPRH	0	0.0000661
IGSF11	7	0.0000493
NR1I2	7	0.000311902
UCN2	7	0.000127052

DCAF1	2	0.000352595
ILDR1	7	0.000194453
DGKQ	1	0.0000369
CORIN	31	0.000154847
OCIAD2	3	0.000188877
SLC10A4	7	0.000190605
SLC10A6	7	0.000215087
KLHL8	0	0.000192253
SNCA	4	0.000150421
TBCK	3	0.000279843
CISD2	4	0.000123391
FABP2	7	0.0000456
CCNA2	14	0.000215386
SETD7	0	0.00030383
NAF1	3	0.000278469
MARCHF1	3	0.000139625
PDGFC	0	0.0000236
CYP4V2	2	0.000159837
ROPN1L	0	0.000116325
SRD5A1	2	0.000195121
MYO10	7	0.000136608
RPL37	3	0.0000694
GZMA	14	0.000049
LHFPL2	5	0.0000469
IQGAP2	0	0.000188693
BTF3	3	0.0000612
FEM1C	0	0.00022438
ARHGAP26	0	0.00017238
TIMD4	2	0.0000245
PCYOX1L	2	0.000269165
TNIP1	1	0.000268543
N4BP3	1	0.0000409
NHP2	3	0.000220866

TBC1D7	2	0.0000476
FARS2	2	0.000292939
GFOD1	2	0.0000314
LRRTM2	7	0.00027005
ZMAT2	4	0.0000378
KLHL3	2	0.000166806
HIGD2A	3	0.0000469
MMUT	3	0.000231995
DOK3	1	0.000153229
ABT1	3	0.000313267
PPP1R18	1	0.00015128
TCTE1	7	0.000202636
IRAK1BP1	3	0.0000628
PNRC1	0	0.0001114
SCML4	2	0.000229335
TBC1D32	3	0.000225329
SLC18B1	3	0.000125185
SLC2A12	7	0.0000994
SHPRH	3	0.000331788
AIG1	0	0.000132733
TMEM181	2	0.000267738
VIP	7	0.0000245
ARMT1	3	0.000201074
GNA12	4	0.0000279
CCZ1B	3	0.0000302
CREB5	0	0.000073
PURB	3	0.000302579
ATXN7L1	2	0.000264686
SLC12A9	1	0.000216018
AGBL3	3	0.000120292
TMEM140	0	0.000111654
EPHA1	2	0.0000414
NLGN4X	7	0.000199757

SHROOM2	7	0.000133611
SYTL5	7	0.0000311
CASK	3	0.000282298
KDM6A	2	0.0000542
MSN	1	0.000215551
CHST7	0	0.0000236
NDUFB11	3	0.000161665
CCDC120	1	0.000107235
OGT	2	0.000334763
ITGB1BP2	2	0.0000459
IL2RG	2	0.0000243
GCNA	2	0.000227183
DIAPH2	3	0.000297307
FRMPD3	3	0.000125149
DOCK11	0	0.000354193
GPC3	7	0.000110021
MFHAS1	2	0.0000538
FBXO25	2	0.000351121
NSDHL	3	0.000223003
RPL10	3	0.000117127
ATP6V1B2	0	0.000284741
HMBOX1	2	0.000280945
CHRNA6	7	0.000229929
GNRH1	2	0.000252676
BIN3	1	0.000352687
DOK2	3	0.000126791
SLC25A37	4	0.000270193
CHMP7	3	0.000219716
DOCK5	0	0.000177579
STAR	7	0.000260725
TACC1	0	0.000341987
PLPP5	11	0.000102789
GINS4	14	0.00005

SLC26A7	7	0.000125847
SYBU	7	0.000241818
MAL2	7	0.000270425
FAM83A	7	0.0000697
GSDMC	7	0.000262121
SLC39A4	7	0.0000346
NAPRT	0	0.0000371
NFIB	7	0.00023854
PLIN2	3	0.0000999
CBWD5	0	0.000133437
CEP78	3	0.000109201
IDNK	3	0.000298759
MFSD14B	0	0.0000948
SNX30	0	0.000336994
STOM	4	0.000102431
GSN	1	0.0000736
MRRF	2	0.000365519
NR6A1	1	0.000116325
ALAD	0	0.000247879
WDR31	7	0.000122033
GBGT1	1	0.0000826
SURF1	3	0.000335712
MED22	1	0.000288561
SH3GLB2	4	0.0000625
MIGA2	1	0.000295293
LCN2	10	0.000155412
SEC16A	1	0.000224245
NACC2	1	0.000200486
PROSER2	7	0.0000494
USP6NL	3	0.000163898
MSRB2	0	0.0000585
FAM171A1	2	0.0000335
RSU1	0	0.0000334

ST8SIA6	3	0.0000431
PARD3	4	0.000162551
ZEB1	3	0.000308554
NRBF2	0	0.000172234
CDHR1	7	0.0000365
LRMDA	2	0.0000252
CAMK2G	1	0.000277136
ADIRF	7	0.0000696
DNAJB12	1	0.000392957
TCF7L2	2	0.0000335
MKI67	14	0.000151294
FUOM	3	0.0000904
LRRC27	2	0.0000784
TAF5	3	0.000350002
PPRC1	1	0.000218693
ITPRIP	1	0.000250905
RGS10	4	0.0000821
ADM	0	0.0000264
GAS2	3	0.0000393
TUT1	2	0.000139559
APIP	3	0.00014772
SERPING1	5	0.00012527
SLC43A1	4	0.0000489
PTPRJ	1	0.00019734
CCDC81	7	0.000124841
SESN3	4	0.0000952
ENDOD1	4	0.000113845
KLHL35	7	0.000208491
TENM4	7	0.0000288
SERPINH1	2	0.000106956
CAPN5	4	0.0000429
PAK1	0	0.000214624
ZC3H12C	0	0.0000239

TTC12	2	0.00018829
NCAM1	3	0.0000379
HTR3B	7	0.000248474
GLB1L2	7	0.0000523
SLX4IP	2	0.000273768
LAMTOR1	0	0.000316547
HYOU1	1	0.00016562
TKFC	1	0.0001317
MTA2	3	0.000221891
ROM1	7	0.0000318
INCENP	2	0.000145455
ZP1	7	0.000231975
MS4A3	10	0.0000753
MS4A2	23	0.000211123
ESAM	4	0.000225016
MPZL2	0	0.000025
TMEM25	2	0.0000829
COMMD7	2	0.0000887
C20orf144	7	0.0000237
LSM14B	1	0.000308925
CABLES2	1	0.0000921
GPHA2	7	0.000066
TRPT1	1	0.000099
FAU	3	0.0000908
VPS51	3	0.00018474
PPP4C	0	0.000210436
TAOK2	1	0.000333529
CNKSR2	3	0.000101595
KLRF1	3	0.000081
ITGB1	3	0.000124836
CTF1	7	0.000117824
FCGR1A	5	0.000100854
ARID5B	3	0.000219057

DCUN1D2	2	0.000412113
TMCO3	0	0.000245589
TIRAP	1	0.000211344
EEF1AKMT1	3	0.000111421
LATS2	1	0.000235527
HNMT	3	0.0000615
ADRA2A	7	0.0000661
GPM6A	11	0.0000422
VEGFC	7	0.0000773
CCDC102B	3	0.000144632
CD226	3	0.000171676
CNDP1	7	0.000317947
FSIP1	24	0.0000926
DLG2	7	0.0000386
RGS18	0	0.000054
PRSS23	3	0.0000605
MTMR12	2	0.000364374
ATPSCKMT	3	0.000316855
PIH1D2	7	0.000224035
IL18	0	0.0000566
PIP4K2A	4	0.000264504
C2orf50	7	0.0000252
FOXO1	2	0.000297952
CRIM1	3	0.0000254
RILPL2	0	0.000268408
DHX37	1	0.000263117
UBC	0	0.000201706
ITPR1	2	0.000260148
TKTL2	7	0.0000236
PRSS53	1	0.000133627
ENKUR	4	0.0000369
DCP1B	3	0.000274441
THRB	7	0.000130615

UEVLD	0	0.000340657
TMEM86A	2	0.0000351
C12orf45	2	0.00022112
TMEM263	3	0.000228405
BTBD11	2	0.0000257
UBE3B	2	0.000381289
ANK3	2	0.000096
IPMK	0	0.000115105
DLG5	3	0.0000497
DIP2C	2	0.0000263
NUBPL	3	0.000308117
ANKRD50	0	0.000194788
UPF2	0	0.000286549
SCLT1	0	0.000160546
CCDC3	7	0.000259729
FRMD4A	21	0.0000545
PTPRO	3	0.0000431
EPS8	32	0.000154847
QDPR	2	0.0000876
FAM160B1	0	0.000165689
DRD3	7	0.0000262
ZNF827	2	0.000147396
NR3C2	2	0.000204532
DPYSL4	7	0.0000259
VENTX	1	0.0000356
ADAM8	1	0.00016607
RNF144A	2	0.000115884
ASAP2	4	0.0000959
FLI1	0	0.000421895
WWC2	2	0.0000702
CENPU	14	0.000130648
ACSL1	0	0.000092
BICD1	2	0.000153713

SAV1	3	0.000152146
SERP2	7	0.00012363
CENPJ	3	0.000261961
CCL28	3	0.000226983
PARP8	0	0.00023367
DST	0	0.0000236
BAG3	2	0.0000788
GLT1D1	1	0.000200245
AP1S3	11	0.0000906
TLCD4	4	0.0000992
MZT2B	3	0.0000511
FAM168B	2	0.000403193
MGAT5	2	0.000279648
CYSLTR2	13	0.000159318
ARL11	0	0.000225932
SETBP1	11	0.0000872
PSTPIP2	5	0.0000881
PDE3B	0	0.000176313
ATG10	3	0.000104631
SPOCK1	7	0.0000848
FAM151B	0	0.0000714
HOMER1	3	0.0000359
ZNF773	3	0.000218206
ZNF776	0	0.000251381
AC010326.1	3	0.000178278
SUV39H2	3	0.000109626
NMT2	3	0.000176793
ZNF837	3	0.0000511
USP12	4	0.0000998
CAMK4	3	0.00013925
TRIM36	32	0.000154847
ZFP36L2	0	0.0000645
PFKM	2	0.000199097

IGSF10	7	0.000146652
SPEF2	3	0.000113694
SPARCL1	7	0.0000728
RASGRP3	11	0.0000703
CATSPER3	7	0.0000285
TCTEX1D1	10	0.0000605
ANKRD22	5	0.0000891
PANK1	3	0.000026
PRDM8	1	0.0000307
HNRNPDL	3	0.000329199
HHEX	0	0.000102708
PTPRK	11	0.000109411
GGPS1	3	0.000313158
ZNF117	0	0.0000487
RAB3C	7	0.000125873
PLOD2	4	0.0000464
STK32B	7	0.000296209
JAKMIP1	3	0.0000325
CDYL	4	0.0000525
CARHSP1	0	0.0000665
BANK1	11	0.000159994
DAB2	2	0.0000257
RGPD3	7	0.0000339
RASSF3	1	0.000228697
AHCTF1	0	0.000220849
TMEM87B	3	0.00028262
PTPRR	7	0.000274198
CCDC148	7	0.000192519
FAM49B	0	0.000304344
ASAP1	1	0.000135888
LPCAT1	1	0.00015527
ING1	0	0.000329355
TEX29	7	0.0000464

CMTM7	2	0.000243351
UBP1	2	0.000338938
RMND5A	0	0.00018056
CD8A	3	0.0000841
RPIA	4	0.000120508
LURAP1L	7	0.0000812
CNKSR3	7	0.000289809
JAZF1	4	0.0000581
PID1	2	0.0000244
KCTD15	1	0.0000424
ZNF599	2	0.000267221
MCOLN2	2	0.0000253
DDAH1	7	0.00027645
HS3ST3A1	7	0.000154533
SEMA3D	7	0.0000236
SLC5A10	7	0.000298633
AK5	3	0.0000329
CABYR	7	0.0000898
SDHAF4	3	0.000203919
C16orf74	2	0.0000353
TBCEL	4	0.0000467
ANKH	4	0.0000716
UBASH3B	2	0.000217294
ROBO3	7	0.000273596
RETREG1	3	0.000132392
PRKCA	2	0.000148151
LRRK1	1	0.000231565
ABCA9	11	0.000154476
ABCA6	11	0.000167297
ENPP3	23	0.000181594
DISP1	2	0.000212038
OBSCN	1	0.0000657
LONRF1	0	0.000207722

TRIM11	1	0.000263113
ENAH	7	0.00030004
CCSAP	0	0.000164398
GBP5	5	0.000162514
SORBS2	7	0.000237961
LY96	0	0.0000515
TMSB4Y	12	0.000165137
CHODL	7	0.000148141
L3MBTL4	2	0.0000235
RABGEF1	2	0.00031708
JAM2	7	0.000262887
TSEN2	2	0.000293953
SLFN13	2	0.000173467
WNT7A	2	0.0000423
FLCN	1	0.000164901
OXNAD1	3	0.000140997
SKA1	14	0.000194587
PPP4R1	0	0.000212839
APCDD1	7	0.000210485
MPPE1	0	0.000228475
EME1	14	0.000064
EPHB1	0	0.000100096
ANKRD40	2	0.00031482
VOPP1	2	0.000199762
KLF10	0	0.0000257
ATP6V1C1	0	0.000243583
PIP4P2	0	0.000176742
CDK19	0	0.000246118
TTC39B	3	0.000222234
MMS19	2	0.000360296
GOLGA7B	2	0.0000635
SAMSN1	0	0.000136231
GRAMD2B	3	0.000166139

RHOC	3	0.0000382
PPM1J	7	0.0000574
SLC16A1	3	0.000107614
OXA1L	3	0.000308718
SLC7A7	0	0.000144841
PIK3AP1	0	0.000150475
TTN	11	0.0000423
FAM126B	0	0.000118841
FLACC1	4	0.0000411
SPAG17	7	0.000329875
RNF20	0	0.000261944
PPARGC1B	2	0.0000759
PXYLP1	2	0.0000243
SLA	0	0.000240742
TMBIM4	0	0.000227845
RAB39B	3	0.000285342
CLIC2	4	0.000099
MICU3	3	0.0000299
GRIP1	7	0.0000239
KIF5A	7	0.00028906
NAT2	7	0.000176641
PSD3	7	0.0000247
CFAP70	2	0.0000543
GNA14	7	0.000258656
GNAQ	0	0.000239052
GPR61	7	0.000278288
ADK	3	0.000157965
BATF	3	0.0000599
NDUFAF6	2	0.000323655
C8orf37	3	0.0000667
CFAP161	4	0.0000311
ADAMTSL3	7	0.000299123
WHAMM	2	0.00034091

N6AMT1	3	0.000261849
RWDD2B	3	0.0000845
BACH1	0	0.000225983
TIAM1	2	0.000151071
SCAF4	2	0.000291169
RPGR	0	0.000168848
ANKRD9	4	0.0000408
SFXN2	2	0.000172905
SH3RF2	7	0.0000614
PPP2R2B	3	0.000192976
FAM122C	2	0.000220853
FBXO43	7	0.000218845
HK1	4	0.0000977
UBE2L6	5	0.000164318
MED19	3	0.00011139
NPTN	0	0.000350663
SAMD8	0	0.000264135
RAB11FIP1	0	0.000205927
AIFM1	3	0.000340877
MAPK13	1	0.000172012
MS4A1	11	0.0000896
TBC1D31	3	0.000186421
FBXO32	2	0.000168625
FBRS	1	0.000218526
FRRS1	13	0.0000984
MPV17L	7	0.000150116
GHRL	1	0.0000247
EXOG	2	0.000253093
NTAN1	4	0.000068
NMNAT2	7	0.00032886
ZFYVE9	3	0.00014396
LRP8	2	0.000192462
SSBP3	1	0.0000616

FZD1	1	0.0000287
SUSD3	3	0.000107076
DHRS4	3	0.000174291
CLEC18C	7	0.0000275
IL34	7	0.000116964
CACNA2D3	0	0.0000236
CCNB2	14	0.000180129
MYO1E	11	0.0000328
AFAP1L1	7	0.000314257
TSC22D3	1	0.0000395
KCNJ15	0	0.0000383
ERG	10	0.000180192
ETS2	0	0.000199981
TSPAN18	1	0.0000764
MX1	5	0.000178114
SLC38A10	1	0.00014936
C9orf43	7	0.0000308
ZNF618	2	0.0000668
TMEM268	1	0.0000629
SNX22	11	0.0000356
PSMG3	3	0.000163638
SLC37A3	0	0.0000534
GAREM2	7	0.0000954
SPPL3	2	0.000252321
WIPI2	1	0.000317008
LDLRAP1	2	0.000131891
AGAP1	3	0.0000794
KRTCAP3	1	0.0000401
WDR66	7	0.000243654
MRPL17	3	0.000271287
PTPDC1	3	0.000262695
HPD	7	0.000126399
RHPN1	1	0.0000541

TPRG1L	0	0.000205425
LRRC43	7	0.000245918
PRXL2C	3	0.000226957
XKR8	1	0.00015464
TMSB15A	7	0.0000499
FANCC	2	0.000204786
ABHD3	0	0.000198024
TENT5B	7	0.00011213
COLEC12	7	0.000189803
RNF207	7	0.0000288
GPRASP2	2	0.000025
AUTS2	2	0.0000297
SHROOM4	7	0.0000272
H2BC5	3	0.0000953
CDC25C	7	0.0000697
H4C8	3	0.0000604
TMSB15B	2	0.000113616
TSPAN33	4	0.00010385
AHCYL2	2	0.000310354
B4GALT5	0	0.000122588
CD1D	0	0.0000917
SPATA2	1	0.000224324
CD1B	7	0.0000641
NCF1	0	0.0000524
GDPD5	1	0.0000401
ALAS2	4	0.0000561
PPP1R15B	0	0.000397675
COPG2	3	0.000242195
PKD1L1	7	0.000117947
TAGLN2	0	0.0000847
SLC45A3	23	0.000119652
HTR6	7	0.0000976
ITLN2	7	0.000208807

F11R	1	0.000157032
SPATA2L	1	0.000058
NPM2	7	0.000283288
CDA	1	0.0000571
PINK1	4	0.000196209
B4GALT3	4	0.0000715
DMTN	4	0.00013674
SLC5A11	7	0.00016943
TOMM40L	3	0.000198471
MPZ	0	0.0000588
RAPGEF6	3	0.000318594
EPB41	4	0.000222164
MIS18A	2	0.0000262
ALG8	3	0.000380946
MRPL10	2	0.000105895
IFNGR2	0	0.000235652
C1QC	7	0.000116838
KCNE2	7	0.000133411
RUNX1	2	0.000303835
CBR1	3	0.0000953
PADI4	1	0.0000543
ADIPOR1	4	0.000264859
M1AP	7	0.000147538
BTG2	1	0.000126726
HK2	0	0.000107241
C1R	7	0.0000551
THEM4	2	0.00012711
AMFR	4	0.0000929
RGL4	0	0.0000287
ACE	7	0.0000522
TEPP	7	0.0000614
SPON2	3	0.0000753
CHCHD6	2	0.000180819

LRRC36	7	0.000237246
ATP6V0D1	0	0.000216338
CARMIL2	1	0.000135877
C16orf86	3	0.0000708
PIP	7	0.000128517
RGS12	2	0.0000854
ZYX	1	0.000111543
ZNF230	0	0.000153365
ZNF221	3	0.0000331
GNE	3	0.000385184
TNFRSF13C	11	0.000072
PTGIR	1	0.000024
CALM3	1	0.00006
CCDC28B	3	0.000073
TMEM234	1	0.000248052
ZBTB8A	7	0.000233459
ATAD3B	3	0.000118849
UBE2J2	1	0.000422511
CCDC58	3	0.000195422
KALRN	7	0.000159284
ABCG1	0	0.0000413
UBASH3A	2	0.00011429
RSPH1	7	0.000295217
PKNOX1	2	0.000220134
HSF2BP	7	0.0000557
RRP1B	2	0.000276399
PDXK	1	0.00025363
G6PD	1	0.000149621
CSTB	3	0.0000242
GATD3A	33	0.000154847
ZNF66	0	0.0000423
LRRC3	7	0.000129092
ITGB2	0	0.0000973

FTCD	7	0.000225292
SPATC1L	7	0.0000517
LSS	2	0.000142533
VAV2	1	0.0000468
MCM3AP	2	0.000322498
C21orf58	1	0.0000422
PCNT	2	0.000197058
DIP2A	2	0.0000731
PRMT2	3	0.000320019
CLDND2	3	0.0000781
CACFD1	1	0.0000264
SLC2A6	1	0.0000636
ZNF761	3	0.000133421
FCN2	7	0.000240594
C9orf116	7	0.000172799
LCN1	7	0.000193071
ZNF714	3	0.0000882
GPSM1	2	0.0000502
TOR2A	1	0.000160858
SHKBP1	1	0.000103126
RDH13	2	0.0000289
ZER1	4	0.000157938
ZDHHC12	1	0.0000417
BRSK1	7	0.000305166
NLRP4	7	0.000215583
MED27	2	0.000342865
DEDD2	0	0.000319403
MPZL3	0	0.000190132
JAML	0	0.000249094
NEK8	1	0.000100475
TLCD1	7	0.000164451
CD3G	3	0.0000956
CXCR5	11	0.000123404

ZBTB7B	1	0.000193993
NLRX1	1	0.000187597
IL6R	0	0.000271397
UBE2Q1	0	0.000337
ANO10	0	0.000231118
PAQR6	1	0.0000254
PMF1	0	0.000113916
SLC25A44	1	0.000257482
CCR5	3	0.0000617
UBQLN4	2	0.000234928
FCRL3	11	0.0000305
NACC1	1	0.000178391
HK3	0	0.0000543
IER2	0	0.000155743
LY6E	5	0.000128607
VPS28	0	0.0000261
TONSL	7	0.00017754
PTGER1	7	0.000148236
ZNF333	0	0.000265724
COL26A1	7	0.000100162
PPP1R16A	1	0.0000567
ORAI2	1	0.0001325
SH2B2	1	0.000088
MRNIP	2	0.0000909
SQSTM1	0	0.000253779
RPL8	3	0.000136149
MAML1	1	0.000264333
LRWD1	1	0.000128449
FBXL13	34	0.000154847
MFSD12	1	0.000138438
USP41	7	0.000247569
YDJC	3	0.000141064
AP2M1	4	0.000147918

DMKN	7	0.000231878
U2AF1L4	1	0.0000387
BDH1	2	0.000164163
PLXDC1	2	0.0000309
IKZF3	2	0.000120991
FDXR	2	0.0000328
ACOX1	0	0.000168775
PRPSAP1	2	0.000338088
CYGB	7	0.000228693
ZNF577	3	0.000259227
ALDH16A1	3	0.0000711
ITGA5	1	0.000254
ZNF385A	1	0.000101316
EMC10	3	0.000191475
JOSD2	3	0.0000738
PLCD3	7	0.000292679
FMNL3	2	0.000203882
RAVER1	1	0.000185261
SPC24	14	0.000167261
ALOX15	13	0.000105312
TREML1	4	0.000216911
CXCL16	1	0.0000298
SCIMP	2	0.0000265
TNFSF13	0	0.0000817
EIF4A1	2	0.000238086
CCDC42	7	0.000276213
POLR3K	2	0.000245933
SNRNP25	3	0.0000602
JMJD8	1	0.000188332
CCDC78	7	0.0000393
TEDC2	7	0.000224657
CCNF	14	0.0000411
FLYWCH2	3	0.000112347

ADCY9	2	0.0000432
CYB561A3	2	0.000135272
PPP1R32	1	0.0000545
ASRGL1	13	0.0000315
GNG3	7	0.000109026
UBXN1	3	0.000345853
LBHD1	2	0.000234511
RPL29	3	0.0000926
RPS6KA4	1	0.000151061
LRP5	7	0.000286439
TAL1	4	0.00021115
BEND5	11	0.0000413
ZYG11B	0	0.000256537
ACOT11	7	0.0001
PARS2	2	0.0000413
LEXM	3	0.000073
KLHL21	1	0.000220487
ZSWIM5	2	0.0000235
SELENON	1	0.000099
JAK1	0	0.000251571
CTRC	7	0.000191566
RBP7	0	0.000033
SLC25A34	1	0.0000369
DHRS3	3	0.0000657
LAPTM5	1	0.00021139
SDC3	7	0.0000781
PEF1	0	0.000329626
TSSK3	7	0.0000803
TMCO4	2	0.000254216
CAMK2N1	7	0.000176323
ALPL	1	0.0000517
SCNN1D	1	0.0000529
MXRA8	7	0.000127242

C1orf87	7	0.000159758
NFIA	4	0.0000468
DNAJB4	3	0.000134041
GBP4	5	0.00015384
GFI1	3	0.000100621
VCAM1	7	0.0000292
ZNF281	0	0.000171947
ARPC5	0	0.000223061
NLRP3	0	0.000100191
ZNF496	1	0.000065
TRIM58	4	0.000214235
IGSF8	1	0.000165973
DDR2	7	0.000297827
PEA15	3	0.0000772
SLAMF6	2	0.000200808
OLFML2B	7	0.00014159
FCGR3B	0	0.0000271
FLVCR1	3	0.0000385
ATF3	5	0.0000538
DENND2D	2	0.000282441
IER5	3	0.000162711
ACP6	2	0.000161894
WDR64	7	0.000189063
TFB2M	3	0.000256635
PKDCC	7	0.000233849
OXER1	1	0.0000377
MAPKAPK2	1	0.000261842
IL24	7	0.000237846
FCMR	2	0.0000901
PIGR	11	0.00015883
WDR26	0	0.000143691
REL	2	0.000307666
KIAA1841	0	0.00019283

RFTN2	7	0.000269173
DISC1	2	0.000037
MEMO1	2	0.000150736
MAIP1	3	0.000225686
DUSP19	3	0.0000855
NUP35	3	0.000315143
H3-3A	0	0.000180808
COQ8A	0	0.0000829
SGCB	3	0.000145628
BBS5	7	0.000140911
PDLIM5	0	0.000326142
NEURL3	7	0.000254043
ANKRD23	2	0.00020727
CTSS	0	0.000199677
BNIPL	1	0.0000259
SCNM1	1	0.0000444
VPS72	3	0.000314676
ERCC3	2	0.000417463
RNF149	0	0.000262748
BOLA3	3	0.0000277
CDC42EP3	0	0.0000902
S100A11	1	0.0000427
ARHGAP25	0	0.00031028
S100A9	0	0.0000578
S100A12	3	0.0000281
TGFA	0	0.000110205
NIPAL1	31	0.000154847
GPR155	0	0.000170343
PMVK	3	0.000191451
PBXIP1	1	0.000112126
PYGO2	1	0.000241688
HIPK1	0	0.000232318
DCST2	7	0.0001899

DCST1	7	0.000207914
COL6A3	7	0.000168497
YY1AP1	2	0.00011231
KBTBD8	3	0.0000715
SLC22A15	0	0.000158683
ATP1A1	2	0.000340187
SLC15A2	2	0.0000768
EIF4E3	0	0.000143898
PROK2	0	0.0000376
ELF3	7	0.000173958
IGFBP7	3	0.0000728
TRIM46	1	0.000064
CXCR1	0	0.0000984
TSACC	7	0.000179058
STK36	2	0.000215191
ADORA1	7	0.000285628
CCDC141	11	0.0000461
IHH	7	0.000271108
CIP2A	3	0.0000573
EOMES	3	0.0000923
HDAC11	2	0.0000244
FCRL1	11	0.000169968
SGO2	3	0.000082
SERPINI1	3	0.00014297
NUAK2	1	0.00021519
SPTA1	4	0.000062
MNDA	0	0.000118982
PYHIN1	3	0.000174336
PPM1L	2	0.0000725
ICA1L	24	0.0000479
CTLA4	3	0.0000341
ICOS	3	0.000151714
RYBP	0	0.000244954

PPP4R2	3	0.000155737
CCDC191	3	0.000095
CDS1	7	0.0000319
WDFY3	0	0.000147934
PTPN13	2	0.0000332
C4orf36	7	0.000150253
ERICH6	7	0.000209565
HESX1	5	0.0000932
RPL9	3	0.0000445
SMIM14	3	0.000274475
RBM47	0	0.000173172
APBB2	7	0.0000859
IL17RE	7	0.000102609
CRELD1	2	0.000330567
PCOLCE2	7	0.000110653
CXCL3	7	0.000224565
PPBP	4	0.0000686
PF4	4	0.0000815
CXCL1	0	0.0000251
RCHY1	3	0.000253817
PLSCR2	5	0.0000276
CPA3	23	0.000188573
GYG1	0	0.0000822
SNRK	0	0.000300196
UCN	1	0.0000237
SPDYA	0	0.000114277
KIF15	14	0.000106307
CDCP1	7	0.000196568
LRRC2	21	0.000124968
ZNF148	0	0.000278056
NMNAT3	2	0.0000393
LIPH	4	0.0000469
IFT122	2	0.00027603

BAP1	0	0.000278929
TKT	0	0.000221353
PRKCD	1	0.00020791
RFT1	2	0.000355438
UVSSA	1	0.0000537
SLBP	0	0.000268383
SLC51A	7	0.000244453
UBXN7	2	0.000339833
PIGX	0	0.000194652
C1orf50	2	0.000250333
ERMAP	4	0.0000463
ZNF691	3	0.000436666
SGMS2	0	0.0000639
SLC9B1	3	0.00010643
SLC9B2	3	0.00023798
BDH2	3	0.00010829
CDC25A	14	0.00014003
CAMP	10	0.000228888
SHISA5	1	0.0000897
RNF123	4	0.000204348
MON1A	3	0.000177054
GRM2	7	0.000295979
DUSP7	1	0.000142335
NDST3	7	0.000104308
HMGB2	3	0.0000536
SCRG1	7	0.000171273
ANXA5	0	0.000102942
GUCY1A1	4	0.000109105
TMEM144	2	0.0000257
GASK1B	0	0.0000257
ARFIP1	0	0.000180423
ELOVL7	4	0.000214488
NDUFAF2	3	0.000196013

STARD4	3	0.000108535
CCDC112	3	0.000149968
ANKRD33B	1	0.0000236
C5orf63	3	0.000203843
WDR41	3	0.000297808
RHOBTB3	3	0.0000235
SERINC5	2	0.000181142
EBF1	11	0.000101145
TLR3	3	0.0000273
ACSL6	3	0.0000337
LEAP2	2	0.00019609
GJB7	7	0.00027045
GRIK2	7	0.000169332
CREBRF	0	0.000244172
SAMD3	3	0.000133333
STXBP5	0	0.00017725
KIAA0895	7	0.000207042
RPS14	3	0.000100219
COG5	3	0.000218383
GPR85	7	0.0000365
SLU7	0	0.000207599
RP9	3	0.000242367
PTTG1	14	0.000222197
STEAP1	7	0.00026084
CDCA7L	3	0.0000598
USP49	0	0.000132507
SYTL3	1	0.0000833
IQUB	7	0.000267797
FABP5	14	0.000191409
TAGAP	0	0.000166737
CHMP4C	7	0.000176025
LMTK2	1	0.000168295
CTSB	4	0.000126597

C8orf48	7	0.000228938
SUN3	7	0.00024325
C7orf57	7	0.000240736
DEFA4	10	0.000249766
OSGIN2	0	0.00012406
DEFB1	7	0.00010829
SUN1	2	0.000307582
GPER1	7	0.0000279
NOS3	7	0.0000771
MICALL2	7	0.0000257
CDK5	3	0.000158452
TMUB1	3	0.0000849
FMC1	3	0.0000853
BAALC	7	0.000229842
SLC25A32	3	0.000211272
TP53INP1	0	0.00018874
GEM	7	0.000151844
TMEM67	3	0.000169566
C9orf24	7	0.000215352
MYORG	7	0.000316141
CCDC171	3	0.0000236
UBAP1	4	0.0000845
DIRAS2	7	0.000270372
SYK	0	0.000259678
ABCA1	0	0.0000412
NFIL3	0	0.000142495
LETM2	0	0.0000431
NKX6-3	7	0.00014332
TMEM71	0	0.000210979
CPA6	7	0.0002594
ALDH1A1	0	0.0000262
KDM1B	1	0.000204711
HGSNAT	2	0.000301656

RASEF	7	0.000304499
KIF27	0	0.0000889
C9orf64	0	0.000288232
HNRNPK	0	0.000279655
ANKS6	2	0.000216894
FBP1	0	0.000055
CYBB	0	0.000120182
MID1IP1	0	0.000136629
KIAA1958	5	0.0000742
STRBP	3	0.000201623
ZNF367	3	0.0000879
NLGN4Y	12	0.0000936
AQP7	7	0.000343015
NOL6	1	0.000236712
VCP	0	0.000207527
STOML2	4	0.000129425
HECTD2	3	0.0000242
INTS6L	3	0.000361703
SPTSSA	3	0.000120142
MARCHF8	4	0.000237684
TSHR	7	0.000233067
ZCCHC24	1	0.0000259
PGM2L1	3	0.0000623
CRYL1	3	0.000253881
SKA3	14	0.000117589
DDIAS	0	0.0000258
EML5	3	0.000169004
ARF6	3	0.000337354
TTC8	3	0.0000506
NOXRED1	0	0.000138055
FAAH2	3	0.0000831
OXGR1	7	0.000233974
TAF3	2	0.000302611

COMTD1	3	0.0000572
PDZD8	0	0.000127918
ZNF503	7	0.0000606
QSOX2	2	0.000149172
CLEC1B	4	0.0000335
TMEM52B	7	0.000216705
ENTR1	3	0.00027757
SPACA9	7	0.0000726
BORCS5	2	0.000176812
ZMYND19	3	0.00024259
DDX21	3	0.000282753
STK32C	3	0.000055
JCAD	7	0.000301138
FUNDC2	3	0.000138808
METTL17	2	0.000330627
NDRG2	2	0.0000509
ARHGEF40	1	0.0000943
CASP7	3	0.0000947
CCDC186	0	0.000207578
SALL2	7	0.000150888
ZFYVE1	1	0.000312867
C10orf82	7	0.0000383
FRAT1	0	0.0000836
UBTD1	1	0.00007
ANKRD2	7	0.000224083
LARGE2	1	0.0000688
TTC7B	7	0.000133804
PSMC3	3	0.000188852
RAPSN	1	0.0000564
AGBL2	2	0.0000269
TC2N	3	0.000188454
IFI27L1	3	0.0000364
CLMN	2	0.0000584

SMCO4	0	0.0000244
AMOTL1	7	0.0000483
HTRA1	7	0.0000236
TCP11L2	0	0.000110405
CMTM5	4	0.0002154
GLB1L3	7	0.000343783
AMN	7	0.0000542
RAB8B	0	0.000202977
IKBIP	0	0.00010143
RPUSD2	3	0.000278148
NDUFB8	3	0.000153055
ZFYVE19	2	0.000253635
PPP1R14D	7	0.000194613
SPINT1	1	0.000037
FBN1	7	0.0000481
LRTM2	7	0.000297969
BRD7	3	0.000314783
BTRC	2	0.000295814
POLL	4	0.000128612
DPCD	4	0.0000292
HPS6	1	0.000140455
SENP8	24	0.000111263
PCBD1	3	0.000137941
C16orf71	7	0.000244386
CLMP	7	0.000291324
ZNF202	3	0.000392418
MYRFL	7	0.000255461
C2	2	0.0000942
PLEKHF1	3	0.0000626
APBB1	2	0.000120196
C11orf65	7	0.0000297
DCHS1	1	0.0000249
CYB5A	3	0.000138999

WDR88	7	0.0002453
PPFIBP2	2	0.0000529
CYB5R2	7	0.000225754
SERPINB8	0	0.000190291
RIC3	3	0.000115403
ZMAT1	3	0.000272277
TRIM66	2	0.0000655
RNF169	0	0.000349199
RPL27A	3	0.000105071
CDYL2	2	0.000129733
C16orf46	7	0.0000495
PRKCB	0	0.000265115
CLEC4E	0	0.0000466
RIMKLB	3	0.000253746
TK2	0	0.0000866
SLC38A8	7	0.000298369
IQCD	7	0.0000248
NDEL1	0	0.000225588
CENPV	3	0.0000293
RRAD	7	0.000237031
CIAO2B	3	0.000270721
ATF7IP2	3	0.000213372
TVP23A	7	0.000332051
BEX3	4	0.0000306
COG1	2	0.000345557
PLEKHA7	7	0.0000718
ZCCHC18	11	0.0000346
NNMT	7	0.000259648
ACSM1	7	0.0000524
CATSPER2	2	0.000101912
MARF1	1	0.000135537
LDHAL6A	7	0.0000606
PCLAF	14	0.000218687

ANPEP	1	0.0000954
RBPMS2	7	0.000102475
PLK1	2	0.0000361
TAC3	7	0.000222134
MYO1A	7	0.000310416
NEMP1	3	0.000311289
NAB2	3	0.000113969
STAT6	1	0.000252174
PATL1	1	0.000148591
ATP23	3	0.0000467
STX3	0	0.000189545
PIP4K2C	2	0.000307201
YWHAB	0	0.000347595
SCG5	7	0.0000246
MS4A6E	7	0.000167296
MS4A7	0	0.00004
MS4A14	2	0.0000267
EPB42	4	0.000172536
SMAD3	2	0.000182807
MAP1A	4	0.0000707
MAPRE2	2	0.000264142
EVA1C	2	0.0000757
TCP10L2	7	0.000313512
MARS1	1	0.000340566
PDIA3	2	0.000279116
DUSP18	1	0.000153049
TEF	2	0.0000952
MEI1	2	0.00020085
GNGT2	3	0.0000403
TTC16	3	0.0000787
FAM102A	1	0.0000711
GOLGA2	0	0.000215285
COQ4	2	0.000212463

SLC27A4	1	0.000143639
URM1	2	0.000160293
CCDC103	7	0.0000508
ENDOG	3	0.0000443
GPRC5B	7	0.000243788
CRK	0	0.000344993
RNF214	2	0.000225146
DPEP2	1	0.000178285
DUS2	0	0.000153141
MYO5B	7	0.000280842
OR51B5	7	0.000163002
PRRT2	7	0.000111467
ZNF23	3	0.000186984
ZNF668	1	0.0000951
ZNF646	1	0.000264296
VKORC1	3	0.0000948
CA4	0	0.0000311
TPM4	0	0.00013966
GPX4	3	0.0000607
JSRP1	4	0.0000259
NIBAN3	11	0.000187878
KLHL26	1	0.000150834
CDT1	7	0.000081
PROCA1	1	0.0000854
RPL13	3	0.000100071
ZNF641	0	0.0000842
CACNB3	7	0.0000378
DHRS13	0	0.0000843
TUBA1A	0	0.000174484
TUBA1C	0	0.0000914
SERTAD3	0	0.000137078
RAB4B	1	0.0000909
GPD1	7	0.000290177

PROSER3	1	0.000114106
CYP2S1	7	0.0000327
NFKBID	1	0.000159141
TRAPPC9	2	0.000209852
NLRP7	7	0.00025857
ZNF283	3	0.000301013
PPP1R14A	7	0.0000236
SPINT2	0	0.000162364
PSCA	7	0.0000912
DAPK3	0	0.000192885
CHAF1A	1	0.0000904
UBXN6	4	0.000167924
HDGFL2	0	0.000118089
PLIN4	1	0.0000684
SEMA6B	7	0.0000961
TLCD3A	7	0.000027
MFSD3	3	0.0000938
GPT	7	0.000259354
SLC43A2	1	0.000207504
RILP	4	0.000156361
TRPV3	7	0.000299109
ZNF83	3	0.0000553
CD320	1	0.0000289
SPRYD3	1	0.000214778
SOAT2	7	0.0000977
PRDX2	4	0.0000992
CD300C	1	0.0000507
CD300A	0	0.000159983
TMEM88	1	0.0000866
TMC8	1	0.000185802
MLST8	3	0.000101973
DNASE1L2	1	0.0000258
NLRC3	2	0.000237176

DDB1	4	0.000101497
VPS37C	1	0.000206618
BEST1	1	0.000161124
FTH1	1	0.0000249
BSCL2	2	0.000160962
SLC3A2	3	0.000234011
PLAAT5	20	0.000176902
ATG16L2	1	0.00020049
TTC21A	1	0.0000422
ULK4	2	0.000237107
FADD	0	0.000239734
LTBP3	1	0.0000758
NAALADL1	3	0.000113692
SAC3D1	3	0.0000245
BATF2	5	0.000160195
SF1	2	0.000280204
CCDC88B	1	0.00011541
SCARA5	7	0.000130219
PNOC	11	0.0000999
VASN	7	0.000157676
RNF187	4	0.0000477
DDIT4	3	0.0000236
PTGDR	3	0.0000624
TTC39C	2	0.000270595
GLYCTK	1	0.000253956
POLR2J3	35	0.000154847
IRF2BP2	3	0.000423794
KIF5C	2	0.0000586
BMI1	3	0.000185238
PXK	0	0.000195311
H1-4	3	0.0000364
KCTD6	3	0.00035567
ACOX2	7	0.000296775

CX3CR1	3	0.0000373
SEPTIN2	3	0.000378026
MFSD2A	1	0.0000251
TAP1	5	0.00013454
MLKL	0	0.000135657
RHOH	3	0.000277185
TXNDC2	7	0.0000239
RAB31	0	0.00020282
REEP4	1	0.0000243
BMP1	1	0.000102241
CCDC110	7	0.000232865
CAVIN2	4	0.000185056
MTCL1	7	0.000269955
GFRA2	7	0.000153708
SLC20A2	2	0.000342258
DYNLRB2	7	0.0000926
ADAM29	11	0.0000913
STAT3	1	0.000244225
ZSWIM1	1	0.000123118
ADAM9	0	0.000208587
AXIN2	2	0.0000446
LRATD2	3	0.000138568
LDLRAD4	2	0.000284535
IL7R	3	0.000150556
PKIG	11	0.000097
TET2	0	0.000223733
TCTN2	2	0.0000309
PPIP5K1	2	0.000240637
TSPAN5	4	0.000258717
ABHD15	3	0.000256318
ZBTB5	3	0.000357811
VAMP5	5	0.0000267
LRRC28	2	0.000288965

LGALS9	1	0.0000658
ATP5ME	3	0.0000392
SLC49A3	1	0.0000505
COL4A3	11	0.0000953
MAP2K1	2	0.000358182
HNRNPH1	3	0.000326689
MECP2	1	0.000224151
AR	7	0.0000396
HSPBAP1	0	0.000186231
FAM110B	7	0.00017021
ZNF354A	0	0.0000419
NPIPB12	2	0.0000239
CD2BP2	0	0.000319837
RGS14	1	0.000216262
TBC1D10B	1	0.000222575
GCSAML	23	0.000179366
RAB24	0	0.0000818
PRELID1	0	0.000179871
THBS3	1	0.000135543
CA5B	3	0.000185983
SH3TC2	4	0.000188728
ZRSR2	3	0.000100122
ADRB2	3	0.0000743
GPRIN1	7	0.000178545
HSPB3	7	0.0000242
KCNAB1	7	0.000290656
PGM2	0	0.000229729
P2RY12	4	0.0000771
CRADD	2	0.000291067
ARL13B	3	0.0002168
PTK2	2	0.0000407
RSPH10B2	7	0.000126041
PTAFR	0	0.000127831

MMGT1	3	0.000197814
HTRA4	2	0.0000255
CLIC4	3	0.0000497
ZEB2	2	0.000195965
DTWD2	3	0.000251444
VPREB1	7	0.000130358
INO80E	2	0.000150206
CKAP2L	14	0.000189341
CHRNA5	7	0.00013034
AGPAT2	0	0.000134534
GP9	4	0.0000825
MT1E	14	0.000136655
DUS1L	3	0.000181724
RFNG	1	0.000127547
DCXR	3	0.0000931
ZNF32	3	0.000174804
RAC3	7	0.0000685
LIMS1	0	0.000159698
BTD	2	0.000372634
ROBO1	7	0.0000262
P2RY1	4	0.0000313
AHSP	4	0.0000412
CALML6	7	0.000202513
REPS2	0	0.000133215
SYAP1	0	0.000252083
ITGAM	1	0.000170584
TPST1	0	0.0000289
TM4SF1	7	0.0000822
OTUD7A	2	0.0000344
BRD3	1	0.000329158
KLF13	1	0.000319123
ZNF764	1	0.000204787
ZNF768	0	0.000113856

TAS1R3	7	0.0000623
TMEM42	3	0.000274864
MAP3K2	0	0.000216255
PUSL1	1	0.0000859
ZNF35	3	0.000210867
IFFO2	3	0.000167239
TMEM154	0	0.000236072
ALCAM	0	0.000104659
UBE2E3	3	0.000160053
CNTROB	2	0.000205692
TRAPPC1	1	0.0000507
SIK2	2	0.00030687
GYPA	4	0.0000593
SLC16A5	1	0.000190893
ANKK1	7	0.0000249
ADPRM	3	0.000149857
USP50	7	0.0000478
FAM161A	3	0.0000976
ZNF282	1	0.000257179
CRTAP	3	0.000219752
GABARAP	0	0.000114046
STX8	3	0.000293845
UBB	4	0.0000508
NFRKB	2	0.000305076
FABP4	7	0.0000598
PRDM10	2	0.000402253
SLC30A1	3	0.00010068
GPRC5C	7	0.000207295
TMEM182	3	0.000213822
MGMT	3	0.000209073
AC021066.1	7	0.000238972
DENND5B	11	0.000155819
CD14	0	0.0000381

DNAJC18	3	0.000116678
MZB1	15	0.000201145
SLC23A1	7	0.000266351
NPAS2	7	0.00023106
KRT72	36	0.000154847
LONRF2	7	0.000286888
PA2G4	4	0.0000962
ELOVL6	3	0.0000711
PFKFB3	1	0.0000612
ARL6IP1	3	0.0000999
SERPINB9	2	0.000154842
STAT2	5	0.000175784
ZNF16	3	0.00030228
CAVIN4	7	0.000171001
HOXB9	7	0.000251877
AKAP13	0	0.000278092
CDCA4	3	0.00017424
USP32	0	0.00026647
CEL	7	0.000235581
GPR27	0	0.0000658
LSM3	3	0.0000756
KIAA0232	0	0.000267355
MTSS1	2	0.0000659
TMEM43	0	0.000294216
RPS9	3	0.000122165
PLA2G1B	7	0.0000482
TSEN34	0	0.000187347
NDUFA3	3	0.0000359
OSCAR	1	0.0000526
PAQR8	2	0.000210446
NUDT6	3	0.0000741
TANC2	0	0.000145988
ZNF160	3	0.000341919

CEACAM3	1	0.000125801
PDGFD	3	0.0000637
S1PR1	3	0.000231579
PKIA	3	0.0000754
XKR6	2	0.00019184
TSNARE1	1	0.000106067
FPR2	0	0.0000888
FPR1	0	0.0000931
C11orf24	2	0.000106841
INSR	1	0.0000889
KCNMB3	3	0.0000699
KCNG3	7	0.000238195
ATP6V0E2	2	0.000128882
PRKCE	2	0.00009
MORN4	7	0.0000243
ZNF692	2	0.000213541
NAIF1	0	0.000332073
RBKS	3	0.0000531
TMEM126A	3	0.00015067
TRIM8	1	0.000238079
NETO2	3	0.0000253
JUNB	0	0.0000567
TMEM37	7	0.000269765
LRG1	0	0.0000555
ZNF440	3	0.000293401
GAA	1	0.0000916
CHST11	0	0.000248081
PGAM1	0	0.000212159
CHD7	2	0.000225712
LURAP1	7	0.0000489
CLCN5	2	0.000156947
KCND3	7	0.0000491
ZNF581	1	0.0000554

POLR1C	3	0.000343597
ASXL1	2	0.000328603
ZNF318	2	0.000250408
WIPF2	1	0.000219612
HOPX	3	0.0000574
LRRC8C	3	0.000264255
SPACA5	7	0.0000271
LRRC8D	3	0.000385683
OR1L8	7	0.000134933
PPID	0	0.000301652
COL24A1	7	0.0000798
PTGER4	0	0.00025854
BCL2L1	4	0.000223494
NMUR1	3	0.00010132
CXXC5	2	0.0000668
PTCRA	4	0.000229358
SLC25A33	3	0.000203103
P2RY6	7	0.000029
S100Z	2	0.0000545
ZIK1	3	0.000160476
GPR82	22	0.000154847
PLEKHG5	7	0.000197651
GPHN	2	0.00015229
SPATA5L1	0	0.000144121
SYCE1	7	0.000134826
RASGRP4	1	0.000128433
NHLH1	7	0.000173606
BCL2	3	0.000157764
CTPS1	2	0.000282434
KNDC1	7	0.0000475
MLLT3	3	0.000200114
FAM90A1	7	0.000209042
RRM2	14	0.000189098

RPS21	3	0.0000512
PTEN	0	0.000179771
RNASEH1	2	0.000380809
ZNF217	0	0.000314125
SRGAP2C	0	0.0000285
CYP4F22	7	0.0000241
DRC3	7	0.000209238
SHLD1	3	0.0000353
JMJD1C	0	0.000220749
LDHAL6B	7	0.000211271
ZNF556	7	0.000241962
MAL	3	0.0000399
ZNF554	2	0.000162196
RAB33B	0	0.000162886
EPHX4	7	0.0000481
ORMDL3	3	0.0000508
KLF11	2	0.0000656
SMN1	3	0.0000853
EIF2AK3	3	0.000316504
CD8B	3	0.0000336
SLFN12	0	0.0000739
SLC9C1	7	0.000149677
FRMD3	5	0.0000302
SNTB1	2	0.000200977
OR4X2	7	0.0000671
CXCR6	3	0.0000364
CEBPB	0	0.0000637
AZU1	10	0.000102597
TPSAB1	7	0.0000739
CLEC7A	0	0.000160498
DPAGT1	2	0.000148142
BSG	4	0.00018073
HINFP	2	0.00029053

CERS6	3	0.000304814
COPRS	3	0.000082
CLEC12A	37	0.000154847
BPGM	4	0.000158578
POP7	3	0.0000935
ALG14	3	0.000115779
STARD5	2	0.0000472
CSDC2	7	0.000219484
GNB2	1	0.000190872
OR5B12	7	0.0000239
OR5B2	7	0.0000246
PDZD3	7	0.00017149
PRSS27	7	0.000285907
SYNPO2	7	0.0000242
FGGY	0	0.0000874
ACOT12	7	0.000277602
CARNS1	1	0.0000558
PPP1CA	0	0.000175254
NIPAL4	7	0.000162715
MUCL1	7	0.000230002
PDE3A	4	0.000135037
RASGRP1	3	0.000270939
KLHL6	2	0.000290063
SMPDL3A	0	0.0000294
RND1	7	0.000244254
AGAP5	2	0.000123043
ZFAND4	3	0.000204215
THEMIS	3	0.0000958
CORO1B	3	0.000194285
TMEM217	7	0.0000277
TMCC1	0	0.000169563
EFCAB12	7	0.0000321
RAB43	1	0.0000774

CBWD1	3	0.000145837
HOXC5	7	0.0000686
RAB37	0	0.000173525
DCP2	0	0.000239579
SNX32	7	0.000223031
RPL38	3	0.0000769
RARG	1	0.000125478
SSH3	1	0.000169869
SP3	0	0.000241402
KRT2	7	0.00026397
METAP1D	2	0.000169511
NBEA	3	0.0000402
ANKRD13D	1	0.000229487
MYD88	1	0.00012225
OXSR1	0	0.000355279
SLC22A13	7	0.000178069
LCLAT1	2	0.0000976
KAT5	0	0.000329802
GXYLT2	7	0.000242101
ARPP21	7	0.000199901
EVC2	7	0.0000237
HECTD4	2	0.000224573
FAM222B	1	0.000108103
BNC2	3	0.0000476
RXFP4	7	0.000105976
HSPA6	0	0.000103508
ADCK5	1	0.000117175
MTX1	0	0.000135987
PARP14	5	0.000158335
PARP15	2	0.000170036
CKS1B	3	0.000104154
ABCD2	3	0.000135666
TUBB8B	7	0.0000236

MFSD4B	2	0.00035253
VANGL1	2	0.0000275
GLRX	0	0.00015696
GOLGB1	0	0.000275626
GPR137	1	0.000113289
SNCG	7	0.000176003
MZT2A	3	0.0000796
ZNF449	3	0.000278743
PPP1R3B	0	0.000171322
MAP3K11	1	0.000240488
TRIB1	0	0.0000882
KCNK7	1	0.0000241
C1QB	38	0.000154847
C1QA	38	0.000154847
IQCF1	7	0.000217222
OLR1	10	0.000184422
DAB1	7	0.0000374
EHBP1L1	1	0.000188601
PPP1R14B	3	0.0000683
ZNRD2	3	0.0000729
VEGFB	1	0.0000295
TNFRSF10D	2	0.000188599
MST1	2	0.0000287
TNFRSF10C	0	0.000152455
SNX33	1	0.000110257
NABP1	0	0.000178732
ADGRF3	3	0.0000275
CCDC106	7	0.0000274
CEP83	3	0.000299219
SULT1B1	0	0.0000915
NUDT4	4	0.000123697
PC	7	0.000137126
UGT2A1	7	0.00010448

NMNAT1	0	0.000291346
TRAPPC3L	4	0.0000435
SLC19A1	1	0.000187079
EIF1AX	3	0.0000588
C11orf80	11	0.000142848
STAT5B	1	0.000261011
CD7	1	0.0000408
CNP	1	0.0000507
EIF1	0	0.0000391
TIGD3	1	0.0000405
NET1	3	0.0000501
DPY19L1	3	0.000168636
AC013489.1	3	0.0000299
PHOSPHO1	4	0.000053
GPR160	0	0.0000818
SWSAP1	0	0.0000808
SLCO4C1	0	0.0000822
TCAP	1	0.0000409
CCS	4	0.000109676
CEP19	0	0.000100723
GNG5	0	0.0000729
CTSF	2	0.0000385
TLR10	3	0.0000332
TLR1	0	0.000154554
TLR6	0	0.000182882
FAM174A	0	0.000111281
FAM53A	7	0.0000713
ZDHHC24	1	0.000270458
SELP	4	0.000234112
CTU2	1	0.0000466
C12orf66	3	0.000306179
REP15	7	0.0000268
PITPNA	1	0.000174081

ZBTB4	1	0.000217278
TNK1	7	0.0000376
ZHX3	2	0.000197803
SLC16A11	7	0.000146593
SLC16A13	1	0.0000748
PODN	7	0.000049
C11orf45	7	0.000029
TRHR	7	0.00018648
ATP2A2	2	0.00030315
ZWILCH	3	0.000129283
CNTNAP2	11	0.0000574
BBS1	2	0.0000661
GCSAM	3	0.0000919
MFSD4A	7	0.000047
PELI3	1	0.0000839
TMEM81	0	0.000320969
IL20RB	2	0.0000397
GOLT1A	7	0.00016151
ZNF497	2	0.0000287
CMKLR1	3	0.0000392
UGT8	11	0.000119446
IQCK	7	0.000189558
SLC29A2	1	0.0000856
B4GAT1	3	0.0000486
TMEM167A	3	0.000195786
SH3PXD2B	7	0.0000641
RESF1	0	0.000161665
LARP7	3	0.000140453
BRMS1	0	0.00019483
HRAS	1	0.0000474
WDR49	0	0.0000596
ODAPH	7	0.000129216
PDZK1	7	0.0000639

GLMN	3	0.00026109
NLRP6	1	0.0000245
NDUFA11	3	0.0000267
CATSPERD	7	0.000294087
RAB1B	1	0.000183803
P2RY14	5	0.000146197
AMZ1	7	0.0000338
GPR171	3	0.000073
FBXW8	2	0.0000967
KLC2	1	0.000185328
SLC22A1	1	0.0000799
CTBP2	0	0.000267993
CHST2	3	0.0000541
ZDHHC14	2	0.0000352
GK5	3	0.000130874
MARCKSL1	1	0.0000291
SH3BP5L	0	0.000274616
YPEL2	2	0.000394434
PSMD2	0	0.000283554
PARL	2	0.000215357
DDIT3	0	0.000123926
PCCA	2	0.000273703
GOLGA8A	2	0.0000575
TP53I11	1	0.000101904
DOLK	3	0.000261694
PHYHD1	7	0.00020826
CATSPER1	3	0.0000266
CCNE2	14	0.000228242
PHYKPL	1	0.000299676
BANF1	3	0.0000266
ARL10	2	0.000218747
RFESD	3	0.0000981
SART1	0	0.00011375

MCTP1	0	0.000184189
POLD4	0	0.000182767
LRRC25	1	0.000167761
CLCF1	1	0.0000239
TSGA10IP	7	0.000158729
LIPT2	3	0.0000737
KCNE3	0	0.000210239
LONRF3	2	0.000152926
UCP3	2	0.000136224
PAAF1	2	0.000341344
P2RY2	13	0.000114857
FOSL1	7	0.0000743
SUGCT	3	0.00012046
OR4B1	7	0.000188693
B3GNTL1	1	0.000109339
AURKAIP1	0	0.000220132
TTLL11	2	0.00016459
ZNF169	2	0.00034617
MSRA	2	0.000138303
CBLL2	7	0.000029
CCDC168	7	0.000250608
SWI5	3	0.000133064
BAIAP2	7	0.000105689
A2M	7	0.000170634
DOK7	7	0.0000724
UBE2O	4	0.000114817
ORAI3	0	0.000251504
ZNF613	3	0.000150765
JAKMIP2	3	0.0000394
TPRN	1	0.00011791
CRYBG2	7	0.0000278
SSNA1	3	0.000147728
TCP11L1	2	0.00015291

HSF5	3	0.000106037
ACBD7	7	0.00019461
SLC35G1	3	0.000120837
COX8A	3	0.0000685
ZSCAN2	3	0.000362437
RNPEP	1	0.0000974
GJC3	7	0.000222988
SPRYD4	2	0.000226891
VPS37D	7	0.000242306
SYNE3	2	0.000297185
SLCO3A1	1	0.000264863
ZNF575	7	0.000150919
WDR25	2	0.000226853
DIRAS1	7	0.000206601
PHLDB3	2	0.0000504
USF3	0	0.000311046
KBTBD11	0	0.0000924
B3GNT5	0	0.0000624
LMNB2	1	0.00016335
RNF152	21	0.000129044
MYO1D	2	0.0000772
LRRC37A	2	0.0000267
ACSF3	2	0.000253663
PFN4	7	0.0000239
CDK5R1	0	0.0000236
NCKAP5	7	0.00030088
RUFY1	0	0.000127638
BASP1	0	0.0000436
METRNL	0	0.0000467
WSB2	0	0.0000787
TCEANC	3	0.0000516
PNMA1	2	0.000204877
TCIM	7	0.000191772

C8G	7	0.0000289
EFCAB5	7	0.0000588
GCNT4	3	0.000109465
FAM89B	0	0.0000243
SMCR8	1	0.000372075
MTHFR	1	0.000208481
FBXO46	1	0.000130558
ACER2	2	0.0000287
POLE	2	0.000294127
PHETA2	3	0.0000602
RHOG	1	0.000193865
EPS8L2	3	0.0000288
ZBTB34	0	0.000251546
FAM9B	7	0.0000691
TALDO1	0	0.000133716
ULK1	1	0.000139066
B3GNT8	0	0.0000684
OR2T33	7	0.0000312
KCNA3	2	0.000110866
OR2AJ1	7	0.0000981
FBXO39	5	0.0000374
CLDN22	39	0.000154847
BEND2	4	0.0000938
LRRN4CL	7	0.000219549
TIMM22	3	0.000402664
UMODL1	7	0.000316122
SAMD9L	5	0.000118812
PAWR	11	0.000150529
CD19	11	0.000175105
CAVIN1	7	0.0000564
ZBED2	7	0.000230092
ATOX1	2	0.00025759
CD163	0	0.0000278

C18orf32	0	0.000295727
ZNF491	3	0.0000799
HASPIN	14	0.0000794
GBA	1	0.00016848
IL17RA	1	0.000210104
AGTRAP	1	0.000222761
NAALADL2	7	0.000147295
NECTIN3	3	0.0000236
FLII	1	0.000217017
HNRNPA0	3	0.000236207
YIPF7	7	0.0000444
CHID1	4	0.0000984
ZNF620	2	0.000229691
TMEM187	3	0.000102185
SVBP	4	0.0000356
GRB2	0	0.000315151
CENPBD1	0	0.00011248
RPS27	3	0.0000456
RIC8A	0	0.000300965
LCN15	7	0.000226393
ODF3B	5	0.0000528
CALHM5	7	0.0000641
IMPDH2	3	0.000242599
ALS2CL	2	0.0000532
NDUFAF3	3	0.00012586
GRAMD1C	0	0.0000303
STAP2	7	0.000263825
TSSK6	3	0.0000571
PDE4DIP	3	0.000175522
DDX10	3	0.000328628
NDUFV2	2	0.000306949
DALRD3	3	0.000220758
PARD6G	7	0.0000804

ZNF454	7	0.000287246
ZC3H12D	2	0.0000989
POGLUT3	3	0.000159343
PLEC	1	0.000101593
WDR6	2	0.000254593
GEN1	11	0.0000273
AQP11	7	0.0000236
TMEM11	1	0.000237592
KCNG2	7	0.00017044
ZFAND2A	0	0.000181024
PLEKHM3	2	0.000307278
ZNF223	3	0.0000707
CEP295NL	1	0.0000631
TUBAL3	7	0.000249027
UCN3	7	0.000245784
SLC25A20	0	0.0000607
CA8	23	0.000133285
CD28	3	0.000187702
MAF	3	0.0000432
GPR35	1	0.0000316
PARP10	5	0.0000826
SUZ12	3	0.00014626
GRINA	4	0.0000643
THBD	0	0.0000239
STX19	7	0.0000248
ZHX2	2	0.000240815
MPI	2	0.000263946
EFCAB13	3	0.0000235
EXOSC4	3	0.000127131
TAF7	3	0.000101767
PFAS	1	0.000192927
HYI	2	0.0000244
LGALS7B	7	0.0000619

ZNF552	0	0.000278754
SMIM10L2A	7	0.000039
GAK	1	0.000282267
SELENOW	3	0.000191633
SNX18	0	0.000198719
AURKB	7	0.0000354
MRFAP1	0	0.000134966
KLHDC7A	7	0.000106671
TMEM107	2	0.000133007
RRS1	3	0.000176384
EXOC3L1	5	0.000175824
IGSF22	7	0.000224918
CCDC89	3	0.0000449
DPM3	3	0.0000567
C12orf42	2	0.0000797
HTR1F	7	0.0000277
TMTC2	2	0.000115433
SPTY2D1	0	0.000369698
SAMD4B	1	0.000202086
GIMAP7	3	0.0000887
EDC3	2	0.000291078
FUCA1	0	0.000102011
PXT1	7	0.000122644
SIGLECL1	7	0.000260776
MAGED1	2	0.000123223
LDLRAD3	1	0.0000295
SMCO3	7	0.000132838
RAD23A	4	0.0000789
GADD45GIP1	3	0.000104829
NSUN7	0	0.0000357
RAB39A	0	0.0000357
GATA2	23	0.0000709
TMEM31	7	0.000097

EGR3	25	0.0000942
GPC5	7	0.000224984
GEMIN4	2	0.000292367
ZBTB18	0	0.000260164
C14orf28	3	0.000227534
SHARPIN	4	0.0000939
DNHD1	2	0.000165307
SLITRK4	0	0.0000258
GCC1	0	0.000333403
LSMEM2	7	0.0000472
RNF151	7	0.0000391
ZFPM1	1	0.0000826
ZBTB42	13	0.0000427
MAF1	4	0.000241089
FCER1A	23	0.0000685
PCED1B	2	0.000146825
MYADM	1	0.0000237
NKPD1	7	0.00011856
GIPC3	7	0.000166631
ABCA13	10	0.00023009
TIGD5	3	0.000111917
PDXDC1	2	0.00033578
C1orf194	7	0.0000271
ZNF154	2	0.0000238
R3HDM2	2	0.000234898
SEPHS2	0	0.0000677
GPBAR1	1	0.0000574
ZNF784	0	0.0000789
ZNF648	7	0.000297104
CCR8	3	0.0000321
FIZ1	7	0.00006
PUF60	3	0.000283398
TSHZ1	2	0.000275733

ZNF48	3	0.00014593
TMEM86B	1	0.0000549
OR3A1	40	0.000154847
MED14	2	0.000299635
FAHD1	4	0.000118313
TDRP	4	0.0000517
RRH	3	0.000028
FGD6	2	0.0000626
PLD5	7	0.000276995
OAZ2	1	0.000213497
PNPLA1	1	0.0000246
FZD2	1	0.000027
TIGD2	3	0.0000996
HCLS1	0	0.000234463
ZNF609	2	0.000256048
PAK2	0	0.000197309
HARBI1	0	0.000275527
GAS1	7	0.000106777
AC004151.1	1	0.000199189
KCNE1	0	0.0000826
ZSCAN4	7	0.000116025
H2BC4	3	0.0000541
PRF1	3	0.0000823
YOD1	4	0.0000659
TMEM64	3	0.000162084
CHRM4	7	0.0000747
S1PR5	3	0.000105943
CLRN3	7	0.000192491
GPR157	1	0.0000247
SLC36A4	3	0.000184986
PPA1	3	0.0000977
CXCR2	1	0.00005
SSR4	3	0.000115179

CAPS2	3	0.000110185
SCRIB	1	0.000162828
KCTD2	1	0.000315003
ST20	0	0.000127149
PITPNB	2	0.000252321
TCEAL8	3	0.000143369
LRRC57	0	0.000305551
GPR137C	7	0.000249003
OR52N5	7	0.0000242
LSMEM1	0	0.0000795
FKRP	1	0.000166605
TRAPPC5	0	0.000120495
RPH3AL	7	0.0000699
FCRL6	3	0.000123248
EHMT1	2	0.000279521
F2R	3	0.0000622
ZNF746	1	0.000210599
SLC25A41	7	0.00023367
TLCD5	7	0.00021265
FRAT2	0	0.000118041
TMEM102	3	0.000175768
ZNF322	3	0.000171386
NME9	7	0.000325104
DDX60L	5	0.000120996
SYNE4	7	0.0000551
AATK	1	0.000100096
ZNF467	1	0.000156676
TMEM45A	7	0.000230108
RAP2B	3	0.000311642
ZBTB2	3	0.000197692
RNF135	0	0.000250802
ACBD4	1	0.00013944
SGSH	1	0.000140254

TMIE	7	0.0000302
MEX3D	3	0.0000271
GPR135	2	0.0000244
P2RY13	0	0.0000324
PHLDA2	7	0.000154587
ATG9B	7	0.0000813
GPR88	7	0.00022689
ZNF875	2	0.000245017
PLAG1	3	0.0000727
ZBTB20	2	0.000290189
OR2Z1	7	0.000207236
C5orf30	0	0.0000968
AMIGO1	2	0.000132044
SIAH2	4	0.000126422
COPG1	0	0.000310279
LSM10	0	0.0000865
RELL1	0	0.0000845
SLC35C1	1	0.000179693
TIGIT	3	0.0000359
SLC2A4	7	0.000152359
IBA57	4	0.0000317
OR52K2	1	0.0000258
CCDC149	1	0.0000507
CHST15	1	0.000192423
IDH2	3	0.000123183
GET1	0	0.0000323
TNRC18	1	0.000119401
FAM181B	7	0.000264652
NOP10	0	0.00010285
FAM89A	3	0.0000239
TDRKH	2	0.000199505
TSEN54	2	0.000119601
RAD51B	2	0.000273759

ARL6IP4	3	0.0000607
EXT1	0	0.000135491
SHMT2	3	0.000178752
ATP6AP2	0	0.000267129
ZAR1	7	0.000247415
CYB5D1	3	0.0000577
BACE2	9	0.000154847
UBE2E2	2	0.000128386
AP1S2	3	0.000116431
C8orf33	3	0.000431782
DCAF4L1	0	0.0000235
ZSCAN22	2	0.000233186
FBXL6	1	0.0000672
GLTPD2	7	0.0000834
YBEY	3	0.000025
CLN8	2	0.000228346
TSHZ2	2	0.0000328
KPNA2	2	0.0000235
CEP97	3	0.000253149
FES	0	0.000109022
GLRX5	4	0.000194278
FAM104B	4	0.0000789
LIMK2	1	0.000127922
MFSD5	0	0.000212265
SPNS3	13	0.000198973
SATB1	3	0.000230095
ACTL10	3	0.0000703
EPGN	7	0.0000287
TRAK1	2	0.000238609
TSPAN10	7	0.00024319
PLCB1	0	0.0000522
NDN	7	0.0000273
TTC3	3	0.000305724

ANXA2	0	0.000160596
SLC35D3	7	0.0000542
MAFA	7	0.0000884
GRID1	7	0.000310656
RPS17	3	0.00034927
HCAR2	0	0.0000551
MAGEB17	7	0.0000937
DDX28	3	0.000333249
C16orf72	0	0.000321733
RBM10	0	0.000281064
ADGRG3	1	0.0000899
C11orf54	0	0.000289599
CEP63	0	0.000226471
ODF3L1	7	0.000182568
SPATA13	0	0.000291942
ZNF662	2	0.0000638
ZNF320	2	0.0000774
PYCR1	7	0.0000695
ABAT	1	0.0000771
SLC25A10	7	0.0000257
LYSMD4	3	0.000230461
WBP2NL	2	0.0000563
AFMID	0	0.0000251
FREM3	7	0.000244157
BEGAIN	7	0.0000337
ARHGEF37	4	0.0000265
CSMD1	7	0.0000309
PTGDR2	13	0.000211002
GJD3	7	0.000170994
FANCF	3	0.000233851
CTNNA3	7	0.000194665
DDX41	3	0.000281164
ABHD16B	7	0.000162356

OR52E8	7	0.0000349
DAZAP2	0	0.000158118
SPDYE4	7	0.0000483
CCDC125	0	0.00018951
BCOR	2	0.000254015
GBP6	5	0.0000584
OR56A4	7	0.000255783
TMEM89	7	0.0000428
CCDC159	1	0.000177379
RIPK4	7	0.0000783
NPIPA1	2	0.0000303
GTF2H2C	0	0.000183385
TREX2	1	0.0000412
MX2	1	0.0000787
TENT5C	4	0.000141718
KLRC4	3	0.0000375
TANGO2	4	0.000238531
SFXN4	3	0.0000942
GKN2	7	0.000184
MRPL54	3	0.000139409
ZNF438	0	0.000105567
KLHL25	1	0.000132848
TRMT12	3	0.000147334
GPR1	7	0.000109876
ALYREF	3	0.000102662
RFLNB	1	0.0000405
EFHC2	0	0.0000287
NOG	3	0.0000244
MRGPRX2	7	0.000135531
UPP1	2	0.000104695
LHFPL6	7	0.000253654
CMTM4	0	0.00013737
TMEM50A	0	0.000227154

NPBWR1	7	0.000243528
ASCL2	3	0.0000279
KREMEN1	1	0.0000295
TRAIP	2	0.0000287
CHEK2	3	0.000131249
ZNF703	1	0.000035
CCR4	3	0.000052
NUDT14	3	0.0000309
MAATS1	7	0.000281743
GPR39	7	0.000277358
UTY	12	0.000185105
SH2D1A	3	0.000149603
PRKX	3	0.0000712
KMT5A	1	0.000192038
KCNH8	11	0.0000639
NPW	7	0.0000538
NPB	7	0.0000401
ST6GALNAC3	0	0.0000824
ACTG1	0	0.000149051
VPS33B	2	0.000207844
TBX1	7	0.000271702
ADAP2	1	0.0000594
FAM120C	2	0.000304213
EIF3C	2	0.0000235
CLDN5	4	0.0000317
NR2C2AP	3	0.000246846
C1QTNF12	7	0.0000951
SCFD2	2	0.000326857
GPR173	7	0.000203193
TSPYL2	2	0.000201654
SNRNP35	0	0.000380669
IRAK1	1	0.00024415
CMSS1	2	0.000228073

PCDH9	11	0.0000285
OAF	1	0.0000353
H2AC21	3	0.0000575
POU6F1	2	0.000124796
PRKD1	7	0.000194842
CCSER1	11	0.0000404
ZDHHC23	3	0.000144739
MRGPRE	7	0.000143558
H1-5	14	0.000217287
PKP3	7	0.000221942
MAP7D2	19	0.000154847
CSF1	13	0.000042
ACTRT3	7	0.000247508
MAML2	2	0.000214557
WDR27	2	0.000198929
TXNRD2	4	0.0000605
FOXO4	4	0.000143892
PTP4A3	1	0.000025
PROS1	4	0.0000712
CEND1	7	0.000193527
C6orf58	7	0.00015221
SOCS3	0	0.0000242
STING1	1	0.000237846
SNN	0	0.000131455
NELL2	3	0.0000671
KRBA2	3	0.000211075
MED12	1	0.000170973
ZNF93	0	0.0000841
CDCA2	14	0.000196602
AMER1	2	0.000249632
ZBTB40	2	0.00033047
SERINC4	7	0.000141082
RNLS	3	0.00013953

APOBR	1	0.000130833
SMIM10	7	0.0000243
OSBP2	4	0.000113972
TRARG1	7	0.000254945
TMED9	3	0.00031437
TMEM186	3	0.000335072
SDR42E1	2	0.000158888
BTBD6	3	0.000130759
IMMP2L	2	0.000169725
TCEAL2	7	0.0000349
NUTM2A	7	0.0000349
LCN12	7	0.0000468
OR6A2	7	0.000217427
USP18	5	0.000162813
SORCS2	7	0.000275897
DGAT1	1	0.00015998
CA13	2	0.000118389
SPDYE16	7	0.0000891
CIB1	3	0.000155279
SLC24A3	7	0.0000512
EFCAB10	7	0.000138267
C5orf47	7	0.000140213
ADSS1	7	0.000206694
ANO9	2	0.0000832
H2BC13	14	0.000202864
MIXL1	7	0.000248351
MFSD6L	13	0.0000779
LRRC37B	2	0.000267644
DDX51	1	0.000171741
NOMO2	2	0.000118469
TNFAIP2	1	0.000175869
ZNF445	1	0.000219015
RAB11B	1	0.0000908

GP1BA	4	0.0000936
PPIL6	7	0.00029229
KIAA0825	0	0.0000933
UBALD2	1	0.0000426
KLHL33	7	0.000153689
RBM11	3	0.000027
SOCS1	5	0.0000538
PRKN	2	0.000114179
TEDC1	1	0.00010392
TNFAIP8L1	2	0.000132364
TARS3	3	0.000326302
SMYD3	2	0.000190858
METTL7A	3	0.000086
IFNLR1	11	0.0000325
FAM174B	0	0.0000283
STAC3	0	0.0000718
ROR1	11	0.000197675
MUC1	7	0.000178229
IRF7	5	0.000118138
PCGF3	2	0.000091
LMLN	2	0.00003
P4HB	0	0.00027795
NDUFA4L2	7	0.000280644
ZFP36L1	0	0.0000461
UBE2L3	3	0.000220965
PMEL	7	0.000261266
SYN3	7	0.000174594
SNAI3	1	0.0000817
ZBTB3	2	0.000163455
MYBL1	3	0.000078
IFIT1	5	0.000131994
CLDN24	39	0.000154847
KCNQ5	2	0.000037

ADAMTSL5	7	0.000109298
ARL17A	41	0.000154847
IFITM1	5	0.0000463
LAMP1	0	0.000101148
TAS2R60	42	0.000154847
C16orf54	1	0.000153006
SETD4	2	0.000357718
PTCH1	2	0.00010646
RTN4RL1	7	0.000289023
CALHM1	7	0.000261964
ZNF267	0	0.000142569
IRS2	1	0.0000449
C7orf61	1	0.0000247
BICD2	0	0.000333003
TMLHE	0	0.000328508
SLITRK2	7	0.000215216
RASA3	2	0.000230558
ZNF529	3	0.000296895
KRT73	36	0.000154847
AIDA	3	0.000115663
C15orf41	3	0.00025696
ZPBP2	7	0.0000813
KRT5	7	0.0000259
NBPF6	7	0.0000478
LRRC70	3	0.0000359
PIP5K1C	1	0.000165224
CYP4F2	7	0.0000294
POLR3C	2	0.000380915
WWOX	2	0.000232811
POLR1D	4	0.000143381
1-Mar	0	0.0000467
ZNF749	3	0.0000893
KLHL32	3	0.0000706

MRTFB	2	0.0002528
BTLA	3	0.000109769
TOR3A	3	0.000218928
NAP1L3	3	0.0000414
RXRA	1	0.000215709
NUDT17	2	0.0000497
KRT10	3	0.0000447
CD300E	1	0.000045
CCDC30	2	0.000119137
NKRF	3	0.000287326
FCAR	0	0.0000524
SPATA12	7	0.000240689
RPS23	3	0.0000719
GNG2	0	0.0001935
BTN3A2	2	0.0000357
AKAP14	7	0.0000626
ZNF396	0	0.0000314
ARHGAP30	0	0.000266549
SEPTIN10	43	0.000154847
FAM86B1	7	0.0000249
CYP4F3	0	0.0000689
FOXD2	7	0.000220713
SMIM29	0	0.00012142
SPATC1	7	0.0000847
UBE2H	4	0.000211833
HPDL	7	0.000035
CARMIL3	7	0.0000236
PRG2	7	0.000204244
PRR5	1	0.0000463
CCDC73	7	0.0000492
ZNF732	7	0.000182275
SPIN2B	3	0.000235292
TPCN1	1	0.000146532

LCE1A	7	0.0000319
TRABD2A	3	0.00011174
TMEM17	7	0.000152244
TMEM232	7	0.0000292
FAM183A	7	0.000147005
GPR141	0	0.0000495
TMPRSS6	7	0.000290324
TMEM216	0	0.00017112
C3orf70	7	0.000297126
OR2AK2	44	0.000144969
ENTPD5	4	0.0000785
MITF	2	0.0000312
LILRA5	0	0.0000236
SLIT1	7	0.000127438
VSTM2B	7	0.0000509
SHTN1	0	0.0000239
H1-7	7	0.000228917
MT1X	3	0.0000371
GCNT1	3	0.000102797
SESTD1	0	0.0000494
RSBN1L	0	0.000312145
EPOR	1	0.0000387
CHP1	1	0.000117939
H1-6	0	0.0000413
CDHR4	7	0.000271443
IFT140	2	0.000323031
TLR5	0	0.0000276
COX8C	7	0.000241804
PLEKHN1	7	0.000264191
ISG15	5	0.000189826
PERM1	7	0.000266948
EZHIP	7	0.0000236
SLC18A3	7	0.000126206

NHEJ1	2	0.000110365
FANCA	1	0.0000393
SEMA4D	1	0.000212774
ZNF70	2	0.000168492
CARD9	1	0.0000682
PEAR1	7	0.000140579
ZFP69B	2	0.000177919
SOWAHD	0	0.0000758
ZFP69	3	0.000280568
TMEM220	2	0.0000236
H1-2	3	0.0000651
ASCL4	7	0.000209652
CCDC157	1	0.000111867
FAM122A	3	0.000352757
SHISA7	7	0.0000868
LRRC74B	7	0.000302275
CLEC17A	11	0.000178656
OVCH1	7	0.000245314
CYHR1	1	0.000240614
CPSF4L	7	0.000246612
PLA2G2C	7	0.000027
TPRG1	3	0.0000536
UBQLN2	2	0.000159743
C19orf67	7	0.000184722
CLCN1	7	0.000226887
C11orf95	2	0.000118429
GPR89B	3	0.000296826
MAPK12	7	0.00027317
AGRN	5	0.000114064
NHS	0	0.0000523
ZNF626	3	0.0000563
HEPACAM2	4	0.000051
LAMTOR4	0	0.000134708

NCR3LG1	2	0.0000291
DCUN1D3	2	0.000250978
ZNF793	3	0.0000659
AGAP4	2	0.000235955
COMMD6	3	0.000051
HYKK	7	0.0000262
HES4	5	0.0000646
ZNF669	3	0.000183597
PEAK3	1	0.0000653
LRRIQ4	7	0.000228164
PLSCR1	5	0.000127028
C3orf62	1	0.000221936
ZNF559	3	0.000182075
SBK1	1	0.0000624
OR6N2	45	0.000154847
FOCAD	2	0.000168827
PRR19	7	0.000217474
JAKMIP3	7	0.000323927
SELL	0	0.000124108
IER5L	0	0.0000237
C19orf54	1	0.0000634
FAM83G	1	0.0000432
HBA2	46	0.000154847
CCDC9B	1	0.0000491
NBR1	0	0.000335251
OR2G6	7	0.0000871
FBLL1	7	0.0000242
NKAIN2	7	0.000250563
CLEC20A	7	0.000263456
CLN3	1	0.000201219
ASAH2	7	0.0000573
RTL6	2	0.000120933
DPYD	0	0.000258914

CC2D2B	0	0.0000236
RHCE	4	0.00005
IDO2	7	0.0000367
PARVB	1	0.0000604
SLC4A5	2	0.0000997
ZDHHC9	2	0.0000294
ZBED6CL	2	0.000146947
QRFP	7	0.0000851
TMEM120B	1	0.000181325
NOXA1	1	0.0000341
TMEM198	7	0.000305677
BCL2L15	28	0.000154847
ZNF548	3	0.00030699
BEND4	11	0.000178254
MSL1	0	0.000282821
AC099489.1	1	0.0000645
LRRK2	0	0.000153105
INSYN2A	7	0.000176698
NYX	7	0.0000531
NELFB	3	0.000202735
HUS1B	2	0.0000817
ADAT2	3	0.000287211
RNF222	7	0.000271056
RELN	7	0.000293981
LITAF	0	0.00011177
TMEM120A	0	0.000182882
FAM25G	7	0.0000352
NKAPL	0	0.0000236
FSCB	7	0.000189752
JPT1	0	0.000180708
ZAR1L	7	0.000204835
ZNF33A	0	0.000276181
PCDH18	7	0.0000446

ZNF600	3	0.0000432
TSPYL1	3	0.000274626
FHIT	2	0.0000363
RRP7A	3	0.0000282
SPANXN4	7	0.0000261
SLC35E2B	1	0.000134563
TOGARAM2	3	0.0000647
NEMP2	3	0.000213444
ALG1L	7	0.000044
GSPT2	3	0.000160922
C8orf76	2	0.000364758
ZFP92	1	0.0000289
NCR1	3	0.000116096
SYCP2	0	0.0000507
IL1RAP	0	0.0000897
PAX5	11	0.000126549
SPOCK3	7	0.000156526
TDRD7	0	0.000128245
CCDC189	0	0.0000375
HLA-DRB1	3	0.0000316
VN1R2	7	0.000114409
AKR1C3	3	0.0000555
SPATS2L	5	0.000180517
ZNF250	3	0.000364655
ZNF79	3	0.000360612
S100A4	0	0.000110564
AC040160.1	2	0.0000613
STK40	1	0.000232033
SEMA4A	1	0.000104774
SIRPB2	1	0.000116623
SRGAP3	7	0.000135774
SUPT5H	1	0.000257808
XPNPEP3	2	0.000242088

OR2T2	7	0.0000298
OR2C3	7	0.000215017
ZNF107	3	0.0000371
ZNF471	47	0.000183376
ATP2A1	7	0.000258216
MFSD14C	2	0.000298164
POM121	1	0.00029788
GIMAP5	3	0.000229349
STK31	7	0.000265303
NLGN3	1	0.0000654
ZKSCAN7	3	0.000111349
CD55	0	0.0000781
CPNE4	7	0.000197865
NTNG2	1	0.000045
SRGAP2B	0	0.000053
ZNF781	3	0.0000494
ZNF774	7	0.000190316
PTPN1	2	0.000394873
EVL	2	0.000258046
PRTN3	7	0.0000236
ZNF765	3	0.00010056
C20orf204	1	0.0000266
PPP1R26	13	0.0000406
NBPF4	7	0.00006
TSC22D2	3	0.000241699
CRYBA4	7	0.000239569
YRDC	3	0.000172409
ZNF775	3	0.0000502
RFX8	7	0.000129542
ZNF799	3	0.00029089
NCOR2	1	0.000206059
ARL9	7	0.000243827
TCEAL3	3	0.0000292

SLC6A9	4	0.0000409
BORCS6	0	0.000179471
MAN2A2	1	0.000223077
MME	0	0.00010062
CACNA1H	7	0.000301628
SULF2	0	0.000105869
PLXNB2	1	0.0000807
AJAP1	7	0.0000714
XRCC2	14	0.0000587
MYO6	3	0.000071
TCF4	11	0.000107649
ZKSCAN5	3	0.000402133
TECPR2	1	0.00015565
TLR7	0	0.0000283
ERI2	3	0.000189858
HSH2D	1	0.000114835
TRPV1	1	0.000142328
NF1	2	0.000282373
VKORC1L1	3	0.000342302
ZNF418	2	0.0000238
DAPK1	1	0.0000291
H2AC13	14	0.0000618
S100A2	7	0.000222432
TLE1	2	0.0000289
MAML3	1	0.000192347
H2AC11	3	0.0000682
ZNF239	3	0.000105274
MVB12B	2	0.0000382
POTEI	7	0.0000992
ADA	3	0.0000606
ARID5A	1	0.00015244
ZFP28	3	0.0000709
CBWD3	0	0.00010518

LAMB3	7	0.0000837
H2BU1	4	0.0000368
ANKRD36B	3	0.0000434
ARHGEF12	4	0.00018175
HCAR1	7	0.000195386
PDLIM7	1	0.000142479
FAM3C	3	0.000125404
CASP4	0	0.00019883
AP2A1	4	0.000151886
FUT11	3	0.000296861
LAGE3	3	0.00016776
WDR45	4	0.000221435
ZNF470	3	0.000113236
GMFB	3	0.00014165
ZNF763	0	0.0000442
DTHD1	3	0.000024
H4C3	3	0.0000582
ARRDC1	1	0.000179415
KIAA1671	7	0.0000621
IGF2R	1	0.00016201
LCE1C	7	0.0000928
GAL3ST4	1	0.0000282
PCBP2	2	0.000189452
ZGPAT	3	0.000164097
PGAP1	3	0.0000462
SRC	1	0.0000663
ZNF772	3	0.0000604
LRRC8B	3	0.000121861
H3C12	14	0.000137459
ZNF785	1	0.000174083
CYSRT1	7	0.0000242
SLC22A4	0	0.0000882
H4C11	3	0.0000549

SERPINA1	1	0.000159172
KANK2	4	0.0000469
RAD54B	3	0.0000547
FITM2	2	0.000221936
BLM	3	0.00016483
LRP10	1	0.000176501
PELI1	0	0.000214992
MRPL21	3	0.000150005
ZNF98	7	0.000238005
FBXL22	7	0.0000286
ZNF786	3	0.000325308
ADARB1	2	0.000160993
ZNF860	11	0.000111361
OR6N1	45	0.000154847
C5AR1	1	0.00015699
VEPH1	4	0.000059
MAP3K5	0	0.000294967
OR2L5	44	0.000190257
STMN3	3	0.000129018
PDGFA	4	0.00018404
SPN	1	0.0000442
ZNF628	1	0.000118712
SLC2A10	7	0.000273533
ZNF665	3	0.0000243
RPF2	3	0.000181511
SLC28A3	10	0.0000615
MYO5A	0	0.000314794
GZMM	3	0.0000471
ATG7	2	0.000222206
ELANE	10	0.000117454
RAB40C	1	0.000172837
HOXA4	7	0.000215283
TOPORS	0	0.000125137

BCO2	2	0.0000322
ENPP1	7	0.000278133
CDC42SE1	0	0.00034114
MPEG1	1	0.0000711
DPP4	3	0.000104708
PDCD1LG2	5	0.0000766
DNAH10	7	0.00036681
KLHL14	11	0.0000875
CR1L	4	0.000136893
PSAP	1	0.000188034
S100A10	3	0.0000833
RPL37A	3	0.0000702
TXNRD3	3	0.000112944
MAP1LC3C	7	0.000237183
KLHDC1	3	0.000210517
TAF13	3	0.0000937
CCDC180	2	0.0000295
H4-16	3	0.000149642
INKA2	0	0.0000788
PRB3	7	0.000139123
FAM49A	0	0.000272761
H2BC12	3	0.0000751
ZNF677	3	0.0000239
ERO1A	0	0.00014867
ZNF823	3	0.000137421
PLCG2	1	0.000255443
FCHSD1	1	0.000252574
RPL12	3	0.000102125
DNM3	4	0.000150613
MPZL1	0	0.0000306
GOLGA6L9	3	0.0000328
KEL	4	0.000115391
FCGR1B	5	0.000140785

RPS4X	3	0.0000514
ZNF667	47	0.000126317
SIRPA	1	0.000194089
GRK6	1	0.000280216
SULT1C4	7	0.000195249
ZNF248	3	0.000293913
OR2L3	44	0.000103781
ZNF544	3	0.000265618
SOWAHC	43	0.000154847
ZNF251	2	0.000363293
TFDP1	4	0.000103354
ZNF334	7	0.000121571
HSD17B11	0	0.000226313
RPS6KL1	7	0.000107683
UBL5	3	0.00010928
TMEM116	3	0.000258665
PHETA1	1	0.0000496
MYL4	4	0.0000299
HOXC4	7	0.0000924
PIM3	0	0.0000358
GET3	0	0.000124079
ASPH	0	0.0000469
NTRK1	7	0.000184303
MT1F	3	0.0000336
ZNF69	3	0.000140642
TXNRD1	0	0.000244491
ZNF583	3	0.000123687
ZXDB	2	0.000234008
ZNF587	2	0.000344545
SH3BGRL2	4	0.000265131
ANKRD35	7	0.000181091
C20orf203	7	0.000247172
ZNF789	2	0.000356448

CTNND1	2	0.000146792
SH2D1B	3	0.000113034
NUDT16	0	0.000204758
LRBA	3	0.000306096
MMP17	1	0.000041
BAZ1A	0	0.000184108
CCDC69	1	0.00028594
STK39	2	0.000216562
C6orf89	0	0.000394519
EIF1AY	12	0.000110895
MT-ND6	7	0.0000271
IPO9	2	0.000354288
MT-CO2	48	0.00023372
GLMP	1	0.0000436
MT-CYB	48	0.000184975
PPP1R14C	7	0.000109511
F5	0	0.0000532
MSRB1	0	0.000158468
CDC42BPB	1	0.0000868
MT-ND2	48	0.000209008
RCSD1	0	0.000314879
FAM169A	3	0.000164576
GRIN3A	7	0.000024
MT-ND5	48	0.000141838
TMEM184B	1	0.000194706
SCAMP5	7	0.0000998
LRIG2	2	0.000299507
MT-CO1	48	0.000172001
PNP	4	0.0000735
GK	0	0.0000692
ZNF358	7	0.0000459
CD247	3	0.000119371
CHAMP1	3	0.000105063

ARHGAP11A	14	0.000116129
HMGN2	3	0.000275296
SELENOM	3	0.000033
UBE2J1	0	0.0000698
DENND4B	1	0.000254999
MT-ND3	48	0.0000452
CD3E	2	0.0000861
RUSC2	1	0.0000444
R3HDM4	4	0.000115761
GRK5	0	0.000229117
DCAF12	4	0.000234923
ASB12	7	0.0000323
ITPRIPL1	3	0.00014181
MT-ND4	48	0.000184975
MT-ND1	48	0.000141838
CIPC	0	0.000321067
CAPZA2	0	0.000116728
MT-ATP6	48	0.000209008
RASGEF1A	2	0.0000239
RPL39	3	0.0000426
DZIP3	3	0.000134485
ATG9A	1	0.000227601
APRT	3	0.000122395
GPRASP1	3	0.000164768
TBKBP1	1	0.0000365
MAGEE1	2	0.000122573
CCDC167	3	0.0000541
MT-CO3	48	0.000066
ZFP2	3	0.0000656
L3MBTL3	3	0.000279246
SMG5	1	0.000166079
TGM2	4	0.0000932
OR2L2	44	0.000190257

CR1	0	0.0000979
GPR52	2	0.0000637
CENPW	14	0.00019109
PRR9	7	0.000142248
H3C14	14	0.000167059
H2BC18	0	0.0000696
H3C15	14	0.00016754
C6orf163	2	0.000150954
GDI1	1	0.000284707
LIME1	3	0.0000912
OOEP	7	0.000152907
SAMD13	7	0.000250638
IFIT1B	4	0.0000705
LIPJ	7	0.0000268
TRPC5OS	7	0.000124611
TCEAL6	7	0.000152754
INPP5B	2	0.000243291
RPA4	3	0.000095
MAFB	1	0.0000283
NHSL2	1	0.000152521
PHACTR4	2	0.000147318
ASAH2B	3	0.0000647
AGAP6	2	0.000222056
TIMM23B	2	0.000319566
ZDHHC18	1	0.00021984
SYT15	7	0.000219009
TCEA3	2	0.000052
HSD17B8	3	0.0000541
OR13C3	7	0.0000371
HLA-DOA	11	0.00010623
PSMB8	0	0.000215555
TAP2	0	0.0000737
SPIN3	3	0.000247144

NBDY	3	0.000114387
HLA-DRA	3	0.0000649
NOTCH4	7	0.000221818
PBX2	1	0.000135404
RNF5	3	0.0000797
PJVK	3	0.000145175
FKBPL	0	0.0000485
MRPL38	1	0.0000907
SMIM5	4	0.000136687
BTBD17	7	0.000196568
C9orf129	7	0.000121515
LAYN	7	0.000128798
NEU1	0	0.000298001
HSPA1A	0	0.0000495
LSM2	2	0.0000292
CARD16	0	0.0000454
MBD5	2	0.000232832
MSH5	1	0.0000471
MPIG6B	4	0.0000772
LY6G6C	7	0.0000679
LY6G6F	4	0.000154223
ABHD16A	0	0.000221079
CSNK2B	1	0.000214774
GPANK1	0	0.000366281
PRRC2A	1	0.00015749
AIF1	3	0.0000508
LST1	0	0.000138641
NFKBIL1	1	0.0000715
ZNF814	2	0.000118168
HLA-C	0	0.000029
POU5F1	7	0.0000773
C6orf136	2	0.000153894
KRTAP5-11	7	0.000141548

ABCF1	2	0.000181719
LILRB3	1	0.000160614
HLA-E	0	0.00022408
TRIM39	0	0.000373112
TRIM10	4	0.000199505
TBC1D8	1	0.0000579
HLA-F	0	0.0000794
AKT1S1	1	0.000142642
GABBR1	1	0.000107665
C10orf113	18	0.000154847
MALRD1	7	0.00026842
RANBP17	30	0.000154847
INSYN2B	7	0.000127219
MRPL53	3	0.000333581
MROH6	1	0.0000246
SPATA31A1	7	0.000179705
TCTN1	2	0.0000289
KRT40	7	0.00025488
GRXCR2	7	0.000262389
ZNF783	2	0.00034477
ZNF425	3	0.0000241
FBXO47	7	0.00023665
C12orf73	3	0.000229976
PCDHGA1	7	0.0000236
ERICH4	7	0.000168353
PRSS1	7	0.000143493
SPIRE2	7	0.000225144
AARD	7	0.000234253
PABPN1L	7	0.0000272
PKHD1L1	4	0.000156929
SYCE1L	2	0.0000242
TMEM231	7	0.0000328
CCNI2	2	0.0000468

TMEM240	7	0.0000803
ACCSL	7	0.0000432
C4orf47	7	0.000242914
TRIQK	0	0.000130972
SDHAF1	3	0.000140174
PSENEN	0	0.0000305
SCGB2B2	7	0.0000248
CCDC144NL	7	0.0000389
E2F4	1	0.000190308
PDE7A	3	0.000299733
ADGRG1	3	0.000160865
PRR13	1	0.0000796
TECPR1	1	0.000201244
SAMD9	3	0.0000343
CNEP1R1	0	0.000240864
KRT81	7	0.000231027
CCDC85C	7	0.000195131
RGL3	7	0.000237427
TMSB4X	3	0.000114702
CPT1B	1	0.0000312
MFSD2B	4	0.000280211
CDPF1	2	0.000192725
DPF3	7	0.0000273
CYP2D7	1	0.0000402
C17orf107	25	0.000129026
ITPRIPL2	0	0.000103158
ARRDC5	2	0.0000405
PLPP6	3	0.000231846
KLRC3	49	0.000154847
ZNF316	1	0.000146694
OLIG2	13	0.0000977
DOK6	7	0.0000236
RNASE13	7	0.000179494

ATP10A	2	0.000105118
TSSK2	7	0.0000612
CFAP44	2	0.000221338
LNP1	3	0.0000695
TRIM71	7	0.0003008
ZCWPW2	3	0.0000241
ANKRD28	3	0.000145318
METTL6	3	0.000402439
SELENOH	3	0.000154823
AKR7L	7	0.000277708
STK38L	0	0.000216437
SLC48A1	4	0.0000413
TAS2R50	3	0.0000563
TAS2R13	2	0.0000572
KRTAP16-1	7	0.0000287
OR2A42	7	0.000177741
MT-ND4L	48	0.000184975
MAP10	3	0.0000417
KRTAP10-3	7	0.0000534
POU5F1B	3	0.0000494
ZNF611	0	0.000201694
NUP62	1	0.000109452
SFT2D2	2	0.000219216
FGFR1OP	2	0.00014505
CRYGS	3	0.00029734
LINGO4	7	0.0000502
FAM24B	3	0.000117563
ASIC3	7	0.000194974
SUPT4H1	0	0.0000998
LTC4S	1	0.0000835
QTRT1	2	0.00017582
MXD3	0	0.0000651
ARHGAP19	0	0.000198885

HAUS7	2	0.0000804
LCAT	1	0.0000908
GPC2	2	0.0000279
SIPA1	1	0.000209016
ERV3-1	0	0.0000797
ARL2	3	0.000187807
TTLL13P	7	0.0000239
LEPROT	2	0.000167351
LBH	3	0.0000986
PPP1CB	3	0.000110141
GPSM3	1	0.00010592
LAT	1	0.0000734
NCKIPSD	1	0.000196185
S1PR3	2	0.0000254
SLC35F6	1	0.000215852
CLIC1	0	0.000188032
DDAH2	0	0.000138355
RPS29	3	0.0000482
UGT2B11	7	0.0000768
ZNF134	3	0.000359755
DDX47	2	0.000286534
ZNF888	3	0.000139549
ZNF845	3	0.0000901
KLRK1	3	0.0000734
KCTD11	3	0.0000602
SLC23A3	1	0.0000237
LTB4R	0	0.000103488
IRF9	1	0.0000581
CLDN9	1	0.0000256
NUDT19	3	0.000151615
ZNF726	3	0.0000999
ZNF90	0	0.0000242
NAXD	3	0.000338263

MEF2B	1	0.0000497
GANC	2	0.00033342
FBXO16	7	0.000262753
ARL16	3	0.0000581
LYRM4	3	0.000145165
MYCBP	0	0.000262036
ALG3	2	0.00025606
C19orf38	0	0.0000975
C17orf67	2	0.000174444
FIS1	4	0.000151273
MBLAC1	3	0.0000431
EFCAB9	7	0.0000427
LCNL1	7	0.0000381
PLIN5	1	0.0000655
C10orf105	1	0.0000767
IFRD2	4	0.000113292
HNRNPUL2	2	0.000360387
MS4A4E	0	0.0000307
MTCP1	3	0.000213047
ARHGEF28	7	0.0000678
LRRC69	2	0.000236657
UBXN2B	0	0.000218765
CBWD6	0	0.0000675
PRSS41	13	0.000045
NLRP2B	7	0.000180046
MSMP	7	0.000146008
FAM166B	7	0.000161136
FASTKD5	3	0.000392444
GOLGA8B	2	0.0000672
SCRT2	7	0.000241673
SIAH3	7	0.000291398
EFCAB8	7	0.000076
GAB4	7	0.000173643

C20orf202	7	0.0000299
TNFRSF25	2	0.0000746
SLC35E2A	1	0.0000317
CYB5RL	2	0.000125978
TTC34	1	0.0000481
IFI30	0	0.0000358
CCDC7	3	0.0000881
FNIP1	0	0.000217951
SYCE3	7	0.0000614
CKLF	0	0.000203732
PAM16	1	0.000182331
ZNF579	7	0.0000706
RNASEK	0	0.00003
NBPF1	50	0.000154847
FAM228B	3	0.000242952
C6orf226	3	0.0000251
PPP3R1	4	0.0000673
AP4M1	1	0.0000608
TAS2R41	42	0.000154847
CEBPD	0	0.0000377
OR3A2	40	0.000154847
ARIH2OS	3	0.000110141
ZBED8	3	0.0000932
HMSD	7	0.000267555
NPTXR	7	0.00005
TRIM16	2	0.000195404
FADS3	1	0.0000394
UBA52	4	0.0000741
MYBPHL	7	0.000166697
ZNF630	2	0.0000469
TIAF1	2	0.0000912
FAM185A	3	0.000313878
PSMB11	7	0.000213614

EXOSC6	3	0.000270721
ZNF844	0	0.0000522
HBD	46	0.000154847
HLA-DPB1	3	0.0000626
PRR29	7	0.000212954
HNRNPA1P48	3	0.0000255
NDUFAF8	3	0.0000901
PRRT4	7	0.000228874
Z82206.1	2	0.0000514
C2CD4D	7	0.000201664
NOL7	3	0.00037786
PIGBOS1	0	0.000237524
FTCDNL1	2	0.0000718
TEX22	1	0.0000313
TAS2R46	3	0.0000624
ZNF717	3	0.000200046
SCAMP4	1	0.000117566
LTB	3	0.0000816
TEX46	7	0.0000502
POLR2J2	35	0.000154847
MT-ATP8	48	0.0000847
FAM174C	3	0.0000872
C2orf92	2	0.0000268
FNDC10	7	0.0000342
PROB1	7	0.000297256
ARL17B	41	0.000154847
PATL2	3	0.0000302
PRPS1L1	7	0.000170392
NFE4	34	0.000154847
PPP5D1	7	0.0000263
CFAP97D1	7	0.000251367
HLA-DPA1	3	0.0000379
RPS18	3	0.0000712

TMEM253	7	0.00011093
OR52I1	7	0.000171158
AJM1	1	0.0000263
GOLGA8N	3	0.0000244
RAB6D	7	0.00021491
GPX1	4	0.0000451
TWIST2	7	0.0000795
H2BC15	3	0.0000469
ZNF736	3	0.000252218
PINLYP	7	0.0000527
CLDN34	7	0.000024
HLA-B	0	0.0000854
SHISA8	7	0.000113306
C19orf81	7	0.000275514
BRD3OS	2	0.000150466
NFAM1	1	0.000126803
KIAA0040	0	0.000175377
CLEC2L	7	0.000146661
SLFN14	4	0.0000424
UBE2L5	7	0.000112859
CTAGE9	7	0.00021963
C3orf84	7	0.000140455
B3GNT9	1	0.0000515
ZNF737	3	0.000106027
RGL2	1	0.000232817
KIFC1	14	0.000111762
C2orf74	3	0.0000865
FAM200B	0	0.000174902
OR2I1P	8	0.00010787
LRRC37A2	2	0.0000492
MPC1L	7	0.000026
OR2W3	4	0.0000935
TXNDC5	15	0.000108549

CASTOR1	3	0.0000657
ALKBH6	2	0.0000881
CASTOR3	1	0.000186422
NME1	3	0.0000646
TNFSF12	1	0.0000255
APOBEC3G	3	0.0000836
TLR9	1	0.0000263
DEFA3	10	0.000132304
LILRA2	1	0.000185611
AMY2B	3	0.000292793
PSMB9	0	0.0000927
PCDHGC3	51	0.000137184
DEFA1B	10	0.0000774
ACAD11	3	0.000242056
FOXO3B	4	0.000034
TNFRSF13B	11	0.0000667
ISY1	0	0.000377315
PNMA2	11	0.0000586
LRRD1	7	0.000248421
KRBOX1	7	0.000149924
PCDHGC5	51	0.0000611
ARHGEF25	7	0.000274005
YAE1	3	0.000226256
RPP21	2	0.000025
CD302	0	0.00012193
ARHGAP8	7	0.000201465
AC244197.3	1	0.00011784
ARPC4	0	0.000243967
INMT	7	0.000209259
ARPC1A	0	0.000333751
ATP5PO	3	0.000176705
C8orf58	1	0.00011411
PISD	1	0.00017683

PWP2	33	0.000154847
HYPK	2	0.000288487
MTFP1	3	0.0000429
TCP10L	7	0.0000582
ARFGAP3	0	0.000364882
PEG10	27	0.000154847
PCDHGC4	51	0.0000829
HLA-DMB	3	0.000137654
AP5Z1	1	0.000252657
STRC	7	0.000279164
MICAL3	2	0.000103915
PRAF2	1	0.000109829
STMP1	4	0.0000961
EFNA4	0	0.000137028
TICAM2	0	0.0000434
C4orf48	3	0.0000349
NAA80	1	0.0000583
UPK3B	1	0.0000747
IL10RB	1	0.000256329
ZNF487	0	0.000138434
WDR92	3	0.000309296
PLA2G4B	1	0.0000389
NPIPB5	1	0.0000267
JMJD7	1	0.000104505
APOBEC3D	2	0.0000447
TUBA4B	7	0.000130704
MRPS6	2	0.000209295
DBNDD2	2	0.0000494
ETV5	7	0.000301713
KRTAP5-7	7	0.000195895
SCARF2	7	0.0000976
ASPRV1	1	0.0000398
HBB	46	0.000154847

CEBPA	0	0.0000467
H2AJ	4	0.0000901
UBAP1L	2	0.000152157
MARS2	3	0.000304111
USP51	3	0.0000248
BCKDHA	2	0.0000397
CDK11B	0	0.000238336
TARM1	10	0.0000605
POU5F2	2	0.000141654
ABHD14A	3	0.0000298
STIMATE-MUSTN1	1	0.000041
RBM14-RBM4	2	0.000275819
CCDC153	0	0.0000246
ACTN3	7	0.000288751
HAUS5	2	0.000147949
TAF11L12	7	0.0000991
AP000311.1	3	0.000024
ZNF324B	2	0.000222734
LINC02218	0	0.0000505
ZNF564	1	0.000134062
HS3ST5	7	0.000266737
TERB1	7	0.000137933
YJEFN3	1	0.0000386
ARPC4-TTLL3	2	0.0000235
PTTG2	0	0.0000703
SMIM20	3	0.000143941
GYPB	4	0.0000278
CHCHD10	1	0.0000238
ATP6V1E2	3	0.000117046
AC068580.4	1	0.000026
AC010255.3	7	0.000272845
TMED7-TICAM2	3	0.0000434
FOXD1	7	0.000174629

AC005324.3	7	0.000166015
PTX4	7	0.0000296
PCDHGA12	51	0.000202062
C8orf88	0	0.0000248
CCDC71L	0	0.0000619
HOXA10	7	0.000194363
PCDHGB6	51	0.000178943
PCDHGA5	51	0.000148823
PCDHGA7	51	0.000165842
EIF5AL1	7	0.0000284
PRKDC	2	0.000255315
PCDHGA6	51	0.000148823
PCDHGA8	51	0.000165842
PCDHGA10	51	0.000130565
PCDHGA11	51	0.000130565
PCDHGB2	51	0.000148823
PCDHGB4	51	0.000165842
LYN	0	0.000224497
PCDHGB7	51	0.000130565
PCDHGA3	7	0.0000236
AP002495.1	3	0.000287832
AP5B1	1	0.000146634
ARMS2	7	0.000116335
MEX3A	7	0.000253877
CKLF-CMTM1	1	0.000101495
MPV17L2	3	0.00005
BORCS8	1	0.000175665
ANKHD1-EIF4EBP3	2	0.000179998
CHMP1B	0	0.000237529
HTD2	2	0.0000248
CARD17	5	0.0000573
OOSP4B	7	0.000206218
HCAR3	0	0.0000751

EBLN2	0	0.00025791
FDXACB1	3	0.000116011
RAB44	13	0.000163033
AC068775.1	49	0.000154847
MTRNR2L8	52	0.000154847
TAS2R20	3	0.000066
TRAPPC2B	3	0.0000253
TAS2R30	3	0.000142199
MTRNR2L3	7	0.000046
ZNF10	3	0.000230461
ZNF486	0	0.0000873
SMIM3	0	0.0000771
HMBS	4	0.0000261
DND1	1	0.0000325
CLEC12B	37	0.000154847
PGA5	7	0.000163958
STH	7	0.0000306
KLRF2	7	0.000163863
CAPNS2	0	0.0000451
GPR142	7	0.000186516
C11orf97	7	0.000247257
DENND11	2	0.000315651
NHLRC4	3	0.0000385
MGAM	1	0.0000815
FNTB	2	0.000149685
AC023055.1	3	0.000108311
INAFM1	0	0.0000509
MGAM2	1	0.0000254
C17orf49	2	0.0000246
PDF	3	0.0000924
AC005726.1	1	0.0000352
TRIM34	0	0.0000313
MC1R	2	0.000103407

AC007040.2	7	0.000303723
CEP95	2	0.000336816
TUBB3	7	0.00023284
ITGB3	4	0.000177003
ZHX1-C8orf76	2	0.0000268
INAFM2	0	0.000101119
ST20-MTHFS	0	0.0000258
GH1	7	0.00005
THTPA	2	0.000201817
SLC22A31	7	0.000249466
AC107871.1	2	0.0000482
CCDC187	7	0.000310457
ARMH2	0	0.000024
C16orf95	7	0.000204918
AL049634.2	0	0.000110954
XKR7	7	0.00031442
CCPG1	0	0.000221469
SULT1A3	1	0.0000271
GOLGA8T	7	0.000111503
FIGNL2	7	0.0000597
AC010325.1	7	0.0000954
PECAM1	1	0.000257834
TUBB8	7	0.00016606
TMEM249	7	0.000101796
PCDHGA9	51	0.000242885
OCLM	2	0.0000757
PCDHGB3	51	0.000148823
CORO7	1	0.000233061
CCER2	7	0.000167954
C19orf84	7	0.00025401
GTF2I	2	0.000265577
MYZAP	29	0.000154847
PPIAL4C	2	0.0000235

FAM72C	14	0.0000296
LINC00672	2	0.0000412
NBPF11	2	0.000249427
RHEX	23	0.0000908
NOTCH2NLA	2	0.0000362
OTUD7B	3	0.000217769
AC134669.1	0	0.0000245
FSBP	3	0.0000245
TXNIP	0	0.000196671
NBPF15	2	0.000152039
NCOA4	4	0.0000848
MRPS21	3	0.000190427
MYO15B	1	0.000116508
AC008770.2	2	0.0000238
C17orf113	7	0.0000298
ATF7-NPFF	2	0.0000499
ZNF285	47	0.000126317
S1PR2	1	0.000028
NDUFA7	1	0.0000342
NBPF12	2	0.000204299
SMIM17	7	0.000197171
FAM156A	2	0.000104878
AC008878.3	2	0.0000323
MTRNR2L12	52	0.000154847
IKBKG	1	0.000251948
SPIB	11	0.000153424
TMEM185A	2	0.000347955
NBPF9	2	0.000115501
CCDC194	5	0.0000396
EGLN2	2	0.000225979
MEI4	7	0.000138405
ZNF559-ZNF177	2	0.0000437
NCBP2AS2	3	0.000209894

H4C15	14	0.0000367
HEATR9	3	0.0000894
NBPF14	1	0.000082
GAS2L2	7	0.000292792
C17orf50	7	0.000229062
H4C14	14	0.0000481
RASL10B	7	0.000313865
TLCD4-RWDD3	3	0.0000985
AC240274.1	50	0.000154847
NBPF19	2	0.000103987
NBPF10	0	0.0000661
CCL5	3	0.0000552
GTF2H5	3	0.000112088
IFNL4	7	0.000242788
FAM47E-STBD1	7	0.0000239
DOC2B	7	0.000279619
ATP6V1FNB	7	0.0000653
AL121845.3	3	0.0000247
SNURF	2	0.0000675
AC092042.3	7	0.0000278
FOXD4L6	7	0.0000259
H4C12	3	0.0000271
CWC25	0	0.000321197
EPOP	7	0.000278379
H2BC14	14	0.000198896
CYFIP1	0	0.0000814
H2BC8	14	0.000212576
USP27X	3	0.000217194
H3C8	14	0.000089
CASTOR2	1	0.0000378
NATD1	1	0.000073
H2BC6	14	0.000188755
C8orf89	7	0.0000242

H2BC17	14	0.000171105
KRTAP7-1	7	0.0000867
PANO1	7	0.000071
H2AC12	14	0.00010591
ZNF280B	3	0.0000256
NUDT18	3	0.000116168
H4C13	14	0.0000794
LENG9	3	0.0000747
H2AC15	3	0.0000578
CCL4	3	0.000093
PRAG1	1	0.00006
C7orf77	7	0.0000884
H3C11	3	0.000052
CCL18	7	0.000190259
FP565260.1	53	0.000154847
C17orf98	7	0.0000671
H4C7	7	0.0000248
H2BC9	14	0.000225869
PRSS2	7	0.000190644
DUSP14	3	0.0000243
UHRF1	14	0.000091
H4C9	3	0.0000528
ANKRD20A3	7	0.000145218
PIK3R6	13	0.000219085
OR4E1	7	0.000119713
PIP4K2B	3	0.000354577
H2AC14	14	0.0000603
H2BC3	14	0.000202275
PCDHGB5	51	0.000242885
PADI6	7	0.000110143
H2AC16	14	0.0000572
H4C5	3	0.0000749
H2AC8	3	0.000132966

TYW1B	2	0.000212325
H4C4	14	0.000188313
PIGW	3	0.000265867
H2BC7	14	0.0000687
MARCKS	0	0.0000357
ZNF670	3	0.0000959
H3C7	14	0.000116139
PSMB3	0	0.000166366
GOLGA6L22	7	0.0000531
RDM1	7	0.0000314
TEX28	7	0.0000304
SSTR3	3	0.000025
ZNF229	47	0.000183376
H2AC4	14	0.0000768
ACACA	2	0.000253574
H2BC10	14	0.000232345
MRM1	2	0.000148276
H4C1	14	0.000162685
GOLGA6L10	7	0.0000237
H4C2	3	0.0000584
H3C10	4	0.0000432
MRPL45	3	0.000205738
TSTD3	7	0.000244974
OR2L8	44	0.000144969
OR52R1	7	0.000142621
GATD3B	53	0.000154847
FP565260.6	3	0.0000833
PAGR1	1	0.0000707
TMEM272	0	0.0000249
BLACAT1	7	0.000157823
ADORA3	13	0.000149962
C13orf46	1	0.0000779
AC001226.2	7	0.000223809

AC118553.2	2	0.000215728
AC116366.3	3	0.0000296
PRR33	7	0.000197704
TPTEP2-CSNK1E	1	0.000024
AC004922.1	0	0.0000761
AL590132.1	7	0.0000771
TAF11L2	1	0.000024
THSD8	7	0.000148882
AC092718.8	7	0.0000982
AC015802.6	1	0.0000913
AC073111.4	2	0.000135322
TMDD1	7	0.0000716
AC114490.3	2	0.0000845
SMIM41	7	0.000152885
ARHGAP11B	3	0.0000238
BX255925.3	1	0.0000852
AC093512.2	7	0.0000283
TBCE	3	0.000159865
ATXN7	2	0.000141004
POLR2J3	35	0.000154847
AP000646.1	4	0.0000357
AC097634.4	0	0.0000236
AP001267.5	2	0.0000306
AC008676.3	7	0.000149687
AC072022.2	0	0.0000271
AL135905.2	0	0.0000373

Supplementary Table 9. Functional enrichment analysis of network community maps

Enriched Pathway	P (adj)	(-log10) P	Term size	Intersection size	Enrichment	Community
INNATE IMMUNE SYSTEM (REACTOME)	1.10E-36	35.96	1104	192	0.1739	0
NEUTROPHIL DEGRANULATION (REACTOME)	2.41E-34	33.62	478	116	0.2427	0
RRNA PROCESSING (REACTOME)	1.12E-18	17.95	205	56	0.2732	3
RRNA PROCESSING IN THE NUCLEUS AND CYTOSOL (REACTOME)	1.76E-18	17.75	193	54	0.2798	3
INTERFERON SIGNALING (REACTOME)	7.88E-34	33.10	202	30	0.1485	5
INTERFERON ALPHA BETA SIGNALING (REACTOME)	7.40E-20	19.13	70	16	0.2286	5
CYTOKINE SIGNALING IN IMMUNE SYSTEM (REACTOME)	2.83E-17	16.55	856	32	0.0374	5
NEUTROPHIL DEGRANULATION (REACTOME)	6.97E-23	22.16	478	21	0.0439	10
INNATE IMMUNE SYSTEM (REACTOME)	6.78E-17	16.17	1104	22	0.0199	10
CELL CYCLE (REACTOME)	9.92E-29	28.00	642	37	0.0576	14
CELL CYCLE MITOTIC (REACTOME)	1.21E-28	27.92	536	35	0.0653	14
CELL CYCLE CHECKPOINTS (REACTOME)	4.20E-19	18.38	291	23	0.0790	14
MITOTIC PROMETAPHASE (REACTOME)	2.24E-18	17.65	198	20	0.1010	14
PARKINSONS DISEASE (KEGG)	5.03E-22	21.30	128	12	0.0938	48
OXIDATIVE PHOSPHORYLATION (KEGG)	6.72E-22	21.17	131	12	0.0916	48
RESPIRATORY ELECTRON TRANSPORT ATP SYNTHESIS*** (REACTOME)	3.46E-19	18.46	122	11	0.0902	48
THE CITRIC ACID TCA CYCLE AND RESPIRATORY ELECTRON TRANSPORT (REACTOME)	1.85E-17	16.73	173	11	0.0636	48

P (adj), P-value adjusted; (-log10) P, p-value on a -log10 scale; Enrichment represents ratio between intersection size and term size; *** by chemiosmotic coupling and heat production by uncoupling proteins

Supplementary Table 10. Cell types significantly associated with PD etiology based on expression specificity enrichment analyses

	DISCOVERY			REPLICATION		
**Cell type**	**Beta**	**SE**	**P**	**Beta**	**SE**	**P**
VASCULAR LEPTOMENINGEAL CELLS	5.10E-06	1.78E-06	0.0244	2.64E-06	3.18E-06	0.434
STRIATAL INTERNEURON DECIL	2.57E-06	1.85E-06	0.2069	4.86E-06	2.67E-06	0.111
RADIAL GLIA LIKE CELLS	6.50E-06	1.14E-06	0.0008	6.08E-06	2.83E-06	0.069
PYRAMIDAL SS LIKE CELLS	4.04E-06	7.34E-07	0.0009	1.70E-06	2.59E-06	0.533
PYRAMIDAL CA1	2.40E-06	7.80E-07	0.0179	2.26E-06	1.86E-06	0.265
OXYTOCIN AND VASOPRESSIN EXPRESSING NEURONS	4.06E-06	1.22E-06	0.0126	3.79E-06	1.81E-06	0.075
OLIGODENDROCYTES	5.62E-06	8.08E-07	0.0002	-1.06E-07	1.39E-06	0.941
OLIGODENDROCYTE PRECURSOR	3.88E-06	1.45E-06	0.0319	1.00E-06	2.06E-06	0.642
INTERNEURONS	1.62E-06	1.39E-06	0.2833	-4.18E-07	2.44E-06	0.869
HYPOTHALAMIC GLUTAMATERGIC NEURONS	4.33E-06	1.30E-06	0.0127	2.80E-06	2.61E-06	0.320
HYPOTHALAMIC GABAERGIC NEURONS	6.36E-06	2.13E-06	**0.0204**	7.51E-06	2.21E-06	**0.012**
HYPOTHALAMIC DOPAMINERGIC NEURONS	3.53E-06	2.61E-06	0.2195	-3.33E-06	6.64E-06	0.632
ENDOTHELIAL MURAL	5.70E-06	1.10E-06	0.0012	1.15E-06	2.55E-06	0.665
AE	7.42E-06	1.54E-06	0.0019	2.55E-06	3.39E-06	0.477
MSN	2.72E-06	7.76E-07	0.0099	-4.23E-07	1.40E-06	0.772
MICROGLIA	4.81E-06	1.96E-06	0.0440	8.18E-06	8.20E-06	0.352
NEUROBLASTS CELLS	5.08E-06	1.25E-06	0.0048	4.33E-06	3.44E-06	0.249
NEURAL PROGENITOR	4.86E-06	9.63E-07	**0.0015**	8.15E-06	2.00E-06	**0.005**
DOPAMINERGIC ADULT	5.32E-06	1.18E+00	0.0028	5.40E-06	2.61E-06	0.078
DOPAMINERGIC NEUROBLAST	2.25E-06	1.27E-06	0.1199	4.42E-06	2.17E-06	0.082
DOPAMINERGIC EMBRYONIC	5.20E-06	1.27E-06	**0.0046**	5.71E-06	1.60E-06	**0.009**
DOPAMINERGIC EMBRYONIC NUCLEUS	3.98E-06	8.76E-07	0.0027	2.21E-06	1.04E-06	0.072
GABAergic EMBRYONIC	4.73E-06	2.05E+00	0.0541	9.85E-07	2.59E-06	0.715
SEROTONERGIC NEURON	4.04E-06	5.91E-07	**2.44E-04**	4.9E-06	1.8E-06	**0.031**

Supplementary Table 11. Functional enrichment for expression quantitative trait loci across pathways significantly associated with PD risk

Gene set	Tested eQTL Brain	Tested eQTL SN	Tested eQTL Blood	N_eQTL Brain	N_eQTL SN	N_eQTL Blood	P-value eQTL Enrichment Brain	P-value eQTL Enrichment SN	P-value eQTL Enrichment Blood
ACTIVATION OF AMPK DOWNSTREAM OF NMDARS (REACTOME)	8	1	17	2	0	3	0.009444166	0.8185458	1.67E-02
ADAPTIVE IMMUNE SYSTEM (REACTOME)	223	20	601	8	7	27	0.3331157	7.46664E-10	5.68E-01
ALPHA SYNUCLEIN PATHWAY (PID)	7	1	26	1	0	6	0.2596398	0.8185458	2.34E-05
ALZHEIMERS DISEASE (KEGG)	44	1	116	2	0	5	0.8899694	0.8185458	7.33E-01
AMYLOID FIBER FORMATION (REACTOME)	17	3	45	0	0	6	0.3441969	0.6911022	1.03E-02
APOPTOTIC CLEAVAGE OF CELLULAR PROTEINS (REACTOME)	16	1	30	2	0	4	0.1686686	0.8185458	3.62E-02
APOPTOTIC EXECUTION PHASE (REACTOME)	20	1	41	3	0	6	0.04017387	0.8185458	4.65E-03
ASPARAGINE N LINKED GLYCOSYLATION (REACTOME)	106	4	233	2	0	10	0.1413813	0.6463552	6.20E-01
CASPASE MEDIATED CLEAVAGE OF CYTOSKELETAL PROTEINS (REACTOME)	10	1	13	1	0	2	0.4681599	0.8185458	8.58E-02
CHROMATIN ORGANIZATION (REACTOME)	50	3	157	5	0	16	0.1047575	0.6911022	2.84E-03
CLASS B 2 SECRETIN FAMILY RECEPTORS (REACTOME)	19	4	40	2	1	4	0.2690456	0.06645742	1.47E-01
CLATHRIN MEDIATED ENDOCYTOSIS (REACTOME)	40	2	106	3	0	5	0.4681599	0.7456028	8.94E-01
COPI DEPENDENT GOLGI TO ER RETROGRADE TRAFFIC (REACTOME)	29	2	68	2	0	2	0.6393443	0.7456028	4.36E-01
COPI MEDIATED ANTEROGRADE TRANSPORT (REACTOME)	35	2	77	1	0	3	0.5607856	0.7456028	6.57E-01
COPII MEDIATED VESICLE TRANSPORT (REACTOME)	26	2	52	2	0	3	0.5287667	0.7456028	7.99E-01
ER TO GOLGI ANTEROGRADE TRANSPORT (REACTOME)	52	3	116	2	0	4	0.7026313	0.6911022	4.43E-01
GLUTAMATE BINDING ACTIVATION OF AMPA RECEPTORS AND SYNAPTIC PLASTICITY (REACTOME)	9	1	17	2	0	3	0.01775783	0.8185458	1.67E-02
GOLGI ASSOCIATED VESICLE BIOGENESIS (REACTOME)	21	1	48	2	0	1	0.3415081	0.8185458	3.54E-01
GPCR LIGAND BINDING (REACTOME)	71	7	172	4	1	8	0.8064257	0.2596398	8.34E-01
INNATE IMMUNE SYSTEM (REACTOME)	288	19	773	5	1	30	0.01103878	0.9580255	1.53E-01
INTRA GOLGI AND RETROGRADE GOLGI TO ER TRAFFIC (REACTOME)	68	2	152	1	0	5	0.1817469	0.7456028	3.33E-01
INTRA GOLGI TRAFFIC (REACTOME)	68	2	152	1	0	4	0.1817469	0.7456028	1.80E-01
LKB1 PATHWAY (PID)	17	1	38	2	0	2	0.2006316	0.8185458	9.41E-01
LONG TERM DEPRESSION (KEGG)	26	1	45	1	0	4	0.7871966	0.8185458	2.31E-01
LYSOSOME (KEGG)	47	2	104	3	0	13	0.6635418	0.7456028	4.49E-04
MAPK SIGNALING PATHWAY (KEGG)	63	3	165	3	0	13	0.9309012	0.6911022	8.98E-02
METABOLISM OF LIPIDS (REACTOME)	19	1	38	1	0	2	0.9580255	0.8185458	9.41E-01
METABOLISM OF VITAMINS AND COFACTORS (REACTOME)	252	9	507	5	0	19	0.02804341	0.4912971	1.96E-01
METABOLISM OF WATER SOLUBLE VITAMINS AND COFACTORS (REACTOME)	73	4	140	4	0	11	0.8509099	0.6463552	1.21E-01
NEUROACTIVE LIGAND RECEPTOR INTERACTION (KEGG)	59	5	92	4	0	3	0.5305183	0.6079589	4.44E-01
NEURONAL SYSTEM (REACTOME)	106	7	198	3	0	5	0.3053585	0.5438662	1.10E-01
NEUROTRANSMITTER RECEPTORS AND POSTSYNAPTIC SIGNAL TRANSMISSION (REACTOME)	51	3	101	2	0	4	0.7238104	0.6911022	6.32E-01
NEUTROPHIL DEGRANULATION (REACTOME)	155	11	399	3	0	17	0.08001898	0.4467252	4.98E-01
P38 GAMMA DELTA PATHWAY (PID)	5	2	9	1	0	2	0.1238122	0.7456028	1.78E-02
PARKINSONS DISEASE (KEGG)	31	1	81	2	0	4	0.7107578	0.8185458	9.80E-01
POST TRANSLATIONAL PROTEIN MODIFICATION (REACTOME)	401	16	954	5	1	35	0.00056392	0.8185458	5.92E-02
PTK6 PROMOTES HIF1A STABILIZATION (REACTOME)	4	1	6	2	0	2	3.64E-05	0.8185458	1.45E-03
RETROGRADE TRANSPORT AT THE TRANS GOLGI NETWORK (REACTOME)	24	1	40	1	0	4	0.851412	0.8185458	1.47E-01
SIGNALING BY GPCR (REACTOME)	170	11	391	4	1	15	0.1132883	0.5335849	2.91E-01
SNARE INTERACTIONS IN VESICULAR TRANSPORT (KEGG)	19	1	33	1	0	4	0.9580255	0.8185458	6.05E-02
TRAFFICKING OF GLUR2 CONTAINING AMPA RECEPTORS (REACTOME)	4	1	11	1	0	1	0.06645742	0.8185458	5.34E-01
TRANS GOLGI NETWORK VESICLE BUDDING (REACTOME)	23	1	61	2	0	2	0.4160918	0.8185458	5.37E-01
TRANSMISSION ACROSS CHEMICAL SYNAPSES (REACTOME)	70	6	141	2	0	5	0.4107282	0.5741491	4.28E-01
TRANSPORT TO THE GOLGI AND SUBSEQUENT MODIFICATION (REACTOME)	64	3	140	2	0	4	0.4912971	0.6911022	2.45E-01
VASOPRESSIN REGULATED WATER REABSORPTION (KEGG)	12	1	31	2	0	3	0.06368978	0.8185458	2.32E-01
VESICLE MEDIATED TRANSPORT (REACTOME)	209	10	526	6	0	13	0.1578491	0.4681599	7.80E-03

Supplementary Table 12. Functional associations for genes within significant pathways associated with PD risk by two sample Mendelian randomization

ACTIVATION OF AMPK DOWNSTREAM OF NMDARS (REACTOME)

Gene	probeID	ProbeChr	Probe bp	topSNP	topSNP chr	topSNP bp	A1	A2	Freq	b GWAS	se GWAS	p GWAS	b eQTL	se eQTL	p eQTL	b SMR	se SMR	p SMR	p SMR multi	p HEIDI	nsnp HEIDI	PASS BONF	Data source	Analyte
CAMKK2	ENSG00000110931	12	121705804	rs2686341	12	121684286	C	T	0.52972	0.0097	0.0096	0.3122957	0.227211	0.03909	6.15E-09	0.0426916	0.0428852	0.3194992	0.3194992	0.5932546	10	FALSE	Qi et al., 2018	Brain
PRKAB1	ENSG00000111725	12	120112496	rs7310266	12	120098409	C	T	0.318182	-0.001	0.0106	0.9248394	-0.310638	0.0486166	1.66E-10	0.00321918	0.034127	0.9248475	0.8626762	0.8766144	20	FALSE	Qi et al., 2018	Brain
PRKAB2	ENSG00000131791	1	146635407	rs2289575	1	146696955	G	C	0.445804	0.0033	0.0095	0.7283146	-0.280835	0.051376	4.60E-08	-0.0117507	0.033896	0.7288399	0.7288399	0.4900077	20	FALSE	Qi et al., 2018	Brain
PRKAA2	ENSG00000162409	1	57146001	rs706467	1	57181238	C	T	0.704545	-0.0073	0.0123	0.5528493	-0.238498	0.0394357	1.47E-09	0.0306082	0.0518205	0.5547492	0.5547492	0.8937058	9	FALSE	Qi et al., 2018	Brain
TUBA1C	ENSG00000167553	12	49624816	rs77888738	12	49512929	G	A	0.312937	0.0323	0.0105	0.00209664	0.962472	0.0422804	1.04E-114	0.0335594	0.0110086	0.00230001	0.001930488	0.00833916	20	TRUE	Qi et al., 2018	Brain
TUBB6	ENSG00000176014	18	12325993	rs4073975	18	12279075	T	C	0.353147	0.0093	0.0111	0.4021218	-0.410417	0.0528212	7.85E-15	-0.0226599	0.0272024	0.4048397	0.5111237	0.8437164	13	FALSE	Qi et al., 2018	Brain
MAPT	ENSG00000186868	17	44038724	rs199502	17	44862347	G	A	0.743007	0.1949	0.0156	8.09E-36	0.734607	0.0582562	1.86E-36	0.265312	0.0298938	6.98E-19	3.51E-24	0.01172117	17	TRUE	Qi et al., 2018	Brain
TUBB1	ENSG00000101162	20	57598009	rs463312	20	57597970	C	A	0.0332168	0.0138	0.0273	0.6132116	-0.150253	0.0199191	4.59E-14	-0.0918449	0.182101	0.6140061	0.6754224	0.4085688	3	FALSE	Vosa et al., 2018	Blood
TUBB4A	ENSG00000104833	19	6498594	rs2071347	19	6495736	A	G	0.347902	0.0184	0.0137	0.1792507	0.100279	0.00921581	1.42E-27	0.183488	0.137655	0.1825481	0.1071711	0.9145709	20	FALSE	Vosa et al., 2018	Blood
PRKAG2	ENSG00000106617	7	151413703	rs4726046	7	151253083	C	A	0.333916	0.001	0.0105	0.9241257	0.168136	0.00899471	5.67E-78	0.00594756	0.0624501	0.9241267	0.7620262	0.5978754	20	FALSE	Vosa et al., 2018	Blood
CAMKK2	ENSG00000110931	12	121705804	rs11065504	12	121680460	C	G	0.325175	-0.0023	0.0099	0.816287	0.53841	0.00925542	0	-0.0042718	0.0183876	0.8162884	0.00240685	0.2028056	20	TRUE	Vosa et al., 2018	Blood
PRKAB1	ENSG00000111725	12	120112496	rs11064881	12	120146925	A	G	0.0576923	-0.0055	0.0202	0.7854089	1.36131	0.024664	0	-0.0040402	0.0148388	0.7854114	0.7988889	0.8585428	20	FALSE	Vosa et al., 2018	Blood
PRKAG3	ENSG00000115592	2	219691957	rs7603912	2	219632002	T	G	0.466783	0.008	0.0094	0.3947339	0.12834	0.00904209	1.00E-45	0.0623345	0.0733746	0.395582	0.8657456	0.341287	20	FALSE	Vosa et al., 2018	Blood
TUBA1B	ENSG00000123416	12	49523372	rs2162644	12	49548190	G	A	0.681818	-0.029	0.0101	0.00408804	-0.0869697	0.00893831	2.25E-22	0.333449	0.121083	0.00588928	0.001977113	0.1202147	20	TRUE	Vosa et al., 2018	Blood
TUBA4A	ENSG00000127824	2	220128662	rs11552796	2	220108300	C	T	0.0996503	-0.0027	0.0155	0.8617134	-0.0889864	0.0120535	1.55E-13	0.0303417	0.174232	0.8617514	0.3492557	0.4658161	12	FALSE	Vosa et al., 2018	Blood
PRKAB2	ENSG00000131791	1	146635407	rs930495	1	146634514	T	G	0.638112	-0.0069	0.0097	0.4768734	0.217217	0.00901368	2.59E-128	-0.0317655	0.0446753	0.4770653	0.1452732	0.6632237	20	FALSE	Vosa et al., 2018	Blood
PRKAA1	ENSG00000132356	5	40778978	rs58751240	5	40791501	T	C	0.300699	0.0221	0.0105	0.03531204	0.0854267	0.00928652	3.61E-20	0.258701	0.126089	0.04019478	0.175909	0.02192962	20	FALSE	Vosa et al., 2018	Blood
TUBB2A	ENSG00000137267	6	3155831	rs9392465	6	3162378	A	C	0.681818	0.0051	0.0109	0.6398633	-0.596583	0.00813605	0	-0.0085487	0.0182711	0.6398701	0.6785826	0.2155316	20	FALSE	Vosa et al., 2018	Blood
TUBB2B	ENSG00000137285	6	3228229	rs9328145	6	3144908	A	G	0.697552	0.0137	0.011	0.2129647	0.154385	0.00889662	1.87E-67	0.0887393	0.0714338	0.214141	0.3289312	0.3902203	20	FALSE	Vosa et al., 2018	Blood
TUBA1A	ENSG00000167552	12	49580843	rs11168868	12	49515130	C	T	0.0297203	-0.0293	0.0387	0.4489864	0.467466	0.0446874	1.31E-25	-0.0626784	0.0830034	0.4501705	0.0410797	0.4141611	17	FALSE	Vosa et al., 2018	Blood
TUBB6	ENSG00000176014	18	12325993	rs7244254	18	12317836	A	G	0.146853	-0.0075	0.0145	0.6049877	0.7473	0.00935293	0	-0.0100361	0.0194036	0.6049952	0.6975407	0.5065391	20	FALSE	Vosa et al., 2018	Blood
MAPT	ENSG00000186868	17	44038724	rs62074125	17	44852612	C	A	0.225524	0.0962	0.0129	8.83E-14	-0.0984498	0.0146407	1.76E-11	-0.977148	0.195666	5.92E-07	1.03E-07	4.34E-10	6	TRUE	Vosa et al., 2018	Blood
CALM1	ENSG00000198668	14	90868725	rs7150626	14	90858484	T	C	0.79021	0.0139	0.0127	0.2737409	-0.231433	0.00883439	2.89E-151	-0.0600605	0.0549233	0.2741593	0.2292752	0.3046809	20	FALSE	Vosa et al., 2018	Blood

ADAPTIVE IMMUNE SYSTEM (REACTOME)

Gene	probeID	ProbeChr	Probe bp	topSNP	topSNP chr	topSNP bp	A1	A2	Freq	b GWAS	se GWAS	p GWAS	b eQTL	se eQTL	p eQTL	b SMR	se SMR	p SMR	p SMR multi	p HEIDI	nsnp HEIDI	PASS BONF	Data source	Analyte
MAP3K14	ENSG00000006062	17	43367451	rs72824757	17	42550474	C	A	0.103147	-0.0403	0.025	0.1069619	-0.46698	0.0839898	2.70E-08	0.0862992	0.0557402	0.1215643	0.1215643			FALSE	Qi et al., 2018	Brain
PSMB1	ENSG00000008018	6	170853317	rs3734763	6	170885912	T	C	0.611888	-0.0019	0.0097	0.844707	0.443175	0.0474856	1.03E-20	-0.0042873	0.0218924	0.8447408	0.8448789	0.8763937	20	FALSE	Qi et al., 2018	Brain
CD4	ENSG00000010610	12	6912999	rs10774447	12	6894449	T	C	0.711538	-0.014	0.0103	0.1740758	-0.301204	0.0492543	9.64E-10	0.0464801	0.0350306	0.1845604	0.2499093	0.8630806	8	FALSE	Qi et al., 2018	Brain
UBR2	ENSG00000024048	6	42596521	rs41273820	6	42666293	G	A	0.746503	0.0012	0.0112	0.9146756	-0.421234	0.0575452	2.48E-13	-0.0028488	0.0265914	0.9146848	0.988094	0.9445029	20	FALSE	Qi et al., 2018	Brain
BTN3A1	ENSG00000026950	6	26408954	rs4712984	6	26379537	C	T	0.604895	-0.0415	0.0127	0.00108419	-0.307607	0.0498499	6.80E-10	0.134912	0.0467181	0.00387952	0.003195861	0.00997827	20	FALSE	Qi et al., 2018	Brain
UBA6	ENSG00000033178	4	68524188	rs1586198	4	68571297	A	G	0.272727	-0.01	0.0115	0.3845381	-0.400743	0.056964	1.99E-12	0.0249537	0.0289151	0.3881388	0.5447083	0.9029707	15	FALSE	Qi et al., 2018	Brain
CUL3	ENSG00000036257	2	225392488	rs4402747	2	225457173	A	G	0.472028	-0.0027	0.0101	0.7892176	0.259467	0.0436918	2.88E-09	-0.010406	0.0389654	0.7894258	0.6707717	0.8336902	13	FALSE	Qi et al., 2018	Brain
CDH1	ENSG00000039068	16	68820286	rs9927407	16	68744532	A	G	0.701049	-0.0046	0.0104	0.6582666	-0.475348	0.0490283	3.15E-22	0.00967713	0.0219015	0.6585991	0.9940708	0.9150806	20	FALSE	Qi et al., 2018	Brain
PSMA4	ENSG00000041357	15	78837175	rs58365910	15	78849034	C	T	0.384615	0.0038	0.01	0.7039454	0.360885	0.0525747	6.68E-12	0.0105297	0.0277521	0.7043767	0.613392	0.4011593	20	FALSE	Qi et al., 2018	Brain
LCP2	ENSG00000043462	5	169699236	rs11134621	5	169666187	C	A	0.517483	-0.029	0.0116	0.01241933	0.333054	0.0457817	3.47E-13	-0.0870731	0.0368285	0.01806474	0.02166176	0.0902474	18	FALSE	Qi et al., 2018	Brain
VAMP3	ENSG00000049245	1	7836410	rs1048785	1	7841330	G	A	0.872378	-0.0132	0.0151	0.3820245	-0.680838	0.0667404	1.96E-24	0.0193879	0.0222598	0.383765	0.301999	0.4502215	20	FALSE	Qi et al., 2018	Brain
CYBA	ENSG00000051523	16	88713625	rs3794628	16	88729788	C	T	0.688811	-0.0069	0.0106	0.515083	0.295562	0.0508541	6.17E-09	-0.0233454	0.0360881	0.5176975	0.5176975	0.9731089	20	FALSE	Qi et al., 2018	Brain
ANAPC4	ENSG00000053900	4	25399477	rs1533133	4	25384305	C	T	0.526224	0.0111	0.0095	0.242637	-0.96801	0.0273641	4.11E-274	-0.0114668	0.0098193	0.2428938	0.3648168	0.3549238	20	FALSE	Qi et al., 2018	Brain
UNKL	ENSG00000059145	16	1438979	rs4984824	16	1421797	G	T	0.896853	-0.0002	0.016	0.9900267	-0.477131	0.0807848	3.50E-09	0.00041917	0.0335338	0.9900267	0.8586375	0.0862868	20	FALSE	Qi et al., 2018	Brain
RNF4	ENSG00000063978	4	2545497	rs2282765	4	2512054	G	A	0.45979	-0.0127	0.0099	0.1995522	-0.356625	0.0493728	5.08E-13	0.0356116	0.0281946	0.2065665	0.03094219	0.08921853	20	FALSE	Qi et al., 2018	Brain
CTSA	ENSG00000064601	20	44523525	rs6073967	20	44571220	C	T	0.643357	-0.0213	0.0099	0.03143556	0.324353	0.0508241	1.75E-10	-0.0656692	0.0322101	0.04147268	0.0253848	0.6371675	20	FALSE	Qi et al., 2018	Brain
BTBD1	ENSG00000064726	15	83710640	rs72758319	15	83857434	C	T	0.914336	-0.0151	0.0164	0.3571905	1.69586	0.0462214	1.06E-294	-0.0089041	0.00967367	0.3573419	0.2931133	0.3962818	20	FALSE	Qi et al., 2018	Brain
SEC61A2	ENSG00000065665	10	12191798	rs7099650	10	12201176	T	C	0.498252	-0.0066	0.0094	0.4825996	0.325692	0.0374698	3.56E-18	-0.0202646	0.0289557	0.4840222	0.6536052	0.1143524	20	FALSE	Qi et al., 2018	Brain
ASB1	ENSG00000065802	2	239348137	rs10929275	2	239323169	A	G	0.40035	0.0367	0.0095	0.00011193	0.398725	0.0415934	9.13E-22	0.0920433	0.0256878	0.00033948	0.001500112	0.759247	20	FALSE	Qi et al., 2018	Brain
PPP2R5A	ENSG00000066027	1	212496991	rs4548507	1	212564028	T	A	0.687063	-0.0226	0.0103	0.02822285	-0.378131	0.0475545	1.84E-15	0.0597676	0.0282573	0.03441937	0.07290744	0.8741837	20	FALSE	Qi et al., 2018	Brain
PSME4	ENSG00000068878	2	54144590	rs62139336	2	54212233	G	A	0.734266	0.0053	0.0105	0.6137261	0.264893	0.0484695	4.63E-08	0.020008	0.0398073	0.6152296	0.6152296	0.2785139	20	FALSE	Qi et al., 2018	Brain
NFATC3	ENSG00000072736	16	68190908	rs7206718	16	67449831	G	A	0.447552	-0.0013	0.0107	0.9032987	-0.333879	0.0507645	4.80E-11	0.00389363	0.032053	0.9033151	0.2136227	0.05905215	20	FALSE	Qi et al., 2018	Brain
AP1M1	ENSG00000072958	19	16327274	rs12984337	19	16343847	A	T	0.706294	0.0078	0.0111	0.482241	1.20089	0.0327215	7.16E-295	0.00649516	0.0092448	0.4823213	0.5096056	0.4189146	20	FALSE	Qi et al., 2018	Brain
PVR	ENSG00000073008	19	45158263	rs11540084	19	45147340	G	T	0.802448	-0.0033	0.0117	0.7779042	-0.500027	0.0437004	2.57E-30	0.00659964	0.0234058	0.7779699	0.8699604	0.2719708	20	FALSE	Qi et al., 2018	Brain
RAB7A	ENSG00000075785	3	128489302	rs73198889	3	128547620	C	T	0.924825	0.0362	0.0208	0.08179151	0.760414	0.112317	1.29E-11	0.0476056	0.0282428	0.09187625	0.160095	0.9144053	18	FALSE	Qi et al., 2018	Brain
ICAM3	ENSG00000076662	19	10447475	rs10403396	19	10447819	A	C	0.732517	-0.0013	0.0112	0.9075959	1.18423	0.0319845	4.51E-300	-0.0010978	0.00945766	0.9075964	0.6039226	0.3958415	20	FALSE	Qi et al., 2018	Brain
CAPZB	ENSG00000077549	1	19738666	rs12117356	1	19658162	C	G	0.807692	0.0036	0.0118	0.7603016	1.10409	0.0318829	9.04E-263	0.00326059	0.0106879	0.7603106	0.8477152	0.8377617	20	FALSE	Qi et al., 2018	Brain
PPP2R5C	ENSG00000078304	14	102311230	rs1741143	14	102392122	A	G	0.0646853	-0.0243	0.0177	0.1697892	-0.46509	0.0643689	5.00E-13	0.052248	0.038738	0.1774172	0.1244309	0.6652541	20	FALSE	Qi et al., 2018	Brain
RAPGEF3	ENSG00000079337	12	48146639	rs12424297	12	48142343	T	C	0.854895	0.0229	0.0132	0.08276765	-0.660889	0.0530908	1.43E-35	-0.0346503	0.0201661	0.0857524	0.1028285	0.1029373	20	FALSE	Qi et al., 2018	Brain
HACE1	ENSG00000085382	6	105241881	rs7769270	6	105230729	A	C	0.5	-0.0088	0.0095	0.3542819	-0.460664	0.0388839	2.23E-32	0.0191029	0.0206854	0.3557482	0.3573487	0.8989986	20	FALSE	Qi et al., 2018	Brain
PILRA	ENSG00000085514	7	99981436	rs11768933	7	99967737	T	C	0.176573	-0.0409	0.0122	0.00080097	1.39479	0.0346749	0	-0.0293235	0.00877719	0.00083514	0.000889775	0.01474002	20	FALSE	Qi et al., 2018	Brain
PSMC5	ENSG00000087191	17	61906945	rs62077481	17	61797873	A	G	0.417832	0.0039	0.0099	0.6936258	-0.34227	0.0395611	5.07E-18	-0.0113945	0.0289545	0.6939268	0.8074967	0.1263962	20	FALSE	Qi et al., 2018	Brain
DYNLL1	ENSG00000088986	12	120921974	rs12310837	12	120880572	A	G	0.802448	-0.0254	0.0128	0.04721405	0.313888	0.0464368	1.39E-11	-0.0809206	0.0424998	0.05690721	0.03121166	0.362197	14	FALSE	Qi et al., 2018	Brain
ANAPC5	ENSG00000089053	12	121791873	rs2668237	12	121730941	C	G	0.648601	0.0053	0.0127	0.6764423	0.296436	0.0526331	1.78E-08	0.017879	0.0429597	0.6772769	0.855521	0.4433064	9	FALSE	Qi et al., 2018	Brain
CD209	ENSG00000090659	19	7808671	rs4804802	19	7807610	G	A	0.90035	0.0247	0.0177	0.1628711	0.503771	0.0902708	2.40E-08	0.0490302	0.0362168	0.1758012	0.1758012	0.1863155	4	FALSE	Qi et al., 2018	Brain
SNAP23	ENSG00000092531	15	42810489	rs11635041	15	42778347	C	T	0.162587	0.0428	0.0129	0.0009072	-0.359216	0.062127	7.38E-09	-0.119148	0.0414039	0.00400576	0.006224645	0.3499936	20	FALSE	Qi et al., 2018	Brain
PSMD5	ENSG00000095261	9	123591501	rs10818476	9	123572038	G	A	0.601399	0.0121	0.0099	0.2216236	-0.331402	0.0368338	2.32E-19	-0.0365116	0.0301475	0.2258576	0.2185749	0.7231306	20	FALSE	Qi et al., 2018	Brain
TREM2	ENSG00000095970	6	41128584	rs17644657	6	41240941	G	A	0.368881	-0.0071	0.01	0.4777041	0.232235	0.0402272	7.78E-09	-0.0305724	0.0433842	0.4810027	0.7383943	0.6723545	20	FALSE	Qi et al., 2018	Brain
MADCAM1	ENSG00000099866	19	497261	rs2302217	19	498524	A	G	0.533217	-0.0154	0.0128	0.228928	0.332985	0.0432489	1.37E-14	-0.0462484	0.0389067	0.234558	0.2226989	0.7409659	7	FALSE	Qi et al., 2018	Brain
FBXO7	ENSG00000100225	22	32882740	rs5749449	22	32882440	G	C	0.370629	-0.002	0.0097	0.836646	0.478528	0.0373464	1.38E-37	-0.0041795	0.0202731	0.8366668	0.7841525	0.581596	19	FALSE	Qi et al., 2018	Brain
ASB2	ENSG00000100628	14	94421818	rs4905125	14	94442455	C	T	0.223776	0.0103	0.0123	0.4023687	-0.335053	0.0418503	1.19E-15	-0.0307414	0.0369109	0.4049266	0.6600649	0.3855416	11	FALSE	Qi et al., 2018	Brain
PSMB5	ENSG00000100804	14	23495095	rs941718	14	23495048	T	C	0.688811	0.008	0.0107	0.4546631	-0.36666	0.0526726	3.38E-12	-0.0218186	0.0293502	0.4572468	0.7039184	0.5030934	12	FALSE	Qi et al., 2018	Brain
SEC23A	ENSG00000100934	14	39539986	rs11848164	14	39541159	C	T	0.527972	-0.0179	0.0098	0.06777037	0.305575	0.0480642	2.05E-10	-0.058578	0.0333679	0.07917085	0.4406141	0.05743821	20	FALSE	Qi et al., 2018	Brain
CD40	ENSG00000101017	20	44752706	rs1883832	20	44746982	T	C	0.269231	0.0182	0.0107	0.08895527	-0.439742	0.0575004	2.05E-14	-0.0413879	0.024927	0.09684143	0.1872149	0.3805412	20	FALSE	Qi et al., 2018	Brain
PSMA7	ENSG00000101182	20	60715143	rs6142716	20	60721829	G	C	0.83042	-0.0239	0.0126	0.05785099	-0.410285	0.0554337	1.35E-13	0.0582521	0.0317028	0.06614443	0.1284613	0.8706246	20	FALSE	Qi et al., 2018	Brain
TRIB3	ENSG00000101255	20	369732	rs62191434	20	360062	C	T	0.424825	-0.0092	0.0112	0.4114022	0.525816	0.0382687	5.84E-43	-0.0174966	0.0213382	0.4122363	0.4809791	0.8471523	20	FALSE	Qi et al., 2018	Brain
MGRN1	ENSG00000102858	16	4703734	rs841225	16	4736316	T	C	0.554196	0.0011	0.0097	0.9097118	1.19374	0.0307143	0	0.00092147	0.00812575	0.9097122	0.3536179	0.1806427	20	FALSE	Qi et al., 2018	Brain
CTSH	ENSG00000103811	15	79227658	rs34843303	15	79234470	T	C	0.888112	0.0286	0.0169	0.09058732	0.547853	0.0631447	4.09E-18	0.0522038	0.031429	0.09671232	0.08476048	0.2773457	9	FALSE	Qi et al., 2018	Brain
UBE2W	ENSG00000104343	8	74741738	rs35204486	8	74802859	C	T	0.867133	0.0045	0.016	0.7785187	-0.331773	0.0567926	5.16E-09	-0.0135635	0.0482815	0.7787682	0.7787682	0.9304022	20	FALSE	Qi et al., 2018	Brain
PPP2CB	ENSG00000104695	8	30651901	rs10109293	8	30651206	A	T	0.903846	-0.0058	0.0151	0.7008999	0.435413	0.0716539	1.23E-09	-0.0133207	0.0347489	0.701467	0.7532496	0.8431142	12	FALSE	Qi et al., 2018	Brain
NFKBIB	ENSG00000104825	19	39394936	rs3136642	19	39398416	A	G	0.622378	-0.0131	0.0104	0.2078081	-0.985924	0.0278375	9.31E-275	0.013287	0.0105551	0.2080955	0.2967082	0.6223028	20	FALSE	Qi et al., 2018	Brain
KLC3	ENSG00000104892	19	45844776	rs10416849	19	45845231	C	G	0.624126	-0.0003	0.0106	0.9774214	-0.417433	0.0721858	7.35E-09	0.00071868	0.0253936	0.9774216	0.8390934	0.3370932	20	FALSE	Qi et al., 2018	Brain
LILRB1	ENSG00000104972	19	55117162	rs2114512	19	55134895	T	C	0.659091	-0.0006	0.0102	0.9530927	0.395427	0.0380964	3.07E-25	-0.0015174	0.0257953	0.9530934	0.8651381	0.8597957	9	FALSE	Qi et al., 2018	Brain
FZR1	ENSG00000105325	19	3522299	rs10417370	19	3522132	C	T	0.45979	-0.0114	0.0098	0.2447219	-0.281392	0.0433245	8.30E-11	0.0405129	0.035381	0.2521905	0.2019662	0.7904182	20	FALSE	Qi et al., 2018	Brain
AP1S1	ENSG00000106367	7	100801277	rs10953325	7	100805508	A	G	0.548951	0.001	0.0099	0.9195424	-1.132	0.030539	9.43E-301	-0.0008834	0.0087456	0.9195427	0.5947243	0.265228	20	FALSE	Qi et al., 2018	Brain
PSMA2	ENSG00000106588	7	42964141	rs615770	7	42975857	G	A	0.907343	-0.0229	0.0166	0.1677351	0.645827	0.103898	5.10E-10	-0.0354584	0.0263289	0.1780606	0.1487038	0.5984108	14	FALSE	Qi et al., 2018	Brain
DNM1	ENSG00000106976	9	130991592	rs10760546	9	130957275	T	A	0.370629	-0.0177	0.0099	0.07379557	0.290779	0.043169	1.63E-11	-0.060871	0.0352254	0.0839806	0.3069471	0.01942137	20	FALSE	Qi et al., 2018	Brain
SH3GL2	ENSG00000107295	9	17688103	rs10756899	9	17684784	G	C	0.781469	-0.0836	0.0112	8.38E-14	-0.331527	0.0397159	6.97E-17	0.252166	0.0453196	2.63E-08	9.04E-08	0.07186738	20	TRUE	Qi et al., 2018	Brain
UBE2R2	ENSG00000107341	9	33868983	rs10971896	9	34081760	G	A	0.865385	0.0181	0.0148	0.2213399	0.529933	0.0647837	2.84E-16	0.0341553	0.0282385	0.2264594	0.06007716	0.012556	20	FALSE	Qi et al., 2018	Brain
BLMH	ENSG00000108578	17	28597146	rs8073378	17	28629763	G	A	0.576923	0.0056	0.0097	0.5637236	1.03697	0.0293648	3.67E-273	0.00540035	0.00935542	0.5637757	0.5206789	0.3355456	20	FALSE	Qi et al., 2018	Brain

ICAM2	ENSG00000108622	17	62090230	rs11079520	17	62079334	A	G	0.594406	0.0166	0.0106	0.1173398	0.279733	0.0503148	2.70E-08	0.0593422	0.0393678	0.1317135	0.1317135	0.7029874	6	FALSE	Qi et al., 2018	Brain
UBE2D3	ENSG00000109332	4	103752796	rs7681002	4	103895223	G	A	0.479021	-0.0278	0.0101	0.00591466	-0.294691	0.0452118	7.12E-11	0.094336	0.0372037	0.01122363	0.02404545	0.2881769	20	FALSE	Qi et al., 2018	Brain
CD81	ENSG00000110651	11	2408017	rs739547	11	2335444	C	G	0.216783	-0.0089	0.0116	0.442938	0.455382	0.0507394	2.84E-19	-0.019544	0.025566	0.4445973	0.5872009	0.4950087	20	FALSE	Qi et al., 2018	Brain
SPSB2	ENSG00000111671	12	6989310	rs7311510	12	6987232	C	A	0.333916	0.0192	0.0106	0.07009121	1.13184	0.0295078	0	0.0169636	0.00937575	0.0704037	0.1759453	0.2577626	20	FALSE	Qi et al., 2018	Brain
BTN3A3	ENSG00000111801	6	26447171	rs13220495	6	26441640	T	C	0.0821678	0.0217	0.0172	0.2070827	0.570888	0.0791705	5.56E-13	0.0380109	0.0305861	0.21396	0.00324667	0.01343947	20	FALSE	Qi et al., 2018	Brain
ULBP1	ENSG00000111981	6	150289994	rs1951931	6	150296898	C	A	0.825175	-0.0123	0.0132	0.3514305	0.688492	0.0874702	3.51E-15	-0.0178651	0.0193062	0.3547805	0.6196545	0.684325	13	FALSE	Qi et al., 2018	Brain
FBXL4	ENSG00000112234	6	99358591	rs2743877	6	99319240	T	A	0.47028	-0.0132	0.0102	0.1956248	-0.537066	0.0401491	8.26E-41	0.024578	0.0190807	0.1977098	0.2013189	0.4164068	20	FALSE	Qi et al., 2018	Brain
BTN2A1	ENSG00000112763	6	26467499	rs6456723	6	26455505	C	G	0.795455	-0.0171	0.0115	0.1370263	-0.42886	0.043978	1.81E-22	0.0398731	0.0271252	0.141571	0.2146645	0.05039869	20	FALSE	Qi et al., 2018	Brain
LNPEP	ENSG00000113441	5	96322158	rs2432142	5	96275201	G	A	0.573427	-0.0083	0.0097	0.3921803	-0.301613	0.0409507	1.77E-13	0.0275187	0.0323768	0.395351	0.6584968	0.3968806	20	FALSE	Qi et al., 2018	Brain
RNF7	ENSG00000114125	3	141461724	rs868934	3	141455781	G	A	0.685315	0.0224	0.011	0.04171385	0.301081	0.042434	1.29E-12	0.0743986	0.03801	0.05030695	0.03380671	0.0171931	20	FALSE	Qi et al., 2018	Brain
ACTR1B	ENSG00000115073	2	98276500	rs11683207	2	98333290	T	C	0.840909	-0.0067	0.0135	0.6196854	-0.415408	0.0673283	6.83E-10	0.0161287	0.0326031	0.620813	0.2330629	0.394342	5	FALSE	Qi et al., 2018	Brain
ZAP70	ENSG00000115085	2	98343174	rs11894651	2	98317771	C	A	0.302448	-0.0178	0.0106	0.09310425	0.901224	0.0877934	1.01E-24	-0.0197509	0.0119181	0.09747539	0.05491509	0.331965	18	FALSE	Qi et al., 2018	Brain
ASB3	ENSG00000115239	2	53923553	rs1127767	2	53955982	T	A	0.949301	0.019	0.0233	0.4148144	0.812696	0.102143	1.77E-15	0.023379	0.0288202	0.4172501	0.4172501	0.8119097	20	FALSE	Qi et al., 2018	Brain
CAPZA1	ENSG00000116489	1	113188018	rs4839257	1	113219388	T	A	0.723776	0.0225	0.0108	0.03722085	-0.44122	0.0513318	8.29E-18	-0.0509949	0.0251863	0.04289715	0.02521541	0.4549249	19	FALSE	Qi et al., 2018	Brain
FBXO6	ENSG00000116663	1	11729296	rs3125812	1	11724938	C	A	0.480769	0.0272	0.0104	0.0089127	-0.315213	0.0375008	4.26E-17	-0.0862909	0.0345538	0.01251469	0.01145506	0.7583416	14	FALSE	Qi et al., 2018	Brain
CTSD	ENSG00000117984	11	1779602	rs72850955	11	1777037	T	C	0.917832	0.0161	0.0167	0.3350099	0.551646	0.0863553	1.68E-10	0.0291854	0.0306159	0.3404497	0.333475	0.8351153	20	FALSE	Qi et al., 2018	Brain
UBE3D	ENSG00000118420	6	83688838	rs12210375	6	83700385	C	T	0.131119	-0.0284	0.0143	0.04703177	0.326339	0.0566777	8.52E-09	-0.087026	0.0463529	0.06045404	0.08619865	0.04901647	20	FALSE	Qi et al., 2018	Brain
FBXL5	ENSG00000118564	4	15644732	rs1134634	4	15603069	G	C	0.381119	0.0046	0.0096	0.6318201	0.302496	0.050381	1.92E-09	0.0152068	0.0318369	0.6329007	0.004654579	9.04E-06	20	FALSE	Qi et al., 2018	Brain
VAMP8	ENSG00000118640	2	85798919	rs1446669	2	85762209	T	C	0.673077	0.0211	0.0103	0.04050676	0.285194	0.0505642	1.70E-08	0.0739847	0.0384241	0.05416979	0.05416979	0.1803202	18	FALSE	Qi et al., 2018	Brain
PPL	ENSG00000118898	16	4971625	rs9635542	16	5001380	A	G	0.903846	-0.0113	0.0164	0.4908079	0.400023	0.0546895	2.58E-13	-0.0282484	0.0411791	0.492721	0.58881	0.1824103	17	FALSE	Qi et al., 2018	Brain
CD274	ENSG00000120217	9	5460534	rs7042084	9	5458035	G	T	0.445804	-0.003	0.01	0.7641772	0.436638	0.045478	7.91E-22	-0.0068707	0.0229134	0.7642888	0.4388048	0.2678029	20	FALSE	Qi et al., 2018	Brain
PILRB	ENSG00000121716	7	99949578	rs6965458	7	99973402	A	G	0.176573	-0.0415	0.0122	0.00066983	1.429	0.034191	0	-0.0290413	0.00856567	0.00069783	0.001058656	0.01767239	20	FALSE	Qi et al., 2018	Brain
CLTA	ENSG00000122705	9	36247815	rs7041383	9	36204912	C	T	0.875874	0.0083	0.0169	0.6233386	0.558835	0.0803854	3.60E-12	0.0148523	0.0303168	0.6242023	0.570455	0.1781766	10	FALSE	Qi et al., 2018	Brain
ITPR2	ENSG00000123104	12	26738236	rs4322504	12	26982905	C	T	0.879371	0.0025	0.0168	0.8817039	-0.635378	0.0589142	4.06E-27	-0.0039347	0.0264435	0.8817151	0.4620927	0.3315176	20	FALSE	Qi et al., 2018	Brain
RNF114	ENSG00000124226	20	48561688	rs4809767	20	48568258	T	C	0.858392	0.0005	0.0159	0.9749134	-0.765456	0.0749958	1.85E-24	-0.0006532	0.020772	0.9749135	0.8043091	0.08413274	20	FALSE	Qi et al., 2018	Brain
BTN2A2	ENSG00000124508	6	26389213	rs1131936	6	26394320	A	G	0.875874	-0.0064	0.0133	0.6303722	-0.544315	0.0693263	4.11E-15	0.0117579	0.0244802	0.6310128	0.4671135	0.07395855	20	FALSE	Qi et al., 2018	Brain
PSMF1	ENSG00000125818	20	1121464	rs6074148	20	1098486	T	C	0.732517	-0.003	0.0105	0.775097	0.359486	0.0420357	1.21E-17	-0.0083453	0.0292247	0.775219	0.7171884	0.7486312	15	FALSE	Qi et al., 2018	Brain
RBCK1	ENSG00000125826	20	399876	rs11698154	20	398278	G	T	0.795455	-0.0088	0.0131	0.5017392	-0.286539	0.0494805	7.00E-09	0.0307113	0.0460245	0.5045925	0.2483762	0.3197535	6	FALSE	Qi et al., 2018	Brain
KLC1	ENSG00000126214	14	104098060	rs709399	14	104167545	A	G	0.695804	0.0182	0.0103	0.07722985	0.374091	0.0526291	1.18E-12	0.0486512	0.0283714	0.08638189	0.1263823	0.4879868	20	FALSE	Qi et al., 2018	Brain
CANX	ENSG00000127022	5	179131777	rs13182141	5	179126632	C	T	0.486014	-0.0003	0.0102	0.9765362	0.380574	0.0376294	4.80E-24	-0.0007883	0.0268017	0.9765363	0.5781448	0.4643663	20	FALSE	Qi et al., 2018	Brain
RNF6	ENSG00000127870	13	26751522	rs11558132	13	26795463	C	A	0.454545	-0.0036	0.0101	0.7215143	0.399659	0.0457325	2.35E-18	-0.0090077	0.0252926	0.7217361	0.8719043	0.897804	20	FALSE	Qi et al., 2018	Brain
ANAPC13	ENSG00000129055	3	134201053	rs13090969	3	134307257	A	C	0.321678	0.0055	0.0101	0.58606	-1.03354	0.0290074	4.80E-278	-0.0053215	0.00977342	0.5861037	0.2769441	0.2526478	20	FALSE	Qi et al., 2018	Brain
CDC16	ENSG00000130177	13	115019280	rs7324079	13	115015577	T	C	0.688811	-0.0035	0.0111	0.7525223	-0.41565	0.0417945	2.65E-23	0.00842056	0.0267186	0.7526425	0.6599417	0.2835876	20	FALSE	Qi et al., 2018	Brain
UBAC1	ENSG00000130560	9	138839020	rs11103233	9	138829709	T	C	0.491259	0.0044	0.0101	0.6630954	-0.303992	0.0510809	2.66E-09	-0.014474	0.0333134	0.6639392	0.5737203	0.4170005	14	FALSE	Qi et al., 2018	Brain
ACTR10	ENSG00000131966	14	58684274	rs56759383	14	58653480	A	C	0.575175	-0.01	0.0099	0.3124469	-0.261863	0.0473284	3.15E-08	0.0381879	0.0384309	0.3203799	0.3203799	0.7481786	9	FALSE	Qi et al., 2018	Brain
RAF1	ENSG00000132155	3	12665412	rs55977822	3	12704438	A	C	0.246503	-0.0007	0.0116	0.951881	0.313434	0.0560423	2.23E-08	-0.0022333	0.0370115	0.9518838	0.9518838	0.6146105	20	FALSE	Qi et al., 2018	Brain
SEC61G	ENSG00000132432	7	54823805	rs62449759	7	54807585	C	T	0.942308	-0.0032	0.0191	0.8669458	0.929703	0.0957578	2.76E-22	-0.003442	0.0205472	0.8669654	0.366972	0.2949889	20	FALSE	Qi et al., 2018	Brain
FBXO44	ENSG00000132879	1	11718908	rs3125815	1	11722688	G	A	0.809441	-0.0288	0.0123	0.01920831	0.619603	0.0557752	1.14E-28	-0.0464814	0.0202876	0.02195622	0.009334654	0.3862285	16	FALSE	Qi et al., 2018	Brain
SFTPD	ENSG00000133661	10	81719933	rs4335482	10	81743578	C	T	0.590909	-0.0073	0.0097	0.451704	1.05948	0.0280233	0	-0.0068902	0.00915725	0.4517936	0.6664689	0.4212128	20	FALSE	Qi et al., 2018	Brain
KRAS	ENSG00000133703	12	25380796	rs12818169	12	25291257	C	T	0.566434	-0.0306	0.0095	0.00127721	0.857707	0.02746	3.60E-214	-0.0356765	0.0111348	0.00135504	0.005492356	0.03265921	20	FALSE	Qi et al., 2018	Brain
VHL	ENSG00000134086	3	10188298	rs1642729	3	10187377	G	A	0.868881	0.0215	0.0149	0.1490337	-0.59928	0.0502074	7.67E-33	-0.0358764	0.0250442	0.1519941	0.04493453	0.1038921	14	FALSE	Qi et al., 2018	Brain
PTPN22	ENSG00000134242	1	114385407	rs6665194	1	114417843	G	A	0.582168	-0.0245	0.0098	0.01241933	-0.270859	0.0412868	5.37E-11	0.0904531	0.0387193	0.01948471	0.005915456	0.1662603	19	FALSE	Qi et al., 2018	Brain
KLRC1	ENSG00000134545	12	10602955	rs4763544	12	10650862	T	A	0.809441	0.0274	0.014	0.05033067	-0.993544	0.0965446	7.73E-25	-0.027578	0.0143435	0.05452003	0.0825453	0.9039375	20	FALSE	Qi et al., 2018	Brain
RNF138	ENSG00000134758	18	29691671	rs28378426	18	29687904	G	A	0.646853	0.012	0.01	0.2301393	-0.263737	0.0463112	1.23E-08	-0.0454999	0.0387492	0.2403089	0.4291247	0.23878	15	FALSE	Qi et al., 2018	Brain
FBXO21	ENSG00000135108	12	117604741	rs2036311	12	117599698	C	T	0.45979	-0.0022	0.0101	0.827568	-0.360323	0.0479172	5.49E-14	0.00610564	0.0280422	0.8276391	0.9893753	0.9776838	13	FALSE	Qi et al., 2018	Brain
MAP3K7	ENSG00000135341	6	91260028	rs61207072	6	91304259	T	C	0.445804	0.0132	0.0103	0.1999994	-0.372752	0.049538	5.29E-14	-0.0354123	0.0280302	0.2064595	0.2358085	0.859607	20	FALSE	Qi et al., 2018	Brain
RAC1	ENSG00000136238	7	6428881	rs836551	7	6441799	G	C	0.513986	0.0217	0.0097	0.02527893	-0.457362	0.0472917	4.00E-22	-0.047446	0.0217686	0.02929006	0.002505209	0.05133831	20	FALSE	Qi et al., 2018	Brain
TLR4	ENSG00000136869	9	120472879	rs1329057	9	120441946	T	C	0.795455	0.0307	0.0128	0.01646519	-0.44958	0.0558815	8.61E-16	-0.0682859	0.0297093	0.02153488	0.004993434	0.4748032	20	FALSE	Qi et al., 2018	Brain
PSMB7	ENSG00000136930	9	127146734	rs10818962	9	127182293	C	G	0.65035	0.0019	0.0098	0.8462718	0.302047	0.0446851	1.39E-11	0.00629041	0.0324586	0.8463342	0.9094104	0.9225007	20	FALSE	Qi et al., 2018	Brain
RNF144B	ENSG00000137393	6	18418942	rs7771828	6	18398335	G	T	0.631119	0.0066	0.0109	0.5448438	-0.277426	0.0499839	2.85E-08	-0.0237901	0.0395228	0.5472186	0.5472186	0.5919282	17	FALSE	Qi et al., 2018	Brain
PPP2R1B	ENSG00000137713	11	111617391	rs631318	11	111677695	A	T	0.374126	-0.0052	0.0101	0.6066568	1.02032	0.028388	6.76E-283	-0.0050965	0.00989989	0.6066937	0.7742361	0.5714294	20	FALSE	Qi et al., 2018	Brain
ITGAV	ENSG00000138448	2	187500210	rs1448423	2	187443285	T	C	0.617133	0.0062	0.0104	0.5510725	0.471483	0.0394828	7.19E-33	0.01315	0.0220855	0.5515679	0.8632527	0.9900709	20	FALSE	Qi et al., 2018	Brain
HERC3	ENSG00000138641	4	89535946	rs28409937	4	89608912	T	G	0.97028	-0.0311	0.0293	0.288493	-1.03276	0.16749	7.00E-10	0.0301134	0.0287878	0.2955387	0.331228	0.1406833	15	FALSE	Qi et al., 2018	Brain
HERC6	ENSG00000138642	4	89332077	rs28397254	4	89286868	C	T	0.91958	-0.0005	0.0218	0.9817015	-0.61172	0.0950709	1.24E-10	0.00081737	0.0356375	0.9817016	0.431613	0.09258503	7	FALSE	Qi et al., 2018	Brain
CENPE	ENSG00000138778	4	104073264	rs72666813	4	104122401	A	G	0.206294	-0.0012	0.0118	0.9189988	0.374994	0.0618735	1.36E-09	-0.0032001	0.0314716	0.9190102	0.6013098	0.2185971	20	FALSE	Qi et al., 2018	Brain
KLRG1	ENSG00000139187	12	9132998	rs12816157	12	9183067	G	A	0.730769	-0.004	0.0105	0.7032386	-0.541203	0.0536581	6.36E-24	0.00739095	0.0194151	0.70344	0.7591609	0.8212048	20	FALSE	Qi et al., 2018	Brain
PDPK1	ENSG00000140992	16	2620577	rs11648777	16	2705416	A	G	0.40035	0.001	0.0115	0.9307061	0.960592	0.0287921	4.71E-244	0.00104103	0.0119718	0.9307063	0.6466102	0.277812	15	FALSE	Qi et al., 2018	Brain
FBXO15	ENSG00000141665	18	71777844	rs3813119	18	71814438	C	A	0.882867	-0.0014	0.0158	0.9293938	0.478176	0.0575172	9.28E-17	-0.0029278	0.0330441	0.9293978	0.9391243	0.8848307	20	FALSE	Qi et al., 2018	Brain
AKT1	ENSG00000142208	14	105248887	rs1130233	14	105239894	T	C	0.251748	0.0109	0.0113	0.3347443	0.313444	0.0540874	6.83E-09	0.0347749	0.0365471	0.3413452	0.3413452	0.1883117	19	FALSE	Qi et al., 2018	Brain
SIGLEC10	ENSG00000142512	19	51917166	rs12984091	19	51940265	C	A	0.863636	-0.0083	0.0155	0.5923153	-0.38168	0.0683149	2.31E-08	0.021746	0.040796	0.5940051	0.5940051	0.7281739	6	FALSE	Qi et al., 2018	Brain
CTSK	ENSG00000143387	1	150774741	rs2089081	1	150800117	T	C	0.480769	-0.0244	0.0094	0.00943862	0.377541	0.0484303	6.41E-15	-0.0646287	0.0262419	0.01378559	0.009707904	0.8806857	20	FALSE	Qi et al., 2018	Brain
DYNC1LI1	ENSG00000144635	3	32589914	rs28403572	3	32612434	T	C	0.0979021	0.0093	0.0178	0.6013418	-0.389773	0.0692132	1.79E-08	-0.02386	0.0458637	0.6028979	0.6028979	0.5890162	9	FALSE	Qi et al., 2018	Brain
SKP2	ENSG00000145604	5	36168256	rs37220	5	36175290	G	C	0.93007	-0.0158	0.0201	0.4318267	0.389763	0.0700231	2.60E-08	-0.0405375	0.0520816	0.4363648	0.4363648	0.2559779	4	FALSE	Qi et al., 2018	Brain
TRIM4	ENSG00000146833	7	99502629	rs2527903	7	99524694	G	A	0.555944	0.0222	0.0095	0.0194474	0.461135	0.0490176	5.08E-21	0.0481421	0.0212274	0.02333406	0.02390056	0.312728	20	FALSE	Qi et al., 2018	Brain
ASB6	ENSG00000148331	9	132401807	rs2241246	9	132403241	T	A	0.393357	0.0002	0.0098	0.9837178	-0.320194	0.0471882	1.16E-11	-0.0006246	0.0306066	0.9837178	0.9850461	0.5984115	12	FALSE	Qi et al., 2018	Brain
PAK1	ENSG00000149269	11	77099867	rs3019253	11	77195995	C	T	0.743007	-0.0086	0.0138	0.5331607	0.317139	0.0453983	2.83E-12	-0.0271174	0.0436868	0.5347817	0.7955918	0.2568357	19	FALSE	Qi et al., 2018	Brain
ITGB1	ENSG00000150093	10	33241983	rs61856595	10	33086119	G	A	0.45979	0.0246	0.0102	0.01587552	-0.418884	0.0465332	2.22E-19	-0.0587274	0.0252092	0.01982713	0.01570693	0.8857836	20	FALSE	Qi et al., 2018	Brain
TIRAP	ENSG00000150455	11	126160850	rs659551	11	126076824	A	C	0.774476	0.012	0.0112	0.2839768	-0.548985	0.0489885	3.79E-29	-0.0218585	0.0204943	0.2861683	0.1137821	0.0656736	20	FALSE	Qi et al., 2018	Brain
UBE3B	ENSG00000151148	12	109944857	rs11067394	12	110033355	C	T	0.197552	-0.0208	0.0119	0.08048149	-0.351763	0.0478152	1.88E-13	0.0591308	0.0347714	0.08902571	0.03120092	0.08742926	20	FALSE	Qi et al., 2018	Brain
RASGRP3	ENSG00000152689	2	33725604	rs17013237	2	33762963	A	G	0.884615	0.0249	0.0171	0.1453538	-0.781178	0.0758197	6.82E-25	-0.0318749	0.0221076	0.1493554	0.3379007	0.9537392	20	FALSE	Qi et al., 2018	Brain
CD8A	ENSG00000153563	2	87023624	rs2944254	2	87014377	C	T	0.770979	0.0035	0.0115	0.7608629	0.31317	0.0486876	1.26E-10	0.011176	0.0367624	0.761122	0.8462379	0.9697345	20	FALSE	Qi et al., 2018	Brain
SH3RF1	ENSG00000154447	4	170103831	rs72706417	4	170006181	G	A	0.837413	0.0141	0.0131	0.2817771	-0.475912	0.0579948	2.28E-16	-0.0296273	0.0277619	0.2858837	0.8239963	0.9555305	20	FALSE	Qi et al., 2018	Brain
FBXL18	ENSG00000155034	7	5512197	rs10235653	7	5506906	C	T	0.933566	0.0094	0.0181	0.6035257	-0.483008	0.0800133	1.57E-09	-0.0194614	0.0376119	0.604859	0.8782578	0.5060797	20	FALSE	Qi et al., 2018	Brain
SEC13	ENSG00000157020	3	10348838	rs154236	3	10356314	T	C	0.645105	0.0122	0.0099	0.2178284	-0.269883	0.0430421	3.61E-10	-0.0452048	0.0373843	0.2265895	0.3126181	0.5969388	20	FALSE	Qi et al., 2018	Brain
COLEC12	ENSG00000158270	18	410041	rs2305027	18	334742	C	T	0.524476	-0.0022	0.0107	0.8370975	-0.250374	0.0399077	3.52E-10	0.00878685	0.042759	0.8371837	0.643498	0.5827363	20	FALSE	Qi et al., 2018	Brain
UBE2Z	ENSG00000159202	17	46996074	rs11650158	17	46959208	G	A	0.826923	-0.0248	0.0131	0.05834061	0.647194	0.0613514	5.14E-26	-0.0383193	0.0205646	0.06241142	0.03006231	0.7360167	20	FALSE	Qi et al., 2018	Brain
PSMD4	ENSG00000159352	1	151233567	rs1752385	1	151334407	A	G	0.765734	0.0045	0.0114	0.6930371	0.309968	0.0539516	9.18E-09	0.0145176	0.0368647	0.6937225	0.7773521	0.2399102	20	FALSE	Qi et al., 2018	Brain
PSMB4	ENSG00000159377	1	151373215	rs4246527	1	151403948	T	C	0.86014	-0.0139	0.0124	0.2623016	0.397664	0.0537029	1.31E-13	-0.0349542	0.0315374	0.267715	0.1793486	0.6460573	20	FALSE	Qi et al., 2018	Brain
THEM4	ENSG00000159445	1	151864172	rs2999506	1	151911780	C	G	0.632867	-0.0065	0.0098	0.5071607	0.518989	0.0377222	4.55E-43	-0.0125243	0.0189048	0.5076535	0.5000803	0.754199	20	FALSE	Qi et al., 2018	Brain
ITGB2	ENSG00000160255	21	46328886	rs7282310	21	46336166	G	C	0.804196	0.001	0.0142	0.9438574	-1.22089	0.0377278	9.85E-230	-0.0008191	0.0116309	0.9438575	0.8921807	0.7130974	5	FALSE	Qi et al., 2018	Brain
RCHY1	ENSG00000163743	4	76422110	rs7663549	4	76287541	G	T	0.496503	-0.004	0.0102	0.6949423	1.01615	0.0294469	6.10E-261	-0.0039364	0.0100385	0.694961	0.6205602	0.4529995	20	FALSE	Qi et al., 2018	Brain
KIF15	ENSG00000163808	3	44859038	rs17631716	3	44915603	C	T	0.715035	-0.0232	0.0103	0.02429529	0.356992	0.0539354	3.62E-11	-0.0649874	0.0304771	0.03297896	0.02444567	0.3104787	20	FALSE	Qi et al., 2018	Brain
RNF123	ENSG00000164068	3	49742947	rs3819325	3	49843723	T	C	0.515734	-0.0088	0.0094	0.3491856	-0.372394	0.038859	9.41E-22	0.0236309	0.0253622	0.3514735	0.1726373	0.1358789	20	FALSE	Qi et al., 2018	Brain
ASB5	ENSG00000164122	4	177166773	rs2046263	4	177151403	A	C	0.472028	-0.0074	0.0101	0.4637577	0.550547	0.048054	2.17E-30	-0.0134412	0.0183829	0.4646694	0.4257176	0.7781565	20	FALSE	Qi et al., 2018	Brain
ERAP1	ENSG00000164307	5	96120162	rs28432918	5	96101226	G	A	0.65035	0.0033	0.0101	0.7438701	1.21088	0.0326605	7.19E-301	0.0027253	0.0083414	0.7438797	0.3311764	0.7710368	20	FALSE	Qi et al., 2018	Brain
ERAP2	ENSG00000164308	5	96233531	rs2910686	5	96252589	T	C	0.557692	-0.0067	0.0094	0.4759906	-1.19931	0.032199	1.19E-303	0.00558653	0.00783925	0.4760713	0.4965276	0.02636245	20	FALSE	Qi et al., 2018	Brain
RAET1E	ENSG00000164520	6	150211874	rs2500542	6	149331204	C	T	0.309441	0.0073	0.0107	0.4950853	-0.320591	0.0584449	4.13E-08	-0.0227705	0.0336331	0.4983891	0.4523015	0.02401593	14	FALSE	Qi et al., 2018	Brain
CTSB	ENSG00000164733	8	11713495	rs4631423	8	11699279	C	T	0.823427	-0.0558	0.0119	2.74E-06	0.372278	0.0450667	1.45E-16	-0.149888	0.0367563	4.54E-05	5.76E-09	0.03058109	20	TRUE	Qi et al., 2018	Brain
YWHAZ	ENSG00000164924	8	101947184	rs3105460	8	101937374	G	A	0.615385	0.0072	0.0103	0.4845338	-0.582335	0.0479413	5.96E-34	-0.012364	0.0177167	0.4852557	0.2538884	0.3310276	20	FALSE	Qi et al., 2018	Brain
BTNL9	ENSG00000165810	5	180477874	rs4078420	5	180471823	A	G	0.477273	-0.0021	0.0094	0.8232209	-0.336346	0.0386147	3.03E-18	0.00624357	0.0279566	0.823278	0.8743032	0.4429184	20	FALSE	Qi et al., 2018	Brain

PSMC3	ENSG00000165916	11	47444156	rs61897424	11	47406065	C	T	0.853147	-0.0103	0.0134	0.4420971	0.502298	0.0721296	3.31E-12	-0.0205057	0.0268394	0.4448573	0.6675608	0.4745025	20	FALSE	Qi et al., 2018	Brain
AP1G1	ENSG00000166747	16	71803008	rs12927320	16	71675230	C	A	0.70979	0.02	0.0099	0.04336244	-0.403635	0.0512941	3.57E-15	-0.0495498	0.0253225	0.05037724	0.09014465	0.01207864	20	FALSE	Qi et al., 2018	Brain
YWHAB	ENSG00000166913	20	43525745	rs8356	20	43536455	C	T	0.718531	-0.0028	0.0108	0.7954352	1.25007	0.0336539	5.32E-302	-0.0022399	0.00863974	0.7954401	0.6550018	0.5269678	20	FALSE	Qi et al., 2018	Brain
STIM1	ENSG00000167323	11	3995098	rs3813879	11	3875982	G	T	0.281469	0.0045	0.0116	0.6980671	0.302798	0.0424538	9.86E-13	0.0148614	0.038366	0.6984908	0.5684568	0.3577816	17	FALSE	Qi et al., 2018	Brain
TUBA1C	ENSG00000167553	12	49624816	rs77888738	12	49512929	G	A	0.312937	0.0323	0.0105	0.00209664	0.962472	0.0422804	1.04E-114	0.0335594	0.0110086	0.00230001	0.001930488	0.00833916	20	FALSE	Qi et al., 2018	Brain
RILP	ENSG00000167705	17	1551407	rs7503846	17	1556270	C	G	0.977273	0.0228	0.0339	0.5012232	0.841317	0.126465	2.88E-11	0.0271004	0.0404994	0.5033964	0.725936	0.7838321	6	FALSE	Qi et al., 2018	Brain
MLST8	ENSG00000167965	16	2256833	rs26864	16	2253918	C	T	0.590909	0.0415	0.01	3.32E-05	-0.516563	0.0421322	1.48E-34	-0.0803388	0.0204377	8.46E-05	0.000155474	0.04666303	20	TRUE	Qi et al., 2018	Brain
INPP5D	ENSG00000168918	2	234020613	rs4663834	2	234118583	G	T	0.746503	-0.0044	0.0116	0.7044574	-0.309599	0.0547884	1.60E-08	0.0142119	0.0375522	0.7050899	0.7050899	0.9972283	9	FALSE	Qi et al., 2018	Brain
SIGLEC7	ENSG00000168995	19	51651169	rs12983058	19	51642784	G	T	0.681818	-0.0174	0.013	0.180746	-0.360933	0.0651589	3.04E-08	0.0482084	0.0370543	0.1932517	0.1932517			FALSE	Qi et al., 2018	Brain
CD14	ENSG00000170458	5	140012299	rs4912717	5	140018235	G	A	0.765734	0.0149	0.0105	0.1558851	-0.303303	0.0496208	9.81E-10	-0.0491258	0.0355396	0.166884	0.2108783	0.4290586	19	FALSE	Qi et al., 2018	Brain
PIK3CD	ENSG00000171608	1	9750481	rs4846192	1	9743729	A	G	0.484266	0.0332	0.0104	0.00141141	0.426201	0.0371466	1.79E-30	0.0778976	0.0253286	0.00210162	0.01775345	0.01611703	20	FALSE	Qi et al., 2018	Brain
FBXW10	ENSG00000171931	17	18664994	rs163367	17	18618644	A	G	0.215035	-0.0045	0.0133	0.7351026	0.443414	0.0771184	8.94E-09	-0.0101485	0.0300464	0.7355429	0.3857726	0.8354349	20	FALSE	Qi et al., 2018	Brain
AC006538.1	ENSG00000261342	19	2728533	rs4806874	19	2738352	A	G	0.685315	-0.0034	0.0111	0.7593714	-0.362966	0.0389474	1.17E-20	0.00936728	0.0305979	0.7594973	0.07995392	0.02340335	20	FALSE	Qi et al., 2018	Brain
CTSF	ENSG00000174080	11	66333623	rs7116940	11	66303895	T	C	0.777972	-0.0297	0.011	0.00693395	-0.425806	0.0566236	5.48E-14	0.06975	0.027448	0.01104826	0.001879057	0.1184297	18	FALSE	Qi et al., 2018	Brain
TLR6	ENSG00000174130	4	38841887	rs28392855	4	38925722	G	A	0.763986	0.0127	0.0112	0.2568245	0.294051	0.0527715	2.52E-08	0.0431898	0.0388693	0.2665019	0.2665019	0.5244669	17	FALSE	Qi et al., 2018	Brain
HRAS	ENSG00000174775	11	534764	rs61877775	11	521462	T	C	0.0926573	0.0176	0.0151	0.2437912	-0.867994	0.0711974	3.46E-34	-0.0202766	0.0174757	0.245938	0.01282247	0.5592613	20	FALSE	Qi et al., 2018	Brain
PSMD2	ENSG00000175166	3	184021669	rs34881379	3	184030387	T	C	0.527972	-0.0167	0.0094	0.07563478	0.455505	0.0483633	4.58E-21	-0.0366626	0.0210003	0.08084429	0.0350901	0.04898501	20	FALSE	Qi et al., 2018	Brain
TUBB6	ENSG00000176014	18	12325993	rs4073975	18	12279075	T	C	0.353147	0.0093	0.0111	0.4021218	-0.410417	0.0528212	7.85E-15	-0.0226599	0.0272024	0.4048397	0.5111237	0.8437164	13	FALSE	Qi et al., 2018	Brain
YES1	ENSG00000176105	18	767067	rs62091703	18	725062	C	T	0.856643	0.0241	0.0153	0.1152187	0.72925	0.0737866	4.92E-23	0.0330476	0.0212452	0.1198196	0.1039925	0.1589655	20	FALSE	Qi et al., 2018	Brain
CDC26	ENSG00000176386	9	116027992	rs10981712	9	116046126	G	C	0.715035	-0.0075	0.0103	0.4665185	0.519709	0.0544195	1.30E-21	-0.0144311	0.0198763	0.4678094	0.6279718	0.8036276	20	FALSE	Qi et al., 2018	Brain
ARIH2	ENSG00000177479	3	48990034	rs7653459	3	48838267	G	A	0.662587	0.0588	0.0099	2.86E-09	0.332583	0.0410231	5.18E-16	0.176798	0.0369005	1.66E-06	0.000336241	3.58E-06	20	FALSE	Qi et al., 2018	Brain
ASB8	ENSG00000177981	12	48558283	rs10875746	12	48518264	A	C	0.765734	0.0164	0.0111	0.1395477	-0.464662	0.0469298	4.11E-23	-0.0352945	0.0241528	0.1439338	0.04837174	0.466721	20	FALSE	Qi et al., 2018	Brain
RNF182	ENSG00000180537	6	13952605	rs6930784	6	13995770	T	C	0.484266	0.0025	0.0098	0.7986443	-0.258288	0.0437054	3.43E-09	-0.0096791	0.0379775	0.7988276	0.7988276	0.9039447	9	FALSE	Qi et al., 2018	Brain
UBE2E2	ENSG00000182247	3	23438897	rs68093882	3	23600664	A	C	0.762238	-0.0126	0.0115	0.273231	0.349835	0.0452535	1.07E-14	-0.0360169	0.0332011	0.2780054	0.2939114	0.7769718	20	FALSE	Qi et al., 2018	Brain
CLEC4G	ENSG00000182566	19	7796317	rs4804798	19	7799057	C	G	0.923077	0.0168	0.0213	0.4302684	-0.619698	0.0736844	4.09E-17	-0.02711	0.0345224	0.4322856	0.4768129	0.3970025	18	FALSE	Qi et al., 2018	Brain
AP2A2	ENSG00000183020	11	968566	rs66670048	11	999764	T	C	0.905594	-0.0131	0.0178	0.461758	0.65027	0.0691272	5.11E-21	-0.0201455	0.0274569	0.4631237	0.918838	0.1335585	7	FALSE	Qi et al., 2018	Brain
UBE2F	ENSG00000184182	2	238913352	rs12615308	2	238903399	G	A	0.847902	-0.0209	0.0152	0.1691314	0.346008	0.0530414	6.88E-11	-0.0604033	0.0448949	0.1784842	0.4562855	0.04922426	20	FALSE	Qi et al., 2018	Brain
UBE2G2	ENSG00000184787	21	46205444	rs11701732	21	46239427	C	T	0.666084	0.0203	0.0111	0.06742525	0.341463	0.0415162	1.96E-16	0.0594502	0.0333011	0.07422422	0.1321807	0.8875338	20	FALSE	Qi et al., 2018	Brain
UBOX5	ENSG00000185019	20	3114530	rs306757	20	3119133	G	A	0.701049	-0.0189	0.0102	0.06389079	-0.423569	0.0392957	4.33E-27	0.0446209	0.0244343	0.06782682	0.08108948	0.09059882	15	FALSE	Qi et al., 2018	Brain
PRKG1	ENSG00000185532	10	53404527	rs9415733	10	52914107	G	C	0.517483	-0.0154	0.0096	0.1086773	0.477879	0.0456401	1.18E-25	-0.0322257	0.0203232	0.1128153	0.09888928	0.6949279	14	FALSE	Qi et al., 2018	Brain
PSMD13	ENSG00000185627	11	244764	rs9795476	11	235894	G	T	0.723776	-0.0132	0.0108	0.2216236	-1.17146	0.0307323	0	0.011268	0.00922398	0.221861	0.1930236	0.1038821	20	FALSE	Qi et al., 2018	Brain
TRIM69	ENSG00000185880	15	45040606	rs17588305	15	45021766	C	T	0.877622	0.0153	0.0156	0.3267066	1.55486	0.0421733	1.49E-297	0.00984013	0.0100366	0.3268777	0.6179281	0.8739852	20	FALSE	Qi et al., 2018	Brain
IFITM1	ENSG00000185885	11	314389	rs10902124	11	326317	C	G	0.552448	-0.0113	0.0109	0.299877	-0.225729	0.039537	1.13E-08	0.05006	0.0490776	0.30772	0.2091014	0.873485	13	FALSE	Qi et al., 2018	Brain
BTN3A2	ENSG00000186470	6	26371966	rs9366653	6	26354247	A	G	0.117133	0.012	0.0154	0.4358497	-1.72349	0.0451537	0	-0.0069626	0.00893723	0.4359453	0.01055042	0.04685832	20	FALSE	Qi et al., 2018	Brain
SELL	ENSG00000188404	1	169670323	rs12404085	1	169616386	T	A	0.25	0.0035	0.0136	0.7969063	-0.391678	0.0542764	5.34E-13	-0.0089359	0.0347445	0.7970325	0.8582735	0.8372406	20	FALSE	Qi et al., 2018	Brain
HLA-DRB1	ENSG00000196126	6	32552085	rs9271069	6	32575700	G	A	0.868881	-0.0576	0.0153	0.00016675	-1.54698	0.0380237	0	0.0372338	0.00993248	0.00017776	5.47E-11	0.00279129	20	TRUE	Qi et al., 2018	Brain
ASB13	ENSG00000196372	10	5694799	rs7899908	10	5686473	A	T	0.812937	-0.0249	0.0131	0.05733302	1.40483	0.0399279	3.50E-271	-0.0177246	0.00933859	0.05769665	0.3401414	0.3878336	20	FALSE	Qi et al., 2018	Brain
HLA-DQA1	ENSG00000196735	6	32605397	rs1063355	6	32627714	G	T	0.606643	-0.0439	0.0108	4.81E-05	-1.0961	0.0383449	1.02E-179	0.040051	0.00995221	5.71E-05	3.66E-09	1.56E-05	20	TRUE	Qi et al., 2018	Brain
ATG7	ENSG00000197548	3	11456567	rs346083	3	11280017	C	T	0.681818	0.0202	0.0107	0.05904603	0.303638	0.0404439	6.02E-14	0.0665266	0.0363364	0.06712229	0.09374884	0.2903064	20	FALSE	Qi et al., 2018	Brain
PDCD1LG2	ENSG00000197646	9	5540913	rs16923189	9	5510644	A	G	0.667832	0.0082	0.011	0.455997	-0.502461	0.0859375	5.01E-09	-0.0163197	0.0220695	0.4596225	0.2554057	0.2636125	20	FALSE	Qi et al., 2018	Brain
PLCG2	ENSG00000197943	16	81882300	rs4420523	16	81926780	A	C	0.636364	0.0157	0.0108	0.1460285	-0.315741	0.0519699	1.24E-09	-0.0497242	0.0351708	0.157422	0.1846142	0.7278994	20	FALSE	Qi et al., 2018	Brain
DNM3	ENSG00000197959	1	172099113	rs9425308	1	172383265	A	C	0.305944	-0.0261	0.0103	0.0112775	-0.296366	0.0447271	3.45E-11	0.0880668	0.037209	0.01794201	0.02042339	0.2218689	19	FALSE	Qi et al., 2018	Brain
HLA-DRB5	ENSG00000198502	6	32491592	rs9271069	6	32575700	G	A	0.868881	-0.0576	0.0153	0.00016675	-1.61449	0.0424892	0	0.035677	0.0095231	0.00017942	5.41E-06	2.92E-05	20	TRUE	Qi et al., 2018	Brain
SMURF1	ENSG00000198742	7	98683392	rs9297145	7	98759117	A	C	0.70979	-0.0033	0.0113	0.7702596	0.254085	0.0401537	2.49E-10	-0.0129878	0.0445207	0.770497	0.8051069	0.5246953	16	FALSE	Qi et al., 2018	Brain
MTOR	ENSG00000198793	1	11244578	rs7540890	1	11278289	T	C	0.263986	0.0199	0.0109	0.06789726	0.239052	0.041922	1.18E-08	0.0832455	0.0478767	0.08207958	0.08207958	0.7566068	20	FALSE	Qi et al., 2018	Brain
HLA-DMA	ENSG00000204257	6	32927441	rs116131925	6	32929943	T	A	0.0576923	0.0138	0.019	0.4676452	1.14457	0.106159	4.21E-27	0.012057	0.0166378	0.4686525	0.05818533	0.1127377	20	FALSE	Qi et al., 2018	Brain
TAP2	ENSG00000204267	6	32798083	rs17034	6	32796521	T	C	0.248252	0.0247	0.0108	0.02219366	-0.78471	0.0522664	5.98E-51	-0.0314766	0.0139218	0.02376203	0.02303057	0.1516401	20	FALSE	Qi et al., 2018	Brain
MICB	ENSG00000204516	6	31470779	rs2516412	6	31462321	T	G	0.277972	-0.0228	0.0105	0.02989879	0.826847	0.084155	8.76E-23	-0.0275746	0.0130053	0.03398362	0.02886158	0.1803319	20	FALSE	Qi et al., 2018	Brain
HLA-C	ENSG00000204525	6	31238216	rs2523594	6	31326629	C	T	0.325175	0.0338	0.0107	0.00158378	1.0899	0.0287086	0	0.031012	0.00985132	0.00164392	0.000586471	0.08892532	20	FALSE	Qi et al., 2018	Brain
HLA-G	ENSG00000204632	6	29796823	rs147947139	6	29912938	A	C	0.111888	-0.0266	0.0193	0.1681297	1.26301	0.123553	1.57E-24	-0.0210608	0.0154192	0.1719766	0.2109599	0.664218	9	FALSE	Qi et al., 2018	Brain
HLA-B	ENSG00000234745	6	31323307	rs2523591	6	31326960	G	A	0.564685	0.0115	0.0105	0.2734123	0.461679	0.0394813	1.37E-31	0.0249091	0.0228426	0.2755081	0.05435522	0.02683943	20	FALSE	Qi et al., 2018	Brain
HLA-A	ENSG00000206503	6	29911349	rs2735061	6	29693938	G	A	0.818182	-0.0167	0.0125	0.1815493	0.628189	0.0675609	1.43E-20	-0.0265843	0.0201028	0.1860287	0.01438524	0.00087432	9	FALSE	Qi et al., 2018	Brain
LAT	ENSG00000213658	16	28999125	rs11645306	16	28983936	C	G	0.753497	0.0631	0.0104	1.30E-09	-0.472818	0.0518374	7.43E-20	-0.133455	0.0264176	4.38E-07	6.32E-07	0.7740546	20	TRUE	Qi et al., 2018	Brain
UBA52	ENSG00000221983	19	18685450	rs10409392	19	18699333	A	C	0.458042	0.002	0.0097	0.836646	0.322222	0.0488456	4.20E-11	0.0062069	0.0301182	0.8367246	0.9599301	0.3329377	20	FALSE	Qi et al., 2018	Brain
HLA-DPB1	ENSG00000223865	6	33049340	rs116108798	6	33029925	G	A	0.199301	0.0087	0.0133	0.5130246	-0.593207	0.106715	2.72E-08	-0.014666	0.0225752	0.5159165	0.5159165	0.1641974	16	FALSE	Qi et al., 2018	Brain
HLA-DPA1	ENSG00000231389	6	33040449	rs116108798	6	33029925	G	A	0.199301	0.0087	0.0133	0.5130246	-0.678817	0.0667456	2.69E-24	-0.0128164	0.0196334	0.5138939	0.5885956	0.2828368	20	FALSE	Qi et al., 2018	Brain
HLA-DQB2	ENSG00000232629	6	32727593	rs35779483	6	32626702	G	A	0.332168	-0.0001	0.0114	0.9930011	0.712475	0.103623	6.17E-12	-0.0001404	0.0160006	0.9930011	0.001369855	0.00037765	20	FALSE	Qi et al., 2018	Brain
HLA-DQA2	ENSG00000237541	6	32712055	rs17191234	6	32564681	C	A	0.372378	-0.099	0.0148	2.24E-11	0.711076	0.0999538	1.13E-12	-0.139226	0.0285694	1.10E-06	1.03E-06	0.05320906	20	TRUE	Qi et al., 2018	Brain
KLHL41	ENSG00000239474	2	170374492	rs79810047	2	170327756	T	G	0.0576923	0.0122	0.0222	0.5826284	1.00477	0.113449	8.25E-19	0.0121421	0.0221371	0.5833523	0.9084537	0.7704049	20	FALSE	Qi et al., 2018	Brain
PSMB9	ENSG00000240065	6	32829638	rs2071480	6	32821859	A	C	0.428322	-0.0081	0.0097	0.4036887	0.312405	0.0505263	6.29E-10	-0.0259279	0.0313313	0.4079319	0.4820694	0.1659977	12	FALSE	Qi et al., 2018	Brain
HLA-DOB	ENSG00000241106	6	32780894	rs2071473	6	32782605	T	C	0.340909	0.0131	0.01	0.1901958	-0.705247	0.0924506	2.38E-14	-0.0185751	0.014387	0.1966687	0.01426934	0.04909922	17	FALSE	Qi et al., 2018	Brain
HLA-DMB	ENSG00000242574	6	32905626	rs116131925	6	32929943	T	A	0.0576923	0.0138	0.019	0.4676452	0.79222	0.10945	4.55E-13	0.0174194	0.0241037	0.4698725	0.3904463	0.1638978	13	FALSE	Qi et al., 2018	Brain
KCTD7	ENSG00000243335	7	66185157	rs6957690	7	65582092	T	G	0.597902	0.0253	0.0096	0.00840341	0.413473	0.0468923	1.17E-18	0.061189	0.0242328	0.01156833	0.008633479	0.00485579	15	FALSE	Qi et al., 2018	Brain
UBE2V1	ENSG00000244687	20	48715078	rs1000595	20	48692455	G	A	0.596154	0.0072	0.0103	0.4845338	0.259816	0.0437809	2.95E-09	0.0277119	0.0399176	0.4875383	0.4875383	0.2917798	15	FALSE	Qi et al., 2018	Brain
LYN	ENSG00000254087	8	56858156	rs7005312	8	56806439	C	T	0.512238	-0.0087	0.01	0.3843004	0.289778	0.0454086	1.75E-10	-0.030023	0.0348284	0.3886732	0.3966343	0.8183085	20	FALSE	Qi et al., 2018	Brain
SIGLEC12	ENSG00000254521	19	51999827	rs3810108	19	52004469	C	T	0.368881	-0.0124	0.0097	0.2011259	0.703204	0.116265	1.46E-09	-0.0176336	0.0140988	0.2110368	0.2110368	0.321669	20	FALSE	Qi et al., 2018	Brain
CTSO	ENSG00000256043	4	156860169	rs2878120	4	156909831	C	T	0.702797	-0.0174	0.011	0.1136911	0.34158	0.0432167	2.70E-15	-0.0509397	0.0328418	0.120887	0.1593738	0.1756641	20	FALSE	Qi et al., 2018	Brain
MRC1	ENSG00000120586	10	18149221	rs722381	10	18231002	A	G	0.662587	-0.0146	0.0129	0.2577257	-0.529722	0.0923589	9.72E-09	0.0275616	0.024822	0.2668392	0.2668392	0.4378614	9	FALSE	Qi et al., 2018	Brain
GAN	ENSG00000127688	16	81381248	rs8051314	16	81387476	C	T	0.479021	-0.0049	0.01	0.6241339	0.526582	0.0607412	4.35E-18	-0.0093053	0.0190207	0.6246865	0.802839	0.3450836	20	FALSE	Qi et al., 2018	Brain
DYNLL2	ENSG00000264364	17	56170903	rs10132	17	56168371	G	A	0.230769	-0.0072	0.0118	0.5417495	-0.336308	0.0549146	9.11E-10	0.0214089	0.0352606	0.5437425	0.619949	0.284745	4	FALSE	Qi et al., 2018	Brain
RNF115	ENSG00000121848	1	145650848	rs17354678	1	145689271	G	A	0.365385	-0.0171	0.0103	0.0968754	0.217668	0.038723	1.90E-08	-0.0785602	0.0493406	0.1113394	0.1113394	0.1195154	9	FALSE	Qi et al., 2018	Brain
FBXO17	ENSG00000269190	19	39449297	rs4803046	19	39416967	A	T	0.756993	-0.0062	0.014	0.6578691	0.381763	0.0423215	1.87E-19	-0.0162404	0.0367161	0.6582545	0.413345	0.2112557	20	FALSE	Qi et al., 2018	Brain
ICAM3	ENSG00000076662.5	19	10447475	rs1058154	19	10446897	A	C	0.265734	-0.0014	0.0136	0.9180097	-0.904795	0.128033	1.58E-12	0.00154731	0.0150326	0.9180183	0.9180183			FALSE	GTEx Consortium	Substantia Nigra
RAPGEF3	ENSG00000079337.11	12	48146639	rs12422983	12	48142636	T	C	0.145105	-0.023	0.0131	0.07913534	0.818048	0.136803	2.23E-09	-0.0281157	0.0166897	0.09206324	0.09206324	0.2245885	9	FALSE	GTEx Consortium	Substantia Nigra
PILRA	ENSG00000085514.11	7	99984393	rs6948685	7	99970188	T	C	0.176573	-0.0409	0.0122	0.00080097	0.736299	0.119707	7.71E-10	-0.0555481	0.0188707	0.00324398	0.001150626	0.00772593	5	TRUE	GTEx Consortium	Substantia Nigra
FBXO7	ENSG00000100225.13	22	32882740	rs1029302	22	32888622	G	A	0.578671	0.0065	0.0099	0.5114602	0.414828	0.0724663	1.04E-08	0.0156691	0.0240218	0.5142146	0.5142146	0.6787736	3	FALSE	GTEx Consortium	Substantia Nigra
MGRN1	ENSG00000102858.8	16	4703734	rs841225	16	4736316	T	C	0.554196	0.0011	0.0097	0.9097118	0.937136	0.0975643	7.59E-22	0.00117379	0.0103514	0.9097181	0.4097988	0.0215649	20	FALSE	GTEx Consortium	Substantia Nigra
SPSB2	ENSG00000111671.5	12	6989317	rs2269358	12	6993151	T	G	0.333916	0.0189	0.0107	0.07733629	0.999624	0.128206	6.34E-15	0.0189071	0.0109753	0.08494232	0.1319524	0.4735075	14	FALSE	GTEx Consortium	Substantia Nigra
PILRB	ENSG00000121716.14	7	99957577	rs10085549	7	99983135	T	C	0.176573	-0.0388	0.0123	0.00160789	0.977526	0.12193	1.08E-15	-0.039692	0.0135218	0.00333098	0.000241963	0.03102275	9	TRUE	GTEx Consortium	Substantia Nigra
SFTPD	ENSG00000133661.11	10	81719933	rs2819121	10	81732040	T	G	0.368881	0.0002	0.0099	0.9838822	0.718246	0.1213	3.20E-09	0.00027846	0.0137837	0.9838823	0.9692752	0.6247123	7	FALSE	GTEx Consortium	Substantia Nigra
ASB5	ENSG00000164122.4	4	177166773	rs12645044	4	177168437	T	G	0.465035	-0.0105	0.0102	0.3032862	0.690789	0.122391	1.66E-08	-0.0152	0.0150093	0.3112007	0.3112007	0.697975	4	FALSE	GTEx Consortium	Substantia Nigra
ERAP1	ENSG00000164307.8	5	96120162	rs7063	5	96110211	T	A	0.340909	0.0112	0.0125	0.3702528	-0.661295	0.104177	2.18E-10	-0.0169365	0.0190897	0.3749684	0.7837613	0.02232852	8	FALSE	GTEx Consortium	Substantia Nigra
ERAP2	ENSG00000164308.12	5	96233531	rs2910686	5	96252589	C	T	0.442308	0.0067	0.0094	0.4759906	1.24262	0.107509	6.71E-31	0.00539183	0.00757903	0.4768274	0.6748492	0.2271651	11	FALSE	GTEx Consortium	Substantia Nigra
PSMD13	ENSG00000185627.13	11	244763	rs6421986	11	221659	G	A	0.722028	-0.0115	0.0106	0.2779634	-0.453857	0.0709068	1.55E-10	0.0253384	0.0236885	0.2847771	0.2367658	0.20194	10	FALSE	GTEx Consortium	Substantia Nigra
BTN3A2	ENSG00000186470.9	6	26371966	rs28551159	6	26376368	G	A	0.117133	0.0093	0.0164	0.5706645	-1.15553	0.189101	9.92E-10	-0.0080483	0.0142536	0.5723139	0.3661756	0.00407343	5	FALSE	GTEx Consortium	Substantia Nigra
HLA-DRB1	ENSG00000196126.6	6	32552085	rs1129735	6	32605295	T	C	0.377622	-0.0783	0.014	2.23E-08	-0.406036	0.0661304	8.26E-10	0.19284	0.0466399	3.55E-05	1.63E-05	0.1767461	12	TRUE	GTEx Consortium	Substantia Nigra
HLA-DRB5	ENSG00000198502.5	6	32491592	rs9271152	6	32577531	G	T	0.858392	-0.0675	0.0146	3.78E-06	-0.98767	0.130984	4.68E-14	0.0683427	0.0173396	8.10E-05	2.38E-05	0.1896288	20	TRUE	GTEx Consortium	Substantia Nigra
HLA-C	ENSG00000204525.10	6	31238204	rs2524119	6	31229404	C	T	0.589161	-0.0446	0.0116	0.00012064	-0.79625	0.118153	1.59E-11	0.0560126	0.0167725	0.00083915	0.01254101	0.02247246	20	FALSE	GTEx Consortium	Substantia Nigra
HLA-DQB2	ENSG00000232629.4	6	32727593	rs17612625	6	32615702	C	A	0.583916	-0.0492	0.0135	0.00026797	0.838845	0.108903	1.33E-14	-0.0586521	0.017804	0.00098662	3.68E-05	0.01378545	20	TRUE	GTEx Consortium	Substantia Nigra
HLA-DQA2	ENSG00000237541.3	6	32712055	rs9271488	6	32589000	T	G	0.328671	-0.0852	0.0167	3.36E-07	0.876459	0.129933	1.53E-11	-0.0972093	0.02389	4.72E-05	2.19E-07	0.05983926	20	TRUE	GTEx Consortium	Substantia Nigra

HLA-DOB	ENSG00000241106.2	6	32782682	rs4148876	6	32796793	A	G	0.0769231	-0.1002	0.019	1.34E-07	1.81732	0.327879	2.98E-08	-0.0551361	0.0144312	0.00013312	0.000133123	0.00538909	10	TRUE	GTEx Consortium	Substantia Nigra
FBXL3	ENSG00000005812	13	77584035	rs149794611	13	77925107	T	C	0.013986	0.1065	0.0528	0.04369077	0.373791	0.0580908	1.24E-10	0.284918	0.148033	0.05426715	0.05426715			FALSE	Vosa et al., 2018	Blood
ITGAL	ENSG00000005844	16	30509242	rs11574938	16	30485393	G	C	0.428322	-0.0272	0.0098	0.00551152	0.225275	0.00899569	2.10E-138	-0.120741	0.0437687	0.00580462	1.68E-07	0.05904939	20	TRUE	Vosa et al., 2018	Blood
MAP3K14	ENSG00000006062	17	43367451	rs4792847	17	43383484	G	A	0.531469	0.033	0.0094	0.00044703	0.0726592	0.00919992	2.84E-15	0.454175	0.141576	0.00133668	0.000661151	0.00161434	5	FALSE	Vosa et al., 2018	Blood
AP2B1	ENSG00000006125	17	33979250	rs321600	17	33905468	A	G	0.232517	0.0131	0.0116	0.2587669	-0.272291	0.0127332	1.87E-101	-0.0481103	0.0426609	0.2594302	0.01519758	0.00318242	20	FALSE	Vosa et al., 2018	Blood
CD79B	ENSG00000007312	17	62007907	rs4968674	17	62015048	T	G	0.388112	0.0002	0.0099	0.9838822	-0.0970458	0.00972344	1.85E-23	-0.0020609	0.102014	0.9838822	0.6595333	0.628415	20	FALSE	Vosa et al., 2018	Blood
MYLIP	ENSG00000007944	6	16138917	rs2021900	6	15900330	C	T	0.0664336	-0.0248	0.0182	0.1729968	0.264804	0.011843	9.74E-111	-0.093654	0.0688575	0.1737938	0.8389421	0.3022516	20	FALSE	Vosa et al., 2018	Blood
PSMB1	ENSG00000008018	6	170853317	rs4710839	6	170862051	C	G	0.405594	0.0042	0.0098	0.6682351	0.373619	0.00862837	0	0.0112414	0.0262312	0.6682504	0.6865953	0.9597619	20	FALSE	Vosa et al., 2018	Blood
UBE3C	ENSG00000009335	7	156996836	rs7807	7	157061642	C	A	0.229021	-0.0181	0.0118	0.1250547	-0.403111	0.00952672	0	0.0449008	0.0292915	0.1253026	0.6197804	0.9189999	20	FALSE	Vosa et al., 2018	Blood
CD4	ENSG00000010610	12	6912999	rs7296859	12	6894164	C	G	0.361888	-0.0168	0.0108	0.1198138	0.259706	0.0100973	6.91E-146	-0.0646886	0.0416615	0.1204904	0.5778693	0.7621564	14	FALSE	Vosa et al., 2018	Blood
FYN	ENSG00000010810	6	112088095	rs79756992	6	112039479	C	T	0.0472028	-0.0124	0.0239	0.6038804	-0.187955	0.0201547	1.10E-20	0.0659733	0.127355	0.6044392	0.001946241	7.18E-07	20	FALSE	Vosa et al., 2018	Blood
TYROBP	ENSG00000011600	19	36397257	rs4806227	19	36401012	A	G	0.0297203	0.0216	0.025	0.3875879	-0.250288	0.0139324	3.70E-72	-0.0863007	0.100001	0.3881361	0.116834	0.2197788	20	FALSE	Vosa et al., 2018	Blood
CD22	ENSG00000012124	19	35824211	rs12327820	19	35814384	C	A	0.134615	0.0208	0.015	0.1655434	0.20008	0.0123608	6.27E-59	0.103958	0.0752446	0.1670927	0.1522676	0.9516308	20	FALSE	Vosa et al., 2018	Blood
RNF14	ENSG00000013561	5	141353874	rs403075	5	141374794	T	C	0.447552	-0.0235	0.0099	0.01760907	0.501702	0.00867796	0	-0.0468406	0.0197495	0.01770459	0.007458864	0.3256792	20	FALSE	Vosa et al., 2018	Blood
UFL1	ENSG00000014123	6	96986311	rs9386528	6	96928514	A	G	0.323427	-0.0099	0.0104	0.341136	0.191505	0.00982996	1.57E-84	-0.0516957	0.0543714	0.3417116	0.5765074	0.4874256	20	FALSE	Vosa et al., 2018	Blood
UBR2	ENSG00000024048	6	42596521	rs12191298	6	42537608	G	A	0.0769231	-0.0029	0.0215	0.8927038	-0.309982	0.0256739	1.45E-33	0.00935539	0.0693632	0.8927105	0.996855	0.9587476	14	FALSE	Vosa et al., 2018	Blood
SLAMF7	ENSG00000026751	1	160716824	rs489286	1	160722550	A	G	0.347902	-0.0048	0.0103	0.6412016	-0.199493	0.00959499	5.18E-96	0.024061	0.0516438	0.6412854	0.1903995	0.1402248	20	FALSE	Vosa et al., 2018	Blood
BTN3A1	ENSG00000026950	6	26408954	rs4320356	6	26423560	T	C	0.428322	0.032	0.0096	0.00085812	0.364505	0.00864711	0	0.0877902	0.0264193	0.00089068	1.34E-05	0.00165471	20	TRUE	Vosa et al., 2018	Blood
UBA6	ENSG00000033178	4	68524188	rs28510080	4	68493603	C	T	0.461538	-0.0036	0.01	0.7188471	0.12036	0.00935209	6.66E-38	-0.0299104	0.0831169	0.7189524	0.5515683	0.3575772	16	FALSE	Vosa et al., 2018	Blood
RNF19A	ENSG00000034677	8	101308867	rs7842976	8	101453407	C	T	0.781469	0.0047	0.0133	0.723801	-0.0759511	0.00930249	3.23E-16	-0.0618819	0.175277	0.7240488	0.2855679	0.1386747	15	FALSE	Vosa et al., 2018	Blood
CUL3	ENSG00000036257	2	225392488	rs3768897	2	225338022	A	G	0.125874	0.0154	0.0141	0.2747458	0.17193	0.0111939	3.07E-53	0.0895715	0.0822173	0.2759566	0.4049131	0.03793907	20	FALSE	Vosa et al., 2018	Blood
CDH1	ENSG00000039068	16	68820286	rs11642362	16	68811307	T	C	0.433566	-0.0008	0.0096	0.9335865	-0.315847	0.00872182	3.75E-287	0.00253288	0.0303946	0.9335867	0.9795313	0.8539883	20	FALSE	Vosa et al., 2018	Blood
PSMA4	ENSG00000041357	15	78837175	rs931794	15	78826180	G	A	0.393357	0.0019	0.0098	0.8462718	0.19025	0.00954438	2.10E-88	0.00998686	0.0515136	0.8462789	0.2968888	0.1556868	20	FALSE	Vosa et al., 2018	Blood
AP2S1	ENSG00000042753	19	47347821	rs311379	19	47441846	C	G	0.566434	-0.0194	0.0095	0.04114109	0.162911	0.00895234	5.39E-74	-0.119083	0.05868	0.04242089	0.08909712	0.193584	20	FALSE	Vosa et al., 2018	Blood
LCP2	ENSG00000043462	5	169699236	rs12652307	5	169735262	C	G	0.164336	0.008	0.0176	0.6494363	0.172862	0.0103478	1.20E-62	0.0462798	0.101853	0.6495573	0.7081154	0.5542662	12	FALSE	Vosa et al., 2018	Blood
CUL7	ENSG00000044090	6	43013519	rs2273918	6	43013662	C	T	0.0506993	-0.0115	0.0186	0.5363911	-0.321513	0.0120344	3.05E-157	0.0357684	0.057867	0.5365001	0.2551128	0.3539148	20	FALSE	Vosa et al., 2018	Blood
HSPA5	ENSG00000044574	9	128000370	rs2416962	9	128118864	G	A	0.0192308	-0.0288	0.0399	0.4704147	0.855496	0.0505995	3.98E-64	-0.0336647	0.0466821	0.4708187	0.256575	0.4429144	13	FALSE	Vosa et al., 2018	Blood
VAMP3	ENSG00000049245	1	7836410	rs11590868	1	7833951	G	T	0.176573	0.021	0.0122	0.08519432	0.484979	0.0142429	3.97E-254	0.0433008	0.0251878	0.08559319	0.4524107	0.3088894	20	FALSE	Vosa et al., 2018	Blood
NEDD4L	ENSG00000049759	18	55890185	rs2288774	18	55983330	C	T	0.498252	0.0063	0.01	0.5286946	-0.157312	0.00889675	5.77E-70	-0.0400477	0.0636081	0.528956	0.5829202	0.8856041	20	FALSE	Vosa et al., 2018	Blood
CYBA	ENSG00000051523	16	88713625	rs3794624	16	88717074	A	G	0.335664	0.0069	0.0102	0.4987419	-0.206681	0.0104678	8.96E-87	-0.0333847	0.0493803	0.4989937	0.5879835	0.8022082	20	FALSE	Vosa et al., 2018	Blood
ANAPC4	ENSG00000053900	4	25399477	rs1533133	4	25384305	C	T	0.526224	0.0111	0.0095	0.242637	-0.576174	0.00854511	0	-0.019265	0.0164905	0.2427077	0.2574084	0.3029508	20	FALSE	Vosa et al., 2018	Blood
CUL1	ENSG00000055130	7	148446567	rs243495	7	148441400	T	G	0.38986	0.0082	0.0105	0.4348305	-0.0909579	0.00902718	7.05E-24	-0.0901516	0.115784	0.4362052	0.2867451	0.163409	20	FALSE	Vosa et al., 2018	Blood
TAB2	ENSG00000055208	6	149636263	rs62425952	6	149563894	T	C	0.164336	0.0151	0.0131	0.2490451	-0.147923	0.0107918	9.22E-43	-0.10208	0.0888721	0.2507137	0.1511637	0.3856774	20	FALSE	Vosa et al., 2018	Blood
RNF4	ENSG00000063978	4	2545497	rs10004477	4	2477281	G	A	0.405594	-0.0067	0.0101	0.507096	-0.155884	0.00946206	5.59E-61	0.0429808	0.0648444	0.5074399	0.03734855	0.01785311	20	FALSE	Vosa et al., 2018	Blood
CTSA	ENSG00000064601	20	44523525	rs6017717	20	44603963	C	G	0.138112	-0.0006	0.0132	0.963745	0.133923	0.0102996	1.18E-38	-0.0044802	0.0985645	0.9637452	0.1311072	0.6176353	20	FALSE	Vosa et al., 2018	Blood
BTBD1	ENSG00000064726	15	83710640	rs1986102	15	83758884	T	C	0.318182	-0.0209	0.0103	0.04244544	0.128693	0.00903314	4.69E-46	-0.162402	0.080843	0.04455269	0.07754066	0.0484277	20	FALSE	Vosa et al., 2018	Blood
COL17A1	ENSG00000065618	10	105818402	rs10883958	10	105736284	G	A	0.185315	-0.016	0.0121	0.1860636	0.205467	0.0103916	5.15E-87	-0.0778715	0.0590219	0.1870465	0.575349	0.2209684	20	FALSE	Vosa et al., 2018	Blood
SEC61A2	ENSG00000065665	10	12191798	rs876089	10	12206193	G	T	0.506993	0.0067	0.0094	0.4759906	-0.38627	0.00860923	0	-0.0173454	0.0243383	0.4760463	0.8962416	0.1775498	20	FALSE	Vosa et al., 2018	Blood
PRKCQ	ENSG00000065675	10	6545684	rs2255088	10	6625378	C	T	0.321678	-0.0033	0.0109	0.7620786	-0.171391	0.00904469	4.47E-80	0.0192542	0.0636052	0.7621081	0.6947417	0.4356083	15	FALSE	Vosa et al., 2018	Blood
ASB1	ENSG00000065802	2	239348137	rs10929275	2	239323169	G	A	0.59965	-0.0367	0.0095	0.00011193	0.459047	0.00864674	0	-0.0799483	0.0207498	0.00011669	0.003172486	0.6380923	20	FALSE	Vosa et al., 2018	Blood
PPP2R5A	ENSG00000066027	1	212497039	rs17018853	1	212431200	T	A	0.141608	0.0224	0.0128	0.08011831	0.815219	0.00973036	0	0.0274773	0.0157047	0.08018419	0.006923378	0.2439689	20	FALSE	Vosa et al., 2018	Blood
KIF26A	ENSG00000066735	14	104626145	rs35797471	14	104621383	C	T	0.477273	0.0286	0.0129	0.02661938	-0.30015	0.00882112	9.09E-254	-0.0952856	0.0430696	0.02694159	0.007155438	0.1058412	20	FALSE	Vosa et al., 2018	Blood
KIF2A	ENSG00000068796	5	61717532	rs12659814	5	61569169	T	C	0.494755	0.0149	0.0096	0.1206423	-0.147817	0.00925246	1.88E-57	-0.1008	0.0652508	0.1223922	0.5217932	0.134323	20	FALSE	Vosa et al., 2018	Blood
PSME4	ENSG00000068878	2	54144590	rs2293360	2	54196772	G	T	0.0821678	0.0111	0.0166	0.503703	-0.29638	0.012118	4.16E-132	-0.0374519	0.0560301	0.5038624	0.2694594	0.3648188	20	FALSE	Vosa et al., 2018	Blood
PPP2R5B	ENSG00000068971	11	64693486	rs58717149	11	64781891	G	T	0.0541958	0.0125	0.0259	0.6293617	-0.135782	0.0200044	1.14E-11	-0.0920593	0.191228	0.6302251	0.4197091	0.1991027	11	FALSE	Vosa et al., 2018	Blood
CLEC2D	ENSG00000069493	12	9828841	rs2080211	12	9837998	T	C	0.423077	0.0112	0.0095	0.2384191	0.292709	0.00899165	1.87E-232	0.0382633	0.0324767	0.2387269	0.5345989	0.8888611	20	FALSE	Vosa et al., 2018	Blood
NEDD4	ENSG00000069869	15	56202532	rs28523546	15	56291573	T	C	0.0157343	-0.0288	0.0266	0.2789386	0.32465	0.0102692	2.37E-219	-0.0887108	0.0819823	0.2792204	0.7587941	0.5302687	4	FALSE	Vosa et al., 2018	Blood
DAPP1	ENSG00000070190	4	100764650	rs2866174	4	100794563	A	T	0.386364	-0.0046	0.0104	0.6582666	-0.253129	0.00883156	1.14E-180	0.0181725	0.0410906	0.6583047	0.1806587	0.1328886	20	FALSE	Vosa et al., 2018	Blood
CDC42	ENSG00000070831	1	22399278	rs2473290	1	22358457	T	C	0.227273	0.0103	0.0118	0.3827277	0.379479	0.00974858	0	0.0271425	0.0311031	0.3828473	0.03557259	0.1788731	20	FALSE	Vosa et al., 2018	Blood
LNX1	ENSG00000072201	4	54446520	rs6827269	4	54320617	C	T	0.269231	-0.0078	0.011	0.4782681	-0.093908	0.00948125	3.98E-23	0.08306	0.117436	0.4793921	0.4786888	0.1922131	20	FALSE	Vosa et al., 2018	Blood
UBE2D1	ENSG00000072401	10	60112069	rs4390300	10	60144207	A	G	0.45979	0.016	0.0101	0.1131577	-0.569226	0.00824429	0	-0.0281083	0.0177481	0.1132525	0.3751741	0.5086545	20	FALSE	Vosa et al., 2018	Blood
FCGR2B	ENSG00000072694	1	161599772	rs61803028	1	161601295	A	C	0.127622	-0.0238	0.0206	0.2479513	1.08359	0.0166667	0	-0.0219641	0.0190139	0.248026	3.55E-06	0.01039928	20	TRUE	Vosa et al., 2018	Blood
NFATC3	ENSG00000072736	16	68190908	rs4627335	16	68140639	G	T	0.339161	0.0138	0.0132	0.2958129	-0.273869	0.0111936	3.35E-132	-0.0503891	0.0482423	0.2962535	0.1167823	0.9346117	20	FALSE	Vosa et al., 2018	Blood
AP1M1	ENSG00000072958	19	16327274	rs2362484	19	16330545	T	C	0.206294	-0.0149	0.0126	0.2369916	-0.52386	0.00899204	0	0.0284427	0.0240572	0.2370882	0.425581	0.9925302	20	FALSE	Vosa et al., 2018	Blood
PVR	ENSG00000073008	19	45158263	rs79572806	19	45122146	T	C	0.0891608	0.0001	0.0179	0.9955426	-0.247264	0.0194103	3.60E-37	-0.0004044	0.0723924	0.9955426	0.9961675	0.8741223	20	FALSE	Vosa et al., 2018	Blood
RAB7A	ENSG00000075785	3	128489302	rs9852825	3	128387357	C	G	0.354895	0.0269	0.0109	0.01359121	-0.127037	0.00937776	8.30E-42	-0.21175	0.0872142	0.01518532	0.06099484	0.8593469	20	FALSE	Vosa et al., 2018	Blood
KIFAP3	ENSG00000075945	1	169972408	rs114812663	1	170046290	T	C	0.0576923	0.033	0.022	0.1336144	-0.338612	0.0277319	2.74E-34	-0.0974567	0.0654596	0.136538	0.1751855	0.9067883	13	FALSE	Vosa et al., 2018	Blood
KLHL20	ENSG00000076321	1	173719960	rs9425411	1	173471890	A	G	0.311189	-0.0145	0.0107	0.1753729	-0.180164	0.0113528	1.03E-56	0.0804822	0.0596065	0.1769434	0.6677831	0.3721685	20	FALSE	Vosa et al., 2018	Blood
PAG1	ENSG00000076641	8	81953414	rs7839273	8	82042292	C	T	0.325175	0.0047	0.0111	0.6719864	-0.251914	0.011815	7.16E-101	-0.0186572	0.0440714	0.6720472	0.5865273	0.681023	20	FALSE	Vosa et al., 2018	Blood
ICAM3	ENSG00000076662	19	10447475	rs7409490	19	10447795	G	A	0.176573	0.0288	0.016	0.07186064	0.430743	0.0119557	3.03E-284	0.0668612	0.0371915	0.07221541	0.02017014	0.1138249	20	FALSE	Vosa et al., 2018	Blood
RAP1GAP	ENSG00000076864	1	21959254	rs10493008	1	22013771	A	G	0.428322	0.0068	0.0099	0.4921655	0.725307	0.00767899	0	0.00937534	0.0136498	0.4921769	0.01552653	0.1654102	20	FALSE	Vosa et al., 2018	Blood
DYNC1I2	ENSG00000077380	2	172574424	rs11886820	2	172614207	T	G	0.274476	0.0097	0.0111	0.3821869	-0.158501	0.0103371	4.59E-53	-0.0611984	0.0701448	0.3829585	0.4427406	0.3338509	20	FALSE	Vosa et al., 2018	Blood
CAPZB	ENSG00000077549	1	19738666	rs114891776	1	19648300	A	G	0.0576923	0.0304	0.0217	0.1612375	-0.316618	0.0266442	1.45E-32	-0.0960146	0.0690114	0.1641385	0.07673488	0.3557032	20	FALSE	Vosa et al., 2018	Blood
UBE2K	ENSG00000078140	4	39742038	rs2170896	4	39694396	C	G	0.304196	0.0346	0.0108	0.00135672	0.197483	0.00961288	8.78E-94	0.175205	0.0553492	0.00154846	0.141024	0.04392359	20	FALSE	Vosa et al., 2018	Blood
ITCH	ENSG00000078747	20	33025119	rs1205344	20	32918298	G	A	0.517483	0.032	0.0095	0.000756	0.188375	0.00891334	3.87E-99	0.169874	0.051068	0.00087965	0.005317869	0.1623394	20	FALSE	Vosa et al., 2018	Blood
UBE2D4	ENSG00000078967	7	43979601	rs149625640	7	43972161	A	G	0.117133	-0.0148	0.0164	0.3668237	0.359828	0.0184308	6.97E-85	-0.0411307	0.0456259	0.367335	0.002738563	0.00969799	20	FALSE	Vosa et al., 2018	Blood
RAPGEF3	ENSG00000079337	12	48146639	rs11168220	12	48135590	A	G	0.227273	-0.023	0.0112	0.04001719	0.251807	0.00984491	2.72E-144	-0.0913397	0.0446216	0.04065962	0.1600229	0.3682804	20	FALSE	Vosa et al., 2018	Blood
DNM2	ENSG00000079805	19	10886459	rs1541596	19	10987013	G	A	0.534965	-0.0001	0.0097	0.9917745	-0.0647435	0.00934303	4.22E-12	0.00154456	0.149822	0.9917745	0.2081589	0.06157785	20	FALSE	Vosa et al., 2018	Blood
PTPRC	ENSG00000081237	1	198667173	rs72738034	1	198645827	C	A	0.0122378	0.0261	0.0428	0.5419856	0.416378	0.0523759	1.87E-15	0.0626834	0.103093	0.5431697	0.6048538	0.8191372	14	FALSE	Vosa et al., 2018	Blood
UBA5	ENSG00000081307	3	132385115	rs61023005	3	132281273	T	C	0.0104895	-0.0284	0.0398	0.4754945	-0.273874	0.0270856	4.92E-24	0.103697	0.145684	0.4765901	0.5975753	0.8934574	15	FALSE	Vosa et al., 2018	Blood
FYB1	ENSG00000082074	5	39189984	rs13171671	5	39224003	T	G	0.166084	-0.0239	0.0126	0.05785099	-0.0995084	0.00911332	9.35E-28	0.240181	0.128519	0.0616456	0.05440907	0.2948011	20	FALSE	Vosa et al., 2018	Blood
ITGB5	ENSG00000082781	3	124543734	rs6768588	3	124487335	G	A	0.166084	0.0057	0.0118	0.6290596	0.159129	0.00970784	2.19E-60	0.03582	0.0741858	0.6292085	0.7214446	0.6063979	20	FALSE	Vosa et al., 2018	Blood
KIF3C	ENSG00000084731	2	26177544	rs10201610	2	26149231	G	T	0.256993	-0.0092	0.0111	0.4072013	0.105472	0.00894119	4.08E-32	-0.0872268	0.105501	0.4083558	0.2900426	0.1525701	20	FALSE	Vosa et al., 2018	Blood
HACE1	ENSG00000085382	6	105241881	rs2486135	6	105282263	C	T	0.5	-0.0091	0.0095	0.3381158	-0.0947182	0.00896663	4.40E-26	0.0960745	0.100709	0.3400935	0.2487801	0.7833894	20	FALSE	Vosa et al., 2018	Blood
PILRA	ENSG00000085514	7	99981436	rs7384878	7	99932049	C	T	0.332168	0.0161	0.0108	0.1360296	-0.0727002	0.00923505	3.48E-15	-0.221458	0.151196	0.1430001	0.0198399	0.549171	20	FALSE	Vosa et al., 2018	Blood
PSMC5	ENSG00000087191	17	61906945	rs2854217	17	61943096	G	C	0.648601	0.0073	0.011	0.5069231	-0.105888	0.00918047	8.89E-31	-0.0689411	0.104056	0.5076248	0.6220766	0.431429	20	FALSE	Vosa et al., 2018	Blood
KLHL42	ENSG00000087448	12	27944463	rs12578595	12	27964996	T	C	0.237762	0.009	0.0118	0.4456353	-0.32451	0.0105702	5.63E-207	-0.0277341	0.0363738	0.4457756	0.1444719	0.3208351	20	FALSE	Vosa et al., 2018	Blood
FKBP1A	ENSG00000088832	20	1361714	rs8392	20	1350709	C	T	0.423077	-0.0025	0.0113	0.8249064	-0.356669	0.00925942	0	0.00700931	0.0316826	0.8249092	0.1032871	0.243583	20	FALSE	Vosa et al., 2018	Blood
DYNLL1	ENSG00000088986	12	120921974	rs7956286	12	121029236	A	G	0.431818	-0.0097	0.01	0.3320465	-0.0714923	0.0089381	1.26E-15	0.135679	0.1409	0.3355751	0.1384103	0.04467142	15	FALSE	Vosa et al., 2018	Blood
ANAPC5	ENSG00000089053	12	121791873	rs1799525	12	121789927	C	A	0.36014	-0.0052	0.0126	0.6798276	-0.224326	0.00883836	4.09E-142	0.0231805	0.0561756	0.6798676	0.04822457	0.1473287	20	FALSE	Vosa et al., 2018	Blood
LAG3	ENSG00000089692	12	6884649	rs56115085	12	6853615	T	C	0.155594	0.0112	0.015	0.4552648	-0.108987	0.0133386	3.06E-16	-0.102765	0.138205	0.4571376	0.2853659	0.5064358	20	FALSE	Vosa et al., 2018	Blood
ICAM1	ENSG00000090339	19	10389401	rs281438	19	10399375	G	T	0.281469	0.0018	0.0104	0.862591	0.180279	0.00999609	1.03E-72	0.0099845	0.0576909	0.8625972	0.03397864	0.01595347	20	FALSE	Vosa et al., 2018	Blood
CD209	ENSG00000090659	19	7808671	rs2335524	19	7785698	C	T	0.727273	0.0059	0.0131	0.6524353	0.189038	0.00886892	8.31E-101	0.0312107	0.0693139	0.6525077	0.4985661	0.5534961	20	FALSE	Vosa et al., 2018	Blood
CD200	ENSG00000091972	3	112066787	rs721363	3	112177130	A	G	0.79021	0.006	0.0123	0.6256881	0.11085	0.00913206	6.59E-34	0.054127	0.11105	0.6259668	0.8018337	0.3927098	20	FALSE	Vosa et al., 2018	Blood
PSME1	ENSG00000092010	14	24606771	rs4575	14	24615435	T	C	0.692308	-0.0096	0.011	0.3828117	0.134054	0.00890947	3.66E-51	-0.0716131	0.0821946	0.3836106	0.584243	0.7410312	20	FALSE	Vosa et al., 2018	Blood
HECTD1	ENSG00000092148	14	31623164	rs113367171	14	31428802	C	T	0.041958	-0.0584	0.03	0.05157471	0.326432	0.0360666	1.42E-19	-0.178904	0.0940046	0.05702173	0.2563168	0.8662648	20	FALSE	Vosa et al., 2018	Blood
SNAP23	ENSG00000092531	15	42810489	rs56357092	15	42790529	A	G	0.243007	0.0235	0.011	0.03264979	0.178527	0.0106145	1.77E-63	0.131633	0.0621105	0.03406213	0.0197893	0.0272596	20	FALSE	Vosa et al., 2018	Blood
CDC23	ENSG00000094880	5	137536185	rs74810661	5	137609836	A	G	0.041958	0.0417	0.0269	0.1210969	0.33173	0.0332862	2.15E-23	0.125705	0.0820651	0.1255802	0.000440584	0.0014136	20	FALSE	Vosa et al., 2018	Blood
PSMD5	ENSG00000095261	9	123591501	rs1060817	9	123583192	A	G	0.40035	-0.0123	0.0099	0.2140801	0.1993	0.00886343	5.74E-112	-0.0617162	0.0497497	0.2147787	0.01887613	0.7404615	20	FALSE	Vosa et al., 2018	Blood
ITPR3	ENSG00000096433	6	33626436	rs9469532	6	33601132	G	A	0.657343	0.0063	0.0099	0.5245394	0.495245	0.00851021	0	0.012721	0.0199913	0.5245642	0.4296533	0.9713585	20	FALSE	Vosa et al., 2018	Blood

PSMD8	ENSG00000099341	19	38869820	rs150489116	19	38842140	T	C	0.027972	-0.0687	0.046	0.135312	0.510107	0.0446686	3.33E-30	-0.134678	0.0909451	0.1386417	0.7822404	0.00852529	8	FALSE	Vosa et al., 2018	Blood
MADCAM1	ENSG00000099866	19	497261	rs2238546	19	583698	A	G	0.0996503	0.022	0.0205	0.2831945	-0.104804	0.0124228	3.27E-17	-0.209915	0.197179	0.2870607	0.139169	0.3005007	7	FALSE	Vosa et al., 2018	Blood
KLHL22	ENSG00000099910	22	20816849	rs62219034	22	20800256	T	C	0.263986	-0.0102	0.0112	0.3624459	0.167834	0.0128583	6.14E-39	-0.0607744	0.0668949	0.3636112	0.6975636	0.8584251	20	FALSE	Vosa et al., 2018	Blood
FBXO7	ENSG00000100225	22	32882740	rs7291067	22	32873496	A	C	0.484266	0.0097	0.0096	0.3122957	0.148253	0.00892174	5.25E-62	0.0654289	0.064874	0.3131886	0.61658	0.5854925	20	FALSE	Vosa et al., 2018	Blood
AP1B1	ENSG00000100280	22	29771418	rs3788412	22	29750186	C	T	0.241259	-0.0103	0.0105	0.3266162	-0.168966	0.00903513	4.86E-78	0.060959	0.0622281	0.3272808	0.2066622	0.3534856	20	FALSE	Vosa et al., 2018	Blood
GRAP2	ENSG00000100351	22	40333405	rs137955	22	40291807	T	C	0.409091	0.015	0.0101	0.1375045	-0.115824	0.00973217	1.17E-32	-0.129506	0.0878772	0.1405575	0.03091838	0.1137331	20	FALSE	Vosa et al., 2018	Blood
NCF4	ENSG00000100365	22	37265543	rs5750318	22	37224357	G	C	0.136364	0.0208	0.0146	0.1542562	-0.145726	0.0154429	3.86E-21	-0.142734	0.101324	0.1589258	0.5424971	0.03958463	7	FALSE	Vosa et al., 2018	Blood
TRIM9	ENSG00000100505	14	51502379	rs74598030	14	51528533	C	T	0.0297203	-0.0515	0.031	0.09665515	0.686194	0.0282113	1.11E-130	-0.0750516	0.045282	0.09743278	0.163492	0.4006372	20	FALSE	Vosa et al., 2018	Blood
PSMC6	ENSG00000100519	14	53184597	rs12590590	14	53161736	G	T	0.38986	0.0036	0.0119	0.7622549	-0.155123	0.00914394	1.50E-64	-0.0232073	0.0767254	0.7622916	0.4666856	0.3640215	18	FALSE	Vosa et al., 2018	Blood
LGMN	ENSG00000100600	14	93192599	rs2402189	14	93170901	A	C	0.405594	0.0163	0.01	0.1031015	-0.235829	0.00982555	2.68E-127	-0.0691179	0.0425013	0.1038956	0.8344817	0.2114481	20	FALSE	Vosa et al., 2018	Blood
ASB2	ENSG00000100628	14	94421818	rs4905126	14	94443410	C	T	0.213287	0.0023	0.0125	0.8540135	0.0810707	0.0114303	1.32E-12	0.0283703	0.154238	0.854062	0.7113105	0.5161231	10	FALSE	Vosa et al., 2018	Blood
PSMB5	ENSG00000100804	14	23495095	rs11543947	14	23504021	A	G	0.0611888	0.0126	0.0171	0.4612184	-0.587548	0.0256663	5.61E-116	-0.0214451	0.0291191	0.4614504	0.6572246	0.740391	16	FALSE	Vosa et al., 2018	Blood
PSMA6	ENSG00000100902	14	35767269	rs10135636	14	35668821	C	T	0.0996503	-0.0148	0.0152	0.3302134	0.156943	0.0135075	3.30E-31	-0.0943017	0.0971898	0.3319053	0.6053651	0.3227106	20	FALSE	Vosa et al., 2018	Blood
NFKBIA	ENSG00000100906	14	35872336	rs12100841	14	35571651	G	A	0.157343	-0.0088	0.013	0.4984548	0.287339	0.0130336	1.04E-107	-0.0306258	0.045264	0.4986571	0.8512126	0.9473092	20	FALSE	Vosa et al., 2018	Blood
CD40	ENSG00000101017	20	44752706	rs745307	20	44747086	A	G	0.229021	-0.0106	0.0114	0.3524619	0.745766	0.012453	0	-0.0142136	0.0152881	0.35252	0.5048353	0.3602916	20	FALSE	Vosa et al., 2018	Blood
TUBB1	ENSG00000101162	20	57598009	rs463312	20	57597970	C	A	0.0332168	0.0138	0.0273	0.6132116	-0.150253	0.0199191	4.59E-14	-0.0918449	0.182101	0.6140061	0.6754224	0.4085688	3	FALSE	Vosa et al., 2018	Blood
PSMA7	ENSG00000101182	20	60715143	rs2023265	20	60721132	A	C	0.173077	0.0266	0.0131	0.04230225	-0.0839137	0.00935932	3.08E-19	-0.316992	0.160066	0.04766069	0.07587037	0.8751087	20	FALSE	Vosa et al., 2018	Blood
TRIB3	ENSG00000101255	20	369732	rs6076464	20	355480	C	T	0.438811	-0.0046	0.0111	0.6785706	0.49593	0.0084885	0	-0.0092755	0.0223828	0.6785783	0.5316779	0.7075665	20	FALSE	Vosa et al., 2018	Blood
KIF3B	ENSG00000101350	20	30894140	rs12480429	20	30907865	G	T	0.113636	-0.0085	0.0149	0.5683591	-0.0891452	0.0136183	5.91E-11	0.09535	0.167776	0.5698206	0.5123211	0.3859264	20	FALSE	Vosa et al., 2018	Blood
MGRN1	ENSG00000102858	16	4703734	rs841228	16	4733542	G	T	0.536713	0.0043	0.0096	0.6542133	0.387483	0.00862963	0	0.0110973	0.0247765	0.6542294	0.574653	0.1134524	20	FALSE	Vosa et al., 2018	Blood
PSMD7	ENSG00000103035	16	74335429	rs59681627	16	74311453	A	G	0.11014	-0.011	0.0172	0.522475	0.0699039	0.0124027	1.74E-08	-0.157359	0.247631	0.5251303	0.7900771	0.5322459	4	FALSE	Vosa et al., 2018	Blood
FBXO31	ENSG00000103264	16	87393170	rs8052244	16	87402261	C	G	0.506993	0.0036	0.0102	0.7241325	0.315841	0.010455	1.78E-200	0.0113981	0.032297	0.7241506	0.1446458	0.2613301	20	FALSE	Vosa et al., 2018	Blood
ELOB	ENSG00000103363	16	2824356	rs2285877	16	2822174	A	G	0.222028	0.0225	0.0116	0.05242161	0.215571	0.00975338	3.01E-108	0.104374	0.0540174	0.05333126	0.154771	0.1327026	15	FALSE	Vosa et al., 2018	Blood
STX4	ENSG00000103496	16	31049253	rs58726213	16	31044683	A	G	0.655594	0.0835	0.0099	3.33E-17	-0.391309	0.00914827	0	-0.213387	0.0257869	1.28E-16	8.61E-16	5.97E-07	20	TRUE	Vosa et al., 2018	Blood
CSK	ENSG00000103653	15	75084968	rs3935077	15	75120211	C	G	0.297203	-0.0209	0.0105	0.0465385	0.364371	0.00887611	0	-0.0573592	0.0288507	0.04679616	0.001772971	0.02325858	20	FALSE	Vosa et al., 2018	Blood
HERC1	ENSG00000103657	15	64013479	rs12148696	15	64006923	A	T	0.365385	0.0015	0.0101	0.8819365	0.168301	0.0106212	1.50E-56	0.00891259	0.0600141	0.8819416	0.04852234	0.156687	20	FALSE	Vosa et al., 2018	Blood
CTSH	ENSG00000103811	15	79227658	rs34843303	15	79234470	C	T	0.111888	-0.0286	0.0169	0.09058732	-0.913762	0.0126186	0	0.0312992	0.0185	0.09067539	0.03925814	0.909273	13	FALSE	Vosa et al., 2018	Blood
RIPK2	ENSG00000104312	8	90786633	rs428484	8	90776033	T	C	0.5	0.0111	0.0095	0.242637	0.290255	0.00903306	1.55E-226	0.0382422	0.0327514	0.2429482	0.4344442	0.7574028	20	FALSE	Vosa et al., 2018	Blood
UBE2W	ENSG00000104343	8	74741738	rs184898399	8	74362056	C	G	0.0157343	0.0296	0.0528	0.5750661	-0.415858	0.0559746	1.09E-13	-0.0711782	0.127328	0.5761502	0.2178922	0.1835626	6	FALSE	Vosa et al., 2018	Blood
IKBKB	ENSG00000104365	8	42159396	rs13278372	8	42182241	A	C	0.111888	-0.0157	0.0161	0.3294832	0.143181	0.00989678	1.95E-47	-0.109652	0.1127	0.3305793	0.6671628	0.04581389	20	FALSE	Vosa et al., 2018	Blood
DCTN6	ENSG00000104671	8	30027484	rs111632716	8	30081054	A	G	0.00874126	0.0589	0.0581	0.3106928	0.491123	0.0345146	6.02E-46	0.119929	0.1186	0.3119177	0.5495357	0.7272642	20	FALSE	Vosa et al., 2018	Blood
PPP2CB	ENSG00000104695	8	30651901	rs10955044	8	30663061	C	G	0.0367133	-0.0047	0.0219	0.8300699	0.685413	0.0164559	0	-0.0068572	0.031952	0.8300722	0.6636084	0.6876321	20	FALSE	Vosa et al., 2018	Blood
TUBB4A	ENSG00000104833	19	6498594	rs2071347	19	6495736	A	G	0.347902	0.0184	0.0137	0.1792507	0.100279	0.00921581	1.42E-27	0.183488	0.137655	0.1825481	0.1071711	0.9145709	20	FALSE	Vosa et al., 2018	Blood
KLC3	ENSG00000104892	19	45845735	rs9749618	19	45849909	G	A	0.335664	-0.0027	0.01	0.7871603	0.267171	0.0110403	2.25E-129	-0.0101059	0.0374315	0.7871732	0.188512	0.1532032	20	FALSE	Vosa et al., 2018	Blood
LILRB1	ENSG00000104972	19	55117162	rs10416183	19	55146803	T	C	0.131119	-0.0121	0.0172	0.4817515	0.703135	0.0131473	0	-0.0172086	0.024464	0.4817894	0.7577038	0.4097812	20	FALSE	Vosa et al., 2018	Blood
LILRA1	ENSG00000104974	19	55109301	rs7249811	19	55087313	G	T	0.0856643	-0.003	0.0175	0.8638868	-0.505775	0.0143894	1.19E-270	0.0059315	0.0346008	0.8638884	0.7746021	0.6198313	20	FALSE	Vosa et al., 2018	Blood
AKT2	ENSG00000105221	19	40763833	rs4332845	19	40785505	T	A	0.263986	-0.0202	0.0107	0.05904603	-0.0991122	0.0104535	2.51E-21	0.203809	0.110078	0.06409707	0.1203322	0.701107	15	FALSE	Vosa et al., 2018	Blood
CD33	ENSG00000105383	19	51737717	rs1710398	19	51726911	C	A	0.47028	-0.0054	0.0096	0.5737754	0.290089	0.00913781	3.60E-221	-0.018615	0.0330984	0.5738355	0.5585771	0.1947531	18	FALSE	Vosa et al., 2018	Blood
SIGLEC6	ENSG00000105492	19	52028944	rs2305772	19	52033742	G	A	0.407343	0.0004	0.0099	0.967771	-0.123916	0.00897216	2.18E-43	-0.003228	0.079893	0.9677711	0.8146838	0.3968278	20	FALSE	Vosa et al., 2018	Blood
SIGLEC5	ENSG00000105501	19	52132466	rs2053924	19	52123549	G	A	0.0961538	0.0108	0.0154	0.4831166	-0.532466	0.0144111	7.74E-299	-0.020283	0.0289273	0.4831954	0.3545672	0.6080306	20	FALSE	Vosa et al., 2018	Blood
PPP2R1A	ENSG00000105568	19	52711989	rs61063927	19	52690790	C	T	0.291958	0.0183	0.0114	0.1084359	0.182001	0.010011	7.43E-74	0.100549	0.0628808	0.1098113	0.4475708	0.7685415	20	FALSE	Vosa et al., 2018	Blood
LILRB5	ENSG00000105609	19	54757715	rs3852892	19	54722595	A	G	0.0874126	-0.0249	0.0214	0.2446059	0.152161	0.0152048	1.41E-23	-0.163643	0.141588	0.2477771	0.3718635	0.8480629	12	FALSE	Vosa et al., 2018	Blood
AP1S1	ENSG00000106367	7	100801277	rs2239538	7	100798728	A	G	0.185315	-0.0172	0.0136	0.2059768	0.242551	0.00972796	3.23E-137	-0.0709129	0.0561427	0.2065597	0.1257311	0.6802901	20	FALSE	Vosa et al., 2018	Blood
PSMA2	ENSG00000106588	7	42964141	rs1060304	7	42956933	A	G	0.0909091	0.0218	0.0167	0.1917603	0.553513	0.0189314	6.42E-188	0.0393848	0.030201	0.1922029	0.5138765	0.2288033	20	FALSE	Vosa et al., 2018	Blood
DNM1	ENSG00000106976	9	130991592	rs10760548	9	130965235	G	A	0.00874126	-0.0689	0.0378	0.06834106	0.301054	0.0186702	1.71E-58	-0.228863	0.126359	0.07010741	0.0138652	0.4223475	20	FALSE	Vosa et al., 2018	Blood
NPDC1	ENSG00000107281	9	139937288	rs867476	9	139958583	G	C	0.41958	0.0148	0.0114	0.1942029	0.377482	0.0108719	3.82E-264	0.0392072	0.0302212	0.1945146	0.593885	0.4583499	20	FALSE	Vosa et al., 2018	Blood
UBE2R2	ENSG00000107341	9	33868983	rs10441723	9	34082144	T	C	0.323427	0.0125	0.0102	0.2203906	0.0711452	0.00897392	2.23E-15	0.175697	0.145071	0.2258546	0.1039382	0.00603131	20	FALSE	Vosa et al., 2018	Blood
FBXL15	ENSG00000107872	10	104180919	rs111522958	10	104181006	C	G	0.020979	0.0682	0.0321	0.03361912	0.5158	0.0235822	4.76E-106	0.132222	0.0625263	0.03445914	0.001451761	0.08140175	20	FALSE	Vosa et al., 2018	Blood
MAP3K8	ENSG00000107968	10	30736814	rs588931	10	30781288	T	C	0.201049	0.0129	0.0131	0.3247553	0.226736	0.00904404	1.05E-138	0.0568944	0.0578211	0.3251283	0.1654711	0.1380976	20	FALSE	Vosa et al., 2018	Blood
CUL2	ENSG00000108094	10	35338524	rs2384287	10	35340559	G	A	0.351399	0.0193	0.0098	0.04890858	-0.145455	0.00937908	3.04E-54	-0.132688	0.0679161	0.05073694	0.03780801	0.1560566	20	FALSE	Vosa et al., 2018	Blood
FBXL20	ENSG00000108306	17	37487080	rs3964723	17	37403979	C	T	0.270979	0.0028	0.0108	0.7954352	0.169134	0.00948742	4.35E-71	0.0165549	0.0638614	0.7954564	0.9479156	0.8972993	20	FALSE	Vosa et al., 2018	Blood
PSMD3	ENSG00000108344	17	38145631	rs2302778	17	38179667	A	G	0.26049	-0.0026	0.0113	0.818023	0.195431	0.0101372	8.12E-83	-0.0133039	0.0578249	0.8180357	0.454432	0.06823111	20	FALSE	Vosa et al., 2018	Blood
TRIM37	ENSG00000108395	17	57122140	rs4932693	17	57110327	C	T	0.375874	0.0357	0.0101	0.00040831	-0.588868	0.00833942	0	-0.0606248	0.017173	0.00041519	0.02086933	0.4375931	20	FALSE	Vosa et al., 2018	Blood
ICAM2	ENSG00000108622	17	62088974	rs58370062	17	62105389	G	A	0.395105	-0.0127	0.0102	0.2130957	-0.224929	0.00969496	4.49E-119	0.0564623	0.0454129	0.213754	0.06260534	0.07938663	20	FALSE	Vosa et al., 2018	Blood
PSMD11	ENSG00000108671	17	30790807	rs118092144	17	30778945	A	G	0.513986	-0.0015	0.0096	0.875836	0.0710301	0.00962467	1.58E-13	-0.0211178	0.135184	0.8758636	0.8757945	0.8115853	20	FALSE	Vosa et al., 2018	Blood
COL1A1	ENSG00000108821	17	48269821	rs9898186	17	48278507	T	C	0.229021	0.031	0.0142	0.02902855	0.0980596	0.0109505	3.40E-19	0.316134	0.149051	0.03392356	0.01528183	0.5554212	6	FALSE	Vosa et al., 2018	Blood
SMURF2	ENSG00000108854	17	62598299	rs35018407	17	62690181	A	C	0.272727	0.0215	0.0122	0.07801945	-0.0625389	0.00928869	1.66E-11	-0.343786	0.201651	0.08822037	0.04857027	0.7387063	12	FALSE	Vosa et al., 2018	Blood
WSB1	ENSG00000109046	17	25630879	rs4795561	17	25629890	A	G	0.388112	0.005	0.0094	0.5947849	-0.202981	0.00899157	7.70E-113	-0.0246328	0.0463226	0.5948872	0.8153255	0.6757239	20	FALSE	Vosa et al., 2018	Blood
NFKB1	ENSG00000109320	4	103480472	rs11097787	4	103407342	T	C	0.40035	-0.0052	0.0113	0.6453892	0.183195	0.00892962	1.57E-93	-0.0283851	0.0616985	0.6454722	0.4627897	0.4204278	20	FALSE	Vosa et al., 2018	Blood
UBE2D3	ENSG00000109332	4	103752796	rs223480	4	103688038	T	C	0.519231	0.0211	0.0108	0.05073627	0.0568457	0.00904639	3.30E-10	0.37118	0.198959	0.06209553	0.05393027	0.575103	20	FALSE	Vosa et al., 2018	Blood
KLHL2	ENSG00000109466	4	166186539	rs10016586	4	166199390	T	C	0.0716783	-0.0354	0.0197	0.07234287	0.11704	0.0128987	1.15E-19	-0.302461	0.171588	0.0779476	0.2071904	0.6211573	15	FALSE	Vosa et al., 2018	Blood
FBXW7	ENSG00000109670	4	153349291	rs17028954	4	153438333	G	C	0.274476	0.0083	0.0108	0.4421792	0.120248	0.00905402	2.98E-40	0.0690239	0.0899645	0.4429417	0.4987806	0.5299876	20	FALSE	Vosa et al., 2018	Blood
KLHL5	ENSG00000109790	4	39087568	rs3806735	4	38953128	C	T	0.428322	0.0089	0.0102	0.3829089	-0.474185	0.00904247	0	-0.018769	0.0215136	0.3829748	0.6803549	0.0814226	20	FALSE	Vosa et al., 2018	Blood
CTSC	ENSG00000109861	11	88048857	rs3758833	11	88071656	G	A	0.816434	-0.0184	0.0126	0.1442028	0.453774	0.00907008	0	-0.0405488	0.027779	0.1443737	0.3602217	0.9427195	20	FALSE	Vosa et al., 2018	Blood
CRTAM	ENSG00000109943	11	122726277	rs10444340	11	122746788	G	A	0.685315	-0.0015	0.0107	0.8885123	0.331363	0.00874061	0	-0.0045268	0.0322911	0.8885131	0.7881178	0.4087857	20	FALSE	Vosa et al., 2018	Blood
UBE4A	ENSG00000110344	11	118250113	rs3212262	11	118217854	T	G	0.0979021	-0.0005	0.0198	0.9798535	0.336293	0.0172341	8.48E-85	-0.0014868	0.0588773	0.9798536	0.7957767	0.7239348	20	FALSE	Vosa et al., 2018	Blood
CD81	ENSG00000110651	11	2408028	rs2651828	11	2307694	T	C	0.407343	0.0035	0.0105	0.7388827	-0.23955	0.00978769	2.75E-132	-0.0146107	0.0438363	0.738906	0.8728524	0.7173465	20	FALSE	Vosa et al., 2018	Blood
PSMD9	ENSG00000110801	12	122341420	rs876934	12	122354848	T	C	0.307692	-0.0199	0.0103	0.05335471	-0.213197	0.00959534	2.26E-109	0.0933409	0.0484945	0.05425742	0.00376407	0.00326134	20	FALSE	Vosa et al., 2018	Blood
CLEC2B	ENSG00000110852	12	10014159	rs2908700	12	10004648	C	T	0.417832	0.0101	0.0099	0.3076327	0.152984	0.00892189	6.62E-66	0.0660201	0.0648272	0.3084874	0.8654678	0.983397	20	FALSE	Vosa et al., 2018	Blood
KLRB1	ENSG00000111796	12	9753814	rs10771784	12	9626678	C	T	0.426573	0.0075	0.0116	0.5179221	-0.206034	0.00889672	1.20E-118	-0.0364018	0.0563234	0.5180852	0.36691	0.00148274	20	FALSE	Vosa et al., 2018	Blood
BTN3A3	ENSG00000111801	6	26447171	rs75403944	6	26413087	C	A	0.0034965	0.1534	0.1571	0.3288424	-1.05472	0.055638	3.88E-80	-0.145442	0.149147	0.3294832	0.01040191	0.3856892	20	FALSE	Vosa et al., 2018	Blood
FBXO9	ENSG00000112146	6	52941230	rs222439	6	52948145	A	G	0.216783	0.0236	0.0121	0.05112689	-0.0710251	0.0102501	4.23E-12	-0.332277	0.176983	0.06045565	0.4219404	0.1067068	13	FALSE	Vosa et al., 2018	Blood
TREML2	ENSG00000112195	6	41163473	rs12208563	6	41155815	A	T	0.0227273	-0.0521	0.0281	0.06372592	-0.353413	0.015037	3.82E-122	0.14742	0.0797575	0.06455173	0.04018381	0.2528646	18	FALSE	Vosa et al., 2018	Blood
FBXL4	ENSG00000112234	6	99358591	rs7752268	6	99301013	A	G	0.417832	0.014	0.0107	0.1907339	-0.125287	0.0100675	1.49E-35	-0.111743	0.0858743	0.1931766	0.1689344	0.4799374	20	FALSE	Vosa et al., 2018	Blood
PPP2R5D	ENSG00000112640	6	42966158	rs59541897	6	42969573	C	T	0.134615	-0.0254	0.0144	0.07775069	-0.145703	0.0098049	5.97E-50	0.174328	0.0995252	0.07984389	0.05115537	0.09534958	20	FALSE	Vosa et al., 2018	Blood
BTN2A1	ENSG00000112763	6	26467499	rs2273193	6	26458525	C	G	0.244755	0.0111	0.0115	0.3344359	0.521764	0.00981339	0	0.021274	0.0220443	0.3345156	0.000112127	0.2375275	20	FALSE	Vosa et al., 2018	Blood
ITK	ENSG00000113263	5	156626072	rs6876898	5	156670047	G	A	0.769231	0.0119	0.0116	0.3049566	-0.208801	0.00890898	1.79E-121	-0.0569919	0.0556084	0.3054194	0.1205669	0.4669304	20	FALSE	Vosa et al., 2018	Blood
RNF130	ENSG00000113269	5	179422514	rs28478989	5	179500185	T	C	0.034965	0.0449	0.0255	0.07827492	0.352049	0.0162354	2.90E-104	0.127539	0.0726715	0.07925798	0.5329336	0.7169583	20	FALSE	Vosa et al., 2018	Blood
BTNL8	ENSG00000113303	5	180351991	rs7710895	5	180374019	C	T	0.297203	-0.0078	0.0117	0.5049851	-0.787609	0.00915056	0	0.00990339	0.0148555	0.5049979	0.6978734	0.3450713	20	FALSE	Vosa et al., 2018	Blood
LNPEP	ENSG00000113441	5	96322158	rs2617439	5	96275154	A	G	0.421329	0.0043	0.0096	0.6542133	0.329648	0.0091783	1.72E-282	0.0130442	0.0291242	0.6542385	0.4270724	0.01875789	20	FALSE	Vosa et al., 2018	Blood
SKP1	ENSG00000113558	5	133526922	rs17167302	5	133482926	A	G	0.0559441	-0.0011	0.0209	0.9580255	-0.377895	0.0160443	1.16E-122	0.00291086	0.0553065	0.9580256	0.3550167	0.6252326	20	FALSE	Vosa et al., 2018	Blood
PPP2CA	ENSG00000113575	5	133545929	rs4642415	5	133540726	G	A	0.11014	-0.0268	0.0149	0.07207284	-0.0681286	0.011258	1.43E-09	0.393374	0.22816	0.08468706	0.08595873	0.1541951	7	FALSE	Vosa et al., 2018	Blood
CD86	ENSG00000114013	3	121807098	rs13088702	3	121714019	C	T	0.0751748	-0.0016	0.0183	0.9303284	0.328955	0.0159657	2.54E-94	-0.0048639	0.0556312	0.930329	0.009355413	0.2821881	20	FALSE	Vosa et al., 2018	Blood
RNF7	ENSG00000114125	3	141461724	rs112917579	3	141474908	T	C	0.146853	-0.0417	0.0143	0.00354455	-0.320572	0.0129259	8.79E-136	0.13008	0.044915	0.00377792	0.01150384	0.1508604	20	FALSE	Vosa et al., 2018	Blood
CBLB	ENSG00000114423	3	105481350	rs2301050	3	105401507	T	C	0.270979	-0.0006	0.0111	0.9568921	-0.372358	0.00996133	8.14E-306	0.00161135	0.02981	0.9568921	0.8926566	0.6303566	20	FALSE	Vosa et al., 2018	Blood
ACTR1B	ENSG00000115073	2	98276500	rs11683207	2	98333290	C	T	0.159091	0.0067	0.0135	0.6196854	0.396788	0.015904	2.19E-137	0.0168856	0.0340299	0.6197546	0.124005	0.2176077	6	FALSE	Vosa et al., 2018	Blood
ZAP70	ENSG00000115085	2	98343174	rs746004	2	98362907	T	C	0.129371	-0.0112	0.0144	0.4367	0.250335	0.0121889	9.85E-94	-0.04474	0.0575641	0.4370286	0.1351177	0.03647407	20	FALSE	Vosa et al., 2018	Blood
ITGA4	ENSG00000115232	2	182361421	rs1375493	2	182323766	G	A	0.461538	-0.0012	0.0099	0.903523	0.490155	0.00839727	0	-0.0024482	0.0201977	0.9035232	0.0172215	0.753468	20	FALSE	Vosa et al., 2018	Blood
ASB3	ENSG00000115239	2	53923553	rs13408029	2	53928465	G	C	0.166084	-0.0115	0.0123	0.3498092	-0.183683	0.0110701	7.86E-62	0.0626078	0.0670693	0.3505729	0.1980727	0.3398863	20	FALSE	Vosa et al., 2018	Blood
SOS1	ENSG00000115904	2	39280011	rs4670904	2	39228883	G	C	0.0804196	-0.0183	0.0177	0.3011837	0.361914	0.0101914	3.25E-276	-0.0505646	0.0489274	0.3013885	0.07395184	0.5151728	20	FALSE	Vosa et al., 2018	Blood

CD207	ENSG00000116031	2	71060149	rs7561215	2	71053852	A	G	0.332168	0.0059	0.0101	0.5591137	0.108798	0.00910914	7.00E-33	0.0542291	0.0929438	0.559583	0.3403497	0.1843462	20	FALSE	Vosa et al., 2018	Blood
RAP1A	ENSG00000116473	1	112172076	rs72695272	1	112167372	T	C	0.143357	0.0083	0.0133	0.5325881	-0.221084	0.0134928	2.43E-60	-0.0375423	0.0602017	0.532885	0.2010764	0.4943781	20	FALSE	Vosa et al., 2018	Blood
CAPZA1	ENSG00000116489	1	113188018	rs3790597	1	113162037	A	C	0.229021	0.0155	0.0159	0.3296383	-0.0714335	0.0129114	3.16E-08	-0.216985	0.226013	0.3370282	0.3370282			FALSE	Vosa et al., 2018	Blood
RNF19B	ENSG00000116514	1	33416166	rs59669045	1	33444351	A	G	0.27972	-0.005	0.0116	0.6664433	0.133899	0.00935636	1.87E-46	-0.0373416	0.0866719	0.6665854	0.4652388	0.2014383	20	FALSE	Vosa et al., 2018	Blood
FBXO2	ENSG00000116661	1	11712133	rs2272983	1	11714368	T	G	0.22028	-0.0118	0.0127	0.3528197	0.525386	0.0106038	0	-0.0224597	0.024177	0.3529044	0.1393572	0.2351225	13	FALSE	Vosa et al., 2018	Blood
FBXO6	ENSG00000116663	1	11729296	rs3125812	1	11724938	A	C	0.519231	-0.0272	0.0104	0.0089127	0.214106	0.00902707	2.34E-124	-0.12704	0.0488685	0.0093325	0.01151007	0.7217176	20	FALSE	Vosa et al., 2018	Blood
NCF2	ENSG00000116701	1	183542354	rs10797890	1	183563709	T	C	0.447552	-0.0197	0.0096	0.04016156	0.13696	0.00910843	4.23E-51	-0.143838	0.0707432	0.04202864	0.0111674	0.1256715	20	FALSE	Vosa et al., 2018	Blood
AKT3	ENSG00000117020	1	243832958	rs12134270	1	243662668	G	T	0.201049	0.0278	0.0121	0.02158909	0.132603	0.0111858	2.04E-32	0.209648	0.0929474	0.02409883	4.90E-05	0.00238409	20	TRUE	Vosa et al., 2018	Blood
CD160	ENSG00000117281	1	145705706	rs10910844	1	145709869	T	C	0.512238	-0.0432	0.0104	3.27E-05	0.159597	0.0090177	4.33E-70	-0.270682	0.0669349	5.26E-05	0.000581398	0.4240653	20	FALSE	Vosa et al., 2018	Blood
CDC20	ENSG00000117399	1	43826750	rs839764	1	43833374	A	T	0.395105	0.0236	0.0098	0.01603301	-0.0834214	0.0102645	4.39E-16	-0.282901	0.122525	0.02094722	0.0129275	0.02453683	11	FALSE	Vosa et al., 2018	Blood
PIK3R3	ENSG00000117461	1	46573986	rs4660322	1	46367773	C	T	0.451049	-0.0132	0.0094	0.1602428	0.176608	0.00887471	4.05E-88	-0.0747416	0.0533574	0.1612822	0.1244461	0.5860807	18	FALSE	Vosa et al., 2018	Blood
CTSD	ENSG00000117984	11	1779602	rs59571891	11	1771188	G	T	0.237762	-0.0047	0.0113	0.6774618	-0.123577	0.00961079	7.74E-38	0.0380328	0.0914885	0.6776209	0.2161897	0.3945402	20	FALSE	Vosa et al., 2018	Blood
UBE3D	ENSG00000118420	6	83688838	rs4235816	6	83733785	C	T	0.323427	-0.0002	0.0104	0.984657	0.087264	0.00937254	1.27E-20	-0.0022919	0.119179	0.984657	0.1739488	0.1233676	20	FALSE	Vosa et al., 2018	Blood
FBXO30	ENSG00000118496	6	146127580	rs34211445	6	146188902	G	T	0.0786713	0.0062	0.0176	0.7246337	-0.220889	0.0194987	9.49E-30	-0.0280684	0.0797164	0.7247614	0.9823116	0.930393	20	FALSE	Vosa et al., 2018	Blood
FBXL5	ENSG00000118564	4	15644732	rs11947383	4	15706193	A	G	0.40035	-0.0663	0.0117	1.46E-08	0.190668	0.00902279	4.06E-99	-0.347725	0.0635313	4.42E-08	2.09E-12	5.20E-08	20	TRUE	Vosa et al., 2018	Blood
VAMP8	ENSG00000118640	2	85798919	rs1010	2	85808982	C	T	0.409091	-0.0309	0.0095	0.00114342	-0.428287	0.0085758	0	0.0721479	0.0222284	0.0011714	0.00507522	0.05092163	20	FALSE	Vosa et al., 2018	Blood
PPL	ENSG00000118898	16	4971625	rs12102426	16	4922450	T	G	0.543706	-0.0036	0.0096	0.7076605	0.226769	0.00898125	1.16E-140	-0.0158752	0.0423384	0.7076912	0.3416531	0.7017679	20	FALSE	Vosa et al., 2018	Blood
UBE2B	ENSG00000119048	5	133717276	rs2436323	5	133447061	C	T	0.0699301	0.0363	0.0173	0.03588166	-0.144465	0.0177136	3.47E-16	-0.251272	0.123652	0.04214466	0.04431547	0.811949	15	FALSE	Vosa et al., 2018	Blood
TRIM32	ENSG00000119401	9	119456580	rs12351794	9	119432650	A	G	0.307692	-0.011	0.0104	0.2901957	0.193054	0.0097494	2.88E-87	-0.0569789	0.0539478	0.2908829	0.8808089	0.8390219	20	FALSE	Vosa et al., 2018	Blood
FBXW2	ENSG00000119402	9	123534973	rs34025993	9	123516572	A	G	0.40035	-0.011	0.0103	0.285538	-0.0878161	0.00893603	8.60E-23	0.125262	0.117981	0.2883673	0.2589993	0.6028003	20	FALSE	Vosa et al., 2018	Blood
MAPKAP1	ENSG00000119487	9	128334592	rs509753	9	128303518	G	A	0.45979	0.0377	0.0095	7.24E-05	0.121088	0.00891399	4.98E-42	0.311345	0.081735	0.00013942	0.00045674	0.8367115	20	FALSE	Vosa et al., 2018	Blood
CD274	ENSG00000120217	9	5460534	rs2890658	9	5465130	A	C	0.108392	0.0042	0.0177	0.8124333	0.222335	0.01224	9.84E-74	0.0188904	0.0796162	0.812449	0.6958256	0.1906846	15	FALSE	Vosa et al., 2018	Blood
KBTBD7	ENSG00000120696	13	41766335	rs498883	13	41747920	A	G	0.15035	0.0028	0.0164	0.8644347	0.168719	0.00897871	8.95E-79	0.0165957	0.0972071	0.8644403	0.2148152	0.04402304	20	FALSE	Vosa et al., 2018	Blood
PILRB	ENSG00000121716	7	99949578	rs11761426	7	99956893	T	A	0.176573	-0.04	0.0122	0.00104291	1.36039	0.0118624	0	-0.0294034	0.0089717	0.00104787	0.005593001	0.00289441	20	FALSE	Vosa et al., 2018	Blood
PIK3CA	ENSG00000121879	3	178911891	rs10936990	3	178800879	G	T	0.440559	0.0227	0.0099	0.02185208	-0.0863763	0.00930659	1.68E-20	-0.262804	0.118061	0.0260139	0.01904223	3.14E-06	20	FALSE	Vosa et al., 2018	Blood
RBBP6	ENSG00000122257	16	24566599	rs139667503	16	24546453	T	A	0.127622	0.0034	0.0157	0.8285509	0.297183	0.0174739	7.26E-65	0.0114408	0.0528337	0.8285645	0.4881735	0.2655075	20	FALSE	Vosa et al., 2018	Blood
CLTA	ENSG00000122705	9	36247815	rs2025879	9	36204455	T	C	0.332168	0.0006	0.0105	0.9544314	0.101456	0.00920872	3.15E-28	0.00591389	0.103494	0.954432	0.1299417	0.1045278	20	FALSE	Vosa et al., 2018	Blood
ITPR2	ENSG00000123104	12	26738236	rs74976642	12	26998022	A	G	0.120629	-0.0029	0.0163	0.8587904	-0.583457	0.016515	2.11E-273	0.00497038	0.0279373	0.8587922	0.6639374	0.4636295	20	FALSE	Vosa et al., 2018	Blood
WWP1	ENSG00000123124	8	87422808	rs3213911	8	87556691	A	G	0.412587	0.0108	0.0105	0.3036811	0.168165	0.0097365	7.70E-67	0.0642227	0.0625493	0.3045371	0.6355509	0.5068527	20	FALSE	Vosa et al., 2018	Blood
TUBA1B	ENSG00000123416	12	49523372	rs2162644	12	49548190	G	A	0.681818	-0.029	0.0101	0.00408804	-0.0869697	0.00893831	2.25E-22	0.333449	0.121083	0.00588928	0.001977113	0.1202147	20	FALSE	Vosa et al., 2018	Blood
PLCG1	ENSG00000124181	20	39795513	rs2072967	20	40003221	A	G	0.0297203	-0.0303	0.0246	0.2180584	-0.0899972	0.0106139	2.27E-17	0.336677	0.276211	0.2228767	0.1510372	0.1942222	20	FALSE	Vosa et al., 2018	Blood
RNF114	ENSG00000124226	20	48561688	rs683954	20	48509861	A	G	0.13986	-0.0031	0.0161	0.8473141	0.333722	0.014894	3.41E-111	-0.0092892	0.0482456	0.8473196	0.6476047	0.112488	20	FALSE	Vosa et al., 2018	Blood
BTN2A2	ENSG00000124508	6	26389213	rs3799378	6	26404374	G	A	0.227273	0.0035	0.0117	0.7648292	0.351899	0.0113574	8.83E-211	0.00994602	0.0332497	0.7648398	0.000415267	0.2709262	20	FALSE	Vosa et al., 2018	Blood
TREM1	ENSG00000124731	6	41245060	rs4714449	6	41239936	T	C	0.113636	-0.0037	0.0154	0.8101289	-0.794209	0.0132238	0	0.00465872	0.0193905	0.8101304	0.1841454	0.3026593	20	FALSE	Vosa et al., 2018	Blood
KIR2DL1	ENSG00000125498	19	55288518	rs56162245	19	55379326	T	A	0.281469	-0.0035	0.0117	0.7648292	0.154276	0.0102258	1.97E-51	-0.0226866	0.075853	0.764874	0.6480384	0.5730026	20	FALSE	Vosa et al., 2018	Blood
C3	ENSG00000125730	19	6704144	rs339392	19	6722022	G	T	0.20979	-0.0143	0.0125	0.2526236	-0.0907926	0.0105123	5.78E-18	0.157502	0.138879	0.2567549	0.2491017	0.6904812	12	FALSE	Vosa et al., 2018	Blood
VASP	ENSG00000125753	19	46020039	rs7252959	19	46033058	A	G	0.276224	-0.0086	0.0109	0.4301174	0.711542	0.0095491	0	-0.0120864	0.0153197	0.4301432	0.0353882	0.3919588	20	FALSE	Vosa et al., 2018	Blood
PSMF1	ENSG00000125818	20	1121464	rs3787573	20	1100669	G	A	0.104895	-0.0034	0.0153	0.8241409	-0.307373	0.0148538	3.99E-95	0.0110615	0.0497795	0.8241509	0.8660415	0.7000919	17	FALSE	Vosa et al., 2018	Blood
RBCK1	ENSG00000125826	20	399876	rs6139109	20	391025	C	T	0.106643	-0.0163	0.0156	0.2960822	0.569514	0.0118287	0	-0.0286209	0.0273982	0.2961959	0.09967512	0.1784345	20	FALSE	Vosa et al., 2018	Blood
PSMB2	ENSG00000126067	1	36087315	rs145444909	1	35661148	A	C	0.00174825	-0.1911	0.1452	0.1881352	-0.252243	0.0240456	9.58E-26	0.757601	0.580147	0.191594	0.5246851	0.08415686	3	FALSE	Vosa et al., 2018	Blood
HECTD3	ENSG00000126107	1	45472606	rs184495791	1	45713753	T	C	0.0227273	0.0488	0.044	0.267391	-0.462086	0.0396443	2.14E-31	-0.105608	0.0956505	0.2695481	0.4439509	0.3346533	11	FALSE	Vosa et al., 2018	Blood
KLC1	ENSG00000126214	14	104098060	rs2403197	14	104053764	T	C	0.258741	0.0067	0.0108	0.535014	0.340022	0.00996526	3.63E-255	0.0197046	0.0317679	0.5350815	0.01720556	0.3070526	20	FALSE	Vosa et al., 2018	Blood
HCST	ENSG00000126264	19	36394330	rs7252902	19	36390252	C	G	0.00874126	-0.0623	0.0513	0.2245855	-0.116809	0.0164127	1.10E-12	0.533349	0.445526	0.2312591	0.3857503	0.09079827	6	FALSE	Vosa et al., 2018	Blood
CANX	ENSG00000127022	5	179131777	rs13182141	5	179126632	C	T	0.486014	-0.0003	0.0102	0.9765362	0.197887	0.00889219	1.03E-109	-0.001516	0.0515447	0.9765362	0.7429421	0.6697677	20	FALSE	Vosa et al., 2018	Blood
FBXL12	ENSG00000127452	19	9929717	rs11085311	19	9909567	A	G	0.227273	-0.0004	0.0121	0.9736285	-0.125644	0.00949357	5.54E-40	0.00318361	0.0963045	0.9736285	0.9849022	0.8989435	20	FALSE	Vosa et al., 2018	Blood
UBR4	ENSG00000127481	1	19468885	rs10799710	1	19483209	A	G	0.566434	0.0265	0.0095	0.00527938	0.118883	0.00899741	7.38E-40	0.222909	0.081672	0.00634662	0.01127687	0.2461948	20	FALSE	Vosa et al., 2018	Blood
FBXL16	ENSG00000127585	16	749164	rs11643113	16	694174	C	T	0.256993	-0.0055	0.0112	0.6233759	-0.121097	0.00894341	9.03E-42	0.045418	0.0925484	0.6236041	0.05696333	0.2529944	19	FALSE	Vosa et al., 2018	Blood
TUBA4A	ENSG00000127824	2	220128662	rs11552796	2	220108300	C	T	0.0996503	-0.0027	0.0155	0.8617134	-0.0889864	0.0120535	1.55E-13	0.0303417	0.174232	0.8617514	0.3492557	0.4658161	12	FALSE	Vosa et al., 2018	Blood
HERC2	ENSG00000128731	15	28461742	rs8182077	15	28467970	A	G	0.0856643	0.0136	0.0234	0.561108	0.730191	0.0119534	0	0.0186253	0.0320479	0.5611257	0.05780186	0.00456956	10	FALSE	Vosa et al., 2018	Blood
ANAPC13	ENSG00000129055	3	134201053	rs4498009	3	134198008	C	G	0.321678	0.0056	0.01	0.5754794	-0.729542	0.00886985	0	-0.007676	0.0137075	0.5754883	0.2813287	0.1062642	20	FALSE	Vosa et al., 2018	Blood
AP1M2	ENSG00000129354	19	10690669	rs73018643	19	10688969	C	G	0.167832	0.0167	0.0124	0.178053	0.276475	0.00922738	3.03E-197	0.0604033	0.0448956	0.178491	0.003009187	0.06076582	20	FALSE	Vosa et al., 2018	Blood
SIGLEC9	ENSG00000129450	19	51634036	rs12983058	19	51642784	T	G	0.318182	0.0174	0.013	0.180746	0.178293	0.00953488	5.04E-78	0.0975923	0.0731003	0.1818614	0.1245137	0.07313942	3	FALSE	Vosa et al., 2018	Blood
CDC16	ENSG00000130177	13	115019280	rs9590404	13	114999059	C	G	0.253497	0.0028	0.0119	0.8139805	0.872258	0.00767861	0	0.00321006	0.0136428	0.8139809	0.3084648	0.3112486	20	FALSE	Vosa et al., 2018	Blood
NECTIN2	ENSG00000130202	19	45370958	rs4803767	19	45372959	T	C	0.298951	-0.0049	0.011	0.6559913	0.785777	0.00920266	0	-0.0062359	0.0139991	0.6559957	0.1847091	0.2611781	20	FALSE	Vosa et al., 2018	Blood
UBAC1	ENSG00000130560	9	138839020	rs62585226	9	138826100	C	T	0.162587	-0.0107	0.0136	0.4314196	-0.302545	0.0110926	8.51E-164	0.0353666	0.0449706	0.4316112	0.9561311	0.6170134	17	FALSE	Vosa et al., 2018	Blood
UBE2M	ENSG00000130725	19	59068703	rs3794964	19	59070916	C	T	0.265734	0.0006	0.012	0.9601224	0.122833	0.00944537	1.15E-38	0.00488467	0.0976942	0.9601227	0.9298929	0.8520516	20	FALSE	Vosa et al., 2018	Blood
LILRB2	ENSG00000131042	19	54781357	rs12984962	19	54769205	C	T	0.325175	-0.0182	0.0127	0.1518376	-0.240218	0.0108445	1.02E-108	0.0757644	0.0529791	0.1526937	0.112938	0.5909579	20	FALSE	Vosa et al., 2018	Blood
NFATC1	ENSG00000131196	18	77222590	rs28723685	18	77204231	T	G	0.211538	0.0014	0.0119	0.9063473	0.19215	0.0100531	1.95E-81	0.00728598	0.061932	0.9063491	0.7476826	0.3975197	20	FALSE	Vosa et al., 2018	Blood
KIF3A	ENSG00000131437	5	132050825	rs7731422	5	132075663	C	A	0.0891608	0.0178	0.0166	0.2835902	-0.0889079	0.00929279	1.10E-21	-0.200207	0.187879	0.2865967	0.3177243	0.4315601	10	FALSE	Vosa et al., 2018	Blood
ACTR10	ENSG00000131966	14	58684274	rs17832891	14	58678057	A	G	0.041958	-0.0912	0.0328	0.00542773	-0.699263	0.0276999	1.31E-140	0.130423	0.0471902	0.00571371	0.020989	0.1823135	19	FALSE	Vosa et al., 2018	Blood
FBXW9	ENSG00000132004	19	12803162	rs45478502	19	12802153	A	C	0.0734266	-0.0349	0.0254	0.1694368	0.22075	0.0242088	7.61E-20	-0.158097	0.116361	0.1742487	0.2496117	0.07371654	19	FALSE	Vosa et al., 2018	Blood
LRRC41	ENSG00000132128	1	46748074	rs78378261	1	46897560	T	C	0.027972	0.0342	0.0404	0.3972546	-0.398479	0.0494011	7.25E-16	-0.0858264	0.101942	0.3998378	0.2032555	0.5942655	13	FALSE	Vosa et al., 2018	Blood
RAF1	ENSG00000132155	3	12665412	rs4684871	3	12697498	G	A	0.472028	-0.0044	0.0101	0.6630954	0.157681	0.009299	1.71E-64	-0.0279044	0.0640744	0.6631996	0.2975915	0.00536167	20	FALSE	Vosa et al., 2018	Blood
UBE2G1	ENSG00000132388	17	4221238	rs12944289	17	4254687	A	G	0.0192308	0.0472	0.0428	0.2701124	0.467172	0.0457331	1.70E-24	0.101033	0.0921473	0.2728894	0.2484508	0.2393391	10	FALSE	Vosa et al., 2018	Blood
FBXO44	ENSG00000132879	1	11718908	rs1883575	1	11713166	A	G	0.449301	0.0087	0.0108	0.4204992	0.133429	0.00910508	1.26E-48	0.065203	0.0810638	0.4211999	0.366032	0.03255785	11	FALSE	Vosa et al., 2018	Blood
DCTN4	ENSG00000132912	5	150113336	rs74600056	5	150090164	G	T	0.0699301	-0.0064	0.0224	0.775097	-0.218039	0.0242093	2.13E-19	0.0293526	0.102786	0.775207	0.9247838	0.7883395	12	FALSE	Vosa et al., 2018	Blood
MKRN1	ENSG00000133606	7	140166104	rs28784908	7	140170395	C	A	0.117133	0.0212	0.0159	0.1824224	-0.0890986	0.0100939	1.08E-18	-0.237939	0.180478	0.1873772	0.1686679	0.08852761	16	FALSE	Vosa et al., 2018	Blood
SFTPD	ENSG00000133661	10	81719933	rs2758549	10	81728870	A	G	0.398601	0.0012	0.0098	0.9025435	0.323983	0.00876428	3.98E-299	0.0037039	0.0302487	0.902544	0.69483	0.2704316	20	FALSE	Vosa et al., 2018	Blood
KRAS	ENSG00000133703	12	25380796	rs12581706	12	25335856	A	T	0.561189	-0.0277	0.0096	0.00390896	0.0713001	0.0089591	1.74E-15	-0.388499	0.143218	0.00667516	0.01008796	0.1425489	20	FALSE	Vosa et al., 2018	Blood
VHL	ENSG00000134086	3	10188298	rs1642729	3	10187377	A	G	0.131119	-0.0215	0.0149	0.1490337	0.184602	0.0118011	3.72E-55	-0.116467	0.0810567	0.1507598	0.02105734	0.09969717	19	FALSE	Vosa et al., 2018	Blood
PTPN22	ENSG00000134242	1	114385407	rs2884603	1	114427450	G	A	0.346154	0.015	0.0101	0.1375045	0.172518	0.00899823	6.29E-82	0.0869476	0.0587201	0.1386832	0.02105212	0.1017557	20	FALSE	Vosa et al., 2018	Blood
CD101	ENSG00000134256	1	117561774	rs12130298	1	117544331	C	A	0.0681818	-0.0033	0.0217	0.8791288	-1.2513	0.0239427	0	0.00263726	0.017342	0.8791293	0.05793525	0.3303867	20	FALSE	Vosa et al., 2018	Blood
KLRC1	ENSG00000134545	12	10601073	rs11053802	12	10597207	C	T	0.323427	-0.0126	0.0108	0.243345	0.414541	0.00858191	0	-0.0303951	0.0260605	0.2434825	0.6693216	0.3414017	20	FALSE	Vosa et al., 2018	Blood
RNF138	ENSG00000134758	18	29691671	rs16962699	18	29692874	A	G	0.0437063	-0.004	0.0233	0.863694	-0.592399	0.0153424	0	0.0067522	0.039332	0.8636953	0.5668098	0.4704231	20	FALSE	Vosa et al., 2018	Blood
TPP2	ENSG00000134900	13	103290437	rs4772494	13	103256296	G	A	0.613636	0.006	0.0102	0.5563744	0.450459	0.00900887	0	0.0133198	0.0226452	0.5564017	0.009242789	0.01556718	20	FALSE	Vosa et al., 2018	Blood
CTSL	ENSG00000135047	9	90343371	rs2378756	9	90337671	G	C	0.461538	0.0142	0.0099	0.1514743	-0.551835	0.00826839	0	-0.0257323	0.0179443	0.1515688	0.199192	0.4157323	20	FALSE	Vosa et al., 2018	Blood
FBXO21	ENSG00000135108	12	117604741	rs4766834	12	117640545	C	A	0.47028	0.0053	0.0103	0.6068584	0.0860004	0.0089364	6.36E-22	0.0616276	0.119938	0.6073715	0.8897184	0.920939	20	FALSE	Vosa et al., 2018	Blood
CD36	ENSG00000135218	7	80153742	rs7755	7	80306271	A	G	0.424825	-0.0037	0.0098	0.7057642	-0.689182	0.00808176	0	0.00536868	0.0142199	0.7057669	0.5521269	0.4207042	20	FALSE	Vosa et al., 2018	Blood
MAP3K7	ENSG00000135341	6	91260028	rs791069	6	91217548	T	C	0.370629	0.0115	0.0108	0.2869597	-0.191917	0.0106281	6.67E-73	-0.0599216	0.056372	0.2877963	0.7604862	0.1962766	20	FALSE	Vosa et al., 2018	Blood
DYNC1LI2	ENSG00000135720	16	66770248	rs2305311	16	66759682	A	C	0.423077	-0.0053	0.0096	0.5808913	-0.155846	0.00892396	2.70E-68	0.0340079	0.0616299	0.5810801	0.9453332	0.9058337	20	FALSE	Vosa et al., 2018	Blood
LMO7	ENSG00000136153	13	76314287	rs17706332	13	76323507	A	G	0.143357	-0.0157	0.0158	0.3203831	-0.116213	0.0172978	1.84E-11	0.135097	0.137436	0.3256185	0.5989005	0.3255821	13	FALSE	Vosa et al., 2018	Blood
RAC1	ENSG00000136238	7	6428881	rs836528	7	6468073	C	G	0.136364	-0.026	0.0153	0.08925393	0.241723	0.0103742	4.39E-120	-0.107561	0.0634638	0.09010485	0.003398643	0.1362244	20	FALSE	Vosa et al., 2018	Blood
BLK	ENSG00000136573	8	11386811	rs4840568	8	11351019	A	G	0.262238	0.0109	0.011	0.32173	-0.434204	0.00857443	0	-0.0251034	0.0253386	0.3218226	0.000727356	0.03258953	20	FALSE	Vosa et al., 2018	Blood
TLR4	ENSG00000136869	9	120472879	rs2770150	9	120463139	G	A	0.255245	0.0106	0.0111	0.3396005	-0.745882	0.0115281	0	-0.0142114	0.0148833	0.3396531	0.4561239	0.1966591	20	FALSE	Vosa et al., 2018	Blood
PSMB7	ENSG00000136930	9	127146734	rs13467	9	127114489	G	A	0.0384615	0.0005	0.0304	0.9868775	-0.220547	0.0117636	2.00E-78	-0.0022671	0.137839	0.9868775	0.7528123	0.4946543	20	FALSE	Vosa et al., 2018	Blood
DCTN3	ENSG00000137100	9	34617031	rs72737122	9	34602784	A	G	0.0909091	0.0196	0.0168	0.243345	-0.405435	0.0224735	9.35E-73	-0.0483431	0.0415235	0.2443289	0.003615671	0.5243301	20	FALSE	Vosa et al., 2018	Blood
KLC4	ENSG00000137171	6	43025676	rs75664886	6	43039403	A	G	0.034965	-0.0079	0.0265	0.7656168	0.357367	0.0341286	1.17E-25	-0.0221061	0.0741834	0.7657089	0.7741691	0.3691906	20	FALSE	Vosa et al., 2018	Blood
TUBB2A	ENSG00000137267	6	3155831	rs9392465	6	3162378	A	C	0.681818	0.0051	0.0109	0.6398633	-0.596583	0.00813605	0	-0.0085487	0.0182711	0.6398701	0.6785826	0.2155316	20	FALSE	Vosa et al., 2018	Blood
TUBB2B	ENSG00000137285	6	3228229	rs9328145	6	3144908	A	G	0.697552	0.0137	0.011	0.2129647	0.154385	0.00889662	1.87E-67	0.0887393	0.0714338	0.214141	0.3289312	0.3902203	20	FALSE	Vosa et al., 2018	Blood
RNF144B	ENSG00000137393	6	18418942	rs6941991	6	18396689	C	T	0.608392	-0.0066	0.0102	0.5175939	0.333541	0.00882246	0	-0.0197877	0.0305854	0.5176552	0.8869167	0.4702486	20	FALSE	Vosa et al., 2018	Blood

TLR2	ENSG00000137462	4	154624751	rs1439164	4	154622649	C	T	0.20979	0.0083	0.0123	0.4998049	0.118491	0.0113539	1.69E-25	0.0700473	0.104022	0.5006989	0.2959203	0.6499752	20	FALSE	Vosa et al., 2018	Blood
PPP2R1B	ENSG00000137713	11	111617391	rs1788955	11	111630998	C	G	0.326923	-0.0152	0.0132	0.2495204	0.289617	0.0100586	2.63E-182	-0.052483	0.0456138	0.2498987	0.5575104	0.1092098	20	FALSE	Vosa et al., 2018	Blood
ACTR1A	ENSG00000138107	10	104250734	rs13287	10	104240438	G	A	0.300699	0.0133	0.0099	0.1791314	-0.173009	0.00948452	2.43E-74	-0.0768746	0.0573774	0.1803085	0.007321339	0.1209438	20	FALSE	Vosa et al., 2018	Blood
KIF11	ENSG00000138160	10	94384096	rs17875345	10	94413667	A	G	0.0367133	0.0642	0.0253	0.01116316	0.196867	0.0228331	6.58E-18	0.326108	0.133963	0.01492021	0.3551713	0.4014078	6	FALSE	Vosa et al., 2018	Blood
HECW2	ENSG00000138411	2	197261196	rs2045277	2	197106759	T	C	0.0681818	-0.0059	0.0192	0.7586214	-0.331124	0.011417	6.07E-185	0.0178181	0.0579876	0.7586345	0.6449476	0.08344147	20	FALSE	Vosa et al., 2018	Blood
ITGAV	ENSG00000138448	2	187500210	rs10174098	2	187492742	G	A	0.339161	0.0027	0.0104	0.7951605	0.191934	0.00924081	8.05E-96	0.0140674	0.0541897	0.7951761	0.99876	0.8891866	20	FALSE	Vosa et al., 2018	Blood
HERC3	ENSG00000138641	4	89535946	rs1020593	4	89612444	T	C	0.783217	-0.0214	0.0119	0.07212642	-0.144746	0.0092646	5.03E-55	0.147845	0.0827555	0.07401449	0.09384459	0.00438938	20	FALSE	Vosa et al., 2018	Blood
HERC6	ENSG00000138642	4	89332077	rs79719417	4	89295921	C	T	0.0769231	0.0023	0.0223	0.9178526	0.769512	0.0254733	1.83E-200	0.00298891	0.0289796	0.9178531	0.09180114	0.02476012	20	FALSE	Vosa et al., 2018	Blood
SEC31A	ENSG00000138674	4	83781066	rs13118857	4	83808854	T	C	0.263986	0.0165	0.0125	0.186835	-0.073822	0.0105388	2.47E-12	-0.223511	0.172306	0.1945729	0.09605367	0.5611395	19	FALSE	Vosa et al., 2018	Blood
CENPE	ENSG00000138778	4	104073264	rs2623063	4	104055722	A	G	0.206294	-0.0003	0.0116	0.9793674	0.0913569	0.0121634	5.88E-14	-0.0032838	0.126975	0.9793675	0.3122099	0.05820131	20	FALSE	Vosa et al., 2018	Blood
SEC24B	ENSG00000138802	4	110408490	rs68041341	4	110453021	T	A	0.15035	-0.0155	0.0159	0.3296383	0.0788864	0.0122093	1.04E-10	-0.196485	0.203837	0.3350794	0.3441807	0.7164047	12	FALSE	Vosa et al., 2018	Blood
PPP3CA	ENSG00000138814	4	102107000	rs2044044	4	102333865	A	G	0.276224	0.0031	0.0105	0.767812	-0.143232	0.00937136	9.78E-53	-0.0216432	0.0733214	0.7678541	0.1121319	0.2082424	20	FALSE	Vosa et al., 2018	Blood
KLRG1	ENSG00000139187	12	9132998	rs4883209	12	9176780	C	T	0.316434	0.0016	0.0101	0.8741291	-0.464498	0.0091216	0	-0.0034446	0.021744	0.8741297	0.09520039	0.3773437	20	FALSE	Vosa et al., 2018	Blood
PIANP	ENSG00000139200	12	6806462	rs117533088	12	6818252	T	C	0.0821678	0.01	0.0239	0.6756473	0.159776	0.019299	1.24E-16	0.0625876	0.149775	0.6760373	0.6080099	0.5287667	8	FALSE	Vosa et al., 2018	Blood
ITGB7	ENSG00000139626	12	53593096	rs12232003	12	53593632	C	T	0.0629371	0.0209	0.0188	0.2662662	0.564763	0.0113609	0	0.0370067	0.0332966	0.2663858	0.2245427	0.135427	20	FALSE	Vosa et al., 2018	Blood
UBE2Q2	ENSG00000140367	15	76164520	rs78911138	15	76275641	T	G	0.0541958	0.023	0.0238	0.3338508	-0.629919	0.0287884	3.94E-106	-0.0365126	0.0378195	0.3343218	0.5074759	0.5758291	20	FALSE	Vosa et al., 2018	Blood
DET1	ENSG00000140543	15	89072348	rs12915845	15	89042467	T	C	0.43007	0.0075	0.0097	0.4394065	0.302909	0.0097871	2.56E-210	0.0247599	0.0320328	0.4395492	0.178345	0.00705056	20	FALSE	Vosa et al., 2018	Blood
NPEPPS	ENSG00000141279	17	45650474	rs10432035	17	45624913	G	C	0.503497	-0.0033	0.0117	0.7779042	-0.198455	0.00888261	1.44E-110	0.0166284	0.05896	0.7779214	0.5675335	0.1723795	20	FALSE	Vosa et al., 2018	Blood
OSBPL1A	ENSG00000141447	18	21859926	rs10775505	18	21743491	T	C	0.631119	0.001	0.0099	0.9195424	-0.307986	0.00887456	6.77E-264	-0.0032469	0.0321444	0.9195428	0.680727	0.3675153	20	FALSE	Vosa et al., 2018	Blood
FBXO15	ENSG00000141665	18	71777844	rs3813114	18	71815122	A	G	0.0716783	0.0038	0.0185	0.8372554	-0.322762	0.0147789	9.80E-106	-0.0117734	0.0573203	0.8372625	0.9277894	0.9557279	20	FALSE	Vosa et al., 2018	Blood
VAV1	ENSG00000141968	19	6815051	rs56408517	19	6759845	G	T	0.141608	-0.0032	0.0144	0.8241409	-0.0976455	0.0151248	1.08E-10	0.0327716	0.14756	0.8242433	0.9768528	0.8464084	8	FALSE	Vosa et al., 2018	Blood
AKT1	ENSG00000142208	14	105248887	rs45490496	14	105272678	T	A	0.631119	0.0097	0.0104	0.3509789	0.260819	0.00909143	5.32E-181	0.0371906	0.0398955	0.3512333	0.307683	0.1083978	20	FALSE	Vosa et al., 2018	Blood
PSMB6	ENSG00000142507	17	4700614	rs2286672	17	4712617	T	C	0.0821678	-0.0362	0.0178	0.04198106	0.131823	0.0119976	4.39E-28	-0.274611	0.137324	0.04552826	0.1278108	0.4236238	20	FALSE	Vosa et al., 2018	Blood
SIGLEC10	ENSG00000142512	19	51917166	rs35935019	19	51912018	C	T	0.152098	0.001	0.0151	0.9471986	-0.240435	0.0103272	6.78E-120	-0.0041591	0.0628031	0.9471988	0.7860296	0.5726345	20	FALSE	Vosa et al., 2018	Blood
BCL10	ENSG00000142867	1	85737352	rs11161570	1	85721911	T	C	0.508741	0.0064	0.0099	0.5179785	-0.295478	0.00876213	2.73E-249	-0.0216598	0.0335112	0.5180554	0.3191362	0.08981384	20	FALSE	Vosa et al., 2018	Blood
CTSK	ENSG00000143387	1	150774741	rs2089081	1	150800117	C	T	0.519231	0.0244	0.0094	0.00943862	-0.690225	0.00801452	0	-0.0353508	0.0136249	0.00947103	0.01075732	0.1041395	20	FALSE	Vosa et al., 2018	Blood
ARF1	ENSG00000143761	1	228278636	rs964941	1	228193857	G	A	0.444056	-0.0213	0.0096	0.02650374	0.0667856	0.00893501	7.74E-14	-0.318931	0.149943	0.03341852	0.02967317	0.660646	20	FALSE	Vosa et al., 2018	Blood
DYNC1LI1	ENSG00000144635	3	32589914	rs9852881	3	32621359	A	C	0.402098	0.0018	0.0122	0.882705	0.49204	0.00889868	0	0.00365824	0.0247948	0.8827054	0.6834253	0.5241697	20	FALSE	Vosa et al., 2018	Blood
UBA3	ENSG00000144744	3	69116720	rs3853156	3	69126495	T	C	0.409091	-0.0037	0.0102	0.7167953	0.0781159	0.00916509	1.55E-17	-0.0473655	0.130693	0.7170404	0.07101025	0.109161	20	FALSE	Vosa et al., 2018	Blood
TRPC1	ENSG00000144935	3	142484823	rs9849265	3	142435388	C	T	0.435315	-0.0031	0.0099	0.7541809	-0.0905176	0.00921749	9.22E-23	0.0342475	0.109427	0.7543017	0.4466326	0.226022	20	FALSE	Vosa et al., 2018	Blood
SKP2	ENSG00000145604	5	36168256	rs3804439	5	36179044	C	T	0.132867	-0.0237	0.0156	0.1287044	0.356248	0.0102342	1.74E-265	-0.0665267	0.0438314	0.1290684	0.06904513	0.2439687	20	FALSE	Vosa et al., 2018	Blood
PIK3R1	ENSG00000145675	5	67554598	rs3756668	5	67596088	G	A	0.571678	-0.0166	0.0094	0.07740299	-0.136415	0.00896394	2.68E-52	0.121687	0.0693698	0.07939862	0.4542759	0.2437176	20	FALSE	Vosa et al., 2018	Blood
KLHL3	ENSG00000146021	5	137012484	rs17797119	5	137070445	G	A	0.354895	-0.0133	0.01	0.1835183	0.2432	0.00919237	3.05E-154	-0.0546874	0.0411703	0.1840715	0.009333939	0.03137775	20	FALSE	Vosa et al., 2018	Blood
TRIM41	ENSG00000146063	5	180656154	rs2259749	5	180652241	C	T	0.22028	-0.02	0.0118	0.0900915	-0.160999	0.00895775	3.16E-72	0.124224	0.0736174	0.09152094	0.05268637	0.5313457	20	FALSE	Vosa et al., 2018	Blood
NFKBIE	ENSG00000146232	6	44229701	rs28385699	6	44226433	T	C	0.041958	0.0653	0.0272	0.01636217	-0.9388	0.0328195	5.83E-180	-0.0695568	0.029075	0.01674203	0.4207522	0.6267995	12	FALSE	Vosa et al., 2018	Blood
RNF217	ENSG00000146373	6	125348735	rs4896619	6	125281126	G	A	0.167832	-0.007	0.0141	0.6195742	0.212493	0.0127266	1.38E-62	-0.0329422	0.0663844	0.6197289	0.4981265	0.4151187	20	FALSE	Vosa et al., 2018	Blood
TRIM4	ENSG00000146833	7	99495902	rs2572010	7	99488543	C	T	0.447552	-0.0218	0.0095	0.02174821	-0.818767	0.00733362	0	0.0266254	0.0116053	0.02177601	0.01897799	0.3546839	20	FALSE	Vosa et al., 2018	Blood
FBXO10	ENSG00000147912	9	37549880	rs10814577	9	37562721	C	T	0.274476	-0.0137	0.0106	0.1962004	0.130346	0.00962671	9.08E-42	-0.105105	0.0816919	0.1982321	0.2517485	0.09627485	20	FALSE	Vosa et al., 2018	Blood
ASB6	ENSG00000148331	9	132401807	rs4837393	9	132387665	C	A	0.601399	0.0017	0.0095	0.857979	0.111584	0.00892832	7.67E-36	0.0152351	0.0851461	0.8579934	0.5896699	0.3069189	20	FALSE	Vosa et al., 2018	Blood
LRSAM1	ENSG00000148356	9	130239772	rs10987668	9	130251615	A	C	0.152098	0.0379	0.016	0.01784831	-0.146369	0.0108008	7.75E-42	-0.258935	0.11097	0.01962862	0.3028198	0.00728726	20	FALSE	Vosa et al., 2018	Blood
HERC4	ENSG00000148634	10	69758385	rs4746714	10	69607037	C	T	0.718531	-0.0134	0.0106	0.2061759	-0.0848345	0.00894029	2.33E-21	0.157955	0.126053	0.2101767	0.04708898	0.6718594	20	FALSE	Vosa et al., 2018	Blood
PTPRJ	ENSG00000149177	11	48095891	rs1017875	11	47999218	T	C	0.183566	-0.0062	0.0122	0.6113154	0.249695	0.0110664	9.92E-113	-0.0248303	0.048872	0.6114057	0.06586337	0.3250507	20	FALSE	Vosa et al., 2018	Blood
PAK1	ENSG00000149269	11	77109216	rs3019238	11	77204866	G	A	0.582168	0.015	0.0095	0.1143481	0.221651	0.00922746	1.69E-127	0.067674	0.0429527	0.1151302	0.1770078	0.07651519	20	FALSE	Vosa et al., 2018	Blood
KLRF1	ENSG00000150045	12	9988841	rs1351111	12	10555448	T	C	0.318182	-0.0138	0.0123	0.2618832	0.0579439	0.00896964	1.05E-10	-0.238161	0.215452	0.2689847	0.4884933	0.3018985	18	FALSE	Vosa et al., 2018	Blood
ITGB1	ENSG00000150093	10	33241983	rs1815241	10	33307024	C	T	0.222028	-0.002	0.0119	0.8665304	-0.0718112	0.00950915	4.29E-14	0.0278508	0.165753	0.8665631	0.4798383	0.3271441	20	FALSE	Vosa et al., 2018	Blood
FCGR1A	ENSG00000150337	1	149759150	rs72708153	1	149865483	G	A	0.103147	-0.0009	0.02	0.9641073	0.315264	0.0197608	2.67E-57	-0.0028548	0.0634391	0.9641075	0.06852513	0.01962011	20	FALSE	Vosa et al., 2018	Blood
TIRAP	ENSG00000150455	11	126160850	rs3851111	11	126083218	G	T	0.652098	0.0142	0.01	0.1556077	0.120144	0.00892071	2.41E-41	0.118191	0.0836947	0.1578992	0.2375353	0.03515299	20	FALSE	Vosa et al., 2018	Blood
CD226	ENSG00000150637	18	67563716	rs763361	18	67531642	T	C	0.498252	-0.0124	0.0093	0.1824224	-0.396298	0.00858704	0	0.0312896	0.023477	0.1826049	0.2696668	0.5669383	20	FALSE	Vosa et al., 2018	Blood
SEC24D	ENSG00000150961	4	119701908	rs115294251	4	119772833	T	C	0.0384615	0.0178	0.0209	0.3943947	0.215105	0.0271077	2.10E-15	0.0827504	0.09772	0.3971004	0.1262355	0.2947634	20	FALSE	Vosa et al., 2018	Blood
UBC	ENSG00000150991	12	125399032	rs4617684	12	125406966	A	G	0.00174825	0.072	0.1032	0.4853808	-0.0842798	0.011017	2.01E-14	-0.854298	1.22958	0.4871863	0.7652337			FALSE	Vosa et al., 2018	Blood
ITPR1	ENSG00000150995	3	4712278	rs2322800	3	4512462	A	G	0.0699301	-0.0224	0.0192	0.243345	-0.316164	0.0124217	6.61E-143	0.0708493	0.0607918	0.2438397	0.3115873	0.3155295	20	FALSE	Vosa et al., 2018	Blood
UBE3B	ENSG00000151148	12	109944857	rs17181453	12	109937092	G	A	0.0769231	0.0152	0.0196	0.4380382	1.03806	0.0205804	0	0.0146427	0.0188836	0.4380924	0.000471368	0.03890061	20	FALSE	Vosa et al., 2018	Blood
FBXO4	ENSG00000151876	5	41933600	rs604472	5	41696934	G	A	0.0594406	0.0193	0.0211	0.3603534	-0.12008	0.0146343	2.30E-16	-0.160726	0.176804	0.3633179	0.828999	0.428359	17	FALSE	Vosa et al., 2018	Blood
AP1S3	ENSG00000152056	2	224659573	rs6436440	2	224704906	G	A	0.513986	0.0039	0.0094	0.6782198	-0.170991	0.00891144	4.68E-82	-0.0228082	0.0549865	0.6782908	0.8943508	0.8682385	20	FALSE	Vosa et al., 2018	Blood
TRIM36	ENSG00000152503	5	114488351	rs7725831	5	114545170	A	G	0.456294	-0.0025	0.0103	0.8082235	0.261492	0.00946149	3.95E-168	-0.0095605	0.0393909	0.8082308	0.182168	0.8045228	20	FALSE	Vosa et al., 2018	Blood
RASGRP3	ENSG00000152689	2	33725604	rs13404493	2	33756520	T	C	0.115385	-0.0211	0.0169	0.2118404	0.316642	0.0118586	4.53E-157	-0.0666369	0.053431	0.2123395	0.04690489	0.3561129	20	FALSE	Vosa et al., 2018	Blood
SAR1B	ENSG00000152700	5	133960897	rs246347	5	134096294	T	C	0.176573	0.0248	0.0131	0.05834061	-0.331952	0.0151899	7.22E-106	-0.0747097	0.0396114	0.05928606	7.83E-08	0.04403147	20	TRUE	Vosa et al., 2018	Blood
ANAPC1	ENSG00000153107	2	112583057	rs7563616	2	112637504	T	C	0.347902	0.0108	0.0106	0.3082657	0.12775	0.00895627	3.68E-46	0.0845398	0.0831857	0.3094968	0.382553	0.7473406	20	FALSE	Vosa et al., 2018	Blood
CD96	ENSG00000153283	3	111322761	rs6771309	3	111343919	C	T	0.183566	0.0092	0.013	0.4791364	0.459994	0.00988364	0	0.0200003	0.0282645	0.4791872	0.2328936	0.9294118	20	FALSE	Vosa et al., 2018	Blood
CD8A	ENSG00000153563	2	87023624	rs2944250	2	87017069	G	C	0.227273	-0.0021	0.0114	0.8538483	-0.217886	0.0104732	3.97E-96	0.00963806	0.0523229	0.853854	0.9930284	0.956164	20	FALSE	Vosa et al., 2018	Blood
TRIP12	ENSG00000153827	2	230709942	rs4331494	2	230575319	A	G	0.23951	-0.0011	0.0127	0.9309782	0.241235	0.0112031	7.68E-103	-0.0045599	0.0526462	0.9309788	0.5762662	0.3136429	20	FALSE	Vosa et al., 2018	Blood
PPP2R5E	ENSG00000154001	14	63924083	rs28571942	14	63847327	T	C	0.0716783	0.0027	0.018	0.8807646	0.107195	0.0100871	2.23E-26	0.0251877	0.167935	0.8807764	0.9907682	0.6286224	18	FALSE	Vosa et al., 2018	Blood
LONRF1	ENSG00000154359	8	12596492	rs67858908	8	12621916	G	T	0.129371	-0.0008	0.0148	0.9568921	-0.296871	0.0181615	4.63E-60	0.00269477	0.0498536	0.9568923	0.2506276	0.05587438	20	FALSE	Vosa et al., 2018	Blood
TRIM11	ENSG00000154370	1	228587957	rs12072922	1	228687796	A	G	0.0909091	-0.0127	0.0156	0.4155862	0.149762	0.0200013	7.01E-14	-0.0848013	0.104779	0.4183237	0.199881	0.1590261	20	FALSE	Vosa et al., 2018	Blood
SH3RF1	ENSG00000154447	4	170103831	rs10013658	4	170215011	C	A	0.47028	0.0185	0.01	0.06431355	-0.259563	0.00882844	5.37E-190	-0.0712736	0.0386025	0.06484163	5.80E-06	3.94E-06	20	TRUE	Vosa et al., 2018	Blood
LY96	ENSG00000154589	8	74922454	rs10957691	8	74906729	G	A	0.143357	-0.0416	0.0138	0.00257409	0.538593	0.010991	0	-0.0772383	0.0256707	0.00262277	0.1456008	0.3695669	20	FALSE	Vosa et al., 2018	Blood
FBXL18	ENSG00000155034	7	5512197	rs4724703	7	5531559	T	C	0.201049	-0.0148	0.012	0.2174514	0.0876428	0.0105419	9.27E-17	-0.168867	0.138418	0.2224716	0.0329179	0.4767243	20	FALSE	Vosa et al., 2018	Blood
PIK3AP1	ENSG00000155629	10	98416670	rs12571935	10	98508321	A	G	0.0594406	0.0041	0.0202	0.8391582	0.157432	0.0124007	6.27E-37	0.0260429	0.128326	0.8391785	0.5224971	0.5447338	19	FALSE	Vosa et al., 2018	Blood
UBE2L6	ENSG00000156587	11	57327443	rs2234412	11	57320044	T	C	0.43007	-0.015	0.0095	0.1143481	0.160474	0.00906286	3.72E-70	-0.0934729	0.0594344	0.1157867	0.09982762	0.04486782	20	FALSE	Vosa et al., 2018	Blood
FBXO32	ENSG00000156804	8	124531787	rs2280919	8	124551779	A	G	0.405594	-0.0063	0.0097	0.5160252	0.170838	0.00902758	7.23E-80	-0.0368771	0.0568124	0.5162722	0.5855525	0.4200736	20	FALSE	Vosa et al., 2018	Blood
SEC13	ENSG00000157020	3	10348838	rs696221	3	10342876	C	T	0.456294	-0.0027	0.0096	0.7785187	0.502759	0.00972951	0	-0.0053704	0.0190949	0.7785219	0.2353582	0.06292348	20	FALSE	Vosa et al., 2018	Blood
RNF111	ENSG00000157450	15	59273496	rs4775175	15	59816688	C	A	0.589161	0.0082	0.0101	0.4168598	0.0594323	0.00906782	5.59E-11	0.137972	0.17124	0.4204025	0.4684841	0.1848298	4	FALSE	Vosa et al., 2018	Blood
TNFRSF14	ENSG00000157873	1	2491949	rs3748825	1	2483270	T	C	0.508741	-0.004	0.0097	0.6800674	-0.374085	0.00866803	0	0.0106928	0.0259311	0.6800812	0.8310308	0.3930593	20	FALSE	Vosa et al., 2018	Blood
NCK1	ENSG00000158092	3	136624857	rs80201571	3	136659779	T	G	0.251748	-0.0124	0.0118	0.2933287	-0.142786	0.0122578	2.33E-31	0.0868431	0.0829766	0.2952853	0.1256721	0.101332	20	FALSE	Vosa et al., 2018	Blood
COLEC12	ENSG00000158270	18	410041	rs2846667	18	466810	T	G	0.269231	0.012	0.0115	0.2967268	-0.293314	0.0117125	2.09E-138	-0.0409118	0.0392412	0.2971458	0.08511419	0.2386676	20	FALSE	Vosa et al., 2018	Blood
CD1D	ENSG00000158473	1	158152211	rs4657080	1	158436299	C	T	0.594406	-0.0022	0.0098	0.8223762	-0.0993107	0.00912598	1.40E-27	0.0221527	0.0987012	0.8224134	0.6618529	0.6105546	20	FALSE	Vosa et al., 2018	Blood
CD1A	ENSG00000158477	1	158225993	rs3181030	1	158220291	C	T	0.0786713	-0.0189	0.017	0.2662393	-1.01978	0.0193129	0	0.0185334	0.016674	0.2663453	0.6321246	0.7873238	20	FALSE	Vosa et al., 2018	Blood
CD1C	ENSG00000158481	1	158261498	rs12131763	1	158400990	G	A	0.449301	-0.0079	0.0096	0.4105554	-0.120929	0.00914828	6.83E-40	0.0653274	0.0795389	0.4114602	0.8758573	0.9193552	19	FALSE	Vosa et al., 2018	Blood
CD1B	ENSG00000158485	1	158299531	rs6699757	1	158415517	C	T	0.442308	-0.0098	0.0095	0.3022694	-0.238825	0.00926635	1.76E-146	0.0410342	0.0398099	0.3026564	0.5896886	0.8113011	20	FALSE	Vosa et al., 2018	Blood
NCF1	ENSG00000158517	7	74195984	rs810366	7	74140925	C	G	0.208042	0.0201	0.0133	0.1307176	0.486465	0.0129766	1.46E-307	0.0413185	0.0273623	0.1310303	0.185793	0.089866	16	FALSE	Vosa et al., 2018	Blood
DYNC1I1	ENSG00000158560	7	95570750	rs7801111	7	95401417	A	G	0.277972	0.025	0.0112	0.02560552	-0.11007	0.0108346	3.02E-24	-0.227127	0.10418	0.02924711	0.001552761	0.01474494	20	FALSE	Vosa et al., 2018	Blood
FBXW5	ENSG00000159069	9	139837017	rs10747041	9	139827110	T	C	0.248252	-0.0029	0.0111	0.793891	0.0983485	0.0110487	5.51E-19	-0.029487	0.112913	0.7939778	0.855345	0.7380983	20	FALSE	Vosa et al., 2018	Blood
UBE2Z	ENSG00000159202	17	46996074	rs8082345	17	47027118	C	A	0.173077	-0.0078	0.0127	0.5391009	0.401641	0.00994055	0	-0.0194203	0.0316239	0.5391477	0.03456122	0.00592995	20	FALSE	Vosa et al., 2018	Blood
PSMD4	ENSG00000159352	1	151233567	rs11204798	1	151273726	C	G	0.218531	-0.0115	0.0115	0.3173105	-0.189715	0.00951123	1.61E-88	0.0606173	0.0606934	0.3179179	0.04496366	0.1072681	20	FALSE	Vosa et al., 2018	Blood
PSMB4	ENSG00000159377	1	151373215	rs2296840	1	151372047	T	C	0.00174825	0.0665	0.1083	0.5391921	-0.102566	0.0113081	1.19E-19	-0.64836	1.05832	0.5401191	0.5401191			FALSE	Vosa et al., 2018	Blood
THEM4	ENSG00000159445	1	151864172	rs6685187	1	151858888	G	A	0.594406	-0.0073	0.0097	0.451704	0.700507	0.00808564	0	-0.010421	0.0138476	0.451721	0.7425016	0.6136149	20	FALSE	Vosa et al., 2018	Blood
UBR1	ENSG00000159459	15	43316703	rs62020698	15	43237414	T	C	0.104895	-0.0116	0.0219	0.5963336	-0.275211	0.0213243	4.16E-38	0.0421494	0.0796422	0.5966426	0.05127502	0.00016075	20	FALSE	Vosa et al., 2018	Blood
UBE2J2	ENSG00000160087	1	1199277	rs4018608	1	1203938	C	T	0.0244755	0.0136	0.0275	0.620921	0.0843112	0.00971171	3.91E-18	0.161307	0.326701	0.6214863	0.8978849	0.9684923	8	FALSE	Vosa et al., 2018	Blood
ICOSLG	ENSG00000160223	21	45651861	rs2847224	21	45660835	A	C	0.454545	-0.0014	0.0101	0.8897553	-0.247431	0.00955834	9.48E-148	0.00565814	0.04082	0.8897569	0.3227997	0.2569657	20	FALSE	Vosa et al., 2018	Blood
ITGB2	ENSG00000160255	21	46328886	rs760462	21	46328099	T	C	0.162587	0.0062	0.0133	0.6410971	-0.737529	0.0121647	0	-0.0084065	0.0180337	0.641107	0.1836927	0.693872	20	FALSE	Vosa et al., 2018	Blood

JAML	ENSG00000160593	11	118080132	rs7939622	11	118087841	A	G	0.192308	-0.008	0.0118	0.4977932	0.437768	0.0108104	0	-0.0182745	0.0269587	0.4978534	0.2931596	0.03525325	20	FALSE	Vosa et al., 2018	Blood
CD3G	ENSG00000160654	11	118220467	rs112563022	11	118262135	T	C	0.0437063	-0.0129	0.031	0.6773156	0.163719	0.026496	6.45E-10	-0.0787935	0.189778	0.6780039	0.7232884	0.6910372	10	FALSE	Vosa et al., 2018	Blood
UBE2Q1	ENSG00000160714	1	154526278	rs72698179	1	154522394	A	C	0.0402098	-0.0823	0.0366	0.0245358	0.663015	0.0348724	1.34E-80	-0.12413	0.0555871	0.02554432	0.03011092	0.3301673	20	FALSE	Vosa et al., 2018	Blood
ORAI2	ENSG00000160991	7	102085410	rs2411005	7	102069584	A	G	0.0524476	0.0034	0.0188	0.8564845	-0.797222	0.0168357	0	-0.0042648	0.0235821	0.8564855	0.239032	0.2404465	20	FALSE	Vosa et al., 2018	Blood
FBXL13	ENSG00000161040	7	102584297	rs117274931	7	102618968	A	G	0.0244755	-0.0224	0.0285	0.431888	1.33316	0.0374858	4.97E-277	-0.0168022	0.021383	0.4320005	0.1904496	0.6793951	20	FALSE	Vosa et al., 2018	Blood
PSMC2	ENSG00000161057	7	102997271	rs58447419	7	103059817	G	C	0.027972	-0.0209	0.0365	0.5669137	-0.117799	0.0173484	1.12E-11	0.177421	0.31095	0.568286	0.4999206	0.8211014	6	FALSE	Vosa et al., 2018	Blood
AP2M1	ENSG00000161203	3	183897178	rs73185743	3	183912194	T	C	0.136364	-0.0006	0.0128	0.9626129	-0.500564	0.0179819	1.54E-170	0.00119865	0.0255712	0.9626129	0.1036225	0.334544	20	FALSE	Vosa et al., 2018	Blood
SIGLEC11	ENSG00000161640	19	50458335	rs10405621	19	50455351	T	C	0.276224	-0.0157	0.0119	0.1870596	0.537467	0.01032	0	-0.0292111	0.022148	0.187201	0.01867936	0.8350313	20	FALSE	Vosa et al., 2018	Blood
ASB16	ENSG00000161664	17	42252133	rs228768	17	42191893	G	T	0.311189	-0.0382	0.0106	0.00031363	-0.0885949	0.00904806	1.22E-22	0.431176	0.127492	0.00071964	0.000154025	0.023835	20	FALSE	Vosa et al., 2018	Blood
RACGAP1	ENSG00000161800	12	50398812	rs60042968	12	50381442	G	T	0.0769231	-0.0224	0.0189	0.2359442	0.202438	0.0152142	2.14E-40	-0.110651	0.0937318	0.2377969	0.4415594	0.7043189	20	FALSE	Vosa et al., 2018	Blood
TREML1	ENSG00000161911	6	41119577	rs192887145	6	41043668	A	T	0.00524476	0.1716	0.1358	0.2063654	-1.61932	0.0930107	6.93E-68	-0.105971	0.0840832	0.2075584	0.5881336	0.3173469	4	FALSE	Vosa et al., 2018	Blood
CCNF	ENSG00000162063	16	2494125	rs12932220	16	2500604	C	G	0.386364	0.0059	0.013	0.6499396	0.154857	0.0102377	1.09E-51	0.0380996	0.083986	0.6500865	0.5342581	0.4407669	18	FALSE	Vosa et al., 2018	Blood
KLHL21	ENSG00000162413	1	6662725	rs144349639	1	6658874	C	T	0.00874126	-0.0597	0.0648	0.3568958	-0.645542	0.0630412	1.31E-24	0.0924804	0.100786	0.3588336	0.4627172	0.7422178	6	FALSE	Vosa et al., 2018	Blood
VCAM1	ENSG00000162692	1	101194953	rs12566489	1	101139367	T	C	0.195804	0.015	0.0122	0.2188813	-0.379131	0.0118677	6.04E-224	-0.0395641	0.0322027	0.2192223	0.3593438	0.4968467	20	FALSE	Vosa et al., 2018	Blood
SLAMF6	ENSG00000162739	1	160473936	rs1041067	1	160457127	T	C	0.18007	0.0042	0.0127	0.7408646	-0.213086	0.0107316	9.78E-88	-0.0197103	0.0596086	0.7408992	0.3664847	0.8789644	12	FALSE	Vosa et al., 2018	Blood
REL	ENSG00000162924	2	61129650	rs13014803	2	61167216	C	T	0.15035	-0.0082	0.0133	0.5375373	0.24955	0.0166629	1.05E-50	-0.0328592	0.0533412	0.5378816	0.2389225	0.837966	20	FALSE	Vosa et al., 2018	Blood
FBXO41	ENSG00000163013	2	73496684	rs546576	2	73501453	G	A	0.155594	-0.0026	0.0143	0.8557254	-0.278906	0.0170229	2.48E-60	0.00932214	0.0512749	0.8557342	0.8082337	0.7974441	20	FALSE	Vosa et al., 2018	Blood
CTSS	ENSG00000163131	1	150720492	rs7544631	1	150657281	T	C	0.557692	0.026	0.0095	0.00620321	-0.344869	0.00892987	0	-0.075391	0.0276158	0.00633352	0.008212565	0.5586563	20	FALSE	Vosa et al., 2018	Blood
KBTBD8	ENSG00000163376	3	67055180	rs11710610	3	67148937	C	A	0.162587	-0.0071	0.0131	0.587829	-0.0944818	0.00999575	3.32E-21	0.0751467	0.138879	0.5884415	0.6408814	0.9752709	20	FALSE	Vosa et al., 2018	Blood
TRAT1	ENSG00000163519	3	108557698	rs4507261	3	108538384	G	A	0.0856643	0.0375	0.0151	0.01301189	0.418796	0.0117724	3.49E-277	0.0895423	0.0361435	0.01323382	0.5334498	0.6408685	20	FALSE	Vosa et al., 2018	Blood
CTLA4	ENSG00000163599	2	204735596	rs10197010	2	204740171	C	A	0.178322	0.0055	0.0118	0.6411427	-0.161271	0.0118145	2.01E-42	-0.0341041	0.0732115	0.641337	0.2420236	0.6989834	20	FALSE	Vosa et al., 2018	Blood
ICOS	ENSG00000163600	2	204813885	rs11571313	2	204821605	T	C	0.103147	-0.014	0.0178	0.4315647	-0.228099	0.0175151	9.05E-39	0.0613768	0.0781784	0.4324029	0.7506611	0.102157	15	FALSE	Vosa et al., 2018	Blood
CD200R1	ENSG00000163606	3	112667012	rs7432373	3	112646699	T	C	0.293706	-0.0041	0.0106	0.6989099	0.467477	0.0114841	0	-0.0087705	0.0226759	0.6989228	0.8866196	0.5436805	20	FALSE	Vosa et al., 2018	Blood
PSMD6	ENSG00000163636	3	64002941	rs73119035	3	63993966	G	A	0.148601	-0.0345	0.0144	0.01658263	-0.114004	0.0137447	1.09E-16	0.302621	0.131475	0.02135011	0.03877753	0.2317088	20	FALSE	Vosa et al., 2018	Blood
RCHY1	ENSG00000163743	4	76422110	rs10518140	4	76417784	C	T	0.206294	-0.004	0.0117	0.7324409	0.559096	0.00957423	0	-0.0071544	0.020927	0.7324454	0.01155515	0.301335	20	FALSE	Vosa et al., 2018	Blood
FBXO40	ENSG00000163833	3	121330552	rs1873645	3	121348499	C	T	0.255245	0.0044	0.0111	0.6918126	0.135559	0.00964764	7.60E-45	0.0324582	0.0819157	0.6919289	0.05911457	0.0010056	20	FALSE	Vosa et al., 2018	Blood
DTX3L	ENSG00000163840	3	122288567	rs6796228	3	122295235	C	T	0.215035	-0.0104	0.0118	0.3781252	-0.238607	0.00996878	1.31E-126	0.0435864	0.0494873	0.3784483	0.005614066	0.3328335	20	FALSE	Vosa et al., 2018	Blood
RNF123	ENSG00000164068	3	49742947	rs4855882	3	49715354	G	T	0.178322	0.0165	0.015	0.2713321	0.156159	0.0147076	2.47E-26	0.105661	0.09657	0.2738919	0.08777751	0.02808156	20	FALSE	Vosa et al., 2018	Blood
ERAP1	ENSG00000164307	5	96120162	rs26499	5	96132343	T	C	0.424825	-0.0106	0.0094	0.2594637	0.195517	0.00887205	1.26E-107	-0.0542153	0.0481406	0.2600867	0.3152275	0.2419713	20	FALSE	Vosa et al., 2018	Blood
ERAP2	ENSG00000164308	5	96233531	rs2910686	5	96252589	C	T	0.442308	0.0067	0.0094	0.4759906	1.0246	0.00635385	0	0.00653911	0.00917437	0.4759949	0.5394405	0.00261931	20	FALSE	Vosa et al., 2018	Blood
RICTOR	ENSG00000164327	5	39006265	rs10075480	5	39070619	G	T	0.166084	0.0036	0.0129	0.7801913	0.117637	0.0133563	1.28E-18	0.0306026	0.109714	0.7802987	0.7380491	0.8816671	18	FALSE	Vosa et al., 2018	Blood
CTSB	ENSG00000164733	8	11713495	rs1296022	8	11722355	A	G	0.26049	0.0349	0.0107	0.00110753	-0.438323	0.00924354	0	-0.0796216	0.0244689	0.00113795	0.000102126	5.90E-06	20	FALSE	Vosa et al., 2018	Blood
YWHAZ	ENSG00000164924	8	101947184	rs964917	8	101937455	T	C	0.396853	-0.0093	0.0104	0.3711984	0.2587	0.0088804	1.43E-186	-0.035949	0.0402199	0.3714237	0.3677418	0.3292638	20	FALSE	Vosa et al., 2018	Blood
SYK	ENSG00000165025	9	93612450	rs772407	9	93560850	C	A	0.456294	-0.0322	0.01	0.00128191	-0.45664	0.00850267	0	0.0705151	0.0219384	0.00130796	0.03225512	0.2242992	20	FALSE	Vosa et al., 2018	Blood
HECTD2	ENSG00000165338	10	93222341	rs7100516	10	93257155	A	C	0.0244755	-0.0199	0.0314	0.5262388	0.190392	0.017171	1.43E-28	-0.104521	0.165192	0.5269131	0.2400831	0.6888808	10	FALSE	Vosa et al., 2018	Blood
LRR1	ENSG00000165501	14	50073402	rs2281836	14	50065717	G	C	0.256993	-0.0062	0.0109	0.5694869	-0.488799	0.00850365	0	0.0126841	0.0223006	0.5695058	0.7701464	0.06487456	20	FALSE	Vosa et al., 2018	Blood
BTNL9	ENSG00000165810	5	180477874	rs10063135	5	180432024	G	T	0.351399	0.0006	0.0101	0.9526288	-0.365356	0.00904552	0	-0.0016422	0.0276443	0.9526288	0.8835233	0.827607	20	FALSE	Vosa et al., 2018	Blood
PSMC3	ENSG00000165916	11	47444156	rs61895111	11	47558516	T	C	0.146853	0.0132	0.0133	0.3209628	-0.205311	0.0154858	4.06E-40	-0.0642928	0.0649611	0.3223149	0.2986457	0.07166938	20	FALSE	Vosa et al., 2018	Blood
BTRC	ENSG00000166167	10	103215449	rs11191021	10	103231641	G	A	0.141608	0.0003	0.0144	0.9833786	0.156934	0.0105607	5.97E-50	0.00191163	0.0917585	0.9833786	0.01444346	0.04295535	20	FALSE	Vosa et al., 2018	Blood
CUL5	ENSG00000166266	11	107928981	rs73000548	11	107978149	T	G	0.0559441	-0.0102	0.0238	0.6682351	-1.07146	0.025033	0	0.00951973	0.0222138	0.6682508	0.8485762	0.6541378	20	FALSE	Vosa et al., 2018	Blood
PRKCB	ENSG00000166501	16	24039627	rs7404003	16	23864647	C	A	0.431818	0.0036	0.01	0.7188471	-0.54582	0.00838862	0	-0.0065956	0.0183213	0.7188513	0.4766815	0.1181784	20	FALSE	Vosa et al., 2018	Blood
B2M	ENSG00000166710	15	45007375	rs1690313	15	45005660	G	C	0.0437063	0.0176	0.024	0.4633551	-0.165611	0.0154005	5.70E-27	-0.106273	0.145255	0.4643919	0.6242994	0.7614216	6	FALSE	Vosa et al., 2018	Blood
AP1G1	ENSG00000166747	16	71803008	rs12924650	16	71705908	T	C	0.174825	-0.003	0.0132	0.8202117	-0.180809	0.0156778	9.02E-31	0.0165921	0.0730195	0.820246	0.02111568	0.7848995	20	FALSE	Vosa et al., 2018	Blood
DCTN5	ENSG00000166847	16	23666954	rs34514	16	23670597	A	G	0.195804	0.0158	0.0116	0.1731761	0.989109	0.00810945	0	0.015974	0.0117285	0.1732029	0.5277559	0.8465008	20	FALSE	Vosa et al., 2018	Blood
YWHAB	ENSG00000166913	20	43525745	rs6017437	20	43503276	A	C	0.234266	0.0099	0.0146	0.4977196	0.266356	0.0110998	3.03E-127	0.0371683	0.0548357	0.4978911	0.6407339	0.6333963	20	FALSE	Vosa et al., 2018	Blood
PDIA3	ENSG00000167004	15	44052033	rs4924722	15	44056735	G	A	0.316434	-0.0034	0.0105	0.7460822	-0.109172	0.00895382	3.40E-34	0.0311435	0.0962123	0.7461687	0.962871	0.9220995	20	FALSE	Vosa et al., 2018	Blood
FBXO22	ENSG00000167196	15	76211904	rs57737646	15	76299828	T	C	0.0192308	0.0441	0.033	0.1814304	-0.239949	0.0228162	7.24E-26	-0.183789	0.138635	0.184938	0.1152381	0.3126048	18	FALSE	Vosa et al., 2018	Blood
CD3D	ENSG00000167286	11	118211564	rs2276423	11	118210106	G	C	0.230769	0.0095	0.0112	0.3963186	0.155629	0.0101067	1.67E-53	0.0610427	0.0720752	0.3970339	0.5368571	0.2641303	20	FALSE	Vosa et al., 2018	Blood
STIM1	ENSG00000167323	11	3995098	rs4414209	11	3885511	G	T	0.318182	-0.0245	0.011	0.02592905	0.14794	0.0100831	9.74E-49	-0.165608	0.0752066	0.02766215	0.05344966	0.05567469	20	FALSE	Vosa et al., 2018	Blood
TUBA1A	ENSG00000167552	12	49580843	rs11168868	12	49515130	C	T	0.0297203	-0.0293	0.0387	0.4489864	0.467466	0.0446874	1.31E-25	-0.0626784	0.0830034	0.4501705	0.0410797	0.4141611	17	FALSE	Vosa et al., 2018	Blood
LAIR1	ENSG00000167613	19	54873713	rs56092919	19	54855568	A	C	0.162587	-0.0498	0.0216	0.02113548	-0.254368	0.011852	3.53E-102	0.19578	0.085405	0.02188451	0.0182551	0.2278765	16	FALSE	Vosa et al., 2018	Blood
LAIR2	ENSG00000167618	19	55015498	rs149731338	19	55020233	T	C	0.136364	0.023	0.0184	0.2112995	-0.562047	0.0172662	1.97E-232	-0.0409218	0.0327616	0.211636	0.5704012	0.689864	20	FALSE	Vosa et al., 2018	Blood
KIR3DL1	ENSG00000167633	19	55307208	rs10407012	19	55387953	A	G	0.104895	0.0078	0.0172	0.6501971	-0.354593	0.0153422	3.49E-118	-0.0219971	0.0485157	0.6502599	0.5673698	0.8263962	20	FALSE	Vosa et al., 2018	Blood
CD300C	ENSG00000167850	17	72539764	rs72844303	17	72541400	C	T	0.0716783	0.0094	0.0162	0.5617481	-0.867581	0.0101142	0	-0.0108347	0.018673	0.5617571	0.208249	0.2920471	20	FALSE	Vosa et al., 2018	Blood
CD300A	ENSG00000167851	17	72471744	rs62087202	17	72459919	A	G	0.048951	0.0047	0.0239	0.8440993	-0.662954	0.0164434	0	-0.0070895	0.0360512	0.8441011	0.07849707	0.3175775	20	FALSE	Vosa et al., 2018	Blood
MLST8	ENSG00000167965	16	2256833	rs26862	16	2257105	G	C	0.479021	-0.0433	0.0098	9.94E-06	0.301991	0.00887369	7.45E-254	-0.143382	0.0327236	1.18E-05	4.85E-05	0.3223712	20	TRUE	Vosa et al., 2018	Blood
KCTD6	ENSG00000168301	3	58482964	rs73087775	3	58491954	T	C	0.284965	0.0313	0.0109	0.00408452	0.0859749	0.0119227	5.55E-13	0.36406	0.136464	0.00763472	0.02408734	0.01668202	15	FALSE	Vosa et al., 2018	Blood
TAP1	ENSG00000168394	6	32817370	rs2071477	6	32825348	C	T	0.428322	-0.0076	0.0097	0.4333305	0.0922364	0.00910941	4.26E-24	-0.0823969	0.105479	0.4347025	0.1108785	0.2721347	20	FALSE	Vosa et al., 2018	Blood
AHCYL1	ENSG00000168710	1	110546832	rs3754444	1	110534462	A	G	0.166084	-0.026	0.0132	0.04887311	-0.118455	0.0109159	1.96E-27	0.219493	0.113256	0.05261958	0.1215228	0.9944506	20	FALSE	Vosa et al., 2018	Blood
INPP5D	ENSG00000168918	2	234020613	rs62192877	2	234077283	G	T	0.33042	-0.004	0.0104	0.7005224	0.177513	0.00890236	1.83E-88	-0.0225336	0.0585981	0.7005754	0.8152443	0.8934732	20	FALSE	Vosa et al., 2018	Blood
SIGLEC7	ENSG00000168995	19	51651169	rs12983058	19	51642784	T	G	0.318182	0.0174	0.013	0.180746	0.160256	0.00954768	3.15E-63	0.108576	0.0813778	0.1821297	0.2765518	0.2976206	3	FALSE	Vosa et al., 2018	Blood
UBE2E3	ENSG00000170035	2	181886643	rs62180071	2	181829237	C	T	0.286713	0.005	0.0113	0.6581434	0.199771	0.0113935	7.91E-69	0.0250286	0.0565827	0.6582453	0.02974606	0.00032513	20	FALSE	Vosa et al., 2018	Blood
UBE2E1	ENSG00000170142	3	23890100	rs3762767	3	23847211	A	C	0.277972	0.0182	0.0113	0.1072627	-0.0849717	0.00997859	1.66E-17	-0.214189	0.135343	0.1135217	0.03120991	0.07434589	20	FALSE	Vosa et al., 2018	Blood
CD14	ENSG00000170458	5	140012299	rs57599368	5	139992113	G	A	0.244755	-0.0137	0.0115	0.2335341	-0.376047	0.0105017	8.24E-281	0.0364317	0.0305982	0.2337927	0.1069736	0.1784605	20	FALSE	Vosa et al., 2018	Blood
KIF5B	ENSG00000170759	10	32321648	rs211290	10	32336397	A	G	0.132867	-0.0111	0.015	0.4593	0.466431	0.0171125	1.38E-163	-0.0237978	0.032171	0.4594654	0.2324679	0.4790436	20	FALSE	Vosa et al., 2018	Blood
OSCAR	ENSG00000170909	19	54601966	rs10500316	19	54553697	C	T	0.545455	-0.0119	0.0103	0.2479513	-0.653754	0.00806153	0	0.0182026	0.0157568	0.2479993	0.3050406	0.1204223	20	FALSE	Vosa et al., 2018	Blood
PIK3CD	ENSG00000171608	1	9750481	rs7524062	1	9718187	A	T	0.143357	-0.003	0.0153	0.8445488	0.0767612	0.00992644	1.05E-14	-0.0390822	0.199384	0.8445981	0.6346049	0.6042749	6	FALSE	Vosa et al., 2018	Blood
SPSB1	ENSG00000171621	1	9391265	rs9660157	1	9347552	A	G	0.372378	0.0047	0.0108	0.663428	0.299289	0.00934037	2.79E-225	0.0157039	0.0360888	0.6634571	0.1061648	0.2301358	20	FALSE	Vosa et al., 2018	Blood
FBXL14	ENSG00000171823	12	1689245	rs2240506	12	1698010	A	G	0.143357	-0.0068	0.0149	0.6481197	0.0560547	0.00928797	1.59E-09	-0.12131	0.266571	0.6490541	0.2783844	0.5075433	12	FALSE	Vosa et al., 2018	Blood
PTEN	ENSG00000171862	10	89677278	rs2785080	10	89788889	G	C	0.26049	0.0172	0.0114	0.1313571	0.072512	0.0110027	4.39E-11	0.237202	0.161283	0.1413661	0.09743112	0.5258035	19	FALSE	Vosa et al., 2018	Blood
MALT1	ENSG00000172175	18	56377994	rs62093468	18	56403055	G	C	0.237762	0.0224	0.0111	0.04358938	-0.257213	0.0105728	9.96E-131	-0.0870875	0.0433032	0.04431376	0.01592678	0.6440776	20	FALSE	Vosa et al., 2018	Blood
RASGRP1	ENSG00000172575	15	38819040	rs17652674	15	38900312	A	C	0.187063	-0.0061	0.012	0.6112196	0.159308	0.013102	5.14E-34	-0.0382907	0.0753918	0.6115307	0.9143887	0.4611093	20	FALSE	Vosa et al., 2018	Blood
MYD88	ENSG00000172936	3	38182241	rs6796045	3	38189160	T	A	0.0821678	0.0246	0.0176	0.162195	0.198373	0.0207734	1.30E-21	0.124009	0.089667	0.1666675	0.1806103	0.2017402	20	FALSE	Vosa et al., 2018	Blood
PSMD1	ENSG00000173692	2	231979559	rs765458	2	231990667	A	G	0.340909	0.0176	0.0106	0.09683856	0.233558	0.00885819	3.34E-153	0.0753561	0.0454748	0.0975003	0.286355	0.2091942	20	FALSE	Vosa et al., 2018	Blood
CD34	ENSG00000174059	1	208071170	rs7572	1	208060009	T	C	0.195804	-0.005	0.012	0.6769222	-0.11581	0.00964586	3.30E-33	0.0431741	0.10368	0.6771056	0.4219007	0.2736462	15	FALSE	Vosa et al., 2018	Blood
CTSF	ENSG00000174080	11	66333623	rs2242663	11	66335308	C	T	0.227273	-0.0206	0.011	0.06110606	0.72693	0.0093272	0	-0.0283384	0.0151365	0.06118076	0.006488698	0.1141404	20	FALSE	Vosa et al., 2018	Blood
TLR1	ENSG00000174125	4	38825368	rs59672618	4	38783171	C	T	0.0506993	-0.0036	0.0201	0.8578557	0.16028	0.0127919	5.13E-36	-0.0224608	0.125419	0.85787	0.1219759	0.2011859	20	FALSE	Vosa et al., 2018	Blood
TLR6	ENSG00000174130	4	38841887	rs57130162	4	38838464	A	G	0.262238	-0.0216	0.0104	0.03780866	0.120627	0.0092659	9.62E-39	-0.179064	0.0873064	0.04026831	0.3213296	0.05327521	14	FALSE	Vosa et al., 2018	Blood
HRAS	ENSG00000174775	11	534764	rs1870726	11	539648	A	G	0.201049	0.0077	0.0146	0.5979177	-0.293247	0.0117509	1.88E-137	-0.0262577	0.0497985	0.5979995	0.006100286	0.1251272	20	FALSE	Vosa et al., 2018	Blood
FBXW8	ENSG00000174989	12	117408857	rs10774898	12	117468873	G	A	0.31993	0.0205	0.0106	0.05311777	-0.227408	0.0088676	4.83E-145	-0.0901464	0.0467446	0.05379468	0.09233905	0.1329127	20	FALSE	Vosa et al., 2018	Blood
KLC2	ENSG00000174996	11	66030048	rs7951079	11	65980550	T	C	0.18007	-0.0144	0.0125	0.2493211	-0.0863909	0.0113341	2.49E-14	0.166684	0.146334	0.2546765	0.4888804	0.3369406	15	FALSE	Vosa et al., 2018	Blood
UBE2C	ENSG00000175063	20	44443405	rs380421	20	44378577	C	T	0.657343	-0.0145	0.0098	0.1389822	0.076145	0.00905992	4.29E-17	-0.190426	0.130681	0.1450657	0.05354301	0.1044553	20	FALSE	Vosa et al., 2018	Blood
TRAF6	ENSG00000175104	11	36520199	rs331463	11	36501787	A	T	0.132867	0.0192	0.0139	0.1671883	-0.105248	0.0109213	5.58E-22	-0.182426	0.133419	0.1715247	0.5607537	0.5972108	20	FALSE	Vosa et al., 2018	Blood
PSMD2	ENSG00000175166	3	184021669	rs13062950	3	184027539	T	G	0.472028	0.0161	0.0094	0.08675561	-0.252369	0.00968132	8.50E-150	-0.0637955	0.0373274	0.08743599	0.2772684	0.0363079	20	FALSE	Vosa et al., 2018	Blood
UBE2O	ENSG00000175931	17	74417410	rs394873	17	74413449	G	A	0.377622	-0.0089	0.0098	0.363792	-0.191478	0.00886326	1.66E-103	0.0464804	0.0512259	0.3642155	0.7247305	0.9135007	20	FALSE	Vosa et al., 2018	Blood
TUBB6	ENSG00000176014	18	12325993	rs7244254	18	12317836	A	G	0.146853	-0.0075	0.0145	0.6049877	0.7473	0.00935293	0	-0.0100361	0.0194036	0.6049952	0.6975407	0.5065391	20	FALSE	Vosa et al., 2018	Blood
YES1	ENSG00000176105	18	767067	rs4121511	18	815330	T	C	0.321678	-0.0125	0.0112	0.2643916	-0.0966664	0.00915384	4.56E-26	0.129311	0.116508	0.2670464	0.1048314	0.2645566	20	FALSE	Vosa et al., 2018	Blood
ANAPC2	ENSG00000176248	9	140076112	rs66708191	9	140095436	A	C	0.295455	-0.0077	0.0129	0.5505747	0.0603838	0.0108757	2.82E-08	-0.127518	0.214864	0.5528603	0.5528603	0.9078452	3	FALSE	Vosa et al., 2018	Blood
CDC26	ENSG00000176386	9	116027992	rs9299214	9	116035696	A	G	0.652098	-0.0083	0.0098	0.3970292	0.399302	0.00869127	0	-0.0207863	0.024547	0.3971094	0.4994656	0.407289	20	FALSE	Vosa et al., 2018	Blood
MEX3C	ENSG00000176624	18	48722797	rs12458916	18	48731131	C	T	0.440559	0.0554	0.01	3.02E-08	-0.190649	0.00960217	1.00E-87	-0.290586	0.0544559	9.49E-08	2.27E-05	0.2070535	20	TRUE	Vosa et al., 2018	Blood

ARIH2	ENSG00000177479	3	48990034	rs7647093	3	48977398	T	G	0.333916	-0.058	0.01	6.63E-09	-0.19333	0.00979054	8.58E-87	0.300006	0.0539102	2.62E-08	1.74E-07	3.05E-05	20	TRUE	Vosa et al., 2018	Blood
GRB2	ENSG00000177885	17	73357973	rs55653371	17	73309355	G	A	0.270979	0.0247	0.0112	0.02742904	-0.217586	0.0117284	7.86E-77	-0.113518	0.0518364	0.02852871	0.01737699	0.3369027	20	FALSE	Vosa et al., 2018	Blood
UBE2N	ENSG00000177889	12	93817743	rs10777512	12	93859770	C	T	0.372378	-0.016	0.0103	0.1203281	-0.0881546	0.00929039	2.34E-21	0.181499	0.118396	0.125278	0.1661066	0.08106897	20	FALSE	Vosa et al., 2018	Blood
ASB8	ENSG00000177981	12	48558283	rs7485057	12	48578809	T	C	0.208042	0.0182	0.0111	0.1010801	-0.183129	0.00900712	6.76E-92	-0.0993834	0.0608097	0.1021888	0.173381	0.03972435	20	FALSE	Vosa et al., 2018	Blood
KLHL11	ENSG00000178502	17	40015740	rs9797236	17	40002928	G	A	0.274476	0.0147	0.0112	0.1893515	0.0708658	0.00984275	6.03E-13	0.207434	0.16065	0.1966273	0.1998053	0.9659438	11	FALSE	Vosa et al., 2018	Blood
CD28	ENSG00000178562	2	204586877	rs3116492	2	204588382	G	A	0.202797	0.0222	0.0126	0.07808539	-0.244549	0.0134551	8.11E-74	-0.0907794	0.051765	0.07948467	0.1366334	0.3246127	20	FALSE	Vosa et al., 2018	Blood
CD300LB	ENSG00000178789	17	72522463	rs117056610	17	72498108	T	C	0.0244755	0.0815	0.0391	0.03712389	1.1947	0.046337	1.38E-146	0.0682182	0.0328348	0.03774432	0.3394186	0.958811	20	FALSE	Vosa et al., 2018	Blood
CALR	ENSG00000179218	19	13052347	rs2965215	19	13071832	A	G	0.283217	0.0091	0.0104	0.3815739	-0.0600901	0.0103145	5.68E-09	-0.151439	0.175015	0.3868771	0.4623146	0.9476065	9	FALSE	Vosa et al., 2018	Blood
PTPN11	ENSG00000179295	12	112901936	rs58116261	12	112935344	T	C	0.0909091	0.0034	0.0183	0.8526076	0.139561	0.012819	1.33E-27	0.0243621	0.131145	0.8526288	0.8834233	0.7999122	20	FALSE	Vosa et al., 2018	Blood
ZNRF2	ENSG00000180233	7	30388020	rs73685955	7	30286649	C	G	0.0122378	-0.1157	0.0441	0.00870103	-0.175285	0.0169811	5.58E-25	0.660066	0.259589	0.01099876	0.07026822	0.1357561	10	FALSE	Vosa et al., 2018	Blood
PAK2	ENSG00000180370	3	196513123	rs2084385	3	196553867	A	G	0.129371	-0.0055	0.0151	0.71568	0.216416	0.0155511	5.04E-44	-0.0254141	0.069797	0.7157731	0.4243611	0.2745337	19	FALSE	Vosa et al., 2018	Blood
RNF182	ENSG00000180537	6	13952605	rs7760949	6	13920538	C	A	0.321678	-0.0112	0.0101	0.2674686	0.602493	0.00822733	0	-0.0185894	0.0167656	0.2675235	0.01549841	0.6479554	20	FALSE	Vosa et al., 2018	Blood
SIAH2	ENSG00000181788	3	150470089	rs524772	3	150447679	G	A	0.0227273	-0.0062	0.0333	0.8522988	-0.707829	0.0435807	2.55E-59	0.00875918	0.0470483	0.8523083	0.06147016	0.02615532	15	FALSE	Vosa et al., 2018	Blood
RNF41	ENSG00000181852	12	56607001	rs183499024	12	56472052	A	G	0.0174825	0.0015	0.058	0.9793674	0.518971	0.0559417	1.74E-20	0.00289034	0.11176	0.9793674	0.5535248	0.6850964	9	FALSE	Vosa et al., 2018	Blood
UBA7	ENSG00000182179	3	49847009	rs7374277	3	49784777	T	C	0.493007	0.0062	0.0094	0.5095269	-0.622158	0.00830537	0	-0.0099653	0.0151093	0.5095434	6.01E-06	0.05005492	20	TRUE	Vosa et al., 2018	Blood
UBE2E2	ENSG00000182247	3	23438897	rs778472	3	23505899	T	C	0.587413	-0.0155	0.0101	0.124869	0.204457	0.00885101	4.65E-118	-0.0758107	0.0495081	0.1257007	0.4579224	0.3716283	20	FALSE	Vosa et al., 2018	Blood
AP2A2	ENSG00000183020	11	968566	rs66670048	11	999764	C	T	0.0944056	0.0131	0.0178	0.461758	-0.326388	0.0184557	5.47E-70	-0.0401362	0.0545835	0.4621455	0.2652658	0.1721969	20	FALSE	Vosa et al., 2018	Blood
ASB7	ENSG00000183475	15	101167324	rs56363126	15	101105116	G	A	0.183566	-0.0146	0.0127	0.2503061	-0.0707633	0.0100886	2.31E-12	0.206322	0.181866	0.2565974	0.6184649	0.7253543	20	FALSE	Vosa et al., 2018	Blood
KLHL25	ENSG00000183655	15	86320407	rs2614659	15	86308548	T	C	0.367133	-0.0022	0.0097	0.8205761	0.066071	0.00955972	4.80E-12	-0.0332975	0.146891	0.8206709	0.5731537	0.4667573	20	FALSE	Vosa et al., 2018	Blood
UBE2F	ENSG00000184182	2	238913352	rs62194508	2	238877409	C	A	0.40035	0.008	0.0099	0.4190441	0.425968	0.00964438	0	0.0187807	0.023245	0.4191219	0.3678498	0.4852129	20	FALSE	Vosa et al., 2018	Blood
UBE2G2	ENSG00000184787	21	46205444	rs62220109	21	46213074	G	C	0.339161	-0.0216	0.0108	0.04550026	-0.320284	0.0106834	1.81E-197	0.0674401	0.033795	0.04598137	0.2241928	0.9739031	20	FALSE	Vosa et al., 2018	Blood
BTBD6	ENSG00000184887	14	105716128	rs4412909	14	105759226	C	T	0.29021	-0.0208	0.0117	0.07544036	-0.241836	0.010774	1.39E-111	0.0860086	0.0485314	0.07635673	0.2732515	0.6810549	8	FALSE	Vosa et al., 2018	Blood
UBOX5	ENSG00000185019	20	3114530	rs306757	20	3119133	A	G	0.298951	0.0189	0.0102	0.06389079	0.315671	0.010989	1.80E-181	0.0598725	0.0323793	0.06444361	0.008977458	0.03688167	20	FALSE	Vosa et al., 2018	Blood
SOCS1	ENSG00000185338	16	11349149	rs2021511	16	11344903	T	C	0.281469	-0.0077	0.0106	0.4675843	0.113533	0.0112637	6.80E-24	-0.0678215	0.0936068	0.4687367	0.4443209	0.3082934	20	FALSE	Vosa et al., 2018	Blood
PRKG1	ENSG00000185532	10	53404527	rs10996331	10	53010887	A	G	0.0769231	-0.0003	0.0192	0.9875336	0.167857	0.015795	2.23E-26	-0.0017872	0.114383	0.9875336	0.8093561	0.851882	9	FALSE	Vosa et al., 2018	Blood
PSMD13	ENSG00000185627	11	244764	rs1533824	11	214169	C	T	0.332168	-0.0074	0.0109	0.4972018	0.300524	0.0107842	6.69E-171	-0.0246237	0.0362808	0.4973294	0.1835045	0.01008154	20	FALSE	Vosa et al., 2018	Blood
UBE2L3	ENSG00000185651	22	21941029	rs5754234	22	21942978	T	A	0.160839	0.0156	0.0117	0.1824224	0.767251	0.00942381	0	0.0203323	0.0152513	0.1824811	0.2047384	0.9512169	20	FALSE	Vosa et al., 2018	Blood
TRIM69	ENSG00000185880	15	45040606	rs17588305	15	45021766	T	C	0.122378	-0.0153	0.0156	0.3267066	-0.541612	0.0137506	0	0.028249	0.0288118	0.3268565	0.7719237	0.2561004	20	FALSE	Vosa et al., 2018	Blood
IFITM1	ENSG00000185885	11	314389	rs9666761	11	330061	G	A	0.674825	0.002	0.0124	0.8718647	0.284415	0.00876595	6.28E-231	0.00703197	0.0435988	0.8718663	0.6963174	0.4734879	19	FALSE	Vosa et al., 2018	Blood
CD300LF	ENSG00000186074	17	72699784	rs34840376	17	72710996	A	C	0.188811	0.0237	0.0128	0.06408867	-0.408904	0.0105877	0	-0.0579598	0.0313391	0.06439455	0.3947783	0.02227956	20	FALSE	Vosa et al., 2018	Blood
ZNRF1	ENSG00000186187	16	75088910	rs62061437	16	75031931	G	T	0.681818	-0.0142	0.011	0.1967352	-0.170446	0.00912483	7.28E-78	0.0833107	0.0646904	0.1978026	0.2434565	0.6332412	20	FALSE	Vosa et al., 2018	Blood
BTLA	ENSG00000186265	3	112200611	rs2633577	3	112151201	C	T	0.103147	-0.0237	0.0183	0.195292	-0.156443	0.0166627	6.07E-21	0.151493	0.118083	0.1995151	0.6519074	0.6263642	17	FALSE	Vosa et al., 2018	Blood
CD300E	ENSG00000186407	17	72612961	rs566915	17	72591657	C	T	0.312937	0.0085	0.0103	0.4092338	0.212993	0.0092659	6.32E-117	0.0399073	0.0483894	0.4095354	0.5544038	0.8955072	20	FALSE	Vosa et al., 2018	Blood
BTN3A2	ENSG00000186470	6	26371966	rs9357006	6	26364628	C	A	0.117133	0.0101	0.0153	0.5091699	-1.55844	0.0141727	0	-0.0064808	0.00981768	0.5091776	1.17E-06	0.00637678	20	TRUE	Vosa et al., 2018	Blood
UBE2H	ENSG00000186591	7	129531680	rs145942329	7	129509030	A	G	0.0944056	-0.0021	0.0176	0.9050233	0.194308	0.0191414	3.27E-24	-0.0108076	0.0905839	0.9050298	0.9120427	0.4730884	20	FALSE	Vosa et al., 2018	Blood
PRR5	ENSG00000186654	22	45099077	rs1034459	22	45115022	T	C	0.645105	-0.0014	0.0107	0.895901	-0.122926	0.0092139	1.33E-40	0.0113889	0.0870482	0.895906	0.4348812	0.2180915	20	FALSE	Vosa et al., 2018	Blood
LILRB4	ENSG00000186818	19	55168575	rs370156	19	55172539	A	G	0.103147	-0.0021	0.0167	0.899931	0.627262	0.0161222	0	-0.0033479	0.0266238	0.8999315	0.5851434	0.1729802	12	FALSE	Vosa et al., 2018	Blood
LILRA5	ENSG00000187116	19	54821381	rs1761456	19	54814602	A	C	0.262238	0.0069	0.0119	0.562028	-0.218618	0.00908567	6.28E-128	-0.031562	0.0544487	0.5621415	0.3023633	0.295644	20	FALSE	Vosa et al., 2018	Blood
RNF220	ENSG00000187147	1	44994131	rs112104879	1	45134059	T	C	0.136364	-0.0063	0.0137	0.645621	-0.224071	0.0185164	1.04E-33	0.0281161	0.0611855	0.6458591	0.01398317	0.05218907	18	FALSE	Vosa et al., 2018	Blood
TREML4	ENSG00000188056	6	41201341	rs6458200	6	41195420	T	G	0.234266	-0.0164	0.0114	0.1502649	0.980587	0.00778448	0	-0.0167247	0.0116265	0.1502915	0.1053357	0.1369105	20	FALSE	Vosa et al., 2018	Blood
NCR3LG1	ENSG00000188211	11	17386080	rs7118875	11	17403669	A	G	0.312937	-0.0035	0.0105	0.7388827	0.123236	0.010165	7.92E-34	-0.0284008	0.0852345	0.7389777	0.5220792	0.6381957	20	FALSE	Vosa et al., 2018	Blood
PDCD1	ENSG00000188389	2	242796546	rs7422174	2	242850538	A	G	0.47028	0.0017	0.0122	0.8891781	0.168525	0.00898654	1.83E-78	0.0100876	0.072395	0.8891812	0.7910368	0.4233635	20	FALSE	Vosa et al., 2018	Blood
SELL	ENSG00000188404	1	169670323	rs2223286	1	169665632	C	T	0.297203	0.0092	0.0102	0.3670777	-0.670766	0.00887245	0	-0.0137157	0.0152076	0.3671118	0.5351773	0.04270128	20	FALSE	Vosa et al., 2018	Blood
KIR2DL4	ENSG00000189013	19	55280987	rs77887839	19	55386858	G	C	0.0472028	0.0201	0.0305	0.5098852	0.126697	0.019687	1.23E-10	0.158646	0.24199	0.5120892	0.6086485	0.9791009	11	FALSE	Vosa et al., 2018	Blood
NCR1	ENSG00000189430	19	55422508	rs1671160	19	55428459	T	C	0.236014	0.006	0.0122	0.6228584	0.116786	0.0114613	2.21E-24	0.051376	0.104586	0.6232628	0.9436017	0.712338	14	FALSE	Vosa et al., 2018	Blood
HLA-DRB1	ENSG00000196126	6	32552085	rs9271520	6	32589771	G	A	0.325175	-0.0835	0.0145	8.48E-09	-0.538394	0.00941138	0	0.155091	0.0270681	1.01E-08	1.97E-16	3.15E-14	20	TRUE	Vosa et al., 2018	Blood
CTSE	ENSG00000196188	1	206324781	rs28694519	1	206328647	C	G	0.202797	-0.0026	0.0162	0.8724921	-0.109007	0.0103757	8.11E-26	0.0238517	0.148632	0.8725068	0.9552914	0.965628	5	FALSE	Vosa et al., 2018	Blood
PPIA	ENSG00000196262	7	44850221	rs6463247	7	44839616	C	T	0.194056	-0.0273	0.0127	0.03158637	-0.284362	0.00876742	9.12E-231	0.0960043	0.0447593	0.03196079	0.1153947	0.2757172	20	FALSE	Vosa et al., 2018	Blood
ASB13	ENSG00000196372	10	5694694	rs4747977	10	5704786	C	G	0.326923	-0.0008	0.0101	0.9368672	0.243394	0.00946226	6.53E-146	-0.0032869	0.0414967	0.9368675	0.5851123	0.8502237	20	FALSE	Vosa et al., 2018	Blood
EVL	ENSG00000196405	14	100524179	rs1191034	14	100434403	T	C	0.157343	-0.0059	0.014	0.6734422	-0.166655	0.0122606	4.42E-42	0.0354024	0.0840461	0.6735899	0.4289874	0.7675717	20	FALSE	Vosa et al., 2018	Blood
SIAH1	ENSG00000196470	16	48436294	rs8053839	16	48390512	T	G	0.533217	-0.0029	0.0102	0.77617	0.107348	0.0100651	1.48E-26	-0.0270149	0.0950516	0.7762473	0.4427574	0.1475888	15	FALSE	Vosa et al., 2018	Blood
ANAPC7	ENSG00000196510	12	110826120	rs6606686	12	110903380	G	C	0.283217	0.0022	0.011	0.8414806	0.118909	0.0100489	2.64E-32	0.0185015	0.0925208	0.8415029	0.681953	0.5393342	20	FALSE	Vosa et al., 2018	Blood
HLA-DQA1	ENSG00000196735	6	32605397	rs1063355	6	32627714	T	G	0.393357	0.0439	0.0108	4.81E-05	0.652626	0.00802871	0	0.0672667	0.0165692	4.91E-05	2.86E-14	5.02E-10	20	TRUE	Vosa et al., 2018	Blood
AP2A1	ENSG00000196961	19	50290297	rs7247528	19	50291807	A	T	0.0891608	-0.0113	0.0174	0.5160635	0.121795	0.0181221	1.81E-11	-0.0927787	0.143528	0.518011	0.6470848			FALSE	Vosa et al., 2018	Blood
DYNC1H1	ENSG00000197102	14	102473997	rs1190607	14	102511597	G	A	0.18007	-0.002	0.0116	0.8631122	0.0620256	0.00991109	3.89E-10	-0.0322448	0.187091	0.8631636	0.5106168	0.2254994	20	FALSE	Vosa et al., 2018	Blood
SRC	ENSG00000197122	20	36003770	rs6017916	20	35971278	C	A	0.262238	-0.0238	0.0113	0.03518744	-0.0892017	0.00995666	3.28E-19	0.266811	0.130133	0.04033535	0.03529139	0.5998163	17	FALSE	Vosa et al., 2018	Blood
FBXL22	ENSG00000197361	15	63892089	rs9972527	15	64127531	C	T	0.541958	0.022	0.0097	0.02332669	0.0884452	0.0091437	3.93E-22	0.248742	0.112647	0.02723366	0.0870922	0.00182463	20	FALSE	Vosa et al., 2018	Blood
MIB2	ENSG00000197530	1	1558392	rs12735276	1	1526799	A	G	0.171329	0.0518	0.0208	0.01276049	0.0611735	0.00971471	3.03E-10	0.846771	0.365642	0.02056646	0.1117089	0.2787071	9	FALSE	Vosa et al., 2018	Blood
ATG7	ENSG00000197548	3	11456567	rs6442241	3	11272005	G	A	0.153846	-0.0028	0.0138	0.8392143	-0.297883	0.00945676	8.84E-218	0.00939967	0.0463279	0.8392176	0.08147124	0.1702457	20	FALSE	Vosa et al., 2018	Blood
PDCD1LG2	ENSG00000197646	9	5540913	rs16923189	9	5510644	G	A	0.332168	-0.0082	0.011	0.455997	0.198932	0.00984346	8.06E-91	-0.04122	0.0553328	0.4563032	0.2948823	0.1597348	20	FALSE	Vosa et al., 2018	Blood
DNM3	ENSG00000197959	1	172099113	rs9425308	1	172383265	A	C	0.305944	-0.0261	0.0103	0.0112775	-0.157822	0.00981124	3.21E-58	0.165377	0.0660684	0.01231062	1.76E-05	0.3025447	20	TRUE	Vosa et al., 2018	Blood
CARD11	ENSG00000198286	7	3014677	rs6955702	7	3157702	G	A	0.463287	-0.0123	0.0104	0.2369311	0.33659	0.00870623	0	-0.036543	0.0309126	0.2371504	0.7630027	0.5044626	20	FALSE	Vosa et al., 2018	Blood
HLA-DRB5	ENSG00000198502	6	32491592	rs9271055	6	32575369	G	T	0.141608	0.0564	0.0143	8.01E-05	1.19522	0.00960029	0	0.0471878	0.0119703	8.08E-05	1.03E-15	6.24E-08	20	TRUE	Vosa et al., 2018	Blood
SH2D1B	ENSG00000198574	1	162373492	rs12746024	1	162374853	A	C	0.297203	0.0225	0.0109	0.03899684	0.214858	0.0107385	4.67E-89	0.104721	0.0510005	0.0400414	0.06796481	0.1435236	20	FALSE	Vosa et al., 2018	Blood
KLHL9	ENSG00000198642	9	21332524	rs7862940	9	21317985	A	G	0.199301	0.0344	0.0123	0.00516198	0.417269	0.00934857	0	0.0824408	0.0295352	0.0052501	0.03639067	0.3824309	20	FALSE	Vosa et al., 2018	Blood
CALM1	ENSG00000198668	14	90868725	rs7150626	14	90858484	T	C	0.79021	0.0139	0.0127	0.2737409	-0.231433	0.00883439	2.89E-151	-0.0600605	0.0549233	0.2741593	0.2292752	0.3046809	20	FALSE	Vosa et al., 2018	Blood
SMURF1	ENSG00000198742	7	98683392	rs11975761	7	98766688	G	A	0.288462	0.0036	0.0114	0.7521623	0.0712187	0.00981081	3.89E-13	0.0505486	0.160222	0.7523888	0.7919671	0.2565202	8	FALSE	Vosa et al., 2018	Blood
MTOR	ENSG00000198793	1	11244578	rs4845986	1	11288633	G	C	0.265734	0.0202	0.0108	0.0614324	0.21392	0.00934818	6.77E-116	0.0944278	0.0506545	0.06230008	0.1172984	0.3483839	20	FALSE	Vosa et al., 2018	Blood
CD247	ENSG00000198821	1	167443862	rs2949661	1	167424924	T	C	0.40035	0.0077	0.0097	0.4273035	0.334453	0.0110762	2.70E-200	0.0230227	0.0290126	0.4274631	0.6055865	0.5752242	20	FALSE	Vosa et al., 2018	Blood
UBE2J1	ENSG00000198833	6	90049455	rs12209311	6	90067072	C	T	0.0786713	0.0506	0.024	0.03500217	0.535902	0.0231425	1.25E-118	0.0944203	0.0449696	0.03575975	0.1860119	0.03376027	16	FALSE	Vosa et al., 2018	Blood
CD3E	ENSG00000198851	11	118181075	rs894678	11	118172520	A	G	0.34965	0.004	0.01	0.6891565	-0.149168	0.00915007	9.49E-60	-0.0268154	0.0670587	0.6892452	0.3364592	0.2273986	20	FALSE	Vosa et al., 2018	Blood
LTN1	ENSG00000198862	21	30332871	rs2464620	21	30298338	C	T	0.545455	-0.0008	0.0098	0.9349388	0.153982	0.00935277	6.68E-61	-0.0051954	0.0636445	0.9349396	0.4464321	0.5029165	20	FALSE	Vosa et al., 2018	Blood
CAPZA2	ENSG00000198898	7	116505219	rs9791721	7	116524512	C	G	0.244755	0.0136	0.0114	0.2328762	-0.290958	0.00941951	1.68E-209	-0.0467421	0.0392101	0.2332245	0.2892146	0.8146648	20	FALSE	Vosa et al., 2018	Blood
DZIP3	ENSG00000198919	3	108361111	rs4494934	3	108317536	A	G	0.342657	0.0113	0.0103	0.2726032	0.442622	0.0112766	0	0.0255297	0.0232795	0.2727904	0.7493574	0.8130313	20	FALSE	Vosa et al., 2018	Blood
PJA2	ENSG00000198961	5	108708052	rs33730	5	108672755	A	T	0.363636	0.01	0.0098	0.3075349	0.462561	0.00866727	0	0.0216188	0.0211903	0.3076233	0.08119567	0.07104999	20	FALSE	Vosa et al., 2018	Blood
FCGR3A	ENSG00000203747	1	161556233	rs34085961	1	161600995	T	C	0.325175	-0.0333	0.0158	0.03506604	0.0573056	0.00898122	1.76E-10	-0.581095	0.290367	0.04536588	0.003610048	0.001753	9	FALSE	Vosa et al., 2018	Blood
HLA-DOA	ENSG00000204252	6	32974672	rs381218	6	32977420	T	G	0.253497	-0.0245	0.0111	0.02729959	-0.325518	0.0107288	3.34E-202	0.0752646	0.0341896	0.02770844	0.008345556	0.02692337	20	FALSE	Vosa et al., 2018	Blood
HLA-DMA	ENSG00000204257	6	32926630	rs3135029	6	32921774	T	G	0.0664336	0.0448	0.0167	0.00730447	0.218358	0.0147647	1.72E-49	0.205167	0.0777278	0.00830133	0.4712768	0.04807696	20	FALSE	Vosa et al., 2018	Blood
PSMB8	ENSG00000204264	6	32810487	rs2071467	6	32805306	T	C	0.0384615	0.0095	0.0249	0.7028129	0.502303	0.017285	1.15E-185	0.0189129	0.049576	0.7028373	0.000404385	0.03764177	20	FALSE	Vosa et al., 2018	Blood
TAP2	ENSG00000204267	6	32798083	rs4148876	6	32796793	A	G	0.0769231	-0.1002	0.019	1.34E-07	-0.588932	0.0166541	6.41E-274	0.170138	0.0326185	1.83E-07	2.37E-10	0.00011799	20	TRUE	Vosa et al., 2018	Blood
HLA-DRA	ENSG00000204287	6	32410221	rs601945	6	32573415	G	A	0.167832	-0.1521	0.0178	1.29E-17	-0.116966	0.0141512	1.39E-16	1.30038	0.218887	2.83E-09	5.43E-09	0.01158478	20	TRUE	Vosa et al., 2018	Blood
CD300LD	ENSG00000204345	17	72581963	rs59362027	17	72563686	A	G	0.104895	0.0092	0.0149	0.5369382	0.403541	0.00985208	0	0.0227982	0.0369273	0.5369844	0.5830777	0.9206659	20	FALSE	Vosa et al., 2018	Blood
NCR3	ENSG00000204475	6	31558717	rs6916921	6	31520426	T	C	0.0856643	0.0119	0.0157	0.4484739	-0.415766	0.0148753	6.56E-172	-0.0286219	0.0377755	0.4486406	0.001597925	0.3096768	20	FALSE	Vosa et al., 2018	Blood
MICB	ENSG00000204516	6	31470779	rs7757162	6	31478960	A	G	0.0611888	0.0441	0.0249	0.07654668	-0.881459	0.0263443	1.89E-245	-0.0500307	0.0282882	0.07695925	1.21E-05	0.00030421	20	TRUE	Vosa et al., 2018	Blood
HLA-C	ENSG00000204525	6	31238216	rs9264547	6	31234742	C	G	0.190559	0.0204	0.0124	0.09993655	0.780475	0.0104309	0	0.0261379	0.0158916	0.1000185	1.44E-07	0.00958969	20	TRUE	Vosa et al., 2018	Blood
LILRB3	ENSG00000204577	19	54733374	rs17207321	19	54709443	G	T	0.284965	0.0131	0.0117	0.2628595	-0.237411	0.0123496	2.32E-82	-0.0551785	0.0493651	0.2636678	0.2780649	0.2268186	19	FALSE	Vosa et al., 2018	Blood
HLA-E	ENSG00000204592	6	30459613	rs1265046	6	31081700	G	A	0.508741	-0.0046	0.0096	0.6318201	0.0993924	0.00894572	1.11E-28	-0.0462812	0.0966767	0.6321366	0.001616221	0.03065618	20	FALSE	Vosa et al., 2018	Blood
TRIM39	ENSG00000204599	6	30302881	rs150506826	6	29906189	T	G	0.034965	0.0411	0.0334	0.2184954	0.172142	0.0266978	1.13E-10	0.238757	0.197528	0.2267691	0.3740217	0.06069607	12	FALSE	Vosa et al., 2018	Blood
HLA-G	ENSG00000204632	6	29796823	rs2471863	6	29899627	T	C	0.377622	-0.0531	0.0145	0.00025019	0.544628	0.00835738	0	-0.0974978	0.0266657	0.00025588	0.000127517	0.04495121	20	FALSE	Vosa et al., 2018	Blood
HLA-F	ENSG00000204642	6	29698428	rs2523404	6	29695713	T	C	0.465035	-0.0206	0.0099	0.03745148	-0.66592	0.00803416	0	0.0309346	0.0148713	0.03751153	0.000496781	5.12E-05	20	FALSE	Vosa et al., 2018	Blood

DCTN1	ENSG00000204843	2	74603747	rs13021268	2	74553663	T	C	0.0297203	0.0005	0.0363	0.9890102	-0.460306	0.0345311	1.54E-40	-0.0010862	0.0788606	0.9890102	0.9605591	0.8954472	10	FALSE	Vosa et al., 2018	Blood
PSMB10	ENSG00000205220	16	67969697	rs8059990	16	67853923	A	G	0.0472028	0.0682	0.0233	0.00342206	-0.136303	0.0165364	1.69E-16	-0.500356	0.181401	0.00581054	0.004657105	0.1266637	20	FALSE	Vosa et al., 2018	Blood
HLA-B	ENSG00000234745	6	31323307	rs2596496	6	31322782	C	G	0.344406	0.004	0.0112	0.7209849	0.379964	0.00952245	0	0.0105273	0.0294777	0.7209956	3.79E-05	0.2584664	20	TRUE	Vosa et al., 2018	Blood
HLA-A	ENSG00000206503	6	29911349	rs2471863	6	29899627	T	C	0.377622	-0.0531	0.0145	0.00025019	0.349049	0.00876568	0	-0.152127	0.0417167	0.00026565	0.000690199	2.78E-06	20	FALSE	Vosa et al., 2018	Blood
IGKV3D-20	ENSG00000211625	2	90077995	rs13383309	2	89088923	C	T	0.0314685	0.024	0.0337	0.4763619	0.146714	0.0203402	5.47E-13	0.163583	0.230815	0.4784982	0.6174771			FALSE	Vosa et al., 2018	Blood
IGLV6-57	ENSG00000211640	22	22550399	rs2073447	22	22550450	G	C	0.442308	0.002	0.0107	0.8517266	-0.263215	0.00961252	4.42E-165	-0.0075983	0.0406521	0.85173	0.7376776	0.7589971	20	FALSE	Vosa et al., 2018	Blood
IGLV1-51	ENSG00000211644	22	22677082	rs10854724	22	22600088	C	A	0.183566	-0.0041	0.0123	0.7388827	0.119793	0.0124641	7.18E-22	-0.0342257	0.102739	0.7390339	0.8872798	0.9435959	19	FALSE	Vosa et al., 2018	Blood
IGLV1-47	ENSG00000211648	22	22712347	rs2856876	22	23249440	A	C	0.136364	-0.0206	0.0152	0.1753337	0.0666969	0.00933737	9.13E-13	-0.30886	0.231963	0.1830217	0.152274			FALSE	Vosa et al., 2018	Blood
IGLV1-44	ENSG00000211651	22	22735425	rs6002080	22	22734848	C	T	0.176573	-0.0175	0.0154	0.2558044	-0.338863	0.0119583	1.21E-176	0.0516434	0.0454827	0.2561864	0.5646268	0.85209	20	FALSE	Vosa et al., 2018	Blood
IGLV7-43	ENSG00000211652	22	22749591	rs2519501	22	23249354	C	T	0.395105	0.0078	0.0104	0.4532547	-0.0828666	0.00925532	3.45E-19	-0.0941272	0.125943	0.4548328	0.7763141	0.4451104	16	FALSE	Vosa et al., 2018	Blood
IGLV3-27	ENSG00000211658	22	23011017	rs2877064	22	23013171	C	T	0.0769231	-0.0328	0.0239	0.1699436	0.194437	0.012442	4.74E-55	-0.168692	0.123392	0.1715867	0.2891489	0.9516905	9	FALSE	Vosa et al., 2018	Blood
IGLV3-19	ENSG00000211663	22	23063369	rs5996359	22	23063932	A	G	0.332168	-0.0095	0.0144	0.5094321	0.0980417	0.00906015	2.73E-27	-0.0968976	0.147149	0.5102172	0.3334272	0.2799368	8	FALSE	Vosa et al., 2018	Blood
IGLV2-14	ENSG00000211666	22	23101448	rs6003261	22	23069225	C	T	0.335664	-0.0121	0.0152	0.4260014	-0.0599744	0.00907329	3.84E-11	0.201753	0.255273	0.4293282	0.4800397	0.09294733	5	FALSE	Vosa et al., 2018	Blood
IGLV3-1	ENSG00000211673	22	23223231	rs459130	22	23253303	C	G	0.23951	0.0068	0.0116	0.5577365	-0.198609	0.0105548	5.48E-79	-0.0342381	0.0584346	0.5579275	0.4263726	0.523053	15	FALSE	Vosa et al., 2018	Blood
IGLC2	ENSG00000211677	22	23243386	rs459130	22	23253303	C	G	0.23951	0.0068	0.0116	0.5577365	-0.466929	0.0102108	0	-0.0145633	0.0248452	0.5577688	0.5598573	0.6425823	20	FALSE	Vosa et al., 2018	Blood
IGLC3	ENSG00000211679	22	23248742	rs409751	22	23266797	C	T	0.402098	-0.0034	0.0103	0.7413266	0.118564	0.00956468	2.75E-35	-0.0286765	0.0869039	0.741415	0.7729431	0.8033335	20	FALSE	Vosa et al., 2018	Blood
TRAV8-4	ENSG00000211790	14	22362911	rs8003805	14	22370028	G	C	0.281469	0.0061	0.0111	0.5826284	-0.157656	0.00990278	4.57E-57	-0.0386918	0.0704483	0.5828528	0.6038685	0.4722178	20	FALSE	Vosa et al., 2018	Blood
TRAV19	ENSG00000211799	14	22476085	rs11852093	14	22477415	A	G	0.157343	0.0066	0.0158	0.6761507	-0.427112	0.0126708	4.34E-249	-0.0154526	0.0369955	0.6761742	0.9371629	0.3694097	20	FALSE	Vosa et al., 2018	Blood
IGHV1-2	ENSG00000211934	14	106452920	rs7142963	14	107058166	A	T	0.152098	0.0296	0.0238	0.2136109	0.107426	0.0160079	1.94E-11	0.275538	0.22532	0.2213772	0.2010972	0.1106179	8	FALSE	Vosa et al., 2018	Blood
IGHV3-33	ENSG00000211955	14	106815987	rs8011590	14	107038041	C	G	0.0629371	0.0031	0.0363	0.9319438	-0.142088	0.0145136	1.24E-22	-0.0218174	0.255485	0.9319464	0.5605181	0.6708595	7	FALSE	Vosa et al., 2018	Blood
IGHV4-39	ENSG00000211959	14	106877872	rs7151792	14	106978471	G	A	0.222028	-0.0021	0.0186	0.9101073	-0.303081	0.0101614	1.76E-195	0.00692885	0.0613702	0.9101079	0.3887137	0.7857777	12	FALSE	Vosa et al., 2018	Blood
IGHV1-46	ENSG00000211962	14	106967418	rs10132771	14	107021489	G	A	0.487762	0.0011	0.0146	0.9399422	-0.0877518	0.00893002	8.65E-23	-0.0125354	0.166383	0.939944	0.767276	0.8420811	13	FALSE	Vosa et al., 2018	Blood
IGHV3-48	ENSG00000211964	14	106994080	rs34169339	14	107000428	G	C	0.325175	-0.0126	0.0156	0.4192677	0.139827	0.00892173	2.33E-55	-0.0901115	0.111715	0.4198844	0.5509222	0.8799555	20	FALSE	Vosa et al., 2018	Blood
IGHV3-53	ENSG00000211967	14	107049006	rs11623507	14	107083151	G	C	0.223776	0.0056	0.0247	0.820641	-0.288894	0.0101974	1.46E-176	-0.0193842	0.0855011	0.8206466	0.3581942	0.2985322	20	FALSE	Vosa et al., 2018	Blood
IGHV1-69	ENSG00000211973	14	107170179	rs8009594	14	107185733	T	G	0.40035	0.0088	0.0099	0.3740628	-0.392545	0.00860507	0	-0.0224178	0.0252249	0.3741535	0.3430044	0.2258921	20	FALSE	Vosa et al., 2018	Blood
NRAS	ENSG00000213281	1	115253302	rs41310114	1	115313051	C	T	0.208042	-0.0116	0.0118	0.3255824	-0.0840182	0.0152916	3.92E-08	0.138065	0.142676	0.3332021	0.3281468	0.9800144	10	FALSE	Vosa et al., 2018	Blood
CHUK	ENSG00000213341	10	101968715	rs148211064	10	102066889	T	C	0.013986	0.076	0.0538	0.1577618	0.80837	0.0602829	5.31E-41	0.0940163	0.0669219	0.1600613	0.2125302	0.3488556	18	FALSE	Vosa et al., 2018	Blood
SIPA1	ENSG00000213445	11	65411984	rs2306363	11	65405600	T	G	0.201049	-0.0201	0.012	0.09393423	-0.213571	0.0114829	3.27E-77	0.0941139	0.0564148	0.09526642	0.06588937	0.5833139	20	FALSE	Vosa et al., 2018	Blood
KLRK1	ENSG00000213809	12	10534712	rs2255336	12	10532326	T	C	0.16958	-0.0005	0.0117	0.9659127	-0.2134	0.0104482	1.01E-92	0.00234302	0.0548267	0.9659128	0.8772844	0.5236586	20	FALSE	Vosa et al., 2018	Blood
IFI30	ENSG00000216490	19	18286449	rs190415956	19	18262971	C	T	0.0192308	-0.072	0.0457	0.1151428	0.395772	0.0580855	9.52E-12	-0.181923	0.118517	0.1247859	0.2438633			FALSE	Vosa et al., 2018	Blood
PPP3R1	ENSG00000221823	2	68423366	rs6758449	2	68437108	A	G	0.386364	0.0137	0.0123	0.2653559	-0.349588	0.00975589	3.32E-281	-0.039189	0.0352012	0.2655867	0.7320101	0.3625109	20	FALSE	Vosa et al., 2018	Blood
UBA52	ENSG00000221983	19	18685450	rs12608925	19	18687533	T	G	0.557692	-0.0014	0.0095	0.8828412	0.275529	0.00877334	1.71E-216	-0.0050811	0.0344795	0.8828425	0.2893193	0.2874441	20	FALSE	Vosa et al., 2018	Blood
PSMB11	ENSG00000222028	14	23512322	rs113525195	14	23499321	A	C	0.307692	-0.007	0.0108	0.5168891	0.175168	0.00934379	2.05E-78	-0.0399616	0.0616919	0.5171395	0.3613153	0.4102705	20	FALSE	Vosa et al., 2018	Blood
HLA-DPB1	ENSG00000223865	6	33049340	rs4713603	6	32979609	T	C	0.533217	-0.0065	0.0095	0.4938422	-0.203932	0.00887448	7.46E-117	0.0318734	0.0466048	0.4940336	0.08979198	0.4540885	19	FALSE	Vosa et al., 2018	Blood
IGHV4-59	ENSG00000224373	14	107082818	rs12590205	14	107082637	A	T	0.251748	0.0035	0.02	0.8610796	0.189028	0.0099525	1.95E-80	0.0185158	0.105809	0.8610855	0.3435875	0.05951649	20	FALSE	Vosa et al., 2018	Blood
HLA-DPA1	ENSG00000231389	6	33040449	rs34354079	6	32981555	A	G	0.0751748	0.0404	0.0168	0.01618301	-0.412465	0.021769	4.64E-80	-0.0979476	0.0410574	0.01705014	0.368619	0.4553132	12	FALSE	Vosa et al., 2018	Blood
TAPBP	ENSG00000231925	6	33274817	rs3130099	6	33282181	A	G	0.512238	0.014	0.0098	0.1531275	-0.139967	0.00890047	1.01E-55	-0.100024	0.0703048	0.1548193	0.6021892	0.3238752	20	FALSE	Vosa et al., 2018	Blood
HLA-DQB2	ENSG00000232629	6	32727593	rs1063355	6	32627714	T	G	0.393357	0.0439	0.0108	4.81E-05	-0.616725	0.00814144	0	-0.0711825	0.0175371	4.93E-05	6.13E-16	4.72E-09	20	TRUE	Vosa et al., 2018	Blood
HLA-DQA2	ENSG00000237541	6	32712055	rs9271520	6	32589771	G	A	0.325175	-0.0835	0.0145	8.48E-09	0.678712	0.00904117	0	-0.123027	0.0214268	9.37E-09	1.37E-16	1.44E-11	20	TRUE	Vosa et al., 2018	Blood
ASB14	ENSG00000239388	3	57314542	rs1046545	3	57305170	T	C	0.164336	-0.0204	0.0153	0.1824224	0.127317	0.0128592	4.13E-23	-0.16023	0.121258	0.1863664	0.5959439	0.2436803	20	FALSE	Vosa et al., 2018	Blood
IGKV2D-30	ENSG00000239571	2	89976079	rs6747215	2	88986443	G	C	0.0314685	0.0529	0.0274	0.05352549	0.124866	0.0162434	1.50E-14	0.423653	0.226249	0.06113696	0.05636907			FALSE	Vosa et al., 2018	Blood
LILRA4	ENSG00000239961	19	54847438	rs3826754	19	54866045	T	C	0.409091	-0.0168	0.0112	0.1336144	-0.0933082	0.00934446	1.77E-23	0.180049	0.121379	0.1379796	0.1262415	0.00935233	20	FALSE	Vosa et al., 2018	Blood
LILRA2	ENSG00000239998	19	55091707	rs1974208	19	55102898	C	T	0.0909091	-0.0065	0.0176	0.7118906	-0.407616	0.00982761	0	0.0159464	0.0431796	0.7119015	0.7790837	0.5390398	20	FALSE	Vosa et al., 2018	Blood
PSMB9	ENSG00000240065	6	32819637	rs2071477	6	32825348	C	T	0.428322	-0.0076	0.0097	0.4333305	0.384606	0.00879794	0	-0.0197605	0.0252247	0.4334044	7.01E-06	0.0390336	20	TRUE	Vosa et al., 2018	Blood
IGKV1-17	ENSG00000240382	2	89417084	rs17842625	2	90089300	A	G	0.0734266	-0.0327	0.03	0.2757131	-0.357582	0.0191922	1.78E-77	0.0914476	0.0840404	0.2765335	0.02079562	0.4616007	20	FALSE	Vosa et al., 2018	Blood
KIR3DL2	ENSG00000240403	19	55370280	rs6509905	19	55391706	A	T	0.38986	-0.0184	0.0138	0.1824224	-0.311981	0.0105137	1.68E-193	0.058978	0.0442781	0.1828637	0.8567837	0.03324585	20	FALSE	Vosa et al., 2018	Blood
IGKV1-16	ENSG00000240864	2	89399603	rs6745982	2	89015493	T	C	0.0314685	0.054	0.0277	0.0512407	0.189772	0.0192151	5.28E-23	0.284551	0.148781	0.05580499	0.3464158	0.07824198	11	FALSE	Vosa et al., 2018	Blood
HLA-DOB	ENSG00000241106	6	32782682	rs4148876	6	32796793	A	G	0.0769231	-0.1002	0.019	1.34E-07	0.686398	0.0165041	0	-0.145979	0.0279024	1.68E-07	5.11E-09	0.00013471	20	TRUE	Vosa et al., 2018	Blood
IGKV1D-16	ENSG00000241244	2	90139329	rs17620337	2	90112745	T	C	0.0751748	-0.0413	0.028	0.1402125	-0.341372	0.0247867	3.74E-43	0.120982	0.082491	0.1424814	0.1448392	0.413728	6	FALSE	Vosa et al., 2018	Blood
HLA-DMB	ENSG00000242574	6	32905626	rs209473	6	32922908	C	A	0.538462	-0.0212	0.0118	0.07239753	0.0980489	0.00893189	4.91E-28	-0.216219	0.121949	0.07622523	0.07049984	0.00265414	8	FALSE	Vosa et al., 2018	Blood
IGKV1-12	ENSG00000243290	2	89339981	rs112893923	2	90149828	C	G	0.0769231	-0.0829	0.0331	0.01226136	0.158522	0.0240783	4.59E-11	-0.522956	0.223402	0.01923893	0.06244041	0.00798756	6	FALSE	Vosa et al., 2018	Blood
KCTD7	ENSG00000243335	7	66185157	rs1796225	7	66038363	C	G	0.382867	-0.0098	0.0106	0.3552113	0.273955	0.00945492	1.36E-184	-0.0357723	0.0387122	0.3554561	5.12E-05	0.00665214	20	TRUE	Vosa et al., 2018	Blood
IGKV1-5	ENSG00000243466	2	89247147	rs111626937	2	90078462	T	A	0.0716783	-0.0421	0.0301	0.1619116	0.158549	0.0239304	3.46E-11	-0.265534	0.194032	0.1711529	0.06368864	0.7237798	7	FALSE	Vosa et al., 2018	Blood
KIR2DL3	ENSG00000243772	19	55272878	rs11084367	19	55213281	A	G	0.0104895	0.0005	0.0319	0.9874945	0.105199	0.0123622	1.74E-17	0.00475287	0.303234	0.9874945	0.9450956	0.3202972	3	FALSE	Vosa et al., 2018	Blood
LILRA6	ENSG00000244482	19	54733693	rs11666543	19	54711981	A	G	0.284965	0.0148	0.0115	0.1981094	-0.251481	0.012354	4.09E-92	-0.0588514	0.0458204	0.1990048	0.2568668	0.5243952	19	FALSE	Vosa et al., 2018	Blood
IGKV2D-40	ENSG00000251039	2	89890954	rs13392134	2	89088419	G	A	0.0314685	0.0223	0.0331	0.5004919	0.189443	0.0199057	1.78E-21	0.117714	0.17516	0.5015619	0.2512605	0.7896907	5	FALSE	Vosa et al., 2018	Blood
LYN	ENSG00000254087	8	56858155	rs1511724	8	56796697	C	G	0.608392	0.0089	0.0102	0.3829089	-0.157423	0.00890181	5.53E-70	-0.0565355	0.0648724	0.3834873	0.122731	0.3631603	20	FALSE	Vosa et al., 2018	Blood
SIGLEC12	ENSG00000254521	19	51999827	rs3829658	19	52000672	G	A	0.339161	-0.0071	0.0098	0.4687651	0.342189	0.00888049	0	-0.0207488	0.0286442	0.4688437	0.8348448	0.8681826	20	FALSE	Vosa et al., 2018	Blood
CTSO	ENSG00000256043	4	156860169	rs116465180	4	156842432	T	C	0.00699301	0.0227	0.0523	0.6642634	-1.79764	0.062143	5.39E-184	-0.0126277	0.029097	0.6642989	0.1691545	0.1357222	20	FALSE	Vosa et al., 2018	Blood
GAN	ENSG00000261609	16	81420681	rs310019	16	81420955	T	C	0.379371	-0.0011	0.0104	0.9157655	-0.126893	0.00971404	5.37E-39	0.00866875	0.0819618	0.9157682	0.8198123	0.4081642	20	FALSE	Vosa et al., 2018	Blood
DYNLL2	ENSG00000264364	17	56170903	rs9903806	17	56149521	G	A	0.318182	-0.0014	0.0109	0.8978005	0.12585	0.0111694	1.90E-29	-0.0111243	0.0866165	0.8978071	0.5156625	0.4181239	20	FALSE	Vosa et al., 2018	Blood

ALPHA SYNUCLEIN PATHWAY (PID)

Gene	probeID	ProbeChr	Probe bp	topSNP	topSNP chr	topSNP bp	A1	A2	Freq	b GWAS	se GWAS	p GWAS	b eQTL	se eQTL	p eQTL	b SMR	se SMR	p SMR	p SMR multi	p HEIDI	nsnp HEIDI	PASS BONF	Data source	Analyte
TOR1A	ENSG00000136827	9	132580818	rs13283293	9	132574477	G	A	0.713287	-0.0272	0.0111	0.01426776	1.28562	0.0342522	2.59E-308	-0.0211572	0.00865238	0.01447539	0.006742217	0.09684349	20	TRUE	Qi et al., 2018	Brain
UCHL1	ENSG00000154277	4	41264451	rs11556272	4	41259162	C	A	0.881119	0.0286	0.019	0.1322564	-0.57432	0.0919831	4.27E-10	-0.049798	0.0340304	0.1433748	0.1101422	0.01958312	9	FALSE	Qi et al., 2018	Brain
PRKCD	ENSG00000163932	3	53208379	rs13319192	3	53193548	G	T	0.97028	-0.0159	0.0295	0.5898985	0.572714	0.0816018	2.24E-12	-0.0277625	0.0516608	0.5909909	0.03512855	0.1877964	18	FALSE	Qi et al., 2018	Brain
MAPK3	ENSG00000102882	16	30130126	rs28529403	16	30134656	C	T	0.454545	0.0209	0.0105	0.0465385	0.3735	0.0379437	7.31E-23	0.0559571	0.0286814	0.05105843	0.04178409	0.1104368	20	FALSE	Qi et al., 2018	Brain
YES1	ENSG00000176105	18	767067	rs62091703	18	725062	C	T	0.856643	0.0241	0.0153	0.1152187	0.72925	0.0737866	4.92E-23	0.0330476	0.0212452	0.1198196	0.1039925	0.1589655	20	FALSE	Qi et al., 2018	Brain
LYN	ENSG00000254087	8	56858156	rs7005312	8	56806439	C	T	0.512238	-0.0087	0.01	0.3843004	0.289778	0.0454086	1.75E-10	-0.030023	0.0348284	0.3886732	0.3966343	0.8183085	20	FALSE	Qi et al., 2018	Brain
HCK	ENSG00000101336	20	30664825	rs1004910	20	30639430	T	G	0.115385	-0.0135	0.0164	0.4104109	-0.296991	0.0131428	4.62E-113	0.0454559	0.0552572	0.4107212	2.35E-05	0.7326997	20	TRUE	Vosa et al., 2018	Blood
TOR1A	ENSG00000136827	9	132580818	rs7025992	9	132557210	C	T	0.381119	0.0289	0.0102	0.00460653	-0.312434	0.0090402	9.92E-262	-0.0924996	0.0327565	0.00474492	0.00512674	0.2807156	20	FALSE	Vosa et al., 2018	Blood
UBE2L3	ENSG00000185651	22	21941029	rs5754234	22	21942978	T	A	0.160839	0.0156	0.0117	0.1824224	0.767251	0.00942381	0	0.0203323	0.0152513	0.1824811	0.2047384	0.9512169	20	FALSE	Vosa et al., 2018	Blood
SRC	ENSG00000197122	20	36003770	rs6017916	20	35971278	C	A	0.262238	-0.0238	0.0113	0.03518744	-0.0892017	0.00995666	3.28E-19	0.266811	0.130133	0.04033535	0.03529139	0.5998163	17	FALSE	Vosa et al., 2018	Blood
PTK2B	ENSG00000120899	8	27242951	rs6987305	8	27208126	G	A	0.645105	-0.0055	0.0101	0.58606	-0.450431	0.00901878	0	0.0122105	0.0224243	0.5860823	0.7301907	0.02714922	20	FALSE	Vosa et al., 2018	Blood
UCHL1	ENSG00000154277	4	41264451	rs13102133	4	41215837	G	C	0.152098	0.0054	0.0135	0.6891565	0.130288	0.0136054	1.01E-21	0.0414467	0.103707	0.6894132	0.3203532	0.9367355	20	FALSE	Vosa et al., 2018	Blood
PRKCD	ENSG00000163932	3	53208379	rs2230494	3	53220215	A	G	0.288462	0.0465	0.0115	5.27E-05	0.212967	0.00980744	1.49E-104	0.218344	0.0549273	7.03E-05	0.000141071	0.08661588	20	TRUE	Vosa et al., 2018	Blood
MAPK1	ENSG00000100030	22	22165379	rs5754932	22	22100718	T	C	0.034965	0.088	0.0302	0.00356936	0.175255	0.0140029	6.13E-36	0.502126	0.176929	0.00453967	0.00892983	0.00946718	20	FALSE	Vosa et al., 2018	Blood
PLD2	ENSG00000129219	17	4718560	rs2286670	17	4718027	T	G	0.132867	0.0202	0.0134	0.1316921	0.701666	0.0151959	0	0.0287886	0.0191076	0.1318978	0.02004697	0.00889413	20	FALSE	Vosa et al., 2018	Blood
MAPK3	ENSG00000102882	16	30130126	rs55732507	16	30141985	C	T	0.458042	0.0234	0.0102	0.02178374	0.541871	0.00838105	0	0.0431837	0.0188355	0.02186673	6.08E-11	0.3718903	20	TRUE	Vosa et al., 2018	Blood
PPP2R5D	ENSG00000112640	6	42966158	rs59541897	6	42969573	C	T	0.134615	-0.0254	0.0144	0.07775069	-0.145703	0.0098049	5.97E-50	0.174328	0.0995252	0.07984389	0.05115537	0.09534958	20	FALSE	Vosa et al., 2018	Blood
BAD	ENSG00000002330	11	64044739	rs947939	11	63885287	T	C	0.153846	0.0088	0.0132	0.5049851	0.122135	0.015812	1.13E-14	0.0720512	0.108479	0.506564	0.7124687	0.5591158	20	FALSE	Vosa et al., 2018	Blood
FGR	ENSG00000000938	1	27950181	rs34806307	1	27958339	T	C	0.0979021	0.0062	0.0193	0.7480259	0.164304	0.0152659	5.16E-27	0.037735	0.117518	0.7481342	0.5247065	0.3849722	14	FALSE	Vosa et al., 2018	Blood
GRK5	ENSG00000198873	10	121091116	rs4752263	10	120969217	A	G	0.381119	0.0164	0.0101	0.1044265	-0.133502	0.00900607	1.03E-49	-0.122844	0.0761066	0.1065041	0.1935354	0.6761112	20	FALSE	Vosa et al., 2018	Blood
BLK	ENSG00000136573	8	11386811	rs4840568	8	11351019	A	G	0.262238	0.0109	0.011	0.32173	-0.434204	0.00857443	0	-0.0251034	0.0253386	0.3218226	0.000727356	0.03258953	20	TRUE	Vosa et al., 2018	Blood
FKBP1A	ENSG00000088832	20	1361714	rs8392	20	1350709	C	T	0.423077	-0.0025	0.0113	0.8249064	-0.356669	0.00925942	0	0.00700931	0.0316826	0.8249092	0.1032871	0.243583	20	FALSE	Vosa et al., 2018	Blood
SNCA	ENSG00000145335	4	90702358	rs2736994	4	90784528	A	G	0.190559	-0.1709	0.0119	9.05E-47	0.260726	0.0109892	1.96E-124	-0.655478	0.0533522	1.08E-34	1.24E-27	3.95E-36	20	TRUE	Vosa et al., 2018	Blood
CSNK2A1	ENSG00000101266	20	491799	rs58317189	20	500293	T	C	0.0856643	0.0121	0.0199	0.5431608	-0.116546	0.0184918	2.93E-10	-0.103821	0.17154	0.5450261	0.5214933	0.4836248	5	FALSE	Vosa et al., 2018	Blood
FYN	ENSG00000010810	6	112088095	rs79756992	6	112039479	C	T	0.0472028	-0.0124	0.0239	0.6038804	-0.187955	0.0201547	1.10E-20	0.0659733	0.127355	0.6044392	0.001946241	7.18E-07	20	TRUE	Vosa et al., 2018	Blood
PLD1	ENSG00000075651	3	171423467	rs73178205	3	171540195	G	A	0.195804	-0.0008	0.0135	0.9527456	0.291227	0.0136861	1.78E-100	-0.002747	0.0463557	0.9527458	0.3793687	0.0791842	19	FALSE	Vosa et al., 2018	Blood

YES1	ENSG00000176105	18	767067	rs4121511	18	815330	T	C	0.321678	-0.0125	0.0112	0.2643916	-0.0966664	0.00915384	4.56E-26	0.129311	0.116508	0.2670464	0.1048314	0.2645566	20	FALSE	Vosa et al., 2018	Blood
PLCB2	ENSG00000137841	15	40585256	rs2305649	15	40589830	G	A	0.515734	0.0122	0.0099	0.2178284	-0.238311	0.00952017	2.73E-138	-0.0511936	0.0415927	0.2183855	0.4497906	0.6188394	20	FALSE	Vosa et al., 2018	Blood
PARK7	ENSG00000116288	1	8029958	rs35675666	1	8021973	T	G	0.129371	0.0018	0.0128	0.8881662	0.472141	0.0118487	0	0.00381242	0.0271107	0.8881669	0.8883643	0.5382203	20	FALSE	Vosa et al., 2018	Blood
SYK	ENSG00000165025	9	93612450	rs772407	9	93560850	C	A	0.456294	-0.0322	0.01	0.00128191	-0.45664	0.00850267	0	0.0705151	0.0219384	0.00130796	0.03225512	0.2242992	20	FALSE	Vosa et al., 2018	Blood
LYN	ENSG00000254087	8	56858155	rs1511724	8	56796697	C	G	0.608392	0.0089	0.0102	0.3829089	-0.157423	0.00890181	5.53E-70	-0.0565355	0.0648724	0.3834873	0.122731	0.3631603	20	FALSE	Vosa et al., 2018	Blood

ALZHEIMERS DISEASE (KEGG)

Gene	probeID	ProbeChr	Probe bp	topSNP	topSNP chr	topSNP bp	A1	A2	Freq	b GWAS	se GWAS	p GWAS	b eQTL	se eQTL	p eQTL	b SMR	se SMR	p SMR	p SMR multi	p HEIDI	nsnp HEIDI	PASS BONF	Data source	Analyte
UQCR10	ENSG00000184076	22	30164880	rs57122143	22	30220560	G	C	0.0472028	0.0166	0.0211	0.43144	0.587361	0.105526	2.61E-08	0.028262	0.0362805	0.4359874	0.6583984	0.6797582	16	FALSE	Qi et al., 2018	Brain
PPP3CC	ENSG00000120910	8	22348492	rs34649162	8	22314730	C	T	0.461538	-0.0296	0.0101	0.00338207	-1.11009	0.0300984	9.01E-298	0.0266646	0.00912708	0.00348368	0.03144998	0.1728065	20	FALSE	Qi et al., 2018	Brain
ADAM17	ENSG00000151694	2	9662268	rs11689299	2	9669472	C	T	0.673077	0.0206	0.01	0.03939854	0.279956	0.0404041	4.24E-12	0.073583	0.0372651	0.04831578	0.314301	0.4019187	20	FALSE	Qi et al., 2018	Brain
BACE2	ENSG00000182240	21	42594126	rs914187	21	42619749	C	T	0.597902	0.0061	0.0102	0.5498138	-1.105	0.0308358	3.17E-281	-0.0055204	0.00923209	0.5498693	0.9512765	0.8943346	20	FALSE	Qi et al., 2018	Brain
NDUFA2	ENSG00000131495	5	140022847	rs1962649	5	140030243	T	A	0.403846	-0.0074	0.0095	0.4360107	-0.216173	0.0374362	7.72E-09	0.0342318	0.0443442	0.4401408	0.2036837	0.03760531	20	FALSE	Qi et al., 2018	Brain
NDUFA3	ENSG00000170906	19	54610467	rs75622830	19	54611734	G	A	0.0891608	0.0217	0.0187	0.2458747	0.643918	0.0863621	8.92E-14	0.0337	0.0293906	0.2515372	0.2602034	0.7810692	10	FALSE	Qi et al., 2018	Brain
ATF6	ENSG00000118217	1	161834972	rs2499854	1	161930476	T	C	0.748252	-0.0033	0.0105	0.753304	-1.05313	0.0307506	4.78E-257	0.00313353	0.00997075	0.7533141	0.751056	0.5365948	20	FALSE	Qi et al., 2018	Brain
COX7A2L	ENSG00000115944	2	42578418	rs2041354	2	42572675	T	A	0.597902	-0.0146	0.0098	0.1362779	-1.16968	0.0305277	0	0.0124821	0.00838472	0.136574	0.5238639	0.6019102	20	FALSE	Qi et al., 2018	Brain
ADAM10	ENSG00000137845	15	58964790	rs12592302	15	58912586	A	T	0.903846	0.016	0.0157	0.3081516	0.341262	0.0567053	1.76E-09	0.0468849	0.0466607	0.3149915	0.3149915	0.2585262	20	FALSE	Qi et al., 2018	Brain
GNAQ	ENSG00000156052	9	80488688	rs1328526	9	80364301	C	T	0.734266	0.0034	0.011	0.7572524	0.470658	0.052394	2.63E-19	0.00722393	0.0233854	0.7573914	0.663995	0.6515798	20	FALSE	Qi et al., 2018	Brain
SDHA	ENSG00000073578	5	237585	rs62345236	5	283519	G	A	0.940559	-0.0039	0.0274	0.8868148	0.550901	0.100078	3.70E-08	-0.0070793	0.0497534	0.8868524	0.8868524	0.2003052	20	FALSE	Qi et al., 2018	Brain
CASP8	ENSG00000064012	2	202125300	rs10200279	2	202170655	T	C	0.286713	0.0319	0.0128	0.0126959	0.658191	0.089728	2.21E-13	0.0484662	0.020539	0.01828891	0.08317744	0.0531694	18	FALSE	Qi et al., 2018	Brain
CASP7	ENSG00000165806	10	115464802	rs3121487	10	115408679	T	G	0.61014	-0.0145	0.0096	0.1309371	-1.02262	0.0283134	1.18E-285	0.0141792	0.00939581	0.1312738	0.407857	0.8460837	20	FALSE	Qi et al., 2018	Brain
FADD	ENSG00000168040	11	70051382	rs10793035	11	70051966	G	A	0.52972	0.0121	0.0103	0.2400918	-0.373721	0.0427682	2.37E-18	-0.0323771	0.0278086	0.2443093	0.1409239	0.8710926	20	FALSE	Qi et al., 2018	Brain
SDHB	ENSG00000117118	1	17362941	rs2179793	1	17365733	A	C	0.512238	0.0169	0.0097	0.08146155	0.348093	0.0441299	3.07E-15	0.0485503	0.0285378	0.08889373	0.03711505	0.1036086	20	FALSE	Qi et al., 2018	Brain
SDHC	ENSG00000143252	1	161308515	rs113560757	1	161359070	A	C	0.0367133	0.0175	0.0208	0.400154	0.518613	0.0910306	1.22E-08	0.0337439	0.040542	0.4052289	0.4052289	0.3104948	6	FALSE	Qi et al., 2018	Brain
COX7A1	ENSG00000161281	19	36642797	rs35927732	19	36665304	C	T	0.945804	0.0387	0.0214	0.07054252	-0.519142	0.0832007	4.39E-10	-0.074546	0.0429182	0.08239926	0.08239926	0.3173088	11	FALSE	Qi et al., 2018	Brain
COX6C	ENSG00000164919	8	100895859	rs62534633	8	100896761	A	T	0.804196	-0.0277	0.0122	0.02317776	0.319034	0.0498751	1.59E-10	-0.0868247	0.040578	0.03237909	0.1219937	0.05605023	20	FALSE	Qi et al., 2018	Brain
COX6B1	ENSG00000126267	19	36144444	rs9917056	19	36182704	A	C	0.520979	-0.0095	0.0095	0.3173105	0.600986	0.045476	7.15E-40	-0.0158074	0.0158526	0.318692	0.230715	0.8714292	20	FALSE	Qi et al., 2018	Brain
BID	ENSG00000015475	22	18237221	rs113222188	22	18193381	A	G	0.195804	0.0116	0.0125	0.3534076	-0.495729	0.0624728	2.10E-15	-0.0233999	0.0253872	0.3566755	0.3723614	0.7469084	20	FALSE	Qi et al., 2018	Brain
NOS1	ENSG00000089250	12	117767961	rs3844051	12	117882701	A	G	0.597902	-0.0037	0.0101	0.7141139	0.343082	0.0346629	4.26E-23	-0.0107846	0.0294592	0.7143009	0.8817704	0.6490479	20	FALSE	Qi et al., 2018	Brain
MAPK3	ENSG00000102882	16	30130126	rs28529403	16	30134656	C	T	0.454545	0.0209	0.0105	0.0465385	0.3735	0.0379437	7.31E-23	0.0559571	0.0286814	0.05105843	0.04178409	0.1104368	20	FALSE	Qi et al., 2018	Brain
COX6A1	ENSG00000111775	12	120877219	rs1037611	12	120920117	C	T	0.370629	-0.0085	0.0104	0.4137526	-0.508877	0.0485481	1.05E-25	0.0167034	0.0204992	0.4151671	0.2204888	0.03245687	20	FALSE	Qi et al., 2018	Brain
UQCRH	ENSG00000173660	1	46775875	rs62620990	1	46685792	A	G	0.966783	0.0966	0.0344	0.00498286	1.49424	0.188875	2.55E-15	0.0646483	0.024429	0.00813604	0.04879686	0.6331779	14	FALSE	Qi et al., 2018	Brain
UQCRFS1	ENSG00000169021	19	29701310	rs10410803	19	29683404	G	T	0.928322	0.0092	0.017	0.588386	-0.828454	0.0802974	5.88E-25	-0.011105	0.0205484	0.5888981	0.445686	0.4635803	11	FALSE	Qi et al., 2018	Brain
NDUFB1	ENSG00000183648	14	92585363	rs35791930	14	92581579	A	G	0.65035	0.0017	0.0102	0.8676323	0.28941	0.0470031	7.40E-10	0.00587401	0.035257	0.8676804	0.7631045	0.8314183	11	FALSE	Qi et al., 2018	Brain
NDUFA10	ENSG00000130414	2	240898343	rs1465880	2	240932326	G	A	0.641608	0.0031	0.0107	0.7720307	-0.437565	0.0421047	2.69E-25	-0.0070847	0.024463	0.7721168	0.9196105	0.3720606	20	FALSE	Qi et al., 2018	Brain
ITPR2	ENSG00000123104	12	26738236	rs4322504	12	26982905	C	T	0.879371	0.0025	0.0168	0.8817039	-0.635378	0.0589142	4.06E-27	-0.0039347	0.0264435	0.8817151	0.4620927	0.3315176	20	FALSE	Qi et al., 2018	Brain
PSEN2	ENSG00000143801	1	227070845	rs1800674	1	227085824	G	A	0.631119	-0.0023	0.0098	0.8144463	0.401368	0.0406571	5.51E-23	-0.0057304	0.0244234	0.8144978	0.2154509	0.07898593	20	FALSE	Qi et al., 2018	Brain
GSK3B	ENSG00000082701	3	119676717	rs2037547	3	119544615	C	T	0.774476	-0.0186	0.0121	0.1242468	-0.495188	0.056669	2.37E-18	0.0375615	0.0248104	0.1300402	0.1318153	0.3920652	20	FALSE	Qi et al., 2018	Brain
NDUFA6	ENSG00000184983	22	42484244	rs4822099	22	42659964	G	A	0.958042	-0.0055	0.0263	0.8343503	-1.58766	0.13722	5.83E-31	0.00346421	0.0165679	0.8343769	0.3373372	0.03151836	20	FALSE	Qi et al., 2018	Brain
NDUFV3	ENSG00000160194	21	44314768	rs4148974	21	44323720	C	T	0.935315	-0.035	0.0194	0.07121192	0.980782	0.0805804	4.41E-34	-0.0356858	0.0199962	0.07432211	0.005561132	0.00541366	20	FALSE	Qi et al., 2018	Brain
COX4I2	ENSG00000131055	20	30229250	rs6119610	20	30225540	G	T	0.73951	0.0574	0.0125	4.39E-06	-0.386212	0.0600892	1.30E-10	-0.148623	0.0397774	0.0001867	1.11E-05	0.784654	20	TRUE	Qi et al., 2018	Brain
MAPT	ENSG00000186868	17	44038724	rs199502	17	44862347	G	A	0.743007	0.1949	0.0156	8.09E-36	0.734607	0.0582562	1.86E-36	0.265312	0.0298938	6.98E-19	3.51E-24	0.01172117	17	TRUE	Qi et al., 2018	Brain
NDUFS8	ENSG00000110717	11	67801097	rs10896289	11	67811262	C	A	0.823427	0.0057	0.0125	0.64839	0.436246	0.0580376	5.62E-14	0.013066	0.0287062	0.6489918	0.6537591	0.6640882	20	FALSE	Qi et al., 2018	Brain
NDUFS1	ENSG00000023228	2	207005238	rs4147713	2	207015772	C	A	0.494755	0.016	0.0094	0.08873144	-0.387458	0.0397031	1.69E-22	-0.0412948	0.0246269	0.09357906	0.1145175	0.07825864	20	FALSE	Qi et al., 2018	Brain
NDUFS4	ENSG00000164258	5	52917815	rs72751833	5	52884611	A	G	0.851399	-0.0041	0.0135	0.7613536	-0.326692	0.0594593	3.92E-08	0.01255	0.0413864	0.7617064	0.7617064	0.4818882	15	FALSE	Qi et al., 2018	Brain
NDUFV1	ENSG00000167792	11	67377164	rs10896190	11	67406123	T	C	0.41958	-0.0108	0.0098	0.2704439	0.436235	0.0494902	1.20E-18	-0.0247573	0.0226398	0.2741623	0.3459466	0.3289348	20	FALSE	Qi et al., 2018	Brain
NDUFS5	ENSG00000168653	1	39496149	rs4147753	1	39470364	A	G	0.681818	0.0187	0.011	0.08913093	0.49063	0.0418785	1.06E-31	0.0381143	0.022655	0.09249512	0.3675313	0.2352027	20	FALSE	Qi et al., 2018	Brain
NDUFS2	ENSG00000158864	1	161175539	rs4379692	1	161186313	C	T	0.659091	0.0061	0.0102	0.5498138	0.365141	0.0408725	4.12E-19	0.0167059	0.027997	0.5507052	0.03213519	0.01327971	20	FALSE	Qi et al., 2018	Brain
NDUFB5	ENSG00000136521	3	179333956	rs9820574	3	179350391	A	G	0.554196	-0.0042	0.0108	0.6973583	-0.297577	0.0361775	1.94E-16	0.014114	0.0363336	0.6976794	0.9029469	0.9144498	16	FALSE	Qi et al., 2018	Brain
FAS	ENSG00000026103	10	90762978	rs1926196	10	90753748	C	T	0.468531	0.0007	0.0095	0.9412617	1.01794	0.0254522	0	0.00068766	0.0093326	0.9412618	0.8560859	0.6987833	20	FALSE	Qi et al., 2018	Brain
CALML6	ENSG00000169885	1	1847499	rs35991586	1	1841919	G	A	0.807692	-0.0106	0.0165	0.5205978	0.43008	0.0598023	6.40E-13	-0.0246466	0.0385177	0.522253	0.6116863	0.9959391	14	FALSE	Qi et al., 2018	Brain
UQCR10	ENSG00000184076	22	30164880	rs73394838	22	30225973	G	A	0.048951	0.0218	0.0208	0.2946032	0.264438	0.0129579	1.44E-92	0.0824391	0.0787611	0.2952391	0.4664088	0.3186376	20	FALSE	Vosa et al., 2018	Blood
NAE1	ENSG00000159593	16	66871968	rs7206715	16	66750938	T	C	0.423077	-0.005	0.0096	0.6024829	-0.146569	0.00893028	1.55E-60	0.0341136	0.0655311	0.6026655	0.7714493	0.9042439	20	FALSE	Vosa et al., 2018	Blood
CALM2	ENSG00000143933	2	47338208	rs6726419	2	47315031	A	G	0.0874126	-0.0003	0.0211	0.9886561	-0.153701	0.00955926	3.59E-58	0.00195184	0.137279	0.9886561	0.7189064	0.4980201	20	FALSE	Vosa et al., 2018	Blood
NDUFA4	ENSG00000189043	7	10975730	rs4720916	7	10959562	A	G	0.417832	-0.0086	0.01	0.389789	0.0659388	0.00936817	1.94E-12	-0.130424	0.152784	0.393298	0.2383179	0.02267184	20	FALSE	Vosa et al., 2018	Blood
CALM1	ENSG00000198668	14	90868725	rs7150626	14	90858484	T	C	0.79021	0.0139	0.0127	0.2737409	-0.231433	0.00883439	2.89E-151	-0.0600605	0.0549233	0.2741593	0.2292752	0.3046809	20	FALSE	Vosa et al., 2018	Blood
ATP2A1	ENSG00000196296	16	28902778	rs4788101	16	28867804	T	C	0.332168	-0.0513	0.0097	1.23E-07	0.109029	0.0100918	3.30E-27	-0.470516	0.0990545	2.03E-06	2.88E-05	0.1147649	20	TRUE	Vosa et al., 2018	Blood
EIF2AK3	ENSG00000172071	2	88891676	rs867529	2	88913273	C	G	0.27972	0.0366	0.0104	0.0004328	0.0913455	0.0098592	1.95E-20	0.400677	0.12179	0.00100226	0.09206366	0.00135096	20	FALSE	Vosa et al., 2018	Blood
PPP3CC	ENSG00000120910	8	22348492	rs9886589	8	22298839	G	A	0.0629371	-0.0668	0.0259	0.00990436	-0.139039	0.0115399	1.97E-33	0.48044	0.190498	0.01166847	0.0123952	0.1053589	20	FALSE	Vosa et al., 2018	Blood
ATP2A3	ENSG00000074370	17	3847452	rs887387	17	3828702	C	T	0.372378	0.0073	0.0106	0.4910251	-0.122762	0.00958466	1.48E-37	-0.0594646	0.0864705	0.4916506	0.3167747	0.2003425	17	FALSE	Vosa et al., 2018	Blood
PPP3R1	ENSG00000221823	2	68423366	rs6758449	2	68437108	A	G	0.386364	0.0137	0.0123	0.2653559	-0.349588	0.00975589	3.32E-281	-0.039189	0.0352012	0.2655867	0.7320101	0.3625109	20	FALSE	Vosa et al., 2018	Blood
ATP2A2	ENSG00000174437	12	110753729	rs149790157	12	110897107	G	A	0.00524476	0.0225	0.0513	0.6609539	0.465129	0.0508038	5.42E-20	0.0483737	0.110418	0.6613181	0.9985102	0.8475875	4	FALSE	Vosa et al., 2018	Blood
NDUFS7	ENSG00000115286	19	1389554	rs7256647	19	1390839	A	G	0.0244755	0.0303	0.032	0.3437025	0.0689929	0.0115207	2.12E-09	0.439176	0.469578	0.3496561	0.3496561	0.1181989	8	FALSE	Vosa et al., 2018	Blood
ADAM17	ENSG00000151694	2	9662268	rs13392064	2	9626365	G	C	0.587413	0.0154	0.0097	0.1123703	0.252129	0.00909893	5.33E-169	0.0610799	0.0385355	0.1129597	0.2775535	0.7370051	20	FALSE	Vosa et al., 2018	Blood
BACE2	ENSG00000182240	21	42594126	rs2837964	21	42520646	A	C	0.295455	-0.0192	0.011	0.08090578	-0.205667	0.00938089	1.53E-106	0.0933549	0.0536538	0.08186797	0.3996733	0.01396193	20	FALSE	Vosa et al., 2018	Blood
ATP5PO	ENSG00000241837	21	35282020	rs2834294	21	35280630	G	A	0.386364	0.0042	0.01	0.6744855	0.164727	0.00897674	3.28E-75	0.0254968	0.0607225	0.6745658	0.171909	0.129851	20	FALSE	Vosa et al., 2018	Blood
MME	ENSG00000196549	3	154821705	rs11719420	3	154857146	G	A	0.335664	-0.0046	0.0109	0.6730116	0.128874	0.00948178	4.48E-42	-0.0356937	0.0846192	0.67316	0.5570706	0.2609695	13	FALSE	Vosa et al., 2018	Blood
ITPR3	ENSG00000096433	6	33626436	rs9469532	6	33601132	G	A	0.657343	0.0063	0.0099	0.5245394	0.495245	0.00851021	0	0.012721	0.0199913	0.5245642	0.4296533	0.9713585	20	FALSE	Vosa et al., 2018	Blood
LRP1	ENSG00000123384	12	57564705	rs4759273	12	57444567	T	C	0.0594406	0.0147	0.021	0.4839273	0.18224	0.0285205	1.66E-10	0.0806627	0.115922	0.4865309	0.4865309			FALSE	Vosa et al., 2018	Blood
COX4I1	ENSG00000131143	16	85836444	rs96603	16	85815097	C	T	0.195804	0.0031	0.0125	0.8041344	0.176349	0.00896074	3.19E-86	0.0175788	0.0708878	0.8041496	0.8912853	0.6433744	20	FALSE	Vosa et al., 2018	Blood
ATP5PD	ENSG00000167863	17	73039019	rs9915968	17	73059616	C	G	0.138112	0.0044	0.0129	0.7330394	0.144025	0.00900221	1.30E-57	0.0305504	0.0895884	0.7330977	0.006853438	0.02785211	20	FALSE	Vosa et al., 2018	Blood
NDUFA2	ENSG00000131495	5	140022847	rs778593	5	140027216	C	T	0.388112	-0.0082	0.0095	0.3880507	-0.314344	0.00896857	3.96E-269	0.026086	0.0302308	0.3881945	0.02238567	0.00647607	20	FALSE	Vosa et al., 2018	Blood
NDUFA3	ENSG00000170906	19	54609300	rs75622830	19	54611734	G	A	0.0891608	0.0217	0.0187	0.2458747	0.67835	0.0175025	0	0.0319894	0.0275792	0.2460863	0.5203377	0.7768712	18	FALSE	Vosa et al., 2018	Blood
CAPN2	ENSG00000162909	1	223926507	rs142113063	1	223667446	T	C	0.0104895	-0.1265	0.0731	0.08353988	-0.799977	0.0539119	8.25E-50	0.158129	0.0919969	0.08564045	0.1933305	0.7386919	17	FALSE	Vosa et al., 2018	Blood
LPL	ENSG00000175445	8	19791998	rs79445051	8	19933089	G	C	0.0961538	-0.0082	0.0181	0.6505209	0.978118	0.0175786	0	-0.0083835	0.0185055	0.6505317	0.452883	0.06891311	20	FALSE	Vosa et al., 2018	Blood
CAPN1	ENSG00000014216	11	64963757	rs12800339	11	64947240	C	T	0.309441	-0.0031	0.0104	0.7656445	0.46292	0.00936044	0	-0.0066966	0.0224665	0.7656486	0.5234536	0.7123003	20	FALSE	Vosa et al., 2018	Blood
APAF1	ENSG00000120868	12	99084061	rs3847874	12	99027045	T	A	0.183566	-0.0104	0.0134	0.4376785	0.3654	0.0133887	5.34E-164	-0.028462	0.036687	0.4378637	0.6655424	0.1954931	20	FALSE	Vosa et al., 2018	Blood
APH1A	ENSG00000117362	1	150239892	rs113007736	1	150295738	G	T	0.141608	-0.0439	0.015	0.00342616	0.132954	0.0183623	4.47E-13	-0.330189	0.121689	0.00665982	0.02265119	0.0876561	20	FALSE	Vosa et al., 2018	Blood
UQCRQ	ENSG00000164405	5	132202987	rs12514058	5	132285833	T	C	0.162587	0.0181	0.0196	0.3557626	-0.115308	0.0123432	9.47E-21	-0.156971	0.170808	0.3581007	0.5417298	0.00625567	12	FALSE	Vosa et al., 2018	Blood
ATP5F1D	ENSG00000099624	19	1243286	rs12983363	19	1241087	C	G	0.253497	0.0019	0.0126	0.8801385	-0.305439	0.00965684	1.46E-219	-0.0062206	0.0412526	0.8801398	0.653617	0.9865857	20	FALSE	Vosa et al., 2018	Blood
GAPDH	ENSG00000111640	12	6645315	rs9634157	12	6648198	G	A	0.211538	0.0029	0.0117	0.8042402	-0.308401	0.00901343	1.41E-256	-0.0094033	0.0379386	0.8042452	0.6487829	0.454118	20	FALSE	Vosa et al., 2018	Blood
ATF6	ENSG00000118217	1	161834972	rs1136046	1	161928771	A	G	0.0874126	0.0056	0.0151	0.7107411	0.448253	0.00963657	0	0.0124929	0.0336874	0.7107499	0.03404916	0.4368408	20	FALSE	Vosa et al., 2018	Blood
COX7A2L	ENSG00000115944	2	42578418	rs1981664	2	42577170	A	G	0.398601	0.0156	0.0098	0.1114214	0.397109	0.0089974	0	0.0392839	0.0246944	0.111654	0.272528	0.8412455	20	FALSE	Vosa et al., 2018	Blood
ATP5MC3	ENSG00000154518	2	176045160	rs62184512	2	175999582	A	G	0.0332168	0.0053	0.0273	0.8460669	0.294471	0.0304464	3.97E-22	0.0179984	0.0927272	0.8460975	0.7197833	0.8079898	20	FALSE	Vosa et al., 2018	Blood
ATP5MC2	ENSG00000135390	12	54048851	rs916134	12	54069803	G	A	0.286713	0.0155	0.0104	0.1361231	0.12647	0.00939013	2.40E-41	0.122559	0.0827349	0.1385153	0.003241038	0.00440598	20	FALSE	Vosa et al., 2018	Blood
ADAM10	ENSG00000137845	15	58964790	rs7161799	15	58770523	T	C	0.0891608	-0.0223	0.019	0.2405215	0.497122	0.0135183	4.79E-296	-0.0448582	0.0382395	0.240761	0.1511186	0.9392393	20	FALSE	Vosa et al., 2018	Blood
ATP5MC1	ENSG00000159199	17	46971680	rs12948439	17	46967124	G	A	0.444056	0.0185	0.0095	0.05149058	0.333424	0.00870514	0	0.0554849	0.028529	0.05179227	0.02705404	0.1026207	20	FALSE	Vosa et al., 2018	Blood
ATP5PB	ENSG00000116459	1	111998440	rs6673170	1	111974483	A	G	0.293706	0.0111	0.01	0.266999	-0.103747	0.0101617	1.80E-24	-0.106991	0.0969563	0.2698113	0.5037224	0.5931423	20	FALSE	Vosa et al., 2018	Blood
ATP5F1E	ENSG00000124172	20	57603979	rs16984980	20	57607449	A	C	0.113636	-0.0151	0.0196	0.4410578	-0.100203	0.0130496	1.61E-14	0.150694	0.196585	0.4433434	0.3947478	0.2917199	4	FALSE	Vosa et al., 2018	Blood
COX5A	ENSG00000178741	15	75221320	rs7495033	15	75206225	T	A	0.318182	-0.0158	0.0103	0.1250344	-0.088056	0.00926554	2.03E-21	0.179431	0.118485	0.1299295	0.001818658	0.00175507	20	FALSE	Vosa et al., 2018	Blood

CDK5R1	ENSG00000176749	17	30815955	rs62065493	17	30822620	G	C	0.505245	-0.0049	0.0098	0.6170751	0.766212	0.00843309	0	-0.0063951	0.0127904	0.6170804	0.4328684	0.9868228	20	FALSE	Vosa et al., 2018	Blood
ERN1	ENSG00000178607	17	62163928	rs16947438	17	62146498	T	G	0.141608	-0.0226	0.0139	0.1039711	-0.339306	0.0110601	1.10E-206	0.0666065	0.0410234	0.1044568	0.1424763	0.2556949	20	FALSE	Vosa et al., 2018	Blood
GNAQ	ENSG00000156052	9	80488688	rs1041584	9	80638165	C	T	0.314685	0.0053	0.0111	0.6330222	-0.141347	0.00948131	2.92E-50	-0.0374963	0.0785702	0.6331964	0.8881655	0.8630081	20	FALSE	Vosa et al., 2018	Blood
UQCRHL	ENSG00000233954	1	16133936	rs66460266	1	16131729	T	C	0.0856643	-0.0013	0.0147	0.9295306	-0.11513	0.0130264	9.73E-19	0.0112916	0.127688	0.9295342	0.8527658	0.5673836	20	FALSE	Vosa et al., 2018	Blood
ATP5F1A	ENSG00000152234	18	43674205	rs1800640	18	43671400	C	T	0.395105	-0.0307	0.0101	0.00236889	-0.351093	0.00972249	1.50E-285	0.0874411	0.028869	0.00245452	0.005079008	0.1423798	20	FALSE	Vosa et al., 2018	Blood
BACE1	ENSG00000186318	11	117171688	rs477036	11	117183914	T	C	0.538462	-0.0245	0.0098	0.01241933	-0.224934	0.00891019	1.30E-140	0.108921	0.0437814	0.0128525	0.01696624	0.2267902	20	FALSE	Vosa et al., 2018	Blood
SDHA	ENSG00000073578	5	237585	rs35947614	5	224397	G	A	0.127622	0.0074	0.0158	0.6395312	0.216612	0.0169144	1.51E-37	0.0341625	0.0729902	0.6397549	0.4680302	0.15962	20	FALSE	Vosa et al., 2018	Blood
CALM3	ENSG00000160014	19	47109190	rs7260181	19	47098427	C	T	0.475524	0.0009	0.0103	0.9303705	0.167798	0.00891914	5.89E-79	0.00536361	0.0613842	0.9303713	0.224938	0.02891854	20	FALSE	Vosa et al., 2018	Blood
NCSTN	ENSG00000162736	1	160320902	rs2038780	1	160317619	C	T	0.47028	0.0151	0.0101	0.1349015	0.538963	0.00838431	0	0.0280168	0.0187447	0.135007	0.7362723	0.8848456	20	FALSE	Vosa et al., 2018	Blood
CASP9	ENSG00000132906	1	15835178	rs9298	1	15897020	T	C	0.307692	-0.0019	0.0102	0.8522295	0.364971	0.00902445	0	-0.0052059	0.0279477	0.852231	0.3513613	0.1753266	20	FALSE	Vosa et al., 2018	Blood
PLCB2	ENSG00000137841	15	40585256	rs2305649	15	40589830	G	A	0.515734	0.0122	0.0099	0.2178284	-0.238311	0.00952017	2.73E-138	-0.0511936	0.0415927	0.2183855	0.4497906	0.6188394	20	FALSE	Vosa et al., 2018	Blood
COX8A	ENSG00000176340	11	63743047	rs10751004	11	63741510	T	A	0.447552	-0.0197	0.0098	0.04440959	-0.104182	0.00940999	1.73E-28	0.189093	0.0956043	0.04794376	0.07410413	0.5034984	20	FALSE	Vosa et al., 2018	Blood
CASP8	ENSG00000064012	2	202125300	rs7560328	2	202164837	A	C	0.398601	0.0194	0.0096	0.04329702	-0.214284	0.00890084	4.61E-128	-0.090534	0.0449579	0.04403565	0.4704423	0.714529	20	FALSE	Vosa et al., 2018	Blood
CASP7	ENSG00000165806	10	115464802	rs4353229	10	115489589	C	T	0.234266	-0.0125	0.0107	0.2427163	-0.66876	0.00909944	0	0.0186913	0.0160018	0.2427758	0.6036084	0.7677355	20	FALSE	Vosa et al., 2018	Blood
PLCB1	ENSG00000182621	20	8530913	rs2719795	20	8408544	A	G	0.286713	-0.0211	0.0111	0.05731499	-0.576306	0.00899977	0	0.0366125	0.0192691	0.05742473	0.2647413	0.763481	20	FALSE	Vosa et al., 2018	Blood
PLCB3	ENSG00000149782	11	64027808	rs7943988	11	64023971	A	G	0.288462	0.0165	0.0102	0.1057387	-0.107197	0.0118517	1.50E-19	-0.153923	0.096662	0.1112993	0.4351191	0.545469	20	FALSE	Vosa et al., 2018	Blood
TNF	ENSG00000232810	6	31544728	rs1121800	6	31535074	A	T	0.393357	0.0206	0.0098	0.0355497	0.212105	0.00931607	9.61E-115	0.0971219	0.0464001	0.03633677	0.000716509	0.06478115	20	FALSE	Vosa et al., 2018	Blood
FADD	ENSG00000168040	11	70051382	rs10898853	11	70049253	C	T	0.363636	0.0116	0.0105	0.2692628	0.301927	0.00918661	6.72E-237	0.0384199	0.0347962	0.2695332	0.8696505	0.4957978	20	FALSE	Vosa et al., 2018	Blood
SDHB	ENSG00000117118	1	17362941	rs138306655	1	16521702	T	C	0.00874126	0.1803	0.0935	0.05381257	-0.502354	0.0697366	5.86E-13	-0.35891	0.192677	0.06249667	0.06249667	0.02639787	3	FALSE	Vosa et al., 2018	Blood
SDHC	ENSG00000143252	1	161308515	rs59559479	1	161308157	C	T	0.0367133	0.0201	0.0209	0.336189	-0.416317	0.0124713	2.50E-244	-0.0482805	0.0502229	0.3363895	0.002804097	0.02548781	16	FALSE	Vosa et al., 2018	Blood
BAD	ENSG00000002330	11	64044739	rs947939	11	63885287	T	C	0.153846	0.0088	0.0132	0.5049851	0.122135	0.015812	1.13E-14	0.0720512	0.108479	0.506564	0.7124687	0.5591158	20	FALSE	Vosa et al., 2018	Blood
COX7A2	ENSG00000112695	6	75953715	rs3756797	6	75996546	T	C	0.204545	-0.0222	0.0121	0.06654856	0.0727081	0.0116445	4.27E-10	-0.305331	0.173454	0.07835824	0.07082076	0.7454991	6	FALSE	Vosa et al., 2018	Blood
COX6C	ENSG00000164919	8	100895859	rs35031559	8	100876705	T	C	0.195804	0.0267	0.0121	0.02734121	0.27614	0.0108457	5.36E-143	0.09669	0.0439826	0.02792269	0.02272508	0.00701806	20	FALSE	Vosa et al., 2018	Blood
COX6B1	ENSG00000126267	19	36144444	rs4806187	19	36149761	A	G	0.480769	-0.0068	0.0094	0.4694316	0.38699	0.00887791	0	-0.0175715	0.0242934	0.4694927	0.5378458	0.6401565	20	FALSE	Vosa et al., 2018	Blood
CACNA1D	ENSG00000157388	3	53687586	rs3774532	3	53737866	A	C	0.305944	0.0204	0.0108	0.05890672	-0.070617	0.0100086	1.72E-12	-0.288882	0.158323	0.06805637	0.03697435	0.8661658	11	FALSE	Vosa et al., 2018	Blood
BID	ENSG00000015475	22	18237221	rs3788279	22	18200386	G	A	0.512238	0.0075	0.01	0.4532547	-0.110254	0.00906502	4.92E-34	-0.0680246	0.0908718	0.4541115	0.726087	0.8264111	20	FALSE	Vosa et al., 2018	Blood
ATP5F1C	ENSG00000165629	10	7839935	rs4749283	10	7868021	C	G	0.223776	0.0068	0.0117	0.561108	0.072236	0.00939227	1.46E-14	0.0941359	0.162431	0.5622221	0.5463094	0.03365228	13	FALSE	Vosa et al., 2018	Blood
GRIN2D	ENSG00000105464	19	48923160	rs275837	19	48876190	C	A	0.456294	-0.0138	0.01	0.1675866	0.0759207	0.00913342	9.38E-17	-0.181769	0.133519	0.1733978	0.5205338	0.4598098	20	FALSE	Vosa et al., 2018	Blood
CACNA1C	ENSG00000151067	12	2441030	rs10505724	12	2782070	C	T	0.129371	0.0011	0.014	0.9373735	-0.0707963	0.0127213	2.62E-08	-0.0155375	0.19777	0.9373797	0.9546931	0.8755873	7	FALSE	Vosa et al., 2018	Blood
CASP3	ENSG00000164305	4	185559756	rs72689261	4	185590897	A	C	0.111888	0.0045	0.0146	0.7579156	-0.56825	0.0151553	1.11E-307	-0.0079191	0.0256938	0.7579235	0.7005626	0.8843126	20	FALSE	Vosa et al., 2018	Blood
TNFRSF1A	ENSG00000067182	12	6444601	rs1800692	12	6442346	A	G	0.372378	-0.0063	0.0103	0.540769	0.258183	0.00955512	8.52E-161	-0.0244013	0.0399044	0.5408727	0.3115939	0.1080935	11	FALSE	Vosa et al., 2018	Blood
UQCRB	ENSG00000156467	8	97243005	rs871798	8	97257982	C	T	0.384615	0.0158	0.0103	0.1250344	0.180079	0.0100904	3.07E-71	0.0877391	0.0574079	0.1264268	0.5705188	0.7504026	20	FALSE	Vosa et al., 2018	Blood
MAPK3	ENSG00000102882	16	30130126	rs55732507	16	30141985	C	T	0.458042	0.0234	0.0102	0.02178374	0.541871	0.00838105	0	0.0431837	0.0188355	0.02186673	6.08E-11	0.3718903	20	TRUE	Vosa et al., 2018	Blood
UQCRH	ENSG00000173660	1	46775875	rs41292543	1	46774783	G	A	0.115385	-0.0378	0.0156	0.01538967	-0.472657	0.0204094	1.19E-118	0.0799734	0.0331851	0.01595594	0.04502816	0.3848949	20	FALSE	Vosa et al., 2018	Blood
UQCRC2	ENSG00000140740	16	21979481	rs4783432	16	21951415	G	A	0.802448	0.0071	0.0132	0.5906607	0.0793681	0.00894046	6.84E-19	0.0894566	0.166619	0.5913407	0.6886895	0.8618073	20	FALSE	Vosa et al., 2018	Blood
MAPK1	ENSG00000100030	22	22165379	rs5754932	22	22100718	T	C	0.034965	0.088	0.0302	0.00356936	0.175255	0.0140029	6.13E-36	0.502126	0.176929	0.00453967	0.00892983	0.00946718	20	FALSE	Vosa et al., 2018	Blood
NDUFB3	ENSG00000119013	2	201943314	rs7597440	2	201842010	A	G	0.206294	0.0034	0.0124	0.7839359	-0.0817931	0.0126356	9.59E-11	-0.0415683	0.151738	0.7841247	0.7841247	0.5632118	8	FALSE	Vosa et al., 2018	Blood
NDUFB1	ENSG00000183648	14	92585363	rs2295727	14	92600785	G	A	0.041958	-0.0026	0.0193	0.8928372	0.181705	0.0145523	8.86E-36	-0.0143089	0.106222	0.8928434	0.9085113	0.8410617	16	FALSE	Vosa et al., 2018	Blood
NDUFB2	ENSG00000090266	7	140406583	rs2968550	7	140401122	G	A	0.159091	-0.0009	0.0133	0.946049	-0.411647	0.00965856	0	0.00218634	0.0323093	0.9460491	0.2452075	0.1561006	20	FALSE	Vosa et al., 2018	Blood
ITPR1	ENSG00000150995	3	4712278	rs2322800	3	4512462	A	G	0.0699301	-0.0224	0.0192	0.243345	-0.316164	0.0124217	6.61E-143	0.0708493	0.0607918	0.2438397	0.3115873	0.3155295	20	FALSE	Vosa et al., 2018	Blood
NDUFA10	ENSG00000130414	2	240898343	rs10166776	2	240967312	A	G	0.321678	0.0115	0.0108	0.2869597	-0.563001	0.00842622	0	-0.0204263	0.0191854	0.2870209	0.4366444	0.2411654	20	FALSE	Vosa et al., 2018	Blood
ITPR2	ENSG00000123104	12	26738236	rs74976642	12	26998022	A	G	0.120629	-0.0029	0.0163	0.8587904	-0.583457	0.016515	2.11E-273	0.00497038	0.0279373	0.8587922	0.6639374	0.4636295	20	FALSE	Vosa et al., 2018	Blood
NDUFAB1	ENSG00000004779	16	23600000	rs463399	16	23608882	T	C	0.0804196	0.0138	0.0185	0.4557001	-0.111416	0.0132182	3.49E-17	-0.12386	0.166693	0.4574563	0.3752322	0.5499514	19	FALSE	Vosa et al., 2018	Blood
PSEN2	ENSG00000143801	1	227070845	rs3795445	1	227180947	C	A	0.520979	-0.0142	0.0096	0.1390958	0.169701	0.00951194	3.41E-71	-0.0836767	0.0567643	0.1404523	0.2447612	0.1017578	20	FALSE	Vosa et al., 2018	Blood
GSK3B	ENSG00000082701	3	119676717	rs6765483	3	119927508	T	A	0.211538	0.0104	0.0113	0.3573878	0.280376	0.00911885	1.35E-207	0.037093	0.0403211	0.3576031	0.5181322	0.0378644	20	FALSE	Vosa et al., 2018	Blood
PSEN1	ENSG00000080815	14	73646762	rs362429	14	73572825	A	G	0.178322	-0.0137	0.0123	0.2653559	0.366073	0.0130331	1.37E-173	-0.0374242	0.0336263	0.2657314	0.2196905	0.3788381	20	FALSE	Vosa et al., 2018	Blood
NDUFA9	ENSG00000139180	12	4756282	rs2270134	12	4758788	G	A	0.533217	0.0004	0.0101	0.9684089	-0.108187	0.00908205	1.02E-32	-0.0036973	0.0933572	0.968409	0.09044394	0.01583364	20	FALSE	Vosa et al., 2018	Blood
NDUFA7	ENSG00000267855	19	8379885	rs561	19	8376431	A	G	0.183566	-0.0189	0.0127	0.1367011	-0.317211	0.0117261	3.64E-161	0.0595818	0.040097	0.1372944	0.2040446	0.00830077	20	FALSE	Vosa et al., 2018	Blood
NDUFA8	ENSG00000119421	9	124908421	rs77945901	9	124908349	T	C	0.0524476	0.031	0.0224	0.1663803	0.263131	0.0137322	7.75E-82	0.117812	0.0853506	0.1674841	0.8380612	0.4408319	16	FALSE	Vosa et al., 2018	Blood
UQCR11	ENSG00000127540	19	1601324	rs8104174	19	1584309	A	G	0.16958	0.0219	0.0127	0.08463395	-0.169346	0.0140301	1.52E-33	-0.129321	0.0757559	0.08780786	0.0651304	0.2827804	10	FALSE	Vosa et al., 2018	Blood
NDUFA6	ENSG00000184983	22	42484244	rs4822094	22	42551839	T	C	0.041958	0.0034	0.0271	0.9001584	0.89547	0.0192921	0	0.00379689	0.0302635	0.9001588	0.002476211	0.05729889	20	FALSE	Vosa et al., 2018	Blood
NDUFV3	ENSG00000160194	21	44314768	rs4148974	21	44323720	T	C	0.0646853	0.035	0.0194	0.07121192	-0.873303	0.0179357	0	-0.0400777	0.0222297	0.07140603	0.01702241	0.1450178	20	FALSE	Vosa et al., 2018	Blood
IL1B	ENSG00000125538	2	113590904	rs6542082	2	113464709	G	A	0.512238	0.0032	0.0098	0.7440229	-0.0692158	0.00897508	1.24E-14	-0.0462322	0.141713	0.7442441	0.9076418	0.7580593	8	FALSE	Vosa et al., 2018	Blood
SNCA	ENSG00000145335	4	90702358	rs2736994	4	90784528	A	G	0.190559	-0.1709	0.0119	9.05E-47	0.260726	0.0109892	1.96E-124	-0.655478	0.0533522	1.08E-34	1.24E-27	3.95E-36	20	TRUE	Vosa et al., 2018	Blood
MAPT	ENSG00000186868	17	44038724	rs62074125	17	44852612	C	A	0.225524	0.0962	0.0129	8.83E-14	-0.0984498	0.0146407	1.76E-11	-0.977148	0.195666	5.92E-07	1.03E-07	4.34E-10	6	TRUE	Vosa et al., 2018	Blood
NDUFB10	ENSG00000140990	16	2010742	rs30252	16	2005982	C	T	0.323427	0.0073	0.0115	0.5255702	-0.183862	0.00901876	2.20E-92	-0.0397037	0.0625773	0.5257708	0.000165574	0.00281613	20	TRUE	Vosa et al., 2018	Blood
NDUFS8	ENSG00000110717	11	67801097	rs3133268	11	67796801	C	G	0.225524	-0.0038	0.0112	0.7343945	0.409646	0.0103163	0	-0.0092763	0.0273417	0.7344038	0.07356383	0.8458248	20	FALSE	Vosa et al., 2018	Blood
NDUFC1	ENSG00000109390	4	140205869	rs3806766	4	140222371	A	G	0.208042	0.0223	0.0128	0.08147564	0.273843	0.0143551	3.97E-81	0.0814335	0.0469366	0.08274662	0.03689011	0.4499327	20	FALSE	Vosa et al., 2018	Blood
NDUFC2	ENSG00000151366	11	77785307	rs657277	11	77774736	T	C	0.171329	0.0028	0.0131	0.8307495	-0.242332	0.0120799	1.62E-89	-0.0115544	0.0540611	0.8307589	0.06485845	0.6607323	20	FALSE	Vosa et al., 2018	Blood
NDUFS1	ENSG00000023228	2	207005238	rs12694040	2	206982546	T	C	0.466783	0.0185	0.0094	0.04905825	-0.0654283	0.00895346	2.72E-13	-0.282752	0.148788	0.05738441	0.1115688	0.00078886	18	FALSE	Vosa et al., 2018	Blood
NDUFV2	ENSG00000178127	18	9118485	rs11660603	18	9129858	G	A	0.174825	-0.0051	0.0127	0.687997	0.0977436	0.0100102	1.60E-22	-0.0521773	0.130042	0.6882467	0.9846781	0.9320564	20	FALSE	Vosa et al., 2018	Blood
NDUFB6	ENSG00000165264	9	32563078	rs10758142	9	32534499	C	T	0.526224	0.0136	0.0096	0.1565804	-0.0881491	0.00907949	2.77E-22	-0.154284	0.11006	0.1609686	0.3438903	0.09120268	19	FALSE	Vosa et al., 2018	Blood
NDUFS4	ENSG00000164258	5	52917815	rs3958630	5	52853386	A	G	0.148601	0.0028	0.0138	0.8392143	0.171868	0.0126662	6.12E-42	0.0162916	0.0803033	0.8392321	0.6066075	0.6378976	20	FALSE	Vosa et al., 2018	Blood
NDUFB7	ENSG00000099795	19	14679882	rs4926130	19	14672165	C	T	0.536713	0.0131	0.0105	0.2121706	-0.0590463	0.00899657	5.27E-11	-0.22186	0.181011	0.2203227	0.4011834	0.8427522	5	FALSE	Vosa et al., 2018	Blood
NDUFB8	ENSG00000166136	10	102278480	rs11190588	10	102273565	T	C	0.201049	-0.013	0.0113	0.2499617	-0.0911699	0.0122774	1.12E-13	0.142591	0.125423	0.2555885	0.2015305	0.5032616	10	FALSE	Vosa et al., 2018	Blood
NDUFS6	ENSG00000145494	5	1809116	rs4975847	5	1796813	C	T	0.606643	-0.0007	0.0105	0.9468471	0.0550085	0.00909803	1.48E-09	-0.0127253	0.190891	0.9468503	0.9468503	0.5371833	3	FALSE	Vosa et al., 2018	Blood
NDUFB9	ENSG00000147684	8	125566047	rs3829037	8	125565328	C	T	0.222028	0.0288	0.0113	0.01081338	0.199956	0.011437	1.92E-68	0.144032	0.0571097	0.01166846	0.005246763	0.1412939	20	FALSE	Vosa et al., 2018	Blood
NDUFS5	ENSG00000168653	1	39456149	rs34848020	1	39453785	G	A	0.536713	0.0106	0.0105	0.3127235	0.58648	0.00827349	0	0.0180739	0.0179052	0.3127726	0.3281497	0.6984984	20	FALSE	Vosa et al., 2018	Blood
NDUFS2	ENSG00000158864	1	161175539	rs4379692	1	161186313	T	C	0.340909	-0.0061	0.0102	0.5498138	-0.230919	0.00944221	4.34E-132	0.0264162	0.0441846	0.549933	0.02528292	0.00064585	20	FALSE	Vosa et al., 2018	Blood
PPP3CA	ENSG00000138814	4	102107000	rs2044044	4	102333865	A	G	0.276224	0.0031	0.0105	0.767812	-0.143232	0.00937136	9.78E-53	-0.0216432	0.0733214	0.7678541	0.1121319	0.2082424	20	FALSE	Vosa et al., 2018	Blood
APP	ENSG00000142192	21	27398153	rs2830076	21	27502468	T	C	0.368881	0.0235	0.0101	0.0199795	0.215839	0.00885412	2.99E-131	0.108878	0.0470069	0.02054702	0.1651418	0.7189337	20	FALSE	Vosa et al., 2018	Blood
NDUFB5	ENSG00000136521	3	179333956	rs4147789	3	179331272	T	C	0.442308	0.0026	0.0102	0.7987988	0.167942	0.00888439	1.08E-79	0.0154816	0.0607409	0.7988167	0.2770923	0.5080435	20	FALSE	Vosa et al., 2018	Blood
FAS	ENSG00000026103	10	90762978	rs4064	10	90751380	C	G	0.325175	-0.0036	0.01	0.7188471	-0.463245	0.0101268	0	0.00777126	0.0215875	0.7188555	0.4194036	0.4633946	20	FALSE	Vosa et al., 2018	Blood
CYC1	ENSG00000179091	8	145151179	rs12549149	8	145158446	C	T	0.0944056	-0.0122	0.0175	0.4857134	-0.122721	0.0140742	2.79E-18	0.0994122	0.143054	0.4871021	0.3417739	0.03997643	14	FALSE	Vosa et al., 2018	Blood
CHP1	ENSG00000187446	15	41548540	rs72739605	15	41556395	A	G	0.0926573	-0.0558	0.0164	0.00066787	-0.475697	0.0225421	7.53E-99	0.117301	0.0349209	0.00078209	0.01448746	0.04081194	20	FALSE	Vosa et al., 2018	Blood
CALML6	ENSG00000169885	1	1847500	rs2377037	1	1838516	A	C	0.243007	-0.002	0.0115	0.8619338	0.145339	0.0115941	4.77E-36	-0.0137609	0.0791328	0.861947	0.8557209	0.7663732	20	FALSE	Vosa et al., 2018	Blood

AMYLOID FIBER FORMATION (REACTOME)

Gene	probeID	ProbeChr	Probe bp	topSNP	topSNP chr	topSNP bp	A1	A2	Freq	b GWAS	se GWAS	p GWAS	b eQTL	se eQTL	p eQTL	b SMR	se SMR	p SMR	p SMR multi	p HEIDI	nsnp HEIDI	PASS BONF	Data source	Analyte
LYZ	ENSG00000090382	12	69745067	rs4761234	12	69732105	C	T	0.482517	0.004	0.0097	0.6800674	-0.403948	0.0507739	1.78E-15	-0.0099023	0.0240452	0.6804726	0.7366201	0.05814161	20	FALSE	Qi et al., 2018	Brain
TSPAN15	ENSG00000099282	10	71239327	rs1238357	10	71268289	C	T	0.526224	0.023	0.01	0.02144822	-0.5736	0.0451957	6.59E-37	-0.0400977	0.0177177	0.02362724	0.06369312	0.2977924	20	FALSE	Qi et al., 2018	Brain
CST3	ENSG00000101439	20	23613822	rs2104006	20	23607874	G	T	0.634615	0.0003	0.0097	0.9753271	0.248254	0.0388878	1.73E-10	0.00120844	0.0390734	0.9753274	0.2336074	0.3146892	10	FALSE	Qi et al., 2018	Brain
TSPAN14	ENSG00000108219	10	82248154	rs2168721	10	82208192	G	A	0.613636	-0.0023	0.0104	0.8249726	-0.250018	0.0446552	2.16E-08	0.00919932	0.0416294	0.8251068	0.938868	0.289312	20	FALSE	Qi et al., 2018	Brain
ADAM10	ENSG00000137845	15	58964790	rs12592302	15	58912586	A	T	0.903846	0.016	0.0157	0.3081516	0.341262	0.0567053	1.76E-09	0.0468849	0.0466607	0.3149915	0.3149915	0.2585262	20	FALSE	Qi et al., 2018	Brain
MFGE8	ENSG00000140545	15	89449279	rs34880771	15	89460632	T	C	0.648601	0.0195	0.0108	0.07098779	-0.333528	0.0412113	5.82E-16	-0.0584658	0.0331771	0.07802998	0.02117331	0.4581573	20	FALSE	Qi et al., 2018	Brain
FURIN	ENSG00000140564	15	91419255	rs17514846	15	91416550	C	A	0.5	0.0153	0.0115	0.1833751	-0.240801	0.0382393	3.03E-10	-0.0635378	0.0488114	0.1930189	0.3033109	0.7300169	9	FALSE	Qi et al., 2018	Brain
HSPG2	ENSG00000142798	1	22206264	rs10799719	1	22248881	G	A	0.333916	0.0119	0.01	0.2340464	0.30663	0.0414125	1.32E-13	0.038809	0.0330311	0.2400257	0.1808285	0.4644011	20	FALSE	Qi et al., 2018	Brain
NAT8	ENSG00000144035	2	73868739	rs9753079	2	73733313	C	T	0.762238	0.0017	0.0111	0.8782775	-0.447134	0.0773293	7.37E-09	-0.003802	0.0248335	0.8783198	0.9591336	0.8696992	20	FALSE	Qi et al., 2018	Brain
GSN	ENSG00000148180	9	124032598	rs76098787	9	124047836	C	T	0.0559441	-0.0418	0.0238	0.07903677	0.682433	0.10975	5.03E-10	-0.0612514	0.0362397	0.09099427	0.06435921	0.08804399	20	FALSE	Qi et al., 2018	Brain
HIST1H2BD	ENSG00000158373	6	26164963	rs9379831	6	26175852	C	A	0.68007	-0.0001	0.0101	0.9921003	0.282419	0.0493856	1.07E-08	-0.0003541	0.0357625	0.9921003	0.9921003	0.01609664	8	FALSE	Qi et al., 2018	Brain

H3F3A	ENSG00000163041	1	226254627	rs6678547	1	226258009	C	T	0.746503	-0.0143	0.0114	0.2097018	-1.15658	0.0311417	6.53E-302	0.012364	0.00986226	0.2099616	0.04395228	0.4220858	20	FALSE	Qi et al., 2018	Brain
HIST1H4C	ENSG00000197061	6	26104311	rs198847	6	26106619	C	A	0.589161	0.0215	0.012	0.07318638	0.317145	0.034302	2.34E-20	0.0677923	0.0385415	0.0785869	0.01584491	0.3221149	20	FALSE	Qi et al., 2018	Brain
UBA52	ENSG00000221983	19	18685450	rs10409392	19	18699333	A	C	0.458042	0.002	0.0097	0.836646	0.322222	0.0488456	4.20E-11	0.0062069	0.0301182	0.8367246	0.9599301	0.3329377	20	FALSE	Qi et al., 2018	Brain
HIST1H4F	ENSG00000198327	6	26240768	rs11759720	6	26250254	C	G	0.802448	0.0184	0.0124	0.1378432	-0.645065	0.081341	2.18E-15	-0.0285243	0.0195565	0.1446868	0.08360257	0.146912	20	FALSE	Qi et al., 2018	Brain
HIST1H3E	ENSG00000196966	6	26225613	rs11757549	6	26231608	G	A	0.907343	0.02	0.0168	0.2338593	-0.860175	0.125459	7.07E-12	-0.0232511	0.0198231	0.2408254	0.2408254	0.1721667	15	FALSE	Qi et al., 2018	Brain
LTF	ENSG00000012223.8	3	46501930	rs10865941	3	46508547	C	T	0.398601	0.0198	0.0097	0.04122726	0.765707	0.132707	7.93E-09	0.0258585	0.0134374	0.05430896	0.05430896	0.05063085	3	FALSE	GTEx Consortium	Substantia Nigra
HIST1H3E	ENSG00000196966.3	6	26225613	rs74928264	6	26224343	G	C	0.0926573	-0.0199	0.0168	0.2362058	1.22093	0.197454	6.28E-10	-0.0162991	0.0140102	0.2446796	0.2446796	0.4080319	6	FALSE	GTEx Consortium	Substantia Nigra
LTF	ENSG00000012223	3	46501930	rs181225830	3	46369762	A	G	0.027972	-0.0639	0.0533	0.2305768	-0.306387	0.0411877	1.02E-13	0.20856	0.176208	0.2365709	0.1905465	0.3490585	3	FALSE	Vosa et al., 2018	Blood
LYZ	ENSG00000090382	12	69745067	rs7959452	12	69735492	A	G	0.479021	-0.0019	0.0096	0.8431103	0.913524	0.00683088	0	-0.0020799	0.0105088	0.8431104	0.1181828	0.1481425	20	FALSE	Vosa et al., 2018	Blood
TSPAN15	ENSG00000099282	10	71239327	rs17569412	10	71237536	A	C	0.237762	-0.0224	0.012	0.06194815	0.29539	0.0130654	3.57E-113	-0.0758319	0.0407625	0.06283792	0.09597795	0.1024411	20	FALSE	Vosa et al., 2018	Blood
GGA1	ENSG00000100083	22	38017026	rs12710562	22	38029723	G	A	0.197552	0.0383	0.0134	0.0042604	0.239356	0.00966792	2.56E-135	0.160012	0.0563553	0.00452055	0.008359469	0.00678893	20	FALSE	Vosa et al., 2018	Blood
CST3	ENSG00000101439	20	23613822	rs13039536	20	23612305	G	A	0.223776	-0.014	0.0115	0.2234554	-0.709542	0.0105134	0	0.0197311	0.0162103	0.2235307	0.3848932	0.2279816	20	FALSE	Vosa et al., 2018	Blood
GGA2	ENSG00000103365	16	23504089	rs4968016	16	23530089	T	C	0.152098	0.0182	0.0127	0.1518376	-0.298821	0.0107253	7.86E-171	-0.0609061	0.0425566	0.1523789	0.432074	0.962314	20	FALSE	Vosa et al., 2018	Blood
CALB1	ENSG00000104327	8	91089269	rs2178830	8	91162808	T	C	0.706294	0.0018	0.0104	0.862591	-0.0918753	0.00893363	8.30E-25	-0.0195918	0.113213	0.8626102	0.7879029	0.6901929	20	FALSE	Vosa et al., 2018	Blood
TSPAN14	ENSG00000108219	10	82248154	rs12260962	10	82262826	T	C	0.291958	-0.007	0.0111	0.5282821	0.270495	0.0109239	2.31E-135	-0.0258785	0.0410492	0.5284158	0.7942235	0.6190725	20	FALSE	Vosa et al., 2018	Blood
APH1A	ENSG00000117362	1	150239892	rs113007736	1	150295738	G	T	0.141608	-0.0439	0.015	0.00342616	0.132954	0.0183623	4.47E-13	-0.330189	0.121689	0.00665982	0.02265119	0.0876561	20	FALSE	Vosa et al., 2018	Blood
TGFBI	ENSG00000120708	5	135382045	rs10074474	5	135334847	A	G	0.15035	0.0195	0.0142	0.1696779	-0.668693	0.0119533	0	-0.0291614	0.0212418	0.1698065	0.484039	0.136858	15	FALSE	Vosa et al., 2018	Blood
SEMG1	ENSG00000124233	20	43837025	rs2233901	20	43851094	A	G	0.160839	-0.0195	0.0121	0.1070555	-0.394451	0.0137738	2.28E-180	0.0494358	0.0307241	0.107611	0.07308835	0.1397268	20	FALSE	Vosa et al., 2018	Blood
HIST1H2BJ	ENSG00000124635	6	27097299	rs12195360	6	27210032	T	C	0.0821678	0.047	0.0163	0.00393363	0.146672	0.0131852	9.59E-29	0.320444	0.114805	0.00525145	5.64E-05	0.1753802	20	TRUE	Vosa et al., 2018	Blood
GGA3	ENSG00000125447	17	73245569	rs8079345	17	73267461	C	G	0.111888	0.0038	0.0144	0.7918655	-0.0827623	0.0105867	5.38E-15	-0.0459146	0.174091	0.7919813	0.358358	0.8845328	13	FALSE	Vosa et al., 2018	Blood
H3F3B	ENSG00000132475	17	73777244	rs17581498	17	73794047	G	T	0.167832	0.0229	0.0138	0.09703113	-0.141386	0.0143772	8.04E-23	-0.161968	0.0989852	0.1017794	0.480104	0.4895962	20	FALSE	Vosa et al., 2018	Blood
ITM2B	ENSG00000136156	13	48822178	rs3803188	13	48808132	T	C	0.241259	0.0088	0.012	0.4633551	0.276717	0.0112642	2.92E-133	0.0318014	0.0433849	0.4635543	0.7282804	0.2299794	20	FALSE	Vosa et al., 2018	Blood
ADAM10	ENSG00000137845	15	58964790	rs7161799	15	58770523	T	C	0.0891608	-0.0223	0.019	0.2405215	0.497122	0.0135183	4.79E-296	-0.0448582	0.0382395	0.240761	0.1511186	0.9392393	20	FALSE	Vosa et al., 2018	Blood
APH1B	ENSG00000138613	15	63584771	rs111564920	15	63441242	A	G	0.115385	0.0379	0.0235	0.1067954	0.540024	0.019021	2.61E-177	0.0701821	0.0435867	0.1073607	0.000336129	0.01788987	20	TRUE	Vosa et al., 2018	Blood
MFGE8	ENSG00000140545	15	89449279	rs12911703	15	89456948	A	G	0.297203	-0.0259	0.0114	0.02309075	0.620458	0.0106803	0	-0.0417434	0.0183876	0.02319579	0.03790007	0.746285	20	FALSE	Vosa et al., 2018	Blood
FURIN	ENSG00000140564	15	91419255	rs372098	15	91365165	T	C	0.384615	-0.0111	0.0103	0.2811811	-0.210317	0.00950491	1.73E-108	0.0527775	0.0490317	0.2817511	0.473274	0.07358751	20	FALSE	Vosa et al., 2018	Blood
APP	ENSG00000142192	21	27398153	rs2830076	21	27502468	T	C	0.368881	0.0235	0.0101	0.0199795	0.215839	0.00885412	2.99E-131	0.108878	0.0470069	0.02054702	0.1651418	0.7189337	20	FALSE	Vosa et al., 2018	Blood
HSPG2	ENSG00000142798	1	22206264	rs11587857	1	22260308	C	T	0.229021	0.0207	0.0115	0.07186064	0.217397	0.00926742	1.09E-121	0.0952175	0.0530541	0.07269765	0.06180838	0.571467	20	FALSE	Vosa et al., 2018	Blood
SNCA	ENSG00000145335	4	90702358	rs2736994	4	90784528	A	G	0.190559	-0.1709	0.0119	9.05E-47	0.260726	0.0109892	1.96E-124	-0.655478	0.0533522	1.08E-34	1.24E-27	3.95E-36	20	TRUE	Vosa et al., 2018	Blood
GSN	ENSG00000148180	9	124032599	rs10760167	9	124035545	A	G	0.548951	-0.0343	0.0099	0.00053093	-0.662881	0.00800026	0	0.0517438	0.0149479	0.00053693	0.008048225	0.08945405	20	FALSE	Vosa et al., 2018	Blood
UBC	ENSG00000150991	12	125399032	rs4617684	12	125406966	A	G	0.00174825	0.072	0.1032	0.4853808	-0.0842798	0.011017	2.01E-14	-0.854298	1.22958	0.4871863	0.7652337			FALSE	Vosa et al., 2018	Blood
UBE2L6	ENSG00000156587	11	57327443	rs2234412	11	57320044	T	C	0.43007	-0.015	0.0095	0.1143481	0.160474	0.00906286	3.72E-70	-0.0934729	0.0594344	0.1157867	0.09982762	0.04486782	20	FALSE	Vosa et al., 2018	Blood
HIST1H2BD	ENSG00000158373	6	26164963	rs1009181	6	26158993	C	T	0.361888	0.0193	0.0098	0.04890858	-0.808108	0.00823209	0	-0.0238829	0.0121295	0.04895406	9.69E-05	0.03177635	20	TRUE	Vosa et al., 2018	Blood
HIST1H4H	ENSG00000158406	6	26283522	rs2393592	6	26285560	A	G	0.477273	-0.0122	0.0096	0.2037879	0.402229	0.00886424	0	-0.030331	0.0238763	0.2039652	9.69E-05	0.02197982	20	TRUE	Vosa et al., 2018	Blood
TSPAN33	ENSG00000158457	7	128796691	rs12667978	7	128777461	T	C	0.554196	0.0085	0.0142	0.5494453	-0.347932	0.00876371	0	-0.02443	0.0408172	0.5494907	0.5151769	0.2274419	20	FALSE	Vosa et al., 2018	Blood
NCSTN	ENSG00000162736	1	160320902	rs2038780	1	160317619	C	T	0.47028	0.0151	0.0101	0.1349015	0.538963	0.00838431	0	0.0280168	0.0187447	0.135007	0.7362723	0.8848456	20	FALSE	Vosa et al., 2018	Blood
H3F3A	ENSG00000163041	1	226254627	rs6678547	1	226258009	T	C	0.253497	0.0143	0.0114	0.2097018	0.362642	0.00911925	0	0.0394328	0.0314516	0.2099284	0.01405624	0.4660388	20	FALSE	Vosa et al., 2018	Blood
H2AFZ	ENSG00000164032	4	100870394	rs2276939	4	100870375	G	T	0.248252	-0.0175	0.012	0.1447487	-0.24915	0.0102198	2.84E-131	0.0702387	0.0482498	0.145467	0.2421593	0.9207658	20	FALSE	Vosa et al., 2018	Blood
B2M	ENSG00000166710	15	45007375	rs1690313	15	45005660	G	C	0.0437063	0.0176	0.024	0.4633551	-0.165611	0.0154005	5.70E-27	-0.106273	0.145255	0.4643919	0.6242994	0.7614216	6	FALSE	Vosa et al., 2018	Blood
TSPAN5	ENSG00000168785	4	99485649	rs13111424	4	99579160	A	C	0.0402098	-0.0047	0.0269	0.8612987	-0.327808	0.0332874	7.01E-23	0.0143377	0.0820732	0.8613203	0.6829546	0.7978827	5	FALSE	Vosa et al., 2018	Blood
NPPA	ENSG00000175206	1	11907084	rs198411	1	11900385	T	C	0.117133	-0.0241	0.0135	0.07423128	0.286078	0.0134876	7.66E-100	-0.0842428	0.0473568	0.07525688	0.1077089	0.04729562	20	FALSE	Vosa et al., 2018	Blood
HIST1H2AC	ENSG00000180573	6	26131858	rs112544908	6	25766673	C	T	0.0157343	-0.0681	0.0396	0.08548754	-0.380035	0.0419715	1.37E-19	0.179194	0.106064	0.09112451	0.01880431	0.7564943	14	FALSE	Vosa et al., 2018	Blood
HIST1H2BC	ENSG00000180596	6	26119627	rs62396160	6	26075393	C	T	0.0944056	-0.0252	0.0139	0.06983959	0.0980445	0.0124716	3.80E-15	-0.257026	0.145493	0.07729822	0.01172856	0.00318936	18	FALSE	Vosa et al., 2018	Blood
SIAH2	ENSG00000181788	3	150470089	rs524772	3	150447679	G	A	0.0227273	-0.0062	0.0333	0.8522988	-0.707829	0.0435807	2.55E-59	0.00875918	0.0470483	0.8523083	0.06147016	0.02615532	15	FALSE	Vosa et al., 2018	Blood
HIST2H2BE	ENSG00000184678	1	149857121	rs72708144	1	149815740	C	T	0.0402098	-0.0771	0.0313	0.01376805	-0.312251	0.0292672	1.42E-26	0.246916	0.102877	0.01638972	0.0279471	0.1781439	7	FALSE	Vosa et al., 2018	Blood
BACE1	ENSG00000186318	11	117171688	rs477036	11	117183914	T	C	0.538462	-0.0245	0.0098	0.01241933	-0.224934	0.00891019	1.30E-140	0.108921	0.0437814	0.0128525	0.01696624	0.2267902	20	FALSE	Vosa et al., 2018	Blood
SIAH1	ENSG00000196470	16	48436294	rs8053839	16	48390512	T	G	0.533217	-0.0029	0.0102	0.77617	0.107348	0.0100651	1.48E-26	-0.0270149	0.0950516	0.7762473	0.4427574	0.1475888	15	FALSE	Vosa et al., 2018	Blood
HIST1H3D	ENSG00000197409	6	26198294	rs112751982	6	26191958	T	C	0.157343	-0.0404	0.0147	0.00599053	-0.0815559	0.0126033	9.74E-11	0.495365	0.195827	0.01141898	0.028221	0.02983773	9	FALSE	Vosa et al., 2018	Blood
HIST1H2BK	ENSG00000197903	6	27110346	rs12192049	6	27056739	C	T	0.0804196	0.0489	0.0164	0.00286646	0.712042	0.0176943	0	0.0686757	0.0230955	0.00294371	7.42E-09	0.00694096	20	TRUE	Vosa et al., 2018	Blood
UBA52	ENSG00000221983	19	18685450	rs12608925	19	18687533	T	G	0.557692	-0.0014	0.0095	0.8828412	0.275529	0.00877334	1.71E-216	-0.0050811	0.0344795	0.8828425	0.2893193	0.2874441	20	FALSE	Vosa et al., 2018	Blood
HIST1H2BN	ENSG00000233822	6	27813148	rs111718978	6	27162514	G	A	0.0227273	-0.1158	0.0501	0.02081203	-0.318543	0.0508464	3.73E-10	0.36353	0.167642	0.03012105	0.04014739	0.2021641	5	FALSE	Vosa et al., 2018	Blood

APOPTOTIC CLEAVAGE OF CELLULAR PROTEINS (REACTOME)

Gene	probeID	ProbeChr	Probe bp	topSNP	topSNP chr	topSNP bp	A1	A2	Freq	b GWAS	se GWAS	p GWAS	b eQTL	se eQTL	p eQTL	b SMR	se SMR	p SMR	p SMR multi	p HEIDI	nsnp HEIDI	PASS BONF	Data source	Analyte
VIM	ENSG00000026025	10	17274925	rs7898271	10	17052418	T	C	0.568182	0.0067	0.0099	0.4985534	-0.301537	0.0467134	1.08E-10	-0.0222195	0.0330118	0.5008974	0.3971622	0.3634895	20	FALSE	Qi et al., 2018	Brain
CDH1	ENSG00000039068	16	68820286	rs9927407	16	68744532	A	G	0.701049	-0.0046	0.0104	0.6582666	-0.475348	0.0490283	3.15E-22	0.00967713	0.0219015	0.6585991	0.9940708	0.9150806	20	FALSE	Qi et al., 2018	Brain
CASP8	ENSG00000064012	2	202125300	rs10200279	2	202170655	T	C	0.286713	0.0319	0.0128	0.0126959	0.658191	0.089728	2.21E-13	0.0484662	0.020539	0.01828891	0.08317744	0.0531694	18	FALSE	Qi et al., 2018	Brain
PKP1	ENSG00000081277	1	201277350	rs56287879	1	201237953	C	A	0.895105	-0.0118	0.0153	0.4405636	0.465412	0.0632948	1.94E-13	-0.0253539	0.0330544	0.4430612	0.7083541	0.8015372	15	FALSE	Qi et al., 2018	Brain
DBNL	ENSG00000136279	7	44096643	rs2595699	7	44005513	G	A	0.944056	-0.0557	0.0257	0.03021083	1.12128	0.116832	8.20E-22	-0.0496755	0.0234974	0.03450841	0.02629425	0.02548071	12	FALSE	Qi et al., 2018	Brain
CASP6	ENSG00000138794	4	110617262	rs10004377	4	110648124	T	G	0.596154	-0.0036	0.0098	0.7133602	0.42605	0.0460858	2.36E-20	-0.0084497	0.0230201	0.7135763	0.9725797	0.9904615	20	FALSE	Qi et al., 2018	Brain
GSN	ENSG00000148180	9	124032598	rs76098787	9	124047836	C	T	0.0559441	-0.0418	0.0238	0.07903677	0.682433	0.10975	5.03E-10	-0.0612514	0.0362397	0.09099427	0.06435921	0.08804399	20	FALSE	Qi et al., 2018	Brain
GAS2	ENSG00000148935	11	22740894	rs337498	11	22688053	T	C	0.833916	0.0061	0.0149	0.6822491	-0.351179	0.0520057	1.45E-11	-0.0173701	0.0425065	0.6827997	0.8117148	0.576869	10	FALSE	Qi et al., 2018	Brain
PRKCD	ENSG00000163932	3	53208379	rs13319192	3	53193548	G	T	0.97028	-0.0159	0.0295	0.5898985	0.572714	0.0816018	2.24E-12	-0.0277625	0.0516608	0.5909909	0.03512855	0.1877964	18	FALSE	Qi et al., 2018	Brain
CASP7	ENSG00000165806	10	115464802	rs3121487	10	115408679	T	G	0.61014	-0.0145	0.0096	0.1309371	-1.02262	0.0283134	1.18E-285	0.0141792	0.00939581	0.1312738	0.407857	0.8460837	20	FALSE	Qi et al., 2018	Brain
PTK2	ENSG00000169398	8	141840157	rs7815898	8	141876541	G	C	0.396853	0.0358	0.0098	0.00025913	-0.366815	0.0467238	4.14E-15	-0.0975969	0.0294672	0.00092613	0.001104484	0.9644551	20	TRUE	Qi et al., 2018	Brain
PLEC	ENSG00000178209	8	145020111	rs11136341	8	145043543	A	G	0.606643	-0.021	0.0099	0.03390396	1.14122	0.0309453	1.01E-297	-0.0184013	0.00868923	0.03419947	0.02894908	0.07013014	20	FALSE	Qi et al., 2018	Brain
SATB1	ENSG00000182568	3	18436972	rs73040357	3	18482190	C	A	0.818182	-0.0229	0.0141	0.1043516	-0.318744	0.0559465	1.22E-08	0.0718445	0.0459984	0.1183137	0.1183137	0.2059623	13	FALSE	Qi et al., 2018	Brain
MAPT	ENSG00000186868	17	44038724	rs199502	17	44862347	G	A	0.743007	0.1949	0.0156	8.09E-36	0.734607	0.0582562	1.86E-36	0.265312	0.0298938	6.98E-19	3.51E-24	0.01172117	17	TRUE	Qi et al., 2018	Brain
OCLN	ENSG00000197822	5	68821025	rs10045526	5	68592751	G	A	0.41958	0.0027	0.0098	0.7829243	-0.315839	0.0422943	8.16E-14	-0.0085487	0.0310496	0.7830682	0.3217747	0.01404157	20	FALSE	Qi et al., 2018	Brain
VIM	ENSG00000026025	10	17274925	rs7083383	10	17260573	C	G	0.222028	0.0025	0.0143	0.861217	-0.226594	0.0138761	6.06E-60	-0.0110329	0.063112	0.8612249	0.2727151	0.09055329	20	FALSE	Vosa et al., 2018	Blood
CDH1	ENSG00000039068	16	68820286	rs11642362	16	68811307	T	C	0.433566	-0.0008	0.0096	0.9335865	-0.315847	0.00872182	3.75E-287	0.00253288	0.0303946	0.9335867	0.9795313	0.8539883	20	FALSE	Vosa et al., 2018	Blood
CASP8	ENSG00000064012	2	202125300	rs7560328	2	202164837	A	C	0.398601	0.0194	0.0096	0.04329702	-0.214284	0.00890084	4.61E-128	-0.090534	0.0449579	0.04403565	0.4704423	0.714529	20	FALSE	Vosa et al., 2018	Blood
PRKCQ	ENSG00000065675	10	6545684	rs2255088	10	6625378	C	T	0.321678	-0.0033	0.0109	0.7620786	-0.171391	0.00904469	4.47E-80	0.0192542	0.0636052	0.7621081	0.6947417	0.4356083	15	FALSE	Vosa et al., 2018	Blood
ROCK1	ENSG00000067900	18	18610756	rs142716063	18	18952751	A	G	0.013986	-0.0528	0.0434	0.2237603	0.236878	0.0325293	3.29E-13	-0.222899	0.185756	0.2301554	0.1230874	0.131832	10	FALSE	Vosa et al., 2018	Blood
ADD1	ENSG00000087274	4	2888693	rs6819310	4	2869127	C	T	0.293706	0.0511	0.0106	1.43E-06	0.23786	0.0120276	4.77E-87	0.214832	0.045869	2.82E-06	0.000887411	0.00734456	20	TRUE	Vosa et al., 2018	Blood
DSP	ENSG00000096696	6	7564379	rs11243180	6	7439070	G	A	0.438811	-0.0106	0.0101	0.2939458	-0.637654	0.00806203	0	0.0166234	0.0158407	0.2939883	0.7138452	0.5761607	20	FALSE	Vosa et al., 2018	Blood
ACIN1	ENSG00000100813	14	23546298	rs10149569	14	23563895	C	A	0.0821678	-0.0112	0.0163	0.4920093	-0.121407	0.0126066	5.95E-22	0.0922519	0.134601	0.4931078	0.3546254	0.6481822	9	FALSE	Vosa et al., 2018	Blood
STK24	ENSG00000102572	13	99166324	rs4772103	13	99234131	A	G	0.442308	0.0039	0.0096	0.6845589	0.172567	0.00891324	1.65E-83	0.0225999	0.0556427	0.6846246	0.2734283	0.2785907	20	FALSE	Vosa et al., 2018	Blood
BIRC2	ENSG00000110330	11	102233671	rs11827872	11	102262244	T	G	0.0751748	-0.0098	0.0242	0.6855079	-0.136661	0.0215847	2.43E-10	0.07171	0.177442	0.686115	0.8600748			FALSE	Vosa et al., 2018	Blood
LMNB1	ENSG00000113368	5	126142513	rs2271354	5	126145771	A	G	0.276224	0.0195	0.0111	0.07895928	0.181891	0.00977615	2.89E-77	0.107207	0.061297	0.08029487	0.2001857	0.459058	20	FALSE	Vosa et al., 2018	Blood
TJP2	ENSG00000119139	9	71803174	rs1416738	9	71787927	T	C	0.393357	0.024	0.0095	0.01152658	-0.679794	0.00784968	0	-0.0353048	0.0139808	0.01156188	0.07684917	0.7073372	20	FALSE	Vosa et al., 2018	Blood
APC	ENSG00000134982	5	112112565	rs1734242	5	112125419	G	A	0.127622	0.0295	0.0154	0.05541801	-0.0759217	0.0109139	3.49E-12	-0.388558	0.210391	0.06477005	0.03036535	0.03897929	19	FALSE	Vosa et al., 2018	Blood
DBNL	ENSG00000136279	7	44096647	rs2528374	7	43976548	G	C	0.0559441	0.0457	0.0219	0.03691002	-0.539491	0.015575	6.73E-263	-0.0847094	0.0406674	0.03725292	0.02617208	0.0015638	20	FALSE	Vosa et al., 2018	Blood
CASP6	ENSG00000138794	4	110617262	rs13144085	4	110628473	C	T	0.0559441	0.0039	0.0193	0.83986	0.155261	0.0216863	8.10E-13	0.025119	0.124356	0.8399229	0.9717244	0.8813971	12	FALSE	Vosa et al., 2018	Blood
GSN	ENSG00000148180	9	124032599	rs10760167	9	124035545	A	G	0.548951	-0.0343	0.0099	0.00053093	-0.662881	0.00800026	0	0.0517438	0.0149479	0.00053693	0.008048225	0.08945405	20	FALSE	Vosa et al., 2018	Blood
GAS2	ENSG00000148935	11	22740894	rs4447177	11	22646025	G	A	0.15035	0.0033	0.0133	0.8040413	0.15242	0.0101964	1.60E-50	0.0216506	0.0872706	0.8040678	0.9053025	0.4432954	20	FALSE	Vosa et al., 2018	Blood
LMNA	ENSG00000160789	1	156081122	rs609625	1	156089631	G	C	0.0716783	0.0589	0.0209	0.00482965	0.528427	0.0135931	0	0.111463	0.0396552	0.00494176	0.00533209	0.00039882	20	FALSE	Vosa et al., 2018	Blood
PRKCD	ENSG00000163932	3	53208379	rs2230494	3	53220215	A	G	0.288462	0.0465	0.0115	5.27E-05	0.212967	0.00980744	1.49E-104	0.218344	0.0549273	7.03E-05	0.000141071	0.08661588	20	TRUE	Vosa et al., 2018	Blood
CASP3	ENSG00000164305	4	185559756	rs72689261	4	185590897	A	C	0.111888	0.0045	0.0146	0.7579156	-0.56825	0.0151553	1.11E-307	-0.0079191	0.0256938	0.7579235	0.7005626	0.8843126	20	FALSE	Vosa et al., 2018	Blood
CASP7	ENSG00000165806	10	115464802	rs4353229	10	115489589	C	T	0.234266	-0.0125	0.0107	0.2427163	-0.66876	0.00909944	0	0.0186913	0.0160018	0.2427758	0.6036084	0.7677355	20	FALSE	Vosa et al., 2018	Blood

CTNNB1	ENSG00000168036	3	41268957	rs1722846	3	41233889	T	C	0.45979	0.0301	0.0101	0.00288062	-0.250356	0.00906553	7.09E-168	-0.120229	0.0405767	0.00304661	0.1741666	0.6460329	20	FALSE	Vosa et al., 2018	Blood
FNTA	ENSG00000168522	8	42915134	rs9657192	8	43023447	T	A	0.113636	0.0235	0.0154	0.1270163	-0.0569757	0.0102904	3.08E-08	-0.412457	0.280368	0.1412575	0.171546	0.2926599	20	FALSE	Vosa et al., 2018	Blood
PTK2	ENSG00000169398	8	141840157	rs4961234	8	141840881	T	C	0.578671	-0.0343	0.0098	0.00046526	0.180841	0.00888451	4.23E-92	-0.18967	0.0549867	0.00056188	0.000525702	0.9990542	20	TRUE	Vosa et al., 2018	Blood
PLEC	ENSG00000178209	8	145020111	rs11781667	8	145040856	T	G	0.377622	0.0235	0.0099	0.01760907	-0.28405	0.0104024	3.58E-164	-0.082732	0.0349845	0.01803889	0.02280896	0.5832694	20	FALSE	Vosa et al., 2018	Blood
SATB1	ENSG00000182568	3	18436972	rs9851736	3	18430301	T	C	0.424825	0.0105	0.0102	0.3032862	0.175235	0.00956344	5.38E-75	0.0599196	0.0582994	0.3040481	0.002032091	0.00152056	20	FALSE	Vosa et al., 2018	Blood
MAPT	ENSG00000186868	17	44038724	rs62074125	17	44852612	C	A	0.225524	0.0962	0.0129	8.83E-14	-0.0984498	0.0146407	1.76E-11	-0.977148	0.195666	5.92E-07	1.03E-07	4.34E-10	6	TRUE	Vosa et al., 2018	Blood
SPTAN1	ENSG00000197694	9	131355403	rs7036322	9	131417140	A	C	0.129371	-0.0031	0.0159	0.8454176	-0.0555623	0.00930038	2.31E-09	0.0557932	0.286318	0.8454988	0.8778587	0.6220713	6	FALSE	Vosa et al., 2018	Blood
OCLN	ENSG00000197822	5	68821025	rs10042124	5	68658499	A	G	0.305944	-0.0342	0.0111	0.00206251	0.130808	0.00976395	6.30E-41	-0.261453	0.0870727	0.00267601	0.1041364	0.08489406	20	FALSE	Vosa et al., 2018	Blood

APOPTOTIC EXECUTION PHASE (REACTOME)

Gene	probeID	ProbeChr	Probe bp	topSNP	topSNP chr	topSNP bp	A1	A2	Freq	b GWAS	se GWAS	p GWAS	b eQTL	se eQTL	p eQTL	b SMR	se SMR	p SMR	p SMR multi	p HEIDI	nsnp HEIDI	PASS BONF	Data source	Analyte
VIM	ENSG00000026025	10	17274925	rs7898271	10	17052418	T	C	0.568182	0.0067	0.0099	0.4985534	-0.301537	0.0467134	1.08E-10	-0.0222195	0.0330118	0.5008974	0.3971622	0.3634895	20	FALSE	Qi et al., 2018	Brain
CDH1	ENSG00000039068	16	68820286	rs9927407	16	68744532	A	G	0.701049	-0.0046	0.0104	0.6582666	-0.475348	0.0490283	3.15E-22	0.00967713	0.0219015	0.6585991	0.9940708	0.9150806	20	FALSE	Qi et al., 2018	Brain
CASP8	ENSG00000064012	2	202125300	rs10200279	2	202170655	T	C	0.286713	0.0319	0.0128	0.0126959	0.658191	0.089728	2.21E-13	0.0484662	0.020539	0.01828891	0.08317744	0.0531694	18	FALSE	Qi et al., 2018	Brain
PKP1	ENSG00000081277	1	201277350	rs56287879	1	201237953	C	A	0.895105	-0.0118	0.0153	0.4405636	0.465412	0.0632948	1.94E-13	-0.0253539	0.0330544	0.4430612	0.7083541	0.8015372	15	FALSE	Qi et al., 2018	Brain
DBNL	ENSG00000136279	7	44096643	rs2595699	7	44005513	G	A	0.944056	-0.0557	0.0257	0.03021083	1.12128	0.116832	8.20E-22	-0.0496755	0.0234974	0.03450841	0.02629425	0.02548071	12	FALSE	Qi et al., 2018	Brain
CASP6	ENSG00000138794	4	110617262	rs10004377	4	110648124	T	G	0.596154	-0.0036	0.0098	0.7133602	0.42605	0.0460858	2.36E-20	-0.0084497	0.0230201	0.7135763	0.9725797	0.9904615	20	FALSE	Qi et al., 2018	Brain
GSN	ENSG00000148180	9	124032598	rs76098787	9	124047836	C	T	0.0559441	-0.0418	0.0238	0.07903677	0.682433	0.10975	5.03E-10	-0.0612514	0.0362397	0.09099427	0.06435921	0.08804399	20	FALSE	Qi et al., 2018	Brain
GAS2	ENSG00000148935	11	22740894	rs337498	11	22688053	T	C	0.833916	0.0061	0.0149	0.6822491	-0.351179	0.0520057	1.45E-11	-0.0173701	0.0425065	0.6827997	0.8117148	0.576869	10	FALSE	Qi et al., 2018	Brain
PRKCD	ENSG00000163932	3	53208379	rs13319192	3	53193548	G	T	0.97028	-0.0159	0.0295	0.5898985	0.572714	0.0816018	2.24E-12	-0.0277625	0.0516608	0.5909909	0.03512855	0.1877964	18	FALSE	Qi et al., 2018	Brain
CASP7	ENSG00000165806	10	115464802	rs3121487	10	115408679	T	G	0.61014	-0.0145	0.0096	0.1309371	-1.02262	0.0283134	1.18E-285	0.0141792	0.00939581	0.1312738	0.407857	0.8460837	20	FALSE	Qi et al., 2018	Brain
PTK2	ENSG00000169398	8	141840157	rs7815898	8	141876541	G	C	0.396853	0.0358	0.0098	0.00025913	-0.366815	0.0467238	4.14E-15	-0.0975969	0.0294672	0.00092613	0.001104484	0.9644551	20	TRUE	Qi et al., 2018	Brain
DFFB	ENSG00000169598	1	3787919	rs7551237	1	3801973	T	C	0.844406	0.0065	0.0142	0.6471346	0.901311	0.0664512	6.59E-42	0.00721171	0.0157638	0.6473217	0.613616	0.8248225	20	FALSE	Qi et al., 2018	Brain
PLEC	ENSG00000178209	8	145020111	rs11136341	8	145043543	A	G	0.606643	-0.021	0.0099	0.03390396	1.14122	0.0309453	1.01E-297	-0.0184013	0.00868923	0.03419947	0.02894908	0.07013014	20	FALSE	Qi et al., 2018	Brain
SATB1	ENSG00000182568	3	18436972	rs73040357	3	18482190	C	A	0.818182	-0.0229	0.0141	0.1043516	-0.318744	0.0559465	1.22E-08	0.0718445	0.0459984	0.1183137	0.1183137	0.2059623	13	FALSE	Qi et al., 2018	Brain
MAPT	ENSG00000186868	17	44038724	rs199502	17	44862347	G	A	0.743007	0.1949	0.0156	8.09E-36	0.734607	0.0582562	1.86E-36	0.265312	0.0298938	6.98E-19	3.51E-24	0.01172117	17	TRUE	Qi et al., 2018	Brain
HIST1H1C	ENSG00000187837	6	26056333	rs9467664	6	26021813	T	A	0.65035	0.0264	0.0102	0.00964691	0.400225	0.0391894	1.74E-24	0.0659629	0.0262914	0.01211023	0.001054336	0.0790097	20	TRUE	Qi et al., 2018	Brain
H1F0	ENSG00000189060	22	38202278	rs5756823	22	38196837	C	T	0.858392	0.0197	0.0138	0.1534254	-0.462337	0.0582828	2.15E-15	-0.0426097	0.0303278	0.1600308	0.1680911	0.1217625	20	FALSE	Qi et al., 2018	Brain
HMGB1	ENSG00000189403	13	31112309	rs11842871	13	31042452	G	T	0.73951	-0.0233	0.0117	0.04643111	1.19136	0.0332424	2.77E-281	-0.0195574	0.00983584	0.04676906	0.1962611	0.4715744	14	FALSE	Qi et al., 2018	Brain
OCLN	ENSG00000197822	5	68821025	rs10045526	5	68592751	G	A	0.41958	0.0027	0.0098	0.7829243	-0.315839	0.0422943	8.16E-14	-0.0085487	0.0310496	0.7830682	0.3217747	0.01404157	20	FALSE	Qi et al., 2018	Brain
VIM	ENSG00000026025	10	17274925	rs7083383	10	17260573	C	G	0.222028	0.0025	0.0143	0.861217	-0.226594	0.0138761	6.06E-60	-0.0110329	0.063112	0.8612249	0.2727151	0.09055329	20	FALSE	Vosa et al., 2018	Blood
CDH1	ENSG00000039068	16	68820286	rs11642362	16	68811307	T	C	0.433566	-0.0008	0.0096	0.9335865	-0.315847	0.00872182	3.75E-287	0.00253288	0.0303946	0.9335867	0.9795313	0.8539883	20	FALSE	Vosa et al., 2018	Blood
CASP8	ENSG00000064012	2	202125300	rs7560328	2	202164837	A	C	0.398601	0.0194	0.0096	0.04329702	-0.214284	0.00890084	4.61E-128	-0.090534	0.0449579	0.04403565	0.4704423	0.714529	20	FALSE	Vosa et al., 2018	Blood
PRKCQ	ENSG00000065675	10	6545684	rs2255088	10	6625378	C	T	0.321678	-0.0033	0.0109	0.7620786	-0.171391	0.00904469	4.47E-80	0.0192542	0.0636052	0.7621081	0.6947417	0.4356083	15	FALSE	Vosa et al., 2018	Blood
ROCK1	ENSG00000067900	18	18610756	rs142716063	18	18952751	A	G	0.013986	-0.0528	0.0434	0.2237603	0.236878	0.0325293	3.29E-13	-0.222899	0.185756	0.2301554	0.1230874	0.131832	10	FALSE	Vosa et al., 2018	Blood
ADD1	ENSG00000087274	4	2888693	rs6819310	4	2869127	C	T	0.293706	0.0511	0.0106	1.43E-06	0.23786	0.0120276	4.77E-87	0.214832	0.045869	2.82E-06	0.000887411	0.00734456	20	TRUE	Vosa et al., 2018	Blood
DNM1L	ENSG00000087470	12	32865310	rs3600	12	32896944	T	C	0.125874	-0.0382	0.0165	0.02060464	-0.345402	0.0184417	2.85E-78	0.110596	0.048134	0.0215809	0.05332457	0.6017097	20	FALSE	Vosa et al., 2018	Blood
DSP	ENSG00000096696	6	7564379	rs11243180	6	7439070	G	A	0.438811	-0.0106	0.0101	0.2939458	-0.637654	0.00806203	0	0.0166234	0.0158407	0.2939883	0.7138452	0.5761607	20	FALSE	Vosa et al., 2018	Blood
ACIN1	ENSG00000100813	14	23546298	rs10149569	14	23563895	C	A	0.0821678	-0.0112	0.0163	0.4920093	-0.121407	0.0126066	5.95E-22	0.0922519	0.134601	0.4931078	0.3546254	0.6481822	9	FALSE	Vosa et al., 2018	Blood
STK24	ENSG00000102572	13	99166324	rs4772103	13	99234131	A	G	0.442308	0.0039	0.0096	0.6845589	0.172567	0.00891324	1.65E-83	0.0225999	0.0556427	0.6846246	0.2734283	0.2785907	20	FALSE	Vosa et al., 2018	Blood
KPNB1	ENSG00000108424	17	45744856	rs141601713	17	45614919	T	C	0.041958	-0.0289	0.0242	0.2323939	-0.169724	0.0245344	4.59E-12	0.170276	0.144693	0.2392719	0.3543072	0.591248	7	FALSE	Vosa et al., 2018	Blood
BIRC2	ENSG00000110330	11	102233671	rs11827872	11	102262244	T	G	0.0751748	-0.0098	0.0242	0.6855079	-0.136661	0.0215847	2.43E-10	0.07171	0.177442	0.686115	0.8600748			FALSE	Vosa et al., 2018	Blood
LMNB1	ENSG00000113368	5	126142513	rs2271354	5	126145771	A	G	0.276224	0.0195	0.0111	0.07895928	0.181891	0.00977615	2.89E-77	0.107207	0.061297	0.08029487	0.2001857	0.459058	20	FALSE	Vosa et al., 2018	Blood
KPNA1	ENSG00000114030	3	122187294	rs73190142	3	122201610	C	G	0.157343	0.0854	0.0127	1.76E-11	0.275738	0.0137928	6.54E-89	0.309714	0.0485939	1.85E-10	3.60E-07	0.01472589	20	TRUE	Vosa et al., 2018	Blood
TJP2	ENSG00000119139	9	71803174	rs1416738	9	71787927	T	C	0.393357	0.024	0.0095	0.01152658	-0.679794	0.00784968	0	-0.0353048	0.0139808	0.01156188	0.07684917	0.7073372	20	FALSE	Vosa et al., 2018	Blood
APC	ENSG00000134982	5	112112565	rs1734242	5	112125419	G	A	0.127622	0.0295	0.0154	0.05541801	-0.0759217	0.0109139	3.49E-12	-0.388558	0.210391	0.06477005	0.03036535	0.03897929	19	FALSE	Vosa et al., 2018	Blood
DBNL	ENSG00000136279	7	44096647	rs2528374	7	43976548	G	C	0.0559441	0.0457	0.0219	0.03691002	-0.539491	0.015575	6.73E-263	-0.0847094	0.0406674	0.03725292	0.02617208	0.0015638	20	FALSE	Vosa et al., 2018	Blood
CASP6	ENSG00000138794	4	110617262	rs13144085	4	110628473	C	T	0.0559441	0.0039	0.0193	0.83986	0.155261	0.0216863	8.10E-13	0.025119	0.124356	0.8399229	0.9717244	0.8813971	12	FALSE	Vosa et al., 2018	Blood
GSN	ENSG00000148180	9	124032599	rs10760167	9	124035545	A	G	0.548951	-0.0343	0.0099	0.00053093	-0.662881	0.00800026	0	0.0517438	0.0149479	0.00053693	0.008048225	0.08945405	20	FALSE	Vosa et al., 2018	Blood
GAS2	ENSG00000148935	11	22740894	rs4447177	11	22646025	G	A	0.15035	0.0033	0.0133	0.8040413	0.15242	0.0101964	1.60E-50	0.0216506	0.0872706	0.8040678	0.9053025	0.4432954	20	FALSE	Vosa et al., 2018	Blood
DFFA	ENSG00000160049	1	10524581	rs7548453	1	10589836	G	A	0.51049	-0.0055	0.01	0.5823194	0.252037	0.0089825	3.13E-173	-0.0218222	0.0396843	0.5823918	0.03867274	0.7347652	20	FALSE	Vosa et al., 2018	Blood
LMNA	ENSG00000160789	1	156081122	rs609625	1	156089631	G	C	0.0716783	0.0589	0.0209	0.00482965	0.528427	0.0135931	0	0.111463	0.0396552	0.00494176	0.00533209	0.00039882	20	FALSE	Vosa et al., 2018	Blood
PRKCD	ENSG00000163932	3	53208379	rs2230494	3	53220215	A	G	0.288462	0.0465	0.0115	5.27E-05	0.212967	0.00980744	1.49E-104	0.218344	0.0549273	7.03E-05	0.000141071	0.08661588	20	TRUE	Vosa et al., 2018	Blood
HMGB2	ENSG00000164104	4	174254561	rs7656222	4	174335738	A	T	0.527972	0.0005	0.0102	0.9609037	0.0957548	0.00899731	1.89E-26	0.00522167	0.106523	0.9609041	0.9281941	0.9477807	20	FALSE	Vosa et al., 2018	Blood
CASP3	ENSG00000164305	4	185559756	rs72689261	4	185590897	A	C	0.111888	0.0045	0.0146	0.7579156	-0.56825	0.0151553	1.11E-307	-0.0079191	0.0256938	0.7579235	0.7005626	0.8843126	20	FALSE	Vosa et al., 2018	Blood
CASP7	ENSG00000165806	10	115464802	rs4353229	10	115489589	C	T	0.234266	-0.0125	0.0107	0.2427163	-0.66876	0.00909944	0	0.0186913	0.0160018	0.2427758	0.6036084	0.7677355	20	FALSE	Vosa et al., 2018	Blood
CTNNB1	ENSG00000168036	3	41268957	rs1722846	3	41233889	T	C	0.45979	0.0301	0.0101	0.00288062	-0.250356	0.00906553	7.09E-168	-0.120229	0.0405767	0.00304661	0.1741666	0.6460329	20	FALSE	Vosa et al., 2018	Blood
HIST1H1E	ENSG00000168298	6	26156951	rs62625309	6	26085626	C	T	0.0122378	-0.0921	0.0493	0.06174059	0.403898	0.0468799	6.96E-18	-0.228028	0.124897	0.06789138	0.0525982	0.8540779	12	FALSE	Vosa et al., 2018	Blood
FNTA	ENSG00000168522	8	42915134	rs9657192	8	43023447	T	A	0.113636	0.0235	0.0154	0.1270163	-0.0569757	0.0102904	3.08E-08	-0.412457	0.280368	0.1412575	0.171546	0.2926599	20	FALSE	Vosa et al., 2018	Blood
PTK2	ENSG00000169398	8	141840157	rs4961234	8	141840881	T	C	0.578671	-0.0343	0.0098	0.00046526	0.180841	0.00888451	4.23E-92	-0.18967	0.0549867	0.00056188	0.000525702	0.9990542	20	TRUE	Vosa et al., 2018	Blood
DFFB	ENSG00000169598	1	3787919	rs72640980	1	3834764	G	A	0.173077	-0.0016	0.0169	0.9245733	-0.162248	0.0159499	2.63E-24	0.00986143	0.104166	0.9245766	0.7755078	0.8134155	14	FALSE	Vosa et al., 2018	Blood
PLEC	ENSG00000178209	8	145020111	rs11781667	8	145040856	T	G	0.377622	0.0235	0.0099	0.01760907	-0.28405	0.0104024	3.58E-164	-0.082732	0.0349845	0.01803889	0.02280896	0.5832694	20	FALSE	Vosa et al., 2018	Blood
PAK2	ENSG00000180370	3	196513123	rs2084385	3	196553867	A	G	0.129371	-0.0055	0.0151	0.71568	0.216416	0.0155511	5.04E-44	-0.0254141	0.069797	0.7157731	0.4243611	0.2745337	19	FALSE	Vosa et al., 2018	Blood
SATB1	ENSG00000182568	3	18436972	rs9851736	3	18430301	T	C	0.424825	0.0105	0.0102	0.3032862	0.175235	0.00956344	5.38E-75	0.0599196	0.0582994	0.3040481	0.002032091	0.00152056	20	FALSE	Vosa et al., 2018	Blood
MAPT	ENSG00000186868	17	44038724	rs62074125	17	44852612	C	A	0.225524	0.0962	0.0129	8.83E-14	-0.0984498	0.0146407	1.76E-11	-0.977148	0.195666	5.92E-07	1.03E-07	4.34E-10	6	TRUE	Vosa et al., 2018	Blood
HIST1H1C	ENSG00000187837	6	26056333	rs807214	6	26061769	G	C	0.375874	-0.024	0.0099	0.01534036	-0.233772	0.0105945	6.80E-108	0.102664	0.0426038	0.01596378	0.000328948	0.1378694	20	TRUE	Vosa et al., 2018	Blood
H1F0	ENSG00000189060	22	38202278	rs5756825	22	38199950	T	C	0.468531	0.0199	0.0095	0.03619439	0.750783	0.00777016	0	0.0265057	0.0126564	0.03623818	0.00408712	0.01810044	20	FALSE	Vosa et al., 2018	Blood
HMGB1	ENSG00000189403	13	31112309	rs1056130	13	31038651	A	G	0.263986	0.0245	0.0117	0.03625846	0.0800573	0.0113155	1.49E-12	0.306031	0.152412	0.04465225	0.09944262	0.7562546	5	FALSE	Vosa et al., 2018	Blood
SPTAN1	ENSG00000197694	9	131355403	rs7036322	9	131417140	A	C	0.129371	-0.0031	0.0159	0.8454176	-0.0555623	0.00930038	2.31E-09	0.0557932	0.286318	0.8454988	0.8778587	0.6220713	6	FALSE	Vosa et al., 2018	Blood
OCLN	ENSG00000197822	5	68821025	rs10042124	5	68658499	A	G	0.305944	-0.0342	0.0111	0.00206251	0.130808	0.00976395	6.30E-41	-0.261453	0.0870727	0.00267601	0.1041364	0.08489406	20	FALSE	Vosa et al., 2018	Blood

ASPARAGINE N LINKED GLYCOSYLATION (REACTOME)

Gene	probeID	ProbeChr	Probe bp	topSNP	topSNP chr	topSNP bp	A1	A2	Freq	b GWAS	se GWAS	p GWAS	b eQTL	se eQTL	p eQTL	b SMR	se SMR	p SMR	p SMR multi	p HEIDI	nsnp HEIDI	PASS BONF	Data source	Analyte
ST3GAL1	ENSG00000008513	8	134525637	rs16904933	8	134502656	C	T	0.833916	-0.0193	0.014	0.1680269	0.339488	0.0524958	1.00E-10	-0.0568503	0.0421651	0.1775692	0.1706851	0.06438345	13	FALSE	Qi et al., 2018	Brain
ST3GAL6	ENSG00000064225	3	98495562	rs828600	3	98466607	A	G	0.861888	-0.002	0.0142	0.8879923	-0.33525	0.0495358	1.31E-11	0.0059657	0.0423656	0.8880164	0.8005765	0.06788417	20	FALSE	Qi et al., 2018	Brain
CTSA	ENSG00000064601	20	44523525	rs6073967	20	44571220	C	T	0.643357	-0.0213	0.0099	0.03143556	0.324353	0.0508241	1.75E-10	-0.0656692	0.0322101	0.04147268	0.0253848	0.6371675	20	FALSE	Qi et al., 2018	Brain
ST6GALNAC2	ENSG00000070731	17	74571415	rs1867561	17	74582025	C	G	0.812937	-0.0165	0.013	0.2043588	0.430296	0.0550316	5.32E-15	-0.0383457	0.0306072	0.2102667	0.3449138	0.7969583	12	FALSE	Qi et al., 2018	Brain
ST6GAL1	ENSG00000073849	3	186722307	rs2041965	3	186648411	C	T	0.657343	-0.0068	0.0102	0.5049851	0.372816	0.0477243	5.63E-15	-0.0182396	0.0274588	0.5065289	0.8844149	0.5236415	20	FALSE	Qi et al., 2018	Brain
NSF	ENSG00000073969	17	44751432	rs17698176	17	44819595	G	T	0.20979	0.0652	0.0126	2.28E-07	0.410489	0.0534624	1.61E-14	0.158835	0.0370153	1.78E-05	5.66E-06	5.73E-07	8	TRUE	Qi et al., 2018	Brain
CAPZB	ENSG00000077549	1	19738666	rs12117356	1	19658162	C	G	0.807692	0.0036	0.0118	0.7603016	1.10409	0.0318829	9.04E-263	0.00326059	0.0106879	0.7603106	0.8477152	0.8377617	20	FALSE	Qi et al., 2018	Brain
CD59	ENSG00000085063	11	33738899	rs3181269	11	33755956	C	T	0.769231	0.0002	0.0118	0.9864772	0.570688	0.0551161	4.00E-25	0.00035045	0.0206768	0.9864772	0.6003399	0.8547608	20	FALSE	Qi et al., 2018	Brain
ALG6	ENSG00000088035	1	63868747	rs855303	1	64051299	T	C	0.70979	0.0206	0.0106	0.0519683	0.632798	0.0501729	1.81E-36	0.0325538	0.0169487	0.05476597	0.3309264	0.2522155	20	FALSE	Qi et al., 2018	Brain
EDEM2	ENSG00000088298	20	33784544	rs6088735	20	33745676	T	C	0.234266	0.0202	0.0112	0.07129852	-0.372206	0.0417136	4.54E-19	-0.054271	0.0306994	0.077091	0.01818816	0.8077278	20	FALSE	Qi et al., 2018	Brain
DYNLL1	ENSG00000088986	12	120921974	rs12310837	12	120880572	A	G	0.802448	-0.0254	0.0128	0.04721405	0.313888	0.0464368	1.39E-11	-0.0809206	0.0424998	0.05690721	0.03121166	0.362197	14	FALSE	Qi et al., 2018	Brain
GANAB	ENSG00000089597	11	62403201	rs1058678	11	62361037	T	C	0.812937	-0.0041	0.0143	0.7743319	0.391937	0.0552528	1.31E-12	-0.0104609	0.0365153	0.774511	0.6036268	0.04890184	12	FALSE	Qi et al., 2018	Brain
SCFD1	ENSG00000092108	14	31148168	rs2070339	14	31152826	G	A	0.339161	-0.017	0.0103	0.09884369	0.365114	0.0389518	7.02E-21	-0.0465608	0.0286443	0.1040605	0.2087001	0.277088	20	FALSE	Qi et al., 2018	Brain
SEC22C	ENSG00000093183	3	42616016	rs17030458	3	42632238	C	G	0.882867	-0.0096	0.0154	0.5330371	-0.683207	0.072004	2.34E-21	0.0140514	0.0225893	0.5339184	0.57597	0.9559437	20	FALSE	Qi et al., 2018	Brain
ARCN1	ENSG00000095139	11	118458417	rs11216892	11	118424008	C	T	0.874126	-0.0005	0.0141	0.9717121	0.407912	0.0672155	1.29E-09	-0.0012258	0.0345669	0.9717126	0.9945717	0.9967939	11	FALSE	Qi et al., 2018	Brain
SEC23A	ENSG00000100934	14	39539986	rs11848164	14	39541159	C	T	0.527972	-0.0179	0.0098	0.06777037	0.305575	0.0480642	2.05E-10	-0.058578	0.0333679	0.07917085	0.4406141	0.05743821	20	FALSE	Qi et al., 2018	Brain
ST8SIA5	ENSG00000101638	18	44299150	rs72915493	18	44267950	G	A	0.659091	0.0155	0.0107	0.1474498	0.346255	0.0503552	6.15E-12	0.0447648	0.0315804	0.1563411	0.6650563	0.2046751	20	FALSE	Qi et al., 2018	Brain

COG4	ENSG00000103051	16	70535969	rs12445830	16	70502493	A	G	0.898601	-0.0214	0.0175	0.2213837	-0.55291	0.0693795	1.60E-15	0.0387043	0.0320212	0.2267742	0.2612337	0.9393306	20	FALSE	Qi et al., 2018	Brain
TUSC3	ENSG00000104723	8	15449441	rs2111755	8	15562357	G	C	0.342657	-0.0004	0.01	0.9680931	0.302369	0.041997	6.03E-13	-0.0013229	0.0330727	0.9680936	0.1970082	0.1025382	20	FALSE	Qi et al., 2018	Brain
NAPA	ENSG00000105402	19	48004695	rs55800589	19	47990889	G	C	0.924825	-0.0592	0.0195	0.00239821	-0.436473	0.0781223	2.31E-08	0.135633	0.0508459	0.00764128	0.007641279	0.114106	6	FALSE	Qi et al., 2018	Brain
COPE	ENSG00000105669	19	19020264	rs1127821	19	19011244	C	T	0.482517	0.0204	0.0096	0.03358661	0.386158	0.0386055	1.48E-23	0.0528282	0.0254151	0.03765309	0.04182814	0.632602	20	FALSE	Qi et al., 2018	Brain
SEC23IP	ENSG00000107651	10	121677118	rs4752351	10	121685016	T	C	0.762238	0.0699	0.0121	7.61E-09	0.288934	0.0475232	1.20E-09	0.241924	0.0577677	2.82E-05	3.58E-05	0.08467302	20	TRUE	Qi et al., 2018	Brain
GOSR2	ENSG00000108433	17	45052743	rs874044	17	45028458	C	T	0.972028	0.0128	0.0235	0.585973	0.977321	0.159326	8.56E-10	0.013097	0.0241399	0.5874423	0.7286979	0.3274536	4	FALSE	Qi et al., 2018	Brain
GOSR1	ENSG00000108587	17	28829495	rs12449388	17	28714383	T	C	0.515734	-0.0005	0.0098	0.9593093	0.473816	0.0384618	7.15E-35	-0.0010553	0.0206833	0.9593096	0.2147378	0.09749148	20	FALSE	Qi et al., 2018	Brain
ST3GAL4	ENSG00000110080	11	126267887	rs10790802	11	126277812	C	T	0.561189	0.0059	0.0096	0.5388299	-0.284454	0.039799	8.85E-13	-0.0207415	0.0338734	0.5403236	0.8492879	0.8646057	20	FALSE	Qi et al., 2018	Brain
MLEC	ENSG00000110917	12	121132169	rs56232241	12	121173386	T	C	0.947552	0.0724	0.0231	0.00172324	-0.667715	0.119411	2.25E-08	-0.108429	0.0396593	0.00625662	0.006256616	0.1664424	11	FALSE	Qi et al., 2018	Brain
ST8SIA1	ENSG00000111728	12	22403341	rs3819877	12	22439031	C	T	0.763986	0.0012	0.0121	0.9210005	-0.2964	0.0456274	8.24E-11	-0.0040486	0.040828	0.9210096	0.6684275	0.402772	11	FALSE	Qi et al., 2018	Brain
MAN2A1	ENSG00000112893	5	109115196	rs17481197	5	108965109	T	C	0.935315	-0.0215	0.0209	0.3036168	-0.50046	0.0825462	1.34E-09	0.0429604	0.0423584	0.3104815	0.2594268	0.8492741	19	FALSE	Qi et al., 2018	Brain
TFG	ENSG00000114354	3	100448007	rs7653531	3	100433797	T	C	0.638112	0.0061	0.0105	0.5612725	-0.301865	0.0478693	2.86E-10	-0.0202077	0.0349311	0.5629248	0.6723586	0.1007627	20	FALSE	Qi et al., 2018	Brain
LMAN2L	ENSG00000114988	2	97388733	rs6727384	2	97400324	A	G	0.622378	0.0161	0.0096	0.09352619	-0.266466	0.043261	7.30E-10	-0.0604204	0.0373386	0.105625	0.105625	0.6472827	9	FALSE	Qi et al., 2018	Brain
MOGS	ENSG00000115275	2	74690360	rs1047911	2	74699778	C	A	0.858392	-0.0074	0.0135	0.5835902	-0.729585	0.0647021	1.72E-29	0.0101428	0.0185255	0.5840342	0.5331894	0.05209782	20	FALSE	Qi et al., 2018	Brain
CHST10	ENSG00000115526	2	101021222	rs6729552	2	100974413	T	C	0.496503	-0.0205	0.0098	0.03645312	-0.377007	0.0488801	1.23E-14	0.0543757	0.0269333	0.04349736	0.07129121	0.8916475	20	FALSE	Qi et al., 2018	Brain
EDEM3	ENSG00000116406	1	184691842	rs9425635	1	184663898	C	T	0.566434	0.0064	0.0094	0.4959657	0.535284	0.0447027	4.84E-33	0.0119563	0.0175891	0.4966608	0.2624157	0.2074655	20	FALSE	Qi et al., 2018	Brain
CAPZA1	ENSG00000116489	1	113188018	rs4839257	1	113219388	T	A	0.723776	0.0225	0.0108	0.03722085	-0.44122	0.0513318	8.29E-18	-0.0509949	0.0251863	0.04289715	0.02521541	0.4549249	19	FALSE	Qi et al., 2018	Brain
B4GALT6	ENSG00000118276	18	29234004	rs718780	18	29255261	T	C	0.438811	0.0133	0.0095	0.1615133	-0.202776	0.0354836	1.10E-08	-0.0655895	0.048235	0.1738967	0.1902872	0.9148864	20	FALSE	Qi et al., 2018	Brain
PPP6C	ENSG00000119414	9	127930535	rs620677	9	127819261	G	C	0.676573	0.0006	0.0101	0.9526288	0.596341	0.0524999	6.70E-30	0.00100614	0.0169369	0.9526294	0.06030085	0.01125773	20	FALSE	Qi et al., 2018	Brain
SLC17A5	ENSG00000119899	6	74333490	rs5007649	6	74339869	C	T	0.910839	0.0091	0.0184	0.6209071	-0.536704	0.0630929	1.79E-17	-0.0169553	0.0343412	0.6214958	0.7221536	0.9774998	20	FALSE	Qi et al., 2018	Brain
ALG5	ENSG00000120697	13	37549155	rs9576144	13	37518022	T	C	0.386364	-0.0164	0.01	0.1010052	-0.252961	0.0460059	3.83E-08	0.0648321	0.0412527	0.1160473	0.1160473	0.8954874	10	FALSE	Qi et al., 2018	Brain
SEC22A	ENSG00000121542	3	122956875	rs6796833	3	122982226	A	G	0.800699	-0.0081	0.0128	0.5268561	0.771166	0.0916333	3.90E-17	-0.0105036	0.0166451	0.5280202	0.7365612	0.453622	20	FALSE	Qi et al., 2018	Brain
B4GALT4	ENSG00000121578	3	118945264	rs7650288	3	118950288	G	A	0.143357	0.0276	0.0125	0.02724427	0.303369	0.0526348	8.23E-09	0.0909783	0.044124	0.03921927	0.07722709	0.463624	18	FALSE	Qi et al., 2018	Brain
COPA	ENSG00000122218	1	160286126	rs16831718	1	160233897	A	G	0.951049	0.0145	0.0227	0.5229748	0.530668	0.0935909	1.43E-08	0.0273241	0.0430469	0.5255906	0.5255906	0.597705	10	FALSE	Qi et al., 2018	Brain
CHST8	ENSG00000124302	19	34188637	rs17760970	19	33929094	C	T	0.573427	0.0033	0.0097	0.7337013	0.25081	0.0369589	1.15E-11	0.0131574	0.0387233	0.7340226	0.4423787	0.1527832	20	FALSE	Qi et al., 2018	Brain
NAGK	ENSG00000124357	2	71299204	rs13010446	2	71291087	T	G	0.756993	-0.0048	0.011	0.6625729	0.366987	0.0544692	1.61E-11	-0.0130795	0.0300366	0.6632349	0.4752999	0.3287276	20	FALSE	Qi et al., 2018	Brain
NAPB	ENSG00000125814	20	23378642	rs6083085	20	23336119	A	G	0.711538	0.0097	0.0101	0.3368559	1.16048	0.0315164	8.42E-297	0.00835864	0.00870629	0.3370202	0.1172971	0.3125153	20	FALSE	Qi et al., 2018	Brain
TBC1D20	ENSG00000125875	20	429660	rs3746794	20	419067	C	T	0.508741	-0.0063	0.0102	0.536808	0.339864	0.0442151	1.51E-14	-0.0185368	0.0301088	0.5381172	0.5420023	0.4585048	12	FALSE	Qi et al., 2018	Brain
CANX	ENSG00000127022	5	179131777	rs13182141	5	179126632	C	T	0.486014	-0.0003	0.0102	0.9765362	0.380574	0.0376294	4.80E-24	-0.0007883	0.0268017	0.9765363	0.5781448	0.4643663	20	FALSE	Qi et al., 2018	Brain
SRD5A3	ENSG00000128039	4	56225769	rs11935857	4	56204865	C	A	0.821678	-0.0079	0.0148	0.5934911	-0.457069	0.0488819	8.72E-21	0.017284	0.0324329	0.5940916	0.91247	0.7310562	20	FALSE	Qi et al., 2018	Brain
MGAT3	ENSG00000128268	22	39870774	rs5750828	22	39835083	C	T	0.690559	-0.0326	0.0113	0.00391469	0.327271	0.0496747	4.45E-11	-0.0996116	0.0376932	0.00822496	0.04947239	0.2710308	20	FALSE	Qi et al., 2018	Brain
DAD1	ENSG00000129562	14	23045990	rs11628361	14	23032088	A	C	0.912587	0.0072	0.0176	0.6824729	-1.98669	0.054436	1.28E-291	-0.0036241	0.00885949	0.6824918	0.7804472	0.8307931	20	FALSE	Qi et al., 2018	Brain
GFPT2	ENSG00000131459	5	179754038	rs7719192	5	179744488	A	G	0.77972	0.005	0.0125	0.6891565	-0.348108	0.0503433	4.69E-12	-0.0143634	0.0359684	0.6896483	0.6820366	0.6646746	20	FALSE	Qi et al., 2018	Brain
ACTR10	ENSG00000131966	14	58684274	rs56759383	14	58653480	A	C	0.575175	-0.01	0.0099	0.3124469	-0.261863	0.0473284	3.15E-08	0.0381879	0.0384309	0.3203799	0.3203799	0.7481786	9	FALSE	Qi et al., 2018	Brain
COG6	ENSG00000133103	13	40297783	rs73179582	13	40355430	G	A	0.938811	0.0346	0.0227	0.1274515	0.646084	0.116408	2.85E-08	0.0535534	0.0364356	0.1416132	0.1416132	0.4559722	7	FALSE	Qi et al., 2018	Brain
NAPG	ENSG00000134265	18	10539330	rs653624	18	10553789	A	C	0.545455	0.0036	0.01	0.7188471	-0.62377	0.0443143	5.33E-45	-0.0057714	0.0160368	0.7189351	0.3899611	0.4921442	20	FALSE	Qi et al., 2018	Brain
KDELR2	ENSG00000136240	7	6504728	rs4574760	7	6537655	T	C	0.428322	0.0011	0.0098	0.9106292	-0.236195	0.0375401	3.14E-10	-0.0046572	0.0414977	0.9106434	0.5434235	0.2178951	17	FALSE	Qi et al., 2018	Brain
DPM2	ENSG00000136908	9	130699070	rs11789803	9	130706128	G	A	0.86014	0.0037	0.0137	0.7871041	-1.41346	0.0397183	2.22E-277	-0.0026177	0.00969281	0.7871101	0.9926475	0.9950683	20	FALSE	Qi et al., 2018	Brain
SPTBN5	ENSG00000137877	15	42163310	rs57950483	15	42269455	G	A	0.131119	0.0094	0.0156	0.5467987	0.396302	0.0681266	5.99E-09	0.0237193	0.0395745	0.5489347	0.5577615	0.5137165	5	FALSE	Qi et al., 2018	Brain
PREB	ENSG00000138073	2	27355583	rs11695930	2	27354971	C	T	0.818182	-0.0183	0.0129	0.1560143	1.32923	0.0413642	1.44E-226	-0.0137674	0.00971431	0.1564173	0.03077446	0.05297839	15	FALSE	Qi et al., 2018	Brain
USO1	ENSG00000138768	4	76692572	rs3821977	4	76628351	G	A	0.73951	-0.0034	0.0113	0.7635022	-0.264273	0.0438954	1.74E-09	0.0128655	0.0428122	0.7637882	0.6017096	0.02930537	12	FALSE	Qi et al., 2018	Brain
RNF185	ENSG00000138942	22	31579583	rs55783055	22	31557798	C	T	0.893357	0.0376	0.0167	0.02435408	0.421061	0.0631993	2.69E-11	0.0892983	0.0418653	0.03292541	0.008899769	0.09202608	17	FALSE	Qi et al., 2018	Brain
ALG10	ENSG00000139133	12	34178922	rs2389242	12	34001484	A	G	0.370629	0.0127	0.0099	0.1995522	-0.383099	0.0555945	5.54E-12	-0.0331507	0.0262859	0.2072509	0.3915694	0.03914755	13	FALSE	Qi et al., 2018	Brain
PMM2	ENSG00000140650	16	8912934	rs2304469	16	8896094	A	G	0.501748	-0.0062	0.0096	0.5183873	-0.421521	0.0475196	7.28E-19	0.0147086	0.022835	0.5194922	0.3624848	0.1590294	20	FALSE	Qi et al., 2018	Brain
ST6GAL2	ENSG00000144057	2	107460810	rs1034815	2	107569785	T	A	0.370629	0.0053	0.0102	0.6033369	0.240851	0.036188	2.82E-11	0.0220053	0.0424786	0.6044363	0.6882683	0.9229911	20	FALSE	Qi et al., 2018	Brain
DYNC1LI1	ENSG00000144635	3	32589914	rs28403572	3	32612434	T	C	0.0979021	0.0093	0.0178	0.6013418	-0.389773	0.0692132	1.79E-08	-0.02386	0.0458637	0.6028979	0.6028979	0.5890162	9	FALSE	Qi et al., 2018	Brain
ANK3	ENSG00000151150	10	62140703	rs11816516	10	62093251	G	A	0.744755	0.0105	0.0111	0.3441762	-0.316931	0.0488779	8.92E-11	-0.0331302	0.0353941	0.3492538	0.3492538	0.8636608	17	FALSE	Qi et al., 2018	Brain
SEC13	ENSG00000157020	3	10348838	rs154236	3	10356314	T	C	0.645105	0.0122	0.0099	0.2178284	-0.269883	0.0430421	3.61E-10	-0.0452048	0.0373843	0.2265895	0.3126181	0.5969388	20	FALSE	Qi et al., 2018	Brain
B4GALT5	ENSG00000158470	20	48289948	rs6067181	20	48300121	T	C	0.548951	0.0262	0.0094	0.00531601	-0.412147	0.0446042	2.46E-20	-0.0635695	0.0238224	0.00761966	0.03040059	0.3328233	20	FALSE	Qi et al., 2018	Brain
ALG8	ENSG00000159063	11	77831344	rs10793287	11	77846706	C	G	0.561189	0.0008	0.0096	0.9335865	-0.52582	0.0339523	4.25E-54	-0.0015214	0.0182575	0.9335875	0.6266638	0.1943512	20	FALSE	Qi et al., 2018	Brain
AMFR	ENSG00000159461	16	56427407	rs3790112	16	56381197	G	T	0.646853	0.0354	0.0108	0.00104628	0.297982	0.041774	9.81E-13	0.118799	0.0398871	0.00289773	0.002840908	0.4435005	20	FALSE	Qi et al., 2018	Brain
GNE	ENSG00000159921	9	36245745	rs62541810	9	36298229	C	T	0.812937	-0.0061	0.0139	0.660771	-0.437449	0.0615042	1.14E-12	0.0139445	0.0318356	0.6613747	0.6963844	0.1988166	20	FALSE	Qi et al., 2018	Brain
TRAPPC10	ENSG00000160218	21	45479316	rs2838468	21	45437074	G	C	0.83042	-0.0003	0.0124	0.9806982	0.325363	0.0509717	1.73E-10	-0.000922	0.0381115	0.9806984	0.5002005	0.1003961	20	FALSE	Qi et al., 2018	Brain
SPTBN4	ENSG00000160460	19	41027259	rs1165839	19	41076053	C	T	0.606643	-0.0081	0.01	0.4179402	-0.552071	0.0471834	1.27E-31	0.014672	0.018157	0.4190526	0.06283108	0.5251349	13	FALSE	Qi et al., 2018	Brain
TGFA	ENSG00000163235	2	70727868	rs7571789	2	70714793	C	T	0.498252	0.0138	0.01	0.1675866	0.300217	0.035237	1.60E-17	0.0459668	0.0337434	0.1731202	0.6071758	0.3105991	20	FALSE	Qi et al., 2018	Brain
RPN1	ENSG00000163902	3	128369367	rs6806687	3	128391789	T	C	0.354895	0.0269	0.0109	0.01359121	0.290825	0.0393451	1.45E-13	0.0924955	0.0395134	0.01923912	0.0562683	0.3005597	20	FALSE	Qi et al., 2018	Brain
RFT1	ENSG00000163933	3	53143488	rs2082093	3	53122969	A	G	0.583916	-0.026	0.0094	0.00567559	-0.450696	0.0387585	2.96E-31	0.0576885	0.0214385	0.00712637	0.02665972	0.01761546	20	FALSE	Qi et al., 2018	Brain
SLC35A1	ENSG00000164414	6	88201197	rs7772351	6	88361017	G	A	0.617133	0.029	0.0096	0.0025208	1.23954	0.0334416	1.00E-300	0.0233958	0.00777049	0.00260517	0.03638124	0.1974343	20	FALSE	Qi et al., 2018	Brain
COG5	ENSG00000164597	7	107023574	rs10953531	7	106827867	C	T	0.777972	-0.0145	0.0113	0.199427	0.287115	0.0459352	4.09E-10	-0.0505024	0.0401779	0.2087642	0.1750181	0.3812803	20	FALSE	Qi et al., 2018	Brain
COG1	ENSG00000166685	17	71196887	rs9890798	17	71239598	T	C	0.896853	0.0025	0.0163	0.8781032	0.376199	0.0582576	1.06E-10	0.00664541	0.0433403	0.8781373	0.8781373	0.6043874	5	FALSE	Qi et al., 2018	Brain
ENGASE	ENSG00000167280	17	77077851	rs73398101	17	77077452	T	C	0.937063	-0.0225	0.0206	0.2747307	0.671865	0.0826137	4.20E-16	-0.0334889	0.0309362	0.2790243	0.8860645	0.4212289	10	FALSE	Qi et al., 2018	Brain
TRAPPC2L	ENSG00000167515	16	88925861	rs11076728	16	88925567	G	T	0.344406	0.0274	0.0103	0.00780956	-1.21083	0.0324535	1.15E-304	-0.0226291	0.00852816	0.00796717	0.02158729	0.4215325	20	FALSE	Qi et al., 2018	Brain
TUBA1C	ENSG00000167553	12	49624816	rs77888738	12	49512929	G	A	0.312937	0.0323	0.0105	0.00209664	0.962472	0.0422804	1.04E-114	0.0335594	0.0110086	0.00230001	0.001930488	0.00833916	20	FALSE	Qi et al., 2018	Brain
TRAPPC9	ENSG00000167632	8	141105632	rs10099688	8	141441957	T	C	0.77972	0.0112	0.0123	0.3625225	0.534983	0.0582097	3.91E-20	0.0209352	0.0231039	0.3648656	0.2841193	0.910115	16	FALSE	Qi et al., 2018	Brain
ARF4	ENSG00000168374	3	57570518	rs4597680	3	57596105	T	C	0.178322	-0.0084	0.0145	0.5623798	0.362071	0.0433555	6.76E-17	-0.0231999	0.0401436	0.5633171	0.2905735	0.76768	20	FALSE	Qi et al., 2018	Brain
GLB1	ENSG00000170266	3	33088417	rs6780220	3	33087200	A	C	0.879371	0.0208	0.0133	0.1178388	-0.454668	0.0605698	6.07E-14	-0.0457477	0.0298802	0.1257608	0.1684101	0.05850083	17	FALSE	Qi et al., 2018	Brain
MANEA	ENSG00000172469	6	96041376	rs117103160	6	96025388	T	G	0.0926573	-0.0402	0.0173	0.02014161	-0.885145	0.131651	1.78E-11	0.0454163	0.0206792	0.02807546	0.0103901	0.00917287	18	FALSE	Qi et al., 2018	Brain
GMPPB	ENSG00000173540	3	49757830	rs56176327	3	49706736	G	C	0.88986	-0.0033	0.0161	0.8375964	-0.593486	0.0636932	1.19E-20	0.00556037	0.0271344	0.8376351	0.04907146	0.00655965	20	FALSE	Qi et al., 2018	Brain
RAB1B	ENSG00000174903	11	66040483	rs3814738	11	66060538	T	G	0.192308	-0.0149	0.0125	0.2332612	-0.362147	0.0560584	1.05E-10	0.0411435	0.035099	0.2411117	0.5604662	0.2254745	15	FALSE	Qi et al., 2018	Brain
TUBB6	ENSG00000176014	18	12325993	rs4073975	18	12279075	T	C	0.353147	0.0093	0.0111	0.4021218	-0.410417	0.0528212	7.85E-15	-0.0226599	0.0272024	0.4048397	0.5111237	0.8437164	13	FALSE	Qi et al., 2018	Brain
MAN1B1	ENSG00000177239	9	139992507	rs6420280	9	140002501	C	T	0.76049	0.015	0.0115	0.192115	-0.539391	0.0458869	6.67E-32	-0.0278092	0.0214512	0.1948408	0.1874741	0.357705	20	FALSE	Qi et al., 2018	Brain
ST8SIA3	ENSG00000177511	18	55028503	rs1942964	18	55027212	G	T	0.506993	-0.0096	0.0103	0.3513165	0.231267	0.0424208	4.99E-08	-0.0415104	0.0451834	0.3582475	0.3582475	0.4913769	5	FALSE	Qi et al., 2018	Brain
BET1L	ENSG00000177951	11	187606	rs17156153	11	270514	C	T	0.909091	0.029	0.0176	0.09940865	0.533529	0.0822961	8.99E-11	0.0543551	0.0340367	0.1102757	0.2218519	0.04516295	18	FALSE	Qi et al., 2018	Brain
MPI	ENSG00000178802	15	75187072	rs3765066	15	75140854	A	G	0.638112	0.0299	0.0099	0.00252606	0.427923	0.0414205	5.09E-25	0.0698724	0.0241033	0.0037451	0.00220215	0.03334343	20	FALSE	Qi et al., 2018	Brain
MCFD2	ENSG00000180398	2	47149001	rs7601491	2	47150626	A	G	0.800699	0.0041	0.013	0.7524696	1.33826	0.0348179	0	0.00306368	0.00971442	0.7524777	0.5089052	0.4340374	20	FALSE	Qi et al., 2018	Brain
SLC35C1	ENSG00000181830	11	45830094	rs61884469	11	45820502	G	A	0.928322	0.0123	0.0189	0.5151797	-0.65765	0.0942532	3.01E-12	-0.018703	0.0288634	0.5169971	0.2598783	0.7979465	20	FALSE	Qi et al., 2018	Brain
NUDT14	ENSG00000183828	14	105643467	rs3825761	14	105638931	T	C	0.361888	-0.0186	0.0098	0.05770146	-0.501588	0.0372499	2.50E-41	0.0370822	0.0197311	0.06019232	0.01937297	0.02814964	20	FALSE	Qi et al., 2018	Brain
TMED9	ENSG00000184840	5	177021142	rs186803	5	177016095	C	T	0.548951	0.0133	0.0097	0.1703332	0.48477	0.0445706	1.49E-27	0.0274357	0.0201678	0.1737138	0.06208392	0.0575554	20	FALSE	Qi et al., 2018	Brain
CD55	ENSG00000196352	1	207514582	rs61821090	1	207582704	T	C	0.0944056	0.0263	0.0156	0.09181556	0.601031	0.063329	2.30E-21	0.0437582	0.0263617	0.09693231	0.1999508	0.6304809	19	FALSE	Qi et al., 2018	Brain
MAN2A2	ENSG00000196547	15	91455631	rs2677744	15	91450441	G	A	0.653846	-0.006	0.0099	0.5444745	0.264037	0.0451525	4.99E-09	-0.0227241	0.0376956	0.5466207	0.7517574	0.3190821	20	FALSE	Qi et al., 2018	Brain
TRAPPC4	ENSG00000196655	11	118892653	rs7131534	11	118886656	G	A	0.800699	0.0031	0.0117	0.7910424	-1.17905	0.0319874	2.05E-297	-0.0026293	0.00992354	0.7910477	0.8488733	0.7255453	20	FALSE	Qi et al., 2018	Brain
TRIM13	ENSG00000204977	13	50582320	rs61961294	13	50565451	T	C	0.844406	-0.0205	0.0135	0.1288837	-0.273709	0.0447093	9.24E-10	0.0748972	0.0508172	0.1405206	0.1685912	0.03165761	18	FALSE	Qi et al., 2018	Brain
COG8	ENSG00000213380	16	69363806	rs3759979	16	69372927	T	C	0.706294	0.006	0.0104	0.5639914	-0.235988	0.0358082	4.39E-11	-0.025425	0.0442385	0.5654772	0.6341514	0.456792	20	FALSE	Qi et al., 2018	Brain
ALG3	ENSG00000214160	3	183963712	rs706584	3	183960252	A	C	0.774476	-0.0174	0.0112	0.1202867	-0.471353	0.0535276	1.30E-18	0.036915	0.0241283	0.1260308	0.1649184	0.3666767	20	FALSE	Qi et al., 2018	Brain
UBA52	ENSG00000221983	19	18685450	rs10409392	19	18699333	A	C	0.458042	0.002	0.0097	0.836646	0.322222	0.0488456	4.20E-11	0.0062069	0.0301182	0.8367246	0.9599301	0.3329377	20	FALSE	Qi et al., 2018	Brain
ARFGAP3	ENSG00000242247	22	43223310	rs2017874	22	43303687	G	A	0.524476	-0.0278	0.0095	0.00343003	-0.410975	0.038457	1.18E-26	0.0676441	0.0239668	0.00476639	0.009671828	0.2290543	20	FALSE	Qi et al., 2018	Brain
FPGT	ENSG00000254685	1	74681846	rs11210418	1	74582677	T	C	0.496503	0.0014	0.0099	0.8875428	-0.526887	0.0490571	6.58E-27	-0.0026571	0.0187912	0.8875525	0.9859445	0.9933296	20	FALSE	Qi et al., 2018	Brain
DYNLL2	ENSG00000264364	17	56170903	rs10132	17	56168371	G	A	0.230769	-0.0072	0.0118	0.5417495	-0.336308	0.0549146	9.11E-10	0.0214089	0.0352606	0.5437425	0.619949	0.284745	4	FALSE	Qi et al., 2018	Brain
SPTBN5	ENSG00000137877.8	15	42161374	rs8041458	15	42196846	C	G	0.407343	-0.005	0.0096	0.6024829	-0.833893	0.135566	7.69E-10	0.00599597	0.0115535	0.6037773	0.6616821	0.029656	13	FALSE	GTEx Consortium	Substantia Nigra
NUDT14	ENSG00000183828.10	14	105643462	rs3759782	14	105637258	C	T	0.63986	0.0187	0.0099	0.05890672	0.617844	0.0788905	4.81E-15	0.0302665	0.0164829	0.06632273	0.05034422	0.2272025	10	FALSE	GTEx Consortium	Substantia Nigra

TRAPPC4	ENSG00000196655.5	11	118892782	rs11217124	11	118889132	G	A	0.227273	-0.0014	0.0113	0.9013994	0.649057	0.101762	1.79E-10	-0.002157	0.0174132	0.9014179	0.9084339	0.9395391	6	FALSE	GTEx Consortium	Substantia Nigra
DPM1	ENSG00000000419	20	49563248	rs2426214	20	49573810	A	G	0.323427	0.0121	0.0097	0.2122426	-0.075329	0.00896134	4.24E-17	-0.160629	0.130179	0.2172363	0.1748266	0.2255414	17	FALSE	Vosa et al., 2018	Blood
ARF5	ENSG00000004059	7	127230079	rs62483173	7	127230676	C	T	0.351399	-0.0157	0.0102	0.1237516	-0.0772763	0.00896851	6.91E-18	0.203167	0.134084	0.1297144	0.03828408	0.03268268	20	FALSE	Vosa et al., 2018	Blood
COPZ2	ENSG00000005243	17	46109462	rs17617053	17	46050669	C	T	0.0786713	0.0236	0.0165	0.1526301	0.595386	0.0247624	9.65E-128	0.0396381	0.0277621	0.1533555	0.371973	0.3712731	20	FALSE	Vosa et al., 2018	Blood
ST3GAL1	ENSG00000008513	8	134525637	rs59413766	8	134552618	A	C	0.202797	-0.0029	0.013	0.8234756	-0.291439	0.0111966	2.31E-149	0.00995062	0.0446079	0.823482	0.05970837	0.2379221	20	FALSE	Vosa et al., 2018	Blood
PGM3	ENSG00000013375	6	83887262	rs1180178	6	83903626	C	G	0.26049	-0.0146	0.014	0.2970145	-0.127642	0.00975229	3.84E-39	0.114382	0.110029	0.2985432	0.1150372	0.01773441	20	FALSE	Vosa et al., 2018	Blood
ANK1	ENSG00000029534	8	41632509	rs4736819	8	41509915	C	T	0.461538	-0.0133	0.01	0.1835183	-0.172476	0.008895	9.34E-84	0.0771122	0.0581153	0.1845476	0.602295	0.1512058	20	FALSE	Vosa et al., 2018	Blood
FUT8	ENSG00000033170	14	66044074	rs8010726	14	65932597	G	A	0.356643	0.0016	0.0102	0.875353	-0.369218	0.00956731	0	-0.0043335	0.0276262	0.875354	0.5573903	0.5443236	20	FALSE	Vosa et al., 2018	Blood
TRAPPC3	ENSG00000054116	1	36608635	rs7522400	1	36613380	A	G	0.246503	-0.0166	0.0114	0.1453538	0.141837	0.0119271	1.30E-32	-0.117036	0.0809742	0.1483609	0.09907977	0.242062	20	FALSE	Vosa et al., 2018	Blood
ST3GAL6	ENSG00000064225	3	98495562	rs14310	3	98512825	A	T	0.356643	0.024	0.01	0.01639507	0.317944	0.00963601	9.46E-239	0.075485	0.0315352	0.01668047	0.008552715	0.9349998	20	FALSE	Vosa et al., 2018	Blood
CTSA	ENSG00000064601	20	44523525	rs6017717	20	44603963	C	G	0.138112	-0.0006	0.0132	0.963745	0.133923	0.0102996	1.18E-38	-0.0044802	0.0985645	0.9637452	0.1311072	0.6176353	20	FALSE	Vosa et al., 2018	Blood
SPTB	ENSG00000070182	14	65279801	rs229621	14	65200821	T	G	0.297203	0.0025	0.0106	0.8135498	0.100339	0.00975222	7.92E-25	0.0249156	0.10567	0.8135979	0.9747503	0.8587644	20	FALSE	Vosa et al., 2018	Blood
ST6GALNAC1	ENSG00000070526	17	74630381	rs9912009	17	74694320	T	C	0.51049	0.0144	0.01	0.1498674	0.390046	0.00904993	0	0.0369188	0.0256523	0.1500947	0.1714485	0.205006	20	FALSE	Vosa et al., 2018	Blood
ST6GALNAC2	ENSG00000070731	17	74571415	rs9890158	17	74577224	G	A	0.188811	0.0166	0.0126	0.1876844	-0.571825	0.0103452	0	-0.0290299	0.022041	0.1878097	0.5239725	0.7731535	16	FALSE	Vosa et al., 2018	Blood
MGAT4A	ENSG00000071073	2	99291579	rs62158019	2	99316339	A	G	0.36014	0.0297	0.0103	0.00393289	0.167625	0.0117862	6.67E-46	0.177181	0.0626967	0.00471337	0.01109613	0.1107273	20	FALSE	Vosa et al., 2018	Blood
SEL1L	ENSG00000071537	14	81969049	rs2236179	14	81953242	G	A	0.479021	0.0036	0.0103	0.726703	0.0855711	0.00904672	3.11E-21	0.0420703	0.12045	0.726882	0.5269892	0.2700787	8	FALSE	Vosa et al., 2018	Blood
DERL2	ENSG00000072849	17	5382351	rs72827608	17	5391206	A	G	0.104895	0.0072	0.0179	0.6875114	-0.0841831	0.0144491	5.67E-09	-0.0855278	0.213138	0.6882144	0.6882144	0.7786748	11	FALSE	Vosa et al., 2018	Blood
ST6GAL1	ENSG00000073849	3	186722307	rs9814673	3	186647652	T	C	0.27972	0.0025	0.0114	0.8264177	0.294801	0.0115509	1.12E-143	0.0084803	0.0386716	0.826424	0.9661521	0.9878108	20	FALSE	Vosa et al., 2018	Blood
NSF	ENSG00000073969	17	44751432	rs62074125	17	44852612	C	A	0.225524	0.0962	0.0129	8.83E-14	-0.36447	0.0144716	5.82E-140	-0.263945	0.0369129	8.65E-13	7.68E-18	6.47E-17	20	TRUE	Vosa et al., 2018	Blood
LMAN1	ENSG00000074695	18	57011124	rs1984357	18	57022203	C	G	0.267483	0.0014	0.0108	0.8968595	0.354931	0.00951582	1.70E-304	0.00394443	0.0304286	0.8968601	0.9926172	0.9298784	20	FALSE	Vosa et al., 2018	Blood
DYNC1I2	ENSG00000077380	2	172574424	rs11886820	2	172614207	T	G	0.274476	0.0097	0.0111	0.3821869	-0.158501	0.0103371	4.59E-53	-0.0611984	0.0701448	0.3829585	0.4427406	0.3338509	20	FALSE	Vosa et al., 2018	Blood
CAPZB	ENSG00000077549	1	19738666	rs114891776	1	19648300	A	G	0.0576923	0.0304	0.0217	0.1612375	-0.316618	0.0266442	1.45E-32	-0.0960146	0.0690114	0.1641385	0.07673488	0.3557032	20	FALSE	Vosa et al., 2018	Blood
CD59	ENSG00000085063	11	33738899	rs1718066	11	33755355	G	T	0.314685	-0.0107	0.013	0.4104643	0.393696	0.00892455	0	-0.0271783	0.0330261	0.4105457	0.6138083	0.6954143	20	FALSE	Vosa et al., 2018	Blood
B4GALT1	ENSG00000086062	9	33135717	rs1411609	9	33137303	T	C	0.435315	-0.0078	0.0104	0.4532547	0.301627	0.00873849	4.45E-261	-0.0258598	0.0344879	0.4533613	0.3796294	0.4746884	20	FALSE	Vosa et al., 2018	Blood
TMED2	ENSG00000086598	12	124076097	rs4930709	12	124099093	T	C	0.263986	0.0273	0.0108	0.0114787	0.200834	0.0128932	1.05E-54	0.135933	0.0544791	0.01259083	0.04766081	0.1190123	20	FALSE	Vosa et al., 2018	Blood
ALG6	ENSG00000088035	1	63868747	rs76572066	1	63825490	G	A	0.0751748	0.0395	0.0183	0.03089131	-0.292336	0.0166807	9.17E-69	-0.135118	0.0630721	0.03217072	0.2721167	0.3102349	20	FALSE	Vosa et al., 2018	Blood
EDEM2	ENSG00000088298	20	33784544	rs7353271	20	33727431	T	C	0.190559	0.0189	0.0124	0.1274604	-0.524042	0.0134296	0	-0.0360658	0.0236803	0.1277507	0.000791981	0.5946437	20	FALSE	Vosa et al., 2018	Blood
DYNLL1	ENSG00000088986	12	120921974	rs7956286	12	121029236	A	G	0.431818	-0.0097	0.01	0.3320465	-0.0714923	0.0089381	1.26E-15	0.135679	0.1409	0.3355751	0.1384103	0.04467142	15	FALSE	Vosa et al., 2018	Blood
GANAB	ENSG00000089597	11	62403201	rs78278641	11	62413662	T	C	0.162587	-0.0069	0.0184	0.7076605	-0.20363	0.0128733	2.34E-56	0.0338851	0.0903855	0.7077388	0.08290029	0.1971911	20	FALSE	Vosa et al., 2018	Blood
SCFD1	ENSG00000092108	14	31148168	rs7144204	14	31171830	T	C	0.340909	-0.0164	0.0101	0.1044265	-0.233452	0.00893891	2.37E-150	0.07025	0.0433472	0.1050964	0.245719	0.4593023	20	FALSE	Vosa et al., 2018	Blood
SEC22C	ENSG00000093183	3	42616016	rs114958301	3	42701759	A	G	0.0402098	-0.0325	0.0249	0.1918176	-0.445079	0.0286611	2.21E-54	0.0730208	0.0561424	0.1933835	0.429853	0.9025828	12	FALSE	Vosa et al., 2018	Blood
ARCN1	ENSG00000095139	11	118458426	rs55844025	11	118446298	T	C	0.125874	0.0005	0.0145	0.9724922	-0.206456	0.0107123	9.09E-83	-0.0024218	0.0702329	0.9724922	0.2788453	0.6310105	20	FALSE	Vosa et al., 2018	Blood
PMM1	ENSG00000100417	22	41979396	rs5751129	22	42015765	C	T	0.197552	-0.0405	0.0119	0.00066562	-0.306119	0.00968428	2.70E-219	0.132301	0.0390984	0.00071489	1.72E-06	0.00159311	20	TRUE	Vosa et al., 2018	Blood
CNIH1	ENSG00000100528	14	54900901	rs10143412	14	54935759	G	A	0.148601	-0.0262	0.0134	0.05055665	0.194216	0.0114633	2.19E-64	-0.134902	0.0694534	0.05209686	0.09071276	0.372893	20	FALSE	Vosa et al., 2018	Blood
CTSZ	ENSG00000101160	20	57576271	rs163782	20	57565943	G	C	0.185315	0.0085	0.0129	0.5099505	-0.309834	0.0115957	2.78E-157	-0.027434	0.0416478	0.5100791	0.9420647	0.7725816	20	FALSE	Vosa et al., 2018	Blood
TUBB1	ENSG00000101162	20	57598009	rs463312	20	57597970	C	A	0.0332168	0.0138	0.0273	0.6132116	-0.150253	0.0199191	4.59E-14	-0.0918449	0.182101	0.6140061	0.6754224	0.4085688	3	FALSE	Vosa et al., 2018	Blood
ARFGAP1	ENSG00000101199	20	61912639	rs13042357	20	61916379	C	T	0.590909	0.0086	0.0102	0.3991517	0.113752	0.00916022	2.09E-35	0.0756033	0.0898755	0.4002356	0.7658326	0.8321593	20	FALSE	Vosa et al., 2018	Blood
UGGT2	ENSG00000102595	13	96579785	rs2389530	13	96550754	G	A	0.356643	0.0061	0.0098	0.5336467	0.296181	0.010222	1.36E-184	0.0205955	0.0330955	0.5337411	0.4934343	0.1431831	20	FALSE	Vosa et al., 2018	Blood
COG4	ENSG00000103051	16	70535969	rs7192865	16	70506907	C	T	0.479021	-0.007	0.0098	0.4750505	-0.188106	0.00909676	5.42E-95	0.037213	0.0521292	0.4753138	0.1232271	0.08698942	20	FALSE	Vosa et al., 2018	Blood
TSTA3	ENSG00000104522	8	144697503	rs77951814	8	144657152	G	C	0.0786713	0.0104	0.0186	0.5760663	-0.143774	0.0158042	9.27E-20	-0.0723356	0.129614	0.5767852	0.754771	0.6359878	20	FALSE	Vosa et al., 2018	Blood
DCTN6	ENSG00000104671	8	30027484	rs111632716	8	30081054	A	G	0.00874126	0.0589	0.0581	0.3106928	0.491123	0.0345146	6.02E-46	0.119929	0.1186	0.3119177	0.5495357	0.7272642	20	FALSE	Vosa et al., 2018	Blood
TUSC3	ENSG00000104723	8	15449441	rs17121672	8	15444576	G	A	0.027972	0.0034	0.0309	0.9123837	0.268015	0.02516	1.70E-26	0.0126859	0.115298	0.9123884	0.8512332	0.7887477	20	FALSE	Vosa et al., 2018	Blood
TUBB4A	ENSG00000104833	19	6498594	rs2071347	19	6495736	A	G	0.347902	0.0184	0.0137	0.1792507	0.100279	0.00921581	1.42E-27	0.183488	0.137655	0.1825481	0.1071711	0.9145709	20	FALSE	Vosa et al., 2018	Blood
PPP6R1	ENSG00000105063	19	55755755	rs73068668	19	55763262	A	G	0.0996503	0.0211	0.0195	0.2792297	-0.333833	0.0210892	1.94E-56	-0.0632052	0.0585487	0.2803506	0.231071	0.2383561	20	FALSE	Vosa et al., 2018	Blood
NAPA	ENSG00000105402	19	48004695	rs56162770	19	48048515	C	T	0.0227273	-0.0129	0.0315	0.6821553	-0.231804	0.0242043	9.99E-22	0.0556504	0.136015	0.6824297	0.07711796	0.5532164	7	FALSE	Vosa et al., 2018	Blood
KDELR1	ENSG00000105438	19	48890318	rs433956	19	48891537	G	A	0.456294	-0.0112	0.0101	0.2674686	0.0914499	0.00954413	9.54E-22	-0.122471	0.11118	0.2706537	0.2599942	0.8387747	20	FALSE	Vosa et al., 2018	Blood
BET1	ENSG00000105829	7	93612884	rs57401807	7	93618979	T	C	0.353147	0.0024	0.0102	0.8139805	-0.253015	0.00962778	3.27E-152	-0.0094856	0.0403154	0.8139878	0.962545	0.8721719	20	FALSE	Vosa et al., 2018	Blood
SEC23IP	ENSG00000107651	10	121677118	rs12241478	10	121667020	C	T	0.295455	-0.0658	0.011	2.21E-09	-0.0887327	0.0104301	1.78E-17	0.741553	0.151545	9.92E-07	8.76E-07	0.3173988	20	TRUE	Vosa et al., 2018	Blood
GOSR2	ENSG00000108433	17	45052743	rs2316330	17	45040032	G	T	0.519231	0.0293	0.0094	0.00182688	-0.0873752	0.00902114	3.47E-22	-0.335336	0.113016	0.0030057	0.001095263	0.3739723	20	FALSE	Vosa et al., 2018	Blood
GOSR1	ENSG00000108587	17	28829495	rs726663	17	28845774	C	T	0.475524	-0.0089	0.01	0.3734659	0.0645738	0.00907089	1.09E-12	-0.137827	0.156067	0.3771687	0.2686449	0.7328627	18	FALSE	Vosa et al., 2018	Blood
CTSC	ENSG00000109861	11	88048857	rs3758833	11	88071656	G	A	0.816434	-0.0184	0.0126	0.1442028	0.453774	0.00907008	0	-0.0405488	0.027779	0.1443737	0.3602217	0.9427195	20	FALSE	Vosa et al., 2018	Blood
PPP6R3	ENSG00000110075	11	68305494	rs7123591	11	68392358	G	A	0.132867	-0.0134	0.0157	0.3933803	0.269088	0.0160107	2.18E-63	-0.0497979	0.0584204	0.3939895	0.2488895	0.07052562	20	FALSE	Vosa et al., 2018	Blood
ST3GAL4	ENSG00000110080	11	126267887	rs2936613	11	126184045	T	G	0.153846	-0.0016	0.012	0.8939298	-0.159247	0.00979642	2.04E-59	0.0100473	0.0753573	0.8939333	0.3078163	0.1264124	20	FALSE	Vosa et al., 2018	Blood
MLEC	ENSG00000110917	12	121132169	rs695949	12	121149736	A	G	0.519231	0.0088	0.0094	0.3491856	0.204402	0.00887923	2.93E-117	0.0430525	0.0460259	0.3495837	0.1900057	0.2444744	20	FALSE	Vosa et al., 2018	Blood
COPZ1	ENSG00000111481	12	54720309	rs61614746	12	54740214	A	G	0.101399	-0.0021	0.0162	0.8968595	0.171147	0.0134159	2.85E-37	-0.0122702	0.0946604	0.8968648	0.1242202	0.03828737	20	FALSE	Vosa et al., 2018	Blood
CMAS	ENSG00000111726	12	22208858	rs6416226	12	22207944	T	C	0.844406	0.004	0.0136	0.768668	-0.0935263	0.00985815	2.37E-21	-0.0427687	0.145484	0.7687759	0.5665995	0.5963529	12	FALSE	Vosa et al., 2018	Blood
ST8SIA1	ENSG00000111728	12	22403341	rs113147032	12	22236791	G	A	0.0734266	-0.0151	0.0215	0.4824761	0.385162	0.0200428	2.67E-82	-0.0392043	0.055858	0.4827683	0.6572087	0.7756212	16	FALSE	Vosa et al., 2018	Blood
MAN1A1	ENSG00000111885	6	119584650	rs6935126	6	119678881	G	A	0.363636	-0.0171	0.0105	0.1034038	-0.314299	0.0095423	6.41E-238	0.0544069	0.0334485	0.1038254	0.1871873	0.3051899	20	FALSE	Vosa et al., 2018	Blood
GMDS	ENSG00000112699	6	1934983	rs1196399	6	2252711	T	C	0.0157343	0.0358	0.0486	0.4613501	1.47762	0.0655805	2.04E-112	0.0242282	0.0329083	0.4615894	0.1392183	0.02352618	20	FALSE	Vosa et al., 2018	Blood
MAN2A1	ENSG00000112893	5	109115196	rs77852094	5	109159723	G	A	0.0332168	0.0161	0.0246	0.5128081	-0.50469	0.0345063	1.92E-48	-0.0319008	0.0487916	0.5132295	0.2038427	0.00722117	20	FALSE	Vosa et al., 2018	Blood
ST8SIA4	ENSG00000113532	5	100192163	rs17158363	5	100002443	C	T	0.0926573	-0.0172	0.0159	0.2793587	-0.306117	0.0131747	2.01E-119	0.0561876	0.0519972	0.2798793	0.3096805	0.5721861	20	FALSE	Vosa et al., 2018	Blood
COL7A1	ENSG00000114270	3	48617103	rs1961527	3	48764954	A	G	0.81993	-0.0274	0.014	0.05033067	0.0589923	0.00928721	2.13E-10	-0.464467	0.248328	0.0614319	0.07686397	0.01093346	19	FALSE	Vosa et al., 2018	Blood
TFG	ENSG00000114354	3	100448007	rs591728	3	100433592	C	T	0.38986	-0.0059	0.0105	0.5741809	0.665248	0.011114	0	-0.0088689	0.0157843	0.5741978	0.05382908	0.00489148	20	FALSE	Vosa et al., 2018	Blood
GORASP1	ENSG00000114745	3	39144002	rs114555929	3	39139339	A	G	0.027972	0.0069	0.0362	0.8488329	-0.544168	0.0396029	5.80E-43	-0.0126799	0.06653	0.8488473	0.2609427	0.3838748	17	FALSE	Vosa et al., 2018	Blood
LMAN2L	ENSG00000114988	2	97388733	rs6730773	2	97441643	G	A	0.379371	-0.016	0.0096	0.0955807	0.151392	0.00895154	3.65E-64	-0.105686	0.0637187	0.09718993	0.009469703	0.00434141	20	FALSE	Vosa et al., 2018	Blood
MOGS	ENSG00000115275	2	74690360	rs3755448	2	74740961	T	C	0.136364	0.0079	0.0139	0.5698005	0.104857	0.00941607	8.38E-29	0.0753404	0.132734	0.5703021	0.4350982	0.1024723	20	FALSE	Vosa et al., 2018	Blood
SPTBN1	ENSG00000115306	2	54790117	rs6706372	2	54815320	C	A	0.288462	-0.0104	0.0111	0.348791	0.323816	0.00974497	4.10E-242	-0.032117	0.0342924	0.3489825	0.5506992	0.985486	20	FALSE	Vosa et al., 2018	Blood
ST3GAL5	ENSG00000115525	2	86091202	rs2280312	2	86072076	A	G	0.174825	0.0232	0.0116	0.04550026	-0.471944	0.0112528	0	-0.0491584	0.0246071	0.04574596	0.6383735	0.3811117	20	FALSE	Vosa et al., 2018	Blood
CHST10	ENSG00000115526	2	101021222	rs6755846	2	101086799	C	T	0.243007	-0.0251	0.0117	0.03192894	0.11418	0.00935522	2.92E-34	-0.219828	0.10404	0.03460869	0.0318933	0.8273516	20	FALSE	Vosa et al., 2018	Blood
EDEM3	ENSG00000116406	1	184691706	rs11808675	1	184666494	A	T	0.604895	0.0088	0.0096	0.3593173	0.197121	0.00887894	3.36E-109	0.0446425	0.0487424	0.3597265	0.2052923	0.279838	20	FALSE	Vosa et al., 2018	Blood
CAPZA1	ENSG00000116489	1	113188018	rs3790597	1	113162037	A	C	0.229021	0.0155	0.0159	0.3296383	-0.0714335	0.0129114	3.16E-08	-0.216985	0.226013	0.3370282	0.3370282			FALSE	Vosa et al., 2018	Blood
UAP1	ENSG00000117143	1	162550474	rs12748004	1	162526002	A	G	0.512238	0.0084	0.0102	0.410207	-0.185908	0.0088857	3.36E-97	-0.0451835	0.0549082	0.4105693	0.5448734	0.9786662	20	FALSE	Vosa et al., 2018	Blood
MAN1C1	ENSG00000117643	1	26028328	rs2280999	1	26108554	C	T	0.321678	0.0121	0.011	0.2713321	0.112613	0.00934454	1.91E-33	0.107448	0.0980858	0.2733212	0.2709476	0.2642181	20	FALSE	Vosa et al., 2018	Blood
DHDDS	ENSG00000117682	1	26778279	rs3816539	1	26786627	G	A	0.77972	-0.0205	0.0113	0.06965321	0.114314	0.00903393	1.07E-36	-0.179331	0.0998617	0.07252677	0.2310483	0.1245539	20	FALSE	Vosa et al., 2018	Blood
B4GALT6	ENSG00000118276	18	29234004	rs8098191	18	29278676	A	G	0.437063	0.0132	0.0095	0.1646888	-0.124717	0.00891513	1.81E-44	-0.105839	0.0765471	0.166766	0.06089281	0.6528968	20	FALSE	Vosa et al., 2018	Blood
RPN2	ENSG00000118705	20	35838417	rs1744769	20	35769592	T	C	0.185315	0.0052	0.012	0.6647726	-0.0915929	0.0103115	6.53E-19	-0.0567729	0.13117	0.6651466	0.8388255	0.8072501	20	FALSE	Vosa et al., 2018	Blood
RAD23B	ENSG00000119318	9	110069946	rs10816493	9	110100745	C	T	0.304196	0.0136	0.0109	0.2121386	0.182667	0.00966167	1.01E-79	0.0744523	0.0598011	0.2131324	0.2719046	0.3243549	20	FALSE	Vosa et al., 2018	Blood
PPP6C	ENSG00000119414	9	127930535	rs864882	9	127968109	T	C	0.323427	-0.0023	0.0102	0.821598	-0.170377	0.0105448	1.01E-58	0.0134994	0.059873	0.821615	0.004091127	0.00439318	20	FALSE	Vosa et al., 2018	Blood
SLC17A5	ENSG00000119899	6	74333490	rs472294	6	74354175	T	C	0.0839161	-0.0087	0.0181	0.630756	0.140354	0.0134302	1.46E-25	-0.0619862	0.129096	0.6311169	0.7562911	0.961857	20	FALSE	Vosa et al., 2018	Blood
ALG5	ENSG00000120697	13	37549155	rs2078022	13	37559306	A	C	0.613636	0.0168	0.0097	0.0832809	0.158879	0.00898098	4.95E-70	0.105741	0.0613447	0.08475884	0.4341148	0.5360834	20	FALSE	Vosa et al., 2018	Blood
SEC22A	ENSG00000121542	3	122956875	rs7642344	3	122947572	G	A	0.391608	0.0034	0.0102	0.7388827	-0.193497	0.00926899	8.89E-97	-0.0175713	0.0527206	0.7389147	0.4236372	0.9178857	20	FALSE	Vosa et al., 2018	Blood
B4GALT4	ENSG00000121578	3	118945264	rs9842789	3	118957370	A	G	0.167832	0.0212	0.0118	0.07239753	0.565476	0.00962295	0	0.0374905	0.0208771	0.07253093	0.12594	0.1434939	20	FALSE	Vosa et al., 2018	Blood
COPA	ENSG00000122218	1	160286126	rs7523769	1	160257980	T	A	0.426573	0.0171	0.0101	0.09044228	0.241861	0.00898009	9.01E-160	0.0707018	0.0418419	0.09107886	0.4161103	0.6778984	20	FALSE	Vosa et al., 2018	Blood
TUBA1B	ENSG00000123416	12	49523372	rs2162644	12	49548190	G	A	0.681818	-0.029	0.0101	0.00408804	-0.0869697	0.00893831	2.25E-22	0.333449	0.121083	0.00588928	0.001977113	0.1202147	20	FALSE	Vosa et al., 2018	Blood
CHST8	ENSG00000124302	19	34188637	rs55678526	19	34096417	C	A	0.309441	-0.0002	0.0113	0.9858789	0.211092	0.0113209	1.36E-77	-0.0009475	0.0535312	0.9858789	0.6092877	0.5001187	20	FALSE	Vosa et al., 2018	Blood
NAGK	ENSG00000124357	2	71299204	rs12996507	2	71298338	T	C	0.148601	0.0199	0.0129	0.1229192	-0.786761	0.0119618	0	-0.0252936	0.0164008	0.1230222	0.9224566	0.283756	20	FALSE	Vosa et al., 2018	Blood
NAPB	ENSG00000125814	20	23378642	rs6137947	20	23414630	A	C	0.0874126	0.0018	0.0171	0.916167	0.620053	0.0127258	0	0.00290298	0.0275783	0.9161672	0.2018572	0.826956	20	FALSE	Vosa et al., 2018	Blood
TBC1D20	ENSG00000125875	20	429660	rs150207	20	440443	T	C	0.501748	-0.0073	0.0104	0.4827271	0.127194	0.00891749	3.70E-46	-0.0573928	0.081864	0.4832564	0.2024743	0.2542323	20	FALSE	Vosa et al., 2018	Blood
TMEM115	ENSG00000126062	3	50394610	rs78501918	3	50369914	G	A	0.120629	0.0191	0.015	0.2028998	0.103775	0.0167704	6.09E-10	0.184052	0.147572	0.2123235	0.1951408	0.02261021	11	FALSE	Vosa et al., 2018	Blood
ST3GAL3	ENSG00000126091	1	44284163	rs7536221	1	44178846	G	C	0.423077	0.0053	0.0097	0.5847967	-0.101002	0.0090725	8.69E-29	-0.0524741	0.096153	0.5852482	0.2869576	0.162852	20	FALSE	Vosa et al., 2018	Blood

CANX	ENSG00000127022	5	179131777	rs13182141	5	179126632	C	T	0.486014	-0.0003	0.0102	0.9765362	0.197887	0.00889219	1.03E-109	-0.001516	0.0515447	0.9765362	0.7429421	0.6697677	20	FALSE	Vosa et al., 2018	Blood
TUBA4A	ENSG00000127824	2	220128662	rs11552796	2	220108300	C	T	0.0996503	-0.0027	0.0155	0.8617134	-0.0889864	0.0120535	1.55E-13	0.0303417	0.174232	0.8617514	0.3492557	0.4658161	12	FALSE	Vosa et al., 2018	Blood
SRD5A3	ENSG00000128039	4	56225769	rs36110240	4	56217015	T	C	0.164336	-0.0013	0.015	0.9309365	0.818246	0.0166845	0	-0.0015888	0.0183319	0.9309366	0.8344958	0.7324186	20	FALSE	Vosa et al., 2018	Blood
MGAT3	ENSG00000128268	22	39870774	rs1005522	22	39845898	C	A	0.263986	0.0352	0.0116	0.00240949	0.536707	0.0119212	0	0.0655851	0.0216623	0.00246493	0.06918144	0.4387746	20	FALSE	Vosa et al., 2018	Blood
COPB1	ENSG00000129083	11	14493279	rs2575859	11	14510736	C	T	0.365385	-0.0073	0.01	0.4653902	-0.524693	0.00910794	0	0.0139129	0.0190603	0.465426	0.6220089	0.4036075	20	FALSE	Vosa et al., 2018	Blood
MPDU1	ENSG00000129255	17	7491477	rs72827565	17	7469607	A	G	0.120629	0.06	0.0144	3.09E-05	0.200836	0.0155595	4.08E-38	0.298751	0.0753434	7.33E-05	3.35E-05	0.1626306	20	TRUE	Vosa et al., 2018	Blood
DAD1	ENSG00000129562	14	23045990	rs5742737	14	23056894	A	G	0.174825	-0.0052	0.0137	0.7042705	0.500686	0.0113383	0	-0.0103858	0.0273635	0.7042809	0.8430992	0.9896144	20	FALSE	Vosa et al., 2018	Blood
PRKCSH	ENSG00000130175	19	11553946	rs160841	19	11559158	G	A	0.0839161	0.0077	0.0184	0.6755975	0.193049	0.0117956	3.33E-60	0.0398862	0.0953435	0.6756974	0.1749255	0.1921693	20	FALSE	Vosa et al., 2018	Blood
MGAT1	ENSG00000131446	5	180230096	rs62405458	5	180225919	T	C	0.223776	-0.0202	0.0135	0.1345765	0.383434	0.0102667	2.80E-305	-0.0526818	0.0352364	0.1348892	0.1991334	0.8521963	20	FALSE	Vosa et al., 2018	Blood
GFPT2	ENSG00000131459	5	179754038	rs73355909	5	179794325	C	T	0.0192308	0.0018	0.0438	0.9672195	-0.203614	0.0194306	1.08E-25	-0.0088403	0.215115	0.9672197	0.8835536	0.8736913	15	FALSE	Vosa et al., 2018	Blood
ACTR10	ENSG00000131966	14	58684274	rs17832891	14	58678057	A	G	0.041958	-0.0912	0.0328	0.00542773	-0.699263	0.0276999	1.31E-140	0.130423	0.0471902	0.00571371	0.020989	0.1823135	19	FALSE	Vosa et al., 2018	Blood
DCTN4	ENSG00000132912	5	150113336	rs74600056	5	150090164	G	T	0.0699301	-0.0064	0.0224	0.775097	-0.218039	0.0242093	2.13E-19	0.0293526	0.102786	0.775207	0.9247838	0.7883395	12	FALSE	Vosa et al., 2018	Blood
COG6	ENSG00000133103	13	40297783	rs7995647	13	40216240	C	T	0.353147	-0.0113	0.0102	0.2679296	0.52513	0.00950822	0	-0.0215185	0.0194277	0.2680258	0.03811313	0.125005	20	FALSE	Vosa et al., 2018	Blood
EDEM1	ENSG00000134109	3	5245486	rs62255918	3	5263126	A	G	0.0769231	0.0714	0.0203	0.00043606	0.131666	0.013583	3.22E-22	0.542282	0.164014	0.00094532	0.003255458	0.0861726	14	FALSE	Vosa et al., 2018	Blood
NAPG	ENSG00000134265	18	10539330	rs643757	18	10545688	A	T	0.545455	0.0042	0.01	0.6744855	-0.295929	0.00898338	5.55E-238	-0.0141926	0.0337947	0.6745104	0.704584	0.5270544	20	FALSE	Vosa et al., 2018	Blood
ARF3	ENSG00000134287	12	49324310	rs833833	12	49336805	A	G	0.0821678	0.0095	0.0174	0.5850817	-0.532201	0.0125293	0	-0.0178504	0.0326971	0.5851127	0.003525027	0.06587165	20	FALSE	Vosa et al., 2018	Blood
STT3A	ENSG00000134910	11	125478358	rs516262	11	125494215	T	C	0.295455	-0.0105	0.0103	0.3080048	-0.162959	0.00965899	7.33E-64	0.0644335	0.0633214	0.3088863	0.2038441	0.5782141	20	FALSE	Vosa et al., 2018	Blood
TMED7	ENSG00000134970	5	114958947	rs400570	5	114951553	A	G	0.421329	-0.0202	0.0095	0.03347697	0.071852	0.00907909	2.49E-15	-0.281134	0.136905	0.04002519	0.01620505	0.3653903	18	FALSE	Vosa et al., 2018	Blood
OS9	ENSG00000135506	12	58101539	rs1689596	12	58053029	C	T	0.667832	0.0011	0.01	0.9124094	0.0776507	0.00893585	3.63E-18	0.014166	0.128792	0.9124164	0.5768229	0.2250125	20	FALSE	Vosa et al., 2018	Blood
DYNC1LI2	ENSG00000135720	16	66770248	rs2305311	16	66759682	A	C	0.423077	-0.0053	0.0096	0.5808913	-0.155846	0.00892396	2.70E-68	0.0340079	0.0616299	0.5810801	0.9453332	0.9058337	20	FALSE	Vosa et al., 2018	Blood
COG2	ENSG00000135775	1	230803981	rs11122572	1	230825427	G	T	0.0891608	-0.0104	0.017	0.5406934	-0.18294	0.0104221	5.64E-69	0.0568494	0.0929833	0.5409391	0.7255868	0.7406747	20	FALSE	Vosa et al., 2018	Blood
NPL	ENSG00000135838	1	182778973	rs74770891	1	182916729	C	T	0.0576923	-0.044	0.0222	0.04748126	-0.352504	0.0125497	1.35E-173	0.124821	0.0631346	0.04803415	0.4280495	0.8945015	20	FALSE	Vosa et al., 2018	Blood
COG3	ENSG00000136152	13	46074912	rs3014963	13	46080684	G	A	0.134615	-0.0224	0.015	0.13535	0.103286	0.0129034	1.20E-15	-0.216873	0.147733	0.1421028	0.1032915	0.02307997	20	FALSE	Vosa et al., 2018	Blood
KDELR2	ENSG00000136240	7	6504728	rs6796	7	6502367	C	T	0.201049	-0.0077	0.0117	0.5104612	-0.777609	0.00823323	0	0.00990215	0.0150465	0.5104715	0.000262155	0.144612	20	FALSE	Vosa et al., 2018	Blood
UGGT1	ENSG00000136731	2	128901012	rs6754450	2	128874814	T	C	0.0157343	0.0419	0.0396	0.2900186	-0.0976966	0.0130506	7.10E-14	-0.428879	0.409365	0.2947917	0.1764058	0.01151248	18	FALSE	Vosa et al., 2018	Blood
ST6GALNAC4	ENSG00000136840	9	130674741	rs1078697	9	130737250	T	C	0.248252	-0.0079	0.0118	0.503182	-0.184678	0.00957537	6.93E-83	0.0427771	0.0639334	0.503439	0.9957485	0.9939477	20	FALSE	Vosa et al., 2018	Blood
STX17	ENSG00000136874	9	102700766	rs7032333	9	102814960	A	G	0.451049	-0.0114	0.0097	0.2398916	-0.252632	0.00999945	7.83E-141	0.0451249	0.0384373	0.2403997	0.6616069	0.1341818	20	FALSE	Vosa et al., 2018	Blood
DPM2	ENSG00000136908	9	130699070	rs2274426	9	130707081	A	G	0.13986	-0.0054	0.0136	0.6913241	0.186881	0.00984321	2.24E-80	-0.0288953	0.0727894	0.6913881	0.8845962	0.9926941	20	FALSE	Vosa et al., 2018	Blood
DERL1	ENSG00000136986	8	124040033	rs11691	8	124027508	T	C	0.0576923	0.005	0.0174	0.7738395	0.208722	0.0167309	1.02E-35	0.0239553	0.0833867	0.7738978	0.4819926	0.1428149	20	FALSE	Vosa et al., 2018	Blood
DCTN3	ENSG00000137100	9	34617031	rs72737122	9	34602784	A	G	0.0909091	0.0196	0.0168	0.243345	-0.405435	0.0224735	9.35E-73	-0.0483431	0.0415235	0.2443289	0.003615671	0.5243301	20	FALSE	Vosa et al., 2018	Blood
TUBB2A	ENSG00000137267	6	3155831	rs9392465	6	3162378	A	C	0.681818	0.0051	0.0109	0.6398633	-0.596583	0.00813605	0	-0.0085487	0.0182711	0.6398701	0.6785826	0.2155316	20	FALSE	Vosa et al., 2018	Blood
TUBB2B	ENSG00000137285	6	3228229	rs9328145	6	3144908	A	G	0.697552	0.0137	0.011	0.2129647	0.154385	0.00889662	1.87E-67	0.0887393	0.0714338	0.214141	0.3289312	0.3902203	20	FALSE	Vosa et al., 2018	Blood
SPTBN5	ENSG00000137877	15	42163310	rs7166358	15	42191067	A	C	0.409091	-0.0046	0.0096	0.6318201	-0.363834	0.00952027	0	0.0126431	0.0263877	0.6318469	0.000627247	0.02626394	20	FALSE	Vosa et al., 2018	Blood
RAB1A	ENSG00000138069	2	65327537	rs10185243	2	65355744	G	T	0.346154	-0.0222	0.0101	0.02794769	-0.0581237	0.00922129	2.92E-10	0.381944	0.18403	0.03794488	0.05921797	0.4412455	15	FALSE	Vosa et al., 2018	Blood
PREB	ENSG00000138073	2	27355583	rs2289358	2	27355991	T	G	0.181818	0.0175	0.0129	0.1749118	-0.172278	0.0109488	8.73E-56	-0.10158	0.0751568	0.1765112	0.12902	0.2513271	20	FALSE	Vosa et al., 2018	Blood
ACTR1A	ENSG00000138107	10	104250734	rs13287	10	104240438	G	A	0.300699	0.0133	0.0099	0.1791314	-0.173009	0.00948452	2.43E-74	-0.0768746	0.0573774	0.1803085	0.007321339	0.1209438	20	FALSE	Vosa et al., 2018	Blood
SEC31A	ENSG00000138674	4	83781066	rs13118857	4	83808854	T	C	0.263986	0.0165	0.0125	0.186835	-0.073822	0.0105388	2.47E-12	-0.223511	0.172306	0.1945729	0.09605367	0.5611395	19	FALSE	Vosa et al., 2018	Blood
USO1	ENSG00000138768	4	76692572	rs10014281	4	76644120	A	C	0.153846	-0.0317	0.0142	0.02558891	-0.269376	0.0144694	2.34E-77	0.117679	0.053092	0.02665619	0.1884744	0.1163355	20	FALSE	Vosa et al., 2018	Blood
SEC24B	ENSG00000138802	4	110408490	rs68041341	4	110453021	T	A	0.15035	-0.0155	0.0159	0.3296383	0.0788864	0.0122093	1.04E-10	-0.196485	0.203837	0.3350794	0.3441807	0.7164047	12	FALSE	Vosa et al., 2018	Blood
RNF185	ENSG00000138942	22	31579583	rs71324184	22	31752061	G	C	0.27972	0.0089	0.0103	0.3875452	0.424218	0.00908605	0	0.0209798	0.0242841	0.3876265	0.2486556	0.2398081	20	FALSE	Vosa et al., 2018	Blood
ALG10	ENSG00000139133	12	34178922	rs11494828	12	34225870	A	T	0.384615	0.0163	0.0105	0.1205711	-0.163685	0.0112387	4.73E-48	-0.0995817	0.0645111	0.1226763	0.1662557	0.00175069	7	FALSE	Vosa et al., 2018	Blood
PMM2	ENSG00000140650	16	8912934	rs2447927	16	8943277	A	G	0.0367133	-0.0088	0.0241	0.715003	0.425791	0.0178184	3.35E-126	-0.0206674	0.0566071	0.7150348	0.1192276	0.6870214	20	FALSE	Vosa et al., 2018	Blood
ASGR1	ENSG00000141505	17	7079816	rs12945886	17	7073014	G	A	0.20979	-0.0139	0.0156	0.3729154	-0.147881	0.0096687	8.28E-53	0.0939946	0.105669	0.373725	0.05045095	0.2022442	16	FALSE	Vosa et al., 2018	Blood
CSNK1D	ENSG00000141551	17	80214253	rs73366295	17	80249978	T	G	0.0297203	-0.0045	0.0279	0.8718647	-0.872033	0.0300005	9.31E-186	0.00516036	0.0319947	0.8718667	0.1718995	0.5237138	20	FALSE	Vosa et al., 2018	Blood
ARF1	ENSG00000143761	1	228278636	rs964941	1	228193857	G	A	0.444056	-0.0213	0.0096	0.02650374	0.0667856	0.00893501	7.74E-14	-0.318931	0.149943	0.03341852	0.02967317	0.660646	20	FALSE	Vosa et al., 2018	Blood
GMPPA	ENSG00000144591	2	220367649	rs73087210	2	220336723	T	C	0.122378	-0.0077	0.017	0.6505911	-0.0744385	0.0123112	1.48E-09	0.103441	0.229016	0.6515027	0.6656351	0.5958562	14	FALSE	Vosa et al., 2018	Blood
DYNC1LI1	ENSG00000144635	3	32589914	rs9852881	3	32621359	A	C	0.402098	0.0018	0.0122	0.882705	0.49204	0.00889868	0	0.00365824	0.0247948	0.8827054	0.6834253	0.5241697	20	FALSE	Vosa et al., 2018	Blood
6-Mar	ENSG00000145495	5	10394653	rs2640712	5	10382248	A	G	0.0944056	0.0288	0.0156	0.06486987	-0.514658	0.00873889	0	-0.0559595	0.0303263	0.06500155	0.007652806	0.00704033	20	FALSE	Vosa et al., 2018	Blood
SEC16A	ENSG00000148396	9	139353345	rs4378078	9	139375962	G	A	0.458042	0.0263	0.0096	0.00615171	-0.311328	0.00895851	1.28E-264	-0.0844768	0.0309313	0.00631213	0.001642336	0.2779525	20	FALSE	Vosa et al., 2018	Blood
ST8SIA6	ENSG00000148488	10	17428355	rs7077568	10	17498867	C	T	0.173077	-0.0181	0.0143	0.2056082	0.112032	0.0113565	5.90E-23	-0.161561	0.128689	0.2093181	0.2018268	0.454324	20	FALSE	Vosa et al., 2018	Blood
FUOM	ENSG00000148803	10	135170093	rs41283339	10	135209850	T	C	0.0314685	-0.0156	0.0317	0.6226391	-0.4752	0.0345228	4.15E-43	0.0328283	0.0667514	0.6228612	0.8336469	0.8040401	20	FALSE	Vosa et al., 2018	Blood
ARFGAP2	ENSG00000149182	11	47192262	rs189817094	11	47044040	T	C	0.0157343	0.0221	0.038	0.5608503	0.662879	0.0479749	2.01E-43	0.0333394	0.0573764	0.561197	0.7616957	0.6292681	20	FALSE	Vosa et al., 2018	Blood
SEC24D	ENSG00000150961	4	119701908	rs115294251	4	119772833	T	C	0.0384615	0.0178	0.0209	0.3943947	0.215105	0.0271077	2.10E-15	0.0827504	0.09772	0.3971004	0.1262355	0.2947634	20	FALSE	Vosa et al., 2018	Blood
UBC	ENSG00000150991	12	125399032	rs4617684	12	125406966	A	G	0.00174825	0.072	0.1032	0.4853808	-0.0842798	0.011017	2.01E-14	-0.854298	1.22958	0.4871863	0.7652337			FALSE	Vosa et al., 2018	Blood
NGLY1	ENSG00000151092	3	25795982	rs4256092	3	25649565	A	T	0.291958	0.029	0.0109	0.0078013	-0.0946383	0.0094359	1.13E-23	-0.30643	0.119159	0.01012272	0.003268072	0.1970295	20	FALSE	Vosa et al., 2018	Blood
ANK3	ENSG00000151150	10	62140703	rs1904418	10	62488381	G	A	0.328671	-0.0074	0.0108	0.4932271	-0.515119	0.00905938	0	0.0143656	0.0209675	0.4932585	0.1177331	0.1312651	20	FALSE	Vosa et al., 2018	Blood
MGAT5	ENSG00000152127	2	135044873	rs11899700	2	135040051	C	T	0.145105	0.014	0.0156	0.3694864	0.114828	0.0107116	8.20E-27	0.121921	0.13633	0.3711574	0.5029446	0.09535569	20	FALSE	Vosa et al., 2018	Blood
SAR1B	ENSG00000152700	5	133960897	rs246347	5	134096294	T	C	0.176573	0.0248	0.0131	0.05834061	-0.331952	0.0151899	7.22E-106	-0.0747097	0.0396114	0.05928606	7.83E-08	0.04403147	20	TRUE	Vosa et al., 2018	Blood
MIA3	ENSG00000154305	1	222816391	rs2378585	1	222781822	A	G	0.188811	0.0062	0.0132	0.6385715	-0.0782061	0.0117862	3.24E-11	-0.0792777	0.169207	0.6394095	0.6394095	0.8226708	13	FALSE	Vosa et al., 2018	Blood
SEC13	ENSG00000157020	3	10348838	rs696221	3	10342876	C	T	0.456294	-0.0027	0.0096	0.7785187	0.502759	0.00972951	0	-0.0053704	0.0190949	0.7785219	0.2353582	0.06292348	20	FALSE	Vosa et al., 2018	Blood
ST3GAL2	ENSG00000157350	16	70443239	rs12934021	16	70559068	A	G	0.0367133	-0.0038	0.028	0.8920472	0.308789	0.036608	3.31E-17	-0.0123062	0.0906887	0.892061	0.6171383	0.7103354	19	FALSE	Vosa et al., 2018	Blood
B4GALT5	ENSG00000158470	20	48289948	rs2273088	20	48273459	A	G	0.447552	-0.0259	0.0094	0.00586349	0.100897	0.00905604	7.89E-29	-0.256697	0.095971	0.00747872	0.07460562	0.7282789	20	FALSE	Vosa et al., 2018	Blood
DYNC1I1	ENSG00000158560	7	95570750	rs7801111	7	95401417	A	G	0.277972	0.025	0.0112	0.02560552	-0.11007	0.0108346	3.02E-24	-0.227127	0.10418	0.02924711	0.001552761	0.01474494	20	FALSE	Vosa et al., 2018	Blood
COPG2	ENSG00000158623	7	130249843	rs138265226	7	130273974	C	T	0.16958	-0.027	0.0134	0.04391247	-0.479426	0.0100026	0	0.0563173	0.0279748	0.04409914	0.1360468	0.7034579	20	FALSE	Vosa et al., 2018	Blood
B4GALT3	ENSG00000158850	1	161144193	rs11585858	1	161156033	A	C	0.232517	-0.0246	0.0113	0.02948123	-0.168387	0.0105168	1.07E-57	0.146092	0.0677247	0.03099453	0.009751314	0.00790217	20	FALSE	Vosa et al., 2018	Blood
ALG8	ENSG00000159063	11	77831344	rs550458	11	77855254	T	C	0.178322	0.0037	0.0129	0.7742488	0.18121	0.0107715	1.66E-63	0.0204183	0.0711986	0.7742808	0.2655966	0.4083499	20	FALSE	Vosa et al., 2018	Blood
AMFR	ENSG00000159461	16	56427407	rs7205545	16	56381312	A	G	0.356643	-0.0352	0.0109	0.00124069	0.569262	0.00829146	0	-0.0618345	0.0191688	0.00125625	0.06593292	0.1968771	20	FALSE	Vosa et al., 2018	Blood
GNE	ENSG00000159921	9	36245745	rs10114702	9	36262120	T	C	0.5	0.0074	0.01	0.4593	-0.17935	0.008986	1.26E-88	-0.0412602	0.0557953	0.4596084	0.3094149	0.06844352	20	FALSE	Vosa et al., 2018	Blood
TRAPPC10	ENSG00000160218	21	45479316	rs2838502	21	45570988	G	T	0.195804	0.0121	0.0118	0.3051633	-0.114714	0.00927118	3.65E-35	-0.10548	0.103217	0.3068186	0.5937488	0.0889477	20	FALSE	Vosa et al., 2018	Blood
ST6GALNAC6	ENSG00000160408	9	130657643	rs10819317	9	130668957	G	A	0.164336	0.0027	0.0138	0.8448824	-0.636361	0.0102597	0	-0.0042429	0.0216859	0.8448832	0.656766	0.8838159	20	FALSE	Vosa et al., 2018	Blood
MGAT4B	ENSG00000161013	5	179229274	rs73351610	5	179232603	C	T	0.013986	0.0017	0.0337	0.9597677	-0.525689	0.0246573	7.44E-101	-0.0032339	0.0641066	0.9597678	0.7393667	0.1513287	20	FALSE	Vosa et al., 2018	Blood
ASGR2	ENSG00000161944	17	7011830	rs552653	17	7015711	T	C	0.291958	-0.0192	0.0109	0.07815924	0.718962	0.00900485	0	-0.0267052	0.0151644	0.07823174	0.02456416	0.17994	20	FALSE	Vosa et al., 2018	Blood
AMDHD2	ENSG00000162066	16	2575890	rs4278751	16	2471514	G	A	0.347902	0.0068	0.0111	0.5401325	0.150506	0.0118203	3.89E-37	0.0451809	0.0738366	0.5406008	0.5013309	0.5853641	19	FALSE	Vosa et al., 2018	Blood
STX5	ENSG00000162236	11	62586964	rs474209	11	62605067	C	T	0.618881	-0.0371	0.0103	0.00031585	-0.0601216	0.00897605	2.11E-11	0.617083	0.19452	0.00151222	0.005093339	0.2881297	7	FALSE	Vosa et al., 2018	Blood
TGFA	ENSG00000163235	2	70727868	rs113971512	2	70774454	T	C	0.108392	0.0104	0.0151	0.4909858	0.352788	0.0159054	5.31E-109	0.0294795	0.0428226	0.4911947	0.6531362	0.2867675	20	FALSE	Vosa et al., 2018	Blood
SPTA1	ENSG00000163554	1	158618383	rs12405509	1	158515051	A	G	0.230769	-0.0128	0.0113	0.257322	0.0844933	0.0106536	2.17E-15	-0.151491	0.135096	0.262133	0.3127074	0.4142021	19	FALSE	Vosa et al., 2018	Blood
RPN1	ENSG00000163902	3	128369367	rs34690832	3	128375024	A	C	0.26049	0.0289	0.0116	0.01272482	0.268381	0.0106091	3.41E-141	0.107683	0.0434312	0.01316093	0.06066131	0.9370748	20	FALSE	Vosa et al., 2018	Blood
RFT1	ENSG00000163933	3	53143488	rs2336725	3	53118739	T	C	0.583916	-0.0259	0.0093	0.00535366	-0.306549	0.00876797	8.40E-268	0.0844889	0.0304338	0.00550064	4.67E-06	0.02781155	20	TRUE	Vosa et al., 2018	Blood
SLC35A1	ENSG00000164414	6	88201197	rs2754245	6	88382859	G	A	0.473776	0.0159	0.0119	0.1815053	-0.194887	0.00885908	2.97E-107	-0.0815856	0.0611734	0.1823098	0.022909	0.04537371	20	FALSE	Vosa et al., 2018	Blood
COG5	ENSG00000164597	7	107023574	rs10264412	7	107183877	A	G	0.262238	-0.0109	0.0111	0.3261087	0.912896	0.00897809	0	-0.01194	0.0121597	0.3261313	0.612918	0.05008743	20	FALSE	Vosa et al., 2018	Blood
VCP	ENSG00000165280	9	35064653	rs684562	9	35060302	C	T	0.715035	0.015	0.0104	0.1492156	0.307809	0.0088298	2.98E-266	0.0487316	0.0338161	0.1495636	0.6733144	0.6692789	20	FALSE	Vosa et al., 2018	Blood
TMED3	ENSG00000166557	15	79653869	rs117679487	15	79600248	C	T	0.048951	0.0274	0.0313	0.3813566	0.463363	0.0255815	2.51E-73	0.0591329	0.0676284	0.381912	0.06633355	0.348001	20	FALSE	Vosa et al., 2018	Blood
DCTN5	ENSG00000166847	16	23666954	rs34514	16	23670597	A	G	0.195804	0.0158	0.0116	0.1731761	0.989109	0.00810945	0	0.015974	0.0117285	0.1732029	0.5277559	0.8465008	20	FALSE	Vosa et al., 2018	Blood
PDIA3	ENSG00000167004	15	44052033	rs4924722	15	44056735	G	A	0.316434	-0.0034	0.0105	0.7460822	-0.109172	0.00895382	3.40E-34	0.0311435	0.0962123	0.7461687	0.962871	0.9220995	20	FALSE	Vosa et al., 2018	Blood
ENGASE	ENSG00000167280	17	77077851	rs35062036	17	77078660	A	G	0.0559441	0.0199	0.0205	0.3316819	-0.796446	0.018289	0	-0.024986	0.0257457	0.331802	0.9437022	0.5096446	20	FALSE	Vosa et al., 2018	Blood
MVD	ENSG00000167508	16	88723956	rs11649119	16	88720074	G	A	0.197552	0.0012	0.0127	0.9247214	-0.249312	0.0125642	1.27E-87	-0.0048132	0.0509407	0.9247222	0.5600675	0.3543652	20	FALSE	Vosa et al., 2018	Blood
TRAPPC2L	ENSG00000167515	16	88925861	rs11076728	16	88925567	G	T	0.344406	0.0274	0.0103	0.00780956	-0.234254	0.00883682	7.67E-155	-0.116967	0.0441902	0.00812334	0.03058658	0.8767766	20	FALSE	Vosa et al., 2018	Blood
TUBA1A	ENSG00000167552	12	49580843	rs11168868	12	49515130	C	T	0.0297203	-0.0293	0.0387	0.4489864	0.467466	0.0446874	1.31E-25	-0.0626784	0.0830034	0.4501705	0.0410797	0.4141611	17	FALSE	Vosa et al., 2018	Blood
TRAPPC9	ENSG00000167632	8	141105632	rs3735799	8	141467960	G	A	0.484266	-0.0021	0.0097	0.828602	-0.349763	0.00866644	0	0.00600406	0.0277335	0.8286045	0.3665005	0.2006222	20	FALSE	Vosa et al., 2018	Blood
MGAT2	ENSG00000168282	14	50088843	rs11157696	14	50105746	C	G	0.237762	0.0031	0.0111	0.7800305	-0.0961053	0.00897108	8.86E-27	-0.0322563	0.115538	0.7801033	0.5566132	0.3350294	20	FALSE	Vosa et al., 2018	Blood

ARF4	ENSG00000168374	3	57570518	rs149547076	3	57557444	C	T	0.0104895	0.1544	0.0626	0.01364584	1.37819	0.0692642	4.27E-88	0.112031	0.0457695	0.01437632	0.1238314	0.2815744	9	FALSE	Vosa et al., 2018	Blood
COG7	ENSG00000168434	16	23432157	rs181835	16	23397113	C	T	0.18007	0.0157	0.0144	0.2755908	0.150655	0.00945543	3.73E-57	0.104211	0.0958059	0.2767125	0.3848184	0.9758649	20	FALSE	Vosa et al., 2018	Blood
LMAN2	ENSG00000169223	5	176768708	rs34316179	5	176779689	A	G	0.309441	-0.0276	0.0102	0.00681232	0.132278	0.00984051	3.42E-41	-0.208651	0.0786571	0.00798574	0.1429957	0.01541429	20	FALSE	Vosa et al., 2018	Blood
TRAPPC1	ENSG00000170043	17	7834552	rs9894029	17	7835451	G	A	0.0174825	0.0422	0.0386	0.2742778	-0.174708	0.018286	1.25E-21	-0.241546	0.222382	0.277401	0.1123568	0.6126376	7	FALSE	Vosa et al., 2018	Blood
NANP	ENSG00000170191	20	25599191	rs6076369	20	25576783	A	G	0.417832	0.0093	0.0098	0.342631	-0.449587	0.00950741	0	-0.0206856	0.0218022	0.3427282	0.001456846	0.03188156	20	FALSE	Vosa et al., 2018	Blood
GLB1	ENSG00000170266	3	33088411	rs11717007	3	33070666	C	A	0.243007	0.0157	0.0112	0.1609792	0.227156	0.0105318	3.54E-103	0.0691154	0.0494093	0.1618626	0.08413435	0.713218	20	FALSE	Vosa et al., 2018	Blood
TMED10	ENSG00000170348	14	75620753	rs7146787	14	75696922	G	A	0.237762	0.0112	0.0116	0.3342858	-0.103433	0.00961259	5.31E-27	-0.108282	0.1126	0.3362236	0.2031045	0.2055898	18	FALSE	Vosa et al., 2018	Blood
RNF139	ENSG00000170881	8	125493567	rs111419470	8	125471651	T	C	0.25	0.0061	0.0118	0.6051917	0.11726	0.0124651	5.10E-21	0.0520214	0.100783	0.6057354	0.2341398	0.06370197	12	FALSE	Vosa et al., 2018	Blood
DPAGT1	ENSG00000172269	11	118973127	rs649870	11	118971251	A	G	0.555944	0.0432	0.0095	5.43E-06	-0.181335	0.00892169	7.70E-92	-0.238233	0.0536843	9.09E-06	1.15E-05	0.5134361	20	TRUE	Vosa et al., 2018	Blood
ALG14	ENSG00000172339	1	95493399	rs60555923	1	95508584	C	A	0.0472028	-0.0367	0.0265	0.1660813	-0.148817	0.0251985	3.51E-09	0.246612	0.182902	0.1775524	0.1775524	0.5383705	8	FALSE	Vosa et al., 2018	Blood
MANEA	ENSG00000172469	6	96041376	rs6940020	6	96035916	C	T	0.403846	-0.0092	0.0098	0.347846	0.643304	0.00867244	0	-0.0143012	0.0152351	0.3478846	0.01204437	0.00992494	20	FALSE	Vosa et al., 2018	Blood
GOLGB1	ENSG00000173230	3	121425324	rs9968051	3	121384081	G	C	0.124126	0.0406	0.0135	0.00263486	-0.828847	0.0116744	0	-0.0489837	0.0163023	0.00265832	0.002603683	0.00193855	20	FALSE	Vosa et al., 2018	Blood
CNIH2	ENSG00000174871	11	66049216	rs2017969	11	66039032	A	G	0.18007	-0.0133	0.0125	0.2873287	0.133811	0.0113366	3.75E-32	-0.0993943	0.0937944	0.2892796	0.2443188	0.2189593	20	FALSE	Vosa et al., 2018	Blood
ALG10B	ENSG00000175548	12	38714082	rs12313851	12	38721324	T	A	0.0244755	0.04	0.0339	0.2380237	-0.0966681	0.0103351	8.49E-21	-0.413787	0.353464	0.2417344	0.1530261	0.5320902	20	FALSE	Vosa et al., 2018	Blood
TUBB6	ENSG00000176014	18	12325993	rs7244254	18	12317836	A	G	0.146853	-0.0075	0.0145	0.6049877	0.7473	0.00935293	0	-0.0100361	0.0194036	0.6049952	0.6975407	0.5065391	20	FALSE	Vosa et al., 2018	Blood
MAN1B1	ENSG00000177239	9	139992507	rs72763269	9	140005916	A	G	0.398601	0.0089	0.0104	0.3921254	-0.48652	0.010113	0	-0.0182932	0.0213797	0.3922003	0.4324886	0.4614805	20	FALSE	Vosa et al., 2018	Blood
BET1L	ENSG00000177951	11	187606	rs4980320	11	198986	T	C	0.571678	0.0003	0.01	0.9760671	-0.383589	0.00860985	0	-0.0007821	0.0260696	0.9760671	0.2174014	0.01276913	20	FALSE	Vosa et al., 2018	Blood
MPI	ENSG00000178802	15	75187072	rs1130741	15	75189930	G	A	0.531469	0.0323	0.0094	0.00059	0.327392	0.00886104	8.02E-299	0.0986584	0.0288356	0.00062298	0.001955724	0.04243735	20	FALSE	Vosa et al., 2018	Blood
DPM3	ENSG00000179085	1	155112719	rs10157801	1	155120012	G	A	0.409091	-0.0417	0.0096	1.40E-05	-0.098351	0.00894002	3.77E-28	0.423992	0.104943	5.34E-05	8.72E-06	0.00092075	20	TRUE	Vosa et al., 2018	Blood
FUCA1	ENSG00000179163	1	24183175	rs2501431	1	24201643	G	A	0.412587	0.0106	0.0103	0.3034204	-0.497907	0.00875028	0	-0.0212891	0.02069	0.3034994	0.4247298	0.6587864	20	FALSE	Vosa et al., 2018	Blood
CALR	ENSG00000179218	19	13052347	rs2965215	19	13071832	A	G	0.283217	0.0091	0.0104	0.3815739	-0.0600901	0.0103145	5.68E-09	-0.151439	0.175015	0.3868771	0.4623146	0.9476065	9	FALSE	Vosa et al., 2018	Blood
MCFD2	ENSG00000180398	2	47149001	rs34653836	2	47150575	C	T	0.176573	0.0018	0.0137	0.8954693	-0.495594	0.0120552	0	-0.003632	0.0276437	0.8954698	0.1444463	0.2162306	20	FALSE	Vosa et al., 2018	Blood
COPG1	ENSG00000181789	3	128982531	rs6762578	3	128992047	G	A	0.208042	0.0132	0.0121	0.2753129	0.141587	0.0112508	2.57E-36	0.0932287	0.0857801	0.2771105	0.3789393	0.5581697	18	FALSE	Vosa et al., 2018	Blood
SLC35C1	ENSG00000181830	11	45830094	rs3808976	11	45827350	G	A	0.25	-0.0097	0.012	0.4188987	0.351328	0.00992588	2.03E-274	-0.0276095	0.034165	0.41902	0.1118616	0.7176128	20	FALSE	Vosa et al., 2018	Blood
TRAPPC6B	ENSG00000182400	14	39628375	rs1950509	14	39549811	C	T	0.0716783	0.0038	0.019	0.8414806	-0.231861	0.0166729	5.79E-44	-0.0163892	0.0819543	0.8414968	0.2539145	0.9614702	20	FALSE	Vosa et al., 2018	Blood
ALG12	ENSG00000182858	22	50304486	rs11705677	22	50258867	C	T	0.232517	0.0183	0.0144	0.2037879	0.182645	0.00899171	9.95E-92	0.100194	0.0789956	0.2046718	0.08918167	0.712009	20	FALSE	Vosa et al., 2018	Blood
NUDT14	ENSG00000183828	14	105643467	rs11625865	14	105644421	A	G	0.631119	0.0187	0.0098	0.05637012	0.235574	0.00894509	7.52E-153	0.0793807	0.0417096	0.05701783	0.0646937	0.2726622	20	FALSE	Vosa et al., 2018	Blood
ST6GALNAC3	ENSG00000184005	1	76820345	rs1251496	1	76517127	G	C	0.195804	0.0084	0.0125	0.5015837	0.483472	0.0135863	2.37E-277	0.0173743	0.0258592	0.50166	0.2754011	0.04356683	20	FALSE	Vosa et al., 2018	Blood
COPB2	ENSG00000184432	3	139091508	rs7375091	3	139090389	A	T	0.625874	0.0122	0.0103	0.2362286	-0.152809	0.00939589	1.79E-59	-0.0798382	0.0675829	0.2374688	0.3142642	0.4131157	20	FALSE	Vosa et al., 2018	Blood
TMED9	ENSG00000184840	5	177021142	rs186803	5	177016095	C	T	0.548951	0.0133	0.0097	0.1703332	0.475321	0.00848082	0	0.0279811	0.0204134	0.170461	0.05755959	0.09279697	20	FALSE	Vosa et al., 2018	Blood
CD55	ENSG00000196352	1	207514582	rs891376	1	207491489	T	C	0.277972	0.0007	0.0106	0.9473478	-0.701086	0.00835422	0	-0.0009985	0.0151194	0.9473478	0.4172905	0.515627	20	FALSE	Vosa et al., 2018	Blood
MAN2A2	ENSG00000196547	15	91455631	rs12904445	15	91480010	A	C	0.0734266	-0.0244	0.0167	0.1439941	-0.288738	0.0107971	1.52E-157	0.0845058	0.0579242	0.1445921	0.2723639	0.2940103	20	FALSE	Vosa et al., 2018	Blood
TRAPPC4	ENSG00000196655	11	118892651	rs569	11	118894287	G	A	0.199301	-0.0001	0.0119	0.9932952	1.05995	0.0104438	0	-9.43E-05	0.011227	0.9932952	0.1706838	0.7817763	20	FALSE	Vosa et al., 2018	Blood
DYNC1H1	ENSG00000197102	14	102473997	rs1190607	14	102511597	G	A	0.18007	-0.002	0.0116	0.8631122	0.0620256	0.00991109	3.89E-10	-0.0322448	0.187091	0.8631636	0.5106168	0.2254994	20	FALSE	Vosa et al., 2018	Blood
SERPINA1	ENSG00000197249	14	94850057	rs8008466	14	94862149	T	C	0.56993	-0.0062	0.0098	0.5269602	-0.465361	0.0084822	0	0.013323	0.0210603	0.5269877	0.05481184	0.0559753	20	FALSE	Vosa et al., 2018	Blood
SPTAN1	ENSG00000197694	9	131355403	rs7036322	9	131417140	A	C	0.129371	-0.0031	0.0159	0.8454176	-0.0555623	0.00930038	2.31E-09	0.0557932	0.286318	0.8454988	0.8778587	0.6220713	6	FALSE	Vosa et al., 2018	Blood
MAN1A2	ENSG00000198162	1	117990782	rs946520	1	117901462	G	A	0.253497	0.024	0.0117	0.04023949	-0.235792	0.00972995	9.85E-130	-0.101785	0.0497975	0.04095625	0.05443525	0.01866513	20	FALSE	Vosa et al., 2018	Blood
GFPT1	ENSG00000198380	2	69580643	rs6720415	2	69614161	A	G	0.403846	-0.0425	0.0096	9.55E-06	0.491367	0.00866441	0	-0.0864934	0.0195968	1.02E-05	6.90E-05	0.3632576	20	TRUE	Vosa et al., 2018	Blood
F5	ENSG00000198734	1	169519615	rs2227245	1	169540080	T	C	0.276224	0.0157	0.0104	0.1311416	-0.609663	0.00906692	0	-0.025752	0.0170629	0.1312387	0.6722065	0.8429823	15	FALSE	Vosa et al., 2018	Blood
CAPZA2	ENSG00000198898	7	116505219	rs9791721	7	116524512	C	G	0.244755	0.0136	0.0114	0.2328762	-0.290958	0.00941951	1.68E-209	-0.0467421	0.0392101	0.2332245	0.2892146	0.8146648	20	FALSE	Vosa et al., 2018	Blood
RNF5	ENSG00000204308	6	32149030	rs62402717	6	32080838	A	G	0.0402098	0.0256	0.027	0.343054	0.243036	0.0233387	2.15E-25	0.105334	0.111554	0.3450459	0.000114743	0.02093985	20	TRUE	Vosa et al., 2018	Blood
NEU1	ENSG00000204386	6	31828059	rs3101018	6	31705864	T	C	0.0769231	0.0289	0.0163	0.07622769	-0.206749	0.0233525	8.49E-19	-0.139783	0.0804048	0.08212492	0.002918568	0.05836884	20	FALSE	Vosa et al., 2018	Blood
DCTN1	ENSG00000204843	2	74603747	rs13021268	2	74553663	T	C	0.0297203	0.0005	0.0363	0.9890102	-0.460306	0.0345311	1.54E-40	-0.0010862	0.0788606	0.9890102	0.9605591	0.8954472	10	FALSE	Vosa et al., 2018	Blood
TRIM13	ENSG00000204977	13	50582320	rs73196708	13	50505614	T	C	0.0367133	0.0322	0.03	0.2831216	-0.215675	0.0189206	4.23E-30	-0.149298	0.139713	0.285247	0.3432425	0.7349092	20	FALSE	Vosa et al., 2018	Blood
ANKRD28	ENSG00000206560	3	15805010	rs7630642	3	15727823	G	A	0.0979021	-0.02	0.0178	0.2611847	0.463952	0.00975718	0	-0.0431079	0.0383767	0.2613178	0.6981483	0.1381479	20	FALSE	Vosa et al., 2018	Blood
ALG3	ENSG00000214160	3	183963712	rs843332	3	183957467	T	G	0.223776	0.0193	0.0113	0.08764291	0.221111	0.0114437	3.54E-83	0.0872865	0.0513048	0.08888095	0.2010637	0.6757517	20	FALSE	Vosa et al., 2018	Blood
UBA52	ENSG00000221983	19	18685450	rs12608925	19	18687533	T	G	0.557692	-0.0014	0.0095	0.8828412	0.275529	0.00877334	1.71E-216	-0.0050811	0.0344795	0.8828425	0.2893193	0.2874441	20	FALSE	Vosa et al., 2018	Blood
RNF103	ENSG00000239305	2	86840752	rs10201590	2	86816501	G	A	0.512238	0.0121	0.0099	0.2216236	0.114431	0.00894046	1.66E-37	0.105741	0.0869088	0.2237231	0.3405456	0.565255	20	FALSE	Vosa et al., 2018	Blood
ARFGAP3	ENSG00000242247	22	43223310	rs5751391	22	43261499	A	G	0.482517	0.0268	0.0096	0.00524373	-0.21107	0.00938908	6.46E-112	-0.126972	0.0458318	0.00559897	0.03697477	0.1209283	20	FALSE	Vosa et al., 2018	Blood
DDOST	ENSG00000244038	1	20983135	rs2298298	1	20964328	A	G	0.125874	-0.0119	0.0143	0.4053142	-0.351533	0.0116739	3.31E-199	0.0338517	0.0406945	0.4054935	0.4069786	0.4581705	20	FALSE	Vosa et al., 2018	Blood
ALG11	ENSG00000253710	13	52595167	rs9535832	13	52580329	C	T	0.589161	-0.009	0.0095	0.3434511	-0.194915	0.00885995	2.91E-107	0.0461739	0.0487842	0.3438981	0.1443952	0.2439401	20	FALSE	Vosa et al., 2018	Blood
FPGT	ENSG00000254685	1	74681846	rs533371	1	74687012	A	G	0.524476	0.0001	0.0099	0.9919407	0.331565	0.00872999	0	0.0003016	0.0298584	0.9919407	0.998842	0.9517342	20	FALSE	Vosa et al., 2018	Blood
DYNLL2	ENSG00000264364	17	56170903	rs9903806	17	56149521	G	A	0.318182	-0.0014	0.0109	0.8978005	0.12585	0.0111694	1.90E-29	-0.0111243	0.0866165	0.8978071	0.5156625	0.4181239	20	FALSE	Vosa et al., 2018	Blood

CASPASE MEDIATED CLEAVAGE OF CYTOSKELETAL PROTEINS (REACTOME)

Gene	probeID	ProbeChr	Probe bp	topSNP	topSNP chr	topSNP bp	A1	A2	Freq	b GWAS	se GWAS	p GWAS	b eQTL	se eQTL	p eQTL	b SMR	se SMR	p SMR	p SMR multi	p HEIDI	nsnp HEIDI	PASS BONF	Data source	Analyte
VIM	ENSG00000026025	10	17274925	rs7898271	10	17052418	T	C	0.568182	0.0067	0.0099	0.4985534	-0.301537	0.0467134	1.08E-10	-0.0222195	0.0330118	0.5008974	0.3971622	0.3634895	20	FALSE	Qi et al., 2018	Brain
CASP8	ENSG00000064012	2	202125300	rs10200279	2	202170655	T	C	0.286713	0.0319	0.0128	0.0126959	0.658191	0.089728	2.21E-13	0.0484662	0.020539	0.01828891	0.08317744	0.0531694	18	FALSE	Qi et al., 2018	Brain
DBNL	ENSG00000136279	7	44096643	rs2595699	7	44005513	G	A	0.944056	-0.0557	0.0257	0.03021083	1.12128	0.116832	8.20E-22	-0.0496755	0.0234974	0.03450841	0.02629425	0.02548071	12	FALSE	Qi et al., 2018	Brain
CASP6	ENSG00000138794	4	110617262	rs10004377	4	110648124	T	G	0.596154	-0.0036	0.0098	0.7133602	0.42605	0.0460858	2.36E-20	-0.0084497	0.0230201	0.7135763	0.9725797	0.9904615	20	FALSE	Qi et al., 2018	Brain
GSN	ENSG00000148180	9	124032598	rs76098787	9	124047836	C	T	0.0559441	-0.0418	0.0238	0.07903677	0.682433	0.10975	5.03E-10	-0.0612514	0.0362397	0.09099427	0.06435921	0.08804399	20	FALSE	Qi et al., 2018	Brain
GAS2	ENSG00000148935	11	22740894	rs337498	11	22688053	T	C	0.833916	0.0061	0.0149	0.6822491	-0.351179	0.0520057	1.45E-11	-0.0173701	0.0425065	0.6827997	0.8117148	0.576869	10	FALSE	Qi et al., 2018	Brain
CASP7	ENSG00000165806	10	115464802	rs3121487	10	115408679	T	G	0.61014	-0.0145	0.0096	0.1309371	-1.02262	0.0283134	1.18E-285	0.0141792	0.00939581	0.1312738	0.407857	0.8460837	20	FALSE	Qi et al., 2018	Brain
PLEC	ENSG00000178209	8	145020111	rs11136341	8	145043543	A	G	0.606643	-0.021	0.0099	0.03390396	1.14122	0.0309453	1.01E-297	-0.0184013	0.00868923	0.03419947	0.02849908	0.07013014	20	FALSE	Qi et al., 2018	Brain
MAPT	ENSG00000186868	17	44038724	rs199502	17	44862347	G	A	0.743007	0.1949	0.0156	8.09E-36	0.734607	0.0582562	1.86E-36	0.265312	0.0298938	6.98E-19	3.51E-24	0.01172117	17	TRUE	Qi et al., 2018	Brain
VIM	ENSG00000026025	10	17274925	rs7083383	10	17260573	C	G	0.222028	0.0025	0.0143	0.861217	-0.226594	0.0138761	6.06E-60	-0.0110329	0.063112	0.8612249	0.2727151	0.09055329	20	FALSE	Vosa et al., 2018	Blood
CASP8	ENSG00000064012	2	202125300	rs7560328	2	202164837	A	C	0.398601	0.0194	0.0096	0.04329702	-0.214284	0.00890084	4.61E-128	-0.090534	0.0449579	0.04403565	0.4704423	0.714529	20	FALSE	Vosa et al., 2018	Blood
ADD1	ENSG00000087274	4	2888693	rs6819310	4	2869127	C	T	0.293706	0.0511	0.0106	1.43E-06	0.23786	0.0120276	4.77E-87	0.214832	0.045869	2.82E-06	0.000887411	0.00734456	20	TRUE	Vosa et al., 2018	Blood
DBNL	ENSG00000136279	7	44096647	rs2528374	7	43976548	G	C	0.0559441	0.0457	0.0219	0.03691002	-0.539491	0.015575	6.73E-263	-0.0847094	0.0406674	0.03725292	0.02617208	0.0015638	20	FALSE	Vosa et al., 2018	Blood
CASP6	ENSG00000138794	4	110617262	rs13144085	4	110628473	C	T	0.0559441	0.0039	0.0193	0.83986	0.155261	0.0216863	8.10E-13	0.025119	0.124356	0.8399229	0.9717244	0.8813971	12	FALSE	Vosa et al., 2018	Blood
GSN	ENSG00000148180	9	124032599	rs10760167	9	124035545	A	G	0.548951	-0.0343	0.0099	0.00053093	-0.662881	0.00800026	0	0.0517438	0.0149479	0.00053693	0.008048225	0.08945405	20	FALSE	Vosa et al., 2018	Blood
GAS2	ENSG00000148935	11	22740894	rs4447177	11	22646025	G	A	0.15035	0.0033	0.0133	0.8040413	0.15242	0.0101964	1.60E-50	0.0216506	0.0872706	0.8040678	0.9053025	0.4432954	20	FALSE	Vosa et al., 2018	Blood
CASP3	ENSG00000164305	4	185559756	rs72689261	4	185590897	A	C	0.111888	0.0045	0.0146	0.7579156	-0.56825	0.0151553	1.11E-307	-0.0079191	0.0256938	0.7579235	0.7005626	0.8843126	20	FALSE	Vosa et al., 2018	Blood
CASP7	ENSG00000165806	10	115464802	rs4353229	10	115489589	C	T	0.234266	-0.0125	0.0107	0.2427163	-0.66876	0.00909944	0	0.0186913	0.0160018	0.2427758	0.6036084	0.7677355	20	FALSE	Vosa et al., 2018	Blood
PLEC	ENSG00000178209	8	145020111	rs11781667	8	145040856	T	G	0.377622	0.0235	0.0099	0.01760907	-0.28405	0.0104024	3.58E-164	-0.082732	0.0349845	0.01803889	0.02280896	0.5832694	20	FALSE	Vosa et al., 2018	Blood
MAPT	ENSG00000186868	17	44038724	rs62074125	17	44852612	C	A	0.225524	0.0962	0.0129	8.83E-14	-0.0984498	0.0146407	1.76E-11	-0.977148	0.195666	5.92E-07	1.03E-07	4.34E-10	6	TRUE	Vosa et al., 2018	Blood
SPTAN1	ENSG00000197694	9	131355403	rs7036322	9	131417140	A	C	0.129371	-0.0031	0.0159	0.8454176	-0.0555623	0.00930038	2.31E-09	0.0557932	0.286318	0.8454988	0.8778587	0.6220713	6	FALSE	Vosa et al., 2018	Blood

CHROMATIN ORGANIZATION (REACTOME)

Gene	probeID	ProbeChr	Probe bp	topSNP	topSNP chr	topSNP bp	A1	A2	Freq	b GWAS	se GWAS	p GWAS	b eQTL	se eQTL	p eQTL	b SMR	se SMR	p SMR	p SMR multi	p HEIDI	nsnp HEIDI	PASS BONF	Data source	Analyte
KDM1A	ENSG00000004487	1	23378061	rs9426808	1	23484031	C	A	0.286713	-0.0049	0.0109	0.6530412	-0.299064	0.0510297	4.61E-09	0.0163845	0.0365541	0.6539911	0.6539911	0.3184136	20	FALSE	Qi et al., 2018	Brain
SMARCE1	ENSG00000073584	17	38792987	rs9303294	17	38837739	T	C	0.472028	-0.0022	0.0094	0.8149519	-0.239792	0.0350218	7.54E-12	0.00917462	0.0392235	0.815058	0.985473	0.9994426	20	FALSE	Qi et al., 2018	Brain
KAT6A	ENSG00000083168	8	41848252	rs6987070	8	41877317	G	A	0.335664	-0.016	0.0104	0.1239358	0.330856	0.0466734	1.35E-12	-0.0483594	0.0321654	0.1327201	0.1853193	0.990297	10	FALSE	Qi et al., 2018	Brain
NCOA1	ENSG00000084676	2	24854177	rs11125765	2	24979464	T	C	0.88986	-0.0144	0.0159	0.3651156	-0.474717	0.0731016	8.36E-11	0.0303338	0.0338178	0.3697299	0.687024	0.4554889	20	FALSE	Qi et al., 2018	Brain
SMARCB1	ENSG00000099956	22	24152926	rs11703363	22	24179661	A	T	0.891608	-0.0079	0.0155	0.6102775	0.835236	0.0703862	1.77E-32	-0.0094584	0.0185747	0.6106065	0.6829779	0.4098225	20	FALSE	Qi et al., 2018	Brain
EP300	ENSG00000100393	22	41531935	rs139486	22	41627654	C	G	0.645105	-0.0439	0.0096	4.81E-06	-0.290179	0.0485143	2.21E-09	0.151286	0.0416441	0.00028033	0.000278114	0.02301808	20	TRUE	Qi et al., 2018	Brain
SETD6	ENSG00000103037	16	58551907	rs11649173	16	58623520	C	T	0.58042	-0.0105	0.0098	0.2839768	-0.342542	0.0352742	2.71E-22	0.0306531	0.0287832	0.2868913	0.6095596	0.3166162	20	FALSE	Qi et al., 2018	Brain
KAT8	ENSG00000103510	16	31134894	rs12597511	16	31145219	T	C	0.410839	0.0746	0.0098	2.69E-14	-0.50755	0.0392543	3.06E-38	-0.146981	0.0224062	5.39E-11	5.79E-12	0.00464736	20	TRUE	Qi et al., 2018	Brain
KAT2B	ENSG00000114166	3	20138705	rs9833147	3	20278275	C	T	0.811189	-0.0204	0.0121	0.09180535	-1.27692	0.0372389	1.11E-257	0.015976	0.00948741	0.09219786	0.1265781	0.4302525	20	FALSE	Qi et al., 2018	Brain
RBBP5	ENSG00000117222	1	205073206	rs7546850	1	205059066	G	A	0.54021	0.0027	0.0096	0.7785187	0.536775	0.0478204	3.08E-29	0.00503004	0.0178902	0.7785863	0.01388387	0.5438043	20	FALSE	Qi et al., 2018	Brain

DR1	ENSG00000117505	1	93819797	rs10782959	1	93811569	G	A	0.73951	-0.0476	0.0109	1.26E-05	-0.45391	0.0422094	5.69E-27	0.104867	0.025918	5.21E-05	5.37E-05	0.8238303	20	TRUE	Qi et al., 2018	Brain
KANSL1	ENSG00000120071	17	44205007	rs62074125	17	44852612	A	C	0.774476	-0.0962	0.0129	8.83E-14	-0.422379	0.0452452	1.01E-20	0.227757	0.0390896	5.66E-09	6.61E-12	1.72E-09	20	TRUE	Qi et al., 2018	Brain
USP22	ENSG00000124422	17	20924991	rs56323013	17	20900744	C	T	0.762238	-0.0034	0.0117	0.7713586	0.534026	0.0439716	6.11E-34	-0.0063667	0.0219153	0.7714222	0.7921502	0.7938354	20	FALSE	Qi et al., 2018	Brain
YEATS4	ENSG00000127337	12	69769029	rs11177644	12	69785748	G	A	0.472028	0.0029	0.0101	0.7740138	-0.513272	0.0384571	1.24E-40	-0.00565	0.0196822	0.7740646	0.5438267	0.619129	20	FALSE	Qi et al., 2018	Brain
KDM6B	ENSG00000132510	17	7750668	rs3744248	17	7751751	C	T	0.944056	0.0533	0.0186	0.00416231	0.616545	0.099424	5.60E-10	0.0864496	0.0332335	0.00928768	0.009287677	0.1848864	20	FALSE	Qi et al., 2018	Brain
PRMT7	ENSG00000132600	16	68368671	rs1532211	16	68394307	G	A	0.48951	0.017	0.0097	0.07967459	0.604592	0.0492737	1.31E-34	0.0281181	0.0162067	0.08274633	0.08142507	0.8993335	20	FALSE	Qi et al., 2018	Brain
ELP3	ENSG00000134014	8	27997931	rs2305451	8	27950670	C	T	0.678322	-0.0135	0.0103	0.189966	-1.08	0.0291901	1.19E-299	0.0125	0.00954299	0.1902439	0.7277102	0.1641437	20	FALSE	Qi et al., 2018	Brain
CLOCK	ENSG00000134852	4	56353687	rs13138241	4	56423225	G	A	0.636364	0.0111	0.0102	0.2764912	-0.338973	0.0484653	2.67E-12	-0.032746	0.0304529	0.2822417	0.9492662	0.5979075	20	FALSE	Qi et al., 2018	Brain
SETDB2	ENSG00000136169	13	50043783	rs1853324	13	50005780	C	T	0.312937	-0.0132	0.0103	0.1999994	0.313658	0.0454516	5.17E-12	-0.042084	0.0333998	0.2076656	0.04136517	0.02251178	20	FALSE	Qi et al., 2018	Brain
AEBP2	ENSG00000139154	12	19715357	rs67864861	12	19701105	A	G	0.730769	-0.0025	0.0112	0.8233687	-0.333226	0.0481511	4.50E-12	0.00750242	0.0336283	0.823459	0.7799063	0.882112	20	FALSE	Qi et al., 2018	Brain
KANSL2	ENSG00000139620	12	49061602	rs4760739	12	49066794	G	A	0.718531	-0.0102	0.0111	0.358138	-0.487245	0.0530018	3.82E-20	0.020934	0.0228947	0.3605265	0.4634337	0.3769871	20	FALSE	Qi et al., 2018	Brain
CARM1	ENSG00000142453	19	11007821	rs11669166	19	11062139	G	A	0.851399	-0.0503	0.0143	0.00043566	0.409574	0.0640162	1.57E-10	-0.12281	0.039843	0.00205366	0.03522688	0.03202175	20	FALSE	Qi et al., 2018	Brain
SMYD2	ENSG00000143499	1	214482459	rs6691532	1	214466553	G	A	0.56993	0.0036	0.0101	0.7215143	0.320767	0.041757	1.57E-14	0.0112231	0.0315209	0.7218012	0.16058	0.07673144	20	FALSE	Qi et al., 2018	Brain
GATAD2B	ENSG00000143614	1	153836326	rs7521047	1	154076366	T	C	0.479021	-0.0034	0.0098	0.7286373	0.996122	0.0294304	4.05E-251	-0.0034132	0.00983867	0.728651	0.1012758	0.0304693	20	FALSE	Qi et al., 2018	Brain
SAP18	ENSG00000150459	13	21718937	rs9509568	13	21708253	A	T	0.361888	0.0283	0.0125	0.02357411	-0.358682	0.0469844	2.27E-14	-0.0789	0.0363501	0.02996448	0.1199084	0.270071	20	FALSE	Qi et al., 2018	Brain
TADA1	ENSG00000152382	1	166835655	rs7542977	1	167007218	C	T	0.973776	-0.0163	0.0268	0.5430489	0.877326	0.140468	4.22E-10	-0.0185792	0.0306918	0.5449494	0.7547874	0.08538408	20	FALSE	Qi et al., 2018	Brain
KAT6B	ENSG00000156650	10	76688860	rs3862556	10	76540987	C	T	0.935315	0.0284	0.02	0.1556077	0.801942	0.102425	4.90E-15	0.035414	0.0253463	0.1623514	0.3802334	0.5550268	20	FALSE	Qi et al., 2018	Brain
HIST1H2BD	ENSG00000158373	6	26164963	rs9379831	6	26175852	C	A	0.68007	-0.0001	0.0101	0.9921003	0.282419	0.0493856	1.07E-08	-0.0003541	0.0357625	0.9921003	0.9921003	0.01609664	8	FALSE	Qi et al., 2018	Brain
PADI4	ENSG00000159339	1	17662594	rs2301888	1	17672730	G	A	0.655594	0.0066	0.01	0.5092538	-0.587861	0.06824	7.02E-18	-0.0112271	0.0170607	0.5104922	0.6418833	0.9391461	20	FALSE	Qi et al., 2018	Brain
ATXN7	ENSG00000163635	3	63919685	rs2280162	3	63997491	G	A	0.811189	0.0254	0.0127	0.04550026	0.379857	0.0567716	2.22E-11	0.0668672	0.0348952	0.05533626	0.03955461	0.5395873	20	FALSE	Qi et al., 2018	Brain
MEAF6	ENSG00000163875	1	37969275	rs12035095	1	38007121	G	C	0.730769	0.009	0.011	0.4132534	0.497274	0.0527103	3.94E-21	0.0180987	0.0222036	0.4150024	0.4004532	0.7598895	20	FALSE	Qi et al., 2018	Brain
SAP30	ENSG00000164105	4	174294901	rs4696071	4	174325726	G	A	0.520979	-0.0021	0.0102	0.8368828	-0.265307	0.0461995	9.32E-09	0.00791536	0.0384707	0.8369861	0.9207999	0.6846772	16	FALSE	Qi et al., 2018	Brain
KDM1B	ENSG00000165097	6	18189822	rs214598	6	18207876	C	G	0.337413	-0.023	0.0113	0.04181082	-0.356986	0.0402253	7.02E-19	0.0644283	0.0324758	0.04726818	0.3059057	0.4273795	20	FALSE	Qi et al., 2018	Brain
GATAD2A	ENSG00000167491	19	19558189	rs8101499	19	19476984	A	G	0.358392	0.0051	0.0102	0.6170751	-0.308031	0.0374853	2.08E-16	-0.0165568	0.0331748	0.6177251	0.4723851	0.5682482	20	FALSE	Qi et al., 2018	Brain
ELP5	ENSG00000170291	17	7158997	rs402514	17	7158203	T	G	0.382867	-0.0354	0.0098	0.00030356	1.15047	0.0306724	6.77E-308	-0.0307701	0.00855769	0.00032363	0.00060464	0.4636315	20	TRUE	Qi et al., 2018	Brain
TADA2B	ENSG00000173011	4	7050997	rs12502513	4	7054680	C	G	0.912587	0.0523	0.019	0.00591184	0.503019	0.0868257	6.90E-09	0.103972	0.0418186	0.01290914	0.01290914	0.2596585	20	FALSE	Qi et al., 2018	Brain
DMAP1	ENSG00000178028	1	44682740	rs226070	1	44822708	G	A	0.73951	0.0364	0.0109	0.00083945	-0.333553	0.0378365	1.19E-18	-0.109128	0.0349446	0.00179082	0.001594149	0.4911748	20	FALSE	Qi et al., 2018	Brain
RUVBL2	ENSG00000183207	19	49507978	rs3810176	19	49496786	C	T	0.923077	0.0351	0.0185	0.05778871	0.944044	0.0853882	2.05E-28	0.0371805	0.019883	0.06148908	0.1311302	0.5339415	20	FALSE	Qi et al., 2018	Brain
BRWD1	ENSG00000185658	21	40624793	rs2776309	21	40715791	G	A	0.307692	-0.0087	0.0101	0.3890254	-0.297743	0.0411657	4.73E-13	0.0292198	0.0341616	0.392362	0.1468184	0.1417299	20	FALSE	Qi et al., 2018	Brain
MORF4L1	ENSG00000185787	15	79146652	rs734287	15	79173900	G	A	0.809441	0.0146	0.0126	0.2465662	0.426914	0.0588772	4.14E-13	0.034199	0.0298887	0.2525357	0.05248118	0.05741705	20	FALSE	Qi et al., 2018	Brain
MSL1	ENSG00000188895	17	38285795	rs2715554	17	38489170	A	G	0.842657	0.0269	0.0149	0.07101694	0.519753	0.0686503	3.70E-14	0.0517553	0.0294712	0.07906608	0.02826279	0.4469217	20	FALSE	Qi et al., 2018	Brain
SUPT3H	ENSG00000196284	6	45061372	rs12195339	6	44796179	T	C	0.737762	-0.0063	0.0114	0.5805157	-0.638214	0.0425683	8.20E-51	0.0098713	0.0178745	0.5807726	0.9754501	0.9026803	20	FALSE	Qi et al., 2018	Brain
HIST1H4C	ENSG00000197061	6	26104311	rs198847	6	26106619	C	A	0.589161	0.0215	0.012	0.07318638	0.317145	0.034302	2.34E-20	0.0677923	0.0385415	0.0785869	0.01584491	0.3221149	20	FALSE	Qi et al., 2018	Brain
PHF2	ENSG00000197724	9	96390279	rs10761256	9	96446695	C	G	0.666084	0.0144	0.0107	0.1783688	-0.301201	0.0479406	3.33E-10	-0.0478086	0.0363303	0.1881931	0.137956	0.00841398	17	FALSE	Qi et al., 2018	Brain
PRMT6	ENSG00000198890	1	107600591	rs1730862	1	107614003	G	A	0.328671	0.0018	0.0103	0.8612704	-0.459489	0.0383555	4.54E-33	-0.0039174	0.0224186	0.861285	0.946698	0.8797697	13	FALSE	Qi et al., 2018	Brain
TAF9	ENSG00000085231	5	68656325	rs299075	5	68728544	A	G	0.480769	0.0091	0.0094	0.3330019	-0.290072	0.0512089	1.47E-08	-0.0313715	0.0328756	0.3399574	0.2113811	0.117286	20	FALSE	Qi et al., 2018	Brain
HIST1H4F	ENSG00000198327	6	26240768	rs11759720	6	26250254	C	G	0.802448	0.0184	0.0124	0.1378432	-0.645065	0.081341	2.18E-15	-0.0285243	0.0195565	0.1446868	0.08360257	0.146912	20	FALSE	Qi et al., 2018	Brain
HIST1H3E	ENSG00000196966	6	26225613	rs11757549	6	26231608	G	A	0.907343	0.02	0.0168	0.2338593	-0.860175	0.125459	7.07E-12	-0.0232511	0.0198231	0.2408254	0.2408254	0.1721667	15	FALSE	Qi et al., 2018	Brain
TADA2A	ENSG00000108264	17	35803400	rs2522970	17	35831705	G	A	0.606643	0.004	0.0097	0.6800674	0.392188	0.037826	3.46E-25	0.0101992	0.0247526	0.6803062	0.3428815	0.07882699	20	FALSE	Qi et al., 2018	Brain
RUVBL2	ENSG00000183207.8	19	49507970	rs3795051	19	49520890	A	G	0.0839161	-0.0255	0.0215	0.2356039	-0.786554	0.133865	4.21E-09	0.0324199	0.0278857	0.2449927	0.1915228	0.07420583	6	FALSE	GTEx Consortium	Substantia Nigra
HIST1H3E	ENSG00000196966.3	6	26225613	rs74928264	6	26224343	G	C	0.0926573	-0.0199	0.0168	0.2362058	1.22093	0.197454	6.28E-10	-0.0162991	0.0140102	0.2446796	0.2446796	0.4080319	6	FALSE	GTEx Consortium	Substantia Nigra
KDM1A	ENSG00000004487	1	23378061	rs67056484	1	23434819	T	C	0.16958	-0.0241	0.0134	0.07209663	-0.107197	0.0112161	1.21E-21	0.224821	0.127198	0.07714704	0.1392576	0.5804272	20	FALSE	Vosa et al., 2018	Blood
KMT2E	ENSG00000005483	7	104704717	rs10274607	7	104635597	C	A	0.118881	-0.0108	0.015	0.471525	0.0532071	0.00908746	4.77E-09	-0.20298	0.284041	0.4748457	0.1526283	0.2684355	20	FALSE	Vosa et al., 2018	Blood
KDM7A	ENSG00000006459	7	139830690	rs11769879	7	140005193	A	G	0.461538	-0.0218	0.0101	0.03089552	-0.13721	0.00895591	5.57E-53	0.158881	0.0743368	0.03257284	0.04018077	0.5337823	20	FALSE	Vosa et al., 2018	Blood
PHF20	ENSG00000025293	20	34449099	rs56052651	20	34344084	T	C	0.103147	0.0085	0.0152	0.5760181	-0.0985386	0.0142855	5.28E-12	-0.0862606	0.15476	0.5772666	0.684969	0.5952509	19	FALSE	Vosa et al., 2018	Blood
ARID4A	ENSG00000032219	14	58802854	rs10151525	14	58772018	G	A	0.0646853	0.0479	0.0189	0.01126428	0.22127	0.0153385	3.56E-47	0.216478	0.0867243	0.01255452	0.02053051	0.1031097	20	FALSE	Vosa et al., 2018	Blood
JADE2	ENSG00000043143	5	133889460	rs10040679	5	133853332	C	T	0.148601	-0.001	0.0144	0.9446359	0.0868782	0.012926	1.80E-11	-0.0115104	0.165758	0.9446388	0.1025916	0.062705	10	FALSE	Vosa et al., 2018	Blood
ARID4B	ENSG00000054267	1	235393241	rs72756074	1	235397022	G	A	0.111888	0.0281	0.0182	0.122599	0.105421	0.0141554	9.52E-14	0.26655	0.176312	0.1305823	0.1974205	0.4017906	14	FALSE	Vosa et al., 2018	Blood
MTA3	ENSG00000057935	2	42852898	rs10187451	2	42901823	A	C	0.367133	-0.0007	0.01	0.9441937	-0.119952	0.00922953	1.28E-38	0.00583564	0.0833676	0.9441945	0.3382636	0.4122307	20	FALSE	Vosa et al., 2018	Blood
HMG20B	ENSG00000064961	19	3575930	rs10420721	19	3575422	C	T	0.0122378	0.019	0.0447	0.6707959	-0.129154	0.0200065	1.08E-10	-0.147111	0.346848	0.6714655	0.7514432	0.898407	3	FALSE	Vosa et al., 2018	Blood
SMARCD1	ENSG00000066117	12	50486625	rs10875995	12	50462138	C	T	0.332168	-0.0289	0.0106	0.00640264	0.170035	0.0115461	4.35E-49	-0.169965	0.0633993	0.00734335	0.02372349	0.9410278	20	FALSE	Vosa et al., 2018	Blood
ELP1	ENSG00000070061	9	111663096	rs1772033	9	111769543	A	G	0.183566	0.0195	0.0129	0.1306285	-0.235356	0.0131334	8.17E-72	-0.0828532	0.0550052	0.1319957	0.3094501	0.04018503	20	FALSE	Vosa et al., 2018	Blood
JMJD6	ENSG00000070495	17	74715892	rs2286585	17	74712310	G	A	0.486014	0.012	0.0101	0.2347866	-0.376235	0.0091669	0	-0.031895	0.0268562	0.2349826	0.1153294	0.1085676	20	FALSE	Vosa et al., 2018	Blood
ING3	ENSG00000071243	7	120604036	rs2721370	7	120612239	G	A	0.194056	0.0199	0.0119	0.09447131	-0.183332	0.0125443	2.26E-48	-0.108546	0.0653331	0.09662751	0.08000091	0.3632436	20	FALSE	Vosa et al., 2018	Blood
SMARCE1	ENSG00000073584	17	38792987	rs8066541	17	38779942	A	G	0.479021	0.0025	0.0095	0.7924289	-0.730699	0.00783525	0	-0.0034214	0.0130013	0.7924297	0.3312831	0.9954324	20	FALSE	Vosa et al., 2018	Blood
KDM5A	ENSG00000073614	12	443957	rs579526	12	373120	C	T	0.178322	0.0195	0.0114	0.08716859	-0.328317	0.00948939	2.67E-262	-0.0593937	0.0347649	0.0875548	0.03947101	0.1026868	20	FALSE	Vosa et al., 2018	Blood
EED	ENSG00000074266	11	85972720	rs10898448	11	85947751	A	G	0.368881	0.015	0.0107	0.1609543	0.0870934	0.00957828	9.65E-20	0.172229	0.124308	0.1658998	0.211834	0.8891556	18	FALSE	Vosa et al., 2018	Blood
ACTB	ENSG00000075624	7	5585098	rs4296949	7	5508644	C	T	0.335664	-0.0124	0.0105	0.2376216	-0.0542021	0.00962499	1.79E-08	0.228773	0.197933	0.2477585	0.2477585	0.6867758	7	FALSE	Vosa et al., 2018	Blood
JADE1	ENSG00000077684	4	129763579	rs72683669	4	129872122	A	G	0.325175	-0.0086	0.0106	0.4171815	-0.164226	0.00959804	1.24E-65	0.0523668	0.0646176	0.4177044	0.06433169	0.2829252	20	FALSE	Vosa et al., 2018	Blood
SMARCA2	ENSG00000080503	9	2104483	rs10114878	9	2030300	C	T	0.197552	0.0154	0.0124	0.2142604	-0.114176	0.0093813	4.46E-34	-0.134879	0.109168	0.2166372	0.384405	0.07408317	20	FALSE	Vosa et al., 2018	Blood
SMARCD3	ENSG00000082014	7	150955416	rs6972652	7	150965896	T	C	0.288462	-0.0239	0.0116	0.03936559	-0.124824	0.0123247	4.15E-24	0.191469	0.0948342	0.04348812	0.004150775	0.2083572	20	FALSE	Vosa et al., 2018	Blood
KAT6A	ENSG00000083168	8	41848252	rs6987070	8	41877317	G	A	0.335664	-0.016	0.0104	0.1239358	0.198653	0.00996074	1.70E-88	-0.0805424	0.0525081	0.1250532	0.7669414	0.8216023	20	FALSE	Vosa et al., 2018	Blood
REST	ENSG00000084093	4	57788042	rs11728137	4	57751875	T	C	0.300699	-0.0042	0.0106	0.691938	0.0780891	0.00973899	1.07E-15	-0.0537847	0.135908	0.6922942	0.8610857	0.681486	20	FALSE	Vosa et al., 2018	Blood
NCOA1	ENSG00000084676	2	24854177	rs12614742	2	24711484	T	C	0.466783	0.0062	0.0098	0.5269602	0.151895	0.00889369	2.13E-65	0.0408176	0.0645624	0.5272435	0.5477442	0.4123711	20	FALSE	Vosa et al., 2018	Blood
MECOM	ENSG00000085276	3	169091346	rs6444842	3	168874035	G	A	0.0664336	-0.0174	0.0181	0.3363883	0.151612	0.0165758	5.88E-20	-0.114767	0.120041	0.339042	0.8335263	0.2621763	20	FALSE	Vosa et al., 2018	Blood
ATXN7L3	ENSG00000087152	17	42273327	rs117820542	17	42275834	G	C	0.027972	-0.0437	0.0292	0.1345038	-0.27084	0.0411974	4.89E-11	0.16135	0.110571	0.1444983	0.1444983	0.2732269	4	FALSE	Vosa et al., 2018	Blood
BRPF3	ENSG00000096070	6	36182544	rs7766116	6	36222688	T	G	0.370629	-0.0004	0.0098	0.9674423	-0.0596813	0.00903768	4.01E-11	0.00670227	0.164209	0.9674429	0.3122783	0.1110706	11	FALSE	Vosa et al., 2018	Blood
JAK2	ENSG00000096968	9	5056608	rs6476941	9	5133609	C	T	0.503497	-0.0009	0.0102	0.9296897	-0.231532	0.00891384	9.64E-149	0.00388716	0.0440547	0.9296901	0.2785131	0.4012363	20	FALSE	Vosa et al., 2018	Blood
SETD1A	ENSG00000099381	16	30982526	rs7206511	16	30950352	A	G	0.652098	0.0905	0.0099	6.16E-20	-0.12754	0.00919329	9.21E-44	-0.709579	0.0929587	2.29E-14	5.62E-14	0.08522184	20	TRUE	Vosa et al., 2018	Blood
SMARCB1	ENSG00000099956	22	24152926	rs35370296	22	24157537	C	T	0.113636	0.0079	0.0151	0.6008498	-0.614917	0.0124537	0	-0.0128473	0.0245575	0.6008703	0.1162501	0.541565	20	FALSE	Vosa et al., 2018	Blood
EP300	ENSG00000100393	22	41531935	rs5751069	22	41627775	G	C	0.305944	-0.055	0.0106	2.12E-07	0.282262	0.00890112	1.10E-220	-0.194854	0.0380531	3.05E-07	2.43E-06	0.00925567	20	TRUE	Vosa et al., 2018	Blood
BRD1	ENSG00000100425	22	50194045	rs137876801	22	50203152	A	G	0.0646853	0.039	0.0202	0.05352102	-0.471785	0.0286441	5.97E-61	-0.0826647	0.0431093	0.05516635	0.1312357	0.03524274	20	FALSE	Vosa et al., 2018	Blood
HDAC10	ENSG00000100429	22	50686723	rs13058062	22	50684296	T	C	0.29021	0.0136	0.0131	0.2991919	0.112988	0.0116432	2.89E-22	0.120366	0.116603	0.3019421	0.01253791	0.03809274	20	FALSE	Vosa et al., 2018	Blood
PRMT5	ENSG00000100462	14	23394257	rs4981454	14	23422186	G	A	0.225524	-0.006	0.0117	0.6080769	-0.474981	0.0105943	0	0.0126321	0.0246342	0.6081003	0.4575056	0.2706976	20	FALSE	Vosa et al., 2018	Blood
MRGBP	ENSG00000101189	20	61429875	rs140838580	20	61427869	T	C	0.0944056	0.0445	0.0205	0.02995146	-0.312301	0.0165298	1.30E-79	-0.142491	0.0660737	0.03104119	0.2339317	0.4742438	20	FALSE	Vosa et al., 2018	Blood
SUPT20H	ENSG00000102710	13	37608649	rs1924426	13	37609725	C	T	0.0979021	-0.0011	0.0168	0.9477949	-0.766615	0.0196869	0	0.00143488	0.0219145	0.9477949	0.4205784	0.8944358	20	FALSE	Vosa et al., 2018	Blood
SETD6	ENSG00000103037	16	58551907	rs8047769	16	58591987	G	A	0.251748	0.0028	0.0108	0.7954352	-0.250672	0.0098009	2.79E-144	-0.01117	0.0430865	0.7954455	0.6122905	0.243283	20	FALSE	Vosa et al., 2018	Blood
KAT8	ENSG00000103510	16	31134894	rs1060506	16	31133449	C	T	0.723776	0.0625	0.0106	3.72E-09	-0.253319	0.00963612	2.60E-152	-0.246724	0.042884	8.75E-09	1.17E-15	1.74E-05	20	TRUE	Vosa et al., 2018	Blood
DOT1L	ENSG00000104885	19	2198362	rs12459507	19	2224387	C	A	0.0804196	0.029	0.0179	0.1052083	0.084092	0.0109904	1.99E-14	0.344861	0.217582	0.1129729	0.2163533	0.6106312	20	FALSE	Vosa et al., 2018	Blood
H2AFV	ENSG00000105968	7	44877036	rs1297837	7	44869537	C	T	0.108392	-0.0353	0.0159	0.02641023	-0.13577	0.00916251	1.12E-49	0.259998	0.118417	0.02811922	0.06288815	0.8257281	20	FALSE	Vosa et al., 2018	Blood
EZH2	ENSG00000106462	7	148542944	rs3779038	7	148542822	C	T	0.304196	0.0171	0.0108	0.1133455	-0.247711	0.0103068	1.23E-127	-0.0690322	0.0436938	0.1141278	0.06160413	0.09033032	20	FALSE	Vosa et al., 2018	Blood
KDM4C	ENSG00000107077	9	6948255	rs6477108	9	6761229	A	G	0.201049	-0.0067	0.0124	0.5889746	0.118087	0.00937955	2.40E-36	-0.056738	0.105104	0.5893173	0.8428602	0.2413547	20	FALSE	Vosa et al., 2018	Blood
SMARCD2	ENSG00000108604	17	61914934	rs752621	17	61901880	C	G	0.270979	0.0081	0.0104	0.4360703	0.469715	0.0108676	0	0.0172445	0.0221447	0.4361448	0.09778919	0.2581871	20	FALSE	Vosa et al., 2018	Blood
KAT2A	ENSG00000108773	17	40269251	rs35184688	17	40272406	C	G	0.23951	-0.0152	0.0113	0.1785824	-0.0920218	0.00949539	3.29E-22	0.165178	0.123974	0.182742	0.2500572	0.7388519	20	FALSE	Vosa et al., 2018	Blood
NFKB1	ENSG00000109320	4	103480472	rs11097787	4	103407342	T	C	0.40035	-0.0052	0.0113	0.6453892	0.183195	0.00892962	1.57E-93	-0.0283851	0.0616985	0.6454722	0.4627897	0.4204278	20	FALSE	Vosa et al., 2018	Blood
CCND1	ENSG00000110092	11	69462548	rs9651783	11	69373584	G	T	0.379371	-0.0133	0.0103	0.1966128	-0.200925	0.00935956	3.14E-102	0.0661937	0.0513555	0.1974215	0.5774775	0.2684301	20	FALSE	Vosa et al., 2018	Blood
CHD4	ENSG00000111642	12	6697945	rs7303399	12	6708813	T	A	0.131119	-0.0119	0.0131	0.3636685	-0.233145	0.017302	2.19E-41	0.0510413	0.0563158	0.364756	0.4301203	0.7114429	20	FALSE	Vosa et al., 2018	Blood
ING4	ENSG00000111653	12	6765880	rs117012163	12	6761893	A	G	0.0157343	0.0126	0.0461	0.7846081	-0.322163	0.0469037	6.48E-12	-0.0391106	0.143209	0.7847742	0.7349982	0.3367107	9	FALSE	Vosa et al., 2018	Blood
SUDS3	ENSG00000111707	12	118835012	rs11068872	12	118654650	T	C	0.0157343	-0.018	0.0362	0.6190215	0.128095	0.0158856	7.41E-16	-0.140521	0.28314	0.6196864	0.2105596	0.7296096	5	FALSE	Vosa et al., 2018	Blood
KAT2B	ENSG00000114166	3	20138705	rs2948085	3	20098119	T	C	0.157343	0.0141	0.0132	0.2854385	0.24087	0.0141917	1.31E-64	0.0585377	0.0549097	0.2863908	0.02189357	0.2286889	20	FALSE	Vosa et al., 2018	Blood

KANSL3	ENSG00000114982	2	97283715	rs2579499	2	97232656	G	C	0.332168	-0.0041	0.01	0.6818059	0.0740109	0.00907686	3.53E-16	-0.0553973	0.135286	0.6821855	0.1926328	0.06603768	14	FALSE	Vosa et al., 2018	Blood
KDM3A	ENSG00000115548	2	86693804	rs308902	2	86843758	G	C	0.524476	0.0075	0.0098	0.4440893	-0.0616593	0.0094099	5.65E-11	-0.121636	0.160018	0.4471714	0.4075358	0.6228075	20	FALSE	Vosa et al., 2018	Blood
ATF2	ENSG00000115966	2	175985044	rs21818	2	176035845	G	A	0.211538	-0.0067	0.0125	0.5919586	-0.0604832	0.0103461	5.04E-09	0.110775	0.207536	0.5935068	0.6705108	0.9350742	13	FALSE	Vosa et al., 2018	Blood
WDR77	ENSG00000116455	1	111987255	rs6692745	1	112019159	G	A	0.298951	0.0135	0.0101	0.1813422	-0.0812923	0.00930842	2.48E-18	-0.166067	0.12569	0.1864186	0.567442	0.178571	20	FALSE	Vosa et al., 2018	Blood
PADI2	ENSG00000117115	1	17419602	rs2235910	1	17425829	T	G	0.638112	0.0245	0.0109	0.02459492	0.691549	0.00780816	0	0.0354277	0.0157668	0.02464112	0.01760462	0.08848651	20	FALSE	Vosa et al., 2018	Blood
KDM5B	ENSG00000117139	1	202737562	rs10494836	1	202696878	T	C	0.0297203	-0.0584	0.0288	0.04258293	0.28396	0.0141599	1.87E-89	-0.205663	0.10194	0.04364441	0.7067888	0.2827224	20	FALSE	Vosa et al., 2018	Blood
DR1	ENSG00000117505	1	93819797	rs3767964	1	93815490	T	C	0.361888	0.0428	0.0101	2.26E-05	0.448757	0.00849776	0	0.0953746	0.022579	2.40E-05	0.000216115	0.2968573	20	TRUE	Vosa et al., 2018	Blood
ARID1A	ENSG00000117713	1	27065559	rs12728091	1	27143513	T	A	0.0699301	0.0285	0.0216	0.1870206	-1.07423	0.0193811	0	-0.0265306	0.0201131	0.1871455	0.2502195	0.05224423	20	FALSE	Vosa et al., 2018	Blood
SUPT7L	ENSG00000119760	2	27880177	rs8731	2	27873326	G	C	0.211538	-0.011	0.0113	0.330329	0.0912887	0.00971342	5.55E-21	-0.120497	0.124445	0.332909	0.3910108	0.2631209	20	FALSE	Vosa et al., 2018	Blood
DNMT3A	ENSG00000119772	2	25510652	rs754209	2	25502769	T	C	0.396853	0.0123	0.01	0.2186971	-0.116552	0.00918164	6.38E-37	-0.105533	0.0862007	0.2208518	0.1432723	0.09191249	20	FALSE	Vosa et al., 2018	Blood
KANSL1	ENSG00000120071	17	44205007	rs12951057	17	44794558	G	C	0.148601	0.081	0.0148	4.43E-08	-0.236481	0.0170979	1.66E-43	-0.342522	0.067306	3.60E-07	8.58E-21	4.23E-21	13	TRUE	Vosa et al., 2018	Blood
ENY2	ENSG00000120533	8	110352367	rs12334438	8	110381783	T	A	0.449301	-0.0322	0.0095	0.00070027	0.108542	0.00903133	2.84E-33	-0.296659	0.0909377	0.00110543	0.000507578	0.1914088	20	FALSE	Vosa et al., 2018	Blood
EPC1	ENSG00000120616	10	32612202	rs79442609	10	32608749	T	C	0.159091	-0.0047	0.0138	0.73342	-0.181762	0.0120253	1.29E-51	0.025858	0.0759427	0.7334851	0.24645	0.5977995	20	FALSE	Vosa et al., 2018	Blood
KDM3B	ENSG00000120733	5	137730501	rs79976632	5	137772334	G	A	0.0926573	-0.0435	0.0156	0.0052959	-0.631281	0.0210195	3.63E-198	0.0689075	0.0248179	0.00549443	0.005394075	0.00986784	20	FALSE	Vosa et al., 2018	Blood
USP22	ENSG00000124422	17	20924991	rs7207753	17	20919337	G	C	0.216783	-0.0051	0.0122	0.6759231	0.295739	0.00925216	3.42E-224	-0.017245	0.0412562	0.6759493	0.8532573	0.8240275	20	FALSE	Vosa et al., 2018	Blood
HIST1H2BJ	ENSG00000124635	6	27097299	rs12195360	6	27210032	T	C	0.0821678	0.047	0.0163	0.00393363	0.146672	0.0131852	9.59E-29	0.320444	0.114805	0.00525145	5.64E-05	0.1753802	20	TRUE	Vosa et al., 2018	Blood
YEATS4	ENSG00000127337	12	69769029	rs7136238	12	69752509	T	G	0.466783	0.0046	0.0097	0.6353382	-0.703035	0.00795434	0	-0.0065431	0.0137975	0.635343	0.2416824	0.4977383	20	FALSE	Vosa et al., 2018	Blood
SMARCA4	ENSG00000127616	19	11123834	rs73013169	19	11127069	T	G	0.311189	-0.0024	0.0104	0.8174941	-0.188181	0.0106154	2.59E-70	0.0127537	0.0552706	0.8175093	0.1278929	0.1007433	20	FALSE	Vosa et al., 2018	Blood
HAT1	ENSG00000128708	2	172813778	rs10201430	2	172813241	C	T	0.321678	-0.003	0.0131	0.818863	-0.195365	0.0089358	5.82E-106	0.0153559	0.0670576	0.8188727	0.09168819	0.01550414	20	FALSE	Vosa et al., 2018	Blood
ASH2L	ENSG00000129691	8	37982177	rs2507706	8	38009275	G	A	0.0104895	-0.0211	0.0545	0.6986407	-0.164328	0.0125546	3.81E-39	0.128402	0.3318	0.698766	0.5491503	0.9266496	5	FALSE	Vosa et al., 2018	Blood
PRMT7	ENSG00000132600	16	68368671	rs79543001	16	68387341	T	C	0.111888	0.009	0.0157	0.5664765	0.569131	0.0169877	4.43E-246	0.0158136	0.02759	0.5665333	0.80782	0.2924138	20	FALSE	Vosa et al., 2018	Blood
KMT5C	ENSG00000133247	19	55855354	rs1870073	19	55850269	A	G	0.288462	-0.0079	0.0111	0.4766433	-0.122562	0.00926727	6.28E-40	0.0644574	0.0906978	0.4772805	0.2243385	0.2290116	20	FALSE	Vosa et al., 2018	Blood
ELP3	ENSG00000134014	8	27997931	rs11786004	8	27951025	A	G	0.321678	0.0132	0.0103	0.1999994	0.553106	0.0089256	0	0.0238652	0.0186261	0.2000955	0.08383571	0.07424034	20	FALSE	Vosa et al., 2018	Blood
ELP2	ENSG00000134759	18	33733658	rs1789548	18	33724296	A	G	0.384615	-0.0078	0.0096	0.4165048	0.545667	0.00921394	0	-0.0142944	0.0175948	0.4165486	0.8571064	0.5019081	20	FALSE	Vosa et al., 2018	Blood
CLOCK	ENSG00000134852	4	56353687	rs7660517	4	56291528	C	T	0.36014	-0.0109	0.0101	0.280495	0.204978	0.00909236	1.54E-112	-0.0531764	0.0493299	0.2810456	0.9694487	0.4027118	20	FALSE	Vosa et al., 2018	Blood
PHF21A	ENSG00000135365	11	46046928	rs2932512	11	46080899	G	A	0.0769231	-0.0164	0.0177	0.3541583	0.166846	0.0165257	5.74E-24	-0.098294	0.106531	0.3561759	0.2390948	0.3724284	20	FALSE	Vosa et al., 2018	Blood
CDK4	ENSG00000135446	12	58145653	rs111566142	12	58065943	G	A	0.0821678	-0.0384	0.0253	0.1290682	0.190938	0.0257204	1.14E-13	-0.201113	0.135245	0.1370083	0.09319028	0.4665656	12	FALSE	Vosa et al., 2018	Blood
SETDB2	ENSG00000136169	13	50043783	rs7321228	13	50060676	T	G	0.31993	-0.0133	0.0102	0.1922603	0.344887	0.0113775	7.72E-202	-0.0385634	0.0296023	0.1926714	0.000201669	0.01306788	20	TRUE	Vosa et al., 2018	Blood
SAP130	ENSG00000136715	2	128742242	rs185713142	2	128684855	T	C	0.0244755	0.0273	0.0339	0.420641	-0.549144	0.0403231	3.10E-42	-0.0497137	0.0618402	0.4214516	0.1053534	0.2483567	20	FALSE	Vosa et al., 2018	Blood
AEBP2	ENSG00000139154	12	19715357	rs7953332	12	19559046	G	C	0.166084	0.0054	0.0136	0.6913241	0.0756506	0.0100658	5.67E-14	0.0713808	0.180025	0.6917319	0.8968663	0.5733498	20	FALSE	Vosa et al., 2018	Blood
SMARCC2	ENSG00000139613	12	56570059	rs2271192	12	56531154	C	T	0.111888	-0.0154	0.0151	0.3077913	0.218039	0.0119877	6.35E-74	-0.0706296	0.0693625	0.3085506	0.7815076	0.7023291	20	FALSE	Vosa et al., 2018	Blood
KANSL2	ENSG00000139620	12	49061602	rs12299955	12	49064459	T	A	0.718531	-0.0104	0.0111	0.348791	-0.32279	0.00883844	5.12E-292	0.0322191	0.034399	0.3489496	0.532701	0.1513475	20	FALSE	Vosa et al., 2018	Blood
NCOA2	ENSG00000140396	8	71169018	rs13260060	8	71218360	A	G	0.129371	0.0282	0.0156	0.0706544	0.0797072	0.0116897	9.19E-12	0.353795	0.202477	0.08058012	0.1559397	0.00257423	20	FALSE	Vosa et al., 2018	Blood
RPS2	ENSG00000140988	16	2013457	rs6366	16	2014031	C	G	0.222028	0.0288	0.0125	0.02122264	0.145957	0.0120862	1.41E-33	0.197318	0.0871861	0.02362463	0.02581647	0.00141191	11	FALSE	Vosa et al., 2018	Blood
NCOR1	ENSG00000141027	17	16028108	rs10852834	17	16174506	G	C	0.482517	-0.0429	0.0095	6.31E-06	-0.313058	0.00925491	8.12E-251	0.137035	0.030615	7.60E-06	1.73E-07	0.00290101	20	TRUE	Vosa et al., 2018	Blood
CARM1	ENSG00000142453	19	11007821	rs78301016	19	11063046	A	G	0.146853	0.0516	0.0145	0.00037281	-0.348612	0.0147248	6.49E-124	-0.148015	0.0420607	0.00043303	0.000672506	0.08424695	20	FALSE	Vosa et al., 2018	Blood
SETDB1	ENSG00000143379	1	150917976	rs7518156	1	150945794	A	C	0.597902	0.0301	0.0097	0.0019151	0.117095	0.00892165	2.37E-39	0.257056	0.0851226	0.0025291	0.0141737	0.9772087	20	FALSE	Vosa et al., 2018	Blood
SMYD2	ENSG00000143499	1	214482459	rs6687629	1	214495139	A	G	0.447552	-0.0148	0.0101	0.1428264	-0.124924	0.00958725	8.24E-39	0.118472	0.0813589	0.1453463	0.06708746	0.6146916	20	FALSE	Vosa et al., 2018	Blood
GATAD2B	ENSG00000143614	1	153836326	rs9427228	1	153768451	C	T	0.171329	0.0224	0.0124	0.07084783	-0.06273	0.009109	5.71E-12	-0.357086	0.20436	0.08057912	0.02860468	0.08221374	10	FALSE	Vosa et al., 2018	Blood
NSD3	ENSG00000147548	8	38186167	rs2234555	8	38178767	A	C	0.262238	0.0398	0.0114	0.00048081	-0.181614	0.0101297	7.03E-72	-0.219146	0.0639495	0.0006106	0.01361052	0.00565218	20	FALSE	Vosa et al., 2018	Blood
KAT14	ENSG00000149474	20	18143765	rs1205217	20	18125340	T	C	0.372378	0.0155	0.01	0.1211415	-0.122429	0.008938	1.05E-42	-0.126604	0.0822012	0.1235188	0.4081919	0.2371427	20	FALSE	Vosa et al., 2018	Blood
MTA2	ENSG00000149480	11	62364999	rs34065644	11	62371938	T	C	0.297203	-0.0316	0.0105	0.00261658	0.0906371	0.011947	3.28E-14	-0.348643	0.124629	0.0051507	0.0116515	0.6521632	10	FALSE	Vosa et al., 2018	Blood
ARID5B	ENSG00000150347	10	63758881	rs12767995	10	63943292	G	T	0.164336	0.0303	0.0127	0.01704078	0.503027	0.0140494	9.53E-281	0.0602353	0.0253031	0.01728693	0.4194714	0.8544145	20	FALSE	Vosa et al., 2018	Blood
SAP18	ENSG00000150459	13	21718937	rs927911	13	21723281	G	A	0.358392	0.0147	0.0101	0.145546	-0.114281	0.00959631	1.06E-32	-0.128631	0.0890366	0.1485433	0.103831	0.7985082	20	FALSE	Vosa et al., 2018	Blood
MBIP	ENSG00000151332	14	36778823	rs79913452	14	36816264	C	T	0.0541958	0.0016	0.0279	0.9542682	-0.200046	0.0275553	3.88E-13	-0.0079982	0.139472	0.9542697	0.5359131	0.3610708	6	FALSE	Vosa et al., 2018	Blood
TADA1	ENSG00000152382	1	166835655	rs6690192	1	166852434	T	C	0.465035	0.0226	0.0098	0.02110379	0.133038	0.00901428	2.71E-49	0.169876	0.0745569	0.02269832	0.003291111	0.1460374	20	FALSE	Vosa et al., 2018	Blood
SUV39H2	ENSG00000152455	10	14933566	rs34397997	10	14973955	T	C	0.117133	-0.0006	0.0154	0.9689215	-0.0881415	0.0159342	3.17E-08	0.00680723	0.174723	0.9689222	0.9689222	0.9813997	11	FALSE	Vosa et al., 2018	Blood
KAT6B	ENSG00000156650	10	76688860	rs2485957	10	76496963	C	T	0.22028	-0.0085	0.0116	0.4637057	-0.0798179	0.0115849	5.59E-12	0.106492	0.146151	0.4662162	0.4449288	0.1301037	8	FALSE	Vosa et al., 2018	Blood
BRPF1	ENSG00000156983	3	9781557	rs11918935	3	9759922	T	C	0.0244755	0.0632	0.0415	0.1277858	0.353187	0.0298179	2.29E-32	0.178942	0.118469	0.1309265	0.1469331	0.0156758	9	FALSE	Vosa et al., 2018	Blood
HIST1H2BD	ENSG00000158373	6	26164963	rs1009181	6	26158993	C	T	0.361888	0.0193	0.0098	0.04890858	-0.808108	0.00823209	0	-0.0238829	0.0121295	0.04895406	9.69E-05	0.03177635	20	TRUE	Vosa et al., 2018	Blood
HIST1H4H	ENSG00000158406	6	26283522	rs2393592	6	26285560	A	G	0.477273	-0.0122	0.0096	0.2037879	0.402229	0.00886424	0	-0.030331	0.0238763	0.2039652	9.69E-05	0.02197982	20	TRUE	Vosa et al., 2018	Blood
PADI4	ENSG00000159339	1	17662594	rs55912928	1	17621520	T	C	0.208042	-0.0029	0.012	0.8090385	-0.571293	0.0128167	0	0.0050762	0.0210053	0.8090412	0.3128752	0.6335309	20	FALSE	Vosa et al., 2018	Blood
TAF6L	ENSG00000162227	11	62546794	rs12364519	11	62548282	A	G	0.0437063	-0.0044	0.0205	0.8300526	-0.148577	0.0161531	3.65E-20	0.0296143	0.138013	0.8300981	0.6586862	0.08113998	15	FALSE	Vosa et al., 2018	Blood
DPY30	ENSG00000162961	2	32178879	rs13428933	2	32358864	C	A	0.148601	0.0139	0.0129	0.2812483	0.096696	0.0162509	2.68E-09	0.143749	0.135578	0.2890197	0.3634849	0.2282823	13	FALSE	Vosa et al., 2018	Blood
ELP6	ENSG00000163832	3	47546190	rs10490798	3	47328744	T	C	0.157343	0.0203	0.013	0.1183968	0.132868	0.0123215	4.12E-27	0.152783	0.0988621	0.122245	0.1768785	0.00229149	20	FALSE	Vosa et al., 2018	Blood
YEATS2	ENSG00000163872	3	183473009	rs4490385	3	183517657	A	G	0.155594	0.0386	0.0126	0.0021877	0.188367	0.0101544	8.13E-77	0.204919	0.0677968	0.00250653	0.0139947	0.00108579	20	FALSE	Vosa et al., 2018	Blood
MEAF6	ENSG00000163875	1	37969275	rs12734946	1	37974497	A	G	0.11014	0.0129	0.0143	0.3670049	-0.879837	0.016727	0	-0.0146618	0.0162554	0.3670753	0.5137399	0.3810922	20	FALSE	Vosa et al., 2018	Blood
PBRM1	ENSG00000163939	3	52649650	rs3199918	3	52581435	C	T	0.222028	0.0335	0.0145	0.02086907	0.0941385	0.0146711	1.39E-10	0.355859	0.163708	0.02972508	0.01590513	0.5655499	5	FALSE	Vosa et al., 2018	Blood
H2AFZ	ENSG00000164032	4	100870394	rs2276939	4	100870375	G	T	0.248252	-0.0175	0.012	0.1447487	-0.24915	0.0102198	2.84E-131	0.0702387	0.0482498	0.145467	0.2421593	0.9207658	20	FALSE	Vosa et al., 2018	Blood
SAP30	ENSG00000164105	4	174294901	rs13128793	4	174332612	T	G	0.524476	0.0001	0.0101	0.9921003	0.135207	0.00899164	4.20E-51	0.00073961	0.0747002	0.9921003	0.9800858	0.9279489	20	FALSE	Vosa et al., 2018	Blood
SAP30L	ENSG00000164576	5	153830704	rs734200	5	153838284	C	A	0.496503	0.0082	0.0095	0.3880507	-0.160706	0.00890178	7.44E-73	-0.051025	0.0591818	0.3885923	0.6081159	0.9927681	20	FALSE	Vosa et al., 2018	Blood
KDM1B	ENSG00000165097	6	18189822	rs1011620	6	18171477	G	A	0.0926573	-0.0007	0.0167	0.9665655	-0.364347	0.0142468	2.97E-144	0.00192124	0.0458355	0.9665656	0.614564	0.4075999	20	FALSE	Vosa et al., 2018	Blood
TAF10	ENSG00000166337	11	6630712	rs7938444	11	6627131	T	C	0.26049	0.0255	0.0107	0.01716391	-0.0725844	0.0094009	1.15E-14	-0.351315	0.154277	0.0227761	0.007578936	0.08226388	8	FALSE	Vosa et al., 2018	Blood
GATAD2A	ENSG00000167491	19	19558189	rs4808203	19	19568659	T	C	0.659091	-0.0024	0.01	0.8103303	-0.455685	0.0088885	0	0.00526679	0.0219452	0.8103323	0.4455599	0.2551101	20	FALSE	Vosa et al., 2018	Blood
ING5	ENSG00000168395	2	242655171	rs6437282	2	242642853	C	G	0.384615	0.0002	0.0133	0.9880022	-0.415212	0.00911275	0	-0.0004817	0.0320319	0.9880022	0.7293308	0.2072467	20	FALSE	Vosa et al., 2018	Blood
CHD3	ENSG00000170004	17	7802101	rs4290529	17	7767731	T	C	0.143357	-0.012	0.012	0.3173105	-0.0879527	0.00916938	8.64E-22	0.136437	0.137176	0.3199262	0.02245438	0.08092918	20	FALSE	Vosa et al., 2018	Blood
ELP5	ENSG00000170291	17	7158997	rs2074219	17	7156447	A	G	0.382867	-0.0357	0.0098	0.00026963	-0.278787	0.00944927	2.59E-191	0.128055	0.0354192	0.00029988	0.000146051	0.451473	20	TRUE	Vosa et al., 2018	Blood
RIOX2	ENSG00000170854	3	97677193	rs17374916	3	97671742	G	A	0.284965	0.0097	0.0105	0.3555855	0.177534	0.0107658	4.29E-61	0.0546373	0.0592362	0.3563391	0.534161	0.1583548	20	FALSE	Vosa et al., 2018	Blood
TADA3	ENSG00000171148	3	9828119	rs36010010	3	9836839	A	G	0.118881	-0.0001	0.0129	0.9938149	0.506954	0.0122333	0	-0.0001973	0.0254461	0.9938149	0.6051954	0.9682818	20	FALSE	Vosa et al., 2018	Blood
HDAC3	ENSG00000171720	5	141008440	rs1421896	5	141016288	T	G	0.391608	0.0239	0.0097	0.01374277	-0.131104	0.00918028	2.87E-46	-0.182299	0.0750804	0.01518047	0.02968876	0.5194693	20	FALSE	Vosa et al., 2018	Blood
COPRS	ENSG00000172301	17	30182619	rs9906443	17	30185565	G	A	0.159091	0.0077	0.0141	0.5849977	-0.200524	0.0109386	4.61E-75	-0.0383994	0.0703471	0.5851641	0.5319082	0.9800696	20	FALSE	Vosa et al., 2018	Blood
KAT5	ENSG00000172977	11	65483271	rs502468	11	65525119	G	T	0.552448	-0.0237	0.0094	0.01169299	0.0587176	0.00893723	5.03E-11	-0.403627	0.171472	0.01857763	0.02489292	0.4194792	20	FALSE	Vosa et al., 2018	Blood
TADA2B	ENSG00000173011	4	7050997	rs9986037	4	7050610	A	G	0.206294	-0.0085	0.0129	0.5099505	-0.194122	0.00984233	1.36E-86	0.0437869	0.0664901	0.5101865	0.0465576	0.05723576	20	FALSE	Vosa et al., 2018	Blood
SMARCC1	ENSG00000173473	3	47725179	rs78216580	3	47952509	T	C	0.0192308	-0.1827	0.0543	0.00076643	-0.380067	0.0494069	1.44E-14	0.480704	0.155938	0.00205153	5.95E-05	0.416222	16	TRUE	Vosa et al., 2018	Blood
MSL2	ENSG00000174579	3	135891923	rs10935177	3	135851077	A	C	0.56993	-0.0257	0.0098	0.00873003	0.0891322	0.00892697	1.78E-23	-0.288336	0.113678	0.01119921	0.02504974	0.7870427	20	FALSE	Vosa et al., 2018	Blood
BRMS1	ENSG00000174744	11	66108700	rs3016311	11	66091814	G	A	0.104895	-0.0083	0.0167	0.6191848	-0.0897929	0.0104028	6.05E-18	0.0924349	0.186292	0.6197645	0.3071427	0.4545799	20	FALSE	Vosa et al., 2018	Blood
RUVBL1	ENSG00000175792	3	127828189	rs11721156	3	127796627	T	C	0.230769	0.0204	0.0115	0.07607753	0.241823	0.0114047	8.81E-100	0.0843593	0.0477216	0.07710478	0.1924462	0.816934	20	FALSE	Vosa et al., 2018	Blood
SGF29	ENSG00000176476	16	28584173	rs151232	16	28580333	C	T	0.454545	-0.0274	0.0098	0.00517525	-0.614921	0.00819993	0	0.0445586	0.0159481	0.00520642	1.43E-05	3.34E-05	20	TRUE	Vosa et al., 2018	Blood
DMAP1	ENSG00000178028	1	44682740	rs709267	1	44681483	C	G	0.402098	0.022	0.0098	0.02477468	-0.139829	0.00942928	9.49E-50	-0.157335	0.0708843	0.02644535	0.02180543	0.244535	20	FALSE	Vosa et al., 2018	Blood
SUZ12	ENSG00000178691	17	30296050	rs55684446	17	30283327	G	C	0.162587	0.0094	0.0155	0.5442149	-0.133602	0.0178288	6.70E-14	-0.0703584	0.116396	0.5455277	0.7500366	0.9884973	20	FALSE	Vosa et al., 2018	Blood
HIST1H2AC	ENSG00000180573	6	26131858	rs112544908	6	25766673	C	T	0.0157343	-0.0681	0.0396	0.08548754	-0.380035	0.0419715	1.37E-19	0.179194	0.106064	0.09112451	0.01880431	0.7564943	14	FALSE	Vosa et al., 2018	Blood
HIST1H2BC	ENSG00000180596	6	26119627	rs62396160	6	26075393	C	T	0.0944056	-0.0252	0.0139	0.06983959	0.0980445	0.0124716	3.80E-15	-0.257026	0.145493	0.07729822	0.01172856	0.00318936	18	FALSE	Vosa et al., 2018	Blood
EHMT1	ENSG00000181090	9	140638956	rs11137167	9	140542378	T	C	0.124126	0.0064	0.0147	0.6632909	0.299598	0.0124545	7.33E-128	0.021362	0.0490739	0.6633426	0.8156831	0.584631	20	FALSE	Vosa et al., 2018	Blood
HIST3H2A	ENSG00000181218	1	228645312	rs1007686	1	228651457	A	G	0.173077	0.0173	0.0121	0.1527882	-0.283869	0.0113769	2.06E-137	-0.0609436	0.0426952	0.1534616	0.001383834	0.1139896	20	FALSE	Vosa et al., 2018	Blood
SETD2	ENSG00000181555	3	47131688	rs6791062	3	47174343	T	A	0.414336	-0.0123	0.0095	0.195411	-0.15897	0.00955209	3.44E-62	0.0773732	0.0599404	0.1967604	0.01848143	0.05117811	20	FALSE	Vosa et al., 2018	Blood
RUVBL2	ENSG00000183207	19	49507978	rs79267532	19	49530737	C	T	0.0734266	-0.0103	0.026	0.6919915	-0.331578	0.0195131	9.32E-65	0.0310636	0.0784343	0.6920709	0.05062112	0.02834848	20	FALSE	Vosa et al., 2018	Blood
SETD3	ENSG00000183576	14	99905649	rs8011858	14	99936321	A	G	0.578671	-0.0071	0.01	0.4777041	-0.339716	0.0087947	0	0.0208998	0.0294413	0.4777785	0.8347851	0.6800857	20	FALSE	Vosa et al., 2018	Blood
KMT5A	ENSG00000183955	12	123881112	rs61955125	12	123891107	C	T	0.312937	-0.0221	0.0122	0.07006729	0.337162	0.0089033	0	-0.0655471	0.0362258	0.07038788	2.80E-06	0.2052359	20	TRUE	Vosa et al., 2018	Blood
HIST2H2BE	ENSG00000184678	1	149857121	rs72708144	1	149815740	C	T	0.0402098	-0.0771	0.0313	0.01376805	-0.312251	0.0292672	1.42E-26	0.246916	0.102877	0.01638972	0.0279471	0.1781439	7	FALSE	Vosa et al., 2018	Blood
PRMT3	ENSG00000185238	11	20469958	rs11025567	11	20475735	C	G	0.178322	0.0087	0.013	0.5033483	0.104865	0.00916638	2.63E-30	0.0829636	0.124181	0.504077	0.2041108	0.3667257	20	FALSE	Vosa et al., 2018	Blood

SMYD3	ENSG00000185420	1	246291628	rs6673969	1	246671189	T	A	0.13986	0.0237	0.0146	0.1045279	0.259197	0.012066	2.31E-102	0.0914363	0.0564884	0.1055177	0.05389271	0.6406123	20	FALSE	Vosa et al., 2018	Blood
BRWD1	ENSG00000185658	21	40624793	rs73222852	21	40563414	G	A	0.0961538	-0.0062	0.0191	0.7454783	0.399486	0.0223566	2.06E-71	-0.01552	0.0478194	0.7455188	0.01328359	0.02326006	20	FALSE	Vosa et al., 2018	Blood
MSL1	ENSG00000188895	17	38285795	rs41275668	17	38324788	G	A	0.034965	-0.0122	0.0377	0.7462353	-0.731242	0.0402073	6.57E-74	0.0166839	0.0515642	0.746274	0.08176173	0.07258377	18	FALSE	Vosa et al., 2018	Blood
ARID2	ENSG00000189079	12	46212635	rs11183272	12	46382256	C	A	0.480769	0.0443	0.0095	3.11E-06	-0.0605062	0.00894058	1.31E-11	-0.732156	0.190672	0.0001231	0.001674695	0.00171554	17	FALSE	Vosa et al., 2018	Blood
SUPT3H	ENSG00000196284	6	45061372	rs9395084	6	45220175	C	T	0.372378	0.0079	0.0099	0.4248822	-0.450872	0.00899831	0	-0.0175216	0.0219602	0.4249409	0.983986	0.1107252	20	FALSE	Vosa et al., 2018	Blood
WDR5	ENSG00000196363	9	137013151	rs28604373	9	137018130	A	G	0.479021	0.0277	0.0115	0.01600964	0.122369	0.0090284	7.52E-42	0.226364	0.0954505	0.01771424	0.017124	0.7487156	20	FALSE	Vosa et al., 2018	Blood
TRRAP	ENSG00000196367	7	98543211	rs12532598	7	98454973	C	T	0.13986	0.0172	0.0157	0.2732795	0.128689	0.0118183	1.30E-27	0.133655	0.122615	0.2756962	0.02863488	0.1165535	20	FALSE	Vosa et al., 2018	Blood
NCOR2	ENSG00000196498	12	124930548	rs1271309	12	124820705	A	G	0.162587	0.0112	0.0131	0.3925723	0.161135	0.0133587	1.67E-33	0.0695067	0.081502	0.3937579	0.09589916	0.1161979	20	FALSE	Vosa et al., 2018	Blood
HIST1H2AG	ENSG00000196787	6	27101951	rs112108849	6	27196744	A	G	0.0314685	-0.0987	0.0325	0.00239006	0.260424	0.0350852	1.15E-13	-0.378998	0.134838	0.00494245	0.002122028	0.00113563	4	FALSE	Vosa et al., 2018	Blood
HIST1H3D	ENSG00000197409	6	26198294	rs112751982	6	26191958	T	C	0.157343	-0.0404	0.0147	0.00599053	-0.0815559	0.0126033	9.74E-11	0.495365	0.195827	0.01141898	0.028221	0.02983773	9	FALSE	Vosa et al., 2018	Blood
PHF2	ENSG00000197724	9	96390279	rs12339150	9	96424789	A	G	0.255245	0.0116	0.0108	0.2827895	-0.14685	0.010129	1.25E-47	-0.0789924	0.0737462	0.2841071	0.1601971	0.4121478	20	FALSE	Vosa et al., 2018	Blood
HIST1H2BK	ENSG00000197903	6	27110346	rs12192049	6	27056739	C	T	0.0804196	0.0489	0.0164	0.00286646	0.712042	0.0176943	0	0.0686757	0.0230955	0.00294371	7.42E-09	0.00694096	20	TRUE	Vosa et al., 2018	Blood
PRMT6	ENSG00000198890	1	107600591	rs1730850	1	107597988	C	T	0.33042	0.0035	0.0105	0.7388827	-0.825013	0.00936494	0	-0.0042424	0.0127272	0.7388845	0.9795031	0.6145883	20	FALSE	Vosa et al., 2018	Blood
HIST2H2BF	ENSG00000203814	1	149769086	rs67567469	1	149818561	A	G	0.0402098	-0.0818	0.0305	0.00731906	0.141704	0.0150794	5.60E-21	-0.577258	0.223831	0.00990899	0.01235346	0.01835384	3	FALSE	Vosa et al., 2018	Blood
EHMT2	ENSG00000204371	6	31856500	rs2736428	6	31843924	T	C	0.333916	-0.0466	0.0103	6.06E-06	-0.0758847	0.00989241	1.71E-14	0.61409	0.157581	9.74E-05	8.12E-06	0.433706	20	TRUE	Vosa et al., 2018	Blood
HIST1H2BN	ENSG00000233822	6	27813148	rs111718978	6	27162514	G	A	0.0227273	-0.1158	0.0501	0.02081203	-0.318543	0.0508464	3.73E-10	0.36353	0.167642	0.03012105	0.04014739	0.2021641	5	FALSE	Vosa et al., 2018	Blood
H2AFJ	ENSG00000246705	12	14929103	rs7977737	12	14917129	T	G	0.124126	-0.0325	0.0142	0.02209491	0.175854	0.0140272	4.71E-36	-0.184813	0.0820837	0.0243527	0.0189517	0.8550354	20	FALSE	Vosa et al., 2018	Blood

CLASS B 2 SECRETIN FAMILY RECEPTORS (REACTOME)

Gene	probeID	ProbeChr	Probe bp	topSNP	topSNP chr	topSNP bp	A1	A2	Freq	b GWAS	se GWAS	p GWAS	b eQTL	se eQTL	p eQTL	b SMR	se SMR	p SMR	p SMR multi	p HEIDI	nsnp HEIDI	PASS BONF	Data source	Analyte
GIPR	ENSG00000010310	19	46179242	rs35845603	19	46160458	G	A	0.34965	0.0316	0.0101	0.00175574	-0.292551	0.0486049	1.76E-09	-0.108015	0.0389096	0.00550215	0.002563811	0.2786029	14	TRUE	Qi et al., 2018	Brain
GLP2R	ENSG00000065325	17	9760471	rs4239109	17	9741032	T	C	0.903846	0.0019	0.0208	0.9272175	0.631086	0.073849	1.28E-17	0.00301068	0.0329609	0.9272217	0.9208298	0.8460386	7	FALSE	Qi et al., 2018	Brain
GNB5	ENSG00000069966	15	52448341	rs12438190	15	52439065	T	C	0.88986	-0.004	0.0136	0.768668	0.378276	0.0563092	1.84E-11	-0.0105743	0.035987	0.7688831	0.8561139	0.72295	20	FALSE	Qi et al., 2018	Brain
FZD3	ENSG00000104290	8	28391752	rs10110026	8	28395405	C	G	0.403846	-0.0039	0.0095	0.6814199	0.858603	0.0273755	6.29E-216	-0.0045423	0.0110654	0.6814457	0.6747968	0.5615391	15	FALSE	Qi et al., 2018	Brain
VIPR2	ENSG00000106018	7	158879257	rs55807738	7	158915306	A	G	0.75	0.0067	0.0128	0.6006698	0.460599	0.0431175	1.23E-26	0.0145463	0.0278233	0.6011063	0.8474652	0.7820997	20	FALSE	Qi et al., 2018	Brain
CRHR2	ENSG00000106113	7	30715972	rs38413	7	30543249	T	C	0.27972	-0.011	0.0109	0.312891	0.342556	0.0483323	1.37E-12	-0.0321116	0.0321406	0.3177476	0.2502123	0.01553248	20	FALSE	Qi et al., 2018	Brain
WNT3	ENSG00000108379	17	44875196	rs12452064	17	44868187	A	G	0.437063	0.0502	0.0099	3.96E-07	-0.632378	0.0485953	1.03E-38	-0.0793829	0.0168017	2.30E-06	1.28E-09	0.00376694	20	TRUE	Qi et al., 2018	Brain
GNB3	ENSG00000111664	12	6952837	rs6489736	12	6944846	A	G	0.40035	-0.0081	0.0098	0.4085032	0.963183	0.0277621	9.64E-264	-0.0084096	0.0101775	0.4086361	0.2349025	0.3948763	15	FALSE	Qi et al., 2018	Brain
PTCH2	ENSG00000117425	1	45297125	rs72686491	1	45944106	G	T	0.952797	-0.0247	0.0215	0.2506231	-0.663188	0.119984	3.25E-08	0.0372443	0.033112	0.2606747	0.2606747	0.6051758	5	FALSE	Qi et al., 2018	Brain
SMO	ENSG00000128602	7	128841049	rs62480578	7	128837294	G	A	0.454545	-0.0027	0.0101	0.7892176	-0.242371	0.0429197	1.63E-08	0.01114	0.0417183	0.7894478	0.7894478	0.1659355	20	FALSE	Qi et al., 2018	Brain
WNT2B	ENSG00000134245	1	113037225	rs10857961	1	113082153	A	C	0.199301	0.0112	0.0117	0.3384335	0.858478	0.0484866	3.81E-70	0.0130464	0.0136487	0.3391385	0.6417635	0.559383	20	FALSE	Qi et al., 2018	Brain
IHH	ENSG00000163501	2	219922165	rs897476	2	219955093	A	G	0.678322	0.002	0.0099	0.8399009	0.371663	0.0632626	4.23E-09	0.00538121	0.0266527	0.8399942	0.793885	0.3721171	20	FALSE	Qi et al., 2018	Brain
FZD6	ENSG00000164930	8	104327877	rs827538	8	104299079	A	G	0.727273	0.0232	0.0107	0.03014162	0.371482	0.0523086	1.23E-12	0.0624525	0.0301161	0.03810462	0.05516363	0.7087446	20	FALSE	Qi et al., 2018	Brain
GNG4	ENSG00000168243	1	235762520	rs7555242	1	235798566	A	G	0.38986	-0.0313	0.0106	0.00314875	0.304335	0.0356061	1.26E-17	-0.102847	0.03685	0.00525499	0.007557897	0.9691893	18	FALSE	Qi et al., 2018	Brain
WNT10B	ENSG00000169884	12	49362334	rs1690182	12	49356142	G	T	0.465035	0.0011	0.0095	0.9078194	-0.331943	0.0308341	5.01E-27	-0.0033138	0.028621	0.9078247	0.8141299	0.3559964	20	FALSE	Qi et al., 2018	Brain
GNG5	ENSG00000174021	1	84968128	rs11163985	1	84971833	G	C	0.896853	0.0172	0.0181	0.3419719	-0.330554	0.0593443	2.55E-08	-0.0520338	0.0555476	0.3488913	0.3488913	0.3112246	9	FALSE	Qi et al., 2018	Brain
GNG7	ENSG00000176533	19	2606962	rs56211570	19	2518483	A	C	0.659091	0.0029	0.0115	0.8009066	0.335308	0.0398963	4.30E-17	0.00864876	0.0343122	0.8009943	0.9012446	0.6357405	20	FALSE	Qi et al., 2018	Brain
WNT8B	ENSG00000075290.7	10	102233149	rs11190520	10	102175309	C	T	0.234266	-0.0157	0.0112	0.1609792	1.45637	0.222602	6.05E-11	-0.0107802	0.00786489	0.1704756	0.189215	0.9621345	4	FALSE	GTEx Consortium	Substantia Nigra
WNT3	ENSG00000108379.5	17	44875148	rs9904865	17	44908263	T	C	0.636364	0.0622	0.0106	4.41E-09	-0.757966	0.0970181	5.60E-15	-0.0820617	0.0174901	2.71E-06	4.66E-06	0.3283538	4	TRUE	GTEx Consortium	Substantia Nigra
GNB3	ENSG00000111664.6	12	6952966	rs10744717	12	6941654	C	T	0.531469	0.0076	0.0099	0.4426792	-0.525456	0.0925931	1.39E-08	-0.0144636	0.0190124	0.4468078	0.4468078	0.8128756	5	FALSE	GTEx Consortium	Substantia Nigra
CD55	ENSG00000196352	1	207514582	rs61821090	1	207582704	T	C	0.0944056	0.0263	0.0156	0.09181556	0.601031	0.063329	2.30E-21	0.0437582	0.0263617	0.09693231	0.1999508	0.6304809	19	FALSE	GTEx Consortium	Substantia Nigra
WNT16	ENSG00000002745	7	120973289	rs7808138	7	120836416	A	T	0.0594406	0.0345	0.0198	0.08143424	-0.119163	0.0162035	1.92E-13	-0.289518	0.170758	0.08998334	0.214705	0.1278298	16	FALSE	Vosa et al., 2018	Blood
GIPR	ENSG00000010310	19	46179242	rs2238689	19	46178661	C	T	0.402098	0.0243	0.0099	0.01410628	-0.0628482	0.00893542	2.01E-12	-0.386646	0.166839	0.02047741	0.000801139	0.2613462	16	TRUE	Vosa et al., 2018	Blood
CALCRL	ENSG00000064989	2	188260521	rs8176580	2	188335246	C	T	0.304196	0.0159	0.01	0.1118348	-0.222989	0.0102185	1.43E-105	-0.0713038	0.0449641	0.1127855	0.4500263	0.8304331	20	FALSE	Vosa et al., 2018	Blood
GNB5	ENSG00000069966	15	52448341	rs12396	15	52413316	C	G	0.66958	0.001	0.0103	0.922657	-0.296832	0.00948179	3.93E-215	-0.0033689	0.0346999	0.9226574	0.3298604	0.2402109	20	FALSE	Vosa et al., 2018	Blood
WNT11	ENSG00000085741	11	75909586	rs17749202	11	75897374	C	T	0.332168	0.0082	0.0105	0.4348305	0.221497	0.0110425	1.70E-89	0.0370208	0.0474406	0.4351783	0.4424841	0.6188895	20	FALSE	Vosa et al., 2018	Blood
GNAS	ENSG00000087460	20	57450510	rs7121	20	57478807	C	T	0.51049	-0.0129	0.0111	0.2451696	0.252167	0.00904677	5.56E-171	-0.0511565	0.0440567	0.2455795	0.8519242	0.9782212	20	FALSE	Vosa et al., 2018	Blood
FZD3	ENSG00000104290	8	28391752	rs1946583	8	28350753	T	C	0.138112	-0.0003	0.0154	0.9844578	-0.339998	0.0131213	4.88E-148	0.00088236	0.0452943	0.9844578	0.8125408	0.8773974	17	FALSE	Vosa et al., 2018	Blood
VIPR2	ENSG00000106018	7	158879257	rs9969287	7	159069049	G	T	0.234266	-0.0173	0.0131	0.1866312	0.197564	0.0133596	1.75E-49	-0.0875665	0.0665714	0.1883835	0.811	0.876201	20	FALSE	Vosa et al., 2018	Blood
CRHR2	ENSG00000106113	7	30715972	rs1468402	7	30668882	A	G	0.653846	0.0171	0.0099	0.08411869	-0.120751	0.00894714	1.65E-41	-0.141614	0.0826559	0.08665775	0.1991266	0.01324326	20	FALSE	Vosa et al., 2018	Blood
WNT3	ENSG00000108379	17	44875196	rs199530	17	44836653	G	A	0.29021	-0.1976	0.0118	6.08E-63	0.136377	0.00891666	8.31E-53	-1.44893	0.128301	1.42E-29	2.38E-16	1.54E-11	16	TRUE	Vosa et al., 2018	Blood
GNB3	ENSG00000111664	12	6952837	rs1047776	12	6948692	A	G	0.346154	-0.0022	0.0101	0.827568	0.156653	0.0103318	6.29E-52	-0.0140438	0.0644802	0.8275855	0.3438407	0.2708241	20	FALSE	Vosa et al., 2018	Blood
GLP1R	ENSG00000112164	6	39036046	rs9283907	6	39026703	A	G	0.159091	0.0029	0.014	0.8358983	0.109923	0.0136128	6.75E-16	0.026382	0.127403	0.8359515	0.9221131	0.7800742	10	FALSE	Vosa et al., 2018	Blood
GNB4	ENSG00000114450	3	179143184	rs2111279	3	179166083	A	G	0.445804	0.0122	0.0108	0.2586323	-0.0940119	0.00892563	6.10E-26	-0.129771	0.115538	0.2613575	0.3673728	0.6835772	20	FALSE	Vosa et al., 2018	Blood
VIPR1	ENSG00000114812	3	42554925	rs342519	3	42532538	G	A	0.328671	0.01	0.0104	0.3362815	-0.519275	0.0102462	0	-0.0192576	0.0200315	0.3363685	0.3109055	0.345817	20	FALSE	Vosa et al., 2018	Blood
PTCH2	ENSG00000117425	1	45297125	rs11808189	1	45230542	A	G	0.339161	-0.0235	0.01	0.01877341	-0.0719403	0.00897033	1.06E-15	0.32666	0.144849	0.02412235	0.03464167	0.6088616	20	FALSE	Vosa et al., 2018	Blood
RAMP3	ENSG00000122679	7	45211645	rs1294863	7	45257184	G	A	0.183566	-0.0047	0.0126	0.7091366	0.285128	0.00902222	3.38E-219	-0.0164839	0.0441938	0.709156	0.4404675	0.935797	20	FALSE	Vosa et al., 2018	Blood
ADGRE5	ENSG00000123146	19	14505425	rs62122578	19	14493302	A	G	0.244755	0.0144	0.0127	0.256854	0.136487	0.01142	6.37E-33	0.105504	0.0934668	0.2589865	0.8304522	0.5965656	7	FALSE	Vosa et al., 2018	Blood
ADGRE2	ENSG00000127507	19	14866279	rs6511971	19	14877630	A	T	0.624126	0.0036	0.01	0.7188471	-0.680253	0.008259	0	-0.0052922	0.0147006	0.7188497	0.8107661	0.9242744	20	FALSE	Vosa et al., 2018	Blood
GNG11	ENSG00000127920	7	93553421	rs180279	7	93533816	G	T	0.0839161	0.0161	0.0178	0.3657333	-0.25958	0.0155246	9.28E-63	-0.0620232	0.0686725	0.3664336	0.4498315	0.2291208	14	FALSE	Vosa et al., 2018	Blood
ADM2	ENSG00000128165	22	50922427	rs57949435	22	50911404	T	G	0.108392	0.0067	0.0155	0.6655539	0.141043	0.0121442	3.50E-31	0.0475033	0.109972	0.6657712	0.08394082	0.04961808	20	FALSE	Vosa et al., 2018	Blood
SMO	ENSG00000128602	7	128841049	rs2718102	7	128810971	T	C	0.215035	0.0041	0.013	0.7524696	-0.0983573	0.0107494	5.69E-20	-0.0416847	0.13225	0.7526118	0.2993799	0.09504452	20	FALSE	Vosa et al., 2018	Blood
ADGRE3	ENSG00000131355	19	14765384	rs112998291	19	14724007	G	A	0.402098	0.0172	0.0099	0.08232122	-0.30321	0.00923935	3.35E-236	-0.0567264	0.0326964	0.0827507	0.5228631	0.6173883	20	FALSE	Vosa et al., 2018	Blood
RAMP1	ENSG00000132329	2	238794146	rs895572	2	238815252	T	C	0.566434	-0.0137	0.0101	0.1749606	0.248718	0.00887404	7.49E-173	-0.0550825	0.0406558	0.1754654	0.4691201	0.6685674	20	FALSE	Vosa et al., 2018	Blood
PTH2R	ENSG00000144407	2	209471832	rs6733487	2	208966158	G	A	0.451049	0.0186	0.0102	0.06822325	0.0833987	0.00895297	1.22E-20	0.223025	0.124625	0.07352427	0.04861133	0.03444948	11	FALSE	Vosa et al., 2018	Blood
ADM	ENSG00000148926	11	10327585	rs10840385	11	10307636	A	T	0.56993	-0.0064	0.0099	0.5179785	0.37954	0.00866448	0	-0.0168625	0.026087	0.5180241	0.6267785	0.6436303	20	FALSE	Vosa et al., 2018	Blood
WNT7A	ENSG00000154764	3	13889686	rs9310431	3	13811594	G	T	0.347902	0.0099	0.0108	0.3593173	-0.152672	0.0104624	3.14E-48	-0.0648449	0.0708793	0.3602634	0.8284685	0.5416864	20	FALSE	Vosa et al., 2018	Blood
FZD7	ENSG00000155760	2	202901235	rs13034579	2	202901279	T	G	0.27972	0.0079	0.0108	0.4644851	0.100997	0.0114134	8.83E-19	0.0782199	0.107298	0.4660047	0.03471343	0.02617865	20	FALSE	Vosa et al., 2018	Blood
FZD1	ENSG00000157240	7	90895953	rs1429535	7	90955960	G	T	0.199301	-0.0107	0.0125	0.3919978	-0.565629	0.0119962	0	0.018917	0.0221029	0.3920758	0.3448702	0.1996901	20	FALSE	Vosa et al., 2018	Blood
FZD6	ENSG00000164930	8	104327877	rs1403294	8	104345778	G	A	0.300699	-0.0235	0.0103	0.02251572	-0.525676	0.0102332	0	0.0447044	0.0196131	0.02264886	0.343116	0.3958929	20	FALSE	Vosa et al., 2018	Blood
WNT10B	ENSG00000169884	12	49362334	rs833840	12	49366150	C	G	0.557692	0.001	0.0098	0.9187243	0.14706	0.00914323	3.30E-58	0.00679996	0.0666409	0.9187259	0.02537038	0.06893706	20	FALSE	Vosa et al., 2018	Blood
GNB2	ENSG00000172354	7	100273975	rs117756744	7	100277212	A	G	0.00874126	0.0258	0.0567	0.6490902	-0.306488	0.056002	4.43E-08	-0.0841795	0.185638	0.6502162	0.6502162			FALSE	Vosa et al., 2018	Blood
GNG5	ENSG00000174021	1	84968128	rs10874446	1	84933846	T	G	0.0769231	-0.0116	0.0208	0.5770545	0.343549	0.014029	1.96E-132	-0.0337652	0.0605603	0.5771532	0.06459577	0.03496893	20	FALSE	Vosa et al., 2018	Blood
ADGRE1	ENSG00000174837	19	6914023	rs330887	19	6900955	C	T	0.277972	-0.0067	0.0105	0.5234117	0.567454	0.00822982	0	-0.0118071	0.0185045	0.5234295	0.5215233	0.2494853	20	FALSE	Vosa et al., 2018	Blood
GNG7	ENSG00000176533	19	2606962	rs6510696	19	2677012	T	C	0.243007	0.0391	0.012	0.00112069	-0.276379	0.00894183	9.17E-210	-0.141472	0.0436592	0.00119371	0.001192676	0.239439	20	TRUE	Vosa et al., 2018	Blood
FZD8	ENSG00000177283	10	35928769	rs604652	10	35942907	C	A	0.216783	-0.0183	0.0122	0.1336144	0.058212	0.00935343	4.86E-10	-0.314368	0.21558	0.1447735	0.08344234	0.5551261	12	FALSE	Vosa et al., 2018	Blood
FZD2	ENSG00000180340	17	42635916	rs11867858	17	42688460	T	C	0.194056	0.0253	0.0118	0.03202745	0.441101	0.0105712	0	0.0573564	0.0267865	0.03225445	5.55E-08	0.06236095	20	TRUE	Vosa et al., 2018	Blood
PTCH1	ENSG00000185920	9	98242300	rs59925771	9	98210941	T	G	0.118881	0.0293	0.0171	0.08662973	0.457421	0.0129372	7.82E-274	0.0640547	0.0374273	0.08699959	0.04445043	0.7526666	20	FALSE	Vosa et al., 2018	Blood
GNG2	ENSG00000186469	14	52369486	rs7145255	14	52328502	G	A	0.113636	-0.016	0.0156	0.3050608	0.278423	0.0169745	1.84E-60	-0.0574666	0.0561394	0.3060045	0.5878483	0.4335894	20	FALSE	Vosa et al., 2018	Blood
CD55	ENSG00000196352	1	207514582	rs891376	1	207491489	T	C	0.277972	0.0007	0.0106	0.9473478	-0.701086	0.00835422	0	-0.0009985	0.0151194	0.9473478	0.4172905	0.515627	20	FALSE	Vosa et al., 2018	Blood

CLATHRIN MEDIATED ENDOCYTOSIS (REACTOME)

Gene	probeID	ProbeChr	Probe bp	topSNP	topSNP chr	topSNP bp	A1	A2	Freq	b GWAS	se GWAS	p GWAS	b eQTL	se eQTL	p eQTL	b SMR	se SMR	p SMR	p SMR multi	p HEIDI	nsnp HEIDI	PASS BONF	Data source	Analyte
CFTR	ENSG00000001626	7	117230931	rs10255092	7	117012241	A	G	0.715035	0.0175	0.0108	0.1051527	0.339645	0.0492271	5.22E-12	0.0515244	0.032663	0.1146916	0.06391268	0.4927696	13	FALSE	Qi et al., 2018	Brain
M6PR	ENSG00000003056	12	9097755	rs11494758	12	9116542	T	C	0.382867	0.0029	0.0099	0.7695762	1.0743	0.0303306	8.53E-275	0.00269944	0.00921565	0.7695839	0.2513595	0.1308319	20	FALSE	Qi et al., 2018	Brain
CD4	ENSG00000010610	12	6912999	rs10774447	12	6894449	T	C	0.711538	-0.014	0.0103	0.1740758	-0.301204	0.0492543	9.64E-10	0.0464801	0.0350306	0.1845604	0.2499093	0.8630806	8	FALSE	Qi et al., 2018	Brain
VAMP3	ENSG00000049245	1	7836410	rs1048785	1	7841330	G	A	0.872378	-0.0132	0.0151	0.3820245	-0.680838	0.0667454	1.96E-24	0.0193879	0.0222598	0.383765	0.301999	0.4502215	20	FALSE	Qi et al., 2018	Brain
CLTCL1	ENSG00000070371	22	19223112	rs68149483	22	19372712	C	T	0.90035	0.0166	0.0166	0.3173105	-1.00554	0.0669564	5.62E-51	-0.0165086	0.0165452	0.318381	0.1891803	0.0041404	20	FALSE	Qi et al., 2018	Brain
SYNJ2	ENSG00000078269	6	158461548	rs2104186	6	158468771	T	C	0.424825	0.0079	0.0108	0.4644851	0.346431	0.038192	1.18E-19	0.022804	0.0312763	0.4659316	0.2304707	0.2196451	20	FALSE	Qi et al., 2018	Brain
APOB	ENSG00000084674	2	21245623	rs1367117	2	21263900	A	G	0.304196	-0.0088	0.0102	0.3882776	-0.423589	0.0685124	6.30E-10	0.0207749	0.0243133	0.3928468	0.3464219	0.7455607	14	FALSE	Qi et al., 2018	Brain

PACSIN2	ENSG00000100266	22	43321284	rs5759094	22	43410235	T	C	0.449301	-0.0172	0.0098	0.07924186	-0.256535	0.0386806	3.31E-11	0.0670475	0.0395165	0.08975424	0.0354929	0.05380128	20	FALSE	Qi et al., 2018	Brain
DNM1	ENSG00000106976	9	130991592	rs10760546	9	130957275	T	A	0.370629	-0.0177	0.0099	0.07379557	0.290779	0.043169	1.63E-11	-0.060871	0.0352254	0.0839806	0.3069471	0.01942137	20	FALSE	Qi et al., 2018	Brain
SH3GL2	ENSG00000107295	9	17688103	rs10756899	9	17684784	G	C	0.781469	-0.0836	0.0112	8.38E-14	-0.331527	0.0397159	6.97E-17	0.252166	0.0453196	2.63E-08	9.04E-08	0.07186738	20	TRUE	Qi et al., 2018	Brain
RAB5C	ENSG00000108774	17	40292014	rs9915255	17	40284191	C	T	0.762238	0.0148	0.0112	0.1863585	-0.276341	0.0440428	3.51E-10	-0.053557	0.0414187	0.1959894	0.3442227	0.6618252	14	FALSE	Qi et al., 2018	Brain
CBL	ENSG00000110395	11	119127201	rs2509671	11	119175075	C	A	0.687063	0.0163	0.0106	0.1241132	-0.357962	0.0504443	1.28E-12	-0.0455355	0.0302993	0.1328765	0.05677834	0.00278764	16	FALSE	Qi et al., 2018	Brain
AC034102.1	ENSG00000255990	12	56387230	rs10431546	12	55745850	C	G	0.0996503	-0.0277	0.0177	0.1175895	0.43878	0.0775197	1.51E-08	-0.0631296	0.0418526	0.1314573	0.1314573	0.6647162	3	FALSE	Qi et al., 2018	Brain
STAM2	ENSG00000115145	2	153002910	rs35717822	2	152971605	T	G	0.783217	0.0043	0.0123	0.7266438	0.463005	0.0628876	1.81E-13	0.00928715	0.0265955	0.7269391	0.5695114	0.4470448	20	FALSE	Qi et al., 2018	Brain
DNAJC6	ENSG00000116675	1	65797727	rs9436726	1	65853196	C	T	0.566434	0.007	0.0098	0.4750505	-0.336472	0.0424182	2.15E-15	-0.0208041	0.0292436	0.4768319	0.101935	0.00456769	20	FALSE	Qi et al., 2018	Brain
SGIP1	ENSG00000118473	1	67106524	rs10889647	1	67154496	T	A	0.75	-0.016	0.0109	0.1421341	-0.390024	0.0503302	9.24E-15	0.0410231	0.0284439	0.1492337	0.2045495	0.01643829	20	FALSE	Qi et al., 2018	Brain
VAMP8	ENSG00000118640	2	85798919	rs1446669	2	85762209	T	C	0.673077	0.0211	0.0103	0.04050676	0.285194	0.0505642	1.70E-08	0.0739847	0.0384241	0.05416979	0.05416979	0.1803202	18	FALSE	Qi et al., 2018	Brain
CLTA	ENSG00000122705	9	36247815	rs7041383	9	36204912	C	T	0.875874	0.0083	0.0169	0.6233386	0.558835	0.0803854	3.60E-12	0.0148523	0.0303168	0.6242023	0.570455	0.1781766	10	FALSE	Qi et al., 2018	Brain
SNX9	ENSG00000130340	6	158305195	rs9347695	6	158267449	G	A	0.75	-0.0177	0.0117	0.1303253	0.257113	0.043499	3.40E-09	-0.0688414	0.0469721	0.1427629	0.1441248	0.4239413	14	FALSE	Qi et al., 2018	Brain
STAM	ENSG00000136738	10	17722018	rs4748397	10	17712297	G	A	0.872378	0.0034	0.0164	0.8357622	0.609057	0.0770103	2.60E-15	0.0055824	0.0269361	0.8358178	0.9149557	0.6977201	13	FALSE	Qi et al., 2018	Brain
TOR1B	ENSG00000136816	9	132569496	rs2062798	9	132594718	T	C	0.725524	-0.0296	0.0112	0.00822097	-0.511864	0.0546109	7.05E-21	0.0578279	0.022734	0.01096937	0.004254939	0.546765	20	FALSE	Qi et al., 2018	Brain
TOR1A	ENSG00000136827	9	132580818	rs13283293	9	132574477	G	A	0.713287	-0.0272	0.0111	0.01426776	1.28562	0.0342522	2.59E-308	-0.0211572	0.00865238	0.01447539	0.006742217	0.09684349	20	FALSE	Qi et al., 2018	Brain
SLC2A8	ENSG00000136856	9	130165062	rs10819289	9	130172319	A	T	0.379371	0.0021	0.0118	0.8587496	1.14697	0.0308993	1.36E-301	0.00183091	0.0102881	0.8587512	0.1338931	0.01964566	20	FALSE	Qi et al., 2018	Brain
ACTR2	ENSG00000138071	2	65476637	rs268858	2	65471137	A	T	0.576923	0.0024	0.0098	0.8065354	0.549254	0.0476491	9.64E-31	0.00436956	0.0178464	0.8065782	0.07609835	0.03284262	20	FALSE	Qi et al., 2018	Brain
SCARB2	ENSG00000138760	4	77107468	rs1348207	4	77167499	T	A	0.646853	-0.0743	0.0096	9.97E-15	-0.27934	0.037075	4.90E-14	0.265984	0.0492678	6.71E-08	8.89E-08	2.78E-05	20	TRUE	Qi et al., 2018	Brain
SH3GL3	ENSG00000140600	15	84201737	rs2585071	15	84264404	G	A	0.314685	0.0063	0.0103	0.540769	0.372552	0.0476379	5.26E-15	0.0169104	0.0277316	0.5420021	0.4040693	0.1502288	20	FALSE	Qi et al., 2018	Brain
RAB5A	ENSG00000144566	3	20007619	rs6550237	3	20001194	A	T	0.743007	0.0036	0.0117	0.7583165	-0.494703	0.0523673	3.49E-21	-0.0072771	0.0236631	0.7584406	0.2753641	0.06392346	20	FALSE	Qi et al., 2018	Brain
EGFR	ENSG00000146648	7	55205513	rs6979446	7	55015505	C	T	0.910839	-0.0052	0.0207	0.8016535	0.551042	0.0721134	2.15E-14	-0.0094367	0.0375855	0.8017584	0.4287651	0.348635	20	FALSE	Qi et al., 2018	Brain
LDLRAP1	ENSG00000157978	1	25882724	rs6688931	1	25889672	A	G	0.491259	-0.0145	0.0094	0.1229392	-0.979336	0.0273192	1.99E-281	0.0148059	0.00960722	0.1232859	0.1942883	0.5623463	10	FALSE	Qi et al., 2018	Brain
TGFA	ENSG00000163235	2	70727868	rs7571789	2	70714793	C	T	0.498252	0.0138	0.01	0.1675866	0.300217	0.035237	1.60E-17	0.0459668	0.0337434	0.1731202	0.6071758	0.3105991	20	FALSE	Qi et al., 2018	Brain
ARPC2	ENSG00000163466	2	219100448	rs1877715	2	219052546	A	G	0.590909	0.0308	0.0099	0.00186385	0.243529	0.0446497	4.92E-08	0.126473	0.0468005	0.00688423	0.006884227	0.5907526	6	FALSE	Qi et al., 2018	Brain
GAK	ENSG00000178950	4	884612	rs11248057	4	906131	C	G	0.702797	-0.1531	0.013	5.13E-32	-0.301155	0.0552209	4.93E-08	0.508376	0.102727	7.47E-07	7.47E-07	0.0369756	20	TRUE	Qi et al., 2018	Brain
SNX18	ENSG00000178996	5	53828002	rs13188020	5	53833021	A	T	0.781469	-0.0129	0.0122	0.2903395	0.417096	0.048506	8.05E-18	-0.0309282	0.0294702	0.2939608	0.1665751	0.3143101	20	FALSE	Qi et al., 2018	Brain
AP2A2	ENSG00000183020	11	968566	rs66670048	11	999764	T	C	0.905594	-0.0131	0.0178	0.461758	0.65027	0.0691272	5.11E-21	-0.0201455	0.0274569	0.4631237	0.918838	0.1335585	7	FALSE	Qi et al., 2018	Brain
IGF2R	ENSG00000197081	6	160462335	rs755884	6	160524578	G	C	0.251748	-0.014	0.0106	0.1865832	-0.310646	0.0484586	1.45E-10	0.0450674	0.0348391	0.1958087	0.3515865	0.5619807	20	FALSE	Qi et al., 2018	Brain
DNM3	ENSG00000197959	1	172099113	rs9425308	1	172383265	A	C	0.305944	-0.0261	0.0103	0.0112775	-0.296366	0.0447271	3.45E-11	0.0880668	0.037209	0.01794201	0.02042339	0.2218689	19	FALSE	Qi et al., 2018	Brain
VAMP2	ENSG00000220205	17	8064665	rs8064449	17	8079029	C	G	0.618881	0.0262	0.0101	0.00948501	0.658922	0.059324	1.16E-28	0.0397619	0.0157406	0.01153426	0.003542877	0.5685108	20	FALSE	Qi et al., 2018	Brain
UBA52	ENSG00000221983	19	18685450	rs10409392	19	18699333	A	C	0.458042	0.002	0.0097	0.836646	0.322222	0.0488456	4.20E-11	0.0062069	0.0301182	0.8367246	0.9599301	0.3329377	20	FALSE	Qi et al., 2018	Brain
LDLRAP1	ENSG00000157978.7	1	25882724	rs6688931	1	25889672	A	G	0.491259	-0.0145	0.0094	0.1229392	-0.431551	0.0685677	3.10E-10	0.0335997	0.0224266	0.1340785	0.1438711	0.6296735	4	FALSE	GTEx Consortium	Substantia Nigra
STON1	ENSG00000243244	2	48791273	rs62135201	2	48780425	G	A	0.704545	-0.0062	0.0102	0.5432915	-0.526098	0.0493878	1.70E-26	0.0117849	0.0194196	0.5439465	0.6815522	0.7271837	20	FALSE	GTEx Consortium	Substantia Nigra
M6PR	ENSG00000003056	12	9097755	rs11048241	12	9110832	A	G	0.356643	0.0052	0.0101	0.6066568	0.431907	0.00984622	0	0.0120396	0.0233863	0.6066816	0.01998627	0.3034987	20	FALSE	Vosa et al., 2018	Blood
DVL2	ENSG00000004975	17	7133262	rs2654185	17	7183362	A	C	0.402098	-0.0294	0.0099	0.00298094	0.122184	0.00893759	1.52E-42	-0.240621	0.082915	0.0037077	0.000422902	0.1412221	20	TRUE	Vosa et al., 2018	Blood
AP2B1	ENSG00000006125	17	33979250	rs321600	17	33905468	A	G	0.232517	0.0131	0.0116	0.2587669	-0.272291	0.0127332	1.87E-101	-0.0481103	0.0426609	0.2594302	0.01519758	0.00318242	20	FALSE	Vosa et al., 2018	Blood
CD4	ENSG00000010610	12	6912999	rs7296859	12	6894164	C	G	0.361888	-0.0168	0.0108	0.1198138	0.259706	0.0100973	6.91E-146	-0.0646886	0.0416615	0.1204904	0.5778693	0.7621564	14	FALSE	Vosa et al., 2018	Blood
PIK3C2A	ENSG00000011405	11	17164403	rs12577388	11	17101155	T	C	0.270979	0.004	0.0105	0.7032386	-0.530592	0.00967654	0	-0.0075388	0.0197897	0.7032454	0.8978044	0.6464649	20	FALSE	Vosa et al., 2018	Blood
AP2S1	ENSG00000042753	19	47347821	rs311379	19	47441846	C	G	0.566434	-0.0194	0.0095	0.04114109	0.162911	0.00895234	5.39E-74	-0.119083	0.05868	0.04242089	0.08909712	0.193584	20	FALSE	Vosa et al., 2018	Blood
VAMP3	ENSG00000049245	1	7836410	rs11590868	1	7833951	G	T	0.176573	0.021	0.0122	0.08519432	0.484979	0.0142429	3.97E-254	0.0433008	0.0251878	0.08559319	0.4524107	0.3088894	20	FALSE	Vosa et al., 2018	Blood
EPN1	ENSG00000063245	19	56203908	rs73615245	19	56223555	A	G	0.0472028	-0.0018	0.0272	0.9472373	0.288905	0.0167763	1.85E-66	-0.0062304	0.0941492	0.9472377	0.9624708	0.6143443	20	FALSE	Vosa et al., 2018	Blood
SYT1	ENSG00000067715	12	79551780	rs67167203	12	79371460	T	C	0.312937	-0.0159	0.011	0.1483303	-0.133015	0.00967322	5.04E-43	0.119536	0.0831534	0.1505663	0.8065238	0.2347043	19	FALSE	Vosa et al., 2018	Blood
CLTCL1	ENSG00000070371	22	19223112	rs756651	22	19179367	T	C	0.379371	0.0285	0.0098	0.00363559	0.445311	0.0108178	0	0.0640002	0.022062	0.00372054	0.03431045	0.08407779	20	FALSE	Vosa et al., 2018	Blood
EPN2	ENSG00000072134	17	19179478	rs3785784	17	19195431	G	A	0.0734266	-0.0085	0.0198	0.6677101	0.131866	0.0122923	7.55E-27	-0.0644592	0.150272	0.6679599	0.1398878	0.1172599	20	FALSE	Vosa et al., 2018	Blood
TFRC	ENSG00000072274	3	195781557	rs28429906	3	195906747	G	A	0.234266	-0.0064	0.0124	0.6057643	-0.303072	0.00945801	2.67E-225	0.0211171	0.0409197	0.605811	0.3618159	0.85856	20	FALSE	Vosa et al., 2018	Blood
PICALM	ENSG00000073921	11	85724825	rs7131120	11	85690012	G	C	0.234266	-0.0076	0.012	0.526516	0.169259	0.0100584	1.53E-63	-0.0449015	0.0709474	0.5268086	0.4293038	0.00600192	20	FALSE	Vosa et al., 2018	Blood
ACTB	ENSG00000075624	7	5585098	rs4296949	7	5508644	C	T	0.335664	-0.0124	0.0105	0.2376216	-0.0542021	0.00962499	1.79E-08	0.228773	0.197933	0.2477585	0.2477585	0.6867758	7	FALSE	Vosa et al., 2018	Blood
AMPH	ENSG00000078053	7	38547236	rs2736947	7	38386791	A	G	0.222028	0.0003	0.0127	0.9811541	-0.0865848	0.0101625	1.60E-17	-0.0034648	0.146678	0.9811542	0.2942188	0.1467706	20	FALSE	Vosa et al., 2018	Blood
SYNJ2	ENSG00000078269	6	158461548	rs2758274	6	158504089	T	C	0.115385	0.0016	0.0162	0.9213245	1.04274	0.0157398	0	0.00153442	0.015536	0.9213246	0.6295801	0.8820505	20	FALSE	Vosa et al., 2018	Blood
DNM2	ENSG00000079805	19	10886459	rs1541596	19	10987013	G	A	0.534965	-0.0001	0.0097	0.9917745	-0.0647435	0.00934303	4.22E-12	0.00154456	0.149822	0.9917745	0.2081589	0.06157785	20	FALSE	Vosa et al., 2018	Blood
CTTN	ENSG00000085733	11	70263600	rs10431177	11	70261536	G	A	0.286713	0.0031	0.0118	0.7927727	0.116758	0.0110232	3.25E-26	0.0265506	0.101095	0.7928349	0.9484488	0.9406654	17	FALSE	Vosa et al., 2018	Blood
EPS15	ENSG00000085832	1	51902467	rs148516140	1	51928058	G	A	0.0437063	-0.0246	0.0217	0.2569454	0.575374	0.0340088	3.29E-64	-0.0427548	0.0377992	0.2580113	0.7863252	0.8514856	20	FALSE	Vosa et al., 2018	Blood
NECAP1	ENSG00000089818	12	8242587	rs2072448	12	8210697	A	G	0.312937	-0.0198	0.011	0.07186064	-0.458781	0.00848538	0	0.0431578	0.0239899	0.07201816	0.5466339	0.9509202	20	FALSE	Vosa et al., 2018	Blood
TF	ENSG00000091513	3	133481325	rs12634591	3	133367858	C	T	0.0926573	0.0313	0.0157	0.04619244	-0.190692	0.0112624	2.62E-64	-0.164139	0.0829003	0.04770882	0.3184751	0.4194726	20	FALSE	Vosa et al., 2018	Blood
GRK3	ENSG00000100077	22	26043037	rs113465681	22	26122573	T	C	0.0157343	0.0385	0.0472	0.4146843	0.366023	0.0270423	9.69E-42	0.105185	0.129188	0.4155299	0.377178	0.5703371	11	FALSE	Vosa et al., 2018	Blood
PACSIN2	ENSG00000100266	22	43321284	rs4822241	22	43369349	A	T	0.447552	-0.0145	0.0096	0.1309371	-0.186358	0.00929718	2.25E-89	0.0778071	0.0516597	0.1320295	0.03831553	0.00567731	20	FALSE	Vosa et al., 2018	Blood
ARFGAP1	ENSG00000101199	20	61912639	rs13042357	20	61916379	C	T	0.590909	0.0086	0.0102	0.3991517	0.113752	0.00916022	2.09E-35	0.0756033	0.0898755	0.4002356	0.7658326	0.8321593	20	FALSE	Vosa et al., 2018	Blood
WASL	ENSG00000106299	7	123355555	rs17146312	7	123395200	G	A	0.265734	-0.0107	0.0113	0.3436884	-0.182662	0.00969122	3.04E-79	0.0585781	0.0619409	0.3442965	0.8440908	0.5101367	20	FALSE	Vosa et al., 2018	Blood
DNM1	ENSG00000106976	9	130991592	rs10760548	9	130965235	G	A	0.00874126	-0.0689	0.0378	0.06834106	0.301054	0.0186702	1.71E-58	-0.228863	0.126359	0.07010741	0.0138652	0.4223475	20	FALSE	Vosa et al., 2018	Blood
RAB5C	ENSG00000108774	17	40292014	rs2285657	17	40272594	A	C	0.237762	-0.0145	0.0114	0.2033981	0.13531	0.00947479	2.87E-46	-0.107162	0.0845848	0.2051857	0.293168	0.559651	20	FALSE	Vosa et al., 2018	Blood
HSPA8	ENSG00000109971	11	122931067	rs11218941	11	122932409	G	A	0.188811	0.0052	0.013	0.6891565	0.10975	0.0095726	1.98E-30	0.0473805	0.118523	0.6893357	0.6449592	0.4454734	20	FALSE	Vosa et al., 2018	Blood
CBL	ENSG00000110395	11	119127201	rs2186882	11	119168370	G	A	0.695804	0.0186	0.0106	0.07930773	0.193778	0.00949705	1.54E-92	0.0959862	0.0549037	0.08041832	0.001652498	0.00284039	20	FALSE	Vosa et al., 2018	Blood
ARPC3	ENSG00000111229	12	110880428	rs3759384	12	110888857	T	C	0.356643	0.0075	0.0103	0.4665185	-0.142122	0.00908183	3.37E-55	-0.0527717	0.0725516	0.4670003	0.5487282	0.7385622	20	FALSE	Vosa et al., 2018	Blood
COPS7A	ENSG00000111652	12	6836974	rs7138427	12	6836852	A	G	0.215035	0.0149	0.0126	0.2369916	0.267429	0.0117885	6.22E-114	0.0557157	0.0471792	0.237628	0.7609674	0.6449434	20	FALSE	Vosa et al., 2018	Blood
HBEGF	ENSG00000113070	5	139719322	rs73257091	5	139664664	T	C	0.223776	-0.0115	0.0121	0.3419023	0.17726	0.0103119	3.17E-66	-0.0648765	0.0683656	0.342639	0.01280672	0.02558315	20	FALSE	Vosa et al., 2018	Blood
ACTR3	ENSG00000115091	2	114683855	rs10182655	2	114738509	T	C	0.0926573	0.0123	0.0161	0.444882	0.0783423	0.0135997	8.38E-09	0.157003	0.207308	0.4488438	0.4488438	0.7187632	9	FALSE	Vosa et al., 2018	Blood
STAM2	ENSG00000115145	2	153002910	rs10930967	2	153033620	C	T	0.777972	0.0016	0.0121	0.8948013	0.252361	0.00904247	2.11E-171	0.00634013	0.0479478	0.8948024	0.2944927	0.2919552	20	FALSE	Vosa et al., 2018	Blood
VAMP4	ENSG00000117533	1	171690343	rs10913587	1	171717417	T	C	0.178322	-0.067	0.0119	1.80E-08	0.246191	0.0123584	2.68E-88	-0.272146	0.0502299	6.03E-08	2.66E-07	0.05055051	20	TRUE	Vosa et al., 2018	Blood
SGIP1	ENSG00000118473	1	67106524	rs7544505	1	66998618	C	T	0.148601	-0.003	0.0133	0.8215406	-0.179382	0.0113639	3.93E-56	0.0167241	0.0741511	0.8215585	0.03267668	0.06592958	20	FALSE	Vosa et al., 2018	Blood
VAMP8	ENSG00000118640	2	85798919	rs1010	2	85808982	C	T	0.409091	-0.0309	0.0095	0.00114342	-0.428287	0.0085758	0	0.0721479	0.0222284	0.0011714	0.00507522	0.05092163	20	FALSE	Vosa et al., 2018	Blood
CLTA	ENSG00000122705	9	36247815	rs2025879	9	36204455	T	C	0.332168	0.0006	0.0105	0.9544314	0.101456	0.00920872	3.15E-28	0.00591389	0.103494	0.954432	0.1299417	0.1045278	20	FALSE	Vosa et al., 2018	Blood
PACSIN1	ENSG00000124507	6	34468461	rs56370046	6	34449264	A	G	0.512238	-0.0001	0.0104	0.9923282	-0.0901114	0.0091467	6.73E-23	0.00110974	0.115413	0.9923282	0.2807504	0.177821	20	FALSE	Vosa et al., 2018	Blood
EREG	ENSG00000124882	4	75242664	rs58813617	4	75196513	C	G	0.300699	-0.0059	0.0129	0.6474092	-0.138068	0.00900842	5.08E-53	0.0427324	0.0934735	0.6475554	0.7984215	0.3499884	20	FALSE	Vosa et al., 2018	Blood
TRIP10	ENSG00000125733	19	6744736	rs1049232	19	6751293	G	T	0.166084	0.0137	0.0134	0.3065973	-0.25128	0.0103168	4.96E-131	-0.0545208	0.0533739	0.307023	0.4221013	0.8018971	11	FALSE	Vosa et al., 2018	Blood
EPS15L1	ENSG00000127527	19	16524473	rs117989741	19	16463392	G	A	0.0384615	0.012	0.0319	0.7067864	-0.282812	0.0310933	9.41E-20	-0.042431	0.112892	0.7070253	0.3209055	0.1612475	14	FALSE	Vosa et al., 2018	Blood
HIP1	ENSG00000127946	7	75265450	rs1179623	7	75254834	C	G	0.452797	0.0228	0.0105	0.02989879	-0.846979	0.00727817	0	-0.0269192	0.0123992	0.02992734	0.03022993	0.1826144	20	FALSE	Vosa et al., 2018	Blood
NEDD8	ENSG00000129559	14	24693859	rs2766	14	24686145	T	C	0.0297203	-0.0098	0.0251	0.6962125	0.487383	0.0389489	6.31E-36	-0.0201074	0.0515246	0.6963529	0.9735437	0.9396146	10	FALSE	Vosa et al., 2018	Blood
LDLR	ENSG00000130164	19	11222265	rs73013176	19	11147526	C	T	0.00699301	0.0197	0.0576	0.7323404	0.543704	0.0790475	6.06E-12	0.0362329	0.106071	0.7326581	0.5530228			FALSE	Vosa et al., 2018	Blood
SNX9	ENSG00000130340	6	158305202	rs73012207	6	158377294	A	G	0.141608	0.0156	0.0155	0.3141984	0.124004	0.0192368	1.15E-10	0.125802	0.12651	0.3200261	0.2855048	0.4517011	14	FALSE	Vosa et al., 2018	Blood
FCHO1	ENSG00000130475	19	17878952	rs10422213	19	17826804	G	A	0.555944	0.0138	0.0099	0.1633359	-0.267692	0.00880081	3.30E-203	-0.0515518	0.0370216	0.1637777	0.2245183	0.6938012	20	FALSE	Vosa et al., 2018	Blood
HIP1R	ENSG00000130787	12	123333253	rs4426165	12	123293807	T	C	0.0804196	-0.0475	0.0191	0.01288576	0.163999	0.0140328	1.49E-31	-0.289636	0.119072	0.01499718	0.000159417	0.00144692	20	TRUE	Vosa et al., 2018	Blood
SYT11	ENSG00000132718	1	155842145	rs12024187	1	155777840	A	T	0.302448	0.0216	0.0128	0.09150725	-0.182043	0.00904815	4.99E-90	-0.118653	0.0705598	0.09264731	0.000484088	0.00010766	20	FALSE	Vosa et al., 2018	Blood
UBQLN1	ENSG00000135018	9	86298998	rs4146740	9	86244075	A	C	0.241259	-0.001	0.0109	0.9269021	0.110509	0.00893597	3.95E-35	-0.009049	0.0986368	0.9269041	0.1343658	0.4380766	20	FALSE	Vosa et al., 2018	Blood
REPS1	ENSG00000135597	6	139267014	rs76933614	6	139290015	C	T	0.0681818	-0.0246	0.0209	0.2391821	0.855379	0.0268197	3.27E-223	-0.0287592	0.0244503	0.2395019	0.1574784	0.05245889	20	FALSE	Vosa et al., 2018	Blood
BIN1	ENSG00000136717	2	127835267	rs11682128	2	127839474	A	G	0.36014	0.0243	0.0107	0.02314528	0.741675	0.00858815	0	0.0327637	0.0144318	0.02319283	0.01132408	0.5278145	20	FALSE	Vosa et al., 2018	Blood
STAM	ENSG00000136738	10	17722018	rs2570287	10	17726070	A	G	0.575175	-0.003	0.0102	0.768668	-0.194776	0.00898138	2.75E-104	0.0154023	0.0523728	0.7686887	0.7685467	0.2252396	20	FALSE	Vosa et al., 2018	Blood
TOR1B	ENSG00000136816	9	132569496	rs1831105	9	132588281	G	A	0.274476	0.0253	0.0113	0.02515995	-0.382671	0.0103424	1.14E-299	-0.0661142	0.0295833	0.02542719	0.02263399	0.04032073	20	FALSE	Vosa et al., 2018	Blood
TOR1A	ENSG00000136827	9	132580818	rs7025992	9	132557210	C	T	0.381119	0.0289	0.0102	0.00460653	-0.312434	0.0090402	9.92E-262	-0.0924996	0.0327565	0.00474492	0.00512674	0.2807156	20	FALSE	Vosa et al., 2018	Blood
SLC2A8	ENSG00000136856	9	130165062	rs11789887	9	130168840	A	G	0.620629	-0.0019	0.0118	0.8720801	-0.487853	0.00842492	0	0.00389461	0.0241877	0.8720805	0.009259988	0.1270927	20	FALSE	Vosa et al., 2018	Blood
KIAA0319	ENSG00000137261	6	24595357	rs6935076	6	24644322	T	C	0.312937	-0.0123	0.0101	0.2232917	0.326116	0.0100382	1.60E-231	-0.0377166	0.0309923	0.2236168	0.4165095	0.3550425	20	FALSE	Vosa et al., 2018	Blood

ARRB1	ENSG00000137486	11	75019049	rs12271945	11	75085563	A	G	0.117133	-0.0068	0.016	0.6708367	-0.368156	0.0152249	3.51E-129	0.0184704	0.0434665	0.6708845	0.7251257	0.5466856	20	FALSE	Vosa et al., 2018	Blood
FNBP1L	ENSG00000137942	1	93966938	rs12096122	1	93891708	C	T	0.0874126	0.0157	0.0182	0.3883369	0.226247	0.0152676	1.11E-49	0.0693931	0.0805791	0.3891392	0.04108761	0.1104124	20	FALSE	Vosa et al., 2018	Blood
ACTR2	ENSG00000138071	2	65476637	rs4143571	2	65503110	C	T	0.277972	-0.014	0.0104	0.1782529	-0.0902395	0.00903388	1.70E-23	0.155143	0.116291	0.1821731	0.2115537	0.3956686	20	FALSE	Vosa et al., 2018	Blood
SCARB2	ENSG00000138760	4	77107468	rs13111888	4	77131294	A	G	0.185315	0.0619	0.0123	4.84E-07	0.325881	0.011423	5.19E-179	0.189947	0.0383266	7.20E-07	2.35E-20	5.80E-12	20	TRUE	Vosa et al., 2018	Blood
EGF	ENSG00000138798	4	110883731	rs2298979	4	110838126	G	A	0.592657	0.0208	0.0096	0.03026028	-0.175414	0.00900593	1.70E-84	-0.118577	0.0550653	0.03128847	0.05853531	0.09619906	20	FALSE	Vosa et al., 2018	Blood
STON2	ENSG00000140022	14	81814904	rs8008602	14	81890171	T	A	0.277972	0.0077	0.0112	0.4917677	0.174004	0.0108604	9.00E-58	0.0442519	0.0644257	0.492166	0.9817379	0.9653819	20	FALSE	Vosa et al., 2018	Blood
COPS3	ENSG00000141030	17	17167374	rs1708626	17	17144908	G	A	0.445804	-0.0205	0.0096	0.03272699	-0.536888	0.00838547	0	0.038183	0.0178908	0.03282396	0.003175317	0.1688567	20	FALSE	Vosa et al., 2018	Blood
ARRB2	ENSG00000141480	17	4619289	rs55682338	17	4582183	A	G	0.211538	-0.008	0.0111	0.4710814	0.655176	0.0144935	0	-0.0122105	0.0169442	0.4711377	0.07159516	0.5718481	20	FALSE	Vosa et al., 2018	Blood
RAB5A	ENSG00000144566	3	20007619	rs13077307	3	20004642	T	C	0.589161	-0.0027	0.0104	0.7951605	0.221257	0.00902015	7.21E-133	-0.012203	0.0470068	0.7951717	0.0880786	0.04138142	20	FALSE	Vosa et al., 2018	Blood
UBC	ENSG00000150991	12	125399032	rs4617684	12	125406966	A	G	0.00174825	0.072	0.1032	0.4853808	-0.0842798	0.011017	2.01E-14	-0.854298	1.22958	0.4871863	0.7652337			FALSE	Vosa et al., 2018	Blood
TGOLN2	ENSG00000152291	2	85550347	rs2271625	2	85625513	A	T	0.215035	-0.0294	0.0128	0.0216259	-0.155141	0.0101184	4.63E-53	0.189505	0.0834264	0.02311499	0.07001698	0.03660266	20	FALSE	Vosa et al., 2018	Blood
DAB2	ENSG00000153071	5	39417091	rs700234	5	39367548	T	A	0.456294	-0.014	0.0094	0.1363921	-0.421828	0.0093662	0	0.0331888	0.0222961	0.1366064	0.3411682	0.515011	20	FALSE	Vosa et al., 2018	Blood
FCHO2	ENSG00000157107	5	72319078	rs7713398	5	72252475	G	A	0.543706	-0.0109	0.0097	0.2611355	-0.610873	0.00811384	0	0.0178433	0.0158807	0.2611886	0.7163882	0.8704293	20	FALSE	Vosa et al., 2018	Blood
NECAP2	ENSG00000157191	1	16776870	rs14126	1	16795685	A	G	0.307692	-0.0074	0.0112	0.5087956	0.482482	0.0090249	0	-0.0153374	0.0232151	0.5088279	0.09917837	0.6905062	20	FALSE	Vosa et al., 2018	Blood
LDLRAP1	ENSG00000157978	1	25882724	rs4075184	1	25890835	G	A	0.51049	-0.0122	0.0093	0.1895782	-0.597094	0.00811792	0	0.0204323	0.0155779	0.1896486	0.000698549	0.02231415	15	FALSE	Vosa et al., 2018	Blood
SYNJ1	ENSG00000159082	21	34050714	rs13339977	21	34068582	T	C	0.0314685	-0.0052	0.0368	0.8876295	0.274067	0.0238888	1.81E-30	-0.0189735	0.134284	0.887638	0.1422818	0.231534	20	FALSE	Vosa et al., 2018	Blood
CD3G	ENSG00000160654	11	118220467	rs112563022	11	118262135	T	C	0.0437063	-0.0129	0.031	0.6773156	0.163719	0.026496	6.45E-10	-0.0787935	0.189778	0.6780039	0.7232884	0.6910372	10	FALSE	Vosa et al., 2018	Blood
AP2M1	ENSG00000161203	3	183897178	rs73185743	3	183912194	T	C	0.136364	-0.0006	0.0128	0.9626129	-0.500564	0.0179819	1.54E-170	0.00119865	0.0255712	0.9626129	0.1036225	0.334544	20	FALSE	Vosa et al., 2018	Blood
ARPC5	ENSG00000162704	1	183598646	rs2767304	1	183603399	G	A	0.398601	-0.0257	0.0097	0.00806147	0.604327	0.00850042	0	-0.0425266	0.016062	0.00810542	0.009385171	0.8227182	20	FALSE	Vosa et al., 2018	Blood
TGFA	ENSG00000163235	2	70727868	rs113971512	2	70774454	T	C	0.108392	0.0104	0.0151	0.4909858	0.352788	0.0159054	5.31E-109	0.0294795	0.0428226	0.4911947	0.6531362	0.2867675	20	FALSE	Vosa et al., 2018	Blood
ARPC2	ENSG00000163466	2	219100448	rs4674250	2	218977531	A	G	0.461538	-0.022	0.0094	0.01926178	-0.0711002	0.00900208	2.83E-15	0.309423	0.13789	0.02483377	0.002056132	0.2554198	12	FALSE	Vosa et al., 2018	Blood
GAPVD1	ENSG00000165219	9	128076779	rs10986629	9	127954644	G	T	0.543706	0.013	0.0095	0.1711803	0.0531071	0.00918426	7.36E-09	0.244789	0.183825	0.1829785	0.09223214	0.1718097	4	FALSE	Vosa et al., 2018	Blood
ARF6	ENSG00000165527	14	50360650	rs3100921	14	50351141	T	A	0.125874	0.0137	0.017	0.4203107	-0.0928465	0.0125201	1.21E-13	-0.147555	0.184176	0.4230358	0.8297605	0.1349597	11	FALSE	Vosa et al., 2018	Blood
CD3D	ENSG00000167286	11	118211564	rs2276423	11	118210106	G	C	0.230769	0.0095	0.0112	0.3963186	0.155629	0.0101067	1.67E-53	0.0610427	0.0720752	0.3970339	0.5368571	0.2641303	20	FALSE	Vosa et al., 2018	Blood
COPS6	ENSG00000168090	7	99688200	rs1130958	7	99690747	C	T	0.0664336	0.0066	0.0227	0.7712434	-0.901765	0.0198232	0	-0.007319	0.0251734	0.7712479	0.1475864	0.7730073	20	FALSE	Vosa et al., 2018	Blood
IL7R	ENSG00000168685	5	35866251	rs6879529	5	35850278	A	G	0.395105	-0.0027	0.0096	0.7785187	-0.182325	0.00926448	3.20E-86	0.0148087	0.0526586	0.7785407	0.6124498	0.4845569	20	FALSE	Vosa et al., 2018	Blood
ADRB2	ENSG00000169252	5	148207176	rs11959615	5	148201316	T	A	0.423077	-0.0211	0.0095	0.0263474	0.173891	0.00920955	1.62E-79	-0.12134	0.0550086	0.02739492	0.08992423	0.5306762	11	FALSE	Vosa et al., 2018	Blood
AGFG1	ENSG00000173744	2	228379126	rs4673196	2	228311150	G	A	0.347902	-0.0037	0.0104	0.7220134	-0.146053	0.00934024	4.07E-55	0.0253332	0.0712253	0.7220823	0.6306171	0.5101329	20	FALSE	Vosa et al., 2018	Blood
CLTB	ENSG00000175416	5	175831513	rs56184505	5	175559404	C	G	0.410839	-0.024	0.0105	0.02227098	0.415703	0.00939025	0	-0.0577336	0.0252921	0.0224496	0.1349135	0.4466951	20	FALSE	Vosa et al., 2018	Blood
GRB2	ENSG00000177885	17	73357973	rs55653371	17	73309355	G	A	0.270979	0.0247	0.0112	0.02742904	-0.217586	0.0117284	7.86E-77	-0.113518	0.0518364	0.02852871	0.01737699	0.3369027	20	FALSE	Vosa et al., 2018	Blood
GAK	ENSG00000178950	4	884612	rs34509509	4	888361	A	C	0.173077	-0.0413	0.0135	0.00221885	-0.107851	0.0145812	1.40E-13	0.382935	0.135457	0.00469869	0.0111039	7.42E-06	14	FALSE	Vosa et al., 2018	Blood
SNX18	ENSG00000178996	5	53828002	rs2059217	5	53912105	G	A	0.342657	0.001	0.0107	0.9255398	0.160198	0.00998852	6.91E-58	0.00624228	0.0667935	0.925541	0.8173273	0.4384966	20	FALSE	Vosa et al., 2018	Blood
AP2A2	ENSG00000183020	11	968566	rs66670048	11	999764	C	T	0.0944056	0.0131	0.0178	0.461758	-0.326388	0.0184557	5.47E-70	-0.0401362	0.0545835	0.4621455	0.2652658	0.1721969	20	FALSE	Vosa et al., 2018	Blood
ACTG1	ENSG00000184009	17	79483935	rs9897709	17	79454362	C	T	0.784965	0.0175	0.0132	0.18492	0.145017	0.00899277	1.68E-58	0.120675	0.0913306	0.1864014	0.1733493	0.6733801	20	FALSE	Vosa et al., 2018	Blood
HGS	ENSG00000185359	17	79660262	rs112084731	17	79679605	T	C	0.027972	0.0017	0.0399	0.9660152	0.568193	0.0436628	1.03E-38	0.00299194	0.070223	0.9660154	0.2802508	0.2784534	6	FALSE	Vosa et al., 2018	Blood
PIP5K1C	ENSG00000186111	19	3665329	rs1476591	19	3679607	G	C	0.159091	0.0025	0.0127	0.8439446	0.255867	0.0125262	9.70E-93	0.00977069	0.0496374	0.8439518	0.6617041	0.5903132	20	FALSE	Vosa et al., 2018	Blood
FNBP1	ENSG00000187239	9	132727469	rs7863899	9	132635193	T	G	0.113636	0.0156	0.016	0.3295603	0.516877	0.012767	0	0.0301812	0.0309641	0.3297005	0.008535617	0.3276275	20	FALSE	Vosa et al., 2018	Blood
AP2A1	ENSG00000196961	19	50290297	rs7247528	19	50291807	A	T	0.0891608	-0.0113	0.0174	0.5160635	0.121795	0.0181221	1.81E-11	-0.0927787	0.143528	0.518011	0.6470848			FALSE	Vosa et al., 2018	Blood
IGF2R	ENSG00000197081	6	160462335	rs6455672	6	160346153	A	G	0.281469	0.0047	0.0105	0.6544282	0.0893652	0.00893027	1.42E-23	0.0525932	0.117613	0.6547509	0.5419243	0.4179091	20	FALSE	Vosa et al., 2018	Blood
DNM3	ENSG00000197959	1	172099113	rs9425308	1	172383265	A	C	0.305944	-0.0261	0.0103	0.0112775	-0.157822	0.00981124	3.21E-58	0.165377	0.0660684	0.01231062	1.76E-05	0.3025447	20	TRUE	Vosa et al., 2018	Blood
ITSN2	ENSG00000198399	2	24504658	rs10198865	2	24590669	G	A	0.0227273	0.0129	0.0351	0.7132302	-0.158096	0.0255471	6.08E-10	-0.0815961	0.222409	0.7137123	0.8579831	0.344345	17	FALSE	Vosa et al., 2018	Blood
COPS8	ENSG00000198612	2	238001532	rs28727919	2	238003313	T	C	0.206294	-0.0175	0.013	0.1782529	0.0913744	0.0100824	1.27E-19	-0.19152	0.143833	0.1830097	0.1401516	0.9562958	20	FALSE	Vosa et al., 2018	Blood
ITSN1	ENSG00000205726	21	35143435	rs4817592	21	35029572	A	G	0.31993	-0.0281	0.0108	0.00927219	-0.584571	0.00820221	0	0.0480694	0.0184874	0.00931912	0.02393003	0.6690514	20	FALSE	Vosa et al., 2018	Blood
VAMP2	ENSG00000220205	17	8064665	rs80283923	17	8067755	T	C	0.0454545	-0.0119	0.0222	0.5919336	-0.245387	0.0153578	1.82E-57	0.0484948	0.0905202	0.592142	0.008933806	0.6352515	20	FALSE	Vosa et al., 2018	Blood
UBA52	ENSG00000221983	19	18685450	rs12608925	19	18687533	T	G	0.557692	-0.0014	0.0095	0.8828412	0.275529	0.00877334	1.71E-216	-0.0050811	0.0344795	0.8828425	0.2893193	0.2874441	20	FALSE	Vosa et al., 2018	Blood
ARPC4	ENSG00000241553	3	9844607	rs159159	3	9777158	C	A	0.272727	-0.0092	0.0109	0.3986489	0.0923212	0.00994583	1.66E-20	-0.0996521	0.118553	0.4005895	0.9421889	0.7864267	20	FALSE	Vosa et al., 2018	Blood
ARPC1A	ENSG00000241685	7	98954654	rs11973460	7	98980659	G	T	0.122378	-0.0195	0.0143	0.172682	-0.0567124	0.00940816	1.66E-09	0.34384	0.258521	0.1835086	0.3872084	0.3507336	15	FALSE	Vosa et al., 2018	Blood
STON1	ENSG00000243244	2	48791273	rs940389	2	48808152	C	G	0.325175	0.0092	0.01	0.3575728	0.208244	0.009273	1.09E-111	0.044179	0.0480609	0.3579758	0.3481086	0.366213	20	FALSE	Vosa et al., 2018	Blood

COPI DEPENDENT GOLGI TO ER RETROGRADE TRAFFIC (REACTOME)

Gene	probeID	ProbeChr	Probe bp	topSNP	topSNP chr	topSNP bp	A1	A2	Freq	b GWAS	se GWAS	p GWAS	b eQTL	se eQTL	p eQTL	b SMR	se SMR	p SMR	p SMR multi	p HEIDI	nsnp HEIDI	PASS BONF	Data source	Analyte
NSF	ENSG00000073969	17	44751432	rs17698176	17	44819595	G	T	0.20979	0.0652	0.0126	2.28E-07	0.410489	0.0534624	1.61E-14	0.158835	0.0370153	1.78E-05	5.66E-06	5.73E-07	8	TRUE	Qi et al., 2018	Brain
KIF16B	ENSG00000089177	20	16403413	rs4814508	20	16535556	T	C	0.477273	-0.0041	0.01	0.6818059	-0.259382	0.0385615	1.74E-11	0.0158068	0.0386247	0.6823632	0.8006928	0.8791343	20	FALSE	Qi et al., 2018	Brain
ARCN1	ENSG00000095139	11	118458417	rs11216892	11	118424008	C	T	0.874126	-0.0005	0.0141	0.9717121	0.407912	0.0672155	1.29E-09	-0.0012258	0.0345669	0.9717126	0.9945717	0.9967939	11	FALSE	Qi et al., 2018	Brain
KLC3	ENSG00000104892	19	45844776	rs10416849	19	45845231	C	G	0.624126	-0.0003	0.0106	0.9774214	-0.417433	0.0721858	7.35E-09	0.00071868	0.0253936	0.9774216	0.8390934	0.3370932	20	FALSE	Qi et al., 2018	Brain
NAPA	ENSG00000105402	19	48004695	rs55800589	19	47990889	G	C	0.924825	-0.0592	0.0195	0.00239821	-0.436473	0.0781223	2.31E-08	0.135633	0.0508459	0.00764128	0.007641279	0.114106	6	FALSE	Qi et al., 2018	Brain
COPE	ENSG00000105669	19	19020264	rs1127821	19	19011244	C	T	0.482517	0.0204	0.0096	0.03358661	0.386158	0.0386055	1.48E-23	0.0528282	0.0254151	0.03765309	0.04182814	0.632602	20	FALSE	Qi et al., 2018	Brain
BNIP1	ENSG00000113734	5	172581417	rs35387209	5	172560793	C	T	0.870629	-0.0059	0.0149	0.6921248	1.48317	0.0398944	1.58E-302	-0.003978	0.0100466	0.6921414	0.2756726	0.2472114	20	FALSE	Qi et al., 2018	Brain
KIF21B	ENSG00000116852	1	200965674	rs296562	1	200945274	A	C	0.312937	-0.0378	0.01	0.00015683	0.401268	0.0472881	2.15E-17	-0.0942014	0.0272818	0.00055459	0.001436248	0.036342	20	TRUE	Qi et al., 2018	Brain
COPA	ENSG00000122218	1	160286126	rs16831718	1	160233897	A	G	0.951049	0.0145	0.0227	0.5229748	0.530668	0.0935909	1.43E-08	0.0273241	0.0430469	0.5255906	0.5255906	0.597705	10	FALSE	Qi et al., 2018	Brain
KIF25	ENSG00000125337	6	168422770	rs11752225	6	168409629	T	C	0.825175	-0.0153	0.0137	0.2640848	-0.713605	0.0703164	3.37E-24	0.0214404	0.0193142	0.2669615	0.6736215	0.1772548	20	FALSE	Qi et al., 2018	Brain
NAPB	ENSG00000125814	20	23378642	rs6083085	20	23336119	A	G	0.711538	0.0097	0.0101	0.3368559	1.16048	0.0315164	8.42E-297	0.00835864	0.00870629	0.3370202	0.1172971	0.3125153	20	FALSE	Qi et al., 2018	Brain
KLC1	ENSG00000126214	14	104098060	rs709399	14	104167545	A	G	0.695804	0.0182	0.0103	0.07722985	0.374091	0.0526291	1.18E-12	0.0486512	0.0283714	0.08638189	0.1263823	0.4879868	20	FALSE	Qi et al., 2018	Brain
NAPG	ENSG00000134265	18	10539330	rs653624	18	10553789	A	C	0.545455	0.0036	0.01	0.7188471	-0.62377	0.0443143	5.33E-45	-0.0057714	0.0160368	0.7189351	0.3899611	0.4921442	20	FALSE	Qi et al., 2018	Brain
RINT1	ENSG00000135249	7	105190328	rs1728645	7	105205115	A	G	0.730769	0.0184	0.0116	0.1126924	0.279177	0.0487677	1.04E-08	0.0659081	0.0431163	0.1263608	0.1263608	0.5613005	17	FALSE	Qi et al., 2018	Brain
KDELR2	ENSG00000136240	7	6504728	rs4574760	7	6537655	T	C	0.428322	0.0011	0.0098	0.9106292	-0.236195	0.0375401	3.14E-10	-0.0046572	0.0414977	0.9106434	0.5434235	0.2178951	17	FALSE	Qi et al., 2018	Brain
KIF20B	ENSG00000138182	10	91498033	rs12254296	10	91541163	A	G	0.781469	-0.027	0.0136	0.04711176	0.345385	0.0500926	5.39E-12	-0.0781736	0.0409761	0.05641906	0.03032158	0.5467232	20	FALSE	Qi et al., 2018	Brain
CENPE	ENSG00000138778	4	104073264	rs72666813	4	104122401	A	G	0.206294	-0.0012	0.0118	0.9189988	0.374994	0.0618735	1.36E-09	-0.0032001	0.0314716	0.9190102	0.6013098	0.2185971	20	FALSE	Qi et al., 2018	Brain
NBAS	ENSG00000151779	2	15504243	rs11680706	2	15477375	C	T	0.699301	-0.0056	0.0109	0.6074188	-0.374605	0.0406885	3.36E-20	0.0149491	0.0291426	0.6079769	0.8844273	0.6974522	20	FALSE	Qi et al., 2018	Brain
KIF15	ENSG00000163808	3	44859038	rs17631716	3	44915603	C	T	0.715035	-0.0232	0.0103	0.02429529	0.356992	0.0539354	3.62E-11	-0.0649874	0.0304771	0.03297896	0.02444567	0.3104787	20	FALSE	Qi et al., 2018	Brain
KIF6	ENSG00000164627	6	39495473	rs6933563	6	39682934	T	C	0.921329	-0.0041	0.0174	0.8137181	-1.10745	0.0634825	3.75E-68	0.00370219	0.0157131	0.8137348	0.9782633	0.9831837	20	FALSE	Qi et al., 2018	Brain
TUBA1C	ENSG00000167553	12	49624816	rs77888738	12	49512929	G	A	0.312937	0.0323	0.0105	0.00209664	0.962472	0.0422804	1.04E-114	0.0335594	0.0110086	0.00230001	0.001930488	0.00833916	20	FALSE	Qi et al., 2018	Brain
KIFC2	ENSG00000167702	8	145695505	rs1480000	8	145702007	T	C	0.491259	0.0187	0.0096	0.05142494	-0.29177	0.0381031	1.90E-14	-0.0640915	0.0339505	0.05905367	0.04862203	0.5640562	20	FALSE	Qi et al., 2018	Brain
ARF4	ENSG00000168374	3	57570518	rs4597680	3	57596105	T	C	0.178322	-0.0084	0.0145	0.5623798	0.362071	0.0433555	6.76E-17	-0.0231999	0.0401436	0.5633171	0.2905735	0.76768	20	FALSE	Qi et al., 2018	Brain
STX18	ENSG00000168818	4	4480771	rs7685812	4	4413859	T	C	0.79021	-0.0036	0.0133	0.7866397	0.431033	0.0534783	7.63E-16	-0.008352	0.0308735	0.786757	0.4217891	0.250932	20	FALSE	Qi et al., 2018	Brain
RAB1B	ENSG00000174903	11	66040483	rs3814738	11	66060538	T	G	0.192308	-0.0149	0.0125	0.2332612	-0.362147	0.0560584	1.05E-10	0.0411435	0.035099	0.2411117	0.5604662	0.2254745	15	FALSE	Qi et al., 2018	Brain
TUBB6	ENSG00000176014	18	12325993	rs4073975	18	12279075	T	C	0.353147	0.0093	0.0111	0.4021218	-0.410417	0.0528212	7.85E-15	-0.0226599	0.0272024	0.4048397	0.5111237	0.8437164	13	FALSE	Qi et al., 2018	Brain
TMED9	ENSG00000184840	5	177021142	rs186803	5	177016095	C	T	0.548951	0.0133	0.0097	0.1703332	0.48477	0.0445706	1.49E-27	0.0274357	0.0201678	0.1737138	0.06208392	0.0575554	20	FALSE	Qi et al., 2018	Brain
ARFGAP3	ENSG00000242247	22	43223310	rs2017874	22	43303687	G	A	0.524476	-0.0278	0.0095	0.00343003	-0.410975	0.038457	1.18E-26	0.0676441	0.0239668	0.00476639	0.009671828	0.2290543	20	FALSE	Qi et al., 2018	Brain
KIF25	ENSG00000125337.12	6	168422770	rs11964963	6	168383708	C	T	0.258741	0.0116	0.0121	0.3377211	1.00514	0.159598	3.02E-10	0.0115407	0.0121768	0.3432515	0.3432515	0.5657828	7	FALSE	GTEx Consortium	Substantia Nigra
ARF5	ENSG00000004059	7	127230079	rs62483173	7	127230676	C	T	0.351399	-0.0157	0.0102	0.1237516	-0.0772763	0.00896851	6.91E-18	0.203167	0.134084	0.1297144	0.03828408	0.03268268	20	FALSE	Vosa et al., 2018	Blood
COPZ2	ENSG00000005243	17	46109462	rs17617053	17	46050669	C	T	0.0786713	0.0236	0.0165	0.1526301	0.595386	0.0247624	9.65E-128	0.0396381	0.0277621	0.1533555	0.371973	0.3712731	20	FALSE	Vosa et al., 2018	Blood
USE1	ENSG00000053501	19	17328396	rs34180454	19	17300714	T	A	0.183566	-0.0331	0.0125	0.00809695	0.133186	0.0136483	1.70E-22	-0.248525	0.097248	0.01060103	0.03247689	0.2440975	20	FALSE	Vosa et al., 2018	Blood
KIF1B	ENSG00000054523	1	10356262	rs11121529	1	10271688	G	C	0.127622	-0.0264	0.0156	0.09058732	0.849335	0.0107012	0	-0.0310831	0.0183715	0.09066063	0.02875276	0.1699354	20	FALSE	Vosa et al., 2018	Blood
KIF26A	ENSG00000066735	14	104626145	rs35797471	14	104621383	C	T	0.477273	0.0286	0.0129	0.02661938	-0.30015	0.00882112	9.09E-254	-0.0952856	0.0430696	0.02694159	0.007155438	0.1058412	20	FALSE	Vosa et al., 2018	Blood
KIF2A	ENSG00000068796	5	61717532	rs12659814	5	61569169	T	C	0.494755	0.0149	0.0096	0.1206423	-0.147817	0.00925246	1.88E-57	-0.1008	0.0652508	0.1223922	0.5217932	0.134323	20	FALSE	Vosa et al., 2018	Blood
NSF	ENSG00000073969	17	44751432	rs62074125	17	44852612	C	A	0.225524	0.0962	0.0129	8.83E-14	-0.36447	0.0144716	5.82E-140	-0.263945	0.0369129	8.65E-13	7.68E-18	6.47E-17	20	TRUE	Vosa et al., 2018	Blood
KIFAP3	ENSG00000075945	1	169972408	rs114812663	1	170046290	T	C	0.0576923	0.033	0.022	0.1336144	-0.338612	0.0277319	2.74E-34	-0.0974567	0.0654596	0.136538	0.1751855	0.9067883	13	FALSE	Vosa et al., 2018	Blood
KIF3C	ENSG00000084731	2	26177544	rs10201610	2	26149231	G	T	0.256993	-0.0092	0.0111	0.4072013	0.105472	0.00894119	4.08E-32	-0.0872268	0.105501	0.4083558	0.2900426	0.1525701	20	FALSE	Vosa et al., 2018	Blood
TMED2	ENSG00000086598	12	124076097	rs4930709	12	124099093	T	C	0.263986	0.0273	0.0108	0.0114787	0.200834	0.0128932	1.05E-54	0.135933	0.0544791	0.01259083	0.04766081	0.1190123	20	FALSE	Vosa et al., 2018	Blood

ZW10	ENSG00000086827	11	113624221	rs12295684	11	113753178	C	G	0.22028	-0.0145	0.0126	0.2498171	0.0596983	0.0103921	9.21E-09	-0.242888	0.215255	0.2591615	0.3536947	0.4362361	14	FALSE	Vosa et al., 2018	Blood
KIF16B	ENSG00000089177	20	16403413	rs6034535	20	16552964	A	C	0.118881	0.002	0.0144	0.889538	-0.896473	0.0117504	0	-0.002231	0.016063	0.8895381	0.8322402	0.5641599	20	FALSE	Vosa et al., 2018	Blood
ARCN1	ENSG00000095139	11	118458426	rs55844025	11	118446298	T	C	0.125874	0.0005	0.0145	0.9724922	-0.206456	0.0107123	9.09E-83	-0.0024218	0.0702329	0.9724922	0.2788453	0.6310105	20	FALSE	Vosa et al., 2018	Blood
TUBB1	ENSG00000101162	20	57598009	rs463312	20	57597970	C	A	0.0332168	0.0138	0.0273	0.6132116	-0.150253	0.0199191	4.59E-14	-0.0918449	0.182101	0.6140061	0.6754224	0.4085688	3	FALSE	Vosa et al., 2018	Blood
ARFGAP1	ENSG00000101199	20	61912639	rs13042357	20	61916379	C	T	0.590909	0.0086	0.0102	0.3991517	0.113752	0.00916022	2.09E-35	0.0756033	0.0898755	0.4002356	0.7658326	0.8321593	20	FALSE	Vosa et al., 2018	Blood
KIF3B	ENSG00000101350	20	30894140	rs12480429	20	30907865	G	T	0.113636	-0.0085	0.0149	0.5683591	-0.0891452	0.0136183	5.91E-11	0.09535	0.167776	0.5698206	0.5123211	0.3859264	20	FALSE	Vosa et al., 2018	Blood
TUBB4A	ENSG00000104833	19	6498594	rs2071347	19	6495736	A	G	0.347902	0.0184	0.0137	0.1792507	0.100279	0.00921581	1.42E-27	0.183488	0.137655	0.1825481	0.1071711	0.9145709	20	FALSE	Vosa et al., 2018	Blood
KLC3	ENSG00000104892	19	45845735	rs9749618	19	45849909	G	A	0.335664	-0.0027	0.01	0.7871603	0.267171	0.0110403	2.25E-129	-0.0101059	0.0374315	0.7871732	0.188512	0.1532032	20	FALSE	Vosa et al., 2018	Blood
NAPA	ENSG00000105402	19	48004695	rs56162770	19	48048515	C	T	0.0227273	-0.0129	0.0315	0.6821553	-0.231804	0.0242043	9.99E-22	0.0556504	0.136015	0.6824297	0.07711796	0.5532164	7	FALSE	Vosa et al., 2018	Blood
KDELR1	ENSG00000105438	19	48890318	rs433956	19	48891537	G	A	0.456294	-0.0112	0.0101	0.2674686	0.0914499	0.00954413	9.54E-22	-0.122471	0.11118	0.2706537	0.2599942	0.8387747	20	FALSE	Vosa et al., 2018	Blood
COPZ1	ENSG00000111481	12	54720309	rs61614746	12	54740214	A	G	0.101399	-0.0021	0.0162	0.8968595	0.171147	0.0134159	2.85E-37	-0.0122702	0.0946604	0.8968648	0.1242202	0.03828737	20	FALSE	Vosa et al., 2018	Blood
BNIP1	ENSG00000113734	5	172581417	rs72814179	5	172489416	G	C	0.129371	0.0053	0.015	0.7238386	-0.682071	0.0173199	0	-0.0077705	0.0219927	0.7238492	0.298174	0.2885399	20	FALSE	Vosa et al., 2018	Blood
KIF21B	ENSG00000116852	1	200965674	rs296560	1	200949369	A	T	0.0192308	0.0781	0.0308	0.01122182	-0.141175	0.01555	1.10E-19	-0.553214	0.226519	0.01459625	0.01908725	0.1236097	19	FALSE	Vosa et al., 2018	Blood
COPA	ENSG00000122218	1	160286126	rs7523769	1	160257980	T	A	0.426573	0.0171	0.0101	0.09044228	0.241861	0.00898009	9.01E-160	0.0707018	0.0418419	0.09107886	0.4161103	0.6778984	20	FALSE	Vosa et al., 2018	Blood
TUBA1B	ENSG00000123416	12	49523372	rs2162644	12	49548190	G	A	0.681818	-0.029	0.0101	0.00408804	-0.0869697	0.00893831	2.25E-22	0.333449	0.121083	0.00588928	0.001977113	0.1202147	20	FALSE	Vosa et al., 2018	Blood
NAPB	ENSG00000125814	20	23378642	rs6137947	20	23414630	A	C	0.0874126	0.0018	0.0171	0.916167	0.620053	0.0127258	0	0.00290298	0.0275783	0.9161672	0.2018572	0.826956	20	FALSE	Vosa et al., 2018	Blood
KLC1	ENSG00000126214	14	104098060	rs2403197	14	104053764	T	C	0.258741	0.0067	0.0108	0.535014	0.340022	0.00996526	3.63E-255	0.0197046	0.0317679	0.5350815	0.01720556	0.3070526	20	FALSE	Vosa et al., 2018	Blood
TUBA4A	ENSG00000127824	2	220128662	rs11552796	2	220108300	C	T	0.0996503	-0.0027	0.0155	0.8617134	-0.0889864	0.0120535	1.55E-13	0.0303417	0.174232	0.8617514	0.3492557	0.4658161	12	FALSE	Vosa et al., 2018	Blood
COPB1	ENSG00000129083	11	14493279	rs2575859	11	14510736	C	T	0.365385	-0.0073	0.01	0.4653902	-0.524693	0.00910794	0	0.0139129	0.0190603	0.465426	0.6220089	0.4036075	20	FALSE	Vosa et al., 2018	Blood
KIF1C	ENSG00000129250	17	4916469	rs16954257	17	4821992	T	A	0.0944056	0.0312	0.0141	0.02691379	0.123825	0.0105491	8.14E-32	0.251968	0.115876	0.02967015	0.01275133	0.1526035	20	FALSE	Vosa et al., 2018	Blood
KIF3A	ENSG00000131437	5	132050825	rs7731422	5	132075653	C	A	0.0891608	0.0178	0.0166	0.2835902	-0.0889079	0.00929279	1.10E-21	-0.200207	0.187879	0.2865967	0.3177243	0.4315601	10	FALSE	Vosa et al., 2018	Blood
NAPG	ENSG00000134265	18	10539330	rs643757	18	10545688	A	T	0.545455	0.0042	0.01	0.6744855	-0.295929	0.00898338	5.55E-238	-0.0141926	0.0337947	0.6745104	0.704584	0.5270544	20	FALSE	Vosa et al., 2018	Blood
ARF3	ENSG00000134287	12	49324310	rs833833	12	49336805	A	G	0.0821678	0.0095	0.0174	0.5850817	-0.532201	0.0125293	0	-0.0178504	0.0326971	0.5851127	0.003525027	0.06587165	20	FALSE	Vosa et al., 2018	Blood
TMED7	ENSG00000134970	5	114958947	rs400570	5	114951553	A	G	0.421329	-0.0202	0.0095	0.03347697	0.071852	0.00907909	2.49E-15	-0.281134	0.136905	0.04002519	0.01620505	0.3653903	18	FALSE	Vosa et al., 2018	Blood
RINT1	ENSG00000135249	7	105190328	rs113947297	7	105188996	A	G	0.034965	-0.0111	0.0307	0.7176782	0.441616	0.0355042	1.62E-35	-0.025135	0.0695468	0.7177923	0.2752664	0.2704048	16	FALSE	Vosa et al., 2018	Blood
KDELR2	ENSG00000136240	7	6504728	rs6796	7	6502367	C	T	0.201049	-0.0077	0.0117	0.5104612	-0.777609	0.00823323	0	0.00990215	0.0150465	0.5104715	0.000262155	0.144612	20	TRUE	Vosa et al., 2018	Blood
KLC4	ENSG00000137171	6	43025676	rs75664886	6	43039403	A	G	0.034965	-0.0079	0.0265	0.7656168	0.357367	0.0341286	1.17E-25	-0.0221061	0.0741834	0.7657089	0.7741691	0.3691906	20	FALSE	Vosa et al., 2018	Blood
TUBB2A	ENSG00000137267	6	3155831	rs9392465	6	3162378	A	C	0.681818	0.0051	0.0109	0.6398633	-0.596583	0.00813605	0	-0.0085487	0.0182711	0.6398701	0.6785826	0.2155316	20	FALSE	Vosa et al., 2018	Blood
TUBB2B	ENSG00000137285	6	3228229	rs9328145	6	3144908	A	G	0.697552	0.0137	0.011	0.2129647	0.154385	0.00889662	1.87E-67	0.0887393	0.0714338	0.214141	0.3289312	0.3902203	20	FALSE	Vosa et al., 2018	Blood
RAB1A	ENSG00000138069	2	65327537	rs10185243	2	65355744	G	T	0.346154	-0.0222	0.0101	0.02794769	-0.0581237	0.00922129	2.92E-10	0.381944	0.18403	0.03794488	0.05921797	0.4412455	15	FALSE	Vosa et al., 2018	Blood
KIF11	ENSG00000138160	10	94384096	rs17875345	10	94413667	A	G	0.0367133	0.0642	0.0253	0.01116316	0.196867	0.0228331	6.58E-18	0.326108	0.133963	0.01492021	0.3551713	0.4014078	6	FALSE	Vosa et al., 2018	Blood
KIF20B	ENSG00000138182	10	91498033	rs1126480	10	91503660	G	A	0.218531	0.021	0.011	0.05625037	0.262395	0.00969348	2.27E-161	0.0800321	0.0420257	0.05686323	0.1534608	0.6363302	20	FALSE	Vosa et al., 2018	Blood
CENPE	ENSG00000138778	4	104073264	rs2623063	4	104055722	A	G	0.206294	-0.0003	0.0116	0.9793674	0.0913569	0.0121634	5.88E-14	-0.0032838	0.126975	0.9793675	0.3122099	0.05820131	20	FALSE	Vosa et al., 2018	Blood
KIF21A	ENSG00000139116	12	39762111	rs186246651	12	39875785	A	G	0.0034965	0.016	0.1115	0.8858971	-0.576691	0.0853623	1.42E-11	-0.0277445	0.193388	0.8859226	0.9797125	0.8615233	4	FALSE	Vosa et al., 2018	Blood
ARF1	ENSG00000143761	1	228278636	rs964941	1	228193857	G	A	0.444056	-0.0213	0.0096	0.02650374	0.0667856	0.00893501	7.74E-14	-0.318931	0.149943	0.03341852	0.02967317	0.660646	20	FALSE	Vosa et al., 2018	Blood
SURF4	ENSG00000148248	9	136235647	rs9411464	9	136109165	T	C	0.048951	-0.0005	0.0194	0.9794382	0.0960234	0.0148767	1.09E-10	-0.0052071	0.202036	0.9794384	0.6112701	0.4748834	20	FALSE	Vosa et al., 2018	Blood
ARFGAP2	ENSG00000149182	11	47192262	rs189817094	11	47044040	T	C	0.0157343	0.0221	0.038	0.5608503	0.662879	0.0479749	2.01E-43	0.0333394	0.0573764	0.561197	0.7616957	0.6292681	20	FALSE	Vosa et al., 2018	Blood
NBAS	ENSG00000151779	2	15504243	rs13416086	2	15688107	C	T	0.232517	0.0006	0.0109	0.9561019	-0.150735	0.00955747	4.90E-56	-0.0039805	0.0723129	0.9561022	0.8970829	0.5558412	20	FALSE	Vosa et al., 2018	Blood
COPG2	ENSG00000158623	7	130249843	rs138265226	7	130273974	C	T	0.16958	-0.027	0.0134	0.04391247	-0.479426	0.0100026	0	0.0563173	0.0279748	0.04409914	0.1360468	0.7034579	20	FALSE	Vosa et al., 2018	Blood
RACGAP1	ENSG00000161800	12	50398812	rs60042968	12	50381442	G	T	0.0769231	-0.0224	0.0189	0.2359442	0.202438	0.0152142	2.14E-40	-0.110651	0.0937318	0.2377969	0.4415594	0.7043189	20	FALSE	Vosa et al., 2018	Blood
KIF26B	ENSG00000162849	1	245595510	rs79856122	1	245676418	C	A	0.0664336	-0.0023	0.0231	0.920688	0.141062	0.0123934	5.14E-30	-0.0163049	0.163765	0.920691	0.5406792	0.9279367	5	FALSE	Vosa et al., 2018	Blood
KIF27	ENSG00000165115	9	86493977	rs117608988	9	86285391	C	T	0.0104895	-0.0637	0.0627	0.3096537	1.71422	0.0689633	2.17E-136	-0.0371597	0.0366069	0.3100575	0.1990079	0.9853054	8	FALSE	Vosa et al., 2018	Blood
TMED3	ENSG00000166557	15	79653869	rs117679487	15	79600248	C	T	0.048951	0.0274	0.0313	0.3813566	0.463363	0.0255815	2.51E-73	0.0591329	0.0676284	0.381912	0.06633355	0.348001	20	FALSE	Vosa et al., 2018	Blood
TUBA1A	ENSG00000167552	12	49580843	rs11168868	12	49515130	C	T	0.0297203	-0.0293	0.0387	0.4489864	0.467466	0.0446874	1.31E-25	-0.0626784	0.0830034	0.4501705	0.0410797	0.4141611	17	FALSE	Vosa et al., 2018	Blood
KIFC2	ENSG00000167702	8	145695505	rs67557810	8	145750550	T	C	0.0576923	-0.0155	0.0218	0.4770786	-0.535772	0.018259	2.94E-189	0.0289302	0.0407009	0.4772078	0.1560801	0.3503784	20	FALSE	Vosa et al., 2018	Blood
ARF4	ENSG00000168374	3	57570518	rs149547076	3	57557444	C	T	0.0104895	0.1544	0.0626	0.01364584	1.37819	0.0692642	4.27E-88	0.112031	0.0457695	0.01437632	0.1238314	0.2815744	9	FALSE	Vosa et al., 2018	Blood
STX18	ENSG00000168818	4	4480771	rs4689174	4	4579042	A	G	0.262238	0.0037	0.0112	0.7411301	0.0805878	0.0101137	1.61E-15	0.0459127	0.139098	0.7413444	0.8727704	0.8693255	3	FALSE	Vosa et al., 2018	Blood
TMED10	ENSG00000170348	14	75620753	rs7146787	14	75696922	G	A	0.237762	0.0112	0.0116	0.3342858	-0.103433	0.00961259	5.31E-27	-0.108282	0.1126	0.3362236	0.2031045	0.2055898	18	FALSE	Vosa et al., 2018	Blood
KIF5B	ENSG00000170759	10	32321648	rs211290	10	32336397	A	G	0.132867	-0.0111	0.015	0.4593	0.466431	0.0171125	1.38E-163	-0.0237978	0.032171	0.4594654	0.2324679	0.4790436	20	FALSE	Vosa et al., 2018	Blood
KLC2	ENSG00000174996	11	66030048	rs7951079	11	65980550	T	C	0.18007	-0.0144	0.0125	0.2493211	-0.0863909	0.0113341	2.49E-14	0.166684	0.146334	0.2546765	0.4888804	0.3369406	15	FALSE	Vosa et al., 2018	Blood
TUBB6	ENSG00000176014	18	12325993	rs7244254	18	12317836	A	G	0.146853	-0.0075	0.0145	0.6049877	0.7473	0.00935293	0	-0.0100361	0.0194036	0.6049952	0.6975407	0.5065391	20	FALSE	Vosa et al., 2018	Blood
COPG1	ENSG00000181789	3	128982531	rs6762578	3	128992047	G	A	0.208042	0.0132	0.0121	0.2753129	0.141587	0.0112508	2.57E-36	0.0932287	0.0857801	0.2771105	0.3789393	0.5581697	18	FALSE	Vosa et al., 2018	Blood
COPB2	ENSG00000184432	3	139091508	rs7375091	3	139090389	A	T	0.625874	0.0122	0.0103	0.2362286	-0.152809	0.00939589	1.79E-59	-0.0798382	0.0675829	0.2374688	0.3142642	0.4131157	20	FALSE	Vosa et al., 2018	Blood
TMED9	ENSG00000184840	5	177021142	rs186803	5	177016095	C	T	0.548951	0.0133	0.0097	0.1703332	0.475321	0.00848082	0	0.0279811	0.0204134	0.170461	0.05755959	0.09279697	20	FALSE	Vosa et al., 2018	Blood
KIF19	ENSG00000196169	17	72337154	rs2670826	17	72462466	C	T	0.587413	0.0023	0.0101	0.8198618	0.166408	0.00931236	2.03E-71	0.0138214	0.0606991	0.8198761	0.1565188	0.2349242	20	FALSE	Vosa et al., 2018	Blood
KIF13B	ENSG00000197892	8	29022718	rs4732667	8	29057186	T	G	0.106643	0.0062	0.0162	0.7019303	-0.168985	0.0102102	1.59E-61	-0.0366897	0.0958923	0.7020062	0.2864966	0.03157592	20	FALSE	Vosa et al., 2018	Blood
ARFGAP3	ENSG00000242247	22	43223310	rs5751391	22	43261499	A	G	0.482517	0.0268	0.0096	0.00524373	-0.21107	0.00938908	6.46E-112	-0.126972	0.0458318	0.00559897	0.03697477	0.1209283	20	FALSE	Vosa et al., 2018	Blood

COPI MEDIATED ANTEROGRADE TRANSPORT (REACTOME)

Gene	probeID	ProbeChr	Probe bp	topSNP	topSNP chr	topSNP bp	A1	A2	Freq	b GWAS	se GWAS	p GWAS	b eQTL	se eQTL	p eQTL	b SMR	se SMR	p SMR	p SMR multi	p HEIDI	nsnp HEIDI	PASS BONF	Data source	Analyte
NSF	ENSG00000073969	17	44751432	rs17698176	17	44819595	G	T	0.20979	0.0652	0.0126	2.28E-07	0.410489	0.0534624	1.61E-14	0.158835	0.0370153	1.78E-05	5.66E-06	5.73E-07	8	TRUE	Qi et al., 2018	Brain
CAPZB	ENSG00000077549	1	19738666	rs12117356	1	19658162	C	G	0.807692	0.0036	0.0118	0.7603016	1.10409	0.0318829	9.04E-263	0.00326059	0.0106879	0.7603106	0.8477152	0.8377617	20	FALSE	Qi et al., 2018	Brain
CD59	ENSG00000085063	11	33738899	rs3181269	11	33755956	C	T	0.769231	0.0002	0.0118	0.9864772	0.570688	0.0551161	4.00E-25	0.00035045	0.0206768	0.9864772	0.6003399	0.8547608	20	FALSE	Qi et al., 2018	Brain
DYNLL1	ENSG00000088986	12	120921974	rs12310837	12	120880572	A	G	0.802448	-0.0254	0.0128	0.04721405	0.313888	0.0464368	1.39E-11	-0.0809206	0.0424998	0.05690721	0.03121166	0.362197	14	FALSE	Qi et al., 2018	Brain
[truncated: 2,198,086 more chars]
